# Supplementary material for: How large should a cause of death be in order to be included in mortality trend analysis? Deriving a cut-off point from retrospective trend analyses in 21 European countries
Source: BMJ Open. 2020 Jan 21;10(1):e031702. doi: 10.1136/bmjopen-2019-031702 (PMC7044923; doi:10.1136/bmjopen-2019-031702)
Supplement: Author's manuscript [file bmjopen-2019-031702.draft_revisions.pdf]

BMJ Open is committed to open peer review. As part of this commitment we make the peer review history of every article we publish publicly available.

When an article is published we post the peer reviewers' comments and the authors' responses online. We also post the versions of the paper that were used during peer review. These are the versions that the peer review comments apply to.

The versions of the paper that follow are the versions that were submitted during the peer review process. They are not the versions of record or the final published versions. They should not be cited or distributed as the published version of this manuscript.

BMJ Open is an open access journal and the full, final, typeset and author-corrected version of record of the manuscript is available on our site with no access controls, subscription charges or pay-per-view fees (http://bmjopen.bmj.com).

If you have any questions on BMJ Open's open peer review process please email editorial.bmjopen@bmj.com

BMJ Open

How large should a cause of death be in order to be included in mortality trend analysis? deriving a cut-off point from analyses of trends in 21 European countries

Journal:	*BMJ Open*
Manuscript ID	bmjopen-2019-031702
Article Type:	Original research
Date Submitted by the Author:	17-May-2019
Complete List of Authors:	Mitratza, Marianna; Amsterdam UMC, University of Amsterdam, Department of Public Health<br>Kardaun, Jan; Statistics Netherlands, Department of Health and Care, ; Amsterdam UMC, University of Amsterdam, Department of Public Health<br>Kunst, Anton; Amsterdam UMC, University of Amsterdam, Department of Public Health
Keywords:	mortality, trends, cause of death, size criterion, Europe

SCHOLARONE™
Manuscripts

Title

How large should a cause of death be in order to be included in mortality trend analysis? deriving a cut-off point from analyses of trends in 21 European countries

Authors

Marianna Mitratza, Department of Public Health, Amsterdam UMC, University of Amsterdam, Amsterdam, the Netherlands

Jan WPF Kardaun, Department of Health and Care, Statistics Netherlands, The Hague, the Netherlands
& Department of Public Health, Amsterdam UMC, University of Amsterdam, Amsterdam, the Netherlands

Anton E Kunst, Department of Public Health, Amsterdam UMC, University of Amsterdam, Amsterdam, the Netherlands

Author responsible for correspondence about the manuscript

Marianna Mitratza

Address: Meibergdreef 9, 1105 AZ Amsterdam, Netherlands

Telephone : +31205664658

Email : m.mitratza@amc.uva.nl

Disclaimers

None

Funding

For peer review only

None

Word count

2540

Number of figures and tables

2 tables, 3 figures

For peer review only

For peer review only - http://bmjopen.bmj.com/site/about/guidelines.xhtml

Abstract

Objectives: The International Classification of Diseases (ICD10) distinguishes a large number of causes of death (CODs) potentially useful for monitoring time-trends. We aimed to develop recommendations for using the size of CODs as a criterion for their inclusion in long-term trend analysis.

Design: Retrospective trend analysis

Setting: 21 European countries of the WHO Mortality Database

Participants: Deaths from causes of death (3-position ICD10 codes) with ≥5 average annual deaths in a 15-year period between 2000 and 2016

Primary and secondary outcome measures: Fitting regression models, we examined whether each COD in each country had a demonstrable long-term trend and assessed correlates of this outcome. Applying ROC curve diagnostics, we derived COD size thresholds for selecting CODs for trends analysis.

Results: Across all countries, 64.0% of CODs had demonstrable long-term trends. The odds of having a demonstrable trend increased by 18% for every 10% increase of COD size. The independent effect of country was negligible. As compared to circulatory system diseases, the probability of detecting a trend was lower for neoplasms and digestive system diseases, and higher for infectious diseases, mental diseases and signs-and-symptoms. We derived a general threshold of around 30 (range: 28-33) annual deaths for inclusion of a COD in trend analysis. The relevant threshold for neoplasms was around 65 (range: 61-70) and for infectious diseases was 20 (range: 19-20).

For peer review only

For peer review only - http://bmjopen.bmj.com/site/about/guidelines.xhtml

Conclusions: The potential to detect long-term trends is strongly related to COD size and varies between ICD10 chapters, but has no independent relation to country. We recommend a general size criterion of 30 annual deaths to select CODs for long-term mortality-trends analysis in European countries.

- What is already known on this subject ?

Until now, causes of death are selected for monitoring purposes based on arbitrary conventions or criteria, such as being part of established short lists. This approach leaves little room for taking into account properties of the data, such as the number of deaths and the type of disease.

- What this study adds ?

We have developed a criterion for the selection of causes of death based on their annual number of deaths. On the basis of analysis of data from 21 countries with up to 791 causes of death per country, we identified a cut-off point at about 30 deaths annually. This criterion is flexible to variation according to ICD10 chapter, and is applicable to all European countries.

Strengths and limitations of this study

- The first study to develop a criterion to select causes of death for monitoring purposes based on their annual number of deaths.
- The use of a large sample of causes of death covering most European countries, using the WHO Mortality Database.
- Criteria for selection of causes were derived for different types of causes of death.
- Other criteria were not applied, such as causes of death that involve high healthcare costs or that are potentially modifiable.

Introduction

Mortality data are essential for the monitoring of population-wide trends in a large number of diseases and injuries, as well as for the evaluation of health policies. A common source for

For peer review only - http://bmjopen.bmj.com/site/about/guidelines.xhtml

these data are the statistics maintained by national statistical offices **[1, 2]**. National statistics of causes of death (CODs) include many ICD10 codes (10th revision of the International Classification of Diseases) **[3].** Given the detail of this classification **-** there are 1752 3-position ICD-10 codes - a part of it may not be instrumental for monitoring long-term time-trends due to the small number of deaths for specific codes.

When using these statistics to monitor long-term trends in mortality, a main question is which of the many possible CODs to include. At the very least, the selection should include only CODs that are large enough to have a reasonable probability of detecting a long-term mortality trend. This probability may be influenced by several factors. One main factor is the COD size, defined as the mean annual number of deaths, which expresses the rarity of a disease or condition that is selected as underlying cause of death in a population. Incidence changes or effects of interventions are common factors discussed in mortality trends analyses **[4, 5]**. In addition, this probability might depend on other factors, such as the type of COD, or the country of interest. Certain types of CODs may be more likely to present a long-term trend. For example, neoplasms have been shown to be more gradual in their annual changes **[6]**, whereas infectious diseases **[7]** may have high year-to-year variation. As regards to different populations, the likelihood to detect a long-term trend for a COD may vary between countries because of differences in population size, COD coding practices that may also influence observed mortality trends **[8]**, trends in prevalence of risk factors **[9, 10, 11, 12]**, implementation of new prevention strategies **[13, 14]**, treatment protocols **[5]** or healthcare reforms **[15]**.

Due to the fact that the likelihood to detect a long-term trend of a COD may depend on various factors, there is a need for an empirical assessment of such likelihood. Such analysis may provide an empirical basis for the identification of CODs for which long-trends are likely to be detectable. More specifically, it may be used to define a criterion, or rule of thumb, that identifies eligible CODs in terms of a minimum COD size. When such a criterion allows for

For peer review only - http://bmjopen.bmj.com/site/about/guidelines.xhtml

variation by COD type and country, it may be used in national and international trend analysis across a broad range of CODs.

The general objective of this study was to determine a COD size criterion for the study of long-term mortality trends in European countries. The specific objectives were: (1) to assess the association between the size and the type of a COD and the probability of detecting a long-term trend in European countries, (2) to assess how this association varies according to country, and (3) to identify a minimum annual number of deaths recommended to monitor trends in cause-specific mortality.

Methods

Data

We used annual mortality data for 21 European countries of the WHO Mortality Database (1 October 2017 update) **[16]**. We included the 21 countries of the European Union (28 countries) or the European Free Trade Association (4 countries) that had been using ICD10 (3- or 4-position) coding for at least 15 consecutive years. Iceland, Luxemburg and Malta were excluded because of their small population **[17]**. The most recent 15-year period was selected, which was 2001-2015 for all countries with few exceptions (Belgium, France and Switzerland: 2000-2014; Austria: 2002-2016). If the time series of a COD in a country was interrupted by a year without any data on that COD, we assumed that zero cases occurred.

Statistical analysis

For each year and COD in a country, we calculated an age-standardized count of deaths using the direct method. As reference population, we used the age-distribution of the European Standard Population 2013, scaled to the mid-period population of each country. This method

For peer review only - http://bmjopen.bmj.com/site/about/guidelines.xhtml

intended to compensate for annual changes in the age-distribution of the population, while keeping the age-standardized count close to the observed absolute numbers.

For further analysis, we analyzed CODs that had at least 5 average age-standardized annual deaths, because most of the smaller CODs had predominantly zero or only zero annual deaths.

Long-term time-trends of the age-standardized count of deaths of each COD in each country were analyzed using regression models. Trends were fitted by applying linear regression models with polynomial terms of year as continuous, independent covariates. We used orthogonal polynomials in order to account for multicollinearity of the polynomial components **[18]**. We fitted four models: the constant, the linear, the quadratic and the cubic model (with zero, first, second and third degree polynomials respectively). The four models were applied for all CODs in each country. We used the lowest corrected Akaike Information Criterion (AICc) to select the best model for each COD in each country **[19]**. In a next step, the best model was compared with the constant model using the F-test, at the significance level of $\alpha=0.05$. If the best model performed better than the constant model with statistical significance, it was kept as the final best model. Otherwise, the constant model was selected as the best model for this COD. In the rest of the paper, the constant model is referred to as the absence of a demonstrable trend.

Next, using a multilevel logistic regression model, we determined how the categorization of a COD as having a demonstrable trend (i.e. best model being the linear, quadratic or cubic model) was related to COD size and COD type. These variables were included in the model as fixed effects. The COD size was defined as the mean annual number of deaths and the COD type was defined as the ICD10 chapter in which it is classified. The chapter of circulatory diseases was the reference category, as it had the largest number deaths. As the distribution of the number of deaths across CODs was highly skewed, we used its natural logarithm as a measure of COD size. The model also included the level of countries as random effect, in order

For peer review only - http://bmjopen.bmj.com/site/about/guidelines.xhtml

to investigate the variation of European countries in the likelihood of detecting a long-term trend. We calculated the Intra-class Correlation Coefficients (ICC), which expresses the proportion of the variance in the outcome that is attributable to variations between the countries **[20]**. The ICC was calculated both with and without controlling for the fixed effects of the size and type of the COD.

Finally, we used receiver operating characteristic (ROC) curve diagnostics **[21, 22, 23]** to derive COD size thresholds for detecting a long-term time-trend. We calculated the Area Under the Curve (AUC) of the logistic model with COD size as predictor the and the binary categorization of a COD as having a detectable long-term time-trend as the outcome. We derived the COD size thresholds using three indices. Firstly, we used the maximum Youden index **[24, 25, 26]**, which represents the point of the ROC curve with the maximum sum of sensitivity (se) and specificity (sp). Secondly, we used the index measuring the minimum difference between sensitivity and specificity **[22]**. Thirdly, we estimated the index that represents the point closest to the top-left part of the ROC curve **[21, 25]**.

All analyses were conducted using R statistical software (3.5.1 version) **[27]**.

Patient and Public Involvement

No patients were involved in this study.

Results

The number of CODs with at least 5 annual deaths on average varied between 202 (Estonia) and 791 (Germany) **(Table 1)**. Of these CODs, 32.6%, 20.2% and 11.2% had a demonstrable trend following a linear, quadratic or cubic model respectively. The percentage of COD with no demonstrable trend (i.e. constant model) varied from 27.5% to 43.9%, and

For peer review only - http://bmjopen.bmj.com/site/about/guidelines.xhtml

was highest in the Nordic countries, Switzerland and Slovenia. More detailed information on the best model for each COD in each European country can be found in an additional file **(Online Resource 1)**.

For peer review only

For peer review only - http://bmjopen.bmj.com/site/about/guidelines.xhtml

Table 1:

Frequencies of causes of deaths according to estimates of their long-term time-trend in 21 European countries

**Country** (sorted by mean population)	**CODs* analyzed (n)**	**Type of long-term time-trend (%)**				**Mean population (thousands)**	**Mean crude annual deaths from all CODs together**	**Mean crude annual deaths from CODs analyzed***
		**No demonstrable trend**	**Linear**	**Quadratic**	**Cubic**			
**Estonia**	202	34.7	35.6	15.3	14.4	1,343	16,642	16,006
**Slovenia**	228	43.9	29.4	17.1	9.6	2,029	18,830	18,163
**Latvia**	259	38.2	28.6	17.0	16.2	2,157	30,840	30,091
**Lithuania**	308	37.7	32.5	19.5	10.4	3,187	42,101	41,243
**Croatia**	315	34.6	37.1	15.9	12.4	4,381	51,444	50,844
**Norway**	356	42.4	26.4	23.6	7.6	4,804	41,764	40,886
**Finland**	355	42.8	35.2	16.6	5.4	5,302	49,869	48,933
**Denmark**	420	40.0	37.4	13.6	9.0	5,497	54,446	53,319
**Switzerland**	451	43.9	31.0	16.4	8.6	7,558	62,183	61,384
**Austria**	374	35.8	25.4	23.5	15.2	8,359	77,256	75,765
**Sweden**	457	43.3	30.6	19.5	6.6	9,259	91,504	90,516
**Hungary**	482	36.1	27.6	29.3	7.1	10,024	130,830	129,695
**Czech Republic**	443	31.8	35.9	23.0	9.3	10,383	107,448	106,636
**Belgium**	517	37.3	33.7	20.9	8.1	10,678	104,344	103,257

For peer review only - http://bmjopen.bmj.com/site/about/guidelines.xhtml

**Netherlands**	554	33.9	28.0	25.1	13.0	16,489	138,373	137,645
**Romania**	444	31.8	36.0	19.4	12.8	21,594	258,000	257,265
**Poland**	581	27.7	33.2	22.9	16.2	38,114	375,231	374,295
**Spain**	672	33.0	33.9	18.2	14.9	44,594	385,512	383,204
**United Kingdom**	710	30.8	33.8	21.1	14.2	61,690	580,733	578,572
**France**	738	27.5	38.8	22.6	11.1	61,709	535,320	532,293
**Germany**	791	27.8	35.4	24.7	12.1	81,980	852,566	849,400

* coded at ICD10, 3-position level; including all causes of death with at least 5 mean number of deaths in the 15-year-period

Both COD size and COD type were significantly associated with the likelihood of having a demonstrable long-term trend (p-value <0.001) **(Table 2)**. For every 10% increase in the COD size, we observed a 18% increase (1.1^1.73=0.18) in the odds of having a detectable trend (OR=1.73, 95%CI=1.67;1.79). Regarding the COD type, neoplasms and digestive system diseases had lower probability for detecting a trend in comparison to the circulatory system diseases. On the other hand, this probability was higher for infectious diseases, mental diseases and signs-and-symptoms had higher. **Fig1** shows for each COD chapter in each country the estimated probability of having a long-term trend in relation to COD size. The variation between COD chapters is substantial, irrespective of COD size. Neoplasms (chapter C00.D48) as a group of CODs have the lowest probability of having a detectable trend.

We found only small variation of countries in the likelihood of detecting a long-term trend, as the ICC for the country-level random effect was only 0.013 (without fixed effects for chapter and size) and 0.003 (with fixed effects) **(Table 2)**. **Fig2** illustrates the small differences between countries in the estimated probability of having a long-term trend.

Table 2:

For peer review only - http://bmjopen.bmj.com/site/about/guidelines.xhtml

For peer review only

Relationship between the likelihood for a cause of death (COD) to have a demonstrable long-term with its size, corresponding ICD10 chapter and country

COD characteristic		Number of CODs	Total number of deaths	Odds Ratio*** (95% Confidence Interval)
**Size**				
log(mean deaths*)			-	**1.73** (1.67 ; 1.79)
**ICD10 Chapter**				
I00.I99	Diseases of the circulatory system	988	23,610,116	reference
A00.B99	Certain infectious and parasitic diseases	454	823,839	**1.63** (1.25 ; 2.14)
C00.D48	Neoplasms	1871	15,632,543	**0.57** (0.47 ; 0.69)
D50.D89	Diseases of blood and blood-forming organs and certain disorders involving the immune mechanisms	217	143,156	0.82 (0.59 ; 1.14)
E00.E90	Endocrine, nutritional and metabolic diseases	365	1,547,431	1.01 (0.76 ; 1.34)
F00.F99	Mental, behavioural disorders	230	1,725,881	**1.62** (1.13 ; 2.30)
G00.G99	Diseases of the nervous system	536	1,888,629	0.90 (0.70 ; 1.15)
J00.J99	Diseases of the respiratory system	553	4,740,481	1.22 (0.94 ; 1.58)
K00.K93	Diseases of the digestive system	767	2,816,168	**0.75** (0.59 ; 0.94)
M00.M99	Diseases of the musculoskeletal system and connective tissue	367	283,108	1.14 (0.86 ; 1.50)
N00.N99	Diseases of the genitourinary system	385	1,029,118	0.98 (0.74 ; 1.29)
Q00.Q99	Congenital malformations, deformations and chromosomal abnormalities	325	141,848	0.89 (0.67 ; 1.19)
R00.R99	Symptoms, signs and abnormal clinical and laboratory findings, not elsewhere classified (signs-and-symptoms)	258	2,062,587	**1.68** (1.18 ; 2.38)
V01.Y98	External causes of morbidity and mortality	1992	3,005,539	1.20 (0.99 ; 1.46)
Other**		349	240,748	1.29 (0.97 ; 1.71)

For peer review only - http://bmjopen.bmj.com/site/about/guidelines.xhtml

For peer review only

**Intra-class correlation for the country level**	
model with fixed effects for size and ICD10 chapter	0.003
model with no fixed effects	0.013

*mean deaths : mean of the annual number of deaths for a cause of death monitored in the 15-year-period, measured per country. Only including CODs with 5 or more deaths.

** "Other" consists of the causes of death classified in the ICD-10 chapters H00.H59: Diseases of the eye and adnexa, H60.H95: Diseases of the ear and mastoid process, L00.L99: Diseases of the skin and subcutaneous tissue, O00.O99: Pregnancy, childbirth and the puerperium and P00.P96: Certain conditions originating in the perinatal period.

***odds ratios in bold are statistically significant with p-value < 0.05

Fig3a describes the sensitivity (se) and specificity (sp) for detecting a long-term trend using different levels of thresholds in terms of any COD size. The AUC corresponding to these se and sp values was 0.706, with 95%CI:0.695;0.716. The maximized sum index (Youden Index) was 32.7 annual deaths, with sensitivity (se) 61.4% and specificity (sp) 70.3%. The minimum difference index was 27.5 annual deaths (se= 65.5%, sp= 65.5%). The closest top-left index was 29.4 annual deaths (se= 64.0%, sp= 67.5%) (Figure 3a).

The corresponding analysis for the neoplasms yielded a similar ROC curve (AUC= 0.703, 95%CI:0.680;0.727) **(Fig3b)**. The Youden Index was 70.4, with sensitivity 61.5% and specificity 69.6%, and the minimum difference index was 60.5 annual deaths (se= 64.8%, sp= 64.8%). The closest top-left index was also 60.5 annual deaths (se= 64.1%, sp= 66.9%). Infectious and parasitic diseases (AUC= 0.706, 95%CI:0.695;0.716) yielded a Youden Index of 19.7 annual deaths, with sensitivity 67.1% and specificity 69.8%. The closest top-left index was identical, while the minimum difference threshold was 19.3 annual deaths (se= 67.4%, sp= 67.4%) **(Fig3c)**.

Discussion

For peer review only - http://bmjopen.bmj.com/site/about/guidelines.xhtml

COD data are used in widely varying settings, ranging from detailed mortality profiles to macro estimates. Applications include studies in localized areas **[28]**, single countries **[29]** or worldwide **[2, 30]**; for a single-disease **[31, 32]** or disease group **[9]**; monitored for days or a long-term period **[7]**; for specific age groups **[32, 33]** or specific situations (e.g. maternal **[5]** mortality, external causes **[34, 35, 36]**). These settings all impose different requirements on the collected data. Here we focused on one particular application: national estimates of mortality time trends for a reasonably long period (15 years), for a considerable number of countries (21) that have quite comparable CODs data collection and registration systems **[37]**, covering as many CODs as possible. Our aim was to investigate the effect of the size of a COD on the probability to detect a trend, and how this is related to country and type of COD (ICD10 chapter).

Our results indicate that both the size and the type of a cause of death were associated with the probability of detecting a trend, while variations among European countries were negligible. Some types of COD, particularly neoplasms and digestive system diseases, had a lower probability for detecting a trend in comparison to the circulatory system diseases, whereas infectious and mental diseases had a higher probability. The results suggest a general size criterion of 30 annual deaths for selecting causes of death to include in long-term mortality trends analysis, and a more specific criterion on 65 deaths for neoplasms and 20 for infectious diseases.

We should outline the limitations of our study. Firstly, due to the exclusion of causes of death with less than 5 annual deaths on average, smaller countries were represented in our analysis with fewer causes of death. However, this is unlikely to have a strong influence on the results, as the suggested COD size threshold of about 30 deaths is much higher than the lower limit of 5 mean annual deaths. Secondly, although we proposed the COD size as a criterion to select CODs for long-term trend analysis, we acknowledge that other criteria

For peer review only - http://bmjopen.bmj.com/site/about/guidelines.xhtml

could be used, such as greater preference to CODs that involve high healthcare costs or that are potentially modifiable by preventive or curative actions.

We found that mortality from neoplasms was less likely to have a demonstrable trend, for a given size of COD. This may relate to the fact that mortality from neoplasms tend to develop rather gradually throughout the years **[6]**. Additionally, cancers are usually coded reliably and consistently over time **[38, 39, 40]**, so that coding artefacts can rarely induce artificial changes. Conversely, the dynamic nature of infectious diseases may be responsible for their higher likelihood to change over time, and to have detectable trends even with relatively small numbers of deaths. Similarly, the chapter of signs-and-symptoms is sensitive to changes in the coding rules and practices, thus creating detectable changes even with small number of deaths.

For peer review only

Our study showed that European countries did not vary substantially in the probability of detecting a long-term trend in CODs of the same size and type. This finding is surprising given the heterogeneity of the countries in terms of demographic characteristics, disease epidemiology, healthcare systems and coding practices. We found that differences between countries in the proportions of CODs with a detectable trend (shown in Table 1) can be related to differences in COD size, which is strongly related to the differences in population size. Consequently, our analysis provides support for establishing one common COD size threshold, applicable for all European countries and for use in international trend analyses.

Currently there is no gold standard for the selection of CODs to analyze for long-term trends. In this study we attempted to set such a standard, based on the criterion of the COD size, which is easy to measure for each single COD. We calculated thresholds with three common methods, which came close enough (e.g. in the range of 28 to 33 deaths) to support one general recommendation for practical use. Of course, different thresholds may be preferred,

For peer review only - http://bmjopen.bmj.com/site/about/guidelines.xhtml

depending on the user's preference to avoid either false positives (by selecting a higher threshold) or false negatives (lower threshold).

In our data, the number of CODs that surpassed our recommended threshold of 30 annual deaths on average is around 500 for the biggest countries, 200-250 for the middle-sized countries and around 100 for the smaller European countries (results not shown). In total, 52 CODs had over 30 annual deaths on average in each country included in our analysis. This implies that at least 52 CODs could be included in the international comparison of long-term trends, but up to 100 if one is to accept a greater risk of false positives in smaller countries.

From the public health practitioner's perspective, the findings of our study can be used in order to set realistic expectations about the number of CODs that are likely to have a detectable long-term trend in populations. We recommend a size criterion of 30 annual deaths to be considered when planning for national or international monitoring and comparisons of cause-specific mortality.

For peer review only

References

1. Mathers CD, Fat DM, Inoue M, Rao C, Lopez AD. Counting the dead and what they died from: an assessment of the global status of cause of death data. Bull World Health Organ. 2005;83(3):171-7.

2. Naghavi M, Abajobir AA, Abbafati C, Abbas KM, Abd-Allah F, Abera SF, et al. Global, regional, and national age-sex specific mortality for 264 causes of death, 1980-2016: a systematic analysis for the Global Burden of Disease Study 2016. Lancet. 2017;390(10100):1151-210.

For peer review only - http://bmjopen.bmj.com/site/about/guidelines.xhtml

3. World Health Organization. International statistical classification of diseases and related health problems. Tenth revision, vol. 2. Geneva, Switzerland: WHO; 1993.

4. Aregawi M, Malm KL, Wahjib M, Kofi O, Allotey N-K, Yaw PN, et al. Effect of anti-malarial interventions on trends of malaria cases, hospital admissions and deaths, 2005–2015 ,Ghana. Malar J. 2017;16(1):177.

5. Mnyani CN, Buchmann EJ, Chersich MF, Frank KA, McIntyre JA. Trends in maternal deaths in HIV-infected women, on a background of changing HIV management guidelines in South Africa: 1997 to 2015. J Int AIDS Soc. 2017;20(3): e25022.

6. Torre LA, Siegel RL, Ward EM, Jemal A. Global cancer incidence and mortality rates and trends-an update. Cancer Epidemiol Biomarkers Prev. 2016;25(1):16-27.

7. Armstrong GL, Conn, LA, Pinner RW. Trends in infectious disease mortality in the United States during the 20th century. JAMA. 1999;281(1):61-6.

8. Griffiths C, Brock A, Rooney C. The impact of introducing ICD-10 on trends in mortality from circulatory diseases in England and Wales. Health statistics quarterly / Office for National Statistics. 2004(22):14-20.

9. Choe YJ, Choe SA, Cho SI. Trends in infectious disease mortality, South Korea, 1983-2015. Emerg Infect Dis. 2018;24(2):320-7.

10. Van Dyke M, Greer S, Odom E, Schieb L, Vaughan A, Kramer M, et al. Heart disease death rates among blacks and whites Aged ≥35 Years - United States, 1968-2015. MMWR Surveill Summ. 2018;67(5):1-11.

For peer review only

For peer review only - http://bmjopen.bmj.com/site/about/guidelines.xhtml

11. Hertel-Fernandez AW, Giusti AE, Sotelo JM. The Chilean infant mortality decline: improvement for whom? Socioeconomic and geographic inequalities in infant mortality, 1990-2005. Bull World Health Organ. 2007;85(10):798-804.

12. Mesalles-Naranjo O, Grant I, Wyper GMA, Stockton D, Dobbie R, McFadden M, et al. Trends and inequalities in the burden of mortality in Scotland 2000-2015. PloS One. 2018;13(8):e0196906.

13. Medina-Gomez OS, Medina-Reyes IS. Mortality from type 2 diabetes and implementation of the PREVENIMSS program: a time series study in Mexico, 1998-2015. Cad Saude Publica. 2018;34(5):e00103117.

14. Allanson ER, Pattinson RC. Quality-of-care audits and perinatal mortality in South Africa. Bull World Health Organ. 2015;93(6):424-8.

15. Notrica DM, Sayrs LW, Krishna N. The effect of verified pediatric trauma centers, state laws, and crash characteristics on time trends in adolescent motor vehicle fatalities, 1999-2015. J Trauma Acute Care Surg. 2018;85(5):944-52.

16. WHO Mortality Database. Geneva: World Health Organization; 2017. Available from: http://www.who.int/healthinfo/mortality_data/en/ [accessed 5 October 2017].

17. Eurostat - European Union. Chapter 7: Demographic challenges-population projections. In: People in the EU: Who are we and how do we live? Eurostat Statistical Books. Luxemburg: Publications Office of the European Union; 2015. p. 159.

18. Shacham M, Brauner N. Minimizing the effects of collinearity in polynomial regression. Ind Eng Chem Res. 1997;36:4405-12.

19. Wong CS, Li WK. A note on the corrected Akaike information criterion for threshold autoregressive models. J Time Ser Anal. 1998;19(1):113-24.

For peer review only

For peer review only - http://bmjopen.bmj.com/site/about/guidelines.xhtml

20. Diez Roux AV. A glossary for multilevel analysis. J Epidemiol Community Health. 2002;56:588-94.

21. Metz CE. Basic principles of ROC analysis. Semin Nucl Med. 1978;8(4):283-98.

22. Greiner M, Pfeiffer D, Smith RD. Principles and practical application of the receiver-operating characteristic analysis for diagnostic tests. Prev Vet Med. 2000;45(1-2):23–41.

23. Zou KH, O'Malley AJ, Mauri L. Receiver-operating characteristic analysis for evaluating diagnostic tests and predictive models. Circulation. 2007;115(5):654–7.

24. Youden WJ. Index for rating diagnostic tests. Cancer. 1950;3(1):32–5.

25. Perkins NJ, Schisterman EF. The inconsistency of "optimal" cutpoints obtained using two criteria based on the receiver operating characteristic curve. Am J Epidemiol. 2006;163(7):670–5.

26. Fluss R, Faraggi D, Reiser B. Estimation of the Youden Index and its associated cutoff point. Biom J. 2005;47(4):458–72.

27. R Core Team (2018). R: A language and environment for statistical computing. R Foundation for Statistical Computing, Vienna, Austria: https://www.R-project.org.

28. Bradshaw D, Groenewald P, Bourne DE, Mahomed H, Nojilana B, Daniels J, et al. Making COD statistics useful for public health at local level in the city of Cape Town: utilidad para la salud publica a nivel local en Ciudad del Cabo. Bull World Health Organ. 2006;84(3):211-7.

29. Franca EB, Passos VMA, Malta DC, Duncan BB, Ribeiro ALP, Guimaraes MDC, et al. Cause-specific mortality for 249 causes in Brazil and states during 1990-2015: a systematic analysis for the global burden of disease study 2015. Popul Health Metr. 2017;15(1):39.

For peer review only - http://bmjopen.bmj.com/site/about/guidelines.xhtml

30. Ebmeier S, Thayabaran D, Braithwaite I, Benamara C, Weatherall M, Beasley R. Trends in international asthma mortality: analysis of data from the WHO Mortality Database from 46 countries (1993-2012). Lancet. 2017;390(10098):935-45.

31. Burkill S, Montgomery S, Hajiebrahimi M, Hillert J, Olsson T, Bahmanyar S. Mortality trends for multiple sclerosis patients in Sweden from 1968 to 2012. Neurology. 2017;89(6):555-62.

32. Reynales-Shigematsu LM, Guerrero-Lopez CM, Hernandez Avila M, Irving H, Jha P. Divergence and convergence in cause-specific premature adult mortality in Mexico and US Mexican Hispanics from 1995 to 2015: analyses of 4.9 million individual deaths. Int J Epidemiol. 2018;47(1):97-106.

33. Oza S, Lawn JE, Hogan DR, Mathers C, Cousens SN. Neonatal cause-of-death estimates for the early and late neonatal periods for 194 countries: 2000-2013. Bull World Health Organ. 2015;93(1):19-28.

34. Mack K, Clapperton A, Macpherson A, Sleet D, Newton D, Murdoch J, et al. Trends in the leading causes of injury mortality, Australia, Canada, and the United States, 2000-2014. Can J Public Health. 2017;108(2):e185-e91.

35. Padron-Monedero A, Damian J, Pilar Martin M, Fernandez-Cuenca R. Mortality trends for accidental falls in older people in Spain, 2000-2015. BMC Geriatr. 2017;17(1):276.

36. Ajdacic-Gross V, Weiss MG, Ring M, Hepp U, Bopp M, Gutzwiller F, et al. Methods of suicide: international suicide patterns derived from the WHO mortality database. Bull World Health Organ. 2008;86(9):726-32.

37. Commission Regulation (EU) No 328/2011 of 5 April 2011 implementing Regulation (EC) No 1338/2008 of the European Parliament and of the Council on Community statistics on public health and health and safety at work, as regards statistics on causes of death Text with EEA relevance. Official Journal of the European Union.

For peer review only

For peer review only - http://bmjopen.bmj.com/site/about/guidelines.xhtml

38. Harteloh P, de Bruin K, Kardaun J. The reliability of cause-of-death coding in The Netherlands. Eur J Epidemiol. 2010;25(8):531–8.

39. Lu TH, Lee MC, Chou MC. Accuracy of cause-of-death coding in Taiwan: types of miscoding and effects on mortality statistics. Int J Epidemiol. 2000;29(2):336–43.

40. Mieno MN, Tanaka N, Arai T, Kawahara T, Kuchiba A, Ishikawa S, et al. Accuracy of death certificates and assessment of factors for misclassification of underlying cause of death. J Epidemiol. 2016;26(4):191-8.

Footnotes

Contributors: AK, JK and MM designed the study. MM performed the calculations. MM, AK and JK analyzed and interpreted the results and formulated the conclusions, and made major contributions to the manuscript. All authors read and approved the final manuscript.

Funding: This research received no specific grant from any funding agency in the public, commercial or not-for-profit sectors.

Conflict of Interest: The authors declare that they have no conflict of interest.

Ethics approval: No ethical approval was required for this study, as no living subjects were involved and only aggregated administrative data were used.

Data sharing statement: All data used are publically available from the WHO Mortality Database (see https://www.who.int/healthinfo/mortality_data/en/).

Provenance and peer review: Not commissioned; externally peer reviewed.

Patient consent for publication: Not required.

For peer review only - http://bmjopen.bmj.com/site/about/guidelines.xhtml

Figure Captions

Fig1

Estimated probability for an underlying cause of death to have a demonstrable long-term trend according to its size, by ICD10 chapter

Footnote: *See Table 2 for the definition of the chapters.

Fig2

Estimated probability for a disease of the circulatory system to have a demonstrable long-term mortality trend according to its size, by European country

Fig3

Sensitivity and specificity of the cause of death size for the detection of long-term time-trends, with thresholds for the optimal cause of death size for trend analysis

For peer review only

For peer review only - http://bmjopen.bmj.com/site/about/guidelines.xhtml

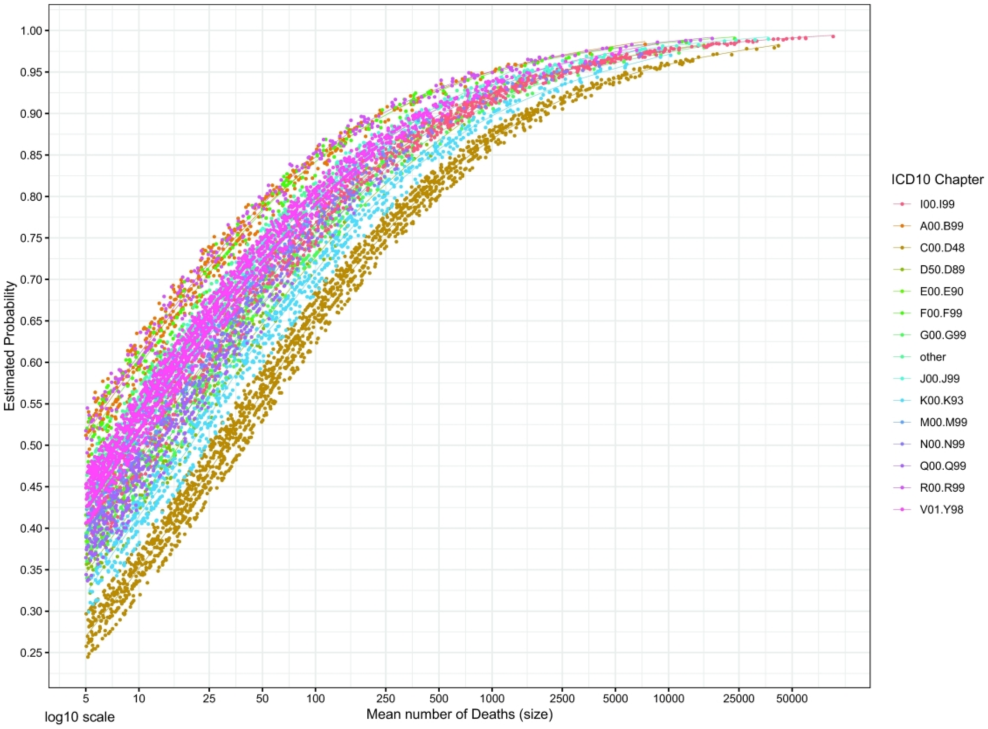

279x215mm (300 x 300 DPI)

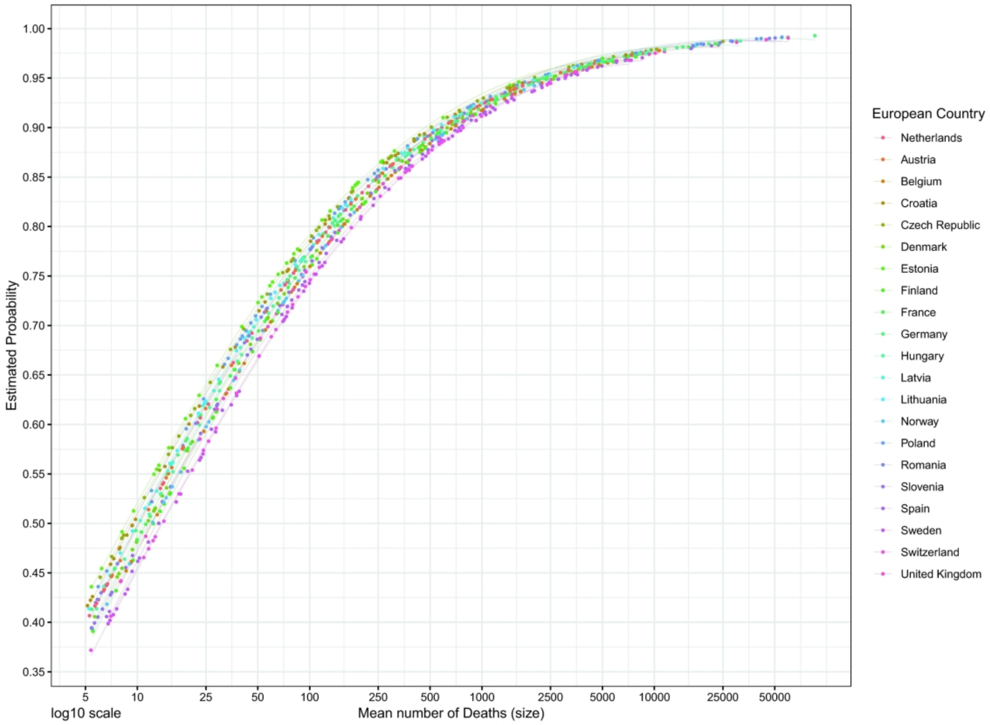

279x215mm (300 x 300 DPI)

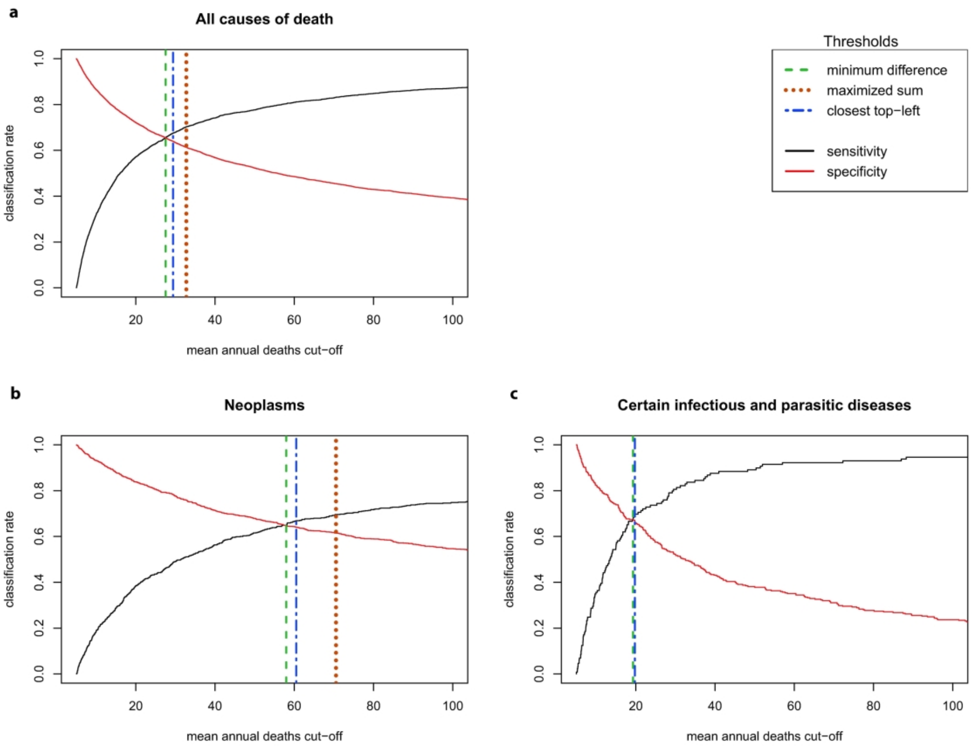

279x217mm (300 x 300 DPI)

For peer review only

Title of the article: How large should a cause of death be in order to be included in mortality trend analysis? d[illegible]

Journal: Journal of Epidemiology and Community Health

Authors: Marianna Mitratza, Jan WPF Kardaun, Anton E Kunst

Corresponding author: Marianna Mitratza
Department of Public Health, Amsterdam UMC, University of Amsterdam, Amsterdam, the Netherlands
Email : m.mitratza@amc.uva.nl

For peer review only

ICD10 code
A02
A02
A02
A02
A02
A02
A02
A02
A02
A04
A04
A04
A04
A04
A04
A04
A04
A04
A04
A04
A04
A04
A04
A04
A04
A05
A05
A05
A08
A08
A08
A08
A08
A08
A08
A08
A08
A08
A08
A08
A08
A09
A09
A09
A09
A09
A09
A09
A09

For peer review only

A09
A09
A09
A09
A09
A09
A09
A09
A09
A15
A15
A15
A15
A15
A15
A15
A15
A15
A16
A16
A16
A16
A16
A16
A16
A16
A16
A16
A16
A16
A16
A16
A16
A16
A16
A16
A17
A17
A17
A17
A17
A17
A18
A18
A18
A18
A18
A18
A19
A19

For peer review only

A19
A19
A19
A19
A19
A19
A19
A19
A26
A27
A31
A31
A31
A31
A31
A31
A32
A32
A32
A32
A35
A35
A35
A37
A39
A39
A39
A39
A39
A39
A39
A39
A39
A39
A39
A39
A39
A39
A40
A40
A40
A40
A40
A40
A40
A40
A40
A40
A40
A40

For peer review only

A40
A40
A40
A41
A41
A41
A41
A41
A41
A41
A41
A41
A41
A41
A41
A41
A41
A41
A41
A41
A41
A41
A41
A41
A43
A43
A46
A46
A46
A46
A46
A46
A46
A46
A46
A46
A46
A46
A46
A46
A46
A46
A48
A48
A48
A48
A48
A48
A48
A48

For peer review only

A48
A48
A48
A48
A48
A49
A49
A49
A49
A49
A49
A49
A49
A49
A49
A49
A49
A49
A49
A52
A52
A69
A81
A81
A81
A81
A81
A81
A81
A81
A81
A81
A81
A81
A81
A81
A81
A86
A86
A86
A86
A86
A86
A87
A87
A87
B00
B00
B00
B00

For peer review only

B00
B00
B00
B00
B00
B01
B01
B01
B01
B02
B02
B02
B02
B02
B02
B02
B02
B02
B02
B15
B16
B16
B16
B16
B16
B16
B16
B17
B17
B17
B17
B17
B17
B17
B17
B17
B18
B18
B18
B18
B18
B18
B18
B18
B18
B18
B18
B18
B18
B18

For peer review only

B18
B18
B18
B18
B18
B18
B19
B19
B19
B19
B19
B20
B20
B20
B20
B20
B20
B20
B20
B20
B20
B20
B20
B20
B20
B21
B21
B21
B21
B21
B21
B21
B21
B21
B22
B22
B22
B22
B22
B22
B22
B22
B22
B22
B23
B23
B23
B23
B23

For peer review only

B23
B23
B24
B24
B24
B24
B24
B24
B24
B24
B24
B24
B25
B25
B25
B25
B33
B34
B34
B34
B34
B34
B34
B34
B34
B34
B34
B34
B34
B37
B37
B37
B37
B37
B37
B37
B37
B37
B37
B44
B44
B44
B44
B44
B44
B44
B49
B49
B49
B49

For peer review only

B50
B54
B55
B59
B59
B59
B59
B59
B59
B67
B67
B67
B90
B90
B90
B90
B90
B90
B90
B90
B90
B90
B90
B90
B90
B91
B91
B91
B91
B91
B91
B91
B91
B91
B91
B91
B91
B94
B94
B94
B94
B94
B94
B94
B99
B99
B99
B99
B99
B99

For peer review only

B99
B99
B99
B99
B99
C00
C00
C00
C00
C00
C00
C00
C00
C00
C00
C00
C00
C00
C00
C01
C01
C01
C01
C01
C01
C01
C01
C01
C01
C01
C01
C01
C01
C01
C01
C01
C01
C01
C01
C02
C02
C02
C02
C02
C02
C02
C02
C02
C02
C02

For peer review only

C02
C02
C02
C02
C02
C02
C02
C02
C02
C02
C03
C03
C03
C03
C03
C03
C03
C03
C03
C03
C03
C03
C03
C03
C03
C03
C04
C04
C04
C04
C04
C04
C04
C04
C04
C04
C04
C04
C04
C04
C04
C04
C04
C04
C04
C04
C04
C05
C05
C05

For peer review only

C05
C05
C05
C05
C05
C05
C05
C05
C05
C05
C05
C05
C05
C05
C05
C06
C06
C06
C06
C06
C06
C06
C06
C06
C06
C06
C06
C06
C06
C06
C06
C06
C06
C06
C06
C07
C07
C07
C07
C07
C07
C07
C07
C07
C07
C07
C07
C07
C07
C07

For peer review only

C07
C07
C07
C07
C07
C08
C08
C08
C08
C08
C08
C08
C08
C08
C08
C08
C08
C08
C08
C08
C08
C08
C08
C09
C09
C09
C09
C09
C09
C09
C09
C09
C09
C09
C09
C09
C09
C09
C09
C09
C09
C09
C09
C10
C10
C10
C10
C10
C10
C10

For peer review only

C10
C10
C10
C10
C10
C10
C10
C10
C10
C10
C10
C10
C10
C10
C11
C11
C11
C11
C11
C11
C11
C11
C11
C11
C11
C11
C11
C11
C11
C11
C11
C11
C11
C11
C12
C12
C12
C12
C12
C12
C12
C12
C12
C12
C12
C12
C12
C12
C13
C13

For peer review only

C13
C13
C13
C13
C13
C13
C13
C13
C13
C13
C13
C13
C13
C13
C13
C13
C13
C13
C13
C14
C14
C14
C14
C14
C14
C14
C14
C14
C14
C14
C14
C14
C14
C14
C14
C14
C14
C14
C14
C15
C15
C15
C15
C15
C15
C15
C15
C15
C15
C15

For peer review only

C15
C15
C15
C15
C15
C15
C15
C15
C15
C15
C16
C16
C16
C16
C16
C16
C16
C16
C16
C16
C16
C16
C16
C16
C16
C16
C16
C16
C16
C16
C16
C17
C17
C17
C17
C17
C17
C17
C17
C17
C17
C17
C17
C17
C17
C17
C17
C17
C17
C17

For peer review only

C17
C17
C18
C18
C18
C18
C18
C18
C18
C18
C18
C18
C18
C18
C18
C18
C18
C18
C18
C18
C18
C18
C18
C19
C19
C19
C19
C19
C19
C19
C19
C19
C19
C19
C19
C19
C19
C19
C19
C19
C19
C19
C19
C19
C20
C20
C20
C20
C20
C20

For peer review only

C20
C20
C20
C20
C20
C20
C20
C20
C20
C20
C20
C20
C20
C20
C20
C21
C21
C21
C21
C21
C21
C21
C21
C21
C21
C21
C21
C21
C21
C21
C21
C21
C21
C21
C21
C21
C22
C22
C22
C22
C22
C22
C22
C22
C22
C22
C22
C22
C22
C22

For peer review only

C22
C22
C22
C22
C22
C22
C22
C23
C23
C23
C23
C23
C23
C23
C23
C23
C23
C23
C23
C23
C23
C23
C23
C23
C23
C23
C23
C23
C24
C24
C24
C24
C24
C24
C24
C24
C24
C24
C24
C24
C24
C24
C24
C24
C24
C24
C24
C24
C24
C25

For peer review only

C25
C25
C25
C25
C25
C25
C25
C25
C25
C25
C25
C25
C25
C25
C25
C25
C25
C25
C25
C25
C26
C26
C26
C26
C26
C26
C26
C26
C26
C26
C26
C26
C26
C26
C26
C26
C26
C26
C26
C26
C26
C30
C30
C30
C30
C30
C30
C30
C30
C30

For peer review only

C30
C30
C30
C30
C30
C31
C31
C31
C31
C31
C31
C31
C31
C31
C31
C31
C31
C31
C31
C31
C31
C31
C31
C31
C31
C31
C32
C32
C32
C32
C32
C32
C32
C32
C32
C32
C32
C32
C32
C32
C32
C32
C32
C32
C32
C32
C32
C33
C33
C33

For peer review only

C33
C33
C33
C33
C33
C33
C33
C33
C34
C34
C34
C34
C34
C34
C34
C34
C34
C34
C34
C34
C34
C34
C34
C34
C34
C34
C34
C34
C34
C37
C37
C37
C37
C37
C37
C37
C37
C37
C37
C37
C37
C38
C38
C38
C38
C38
C38
C38
C38
C38

For peer review only

C38
C38
C38
C38
C38
C38
C38
C38
C38
C38
C38
C38
C39
C39
C39
C39
C39
C39
C39
C39
C39
C39
C39
C39
C40
C40
C40
C40
C40
C40
C40
C40
C40
C40
C40
C40
C40
C40
C40
C40
C40
C40
C40
C41
C41
C41
C41
C41
C41
C41

For peer review only

C41
C41
C41
C41
C41
C41
C41
C41
C41
C41
C41
C41
C41
C41
C43
C43
C43
C43
C43
C43
C43
C43
C43
C43
C43
C43
C43
C43
C43
C43
C43
C43
C43
C43
C43
C44
C44
C44
C44
C44
C44
C44
C44
C44
C44
C44
C44
C44
C44
C44

For peer review only

C44
C44
C44
C44
C44
C44
C45
C45
C45
C45
C45
C45
C45
C45
C45
C45
C45
C45
C45
C45
C45
C45
C45
C45
C45
C45
C46
C46
C46
C46
C46
C46
C46
C47
C47
C47
C47
C47
C47
C47
C47
C47
C47
C47
C47
C48
C48
C48
C48
C48

For peer review only

C48
C48
C48
C48
C48
C48
C48
C48
C48
C48
C48
C48
C48
C48
C48
C48
C49
C49
C49
C49
C49
C49
C49
C49
C49
C49
C49
C49
C49
C49
C49
C49
C49
C49
C49
C49
C49
C50
C50
C50
C50
C50
C50
C50
C50
C50
C50
C50
C50
C50

For peer review only

C50
C50
C50
C50
C50
C50
C50
C50
C51
C51
C51
C51
C51
C51
C51
C51
C51
C51
C51
C51
C51
C51
C51
C51
C51
C51
C51
C51
C51
C52
C52
C52
C52
C52
C52
C52
C52
C52
C52
C52
C52
C52
C52
C52
C52
C52
C52
C52
C52
C52

For peer review only

C53
C53
C53
C53
C53
C53
C53
C53
C53
C53
C53
C53
C53
C53
C53
C53
C53
C53
C53
C53
C53
C54
C54
C54
C54
C54
C54
C54
C54
C54
C54
C54
C54
C54
C54
C54
C54
C54
C54
C54
C54
C54
C55
C55
C55
C55
C55
C55
C55
C55

For peer review only

C55
C55
C55
C55
C55
C55
C55
C55
C55
C55
C55
C55
C55
C56
C56
C56
C56
C56
C56
C56
C56
C56
C56
C56
C56
C56
C56
C56
C56
C56
C56
C56
C56
C56
C57
C57
C57
C57
C57
C57
C57
C57
C57
C57
C57
C57
C57
C57
C57
C57

For peer review only

C57
C57
C57
C57
C57
C60
C60
C60
C60
C60
C60
C60
C60
C60
C60
C60
C60
C60
C60
C60
C60
C60
C60
C60
C61
C61
C61
C61
C61
C61
C61
C61
C61
C61
C61
C61
C61
C61
C61
C61
C61
C61
C61
C61
C61
C62
C62
C62
C62
C62

For peer review only

C62
C62
C62
C62
C62
C62
C62
C62
C62
C62
C62
C62
C62
C62
C62
C63
C63
C63
C63
C63
C63
C63
C63
C64
C64
C64
C64
C64
C64
C64
C64
C64
C64
C64
C64
C64
C64
C64
C64
C64
C64
C64
C64
C64
C65
C65
C65
C65
C65
C65

For peer review only

C65
C65
C65
C65
C65
C65
C65
C65
C65
C65
C65
C65
C66
C66
C66
C66
C66
C66
C66
C66
C66
C66
C66
C66
C66
C66
C66
C66
C66
C67
C67
C67
C67
C67
C67
C67
C67
C67
C67
C67
C67
C67
C67
C67
C67
C67
C67
C67
C67
C67

For peer review only

C68
C68
C68
C68
C68
C68
C68
C68
C68
C68
C68
C68
C68
C68
C68
C68
C68
C69
C69
C69
C69
C69
C69
C69
C69
C69
C69
C69
C69
C69
C69
C69
C69
C69
C69
C69
C69
C70
C70
C70
C70
C70
C70
C70
C70
C70
C70
C70
C70
C70

For peer review only

C70
C70
C70
C70
C70
C71
C71
C71
C71
C71
C71
C71
C71
C71
C71
C71
C71
C71
C71
C71
C71
C71
C71
C71
C71
C71
C72
C72
C72
C72
C72
C72
C72
C72
C72
C72
C72
C72
C72
C72
C72
C73
C73
C73
C73
C73
C73
C73
C73
C73

For peer review only

C73
C73
C73
C73
C73
C73
C73
C73
C73
C73
C73
C73
C74
C74
C74
C74
C74
C74
C74
C74
C74
C74
C74
C74
C74
C74
C74
C74
C74
C74
C74
C74
C75
C75
C75
C75
C75
C75
C75
C75
C75
C75
C75
C75
C75
C76
C76
C76
C76
C76

For peer review only

C76
C76
C76
C76
C76
C76
C76
C76
C76
C76
C76
C76
C76
C76
C76
C76
C77
C78
C78
C78
C78
C78
C79
C79
C79
C79
C80
C80
C80
C80
C80
C80
C80
C80
C80
C80
C80
C80
C80
C80
C80
C80
C80
C80
C80
C80
C80
C81
C81
C81

For peer review only

C81
C81
C81
C81
C81
C81
C81
C81
C81
C81
C81
C81
C81
C81
C81
C81
C81
C81
C82
C82
C82
C82
C82
C82
C82
C82
C82
C82
C82
C82
C82
C82
C82
C82
C82
C82
C83
C83
C83
C83
C83
C83
C83
C83
C83
C83
C83
C83
C83
C83

For peer review only

C83
C83
C83
C83
C83
C83
C83
C84
C84
C84
C84
C84
C84
C84
C84
C84
C84
C84
C84
C84
C84
C84
C84
C84
C84
C84
C84
C85
C85
C85
C85
C85
C85
C85
C85
C85
C85
C85
C85
C85
C85
C85
C85
C85
C85
C85
C85
C88
C88

For peer review only

C88
C88
C88
C88
C88
C88
C88
C88
C88
C88
C88
C88
C88
C88
C88
C90
C90
C90
C90
C90
C90
C90
C90
C90
C90
C90
C90
C90
C90
C90
C90
C90
C90
C90
C90
C90
C91
C91
C91
C91
C91
C91
C91
C91
C91
C91
C91
C91
C91
C91

For peer review only

C91
C91
C91
C91
C91
C91
C91
C92
C92
C92
C92
C92
C92
C92
C92
C92
C92
C92
C92
C92
C92
C92
C92
C92
C92
C92
C92
C92
C93
C93
C93
C93
C93
C93
C93
C93
C93
C93
C93
C93
C93
C93
C93
C93
C94
C94
C94
C94
C94
C94

For peer review only

C94
C94
C94
C94
C95
C95
C95
C95
C95
C95
C95
C95
C95
C95
C95
C95
C95
C95
C95
C95
C95
C95
C95
C95
C95
C96
C96
C96
C96
C96
C96
C96
C96
C96
C96
C96
C96
C96
C96
C96
C97
C97
C97
D01
D01
D02
D04
D07
D07
D09

For peer review only

D12
D12
D12
D12
D12
D12
D12
D12
D13
D13
D13
D13
D13
D13
D13
D14
D14
D15
D15
D15
D15
D15
D18
D18
D18
D18
D18
D18
D18
D18
D19
D21
D21
D21
D23
D25
D25
D27
D27
D29
D30
D30
D30
D32
D32
D32
D32
D32
D32
D32

For peer review only

D32
D32
D32
D32
D32
D32
D32
D32
D32
D32
D32
D32
D32
D32
D33
D33
D33
D33
D33
D33
D33
D33
D33
D33
D33
D33
D33
D33
D33
D33
D35
D35
D35
D35
D35
D35
D35
D35
D35
D35
D35
D35
D35
D36
D36
D36
D36
D37
D37
D37

For peer review only

D37
D37
D37
D37
D37
D37
D37
D37
D37
D37
D37
D37
D37
D37
D38
D38
D38
D38
D38
D38
D38
D38
D38
D38
D38
D38
D38
D38
D38
D38
D39
D39
D39
D39
D39
D39
D39
D39
D39
D39
D39
D39
D39
D40
D40
D40
D40
D40
D40
D41

For peer review only

D41
D41
D41
D41
D41
D41
D41
D41
D41
D41
D41
D41
D41
D41
D41
D42
D42
D42
D42
D43
D43
D43
D43
D43
D43
D43
D43
D43
D43
D43
D43
D43
D43
D43
D43
D43
D43
D43
D43
D44
D44
D44
D44
D44
D44
D44
D44
D44
D44
D44

For peer review only

D44
D45
D45
D45
D45
D45
D45
D45
D45
D45
D45
D45
D45
D45
D45
D45
D45
D45
D46
D46
D46
D46
D46
D46
D46
D46
D46
D46
D46
D46
D46
D46
D46
D46
D46
D46
D46
D46
D46
D47
D47
D47
D47
D47
D47
D47
D47
D47
D47
D47

For peer review only

D47
D47
D47
D47
D47
D47
D47
D47
D47
D47
D48
D48
D48
D48
D48
D48
D48
D48
D48
D48
D48
D48
D48
D48
D48
D48
D50
D50
D50
D50
D50
D50
D50
D50
D50
D50
D50
D50
D50
D50
D50
D50
D51
D51
D51
D51
D51
D51
D51
D51

For peer review only

D52
D52
D52
D53
D53
D53
D53
D53
D53
D53
D56
D56
D56
D57
D57
D57
D58
D58
D58
D58
D58
D59
D59
D59
D59
D59
D59
D59
D59
D59
D59
D59
D59
D59
D59
D60
D60
D60
D61
D61
D61
D61
D61
D61
D61
D61
D61
D61
D61
D61

For peer review only

D61
D61
D61
D61
D61
D61
D61
D62
D64
D64
D64
D64
D64
D64
D64
D64
D64
D64
D64
D64
D64
D64
D64
D64
D64
D65
D65
D65
D65
D65
D65
D65
D65
D65
D66
D66
D66
D66
D66
D66
D68
D68
D68
D68
D68
D68
D68
D68
D68
D68
D68

For peer review only

D68
D68
D69
D69
D69
D69
D69
D69
D69
D69
D69
D69
D69
D69
D69
D69
D69
D69
D69
D69
D70
D70
D70
D70
D70
D70
D70
D70
D70
D70
D70
D72
D72
D72
D73
D73
D73
D73
D73
D73
D73
D73
D75
D75
D75
D75
D75
D75
D75
D75

For peer review only

D75
D75
D75
D75
D76
D76
D76
D76
D76
D76
D80
D80
D81
D84
D84
D84
D84
D84
D84
D84
D84
D86
D86
D86
D86
D86
D86
D86
D86
D86
D86
D86
D86
D86
D86
D86
D89
D89
D89
D89
D89
D89
D89
E03
E03
E03
E03
E03
E03
E03

For peer review only

E03
E03
E03
E03
E03
E03
E04
E04
E04
E04
E04
E04
E04
E04
E04
E05
E05
E05
E05
E05
E05
E05
E05
E05
E05
E05
E05
E05
E05
E05
E06
E06
E07
E07
E07
E07
E07
E07
E10
E10
E10
E10
E10
E10
E10
E10
E10
E10
E10
E10

For peer review only

E10
E10
E10
E10
E10
E10
E10
E10
E10
E11
E11
E11
E11
E11
E11
E11
E11
E11
E11
E11
E11
E11
E11
E11
E11
E11
E11
E11
E11
E11
E12
E12
E12
E12
E13
E13
E13
E13
E14
E14
E14
E14
E14
E14
E14
E14
E14
E14
E14
E14

For peer review only

E14
E14
E14
E14
E14
E14
E14
E14
E14
E15
E15
E15
E15
E15
E15
E16
E16
E16
E16
E16
E16
E16
E21
E21
E21
E21
E21
E21
E21
E21
E22
E22
E22
E22
E22
E23
E23
E23
E23
E23
E23
E23
E24
E24
E24
E24
E27
E27
E27
E27

For peer review only

E27
E27
E27
E27
E27
E27
E27
E27
E27
E34
E34
E34
E34
E34
E41
E41
E41
E41
E41
E41
E41
E41
E41
E41
E41
E43
E43
E43
E43
E43
E43
E43
E43
E43
E46
E46
E46
E46
E46
E46
E46
E46
E46
E46
E46
E51
E51
E51
E51
E63

For peer review only

E63
E63
E64
E66
E66
E66
E66
E66
E66
E66
E66
E66
E66
E66
E66
E66
E66
E66
E66
E66
E66
E66
E66
E71
E71
E71
E71
E72
E72
E72
E72
E72
E74
E74
E74
E74
E75
E75
E75
E75
E75
E75
E75
E75
E75
E75
E75
E76
E76
E76

For peer review only

E76
E76
E77
E77
E77
E77
E78
E78
E78
E78
E78
E78
E78
E78
E78
E78
E78
E78
E78
E79
E80
E83
E83
E83
E83
E83
E83
E83
E83
E83
E83
E83
E83
E83
E83
E84
E84
E84
E84
E84
E84
E84
E84
E84
E84
E84
E84
E84
E85
E85

For peer review only

E85
E85
E85
E85
E85
E85
E85
E85
E85
E85
E85
E85
E85
E85
E85
E85
E86
E86
E86
E86
E86
E86
E86
E86
E86
E86
E86
E86
E86
E87
E87
E87
E87
E87
E87
E87
E87
E87
E87
E87
E87
E87
E87
E88
E88
E88
E88
E88
E88
E88

For peer review only

E88
E88
E88
E88
E88
E88
E88
E88
F01
F01
F01
F01
F01
F01
F01
F01
F01
F01
F01
F01
F01
F01
F01
F01
F01
F03
F03
F03
F03
F03
F03
F03
F03
F03
F03
F03
F03
F03
F03
F03
F03
F03
F04
F04
F05
F05
F05
F05
F05
F05

For peer review only

F05
F05
F05
F06
F06
F06
F06
F06
F07
F07
F07
F07
F07
F07
F07
F07
F10
F10
F10
F10
F10
F10
F10
F10
F10
F10
F10
F10
F10
F10
F10
F10
F10
F10
F10
F10
F10
F11
F11
F11
F11
F11
F11
F11
F11
F11
F11
F11
F13
F14

For peer review only

F14
F15
F17
F17
F17
F17
F17
F17
F18
F19
F19
F19
F19
F19
F19
F19
F19
F19
F19
F19
F19
F20
F20
F20
F20
F20
F20
F20
F20
F20
F20
F20
F20
F20
F20
F20
F20
F20
F22
F22
F22
F22
F22
F22
F22
F22
F23
F25
F25
F28

For peer review only

F29
F29
F29
F29
F29
F29
F29
F29
F31
F31
F31
F31
F31
F31
F31
F31
F31
F31
F32
F32
F32
F32
F32
F32
F32
F32
F32
F32
F32
F32
F33
F33
F33
F33
F33
F33
F33
F34
F41
F41
F41
F41
F42
F43
F44
F44
F44
F48
F50
F50

For peer review only

F50
F50
F50
F50
F50
F50
F50
F50
F54
F54
F55
F55
F60
F71
F72
F72
F73
F73
F73
F73
F79
F79
F79
F79
F79
F79
F79
F79
F79
F81
F84
F84
F84
F99
F99
F99
F99
F99
G00
G00
G00
G00
G00
G00
G00
G00
G00
G00
G00
G00

For peer review only

G00
G00
G00
G00
G00
G00
G00
G00
G00
G03
G03
G03
G03
G03
G03
G03
G03
G03
G03
G03
G03
G03
G03
G03
G04
G04
G04
G04
G04
G04
G04
G04
G04
G04
G04
G04
G04
G04
G04
G04
G04
G04
G04
G06
G06
G06
G06
G06
G06
G06

For peer review only

G06
G06
G06
G06
G06
G06
G08
G08
G08
G09
G09
G09
G09
G09
G09
G09
G09
G10
G10
G10
G10
G10
G10
G10
G10
G10
G10
G10
G10
G10
G10
G10
G10
G10
G10
G11
G11
G11
G11
G11
G11
G11
G11
G11
G11
G11
G11
G11
G12
G12

For peer review only

G12
G12
G12
G12
G12
G12
G12
G12
G12
G12
G12
G12
G12
G12
G12
G12
G12
G12
G12
G20
G20
G20
G20
G20
G20
G20
G20
G20
G20
G20
G20
G20
G20
G20
G20
G20
G20
G20
G20
G20
G21
G21
G21
G21
G21
G21
G21
G21
G21
G21

For peer review only

G21
G23
G23
G23
G23
G23
G23
G23
G23
G23
G23
G23
G23
G23
G23
G24
G24
G24
G25
G25
G25
G25
G25
G25
G25
G25
G30
G30
G30
G30
G30
G30
G30
G30
G30
G30
G30
G30
G30
G30
G30
G30
G30
G30
G30
G30
G30
G31
G31
G31

For peer review only

G31
G31
G31
G31
G31
G31
G31
G31
G31
G31
G31
G31
G31
G31
G31
G31
G31
G31
G35
G35
G35
G35
G35
G35
G35
G35
G35
G35
G35
G35
G35
G35
G35
G35
G35
G35
G35
G35
G35
G36
G37
G37
G37
G37
G37
G37
G37
G37
G37
G37

For peer review only

G40
G40
G40
G40
G40
G40
G40
G40
G40
G40
G40
G40
G40
G40
G40
G40
G40
G40
G40
G40
G40
G41
G41
G41
G41
G41
G41
G41
G41
G41
G41
G41
G41
G41
G41
G41
G41
G41
G41
G41
G45
G45
G45
G45
G45
G45
G45
G45
G45
G45

For peer review only

G45
G47
G47
G47
G47
G47
G47
G47
G52
G58
G60
G60
G60
G60
G60
G60
G60
G61
G61
G61
G61
G61
G61
G61
G61
G61
G61
G61
G61
G62
G62
G62
G62
G62
G62
G62
G62
G62
G62
G62
G62
G62
G62
G62
G62
G70
G70
G70
G70
G70

For peer review only

G70
G70
G70
G70
G70
G70
G70
G70
G70
G70
G70
G70
G70
G71
G71
G71
G71
G71
G71
G71
G71
G71
G71
G71
G71
G71
G71
G71
G71
G71
G71
G72
G72
G72
G72
G72
G72
G72
G80
G80
G80
G80
G80
G80
G80
G80
G80
G80
G80
G80

For peer review only

G80
G80
G80
G80
G80
G80
G80
G80
G81
G81
G81
G81
G81
G81
G81
G81
G82
G82
G82
G82
G82
G82
G82
G82
G82
G82
G82
G82
G82
G82
G83
G83
G83
G83
G83
G83
G83
G83
G90
G90
G90
G90
G90
G90
G90
G90
G90
G90
G90
G91

For peer review only

G91
G91
G91
G91
G91
G91
G91
G91
G91
G91
G91
G91
G91
G91
G91
G91
G91
G92
G92
G92
G92
G92
G92
G92
G92
G93
G93
G93
G93
G93
G93
G93
G93
G93
G93
G93
G93
G93
G93
G93
G93
G93
G93
G93
G93
G95
G95
G95
G95
G95

For peer review only

G95
G95
G95
G95
G95
G95
G95
G95
G96
G96
G96
G96
G96
G96
G98
G98
G98
G98
G98
G98
G98
G98
G98
G98
H05
H60
H66
H66
H66
H66
H66
H70
I00
I01
I01
I05
I05
I05
I05
I05
I05
I05
I05
I05
I05
I05
I05
I05
I05
I05

For peer review only

I05
I05
I05
I05
I05
I05
I06
I06
I06
I06
I06
I06
I06
I06
I06
I06
I06
I06
I06
I06
I07
I07
I07
I07
I07
I07
I07
I07
I07
I07
I08
I08
I08
I08
I08
I08
I08
I08
I08
I08
I08
I08
I08
I08
I08
I08
I08
I08
I08
I08

For peer review only

I08
I09
I09
I09
I09
I09
I09
I09
I09
I09
I09
I09
I09
I10
I10
I10
I10
I10
I10
I10
I10
I10
I10
I10
I10
I10
I10
I10
I10
I10
I10
I10
I10
I11
I11
I11
I11
I11
I11
I11
I11
I11
I11
I11
I11
I11
I11
I11
I11
I11

For peer review only

I11
I11
I11
I11
I12
I12
I12
I12
I12
I12
I12
I12
I12
I12
I12
I12
I12
I12
I12
I12
I12
I12
I12
I12
I13
I13
I13
I13
I13
I13
I13
I13
I13
I13
I13
I13
I13
I13
I13
I13
I13
I13
I13
I13
I20
I20
I20
I20
I20
I20

For peer review only

For peer review only - http://bmjopen.bmj.com/site/about/guidelines.xhtml

I20
I20
I20
I20
I20
I20
I20
I20
I20
I20
I20
I20
I20
I20
I21
I21
I21
I21
I21
I21
I21
I21
I21
I21
I21
I21
I21
I21
I21
I21
I21
I21
I21
I21
I21
I22
I22
I22
I22
I22
I22
I22
I22
I22
I22
I22
I22
I22
I22
I22

For peer review only

I22
I24
I24
I24
I24
I24
I24
I24
I24
I24
I24
I24
I24
I24
I24
I24
I24
I24
I24
I24
I24
I25
I25
I25
I25
I25
I25
I25
I25
I25
I25
I25
I25
I25
I25
I25
I25
I25
I25
I25
I25
I25
I26
I26
I26
I26
I26
I26
I26
I26

For peer review only

I26
I26
I26
I26
I26
I26
I26
I26
I26
I26
I26
I26
I26
I27
I27
I27
I27
I27
I27
I27
I27
I27
I27
I27
I27
I27
I27
I27
I27
I27
I27
I27
I27
I27
I28
I28
I28
I28
I28
I28
I28
I30
I30
I30
I30
I30
I30
I30
I30
I30

For peer review only

I30
I30
I30
I31
I31
I31
I31
I31
I31
I31
I31
I31
I31
I31
I31
I31
I31
I31
I31
I31
I33
I33
I33
I33
I33
I33
I33
I33
I33
I33
I33
I33
I33
I33
I33
I33
I33
I33
I33
I33
I33
I34
I34
I34
I34
I34
I34
I34
I34
I34

For peer review only

I34
I34
I34
I34
I34
I34
I34
I34
I34
I34
I34
I34
I35
I35
I35
I35
I35
I35
I35
I35
I35
I35
I35
I35
I35
I35
I35
I35
I35
I35
I35
I35
I35
I36
I36
I36
I36
I36
I36
I36
I36
I36
I37
I37
I38
I38
I38
I38
I38
I38

For peer review only

I38
I38
I38
I38
I38
I38
I38
I38
I38
I38
I40
I40
I40
I40
I40
I40
I40
I40
I40
I40
I40
I40
I40
I42
I42
I42
I42
I42
I42
I42
I42
I42
I42
I42
I42
I42
I42
I42
I42
I42
I42
I42
I42
I42
I44
I44
I44
I44
I44
I44

For peer review only

I44
I44
I44
I44
I44
I44
I44
I44
I44
I44
I44
I45
I45
I45
I45
I45
I45
I45
I45
I45
I45
I45
I45
I45
I45
I46
I46
I46
I46
I46
I46
I46
I46
I46
I46
I46
I46
I46
I46
I46
I46
I46
I46
I46
I46
I47
I47
I47
I47
I47

For peer review only

I47
I47
I47
I47
I47
I47
I47
I47
I47
I47
I48
I48
I48
I48
I48
I48
I48
I48
I48
I48
I48
I48
I48
I48
I48
I48
I48
I48
I48
I48
I49
I49
I49
I49
I49
I49
I49
I49
I49
I49
I49
I49
I49
I49
I49
I49
I49
I49
I49
I50

For peer review only

I50
I50
I50
I50
I50
I50
I50
I50
I50
I50
I50
I50
I50
I50
I50
I50
I50
I50
I50
I51
I51
I51
I51
I51
I51
I51
I51
I51
I51
I51
I51
I51
I51
I51
I51
I51
I51
I51
I51
I51
I60
I60
I60
I60
I60
I60
I60
I60
I60
I60

For peer review only

I60
I60
I60
I60
I60
I60
I60
I60
I60
I60
I60
I61
I61
I61
I61
I61
I61
I61
I61
I61
I61
I61
I61
I61
I61
I61
I61
I61
I61
I61
I61
I61
I62
I62
I62
I62
I62
I62
I62
I62
I62
I62
I62
I62
I62
I62
I62
I62
I62
I62

For peer review only

I62
I62
I62
I63
I63
I63
I63
I63
I63
I63
I63
I63
I63
I63
I63
I63
I63
I63
I63
I63
I63
I63
I63
I63
I64
I64
I64
I64
I64
I64
I64
I64
I64
I64
I64
I64
I64
I64
I64
I64
I64
I64
I64
I64
I64
I67
I67
I67
I67
I67

For peer review only

I67
I67
I67
I67
I67
I67
I67
I67
I67
I67
I67
I67
I67
I67
I67
I67
I69
I69
I69
I69
I69
I69
I69
I69
I69
I69
I69
I69
I69
I69
I69
I69
I69
I69
I69
I69
I70
I70
I70
I70
I70
I70
I70
I70
I70
I70
I70
I70
I70
I70

For peer review only

I70
I70
I70
I70
I70
I70
I70
I71
I71
I71
I71
I71
I71
I71
I71
I71
I71
I71
I71
I71
I71
I71
I71
I71
I71
I71
I71
I71
I72
I72
I72
I72
I72
I72
I72
I72
I72
I72
I72
I72
I72
I72
I72
I72
I72
I72
I72
I73
I73
I73

For peer review only

For peer review only - http://bmjopen.bmj.com/site/about/guidelines.xhtml

I73
I73
I73
I73
I73
I73
I73
I73
I73
I73
I73
I73
I73
I74
I74
I74
I74
I74
I74
I74
I74
I74
I74
I74
I74
I74
I74
I74
I74
I74
I74
I74
I74
I74
I77
I77
I77
I77
I77
I77
I77
I77
I77
I77
I77
I77
I77
I77
I77
I77

For peer review only

I78
I78
I78
I78
I78
I80
I80
I80
I80
I80
I80
I80
I80
I80
I80
I80
I80
I80
I80
I80
I80
I80
I80
I80
I80
I80
I81
I81
I81
I81
I81
I81
I81
I81
I81
I81
I82
I82
I82
I82
I82
I82
I82
I82
I82
I82
I82
I82
I82
I82

For peer review only

I82
I82
I83
I83
I83
I83
I83
I83
I83
I83
I83
I83
I83
I83
I83
I83
I83
I83
I83
I83
I84
I85
I85
I85
I85
I85
I85
I85
I85
I85
I85
I85
I85
I85
I85
I86
I86
I87
I87
I87
I87
I87
I87
I87
I87
I87
I87
I89
I89
I89

For peer review only

I89
I95
I95
I95
I95
I95
I95
I95
I99
I99
I99
I99
I99
I99
I99
I99
I99
I99
I99
I99
J00
J02
J02
J03
J04
J04
J04
J04
J05
J06
J06
J06
J06
J06
J06
J06
J06
J06
J06
J10
J10
J10
J10
J10
J10
J10
J11
J11
J11
J11

For peer review only

J11
J11
J11
J11
J11
J11
J11
J11
J11
J11
J11
J11
J12
J12
J12
J12
J12
J12
J12
J12
J12
J12
J12
J12
J12
J12
J12
J13
J13
J13
J13
J13
J13
J13
J13
J13
J13
J13
J13
J13
J13
J14
J15
J15
J15
J15
J15
J15
J15
J15

For peer review only

J15
J15
J15
J15
J15
J15
J15
J15
J15
J15
J15
J15
J16
J16
J16
J16
J18
J18
J18
J18
J18
J18
J18
J18
J18
J18
J18
J18
J18
J18
J18
J18
J18
J18
J18
J18
J18
J20
J20
J20
J20
J20
J20
J20
J20
J20
J20
J20
J20
J20

J20
J20
J20
J20
J21
J21
J21
J21
J21
J21
J21
J21
J22
J22
J22
J22
J22
J22
J22
J22
J22
J22
J22
J22
J22
J22
J22
J22
J32
J32
J32
J38
J38
J38
J38
J38
J38
J39
J39
J39
J39
J39
J39
J39
J39
J39
J40
J40
J40
J40

For peer review only

J40
J40
J40
J40
J40
J40
J40
J40
J40
J40
J41
J41
J41
J41
J41
J41
J41
J41
J41
J41
J42
J42
J42
J42
J42
J42
J42
J42
J42
J42
J42
J42
J42
J42
J42
J42
J42
J42
J42
J42
J43
J43
J43
J43
J43
J43
J43
J43
J43
J43

For peer review only

J43
J43
J43
J43
J43
J43
J43
J43
J43
J43
J44
J44
J44
J44
J44
J44
J44
J44
J44
J44
J44
J44
J44
J44
J44
J44
J44
J44
J44
J44
J44
J45
J45
J45
J45
J45
J45
J45
J45
J45
J45
J45
J45
J45
J45
J45
J45
J45
J45
J45

For peer review only

J45
J45
J46
J46
J46
J46
J46
J46
J46
J46
J46
J46
J46
J46
J46
J46
J46
J46
J47
J47
J47
J47
J47
J47
J47
J47
J47
J47
J47
J47
J47
J47
J47
J47
J60
J60
J60
J60
J60
J60
J60
J61
J61
J61
J61
J61
J61
J61
J61
J61

For peer review only

J61
J61
J62
J62
J62
J62
J62
J62
J62
J62
J62
J62
J62
J62
J62
J63
J63
J64
J64
J64
J64
J64
J64
J64
J64
J65
J65
J65
J67
J67
J67
J67
J67
J67
J67
J67
J67
J67
J67
J68
J69
J69
J69
J69
J69
J69
J69
J69
J69
J69

For peer review only

J69
J69
J69
J69
J69
J69
J69
J70
J70
J70
J70
J70
J70
J80
J80
J80
J80
J80
J80
J80
J80
J80
J81
J81
J81
J81
J81
J81
J81
J81
J81
J81
J81
J81
J81
J81
J81
J82
J82
J82
J84
J84
J84
J84
J84
J84
J84
J84
J84
J84

For peer review only

J84
J84
J84
J84
J84
J84
J84
J84
J84
J84
J84
J85
J85
J85
J85
J85
J85
J85
J85
J85
J85
J85
J85
J85
J85
J85
J85
J85
J85
J85
J86
J86
J86
J86
J86
J86
J86
J86
J86
J86
J86
J86
J86
J86
J86
J86
J86
J86
J86
J86

For peer review only

For peer review only - http://bmjopen.bmj.com/site/about/guidelines.xhtml

J90
J90
J90
J90
J90
J90
J90
J90
J90
J90
J90
J90
J90
J90
J92
J92
J92
J93
J93
J93
J93
J93
J93
J93
J93
J93
J93
J93
J93
J94
J94
J94
J94
J94
J94
J94
J94
J94
J94
J94
J94
J96
J96
J96
J96
J96
J96
J96
J96
J96

For peer review only

J96
J96
J96
J96
J96
J96
J96
J96
J98
J98
J98
J98
J98
J98
J98
J98
J98
J98
J98
J98
J98
J98
J98
J98
K04
K10
K11
K11
K11
K12
K12
K12
K13
K20
K20
K20
K20
K20
K20
K20
K20
K20
K20
K21
K21
K21
K21
K21
K21
K21
K21

For peer review only

K21
K21
K22
K22
K22
K22
K22
K22
K22
K22
K22
K22
K22
K22
K22
K22
K22
K22
K22
K22
K22
K22
K22
K25
K25
K25
K25
K25
K25
K25
K25
K25
K25
K25
K25
K25
K25
K25
K25
K25
K25
K25
K25
K25
K26
K26
K26
K26
K26
K26

For peer review only

K26
K26
K26
K26
K26
K26
K26
K26
K26
K26
K26
K26
K26
K26
K26
K27
K27
K27
K27
K27
K27
K27
K27
K27
K27
K27
K27
K27
K27
K27
K27
K28
K28
K28
K28
K28
K28
K28
K28
K29
K29
K29
K29
K29
K29
K29
K29
K29
K29
K29

For peer review only

K29
K29
K29
K29
K29
K29
K29
K29
K29
K30
K31
K31
K31
K31
K31
K31
K31
K31
K31
K31
K31
K31
K31
K31
K31
K35
K35
K35
K35
K35
K35
K35
K35
K35
K35
K35
K35
K35
K35
K35
K35
K35
K35
K37
K37
K37
K37
K37
K38
K38

For peer review only

K40
K40
K40
K40
K40
K40
K40
K40
K40
K40
K40
K40
K40
K40
K40
K40
K40
K40
K40
K40
K41
K41
K41
K41
K41
K41
K41
K41
K41
K41
K41
K41
K41
K41
K41
K42
K42
K42
K42
K42
K42
K42
K42
K42
K42
K42
K42
K42
K42
K42

For peer review only

K42
K42
K43
K43
K43
K43
K43
K43
K43
K43
K43
K43
K43
K43
K43
K43
K43
K43
K43
K43
K43
K44
K44
K44
K44
K44
K44
K44
K44
K44
K44
K44
K44
K44
K44
K44
K45
K45
K45
K45
K45
K45
K45
K45
K46
K46
K46
K46
K46

For peer review only

K46
K46
K46
K46
K46
K46
K46
K46
K46
K50
K50
K50
K50
K50
K50
K50
K50
K50
K50
K50
K50
K50
K50
K50
K50
K50
K51
K51
K51
K51
K51
K51
K51
K51
K51
K51
K51
K51
K51
K51
K51
K51
K51
K51
K51
K51
K51
K52
K52
K52

For peer review only

K52
K52
K52
K52
K52
K52
K52
K52
K52
K52
K52
K52
K52
K55
K55
K55
K55
K55
K55
K55
K55
K55
K55
K55
K55
K55
K55
K55
K55
K55
K55
K55
K55
K55
K56
K56
K56
K56
K56
K56
K56
K56
K56
K56
K56
K56
K56
K56
K56
K56

For peer review only

K56
K56
K56
K56
K56
K57
K57
K57
K57
K57
K57
K57
K57
K57
K57
K57
K57
K57
K57
K57
K57
K57
K57
K57
K57
K57
K58
K59
K59
K59
K59
K59
K59
K59
K59
K59
K59
K59
K59
K59
K60
K60
K61
K61
K61
K61
K61
K61
K61
K61

For peer review only

K61
K62
K62
K62
K62
K62
K62
K62
K62
K62
K62
K62
K62
K62
K63
K63
K63
K63
K63
K63
K63
K63
K63
K63
K63
K63
K63
K63
K63
K63
K63
K63
K63
K63
K63
K65
K65
K65
K65
K65
K65
K65
K65
K65
K65
K65
K65
K65
K65
K65

For peer review only

K65
K65
K65
K65
K65
K66
K66
K66
K66
K66
K66
K66
K66
K66
K66
K66
K66
K70
K70
K70
K70
K70
K70
K70
K70
K70
K70
K70
K70
K70
K70
K70
K70
K70
K70
K70
K70
K70
K71
K71
K71
K71
K71
K71
K71
K71
K71
K71
K71
K71

For peer review only

K71
K71
K71
K72
K72
K72
K72
K72
K72
K72
K72
K72
K72
K72
K72
K72
K72
K72
K72
K72
K72
K73
K73
K73
K73
K73
K73
K73
K73
K73
K73
K73
K74
K74
K74
K74
K74
K74
K74
K74
K74
K74
K74
K74
K74
K74
K74
K74
K74
K74

For peer review only

K74
K74
K74
K75
K75
K75
K75
K75
K75
K75
K75
K75
K75
K75
K75
K75
K75
K75
K75
K75
K75
K76
K76
K76
K76
K76
K76
K76
K76
K76
K76
K76
K76
K76
K76
K76
K76
K76
K76
K76
K76
K76
K80
K80
K80
K80
K80
K80
K80
K80

For peer review only

K80
K80
K80
K80
K80
K80
K80
K80
K80
K80
K80
K80
K80
K81
K81
K81
K81
K81
K81
K81
K81
K81
K81
K81
K81
K81
K81
K81
K81
K81
K81
K81
K81
K81
K82
K82
K82
K82
K82
K82
K82
K82
K82
K82
K82
K82
K82
K82
K82
K82

For peer review only

K83
K83
K83
K83
K83
K83
K83
K83
K83
K83
K83
K83
K83
K83
K83
K83
K83
K83
K83
K85
K85
K85
K85
K85
K85
K85
K85
K85
K85
K85
K85
K85
K85
K85
K85
K85
K85
K85
K85
K85
K86
K86
K86
K86
K86
K86
K86
K86
K86
K86

For peer review only

K86
K86
K86
K86
K86
K86
K86
K86
K86
K86
K86
K90
K90
K90
K90
K90
K90
K90
K90
K92
K92
K92
K92
K92
K92
K92
K92
K92
K92
K92
K92
K92
K92
K92
K92
K92
K92
K92
K92
K92
L02
L02
L02
L02
L02
L02
L02
L02
L02
L02

For peer review only

L02
L02
L02
L02
L03
L03
L03
L03
L03
L03
L03
L03
L03
L03
L03
L03
L03
L08
L08
L08
L08
L08
L08
L08
L08
L08
L08
L10
L10
L10
L10
L10
L12
L12
L12
L12
L12
L12
L12
L13
L30
L40
L40
L40
L40
L51
L51
L51
L51
L53

For peer review only

L53
L88
L88
L88
L89
L89
L89
L89
L89
L89
L89
L89
L89
L89
L89
L89
L89
L89
L89
L89
L89
L93
L93
L93
L93
L93
L93
L97
L97
L97
L97
L97
L97
L97
L97
L97
L97
L97
L98
L98
L98
L98
L98
L98
L98
L98
L98
M00
M00
M00

For peer review only

M00
M00
M00
M00
M00
M00
M00
M00
M00
M00
M00
M00
M05
M05
M05
M05
M05
M05
M05
M05
M05
M05
M05
M05
M05
M05
M05
M05
M05
M06
M06
M06
M06
M06
M06
M06
M06
M06
M06
M06
M06
M06
M06
M06
M06
M06
M06
M06
M06
M10

For peer review only

M10
M10
M10
M10
M10
M10
M11
M13
M13
M13
M13
M13
M13
M13
M13
M13
M13
M13
M13
M15
M15
M15
M15
M15
M15
M15
M15
M15
M15
M15
M15
M16
M16
M16
M16
M16
M16
M16
M16
M16
M16
M16
M16
M17
M17
M17
M17
M17
M17
M17

For peer review only

M17
M17
M17
M17
M19
M19
M19
M19
M19
M19
M19
M19
M24
M24
M25
M25
M25
M25
M25
M25
M30
M30
M30
M30
M30
M30
M31
M31
M31
M31
M31
M31
M31
M31
M31
M31
M31
M31
M31
M31
M31
M31
M31
M31
M31
M32
M32
M32
M32
M32
M32

For peer review only

M32
M32
M32
M32
M32
M32
M32
M32
M32
M32
M32
M32
M32
M32
M33
M33
M33
M33
M33
M33
M33
M33
M33
M33
M33
M34
M34
M34
M34
M34
M34
M34
M34
M34
M34
M34
M34
M34
M34
M34
M34
M34
M35
M35
M35
M35
M35
M35
M35
M35

For peer review only

M35
M35
M35
M35
M35
M35
M35
M35
M40
M40
M40
M41
M41
M41
M41
M41
M41
M41
M41
M41
M43
M45
M45
M45
M45
M45
M45
M45
M46
M46
M46
M46
M46
M46
M46
M46
M46
M46
M46
M46
M46
M47
M47
M47
M47
M47
M47
M48
M48
M48

For peer review only

M48
M48
M48
M48
M48
M48
M48
M48
M50
M50
M51
M51
M51
M51
M54
M54
M54
M54
M60
M60
M60
M60
M62
M62
M62
M62
M62
M62
M62
M62
M62
M62
M62
M62
M70
M70
M72
M72
M72
M72
M72
M72
M72
M72
M72
M79
M79
M79
M79
M79

For peer review only

M80
M80
M80
M80
M80
M80
M80
M80
M80
M80
M80
M80
M80
M80
M80
M80
M81
M81
M81
M81
M81
M81
M81
M81
M81
M81
M81
M81
M84
M84
M84
M84
M84
M84
M86
M86
M86
M86
M86
M86
M86
M86
M86
M86
M86
M86
M86
M86
M86
M86

For peer review only

M86
M87
M87
M87
M87
M88
M88
M88
M88
M89
M89
M89
M89
M95
N00
N00
N00
N00
N00
N01
N02
N02
N02
N03
N03
N03
N03
N03
N03
N03
N03
N03
N03
N03
N03
N03
N03
N03
N03
N03
N03
N04
N04
N04
N04
N04
N04
N04
N04
N04

For peer review only

N04
N04
N04
N05
N05
N05
N05
N05
N05
N05
N05
N05
N05
N05
N05
N05
N05
N10
N10
N10
N10
N10
N10
N10
N10
N10
N10
N10
N10
N10
N10
N10
N10
N10
N10
N11
N11
N11
N11
N11
N11
N11
N11
N11
N11
N11
N11
N11
N11
N11

For peer review only

N11
N11
N11
N11
N11
N12
N12
N12
N12
N12
N12
N12
N12
N12
N12
N12
N12
N12
N12
N12
N12
N12
N13
N13
N13
N13
N13
N13
N13
N13
N13
N13
N13
N13
N13
N13
N13
N13
N13
N13
N13
N14
N14
N14
N14
N14
N14
N15
N15
N15

For peer review only

N15
N15
N15
N15
N15
N15
N15
N15
N17
N17
N17
N17
N17
N17
N17
N17
N17
N17
N17
N17
N17
N17
N17
N17
N17
N17
N18
N18
N18
N18
N18
N18
N18
N18
N18
N18
N18
N18
N18
N18
N18
N18
N18
N18
N18
N18
N18
N19
N19
N19

For peer review only

N19
N19
N19
N19
N19
N19
N19
N19
N19
N19
N19
N19
N19
N19
N19
N19
N20
N20
N20
N20
N20
N20
N20
N20
N20
N20
N20
N20
N20
N20
N20
N20
N20
N20
N20
N21
N21
N21
N23
N25
N26
N26
N26
N26
N26
N26
N26
N26
N26
N26

For peer review only

N26
N26
N26
N27
N28
N28
N28
N28
N28
N28
N28
N28
N28
N28
N28
N28
N28
N28
N28
N28
N28
N28
N30
N30
N30
N30
N30
N30
N30
N30
N30
N30
N30
N30
N30
N30
N30
N30
N30
N31
N31
N32
N32
N32
N32
N32
N32
N32
N32
N32

For peer review only

N32
N32
N34
N35
N35
N36
N39
N39
N39
N39
N39
N39
N39
N39
N39
N39
N39
N39
N39
N39
N39
N39
N39
N39
N39
N39
N40
N40
N40
N40
N40
N40
N40
N40
N40
N40
N40
N40
N40
N40
N40
N40
N40
N40
N40
N40
N41
N41
N41
N41

For peer review only

N41
N41
N41
N42
N42
N42
N42
N42
N42
N42
N45
N45
N45
N49
N49
N49
N49
N49
N49
N49
N70
N70
N70
N71
N71
N71
N71
N73
N73
N73
N76
N76
N81
N81
N81
N81
N82
N82
N82
N82
N82
N83
N83
N85
N85
N93
N93
N95
N95
O06

For peer review only

O72
O88
O88
O99
O99
P00
P00
P00
P00
P00
P01
P01
P01
P01
P01
P01
P01
P01
P01
P01
P01
P01
P02
P02
P02
P02
P02
P02
P02
P02
P02
P02
P02
P02
P02
P02
P03
P03
P03
P05
P05
P05
P05
P07
P07
P07
P07
P07
P07
P07

For peer review only

P07
P07
P07
P07
P07
P07
P07
P07
P07
P07
P07
P10
P10
P20
P20
P20
P20
P20
P20
P20
P20
P21
P21
P21
P21
P21
P21
P21
P21
P21
P21
P21
P21
P21
P21
P21
P21
P21
P21
P22
P22
P22
P22
P22
P22
P22
P22
P22
P22
P22

For peer review only

P22
P22
P22
P22
P22
P22
P23
P23
P23
P23
P23
P23
P24
P24
P24
P24
P24
P24
P25
P25
P25
P25
P26
P26
P26
P26
P26
P26
P27
P27
P27
P27
P27
P27
P27
P28
P28
P28
P28
P28
P28
P28
P28
P28
P28
P28
P28
P29
P29
P29

For peer review only

P29
P29
P29
P29
P29
P35
P35
P36
P36
P36
P36
P36
P36
P36
P36
P36
P36
P36
P36
P36
P36
P37
P37
P37
P39
P39
P39
P39
P39
P39
P52
P52
P52
P52
P52
P52
P52
P52
P52
P52
P52
P52
P52
P52
P52
P53
P54
P55
P60
P60

For peer review only

P61
P76
P77
P77
P77
P77
P77
P77
P77
P77
P77
P77
P77
P77
P78
P78
P78
P78
P83
P83
P83
P83
P83
P91
P91
P91
P91
P91
P96
P96
P96
P96
P96
Q00
Q00
Q00
Q00
Q00
Q00
Q01
Q02
Q02
Q02
Q03
Q03
Q03
Q03
Q03
Q03
Q03

For peer review only

Q03
Q03
Q03
Q04
Q04
Q04
Q04
Q04
Q04
Q04
Q04
Q04
Q04
Q04
Q04
Q04
Q04
Q05
Q05
Q05
Q05
Q05
Q05
Q05
Q07
Q07
Q07
Q07
Q07
Q07
Q20
Q20
Q20
Q20
Q20
Q20
Q20
Q20
Q20
Q20
Q20
Q20
Q20
Q20
Q21
Q21
Q21
Q21
Q21
Q21

For peer review only

Q21
Q21
Q21
Q21
Q21
Q21
Q21
Q21
Q21
Q21
Q21
Q21
Q21
Q21
Q22
Q22
Q22
Q22
Q22
Q22
Q23
Q23
Q23
Q23
Q23
Q23
Q23
Q23
Q23
Q23
Q23
Q23
Q23
Q23
Q23
Q23
Q23
Q23
Q24
Q24
Q24
Q24
Q24
Q24
Q24
Q24
Q24
Q24
Q24
Q24

For peer review only

Q24
Q24
Q24
Q24
Q24
Q24
Q25
Q25
Q25
Q25
Q25
Q25
Q25
Q25
Q25
Q25
Q25
Q25
Q25
Q25
Q26
Q26
Q26
Q26
Q27
Q27
Q27
Q28
Q28
Q28
Q28
Q28
Q28
Q28
Q28
Q28
Q32
Q32
Q32
Q33
Q33
Q33
Q33
Q33
Q33
Q33
Q33
Q33
Q33
Q38

For peer review only

Q39
Q39
Q39
Q39
Q39
Q39
Q41
Q41
Q43
Q43
Q43
Q43
Q43
Q43
Q43
Q43
Q44
Q44
Q44
Q44
Q44
Q60
Q60
Q60
Q60
Q60
Q60
Q61
Q61
Q61
Q61
Q61
Q61
Q61
Q61
Q61
Q61
Q61
Q61
Q61
Q61
Q61
Q61
Q61
Q63
Q63
Q67
Q76
Q77
Q77

For peer review only

For peer review only

Q77
Q78
Q78
Q78
Q78
Q79
Q79
Q79
Q79
Q79
Q79
Q79
Q79
Q79
Q79
Q79
Q79
Q79
Q81
Q81
Q82
Q85
Q85
Q85
Q85
Q85
Q85
Q85
Q85
Q85
Q87
Q87
Q87
Q87
Q87
Q87
Q87
Q87
Q87
Q87
Q87
Q87
Q87
Q87
Q87
Q89
Q89
Q89
Q89
Q89

Q89
Q89
Q89
Q89
Q89
Q89
Q89
Q89
Q89
Q89
Q89
Q90
Q90
Q90
Q90
Q90
Q90
Q90
Q90
Q90
Q90
Q90
Q90
Q90
Q90
Q90
Q90
Q90
Q90
Q90
Q90
Q91
Q91
Q91
Q91
Q91
Q91
Q91
Q91
Q91
Q91
Q91
Q91
Q91
Q91
Q91
Q91
Q92
Q92
Q93

For peer review only

Q93
Q93
Q99
Q99
Q99
Q99
Q99
Q99
R00
R00
R00
R00
R00
R00
R02
R02
R02
R02
R02
R02
R02
R04
R04
R04
R04
R04
R04
R04
R06
R06
R06
R06
R06
R06
R07
R09
R09
R09
R09
R09
R09
R09
R09
R09
R09
R09
R09
R09
R09
R10

For peer review only

R10
R10
R10
R10
R10
R10
R10
R10
R11
R11
R11
R13
R13
R13
R13
R17
R17
R17
R18
R18
R19
R19
R19
R19
R19
R23
R26
R31
R31
R31
R33
R34
R40
R40
R40
R40
R40
R40
R41
R41
R50
R50
R50
R50
R50
R50
R51
R52
R52
R52

For peer review only

R53
R53
R53
R53
R53
R53
R53
R53
R53
R54
R54
R54
R54
R54
R54
R54
R54
R54
R54
R54
R54
R54
R54
R54
R54
R54
R54
R54
R54
R55
R55
R55
R55
R55
R56
R56
R56
R56
R57
R57
R57
R57
R57
R57
R57
R57
R57
R57
R57
R57

For peer review only

R58
R58
R58
R58
R58
R58
R58
R60
R60
R60
R63
R63
R63
R63
R63
R63
R63
R64
R64
R64
R64
R64
R64
R64
R64
R64
R64
R64
R64
R64
R64
R68
R68
R68
R68
R68
R68
R68
R68
R73
R73
R91
R95
R95
R95
R95
R95
R95
R95
R95

For peer review only

R95
R95
R95
R95
R95
R95
R95
R95
R95
R95
R95
R96
R96
R96
R96
R96
R96
R96
R96
R96
R96
R96
R96
R96
R96
R96
R96
R96
R98
R98
R98
R98
R98
R98
R98
R98
R98
R98
R98
R98
R98
R98
R98
R98
R98
R99
R99
R99
R99
R99

For peer review only

R99
R99
R99
R99
R99
R99
R99
R99
R99
R99
R99
R99
R99
R99
R99
R99
V01
V01
V01
V02
V02
V02
V02
V02
V02
V02
V03
V03
V03
V03
V03
V03
V03
V03
V03
V03
V03
V03
V03
V03
V03
V03
V03
V03
V03
V03
V03
V04
V04
V04

For peer review only

V04
V04
V04
V04
V04
V04
V04
V04
V04
V04
V04
V04
V04
V04
V04
V04
V04
V05
V05
V05
V05
V05
V05
V05
V05
V05
V05
V05
V05
V05
V05
V05
V05
V05
V05
V05
V05
V06
V06
V09
V09
V09
V09
V09
V09
V09
V09
V09
V09
V09

For peer review only

V09
V09
V09
V09
V09
V09
V11
V12
V12
V13
V13
V13
V13
V13
V13
V13
V13
V13
V13
V13
V13
V13
V13
V13
V13
V13
V13
V13
V13
V14
V14
V14
V14
V14
V14
V14
V14
V14
V14
V14
V15
V17
V17
V18
V18
V18
V18
V18
V18
V18

For peer review only

V18
V18
V18
V18
V18
V18
V18
V18
V18
V18
V19
V19
V19
V19
V19
V19
V19
V19
V19
V19
V19
V19
V19
V22
V22
V22
V23
V23
V23
V23
V23
V23
V23
V23
V23
V23
V23
V23
V23
V23
V23
V23
V23
V23
V23
V24
V24
V24
V24
V24

For peer review only

V27
V27
V27
V27
V27
V27
V27
V27
V27
V27
V27
V27
V27
V28
V28
V28
V28
V28
V28
V28
V28
V28
V28
V28
V28
V28
V28
V28
V28
V29
V29
V29
V29
V29
V29
V29
V29
V29
V29
V29
V29
V29
V29
V29
V29
V33
V38
V38
V40
V40

For peer review only

V42
V43
V43
V43
V43
V43
V43
V43
V43
V43
V43
V43
V43
V43
V43
V43
V43
V43
V43
V43
V43
V43
V44
V44
V44
V44
V44
V44
V44
V44
V44
V44
V44
V44
V44
V44
V44
V44
V44
V44
V44
V44
V45
V45
V45
V45
V45
V45
V45
V45

For peer review only

V45
V45
V47
V47
V47
V47
V47
V47
V47
V47
V47
V47
V47
V47
V47
V47
V47
V47
V47
V47
V47
V47
V48
V48
V48
V48
V48
V48
V48
V48
V48
V48
V48
V48
V48
V48
V48
V48
V48
V48
V48
V48
V48
V49
V49
V49
V49
V49
V49
V49

For peer review only

V49
V49
V49
V49
V49
V49
V49
V49
V49
V49
V49
V49
V49
V49
V53
V53
V53
V53
V54
V54
V54
V57
V57
V57
V58
V59
V59
V59
V63
V64
V64
V64
V67
V67
V68
V68
V68
V68
V69
V69
V69
V69
V69
V78
V78
V79
V80
V80
V80
V80

For peer review only

V80
V80
V80
V80
V81
V81
V81
V81
V81
V81
V82
V83
V83
V84
V84
V84
V84
V84
V84
V84
V84
V84
V84
V84
V84
V84
V84
V85
V86
V86
V86
V86
V86
V86
V86
V87
V87
V87
V87
V89
V89
V89
V89
V89
V89
V89
V89
V89
V89
V89

For peer review only

V89
V89
V89
V89
V90
V90
V90
V90
V92
V92
V92
V92
V93
V95
V95
V95
V95
V95
V95
V95
V95
V95
V95
V96
V96
V96
V96
V97
V99
V99
V99
V99
V99
V99
V99
W00
W00
W00
W00
W00
W00
W01
W01
W01
W01
W01
W01
W01
W01
W01

For peer review only

W01
W01
W01
W01
W01
W01
W01
W01
W01
W01
W01
W01
W02
W02
W02
W02
W03
W03
W04
W05
W05
W05
W05
W05
W05
W05
W05
W05
W05
W05
W06
W06
W06
W06
W06
W06
W06
W06
W06
W06
W06
W06
W06
W06
W06
W06
W06
W06
W06
W07

For peer review only

W07
W07
W07
W07
W07
W07
W07
W07
W07
W07
W08
W08
W08
W08
W08
W09
W10
W10
W10
W10
W10
W10
W10
W10
W10
W10
W10
W10
W10
W10
W10
W10
W10
W10
W10
W10
W11
W11
W11
W11
W11
W11
W11
W11
W11
W11
W11
W11
W11
W11

For peer review only

W11
W11
W11
W11
W12
W12
W12
W12
W12
W12
W12
W13
W13
W13
W13
W13
W13
W13
W13
W13
W13
W13
W13
W13
W13
W13
W13
W13
W13
W13
W13
W14
W14
W14
W14
W14
W14
W14
W14
W14
W15
W15
W15
W15
W15
W15
W15
W15
W15

For peer review only

W16
W16
W17
W17
W17
W17
W17
W17
W17
W17
W17
W17
W17
W17
W17
W17
W17
W17
W17
W17
W17
W17
W18
W18
W18
W18
W18
W18
W18
W18
W18
W18
W18
W18
W18
W18
W18
W18
W18
W18
W18
W18
W19
W19
W19
W19
W19
W19
W19
W19

For peer review only

W19
W19
W19
W19
W19
W19
W19
W19
W19
W19
W19
W19
W19
W20
W20
W20
W20
W20
W20
W20
W20
W20
W20
W20
W20
W20
W20
W20
W20
W20
W20
W20
W20
W20
W22
W22
W22
W22
W22
W22
W23
W23
W23
W23
W23
W23
W23
W23
W23
W23

For peer review only

W23
W23
W23
W23
W23
W23
W23
W24
W24
W24
W24
W24
W25
W26
W29
W29
W29
W29
W30
W30
W30
W30
W30
W30
W30
W31
W31
W31
W31
W31
W31
W33
W33
W34
W34
W34
W34
W36
W40
W40
W40
W40
W40
W40
W40
W44
W44
W49
W49
W49

For peer review only

W49
W50
W54
W54
W55
W55
W55
W55
W55
W55
W55
W55
W64
W65
W65
W65
W65
W65
W65
W65
W65
W65
W65
W65
W66
W67
W67
W67
W67
W68
W68
W69
W69
W69
W69
W69
W69
W69
W69
W69
W69
W69
W69
W69
W69
W69
W69
W69
W69
W69

For peer review only

W69
W69
W70
W70
W70
W70
W70
W70
W70
W70
W70
W70
W70
W70
W70
W70
W70
W70
W70
W70
W73
W73
W73
W73
W73
W73
W73
W73
W73
W73
W73
W73
W73
W74
W74
W74
W74
W74
W74
W74
W74
W74
W74
W74
W74
W74
W74
W74
W74
W74

For peer review only

W74
W74
W75
W75
W75
W75
W75
W75
W76
W76
W76
W76
W76
W76
W76
W76
W76
W76
W77
W77
W77
W77
W77
W77
W78
W78
W78
W78
W78
W78
W78
W78
W78
W78
W78
W78
W78
W78
W78
W78
W78
W78
W78
W78
W78
W79
W79
W79
W79
W79

For peer review only

W79
W79
W79
W79
W79
W79
W79
W79
W79
W79
W79
W79
W79
W79
W79
W79
W80
W80
W80
W80
W80
W80
W80
W80
W80
W80
W80
W80
W80
W80
W83
W83
W83
W83
W84
W84
W84
W84
W84
W84
W84
W84
W84
W84
W84
W84
W84
W85
W85
W85

For peer review only

W85
W85
W85
W86
W86
W86
W86
W86
W87
W87
W87
W87
W87
W87
W87
W87
W87
W87
W87
W89
W92
W92
W92
W93
X00
X00
X00
X00
X00
X00
X00
X00
X00
X00
X00
X00
X00
X00
X00
X00
X00
X00
X00
X00
X00
X00
X01
X02
X02
X02
X02

For peer review only

X02
X02
X02
X02
X03
X04
X04
X04
X05
X06
X06
X06
X06
X06
X08
X08
X08
X08
X08
X08
X08
X08
X09
X09
X09
X09
X09
X09
X09
X09
X09
X09
X09
X09
X09
X09
X09
X09
X09
X11
X11
X11
X11
X12
X12
X12
X16
X19
X19
X19

For peer review only

X19
X23
X23
X23
X23
X23
X30
X30
X30
X30
X30
X30
X31
X31
X31
X31
X31
X31
X31
X31
X31
X31
X31
X31
X31
X31
X31
X31
X31
X31
X31
X31
X31
X33
X33
X36
X36
X36
X36
X40
X40
X40
X40
X40
X40
X40
X41
X41
X41
X41

For peer review only

X41
X41
X41
X41
X41
X41
X41
X41
X41
X41
X41
X41
X41
X42
X42
X42
X42
X42
X42
X42
X42
X42
X42
X42
X42
X42
X42
X42
X42
X42
X42
X42
X43
X43
X43
X44
X44
X44
X44
X44
X44
X44
X44
X44
X44
X44
X44
X44
X44
X44

For peer review only

X44
X44
X44
X44
X45
X45
X45
X45
X45
X45
X45
X45
X45
X45
X45
X45
X45
X45
X45
X45
X45
X45
X45
X45
X46
X46
X46
X46
X46
X46
X46
X46
X47
X47
X47
X47
X47
X47
X47
X47
X47
X47
X47
X47
X47
X47
X47
X47
X47
X47

For peer review only

X47
X48
X48
X49
X49
X49
X49
X49
X49
X49
X49
X49
X49
X49
X49
X50
X50
X50
X51
X53
X58
X58
X59
X59
X59
X59
X59
X59
X59
X59
X59
X59
X59
X59
X59
X59
X59
X59
X59
X59
X59
X59
X59
X60
X60
X60
X60
X60
X60
X60

For peer review only

X60
X60
X60
X61
X61
X61
X61
X61
X61
X61
X61
X61
X61
X61
X61
X61
X61
X61
X61
X61
X61
X61
X62
X62
X62
X62
X62
X62
X62
X62
X62
X62
X62
X62
X62
X62
X62
X63
X63
X63
X63
X63
X64
X64
X64
X64
X64
X64
X64
X64

For peer review only

X64
X64
X64
X64
X64
X64
X64
X64
X64
X64
X65
X65
X65
X65
X65
X66
X66
X67
X67
X67
X67
X67
X67
X67
X67
X67
X67
X67
X67
X67
X67
X67
X67
X67
X67
X68
X68
X68
X68
X68
X68
X69
X69
X69
X69
X69
X69
X69
X69
X69

For peer review only

X69
X69
X69
X69
X70
X70
X70
X70
X70
X70
X70
X70
X70
X70
X70
X70
X70
X70
X70
X70
X70
X70
X70
X70
X71
X71
X71
X71
X71
X71
X71
X71
X71
X71
X71
X71
X71
X71
X71
X71
X71
X71
X71
X71
X72
X72
X72
X72
X72

For peer review only

X72
X72
X72
X72
X72
X72
X72
X72
X72
X72
X72
X72
X73
X73
X73
X73
X73
X73
X73
X73
X73
X73
X73
X73
X73
X73
X73
X73
X73
X73
X74
X74
X74
X74
X74
X74
X74
X74
X74
X74
X74
X74
X74
X74
X74
X74
X74
X74
X74
X74

For peer review only

X74
X75
X76
X76
X76
X76
X76
X76
X76
X76
X76
X76
X76
X76
X76
X76
X76
X78
X78
X78
X78
X78
X78
X78
X78
X78
X78
X78
X78
X78
X78
X78
X78
X78
X78
X78
X78
X78
X80
X80
X80
X80
X80
X80
X80
X80
X80
X80
X80
X80

For peer review only

X80
X80
X80
X80
X80
X80
X80
X80
X80
X81
X81
X81
X81
X81
X81
X81
X81
X81
X81
X81
X81
X81
X81
X81
X81
X81
X82
X82
X82
X82
X82
X82
X82
X82
X82
X82
X82
X83
X83
X83
X83
X83
X83
X83
X83
X83
X83
X83
X84
X84

For peer review only

X84
X84
X84
X84
X84
X84
X84
X84
X84
X84
X84
X91
X91
X91
X91
X91
X91
X91
X91
X91
X91
X91
X91
X91
X91
X91
X91
X91
X91
X92
X93
X93
X93
X93
X93
X93
X94
X94
X94
X94
X95
X95
X95
X95
X95
X95
X95
X95
X95
X95

X95
X95
X95
X95
X95
X95
X95
X97
X99
X99
X99
X99
X99
X99
X99
X99
X99
X99
X99
X99
X99
X99
X99
X99
X99
X99
X99
X99
X99
Y00
Y00
Y00
Y00
Y00
Y00
Y00
Y00
Y00
Y00
Y00
Y00
Y00
Y00
Y00
Y04
Y04
Y04
Y04
Y04
Y04

For peer review only

Y04
Y04
Y04
Y04
Y04
Y04
Y04
Y04
Y04
Y04
Y06
Y07
Y07
Y07
Y07
Y08
Y09
Y09
Y09
Y09
Y09
Y09
Y09
Y09
Y09
Y09
Y09
Y09
Y09
Y10
Y10
Y11
Y11
Y11
Y11
Y11
Y11
Y11
Y11
Y11
Y12
Y12
Y12
Y12
Y12
Y12
Y12
Y13
Y14
Y14

For peer review only

Y14
Y14
Y14
Y14
Y14
Y14
Y14
Y14
Y14
Y14
Y14
Y14
Y14
Y15
Y15
Y15
Y15
Y15
Y15
Y15
Y16
Y16
Y17
Y17
Y17
Y17
Y17
Y17
Y17
Y17
Y19
Y19
Y19
Y19
Y19
Y19
Y20
Y20
Y20
Y20
Y20
Y20
Y20
Y20
Y20
Y20
Y21
Y21
Y21
Y21

For peer review only

Y21
Y21
Y21
Y21
Y21
Y21
Y21
Y21
Y21
Y21
Y21
Y21
Y21
Y21
Y22
Y22
Y23
Y23
Y24
Y24
Y24
Y24
Y24
Y24
Y24
Y24
Y24
Y26
Y26
Y26
Y26
Y26
Y26
Y26
Y26
Y26
Y28
Y28
Y28
Y28
Y28
Y28
Y28
Y28
Y29
Y29
Y29
Y29
Y29
Y30

For peer review only

Y30
Y30
Y30
Y30
Y30
Y30
Y30
Y30
Y30
Y30
Y30
Y30
Y30
Y30
Y30
Y31
Y31
Y31
Y31
Y31
Y31
Y31
Y32
Y32
Y33
Y33
Y33
Y33
Y33
Y33
Y33
Y33
Y34
Y34
Y34
Y34
Y34
Y34
Y34
Y34
Y34
Y34
Y34
Y34
Y34
Y34
Y34
Y34
Y34
Y34

For peer review only

Y34
Y40
Y40
Y40
Y42
Y43
Y44
Y44
Y44
Y44
Y44
Y44
Y44
Y45
Y45
Y45
Y45
Y48
Y49
Y52
Y52
Y54
Y57
Y57
Y57
Y57
Y57
Y60
Y60
Y60
Y60
Y60
Y60
Y60
Y60
Y62
Y65
Y65
Y65
Y69
Y69
Y71
Y71
Y73
Y83
Y83
Y83
Y83
Y83
Y83

For peer review only

Y83
Y83
Y83
Y83
Y83
Y83
Y83
Y83
Y83
Y83
Y83
Y84
Y84
Y84
Y84
Y84
Y84
Y84
Y84
Y84
Y85
Y85
Y85
Y85
Y85
Y85
Y85
Y85
Y85
Y85
Y86
Y86
Y86
Y86
Y86
Y86
Y86
Y86
Y86
Y86
Y86
Y86
Y86
Y86
Y87
Y87
Y87
Y87
Y87
Y88

For peer review only

Y88
Y88
Y88
Y89
Y89
Y89
Y89
Y89

For peer review only

ICD10 code label
Other salmonella infections
Other salmonella infections
Other salmonella infections
Other salmonella infections
Other salmonella infections
Other salmonella infections
Other salmonella infections
Other salmonella infections
Other salmonella infections
Other bacterial intestinal infections
Other bacterial intestinal infections
Other bacterial intestinal infections
Other bacterial intestinal infections
Other bacterial intestinal infections
Other bacterial intestinal infections
Other bacterial intestinal infections
Other bacterial intestinal infections
Other bacterial intestinal infections
Other bacterial intestinal infections
Other bacterial intestinal infections
Other bacterial intestinal infections
Other bacterial intestinal infections
Other bacterial intestinal infections
Other bacterial intestinal infections
Other bacterial intestinal infections
Other bacterial foodborne intoxications
Other bacterial foodborne intoxications
Other bacterial foodborne intoxications
Viral and other specified intestinal infections
Viral and other specified intestinal infections
Viral and other specified intestinal infections
Viral and other specified intestinal infections
Viral and other specified intestinal infections
Viral and other specified intestinal infections
Viral and other specified intestinal infections
Viral and other specified intestinal infections
Viral and other specified intestinal infections
Viral and other specified intestinal infections
Viral and other specified intestinal infections
Viral and other specified intestinal infections
Viral and other specified intestinal infections
Diarrhoea and gastroenteritis of presumed infectious origin
Diarrhoea and gastroenteritis of presumed infectious origin
Diarrhoea and gastroenteritis of presumed infectious origin
Diarrhoea and gastroenteritis of presumed infectious origin
Diarrhoea and gastroenteritis of presumed infectious origin
Diarrhoea and gastroenteritis of presumed infectious origin
Diarrhoea and gastroenteritis of presumed infectious origin
Diarrhoea and gastroenteritis of presumed infectious origin

For peer review only

Diarrhoea and gastroenteritis of presumed infectious origin
Diarrhoea and gastroenteritis of presumed infectious origin
Diarrhoea and gastroenteritis of presumed infectious origin
Diarrhoea and gastroenteritis of presumed infectious origin
Diarrhoea and gastroenteritis of presumed infectious origin
Diarrhoea and gastroenteritis of presumed infectious origin
Diarrhoea and gastroenteritis of presumed infectious origin
Diarrhoea and gastroenteritis of presumed infectious origin
Diarrhoea and gastroenteritis of presumed infectious origin
Respiratory tuberculosis, bacteriologically and histologically confirmed
Respiratory tuberculosis, bacteriologically and histologically confirmed
Respiratory tuberculosis, bacteriologically and histologically confirmed
Respiratory tuberculosis, bacteriologically and histologically confirmed
Respiratory tuberculosis, bacteriologically and histologically confirmed
Respiratory tuberculosis, bacteriologically and histologically confirmed
Respiratory tuberculosis, bacteriologically and histologically confirmed
Respiratory tuberculosis, bacteriologically and histologically confirmed
Respiratory tuberculosis, bacteriologically and histologically confirmed
Respiratory tuberculosis, not confirmed bacteriologically or histologically
Respiratory tuberculosis, not confirmed bacteriologically or histologically
Respiratory tuberculosis, not confirmed bacteriologically or histologically
Respiratory tuberculosis, not confirmed bacteriologically or histologically
Respiratory tuberculosis, not confirmed bacteriologically or histologically
Respiratory tuberculosis, not confirmed bacteriologically or histologically
Respiratory tuberculosis, not confirmed bacteriologically or histologically
Respiratory tuberculosis, not confirmed bacteriologically or histologically
Respiratory tuberculosis, not confirmed bacteriologically or histologically
Respiratory tuberculosis, not confirmed bacteriologically or histologically
Respiratory tuberculosis, not confirmed bacteriologically or histologically
Respiratory tuberculosis, not confirmed bacteriologically or histologically
Respiratory tuberculosis, not confirmed bacteriologically or histologically
Respiratory tuberculosis, not confirmed bacteriologically or histologically
Respiratory tuberculosis, not confirmed bacteriologically or histologically
Respiratory tuberculosis, not confirmed bacteriologically or histologically
Respiratory tuberculosis, not confirmed bacteriologically or histologically
Respiratory tuberculosis, not confirmed bacteriologically or histologically
Tuberculosis of nervous system
Tuberculosis of nervous system
Tuberculosis of nervous system
Tuberculosis of nervous system
Tuberculosis of nervous system
Tuberculosis of nervous system
Tuberculosis of other organs
Tuberculosis of other organs
Tuberculosis of other organs
Tuberculosis of other organs
Tuberculosis of other organs
Tuberculosis of other organs
Miliary tuberculosis
Miliary tuberculosis

For peer review only

Miliary tuberculosis
Miliary tuberculosis
Miliary tuberculosis
Miliary tuberculosis
Miliary tuberculosis
Miliary tuberculosis
Miliary tuberculosis
Miliary tuberculosis
Erysipeloid
Leptospirosis
Infection due to other mycobacteria
Infection due to other mycobacteria
Infection due to other mycobacteria
Infection due to other mycobacteria
Infection due to other mycobacteria
Infection due to other mycobacteria
Listeriosis
Listeriosis
Listeriosis
Listeriosis
Other tetanus
Other tetanus
Other tetanus
Whooping cough
Meningococcal infection
Meningococcal infection
Meningococcal infection
Meningococcal infection
Meningococcal infection
Meningococcal infection
Meningococcal infection
Meningococcal infection
Meningococcal infection
Meningococcal infection
Meningococcal infection
Meningococcal infection
Meningococcal infection
Meningococcal infection
Streptococcal septicaemia
Streptococcal septicaemia
Streptococcal septicaemia
Streptococcal septicaemia
Streptococcal septicaemia
Streptococcal septicaemia
Streptococcal septicaemia
Streptococcal septicaemia
Streptococcal septicaemia
Streptococcal septicaemia
Streptococcal septicaemia
Streptococcal septicaemia

For peer review only

Streptococcal septicaemia
Streptococcal septicaemia
Streptococcal septicaemia
Other septicaemia
Other septicaemia
Other septicaemia
Other septicaemia
Other septicaemia
Other septicaemia
Other septicaemia
Other septicaemia
Other septicaemia
Other septicaemia
Other septicaemia
Other septicaemia
Other septicaemia
Other septicaemia
Other septicaemia
Other septicaemia
Other septicaemia
Other septicaemia
Other septicaemia
Other septicaemia
Other septicaemia
Nocardiosis
Nocardiosis
Erysipelas
Erysipelas
Erysipelas
Erysipelas
Erysipelas
Erysipelas
Erysipelas
Erysipelas
Erysipelas
Erysipelas
Erysipelas
Erysipelas
Erysipelas
Erysipelas
Erysipelas
Erysipelas
Other bacterial diseases, not elsewhere classified
Other bacterial diseases, not elsewhere classified
Other bacterial diseases, not elsewhere classified
Other bacterial diseases, not elsewhere classified
Other bacterial diseases, not elsewhere classified
Other bacterial diseases, not elsewhere classified
Other bacterial diseases, not elsewhere classified
Other bacterial diseases, not elsewhere classified

For peer review only

Other bacterial diseases, not elsewhere classified
Other bacterial diseases, not elsewhere classified
Other bacterial diseases, not elsewhere classified
Other bacterial diseases, not elsewhere classified
Other bacterial diseases, not elsewhere classified
Bacterial infection of unspecified site
Bacterial infection of unspecified site
Bacterial infection of unspecified site
Bacterial infection of unspecified site
Bacterial infection of unspecified site
Bacterial infection of unspecified site
Bacterial infection of unspecified site
Bacterial infection of unspecified site
Bacterial infection of unspecified site
Bacterial infection of unspecified site
Bacterial infection of unspecified site
Bacterial infection of unspecified site
Bacterial infection of unspecified site
Bacterial infection of unspecified site
Late syphilis
Late syphilis
Other spirochaetal infections
Slow virus infections of central nervous system
Slow virus infections of central nervous system
Slow virus infections of central nervous system
Slow virus infections of central nervous system
Slow virus infections of central nervous system
Slow virus infections of central nervous system
Slow virus infections of central nervous system
Slow virus infections of central nervous system
Slow virus infections of central nervous system
Slow virus infections of central nervous system
Slow virus infections of central nervous system
Slow virus infections of central nervous system
Slow virus infections of central nervous system
Slow virus infections of central nervous system
Slow virus infections of central nervous system
Unspecified viral encephalitis
Unspecified viral encephalitis
Unspecified viral encephalitis
Unspecified viral encephalitis
Unspecified viral encephalitis
Unspecified viral encephalitis
Viral meningitis
Viral meningitis
Viral meningitis
Herpesviral [herpes simplex] infections
Herpesviral [herpes simplex] infections
Herpesviral [herpes simplex] infections
Herpesviral [herpes simplex] infections

For peer review only

Herpesviral [herpes simplex] infections
Herpesviral [herpes simplex] infections
Herpesviral [herpes simplex] infections
Herpesviral [herpes simplex] infections
Herpesviral [herpes simplex] infections
Varicella [chickenpox]
Varicella [chickenpox]
Varicella [chickenpox]
Varicella [chickenpox]
Zoster [herpes zoster]
Zoster [herpes zoster]
Zoster [herpes zoster]
Zoster [herpes zoster]
Zoster [herpes zoster]
Zoster [herpes zoster]
Zoster [herpes zoster]
Zoster [herpes zoster]
Zoster [herpes zoster]
Zoster [herpes zoster]
Acute hepatitis A
Acute hepatitis B
Acute hepatitis B
Acute hepatitis B
Acute hepatitis B
Acute hepatitis B
Acute hepatitis B
Acute hepatitis B
Other acute viral hepatitis
Other acute viral hepatitis
Other acute viral hepatitis
Other acute viral hepatitis
Other acute viral hepatitis
Other acute viral hepatitis
Other acute viral hepatitis
Other acute viral hepatitis
Other acute viral hepatitis
Chronic viral hepatitis
Chronic viral hepatitis
Chronic viral hepatitis
Chronic viral hepatitis
Chronic viral hepatitis
Chronic viral hepatitis
Chronic viral hepatitis
Chronic viral hepatitis
Chronic viral hepatitis
Chronic viral hepatitis
Chronic viral hepatitis
Chronic viral hepatitis
Chronic viral hepatitis
Chronic viral hepatitis

For peer review only

Chronic viral hepatitis
Chronic viral hepatitis
Chronic viral hepatitis
Chronic viral hepatitis
Chronic viral hepatitis
Chronic viral hepatitis
Unspecified viral hepatitis
Unspecified viral hepatitis
Unspecified viral hepatitis
Unspecified viral hepatitis
Unspecified viral hepatitis
Human immunodeficiency virus [HIV] disease resulting in infectious and parasitic diseases
Human immunodeficiency virus [HIV] disease resulting in infectious and parasitic diseases
Human immunodeficiency virus [HIV] disease resulting in infectious and parasitic diseases
Human immunodeficiency virus [HIV] disease resulting in infectious and parasitic diseases
Human immunodeficiency virus [HIV] disease resulting in infectious and parasitic diseases
Human immunodeficiency virus [HIV] disease resulting in infectious and parasitic diseases
Human immunodeficiency virus [HIV] disease resulting in infectious and parasitic diseases
Human immunodeficiency virus [HIV] disease resulting in infectious and parasitic diseases
Human immunodeficiency virus [HIV] disease resulting in infectious and parasitic diseases
Human immunodeficiency virus [HIV] disease resulting in infectious and parasitic diseases
Human immunodeficiency virus [HIV] disease resulting in infectious and parasitic diseases
Human immunodeficiency virus [HIV] disease resulting in infectious and parasitic diseases
Human immunodeficiency virus [HIV] disease resulting in infectious and parasitic diseases
Human immunodeficiency virus [HIV] disease resulting in infectious and parasitic diseases
Human immunodeficiency virus [HIV] disease resulting in infectious and parasitic diseases
Human immunodeficiency virus [HIV] disease resulting in malignant neoplasms
Human immunodeficiency virus [HIV] disease resulting in malignant neoplasms
Human immunodeficiency virus [HIV] disease resulting in malignant neoplasms
Human immunodeficiency virus [HIV] disease resulting in malignant neoplasms
Human immunodeficiency virus [HIV] disease resulting in malignant neoplasms
Human immunodeficiency virus [HIV] disease resulting in malignant neoplasms
Human immunodeficiency virus [HIV] disease resulting in malignant neoplasms
Human immunodeficiency virus [HIV] disease resulting in malignant neoplasms
Human immunodeficiency virus [HIV] disease resulting in malignant neoplasms
Human immunodeficiency virus [HIV] disease resulting in other specified diseases
Human immunodeficiency virus [HIV] disease resulting in other specified diseases
Human immunodeficiency virus [HIV] disease resulting in other specified diseases
Human immunodeficiency virus [HIV] disease resulting in other specified diseases
Human immunodeficiency virus [HIV] disease resulting in other specified diseases
Human immunodeficiency virus [HIV] disease resulting in other specified diseases
Human immunodeficiency virus [HIV] disease resulting in other specified diseases
Human immunodeficiency virus [HIV] disease resulting in other specified diseases
Human immunodeficiency virus [HIV] disease resulting in other specified diseases
Human immunodeficiency virus [HIV] disease resulting in other specified diseases
Human immunodeficiency virus [HIV] disease resulting in other conditions
Human immunodeficiency virus [HIV] disease resulting in other conditions
Human immunodeficiency virus [HIV] disease resulting in other conditions
Human immunodeficiency virus [HIV] disease resulting in other conditions
Human immunodeficiency virus [HIV] disease resulting in other conditions

Human immunodeficiency virus [HIV] disease resulting in other conditions
Human immunodeficiency virus [HIV] disease resulting in other conditions
Unspecified human immunodeficiency virus [HIV] disease
Unspecified human immunodeficiency virus [HIV] disease
Unspecified human immunodeficiency virus [HIV] disease
Unspecified human immunodeficiency virus [HIV] disease
Unspecified human immunodeficiency virus [HIV] disease
Unspecified human immunodeficiency virus [HIV] disease
Unspecified human immunodeficiency virus [HIV] disease
Unspecified human immunodeficiency virus [HIV] disease
Unspecified human immunodeficiency virus [HIV] disease
Unspecified human immunodeficiency virus [HIV] disease
Cytomegaloviral disease
Cytomegaloviral disease
Cytomegaloviral disease
Cytomegaloviral disease
Other viral diseases, not elsewhere classified
Viral infection of unspecified site
Viral infection of unspecified site
Viral infection of unspecified site
Viral infection of unspecified site
Viral infection of unspecified site
Viral infection of unspecified site
Viral infection of unspecified site
Viral infection of unspecified site
Viral infection of unspecified site
Viral infection of unspecified site
Viral infection of unspecified site
Viral infection of unspecified site
Candidiasis
Candidiasis
Candidiasis
Candidiasis
Candidiasis
Candidiasis
Candidiasis
Candidiasis
Candidiasis
Candidiasis
Aspergillosis
Aspergillosis
Aspergillosis
Aspergillosis
Aspergillosis
Aspergillosis
Aspergillosis
Unspecified mycosis
Unspecified mycosis
Unspecified mycosis
Unspecified mycosis

For peer review only

Plasmodium falciparum malaria
Unspecified malaria
Leishmaniasis
Pneumocystosis
Pneumocystosis
Pneumocystosis
Pneumocystosis
Pneumocystosis
Pneumocystosis
Echinococcosis
Echinococcosis
Echinococcosis
Sequelae of tuberculosis
Sequelae of tuberculosis
Sequelae of tuberculosis
Sequelae of tuberculosis
Sequelae of tuberculosis
Sequelae of tuberculosis
Sequelae of tuberculosis
Sequelae of tuberculosis
Sequelae of tuberculosis
Sequelae of tuberculosis
Sequelae of tuberculosis
Sequelae of tuberculosis
Sequelae of tuberculosis
Sequelae of poliomyelitis
Sequelae of poliomyelitis
Sequelae of poliomyelitis
Sequelae of poliomyelitis
Sequelae of poliomyelitis
Sequelae of poliomyelitis
Sequelae of poliomyelitis
Sequelae of poliomyelitis
Sequelae of poliomyelitis
Sequelae of poliomyelitis
Sequelae of poliomyelitis
Sequelae of poliomyelitis
Sequelae of other and unspecified infectious and parasitic diseases
Sequelae of other and unspecified infectious and parasitic diseases
Sequelae of other and unspecified infectious and parasitic diseases
Sequelae of other and unspecified infectious and parasitic diseases
Sequelae of other and unspecified infectious and parasitic diseases
Sequelae of other and unspecified infectious and parasitic diseases
Sequelae of other and unspecified infectious and parasitic diseases
Other and unspecified infectious diseases
Other and unspecified infectious diseases
Other and unspecified infectious diseases
Other and unspecified infectious diseases
Other and unspecified infectious diseases
Other and unspecified infectious diseases

For peer review only

Other and unspecified infectious diseases
Other and unspecified infectious diseases
Other and unspecified infectious diseases
Other and unspecified infectious diseases
Other and unspecified infectious diseases
Malignant neoplasm of lip
Malignant neoplasm of lip
Malignant neoplasm of lip
Malignant neoplasm of lip
Malignant neoplasm of lip
Malignant neoplasm of lip
Malignant neoplasm of lip
Malignant neoplasm of lip
Malignant neoplasm of lip
Malignant neoplasm of lip
Malignant neoplasm of lip
Malignant neoplasm of lip
Malignant neoplasm of lip
Malignant neoplasm of lip
Malignant neoplasm of base of tongue
Malignant neoplasm of base of tongue
Malignant neoplasm of base of tongue
Malignant neoplasm of base of tongue
Malignant neoplasm of base of tongue
Malignant neoplasm of base of tongue
Malignant neoplasm of base of tongue
Malignant neoplasm of base of tongue
Malignant neoplasm of base of tongue
Malignant neoplasm of base of tongue
Malignant neoplasm of base of tongue
Malignant neoplasm of base of tongue
Malignant neoplasm of base of tongue
Malignant neoplasm of base of tongue
Malignant neoplasm of base of tongue
Malignant neoplasm of base of tongue
Malignant neoplasm of base of tongue
Malignant neoplasm of base of tongue
Malignant neoplasm of base of tongue
Malignant neoplasm of base of tongue
Malignant neoplasm of other and unspecified parts of tongue
Malignant neoplasm of other and unspecified parts of tongue
Malignant neoplasm of other and unspecified parts of tongue
Malignant neoplasm of other and unspecified parts of tongue
Malignant neoplasm of other and unspecified parts of tongue
Malignant neoplasm of other and unspecified parts of tongue
Malignant neoplasm of other and unspecified parts of tongue
Malignant neoplasm of other and unspecified parts of tongue
Malignant neoplasm of other and unspecified parts of tongue
Malignant neoplasm of other and unspecified parts of tongue
Malignant neoplasm of other and unspecified parts of tongue

For peer review only

Malignant neoplasm of other and unspecified parts of tongue
Malignant neoplasm of other and unspecified parts of tongue
Malignant neoplasm of other and unspecified parts of tongue
Malignant neoplasm of other and unspecified parts of tongue
Malignant neoplasm of other and unspecified parts of tongue
Malignant neoplasm of other and unspecified parts of tongue
Malignant neoplasm of other and unspecified parts of tongue
Malignant neoplasm of other and unspecified parts of tongue
Malignant neoplasm of other and unspecified parts of tongue
Malignant neoplasm of other and unspecified parts of tongue
Malignant neoplasm of gum
Malignant neoplasm of gum
Malignant neoplasm of gum
Malignant neoplasm of gum
Malignant neoplasm of gum
Malignant neoplasm of gum
Malignant neoplasm of gum
Malignant neoplasm of gum
Malignant neoplasm of gum
Malignant neoplasm of gum
Malignant neoplasm of gum
Malignant neoplasm of gum
Malignant neoplasm of gum
Malignant neoplasm of gum
Malignant neoplasm of gum
Malignant neoplasm of gum
Malignant neoplasm of floor of mouth
Malignant neoplasm of floor of mouth
Malignant neoplasm of floor of mouth
Malignant neoplasm of floor of mouth
Malignant neoplasm of floor of mouth
Malignant neoplasm of floor of mouth
Malignant neoplasm of floor of mouth
Malignant neoplasm of floor of mouth
Malignant neoplasm of floor of mouth
Malignant neoplasm of floor of mouth
Malignant neoplasm of floor of mouth
Malignant neoplasm of floor of mouth
Malignant neoplasm of floor of mouth
Malignant neoplasm of floor of mouth
Malignant neoplasm of floor of mouth
Malignant neoplasm of floor of mouth
Malignant neoplasm of floor of mouth
Malignant neoplasm of floor of mouth
Malignant neoplasm of floor of mouth
Malignant neoplasm of floor of mouth
Malignant neoplasm of floor of mouth
Malignant neoplasm of palate
Malignant neoplasm of palate
Malignant neoplasm of palate

For peer review only

Malignant neoplasm of palate
Malignant neoplasm of palate
Malignant neoplasm of palate
Malignant neoplasm of palate
Malignant neoplasm of palate
Malignant neoplasm of palate
Malignant neoplasm of palate
Malignant neoplasm of palate
Malignant neoplasm of palate
Malignant neoplasm of palate
Malignant neoplasm of palate
Malignant neoplasm of palate
Malignant neoplasm of palate
Malignant neoplasm of palate
Malignant neoplasm of palate
Malignant neoplasm of other and unspecified parts of mouth
Malignant neoplasm of other and unspecified parts of mouth
Malignant neoplasm of other and unspecified parts of mouth
Malignant neoplasm of other and unspecified parts of mouth
Malignant neoplasm of other and unspecified parts of mouth
Malignant neoplasm of other and unspecified parts of mouth
Malignant neoplasm of other and unspecified parts of mouth
Malignant neoplasm of other and unspecified parts of mouth
Malignant neoplasm of other and unspecified parts of mouth
Malignant neoplasm of other and unspecified parts of mouth
Malignant neoplasm of other and unspecified parts of mouth
Malignant neoplasm of other and unspecified parts of mouth
Malignant neoplasm of other and unspecified parts of mouth
Malignant neoplasm of other and unspecified parts of mouth
Malignant neoplasm of other and unspecified parts of mouth
Malignant neoplasm of other and unspecified parts of mouth
Malignant neoplasm of other and unspecified parts of mouth
Malignant neoplasm of other and unspecified parts of mouth
Malignant neoplasm of other and unspecified parts of mouth
Malignant neoplasm of other and unspecified parts of mouth
Malignant neoplasm of parotid gland
Malignant neoplasm of parotid gland
Malignant neoplasm of parotid gland
Malignant neoplasm of parotid gland
Malignant neoplasm of parotid gland
Malignant neoplasm of parotid gland
Malignant neoplasm of parotid gland
Malignant neoplasm of parotid gland
Malignant neoplasm of parotid gland
Malignant neoplasm of parotid gland
Malignant neoplasm of parotid gland
Malignant neoplasm of parotid gland
Malignant neoplasm of parotid gland
Malignant neoplasm of parotid gland
Malignant neoplasm of parotid gland

For peer review only

Malignant neoplasm of parotid gland
Malignant neoplasm of parotid gland
Malignant neoplasm of parotid gland
Malignant neoplasm of parotid gland
Malignant neoplasm of parotid gland
Malignant neoplasm of other and unspecified major salivary glands
Malignant neoplasm of other and unspecified major salivary glands
Malignant neoplasm of other and unspecified major salivary glands
Malignant neoplasm of other and unspecified major salivary glands
Malignant neoplasm of other and unspecified major salivary glands
Malignant neoplasm of other and unspecified major salivary glands
Malignant neoplasm of other and unspecified major salivary glands
Malignant neoplasm of other and unspecified major salivary glands
Malignant neoplasm of other and unspecified major salivary glands
Malignant neoplasm of other and unspecified major salivary glands
Malignant neoplasm of other and unspecified major salivary glands
Malignant neoplasm of other and unspecified major salivary glands
Malignant neoplasm of other and unspecified major salivary glands
Malignant neoplasm of other and unspecified major salivary glands
Malignant neoplasm of other and unspecified major salivary glands
Malignant neoplasm of other and unspecified major salivary glands
Malignant neoplasm of other and unspecified major salivary glands
Malignant neoplasm of other and unspecified major salivary glands
Malignant neoplasm of tonsil
Malignant neoplasm of tonsil
Malignant neoplasm of tonsil
Malignant neoplasm of tonsil
Malignant neoplasm of tonsil
Malignant neoplasm of tonsil
Malignant neoplasm of tonsil
Malignant neoplasm of tonsil
Malignant neoplasm of tonsil
Malignant neoplasm of tonsil
Malignant neoplasm of tonsil
Malignant neoplasm of tonsil
Malignant neoplasm of tonsil
Malignant neoplasm of tonsil
Malignant neoplasm of tonsil
Malignant neoplasm of tonsil
Malignant neoplasm of tonsil
Malignant neoplasm of tonsil
Malignant neoplasm of tonsil
Malignant neoplasm of tonsil
Malignant neoplasm of oropharynx
Malignant neoplasm of oropharynx
Malignant neoplasm of oropharynx
Malignant neoplasm of oropharynx
Malignant neoplasm of oropharynx
Malignant neoplasm of oropharynx
Malignant neoplasm of oropharynx

For peer review only

Malignant neoplasm of oropharynx
Malignant neoplasm of oropharynx
Malignant neoplasm of oropharynx
Malignant neoplasm of oropharynx
Malignant neoplasm of oropharynx
Malignant neoplasm of oropharynx
Malignant neoplasm of oropharynx
Malignant neoplasm of oropharynx
Malignant neoplasm of oropharynx
Malignant neoplasm of oropharynx
Malignant neoplasm of oropharynx
Malignant neoplasm of oropharynx
Malignant neoplasm of oropharynx
Malignant neoplasm of oropharynx
Malignant neoplasm of nasopharynx
Malignant neoplasm of nasopharynx
Malignant neoplasm of nasopharynx
Malignant neoplasm of nasopharynx
Malignant neoplasm of nasopharynx
Malignant neoplasm of nasopharynx
Malignant neoplasm of nasopharynx
Malignant neoplasm of nasopharynx
Malignant neoplasm of nasopharynx
Malignant neoplasm of nasopharynx
Malignant neoplasm of nasopharynx
Malignant neoplasm of nasopharynx
Malignant neoplasm of nasopharynx
Malignant neoplasm of nasopharynx
Malignant neoplasm of nasopharynx
Malignant neoplasm of nasopharynx
Malignant neoplasm of nasopharynx
Malignant neoplasm of nasopharynx
Malignant neoplasm of nasopharynx
Malignant neoplasm of nasopharynx
Malignant neoplasm of pyriform sinus
Malignant neoplasm of pyriform sinus
Malignant neoplasm of pyriform sinus
Malignant neoplasm of pyriform sinus
Malignant neoplasm of pyriform sinus
Malignant neoplasm of pyriform sinus
Malignant neoplasm of pyriform sinus
Malignant neoplasm of pyriform sinus
Malignant neoplasm of pyriform sinus
Malignant neoplasm of pyriform sinus
Malignant neoplasm of pyriform sinus
Malignant neoplasm of pyriform sinus
Malignant neoplasm of pyriform sinus
Malignant neoplasm of pyriform sinus
Malignant neoplasm of hypopharynx
Malignant neoplasm of hypopharynx

For peer review only

Malignant neoplasm of hypopharynx
Malignant neoplasm of hypopharynx
Malignant neoplasm of hypopharynx
Malignant neoplasm of hypopharynx
Malignant neoplasm of hypopharynx
Malignant neoplasm of hypopharynx
Malignant neoplasm of hypopharynx
Malignant neoplasm of hypopharynx
Malignant neoplasm of hypopharynx
Malignant neoplasm of hypopharynx
Malignant neoplasm of hypopharynx
Malignant neoplasm of hypopharynx
Malignant neoplasm of hypopharynx
Malignant neoplasm of hypopharynx
Malignant neoplasm of hypopharynx
Malignant neoplasm of hypopharynx
Malignant neoplasm of hypopharynx
Malignant neoplasm of hypopharynx
Malignant neoplasm of hypopharynx
Malignant neoplasm of other and ill-defined sites in the lip, oral cavity and pharynx
Malignant neoplasm of other and ill-defined sites in the lip, oral cavity and pharynx
Malignant neoplasm of other and ill-defined sites in the lip, oral cavity and pharynx
Malignant neoplasm of other and ill-defined sites in the lip, oral cavity and pharynx
Malignant neoplasm of other and ill-defined sites in the lip, oral cavity and pharynx
Malignant neoplasm of other and ill-defined sites in the lip, oral cavity and pharynx
Malignant neoplasm of other and ill-defined sites in the lip, oral cavity and pharynx
Malignant neoplasm of other and ill-defined sites in the lip, oral cavity and pharynx
Malignant neoplasm of other and ill-defined sites in the lip, oral cavity and pharynx
Malignant neoplasm of other and ill-defined sites in the lip, oral cavity and pharynx
Malignant neoplasm of other and ill-defined sites in the lip, oral cavity and pharynx
Malignant neoplasm of other and ill-defined sites in the lip, oral cavity and pharynx
Malignant neoplasm of other and ill-defined sites in the lip, oral cavity and pharynx
Malignant neoplasm of other and ill-defined sites in the lip, oral cavity and pharynx
Malignant neoplasm of other and ill-defined sites in the lip, oral cavity and pharynx
Malignant neoplasm of other and ill-defined sites in the lip, oral cavity and pharynx
Malignant neoplasm of other and ill-defined sites in the lip, oral cavity and pharynx
Malignant neoplasm of other and ill-defined sites in the lip, oral cavity and pharynx
Malignant neoplasm of other and ill-defined sites in the lip, oral cavity and pharynx
Malignant neoplasm of other and ill-defined sites in the lip, oral cavity and pharynx
Malignant neoplasm of oesophagus
Malignant neoplasm of oesophagus
Malignant neoplasm of oesophagus
Malignant neoplasm of oesophagus
Malignant neoplasm of oesophagus
Malignant neoplasm of oesophagus
Malignant neoplasm of oesophagus
Malignant neoplasm of oesophagus
Malignant neoplasm of oesophagus
Malignant neoplasm of oesophagus
Malignant neoplasm of oesophagus

For peer review only

Malignant neoplasm of oesophagus
Malignant neoplasm of oesophagus
Malignant neoplasm of oesophagus
Malignant neoplasm of oesophagus
Malignant neoplasm of oesophagus
Malignant neoplasm of oesophagus
Malignant neoplasm of oesophagus
Malignant neoplasm of oesophagus
Malignant neoplasm of oesophagus
Malignant neoplasm of oesophagus
Malignant neoplasm of stomach
Malignant neoplasm of stomach
Malignant neoplasm of stomach
Malignant neoplasm of stomach
Malignant neoplasm of stomach
Malignant neoplasm of stomach
Malignant neoplasm of stomach
Malignant neoplasm of stomach
Malignant neoplasm of stomach
Malignant neoplasm of stomach
Malignant neoplasm of stomach
Malignant neoplasm of stomach
Malignant neoplasm of stomach
Malignant neoplasm of stomach
Malignant neoplasm of stomach
Malignant neoplasm of stomach
Malignant neoplasm of stomach
Malignant neoplasm of stomach
Malignant neoplasm of stomach
Malignant neoplasm of stomach
Malignant neoplasm of stomach
Malignant neoplasm of small intestine
Malignant neoplasm of small intestine
Malignant neoplasm of small intestine
Malignant neoplasm of small intestine
Malignant neoplasm of small intestine
Malignant neoplasm of small intestine
Malignant neoplasm of small intestine
Malignant neoplasm of small intestine
Malignant neoplasm of small intestine
Malignant neoplasm of small intestine
Malignant neoplasm of small intestine
Malignant neoplasm of small intestine
Malignant neoplasm of small intestine
Malignant neoplasm of small intestine
Malignant neoplasm of small intestine
Malignant neoplasm of small intestine
Malignant neoplasm of small intestine
Malignant neoplasm of small intestine
Malignant neoplasm of small intestine

For peer review only

Malignant neoplasm of small intestine
Malignant neoplasm of small intestine
Malignant neoplasm of colon
Malignant neoplasm of colon
Malignant neoplasm of colon
Malignant neoplasm of colon
Malignant neoplasm of colon
Malignant neoplasm of colon
Malignant neoplasm of colon
Malignant neoplasm of colon
Malignant neoplasm of colon
Malignant neoplasm of colon
Malignant neoplasm of colon
Malignant neoplasm of colon
Malignant neoplasm of colon
Malignant neoplasm of colon
Malignant neoplasm of colon
Malignant neoplasm of colon
Malignant neoplasm of colon
Malignant neoplasm of colon
Malignant neoplasm of colon
Malignant neoplasm of colon
Malignant neoplasm of colon
Malignant neoplasm of rectosigmoid junction
Malignant neoplasm of rectosigmoid junction
Malignant neoplasm of rectosigmoid junction
Malignant neoplasm of rectosigmoid junction
Malignant neoplasm of rectosigmoid junction
Malignant neoplasm of rectosigmoid junction
Malignant neoplasm of rectosigmoid junction
Malignant neoplasm of rectosigmoid junction
Malignant neoplasm of rectosigmoid junction
Malignant neoplasm of rectosigmoid junction
Malignant neoplasm of rectosigmoid junction
Malignant neoplasm of rectosigmoid junction
Malignant neoplasm of rectosigmoid junction
Malignant neoplasm of rectosigmoid junction
Malignant neoplasm of rectosigmoid junction
Malignant neoplasm of rectosigmoid junction
Malignant neoplasm of rectosigmoid junction
Malignant neoplasm of rectosigmoid junction
Malignant neoplasm of rectosigmoid junction
Malignant neoplasm of rectosigmoid junction
Malignant neoplasm of rectosigmoid junction
Malignant neoplasm of rectum
Malignant neoplasm of rectum
Malignant neoplasm of rectum
Malignant neoplasm of rectum
Malignant neoplasm of rectum
Malignant neoplasm of rectum

For peer review only

Malignant neoplasm of rectum
Malignant neoplasm of rectum
Malignant neoplasm of rectum
Malignant neoplasm of rectum
Malignant neoplasm of rectum
Malignant neoplasm of rectum
Malignant neoplasm of rectum
Malignant neoplasm of rectum
Malignant neoplasm of rectum
Malignant neoplasm of rectum
Malignant neoplasm of rectum
Malignant neoplasm of rectum
Malignant neoplasm of rectum
Malignant neoplasm of rectum
Malignant neoplasm of rectum
Malignant neoplasm of anus and anal canal
Malignant neoplasm of anus and anal canal
Malignant neoplasm of anus and anal canal
Malignant neoplasm of anus and anal canal
Malignant neoplasm of anus and anal canal
Malignant neoplasm of anus and anal canal
Malignant neoplasm of anus and anal canal
Malignant neoplasm of anus and anal canal
Malignant neoplasm of anus and anal canal
Malignant neoplasm of anus and anal canal
Malignant neoplasm of anus and anal canal
Malignant neoplasm of anus and anal canal
Malignant neoplasm of anus and anal canal
Malignant neoplasm of anus and anal canal
Malignant neoplasm of anus and anal canal
Malignant neoplasm of anus and anal canal
Malignant neoplasm of anus and anal canal
Malignant neoplasm of anus and anal canal
Malignant neoplasm of anus and anal canal
Malignant neoplasm of anus and anal canal
Malignant neoplasm of anus and anal canal
Malignant neoplasm of liver and intrahepatic bile ducts
Malignant neoplasm of liver and intrahepatic bile ducts
Malignant neoplasm of liver and intrahepatic bile ducts
Malignant neoplasm of liver and intrahepatic bile ducts
Malignant neoplasm of liver and intrahepatic bile ducts
Malignant neoplasm of liver and intrahepatic bile ducts
Malignant neoplasm of liver and intrahepatic bile ducts
Malignant neoplasm of liver and intrahepatic bile ducts
Malignant neoplasm of liver and intrahepatic bile ducts
Malignant neoplasm of liver and intrahepatic bile ducts
Malignant neoplasm of liver and intrahepatic bile ducts
Malignant neoplasm of liver and intrahepatic bile ducts
Malignant neoplasm of liver and intrahepatic bile ducts
Malignant neoplasm of liver and intrahepatic bile ducts

For peer review only

Malignant neoplasm of liver and intrahepatic bile ducts
Malignant neoplasm of liver and intrahepatic bile ducts
Malignant neoplasm of liver and intrahepatic bile ducts
Malignant neoplasm of liver and intrahepatic bile ducts
Malignant neoplasm of liver and intrahepatic bile ducts
Malignant neoplasm of liver and intrahepatic bile ducts
Malignant neoplasm of liver and intrahepatic bile ducts
Malignant neoplasm of gallbladder
Malignant neoplasm of gallbladder
Malignant neoplasm of gallbladder
Malignant neoplasm of gallbladder
Malignant neoplasm of gallbladder
Malignant neoplasm of gallbladder
Malignant neoplasm of gallbladder
Malignant neoplasm of gallbladder
Malignant neoplasm of gallbladder
Malignant neoplasm of gallbladder
Malignant neoplasm of gallbladder
Malignant neoplasm of gallbladder
Malignant neoplasm of gallbladder
Malignant neoplasm of gallbladder
Malignant neoplasm of gallbladder
Malignant neoplasm of gallbladder
Malignant neoplasm of gallbladder
Malignant neoplasm of gallbladder
Malignant neoplasm of gallbladder
Malignant neoplasm of gallbladder
Malignant neoplasm of gallbladder
Malignant neoplasm of other and unspecified parts of biliary tract
Malignant neoplasm of other and unspecified parts of biliary tract
Malignant neoplasm of other and unspecified parts of biliary tract
Malignant neoplasm of other and unspecified parts of biliary tract
Malignant neoplasm of other and unspecified parts of biliary tract
Malignant neoplasm of other and unspecified parts of biliary tract
Malignant neoplasm of other and unspecified parts of biliary tract
Malignant neoplasm of other and unspecified parts of biliary tract
Malignant neoplasm of other and unspecified parts of biliary tract
Malignant neoplasm of other and unspecified parts of biliary tract
Malignant neoplasm of other and unspecified parts of biliary tract
Malignant neoplasm of other and unspecified parts of biliary tract
Malignant neoplasm of other and unspecified parts of biliary tract
Malignant neoplasm of other and unspecified parts of biliary tract
Malignant neoplasm of other and unspecified parts of biliary tract
Malignant neoplasm of other and unspecified parts of biliary tract
Malignant neoplasm of other and unspecified parts of biliary tract
Malignant neoplasm of other and unspecified parts of biliary tract
Malignant neoplasm of other and unspecified parts of biliary tract
Malignant neoplasm of other and unspecified parts of biliary tract
Malignant neoplasm of other and unspecified parts of biliary tract
Malignant neoplasm of pancreas

For peer review only

Malignant neoplasm of pancreas
Malignant neoplasm of pancreas
Malignant neoplasm of pancreas
Malignant neoplasm of pancreas
Malignant neoplasm of pancreas
Malignant neoplasm of pancreas
Malignant neoplasm of pancreas
Malignant neoplasm of pancreas
Malignant neoplasm of pancreas
Malignant neoplasm of pancreas
Malignant neoplasm of pancreas
Malignant neoplasm of pancreas
Malignant neoplasm of pancreas
Malignant neoplasm of pancreas
Malignant neoplasm of pancreas
Malignant neoplasm of pancreas
Malignant neoplasm of pancreas
Malignant neoplasm of pancreas
Malignant neoplasm of pancreas
Malignant neoplasm of pancreas
Malignant neoplasm of other and ill-defined digestive organs
Malignant neoplasm of other and ill-defined digestive organs
Malignant neoplasm of other and ill-defined digestive organs
Malignant neoplasm of other and ill-defined digestive organs
Malignant neoplasm of other and ill-defined digestive organs
Malignant neoplasm of other and ill-defined digestive organs
Malignant neoplasm of other and ill-defined digestive organs
Malignant neoplasm of other and ill-defined digestive organs
Malignant neoplasm of other and ill-defined digestive organs
Malignant neoplasm of other and ill-defined digestive organs
Malignant neoplasm of other and ill-defined digestive organs
Malignant neoplasm of other and ill-defined digestive organs
Malignant neoplasm of other and ill-defined digestive organs
Malignant neoplasm of other and ill-defined digestive organs
Malignant neoplasm of other and ill-defined digestive organs
Malignant neoplasm of other and ill-defined digestive organs
Malignant neoplasm of other and ill-defined digestive organs
Malignant neoplasm of other and ill-defined digestive organs
Malignant neoplasm of other and ill-defined digestive organs
Malignant neoplasm of other and ill-defined digestive organs
Malignant neoplasm of other and ill-defined digestive organs
Malignant neoplasm of nasal cavity and middle ear
Malignant neoplasm of nasal cavity and middle ear
Malignant neoplasm of nasal cavity and middle ear
Malignant neoplasm of nasal cavity and middle ear
Malignant neoplasm of nasal cavity and middle ear
Malignant neoplasm of nasal cavity and middle ear
Malignant neoplasm of nasal cavity and middle ear
Malignant neoplasm of nasal cavity and middle ear
Malignant neoplasm of nasal cavity and middle ear

For peer review only

Malignant neoplasm of nasal cavity and middle ear
Malignant neoplasm of nasal cavity and middle ear
Malignant neoplasm of nasal cavity and middle ear
Malignant neoplasm of nasal cavity and middle ear
Malignant neoplasm of nasal cavity and middle ear
Malignant neoplasm of accessory sinuses
Malignant neoplasm of accessory sinuses
Malignant neoplasm of accessory sinuses
Malignant neoplasm of accessory sinuses
Malignant neoplasm of accessory sinuses
Malignant neoplasm of accessory sinuses
Malignant neoplasm of accessory sinuses
Malignant neoplasm of accessory sinuses
Malignant neoplasm of accessory sinuses
Malignant neoplasm of accessory sinuses
Malignant neoplasm of accessory sinuses
Malignant neoplasm of accessory sinuses
Malignant neoplasm of accessory sinuses
Malignant neoplasm of accessory sinuses
Malignant neoplasm of accessory sinuses
Malignant neoplasm of accessory sinuses
Malignant neoplasm of accessory sinuses
Malignant neoplasm of accessory sinuses
Malignant neoplasm of accessory sinuses
Malignant neoplasm of accessory sinuses
Malignant neoplasm of accessory sinuses
Malignant neoplasm of larynx
Malignant neoplasm of larynx
Malignant neoplasm of larynx
Malignant neoplasm of larynx
Malignant neoplasm of larynx
Malignant neoplasm of larynx
Malignant neoplasm of larynx
Malignant neoplasm of larynx
Malignant neoplasm of larynx
Malignant neoplasm of larynx
Malignant neoplasm of larynx
Malignant neoplasm of larynx
Malignant neoplasm of larynx
Malignant neoplasm of larynx
Malignant neoplasm of larynx
Malignant neoplasm of larynx
Malignant neoplasm of larynx
Malignant neoplasm of larynx
Malignant neoplasm of larynx
Malignant neoplasm of larynx
Malignant neoplasm of larynx
Malignant neoplasm of trachea
Malignant neoplasm of trachea
Malignant neoplasm of trachea

For peer review only

Malignant neoplasm of trachea
Malignant neoplasm of trachea
Malignant neoplasm of trachea
Malignant neoplasm of trachea
Malignant neoplasm of trachea
Malignant neoplasm of trachea
Malignant neoplasm of trachea
Malignant neoplasm of trachea
Malignant neoplasm of bronchus and lung
Malignant neoplasm of bronchus and lung
Malignant neoplasm of bronchus and lung
Malignant neoplasm of bronchus and lung
Malignant neoplasm of bronchus and lung
Malignant neoplasm of bronchus and lung
Malignant neoplasm of bronchus and lung
Malignant neoplasm of bronchus and lung
Malignant neoplasm of bronchus and lung
Malignant neoplasm of bronchus and lung
Malignant neoplasm of bronchus and lung
Malignant neoplasm of bronchus and lung
Malignant neoplasm of bronchus and lung
Malignant neoplasm of bronchus and lung
Malignant neoplasm of bronchus and lung
Malignant neoplasm of bronchus and lung
Malignant neoplasm of bronchus and lung
Malignant neoplasm of bronchus and lung
Malignant neoplasm of bronchus and lung
Malignant neoplasm of bronchus and lung
Malignant neoplasm of bronchus and lung
Malignant neoplasm of thymus
Malignant neoplasm of thymus
Malignant neoplasm of thymus
Malignant neoplasm of thymus
Malignant neoplasm of thymus
Malignant neoplasm of thymus
Malignant neoplasm of thymus
Malignant neoplasm of thymus
Malignant neoplasm of thymus
Malignant neoplasm of thymus
Malignant neoplasm of thymus
Malignant neoplasm of thymus
Malignant neoplasm of heart, mediastinum and pleura
Malignant neoplasm of heart, mediastinum and pleura
Malignant neoplasm of heart, mediastinum and pleura
Malignant neoplasm of heart, mediastinum and pleura
Malignant neoplasm of heart, mediastinum and pleura
Malignant neoplasm of heart, mediastinum and pleura
Malignant neoplasm of heart, mediastinum and pleura
Malignant neoplasm of heart, mediastinum and pleura
Malignant neoplasm of heart, mediastinum and pleura

For peer review only

Malignant neoplasm of heart, mediastinum and pleura
Malignant neoplasm of heart, mediastinum and pleura
Malignant neoplasm of heart, mediastinum and pleura
Malignant neoplasm of heart, mediastinum and pleura
Malignant neoplasm of heart, mediastinum and pleura
Malignant neoplasm of heart, mediastinum and pleura
Malignant neoplasm of heart, mediastinum and pleura
Malignant neoplasm of heart, mediastinum and pleura
Malignant neoplasm of heart, mediastinum and pleura
Malignant neoplasm of heart, mediastinum and pleura
Malignant neoplasm of heart, mediastinum and pleura
Malignant neoplasm of heart, mediastinum and pleura
Malignant neoplasm of other and ill-defined sites in the respiratory system and intrathoracic organs
Malignant neoplasm of other and ill-defined sites in the respiratory system and intrathoracic organs
Malignant neoplasm of other and ill-defined sites in the respiratory system and intrathoracic organs
Malignant neoplasm of other and ill-defined sites in the respiratory system and intrathoracic organs
Malignant neoplasm of other and ill-defined sites in the respiratory system and intrathoracic organs
Malignant neoplasm of other and ill-defined sites in the respiratory system and intrathoracic organs
Malignant neoplasm of other and ill-defined sites in the respiratory system and intrathoracic organs
Malignant neoplasm of other and ill-defined sites in the respiratory system and intrathoracic organs
Malignant neoplasm of other and ill-defined sites in the respiratory system and intrathoracic organs
Malignant neoplasm of other and ill-defined sites in the respiratory system and intrathoracic organs
Malignant neoplasm of other and ill-defined sites in the respiratory system and intrathoracic organs
Malignant neoplasm of other and ill-defined sites in the respiratory system and intrathoracic organs
Malignant neoplasm of bone and articular cartilage of limbs
Malignant neoplasm of bone and articular cartilage of limbs
Malignant neoplasm of bone and articular cartilage of limbs
Malignant neoplasm of bone and articular cartilage of limbs
Malignant neoplasm of bone and articular cartilage of limbs
Malignant neoplasm of bone and articular cartilage of limbs
Malignant neoplasm of bone and articular cartilage of limbs
Malignant neoplasm of bone and articular cartilage of limbs
Malignant neoplasm of bone and articular cartilage of limbs
Malignant neoplasm of bone and articular cartilage of limbs
Malignant neoplasm of bone and articular cartilage of limbs
Malignant neoplasm of bone and articular cartilage of limbs
Malignant neoplasm of bone and articular cartilage of limbs
Malignant neoplasm of bone and articular cartilage of limbs
Malignant neoplasm of bone and articular cartilage of limbs
Malignant neoplasm of bone and articular cartilage of limbs
Malignant neoplasm of bone and articular cartilage of limbs
Malignant neoplasm of bone and articular cartilage of limbs
Malignant neoplasm of bone and articular cartilage of limbs
Malignant neoplasm of bone and articular cartilage of other and unspecified sites
Malignant neoplasm of bone and articular cartilage of other and unspecified sites
Malignant neoplasm of bone and articular cartilage of other and unspecified sites
Malignant neoplasm of bone and articular cartilage of other and unspecified sites
Malignant neoplasm of bone and articular cartilage of other and unspecified sites
Malignant neoplasm of bone and articular cartilage of other and unspecified sites
Malignant neoplasm of bone and articular cartilage of other and unspecified sites

For peer review only

Malignant neoplasm of bone and articular cartilage of other and unspecified sites
Malignant neoplasm of bone and articular cartilage of other and unspecified sites
Malignant neoplasm of bone and articular cartilage of other and unspecified sites
Malignant neoplasm of bone and articular cartilage of other and unspecified sites
Malignant neoplasm of bone and articular cartilage of other and unspecified sites
Malignant neoplasm of bone and articular cartilage of other and unspecified sites
Malignant neoplasm of bone and articular cartilage of other and unspecified sites
Malignant neoplasm of bone and articular cartilage of other and unspecified sites
Malignant neoplasm of bone and articular cartilage of other and unspecified sites
Malignant neoplasm of bone and articular cartilage of other and unspecified sites
Malignant neoplasm of bone and articular cartilage of other and unspecified sites
Malignant neoplasm of bone and articular cartilage of other and unspecified sites
Malignant neoplasm of bone and articular cartilage of other and unspecified sites
Malignant neoplasm of bone and articular cartilage of other and unspecified sites
Malignant melanoma of skin
Malignant melanoma of skin
Malignant melanoma of skin
Malignant melanoma of skin
Malignant melanoma of skin
Malignant melanoma of skin
Malignant melanoma of skin
Malignant melanoma of skin
Malignant melanoma of skin
Malignant melanoma of skin
Malignant melanoma of skin
Malignant melanoma of skin
Malignant melanoma of skin
Malignant melanoma of skin
Malignant melanoma of skin
Malignant melanoma of skin
Malignant melanoma of skin
Malignant melanoma of skin
Malignant melanoma of skin
Malignant melanoma of skin
Malignant melanoma of skin
Other malignant neoplasms of skin
Other malignant neoplasms of skin
Other malignant neoplasms of skin
Other malignant neoplasms of skin
Other malignant neoplasms of skin
Other malignant neoplasms of skin
Other malignant neoplasms of skin
Other malignant neoplasms of skin
Other malignant neoplasms of skin
Other malignant neoplasms of skin
Other malignant neoplasms of skin
Other malignant neoplasms of skin
Other malignant neoplasms of skin
Other malignant neoplasms of skin
Other malignant neoplasms of skin

For peer review only

Other malignant neoplasms of skin
Other malignant neoplasms of skin
Other malignant neoplasms of skin
Other malignant neoplasms of skin
Other malignant neoplasms of skin
Other malignant neoplasms of skin
Mesothelioma
Mesothelioma
Mesothelioma
Mesothelioma
Mesothelioma
Mesothelioma
Mesothelioma
Mesothelioma
Mesothelioma
Mesothelioma
Mesothelioma
Mesothelioma
Mesothelioma
Mesothelioma
Mesothelioma
Mesothelioma
Mesothelioma
Mesothelioma
Mesothelioma
Mesothelioma
Kaposi's sarcoma
Kaposi's sarcoma
Kaposi's sarcoma
Kaposi's sarcoma
Kaposi's sarcoma
Kaposi's sarcoma
Kaposi's sarcoma
Malignant neoplasm of peripheral nerves and autonomic nervous system
Malignant neoplasm of peripheral nerves and autonomic nervous system
Malignant neoplasm of peripheral nerves and autonomic nervous system
Malignant neoplasm of peripheral nerves and autonomic nervous system
Malignant neoplasm of peripheral nerves and autonomic nervous system
Malignant neoplasm of peripheral nerves and autonomic nervous system
Malignant neoplasm of peripheral nerves and autonomic nervous system
Malignant neoplasm of peripheral nerves and autonomic nervous system
Malignant neoplasm of peripheral nerves and autonomic nervous system
Malignant neoplasm of peripheral nerves and autonomic nervous system
Malignant neoplasm of peripheral nerves and autonomic nervous system
Malignant neoplasm of peripheral nerves and autonomic nervous system
Malignant neoplasm of retroperitoneum and peritoneum
Malignant neoplasm of retroperitoneum and peritoneum
Malignant neoplasm of retroperitoneum and peritoneum
Malignant neoplasm of retroperitoneum and peritoneum
Malignant neoplasm of retroperitoneum and peritoneum

For peer review only

Malignant neoplasm of retroperitoneum and peritoneum
Malignant neoplasm of retroperitoneum and peritoneum
Malignant neoplasm of retroperitoneum and peritoneum
Malignant neoplasm of retroperitoneum and peritoneum
Malignant neoplasm of retroperitoneum and peritoneum
Malignant neoplasm of retroperitoneum and peritoneum
Malignant neoplasm of retroperitoneum and peritoneum
Malignant neoplasm of retroperitoneum and peritoneum
Malignant neoplasm of retroperitoneum and peritoneum
Malignant neoplasm of retroperitoneum and peritoneum
Malignant neoplasm of retroperitoneum and peritoneum
Malignant neoplasm of retroperitoneum and peritoneum
Malignant neoplasm of retroperitoneum and peritoneum
Malignant neoplasm of retroperitoneum and peritoneum
Malignant neoplasm of retroperitoneum and peritoneum
Malignant neoplasm of retroperitoneum and peritoneum
Malignant neoplasm of other connective and soft tissue
Malignant neoplasm of other connective and soft tissue
Malignant neoplasm of other connective and soft tissue
Malignant neoplasm of other connective and soft tissue
Malignant neoplasm of other connective and soft tissue
Malignant neoplasm of other connective and soft tissue
Malignant neoplasm of other connective and soft tissue
Malignant neoplasm of other connective and soft tissue
Malignant neoplasm of other connective and soft tissue
Malignant neoplasm of other connective and soft tissue
Malignant neoplasm of other connective and soft tissue
Malignant neoplasm of other connective and soft tissue
Malignant neoplasm of other connective and soft tissue
Malignant neoplasm of other connective and soft tissue
Malignant neoplasm of other connective and soft tissue
Malignant neoplasm of other connective and soft tissue
Malignant neoplasm of other connective and soft tissue
Malignant neoplasm of other connective and soft tissue
Malignant neoplasm of other connective and soft tissue
Malignant neoplasm of other connective and soft tissue
Malignant neoplasm of other connective and soft tissue
Malignant neoplasm of breast
Malignant neoplasm of breast
Malignant neoplasm of breast
Malignant neoplasm of breast
Malignant neoplasm of breast
Malignant neoplasm of breast
Malignant neoplasm of breast
Malignant neoplasm of breast
Malignant neoplasm of breast
Malignant neoplasm of breast
Malignant neoplasm of breast
Malignant neoplasm of breast
Malignant neoplasm of breast

For peer review only

Malignant neoplasm of breast
Malignant neoplasm of breast
Malignant neoplasm of breast
Malignant neoplasm of breast
Malignant neoplasm of breast
Malignant neoplasm of breast
Malignant neoplasm of breast
Malignant neoplasm of breast
Malignant neoplasm of vulva
Malignant neoplasm of vulva
Malignant neoplasm of vulva
Malignant neoplasm of vulva
Malignant neoplasm of vulva
Malignant neoplasm of vulva
Malignant neoplasm of vulva
Malignant neoplasm of vulva
Malignant neoplasm of vulva
Malignant neoplasm of vulva
Malignant neoplasm of vulva
Malignant neoplasm of vulva
Malignant neoplasm of vulva
Malignant neoplasm of vulva
Malignant neoplasm of vulva
Malignant neoplasm of vulva
Malignant neoplasm of vulva
Malignant neoplasm of vulva
Malignant neoplasm of vulva
Malignant neoplasm of vulva
Malignant neoplasm of vulva
Malignant neoplasm of vagina
Malignant neoplasm of vagina
Malignant neoplasm of vagina
Malignant neoplasm of vagina
Malignant neoplasm of vagina
Malignant neoplasm of vagina
Malignant neoplasm of vagina
Malignant neoplasm of vagina
Malignant neoplasm of vagina
Malignant neoplasm of vagina
Malignant neoplasm of vagina
Malignant neoplasm of vagina
Malignant neoplasm of vagina
Malignant neoplasm of vagina
Malignant neoplasm of vagina
Malignant neoplasm of vagina
Malignant neoplasm of vagina
Malignant neoplasm of vagina
Malignant neoplasm of vagina
Malignant neoplasm of vagina
Malignant neoplasm of vagina

For peer review only

Malignant neoplasm of cervix uteri
Malignant neoplasm of cervix uteri
Malignant neoplasm of cervix uteri
Malignant neoplasm of cervix uteri
Malignant neoplasm of cervix uteri
Malignant neoplasm of cervix uteri
Malignant neoplasm of cervix uteri
Malignant neoplasm of cervix uteri
Malignant neoplasm of cervix uteri
Malignant neoplasm of cervix uteri
Malignant neoplasm of cervix uteri
Malignant neoplasm of cervix uteri
Malignant neoplasm of cervix uteri
Malignant neoplasm of cervix uteri
Malignant neoplasm of cervix uteri
Malignant neoplasm of cervix uteri
Malignant neoplasm of cervix uteri
Malignant neoplasm of cervix uteri
Malignant neoplasm of cervix uteri
Malignant neoplasm of cervix uteri
Malignant neoplasm of cervix uteri
Malignant neoplasm of corpus uteri
Malignant neoplasm of corpus uteri
Malignant neoplasm of corpus uteri
Malignant neoplasm of corpus uteri
Malignant neoplasm of corpus uteri
Malignant neoplasm of corpus uteri
Malignant neoplasm of corpus uteri
Malignant neoplasm of corpus uteri
Malignant neoplasm of corpus uteri
Malignant neoplasm of corpus uteri
Malignant neoplasm of corpus uteri
Malignant neoplasm of corpus uteri
Malignant neoplasm of corpus uteri
Malignant neoplasm of corpus uteri
Malignant neoplasm of corpus uteri
Malignant neoplasm of corpus uteri
Malignant neoplasm of corpus uteri
Malignant neoplasm of corpus uteri
Malignant neoplasm of corpus uteri
Malignant neoplasm of corpus uteri
Malignant neoplasm of corpus uteri
Malignant neoplasm of uterus, part unspecified
Malignant neoplasm of uterus, part unspecified
Malignant neoplasm of uterus, part unspecified
Malignant neoplasm of uterus, part unspecified
Malignant neoplasm of uterus, part unspecified
Malignant neoplasm of uterus, part unspecified
Malignant neoplasm of uterus, part unspecified
Malignant neoplasm of uterus, part unspecified

For peer review only

Malignant neoplasm of uterus, part unspecified
Malignant neoplasm of uterus, part unspecified
Malignant neoplasm of uterus, part unspecified
Malignant neoplasm of uterus, part unspecified
Malignant neoplasm of uterus, part unspecified
Malignant neoplasm of uterus, part unspecified
Malignant neoplasm of uterus, part unspecified
Malignant neoplasm of uterus, part unspecified
Malignant neoplasm of uterus, part unspecified
Malignant neoplasm of uterus, part unspecified
Malignant neoplasm of uterus, part unspecified
Malignant neoplasm of uterus, part unspecified
Malignant neoplasm of uterus, part unspecified
Malignant neoplasm of ovary
Malignant neoplasm of ovary
Malignant neoplasm of ovary
Malignant neoplasm of ovary
Malignant neoplasm of ovary
Malignant neoplasm of ovary
Malignant neoplasm of ovary
Malignant neoplasm of ovary
Malignant neoplasm of ovary
Malignant neoplasm of ovary
Malignant neoplasm of ovary
Malignant neoplasm of ovary
Malignant neoplasm of ovary
Malignant neoplasm of ovary
Malignant neoplasm of ovary
Malignant neoplasm of ovary
Malignant neoplasm of ovary
Malignant neoplasm of ovary
Malignant neoplasm of ovary
Malignant neoplasm of ovary
Malignant neoplasm of ovary
Malignant neoplasm of other and unspecified female genital organs
Malignant neoplasm of other and unspecified female genital organs
Malignant neoplasm of other and unspecified female genital organs
Malignant neoplasm of other and unspecified female genital organs
Malignant neoplasm of other and unspecified female genital organs
Malignant neoplasm of other and unspecified female genital organs
Malignant neoplasm of other and unspecified female genital organs
Malignant neoplasm of other and unspecified female genital organs
Malignant neoplasm of other and unspecified female genital organs
Malignant neoplasm of other and unspecified female genital organs
Malignant neoplasm of other and unspecified female genital organs
Malignant neoplasm of other and unspecified female genital organs
Malignant neoplasm of other and unspecified female genital organs
Malignant neoplasm of other and unspecified female genital organs
Malignant neoplasm of other and unspecified female genital organs
Malignant neoplasm of other and unspecified female genital organs

For peer review only

Malignant neoplasm of other and unspecified female genital organs
Malignant neoplasm of other and unspecified female genital organs
Malignant neoplasm of other and unspecified female genital organs
Malignant neoplasm of other and unspecified female genital organs
Malignant neoplasm of other and unspecified female genital organs
Malignant neoplasm of penis
Malignant neoplasm of penis
Malignant neoplasm of penis
Malignant neoplasm of penis
Malignant neoplasm of penis
Malignant neoplasm of penis
Malignant neoplasm of penis
Malignant neoplasm of penis
Malignant neoplasm of penis
Malignant neoplasm of penis
Malignant neoplasm of penis
Malignant neoplasm of penis
Malignant neoplasm of penis
Malignant neoplasm of penis
Malignant neoplasm of penis
Malignant neoplasm of penis
Malignant neoplasm of penis
Malignant neoplasm of penis
Malignant neoplasm of penis
Malignant neoplasm of prostate
Malignant neoplasm of prostate
Malignant neoplasm of prostate
Malignant neoplasm of prostate
Malignant neoplasm of prostate
Malignant neoplasm of prostate
Malignant neoplasm of prostate
Malignant neoplasm of prostate
Malignant neoplasm of prostate
Malignant neoplasm of prostate
Malignant neoplasm of prostate
Malignant neoplasm of prostate
Malignant neoplasm of prostate
Malignant neoplasm of prostate
Malignant neoplasm of prostate
Malignant neoplasm of prostate
Malignant neoplasm of prostate
Malignant neoplasm of prostate
Malignant neoplasm of prostate
Malignant neoplasm of prostate
Malignant neoplasm of prostate
Malignant neoplasm of testis
Malignant neoplasm of testis
Malignant neoplasm of testis
Malignant neoplasm of testis
Malignant neoplasm of testis

For peer review only

Malignant neoplasm of testis
Malignant neoplasm of testis
Malignant neoplasm of testis
Malignant neoplasm of testis
Malignant neoplasm of testis
Malignant neoplasm of testis
Malignant neoplasm of testis
Malignant neoplasm of testis
Malignant neoplasm of testis
Malignant neoplasm of testis
Malignant neoplasm of testis
Malignant neoplasm of testis
Malignant neoplasm of testis
Malignant neoplasm of testis
Malignant neoplasm of testis
Malignant neoplasm of other and unspecified male genital organs
Malignant neoplasm of other and unspecified male genital organs
Malignant neoplasm of other and unspecified male genital organs
Malignant neoplasm of other and unspecified male genital organs
Malignant neoplasm of other and unspecified male genital organs
Malignant neoplasm of other and unspecified male genital organs
Malignant neoplasm of other and unspecified male genital organs
Malignant neoplasm of other and unspecified male genital organs
Malignant neoplasm of kidney, except renal pelvis
Malignant neoplasm of kidney, except renal pelvis
Malignant neoplasm of kidney, except renal pelvis
Malignant neoplasm of kidney, except renal pelvis
Malignant neoplasm of kidney, except renal pelvis
Malignant neoplasm of kidney, except renal pelvis
Malignant neoplasm of kidney, except renal pelvis
Malignant neoplasm of kidney, except renal pelvis
Malignant neoplasm of kidney, except renal pelvis
Malignant neoplasm of kidney, except renal pelvis
Malignant neoplasm of kidney, except renal pelvis
Malignant neoplasm of kidney, except renal pelvis
Malignant neoplasm of kidney, except renal pelvis
Malignant neoplasm of kidney, except renal pelvis
Malignant neoplasm of kidney, except renal pelvis
Malignant neoplasm of kidney, except renal pelvis
Malignant neoplasm of kidney, except renal pelvis
Malignant neoplasm of kidney, except renal pelvis
Malignant neoplasm of kidney, except renal pelvis
Malignant neoplasm of kidney, except renal pelvis
Malignant neoplasm of kidney, except renal pelvis
Malignant neoplasm of renal pelvis
Malignant neoplasm of renal pelvis
Malignant neoplasm of renal pelvis
Malignant neoplasm of renal pelvis
Malignant neoplasm of renal pelvis
Malignant neoplasm of renal pelvis

For peer review only

Malignant neoplasm of renal pelvis
Malignant neoplasm of renal pelvis
Malignant neoplasm of renal pelvis
Malignant neoplasm of renal pelvis
Malignant neoplasm of renal pelvis
Malignant neoplasm of renal pelvis
Malignant neoplasm of renal pelvis
Malignant neoplasm of renal pelvis
Malignant neoplasm of renal pelvis
Malignant neoplasm of renal pelvis
Malignant neoplasm of renal pelvis
Malignant neoplasm of renal pelvis
Malignant neoplasm of ureter
Malignant neoplasm of ureter
Malignant neoplasm of ureter
Malignant neoplasm of ureter
Malignant neoplasm of ureter
Malignant neoplasm of ureter
Malignant neoplasm of ureter
Malignant neoplasm of ureter
Malignant neoplasm of ureter
Malignant neoplasm of ureter
Malignant neoplasm of ureter
Malignant neoplasm of ureter
Malignant neoplasm of ureter
Malignant neoplasm of ureter
Malignant neoplasm of ureter
Malignant neoplasm of ureter
Malignant neoplasm of ureter
Malignant neoplasm of bladder
Malignant neoplasm of bladder
Malignant neoplasm of bladder
Malignant neoplasm of bladder
Malignant neoplasm of bladder
Malignant neoplasm of bladder
Malignant neoplasm of bladder
Malignant neoplasm of bladder
Malignant neoplasm of bladder
Malignant neoplasm of bladder
Malignant neoplasm of bladder
Malignant neoplasm of bladder
Malignant neoplasm of bladder
Malignant neoplasm of bladder
Malignant neoplasm of bladder
Malignant neoplasm of bladder
Malignant neoplasm of bladder
Malignant neoplasm of bladder
Malignant neoplasm of bladder
Malignant neoplasm of bladder
Malignant neoplasm of bladder

For peer review only

Malignant neoplasm of other and unspecified urinary organs
Malignant neoplasm of other and unspecified urinary organs
Malignant neoplasm of other and unspecified urinary organs
Malignant neoplasm of other and unspecified urinary organs
Malignant neoplasm of other and unspecified urinary organs
Malignant neoplasm of other and unspecified urinary organs
Malignant neoplasm of other and unspecified urinary organs
Malignant neoplasm of other and unspecified urinary organs
Malignant neoplasm of other and unspecified urinary organs
Malignant neoplasm of other and unspecified urinary organs
Malignant neoplasm of other and unspecified urinary organs
Malignant neoplasm of other and unspecified urinary organs
Malignant neoplasm of other and unspecified urinary organs
Malignant neoplasm of other and unspecified urinary organs
Malignant neoplasm of other and unspecified urinary organs
Malignant neoplasm of other and unspecified urinary organs
Malignant neoplasm of other and unspecified urinary organs
Malignant neoplasm of eye and adnexa
Malignant neoplasm of eye and adnexa
Malignant neoplasm of eye and adnexa
Malignant neoplasm of eye and adnexa
Malignant neoplasm of eye and adnexa
Malignant neoplasm of eye and adnexa
Malignant neoplasm of eye and adnexa
Malignant neoplasm of eye and adnexa
Malignant neoplasm of eye and adnexa
Malignant neoplasm of eye and adnexa
Malignant neoplasm of eye and adnexa
Malignant neoplasm of eye and adnexa
Malignant neoplasm of eye and adnexa
Malignant neoplasm of eye and adnexa
Malignant neoplasm of eye and adnexa
Malignant neoplasm of eye and adnexa
Malignant neoplasm of eye and adnexa
Malignant neoplasm of eye and adnexa
Malignant neoplasm of eye and adnexa
Malignant neoplasm of eye and adnexa
Malignant neoplasm of meninges
Malignant neoplasm of meninges
Malignant neoplasm of meninges
Malignant neoplasm of meninges
Malignant neoplasm of meninges
Malignant neoplasm of meninges
Malignant neoplasm of meninges
Malignant neoplasm of meninges
Malignant neoplasm of meninges
Malignant neoplasm of meninges
Malignant neoplasm of meninges
Malignant neoplasm of meninges
Malignant neoplasm of meninges

For peer review only

Malignant neoplasm of meninges
Malignant neoplasm of meninges
Malignant neoplasm of meninges
Malignant neoplasm of meninges
Malignant neoplasm of meninges
Malignant neoplasm of brain
Malignant neoplasm of brain
Malignant neoplasm of brain
Malignant neoplasm of brain
Malignant neoplasm of brain
Malignant neoplasm of brain
Malignant neoplasm of brain
Malignant neoplasm of brain
Malignant neoplasm of brain
Malignant neoplasm of brain
Malignant neoplasm of brain
Malignant neoplasm of brain
Malignant neoplasm of brain
Malignant neoplasm of brain
Malignant neoplasm of brain
Malignant neoplasm of brain
Malignant neoplasm of brain
Malignant neoplasm of brain
Malignant neoplasm of brain
Malignant neoplasm of brain
Malignant neoplasm of brain
Malignant neoplasm of spinal cord, cranial nerves and other parts of central nervous system
Malignant neoplasm of spinal cord, cranial nerves and other parts of central nervous system
Malignant neoplasm of spinal cord, cranial nerves and other parts of central nervous system
Malignant neoplasm of spinal cord, cranial nerves and other parts of central nervous system
Malignant neoplasm of spinal cord, cranial nerves and other parts of central nervous system
Malignant neoplasm of spinal cord, cranial nerves and other parts of central nervous system
Malignant neoplasm of spinal cord, cranial nerves and other parts of central nervous system
Malignant neoplasm of spinal cord, cranial nerves and other parts of central nervous system
Malignant neoplasm of spinal cord, cranial nerves and other parts of central nervous system
Malignant neoplasm of spinal cord, cranial nerves and other parts of central nervous system
Malignant neoplasm of spinal cord, cranial nerves and other parts of central nervous system
Malignant neoplasm of spinal cord, cranial nerves and other parts of central nervous system
Malignant neoplasm of spinal cord, cranial nerves and other parts of central nervous system
Malignant neoplasm of spinal cord, cranial nerves and other parts of central nervous system
Malignant neoplasm of spinal cord, cranial nerves and other parts of central nervous system
Malignant neoplasm of thyroid gland
Malignant neoplasm of thyroid gland
Malignant neoplasm of thyroid gland
Malignant neoplasm of thyroid gland
Malignant neoplasm of thyroid gland
Malignant neoplasm of thyroid gland
Malignant neoplasm of thyroid gland
Malignant neoplasm of thyroid gland
Malignant neoplasm of thyroid gland

For peer review only

Malignant neoplasm of thyroid gland
Malignant neoplasm of thyroid gland
Malignant neoplasm of thyroid gland
Malignant neoplasm of thyroid gland
Malignant neoplasm of thyroid gland
Malignant neoplasm of thyroid gland
Malignant neoplasm of thyroid gland
Malignant neoplasm of thyroid gland
Malignant neoplasm of thyroid gland
Malignant neoplasm of thyroid gland
Malignant neoplasm of thyroid gland
Malignant neoplasm of thyroid gland
Malignant neoplasm of adrenal gland
Malignant neoplasm of adrenal gland
Malignant neoplasm of adrenal gland
Malignant neoplasm of adrenal gland
Malignant neoplasm of adrenal gland
Malignant neoplasm of adrenal gland
Malignant neoplasm of adrenal gland
Malignant neoplasm of adrenal gland
Malignant neoplasm of adrenal gland
Malignant neoplasm of adrenal gland
Malignant neoplasm of adrenal gland
Malignant neoplasm of adrenal gland
Malignant neoplasm of adrenal gland
Malignant neoplasm of adrenal gland
Malignant neoplasm of adrenal gland
Malignant neoplasm of adrenal gland
Malignant neoplasm of adrenal gland
Malignant neoplasm of adrenal gland
Malignant neoplasm of adrenal gland
Malignant neoplasm of adrenal gland
Malignant neoplasm of other endocrine glands and related structures
Malignant neoplasm of other endocrine glands and related structures
Malignant neoplasm of other endocrine glands and related structures
Malignant neoplasm of other endocrine glands and related structures
Malignant neoplasm of other endocrine glands and related structures
Malignant neoplasm of other endocrine glands and related structures
Malignant neoplasm of other endocrine glands and related structures
Malignant neoplasm of other endocrine glands and related structures
Malignant neoplasm of other endocrine glands and related structures
Malignant neoplasm of other endocrine glands and related structures
Malignant neoplasm of other endocrine glands and related structures
Malignant neoplasm of other endocrine glands and related structures
Malignant neoplasm of other endocrine glands and related structures
Malignant neoplasm of other and ill-defined sites
Malignant neoplasm of other and ill-defined sites
Malignant neoplasm of other and ill-defined sites
Malignant neoplasm of other and ill-defined sites
Malignant neoplasm of other and ill-defined sites

For peer review only

Malignant neoplasm of other and ill-defined sites
Malignant neoplasm of other and ill-defined sites
Malignant neoplasm of other and ill-defined sites
Malignant neoplasm of other and ill-defined sites
Malignant neoplasm of other and ill-defined sites
Malignant neoplasm of other and ill-defined sites
Malignant neoplasm of other and ill-defined sites
Malignant neoplasm of other and ill-defined sites
Malignant neoplasm of other and ill-defined sites
Malignant neoplasm of other and ill-defined sites
Malignant neoplasm of other and ill-defined sites
Malignant neoplasm of other and ill-defined sites
Malignant neoplasm of other and ill-defined sites
Malignant neoplasm of other and ill-defined sites
Malignant neoplasm of other and ill-defined sites
Malignant neoplasm of other and ill-defined sites
Secondary and unspecified malignant neoplasm of lymph nodes
Secondary malignant neoplasm of respiratory and digestive organs
Secondary malignant neoplasm of respiratory and digestive organs
Secondary malignant neoplasm of respiratory and digestive organs
Secondary malignant neoplasm of respiratory and digestive organs
Secondary malignant neoplasm of respiratory and digestive organs
Secondary malignant neoplasm of other sites
Secondary malignant neoplasm of other sites
Secondary malignant neoplasm of other sites
Secondary malignant neoplasm of other sites
Malignant neoplasm without specification of site
Malignant neoplasm without specification of site
Malignant neoplasm without specification of site
Malignant neoplasm without specification of site
Malignant neoplasm without specification of site
Malignant neoplasm without specification of site
Malignant neoplasm without specification of site
Malignant neoplasm without specification of site
Malignant neoplasm without specification of site
Malignant neoplasm without specification of site
Malignant neoplasm without specification of site
Malignant neoplasm without specification of site
Malignant neoplasm without specification of site
Malignant neoplasm without specification of site
Malignant neoplasm without specification of site
Malignant neoplasm without specification of site
Malignant neoplasm without specification of site
Malignant neoplasm without specification of site
Malignant neoplasm without specification of site
Malignant neoplasm without specification of site
Malignant neoplasm without specification of site
Hodgkin's disease
Hodgkin's disease
Hodgkin's disease

For peer review only

Hodgkin's disease
Hodgkin's disease
Hodgkin's disease
Hodgkin's disease
Hodgkin's disease
Hodgkin's disease
Hodgkin's disease
Hodgkin's disease
Hodgkin's disease
Hodgkin's disease
Hodgkin's disease
Hodgkin's disease
Hodgkin's disease
Hodgkin's disease
Hodgkin's disease
Hodgkin's disease
Hodgkin's disease
Hodgkin's disease
Follicular [nodular] non-Hodgkin's lymphoma
Follicular [nodular] non-Hodgkin's lymphoma
Follicular [nodular] non-Hodgkin's lymphoma
Follicular [nodular] non-Hodgkin's lymphoma
Follicular [nodular] non-Hodgkin's lymphoma
Follicular [nodular] non-Hodgkin's lymphoma
Follicular [nodular] non-Hodgkin's lymphoma
Follicular [nodular] non-Hodgkin's lymphoma
Follicular [nodular] non-Hodgkin's lymphoma
Follicular [nodular] non-Hodgkin's lymphoma
Follicular [nodular] non-Hodgkin's lymphoma
Follicular [nodular] non-Hodgkin's lymphoma
Follicular [nodular] non-Hodgkin's lymphoma
Follicular [nodular] non-Hodgkin's lymphoma
Follicular [nodular] non-Hodgkin's lymphoma
Follicular [nodular] non-Hodgkin's lymphoma
Follicular [nodular] non-Hodgkin's lymphoma
Follicular [nodular] non-Hodgkin's lymphoma
Diffuse non-Hodgkin's lymphoma
Diffuse non-Hodgkin's lymphoma
Diffuse non-Hodgkin's lymphoma
Diffuse non-Hodgkin's lymphoma
Diffuse non-Hodgkin's lymphoma
Diffuse non-Hodgkin's lymphoma
Diffuse non-Hodgkin's lymphoma
Diffuse non-Hodgkin's lymphoma
Diffuse non-Hodgkin's lymphoma
Diffuse non-Hodgkin's lymphoma
Diffuse non-Hodgkin's lymphoma
Diffuse non-Hodgkin's lymphoma
Diffuse non-Hodgkin's lymphoma
Diffuse non-Hodgkin's lymphoma

For peer review only

Diffuse non-Hodgkin's lymphoma
Diffuse non-Hodgkin's lymphoma
Diffuse non-Hodgkin's lymphoma
Diffuse non-Hodgkin's lymphoma
Diffuse non-Hodgkin's lymphoma
Diffuse non-Hodgkin's lymphoma
Diffuse non-Hodgkin's lymphoma
Peripheral and cutaneous T-cell lymphomas
Peripheral and cutaneous T-cell lymphomas
Peripheral and cutaneous T-cell lymphomas
Peripheral and cutaneous T-cell lymphomas
Peripheral and cutaneous T-cell lymphomas
Peripheral and cutaneous T-cell lymphomas
Peripheral and cutaneous T-cell lymphomas
Peripheral and cutaneous T-cell lymphomas
Peripheral and cutaneous T-cell lymphomas
Peripheral and cutaneous T-cell lymphomas
Peripheral and cutaneous T-cell lymphomas
Peripheral and cutaneous T-cell lymphomas
Peripheral and cutaneous T-cell lymphomas
Peripheral and cutaneous T-cell lymphomas
Peripheral and cutaneous T-cell lymphomas
Peripheral and cutaneous T-cell lymphomas
Peripheral and cutaneous T-cell lymphomas
Peripheral and cutaneous T-cell lymphomas
Peripheral and cutaneous T-cell lymphomas
Peripheral and cutaneous T-cell lymphomas
Other and unspecified types of non-Hodgkin's lymphoma
Other and unspecified types of non-Hodgkin's lymphoma
Other and unspecified types of non-Hodgkin's lymphoma
Other and unspecified types of non-Hodgkin's lymphoma
Other and unspecified types of non-Hodgkin's lymphoma
Other and unspecified types of non-Hodgkin's lymphoma
Other and unspecified types of non-Hodgkin's lymphoma
Other and unspecified types of non-Hodgkin's lymphoma
Other and unspecified types of non-Hodgkin's lymphoma
Other and unspecified types of non-Hodgkin's lymphoma
Other and unspecified types of non-Hodgkin's lymphoma
Other and unspecified types of non-Hodgkin's lymphoma
Other and unspecified types of non-Hodgkin's lymphoma
Other and unspecified types of non-Hodgkin's lymphoma
Other and unspecified types of non-Hodgkin's lymphoma
Other and unspecified types of non-Hodgkin's lymphoma
Other and unspecified types of non-Hodgkin's lymphoma
Other and unspecified types of non-Hodgkin's lymphoma
Other and unspecified types of non-Hodgkin's lymphoma
Other and unspecified types of non-Hodgkin's lymphoma
Other and unspecified types of non-Hodgkin's lymphoma
Malignant immunoproliferative diseases
Malignant immunoproliferative diseases

For peer review only

Malignant immunoproliferative diseases
Malignant immunoproliferative diseases
Malignant immunoproliferative diseases
Malignant immunoproliferative diseases
Malignant immunoproliferative diseases
Malignant immunoproliferative diseases
Malignant immunoproliferative diseases
Malignant immunoproliferative diseases
Malignant immunoproliferative diseases
Malignant immunoproliferative diseases
Malignant immunoproliferative diseases
Malignant immunoproliferative diseases
Malignant immunoproliferative diseases
Malignant immunoproliferative diseases
Malignant immunoproliferative diseases
Multiple myeloma and malignant plasma cell neoplasms
Multiple myeloma and malignant plasma cell neoplasms
Multiple myeloma and malignant plasma cell neoplasms
Multiple myeloma and malignant plasma cell neoplasms
Multiple myeloma and malignant plasma cell neoplasms
Multiple myeloma and malignant plasma cell neoplasms
Multiple myeloma and malignant plasma cell neoplasms
Multiple myeloma and malignant plasma cell neoplasms
Multiple myeloma and malignant plasma cell neoplasms
Multiple myeloma and malignant plasma cell neoplasms
Multiple myeloma and malignant plasma cell neoplasms
Multiple myeloma and malignant plasma cell neoplasms
Multiple myeloma and malignant plasma cell neoplasms
Multiple myeloma and malignant plasma cell neoplasms
Multiple myeloma and malignant plasma cell neoplasms
Multiple myeloma and malignant plasma cell neoplasms
Multiple myeloma and malignant plasma cell neoplasms
Multiple myeloma and malignant plasma cell neoplasms
Multiple myeloma and malignant plasma cell neoplasms
Multiple myeloma and malignant plasma cell neoplasms
Multiple myeloma and malignant plasma cell neoplasms
Lymphoid leukaemia
Lymphoid leukaemia
Lymphoid leukaemia
Lymphoid leukaemia
Lymphoid leukaemia
Lymphoid leukaemia
Lymphoid leukaemia
Lymphoid leukaemia
Lymphoid leukaemia
Lymphoid leukaemia
Lymphoid leukaemia
Lymphoid leukaemia
Lymphoid leukaemia
Lymphoid leukaemia

For peer review only

Lymphoid leukaemia
Lymphoid leukaemia
Lymphoid leukaemia
Lymphoid leukaemia
Lymphoid leukaemia
Lymphoid leukaemia
Lymphoid leukaemia
Myeloid leukaemia
Myeloid leukaemia
Myeloid leukaemia
Myeloid leukaemia
Myeloid leukaemia
Myeloid leukaemia
Myeloid leukaemia
Myeloid leukaemia
Myeloid leukaemia
Myeloid leukaemia
Myeloid leukaemia
Myeloid leukaemia
Myeloid leukaemia
Myeloid leukaemia
Myeloid leukaemia
Myeloid leukaemia
Myeloid leukaemia
Myeloid leukaemia
Myeloid leukaemia
Myeloid leukaemia
Myeloid leukaemia
Monocytic leukaemia
Monocytic leukaemia
Monocytic leukaemia
Monocytic leukaemia
Monocytic leukaemia
Monocytic leukaemia
Monocytic leukaemia
Monocytic leukaemia
Monocytic leukaemia
Monocytic leukaemia
Monocytic leukaemia
Monocytic leukaemia
Monocytic leukaemia
Monocytic leukaemia
Monocytic leukaemia
Monocytic leukaemia
Other leukaemias of specified cell type
Other leukaemias of specified cell type
Other leukaemias of specified cell type
Other leukaemias of specified cell type
Other leukaemias of specified cell type
Other leukaemias of specified cell type

For peer review only

Other leukaemias of specified cell type
Other leukaemias of specified cell type
Other leukaemias of specified cell type
Other leukaemias of specified cell type
Leukaemia of unspecified cell type
Leukaemia of unspecified cell type
Leukaemia of unspecified cell type
Leukaemia of unspecified cell type
Leukaemia of unspecified cell type
Leukaemia of unspecified cell type
Leukaemia of unspecified cell type
Leukaemia of unspecified cell type
Leukaemia of unspecified cell type
Leukaemia of unspecified cell type
Leukaemia of unspecified cell type
Leukaemia of unspecified cell type
Leukaemia of unspecified cell type
Leukaemia of unspecified cell type
Leukaemia of unspecified cell type
Leukaemia of unspecified cell type
Leukaemia of unspecified cell type
Leukaemia of unspecified cell type
Leukaemia of unspecified cell type
Leukaemia of unspecified cell type
Leukaemia of unspecified cell type
Other and unspecified malignant neoplasms of lymphoid, haematopoietic and related tissue
Other and unspecified malignant neoplasms of lymphoid, haematopoietic and related tissue
Other and unspecified malignant neoplasms of lymphoid, haematopoietic and related tissue
Other and unspecified malignant neoplasms of lymphoid, haematopoietic and related tissue
Other and unspecified malignant neoplasms of lymphoid, haematopoietic and related tissue
Other and unspecified malignant neoplasms of lymphoid, haematopoietic and related tissue
Other and unspecified malignant neoplasms of lymphoid, haematopoietic and related tissue
Other and unspecified malignant neoplasms of lymphoid, haematopoietic and related tissue
Other and unspecified malignant neoplasms of lymphoid, haematopoietic and related tissue
Other and unspecified malignant neoplasms of lymphoid, haematopoietic and related tissue
Other and unspecified malignant neoplasms of lymphoid, haematopoietic and related tissue
Other and unspecified malignant neoplasms of lymphoid, haematopoietic and related tissue
Other and unspecified malignant neoplasms of lymphoid, haematopoietic and related tissue
Other and unspecified malignant neoplasms of lymphoid, haematopoietic and related tissue
Other and unspecified malignant neoplasms of lymphoid, haematopoietic and related tissue
Malignant neoplasms of independent (primary) multiple sites
Malignant neoplasms of independent (primary) multiple sites
Malignant neoplasms of independent (primary) multiple sites
Carcinoma in situ of other and unspecified digestive organs
Carcinoma in situ of other and unspecified digestive organs
Carcinoma in situ of middle ear and respiratory system
Carcinoma in situ of skin
Carcinoma in situ of other and unspecified genital organs
Carcinoma in situ of other and unspecified genital organs
Carcinoma in situ of other and unspecified sites

For peer review only

Benign neoplasm of colon, rectum, anus and anal canal
Benign neoplasm of colon, rectum, anus and anal canal
Benign neoplasm of colon, rectum, anus and anal canal
Benign neoplasm of colon, rectum, anus and anal canal
Benign neoplasm of colon, rectum, anus and anal canal
Benign neoplasm of colon, rectum, anus and anal canal
Benign neoplasm of colon, rectum, anus and anal canal
Benign neoplasm of colon, rectum, anus and anal canal
Benign neoplasm of other and ill-defined parts of digestive system
Benign neoplasm of other and ill-defined parts of digestive system
Benign neoplasm of other and ill-defined parts of digestive system
Benign neoplasm of other and ill-defined parts of digestive system
Benign neoplasm of other and ill-defined parts of digestive system
Benign neoplasm of other and ill-defined parts of digestive system
Benign neoplasm of other and ill-defined parts of digestive system
Benign neoplasm of middle ear and respiratory system
Benign neoplasm of middle ear and respiratory system
Benign neoplasm of other and unspecified intrathoracic organs
Benign neoplasm of other and unspecified intrathoracic organs
Benign neoplasm of other and unspecified intrathoracic organs
Benign neoplasm of other and unspecified intrathoracic organs
Benign neoplasm of other and unspecified intrathoracic organs
Haemangioma and lymphangioma, any site
Haemangioma and lymphangioma, any site
Haemangioma and lymphangioma, any site
Haemangioma and lymphangioma, any site
Haemangioma and lymphangioma, any site
Haemangioma and lymphangioma, any site
Haemangioma and lymphangioma, any site
Haemangioma and lymphangioma, any site
Benign neoplasm of mesothelial tissue
Other benign neoplasms of connective and other soft tissue
Other benign neoplasms of connective and other soft tissue
Other benign neoplasms of connective and other soft tissue
Other benign neoplasms of skin
Leiomyoma of uterus
Leiomyoma of uterus
Benign neoplasm of ovary
Benign neoplasm of ovary
Benign neoplasm of male genital organs
Benign neoplasm of urinary organs
Benign neoplasm of urinary organs
Benign neoplasm of urinary organs
Benign neoplasm of meninges
Benign neoplasm of meninges
Benign neoplasm of meninges
Benign neoplasm of meninges
Benign neoplasm of meninges
Benign neoplasm of meninges
Benign neoplasm of meninges

For peer review only

Benign neoplasm of meninges
Benign neoplasm of meninges
Benign neoplasm of meninges
Benign neoplasm of meninges
Benign neoplasm of meninges
Benign neoplasm of meninges
Benign neoplasm of meninges
Benign neoplasm of meninges
Benign neoplasm of meninges
Benign neoplasm of meninges
Benign neoplasm of meninges
Benign neoplasm of meninges
Benign neoplasm of meninges
Benign neoplasm of meninges
Benign neoplasm of brain and other parts of central nervous system
Benign neoplasm of brain and other parts of central nervous system
Benign neoplasm of brain and other parts of central nervous system
Benign neoplasm of brain and other parts of central nervous system
Benign neoplasm of brain and other parts of central nervous system
Benign neoplasm of brain and other parts of central nervous system
Benign neoplasm of brain and other parts of central nervous system
Benign neoplasm of brain and other parts of central nervous system
Benign neoplasm of brain and other parts of central nervous system
Benign neoplasm of brain and other parts of central nervous system
Benign neoplasm of brain and other parts of central nervous system
Benign neoplasm of brain and other parts of central nervous system
Benign neoplasm of brain and other parts of central nervous system
Benign neoplasm of brain and other parts of central nervous system
Benign neoplasm of brain and other parts of central nervous system
Benign neoplasm of brain and other parts of central nervous system
Benign neoplasm of other and unspecified endocrine glands
Benign neoplasm of other and unspecified endocrine glands
Benign neoplasm of other and unspecified endocrine glands
Benign neoplasm of other and unspecified endocrine glands
Benign neoplasm of other and unspecified endocrine glands
Benign neoplasm of other and unspecified endocrine glands
Benign neoplasm of other and unspecified endocrine glands
Benign neoplasm of other and unspecified endocrine glands
Benign neoplasm of other and unspecified endocrine glands
Benign neoplasm of other and unspecified endocrine glands
Benign neoplasm of other and unspecified endocrine glands
Benign neoplasm of other and unspecified endocrine glands
Benign neoplasm of other and unspecified endocrine glands
Benign neoplasm of other and unspecified sites
Benign neoplasm of other and unspecified sites
Benign neoplasm of other and unspecified sites
Benign neoplasm of other and unspecified sites
Neoplasm of uncertain or unknown behaviour of oral cavity and digestive organs
Neoplasm of uncertain or unknown behaviour of oral cavity and digestive organs
Neoplasm of uncertain or unknown behaviour of oral cavity and digestive organs

For peer review only

Neoplasm of uncertain or unknown behaviour of oral cavity and digestive organs
Neoplasm of uncertain or unknown behaviour of oral cavity and digestive organs
Neoplasm of uncertain or unknown behaviour of oral cavity and digestive organs
Neoplasm of uncertain or unknown behaviour of oral cavity and digestive organs
Neoplasm of uncertain or unknown behaviour of oral cavity and digestive organs
Neoplasm of uncertain or unknown behaviour of oral cavity and digestive organs
Neoplasm of uncertain or unknown behaviour of oral cavity and digestive organs
Neoplasm of uncertain or unknown behaviour of oral cavity and digestive organs
Neoplasm of uncertain or unknown behaviour of oral cavity and digestive organs
Neoplasm of uncertain or unknown behaviour of oral cavity and digestive organs
Neoplasm of uncertain or unknown behaviour of oral cavity and digestive organs
Neoplasm of uncertain or unknown behaviour of oral cavity and digestive organs
Neoplasm of uncertain or unknown behaviour of oral cavity and digestive organs
Neoplasm of uncertain or unknown behaviour of oral cavity and digestive organs
Neoplasm of uncertain or unknown behaviour of middle ear and respiratory and intrathoracic organs
Neoplasm of uncertain or unknown behaviour of middle ear and respiratory and intrathoracic organs
Neoplasm of uncertain or unknown behaviour of middle ear and respiratory and intrathoracic organs
Neoplasm of uncertain or unknown behaviour of middle ear and respiratory and intrathoracic organs
Neoplasm of uncertain or unknown behaviour of middle ear and respiratory and intrathoracic organs
Neoplasm of uncertain or unknown behaviour of middle ear and respiratory and intrathoracic organs
Neoplasm of uncertain or unknown behaviour of middle ear and respiratory and intrathoracic organs
Neoplasm of uncertain or unknown behaviour of middle ear and respiratory and intrathoracic organs
Neoplasm of uncertain or unknown behaviour of middle ear and respiratory and intrathoracic organs
Neoplasm of uncertain or unknown behaviour of middle ear and respiratory and intrathoracic organs
Neoplasm of uncertain or unknown behaviour of middle ear and respiratory and intrathoracic organs
Neoplasm of uncertain or unknown behaviour of middle ear and respiratory and intrathoracic organs
Neoplasm of uncertain or unknown behaviour of middle ear and respiratory and intrathoracic organs
Neoplasm of uncertain or unknown behaviour of middle ear and respiratory and intrathoracic organs
Neoplasm of uncertain or unknown behaviour of middle ear and respiratory and intrathoracic organs
Neoplasm of uncertain or unknown behaviour of middle ear and respiratory and intrathoracic organs
Neoplasm of uncertain or unknown behaviour of female genital organs
Neoplasm of uncertain or unknown behaviour of female genital organs
Neoplasm of uncertain or unknown behaviour of female genital organs
Neoplasm of uncertain or unknown behaviour of female genital organs
Neoplasm of uncertain or unknown behaviour of female genital organs
Neoplasm of uncertain or unknown behaviour of female genital organs
Neoplasm of uncertain or unknown behaviour of female genital organs
Neoplasm of uncertain or unknown behaviour of female genital organs
Neoplasm of uncertain or unknown behaviour of female genital organs
Neoplasm of uncertain or unknown behaviour of female genital organs
Neoplasm of uncertain or unknown behaviour of female genital organs
Neoplasm of uncertain or unknown behaviour of female genital organs
Neoplasm of uncertain or unknown behaviour of female genital organs
Neoplasm of uncertain or unknown behaviour of male genital organs
Neoplasm of uncertain or unknown behaviour of male genital organs
Neoplasm of uncertain or unknown behaviour of male genital organs
Neoplasm of uncertain or unknown behaviour of male genital organs
Neoplasm of uncertain or unknown behaviour of male genital organs
Neoplasm of uncertain or unknown behaviour of male genital organs
Neoplasm of uncertain or unknown behaviour of urinary organs

For peer review only

Neoplasm of uncertain or unknown behaviour of urinary organs
Neoplasm of uncertain or unknown behaviour of urinary organs
Neoplasm of uncertain or unknown behaviour of urinary organs
Neoplasm of uncertain or unknown behaviour of urinary organs
Neoplasm of uncertain or unknown behaviour of urinary organs
Neoplasm of uncertain or unknown behaviour of urinary organs
Neoplasm of uncertain or unknown behaviour of urinary organs
Neoplasm of uncertain or unknown behaviour of urinary organs
Neoplasm of uncertain or unknown behaviour of urinary organs
Neoplasm of uncertain or unknown behaviour of urinary organs
Neoplasm of uncertain or unknown behaviour of urinary organs
Neoplasm of uncertain or unknown behaviour of urinary organs
Neoplasm of uncertain or unknown behaviour of urinary organs
Neoplasm of uncertain or unknown behaviour of urinary organs
Neoplasm of uncertain or unknown behaviour of urinary organs
Neoplasm of uncertain or unknown behaviour of meninges
Neoplasm of uncertain or unknown behaviour of meninges
Neoplasm of uncertain or unknown behaviour of meninges
Neoplasm of uncertain or unknown behaviour of meninges
Neoplasm of uncertain or unknown behaviour of brain and central nervous system
Neoplasm of uncertain or unknown behaviour of brain and central nervous system
Neoplasm of uncertain or unknown behaviour of brain and central nervous system
Neoplasm of uncertain or unknown behaviour of brain and central nervous system
Neoplasm of uncertain or unknown behaviour of brain and central nervous system
Neoplasm of uncertain or unknown behaviour of brain and central nervous system
Neoplasm of uncertain or unknown behaviour of brain and central nervous system
Neoplasm of uncertain or unknown behaviour of brain and central nervous system
Neoplasm of uncertain or unknown behaviour of brain and central nervous system
Neoplasm of uncertain or unknown behaviour of brain and central nervous system
Neoplasm of uncertain or unknown behaviour of brain and central nervous system
Neoplasm of uncertain or unknown behaviour of brain and central nervous system
Neoplasm of uncertain or unknown behaviour of brain and central nervous system
Neoplasm of uncertain or unknown behaviour of brain and central nervous system
Neoplasm of uncertain or unknown behaviour of brain and central nervous system
Neoplasm of uncertain or unknown behaviour of brain and central nervous system
Neoplasm of uncertain or unknown behaviour of brain and central nervous system
Neoplasm of uncertain or unknown behaviour of brain and central nervous system
Neoplasm of uncertain or unknown behaviour of brain and central nervous system
Neoplasm of uncertain or unknown behaviour of brain and central nervous system
Neoplasm of uncertain or unknown behaviour of endocrine glands
Neoplasm of uncertain or unknown behaviour of endocrine glands
Neoplasm of uncertain or unknown behaviour of endocrine glands
Neoplasm of uncertain or unknown behaviour of endocrine glands
Neoplasm of uncertain or unknown behaviour of endocrine glands
Neoplasm of uncertain or unknown behaviour of endocrine glands
Neoplasm of uncertain or unknown behaviour of endocrine glands
Neoplasm of uncertain or unknown behaviour of endocrine glands
Neoplasm of uncertain or unknown behaviour of endocrine glands
Neoplasm of uncertain or unknown behaviour of endocrine glands
Neoplasm of uncertain or unknown behaviour of endocrine glands

For peer review only

Neoplasm of uncertain or unknown behaviour of endocrine glands
Polycythaemia vera
Polycythaemia vera
Polycythaemia vera
Polycythaemia vera
Polycythaemia vera
Polycythaemia vera
Polycythaemia vera
Polycythaemia vera
Polycythaemia vera
Polycythaemia vera
Polycythaemia vera
Polycythaemia vera
Polycythaemia vera
Polycythaemia vera
Polycythaemia vera
Polycythaemia vera
Polycythaemia vera
Myelodysplastic syndromes
Myelodysplastic syndromes
Myelodysplastic syndromes
Myelodysplastic syndromes
Myelodysplastic syndromes
Myelodysplastic syndromes
Myelodysplastic syndromes
Myelodysplastic syndromes
Myelodysplastic syndromes
Myelodysplastic syndromes
Myelodysplastic syndromes
Myelodysplastic syndromes
Myelodysplastic syndromes
Myelodysplastic syndromes
Myelodysplastic syndromes
Myelodysplastic syndromes
Myelodysplastic syndromes
Myelodysplastic syndromes
Myelodysplastic syndromes
Myelodysplastic syndromes
Other neoplasms of uncertain or unknown behaviour of lymphoid, haematopoietic and related tissue
Other neoplasms of uncertain or unknown behaviour of lymphoid, haematopoietic and related tissue
Other neoplasms of uncertain or unknown behaviour of lymphoid, haematopoietic and related tissue
Other neoplasms of uncertain or unknown behaviour of lymphoid, haematopoietic and related tissue
Other neoplasms of uncertain or unknown behaviour of lymphoid, haematopoietic and related tissue
Other neoplasms of uncertain or unknown behaviour of lymphoid, haematopoietic and related tissue
Other neoplasms of uncertain or unknown behaviour of lymphoid, haematopoietic and related tissue
Other neoplasms of uncertain or unknown behaviour of lymphoid, haematopoietic and related tissue
Other neoplasms of uncertain or unknown behaviour of lymphoid, haematopoietic and related tissue
Other neoplasms of uncertain or unknown behaviour of lymphoid, haematopoietic and related tissue
Other neoplasms of uncertain or unknown behaviour of lymphoid, haematopoietic and related tissue

For peer review only

Other neoplasms of uncertain or unknown behaviour of lymphoid, haematopoietic and related tissue
Other neoplasms of uncertain or unknown behaviour of lymphoid, haematopoietic and related tissue
Other neoplasms of uncertain or unknown behaviour of lymphoid, haematopoietic and related tissue
Other neoplasms of uncertain or unknown behaviour of lymphoid, haematopoietic and related tissue
Other neoplasms of uncertain or unknown behaviour of lymphoid, haematopoietic and related tissue
Other neoplasms of uncertain or unknown behaviour of lymphoid, haematopoietic and related tissue
Other neoplasms of uncertain or unknown behaviour of lymphoid, haematopoietic and related tissue
Other neoplasms of uncertain or unknown behaviour of lymphoid, haematopoietic and related tissue
Other neoplasms of uncertain or unknown behaviour of lymphoid, haematopoietic and related tissue
Other neoplasms of uncertain or unknown behaviour of lymphoid, haematopoietic and related tissue
Neoplasm of uncertain or unknown behaviour of other and unspecified sites
Neoplasm of uncertain or unknown behaviour of other and unspecified sites
Neoplasm of uncertain or unknown behaviour of other and unspecified sites
Neoplasm of uncertain or unknown behaviour of other and unspecified sites
Neoplasm of uncertain or unknown behaviour of other and unspecified sites
Neoplasm of uncertain or unknown behaviour of other and unspecified sites
Neoplasm of uncertain or unknown behaviour of other and unspecified sites
Neoplasm of uncertain or unknown behaviour of other and unspecified sites
Neoplasm of uncertain or unknown behaviour of other and unspecified sites
Neoplasm of uncertain or unknown behaviour of other and unspecified sites
Neoplasm of uncertain or unknown behaviour of other and unspecified sites
Neoplasm of uncertain or unknown behaviour of other and unspecified sites
Neoplasm of uncertain or unknown behaviour of other and unspecified sites
Neoplasm of uncertain or unknown behaviour of other and unspecified sites
Neoplasm of uncertain or unknown behaviour of other and unspecified sites
Neoplasm of uncertain or unknown behaviour of other and unspecified sites
Iron deficiency anaemia
Iron deficiency anaemia
Iron deficiency anaemia
Iron deficiency anaemia
Iron deficiency anaemia
Iron deficiency anaemia
Iron deficiency anaemia
Iron deficiency anaemia
Iron deficiency anaemia
Iron deficiency anaemia
Iron deficiency anaemia
Iron deficiency anaemia
Iron deficiency anaemia
Iron deficiency anaemia
Iron deficiency anaemia
Iron deficiency anaemia
Vitamin B12 deficiency anaemia
Vitamin B12 deficiency anaemia
Vitamin B12 deficiency anaemia
Vitamin B12 deficiency anaemia
Vitamin B12 deficiency anaemia
Vitamin B12 deficiency anaemia
Vitamin B12 deficiency anaemia
Vitamin B12 deficiency anaemia

For peer review only

Folate deficiency anaemia
Folate deficiency anaemia
Folate deficiency anaemia
Other nutritional anaemias
Other nutritional anaemias
Other nutritional anaemias
Other nutritional anaemias
Other nutritional anaemias
Other nutritional anaemias
Other nutritional anaemias
Thalassaemia
Thalassaemia
Thalassaemia
Sickle-cell disorders
Sickle-cell disorders
Sickle-cell disorders
Other hereditary haemolytic anaemias
Other hereditary haemolytic anaemias
Other hereditary haemolytic anaemias
Other hereditary haemolytic anaemias
Other hereditary haemolytic anaemias
Acquired haemolytic anaemia
Acquired haemolytic anaemia
Acquired haemolytic anaemia
Acquired haemolytic anaemia
Acquired haemolytic anaemia
Acquired haemolytic anaemia
Acquired haemolytic anaemia
Acquired haemolytic anaemia
Acquired haemolytic anaemia
Acquired haemolytic anaemia
Acquired haemolytic anaemia
Acquired haemolytic anaemia
Acquired haemolytic anaemia
Acquired haemolytic anaemia
Acquired pure red cell aplasia [erythroblastopenia]
Acquired pure red cell aplasia [erythroblastopenia]
Acquired pure red cell aplasia [erythroblastopenia]
Other aplastic anaemias
Other aplastic anaemias
Other aplastic anaemias
Other aplastic anaemias
Other aplastic anaemias
Other aplastic anaemias
Other aplastic anaemias
Other aplastic anaemias
Other aplastic anaemias
Other aplastic anaemias
Other aplastic anaemias
Other aplastic anaemias

For peer review only

Other aplastic anaemias
Other aplastic anaemias
Other aplastic anaemias
Other aplastic anaemias
Other aplastic anaemias
Other aplastic anaemias
Other aplastic anaemias
Acute posthaemorrhagic anaemia
Other anaemias
Other anaemias
Other anaemias
Other anaemias
Other anaemias
Other anaemias
Other anaemias
Other anaemias
Other anaemias
Other anaemias
Other anaemias
Other anaemias
Other anaemias
Other anaemias
Other anaemias
Other anaemias
Disseminated intravascular coagulation [defibrination syndrome]
Disseminated intravascular coagulation [defibrination syndrome]
Disseminated intravascular coagulation [defibrination syndrome]
Disseminated intravascular coagulation [defibrination syndrome]
Disseminated intravascular coagulation [defibrination syndrome]
Disseminated intravascular coagulation [defibrination syndrome]
Disseminated intravascular coagulation [defibrination syndrome]
Disseminated intravascular coagulation [defibrination syndrome]
Disseminated intravascular coagulation [defibrination syndrome]
Hereditary factor VIII deficiency
Hereditary factor VIII deficiency
Hereditary factor VIII deficiency
Hereditary factor VIII deficiency
Hereditary factor VIII deficiency
Hereditary factor VIII deficiency
Other coagulation defects
Other coagulation defects
Other coagulation defects
Other coagulation defects
Other coagulation defects
Other coagulation defects
Other coagulation defects
Other coagulation defects
Other coagulation defects
Other coagulation defects
Other coagulation defects

For peer review only

Other coagulation defects
Other coagulation defects
Purpura and other haemorrhagic conditions
Purpura and other haemorrhagic conditions
Purpura and other haemorrhagic conditions
Purpura and other haemorrhagic conditions
Purpura and other haemorrhagic conditions
Purpura and other haemorrhagic conditions
Purpura and other haemorrhagic conditions
Purpura and other haemorrhagic conditions
Purpura and other haemorrhagic conditions
Purpura and other haemorrhagic conditions
Purpura and other haemorrhagic conditions
Purpura and other haemorrhagic conditions
Purpura and other haemorrhagic conditions
Purpura and other haemorrhagic conditions
Purpura and other haemorrhagic conditions
Purpura and other haemorrhagic conditions
Purpura and other haemorrhagic conditions
Purpura and other haemorrhagic conditions
Agranulocytosis
Agranulocytosis
Agranulocytosis
Agranulocytosis
Agranulocytosis
Agranulocytosis
Agranulocytosis
Agranulocytosis
Agranulocytosis
Agranulocytosis
Agranulocytosis
Other disorders of white blood cells
Other disorders of white blood cells
Other disorders of white blood cells
Diseases of spleen
Diseases of spleen
Diseases of spleen
Diseases of spleen
Diseases of spleen
Diseases of spleen
Diseases of spleen
Diseases of spleen
Other diseases of blood and blood-forming organs
Other diseases of blood and blood-forming organs
Other diseases of blood and blood-forming organs
Other diseases of blood and blood-forming organs
Other diseases of blood and blood-forming organs
Other diseases of blood and blood-forming organs
Other diseases of blood and blood-forming organs
Other diseases of blood and blood-forming organs

For peer review only

Other diseases of blood and blood-forming organs
Other diseases of blood and blood-forming organs
Other diseases of blood and blood-forming organs
Other diseases of blood and blood-forming organs
Certain diseases involving lymphoreticular tissue and reticulohistiocytic system
Certain diseases involving lymphoreticular tissue and reticulohistiocytic system
Certain diseases involving lymphoreticular tissue and reticulohistiocytic system
Certain diseases involving lymphoreticular tissue and reticulohistiocytic system
Certain diseases involving lymphoreticular tissue and reticulohistiocytic system
Certain diseases involving lymphoreticular tissue and reticulohistiocytic system
Immunodeficiency with predominantly antibody defects
Immunodeficiency with predominantly antibody defects
Combined immunodeficiencies
Other immunodeficiencies
Other immunodeficiencies
Other immunodeficiencies
Other immunodeficiencies
Other immunodeficiencies
Other immunodeficiencies
Other immunodeficiencies
Other immunodeficiencies
Sarcoidosis
Sarcoidosis
Sarcoidosis
Sarcoidosis
Sarcoidosis
Sarcoidosis
Sarcoidosis
Sarcoidosis
Sarcoidosis
Sarcoidosis
Sarcoidosis
Sarcoidosis
Sarcoidosis
Sarcoidosis
Sarcoidosis
Other disorders involving the immune mechanism, not elsewhere classified
Other disorders involving the immune mechanism, not elsewhere classified
Other disorders involving the immune mechanism, not elsewhere classified
Other disorders involving the immune mechanism, not elsewhere classified
Other disorders involving the immune mechanism, not elsewhere classified
Other disorders involving the immune mechanism, not elsewhere classified
Other disorders involving the immune mechanism, not elsewhere classified
Other hypothyroidism
Other hypothyroidism
Other hypothyroidism
Other hypothyroidism
Other hypothyroidism
Other hypothyroidism
Other hypothyroidism

For peer review only

Other hypothyroidism
Other hypothyroidism
Other hypothyroidism
Other hypothyroidism
Other hypothyroidism
Other hypothyroidism
Other nontoxic goitre
Other nontoxic goitre
Other nontoxic goitre
Other nontoxic goitre
Other nontoxic goitre
Other nontoxic goitre
Other nontoxic goitre
Other nontoxic goitre
Other nontoxic goitre
Thyrotoxicosis [hyperthyroidism]
Thyrotoxicosis [hyperthyroidism]
Thyrotoxicosis [hyperthyroidism]
Thyrotoxicosis [hyperthyroidism]
Thyrotoxicosis [hyperthyroidism]
Thyrotoxicosis [hyperthyroidism]
Thyrotoxicosis [hyperthyroidism]
Thyrotoxicosis [hyperthyroidism]
Thyrotoxicosis [hyperthyroidism]
Thyrotoxicosis [hyperthyroidism]
Thyrotoxicosis [hyperthyroidism]
Thyrotoxicosis [hyperthyroidism]
Thyrotoxicosis [hyperthyroidism]
Thyrotoxicosis [hyperthyroidism]
Thyrotoxicosis [hyperthyroidism]
Thyroiditis
Thyroiditis
Other disorders of thyroid
Other disorders of thyroid
Other disorders of thyroid
Other disorders of thyroid
Other disorders of thyroid
Other disorders of thyroid
Insulin-dependent diabetes mellitus
Insulin-dependent diabetes mellitus
Insulin-dependent diabetes mellitus
Insulin-dependent diabetes mellitus
Insulin-dependent diabetes mellitus
Insulin-dependent diabetes mellitus
Insulin-dependent diabetes mellitus
Insulin-dependent diabetes mellitus
Insulin-dependent diabetes mellitus
Insulin-dependent diabetes mellitus
Insulin-dependent diabetes mellitus
Insulin-dependent diabetes mellitus

For peer review only

Insulin-dependent diabetes mellitus
Insulin-dependent diabetes mellitus
Insulin-dependent diabetes mellitus
Insulin-dependent diabetes mellitus
Insulin-dependent diabetes mellitus
Insulin-dependent diabetes mellitus
Insulin-dependent diabetes mellitus
Insulin-dependent diabetes mellitus
Insulin-dependent diabetes mellitus
Non-insulin-dependent diabetes mellitus
Non-insulin-dependent diabetes mellitus
Non-insulin-dependent diabetes mellitus
Non-insulin-dependent diabetes mellitus
Non-insulin-dependent diabetes mellitus
Non-insulin-dependent diabetes mellitus
Non-insulin-dependent diabetes mellitus
Non-insulin-dependent diabetes mellitus
Non-insulin-dependent diabetes mellitus
Non-insulin-dependent diabetes mellitus
Non-insulin-dependent diabetes mellitus
Non-insulin-dependent diabetes mellitus
Non-insulin-dependent diabetes mellitus
Non-insulin-dependent diabetes mellitus
Non-insulin-dependent diabetes mellitus
Non-insulin-dependent diabetes mellitus
Non-insulin-dependent diabetes mellitus
Non-insulin-dependent diabetes mellitus
Non-insulin-dependent diabetes mellitus
Non-insulin-dependent diabetes mellitus
Non-insulin-dependent diabetes mellitus
Malnutrition-related diabetes mellitus
Malnutrition-related diabetes mellitus
Malnutrition-related diabetes mellitus
Malnutrition-related diabetes mellitus
Other specified diabetes mellitus
Other specified diabetes mellitus
Other specified diabetes mellitus
Other specified diabetes mellitus
Unspecified diabetes mellitus
Unspecified diabetes mellitus
Unspecified diabetes mellitus
Unspecified diabetes mellitus
Unspecified diabetes mellitus
Unspecified diabetes mellitus
Unspecified diabetes mellitus
Unspecified diabetes mellitus
Unspecified diabetes mellitus
Unspecified diabetes mellitus
Unspecified diabetes mellitus
Unspecified diabetes mellitus

For peer review only

Unspecified diabetes mellitus
Unspecified diabetes mellitus
Unspecified diabetes mellitus
Unspecified diabetes mellitus
Unspecified diabetes mellitus
Unspecified diabetes mellitus
Unspecified diabetes mellitus
Unspecified diabetes mellitus
Unspecified diabetes mellitus
Nondiabetic hypoglycaemic coma
Nondiabetic hypoglycaemic coma
Nondiabetic hypoglycaemic coma
Nondiabetic hypoglycaemic coma
Nondiabetic hypoglycaemic coma
Nondiabetic hypoglycaemic coma
Other disorders of pancreatic internal secretion
Other disorders of pancreatic internal secretion
Other disorders of pancreatic internal secretion
Other disorders of pancreatic internal secretion
Other disorders of pancreatic internal secretion
Other disorders of pancreatic internal secretion
Other disorders of pancreatic internal secretion
Hyperparathyroidism and other disorders of parathyroid gland
Hyperparathyroidism and other disorders of parathyroid gland
Hyperparathyroidism and other disorders of parathyroid gland
Hyperparathyroidism and other disorders of parathyroid gland
Hyperparathyroidism and other disorders of parathyroid gland
Hyperparathyroidism and other disorders of parathyroid gland
Hyperparathyroidism and other disorders of parathyroid gland
Hyperparathyroidism and other disorders of parathyroid gland
Hyperfunction of pituitary gland
Hyperfunction of pituitary gland
Hyperfunction of pituitary gland
Hyperfunction of pituitary gland
Hyperfunction of pituitary gland
Hypofunction and other disorders of pituitary gland
Hypofunction and other disorders of pituitary gland
Hypofunction and other disorders of pituitary gland
Hypofunction and other disorders of pituitary gland
Hypofunction and other disorders of pituitary gland
Hypofunction and other disorders of pituitary gland
Hypofunction and other disorders of pituitary gland
Cushing's syndrome
Cushing's syndrome
Cushing's syndrome
Cushing's syndrome
Other disorders of adrenal gland
Other disorders of adrenal gland
Other disorders of adrenal gland
Other disorders of adrenal gland

For peer review only

Other disorders of adrenal gland
Other disorders of adrenal gland
Other disorders of adrenal gland
Other disorders of adrenal gland
Other disorders of adrenal gland
Other disorders of adrenal gland
Other disorders of adrenal gland
Other disorders of adrenal gland
Other disorders of adrenal gland
Other endocrine disorders
Other endocrine disorders
Other endocrine disorders
Other endocrine disorders
Other endocrine disorders
Nutritional marasmus
Nutritional marasmus
Nutritional marasmus
Nutritional marasmus
Nutritional marasmus
Nutritional marasmus
Nutritional marasmus
Nutritional marasmus
Nutritional marasmus
Nutritional marasmus
Nutritional marasmus
Unspecified severe protein-energy malnutrition
Unspecified severe protein-energy malnutrition
Unspecified severe protein-energy malnutrition
Unspecified severe protein-energy malnutrition
Unspecified severe protein-energy malnutrition
Unspecified severe protein-energy malnutrition
Unspecified severe protein-energy malnutrition
Unspecified severe protein-energy malnutrition
Unspecified severe protein-energy malnutrition
Unspecified protein-energy malnutrition
Unspecified protein-energy malnutrition
Unspecified protein-energy malnutrition
Unspecified protein-energy malnutrition
Unspecified protein-energy malnutrition
Unspecified protein-energy malnutrition
Unspecified protein-energy malnutrition
Unspecified protein-energy malnutrition
Unspecified protein-energy malnutrition
Unspecified protein-energy malnutrition
Unspecified protein-energy malnutrition
Thiamine deficiency
Thiamine deficiency
Thiamine deficiency
Thiamine deficiency
Other nutritional deficiencies

For peer review only

Other nutritional deficiencies
Other nutritional deficiencies
Sequelae of malnutrition and other nutritional deficiencies
Obesity
Obesity
Obesity
Obesity
Obesity
Obesity
Obesity
Obesity
Obesity
Obesity
Obesity
Obesity
Obesity
Obesity
Obesity
Obesity
Obesity
Obesity
Obesity
Obesity
Disorders of branched-chain amino-acid metabolism and fatty-acid metabolism
Disorders of branched-chain amino-acid metabolism and fatty-acid metabolism
Disorders of branched-chain amino-acid metabolism and fatty-acid metabolism
Disorders of branched-chain amino-acid metabolism and fatty-acid metabolism
Other disorders of amino-acid metabolism
Other disorders of amino-acid metabolism
Other disorders of amino-acid metabolism
Other disorders of amino-acid metabolism
Other disorders of amino-acid metabolism
Other disorders of carbohydrate metabolism
Other disorders of carbohydrate metabolism
Other disorders of carbohydrate metabolism
Other disorders of carbohydrate metabolism
Disorders of sphingolipid metabolism and other lipid storage disorders
Disorders of sphingolipid metabolism and other lipid storage disorders
Disorders of sphingolipid metabolism and other lipid storage disorders
Disorders of sphingolipid metabolism and other lipid storage disorders
Disorders of sphingolipid metabolism and other lipid storage disorders
Disorders of sphingolipid metabolism and other lipid storage disorders
Disorders of sphingolipid metabolism and other lipid storage disorders
Disorders of sphingolipid metabolism and other lipid storage disorders
Disorders of sphingolipid metabolism and other lipid storage disorders
Disorders of sphingolipid metabolism and other lipid storage disorders
Disorders of sphingolipid metabolism and other lipid storage disorders
Disorders of glycosaminoglycan metabolism
Disorders of glycosaminoglycan metabolism
Disorders of glycosaminoglycan metabolism

For peer review only

Disorders of glycosaminoglycan metabolism
Disorders of glycosaminoglycan metabolism
Disorders of glycoprotein metabolism
Disorders of glycoprotein metabolism
Disorders of glycoprotein metabolism
Disorders of glycoprotein metabolism
Disorders of lipoprotein metabolism and other lipidaemias
Disorders of lipoprotein metabolism and other lipidaemias
Disorders of lipoprotein metabolism and other lipidaemias
Disorders of lipoprotein metabolism and other lipidaemias
Disorders of lipoprotein metabolism and other lipidaemias
Disorders of lipoprotein metabolism and other lipidaemias
Disorders of lipoprotein metabolism and other lipidaemias
Disorders of lipoprotein metabolism and other lipidaemias
Disorders of lipoprotein metabolism and other lipidaemias
Disorders of lipoprotein metabolism and other lipidaemias
Disorders of lipoprotein metabolism and other lipidaemias
Disorders of lipoprotein metabolism and other lipidaemias
Disorders of lipoprotein metabolism and other lipidaemias
Disorders of purine and pyrimidine metabolism
Disorders of porphyrin and bilirubin metabolism
Disorders of mineral metabolism
Disorders of mineral metabolism
Disorders of mineral metabolism
Disorders of mineral metabolism
Disorders of mineral metabolism
Disorders of mineral metabolism
Disorders of mineral metabolism
Disorders of mineral metabolism
Disorders of mineral metabolism
Disorders of mineral metabolism
Disorders of mineral metabolism
Disorders of mineral metabolism
Disorders of mineral metabolism
Disorders of mineral metabolism
Cystic fibrosis
Cystic fibrosis
Cystic fibrosis
Cystic fibrosis
Cystic fibrosis
Cystic fibrosis
Cystic fibrosis
Cystic fibrosis
Cystic fibrosis
Cystic fibrosis
Cystic fibrosis
Cystic fibrosis
Cystic fibrosis
Amyloidosis
Amyloidosis

For peer review only

Amyloidosis
Amyloidosis
Amyloidosis
Amyloidosis
Amyloidosis
Amyloidosis
Amyloidosis
Amyloidosis
Amyloidosis
Amyloidosis
Amyloidosis
Amyloidosis
Amyloidosis
Amyloidosis
Amyloidosis
Amyloidosis
Volume depletion
Volume depletion
Volume depletion
Volume depletion
Volume depletion
Volume depletion
Volume depletion
Volume depletion
Volume depletion
Volume depletion
Volume depletion
Volume depletion
Volume depletion
Other disorders of fluid, electrolyte and acid-base balance
Other disorders of fluid, electrolyte and acid-base balance
Other disorders of fluid, electrolyte and acid-base balance
Other disorders of fluid, electrolyte and acid-base balance
Other disorders of fluid, electrolyte and acid-base balance
Other disorders of fluid, electrolyte and acid-base balance
Other disorders of fluid, electrolyte and acid-base balance
Other disorders of fluid, electrolyte and acid-base balance
Other disorders of fluid, electrolyte and acid-base balance
Other disorders of fluid, electrolyte and acid-base balance
Other disorders of fluid, electrolyte and acid-base balance
Other disorders of fluid, electrolyte and acid-base balance
Other disorders of fluid, electrolyte and acid-base balance
Other disorders of fluid, electrolyte and acid-base balance
Other metabolic disorders
Other metabolic disorders
Other metabolic disorders
Other metabolic disorders
Other metabolic disorders
Other metabolic disorders
Other metabolic disorders

For peer review only

Other metabolic disorders
Other metabolic disorders
Other metabolic disorders
Other metabolic disorders
Other metabolic disorders
Other metabolic disorders
Other metabolic disorders
Other metabolic disorders
Vascular dementia
Vascular dementia
Vascular dementia
Vascular dementia
Vascular dementia
Vascular dementia
Vascular dementia
Vascular dementia
Vascular dementia
Vascular dementia
Vascular dementia
Vascular dementia
Vascular dementia
Vascular dementia
Vascular dementia
Vascular dementia
Vascular dementia
Unspecified dementia
Unspecified dementia
Unspecified dementia
Unspecified dementia
Unspecified dementia
Unspecified dementia
Unspecified dementia
Unspecified dementia
Unspecified dementia
Unspecified dementia
Unspecified dementia
Unspecified dementia
Unspecified dementia
Unspecified dementia
Unspecified dementia
Unspecified dementia
Unspecified dementia
Organic amnesic syndrome, not induced by alcohol and other psychoactive substances
Organic amnesic syndrome, not induced by alcohol and other psychoactive substances
Delirium, not induced by alcohol and other psychoactive substances
Delirium, not induced by alcohol and other psychoactive substances
Delirium, not induced by alcohol and other psychoactive substances
Delirium, not induced by alcohol and other psychoactive substances
Delirium, not induced by alcohol and other psychoactive substances
Delirium, not induced by alcohol and other psychoactive substances

For peer review only

Delirium, not induced by alcohol and other psychoactive substances
Delirium, not induced by alcohol and other psychoactive substances
Delirium, not induced by alcohol and other psychoactive substances
Other mental disorders due to brain damage and dysfunction and to physical disease
Other mental disorders due to brain damage and dysfunction and to physical disease
Other mental disorders due to brain damage and dysfunction and to physical disease
Other mental disorders due to brain damage and dysfunction and to physical disease
Other mental disorders due to brain damage and dysfunction and to physical disease
Personality and behavioural disorders due to brain disease, damage and dysfunction
Personality and behavioural disorders due to brain disease, damage and dysfunction
Personality and behavioural disorders due to brain disease, damage and dysfunction
Personality and behavioural disorders due to brain disease, damage and dysfunction
Personality and behavioural disorders due to brain disease, damage and dysfunction
Personality and behavioural disorders due to brain disease, damage and dysfunction
Personality and behavioural disorders due to brain disease, damage and dysfunction
Personality and behavioural disorders due to brain disease, damage and dysfunction
Mental and behavioural disorders due to use of alcohol
Mental and behavioural disorders due to use of alcohol
Mental and behavioural disorders due to use of alcohol
Mental and behavioural disorders due to use of alcohol
Mental and behavioural disorders due to use of alcohol
Mental and behavioural disorders due to use of alcohol
Mental and behavioural disorders due to use of alcohol
Mental and behavioural disorders due to use of alcohol
Mental and behavioural disorders due to use of alcohol
Mental and behavioural disorders due to use of alcohol
Mental and behavioural disorders due to use of alcohol
Mental and behavioural disorders due to use of alcohol
Mental and behavioural disorders due to use of alcohol
Mental and behavioural disorders due to use of alcohol
Mental and behavioural disorders due to use of alcohol
Mental and behavioural disorders due to use of alcohol
Mental and behavioural disorders due to use of alcohol
Mental and behavioural disorders due to use of alcohol
Mental and behavioural disorders due to use of alcohol
Mental and behavioural disorders due to use of alcohol
Mental and behavioural disorders due to use of alcohol
Mental and behavioural disorders due to use of opioids
Mental and behavioural disorders due to use of opioids
Mental and behavioural disorders due to use of opioids
Mental and behavioural disorders due to use of opioids
Mental and behavioural disorders due to use of opioids
Mental and behavioural disorders due to use of opioids
Mental and behavioural disorders due to use of opioids
Mental and behavioural disorders due to use of opioids
Mental and behavioural disorders due to use of opioids
Mental and behavioural disorders due to use of opioids
Mental and behavioural disorders due to use of opioids
Mental and behavioural disorders due to use of sedatives or hypnotics
Mental and behavioural disorders due to use of cocaine

For peer review only

Mental and behavioural disorders due to use of cocaine
Mental and behavioural disorders due to use of other stimulants, including caffeine
Mental and behavioural disorders due to use of tobacco
Mental and behavioural disorders due to use of tobacco
Mental and behavioural disorders due to use of tobacco
Mental and behavioural disorders due to use of tobacco
Mental and behavioural disorders due to use of tobacco
Mental and behavioural disorders due to use of tobacco
Mental and behavioural disorders due to use of volatile solvents
Mental and behavioural disorders due to multiple drug use and use of other psychoactive substances
Mental and behavioural disorders due to multiple drug use and use of other psychoactive substances
Mental and behavioural disorders due to multiple drug use and use of other psychoactive substances
Mental and behavioural disorders due to multiple drug use and use of other psychoactive substances
Mental and behavioural disorders due to multiple drug use and use of other psychoactive substances
Mental and behavioural disorders due to multiple drug use and use of other psychoactive substances
Mental and behavioural disorders due to multiple drug use and use of other psychoactive substances
Mental and behavioural disorders due to multiple drug use and use of other psychoactive substances
Mental and behavioural disorders due to multiple drug use and use of other psychoactive substances
Mental and behavioural disorders due to multiple drug use and use of other psychoactive substances
Mental and behavioural disorders due to multiple drug use and use of other psychoactive substances
Mental and behavioural disorders due to multiple drug use and use of other psychoactive substances
Schizophrenia
Schizophrenia
Schizophrenia
Schizophrenia
Schizophrenia
Schizophrenia
Schizophrenia
Schizophrenia
Schizophrenia
Schizophrenia
Schizophrenia
Schizophrenia
Schizophrenia
Schizophrenia
Schizophrenia
Schizophrenia
Schizophrenia
Persistent delusional disorders
Persistent delusional disorders
Persistent delusional disorders
Persistent delusional disorders
Persistent delusional disorders
Persistent delusional disorders
Persistent delusional disorders
Persistent delusional disorders
Acute and transient psychotic disorders
Schizoaffective disorders
Schizoaffective disorders
Other nonorganic psychotic disorders

For peer review only

Unspecified nonorganic psychosis
Unspecified nonorganic psychosis
Unspecified nonorganic psychosis
Unspecified nonorganic psychosis
Unspecified nonorganic psychosis
Unspecified nonorganic psychosis
Unspecified nonorganic psychosis
Unspecified nonorganic psychosis
Bipolar affective disorder
Bipolar affective disorder
Bipolar affective disorder
Bipolar affective disorder
Bipolar affective disorder
Bipolar affective disorder
Bipolar affective disorder
Bipolar affective disorder
Bipolar affective disorder
Bipolar affective disorder
Depressive episodes
Depressive episodes
Depressive episodes
Depressive episodes
Depressive episodes
Depressive episodes
Depressive episodes
Depressive episodes
Depressive episodes
Depressive episodes
Depressive episodes
Depressive episodes
Recurrent depressive disorder
Recurrent depressive disorder
Recurrent depressive disorder
Recurrent depressive disorder
Recurrent depressive disorder
Recurrent depressive disorder
Recurrent depressive disorder
Persistent mood [affective] disorders
Other anxiety disorders
Other anxiety disorders
Other anxiety disorders
Other anxiety disorders
Obsessive-compulsive disorder
Reaction to severe stress, and adjustment disorders
Dissociative [conversion] disorders
Dissociative [conversion] disorders
Dissociative [conversion] disorders
Other neurotic disorders
Eating disorders
Eating disorders

For peer review only

Eating disorders
Eating disorders
Eating disorders
Eating disorders
Eating disorders
Eating disorders
Eating disorders
Eating disorders
Psychological and behavioural factors associated with disorders or diseases classified elsewhere
Psychological and behavioural factors associated with disorders or diseases classified elsewhere
Abuse of non-dependence-producing substances
Abuse of non-dependence-producing substances
Specific personality disorders
MModerate mental retardation
MSevere mental retardation
MSevere mental retardation
MProfound mental retardation
MProfound mental retardation
MProfound mental retardation
MProfound mental retardation
MUnspecified mental retardation
MUnspecified mental retardation
MUnspecified mental retardation
MUnspecified mental retardation
MUnspecified mental retardation
MUnspecified mental retardation
MUnspecified mental retardation
MUnspecified mental retardation
MUnspecified mental retardation
Specific developmental disorders of scholastic skills
Pervasive developmental disorders
Pervasive developmental disorders
Pervasive developmental disorders
Mental disorder, not otherwise specified
Mental disorder, not otherwise specified
Mental disorder, not otherwise specified
Mental disorder, not otherwise specified
Mental disorder, not otherwise specified
Bacterial meningitis, not elsewhere classified
Bacterial meningitis, not elsewhere classified
Bacterial meningitis, not elsewhere classified
Bacterial meningitis, not elsewhere classified
Bacterial meningitis, not elsewhere classified
Bacterial meningitis, not elsewhere classified
Bacterial meningitis, not elsewhere classified
Bacterial meningitis, not elsewhere classified
Bacterial meningitis, not elsewhere classified
Bacterial meningitis, not elsewhere classified
Bacterial meningitis, not elsewhere classified
Bacterial meningitis, not elsewhere classified

For peer review only

Bacterial meningitis, not elsewhere classified
Bacterial meningitis, not elsewhere classified
Bacterial meningitis, not elsewhere classified
Bacterial meningitis, not elsewhere classified
Bacterial meningitis, not elsewhere classified
Bacterial meningitis, not elsewhere classified
Bacterial meningitis, not elsewhere classified
Bacterial meningitis, not elsewhere classified
Bacterial meningitis, not elsewhere classified
Meningitis due to other and unspecified causes
Meningitis due to other and unspecified causes
Meningitis due to other and unspecified causes
Meningitis due to other and unspecified causes
Meningitis due to other and unspecified causes
Meningitis due to other and unspecified causes
Meningitis due to other and unspecified causes
Meningitis due to other and unspecified causes
Meningitis due to other and unspecified causes
Meningitis due to other and unspecified causes
Meningitis due to other and unspecified causes
Meningitis due to other and unspecified causes
Meningitis due to other and unspecified causes
Meningitis due to other and unspecified causes
Meningitis due to other and unspecified causes
Encephalitis, myelitis and encephalomyelitis
Encephalitis, myelitis and encephalomyelitis
Encephalitis, myelitis and encephalomyelitis
Encephalitis, myelitis and encephalomyelitis
Encephalitis, myelitis and encephalomyelitis
Encephalitis, myelitis and encephalomyelitis
Encephalitis, myelitis and encephalomyelitis
Encephalitis, myelitis and encephalomyelitis
Encephalitis, myelitis and encephalomyelitis
Encephalitis, myelitis and encephalomyelitis
Encephalitis, myelitis and encephalomyelitis
Encephalitis, myelitis and encephalomyelitis
Encephalitis, myelitis and encephalomyelitis
Encephalitis, myelitis and encephalomyelitis
Encephalitis, myelitis and encephalomyelitis
Encephalitis, myelitis and encephalomyelitis
Encephalitis, myelitis and encephalomyelitis
Encephalitis, myelitis and encephalomyelitis
Encephalitis, myelitis and encephalomyelitis
Intracranial and intraspinal abscess and granuloma
Intracranial and intraspinal abscess and granuloma
Intracranial and intraspinal abscess and granuloma
Intracranial and intraspinal abscess and granuloma
Intracranial and intraspinal abscess and granuloma
Intracranial and intraspinal abscess and granuloma
Intracranial and intraspinal abscess and granuloma

For peer review only

Intracranial and intraspinal abscess and granuloma
Intracranial and intraspinal abscess and granuloma
Intracranial and intraspinal abscess and granuloma
Intracranial and intraspinal abscess and granuloma
Intracranial and intraspinal abscess and granuloma
Intracranial and intraspinal abscess and granuloma
Intracranial and intraspinal phlebitis and thrombophlebitis
Intracranial and intraspinal phlebitis and thrombophlebitis
Intracranial and intraspinal phlebitis and thrombophlebitis
Sequelae of inflammatory diseases of central nervous system
Sequelae of inflammatory diseases of central nervous system
Sequelae of inflammatory diseases of central nervous system
Sequelae of inflammatory diseases of central nervous system
Sequelae of inflammatory diseases of central nervous system
Sequelae of inflammatory diseases of central nervous system
Sequelae of inflammatory diseases of central nervous system
Sequelae of inflammatory diseases of central nervous system
Huntington's disease
Huntington's disease
Huntington's disease
Huntington's disease
Huntington's disease
Huntington's disease
Huntington's disease
Huntington's disease
Huntington's disease
Huntington's disease
Huntington's disease
Huntington's disease
Huntington's disease
Huntington's disease
Huntington's disease
Huntington's disease
Huntington's disease
Huntington's disease
Hereditary ataxia
Hereditary ataxia
Hereditary ataxia
Hereditary ataxia
Hereditary ataxia
Hereditary ataxia
Hereditary ataxia
Hereditary ataxia
Hereditary ataxia
Hereditary ataxia
Hereditary ataxia
Hereditary ataxia
Hereditary ataxia
Spinal muscular atrophy and related syndromes
Spinal muscular atrophy and related syndromes

For peer review only

Spinal muscular atrophy and related syndromes
Spinal muscular atrophy and related syndromes
Spinal muscular atrophy and related syndromes
Spinal muscular atrophy and related syndromes
Spinal muscular atrophy and related syndromes
Spinal muscular atrophy and related syndromes
Spinal muscular atrophy and related syndromes
Spinal muscular atrophy and related syndromes
Spinal muscular atrophy and related syndromes
Spinal muscular atrophy and related syndromes
Spinal muscular atrophy and related syndromes
Spinal muscular atrophy and related syndromes
Spinal muscular atrophy and related syndromes
Spinal muscular atrophy and related syndromes
Spinal muscular atrophy and related syndromes
Spinal muscular atrophy and related syndromes
Spinal muscular atrophy and related syndromes
Spinal muscular atrophy and related syndromes
Spinal muscular atrophy and related syndromes
Parkinson's disease
Parkinson's disease
Parkinson's disease
Parkinson's disease
Parkinson's disease
Parkinson's disease
Parkinson's disease
Parkinson's disease
Parkinson's disease
Parkinson's disease
Parkinson's disease
Parkinson's disease
Parkinson's disease
Parkinson's disease
Parkinson's disease
Parkinson's disease
Parkinson's disease
Parkinson's disease
Parkinson's disease
Parkinson's disease
Parkinson's disease
Secondary parkinsonism
Secondary parkinsonism
Secondary parkinsonism
Secondary parkinsonism
Secondary parkinsonism
Secondary parkinsonism
Secondary parkinsonism
Secondary parkinsonism
Secondary parkinsonism
Secondary parkinsonism

For peer review only

Secondary parkinsonism
Other degenerative diseases of the basal ganglia
Other degenerative diseases of the basal ganglia
Other degenerative diseases of the basal ganglia
Other degenerative diseases of the basal ganglia
Other degenerative diseases of the basal ganglia
Other degenerative diseases of the basal ganglia
Other degenerative diseases of the basal ganglia
Other degenerative diseases of the basal ganglia
Other degenerative diseases of the basal ganglia
Other degenerative diseases of the basal ganglia
Other degenerative diseases of the basal ganglia
Other degenerative diseases of the basal ganglia
Other degenerative diseases of the basal ganglia
Other degenerative diseases of the basal ganglia
Dystonia
Dystonia
Dystonia
Other extrapyramidal and movement disorders
Other extrapyramidal and movement disorders
Other extrapyramidal and movement disorders
Other extrapyramidal and movement disorders
Other extrapyramidal and movement disorders
Other extrapyramidal and movement disorders
Other extrapyramidal and movement disorders
Other extrapyramidal and movement disorders
Alzheimer's disease
Alzheimer's disease
Alzheimer's disease
Alzheimer's disease
Alzheimer's disease
Alzheimer's disease
Alzheimer's disease
Alzheimer's disease
Alzheimer's disease
Alzheimer's disease
Alzheimer's disease
Alzheimer's disease
Alzheimer's disease
Alzheimer's disease
Alzheimer's disease
Alzheimer's disease
Alzheimer's disease
Alzheimer's disease
Alzheimer's disease
Alzheimer's disease
Alzheimer's disease
Other degenerative diseases of nervous system, not elsewhere classified
Other degenerative diseases of nervous system, not elsewhere classified
Other degenerative diseases of nervous system, not elsewhere classified

For peer review only

Other degenerative diseases of nervous system, not elsewhere classified
Other degenerative diseases of nervous system, not elsewhere classified
Other degenerative diseases of nervous system, not elsewhere classified
Other degenerative diseases of nervous system, not elsewhere classified
Other degenerative diseases of nervous system, not elsewhere classified
Other degenerative diseases of nervous system, not elsewhere classified
Other degenerative diseases of nervous system, not elsewhere classified
Other degenerative diseases of nervous system, not elsewhere classified
Other degenerative diseases of nervous system, not elsewhere classified
Other degenerative diseases of nervous system, not elsewhere classified
Other degenerative diseases of nervous system, not elsewhere classified
Other degenerative diseases of nervous system, not elsewhere classified
Other degenerative diseases of nervous system, not elsewhere classified
Other degenerative diseases of nervous system, not elsewhere classified
Other degenerative diseases of nervous system, not elsewhere classified
Other degenerative diseases of nervous system, not elsewhere classified
Other degenerative diseases of nervous system, not elsewhere classified
Other degenerative diseases of nervous system, not elsewhere classified
Multiple sclerosis
Multiple sclerosis
Multiple sclerosis
Multiple sclerosis
Multiple sclerosis
Multiple sclerosis
Multiple sclerosis
Multiple sclerosis
Multiple sclerosis
Multiple sclerosis
Multiple sclerosis
Multiple sclerosis
Multiple sclerosis
Multiple sclerosis
Multiple sclerosis
Multiple sclerosis
Multiple sclerosis
Multiple sclerosis
Multiple sclerosis
Multiple sclerosis
Multiple sclerosis
Other acute disseminated demyelination
Other demyelinating diseases of central nervous system
Other demyelinating diseases of central nervous system
Other demyelinating diseases of central nervous system
Other demyelinating diseases of central nervous system
Other demyelinating diseases of central nervous system
Other demyelinating diseases of central nervous system
Other demyelinating diseases of central nervous system
Other demyelinating diseases of central nervous system
Other demyelinating diseases of central nervous system
Other demyelinating diseases of central nervous system

For peer review only

Epilepsy
Epilepsy
Epilepsy
Epilepsy
Epilepsy
Epilepsy
Epilepsy
Epilepsy
Epilepsy
Epilepsy
Epilepsy
Epilepsy
Epilepsy
Epilepsy
Epilepsy
Epilepsy
Epilepsy
Epilepsy
Epilepsy
Epilepsy
Epilepsy
Status epilepticus
Status epilepticus
Status epilepticus
Status epilepticus
Status epilepticus
Status epilepticus
Status epilepticus
Status epilepticus
Status epilepticus
Status epilepticus
Status epilepticus
Status epilepticus
Status epilepticus
Status epilepticus
Status epilepticus
Status epilepticus
Status epilepticus
Status epilepticus
Status epilepticus
Transient cerebral ischaemic attacks and related syndromes
Transient cerebral ischaemic attacks and related syndromes
Transient cerebral ischaemic attacks and related syndromes
Transient cerebral ischaemic attacks and related syndromes
Transient cerebral ischaemic attacks and related syndromes
Transient cerebral ischaemic attacks and related syndromes
Transient cerebral ischaemic attacks and related syndromes
Transient cerebral ischaemic attacks and related syndromes
Transient cerebral ischaemic attacks and related syndromes
Transient cerebral ischaemic attacks and related syndromes

For peer review only

Transient cerebral ischaemic attacks and related syndromes
Sleep disorders
Sleep disorders
Sleep disorders
Sleep disorders
Sleep disorders
Sleep disorders
Sleep disorders
Disorders of other cranial nerves
Other mononeuropathies
Hereditary and idiopathic neuropathy
Hereditary and idiopathic neuropathy
Hereditary and idiopathic neuropathy
Hereditary and idiopathic neuropathy
Hereditary and idiopathic neuropathy
Hereditary and idiopathic neuropathy
Hereditary and idiopathic neuropathy
Inflammatory polyneuropathy
Inflammatory polyneuropathy
Inflammatory polyneuropathy
Inflammatory polyneuropathy
Inflammatory polyneuropathy
Inflammatory polyneuropathy
Inflammatory polyneuropathy
Inflammatory polyneuropathy
Inflammatory polyneuropathy
Inflammatory polyneuropathy
Inflammatory polyneuropathy
Inflammatory polyneuropathy
Other polyneuropathies
Other polyneuropathies
Other polyneuropathies
Other polyneuropathies
Other polyneuropathies
Other polyneuropathies
Other polyneuropathies
Other polyneuropathies
Other polyneuropathies
Other polyneuropathies
Other polyneuropathies
Other polyneuropathies
Other polyneuropathies
Other polyneuropathies
Other polyneuropathies
Other polyneuropathies
Myasthenia gravis and other myoneural disorders
Myasthenia gravis and other myoneural disorders
Myasthenia gravis and other myoneural disorders
Myasthenia gravis and other myoneural disorders
Myasthenia gravis and other myoneural disorders

For peer review only

Myasthenia gravis and other myoneural disorders
Myasthenia gravis and other myoneural disorders
Myasthenia gravis and other myoneural disorders
Myasthenia gravis and other myoneural disorders
Myasthenia gravis and other myoneural disorders
Myasthenia gravis and other myoneural disorders
Myasthenia gravis and other myoneural disorders
Myasthenia gravis and other myoneural disorders
Myasthenia gravis and other myoneural disorders
Myasthenia gravis and other myoneural disorders
Myasthenia gravis and other myoneural disorders
Myasthenia gravis and other myoneural disorders
Myasthenia gravis and other myoneural disorders
Primary disorders of muscles
Primary disorders of muscles
Primary disorders of muscles
Primary disorders of muscles
Primary disorders of muscles
Primary disorders of muscles
Primary disorders of muscles
Primary disorders of muscles
Primary disorders of muscles
Primary disorders of muscles
Primary disorders of muscles
Primary disorders of muscles
Primary disorders of muscles
Primary disorders of muscles
Primary disorders of muscles
Primary disorders of muscles
Primary disorders of muscles
Primary disorders of muscles
Other myopathies
Other myopathies
Other myopathies
Other myopathies
Other myopathies
Other myopathies
Other myopathies
Infantile cerebral palsy
Infantile cerebral palsy
Infantile cerebral palsy
Infantile cerebral palsy
Infantile cerebral palsy
Infantile cerebral palsy
Infantile cerebral palsy
Infantile cerebral palsy
Infantile cerebral palsy
Infantile cerebral palsy
Infantile cerebral palsy
Infantile cerebral palsy

For peer review only

Infantile cerebral palsy
Infantile cerebral palsy
Infantile cerebral palsy
Infantile cerebral palsy
Infantile cerebral palsy
Infantile cerebral palsy
Infantile cerebral palsy
Infantile cerebral palsy
Hemiplegia
Hemiplegia
Hemiplegia
Hemiplegia
Hemiplegia
Hemiplegia
Hemiplegia
Hemiplegia
Paraplegia and tetraplegia
Paraplegia and tetraplegia
Paraplegia and tetraplegia
Paraplegia and tetraplegia
Paraplegia and tetraplegia
Paraplegia and tetraplegia
Paraplegia and tetraplegia
Paraplegia and tetraplegia
Paraplegia and tetraplegia
Paraplegia and tetraplegia
Paraplegia and tetraplegia
Paraplegia and tetraplegia
Paraplegia and tetraplegia
Paraplegia and tetraplegia
Other paralytic syndromes
Other paralytic syndromes
Other paralytic syndromes
Other paralytic syndromes
Other paralytic syndromes
Other paralytic syndromes
Other paralytic syndromes
Other paralytic syndromes
Disorders of autonomic nervous system
Disorders of autonomic nervous system
Disorders of autonomic nervous system
Disorders of autonomic nervous system
Disorders of autonomic nervous system
Disorders of autonomic nervous system
Disorders of autonomic nervous system
Disorders of autonomic nervous system
Disorders of autonomic nervous system
Disorders of autonomic nervous system
Disorders of autonomic nervous system
Hydrocephalus

For peer review only

Hydrocephalus
Hydrocephalus
Hydrocephalus
Hydrocephalus
Hydrocephalus
Hydrocephalus
Hydrocephalus
Hydrocephalus
Hydrocephalus
Hydrocephalus
Hydrocephalus
Hydrocephalus
Hydrocephalus
Hydrocephalus
Hydrocephalus
Hydrocephalus
Hydrocephalus
Toxic encephalopathy
Toxic encephalopathy
Toxic encephalopathy
Toxic encephalopathy
Toxic encephalopathy
Toxic encephalopathy
Toxic encephalopathy
Toxic encephalopathy
Other disorders of brain
Other disorders of brain
Other disorders of brain
Other disorders of brain
Other disorders of brain
Other disorders of brain
Other disorders of brain
Other disorders of brain
Other disorders of brain
Other disorders of brain
Other disorders of brain
Other disorders of brain
Other disorders of brain
Other disorders of brain
Other disorders of brain
Other disorders of brain
Other disorders of brain
Other disorders of brain
Other disorders of brain
Other disorders of brain
Other diseases of spinal cord
Other diseases of spinal cord
Other diseases of spinal cord
Other diseases of spinal cord
Other diseases of spinal cord

For peer review only

Other diseases of spinal cord
Other diseases of spinal cord
Other diseases of spinal cord
Other diseases of spinal cord
Other diseases of spinal cord
Other diseases of spinal cord
Other diseases of spinal cord
Other diseases of spinal cord
Other disorders of central nervous system
Other disorders of central nervous system
Other disorders of central nervous system
Other disorders of central nervous system
Other disorders of central nervous system
Other disorders of central nervous system
Other disorders of nervous system, not elsewhere classified
Other disorders of nervous system, not elsewhere classified
Other disorders of nervous system, not elsewhere classified
Other disorders of nervous system, not elsewhere classified
Other disorders of nervous system, not elsewhere classified
Other disorders of nervous system, not elsewhere classified
Other disorders of nervous system, not elsewhere classified
Other disorders of nervous system, not elsewhere classified
Other disorders of nervous system, not elsewhere classified
Other disorders of nervous system, not elsewhere classified
Disorders of orbit
Otitis externa
Suppurative and unspecified otitis media
Suppurative and unspecified otitis media
Suppurative and unspecified otitis media
Suppurative and unspecified otitis media
Suppurative and unspecified otitis media
Mastoiditis and related conditions
Rheumatic fever without mention of heart involvement
Rheumatic fever with heart involvement
Rheumatic fever with heart involvement
Rheumatic mitral valve diseases
Rheumatic mitral valve diseases
Rheumatic mitral valve diseases
Rheumatic mitral valve diseases
Rheumatic mitral valve diseases
Rheumatic mitral valve diseases
Rheumatic mitral valve diseases
Rheumatic mitral valve diseases
Rheumatic mitral valve diseases
Rheumatic mitral valve diseases
Rheumatic mitral valve diseases
Rheumatic mitral valve diseases
Rheumatic mitral valve diseases
Rheumatic mitral valve diseases
Rheumatic mitral valve diseases

For peer review only

Rheumatic mitral valve diseases
Rheumatic mitral valve diseases
Rheumatic mitral valve diseases
Rheumatic mitral valve diseases
Rheumatic mitral valve diseases
Rheumatic mitral valve diseases
Rheumatic aortic valve diseases
Rheumatic aortic valve diseases
Rheumatic aortic valve diseases
Rheumatic aortic valve diseases
Rheumatic aortic valve diseases
Rheumatic aortic valve diseases
Rheumatic aortic valve diseases
Rheumatic aortic valve diseases
Rheumatic aortic valve diseases
Rheumatic aortic valve diseases
Rheumatic aortic valve diseases
Rheumatic aortic valve diseases
Rheumatic aortic valve diseases
Rheumatic aortic valve diseases
Rheumatic tricuspid valve diseases
Rheumatic tricuspid valve diseases
Rheumatic tricuspid valve diseases
Rheumatic tricuspid valve diseases
Rheumatic tricuspid valve diseases
Rheumatic tricuspid valve diseases
Rheumatic tricuspid valve diseases
Rheumatic tricuspid valve diseases
Rheumatic tricuspid valve diseases
Rheumatic tricuspid valve diseases
Multiple valve diseases
Multiple valve diseases
Multiple valve diseases
Multiple valve diseases
Multiple valve diseases
Multiple valve diseases
Multiple valve diseases
Multiple valve diseases
Multiple valve diseases
Multiple valve diseases
Multiple valve diseases
Multiple valve diseases
Multiple valve diseases
Multiple valve diseases
Multiple valve diseases
Multiple valve diseases
Multiple valve diseases
Multiple valve diseases
Multiple valve diseases
Multiple valve diseases

For peer review only

Multiple valve diseases
Other rheumatic heart diseases
Other rheumatic heart diseases
Other rheumatic heart diseases
Other rheumatic heart diseases
Other rheumatic heart diseases
Other rheumatic heart diseases
Other rheumatic heart diseases
Other rheumatic heart diseases
Other rheumatic heart diseases
Other rheumatic heart diseases
Other rheumatic heart diseases
Other rheumatic heart diseases
Essential (primary) hypertension
Essential (primary) hypertension
Essential (primary) hypertension
Essential (primary) hypertension
Essential (primary) hypertension
Essential (primary) hypertension
Essential (primary) hypertension
Essential (primary) hypertension
Essential (primary) hypertension
Essential (primary) hypertension
Essential (primary) hypertension
Essential (primary) hypertension
Essential (primary) hypertension
Essential (primary) hypertension
Essential (primary) hypertension
Essential (primary) hypertension
Essential (primary) hypertension
Essential (primary) hypertension
Essential (primary) hypertension
Essential (primary) hypertension
Hypertensive heart disease
Hypertensive heart disease
Hypertensive heart disease
Hypertensive heart disease
Hypertensive heart disease
Hypertensive heart disease
Hypertensive heart disease
Hypertensive heart disease
Hypertensive heart disease
Hypertensive heart disease
Hypertensive heart disease
Hypertensive heart disease
Hypertensive heart disease
Hypertensive heart disease
Hypertensive heart disease
Hypertensive heart disease
Hypertensive heart disease

For peer review only

Hypertensive heart disease
Hypertensive heart disease
Hypertensive heart disease
Hypertensive heart disease
Hypertensive renal disease
Hypertensive renal disease
Hypertensive renal disease
Hypertensive renal disease
Hypertensive renal disease
Hypertensive renal disease
Hypertensive renal disease
Hypertensive renal disease
Hypertensive renal disease
Hypertensive renal disease
Hypertensive renal disease
Hypertensive renal disease
Hypertensive renal disease
Hypertensive renal disease
Hypertensive renal disease
Hypertensive renal disease
Hypertensive renal disease
Hypertensive renal disease
Hypertensive renal disease
Hypertensive renal disease
Hypertensive heart and renal disease
Hypertensive heart and renal disease
Hypertensive heart and renal disease
Hypertensive heart and renal disease
Hypertensive heart and renal disease
Hypertensive heart and renal disease
Hypertensive heart and renal disease
Hypertensive heart and renal disease
Hypertensive heart and renal disease
Hypertensive heart and renal disease
Hypertensive heart and renal disease
Hypertensive heart and renal disease
Hypertensive heart and renal disease
Hypertensive heart and renal disease
Hypertensive heart and renal disease
Hypertensive heart and renal disease
Hypertensive heart and renal disease
Hypertensive heart and renal disease
Hypertensive heart and renal disease
Hypertensive heart and renal disease
Angina pectoris
Angina pectoris
Angina pectoris
Angina pectoris
Angina pectoris
Angina pectoris

For peer review only

Angina pectoris
Angina pectoris
Angina pectoris
Angina pectoris
Angina pectoris
Angina pectoris
Angina pectoris
Angina pectoris
Angina pectoris
Angina pectoris
Angina pectoris
Angina pectoris
Angina pectoris
Angina pectoris
Acute myocardial infarction
Acute myocardial infarction
Acute myocardial infarction
Acute myocardial infarction
Acute myocardial infarction
Acute myocardial infarction
Acute myocardial infarction
Acute myocardial infarction
Acute myocardial infarction
Acute myocardial infarction
Acute myocardial infarction
Acute myocardial infarction
Acute myocardial infarction
Acute myocardial infarction
Acute myocardial infarction
Acute myocardial infarction
Acute myocardial infarction
Acute myocardial infarction
Acute myocardial infarction
Acute myocardial infarction
Acute myocardial infarction
Subsequent myocardial infarction
Subsequent myocardial infarction
Subsequent myocardial infarction
Subsequent myocardial infarction
Subsequent myocardial infarction
Subsequent myocardial infarction
Subsequent myocardial infarction
Subsequent myocardial infarction
Subsequent myocardial infarction
Subsequent myocardial infarction
Subsequent myocardial infarction
Subsequent myocardial infarction
Subsequent myocardial infarction
Subsequent myocardial infarction
Subsequent myocardial infarction

For peer review only

Subsequent myocardial infarction
Other acute ischaemic heart diseases
Other acute ischaemic heart diseases
Other acute ischaemic heart diseases
Other acute ischaemic heart diseases
Other acute ischaemic heart diseases
Other acute ischaemic heart diseases
Other acute ischaemic heart diseases
Other acute ischaemic heart diseases
Other acute ischaemic heart diseases
Other acute ischaemic heart diseases
Other acute ischaemic heart diseases
Other acute ischaemic heart diseases
Other acute ischaemic heart diseases
Other acute ischaemic heart diseases
Other acute ischaemic heart diseases
Other acute ischaemic heart diseases
Other acute ischaemic heart diseases
Other acute ischaemic heart diseases
Other acute ischaemic heart diseases
Other acute ischaemic heart diseases
Chronic ischaemic heart disease
Chronic ischaemic heart disease
Chronic ischaemic heart disease
Chronic ischaemic heart disease
Chronic ischaemic heart disease
Chronic ischaemic heart disease
Chronic ischaemic heart disease
Chronic ischaemic heart disease
Chronic ischaemic heart disease
Chronic ischaemic heart disease
Chronic ischaemic heart disease
Chronic ischaemic heart disease
Chronic ischaemic heart disease
Chronic ischaemic heart disease
Chronic ischaemic heart disease
Chronic ischaemic heart disease
Chronic ischaemic heart disease
Chronic ischaemic heart disease
Chronic ischaemic heart disease
Chronic ischaemic heart disease
Chronic ischaemic heart disease
Pulmonary embolism
Pulmonary embolism
Pulmonary embolism
Pulmonary embolism
Pulmonary embolism
Pulmonary embolism
Pulmonary embolism
Pulmonary embolism

For peer review only

Pulmonary embolism
Pulmonary embolism
Pulmonary embolism
Pulmonary embolism
Pulmonary embolism
Pulmonary embolism
Pulmonary embolism
Pulmonary embolism
Pulmonary embolism
Pulmonary embolism
Pulmonary embolism
Pulmonary embolism
Pulmonary embolism
Other pulmonary heart diseases
Other pulmonary heart diseases
Other pulmonary heart diseases
Other pulmonary heart diseases
Other pulmonary heart diseases
Other pulmonary heart diseases
Other pulmonary heart diseases
Other pulmonary heart diseases
Other pulmonary heart diseases
Other pulmonary heart diseases
Other pulmonary heart diseases
Other pulmonary heart diseases
Other pulmonary heart diseases
Other pulmonary heart diseases
Other pulmonary heart diseases
Other pulmonary heart diseases
Other pulmonary heart diseases
Other pulmonary heart diseases
Other pulmonary heart diseases
Other pulmonary heart diseases
Other pulmonary heart diseases
Other diseases of pulmonary vessels
Other diseases of pulmonary vessels
Other diseases of pulmonary vessels
Other diseases of pulmonary vessels
Other diseases of pulmonary vessels
Other diseases of pulmonary vessels
Other diseases of pulmonary vessels
Acute pericarditis
Acute pericarditis
Acute pericarditis
Acute pericarditis
Acute pericarditis
Acute pericarditis
Acute pericarditis
Acute pericarditis
Acute pericarditis

For peer review only

Acute pericarditis
Acute pericarditis
Acute pericarditis
Other diseases of pericardium
Other diseases of pericardium
Other diseases of pericardium
Other diseases of pericardium
Other diseases of pericardium
Other diseases of pericardium
Other diseases of pericardium
Other diseases of pericardium
Other diseases of pericardium
Other diseases of pericardium
Other diseases of pericardium
Other diseases of pericardium
Other diseases of pericardium
Other diseases of pericardium
Other diseases of pericardium
Other diseases of pericardium
Other diseases of pericardium
Acute and subacute endocarditis
Acute and subacute endocarditis
Acute and subacute endocarditis
Acute and subacute endocarditis
Acute and subacute endocarditis
Acute and subacute endocarditis
Acute and subacute endocarditis
Acute and subacute endocarditis
Acute and subacute endocarditis
Acute and subacute endocarditis
Acute and subacute endocarditis
Acute and subacute endocarditis
Acute and subacute endocarditis
Acute and subacute endocarditis
Acute and subacute endocarditis
Acute and subacute endocarditis
Acute and subacute endocarditis
Acute and subacute endocarditis
Acute and subacute endocarditis
Acute and subacute endocarditis
Acute and subacute endocarditis
Nonrheumatic mitral valve disorders
Nonrheumatic mitral valve disorders
Nonrheumatic mitral valve disorders
Nonrheumatic mitral valve disorders
Nonrheumatic mitral valve disorders
Nonrheumatic mitral valve disorders
Nonrheumatic mitral valve disorders
Nonrheumatic mitral valve disorders
Nonrheumatic mitral valve disorders

For peer review only

Nonrheumatic mitral valve disorders
Nonrheumatic mitral valve disorders
Nonrheumatic mitral valve disorders
Nonrheumatic mitral valve disorders
Nonrheumatic mitral valve disorders
Nonrheumatic mitral valve disorders
Nonrheumatic mitral valve disorders
Nonrheumatic mitral valve disorders
Nonrheumatic mitral valve disorders
Nonrheumatic mitral valve disorders
Nonrheumatic mitral valve disorders
Nonrheumatic mitral valve disorders
Nonrheumatic aortic valve disorders
Nonrheumatic aortic valve disorders
Nonrheumatic aortic valve disorders
Nonrheumatic aortic valve disorders
Nonrheumatic aortic valve disorders
Nonrheumatic aortic valve disorders
Nonrheumatic aortic valve disorders
Nonrheumatic aortic valve disorders
Nonrheumatic aortic valve disorders
Nonrheumatic aortic valve disorders
Nonrheumatic aortic valve disorders
Nonrheumatic aortic valve disorders
Nonrheumatic aortic valve disorders
Nonrheumatic aortic valve disorders
Nonrheumatic aortic valve disorders
Nonrheumatic aortic valve disorders
Nonrheumatic aortic valve disorders
Nonrheumatic aortic valve disorders
Nonrheumatic aortic valve disorders
Nonrheumatic aortic valve disorders
Nonrheumatic aortic valve disorders
Nonrheumatic tricuspid valve disorders
Nonrheumatic tricuspid valve disorders
Nonrheumatic tricuspid valve disorders
Nonrheumatic tricuspid valve disorders
Nonrheumatic tricuspid valve disorders
Nonrheumatic tricuspid valve disorders
Nonrheumatic tricuspid valve disorders
Nonrheumatic tricuspid valve disorders
Nonrheumatic tricuspid valve disorders
Pulmonary valve disorders
Pulmonary valve disorders
Endocarditis, valve unspecified
Endocarditis, valve unspecified
Endocarditis, valve unspecified
Endocarditis, valve unspecified
Endocarditis, valve unspecified
Endocarditis, valve unspecified

For peer review only

Endocarditis, valve unspecified
Endocarditis, valve unspecified
Endocarditis, valve unspecified
Endocarditis, valve unspecified
Endocarditis, valve unspecified
Endocarditis, valve unspecified
Endocarditis, valve unspecified
Endocarditis, valve unspecified
Endocarditis, valve unspecified
Endocarditis, valve unspecified
Acute myocarditis
Acute myocarditis
Acute myocarditis
Acute myocarditis
Acute myocarditis
Acute myocarditis
Acute myocarditis
Acute myocarditis
Acute myocarditis
Acute myocarditis
Acute myocarditis
Acute myocarditis
Acute myocarditis
Cardiomyopathy
Cardiomyopathy
Cardiomyopathy
Cardiomyopathy
Cardiomyopathy
Cardiomyopathy
Cardiomyopathy
Cardiomyopathy
Cardiomyopathy
Cardiomyopathy
Cardiomyopathy
Cardiomyopathy
Cardiomyopathy
Cardiomyopathy
Cardiomyopathy
Cardiomyopathy
Cardiomyopathy
Cardiomyopathy
Cardiomyopathy
Cardiomyopathy
Cardiomyopathy
Atrioventricular and left bundle-branch block
Atrioventricular and left bundle-branch block
Atrioventricular and left bundle-branch block
Atrioventricular and left bundle-branch block
Atrioventricular and left bundle-branch block
Atrioventricular and left bundle-branch block

For peer review only

Atrioventricular and left bundle-branch block
Atrioventricular and left bundle-branch block
Atrioventricular and left bundle-branch block
Atrioventricular and left bundle-branch block
Atrioventricular and left bundle-branch block
Atrioventricular and left bundle-branch block
Atrioventricular and left bundle-branch block
Atrioventricular and left bundle-branch block
Atrioventricular and left bundle-branch block
Atrioventricular and left bundle-branch block
Atrioventricular and left bundle-branch block
Other conduction disorders
Other conduction disorders
Other conduction disorders
Other conduction disorders
Other conduction disorders
Other conduction disorders
Other conduction disorders
Other conduction disorders
Other conduction disorders
Other conduction disorders
Other conduction disorders
Other conduction disorders
Other conduction disorders
Other conduction disorders
Cardiac arrest
Cardiac arrest
Cardiac arrest
Cardiac arrest
Cardiac arrest
Cardiac arrest
Cardiac arrest
Cardiac arrest
Cardiac arrest
Cardiac arrest
Cardiac arrest
Cardiac arrest
Cardiac arrest
Cardiac arrest
Cardiac arrest
Cardiac arrest
Cardiac arrest
Cardiac arrest
Cardiac arrest
Cardiac arrest
Paroxysmal tachycardia
Paroxysmal tachycardia
Paroxysmal tachycardia
Paroxysmal tachycardia
Paroxysmal tachycardia

For peer review only

Paroxysmal tachycardia
Paroxysmal tachycardia
Paroxysmal tachycardia
Paroxysmal tachycardia
Paroxysmal tachycardia
Paroxysmal tachycardia
Paroxysmal tachycardia
Paroxysmal tachycardia
Paroxysmal tachycardia
Paroxysmal tachycardia
Atrial fibrillation and flutter
Atrial fibrillation and flutter
Atrial fibrillation and flutter
Atrial fibrillation and flutter
Atrial fibrillation and flutter
Atrial fibrillation and flutter
Atrial fibrillation and flutter
Atrial fibrillation and flutter
Atrial fibrillation and flutter
Atrial fibrillation and flutter
Atrial fibrillation and flutter
Atrial fibrillation and flutter
Atrial fibrillation and flutter
Atrial fibrillation and flutter
Atrial fibrillation and flutter
Atrial fibrillation and flutter
Atrial fibrillation and flutter
Atrial fibrillation and flutter
Atrial fibrillation and flutter
Atrial fibrillation and flutter
Other cardiac arrhythmias
Other cardiac arrhythmias
Other cardiac arrhythmias
Other cardiac arrhythmias
Other cardiac arrhythmias
Other cardiac arrhythmias
Other cardiac arrhythmias
Other cardiac arrhythmias
Other cardiac arrhythmias
Other cardiac arrhythmias
Other cardiac arrhythmias
Other cardiac arrhythmias
Other cardiac arrhythmias
Other cardiac arrhythmias
Other cardiac arrhythmias
Other cardiac arrhythmias
Other cardiac arrhythmias
Other cardiac arrhythmias
Other cardiac arrhythmias
Heart failure

For peer review only

For peer review only - http://bmjopen.bmj.com/site/about/guidelines.xhtml

Heart failure
Heart failure
Heart failure
Heart failure
Heart failure
Heart failure
Heart failure
Heart failure
Heart failure
Heart failure
Heart failure
Heart failure
Heart failure
Heart failure
Heart failure
Heart failure
Heart failure
Heart failure
Heart failure
Complications and ill-defined descriptions of heart disease
Complications and ill-defined descriptions of heart disease
Complications and ill-defined descriptions of heart disease
Complications and ill-defined descriptions of heart disease
Complications and ill-defined descriptions of heart disease
Complications and ill-defined descriptions of heart disease
Complications and ill-defined descriptions of heart disease
Complications and ill-defined descriptions of heart disease
Complications and ill-defined descriptions of heart disease
Complications and ill-defined descriptions of heart disease
Complications and ill-defined descriptions of heart disease
Complications and ill-defined descriptions of heart disease
Complications and ill-defined descriptions of heart disease
Complications and ill-defined descriptions of heart disease
Complications and ill-defined descriptions of heart disease
Complications and ill-defined descriptions of heart disease
Complications and ill-defined descriptions of heart disease
Complications and ill-defined descriptions of heart disease
Complications and ill-defined descriptions of heart disease
Complications and ill-defined descriptions of heart disease
Complications and ill-defined descriptions of heart disease
Subarachnoid haemorrhage
Subarachnoid haemorrhage
Subarachnoid haemorrhage
Subarachnoid haemorrhage
Subarachnoid haemorrhage
Subarachnoid haemorrhage
Subarachnoid haemorrhage
Subarachnoid haemorrhage
Subarachnoid haemorrhage
Subarachnoid haemorrhage

For peer review only

Subarachnoid haemorrhage
Subarachnoid haemorrhage
Subarachnoid haemorrhage
Subarachnoid haemorrhage
Subarachnoid haemorrhage
Subarachnoid haemorrhage
Subarachnoid haemorrhage
Subarachnoid haemorrhage
Subarachnoid haemorrhage
Subarachnoid haemorrhage
Subarachnoid haemorrhage
Intracerebral haemorrhage
Intracerebral haemorrhage
Intracerebral haemorrhage
Intracerebral haemorrhage
Intracerebral haemorrhage
Intracerebral haemorrhage
Intracerebral haemorrhage
Intracerebral haemorrhage
Intracerebral haemorrhage
Intracerebral haemorrhage
Intracerebral haemorrhage
Intracerebral haemorrhage
Intracerebral haemorrhage
Intracerebral haemorrhage
Intracerebral haemorrhage
Intracerebral haemorrhage
Intracerebral haemorrhage
Intracerebral haemorrhage
Intracerebral haemorrhage
Intracerebral haemorrhage
Intracerebral haemorrhage
Other nontraumatic intracranial haemorrhage
Other nontraumatic intracranial haemorrhage
Other nontraumatic intracranial haemorrhage
Other nontraumatic intracranial haemorrhage
Other nontraumatic intracranial haemorrhage
Other nontraumatic intracranial haemorrhage
Other nontraumatic intracranial haemorrhage
Other nontraumatic intracranial haemorrhage
Other nontraumatic intracranial haemorrhage
Other nontraumatic intracranial haemorrhage
Other nontraumatic intracranial haemorrhage
Other nontraumatic intracranial haemorrhage
Other nontraumatic intracranial haemorrhage
Other nontraumatic intracranial haemorrhage
Other nontraumatic intracranial haemorrhage
Other nontraumatic intracranial haemorrhage
Other nontraumatic intracranial haemorrhage
Other nontraumatic intracranial haemorrhage

For peer review only

Other nontraumatic intracranial haemorrhage
Other nontraumatic intracranial haemorrhage
Other nontraumatic intracranial haemorrhage
Cerebral infarction
Cerebral infarction
Cerebral infarction
Cerebral infarction
Cerebral infarction
Cerebral infarction
Cerebral infarction
Cerebral infarction
Cerebral infarction
Cerebral infarction
Cerebral infarction
Cerebral infarction
Cerebral infarction
Cerebral infarction
Cerebral infarction
Cerebral infarction
Cerebral infarction
Cerebral infarction
Cerebral infarction
Cerebral infarction
Cerebral infarction
Stroke, not specified as haemorrhage or infarction
Stroke, not specified as haemorrhage or infarction
Stroke, not specified as haemorrhage or infarction
Stroke, not specified as haemorrhage or infarction
Stroke, not specified as haemorrhage or infarction
Stroke, not specified as haemorrhage or infarction
Stroke, not specified as haemorrhage or infarction
Stroke, not specified as haemorrhage or infarction
Stroke, not specified as haemorrhage or infarction
Stroke, not specified as haemorrhage or infarction
Stroke, not specified as haemorrhage or infarction
Stroke, not specified as haemorrhage or infarction
Stroke, not specified as haemorrhage or infarction
Stroke, not specified as haemorrhage or infarction
Stroke, not specified as haemorrhage or infarction
Stroke, not specified as haemorrhage or infarction
Stroke, not specified as haemorrhage or infarction
Stroke, not specified as haemorrhage or infarction
Stroke, not specified as haemorrhage or infarction
Stroke, not specified as haemorrhage or infarction
Stroke, not specified as haemorrhage or infarction
Other cerebrovascular diseases
Other cerebrovascular diseases
Other cerebrovascular diseases
Other cerebrovascular diseases
Other cerebrovascular diseases

For peer review only

Other cerebrovascular diseases
Other cerebrovascular diseases
Other cerebrovascular diseases
Other cerebrovascular diseases
Other cerebrovascular diseases
Other cerebrovascular diseases
Other cerebrovascular diseases
Other cerebrovascular diseases
Other cerebrovascular diseases
Other cerebrovascular diseases
Other cerebrovascular diseases
Other cerebrovascular diseases
Other cerebrovascular diseases
Other cerebrovascular diseases
Other cerebrovascular diseases
Other cerebrovascular diseases
Sequelae of cerebrovascular disease
Sequelae of cerebrovascular disease
Sequelae of cerebrovascular disease
Sequelae of cerebrovascular disease
Sequelae of cerebrovascular disease
Sequelae of cerebrovascular disease
Sequelae of cerebrovascular disease
Sequelae of cerebrovascular disease
Sequelae of cerebrovascular disease
Sequelae of cerebrovascular disease
Sequelae of cerebrovascular disease
Sequelae of cerebrovascular disease
Sequelae of cerebrovascular disease
Sequelae of cerebrovascular disease
Sequelae of cerebrovascular disease
Sequelae of cerebrovascular disease
Sequelae of cerebrovascular disease
Sequelae of cerebrovascular disease
Sequelae of cerebrovascular disease
Sequelae of cerebrovascular disease
Atherosclerosis
Atherosclerosis
Atherosclerosis
Atherosclerosis
Atherosclerosis
Atherosclerosis
Atherosclerosis
Atherosclerosis
Atherosclerosis
Atherosclerosis
Atherosclerosis
Atherosclerosis
Atherosclerosis
Atherosclerosis

For peer review only

Atherosclerosis
Atherosclerosis
Atherosclerosis
Atherosclerosis
Atherosclerosis
Atherosclerosis
Atherosclerosis
Aortic aneurysm and dissection
Aortic aneurysm and dissection
Aortic aneurysm and dissection
Aortic aneurysm and dissection
Aortic aneurysm and dissection
Aortic aneurysm and dissection
Aortic aneurysm and dissection
Aortic aneurysm and dissection
Aortic aneurysm and dissection
Aortic aneurysm and dissection
Aortic aneurysm and dissection
Aortic aneurysm and dissection
Aortic aneurysm and dissection
Aortic aneurysm and dissection
Aortic aneurysm and dissection
Aortic aneurysm and dissection
Aortic aneurysm and dissection
Aortic aneurysm and dissection
Aortic aneurysm and dissection
Aortic aneurysm and dissection
Aortic aneurysm and dissection
Other aneurysm
Other aneurysm
Other aneurysm
Other aneurysm
Other aneurysm
Other aneurysm
Other aneurysm
Other aneurysm
Other aneurysm
Other aneurysm
Other aneurysm
Other aneurysm
Other aneurysm
Other aneurysm
Other aneurysm
Other aneurysm
Other aneurysm
Other aneurysm
Other aneurysm
Other peripheral vascular diseases
Other peripheral vascular diseases
Other peripheral vascular diseases

For peer review only

Other peripheral vascular diseases
Other peripheral vascular diseases
Other peripheral vascular diseases
Other peripheral vascular diseases
Other peripheral vascular diseases
Other peripheral vascular diseases
Other peripheral vascular diseases
Other peripheral vascular diseases
Other peripheral vascular diseases
Other peripheral vascular diseases
Other peripheral vascular diseases
Other peripheral vascular diseases
Other peripheral vascular diseases
Arterial embolism and thrombosis
Arterial embolism and thrombosis
Arterial embolism and thrombosis
Arterial embolism and thrombosis
Arterial embolism and thrombosis
Arterial embolism and thrombosis
Arterial embolism and thrombosis
Arterial embolism and thrombosis
Arterial embolism and thrombosis
Arterial embolism and thrombosis
Arterial embolism and thrombosis
Arterial embolism and thrombosis
Arterial embolism and thrombosis
Arterial embolism and thrombosis
Arterial embolism and thrombosis
Arterial embolism and thrombosis
Arterial embolism and thrombosis
Arterial embolism and thrombosis
Arterial embolism and thrombosis
Arterial embolism and thrombosis
Arterial embolism and thrombosis
Other disorders of arteries and arterioles
Other disorders of arteries and arterioles
Other disorders of arteries and arterioles
Other disorders of arteries and arterioles
Other disorders of arteries and arterioles
Other disorders of arteries and arterioles
Other disorders of arteries and arterioles
Other disorders of arteries and arterioles
Other disorders of arteries and arterioles
Other disorders of arteries and arterioles
Other disorders of arteries and arterioles
Other disorders of arteries and arterioles
Other disorders of arteries and arterioles
Other disorders of arteries and arterioles
Other disorders of arteries and arterioles
Other disorders of arteries and arterioles

For peer review only

Diseases of capillaries
Diseases of capillaries
Diseases of capillaries
Diseases of capillaries
Diseases of capillaries
Phlebitis and thrombophlebitis
Phlebitis and thrombophlebitis
Phlebitis and thrombophlebitis
Phlebitis and thrombophlebitis
Phlebitis and thrombophlebitis
Phlebitis and thrombophlebitis
Phlebitis and thrombophlebitis
Phlebitis and thrombophlebitis
Phlebitis and thrombophlebitis
Phlebitis and thrombophlebitis
Phlebitis and thrombophlebitis
Phlebitis and thrombophlebitis
Phlebitis and thrombophlebitis
Phlebitis and thrombophlebitis
Phlebitis and thrombophlebitis
Phlebitis and thrombophlebitis
Phlebitis and thrombophlebitis
Phlebitis and thrombophlebitis
Phlebitis and thrombophlebitis
Phlebitis and thrombophlebitis
Phlebitis and thrombophlebitis
Portal vein thrombosis
Portal vein thrombosis
Portal vein thrombosis
Portal vein thrombosis
Portal vein thrombosis
Portal vein thrombosis
Portal vein thrombosis
Portal vein thrombosis
Portal vein thrombosis
Portal vein thrombosis
Other venous embolism and thrombosis
Other venous embolism and thrombosis
Other venous embolism and thrombosis
Other venous embolism and thrombosis
Other venous embolism and thrombosis
Other venous embolism and thrombosis
Other venous embolism and thrombosis
Other venous embolism and thrombosis
Other venous embolism and thrombosis
Other venous embolism and thrombosis
Other venous embolism and thrombosis
Other venous embolism and thrombosis
Other venous embolism and thrombosis
Other venous embolism and thrombosis

For peer review only

Other venous embolism and thrombosis
Other venous embolism and thrombosis
Varicose veins of lower extremities
Varicose veins of lower extremities
Varicose veins of lower extremities
Varicose veins of lower extremities
Varicose veins of lower extremities
Varicose veins of lower extremities
Varicose veins of lower extremities
Varicose veins of lower extremities
Varicose veins of lower extremities
Varicose veins of lower extremities
Varicose veins of lower extremities
Varicose veins of lower extremities
Varicose veins of lower extremities
Varicose veins of lower extremities
Varicose veins of lower extremities
Varicose veins of lower extremities
Varicose veins of lower extremities
Varicose veins of lower extremities
Haemorrhoids
Oesophageal varices
Oesophageal varices
Oesophageal varices
Oesophageal varices
Oesophageal varices
Oesophageal varices
Oesophageal varices
Oesophageal varices
Oesophageal varices
Oesophageal varices
Oesophageal varices
Oesophageal varices
Oesophageal varices
Oesophageal varices
Varicose veins of other sites
Varicose veins of other sites
Other disorders of veins
Other disorders of veins
Other disorders of veins
Other disorders of veins
Other disorders of veins
Other disorders of veins
Other disorders of veins
Other disorders of veins
Other disorders of veins
Other disorders of veins
Other noninfective disorders of lymphatic vessels and lymph nodes
Other noninfective disorders of lymphatic vessels and lymph nodes
Other noninfective disorders of lymphatic vessels and lymph nodes

For peer review only

Other noninfective disorders of lymphatic vessels and lymph nodes
Hypotension
Hypotension
Hypotension
Hypotension
Hypotension
Hypotension
Hypotension
Other and unspecified disorders of circulatory system
Other and unspecified disorders of circulatory system
Other and unspecified disorders of circulatory system
Other and unspecified disorders of circulatory system
Other and unspecified disorders of circulatory system
Other and unspecified disorders of circulatory system
Other and unspecified disorders of circulatory system
Other and unspecified disorders of circulatory system
Other and unspecified disorders of circulatory system
Other and unspecified disorders of circulatory system
Other and unspecified disorders of circulatory system
Other and unspecified disorders of circulatory system
Acute nasopharyngitis [common cold]
Acute pharyngitis
Acute pharyngitis
Acute tonsillitis
Acute laryngitis and tracheitis
Acute laryngitis and tracheitis
Acute laryngitis and tracheitis
Acute laryngitis and tracheitis
Acute obstructive laryngitis [croup] and epiglottitis
Acute upper respiratory infections of multiple or unspecified sites
Acute upper respiratory infections of multiple or unspecified sites
Acute upper respiratory infections of multiple or unspecified sites
Acute upper respiratory infections of multiple or unspecified sites
Acute upper respiratory infections of multiple or unspecified sites
Acute upper respiratory infections of multiple or unspecified sites
Acute upper respiratory infections of multiple or unspecified sites
Acute upper respiratory infections of multiple or unspecified sites
Acute upper respiratory infections of multiple or unspecified sites
Acute upper respiratory infections of multiple or unspecified sites
Influenza due to identified influenza virus
Influenza due to identified influenza virus
Influenza due to identified influenza virus
Influenza due to identified influenza virus
Influenza due to identified influenza virus
Influenza due to identified influenza virus
Influenza due to identified influenza virus
Influenza, virus not identified
Influenza, virus not identified
Influenza, virus not identified
Influenza, virus not identified

For peer review only

Influenza, virus not identified
Influenza, virus not identified
Influenza, virus not identified
Influenza, virus not identified
Influenza, virus not identified
Influenza, virus not identified
Influenza, virus not identified
Influenza, virus not identified
Influenza, virus not identified
Influenza, virus not identified
Influenza, virus not identified
Influenza, virus not identified
Viral pneumonia, not elsewhere classified
Viral pneumonia, not elsewhere classified
Viral pneumonia, not elsewhere classified
Viral pneumonia, not elsewhere classified
Viral pneumonia, not elsewhere classified
Viral pneumonia, not elsewhere classified
Viral pneumonia, not elsewhere classified
Viral pneumonia, not elsewhere classified
Viral pneumonia, not elsewhere classified
Viral pneumonia, not elsewhere classified
Viral pneumonia, not elsewhere classified
Viral pneumonia, not elsewhere classified
Viral pneumonia, not elsewhere classified
Viral pneumonia, not elsewhere classified
Viral pneumonia, not elsewhere classified
Pneumonia due to Streptococcus pneumoniae
Pneumonia due to Streptococcus pneumoniae
Pneumonia due to Streptococcus pneumoniae
Pneumonia due to Streptococcus pneumoniae
Pneumonia due to Streptococcus pneumoniae
Pneumonia due to Streptococcus pneumoniae
Pneumonia due to Streptococcus pneumoniae
Pneumonia due to Streptococcus pneumoniae
Pneumonia due to Streptococcus pneumoniae
Pneumonia due to Streptococcus pneumoniae
Pneumonia due to Streptococcus pneumoniae
Pneumonia due to Streptococcus pneumoniae
Pneumonia due to Streptococcus pneumoniae
Pneumonia due to Streptococcus pneumoniae
Pneumonia due to Haemophilus influenzae
Bacterial pneumonia, not elsewhere classified
Bacterial pneumonia, not elsewhere classified
Bacterial pneumonia, not elsewhere classified
Bacterial pneumonia, not elsewhere classified
Bacterial pneumonia, not elsewhere classified
Bacterial pneumonia, not elsewhere classified
Bacterial pneumonia, not elsewhere classified
Bacterial pneumonia, not elsewhere classified

For peer review only

Bacterial pneumonia, not elsewhere classified
Bacterial pneumonia, not elsewhere classified
Bacterial pneumonia, not elsewhere classified
Bacterial pneumonia, not elsewhere classified
Bacterial pneumonia, not elsewhere classified
Bacterial pneumonia, not elsewhere classified
Bacterial pneumonia, not elsewhere classified
Bacterial pneumonia, not elsewhere classified
Bacterial pneumonia, not elsewhere classified
Bacterial pneumonia, not elsewhere classified
Bacterial pneumonia, not elsewhere classified
Bacterial pneumonia, not elsewhere classified
Pneumonia due to other infectious organisms, not elsewhere classified
Pneumonia due to other infectious organisms, not elsewhere classified
Pneumonia due to other infectious organisms, not elsewhere classified
Pneumonia due to other infectious organisms, not elsewhere classified
Pneumonia, organism unspecified
Pneumonia, organism unspecified
Pneumonia, organism unspecified
Pneumonia, organism unspecified
Pneumonia, organism unspecified
Pneumonia, organism unspecified
Pneumonia, organism unspecified
Pneumonia, organism unspecified
Pneumonia, organism unspecified
Pneumonia, organism unspecified
Pneumonia, organism unspecified
Pneumonia, organism unspecified
Pneumonia, organism unspecified
Pneumonia, organism unspecified
Pneumonia, organism unspecified
Pneumonia, organism unspecified
Pneumonia, organism unspecified
Pneumonia, organism unspecified
Pneumonia, organism unspecified
Pneumonia, organism unspecified
Pneumonia, organism unspecified
Acute bronchitis
Acute bronchitis
Acute bronchitis
Acute bronchitis
Acute bronchitis
Acute bronchitis
Acute bronchitis
Acute bronchitis
Acute bronchitis
Acute bronchitis
Acute bronchitis
Acute bronchitis
Acute bronchitis

For peer review only

Acute bronchitis
Acute bronchitis
Acute bronchitis
Acute bronchitis
Acute bronchiolitis
Acute bronchiolitis
Acute bronchiolitis
Acute bronchiolitis
Acute bronchiolitis
Acute bronchiolitis
Acute bronchiolitis
Acute bronchiolitis
Unspecified acute lower respiratory infection
Unspecified acute lower respiratory infection
Unspecified acute lower respiratory infection
Unspecified acute lower respiratory infection
Unspecified acute lower respiratory infection
Unspecified acute lower respiratory infection
Unspecified acute lower respiratory infection
Unspecified acute lower respiratory infection
Unspecified acute lower respiratory infection
Unspecified acute lower respiratory infection
Unspecified acute lower respiratory infection
Unspecified acute lower respiratory infection
Unspecified acute lower respiratory infection
Unspecified acute lower respiratory infection
Unspecified acute lower respiratory infection
Unspecified acute lower respiratory infection
Chronic sinusitis
Chronic sinusitis
Chronic sinusitis
Diseases of vocal cords and larynx, not elsewhere classified
Diseases of vocal cords and larynx, not elsewhere classified
Diseases of vocal cords and larynx, not elsewhere classified
Diseases of vocal cords and larynx, not elsewhere classified
Diseases of vocal cords and larynx, not elsewhere classified
Diseases of vocal cords and larynx, not elsewhere classified
Other diseases of upper respiratory tract
Other diseases of upper respiratory tract
Other diseases of upper respiratory tract
Other diseases of upper respiratory tract
Other diseases of upper respiratory tract
Other diseases of upper respiratory tract
Other diseases of upper respiratory tract
Other diseases of upper respiratory tract
Other diseases of upper respiratory tract
Bronchitis, not specified as acute or chronic
Bronchitis, not specified as acute or chronic
Bronchitis, not specified as acute or chronic
Bronchitis, not specified as acute or chronic

For peer review only

Bronchitis, not specified as acute or chronic
Bronchitis, not specified as acute or chronic
Bronchitis, not specified as acute or chronic
Bronchitis, not specified as acute or chronic
Bronchitis, not specified as acute or chronic
Bronchitis, not specified as acute or chronic
Bronchitis, not specified as acute or chronic
Bronchitis, not specified as acute or chronic
Bronchitis, not specified as acute or chronic
Bronchitis, not specified as acute or chronic
Simple and mucopurulent chronic bronchitis
Simple and mucopurulent chronic bronchitis
Simple and mucopurulent chronic bronchitis
Simple and mucopurulent chronic bronchitis
Simple and mucopurulent chronic bronchitis
Simple and mucopurulent chronic bronchitis
Simple and mucopurulent chronic bronchitis
Simple and mucopurulent chronic bronchitis
Simple and mucopurulent chronic bronchitis
Simple and mucopurulent chronic bronchitis
Unspecified chronic bronchitis
Unspecified chronic bronchitis
Unspecified chronic bronchitis
Unspecified chronic bronchitis
Unspecified chronic bronchitis
Unspecified chronic bronchitis
Unspecified chronic bronchitis
Unspecified chronic bronchitis
Unspecified chronic bronchitis
Unspecified chronic bronchitis
Unspecified chronic bronchitis
Unspecified chronic bronchitis
Unspecified chronic bronchitis
Unspecified chronic bronchitis
Unspecified chronic bronchitis
Unspecified chronic bronchitis
Unspecified chronic bronchitis
Unspecified chronic bronchitis
Unspecified chronic bronchitis
Unspecified chronic bronchitis
Emphysema
Emphysema
Emphysema
Emphysema
Emphysema
Emphysema
Emphysema
Emphysema
Emphysema
Emphysema

For peer review only

Emphysema
Emphysema
Emphysema
Emphysema
Emphysema
Emphysema
Emphysema
Emphysema
Emphysema
Emphysema
Other chronic obstructive pulmonary disease
Other chronic obstructive pulmonary disease
Other chronic obstructive pulmonary disease
Other chronic obstructive pulmonary disease
Other chronic obstructive pulmonary disease
Other chronic obstructive pulmonary disease
Other chronic obstructive pulmonary disease
Other chronic obstructive pulmonary disease
Other chronic obstructive pulmonary disease
Other chronic obstructive pulmonary disease
Other chronic obstructive pulmonary disease
Other chronic obstructive pulmonary disease
Other chronic obstructive pulmonary disease
Other chronic obstructive pulmonary disease
Other chronic obstructive pulmonary disease
Other chronic obstructive pulmonary disease
Other chronic obstructive pulmonary disease
Other chronic obstructive pulmonary disease
Other chronic obstructive pulmonary disease
Other chronic obstructive pulmonary disease
Other chronic obstructive pulmonary disease
Asthma
Asthma
Asthma
Asthma
Asthma
Asthma
Asthma
Asthma
Asthma
Asthma
Asthma
Asthma
Asthma
Asthma
Asthma
Asthma
Asthma
Asthma
Asthma

For peer review only

Asthma
Asthma
Status asthmaticus
Status asthmaticus
Status asthmaticus
Status asthmaticus
Status asthmaticus
Status asthmaticus
Status asthmaticus
Status asthmaticus
Status asthmaticus
Status asthmaticus
Status asthmaticus
Status asthmaticus
Status asthmaticus
Status asthmaticus
Status asthmaticus
Status asthmaticus
Bronchiectasis
Bronchiectasis
Bronchiectasis
Bronchiectasis
Bronchiectasis
Bronchiectasis
Bronchiectasis
Bronchiectasis
Bronchiectasis
Bronchiectasis
Bronchiectasis
Bronchiectasis
Bronchiectasis
Bronchiectasis
Bronchiectasis
Bronchiectasis
Coalworker's pneumoconiosis
Coalworker's pneumoconiosis
Coalworker's pneumoconiosis
Coalworker's pneumoconiosis
Coalworker's pneumoconiosis
Coalworker's pneumoconiosis
Coalworker's pneumoconiosis
Pneumoconiosis due to asbestos and other mineral fibres
Pneumoconiosis due to asbestos and other mineral fibres
Pneumoconiosis due to asbestos and other mineral fibres
Pneumoconiosis due to asbestos and other mineral fibres
Pneumoconiosis due to asbestos and other mineral fibres
Pneumoconiosis due to asbestos and other mineral fibres
Pneumoconiosis due to asbestos and other mineral fibres
Pneumoconiosis due to asbestos and other mineral fibres
Pneumoconiosis due to asbestos and other mineral fibres

For peer review only

Pneumoconiosis due to asbestos and other mineral fibres
Pneumoconiosis due to asbestos and other mineral fibres
Pneumoconiosis due to dust containing silica
Pneumoconiosis due to dust containing silica
Pneumoconiosis due to dust containing silica
Pneumoconiosis due to dust containing silica
Pneumoconiosis due to dust containing silica
Pneumoconiosis due to dust containing silica
Pneumoconiosis due to dust containing silica
Pneumoconiosis due to dust containing silica
Pneumoconiosis due to dust containing silica
Pneumoconiosis due to dust containing silica
Pneumoconiosis due to dust containing silica
Pneumoconiosis due to dust containing silica
Pneumoconiosis due to dust containing silica
Pneumoconiosis due to other inorganic dusts
Pneumoconiosis due to other inorganic dusts
Unspecified pneumoconiosis
Unspecified pneumoconiosis
Unspecified pneumoconiosis
Unspecified pneumoconiosis
Unspecified pneumoconiosis
Unspecified pneumoconiosis
Unspecified pneumoconiosis
Unspecified pneumoconiosis
Pneumoconiosis associated with tuberculosis
Pneumoconiosis associated with tuberculosis
Pneumoconiosis associated with tuberculosis
Hypersensitivity pneumonitis due to organic dust
Hypersensitivity pneumonitis due to organic dust
Hypersensitivity pneumonitis due to organic dust
Hypersensitivity pneumonitis due to organic dust
Hypersensitivity pneumonitis due to organic dust
Hypersensitivity pneumonitis due to organic dust
Hypersensitivity pneumonitis due to organic dust
Hypersensitivity pneumonitis due to organic dust
Hypersensitivity pneumonitis due to organic dust
Hypersensitivity pneumonitis due to organic dust
Hypersensitivity pneumonitis due to organic dust
Respiratory conditions due to inhalation of chemicals, gases, fumes and vapours
Pneumonitis due to solids and liquids
Pneumonitis due to solids and liquids
Pneumonitis due to solids and liquids
Pneumonitis due to solids and liquids
Pneumonitis due to solids and liquids
Pneumonitis due to solids and liquids
Pneumonitis due to solids and liquids
Pneumonitis due to solids and liquids
Pneumonitis due to solids and liquids
Pneumonitis due to solids and liquids

For peer review only

Pneumonitis due to solids and liquids
Pneumonitis due to solids and liquids
Pneumonitis due to solids and liquids
Pneumonitis due to solids and liquids
Pneumonitis due to solids and liquids
Pneumonitis due to solids and liquids
Pneumonitis due to solids and liquids
Respiratory conditions due to other external agents
Respiratory conditions due to other external agents
Respiratory conditions due to other external agents
Respiratory conditions due to other external agents
Respiratory conditions due to other external agents
Respiratory conditions due to other external agents
Adult respiratory distress syndrome
Adult respiratory distress syndrome
Adult respiratory distress syndrome
Adult respiratory distress syndrome
Adult respiratory distress syndrome
Adult respiratory distress syndrome
Adult respiratory distress syndrome
Adult respiratory distress syndrome
Adult respiratory distress syndrome
Pulmonary oedema
Pulmonary oedema
Pulmonary oedema
Pulmonary oedema
Pulmonary oedema
Pulmonary oedema
Pulmonary oedema
Pulmonary oedema
Pulmonary oedema
Pulmonary oedema
Pulmonary oedema
Pulmonary oedema
Pulmonary oedema
Pulmonary oedema
Pulmonary oedema
Pulmonary eosinophilia, not elsewhere classified
Pulmonary eosinophilia, not elsewhere classified
Pulmonary eosinophilia, not elsewhere classified
Other interstitial pulmonary diseases
Other interstitial pulmonary diseases
Other interstitial pulmonary diseases
Other interstitial pulmonary diseases
Other interstitial pulmonary diseases
Other interstitial pulmonary diseases
Other interstitial pulmonary diseases
Other interstitial pulmonary diseases
Other interstitial pulmonary diseases
Other interstitial pulmonary diseases

For peer review only

Other interstitial pulmonary diseases
Other interstitial pulmonary diseases
Other interstitial pulmonary diseases
Other interstitial pulmonary diseases
Other interstitial pulmonary diseases
Other interstitial pulmonary diseases
Other interstitial pulmonary diseases
Other interstitial pulmonary diseases
Other interstitial pulmonary diseases
Other interstitial pulmonary diseases
Other interstitial pulmonary diseases
Abscess of lung and mediastinum
Abscess of lung and mediastinum
Abscess of lung and mediastinum
Abscess of lung and mediastinum
Abscess of lung and mediastinum
Abscess of lung and mediastinum
Abscess of lung and mediastinum
Abscess of lung and mediastinum
Abscess of lung and mediastinum
Abscess of lung and mediastinum
Abscess of lung and mediastinum
Abscess of lung and mediastinum
Abscess of lung and mediastinum
Abscess of lung and mediastinum
Abscess of lung and mediastinum
Abscess of lung and mediastinum
Abscess of lung and mediastinum
Abscess of lung and mediastinum
Abscess of lung and mediastinum
Pyothorax
Pyothorax
Pyothorax
Pyothorax
Pyothorax
Pyothorax
Pyothorax
Pyothorax
Pyothorax
Pyothorax
Pyothorax
Pyothorax
Pyothorax
Pyothorax
Pyothorax
Pyothorax
Pyothorax
Pyothorax
Pyothorax
Pyothorax

For peer review only

Pleural effusion, not elsewhere classified
Pleural effusion, not elsewhere classified
Pleural effusion, not elsewhere classified
Pleural effusion, not elsewhere classified
Pleural effusion, not elsewhere classified
Pleural effusion, not elsewhere classified
Pleural effusion, not elsewhere classified
Pleural effusion, not elsewhere classified
Pleural effusion, not elsewhere classified
Pleural effusion, not elsewhere classified
Pleural effusion, not elsewhere classified
Pleural effusion, not elsewhere classified
Pleural effusion, not elsewhere classified
Pleural effusion, not elsewhere classified
Pleural plaque
Pleural plaque
Pleural plaque
Pneumothorax
Pneumothorax
Pneumothorax
Pneumothorax
Pneumothorax
Pneumothorax
Pneumothorax
Pneumothorax
Pneumothorax
Pneumothorax
Pneumothorax
Pneumothorax
Other pleural conditions
Other pleural conditions
Other pleural conditions
Other pleural conditions
Other pleural conditions
Other pleural conditions
Other pleural conditions
Other pleural conditions
Other pleural conditions
Other pleural conditions
Other pleural conditions
Other pleural conditions
Respiratory failure, not elsewhere classified
Respiratory failure, not elsewhere classified
Respiratory failure, not elsewhere classified
Respiratory failure, not elsewhere classified
Respiratory failure, not elsewhere classified
Respiratory failure, not elsewhere classified
Respiratory failure, not elsewhere classified
Respiratory failure, not elsewhere classified
Respiratory failure, not elsewhere classified

For peer review only

For peer review only - http://bmjopen.bmj.com/site/about/guidelines.xhtml

Respiratory failure, not elsewhere classified
Respiratory failure, not elsewhere classified
Respiratory failure, not elsewhere classified
Respiratory failure, not elsewhere classified
Respiratory failure, not elsewhere classified
Respiratory failure, not elsewhere classified
Respiratory failure, not elsewhere classified
Respiratory failure, not elsewhere classified
Other respiratory disorders
Other respiratory disorders
Other respiratory disorders
Other respiratory disorders
Other respiratory disorders
Other respiratory disorders
Other respiratory disorders
Other respiratory disorders
Other respiratory disorders
Other respiratory disorders
Other respiratory disorders
Other respiratory disorders
Other respiratory disorders
Other respiratory disorders
Other respiratory disorders
Diseases of pulp and periapical tissues
Other diseases of jaws
Diseases of salivary glands
Diseases of salivary glands
Diseases of salivary glands
Stomatitis and related lesions
Stomatitis and related lesions
Stomatitis and related lesions
Other diseases of lip and oral mucosa
Oesophagitis
Oesophagitis
Oesophagitis
Oesophagitis
Oesophagitis
Oesophagitis
Oesophagitis
Oesophagitis
Oesophagitis
Oesophagitis
Gastro-oesophageal reflux disease
Gastro-oesophageal reflux disease
Gastro-oesophageal reflux disease
Gastro-oesophageal reflux disease
Gastro-oesophageal reflux disease
Gastro-oesophageal reflux disease
Gastro-oesophageal reflux disease
Gastro-oesophageal reflux disease

For peer review only

Gastro-oesophageal reflux disease
Gastro-oesophageal reflux disease
Other diseases of oesophagus
Other diseases of oesophagus
Other diseases of oesophagus
Other diseases of oesophagus
Other diseases of oesophagus
Other diseases of oesophagus
Other diseases of oesophagus
Other diseases of oesophagus
Other diseases of oesophagus
Other diseases of oesophagus
Other diseases of oesophagus
Other diseases of oesophagus
Other diseases of oesophagus
Other diseases of oesophagus
Other diseases of oesophagus
Other diseases of oesophagus
Other diseases of oesophagus
Other diseases of oesophagus
Other diseases of oesophagus
Other diseases of oesophagus
Gastric ulcer
Gastric ulcer
Gastric ulcer
Gastric ulcer
Gastric ulcer
Gastric ulcer
Gastric ulcer
Gastric ulcer
Gastric ulcer
Gastric ulcer
Gastric ulcer
Gastric ulcer
Gastric ulcer
Gastric ulcer
Gastric ulcer
Gastric ulcer
Gastric ulcer
Gastric ulcer
Gastric ulcer
Gastric ulcer
Gastric ulcer
Duodenal ulcer
Duodenal ulcer
Duodenal ulcer
Duodenal ulcer
Duodenal ulcer
Duodenal ulcer

For peer review only

Duodenal ulcer
Duodenal ulcer
Duodenal ulcer
Duodenal ulcer
Duodenal ulcer
Duodenal ulcer
Duodenal ulcer
Duodenal ulcer
Duodenal ulcer
Duodenal ulcer
Duodenal ulcer
Duodenal ulcer
Duodenal ulcer
Duodenal ulcer
Duodenal ulcer
Peptic ulcer, site unspecified
Peptic ulcer, site unspecified
Peptic ulcer, site unspecified
Peptic ulcer, site unspecified
Peptic ulcer, site unspecified
Peptic ulcer, site unspecified
Peptic ulcer, site unspecified
Peptic ulcer, site unspecified
Peptic ulcer, site unspecified
Peptic ulcer, site unspecified
Peptic ulcer, site unspecified
Peptic ulcer, site unspecified
Peptic ulcer, site unspecified
Peptic ulcer, site unspecified
Peptic ulcer, site unspecified
Peptic ulcer, site unspecified
Gastrojejunal ulcer
Gastrojejunal ulcer
Gastrojejunal ulcer
Gastrojejunal ulcer
Gastrojejunal ulcer
Gastrojejunal ulcer
Gastrojejunal ulcer
Gastrojejunal ulcer
Gastritis and duodenitis
Gastritis and duodenitis
Gastritis and duodenitis
Gastritis and duodenitis
Gastritis and duodenitis
Gastritis and duodenitis
Gastritis and duodenitis
Gastritis and duodenitis
Gastritis and duodenitis
Gastritis and duodenitis
Gastritis and duodenitis

For peer review only

Gastritis and duodenitis
Gastritis and duodenitis
Gastritis and duodenitis
Gastritis and duodenitis
Gastritis and duodenitis
Gastritis and duodenitis
Gastritis and duodenitis
Gastritis and duodenitis
Gastritis and duodenitis
Dyspepsia
Other diseases of stomach and duodenum
Other diseases of stomach and duodenum
Other diseases of stomach and duodenum
Other diseases of stomach and duodenum
Other diseases of stomach and duodenum
Other diseases of stomach and duodenum
Other diseases of stomach and duodenum
Other diseases of stomach and duodenum
Other diseases of stomach and duodenum
Other diseases of stomach and duodenum
Other diseases of stomach and duodenum
Other diseases of stomach and duodenum
Other diseases of stomach and duodenum
Other diseases of stomach and duodenum
Other diseases of stomach and duodenum
Acute appendicitis
Acute appendicitis
Acute appendicitis
Acute appendicitis
Acute appendicitis
Acute appendicitis
Acute appendicitis
Acute appendicitis
Acute appendicitis
Acute appendicitis
Acute appendicitis
Acute appendicitis
Acute appendicitis
Acute appendicitis
Acute appendicitis
Acute appendicitis
Acute appendicitis
Acute appendicitis
Unspecified appendicitis
Unspecified appendicitis
Unspecified appendicitis
Unspecified appendicitis
Unspecified appendicitis
Other diseases of appendix
Other diseases of appendix

For peer review only

Inguinal hernia
Inguinal hernia
Inguinal hernia
Inguinal hernia
Inguinal hernia
Inguinal hernia
Inguinal hernia
Inguinal hernia
Inguinal hernia
Inguinal hernia
Inguinal hernia
Inguinal hernia
Inguinal hernia
Inguinal hernia
Inguinal hernia
Inguinal hernia
Inguinal hernia
Inguinal hernia
Inguinal hernia
Inguinal hernia
Femoral hernia
Femoral hernia
Femoral hernia
Femoral hernia
Femoral hernia
Femoral hernia
Femoral hernia
Femoral hernia
Femoral hernia
Femoral hernia
Femoral hernia
Femoral hernia
Femoral hernia
Femoral hernia
Femoral hernia
Umbilical hernia
Umbilical hernia
Umbilical hernia
Umbilical hernia
Umbilical hernia
Umbilical hernia
Umbilical hernia
Umbilical hernia
Umbilical hernia
Umbilical hernia
Umbilical hernia
Umbilical hernia
Umbilical hernia
Umbilical hernia
Umbilical hernia

For peer review only

Umbilical hernia
Umbilical hernia
Ventral hernia
Ventral hernia
Ventral hernia
Ventral hernia
Ventral hernia
Ventral hernia
Ventral hernia
Ventral hernia
Ventral hernia
Ventral hernia
Ventral hernia
Ventral hernia
Ventral hernia
Ventral hernia
Ventral hernia
Ventral hernia
Ventral hernia
Ventral hernia
Ventral hernia
Diaphragmatic hernia
Diaphragmatic hernia
Diaphragmatic hernia
Diaphragmatic hernia
Diaphragmatic hernia
Diaphragmatic hernia
Diaphragmatic hernia
Diaphragmatic hernia
Diaphragmatic hernia
Diaphragmatic hernia
Diaphragmatic hernia
Diaphragmatic hernia
Diaphragmatic hernia
Diaphragmatic hernia
Diaphragmatic hernia
Diaphragmatic hernia
Other abdominal hernia
Other abdominal hernia
Other abdominal hernia
Other abdominal hernia
Other abdominal hernia
Other abdominal hernia
Other abdominal hernia
Other abdominal hernia
Unspecified hernia of abdominal cavity
Unspecified hernia of abdominal cavity
Unspecified hernia of abdominal cavity
Unspecified hernia of abdominal cavity
Unspecified hernia of abdominal cavity

For peer review only

Unspecified hernia of abdominal cavity
Unspecified hernia of abdominal cavity
Unspecified hernia of abdominal cavity
Unspecified hernia of abdominal cavity
Unspecified hernia of abdominal cavity
Unspecified hernia of abdominal cavity
Unspecified hernia of abdominal cavity
Unspecified hernia of abdominal cavity
Unspecified hernia of abdominal cavity
Crohn's disease [regional enteritis]
Crohn's disease [regional enteritis]
Crohn's disease [regional enteritis]
Crohn's disease [regional enteritis]
Crohn's disease [regional enteritis]
Crohn's disease [regional enteritis]
Crohn's disease [regional enteritis]
Crohn's disease [regional enteritis]
Crohn's disease [regional enteritis]
Crohn's disease [regional enteritis]
Crohn's disease [regional enteritis]
Crohn's disease [regional enteritis]
Crohn's disease [regional enteritis]
Crohn's disease [regional enteritis]
Crohn's disease [regional enteritis]
Crohn's disease [regional enteritis]
Crohn's disease [regional enteritis]
Ulcerative colitis
Ulcerative colitis
Ulcerative colitis
Ulcerative colitis
Ulcerative colitis
Ulcerative colitis
Ulcerative colitis
Ulcerative colitis
Ulcerative colitis
Ulcerative colitis
Ulcerative colitis
Ulcerative colitis
Ulcerative colitis
Ulcerative colitis
Ulcerative colitis
Ulcerative colitis
Ulcerative colitis
Ulcerative colitis
Ulcerative colitis
Ulcerative colitis
Ulcerative colitis
Other noninfective gastroenteritis and colitis
Other noninfective gastroenteritis and colitis
Other noninfective gastroenteritis and colitis

For peer review only

Other noninfective gastroenteritis and colitis
Other noninfective gastroenteritis and colitis
Other noninfective gastroenteritis and colitis
Other noninfective gastroenteritis and colitis
Other noninfective gastroenteritis and colitis
Other noninfective gastroenteritis and colitis
Other noninfective gastroenteritis and colitis
Other noninfective gastroenteritis and colitis
Other noninfective gastroenteritis and colitis
Other noninfective gastroenteritis and colitis
Other noninfective gastroenteritis and colitis
Other noninfective gastroenteritis and colitis
Other noninfective gastroenteritis and colitis
Vascular disorders of intestine
Vascular disorders of intestine
Vascular disorders of intestine
Vascular disorders of intestine
Vascular disorders of intestine
Vascular disorders of intestine
Vascular disorders of intestine
Vascular disorders of intestine
Vascular disorders of intestine
Vascular disorders of intestine
Vascular disorders of intestine
Vascular disorders of intestine
Vascular disorders of intestine
Vascular disorders of intestine
Vascular disorders of intestine
Vascular disorders of intestine
Vascular disorders of intestine
Vascular disorders of intestine
Vascular disorders of intestine
Vascular disorders of intestine
Vascular disorders of intestine
Paralytic ileus and intestinal obstruction without hernia
Paralytic ileus and intestinal obstruction without hernia
Paralytic ileus and intestinal obstruction without hernia
Paralytic ileus and intestinal obstruction without hernia
Paralytic ileus and intestinal obstruction without hernia
Paralytic ileus and intestinal obstruction without hernia
Paralytic ileus and intestinal obstruction without hernia
Paralytic ileus and intestinal obstruction without hernia
Paralytic ileus and intestinal obstruction without hernia
Paralytic ileus and intestinal obstruction without hernia
Paralytic ileus and intestinal obstruction without hernia
Paralytic ileus and intestinal obstruction without hernia
Paralytic ileus and intestinal obstruction without hernia
Paralytic ileus and intestinal obstruction without hernia
Paralytic ileus and intestinal obstruction without hernia
Paralytic ileus and intestinal obstruction without hernia

For peer review only

Paralytic ileus and intestinal obstruction without hernia
Paralytic ileus and intestinal obstruction without hernia
Paralytic ileus and intestinal obstruction without hernia
Paralytic ileus and intestinal obstruction without hernia
Paralytic ileus and intestinal obstruction without hernia
Diverticular disease of intestine
Diverticular disease of intestine
Diverticular disease of intestine
Diverticular disease of intestine
Diverticular disease of intestine
Diverticular disease of intestine
Diverticular disease of intestine
Diverticular disease of intestine
Diverticular disease of intestine
Diverticular disease of intestine
Diverticular disease of intestine
Diverticular disease of intestine
Diverticular disease of intestine
Diverticular disease of intestine
Diverticular disease of intestine
Diverticular disease of intestine
Diverticular disease of intestine
Diverticular disease of intestine
Diverticular disease of intestine
Diverticular disease of intestine
Diverticular disease of intestine
Irritable bowel syndrome
Other functional intestinal disorders
Other functional intestinal disorders
Other functional intestinal disorders
Other functional intestinal disorders
Other functional intestinal disorders
Other functional intestinal disorders
Other functional intestinal disorders
Other functional intestinal disorders
Other functional intestinal disorders
Other functional intestinal disorders
Other functional intestinal disorders
Other functional intestinal disorders
Other functional intestinal disorders
Fissure and fistula of anal and rectal regions
Fissure and fistula of anal and rectal regions
Abscess of anal and rectal regions
Abscess of anal and rectal regions
Abscess of anal and rectal regions
Abscess of anal and rectal regions
Abscess of anal and rectal regions
Abscess of anal and rectal regions
Abscess of anal and rectal regions
Abscess of anal and rectal regions

For peer review only

Abscess of anal and rectal regions
Other diseases of anus and rectum
Other diseases of anus and rectum
Other diseases of anus and rectum
Other diseases of anus and rectum
Other diseases of anus and rectum
Other diseases of anus and rectum
Other diseases of anus and rectum
Other diseases of anus and rectum
Other diseases of anus and rectum
Other diseases of anus and rectum
Other diseases of anus and rectum
Other diseases of anus and rectum
Other diseases of anus and rectum
Other diseases of intestine
Other diseases of intestine
Other diseases of intestine
Other diseases of intestine
Other diseases of intestine
Other diseases of intestine
Other diseases of intestine
Other diseases of intestine
Other diseases of intestine
Other diseases of intestine
Other diseases of intestine
Other diseases of intestine
Other diseases of intestine
Other diseases of intestine
Other diseases of intestine
Other diseases of intestine
Other diseases of intestine
Other diseases of intestine
Other diseases of intestine
Other diseases of intestine
Other diseases of intestine
Peritonitis
Peritonitis
Peritonitis
Peritonitis
Peritonitis
Peritonitis
Peritonitis
Peritonitis
Peritonitis
Peritonitis
Peritonitis
Peritonitis
Peritonitis
Peritonitis
Peritonitis

For peer review only

Peritonitis
Peritonitis
Peritonitis
Peritonitis
Peritonitis
Other disorders of peritoneum
Other disorders of peritoneum
Other disorders of peritoneum
Other disorders of peritoneum
Other disorders of peritoneum
Other disorders of peritoneum
Other disorders of peritoneum
Other disorders of peritoneum
Other disorders of peritoneum
Other disorders of peritoneum
Other disorders of peritoneum
Other disorders of peritoneum
Alcoholic liver disease
Alcoholic liver disease
Alcoholic liver disease
Alcoholic liver disease
Alcoholic liver disease
Alcoholic liver disease
Alcoholic liver disease
Alcoholic liver disease
Alcoholic liver disease
Alcoholic liver disease
Alcoholic liver disease
Alcoholic liver disease
Alcoholic liver disease
Alcoholic liver disease
Alcoholic liver disease
Alcoholic liver disease
Alcoholic liver disease
Alcoholic liver disease
Alcoholic liver disease
Alcoholic liver disease
Alcoholic liver disease
Toxic liver disease
Toxic liver disease
Toxic liver disease
Toxic liver disease
Toxic liver disease
Toxic liver disease
Toxic liver disease
Toxic liver disease
Toxic liver disease
Toxic liver disease
Toxic liver disease
Toxic liver disease

For peer review only

Toxic liver disease
Toxic liver disease
Toxic liver disease
Hepatic failure, not elsewhere classified
Hepatic failure, not elsewhere classified
Hepatic failure, not elsewhere classified
Hepatic failure, not elsewhere classified
Hepatic failure, not elsewhere classified
Hepatic failure, not elsewhere classified
Hepatic failure, not elsewhere classified
Hepatic failure, not elsewhere classified
Hepatic failure, not elsewhere classified
Hepatic failure, not elsewhere classified
Hepatic failure, not elsewhere classified
Hepatic failure, not elsewhere classified
Hepatic failure, not elsewhere classified
Hepatic failure, not elsewhere classified
Hepatic failure, not elsewhere classified
Hepatic failure, not elsewhere classified
Hepatic failure, not elsewhere classified
Hepatic failure, not elsewhere classified
Chronic hepatitis, not elsewhere classified
Chronic hepatitis, not elsewhere classified
Chronic hepatitis, not elsewhere classified
Chronic hepatitis, not elsewhere classified
Chronic hepatitis, not elsewhere classified
Chronic hepatitis, not elsewhere classified
Chronic hepatitis, not elsewhere classified
Chronic hepatitis, not elsewhere classified
Chronic hepatitis, not elsewhere classified
Chronic hepatitis, not elsewhere classified
Chronic hepatitis, not elsewhere classified
Fibrosis and cirrhosis of liver
Fibrosis and cirrhosis of liver
Fibrosis and cirrhosis of liver
Fibrosis and cirrhosis of liver
Fibrosis and cirrhosis of liver
Fibrosis and cirrhosis of liver
Fibrosis and cirrhosis of liver
Fibrosis and cirrhosis of liver
Fibrosis and cirrhosis of liver
Fibrosis and cirrhosis of liver
Fibrosis and cirrhosis of liver
Fibrosis and cirrhosis of liver
Fibrosis and cirrhosis of liver
Fibrosis and cirrhosis of liver
Fibrosis and cirrhosis of liver
Fibrosis and cirrhosis of liver
Fibrosis and cirrhosis of liver
Fibrosis and cirrhosis of liver

For peer review only

Fibrosis and cirrhosis of liver
Fibrosis and cirrhosis of liver
Fibrosis and cirrhosis of liver
Other inflammatory liver diseases
Other inflammatory liver diseases
Other inflammatory liver diseases
Other inflammatory liver diseases
Other inflammatory liver diseases
Other inflammatory liver diseases
Other inflammatory liver diseases
Other inflammatory liver diseases
Other inflammatory liver diseases
Other inflammatory liver diseases
Other inflammatory liver diseases
Other inflammatory liver diseases
Other inflammatory liver diseases
Other inflammatory liver diseases
Other inflammatory liver diseases
Other inflammatory liver diseases
Other inflammatory liver diseases
Other inflammatory liver diseases
Other diseases of liver
Other diseases of liver
Other diseases of liver
Other diseases of liver
Other diseases of liver
Other diseases of liver
Other diseases of liver
Other diseases of liver
Other diseases of liver
Other diseases of liver
Other diseases of liver
Other diseases of liver
Other diseases of liver
Other diseases of liver
Other diseases of liver
Other diseases of liver
Other diseases of liver
Other diseases of liver
Other diseases of liver
Other diseases of liver
Other diseases of liver
Cholelithiasis
Cholelithiasis
Cholelithiasis
Cholelithiasis
Cholelithiasis
Cholelithiasis
Cholelithiasis
Cholelithiasis

For peer review only

Cholelithiasis
Cholelithiasis
Cholelithiasis
Cholelithiasis
Cholelithiasis
Cholelithiasis
Cholelithiasis
Cholelithiasis
Cholelithiasis
Cholelithiasis
Cholelithiasis
Cholelithiasis
Cholelithiasis
Cholecystitis
Cholecystitis
Cholecystitis
Cholecystitis
Cholecystitis
Cholecystitis
Cholecystitis
Cholecystitis
Cholecystitis
Cholecystitis
Cholecystitis
Cholecystitis
Cholecystitis
Cholecystitis
Cholecystitis
Cholecystitis
Cholecystitis
Cholecystitis
Cholecystitis
Cholecystitis
Cholecystitis
Other diseases of gallbladder
Other diseases of gallbladder
Other diseases of gallbladder
Other diseases of gallbladder
Other diseases of gallbladder
Other diseases of gallbladder
Other diseases of gallbladder
Other diseases of gallbladder
Other diseases of gallbladder
Other diseases of gallbladder
Other diseases of gallbladder
Other diseases of gallbladder
Other diseases of gallbladder
Other diseases of gallbladder
Other diseases of gallbladder
Other diseases of gallbladder

For peer review only

Other diseases of biliary tract
Other diseases of biliary tract
Other diseases of biliary tract
Other diseases of biliary tract
Other diseases of biliary tract
Other diseases of biliary tract
Other diseases of biliary tract
Other diseases of biliary tract
Other diseases of biliary tract
Other diseases of biliary tract
Other diseases of biliary tract
Other diseases of biliary tract
Other diseases of biliary tract
Other diseases of biliary tract
Other diseases of biliary tract
Other diseases of biliary tract
Other diseases of biliary tract
Other diseases of biliary tract
Other diseases of biliary tract
Acute pancreatitis
Acute pancreatitis
Acute pancreatitis
Acute pancreatitis
Acute pancreatitis
Acute pancreatitis
Acute pancreatitis
Acute pancreatitis
Acute pancreatitis
Acute pancreatitis
Acute pancreatitis
Acute pancreatitis
Acute pancreatitis
Acute pancreatitis
Acute pancreatitis
Acute pancreatitis
Acute pancreatitis
Acute pancreatitis
Acute pancreatitis
Acute pancreatitis
Acute pancreatitis
Other diseases of pancreas
Other diseases of pancreas
Other diseases of pancreas
Other diseases of pancreas
Other diseases of pancreas
Other diseases of pancreas
Other diseases of pancreas
Other diseases of pancreas
Other diseases of pancreas
Other diseases of pancreas

For peer review only

Other diseases of pancreas
Other diseases of pancreas
Other diseases of pancreas
Other diseases of pancreas
Other diseases of pancreas
Other diseases of pancreas
Other diseases of pancreas
Other diseases of pancreas
Other diseases of pancreas
Other diseases of pancreas
Other diseases of pancreas
Intestinal malabsorption
Intestinal malabsorption
Intestinal malabsorption
Intestinal malabsorption
Intestinal malabsorption
Intestinal malabsorption
Intestinal malabsorption
Intestinal malabsorption
Other diseases of digestive system
Other diseases of digestive system
Other diseases of digestive system
Other diseases of digestive system
Other diseases of digestive system
Other diseases of digestive system
Other diseases of digestive system
Other diseases of digestive system
Other diseases of digestive system
Other diseases of digestive system
Other diseases of digestive system
Other diseases of digestive system
Other diseases of digestive system
Other diseases of digestive system
Other diseases of digestive system
Other diseases of digestive system
Other diseases of digestive system
Other diseases of digestive system
Other diseases of digestive system
Other diseases of digestive system
Other diseases of digestive system
Cutaneous abscess, furuncle and carbuncle
Cutaneous abscess, furuncle and carbuncle
Cutaneous abscess, furuncle and carbuncle
Cutaneous abscess, furuncle and carbuncle
Cutaneous abscess, furuncle and carbuncle
Cutaneous abscess, furuncle and carbuncle
Cutaneous abscess, furuncle and carbuncle
Cutaneous abscess, furuncle and carbuncle
Cutaneous abscess, furuncle and carbuncle
Cutaneous abscess, furuncle and carbuncle

For peer review only

For peer review only - http://bmjopen.bmj.com/site/about/guidelines.xhtml

Cutaneous abscess, furuncle and carbuncle
Cutaneous abscess, furuncle and carbuncle
Cutaneous abscess, furuncle and carbuncle
Cutaneous abscess, furuncle and carbuncle
Cellulitis
Cellulitis
Cellulitis
Cellulitis
Cellulitis
Cellulitis
Cellulitis
Cellulitis
Cellulitis
Cellulitis
Cellulitis
Cellulitis
Cellulitis
Other local infections of skin and subcutaneous tissue
Other local infections of skin and subcutaneous tissue
Other local infections of skin and subcutaneous tissue
Other local infections of skin and subcutaneous tissue
Other local infections of skin and subcutaneous tissue
Other local infections of skin and subcutaneous tissue
Other local infections of skin and subcutaneous tissue
Other local infections of skin and subcutaneous tissue
Other local infections of skin and subcutaneous tissue
Other local infections of skin and subcutaneous tissue
Pemphigus
Pemphigus
Pemphigus
Pemphigus
Pemphigus
Pemphigoid
Pemphigoid
Pemphigoid
Pemphigoid
Pemphigoid
Pemphigoid
Pemphigoid
Other bullous disorders
Other dermatitis
Psoriasis
Psoriasis
Psoriasis
Psoriasis
Erythema multiforme
Erythema multiforme
Erythema multiforme
Erythema multiforme
Other erythematous conditions

For peer review only

Other erythematous conditions
Pyoderma gangrenosum
Pyoderma gangrenosum
Pyoderma gangrenosum
Decubitus ulcer
Decubitus ulcer
Decubitus ulcer
Decubitus ulcer
Decubitus ulcer
Decubitus ulcer
Decubitus ulcer
Decubitus ulcer
Decubitus ulcer
Decubitus ulcer
Decubitus ulcer
Decubitus ulcer
Decubitus ulcer
Decubitus ulcer
Decubitus ulcer
Decubitus ulcer
Decubitus ulcer
Lupus erythematosus
Lupus erythematosus
Lupus erythematosus
Lupus erythematosus
Lupus erythematosus
Lupus erythematosus
Ulcer of lower limb, not elsewhere classified
Ulcer of lower limb, not elsewhere classified
Ulcer of lower limb, not elsewhere classified
Ulcer of lower limb, not elsewhere classified
Ulcer of lower limb, not elsewhere classified
Ulcer of lower limb, not elsewhere classified
Ulcer of lower limb, not elsewhere classified
Ulcer of lower limb, not elsewhere classified
Ulcer of lower limb, not elsewhere classified
Ulcer of lower limb, not elsewhere classified
Ulcer of lower limb, not elsewhere classified
Other disorders of skin and subcutaneous tissue, not elsewhere classified
Other disorders of skin and subcutaneous tissue, not elsewhere classified
Other disorders of skin and subcutaneous tissue, not elsewhere classified
Other disorders of skin and subcutaneous tissue, not elsewhere classified
Other disorders of skin and subcutaneous tissue, not elsewhere classified
Other disorders of skin and subcutaneous tissue, not elsewhere classified
Other disorders of skin and subcutaneous tissue, not elsewhere classified
Other disorders of skin and subcutaneous tissue, not elsewhere classified
Other disorders of skin and subcutaneous tissue, not elsewhere classified
Pyogenic arthritis
Pyogenic arthritis
Pyogenic arthritis

For peer review only

Pyogenic arthritis
Pyogenic arthritis
Pyogenic arthritis
Pyogenic arthritis
Pyogenic arthritis
Pyogenic arthritis
Pyogenic arthritis
Pyogenic arthritis
Pyogenic arthritis
Pyogenic arthritis
Pyogenic arthritis
Pyogenic arthritis
Seropositive rheumatoid arthritis
Seropositive rheumatoid arthritis
Seropositive rheumatoid arthritis
Seropositive rheumatoid arthritis
Seropositive rheumatoid arthritis
Seropositive rheumatoid arthritis
Seropositive rheumatoid arthritis
Seropositive rheumatoid arthritis
Seropositive rheumatoid arthritis
Seropositive rheumatoid arthritis
Seropositive rheumatoid arthritis
Seropositive rheumatoid arthritis
Seropositive rheumatoid arthritis
Seropositive rheumatoid arthritis
Seropositive rheumatoid arthritis
Seropositive rheumatoid arthritis
Seropositive rheumatoid arthritis
Other rheumatoid arthritis
Other rheumatoid arthritis
Other rheumatoid arthritis
Other rheumatoid arthritis
Other rheumatoid arthritis
Other rheumatoid arthritis
Other rheumatoid arthritis
Other rheumatoid arthritis
Other rheumatoid arthritis
Other rheumatoid arthritis
Other rheumatoid arthritis
Other rheumatoid arthritis
Other rheumatoid arthritis
Other rheumatoid arthritis
Other rheumatoid arthritis
Other rheumatoid arthritis
Other rheumatoid arthritis
Other rheumatoid arthritis
Other rheumatoid arthritis
Other rheumatoid arthritis
Gout

For peer review only

Gout
Gout
Gout
Gout
Gout
Gout
Other crystal arthropathies
Other arthritis
Other arthritis
Other arthritis
Other arthritis
Other arthritis
Other arthritis
Other arthritis
Other arthritis
Other arthritis
Other arthritis
Other arthritis
Other arthritis
Polyarthrosis
Polyarthrosis
Polyarthrosis
Polyarthrosis
Polyarthrosis
Polyarthrosis
Polyarthrosis
Polyarthrosis
Polyarthrosis
Polyarthrosis
Polyarthrosis
Polyarthrosis
Coxarthrosis [arthrosis of hip]
Coxarthrosis [arthrosis of hip]
Coxarthrosis [arthrosis of hip]
Coxarthrosis [arthrosis of hip]
Coxarthrosis [arthrosis of hip]
Coxarthrosis [arthrosis of hip]
Coxarthrosis [arthrosis of hip]
Coxarthrosis [arthrosis of hip]
Coxarthrosis [arthrosis of hip]
Coxarthrosis [arthrosis of hip]
Coxarthrosis [arthrosis of hip]
Coxarthrosis [arthrosis of hip]
Gonarthrosis [arthrosis of knee]
Gonarthrosis [arthrosis of knee]
Gonarthrosis [arthrosis of knee]
Gonarthrosis [arthrosis of knee]
Gonarthrosis [arthrosis of knee]
Gonarthrosis [arthrosis of knee]
Gonarthrosis [arthrosis of knee]

For peer review only

Gonarthrosis [arthrosis of knee]
Gonarthrosis [arthrosis of knee]
Gonarthrosis [arthrosis of knee]
Gonarthrosis [arthrosis of knee]
Other arthrosis
Other arthrosis
Other arthrosis
Other arthrosis
Other arthrosis
Other arthrosis
Other arthrosis
Other arthrosis
Other specific joint derangements
Other specific joint derangements
Other joint disorders, not elsewhere classified
Other joint disorders, not elsewhere classified
Other joint disorders, not elsewhere classified
Other joint disorders, not elsewhere classified
Other joint disorders, not elsewhere classified
Other joint disorders, not elsewhere classified
Polyarteritis nodosa and related conditions
Polyarteritis nodosa and related conditions
Polyarteritis nodosa and related conditions
Polyarteritis nodosa and related conditions
Polyarteritis nodosa and related conditions
Polyarteritis nodosa and related conditions
Other necrotizing vasculopathies
Other necrotizing vasculopathies
Other necrotizing vasculopathies
Other necrotizing vasculopathies
Other necrotizing vasculopathies
Other necrotizing vasculopathies
Other necrotizing vasculopathies
Other necrotizing vasculopathies
Other necrotizing vasculopathies
Other necrotizing vasculopathies
Other necrotizing vasculopathies
Other necrotizing vasculopathies
Other necrotizing vasculopathies
Other necrotizing vasculopathies
Other necrotizing vasculopathies
Other necrotizing vasculopathies
Other necrotizing vasculopathies
Other necrotizing vasculopathies
Systemic lupus erythematosus
Systemic lupus erythematosus
Systemic lupus erythematosus
Systemic lupus erythematosus
Systemic lupus erythematosus
Systemic lupus erythematosus

For peer review only

Systemic lupus erythematosus
Systemic lupus erythematosus
Systemic lupus erythematosus
Systemic lupus erythematosus
Systemic lupus erythematosus
Systemic lupus erythematosus
Systemic lupus erythematosus
Systemic lupus erythematosus
Systemic lupus erythematosus
Systemic lupus erythematosus
Systemic lupus erythematosus
Systemic lupus erythematosus
Systemic lupus erythematosus
Systemic lupus erythematosus
Dermatopolymyositis
Dermatopolymyositis
Dermatopolymyositis
Dermatopolymyositis
Dermatopolymyositis
Dermatopolymyositis
Dermatopolymyositis
Dermatopolymyositis
Dermatopolymyositis
Dermatopolymyositis
Dermatopolymyositis
Systemic sclerosis
Systemic sclerosis
Systemic sclerosis
Systemic sclerosis
Systemic sclerosis
Systemic sclerosis
Systemic sclerosis
Systemic sclerosis
Systemic sclerosis
Systemic sclerosis
Systemic sclerosis
Systemic sclerosis
Systemic sclerosis
Systemic sclerosis
Systemic sclerosis
Systemic sclerosis
Systemic sclerosis
Other systemic involvement of connective tissue
Other systemic involvement of connective tissue
Other systemic involvement of connective tissue
Other systemic involvement of connective tissue
Other systemic involvement of connective tissue
Other systemic involvement of connective tissue
Other systemic involvement of connective tissue
Other systemic involvement of connective tissue

For peer review only

Other systemic involvement of connective tissue
Other systemic involvement of connective tissue
Other systemic involvement of connective tissue
Other systemic involvement of connective tissue
Other systemic involvement of connective tissue
Other systemic involvement of connective tissue
Other systemic involvement of connective tissue
Other systemic involvement of connective tissue
Kyphosis and lordosis
Kyphosis and lordosis
Kyphosis and lordosis
Scoliosis
Scoliosis
Scoliosis
Scoliosis
Scoliosis
Scoliosis
Scoliosis
Scoliosis
Scoliosis
Other deforming dorsopathies
Ankylosing spondylitis
Ankylosing spondylitis
Ankylosing spondylitis
Ankylosing spondylitis
Ankylosing spondylitis
Ankylosing spondylitis
Ankylosing spondylitis
Other inflammatory spondylopathies
Other inflammatory spondylopathies
Other inflammatory spondylopathies
Other inflammatory spondylopathies
Other inflammatory spondylopathies
Other inflammatory spondylopathies
Other inflammatory spondylopathies
Other inflammatory spondylopathies
Other inflammatory spondylopathies
Other inflammatory spondylopathies
Other inflammatory spondylopathies
Other inflammatory spondylopathies
Other inflammatory spondylopathies
Spondylosis
Spondylosis
Spondylosis
Spondylosis
Spondylosis
Spondylosis
Other spondylopathies
Other spondylopathies
Other spondylopathies

For peer review only

Other spondylopathies
Other spondylopathies
Other spondylopathies
Other spondylopathies
Other spondylopathies
Other spondylopathies
Other spondylopathies
Other spondylopathies
Cervical disc disorders
Cervical disc disorders
Other intervertebral disc disorders
Other intervertebral disc disorders
Other intervertebral disc disorders
Other intervertebral disc disorders
Dorsalgia
Dorsalgia
Dorsalgia
Dorsalgia
Myositis
Myositis
Myositis
Myositis
Other disorders of muscle
Other disorders of muscle
Other disorders of muscle
Other disorders of muscle
Other disorders of muscle
Other disorders of muscle
Other disorders of muscle
Other disorders of muscle
Other disorders of muscle
Other disorders of muscle
Other disorders of muscle
Other disorders of muscle
Soft tissue disorders related to use, overuse and pressure
Soft tissue disorders related to use, overuse and pressure
Fibroblastic disorders
Fibroblastic disorders
Fibroblastic disorders
Fibroblastic disorders
Fibroblastic disorders
Fibroblastic disorders
Fibroblastic disorders
Fibroblastic disorders
Fibroblastic disorders
Other soft tissue disorders, not elsewhere classified
Other soft tissue disorders, not elsewhere classified
Other soft tissue disorders, not elsewhere classified
Other soft tissue disorders, not elsewhere classified
Other soft tissue disorders, not elsewhere classified

For peer review only

Osteoporosis with pathological fracture
Osteoporosis with pathological fracture
Osteoporosis with pathological fracture
Osteoporosis with pathological fracture
Osteoporosis with pathological fracture
Osteoporosis with pathological fracture
Osteoporosis with pathological fracture
Osteoporosis with pathological fracture
Osteoporosis with pathological fracture
Osteoporosis with pathological fracture
Osteoporosis with pathological fracture
Osteoporosis with pathological fracture
Osteoporosis with pathological fracture
Osteoporosis with pathological fracture
Osteoporosis with pathological fracture
Osteoporosis with pathological fracture
Osteoporosis without pathological fracture
Osteoporosis without pathological fracture
Osteoporosis without pathological fracture
Osteoporosis without pathological fracture
Osteoporosis without pathological fracture
Osteoporosis without pathological fracture
Osteoporosis without pathological fracture
Osteoporosis without pathological fracture
Osteoporosis without pathological fracture
Osteoporosis without pathological fracture
Osteoporosis without pathological fracture
Osteoporosis without pathological fracture
Disorders of continuity of bone
Disorders of continuity of bone
Disorders of continuity of bone
Disorders of continuity of bone
Disorders of continuity of bone
Disorders of continuity of bone
Osteomyelitis
Osteomyelitis
Osteomyelitis
Osteomyelitis
Osteomyelitis
Osteomyelitis
Osteomyelitis
Osteomyelitis
Osteomyelitis
Osteomyelitis
Osteomyelitis
Osteomyelitis
Osteomyelitis
Osteomyelitis
Osteomyelitis
Osteomyelitis

For peer review only

Osteomyelitis
Osteonecrosis
Osteonecrosis
Osteonecrosis
Osteonecrosis
Paget's disease of bone [osteitis deformans]
Paget's disease of bone [osteitis deformans]
Paget's disease of bone [osteitis deformans]
Paget's disease of bone [osteitis deformans]
Other disorders of bone
Other disorders of bone
Other disorders of bone
Other disorders of bone
Other acquired deformities of musculoskeletal system
Acute nephritic syndrome
Acute nephritic syndrome
Acute nephritic syndrome
Acute nephritic syndrome
Acute nephritic syndrome
Rapidly progressive nephritic syndrome
Recurrent and persistent haematuria
Recurrent and persistent haematuria
Recurrent and persistent haematuria
Chronic nephritic syndrome
Chronic nephritic syndrome
Chronic nephritic syndrome
Chronic nephritic syndrome
Chronic nephritic syndrome
Chronic nephritic syndrome
Chronic nephritic syndrome
Chronic nephritic syndrome
Chronic nephritic syndrome
Chronic nephritic syndrome
Chronic nephritic syndrome
Chronic nephritic syndrome
Chronic nephritic syndrome
Chronic nephritic syndrome
Chronic nephritic syndrome
Chronic nephritic syndrome
Chronic nephritic syndrome
Chronic nephritic syndrome
Nephrotic syndrome
Nephrotic syndrome
Nephrotic syndrome
Nephrotic syndrome
Nephrotic syndrome
Nephrotic syndrome
Nephrotic syndrome
Nephrotic syndrome
Nephrotic syndrome

For peer review only

Nephrotic syndrome
Nephrotic syndrome
Nephrotic syndrome
Unspecified nephritic syndrome
Unspecified nephritic syndrome
Unspecified nephritic syndrome
Unspecified nephritic syndrome
Unspecified nephritic syndrome
Unspecified nephritic syndrome
Unspecified nephritic syndrome
Unspecified nephritic syndrome
Unspecified nephritic syndrome
Unspecified nephritic syndrome
Unspecified nephritic syndrome
Unspecified nephritic syndrome
Unspecified nephritic syndrome
Unspecified nephritic syndrome
Acute tubulo-interstitial nephritis
Acute tubulo-interstitial nephritis
Acute tubulo-interstitial nephritis
Acute tubulo-interstitial nephritis
Acute tubulo-interstitial nephritis
Acute tubulo-interstitial nephritis
Acute tubulo-interstitial nephritis
Acute tubulo-interstitial nephritis
Acute tubulo-interstitial nephritis
Acute tubulo-interstitial nephritis
Acute tubulo-interstitial nephritis
Acute tubulo-interstitial nephritis
Acute tubulo-interstitial nephritis
Acute tubulo-interstitial nephritis
Acute tubulo-interstitial nephritis
Acute tubulo-interstitial nephritis
Acute tubulo-interstitial nephritis
Acute tubulo-interstitial nephritis
Chronic tubulo-interstitial nephritis
Chronic tubulo-interstitial nephritis
Chronic tubulo-interstitial nephritis
Chronic tubulo-interstitial nephritis
Chronic tubulo-interstitial nephritis
Chronic tubulo-interstitial nephritis
Chronic tubulo-interstitial nephritis
Chronic tubulo-interstitial nephritis
Chronic tubulo-interstitial nephritis
Chronic tubulo-interstitial nephritis
Chronic tubulo-interstitial nephritis
Chronic tubulo-interstitial nephritis
Chronic tubulo-interstitial nephritis
Chronic tubulo-interstitial nephritis
Chronic tubulo-interstitial nephritis

For peer review only

Chronic tubulo-interstitial nephritis
Chronic tubulo-interstitial nephritis
Chronic tubulo-interstitial nephritis
Chronic tubulo-interstitial nephritis
Chronic tubulo-interstitial nephritis
Tubulo-interstitial nephritis, not specified as acute or chronic
Tubulo-interstitial nephritis, not specified as acute or chronic
Tubulo-interstitial nephritis, not specified as acute or chronic
Tubulo-interstitial nephritis, not specified as acute or chronic
Tubulo-interstitial nephritis, not specified as acute or chronic
Tubulo-interstitial nephritis, not specified as acute or chronic
Tubulo-interstitial nephritis, not specified as acute or chronic
Tubulo-interstitial nephritis, not specified as acute or chronic
Tubulo-interstitial nephritis, not specified as acute or chronic
Tubulo-interstitial nephritis, not specified as acute or chronic
Tubulo-interstitial nephritis, not specified as acute or chronic
Tubulo-interstitial nephritis, not specified as acute or chronic
Tubulo-interstitial nephritis, not specified as acute or chronic
Tubulo-interstitial nephritis, not specified as acute or chronic
Tubulo-interstitial nephritis, not specified as acute or chronic
Tubulo-interstitial nephritis, not specified as acute or chronic
Tubulo-interstitial nephritis, not specified as acute or chronic
Obstructive and reflux uropathy
Obstructive and reflux uropathy
Obstructive and reflux uropathy
Obstructive and reflux uropathy
Obstructive and reflux uropathy
Obstructive and reflux uropathy
Obstructive and reflux uropathy
Obstructive and reflux uropathy
Obstructive and reflux uropathy
Obstructive and reflux uropathy
Obstructive and reflux uropathy
Obstructive and reflux uropathy
Obstructive and reflux uropathy
Obstructive and reflux uropathy
Obstructive and reflux uropathy
Obstructive and reflux uropathy
Obstructive and reflux uropathy
Obstructive and reflux uropathy
Obstructive and reflux uropathy
Drug- and heavy-metal-induced tubulo-interstitial and tubular conditions
Drug- and heavy-metal-induced tubulo-interstitial and tubular conditions
Drug- and heavy-metal-induced tubulo-interstitial and tubular conditions
Drug- and heavy-metal-induced tubulo-interstitial and tubular conditions
Drug- and heavy-metal-induced tubulo-interstitial and tubular conditions
Drug- and heavy-metal-induced tubulo-interstitial and tubular conditions
Other renal tubulo-interstitial diseases
Other renal tubulo-interstitial diseases
Other renal tubulo-interstitial diseases

For peer review only

Other renal tubulo-interstitial diseases
Other renal tubulo-interstitial diseases
Other renal tubulo-interstitial diseases
Other renal tubulo-interstitial diseases
Other renal tubulo-interstitial diseases
Other renal tubulo-interstitial diseases
Other renal tubulo-interstitial diseases
Other renal tubulo-interstitial diseases
Acute renal failure
Acute renal failure
Acute renal failure
Acute renal failure
Acute renal failure
Acute renal failure
Acute renal failure
Acute renal failure
Acute renal failure
Acute renal failure
Acute renal failure
Acute renal failure
Acute renal failure
Acute renal failure
Acute renal failure
Acute renal failure
Acute renal failure
Acute renal failure
Chronic renal failure
Chronic renal failure
Chronic renal failure
Chronic renal failure
Chronic renal failure
Chronic renal failure
Chronic renal failure
Chronic renal failure
Chronic renal failure
Chronic renal failure
Chronic renal failure
Chronic renal failure
Chronic renal failure
Chronic renal failure
Chronic renal failure
Chronic renal failure
Chronic renal failure
Chronic renal failure
Chronic renal failure
Chronic renal failure
Chronic renal failure
Unspecified renal failure
Unspecified renal failure
Unspecified renal failure

For peer review only

Unspecified renal failure
Unspecified renal failure
Unspecified renal failure
Unspecified renal failure
Unspecified renal failure
Unspecified renal failure
Unspecified renal failure
Unspecified renal failure
Unspecified renal failure
Unspecified renal failure
Unspecified renal failure
Unspecified renal failure
Unspecified renal failure
Unspecified renal failure
Unspecified renal failure
Unspecified renal failure
Calculus of kidney and ureter
Calculus of kidney and ureter
Calculus of kidney and ureter
Calculus of kidney and ureter
Calculus of kidney and ureter
Calculus of kidney and ureter
Calculus of kidney and ureter
Calculus of kidney and ureter
Calculus of kidney and ureter
Calculus of kidney and ureter
Calculus of kidney and ureter
Calculus of kidney and ureter
Calculus of kidney and ureter
Calculus of kidney and ureter
Calculus of kidney and ureter
Calculus of kidney and ureter
Calculus of kidney and ureter
Calculus of kidney and ureter
Calculus of kidney and ureter
Calculus of lower urinary tract
Calculus of lower urinary tract
Calculus of lower urinary tract
Unspecified renal colic
Disorders resulting from impaired renal tubular function
Unspecified contracted kidney
Unspecified contracted kidney
Unspecified contracted kidney
Unspecified contracted kidney
Unspecified contracted kidney
Unspecified contracted kidney
Unspecified contracted kidney
Unspecified contracted kidney
Unspecified contracted kidney
Unspecified contracted kidney

For peer review only

Unspecified contracted kidney
Unspecified contracted kidney
Unspecified contracted kidney
Small kidney of unknown cause
Other disorders of kidney and ureter, not elsewhere classified
Other disorders of kidney and ureter, not elsewhere classified
Other disorders of kidney and ureter, not elsewhere classified
Other disorders of kidney and ureter, not elsewhere classified
Other disorders of kidney and ureter, not elsewhere classified
Other disorders of kidney and ureter, not elsewhere classified
Other disorders of kidney and ureter, not elsewhere classified
Other disorders of kidney and ureter, not elsewhere classified
Other disorders of kidney and ureter, not elsewhere classified
Other disorders of kidney and ureter, not elsewhere classified
Other disorders of kidney and ureter, not elsewhere classified
Other disorders of kidney and ureter, not elsewhere classified
Other disorders of kidney and ureter, not elsewhere classified
Other disorders of kidney and ureter, not elsewhere classified
Other disorders of kidney and ureter, not elsewhere classified
Other disorders of kidney and ureter, not elsewhere classified
Other disorders of kidney and ureter, not elsewhere classified
Other disorders of kidney and ureter, not elsewhere classified
Cystitis
Cystitis
Cystitis
Cystitis
Cystitis
Cystitis
Cystitis
Cystitis
Cystitis
Cystitis
Cystitis
Cystitis
Cystitis
Cystitis
Cystitis
Cystitis
Cystitis
Neuromuscular dysfunction of bladder, not elsewhere classified
Neuromuscular dysfunction of bladder, not elsewhere classified
Other disorders of bladder
Other disorders of bladder
Other disorders of bladder
Other disorders of bladder
Other disorders of bladder
Other disorders of bladder
Other disorders of bladder
Other disorders of bladder
Other disorders of bladder

For peer review only

Other disorders of bladder
Other disorders of bladder
Urethritis and urethral syndrome
Urethral stricture
Urethral stricture
Other disorders of urethra
Other disorders of urinary system
Other disorders of urinary system
Other disorders of urinary system
Other disorders of urinary system
Other disorders of urinary system
Other disorders of urinary system
Other disorders of urinary system
Other disorders of urinary system
Other disorders of urinary system
Other disorders of urinary system
Other disorders of urinary system
Other disorders of urinary system
Other disorders of urinary system
Other disorders of urinary system
Other disorders of urinary system
Other disorders of urinary system
Other disorders of urinary system
Other disorders of urinary system
Other disorders of urinary system
Other disorders of urinary system
Hyperplasia of prostate
Hyperplasia of prostate
Hyperplasia of prostate
Hyperplasia of prostate
Hyperplasia of prostate
Hyperplasia of prostate
Hyperplasia of prostate
Hyperplasia of prostate
Hyperplasia of prostate
Hyperplasia of prostate
Hyperplasia of prostate
Hyperplasia of prostate
Hyperplasia of prostate
Hyperplasia of prostate
Hyperplasia of prostate
Hyperplasia of prostate
Hyperplasia of prostate
Hyperplasia of prostate
Hyperplasia of prostate
Hyperplasia of prostate
Inflammatory diseases of prostate
Inflammatory diseases of prostate
Inflammatory diseases of prostate
Inflammatory diseases of prostate

For peer review only

Inflammatory diseases of prostate
Inflammatory diseases of prostate
Inflammatory diseases of prostate
Other disorders of prostate
Other disorders of prostate
Other disorders of prostate
Other disorders of prostate
Other disorders of prostate
Other disorders of prostate
Other disorders of prostate
Orchitis and epididymitis
Orchitis and epididymitis
Orchitis and epididymitis
Inflammatory disorders of male genital organs, not elsewhere classified
Inflammatory disorders of male genital organs, not elsewhere classified
Inflammatory disorders of male genital organs, not elsewhere classified
Inflammatory disorders of male genital organs, not elsewhere classified
Inflammatory disorders of male genital organs, not elsewhere classified
Inflammatory disorders of male genital organs, not elsewhere classified
Inflammatory disorders of male genital organs, not elsewhere classified
Salpingitis and oophoritis
Salpingitis and oophoritis
Salpingitis and oophoritis
Inflammatory disease of uterus, except cervix
Inflammatory disease of uterus, except cervix
Inflammatory disease of uterus, except cervix
Inflammatory disease of uterus, except cervix
Other female pelvic inflammatory diseases
Other female pelvic inflammatory diseases
Other female pelvic inflammatory diseases
Other inflammation of vagina and vulva
Other inflammation of vagina and vulva
Female genital prolapse
Female genital prolapse
Female genital prolapse
Female genital prolapse
Fistulae involving female genital tract
Fistulae involving female genital tract
Fistulae involving female genital tract
Fistulae involving female genital tract
Fistulae involving female genital tract
Noninflammatory disorders of ovary, fallopian tube and broad ligament
Noninflammatory disorders of ovary, fallopian tube and broad ligament
Other noninflammatory disorders of uterus, except cervix
Other noninflammatory disorders of uterus, except cervix
Other abnormal uterine and vaginal bleeding
Other abnormal uterine and vaginal bleeding
Menopausal and other perimenopausal disorders
Menopausal and other perimenopausal disorders
Unspecified abortion

For peer review only

Postpartum haemorrhage
Obstetric embolism
Obstetric embolism
Other maternal diseases classifiable elsewhere but complicating pregnancy, childbirth and the puerperium
Other maternal diseases classifiable elsewhere but complicating pregnancy, childbirth and the puerperium
Fetus and newborn affected by maternal conditions that may be unrelated to present pregnancy
Fetus and newborn affected by maternal conditions that may be unrelated to present pregnancy
Fetus and newborn affected by maternal conditions that may be unrelated to present pregnancy
Fetus and newborn affected by maternal conditions that may be unrelated to present pregnancy
Fetus and newborn affected by maternal conditions that may be unrelated to present pregnancy
Fetus and newborn affected by maternal complications of pregnancy
Fetus and newborn affected by maternal complications of pregnancy
Fetus and newborn affected by maternal complications of pregnancy
Fetus and newborn affected by maternal complications of pregnancy
Fetus and newborn affected by maternal complications of pregnancy
Fetus and newborn affected by maternal complications of pregnancy
Fetus and newborn affected by maternal complications of pregnancy
Fetus and newborn affected by maternal complications of pregnancy
Fetus and newborn affected by maternal complications of pregnancy
Fetus and newborn affected by maternal complications of pregnancy
Fetus and newborn affected by maternal complications of pregnancy
Fetus and newborn affected by maternal complications of pregnancy
Fetus and newborn affected by complications of placenta, cord and membranes
Fetus and newborn affected by complications of placenta, cord and membranes
Fetus and newborn affected by complications of placenta, cord and membranes
Fetus and newborn affected by complications of placenta, cord and membranes
Fetus and newborn affected by complications of placenta, cord and membranes
Fetus and newborn affected by complications of placenta, cord and membranes
Fetus and newborn affected by complications of placenta, cord and membranes
Fetus and newborn affected by complications of placenta, cord and membranes
Fetus and newborn affected by complications of placenta, cord and membranes
Fetus and newborn affected by complications of placenta, cord and membranes
Fetus and newborn affected by complications of placenta, cord and membranes
Fetus and newborn affected by complications of placenta, cord and membranes
Fetus and newborn affected by complications of placenta, cord and membranes
Fetus and newborn affected by complications of placenta, cord and membranes
Fetus and newborn affected by other complications of labour and delivery
Fetus and newborn affected by other complications of labour and delivery
Fetus and newborn affected by other complications of labour and delivery
Slow fetal growth and fetal malnutrition
Slow fetal growth and fetal malnutrition
Slow fetal growth and fetal malnutrition
Slow fetal growth and fetal malnutrition
Disorders related to short gestation and low birth weight, not elsewhere classified
Disorders related to short gestation and low birth weight, not elsewhere classified
Disorders related to short gestation and low birth weight, not elsewhere classified
Disorders related to short gestation and low birth weight, not elsewhere classified
Disorders related to short gestation and low birth weight, not elsewhere classified
Disorders related to short gestation and low birth weight, not elsewhere classified
Disorders related to short gestation and low birth weight, not elsewhere classified

For peer review only

Disorders related to short gestation and low birth weight, not elsewhere classified
Disorders related to short gestation and low birth weight, not elsewhere classified
Disorders related to short gestation and low birth weight, not elsewhere classified
Disorders related to short gestation and low birth weight, not elsewhere classified
Disorders related to short gestation and low birth weight, not elsewhere classified
Disorders related to short gestation and low birth weight, not elsewhere classified
Disorders related to short gestation and low birth weight, not elsewhere classified
Disorders related to short gestation and low birth weight, not elsewhere classified
Disorders related to short gestation and low birth weight, not elsewhere classified
Disorders related to short gestation and low birth weight, not elsewhere classified
Disorders related to short gestation and low birth weight, not elsewhere classified
Intracranial laceration and haemorrhage due to birth injury
Intracranial laceration and haemorrhage due to birth injury
Intrauterine hypoxia
Intrauterine hypoxia
Intrauterine hypoxia
Intrauterine hypoxia
Intrauterine hypoxia
Intrauterine hypoxia
Intrauterine hypoxia
Intrauterine hypoxia
Birth asphyxia
Birth asphyxia
Birth asphyxia
Birth asphyxia
Birth asphyxia
Birth asphyxia
Birth asphyxia
Birth asphyxia
Birth asphyxia
Birth asphyxia
Birth asphyxia
Birth asphyxia
Birth asphyxia
Birth asphyxia
Birth asphyxia
Birth asphyxia
Birth asphyxia
Birth asphyxia
Respiratory distress of newborn
Respiratory distress of newborn
Respiratory distress of newborn
Respiratory distress of newborn
Respiratory distress of newborn
Respiratory distress of newborn
Respiratory distress of newborn
Respiratory distress of newborn
Respiratory distress of newborn
Respiratory distress of newborn
Respiratory distress of newborn

For peer review only

Respiratory distress of newborn
Respiratory distress of newborn
Respiratory distress of newborn
Respiratory distress of newborn
Respiratory distress of newborn
Respiratory distress of newborn
Congenital pneumonia
Congenital pneumonia
Congenital pneumonia
Congenital pneumonia
Congenital pneumonia
Congenital pneumonia
Neonatal aspiration syndromes
Neonatal aspiration syndromes
Neonatal aspiration syndromes
Neonatal aspiration syndromes
Neonatal aspiration syndromes
Neonatal aspiration syndromes
Interstitial emphysema and related conditions originating in the perinatal period
Interstitial emphysema and related conditions originating in the perinatal period
Interstitial emphysema and related conditions originating in the perinatal period
Interstitial emphysema and related conditions originating in the perinatal period
Pulmonary haemorrhage originating in the perinatal period
Pulmonary haemorrhage originating in the perinatal period
Pulmonary haemorrhage originating in the perinatal period
Pulmonary haemorrhage originating in the perinatal period
Pulmonary haemorrhage originating in the perinatal period
Pulmonary haemorrhage originating in the perinatal period
Chronic respiratory disease originating in the perinatal period
Chronic respiratory disease originating in the perinatal period
Chronic respiratory disease originating in the perinatal period
Chronic respiratory disease originating in the perinatal period
Chronic respiratory disease originating in the perinatal period
Chronic respiratory disease originating in the perinatal period
Chronic respiratory disease originating in the perinatal period
Other respiratory conditions originating in the perinatal period
Other respiratory conditions originating in the perinatal period
Other respiratory conditions originating in the perinatal period
Other respiratory conditions originating in the perinatal period
Other respiratory conditions originating in the perinatal period
Other respiratory conditions originating in the perinatal period
Other respiratory conditions originating in the perinatal period
Other respiratory conditions originating in the perinatal period
Other respiratory conditions originating in the perinatal period
Other respiratory conditions originating in the perinatal period
Other respiratory conditions originating in the perinatal period
Other respiratory conditions originating in the perinatal period
Cardiovascular disorders originating in the perinatal period
Cardiovascular disorders originating in the perinatal period
Cardiovascular disorders originating in the perinatal period

For peer review only

Cardiovascular disorders originating in the perinatal period
Cardiovascular disorders originating in the perinatal period
Cardiovascular disorders originating in the perinatal period
Cardiovascular disorders originating in the perinatal period
Cardiovascular disorders originating in the perinatal period
Congenital viral diseases
Congenital viral diseases
Bacterial sepsis of newborn
Bacterial sepsis of newborn
Bacterial sepsis of newborn
Bacterial sepsis of newborn
Bacterial sepsis of newborn
Bacterial sepsis of newborn
Bacterial sepsis of newborn
Bacterial sepsis of newborn
Bacterial sepsis of newborn
Bacterial sepsis of newborn
Bacterial sepsis of newborn
Bacterial sepsis of newborn
Bacterial sepsis of newborn
Bacterial sepsis of newborn
Other congenital infectious and parasitic diseases
Other congenital infectious and parasitic diseases
Other congenital infectious and parasitic diseases
Other infections specific to the perinatal period
Other infections specific to the perinatal period
Other infections specific to the perinatal period
Other infections specific to the perinatal period
Other infections specific to the perinatal period
Other infections specific to the perinatal period
Intracranial nontraumatic haemorrhage of fetus and newborn
Intracranial nontraumatic haemorrhage of fetus and newborn
Intracranial nontraumatic haemorrhage of fetus and newborn
Intracranial nontraumatic haemorrhage of fetus and newborn
Intracranial nontraumatic haemorrhage of fetus and newborn
Intracranial nontraumatic haemorrhage of fetus and newborn
Intracranial nontraumatic haemorrhage of fetus and newborn
Intracranial nontraumatic haemorrhage of fetus and newborn
Intracranial nontraumatic haemorrhage of fetus and newborn
Intracranial nontraumatic haemorrhage of fetus and newborn
Intracranial nontraumatic haemorrhage of fetus and newborn
Intracranial nontraumatic haemorrhage of fetus and newborn
Intracranial nontraumatic haemorrhage of fetus and newborn
Intracranial nontraumatic haemorrhage of fetus and newborn
Intracranial nontraumatic haemorrhage of fetus and newborn
Haemorrhagic disease of fetus and newborn
Other neonatal haemorrhages
Haemolytic disease of fetus and newborn
Disseminated intravascular coagulation of fetus and newborn
Disseminated intravascular coagulation of fetus and newborn

For peer review only

Other perinatal haematological disorders
Other intestinal obstruction of newborn
Necrotizing enterocolitis of fetus and newborn
Necrotizing enterocolitis of fetus and newborn
Necrotizing enterocolitis of fetus and newborn
Necrotizing enterocolitis of fetus and newborn
Necrotizing enterocolitis of fetus and newborn
Necrotizing enterocolitis of fetus and newborn
Necrotizing enterocolitis of fetus and newborn
Necrotizing enterocolitis of fetus and newborn
Necrotizing enterocolitis of fetus and newborn
Necrotizing enterocolitis of fetus and newborn
Necrotizing enterocolitis of fetus and newborn
Necrotizing enterocolitis of fetus and newborn
Other perinatal digestive system disorders
Other perinatal digestive system disorders
Other perinatal digestive system disorders
Other perinatal digestive system disorders
Other conditions of integument specific to fetus and newborn
Other conditions of integument specific to fetus and newborn
Other conditions of integument specific to fetus and newborn
Other conditions of integument specific to fetus and newborn
Other conditions of integument specific to fetus and newborn
Other disturbances of cerebral status of newborn
Other disturbances of cerebral status of newborn
Other disturbances of cerebral status of newborn
Other disturbances of cerebral status of newborn
Other disturbances of cerebral status of newborn
Other conditions originating in the perinatal period
Other conditions originating in the perinatal period
Other conditions originating in the perinatal period
Other conditions originating in the perinatal period
Other conditions originating in the perinatal period
Anencephaly and similar malformations
Anencephaly and similar malformations
Anencephaly and similar malformations
Anencephaly and similar malformations
Anencephaly and similar malformations
Anencephaly and similar malformations
Encephalocele
Microcephaly
Microcephaly
Microcephaly
Congenital hydrocephalus
Congenital hydrocephalus
Congenital hydrocephalus
Congenital hydrocephalus
Congenital hydrocephalus
Congenital hydrocephalus
Congenital hydrocephalus

For peer review only

Congenital hydrocephalus
Congenital hydrocephalus
Congenital hydrocephalus
Other congenital malformations of brain
Other congenital malformations of brain
Other congenital malformations of brain
Other congenital malformations of brain
Other congenital malformations of brain
Other congenital malformations of brain
Other congenital malformations of brain
Other congenital malformations of brain
Other congenital malformations of brain
Other congenital malformations of brain
Other congenital malformations of brain
Other congenital malformations of brain
Other congenital malformations of brain
Other congenital malformations of brain
Spina bifida
Spina bifida
Spina bifida
Spina bifida
Spina bifida
Spina bifida
Spina bifida
Other congenital malformations of nervous system
Other congenital malformations of nervous system
Other congenital malformations of nervous system
Other congenital malformations of nervous system
Other congenital malformations of nervous system
Other congenital malformations of nervous system
Congenital malformations of cardiac chambers and connections
Congenital malformations of cardiac chambers and connections
Congenital malformations of cardiac chambers and connections
Congenital malformations of cardiac chambers and connections
Congenital malformations of cardiac chambers and connections
Congenital malformations of cardiac chambers and connections
Congenital malformations of cardiac chambers and connections
Congenital malformations of cardiac chambers and connections
Congenital malformations of cardiac chambers and connections
Congenital malformations of cardiac chambers and connections
Congenital malformations of cardiac chambers and connections
Congenital malformations of cardiac chambers and connections
Congenital malformations of cardiac chambers and connections
Congenital malformations of cardiac chambers and connections
Congenital malformations of cardiac septa
Congenital malformations of cardiac septa
Congenital malformations of cardiac septa
Congenital malformations of cardiac septa
Congenital malformations of cardiac septa
Congenital malformations of cardiac septa

For peer review only

Congenital malformations of cardiac septa
Congenital malformations of cardiac septa
Congenital malformations of cardiac septa
Congenital malformations of cardiac septa
Congenital malformations of cardiac septa
Congenital malformations of cardiac septa
Congenital malformations of cardiac septa
Congenital malformations of cardiac septa
Congenital malformations of cardiac septa
Congenital malformations of cardiac septa
Congenital malformations of cardiac septa
Congenital malformations of cardiac septa
Congenital malformations of cardiac septa
Congenital malformations of cardiac septa
Congenital malformations of pulmonary and tricuspid valves
Congenital malformations of pulmonary and tricuspid valves
Congenital malformations of pulmonary and tricuspid valves
Congenital malformations of pulmonary and tricuspid valves
Congenital malformations of pulmonary and tricuspid valves
Congenital malformations of pulmonary and tricuspid valves
Congenital malformations of aortic and mitral valves
Congenital malformations of aortic and mitral valves
Congenital malformations of aortic and mitral valves
Congenital malformations of aortic and mitral valves
Congenital malformations of aortic and mitral valves
Congenital malformations of aortic and mitral valves
Congenital malformations of aortic and mitral valves
Congenital malformations of aortic and mitral valves
Congenital malformations of aortic and mitral valves
Congenital malformations of aortic and mitral valves
Congenital malformations of aortic and mitral valves
Congenital malformations of aortic and mitral valves
Congenital malformations of aortic and mitral valves
Congenital malformations of aortic and mitral valves
Congenital malformations of aortic and mitral valves
Congenital malformations of aortic and mitral valves
Congenital malformations of aortic and mitral valves
Congenital malformations of aortic and mitral valves
Other congenital malformations of the heart
Other congenital malformations of the heart
Other congenital malformations of the heart
Other congenital malformations of the heart
Other congenital malformations of the heart
Other congenital malformations of the heart
Other congenital malformations of the heart
Other congenital malformations of the heart
Other congenital malformations of the heart
Other congenital malformations of the heart
Other congenital malformations of the heart
Other congenital malformations of the heart

For peer review only

Other congenital malformations of the heart
Other congenital malformations of the heart
Other congenital malformations of the heart
Other congenital malformations of the heart
Other congenital malformations of the heart
Other congenital malformations of the heart
Congenital malformations of great arteries
Congenital malformations of great arteries
Congenital malformations of great arteries
Congenital malformations of great arteries
Congenital malformations of great arteries
Congenital malformations of great arteries
Congenital malformations of great arteries
Congenital malformations of great arteries
Congenital malformations of great arteries
Congenital malformations of great arteries
Congenital malformations of great arteries
Congenital malformations of great arteries
Congenital malformations of great arteries
Congenital malformations of great arteries
Congenital malformations of great veins
Congenital malformations of great veins
Congenital malformations of great veins
Congenital malformations of great veins
Other congenital malformations of peripheral vascular system
Other congenital malformations of peripheral vascular system
Other congenital malformations of peripheral vascular system
Other congenital malformations of circulatory system
Other congenital malformations of circulatory system
Other congenital malformations of circulatory system
Other congenital malformations of circulatory system
Other congenital malformations of circulatory system
Other congenital malformations of circulatory system
Other congenital malformations of circulatory system
Other congenital malformations of circulatory system
Other congenital malformations of circulatory system
Congenital malformations of trachea and bronchus
Congenital malformations of trachea and bronchus
Congenital malformations of trachea and bronchus
Congenital malformations of lung
Congenital malformations of lung
Congenital malformations of lung
Congenital malformations of lung
Congenital malformations of lung
Congenital malformations of lung
Congenital malformations of lung
Congenital malformations of lung
Congenital malformations of lung
Congenital malformations of lung
Other congenital malformations of tongue, mouth and pharynx

For peer review only

Congenital malformations of oesophagus
Congenital malformations of oesophagus
Congenital malformations of oesophagus
Congenital malformations of oesophagus
Congenital malformations of oesophagus
Congenital malformations of oesophagus
Congenital absence, atresia and stenosis of small intestine
Congenital absence, atresia and stenosis of small intestine
Other congenital malformations of intestine
Other congenital malformations of intestine
Other congenital malformations of intestine
Other congenital malformations of intestine
Other congenital malformations of intestine
Other congenital malformations of intestine
Other congenital malformations of intestine
Other congenital malformations of intestine
Congenital malformations of gallbladder, bile ducts and liver
Congenital malformations of gallbladder, bile ducts and liver
Congenital malformations of gallbladder, bile ducts and liver
Congenital malformations of gallbladder, bile ducts and liver
Congenital malformations of gallbladder, bile ducts and liver
Renal agenesis and other reduction defects of kidney
Renal agenesis and other reduction defects of kidney
Renal agenesis and other reduction defects of kidney
Renal agenesis and other reduction defects of kidney
Renal agenesis and other reduction defects of kidney
Renal agenesis and other reduction defects of kidney
Cystic kidney disease
Cystic kidney disease
Cystic kidney disease
Cystic kidney disease
Cystic kidney disease
Cystic kidney disease
Cystic kidney disease
Cystic kidney disease
Cystic kidney disease
Cystic kidney disease
Cystic kidney disease
Cystic kidney disease
Cystic kidney disease
Cystic kidney disease
Cystic kidney disease
Cystic kidney disease
Cystic kidney disease
Other congenital malformations of kidney
Other congenital malformations of kidney
Congenital musculoskeletal deformities of head, face, spine and chest
Congenital malformations of spine and bony thorax
Osteochondrodysplasia with defects of growth of tubular bones and spine
Osteochondrodysplasia with defects of growth of tubular bones and spine

For peer review only

Osteochondrodysplasia with defects of growth of tubular bones and spine
Other osteochondrodysplasias
Other osteochondrodysplasias
Other osteochondrodysplasias
Other osteochondrodysplasias
Congenital malformations of the musculoskeletal system, not elsewhere classified
Congenital malformations of the musculoskeletal system, not elsewhere classified
Congenital malformations of the musculoskeletal system, not elsewhere classified
Congenital malformations of the musculoskeletal system, not elsewhere classified
Congenital malformations of the musculoskeletal system, not elsewhere classified
Congenital malformations of the musculoskeletal system, not elsewhere classified
Congenital malformations of the musculoskeletal system, not elsewhere classified
Congenital malformations of the musculoskeletal system, not elsewhere classified
Congenital malformations of the musculoskeletal system, not elsewhere classified
Congenital malformations of the musculoskeletal system, not elsewhere classified
Congenital malformations of the musculoskeletal system, not elsewhere classified
Congenital malformations of the musculoskeletal system, not elsewhere classified
Congenital malformations of the musculoskeletal system, not elsewhere classified
Epidermolysis bullosa
Epidermolysis bullosa
Other congenital malformations of skin
Phakomatoses, not elsewhere classified
Phakomatoses, not elsewhere classified
Phakomatoses, not elsewhere classified
Phakomatoses, not elsewhere classified
Phakomatoses, not elsewhere classified
Phakomatoses, not elsewhere classified
Phakomatoses, not elsewhere classified
Phakomatoses, not elsewhere classified
Phakomatoses, not elsewhere classified
Other specified congenital malformation syndromes affecting multiple systems
Other specified congenital malformation syndromes affecting multiple systems
Other specified congenital malformation syndromes affecting multiple systems
Other specified congenital malformation syndromes affecting multiple systems
Other specified congenital malformation syndromes affecting multiple systems
Other specified congenital malformation syndromes affecting multiple systems
Other specified congenital malformation syndromes affecting multiple systems
Other specified congenital malformation syndromes affecting multiple systems
Other specified congenital malformation syndromes affecting multiple systems
Other specified congenital malformation syndromes affecting multiple systems
Other specified congenital malformation syndromes affecting multiple systems
Other specified congenital malformation syndromes affecting multiple systems
Other specified congenital malformation syndromes affecting multiple systems
Other specified congenital malformation syndromes affecting multiple systems
Other specified congenital malformation syndromes affecting multiple systems
Other congenital malformations, not elsewhere classified
Other congenital malformations, not elsewhere classified
Other congenital malformations, not elsewhere classified
Other congenital malformations, not elsewhere classified
Other congenital malformations, not elsewhere classified

For peer review only

Other congenital malformations, not elsewhere classified
Other congenital malformations, not elsewhere classified
Other congenital malformations, not elsewhere classified
Other congenital malformations, not elsewhere classified
Other congenital malformations, not elsewhere classified
Other congenital malformations, not elsewhere classified
Other congenital malformations, not elsewhere classified
Other congenital malformations, not elsewhere classified
Other congenital malformations, not elsewhere classified
Other congenital malformations, not elsewhere classified
Other congenital malformations, not elsewhere classified
Down's syndrome
Down's syndrome
Down's syndrome
Down's syndrome
Down's syndrome
Down's syndrome
Down's syndrome
Down's syndrome
Down's syndrome
Down's syndrome
Down's syndrome
Down's syndrome
Down's syndrome
Down's syndrome
Down's syndrome
Down's syndrome
Down's syndrome
Down's syndrome
Down's syndrome
Down's syndrome
Edwards' syndrome and Patau's syndrome
Edwards' syndrome and Patau's syndrome
Edwards' syndrome and Patau's syndrome
Edwards' syndrome and Patau's syndrome
Edwards' syndrome and Patau's syndrome
Edwards' syndrome and Patau's syndrome
Edwards' syndrome and Patau's syndrome
Edwards' syndrome and Patau's syndrome
Edwards' syndrome and Patau's syndrome
Edwards' syndrome and Patau's syndrome
Edwards' syndrome and Patau's syndrome
Edwards' syndrome and Patau's syndrome
Edwards' syndrome and Patau's syndrome
Edwards' syndrome and Patau's syndrome
Edwards' syndrome and Patau's syndrome
Edwards' syndrome and Patau's syndrome
Other trisomies and partial trisomies of the autosomes, not elsewhere classified
Other trisomies and partial trisomies of the autosomes, not elsewhere classified
Monosomies and deletions from the autosomes, not elsewhere classified

For peer review only

Monosomies and deletions from the autosomes, not elsewhere classified
Monosomies and deletions from the autosomes, not elsewhere classified
Other chromosome abnormalities, not elsewhere classified
Other chromosome abnormalities, not elsewhere classified
Other chromosome abnormalities, not elsewhere classified
Other chromosome abnormalities, not elsewhere classified
Other chromosome abnormalities, not elsewhere classified
Other chromosome abnormalities, not elsewhere classified
Abnormalities of heart beat
Abnormalities of heart beat
Abnormalities of heart beat
Abnormalities of heart beat
Abnormalities of heart beat
Abnormalities of heart beat
Gangrene, not elsewhere classified
Gangrene, not elsewhere classified
Gangrene, not elsewhere classified
Gangrene, not elsewhere classified
Gangrene, not elsewhere classified
Gangrene, not elsewhere classified
Gangrene, not elsewhere classified
Haemorrhage from respiratory passages
Haemorrhage from respiratory passages
Haemorrhage from respiratory passages
Haemorrhage from respiratory passages
Haemorrhage from respiratory passages
Haemorrhage from respiratory passages
Haemorrhage from respiratory passages
Abnormalities of breathing
Abnormalities of breathing
Abnormalities of breathing
Abnormalities of breathing
Abnormalities of breathing
Abnormalities of breathing
Pain in throat and chest
Other symptoms and signs involving the circulatory and respiratory systems
Other symptoms and signs involving the circulatory and respiratory systems
Other symptoms and signs involving the circulatory and respiratory systems
Other symptoms and signs involving the circulatory and respiratory systems
Other symptoms and signs involving the circulatory and respiratory systems
Other symptoms and signs involving the circulatory and respiratory systems
Other symptoms and signs involving the circulatory and respiratory systems
Other symptoms and signs involving the circulatory and respiratory systems
Other symptoms and signs involving the circulatory and respiratory systems
Other symptoms and signs involving the circulatory and respiratory systems
Other symptoms and signs involving the circulatory and respiratory systems
Other symptoms and signs involving the circulatory and respiratory systems
Other symptoms and signs involving the circulatory and respiratory systems
Other symptoms and signs involving the circulatory and respiratory systems
Abdominal and pelvic pain

For peer review only

Abdominal and pelvic pain
Abdominal and pelvic pain
Abdominal and pelvic pain
Abdominal and pelvic pain
Abdominal and pelvic pain
Abdominal and pelvic pain
Abdominal and pelvic pain
Abdominal and pelvic pain
Nausea and vomiting
Nausea and vomiting
Nausea and vomiting
Dysphagia
Dysphagia
Dysphagia
Dysphagia
Unspecified jaundice
Unspecified jaundice
Unspecified jaundice
Ascites
Ascites
Other symptoms and signs involving the digestive system and abdomen
Other symptoms and signs involving the digestive system and abdomen
Other symptoms and signs involving the digestive system and abdomen
Other symptoms and signs involving the digestive system and abdomen
Other symptoms and signs involving the digestive system and abdomen
Other skin changes
Abnormalities of gait and mobility
Unspecified haematuria
Unspecified haematuria
Unspecified haematuria
Retention of urine
Anuria and oliguria
Somnolence, stupor and coma
Somnolence, stupor and coma
Somnolence, stupor and coma
Somnolence, stupor and coma
Somnolence, stupor and coma
Somnolence, stupor and coma
Other symptoms and signs involving cognitive functions and awareness
Other symptoms and signs involving cognitive functions and awareness
Fever of unknown origin
Fever of unknown origin
Fever of unknown origin
Fever of unknown origin
Fever of unknown origin
Fever of unknown origin
Headache
Pain, not elsewhere classified
Pain, not elsewhere classified
Pain, not elsewhere classified

For peer review only

Malaise and fatigue
Malaise and fatigue
Malaise and fatigue
Malaise and fatigue
Malaise and fatigue
Malaise and fatigue
Malaise and fatigue
Malaise and fatigue
Malaise and fatigue
Senility
Senility
Senility
Senility
Senility
Senility
Senility
Senility
Senility
Senility
Senility
Senility
Senility
Senility
Senility
Senility
Senility
Senility
Senility
Senility
Syncope and collapse
Syncope and collapse
Syncope and collapse
Syncope and collapse
Syncope and collapse
Convulsions, not elsewhere classified
Convulsions, not elsewhere classified
Convulsions, not elsewhere classified
Convulsions, not elsewhere classified
Shock, not elsewhere classified
Shock, not elsewhere classified
Shock, not elsewhere classified
Shock, not elsewhere classified
Shock, not elsewhere classified
Shock, not elsewhere classified
Shock, not elsewhere classified
Shock, not elsewhere classified
Shock, not elsewhere classified
Shock, not elsewhere classified
Shock, not elsewhere classified
Shock, not elsewhere classified

For peer review only

Haemorrhage, not elsewhere classified
Haemorrhage, not elsewhere classified
Haemorrhage, not elsewhere classified
Haemorrhage, not elsewhere classified
Haemorrhage, not elsewhere classified
Haemorrhage, not elsewhere classified
Haemorrhage, not elsewhere classified
Oedema, not elsewhere classified
Oedema, not elsewhere classified
Oedema, not elsewhere classified
Symptoms and signs concerning food and fluid intake
Symptoms and signs concerning food and fluid intake
Symptoms and signs concerning food and fluid intake
Symptoms and signs concerning food and fluid intake
Symptoms and signs concerning food and fluid intake
Symptoms and signs concerning food and fluid intake
Symptoms and signs concerning food and fluid intake
Cachexia
Cachexia
Cachexia
Cachexia
Cachexia
Cachexia
Cachexia
Cachexia
Cachexia
Cachexia
Cachexia
Cachexia
Cachexia
Cachexia
Other general symptoms and signs
Other general symptoms and signs
Other general symptoms and signs
Other general symptoms and signs
Other general symptoms and signs
Other general symptoms and signs
Other general symptoms and signs
Other general symptoms and signs
Elevated blood glucose level
Elevated blood glucose level
Abnormal findings on diagnostic imaging of lung
Sudden infant death syndrome
Sudden infant death syndrome
Sudden infant death syndrome
Sudden infant death syndrome
Sudden infant death syndrome
Sudden infant death syndrome
Sudden infant death syndrome
Sudden infant death syndrome

For peer review only

Sudden infant death syndrome
Sudden infant death syndrome
Sudden infant death syndrome
Sudden infant death syndrome
Sudden infant death syndrome
Sudden infant death syndrome
Sudden infant death syndrome
Sudden infant death syndrome
Sudden infant death syndrome
Sudden infant death syndrome
Sudden infant death syndrome
Other sudden death, cause unknown
Other sudden death, cause unknown
Other sudden death, cause unknown
Other sudden death, cause unknown
Other sudden death, cause unknown
Other sudden death, cause unknown
Other sudden death, cause unknown
Other sudden death, cause unknown
Other sudden death, cause unknown
Other sudden death, cause unknown
Other sudden death, cause unknown
Other sudden death, cause unknown
Other sudden death, cause unknown
Other sudden death, cause unknown
Other sudden death, cause unknown
Other sudden death, cause unknown
Other sudden death, cause unknown
Unattended death
Unattended death
Unattended death
Unattended death
Unattended death
Unattended death
Unattended death
Unattended death
Unattended death
Unattended death
Unattended death
Unattended death
Unattended death
Unattended death
Unattended death
Unattended death
Unattended death
Other ill-defined and unspecified causes of mortality
Other ill-defined and unspecified causes of mortality
Other ill-defined and unspecified causes of mortality
Other ill-defined and unspecified causes of mortality
Other ill-defined and unspecified causes of mortality

For peer review only

Other ill-defined and unspecified causes of mortality
Other ill-defined and unspecified causes of mortality
Other ill-defined and unspecified causes of mortality
Other ill-defined and unspecified causes of mortality
Other ill-defined and unspecified causes of mortality
Other ill-defined and unspecified causes of mortality
Other ill-defined and unspecified causes of mortality
Other ill-defined and unspecified causes of mortality
Other ill-defined and unspecified causes of mortality
Other ill-defined and unspecified causes of mortality
Other ill-defined and unspecified causes of mortality
Other ill-defined and unspecified causes of mortality
Other ill-defined and unspecified causes of mortality
Other ill-defined and unspecified causes of mortality
Other ill-defined and unspecified causes of mortality
Other ill-defined and unspecified causes of mortality
Pedestrian injured in collision with pedal cycle
Pedestrian injured in collision with pedal cycle
Pedestrian injured in collision with pedal cycle
Pedestrian injured in collision with two- or three-wheeled motor vehicle
Pedestrian injured in collision with two- or three-wheeled motor vehicle
Pedestrian injured in collision with two- or three-wheeled motor vehicle
Pedestrian injured in collision with two- or three-wheeled motor vehicle
Pedestrian injured in collision with two- or three-wheeled motor vehicle
Pedestrian injured in collision with two- or three-wheeled motor vehicle
Pedestrian injured in collision with two- or three-wheeled motor vehicle
Pedestrian injured in collision with car, pick-up truck or van
Pedestrian injured in collision with car, pick-up truck or van
Pedestrian injured in collision with car, pick-up truck or van
Pedestrian injured in collision with car, pick-up truck or van
Pedestrian injured in collision with car, pick-up truck or van
Pedestrian injured in collision with car, pick-up truck or van
Pedestrian injured in collision with car, pick-up truck or van
Pedestrian injured in collision with car, pick-up truck or van
Pedestrian injured in collision with car, pick-up truck or van
Pedestrian injured in collision with car, pick-up truck or van
Pedestrian injured in collision with car, pick-up truck or van
Pedestrian injured in collision with car, pick-up truck or van
Pedestrian injured in collision with car, pick-up truck or van
Pedestrian injured in collision with car, pick-up truck or van
Pedestrian injured in collision with car, pick-up truck or van
Pedestrian injured in collision with car, pick-up truck or van
Pedestrian injured in collision with car, pick-up truck or van
Pedestrian injured in collision with car, pick-up truck or van
Pedestrian injured in collision with car, pick-up truck or van
Pedestrian injured in collision with car, pick-up truck or van
Pedestrian injured in collision with car, pick-up truck or van
Pedestrian injured in collision with heavy transport vehicle or bus
Pedestrian injured in collision with heavy transport vehicle or bus
Pedestrian injured in collision with heavy transport vehicle or bus

For peer review only

Pedestrian injured in collision with heavy transport vehicle or bus
Pedestrian injured in collision with heavy transport vehicle or bus
Pedestrian injured in collision with heavy transport vehicle or bus
Pedestrian injured in collision with heavy transport vehicle or bus
Pedestrian injured in collision with heavy transport vehicle or bus
Pedestrian injured in collision with heavy transport vehicle or bus
Pedestrian injured in collision with heavy transport vehicle or bus
Pedestrian injured in collision with heavy transport vehicle or bus
Pedestrian injured in collision with heavy transport vehicle or bus
Pedestrian injured in collision with heavy transport vehicle or bus
Pedestrian injured in collision with heavy transport vehicle or bus
Pedestrian injured in collision with heavy transport vehicle or bus
Pedestrian injured in collision with heavy transport vehicle or bus
Pedestrian injured in collision with heavy transport vehicle or bus
Pedestrian injured in collision with heavy transport vehicle or bus
Pedestrian injured in collision with heavy transport vehicle or bus
Pedestrian injured in collision with heavy transport vehicle or bus
Pedestrian injured in collision with railway train or railway vehicle
Pedestrian injured in collision with railway train or railway vehicle
Pedestrian injured in collision with railway train or railway vehicle
Pedestrian injured in collision with railway train or railway vehicle
Pedestrian injured in collision with railway train or railway vehicle
Pedestrian injured in collision with railway train or railway vehicle
Pedestrian injured in collision with railway train or railway vehicle
Pedestrian injured in collision with railway train or railway vehicle
Pedestrian injured in collision with railway train or railway vehicle
Pedestrian injured in collision with railway train or railway vehicle
Pedestrian injured in collision with railway train or railway vehicle
Pedestrian injured in collision with railway train or railway vehicle
Pedestrian injured in collision with railway train or railway vehicle
Pedestrian injured in collision with railway train or railway vehicle
Pedestrian injured in collision with railway train or railway vehicle
Pedestrian injured in collision with railway train or railway vehicle
Pedestrian injured in collision with railway train or railway vehicle
Pedestrian injured in collision with railway train or railway vehicle
Pedestrian injured in collision with railway train or railway vehicle
Pedestrian injured in collision with railway train or railway vehicle
Pedestrian injured in collision with other nonmotor vehicle
Pedestrian injured in collision with other nonmotor vehicle
Pedestrian injured in other and unspecified transport accidents
Pedestrian injured in other and unspecified transport accidents
Pedestrian injured in other and unspecified transport accidents
Pedestrian injured in other and unspecified transport accidents
Pedestrian injured in other and unspecified transport accidents
Pedestrian injured in other and unspecified transport accidents
Pedestrian injured in other and unspecified transport accidents
Pedestrian injured in other and unspecified transport accidents
Pedestrian injured in other and unspecified transport accidents
Pedestrian injured in other and unspecified transport accidents
Pedestrian injured in other and unspecified transport accidents

For peer review only

Pedestrian injured in other and unspecified transport accidents
Pedestrian injured in other and unspecified transport accidents
Pedestrian injured in other and unspecified transport accidents
Pedestrian injured in other and unspecified transport accidents
Pedestrian injured in other and unspecified transport accidents
Pedestrian injured in other and unspecified transport accidents
Pedal cyclist injured in collision with other pedal cycle
Pedal cyclist injured in collision with two- or three-wheeled motor vehicle
Pedal cyclist injured in collision with two- or three-wheeled motor vehicle
Pedal cyclist injured in collision with car, pick-up truck or van
Pedal cyclist injured in collision with car, pick-up truck or van
Pedal cyclist injured in collision with car, pick-up truck or van
Pedal cyclist injured in collision with car, pick-up truck or van
Pedal cyclist injured in collision with car, pick-up truck or van
Pedal cyclist injured in collision with car, pick-up truck or van
Pedal cyclist injured in collision with car, pick-up truck or van
Pedal cyclist injured in collision with car, pick-up truck or van
Pedal cyclist injured in collision with car, pick-up truck or van
Pedal cyclist injured in collision with car, pick-up truck or van
Pedal cyclist injured in collision with car, pick-up truck or van
Pedal cyclist injured in collision with car, pick-up truck or van
Pedal cyclist injured in collision with car, pick-up truck or van
Pedal cyclist injured in collision with car, pick-up truck or van
Pedal cyclist injured in collision with car, pick-up truck or van
Pedal cyclist injured in collision with car, pick-up truck or van
Pedal cyclist injured in collision with car, pick-up truck or van
Pedal cyclist injured in collision with car, pick-up truck or van
Pedal cyclist injured in collision with car, pick-up truck or van
Pedal cyclist injured in collision with car, pick-up truck or van
Pedal cyclist injured in collision with car, pick-up truck or van
Pedal cyclist injured in collision with heavy transport vehicle or bus
Pedal cyclist injured in collision with heavy transport vehicle or bus
Pedal cyclist injured in collision with heavy transport vehicle or bus
Pedal cyclist injured in collision with heavy transport vehicle or bus
Pedal cyclist injured in collision with heavy transport vehicle or bus
Pedal cyclist injured in collision with heavy transport vehicle or bus
Pedal cyclist injured in collision with heavy transport vehicle or bus
Pedal cyclist injured in collision with heavy transport vehicle or bus
Pedal cyclist injured in collision with heavy transport vehicle or bus
Pedal cyclist injured in collision with heavy transport vehicle or bus
Pedal cyclist injured in collision with heavy transport vehicle or bus
Pedal cyclist injured in collision with railway train or railway vehicle
Pedal cyclist injured in collision with fixed or stationary object
Pedal cyclist injured in collision with fixed or stationary object
Pedal cyclist injured in noncollision transport accident
Pedal cyclist injured in noncollision transport accident
Pedal cyclist injured in noncollision transport accident
Pedal cyclist injured in noncollision transport accident
Pedal cyclist injured in noncollision transport accident
Pedal cyclist injured in noncollision transport accident
Pedal cyclist injured in noncollision transport accident

For peer review only

Pedal cyclist injured in noncollision transport accident
Pedal cyclist injured in noncollision transport accident
Pedal cyclist injured in noncollision transport accident
Pedal cyclist injured in noncollision transport accident
Pedal cyclist injured in noncollision transport accident
Pedal cyclist injured in noncollision transport accident
Pedal cyclist injured in noncollision transport accident
Pedal cyclist injured in noncollision transport accident
Pedal cyclist injured in noncollision transport accident
Pedal cyclist injured in noncollision transport accident
Pedal cyclist injured in other and unspecified transport accidents
Pedal cyclist injured in other and unspecified transport accidents
Pedal cyclist injured in other and unspecified transport accidents
Pedal cyclist injured in other and unspecified transport accidents
Pedal cyclist injured in other and unspecified transport accidents
Pedal cyclist injured in other and unspecified transport accidents
Pedal cyclist injured in other and unspecified transport accidents
Pedal cyclist injured in other and unspecified transport accidents
Pedal cyclist injured in other and unspecified transport accidents
Pedal cyclist injured in other and unspecified transport accidents
Pedal cyclist injured in other and unspecified transport accidents
Pedal cyclist injured in other and unspecified transport accidents
Pedal cyclist injured in other and unspecified transport accidents
Motorcyle rider injured in collision with two- or three-wheeled motor vehicle
Motorcyle rider injured in collision with two- or three-wheeled motor vehicle
Motorcyle rider injured in collision with two- or three-wheeled motor vehicle
Motorcyle rider injured in collision with car, pick-up truck or van
Motorcyle rider injured in collision with car, pick-up truck or van
Motorcyle rider injured in collision with car, pick-up truck or van
Motorcyle rider injured in collision with car, pick-up truck or van
Motorcyle rider injured in collision with car, pick-up truck or van
Motorcyle rider injured in collision with car, pick-up truck or van
Motorcyle rider injured in collision with car, pick-up truck or van
Motorcyle rider injured in collision with car, pick-up truck or van
Motorcyle rider injured in collision with car, pick-up truck or van
Motorcyle rider injured in collision with car, pick-up truck or van
Motorcyle rider injured in collision with car, pick-up truck or van
Motorcyle rider injured in collision with car, pick-up truck or van
Motorcyle rider injured in collision with car, pick-up truck or van
Motorcyle rider injured in collision with car, pick-up truck or van
Motorcyle rider injured in collision with car, pick-up truck or van
Motorcyle rider injured in collision with car, pick-up truck or van
Motorcyle rider injured in collision with car, pick-up truck or van
Motorcyle rider injured in collision with car, pick-up truck or van
Motorcyle rider injured in collision with car, pick-up truck or van
Motorcyle rider injured in collision with heavy transport vehicle or bus
Motorcyle rider injured in collision with heavy transport vehicle or bus
Motorcyle rider injured in collision with heavy transport vehicle or bus
Motorcyle rider injured in collision with heavy transport vehicle or bus
Motorcyle rider injured in collision with heavy transport vehicle or bus

For peer review only

Motorcyle rider injured in collision with fixed or stationary object
Motorcyle rider injured in collision with fixed or stationary object
Motorcyle rider injured in collision with fixed or stationary object
Motorcyle rider injured in collision with fixed or stationary object
Motorcyle rider injured in collision with fixed or stationary object
Motorcyle rider injured in collision with fixed or stationary object
Motorcyle rider injured in collision with fixed or stationary object
Motorcyle rider injured in collision with fixed or stationary object
Motorcyle rider injured in collision with fixed or stationary object
Motorcyle rider injured in collision with fixed or stationary object
Motorcyle rider injured in collision with fixed or stationary object
Motorcyle rider injured in collision with fixed or stationary object
Motorcyle rider injured in collision with fixed or stationary object
Motorcyle rider injured in noncollision transport accident
Motorcyle rider injured in noncollision transport accident
Motorcyle rider injured in noncollision transport accident
Motorcyle rider injured in noncollision transport accident
Motorcyle rider injured in noncollision transport accident
Motorcyle rider injured in noncollision transport accident
Motorcyle rider injured in noncollision transport accident
Motorcyle rider injured in noncollision transport accident
Motorcyle rider injured in noncollision transport accident
Motorcyle rider injured in noncollision transport accident
Motorcyle rider injured in noncollision transport accident
Motorcyle rider injured in noncollision transport accident
Motorcyle rider injured in noncollision transport accident
Motorcyle rider injured in noncollision transport accident
Motorcyle rider injured in noncollision transport accident
Motorcyle rider injured in noncollision transport accident
Motorcyle rider injured in other and unspecified transport accidents
Motorcyle rider injured in other and unspecified transport accidents
Motorcyle rider injured in other and unspecified transport accidents
Motorcyle rider injured in other and unspecified transport accidents
Motorcyle rider injured in other and unspecified transport accidents
Motorcyle rider injured in other and unspecified transport accidents
Motorcyle rider injured in other and unspecified transport accidents
Motorcyle rider injured in other and unspecified transport accidents
Motorcyle rider injured in other and unspecified transport accidents
Motorcyle rider injured in other and unspecified transport accidents
Motorcyle rider injured in other and unspecified transport accidents
Motorcyle rider injured in other and unspecified transport accidents
Motorcyle rider injured in other and unspecified transport accidents
Motorcyle rider injured in other and unspecified transport accidents
Motorcyle rider injured in other and unspecified transport accidents
Motorcyle rider injured in other and unspecified transport accidents
Occupant of three-wheeled motor vehicle injured in collision with car, pick-up truck or van
Occupant of three-wheeled motor vehicle injured in noncollision transport accident
Occupant of three-wheeled motor vehicle injured in noncollision transport accident
Car occupant injured in collision with pedestrian or animal
Car occupant injured in collision with pedestrian or animal

For peer review only

Car occupant injured in collision with two- or three-wheeled motor vehicle
Car occupant injured in collision with car, pick-up truck or van
Car occupant injured in collision with car, pick-up truck or van
Car occupant injured in collision with car, pick-up truck or van
Car occupant injured in collision with car, pick-up truck or van
Car occupant injured in collision with car, pick-up truck or van
Car occupant injured in collision with car, pick-up truck or van
Car occupant injured in collision with car, pick-up truck or van
Car occupant injured in collision with car, pick-up truck or van
Car occupant injured in collision with car, pick-up truck or van
Car occupant injured in collision with car, pick-up truck or van
Car occupant injured in collision with car, pick-up truck or van
Car occupant injured in collision with car, pick-up truck or van
Car occupant injured in collision with car, pick-up truck or van
Car occupant injured in collision with car, pick-up truck or van
Car occupant injured in collision with car, pick-up truck or van
Car occupant injured in collision with car, pick-up truck or van
Car occupant injured in collision with car, pick-up truck or van
Car occupant injured in collision with car, pick-up truck or van
Car occupant injured in collision with car, pick-up truck or van
Car occupant injured in collision with car, pick-up truck or van
Car occupant injured in collision with car, pick-up truck or van
Car occupant injured in collision with heavy transport vehicle or bus
Car occupant injured in collision with heavy transport vehicle or bus
Car occupant injured in collision with heavy transport vehicle or bus
Car occupant injured in collision with heavy transport vehicle or bus
Car occupant injured in collision with heavy transport vehicle or bus
Car occupant injured in collision with heavy transport vehicle or bus
Car occupant injured in collision with heavy transport vehicle or bus
Car occupant injured in collision with heavy transport vehicle or bus
Car occupant injured in collision with heavy transport vehicle or bus
Car occupant injured in collision with heavy transport vehicle or bus
Car occupant injured in collision with heavy transport vehicle or bus
Car occupant injured in collision with heavy transport vehicle or bus
Car occupant injured in collision with heavy transport vehicle or bus
Car occupant injured in collision with heavy transport vehicle or bus
Car occupant injured in collision with heavy transport vehicle or bus
Car occupant injured in collision with heavy transport vehicle or bus
Car occupant injured in collision with heavy transport vehicle or bus
Car occupant injured in collision with heavy transport vehicle or bus
Car occupant injured in collision with heavy transport vehicle or bus
Car occupant injured in collision with heavy transport vehicle or bus
Car occupant injured in collision with railway train or railway vehicle
Car occupant injured in collision with railway train or railway vehicle
Car occupant injured in collision with railway train or railway vehicle
Car occupant injured in collision with railway train or railway vehicle
Car occupant injured in collision with railway train or railway vehicle
Car occupant injured in collision with railway train or railway vehicle
Car occupant injured in collision with railway train or railway vehicle
Car occupant injured in collision with railway train or railway vehicle

For peer review only

Car occupant injured in collision with railway train or railway vehicle
Car occupant injured in collision with railway train or railway vehicle
Car occupant injured in collision with fixed or stationary object
Car occupant injured in collision with fixed or stationary object
Car occupant injured in collision with fixed or stationary object
Car occupant injured in collision with fixed or stationary object
Car occupant injured in collision with fixed or stationary object
Car occupant injured in collision with fixed or stationary object
Car occupant injured in collision with fixed or stationary object
Car occupant injured in collision with fixed or stationary object
Car occupant injured in collision with fixed or stationary object
Car occupant injured in collision with fixed or stationary object
Car occupant injured in collision with fixed or stationary object
Car occupant injured in collision with fixed or stationary object
Car occupant injured in collision with fixed or stationary object
Car occupant injured in collision with fixed or stationary object
Car occupant injured in collision with fixed or stationary object
Car occupant injured in collision with fixed or stationary object
Car occupant injured in collision with fixed or stationary object
Car occupant injured in collision with fixed or stationary object
Car occupant injured in collision with fixed or stationary object
Car occupant injured in collision with fixed or stationary object
Car occupant injured in noncollision transport accident
Car occupant injured in noncollision transport accident
Car occupant injured in noncollision transport accident
Car occupant injured in noncollision transport accident
Car occupant injured in noncollision transport accident
Car occupant injured in noncollision transport accident
Car occupant injured in noncollision transport accident
Car occupant injured in noncollision transport accident
Car occupant injured in noncollision transport accident
Car occupant injured in noncollision transport accident
Car occupant injured in noncollision transport accident
Car occupant injured in noncollision transport accident
Car occupant injured in noncollision transport accident
Car occupant injured in noncollision transport accident
Car occupant injured in noncollision transport accident
Car occupant injured in noncollision transport accident
Car occupant injured in noncollision transport accident
Car occupant injured in noncollision transport accident
Car occupant injured in noncollision transport accident
Car occupant injured in noncollision transport accident
Car occupant injured in noncollision transport accident
Car occupant injured in other and unspecified transport accidents
Car occupant injured in other and unspecified transport accidents
Car occupant injured in other and unspecified transport accidents
Car occupant injured in other and unspecified transport accidents
Car occupant injured in other and unspecified transport accidents
Car occupant injured in other and unspecified transport accidents
Car occupant injured in other and unspecified transport accidents

For peer review only

Car occupant injured in other and unspecified transport accidents
Car occupant injured in other and unspecified transport accidents
Car occupant injured in other and unspecified transport accidents
Car occupant injured in other and unspecified transport accidents
Car occupant injured in other and unspecified transport accidents
Car occupant injured in other and unspecified transport accidents
Car occupant injured in other and unspecified transport accidents
Car occupant injured in other and unspecified transport accidents
Car occupant injured in other and unspecified transport accidents
Car occupant injured in other and unspecified transport accidents
Car occupant injured in other and unspecified transport accidents
Car occupant injured in other and unspecified transport accidents
Car occupant injured in other and unspecified transport accidents
Car occupant injured in other and unspecified transport accidents
Occupant of pick-up truck or van injured in collision with car, pick-up truck or van
Occupant of pick-up truck or van injured in collision with car, pick-up truck or van
Occupant of pick-up truck or van injured in collision with car, pick-up truck or van
Occupant of pick-up truck or van injured in collision with car, pick-up truck or van
Occupant of pick-up truck or van injured in collision with heavy transport vehicle or bus
Occupant of pick-up truck or van injured in collision with heavy transport vehicle or bus
Occupant of pick-up truck or van injured in collision with heavy transport vehicle or bus
Occupant of pick-up truck or van injured in collision with fixed or stationary object
Occupant of pick-up truck or van injured in collision with fixed or stationary object
Occupant of pick-up truck or van injured in collision with fixed or stationary object
Occupant of pick-up truck or van injured in noncollision transport accident
Occupant of pick-up truck or van injured in other and unspecified transport accidents
Occupant of pick-up truck or van injured in other and unspecified transport accidents
Occupant of pick-up truck or van injured in other and unspecified transport accidents
Occupant of heavy transport vehicle injured in collision with car, pick-up truck or van
Occupant of heavy transport vehicle injured in collision with heavy transport vehicle or bus
Occupant of heavy transport vehicle injured in collision with heavy transport vehicle or bus
Occupant of heavy transport vehicle injured in collision with heavy transport vehicle or bus
Occupant of heavy transport vehicle injured in collision with fixed or stationary object
Occupant of heavy transport vehicle injured in collision with fixed or stationary object
Occupant of heavy transport vehicle injured in noncollision transport accident
Occupant of heavy transport vehicle injured in noncollision transport accident
Occupant of heavy transport vehicle injured in noncollision transport accident
Occupant of heavy transport vehicle injured in noncollision transport accident
Occupant of heavy transport vehicle injured in other and unspecified transport accidents
Occupant of heavy transport vehicle injured in other and unspecified transport accidents
Occupant of heavy transport vehicle injured in other and unspecified transport accidents
Occupant of heavy transport vehicle injured in other and unspecified transport accidents
Occupant of heavy transport vehicle injured in other and unspecified transport accidents
Bus occupant injured in noncollision transport accident
Bus occupant injured in noncollision transport accident
Bus occupant injured in other and unspecified transport accidents
Animal-rider or occupant of animal-drawn vehicle injured in transport accident
Animal-rider or occupant of animal-drawn vehicle injured in transport accident
Animal-rider or occupant of animal-drawn vehicle injured in transport accident
Animal-rider or occupant of animal-drawn vehicle injured in transport accident

For peer review only

Animal-rider or occupant of animal-drawn vehicle injured in transport accident
Animal-rider or occupant of animal-drawn vehicle injured in transport accident
Animal-rider or occupant of animal-drawn vehicle injured in transport accident
Animal-rider or occupant of animal-drawn vehicle injured in transport accident
Occupant of railway train or railway vehicle injured in transport accident
Occupant of railway train or railway vehicle injured in transport accident
Occupant of railway train or railway vehicle injured in transport accident
Occupant of railway train or railway vehicle injured in transport accident
Occupant of railway train or railway vehicle injured in transport accident
Occupant of railway train or railway vehicle injured in transport accident
Occupant of streetcar injured in transport accident
Occupant of special vehicle mainly used on industrial premises injured in transport accident
Occupant of special vehicle mainly used on industrial premises injured in transport accident
Occupant of special vehicle mainly used in agriculture injured in transport accident
Occupant of special vehicle mainly used in agriculture injured in transport accident
Occupant of special vehicle mainly used in agriculture injured in transport accident
Occupant of special vehicle mainly used in agriculture injured in transport accident
Occupant of special vehicle mainly used in agriculture injured in transport accident
Occupant of special vehicle mainly used in agriculture injured in transport accident
Occupant of special vehicle mainly used in agriculture injured in transport accident
Occupant of special vehicle mainly used in agriculture injured in transport accident
Occupant of special vehicle mainly used in agriculture injured in transport accident
Occupant of special vehicle mainly used in agriculture injured in transport accident
Occupant of special vehicle mainly used in agriculture injured in transport accident
Occupant of special vehicle mainly used in agriculture injured in transport accident
Occupant of special vehicle mainly used in agriculture injured in transport accident
Occupant of special vehicle mainly used in agriculture injured in transport accident
Occupant of special construction vehicle injured in transport accident
Occupant of special all-terrain or other motor vehicles designed primarily for off-road use, injured in transport
Occupant of special all-terrain or other motor vehicles designed primarily for off-road use, injured in transport
Occupant of special all-terrain or other motor vehicles designed primarily for off-road use, injured in transport
Occupant of special all-terrain or other motor vehicles designed primarily for off-road use, injured in transport
Occupant of special all-terrain or other motor vehicles designed primarily for off-road use, injured in transport
Occupant of special all-terrain or other motor vehicles designed primarily for off-road use, injured in transport
Occupant of special all-terrain or other motor vehicles designed primarily for off-road use, injured in transport
Traffic accident of specified type but victim's mode of transport unknown
Traffic accident of specified type but victim's mode of transport unknown
Traffic accident of specified type but victim's mode of transport unknown
Traffic accident of specified type but victim's mode of transport unknown
Motor- or nonmotor-vehicle accident, type of vehicle unspecified
Motor- or nonmotor-vehicle accident, type of vehicle unspecified
Motor- or nonmotor-vehicle accident, type of vehicle unspecified
Motor- or nonmotor-vehicle accident, type of vehicle unspecified
Motor- or nonmotor-vehicle accident, type of vehicle unspecified
Motor- or nonmotor-vehicle accident, type of vehicle unspecified
Motor- or nonmotor-vehicle accident, type of vehicle unspecified
Motor- or nonmotor-vehicle accident, type of vehicle unspecified
Motor- or nonmotor-vehicle accident, type of vehicle unspecified
Motor- or nonmotor-vehicle accident, type of vehicle unspecified
Motor- or nonmotor-vehicle accident, type of vehicle unspecified

For peer review only

Motor- or nonmotor-vehicle accident, type of vehicle unspecified
Motor- or nonmotor-vehicle accident, type of vehicle unspecified
Motor- or nonmotor-vehicle accident, type of vehicle unspecified
Motor- or nonmotor-vehicle accident, type of vehicle unspecified
Accident to watercraft causing drowning and submersion
Accident to watercraft causing drowning and submersion
Accident to watercraft causing drowning and submersion
Accident to watercraft causing drowning and submersion
Water-transport-related drowning and submersion without accident to watercraft
Water-transport-related drowning and submersion without accident to watercraft
Water-transport-related drowning and submersion without accident to watercraft
Water-transport-related drowning and submersion without accident to watercraft
Accident on board watercraft without accident to watercraft, not causing drowning and submersion
Accident to powered aircraft causing injury to occupant
Accident to powered aircraft causing injury to occupant
Accident to powered aircraft causing injury to occupant
Accident to powered aircraft causing injury to occupant
Accident to powered aircraft causing injury to occupant
Accident to powered aircraft causing injury to occupant
Accident to powered aircraft causing injury to occupant
Accident to powered aircraft causing injury to occupant
Accident to powered aircraft causing injury to occupant
Accident to powered aircraft causing injury to occupant
Accident to nonpowered aircraft causing injury to occupant
Accident to nonpowered aircraft causing injury to occupant
Accident to nonpowered aircraft causing injury to occupant
Accident to nonpowered aircraft causing injury to occupant
Other specified air transport accidents
Unspecified transport accident
Unspecified transport accident
Unspecified transport accident
Unspecified transport accident
Unspecified transport accident
Unspecified transport accident
Unspecified transport accident
Fall on same level involving ice and snow
Fall on same level involving ice and snow
Fall on same level involving ice and snow
Fall on same level involving ice and snow
Fall on same level involving ice and snow
Fall on same level involving ice and snow
Fall on same level from slipping, tripping and stumbling
Fall on same level from slipping, tripping and stumbling
Fall on same level from slipping, tripping and stumbling
Fall on same level from slipping, tripping and stumbling
Fall on same level from slipping, tripping and stumbling
Fall on same level from slipping, tripping and stumbling
Fall on same level from slipping, tripping and stumbling
Fall on same level from slipping, tripping and stumbling
Fall on same level from slipping, tripping and stumbling

For peer review only

Fall on same level from slipping, tripping and stumbling
Fall on same level from slipping, tripping and stumbling
Fall on same level from slipping, tripping and stumbling
Fall on same level from slipping, tripping and stumbling
Fall on same level from slipping, tripping and stumbling
Fall on same level from slipping, tripping and stumbling
Fall on same level from slipping, tripping and stumbling
Fall on same level from slipping, tripping and stumbling
Fall on same level from slipping, tripping and stumbling
Fall on same level from slipping, tripping and stumbling
Fall on same level from slipping, tripping and stumbling
Fall on same level from slipping, tripping and stumbling
Fall involving ice-skates, skis, roller-skates or skateboards
Fall involving ice-skates, skis, roller-skates or skateboards
Fall involving ice-skates, skis, roller-skates or skateboards
Fall involving ice-skates, skis, roller-skates or skateboards
Other fall on same level due to collision with, or pushing by, another person
Other fall on same level due to collision with, or pushing by, another person
Fall while being carried or supported by other persons
Fall involving wheelchair
Fall involving wheelchair
Fall involving wheelchair
Fall involving wheelchair
Fall involving wheelchair
Fall involving wheelchair
Fall involving wheelchair
Fall involving wheelchair
Fall involving wheelchair
Fall involving wheelchair
Fall involving wheelchair
Fall involving bed
Fall involving bed
Fall involving bed
Fall involving bed
Fall involving bed
Fall involving bed
Fall involving bed
Fall involving bed
Fall involving bed
Fall involving bed
Fall involving bed
Fall involving bed
Fall involving bed
Fall involving bed
Fall involving bed
Fall involving bed
Fall involving bed
Fall involving bed
Fall involving bed
Fall involving chair

For peer review only

Fall involving chair
Fall involving chair
Fall involving chair
Fall involving chair
Fall involving chair
Fall involving chair
Fall involving chair
Fall involving chair
Fall involving chair
Fall involving chair
Fall involving other furniture
Fall involving other furniture
Fall involving other furniture
Fall involving other furniture
Fall involving other furniture
Fall involving playground equipment
Fall on and from stairs and steps
Fall on and from stairs and steps
Fall on and from stairs and steps
Fall on and from stairs and steps
Fall on and from stairs and steps
Fall on and from stairs and steps
Fall on and from stairs and steps
Fall on and from stairs and steps
Fall on and from stairs and steps
Fall on and from stairs and steps
Fall on and from stairs and steps
Fall on and from stairs and steps
Fall on and from stairs and steps
Fall on and from stairs and steps
Fall on and from stairs and steps
Fall on and from stairs and steps
Fall on and from stairs and steps
Fall on and from stairs and steps
Fall on and from stairs and steps
Fall on and from stairs and steps
Fall on and from ladder
Fall on and from ladder
Fall on and from ladder
Fall on and from ladder
Fall on and from ladder
Fall on and from ladder
Fall on and from ladder
Fall on and from ladder
Fall on and from ladder
Fall on and from ladder
Fall on and from ladder
Fall on and from ladder
Fall on and from ladder
Fall on and from ladder

For peer review only

For peer review only - http://bmjopen.bmj.com/site/about/guidelines.xhtml

Fall on and from ladder
Fall on and from ladder
Fall on and from ladder
Fall on and from ladder
Fall on and from scaffolding
Fall on and from scaffolding
Fall on and from scaffolding
Fall on and from scaffolding
Fall on and from scaffolding
Fall on and from scaffolding
Fall on and from scaffolding
Fall from, out of or through building or structure
Fall from, out of or through building or structure
Fall from, out of or through building or structure
Fall from, out of or through building or structure
Fall from, out of or through building or structure
Fall from, out of or through building or structure
Fall from, out of or through building or structure
Fall from, out of or through building or structure
Fall from, out of or through building or structure
Fall from, out of or through building or structure
Fall from, out of or through building or structure
Fall from, out of or through building or structure
Fall from, out of or through building or structure
Fall from, out of or through building or structure
Fall from, out of or through building or structure
Fall from, out of or through building or structure
Fall from, out of or through building or structure
Fall from, out of or through building or structure
Fall from, out of or through building or structure
Fall from, out of or through building or structure
Fall from, out of or through building or structure
Fall from tree
Fall from tree
Fall from tree
Fall from tree
Fall from tree
Fall from tree
Fall from tree
Fall from tree
Fall from tree
Fall from cliff
Fall from cliff
Fall from cliff
Fall from cliff
Fall from cliff
Fall from cliff
Fall from cliff
Fall from cliff
Fall from cliff

For peer review only

Diving or jumping into water causing injury other than drowning or submersion
Diving or jumping into water causing injury other than drowning or submersion
Other fall from one level to another
Other fall from one level to another
Other fall from one level to another
Other fall from one level to another
Other fall from one level to another
Other fall from one level to another
Other fall from one level to another
Other fall from one level to another
Other fall from one level to another
Other fall from one level to another
Other fall from one level to another
Other fall from one level to another
Other fall from one level to another
Other fall from one level to another
Other fall from one level to another
Other fall from one level to another
Other fall from one level to another
Other fall from one level to another
Other fall from one level to another
Other fall from one level to another
Other fall on same level
Other fall on same level
Other fall on same level
Other fall on same level
Other fall on same level
Other fall on same level
Other fall on same level
Other fall on same level
Other fall on same level
Other fall on same level
Other fall on same level
Other fall on same level
Other fall on same level
Other fall on same level
Other fall on same level
Other fall on same level
Other fall on same level
Other fall on same level
Other fall on same level
Other fall on same level
Unspecified fall
Unspecified fall
Unspecified fall
Unspecified fall
Unspecified fall
Unspecified fall
Unspecified fall
Unspecified fall

For peer review only

Unspecified fall
Unspecified fall
Unspecified fall
Unspecified fall
Unspecified fall
Unspecified fall
Unspecified fall
Unspecified fall
Unspecified fall
Unspecified fall
Unspecified fall
Unspecified fall
Unspecified fall
Struck by thrown, projected or falling object
Struck by thrown, projected or falling object
Struck by thrown, projected or falling object
Struck by thrown, projected or falling object
Struck by thrown, projected or falling object
Struck by thrown, projected or falling object
Struck by thrown, projected or falling object
Struck by thrown, projected or falling object
Struck by thrown, projected or falling object
Struck by thrown, projected or falling object
Struck by thrown, projected or falling object
Struck by thrown, projected or falling object
Struck by thrown, projected or falling object
Struck by thrown, projected or falling object
Struck by thrown, projected or falling object
Struck by thrown, projected or falling object
Struck by thrown, projected or falling object
Struck by thrown, projected or falling object
Struck by thrown, projected or falling object
Struck by thrown, projected or falling object
Struck by thrown, projected or falling object
Striking against or struck by other objects
Striking against or struck by other objects
Striking against or struck by other objects
Striking against or struck by other objects
Striking against or struck by other objects
Striking against or struck by other objects
Caught, crushed, jammed or pinched in or between objects
Caught, crushed, jammed or pinched in or between objects
Caught, crushed, jammed or pinched in or between objects
Caught, crushed, jammed or pinched in or between objects
Caught, crushed, jammed or pinched in or between objects
Caught, crushed, jammed or pinched in or between objects
Caught, crushed, jammed or pinched in or between objects
Caught, crushed, jammed or pinched in or between objects
Caught, crushed, jammed or pinched in or between objects
Caught, crushed, jammed or pinched in or between objects

For peer review only

Caught, crushed, jammed or pinched in or between objects
Caught, crushed, jammed or pinched in or between objects
Caught, crushed, jammed or pinched in or between objects
Caught, crushed, jammed or pinched in or between objects
Caught, crushed, jammed or pinched in or between objects
Caught, crushed, jammed or pinched in or between objects
Caught, crushed, jammed or pinched in or between objects
Contact with lifting and transmission devices, not elsewhere classified
Contact with lifting and transmission devices, not elsewhere classified
Contact with lifting and transmission devices, not elsewhere classified
Contact with lifting and transmission devices, not elsewhere classified
Contact with lifting and transmission devices, not elsewhere classified
Contact with sharp glass
Contact with knife, sword or dagger
Contact with other powered hand tools and household machinery
Contact with other powered hand tools and household machinery
Contact with other powered hand tools and household machinery
Contact with other powered hand tools and household machinery
Contact with agricultural machinery
Contact with agricultural machinery
Contact with agricultural machinery
Contact with agricultural machinery
Contact with agricultural machinery
Contact with agricultural machinery
Contact with agricultural machinery
Contact with other and unspecified machinery
Contact with other and unspecified machinery
Contact with other and unspecified machinery
Contact with other and unspecified machinery
Contact with other and unspecified machinery
Contact with other and unspecified machinery
Rifle, shotgun and larger firearm discharge
Rifle, shotgun and larger firearm discharge
Discharge from other and unspecified firearms
Discharge from other and unspecified firearms
Discharge from other and unspecified firearms
Discharge from other and unspecified firearms
Explosion and rupture of gas cylinder
Explosion of other materials
Explosion of other materials
Explosion of other materials
Explosion of other materials
Explosion of other materials
Explosion of other materials
Explosion of other materials
Foreign body entering into or through eye or natural orifice
Foreign body entering into or through eye or natural orifice
Exposure to other and unspecified inanimate mechanical forces
Exposure to other and unspecified inanimate mechanical forces
Exposure to other and unspecified inanimate mechanical forces

For peer review only

Exposure to other and unspecified inanimate mechanical forces
Hit, struck, kicked, twisted, bitten or scratched by another person
Bitten or struck by dog
Bitten or struck by dog
Bitten or struck by other mammals
Bitten or struck by other mammals
Bitten or struck by other mammals
Bitten or struck by other mammals
Bitten or struck by other mammals
Bitten or struck by other mammals
Bitten or struck by other mammals
Bitten or struck by other mammals
Exposure to other and unspecified animate mechanical forces
Drowning and submersion while in bath-tub
Drowning and submersion while in bath-tub
Drowning and submersion while in bath-tub
Drowning and submersion while in bath-tub
Drowning and submersion while in bath-tub
Drowning and submersion while in bath-tub
Drowning and submersion while in bath-tub
Drowning and submersion while in bath-tub
Drowning and submersion while in bath-tub
Drowning and submersion while in bath-tub
Drowning and submersion while in bath-tub
Drowning and submersion following fall into bath-tub
Drowning and submersion while in swimming-pool
Drowning and submersion while in swimming-pool
Drowning and submersion while in swimming-pool
Drowning and submersion while in swimming-pool
Drowning and submersion following fall into swimming-pool
Drowning and submersion following fall into swimming-pool
Drowning and submersion while in natural water
Drowning and submersion while in natural water
Drowning and submersion while in natural water
Drowning and submersion while in natural water
Drowning and submersion while in natural water
Drowning and submersion while in natural water
Drowning and submersion while in natural water
Drowning and submersion while in natural water
Drowning and submersion while in natural water
Drowning and submersion while in natural water
Drowning and submersion while in natural water
Drowning and submersion while in natural water
Drowning and submersion while in natural water
Drowning and submersion while in natural water
Drowning and submersion while in natural water
Drowning and submersion while in natural water
Drowning and submersion while in natural water
Drowning and submersion while in natural water
Drowning and submersion while in natural water

For peer review only

Drowning and submersion while in natural water
Drowning and submersion while in natural water
Drowning and submersion following fall into natural water
Drowning and submersion following fall into natural water
Drowning and submersion following fall into natural water
Drowning and submersion following fall into natural water
Drowning and submersion following fall into natural water
Drowning and submersion following fall into natural water
Drowning and submersion following fall into natural water
Drowning and submersion following fall into natural water
Drowning and submersion following fall into natural water
Drowning and submersion following fall into natural water
Drowning and submersion following fall into natural water
Drowning and submersion following fall into natural water
Drowning and submersion following fall into natural water
Drowning and submersion following fall into natural water
Drowning and submersion following fall into natural water
Drowning and submersion following fall into natural water
Drowning and submersion following fall into natural water
Drowning and submersion following fall into natural water
Drowning and submersion following fall into natural water
Drowning and submersion following fall into natural water
Other specified drowning and submersion
Other specified drowning and submersion
Other specified drowning and submersion
Other specified drowning and submersion
Other specified drowning and submersion
Other specified drowning and submersion
Other specified drowning and submersion
Other specified drowning and submersion
Other specified drowning and submersion
Other specified drowning and submersion
Other specified drowning and submersion
Other specified drowning and submersion
Unspecified drowning and submersion
Unspecified drowning and submersion
Unspecified drowning and submersion
Unspecified drowning and submersion
Unspecified drowning and submersion
Unspecified drowning and submersion
Unspecified drowning and submersion
Unspecified drowning and submersion
Unspecified drowning and submersion
Unspecified drowning and submersion
Unspecified drowning and submersion
Unspecified drowning and submersion
Unspecified drowning and submersion
Unspecified drowning and submersion
Unspecified drowning and submersion
Unspecified drowning and submersion
Unspecified drowning and submersion

For peer review only

Unspecified drowning and submersion
Unspecified drowning and submersion
Accidental suffocation and strangulation in bed
Accidental suffocation and strangulation in bed
Accidental suffocation and strangulation in bed
Accidental suffocation and strangulation in bed
Accidental suffocation and strangulation in bed
Accidental suffocation and strangulation in bed
Other accidental hanging and strangulation
Other accidental hanging and strangulation
Other accidental hanging and strangulation
Other accidental hanging and strangulation
Other accidental hanging and strangulation
Other accidental hanging and strangulation
Other accidental hanging and strangulation
Other accidental hanging and strangulation
Other accidental hanging and strangulation
Other accidental hanging and strangulation
Threat to breathing due to cave-in, falling earth and other substances
Threat to breathing due to cave-in, falling earth and other substances
Threat to breathing due to cave-in, falling earth and other substances
Threat to breathing due to cave-in, falling earth and other substances
Threat to breathing due to cave-in, falling earth and other substances
Threat to breathing due to cave-in, falling earth and other substances
Inhalation of gastric contents
Inhalation of gastric contents
Inhalation of gastric contents
Inhalation of gastric contents
Inhalation of gastric contents
Inhalation of gastric contents
Inhalation of gastric contents
Inhalation of gastric contents
Inhalation of gastric contents
Inhalation of gastric contents
Inhalation of gastric contents
Inhalation of gastric contents
Inhalation of gastric contents
Inhalation of gastric contents
Inhalation of gastric contents
Inhalation of gastric contents
Inhalation of gastric contents
Inhalation of gastric contents
Inhalation of gastric contents
Inhalation of gastric contents
Inhalation of gastric contents
Inhalation and ingestion of food causing obstruction of respiratory tract
Inhalation and ingestion of food causing obstruction of respiratory tract
Inhalation and ingestion of food causing obstruction of respiratory tract
Inhalation and ingestion of food causing obstruction of respiratory tract
Inhalation and ingestion of food causing obstruction of respiratory tract

For peer review only

Inhalation and ingestion of food causing obstruction of respiratory tract
Inhalation and ingestion of food causing obstruction of respiratory tract
Inhalation and ingestion of food causing obstruction of respiratory tract
Inhalation and ingestion of food causing obstruction of respiratory tract
Inhalation and ingestion of food causing obstruction of respiratory tract
Inhalation and ingestion of food causing obstruction of respiratory tract
Inhalation and ingestion of food causing obstruction of respiratory tract
Inhalation and ingestion of food causing obstruction of respiratory tract
Inhalation and ingestion of food causing obstruction of respiratory tract
Inhalation and ingestion of food causing obstruction of respiratory tract
Inhalation and ingestion of food causing obstruction of respiratory tract
Inhalation and ingestion of food causing obstruction of respiratory tract
Inhalation and ingestion of food causing obstruction of respiratory tract
Inhalation and ingestion of food causing obstruction of respiratory tract
Inhalation and ingestion of food causing obstruction of respiratory tract
Inhalation and ingestion of food causing obstruction of respiratory tract
Inhalation and ingestion of other objects causing obstruction of respiratory tract
Inhalation and ingestion of other objects causing obstruction of respiratory tract
Inhalation and ingestion of other objects causing obstruction of respiratory tract
Inhalation and ingestion of other objects causing obstruction of respiratory tract
Inhalation and ingestion of other objects causing obstruction of respiratory tract
Inhalation and ingestion of other objects causing obstruction of respiratory tract
Inhalation and ingestion of other objects causing obstruction of respiratory tract
Inhalation and ingestion of other objects causing obstruction of respiratory tract
Inhalation and ingestion of other objects causing obstruction of respiratory tract
Inhalation and ingestion of other objects causing obstruction of respiratory tract
Inhalation and ingestion of other objects causing obstruction of respiratory tract
Inhalation and ingestion of other objects causing obstruction of respiratory tract
Inhalation and ingestion of other objects causing obstruction of respiratory tract
Inhalation and ingestion of other objects causing obstruction of respiratory tract
Other specified threats to breathing
Other specified threats to breathing
Other specified threats to breathing
Other specified threats to breathing
Unspecified threat to breathing
Unspecified threat to breathing
Unspecified threat to breathing
Unspecified threat to breathing
Unspecified threat to breathing
Unspecified threat to breathing
Unspecified threat to breathing
Unspecified threat to breathing
Unspecified threat to breathing
Unspecified threat to breathing
Unspecified threat to breathing
Unspecified threat to breathing
Unspecified threat to breathing
Exposure to electric transmission lines
Exposure to electric transmission lines
Exposure to electric transmission lines

For peer review only

Exposure to electric transmission lines
Exposure to electric transmission lines
Exposure to electric transmission lines
Exposure to other specified electric current
Exposure to other specified electric current
Exposure to other specified electric current
Exposure to other specified electric current
Exposure to other specified electric current
Exposure to unspecified electric current
Exposure to unspecified electric current
Exposure to unspecified electric current
Exposure to unspecified electric current
Exposure to unspecified electric current
Exposure to unspecified electric current
Exposure to unspecified electric current
Exposure to unspecified electric current
Exposure to unspecified electric current
Exposure to unspecified electric current
Exposure to unspecified electric current
Exposure to man-made visible and ultraviolet light
Exposure to excessive heat of man-made origin
Exposure to excessive heat of man-made origin
Exposure to excessive heat of man-made origin
Exposure to excessive cold of man-made origin
Exposure to uncontrolled fire in building or structure
Exposure to uncontrolled fire in building or structure
Exposure to uncontrolled fire in building or structure
Exposure to uncontrolled fire in building or structure
Exposure to uncontrolled fire in building or structure
Exposure to uncontrolled fire in building or structure
Exposure to uncontrolled fire in building or structure
Exposure to uncontrolled fire in building or structure
Exposure to uncontrolled fire in building or structure
Exposure to uncontrolled fire in building or structure
Exposure to uncontrolled fire in building or structure
Exposure to uncontrolled fire in building or structure
Exposure to uncontrolled fire in building or structure
Exposure to uncontrolled fire in building or structure
Exposure to uncontrolled fire in building or structure
Exposure to uncontrolled fire in building or structure
Exposure to uncontrolled fire in building or structure
Exposure to uncontrolled fire in building or structure
Exposure to uncontrolled fire in building or structure
Exposure to uncontrolled fire in building or structure
Exposure to uncontrolled fire in building or structure
Exposure to uncontrolled fire, not in building or structure
Exposure to controlled fire in building or structure
Exposure to controlled fire in building or structure
Exposure to controlled fire in building or structure
Exposure to controlled fire in building or structure

For peer review only

Exposure to controlled fire in building or structure
Exposure to controlled fire in building or structure
Exposure to controlled fire in building or structure
Exposure to controlled fire in building or structure
Exposure to controlled fire, not in building or structure
Exposure to ignition of highly flammable material
Exposure to ignition of highly flammable material
Exposure to ignition of highly flammable material
Exposure to ignition or melting of nightwear
Exposure to ignition or melting of other clothing and apparel
Exposure to ignition or melting of other clothing and apparel
Exposure to ignition or melting of other clothing and apparel
Exposure to ignition or melting of other clothing and apparel
Exposure to ignition or melting of other clothing and apparel
Exposure to other specified smoke, fire and flames
Exposure to other specified smoke, fire and flames
Exposure to other specified smoke, fire and flames
Exposure to other specified smoke, fire and flames
Exposure to other specified smoke, fire and flames
Exposure to other specified smoke, fire and flames
Exposure to other specified smoke, fire and flames
Exposure to other specified smoke, fire and flames
Exposure to unspecified smoke, fire and flames
Exposure to unspecified smoke, fire and flames
Exposure to unspecified smoke, fire and flames
Exposure to unspecified smoke, fire and flames
Exposure to unspecified smoke, fire and flames
Exposure to unspecified smoke, fire and flames
Exposure to unspecified smoke, fire and flames
Exposure to unspecified smoke, fire and flames
Exposure to unspecified smoke, fire and flames
Exposure to unspecified smoke, fire and flames
Exposure to unspecified smoke, fire and flames
Exposure to unspecified smoke, fire and flames
Exposure to unspecified smoke, fire and flames
Exposure to unspecified smoke, fire and flames
Exposure to unspecified smoke, fire and flames
Exposure to unspecified smoke, fire and flames
Exposure to unspecified smoke, fire and flames
Exposure to unspecified smoke, fire and flames
Contact with hot tap-water
Contact with hot tap-water
Contact with hot tap-water
Contact with hot tap-water
Contact with other hot fluids
Contact with other hot fluids
Contact with other hot fluids
Contact with hot heating appliances, radiators and pipes
Contact with other and unspecified heat and hot substances
Contact with other and unspecified heat and hot substances
Contact with other and unspecified heat and hot substances

For peer review only

Contact with other and unspecified heat and hot substances
Contact with hornets, wasps and bees
Contact with hornets, wasps and bees
Contact with hornets, wasps and bees
Contact with hornets, wasps and bees
Contact with hornets, wasps and bees
Exposure to excessive natural heat
Exposure to excessive natural heat
Exposure to excessive natural heat
Exposure to excessive natural heat
Exposure to excessive natural heat
Exposure to excessive natural heat
Exposure to excessive natural cold
Exposure to excessive natural cold
Exposure to excessive natural cold
Exposure to excessive natural cold
Exposure to excessive natural cold
Exposure to excessive natural cold
Exposure to excessive natural cold
Exposure to excessive natural cold
Exposure to excessive natural cold
Exposure to excessive natural cold
Exposure to excessive natural cold
Exposure to excessive natural cold
Exposure to excessive natural cold
Exposure to excessive natural cold
Exposure to excessive natural cold
Exposure to excessive natural cold
Exposure to excessive natural cold
Exposure to excessive natural cold
Exposure to excessive natural cold
Exposure to excessive natural cold
Exposure to excessive natural cold
Victim of lightning
Victim of lightning
Victim of avalanche, landslide and other earth movements
Victim of avalanche, landslide and other earth movements
Victim of avalanche, landslide and other earth movements
Victim of avalanche, landslide and other earth movements
Accidental poisoning by and exposure to nonopioid analgesics, antipyretics and antirheumatics
Accidental poisoning by and exposure to nonopioid analgesics, antipyretics and antirheumatics
Accidental poisoning by and exposure to nonopioid analgesics, antipyretics and antirheumatics
Accidental poisoning by and exposure to nonopioid analgesics, antipyretics and antirheumatics
Accidental poisoning by and exposure to nonopioid analgesics, antipyretics and antirheumatics
Accidental poisoning by and exposure to nonopioid analgesics, antipyretics and antirheumatics
Accidental poisoning by and exposure to nonopioid analgesics, antipyretics and antirheumatics
Accidental poisoning by and exposure to antiepileptic, sedative-hypnotic, antiparkinsonism and psychotropic c
Accidental poisoning by and exposure to antiepileptic, sedative-hypnotic, antiparkinsonism and psychotropic c
Accidental poisoning by and exposure to antiepileptic, sedative-hypnotic, antiparkinsonism and psychotropic c
Accidental poisoning by and exposure to antiepileptic, sedative-hypnotic, antiparkinsonism and psychotropic c

For peer review only

Accidental poisoning by and exposure to antiepileptic, sedative-hypnotic, antiparkinsonism and psychotropic c
Accidental poisoning by and exposure to antiepileptic, sedative-hypnotic, antiparkinsonism and psychotropic c
Accidental poisoning by and exposure to antiepileptic, sedative-hypnotic, antiparkinsonism and psychotropic c
Accidental poisoning by and exposure to antiepileptic, sedative-hypnotic, antiparkinsonism and psychotropic c
Accidental poisoning by and exposure to antiepileptic, sedative-hypnotic, antiparkinsonism and psychotropic c
Accidental poisoning by and exposure to antiepileptic, sedative-hypnotic, antiparkinsonism and psychotropic c
Accidental poisoning by and exposure to antiepileptic, sedative-hypnotic, antiparkinsonism and psychotropic c
Accidental poisoning by and exposure to antiepileptic, sedative-hypnotic, antiparkinsonism and psychotropic c
Accidental poisoning by and exposure to antiepileptic, sedative-hypnotic, antiparkinsonism and psychotropic c
Accidental poisoning by and exposure to antiepileptic, sedative-hypnotic, antiparkinsonism and psychotropic c
Accidental poisoning by and exposure to antiepileptic, sedative-hypnotic, antiparkinsonism and psychotropic c
Accidental poisoning by and exposure to antiepileptic, sedative-hypnotic, antiparkinsonism and psychotropic c
Accidental poisoning by and exposure to antiepileptic, sedative-hypnotic, antiparkinsonism and psychotropic c
Accidental poisoning by and exposure to narcotics and psychodysleptics [hallucinogens], not elsewhere classifi
Accidental poisoning by and exposure to narcotics and psychodysleptics [hallucinogens], not elsewhere classifi
Accidental poisoning by and exposure to narcotics and psychodysleptics [hallucinogens], not elsewhere classifi
Accidental poisoning by and exposure to narcotics and psychodysleptics [hallucinogens], not elsewhere classifi
Accidental poisoning by and exposure to narcotics and psychodysleptics [hallucinogens], not elsewhere classifi
Accidental poisoning by and exposure to narcotics and psychodysleptics [hallucinogens], not elsewhere classifi
Accidental poisoning by and exposure to narcotics and psychodysleptics [hallucinogens], not elsewhere classifi
Accidental poisoning by and exposure to narcotics and psychodysleptics [hallucinogens], not elsewhere classifi
Accidental poisoning by and exposure to narcotics and psychodysleptics [hallucinogens], not elsewhere classifi
Accidental poisoning by and exposure to narcotics and psychodysleptics [hallucinogens], not elsewhere classifi
Accidental poisoning by and exposure to narcotics and psychodysleptics [hallucinogens], not elsewhere classifi
Accidental poisoning by and exposure to narcotics and psychodysleptics [hallucinogens], not elsewhere classifi
Accidental poisoning by and exposure to narcotics and psychodysleptics [hallucinogens], not elsewhere classifi
Accidental poisoning by and exposure to narcotics and psychodysleptics [hallucinogens], not elsewhere classifi
Accidental poisoning by and exposure to narcotics and psychodysleptics [hallucinogens], not elsewhere classifi
Accidental poisoning by and exposure to narcotics and psychodysleptics [hallucinogens], not elsewhere classifi
Accidental poisoning by and exposure to narcotics and psychodysleptics [hallucinogens], not elsewhere classifi
Accidental poisoning by and exposure to narcotics and psychodysleptics [hallucinogens], not elsewhere classifi
Accidental poisoning by and exposure to narcotics and psychodysleptics [hallucinogens], not elsewhere classifi
Accidental poisoning by and exposure to other drugs acting on the autonomic nervous system
Accidental poisoning by and exposure to other drugs acting on the autonomic nervous system
Accidental poisoning by and exposure to other drugs acting on the autonomic nervous system
Accidental poisoning by and exposure to other and unspecified drugs, medicaments and biological substances
Accidental poisoning by and exposure to other and unspecified drugs, medicaments and biological substances
Accidental poisoning by and exposure to other and unspecified drugs, medicaments and biological substances
Accidental poisoning by and exposure to other and unspecified drugs, medicaments and biological substances
Accidental poisoning by and exposure to other and unspecified drugs, medicaments and biological substances
Accidental poisoning by and exposure to other and unspecified drugs, medicaments and biological substances
Accidental poisoning by and exposure to other and unspecified drugs, medicaments and biological substances
Accidental poisoning by and exposure to other and unspecified drugs, medicaments and biological substances
Accidental poisoning by and exposure to other and unspecified drugs, medicaments and biological substances
Accidental poisoning by and exposure to other and unspecified drugs, medicaments and biological substances
Accidental poisoning by and exposure to other and unspecified drugs, medicaments and biological substances
Accidental poisoning by and exposure to other and unspecified drugs, medicaments and biological substances
Accidental poisoning by and exposure to other and unspecified drugs, medicaments and biological substances
Accidental poisoning by and exposure to other and unspecified drugs, medicaments and biological substances
Accidental poisoning by and exposure to other and unspecified drugs, medicaments and biological substances

For peer review only

Accidental poisoning by and exposure to other and unspecified drugs, medicaments and biological substances
Accidental poisoning by and exposure to other and unspecified drugs, medicaments and biological substances
Accidental poisoning by and exposure to other and unspecified drugs, medicaments and biological substances
Accidental poisoning by and exposure to other and unspecified drugs, medicaments and biological substances
Accidental poisoning by and exposure to alcohol
Accidental poisoning by and exposure to alcohol
Accidental poisoning by and exposure to alcohol
Accidental poisoning by and exposure to alcohol
Accidental poisoning by and exposure to alcohol
Accidental poisoning by and exposure to alcohol
Accidental poisoning by and exposure to alcohol
Accidental poisoning by and exposure to alcohol
Accidental poisoning by and exposure to alcohol
Accidental poisoning by and exposure to alcohol
Accidental poisoning by and exposure to alcohol
Accidental poisoning by and exposure to alcohol
Accidental poisoning by and exposure to alcohol
Accidental poisoning by and exposure to alcohol
Accidental poisoning by and exposure to alcohol
Accidental poisoning by and exposure to alcohol
Accidental poisoning by and exposure to alcohol
Accidental poisoning by and exposure to alcohol
Accidental poisoning by and exposure to alcohol
Accidental poisoning by and exposure to alcohol
Accidental poisoning by and exposure to organic solvents and halogenated hydrocarbons and their vapours
Accidental poisoning by and exposure to organic solvents and halogenated hydrocarbons and their vapours
Accidental poisoning by and exposure to organic solvents and halogenated hydrocarbons and their vapours
Accidental poisoning by and exposure to organic solvents and halogenated hydrocarbons and their vapours
Accidental poisoning by and exposure to organic solvents and halogenated hydrocarbons and their vapours
Accidental poisoning by and exposure to organic solvents and halogenated hydrocarbons and their vapours
Accidental poisoning by and exposure to organic solvents and halogenated hydrocarbons and their vapours
Accidental poisoning by and exposure to organic solvents and halogenated hydrocarbons and their vapours
Accidental poisoning by and exposure to other gases and vapours
Accidental poisoning by and exposure to other gases and vapours
Accidental poisoning by and exposure to other gases and vapours
Accidental poisoning by and exposure to other gases and vapours
Accidental poisoning by and exposure to other gases and vapours
Accidental poisoning by and exposure to other gases and vapours
Accidental poisoning by and exposure to other gases and vapours
Accidental poisoning by and exposure to other gases and vapours
Accidental poisoning by and exposure to other gases and vapours
Accidental poisoning by and exposure to other gases and vapours
Accidental poisoning by and exposure to other gases and vapours
Accidental poisoning by and exposure to other gases and vapours
Accidental poisoning by and exposure to other gases and vapours
Accidental poisoning by and exposure to other gases and vapours
Accidental poisoning by and exposure to other gases and vapours
Accidental poisoning by and exposure to other gases and vapours
Accidental poisoning by and exposure to other gases and vapours
Accidental poisoning by and exposure to other gases and vapours

For peer review only

Accidental poisoning by and exposure to other gases and vapours
Accidental poisoning by and exposure to pesticides
Accidental poisoning by and exposure to pesticides
Accidental poisoning by and exposure to other and unspecified chemicals and noxious substances
Accidental poisoning by and exposure to other and unspecified chemicals and noxious substances
Accidental poisoning by and exposure to other and unspecified chemicals and noxious substances
Accidental poisoning by and exposure to other and unspecified chemicals and noxious substances
Accidental poisoning by and exposure to other and unspecified chemicals and noxious substances
Accidental poisoning by and exposure to other and unspecified chemicals and noxious substances
Accidental poisoning by and exposure to other and unspecified chemicals and noxious substances
Accidental poisoning by and exposure to other and unspecified chemicals and noxious substances
Accidental poisoning by and exposure to other and unspecified chemicals and noxious substances
Accidental poisoning by and exposure to other and unspecified chemicals and noxious substances
Accidental poisoning by and exposure to other and unspecified chemicals and noxious substances
Accidental poisoning by and exposure to other and unspecified chemicals and noxious substances
Overexertion and strenuous or repetitive movements
Overexertion and strenuous or repetitive movements
Overexertion and strenuous or repetitive movements
Travel and motion
Lack of food
Exposure to other specified factors
Exposure to other specified factors
Exposure to unspecified factor
Exposure to unspecified factor
Exposure to unspecified factor
Exposure to unspecified factor
Exposure to unspecified factor
Exposure to unspecified factor
Exposure to unspecified factor
Exposure to unspecified factor
Exposure to unspecified factor
Exposure to unspecified factor
Exposure to unspecified factor
Exposure to unspecified factor
Exposure to unspecified factor
Exposure to unspecified factor
Exposure to unspecified factor
Exposure to unspecified factor
Exposure to unspecified factor
Exposure to unspecified factor
Exposure to unspecified factor
Exposure to unspecified factor
Exposure to unspecified factor
Intentional self-poisoning by and exposure to nonopioid analgesics, antipyretics and antirheumatics
Intentional self-poisoning by and exposure to nonopioid analgesics, antipyretics and antirheumatics
Intentional self-poisoning by and exposure to nonopioid analgesics, antipyretics and antirheumatics
Intentional self-poisoning by and exposure to nonopioid analgesics, antipyretics and antirheumatics
Intentional self-poisoning by and exposure to nonopioid analgesics, antipyretics and antirheumatics
Intentional self-poisoning by and exposure to nonopioid analgesics, antipyretics and antirheumatics
Intentional self-poisoning by and exposure to nonopioid analgesics, antipyretics and antirheumatics

For peer review only

Intentional self-poisoning by and exposure to nonopioid analgesics, antipyretics and antirheumatics
Intentional self-poisoning by and exposure to nonopioid analgesics, antipyretics and antirheumatics
Intentional self-poisoning by and exposure to nonopioid analgesics, antipyretics and antirheumatics
Intentional self-poisoning by and exposure to antiepileptic, sedative-hypnotic, antiparkinsonism and psychotro
Intentional self-poisoning by and exposure to antiepileptic, sedative-hypnotic, antiparkinsonism and psychotro
Intentional self-poisoning by and exposure to antiepileptic, sedative-hypnotic, antiparkinsonism and psychotro
Intentional self-poisoning by and exposure to antiepileptic, sedative-hypnotic, antiparkinsonism and psychotro
Intentional self-poisoning by and exposure to antiepileptic, sedative-hypnotic, antiparkinsonism and psychotro
Intentional self-poisoning by and exposure to antiepileptic, sedative-hypnotic, antiparkinsonism and psychotro
Intentional self-poisoning by and exposure to antiepileptic, sedative-hypnotic, antiparkinsonism and psychotro
Intentional self-poisoning by and exposure to antiepileptic, sedative-hypnotic, antiparkinsonism and psychotro
Intentional self-poisoning by and exposure to antiepileptic, sedative-hypnotic, antiparkinsonism and psychotro
Intentional self-poisoning by and exposure to antiepileptic, sedative-hypnotic, antiparkinsonism and psychotro
Intentional self-poisoning by and exposure to antiepileptic, sedative-hypnotic, antiparkinsonism and psychotro
Intentional self-poisoning by and exposure to antiepileptic, sedative-hypnotic, antiparkinsonism and psychotro
Intentional self-poisoning by and exposure to antiepileptic, sedative-hypnotic, antiparkinsonism and psychotro
Intentional self-poisoning by and exposure to antiepileptic, sedative-hypnotic, antiparkinsonism and psychotro
Intentional self-poisoning by and exposure to antiepileptic, sedative-hypnotic, antiparkinsonism and psychotro
Intentional self-poisoning by and exposure to antiepileptic, sedative-hypnotic, antiparkinsonism and psychotro
Intentional self-poisoning by and exposure to antiepileptic, sedative-hypnotic, antiparkinsonism and psychotro
Intentional self-poisoning by and exposure to antiepileptic, sedative-hypnotic, antiparkinsonism and psychotro
Intentional self-poisoning by and exposure to antiepileptic, sedative-hypnotic, antiparkinsonism and psychotro
Intentional self-poisoning by and exposure to narcotics and psychodysleptics [hallucinogens], not elsewhere cl
Intentional self-poisoning by and exposure to narcotics and psychodysleptics [hallucinogens], not elsewhere cl
Intentional self-poisoning by and exposure to narcotics and psychodysleptics [hallucinogens], not elsewhere cl
Intentional self-poisoning by and exposure to narcotics and psychodysleptics [hallucinogens], not elsewhere cl
Intentional self-poisoning by and exposure to narcotics and psychodysleptics [hallucinogens], not elsewhere cl
Intentional self-poisoning by and exposure to narcotics and psychodysleptics [hallucinogens], not elsewhere cl
Intentional self-poisoning by and exposure to narcotics and psychodysleptics [hallucinogens], not elsewhere cl
Intentional self-poisoning by and exposure to narcotics and psychodysleptics [hallucinogens], not elsewhere cl
Intentional self-poisoning by and exposure to narcotics and psychodysleptics [hallucinogens], not elsewhere cl
Intentional self-poisoning by and exposure to narcotics and psychodysleptics [hallucinogens], not elsewhere cl
Intentional self-poisoning by and exposure to narcotics and psychodysleptics [hallucinogens], not elsewhere cl
Intentional self-poisoning by and exposure to narcotics and psychodysleptics [hallucinogens], not elsewhere cl
Intentional self-poisoning by and exposure to narcotics and psychodysleptics [hallucinogens], not elsewhere cl
Intentional self-poisoning by and exposure to narcotics and psychodysleptics [hallucinogens], not elsewhere cl
Intentional self-poisoning by and exposure to narcotics and psychodysleptics [hallucinogens], not elsewhere cl
Intentional self-poisoning by and exposure to other drugs acting on the autonomic nervous system
Intentional self-poisoning by and exposure to other drugs acting on the autonomic nervous system
Intentional self-poisoning by and exposure to other drugs acting on the autonomic nervous system
Intentional self-poisoning by and exposure to other drugs acting on the autonomic nervous system
Intentional self-poisoning by and exposure to other drugs acting on the autonomic nervous system
Intentional self-poisoning by and exposure to other and unspecified drugs, medicaments and biological substa
Intentional self-poisoning by and exposure to other and unspecified drugs, medicaments and biological substa
Intentional self-poisoning by and exposure to other and unspecified drugs, medicaments and biological substa
Intentional self-poisoning by and exposure to other and unspecified drugs, medicaments and biological substa
Intentional self-poisoning by and exposure to other and unspecified drugs, medicaments and biological substa
Intentional self-poisoning by and exposure to other and unspecified drugs, medicaments and biological substa
Intentional self-poisoning by and exposure to other and unspecified drugs, medicaments and biological substa
Intentional self-poisoning by and exposure to other and unspecified drugs, medicaments and biological substa

For peer review only

Intentional self-poisoning by and exposure to other and unspecified drugs, medicaments and biological substa
Intentional self-poisoning by and exposure to other and unspecified drugs, medicaments and biological substa
Intentional self-poisoning by and exposure to other and unspecified drugs, medicaments and biological substa
Intentional self-poisoning by and exposure to other and unspecified drugs, medicaments and biological substa
Intentional self-poisoning by and exposure to other and unspecified drugs, medicaments and biological substa
Intentional self-poisoning by and exposure to other and unspecified drugs, medicaments and biological substa
Intentional self-poisoning by and exposure to other and unspecified drugs, medicaments and biological substa
Intentional self-poisoning by and exposure to other and unspecified drugs, medicaments and biological substa
Intentional self-poisoning by and exposure to other and unspecified drugs, medicaments and biological substa
Intentional self-poisoning by and exposure to other and unspecified drugs, medicaments and biological substa
Intentional self-poisoning by and exposure to alcohol
Intentional self-poisoning by and exposure to alcohol
Intentional self-poisoning by and exposure to alcohol
Intentional self-poisoning by and exposure to alcohol
Intentional self-poisoning by and exposure to alcohol
Intentional self-poisoning by and exposure to organic solvents and halogenated hydrocarbons and their vapou
Intentional self-poisoning by and exposure to organic solvents and halogenated hydrocarbons and their vapou
Intentional self-poisoning by and exposure to other gases and vapours
Intentional self-poisoning by and exposure to other gases and vapours
Intentional self-poisoning by and exposure to other gases and vapours
Intentional self-poisoning by and exposure to other gases and vapours
Intentional self-poisoning by and exposure to other gases and vapours
Intentional self-poisoning by and exposure to other gases and vapours
Intentional self-poisoning by and exposure to other gases and vapours
Intentional self-poisoning by and exposure to other gases and vapours
Intentional self-poisoning by and exposure to other gases and vapours
Intentional self-poisoning by and exposure to other gases and vapours
Intentional self-poisoning by and exposure to other gases and vapours
Intentional self-poisoning by and exposure to other gases and vapours
Intentional self-poisoning by and exposure to other gases and vapours
Intentional self-poisoning by and exposure to other gases and vapours
Intentional self-poisoning by and exposure to other gases and vapours
Intentional self-poisoning by and exposure to other gases and vapours
Intentional self-poisoning by and exposure to other gases and vapours
Intentional self-poisoning by and exposure to other gases and vapours
Intentional self-poisoning by and exposure to pesticides
Intentional self-poisoning by and exposure to pesticides
Intentional self-poisoning by and exposure to pesticides
Intentional self-poisoning by and exposure to pesticides
Intentional self-poisoning by and exposure to pesticides
Intentional self-poisoning by and exposure to pesticides
Intentional self-poisoning by and exposure to other and unspecified chemicals and noxious substances
Intentional self-poisoning by and exposure to other and unspecified chemicals and noxious substances
Intentional self-poisoning by and exposure to other and unspecified chemicals and noxious substances
Intentional self-poisoning by and exposure to other and unspecified chemicals and noxious substances
Intentional self-poisoning by and exposure to other and unspecified chemicals and noxious substances
Intentional self-poisoning by and exposure to other and unspecified chemicals and noxious substances
Intentional self-poisoning by and exposure to other and unspecified chemicals and noxious substances
Intentional self-poisoning by and exposure to other and unspecified chemicals and noxious substances
Intentional self-poisoning by and exposure to other and unspecified chemicals and noxious substances

For peer review only

Intentional self-poisoning by and exposure to other and unspecified chemicals and noxious substances
Intentional self-poisoning by and exposure to other and unspecified chemicals and noxious substances
Intentional self-poisoning by and exposure to other and unspecified chemicals and noxious substances
Intentional self-poisoning by and exposure to other and unspecified chemicals and noxious substances
Intentional self-harm by hanging, strangulation and suffocation
Intentional self-harm by hanging, strangulation and suffocation
Intentional self-harm by hanging, strangulation and suffocation
Intentional self-harm by hanging, strangulation and suffocation
Intentional self-harm by hanging, strangulation and suffocation
Intentional self-harm by hanging, strangulation and suffocation
Intentional self-harm by hanging, strangulation and suffocation
Intentional self-harm by hanging, strangulation and suffocation
Intentional self-harm by hanging, strangulation and suffocation
Intentional self-harm by hanging, strangulation and suffocation
Intentional self-harm by hanging, strangulation and suffocation
Intentional self-harm by hanging, strangulation and suffocation
Intentional self-harm by hanging, strangulation and suffocation
Intentional self-harm by hanging, strangulation and suffocation
Intentional self-harm by hanging, strangulation and suffocation
Intentional self-harm by hanging, strangulation and suffocation
Intentional self-harm by hanging, strangulation and suffocation
Intentional self-harm by hanging, strangulation and suffocation
Intentional self-harm by hanging, strangulation and suffocation
Intentional self-harm by hanging, strangulation and suffocation
Intentional self-harm by hanging, strangulation and suffocation
Intentional self-harm by drowning and submersion
Intentional self-harm by drowning and submersion
Intentional self-harm by drowning and submersion
Intentional self-harm by drowning and submersion
Intentional self-harm by drowning and submersion
Intentional self-harm by drowning and submersion
Intentional self-harm by drowning and submersion
Intentional self-harm by drowning and submersion
Intentional self-harm by drowning and submersion
Intentional self-harm by drowning and submersion
Intentional self-harm by drowning and submersion
Intentional self-harm by drowning and submersion
Intentional self-harm by drowning and submersion
Intentional self-harm by drowning and submersion
Intentional self-harm by drowning and submersion
Intentional self-harm by drowning and submersion
Intentional self-harm by drowning and submersion
Intentional self-harm by drowning and submersion
Intentional self-harm by drowning and submersion
Intentional self-harm by drowning and submersion
Intentional self-harm by handgun discharge
Intentional self-harm by handgun discharge
Intentional self-harm by handgun discharge
Intentional self-harm by handgun discharge
Intentional self-harm by handgun discharge

For peer review only

Intentional self-harm by handgun discharge
Intentional self-harm by handgun discharge
Intentional self-harm by handgun discharge
Intentional self-harm by handgun discharge
Intentional self-harm by handgun discharge
Intentional self-harm by handgun discharge
Intentional self-harm by handgun discharge
Intentional self-harm by handgun discharge
Intentional self-harm by handgun discharge
Intentional self-harm by handgun discharge
Intentional self-harm by handgun discharge
Intentional self-harm by handgun discharge
Intentional self-harm by rifle, shotgun and larger firearm discharge
Intentional self-harm by rifle, shotgun and larger firearm discharge
Intentional self-harm by rifle, shotgun and larger firearm discharge
Intentional self-harm by rifle, shotgun and larger firearm discharge
Intentional self-harm by rifle, shotgun and larger firearm discharge
Intentional self-harm by rifle, shotgun and larger firearm discharge
Intentional self-harm by rifle, shotgun and larger firearm discharge
Intentional self-harm by rifle, shotgun and larger firearm discharge
Intentional self-harm by rifle, shotgun and larger firearm discharge
Intentional self-harm by rifle, shotgun and larger firearm discharge
Intentional self-harm by rifle, shotgun and larger firearm discharge
Intentional self-harm by rifle, shotgun and larger firearm discharge
Intentional self-harm by rifle, shotgun and larger firearm discharge
Intentional self-harm by rifle, shotgun and larger firearm discharge
Intentional self-harm by rifle, shotgun and larger firearm discharge
Intentional self-harm by rifle, shotgun and larger firearm discharge
Intentional self-harm by rifle, shotgun and larger firearm discharge
Intentional self-harm by rifle, shotgun and larger firearm discharge
Intentional self-harm by other and unspecified firearm discharge
Intentional self-harm by other and unspecified firearm discharge
Intentional self-harm by other and unspecified firearm discharge
Intentional self-harm by other and unspecified firearm discharge
Intentional self-harm by other and unspecified firearm discharge
Intentional self-harm by other and unspecified firearm discharge
Intentional self-harm by other and unspecified firearm discharge
Intentional self-harm by other and unspecified firearm discharge
Intentional self-harm by other and unspecified firearm discharge
Intentional self-harm by other and unspecified firearm discharge
Intentional self-harm by other and unspecified firearm discharge
Intentional self-harm by other and unspecified firearm discharge
Intentional self-harm by other and unspecified firearm discharge
Intentional self-harm by other and unspecified firearm discharge
Intentional self-harm by other and unspecified firearm discharge
Intentional self-harm by other and unspecified firearm discharge
Intentional self-harm by other and unspecified firearm discharge
Intentional self-harm by other and unspecified firearm discharge
Intentional self-harm by other and unspecified firearm discharge
Intentional self-harm by other and unspecified firearm discharge

For peer review only

For peer review only - http://bmjopen.bmj.com/site/about/guidelines.xhtml

Intentional self-harm by other and unspecified firearm discharge
Intentional self-harm by explosive material
Intentional self-harm by smoke, fire and flames
Intentional self-harm by smoke, fire and flames
Intentional self-harm by smoke, fire and flames
Intentional self-harm by smoke, fire and flames
Intentional self-harm by smoke, fire and flames
Intentional self-harm by smoke, fire and flames
Intentional self-harm by smoke, fire and flames
Intentional self-harm by smoke, fire and flames
Intentional self-harm by smoke, fire and flames
Intentional self-harm by smoke, fire and flames
Intentional self-harm by smoke, fire and flames
Intentional self-harm by smoke, fire and flames
Intentional self-harm by smoke, fire and flames
Intentional self-harm by smoke, fire and flames
Intentional self-harm by smoke, fire and flames
Intentional self-harm by sharp object
Intentional self-harm by sharp object
Intentional self-harm by sharp object
Intentional self-harm by sharp object
Intentional self-harm by sharp object
Intentional self-harm by sharp object
Intentional self-harm by sharp object
Intentional self-harm by sharp object
Intentional self-harm by sharp object
Intentional self-harm by sharp object
Intentional self-harm by sharp object
Intentional self-harm by sharp object
Intentional self-harm by sharp object
Intentional self-harm by sharp object
Intentional self-harm by sharp object
Intentional self-harm by sharp object
Intentional self-harm by sharp object
Intentional self-harm by sharp object
Intentional self-harm by sharp object
Intentional self-harm by sharp object
Intentional self-harm by sharp object
Intentional self-harm by jumping from a high place
Intentional self-harm by jumping from a high place
Intentional self-harm by jumping from a high place
Intentional self-harm by jumping from a high place
Intentional self-harm by jumping from a high place
Intentional self-harm by jumping from a high place
Intentional self-harm by jumping from a high place
Intentional self-harm by jumping from a high place
Intentional self-harm by jumping from a high place
Intentional self-harm by jumping from a high place
Intentional self-harm by jumping from a high place
Intentional self-harm by jumping from a high place

For peer review only

Intentional self-harm by jumping from a high place
Intentional self-harm by jumping from a high place
Intentional self-harm by jumping from a high place
Intentional self-harm by jumping from a high place
Intentional self-harm by jumping from a high place
Intentional self-harm by jumping from a high place
Intentional self-harm by jumping from a high place
Intentional self-harm by jumping from a high place
Intentional self-harm by jumping from a high place
Intentional self-harm by jumping or lying before moving object
Intentional self-harm by jumping or lying before moving object
Intentional self-harm by jumping or lying before moving object
Intentional self-harm by jumping or lying before moving object
Intentional self-harm by jumping or lying before moving object
Intentional self-harm by jumping or lying before moving object
Intentional self-harm by jumping or lying before moving object
Intentional self-harm by jumping or lying before moving object
Intentional self-harm by jumping or lying before moving object
Intentional self-harm by jumping or lying before moving object
Intentional self-harm by jumping or lying before moving object
Intentional self-harm by jumping or lying before moving object
Intentional self-harm by jumping or lying before moving object
Intentional self-harm by jumping or lying before moving object
Intentional self-harm by jumping or lying before moving object
Intentional self-harm by jumping or lying before moving object
Intentional self-harm by jumping or lying before moving object
Intentional self-harm by crashing of motor vehicle
Intentional self-harm by crashing of motor vehicle
Intentional self-harm by crashing of motor vehicle
Intentional self-harm by crashing of motor vehicle
Intentional self-harm by crashing of motor vehicle
Intentional self-harm by crashing of motor vehicle
Intentional self-harm by crashing of motor vehicle
Intentional self-harm by crashing of motor vehicle
Intentional self-harm by crashing of motor vehicle
Intentional self-harm by crashing of motor vehicle
Intentional self-harm by crashing of motor vehicle
Intentional self-harm by other specified means
Intentional self-harm by other specified means
Intentional self-harm by other specified means
Intentional self-harm by other specified means
Intentional self-harm by other specified means
Intentional self-harm by other specified means
Intentional self-harm by other specified means
Intentional self-harm by other specified means
Intentional self-harm by other specified means
Intentional self-harm by other specified means
Intentional self-harm by other specified means
Intentional self-harm by unspecified means
Intentional self-harm by unspecified means

For peer review only

Intentional self-harm by unspecified means
Intentional self-harm by unspecified means
Intentional self-harm by unspecified means
Intentional self-harm by unspecified means
Intentional self-harm by unspecified means
Intentional self-harm by unspecified means
Intentional self-harm by unspecified means
Intentional self-harm by unspecified means
Intentional self-harm by unspecified means
Intentional self-harm by unspecified means
Intentional self-harm by unspecified means
Assault by hanging, strangulation and suffocation
Assault by hanging, strangulation and suffocation
Assault by hanging, strangulation and suffocation
Assault by hanging, strangulation and suffocation
Assault by hanging, strangulation and suffocation
Assault by hanging, strangulation and suffocation
Assault by hanging, strangulation and suffocation
Assault by hanging, strangulation and suffocation
Assault by hanging, strangulation and suffocation
Assault by hanging, strangulation and suffocation
Assault by hanging, strangulation and suffocation
Assault by hanging, strangulation and suffocation
Assault by hanging, strangulation and suffocation
Assault by hanging, strangulation and suffocation
Assault by hanging, strangulation and suffocation
Assault by hanging, strangulation and suffocation
Assault by hanging, strangulation and suffocation
Assault by hanging, strangulation and suffocation
Assault by drowning and submersion
Assault by handgun discharge
Assault by handgun discharge
Assault by handgun discharge
Assault by handgun discharge
Assault by handgun discharge
Assault by handgun discharge
Assault by rifle, shotgun and larger firearm discharge
Assault by rifle, shotgun and larger firearm discharge
Assault by rifle, shotgun and larger firearm discharge
Assault by rifle, shotgun and larger firearm discharge
Assault by other and unspecified firearm discharge
Assault by other and unspecified firearm discharge
Assault by other and unspecified firearm discharge
Assault by other and unspecified firearm discharge
Assault by other and unspecified firearm discharge
Assault by other and unspecified firearm discharge
Assault by other and unspecified firearm discharge
Assault by other and unspecified firearm discharge
Assault by other and unspecified firearm discharge
Assault by other and unspecified firearm discharge

For peer review only

Assault by other and unspecified firearm discharge
Assault by other and unspecified firearm discharge
Assault by other and unspecified firearm discharge
Assault by other and unspecified firearm discharge
Assault by other and unspecified firearm discharge
Assault by other and unspecified firearm discharge
Assault by other and unspecified firearm discharge
Assault by fire and flames
Assault by sharp object
Assault by sharp object
Assault by sharp object
Assault by sharp object
Assault by sharp object
Assault by sharp object
Assault by sharp object
Assault by sharp object
Assault by sharp object
Assault by sharp object
Assault by sharp object
Assault by sharp object
Assault by sharp object
Assault by sharp object
Assault by sharp object
Assault by sharp object
Assault by sharp object
Assault by sharp object
Assault by sharp object
Assault by sharp object
Assault by sharp object
Assault by blunt object
Assault by blunt object
Assault by blunt object
Assault by blunt object
Assault by blunt object
Assault by blunt object
Assault by blunt object
Assault by blunt object
Assault by blunt object
Assault by blunt object
Assault by blunt object
Assault by blunt object
Assault by blunt object
Assault by blunt object
Assault by blunt object
Assault by bodily force
Assault by bodily force
Assault by bodily force
Assault by bodily force
Assault by bodily force
Assault by bodily force

For peer review only

Assault by bodily force
Assault by bodily force
Assault by bodily force
Assault by bodily force
Assault by bodily force
Assault by bodily force
Assault by bodily force
Assault by bodily force
Assault by bodily force
Assault by bodily force
Neglect and abandonment
Other maltreatment syndromes
Other maltreatment syndromes
Other maltreatment syndromes
Other maltreatment syndromes
Assault by other specified means
Assault by unspecified means
Assault by unspecified means
Assault by unspecified means
Assault by unspecified means
Assault by unspecified means
Assault by unspecified means
Assault by unspecified means
Assault by unspecified means
Assault by unspecified means
Assault by unspecified means
Assault by unspecified means
Assault by unspecified means
Assault by unspecified means
Poisoning by and exposure to nonopioid analgesics, antipyretics and antirheumatics, undetermined intent
Poisoning by and exposure to nonopioid analgesics, antipyretics and antirheumatics, undetermined intent
Poisoning by and exposure to antiepileptic, sedative-hypnotic, antiparkinsonism and psychotropic drugs, not e
Poisoning by and exposure to antiepileptic, sedative-hypnotic, antiparkinsonism and psychotropic drugs, not e
Poisoning by and exposure to antiepileptic, sedative-hypnotic, antiparkinsonism and psychotropic drugs, not e
Poisoning by and exposure to antiepileptic, sedative-hypnotic, antiparkinsonism and psychotropic drugs, not e
Poisoning by and exposure to antiepileptic, sedative-hypnotic, antiparkinsonism and psychotropic drugs, not e
Poisoning by and exposure to antiepileptic, sedative-hypnotic, antiparkinsonism and psychotropic drugs, not e
Poisoning by and exposure to antiepileptic, sedative-hypnotic, antiparkinsonism and psychotropic drugs, not e
Poisoning by and exposure to antiepileptic, sedative-hypnotic, antiparkinsonism and psychotropic drugs, not e
Poisoning by and exposure to antiepileptic, sedative-hypnotic, antiparkinsonism and psychotropic drugs, not e
Poisoning by and exposure to narcotics and psychodysleptics [hallucinogens], not elsewhere classified, undete
Poisoning by and exposure to narcotics and psychodysleptics [hallucinogens], not elsewhere classified, undete
Poisoning by and exposure to narcotics and psychodysleptics [hallucinogens], not elsewhere classified, undete
Poisoning by and exposure to narcotics and psychodysleptics [hallucinogens], not elsewhere classified, undete
Poisoning by and exposure to narcotics and psychodysleptics [hallucinogens], not elsewhere classified, undete
Poisoning by and exposure to narcotics and psychodysleptics [hallucinogens], not elsewhere classified, undete
Poisoning by and exposure to narcotics and psychodysleptics [hallucinogens], not elsewhere classified, undete
Poisoning by and exposure to other drugs acting on the autonomic nervous system, undetermined intent
Poisoning by and exposure to other and unspecified drugs, medicaments and biological substances, undeterm
Poisoning by and exposure to other and unspecified drugs, medicaments and biological substances, undeterm

For peer review only

Poisoning by and exposure to other and unspecified drugs, medicaments and biological substances, undeterm
Poisoning by and exposure to other and unspecified drugs, medicaments and biological substances, undeterm
Poisoning by and exposure to other and unspecified drugs, medicaments and biological substances, undeterm
Poisoning by and exposure to other and unspecified drugs, medicaments and biological substances, undeterm
Poisoning by and exposure to other and unspecified drugs, medicaments and biological substances, undeterm
Poisoning by and exposure to other and unspecified drugs, medicaments and biological substances, undeterm
Poisoning by and exposure to other and unspecified drugs, medicaments and biological substances, undeterm
Poisoning by and exposure to other and unspecified drugs, medicaments and biological substances, undeterm
Poisoning by and exposure to other and unspecified drugs, medicaments and biological substances, undeterm
Poisoning by and exposure to other and unspecified drugs, medicaments and biological substances, undeterm
Poisoning by and exposure to other and unspecified drugs, medicaments and biological substances, undeterm
Poisoning by and exposure to other and unspecified drugs, medicaments and biological substances, undeterm
Poisoning by and exposure to other and unspecified drugs, medicaments and biological substances, undeterm
Poisoning by and exposure to alcohol, undetermined intent
Poisoning by and exposure to alcohol, undetermined intent
Poisoning by and exposure to alcohol, undetermined intent
Poisoning by and exposure to alcohol, undetermined intent
Poisoning by and exposure to alcohol, undetermined intent
Poisoning by and exposure to alcohol, undetermined intent
Poisoning by and exposure to alcohol, undetermined intent
Poisoning by and exposure to organic solvents and halogenated hydrocarbons and their vapours, undetermine
Poisoning by and exposure to organic solvents and halogenated hydrocarbons and their vapours, undetermine
Poisoning by and exposure to other gases and vapours, undetermined intent
Poisoning by and exposure to other gases and vapours, undetermined intent
Poisoning by and exposure to other gases and vapours, undetermined intent
Poisoning by and exposure to other gases and vapours, undetermined intent
Poisoning by and exposure to other gases and vapours, undetermined intent
Poisoning by and exposure to other gases and vapours, undetermined intent
Poisoning by and exposure to other gases and vapours, undetermined intent
Poisoning by and exposure to other gases and vapours, undetermined intent
Poisoning by and exposure to other and unspecified chemicals and noxious substances, undetermined intent
Poisoning by and exposure to other and unspecified chemicals and noxious substances, undetermined intent
Poisoning by and exposure to other and unspecified chemicals and noxious substances, undetermined intent
Poisoning by and exposure to other and unspecified chemicals and noxious substances, undetermined intent
Poisoning by and exposure to other and unspecified chemicals and noxious substances, undetermined intent
Poisoning by and exposure to other and unspecified chemicals and noxious substances, undetermined intent
Hanging, strangulation and suffocation, undetermined intent
Hanging, strangulation and suffocation, undetermined intent
Hanging, strangulation and suffocation, undetermined intent
Hanging, strangulation and suffocation, undetermined intent
Hanging, strangulation and suffocation, undetermined intent
Hanging, strangulation and suffocation, undetermined intent
Hanging, strangulation and suffocation, undetermined intent
Hanging, strangulation and suffocation, undetermined intent
Hanging, strangulation and suffocation, undetermined intent
Hanging, strangulation and suffocation, undetermined intent
Drowning and submersion, undetermined intent
Drowning and submersion, undetermined intent
Drowning and submersion, undetermined intent
Drowning and submersion, undetermined intent

For peer review only

Drowning and submersion, undetermined intent
Drowning and submersion, undetermined intent
Drowning and submersion, undetermined intent
Drowning and submersion, undetermined intent
Drowning and submersion, undetermined intent
Drowning and submersion, undetermined intent
Drowning and submersion, undetermined intent
Drowning and submersion, undetermined intent
Drowning and submersion, undetermined intent
Drowning and submersion, undetermined intent
Drowning and submersion, undetermined intent
Drowning and submersion, undetermined intent
Drowning and submersion, undetermined intent
Drowning and submersion, undetermined intent
Handgun discharge, undetermined intent
Handgun discharge, undetermined intent
Rifle, shotgun and larger firearm discharge, undetermined intent
Rifle, shotgun and larger firearm discharge, undetermined intent
Other and unspecified firearm discharge, undetermined intent
Other and unspecified firearm discharge, undetermined intent
Other and unspecified firearm discharge, undetermined intent
Other and unspecified firearm discharge, undetermined intent
Other and unspecified firearm discharge, undetermined intent
Other and unspecified firearm discharge, undetermined intent
Other and unspecified firearm discharge, undetermined intent
Other and unspecified firearm discharge, undetermined intent
Other and unspecified firearm discharge, undetermined intent
Exposure to smoke, fire and flames, undetermined intent
Exposure to smoke, fire and flames, undetermined intent
Exposure to smoke, fire and flames, undetermined intent
Exposure to smoke, fire and flames, undetermined intent
Exposure to smoke, fire and flames, undetermined intent
Exposure to smoke, fire and flames, undetermined intent
Exposure to smoke, fire and flames, undetermined intent
Exposure to smoke, fire and flames, undetermined intent
Exposure to smoke, fire and flames, undetermined intent
Contact with sharp object, undetermined intent
Contact with sharp object, undetermined intent
Contact with sharp object, undetermined intent
Contact with sharp object, undetermined intent
Contact with sharp object, undetermined intent
Contact with sharp object, undetermined intent
Contact with sharp object, undetermined intent
Contact with sharp object, undetermined intent
Contact with blunt object, undetermined intent
Contact with blunt object, undetermined intent
Contact with blunt object, undetermined intent
Contact with blunt object, undetermined intent
Contact with blunt object, undetermined intent
Falling, jumping or pushed from a high place, undetermined intent

For peer review only

Falling, jumping or pushed from a high place, undetermined intent
Falling, jumping or pushed from a high place, undetermined intent
Falling, jumping or pushed from a high place, undetermined intent
Falling, jumping or pushed from a high place, undetermined intent
Falling, jumping or pushed from a high place, undetermined intent
Falling, jumping or pushed from a high place, undetermined intent
Falling, jumping or pushed from a high place, undetermined intent
Falling, jumping or pushed from a high place, undetermined intent
Falling, jumping or pushed from a high place, undetermined intent
Falling, jumping or pushed from a high place, undetermined intent
Falling, jumping or pushed from a high place, undetermined intent
Falling, jumping or pushed from a high place, undetermined intent
Falling, jumping or pushed from a high place, undetermined intent
Falling, jumping or pushed from a high place, undetermined intent
Falling, jumping or pushed from a high place, undetermined intent
Falling, lying or running before or into moving object, undetermined intent
Falling, lying or running before or into moving object, undetermined intent
Falling, lying or running before or into moving object, undetermined intent
Falling, lying or running before or into moving object, undetermined intent
Falling, lying or running before or into moving object, undetermined intent
Falling, lying or running before or into moving object, undetermined intent
Falling, lying or running before or into moving object, undetermined intent
Crashing of motor vehicle, undetermined intent
Crashing of motor vehicle, undetermined intent
Other specified events, undetermined intent
Other specified events, undetermined intent
Other specified events, undetermined intent
Other specified events, undetermined intent
Other specified events, undetermined intent
Other specified events, undetermined intent
Other specified events, undetermined intent
Other specified events, undetermined intent
Unspecified event, undetermined intent
Unspecified event, undetermined intent
Unspecified event, undetermined intent
Unspecified event, undetermined intent
Unspecified event, undetermined intent
Unspecified event, undetermined intent
Unspecified event, undetermined intent
Unspecified event, undetermined intent
Unspecified event, undetermined intent
Unspecified event, undetermined intent
Unspecified event, undetermined intent
Unspecified event, undetermined intent
Unspecified event, undetermined intent
Unspecified event, undetermined intent
Unspecified event, undetermined intent
Unspecified event, undetermined intent
Unspecified event, undetermined intent
Unspecified event, undetermined intent

For peer review only

Unspecified event, undetermined intent
Systemic antibiotics
Systemic antibiotics
Systemic antibiotics
Hormones, and their synthetic substitutes and antagonists, not elsewhere classified
Primarily systemic agents
Agents primarily affecting blood constituents
Agents primarily affecting blood constituents
Agents primarily affecting blood constituents
Agents primarily affecting blood constituents
Agents primarily affecting blood constituents
Agents primarily affecting blood constituents
Agents primarily affecting blood constituents
Analgesics, antipyretics and anti-inflammatory drugs
Analgesics, antipyretics and anti-inflammatory drugs
Analgesics, antipyretics and anti-inflammatory drugs
Analgesics, antipyretics and anti-inflammatory drugs
Anaesthetics and therapeutic gases
Psychotropic drugs, not elsewhere classified
Agents primarily affecting the cardiovascular system
Agents primarily affecting the cardiovascular system
Agents primarily affecting water-balance and mineral and uric acid metabolism
Other and unspecified drugs and medicaments
Other and unspecified drugs and medicaments
Other and unspecified drugs and medicaments
Other and unspecified drugs and medicaments
Other and unspecified drugs and medicaments
Unintentional cut, puncture, perforation or haemorrhage during surgical and medical care
Unintentional cut, puncture, perforation or haemorrhage during surgical and medical care
Unintentional cut, puncture, perforation or haemorrhage during surgical and medical care
Unintentional cut, puncture, perforation or haemorrhage during surgical and medical care
Unintentional cut, puncture, perforation or haemorrhage during surgical and medical care
Unintentional cut, puncture, perforation or haemorrhage during surgical and medical care
Unintentional cut, puncture, perforation or haemorrhage during surgical and medical care
Unintentional cut, puncture, perforation or haemorrhage during surgical and medical care
Failure of sterile precautions during surgical and medical care
Other misadventures during surgical and medical care
Other misadventures during surgical and medical care
Other misadventures during surgical and medical care
Unspecified misadventure during surgical and medical care
Unspecified misadventure during surgical and medical care
Cardiovascular devices associated with adverse incidents
Cardiovascular devices associated with adverse incidents
Gastroenterology and urology devices associated with adverse incidents
Surgical operation and other surgical procedures as the cause of abnormal reaction of the patient, or of later c
Surgical operation and other surgical procedures as the cause of abnormal reaction of the patient, or of later c
Surgical operation and other surgical procedures as the cause of abnormal reaction of the patient, or of later c
Surgical operation and other surgical procedures as the cause of abnormal reaction of the patient, or of later c
Surgical operation and other surgical procedures as the cause of abnormal reaction of the patient, or of later c
Surgical operation and other surgical procedures as the cause of abnormal reaction of the patient, or of later c

For peer review only

Surgical operation and other surgical procedures as the cause of abnormal reaction of the patient, or of later c
Surgical operation and other surgical procedures as the cause of abnormal reaction of the patient, or of later c
Surgical operation and other surgical procedures as the cause of abnormal reaction of the patient, or of later c
Surgical operation and other surgical procedures as the cause of abnormal reaction of the patient, or of later c
Surgical operation and other surgical procedures as the cause of abnormal reaction of the patient, or of later c
Surgical operation and other surgical procedures as the cause of abnormal reaction of the patient, or of later c
Surgical operation and other surgical procedures as the cause of abnormal reaction of the patient, or of later c
Surgical operation and other surgical procedures as the cause of abnormal reaction of the patient, or of later c
Surgical operation and other surgical procedures as the cause of abnormal reaction of the patient, or of later c
Surgical operation and other surgical procedures as the cause of abnormal reaction of the patient, or of later c
Surgical operation and other surgical procedures as the cause of abnormal reaction of the patient, or of later c
Other medical procedures as the cause of abnormal reaction of the patient, or of later complication, without n
Other medical procedures as the cause of abnormal reaction of the patient, or of later complication, without n
Other medical procedures as the cause of abnormal reaction of the patient, or of later complication, without n
Other medical procedures as the cause of abnormal reaction of the patient, or of later complication, without n
Other medical procedures as the cause of abnormal reaction of the patient, or of later complication, without n
Other medical procedures as the cause of abnormal reaction of the patient, or of later complication, without n
Other medical procedures as the cause of abnormal reaction of the patient, or of later complication, without n
Other medical procedures as the cause of abnormal reaction of the patient, or of later complication, without n
Other medical procedures as the cause of abnormal reaction of the patient, or of later complication, without n
Sequelae of transport accidents
Sequelae of transport accidents
Sequelae of transport accidents
Sequelae of transport accidents
Sequelae of transport accidents
Sequelae of transport accidents
Sequelae of transport accidents
Sequelae of transport accidents
Sequelae of transport accidents
Sequelae of transport accidents
Sequelae of other accidents
Sequelae of other accidents
Sequelae of other accidents
Sequelae of other accidents
Sequelae of other accidents
Sequelae of other accidents
Sequelae of other accidents
Sequelae of other accidents
Sequelae of other accidents
Sequelae of other accidents
Sequelae of other accidents
Sequelae of other accidents
Sequelae of other accidents
Sequelae of other accidents
Sequelae of intentional self-harm, assault and events of undetermined intent
Sequelae of intentional self-harm, assault and events of undetermined intent
Sequelae of intentional self-harm, assault and events of undetermined intent
Sequelae of intentional self-harm, assault and events of undetermined intent
Sequelae of intentional self-harm, assault and events of undetermined intent
Sequelae of surgical and medical care as external cause

For peer review only

Sequelae of surgical and medical care as external cause
Sequelae of surgical and medical care as external cause
Sequelae of surgical and medical care as external cause
Sequelae of other external causes
Sequelae of other external causes
Sequelae of other external causes
Sequelae of other external causes
Sequelae of other external causes

For peer review only

chapter	country	average age-standardized deaths	final best model polynomial degree
A00.B99	Belgium	9.78	3
A00.B99	Czech Republic	8.64	0
A00.B99	France	21.97	1
A00.B99	Germany	54.17	1
A00.B99	Hungary	6.18	0
A00.B99	Netherlands	7.44	1
A00.B99	Poland	9.07	0
A00.B99	Spain	15.93	1
A00.B99	United Kingdom	16.29	1
A00.B99	Belgium	132.83	2
A00.B99	Croatia	5.86	2
A00.B99	Czech Republic	103.51	3
A00.B99	Denmark	77.43	3
A00.B99	Finland	16.69	0
A00.B99	France	290.13	3
A00.B99	Germany	1221.13	3
A00.B99	Hungary	108.85	2
A00.B99	Netherlands	23.73	3
A00.B99	Norway	47.71	2
A00.B99	Poland	139.10	3
A00.B99	Romania	26.54	3
A00.B99	Spain	62.40	1
A00.B99	Sweden	65.24	3
A00.B99	Switzerland	23.09	1
A00.B99	United Kingdom	1817.62	2
A00.B99	France	11.53	1
A00.B99	Germany	9.54	2
A00.B99	Poland	5.75	0
A00.B99	Belgium	16.42	0
A00.B99	Denmark	17.33	2
A00.B99	Finland	18.39	2
A00.B99	France	33.75	1
A00.B99	Germany	171.25	2
A00.B99	Hungary	6.08	1
A00.B99	Netherlands	70.77	1
A00.B99	Norway	7.43	3
A00.B99	Poland	5.19	1
A00.B99	Spain	7.01	0
A00.B99	Sweden	40.71	2
A00.B99	Switzerland	12.96	3
A00.B99	United Kingdom	76.31	3
A00.B99	Austria	31.23	3
A00.B99	Belgium	147.79	1
A00.B99	Croatia	16.85	1
A00.B99	Czech Republic	40.34	1
A00.B99	Denmark	96.17	1
A00.B99	Finland	27.69	1
A00.B99	France	1031.42	3
A00.B99	Germany	1349.34	1

For peer review only

A00.B99	Hungary	62.70	3
A00.B99	Netherlands	157.63	2
A00.B99	Norway	121.17	2
A00.B99	Poland	30.26	0
A00.B99	Romania	60.39	2
A00.B99	Spain	538.56	1
A00.B99	Sweden	96.00	3
A00.B99	Switzerland	42.04	1
A00.B99	United Kingdom	377.61	1
A00.B99	Croatia	13.42	2
A00.B99	Czech Republic	41.62	2
A00.B99	Estonia	26.69	1
A00.B99	Finland	20.85	2
A00.B99	Germany	52.81	1
A00.B99	Hungary	45.07	2
A00.B99	Poland	345.67	0
A00.B99	Romania	1051.27	1
A00.B99	Slovenia	9.30	0
A00.B99	Austria	34.91	0
A00.B99	Belgium	38.61	1
A00.B99	Croatia	101.52	1
A00.B99	Czech Republic	23.82	1
A00.B99	Denmark	20.54	0
A00.B99	Estonia	23.02	2
A00.B99	France	357.23	3
A00.B99	Germany	229.49	2
A00.B99	Hungary	115.32	1
A00.B99	Latvia	52.58	2
A00.B99	Lithuania	155.24	1
A00.B99	Netherlands	24.40	1
A00.B99	Poland	514.49	2
A00.B99	Romania	679.40	2
A00.B99	Spain	247.81	2
A00.B99	Sweden	13.93	0
A00.B99	Switzerland	10.23	3
A00.B99	United Kingdom	302.85	1
A00.B99	France	14.06	1
A00.B99	Germany	9.64	0
A00.B99	Poland	9.37	1
A00.B99	Romania	17.09	1
A00.B99	Spain	16.08	1
A00.B99	United Kingdom	14.54	1
A00.B99	France	39.71	2
A00.B99	Germany	17.54	1
A00.B99	Poland	12.29	0
A00.B99	Romania	14.98	1
A00.B99	Spain	30.10	1
A00.B99	United Kingdom	41.12	1
A00.B99	Finland	7.44	3
A00.B99	France	26.15	1

For peer review only

A00.B99	Germany	21.82	1
A00.B99	Hungary	8.07	1
A00.B99	Latvia	5.32	0
A00.B99	Lithuania	74.55	3
A00.B99	Poland	9.24	1
A00.B99	Romania	13.24	0
A00.B99	Spain	30.55	1
A00.B99	United Kingdom	36.87	3
A00.B99	Germany	5.18	2
A00.B99	Romania	8.07	1
A00.B99	Finland	7.60	0
A00.B99	France	19.16	0
A00.B99	Germany	8.02	1
A00.B99	Poland	5.68	0
A00.B99	Sweden	6.62	1
A00.B99	United Kingdom	41.26	3
A00.B99	France	21.74	0
A00.B99	Germany	16.87	3
A00.B99	Spain	18.11	0
A00.B99	United Kingdom	16.21	0
A00.B99	France	6.02	1
A00.B99	Poland	8.94	1
A00.B99	Romania	5.33	1
A00.B99	France	5.01	0
A00.B99	Austria	6.07	0
A00.B99	Belgium	13.33	1
A00.B99	Czech Republic	6.68	1
A00.B99	Denmark	5.44	1
A00.B99	France	30.02	1
A00.B99	Germany	50.01	3
A00.B99	Hungary	7.47	0
A00.B99	Lithuania	7.37	0
A00.B99	Netherlands	12.96	3
A00.B99	Poland	15.10	0
A00.B99	Romania	16.17	2
A00.B99	Spain	42.73	1
A00.B99	Switzerland	6.75	2
A00.B99	United Kingdom	82.51	3
A00.B99	Austria	6.16	0
A00.B99	Belgium	34.18	2
A00.B99	Czech Republic	10.74	1
A00.B99	Denmark	23.52	2
A00.B99	Finland	28.34	0
A00.B99	France	121.57	0
A00.B99	Germany	152.87	2
A00.B99	Lithuania	6.30	1
A00.B99	Netherlands	56.51	1
A00.B99	Norway	26.55	1
A00.B99	Poland	22.38	3
A00.B99	Spain	32.47	0

For peer review only

A00.B99	Sweden	45.71	1
A00.B99	Switzerland	22.52	0
A00.B99	United Kingdom	118.54	3
A00.B99	Austria	87.99	2
A00.B99	Belgium	1466.24	3
A00.B99	Croatia	232.07	2
A00.B99	Czech Republic	759.13	1
A00.B99	Denmark	402.79	0
A00.B99	Estonia	13.02	2
A00.B99	Finland	129.82	0
A00.B99	France	4658.94	0
A00.B99	Germany	7361.90	1
A00.B99	Hungary	87.44	1
A00.B99	Latvia	29.51	0
A00.B99	Lithuania	181.40	2
A00.B99	Netherlands	1360.05	3
A00.B99	Norway	363.57	3
A00.B99	Poland	1236.95	3
A00.B99	Romania	625.64	2
A00.B99	Slovenia	86.99	0
A00.B99	Spain	3600.30	3
A00.B99	Sweden	705.38	1
A00.B99	Switzerland	192.17	0
A00.B99	United Kingdom	2963.53	3
A00.B99	France	5.41	0
A00.B99	Spain	5.24	0
A00.B99	Austria	15.53	2
A00.B99	Belgium	34.20	1
A00.B99	Czech Republic	15.71	2
A00.B99	Denmark	36.34	1
A00.B99	Estonia	6.24	0
A00.B99	Finland	27.89	0
A00.B99	France	209.88	1
A00.B99	Germany	236.24	1
A00.B99	Hungary	26.58	1
A00.B99	Latvia	15.88	1
A00.B99	Lithuania	9.46	0
A00.B99	Netherlands	121.16	1
A00.B99	Norway	26.92	0
A00.B99	Poland	17.45	0
A00.B99	Sweden	46.96	2
A00.B99	Switzerland	21.43	1
A00.B99	Belgium	25.29	0
A00.B99	Czech Republic	10.61	2
A00.B99	Denmark	13.04	1
A00.B99	France	125.58	2
A00.B99	Germany	58.22	1
A00.B99	Hungary	8.70	1
A00.B99	Netherlands	34.83	1
A00.B99	Poland	26.50	2

For peer review only

A00.B99	Romania	31.21	0
A00.B99	Spain	42.04	1
A00.B99	Sweden	7.39	0
A00.B99	Switzerland	12.08	1
A00.B99	United Kingdom	36.30	2
A00.B99	Belgium	52.93	3
A00.B99	Czech Republic	24.85	1
A00.B99	Denmark	62.50	2
A00.B99	Finland	13.65	0
A00.B99	France	138.52	1
A00.B99	Germany	166.82	3
A00.B99	Netherlands	19.82	2
A00.B99	Norway	15.89	1
A00.B99	Poland	22.69	1
A00.B99	Romania	22.55	1
A00.B99	Spain	116.89	3
A00.B99	Sweden	45.38	2
A00.B99	Switzerland	7.34	2
A00.B99	United Kingdom	198.85	2
A00.B99	Germany	6.36	2
A00.B99	Spain	7.60	0
A00.B99	Germany	9.70	0
A00.B99	Austria	23.65	0
A00.B99	Belgium	19.31	0
A00.B99	Denmark	8.60	0
A00.B99	Finland	12.41	0
A00.B99	France	154.72	0
A00.B99	Germany	160.70	1
A00.B99	Hungary	17.77	0
A00.B99	Netherlands	31.41	1
A00.B99	Norway	8.55	0
A00.B99	Poland	19.75	2
A00.B99	Romania	6.70	1
A00.B99	Spain	88.26	0
A00.B99	Sweden	19.47	0
A00.B99	Switzerland	20.16	1
A00.B99	United Kingdom	123.97	1
A00.B99	France	15.02	0
A00.B99	Germany	12.12	0
A00.B99	Poland	11.05	1
A00.B99	Romania	12.95	0
A00.B99	Spain	28.04	0
A00.B99	United Kingdom	42.92	1
A00.B99	France	10.96	1
A00.B99	Romania	6.33	0
A00.B99	Spain	9.28	1
A00.B99	Austria	5.57	1
A00.B99	Belgium	14.94	0
A00.B99	France	50.36	0
A00.B99	Germany	45.32	1

For peer review only

A00.B99	Netherlands	15.59	3
A00.B99	Spain	37.37	2
A00.B99	Sweden	6.65	0
A00.B99	Switzerland	5.24	0
A00.B99	United Kingdom	32.88	0
A00.B99	France	11.50	0
A00.B99	Germany	6.71	0
A00.B99	Spain	7.71	1
A00.B99	United Kingdom	21.80	3
A00.B99	Belgium	13.86	0
A00.B99	Denmark	5.27	1
A00.B99	France	71.15	1
A00.B99	Germany	75.74	1
A00.B99	Netherlands	28.33	0
A00.B99	Norway	9.75	2
A00.B99	Spain	21.28	0
A00.B99	Sweden	12.61	0
A00.B99	Switzerland	9.43	0
A00.B99	United Kingdom	69.17	3
A00.B99	Germany	8.72	3
A00.B99	Belgium	6.18	2
A00.B99	France	13.26	1
A00.B99	Germany	69.13	2
A00.B99	Poland	25.77	3
A00.B99	Romania	11.03	3
A00.B99	Spain	38.79	0
A00.B99	United Kingdom	37.60	1
A00.B99	Belgium	22.54	3
A00.B99	Czech Republic	17.33	0
A00.B99	France	70.84	1
A00.B99	Germany	72.19	2
A00.B99	Netherlands	6.25	2
A00.B99	Poland	9.47	0
A00.B99	Romania	7.36	1
A00.B99	Spain	139.58	3
A00.B99	United Kingdom	80.13	1
A00.B99	Austria	277.69	2
A00.B99	Belgium	89.56	2
A00.B99	Croatia	36.46	3
A00.B99	Czech Republic	23.05	1
A00.B99	Denmark	19.70	0
A00.B99	Estonia	5.65	1
A00.B99	Finland	8.66	0
A00.B99	France	702.43	1
A00.B99	Germany	876.32	1
A00.B99	Latvia	26.31	1
A00.B99	Lithuania	17.12	2
A00.B99	Netherlands	48.28	1
A00.B99	Norway	9.94	0
A00.B99	Poland	254.57	1

For peer review only

A00.B99	Romania	34.80	3
A00.B99	Slovenia	6.70	0
A00.B99	Spain	860.57	1
A00.B99	Sweden	57.65	1
A00.B99	Switzerland	14.33	1
A00.B99	United Kingdom	140.70	1
A00.B99	France	19.38	1
A00.B99	Germany	10.24	1
A00.B99	Poland	8.67	1
A00.B99	Spain	19.80	1
A00.B99	United Kingdom	5.84	1
A00.B99	Belgium	20.40	1
A00.B99	Denmark	11.14	0
A00.B99	Estonia	15.62	2
A00.B99	France	279.73	3
A00.B99	Germany	93.60	2
A00.B99	Latvia	31.93	2
A00.B99	Lithuania	7.86	1
A00.B99	Netherlands	27.18	3
A00.B99	Norway	7.12	0
A00.B99	Poland	57.00	0
A00.B99	Romania	77.78	2
A00.B99	Spain	530.89	2
A00.B99	Sweden	8.82	1
A00.B99	Switzerland	20.84	2
A00.B99	United Kingdom	131.09	2
A00.B99	Belgium	7.34	0
A00.B99	France	92.20	1
A00.B99	Germany	52.68	1
A00.B99	Netherlands	13.24	1
A00.B99	Poland	11.82	1
A00.B99	Romania	6.51	2
A00.B99	Spain	118.70	1
A00.B99	Switzerland	8.88	2
A00.B99	United Kingdom	29.58	1
A00.B99	Belgium	6.69	0
A00.B99	Estonia	9.72	1
A00.B99	France	145.50	1
A00.B99	Germany	29.52	2
A00.B99	Netherlands	6.48	2
A00.B99	Poland	25.47	3
A00.B99	Romania	24.72	3
A00.B99	Spain	112.55	1
A00.B99	Switzerland	21.14	1
A00.B99	United Kingdom	24.31	3
A00.B99	Belgium	6.08	0
A00.B99	France	142.60	3
A00.B99	Germany	37.80	3
A00.B99	Poland	15.95	0
A00.B99	Romania	5.74	2

For peer review only

A00.B99	Spain	113.78	2
A00.B99	United Kingdom	27.82	2
A00.B99	Austria	32.58	1
A00.B99	Belgium	11.76	0
A00.B99	Denmark	9.26	0
A00.B99	France	63.94	2
A00.B99	Germany	219.85	2
A00.B99	Netherlands	13.61	1
A00.B99	Romania	71.17	3
A00.B99	Spain	258.75	3
A00.B99	Switzerland	13.21	0
A00.B99	United Kingdom	17.46	3
A00.B99	France	15.61	3
A00.B99	Germany	11.67	0
A00.B99	Spain	7.81	0
A00.B99	United Kingdom	17.72	0
A00.B99	United Kingdom	10.26	0
A00.B99	Belgium	24.82	1
A00.B99	Denmark	8.54	0
A00.B99	France	63.32	1
A00.B99	Germany	151.72	1
A00.B99	Hungary	21.79	1
A00.B99	Latvia	14.16	0
A00.B99	Netherlands	89.62	3
A00.B99	Norway	5.59	1
A00.B99	Spain	13.75	1
A00.B99	Sweden	34.72	1
A00.B99	Switzerland	11.42	1
A00.B99	United Kingdom	38.05	0
A00.B99	Belgium	35.82	1
A00.B99	Denmark	5.29	1
A00.B99	France	95.35	3
A00.B99	Germany	95.35	2
A00.B99	Netherlands	11.49	0
A00.B99	Poland	5.68	0
A00.B99	Spain	26.54	0
A00.B99	Sweden	8.58	1
A00.B99	Switzerland	5.20	2
A00.B99	United Kingdom	29.19	0
A00.B99	Belgium	40.93	0
A00.B99	France	67.09	3
A00.B99	Germany	51.42	0
A00.B99	Netherlands	18.15	0
A00.B99	Poland	13.47	0
A00.B99	Spain	48.22	0
A00.B99	United Kingdom	68.90	1
A00.B99	Belgium	6.29	3
A00.B99	France	14.11	0
A00.B99	Germany	10.88	0
A00.B99	United Kingdom	16.74	0

For peer review only

A00.B99	France	12.59	1
A00.B99	France	5.15	1
A00.B99	Spain	6.24	2
A00.B99	Belgium	6.91	1
A00.B99	France	33.29	3
A00.B99	Germany	16.70	1
A00.B99	Netherlands	5.67	1
A00.B99	Spain	5.43	0
A00.B99	United Kingdom	37.53	0
A00.B99	Germany	6.30	0
A00.B99	Romania	16.87	1
A00.B99	Spain	19.74	1
A00.B99	Belgium	29.48	1
A00.B99	Finland	38.58	1
A00.B99	France	382.27	1
A00.B99	Germany	84.94	1
A00.B99	Hungary	57.02	2
A00.B99	Latvia	14.96	0
A00.B99	Netherlands	38.94	1
A00.B99	Norway	28.05	1
A00.B99	Poland	32.50	3
A00.B99	Spain	103.14	1
A00.B99	Sweden	37.15	1
A00.B99	Switzerland	11.52	1
A00.B99	United Kingdom	66.69	2
A00.B99	Austria	11.54	0
A00.B99	Belgium	7.38	1
A00.B99	Finland	7.76	0
A00.B99	France	51.98	0
A00.B99	Germany	23.72	2
A00.B99	Hungary	5.84	1
A00.B99	Netherlands	6.80	0
A00.B99	Norway	15.36	0
A00.B99	Spain	10.77	0
A00.B99	Sweden	28.64	1
A00.B99	Switzerland	10.82	0
A00.B99	United Kingdom	37.17	0
A00.B99	Finland	6.64	0
A00.B99	France	37.63	2
A00.B99	Germany	137.31	2
A00.B99	Poland	11.66	0
A00.B99	Sweden	6.05	2
A00.B99	Switzerland	72.26	0
A00.B99	United Kingdom	10.02	3
A00.B99	Austria	31.26	3
A00.B99	Belgium	349.03	1
A00.B99	Denmark	60.41	3
A00.B99	France	1540.91	2
A00.B99	Germany	1104.45	1
A00.B99	Netherlands	452.61	3

For peer review only

A00.B99	Norway	193.70	2
A00.B99	Spain	76.30	3
A00.B99	Sweden	352.47	1
A00.B99	Switzerland	116.27	1
A00.B99	United Kingdom	22.50	2
C00.D48	Croatia	13.54	1
C00.D48	Czech Republic	14.75	0
C00.D48	Denmark	6.12	0
C00.D48	France	42.06	1
C00.D48	Germany	44.31	0
C00.D48	Hungary	31.29	1
C00.D48	Latvia	10.48	0
C00.D48	Lithuania	11.79	1
C00.D48	Netherlands	9.01	0
C00.D48	Poland	177.06	3
C00.D48	Romania	168.30	1
C00.D48	Spain	66.63	1
C00.D48	Sweden	5.83	0
C00.D48	United Kingdom	24.67	0
C00.D48	Austria	79.35	2
C00.D48	Belgium	37.74	0
C00.D48	Croatia	26.88	0
C00.D48	Czech Republic	62.68	0
C00.D48	Denmark	9.64	0
C00.D48	Estonia	5.03	1
C00.D48	Finland	8.20	0
C00.D48	France	375.84	1
C00.D48	Germany	525.23	0
C00.D48	Hungary	251.33	0
C00.D48	Latvia	14.33	1
C00.D48	Lithuania	29.48	0
C00.D48	Netherlands	38.32	0
C00.D48	Norway	6.53	1
C00.D48	Poland	125.22	3
C00.D48	Romania	117.99	1
C00.D48	Spain	133.89	2
C00.D48	Sweden	30.14	1
C00.D48	Switzerland	40.96	0
C00.D48	United Kingdom	59.67	0
C00.D48	Austria	40.61	0
C00.D48	Belgium	70.54	3
C00.D48	Croatia	62.73	1
C00.D48	Czech Republic	103.43	1
C00.D48	Denmark	44.16	0
C00.D48	Estonia	19.27	0
C00.D48	Finland	41.67	0
C00.D48	France	560.41	1
C00.D48	Germany	301.54	3
C00.D48	Hungary	174.13	1
C00.D48	Latvia	21.68	0

For peer review only

C00.D48	Lithuania	28.45	0
C00.D48	Netherlands	88.33	0
C00.D48	Norway	30.23	0
C00.D48	Poland	345.93	3
C00.D48	Romania	278.13	2
C00.D48	Slovenia	13.17	0
C00.D48	Spain	455.86	0
C00.D48	Sweden	39.11	0
C00.D48	Switzerland	37.96	1
C00.D48	United Kingdom	586.43	2
C00.D48	Austria	19.94	3
C00.D48	Croatia	7.63	0
C00.D48	Czech Republic	17.00	0
C00.D48	Denmark	8.38	0
C00.D48	Finland	16.64	0
C00.D48	France	49.26	0
C00.D48	Germany	49.45	1
C00.D48	Hungary	33.09	2
C00.D48	Netherlands	5.92	0
C00.D48	Norway	10.66	2
C00.D48	Poland	38.53	1
C00.D48	Romania	26.53	1
C00.D48	Spain	36.90	0
C00.D48	Sweden	20.83	0
C00.D48	Switzerland	9.34	1
C00.D48	United Kingdom	95.16	1
C00.D48	Austria	68.75	0
C00.D48	Belgium	90.69	1
C00.D48	Croatia	21.15	1
C00.D48	Czech Republic	72.96	0
C00.D48	Denmark	16.38	2
C00.D48	Estonia	8.64	0
C00.D48	Finland	16.71	0
C00.D48	France	308.86	1
C00.D48	Germany	897.46	3
C00.D48	Hungary	117.26	1
C00.D48	Latvia	14.46	1
C00.D48	Lithuania	33.08	1
C00.D48	Netherlands	108.32	1
C00.D48	Norway	10.56	2
C00.D48	Poland	288.98	1
C00.D48	Romania	178.40	0
C00.D48	Slovenia	10.98	0
C00.D48	Spain	182.01	1
C00.D48	Sweden	20.90	2
C00.D48	Switzerland	34.98	0
C00.D48	United Kingdom	76.80	2
C00.D48	Austria	8.90	0
C00.D48	Belgium	12.69	0
C00.D48	Croatia	12.38	0

For peer review only

C00.D48	Czech Republic	25.30	0
C00.D48	Denmark	5.40	0
C00.D48	Finland	7.06	0
C00.D48	France	125.55	0
C00.D48	Germany	72.64	0
C00.D48	Hungary	43.92	0
C00.D48	Latvia	6.54	0
C00.D48	Lithuania	9.36	1
C00.D48	Netherlands	10.53	0
C00.D48	Poland	73.60	2
C00.D48	Romania	58.09	0
C00.D48	Spain	61.92	0
C00.D48	Sweden	7.42	0
C00.D48	Switzerland	9.19	2
C00.D48	United Kingdom	81.77	0
C00.D48	Austria	9.67	0
C00.D48	Belgium	30.27	0
C00.D48	Croatia	18.86	0
C00.D48	Czech Republic	33.08	1
C00.D48	Denmark	69.13	1
C00.D48	Finland	12.15	1
C00.D48	France	189.13	2
C00.D48	Germany	108.38	2
C00.D48	Hungary	76.93	0
C00.D48	Latvia	15.00	1
C00.D48	Lithuania	11.43	1
C00.D48	Netherlands	39.38	1
C00.D48	Norway	21.22	0
C00.D48	Poland	138.57	0
C00.D48	Romania	77.35	0
C00.D48	Slovenia	14.28	0
C00.D48	Spain	246.18	0
C00.D48	Sweden	27.61	0
C00.D48	Switzerland	16.66	0
C00.D48	United Kingdom	304.74	2
C00.D48	Austria	33.33	0
C00.D48	Belgium	29.77	2
C00.D48	Croatia	16.90	0
C00.D48	Czech Republic	45.97	1
C00.D48	Denmark	19.56	0
C00.D48	Estonia	6.31	0
C00.D48	Finland	16.02	0
C00.D48	France	197.64	0
C00.D48	Germany	233.79	0
C00.D48	Hungary	52.13	0
C00.D48	Latvia	9.48	1
C00.D48	Lithuania	14.50	0
C00.D48	Netherlands	41.98	0
C00.D48	Norway	13.37	0
C00.D48	Poland	167.35	0

For peer review only

C00.D48	Romania	87.04	0
C00.D48	Spain	152.25	0
C00.D48	Sweden	22.56	0
C00.D48	Switzerland	20.60	0
C00.D48	United Kingdom	178.31	0
C00.D48	Austria	8.32	0
C00.D48	Belgium	6.80	2
C00.D48	Croatia	5.62	0
C00.D48	Czech Republic	19.83	0
C00.D48	Denmark	6.32	1
C00.D48	Finland	5.97	0
C00.D48	France	39.21	0
C00.D48	Germany	52.63	0
C00.D48	Hungary	25.41	0
C00.D48	Latvia	8.95	0
C00.D48	Lithuania	9.96	0
C00.D48	Netherlands	14.73	2
C00.D48	Poland	85.62	2
C00.D48	Romania	20.21	1
C00.D48	Spain	25.15	1
C00.D48	Sweden	12.83	0
C00.D48	Switzerland	5.53	0
C00.D48	United Kingdom	45.06	0
C00.D48	Austria	58.14	0
C00.D48	Belgium	45.70	2
C00.D48	Croatia	33.59	0
C00.D48	Czech Republic	101.99	1
C00.D48	Denmark	49.01	0
C00.D48	Estonia	5.50	1
C00.D48	Finland	19.56	1
C00.D48	France	416.60	2
C00.D48	Germany	373.42	2
C00.D48	Hungary	111.34	1
C00.D48	Latvia	15.96	1
C00.D48	Lithuania	18.23	0
C00.D48	Netherlands	32.47	1
C00.D48	Norway	20.45	1
C00.D48	Poland	267.79	1
C00.D48	Romania	240.86	1
C00.D48	Spain	157.66	0
C00.D48	Sweden	43.23	0
C00.D48	Switzerland	33.90	0
C00.D48	United Kingdom	245.10	1
C00.D48	Austria	57.21	1
C00.D48	Belgium	49.14	1
C00.D48	Croatia	37.31	1
C00.D48	Czech Republic	75.43	1
C00.D48	Denmark	35.20	2
C00.D48	Estonia	13.57	0
C00.D48	Finland	5.95	2

For peer review only

C00.D48	France	567.96	2
C00.D48	Germany	918.14	1
C00.D48	Hungary	159.65	1
C00.D48	Latvia	7.71	3
C00.D48	Lithuania	37.27	3
C00.D48	Netherlands	87.43	0
C00.D48	Norway	11.87	2
C00.D48	Poland	158.88	1
C00.D48	Romania	238.87	1
C00.D48	Slovenia	60.48	0
C00.D48	Spain	306.33	2
C00.D48	Sweden	8.94	0
C00.D48	Switzerland	51.20	0
C00.D48	United Kingdom	233.90	3
C00.D48	Austria	20.86	0
C00.D48	Belgium	18.95	0
C00.D48	Croatia	16.97	0
C00.D48	Czech Republic	33.27	0
C00.D48	Denmark	11.03	0
C00.D48	Estonia	5.33	0
C00.D48	Finland	7.62	0
C00.D48	France	142.89	1
C00.D48	Germany	155.82	0
C00.D48	Hungary	48.80	0
C00.D48	Latvia	6.37	0
C00.D48	Lithuania	10.94	0
C00.D48	Netherlands	34.16	1
C00.D48	Norway	6.87	2
C00.D48	Poland	160.56	1
C00.D48	Romania	183.56	0
C00.D48	Spain	221.66	1
C00.D48	Sweden	16.23	1
C00.D48	Switzerland	13.72	1
C00.D48	United Kingdom	132.46	0
C00.D48	Austria	5.71	0
C00.D48	Belgium	20.60	0
C00.D48	Czech Republic	12.21	0
C00.D48	Estonia	5.59	1
C00.D48	France	452.13	2
C00.D48	Germany	6.04	0
C00.D48	Hungary	10.41	0
C00.D48	Lithuania	7.18	0
C00.D48	Netherlands	10.51	1
C00.D48	Poland	26.12	3
C00.D48	Romania	56.71	1
C00.D48	Spain	113.60	1
C00.D48	Switzerland	14.19	1
C00.D48	United Kingdom	86.45	0
C00.D48	Austria	77.90	0
C00.D48	Belgium	37.78	0

For peer review only

C00.D48	Croatia	84.24	1
C00.D48	Czech Republic	81.59	1
C00.D48	Denmark	48.97	1
C00.D48	Estonia	9.07	0
C00.D48	Finland	21.90	0
C00.D48	France	337.88	2
C00.D48	Germany	984.18	1
C00.D48	Hungary	291.49	1
C00.D48	Latvia	24.63	1
C00.D48	Lithuania	49.68	0
C00.D48	Netherlands	54.44	0
C00.D48	Norway	17.76	1
C00.D48	Poland	211.55	2
C00.D48	Romania	164.15	3
C00.D48	Slovenia	32.51	0
C00.D48	Spain	189.71	0
C00.D48	Sweden	41.51	1
C00.D48	Switzerland	55.32	0
C00.D48	United Kingdom	134.21	2
C00.D48	Austria	21.73	0
C00.D48	Belgium	110.39	0
C00.D48	Croatia	24.02	1
C00.D48	Czech Republic	35.50	1
C00.D48	Denmark	19.52	1
C00.D48	Estonia	5.59	1
C00.D48	France	769.81	1
C00.D48	Germany	243.93	1
C00.D48	Hungary	210.09	2
C00.D48	Latvia	19.44	3
C00.D48	Lithuania	5.51	0
C00.D48	Netherlands	99.59	0
C00.D48	Norway	6.04	0
C00.D48	Poland	290.56	2
C00.D48	Romania	293.84	2
C00.D48	Slovenia	9.17	0
C00.D48	Spain	188.71	1
C00.D48	Sweden	8.26	1
C00.D48	Switzerland	71.73	1
C00.D48	United Kingdom	238.72	0
C00.D48	Austria	356.66	0
C00.D48	Belgium	735.66	0
C00.D48	Croatia	223.12	1
C00.D48	Czech Republic	515.38	0
C00.D48	Denmark	449.43	1
C00.D48	Estonia	63.45	1
C00.D48	Finland	244.80	0
C00.D48	France	4390.10	2
C00.D48	Germany	4879.38	1
C00.D48	Hungary	659.75	1
C00.D48	Latvia	121.07	1

For peer review only

C00.D48	Lithuania	193.08	1
C00.D48	Netherlands	1803.26	2
C00.D48	Norway	224.27	0
C00.D48	Poland	1818.22	1
C00.D48	Romania	737.89	2
C00.D48	Slovenia	97.39	1
C00.D48	Spain	2061.63	2
C00.D48	Sweden	425.26	0
C00.D48	Switzerland	475.80	0
C00.D48	United Kingdom	8568.89	3
C00.D48	Austria	1070.57	2
C00.D48	Belgium	926.52	2
C00.D48	Croatia	1020.83	1
C00.D48	Czech Republic	1573.28	2
C00.D48	Denmark	451.24	1
C00.D48	Estonia	350.16	1
C00.D48	Finland	572.62	1
C00.D48	France	5362.07	2
C00.D48	Germany	10971.18	2
C00.D48	Hungary	2120.23	2
C00.D48	Latvia	591.28	1
C00.D48	Lithuania	853.87	2
C00.D48	Netherlands	1810.99	1
C00.D48	Norway	454.29	2
C00.D48	Poland	7313.83	2
C00.D48	Romania	4575.15	1
C00.D48	Slovenia	432.00	1
C00.D48	Spain	6473.17	3
C00.D48	Sweden	747.80	2
C00.D48	Switzerland	612.56	2
C00.D48	United Kingdom	6114.85	2
C00.D48	Austria	55.76	1
C00.D48	Belgium	58.20	0
C00.D48	Croatia	30.79	1
C00.D48	Czech Republic	87.88	0
C00.D48	Denmark	53.46	1
C00.D48	Estonia	12.00	0
C00.D48	Finland	49.50	1
C00.D48	France	412.64	2
C00.D48	Germany	454.50	2
C00.D48	Hungary	77.66	2
C00.D48	Latvia	13.67	0
C00.D48	Lithuania	19.97	0
C00.D48	Netherlands	152.30	2
C00.D48	Norway	59.95	0
C00.D48	Poland	213.91	0
C00.D48	Romania	113.53	2
C00.D48	Slovenia	15.87	0
C00.D48	Spain	198.52	1
C00.D48	Sweden	113.99	0

For peer review only

C00.D48	Switzerland	58.17	0
C00.D48	United Kingdom	431.82	1
C00.D48	Austria	1700.02	2
C00.D48	Belgium	2543.42	2
C00.D48	Croatia	1285.25	2
C00.D48	Czech Republic	2985.96	1
C00.D48	Denmark	1627.29	1
C00.D48	Estonia	289.28	0
C00.D48	Finland	774.46	0
C00.D48	France	13674.13	2
C00.D48	Germany	18779.55	3
C00.D48	Hungary	3741.13	1
C00.D48	Latvia	443.31	0
C00.D48	Lithuania	574.65	2
C00.D48	Netherlands	4612.69	2
C00.D48	Norway	1370.52	1
C00.D48	Poland	9098.25	0
C00.D48	Romania	3919.18	1
C00.D48	Slovenia	502.93	2
C00.D48	Spain	11910.60	3
C00.D48	Sweden	1885.22	1
C00.D48	Switzerland	1340.83	3
C00.D48	United Kingdom	11554.54	1
C00.D48	Austria	41.14	1
C00.D48	Belgium	137.27	0
C00.D48	Croatia	97.35	1
C00.D48	Czech Republic	662.87	1
C00.D48	Denmark	24.36	1
C00.D48	Estonia	29.59	1
C00.D48	Finland	55.83	1
C00.D48	France	1022.25	1
C00.D48	Germany	477.69	1
C00.D48	Hungary	208.50	2
C00.D48	Latvia	42.27	3
C00.D48	Lithuania	68.07	0
C00.D48	Netherlands	133.45	0
C00.D48	Norway	35.63	2
C00.D48	Poland	383.56	2
C00.D48	Romania	759.16	0
C00.D48	Slovenia	73.77	1
C00.D48	Spain	710.54	0
C00.D48	Sweden	55.67	0
C00.D48	Switzerland	86.25	1
C00.D48	United Kingdom	1996.47	1
C00.D48	Austria	744.27	1
C00.D48	Belgium	572.53	1
C00.D48	Croatia	722.33	1
C00.D48	Czech Republic	1328.01	1
C00.D48	Denmark	609.46	1
C00.D48	Estonia	146.78	1

For peer review only

C00.D48	Finland	401.33	1
C00.D48	France	3471.90	1
C00.D48	Germany	7909.38	1
C00.D48	Hungary	1790.47	1
C00.D48	Latvia	286.46	1
C00.D48	Lithuania	435.08	1
C00.D48	Netherlands	1189.43	0
C00.D48	Norway	462.34	2
C00.D48	Poland	3669.19	3
C00.D48	Romania	1627.47	2
C00.D48	Slovenia	293.89	1
C00.D48	Spain	2987.13	0
C00.D48	Sweden	773.71	0
C00.D48	Switzerland	448.72	1
C00.D48	United Kingdom	4641.80	1
C00.D48	Austria	38.45	0
C00.D48	Belgium	25.74	1
C00.D48	Croatia	10.64	0
C00.D48	Czech Republic	87.52	2
C00.D48	Denmark	31.92	3
C00.D48	Estonia	8.74	0
C00.D48	Finland	16.80	0
C00.D48	France	321.18	1
C00.D48	Germany	383.44	3
C00.D48	Hungary	37.52	2
C00.D48	Latvia	10.42	0
C00.D48	Lithuania	14.96	1
C00.D48	Netherlands	39.39	0
C00.D48	Norway	18.97	0
C00.D48	Poland	736.10	3
C00.D48	Romania	164.76	2
C00.D48	Slovenia	6.64	1
C00.D48	Spain	94.19	1
C00.D48	Sweden	38.64	0
C00.D48	Switzerland	42.06	0
C00.D48	United Kingdom	319.50	2
C00.D48	Austria	901.16	0
C00.D48	Belgium	818.27	3
C00.D48	Croatia	512.11	3
C00.D48	Czech Republic	1042.29	2
C00.D48	Denmark	372.93	1
C00.D48	Estonia	97.51	0
C00.D48	Finland	473.65	0
C00.D48	France	8425.34	1
C00.D48	Germany	6839.91	2
C00.D48	Hungary	940.63	2
C00.D48	Latvia	149.78	0
C00.D48	Lithuania	190.35	1
C00.D48	Netherlands	809.49	2
C00.D48	Norway	201.73	1

For peer review only

C00.D48	Poland	2658.83	3
C00.D48	Romania	3121.36	1
C00.D48	Slovenia	210.22	1
C00.D48	Spain	5259.94	3
C00.D48	Sweden	660.07	0
C00.D48	Switzerland	680.96	1
C00.D48	United Kingdom	4051.88	2
C00.D48	Austria	156.39	1
C00.D48	Belgium	84.86	1
C00.D48	Croatia	190.45	2
C00.D48	Czech Republic	577.63	1
C00.D48	Denmark	34.06	0
C00.D48	Estonia	17.86	0
C00.D48	Finland	92.55	2
C00.D48	France	487.65	2
C00.D48	Germany	1739.51	2
C00.D48	Hungary	479.73	2
C00.D48	Latvia	20.41	1
C00.D48	Lithuania	54.78	2
C00.D48	Netherlands	157.11	1
C00.D48	Norway	29.39	0
C00.D48	Poland	1669.34	2
C00.D48	Romania	315.12	3
C00.D48	Slovenia	70.04	2
C00.D48	Spain	614.57	1
C00.D48	Sweden	202.71	1
C00.D48	Switzerland	91.88	1
C00.D48	United Kingdom	523.34	1
C00.D48	Austria	186.41	0
C00.D48	Belgium	106.65	1
C00.D48	Croatia	95.39	1
C00.D48	Czech Republic	427.51	0
C00.D48	Denmark	104.90	0
C00.D48	Estonia	29.80	1
C00.D48	Finland	132.69	1
C00.D48	France	925.30	1
C00.D48	Germany	2002.50	2
C00.D48	Hungary	368.39	2
C00.D48	Latvia	34.53	0
C00.D48	Lithuania	51.98	0
C00.D48	Netherlands	255.09	2
C00.D48	Norway	62.42	2
C00.D48	Poland	911.98	1
C00.D48	Romania	305.84	2
C00.D48	Slovenia	66.74	0
C00.D48	Spain	835.07	3
C00.D48	Sweden	325.28	1
C00.D48	Switzerland	118.69	0
C00.D48	United Kingdom	283.90	2
C00.D48	Austria	1598.11	0

For peer review only

C00.D48	Belgium	1606.64	1
C00.D48	Croatia	730.98	1
C00.D48	Czech Republic	2212.87	0
C00.D48	Denmark	990.02	2
C00.D48	Estonia	243.19	1
C00.D48	Finland	1050.69	0
C00.D48	France	9530.08	1
C00.D48	Germany	14771.19	1
C00.D48	Hungary	2051.51	1
C00.D48	Latvia	395.34	1
C00.D48	Lithuania	513.50	0
C00.D48	Netherlands	2768.64	2
C00.D48	Norway	793.44	0
C00.D48	Poland	5692.00	0
C00.D48	Romania	3110.30	2
C00.D48	Slovenia	363.35	3
C00.D48	Spain	5893.56	1
C00.D48	Sweden	1643.02	1
C00.D48	Switzerland	1169.38	1
C00.D48	United Kingdom	8902.67	1
C00.D48	Austria	62.47	1
C00.D48	Belgium	450.82	1
C00.D48	Croatia	42.32	0
C00.D48	Czech Republic	251.87	1
C00.D48	Denmark	55.63	0
C00.D48	Estonia	16.87	2
C00.D48	Finland	114.67	0
C00.D48	France	1914.40	1
C00.D48	Germany	1202.50	1
C00.D48	Hungary	109.11	2
C00.D48	Latvia	18.82	1
C00.D48	Lithuania	18.99	3
C00.D48	Netherlands	1154.93	3
C00.D48	Norway	71.38	1
C00.D48	Poland	967.63	0
C00.D48	Romania	95.86	3
C00.D48	Slovenia	27.50	2
C00.D48	Spain	834.34	3
C00.D48	Sweden	173.19	0
C00.D48	Switzerland	132.65	1
C00.D48	United Kingdom	3207.88	2
C00.D48	Croatia	6.07	0
C00.D48	Czech Republic	16.92	0
C00.D48	Denmark	13.41	0
C00.D48	Finland	8.81	0
C00.D48	France	39.66	0
C00.D48	Germany	32.76	1
C00.D48	Hungary	12.28	1
C00.D48	Netherlands	9.26	0
C00.D48	Poland	58.23	2

For peer review only

C00.D48	Romania	60.71	1
C00.D48	Spain	31.56	0
C00.D48	Sweden	5.14	0
C00.D48	Switzerland	8.16	1
C00.D48	United Kingdom	37.21	1
C00.D48	Austria	18.52	0
C00.D48	Belgium	26.35	0
C00.D48	Croatia	12.34	0
C00.D48	Czech Republic	27.32	0
C00.D48	Denmark	9.97	0
C00.D48	Estonia	6.38	0
C00.D48	Finland	11.64	0
C00.D48	France	162.75	1
C00.D48	Germany	144.54	0
C00.D48	Hungary	29.21	0
C00.D48	Latvia	7.52	0
C00.D48	Lithuania	19.03	2
C00.D48	Netherlands	27.78	2
C00.D48	Norway	9.38	0
C00.D48	Poland	98.28	2
C00.D48	Romania	71.89	2
C00.D48	Slovenia	7.07	0
C00.D48	Spain	74.30	1
C00.D48	Sweden	15.03	0
C00.D48	Switzerland	13.62	0
C00.D48	United Kingdom	107.18	2
C00.D48	Austria	152.61	2
C00.D48	Belgium	247.59	2
C00.D48	Croatia	227.88	1
C00.D48	Czech Republic	287.98	1
C00.D48	Denmark	113.47	1
C00.D48	Estonia	41.42	1
C00.D48	Finland	41.83	0
C00.D48	France	1565.17	2
C00.D48	Germany	1495.38	1
C00.D48	Hungary	586.14	1
C00.D48	Latvia	92.67	0
C00.D48	Lithuania	143.30	0
C00.D48	Netherlands	260.40	2
C00.D48	Norway	48.05	1
C00.D48	Poland	1937.08	1
C00.D48	Romania	1118.26	3
C00.D48	Slovenia	63.08	1
C00.D48	Spain	1765.89	3
C00.D48	Sweden	58.28	0
C00.D48	Switzerland	103.44	2
C00.D48	United Kingdom	935.09	2
C00.D48	Belgium	8.51	1
C00.D48	Croatia	13.96	0
C00.D48	Czech Republic	14.81	0

For peer review only

C00.D48	France	62.98	1
C00.D48	Germany	35.01	0
C00.D48	Hungary	28.67	0
C00.D48	Netherlands	7.86	0
C00.D48	Poland	51.14	1
C00.D48	Romania	14.38	0
C00.D48	Spain	25.54	0
C00.D48	United Kingdom	29.53	1
C00.D48	Austria	3922.22	2
C00.D48	Belgium	7008.28	1
C00.D48	Croatia	2920.29	0
C00.D48	Czech Republic	6359.29	1
C00.D48	Denmark	4156.65	2
C00.D48	Estonia	735.25	1
C00.D48	Finland	2253.31	1
C00.D48	France	31489.35	2
C00.D48	Germany	41859.76	0
C00.D48	Hungary	8998.97	0
C00.D48	Latvia	1113.97	2
C00.D48	Lithuania	1571.89	1
C00.D48	Netherlands	11699.38	2
C00.D48	Norway	2501.41	2
C00.D48	Poland	27661.54	1
C00.D48	Romania	10946.56	1
C00.D48	Slovenia	1196.23	3
C00.D48	Spain	22762.25	3
C00.D48	Sweden	3644.45	2
C00.D48	Switzerland	3370.25	1
C00.D48	United Kingdom	39651.31	2
C00.D48	Austria	12.19	2
C00.D48	Belgium	10.59	0
C00.D48	Czech Republic	11.42	1
C00.D48	France	56.81	0
C00.D48	Germany	72.24	1
C00.D48	Hungary	8.60	1
C00.D48	Netherlands	17.64	0
C00.D48	Poland	16.00	0
C00.D48	Spain	39.06	1
C00.D48	Sweden	7.81	0
C00.D48	Switzerland	8.41	0
C00.D48	United Kingdom	45.19	1
C00.D48	Austria	18.32	3
C00.D48	Belgium	60.23	1
C00.D48	Croatia	26.35	1
C00.D48	Czech Republic	90.03	1
C00.D48	Denmark	31.42	0
C00.D48	Estonia	7.14	0
C00.D48	Finland	28.43	1
C00.D48	France	501.41	1
C00.D48	Germany	225.22	2

For peer review only

C00.D48	Hungary	80.11	2
C00.D48	Latvia	11.94	0
C00.D48	Lithuania	19.42	0
C00.D48	Netherlands	36.72	1
C00.D48	Norway	14.73	2
C00.D48	Poland	425.78	1
C00.D48	Romania	289.76	0
C00.D48	Slovenia	16.90	1
C00.D48	Spain	165.56	1
C00.D48	Sweden	33.40	2
C00.D48	Switzerland	20.58	1
C00.D48	United Kingdom	120.09	1
C00.D48	Belgium	13.66	2
C00.D48	Czech Republic	48.60	0
C00.D48	Denmark	18.68	1
C00.D48	Finland	5.04	0
C00.D48	France	938.81	2
C00.D48	Germany	24.14	1
C00.D48	Hungary	14.41	1
C00.D48	Poland	75.35	1
C00.D48	Romania	12.86	2
C00.D48	Spain	62.05	1
C00.D48	Sweden	7.08	1
C00.D48	United Kingdom	23.66	0
C00.D48	Austria	6.69	0
C00.D48	Belgium	9.62	0
C00.D48	Croatia	28.36	0
C00.D48	Czech Republic	32.02	1
C00.D48	Denmark	10.87	2
C00.D48	Estonia	5.78	0
C00.D48	Finland	7.99	0
C00.D48	France	62.95	1
C00.D48	Germany	53.13	0
C00.D48	Hungary	13.09	2
C00.D48	Latvia	7.76	0
C00.D48	Lithuania	11.95	1
C00.D48	Netherlands	7.48	1
C00.D48	Poland	139.48	2
C00.D48	Romania	122.38	1
C00.D48	Slovenia	5.22	0
C00.D48	Spain	29.24	0
C00.D48	Switzerland	6.21	0
C00.D48	United Kingdom	37.37	1
C00.D48	Austria	35.90	0
C00.D48	Belgium	81.49	0
C00.D48	Croatia	51.52	0
C00.D48	Czech Republic	68.07	1
C00.D48	Denmark	19.75	0
C00.D48	Estonia	6.76	0
C00.D48	Finland	13.43	1

For peer review only

C00.D48	France	530.20	1
C00.D48	Germany	377.75	0
C00.D48	Hungary	58.02	2
C00.D48	Latvia	21.58	1
C00.D48	Lithuania	18.49	0
C00.D48	Netherlands	103.80	0
C00.D48	Norway	21.07	0
C00.D48	Poland	320.78	2
C00.D48	Romania	441.29	1
C00.D48	Slovenia	10.49	0
C00.D48	Spain	282.85	0
C00.D48	Sweden	39.14	1
C00.D48	Switzerland	34.36	0
C00.D48	United Kingdom	283.26	0
C00.D48	Austria	351.56	0
C00.D48	Belgium	321.60	1
C00.D48	Croatia	208.57	0
C00.D48	Czech Republic	423.28	1
C00.D48	Denmark	280.41	1
C00.D48	Estonia	57.67	2
C00.D48	Finland	206.27	1
C00.D48	France	1725.80	1
C00.D48	Germany	2579.33	3
C00.D48	Hungary	388.21	0
C00.D48	Latvia	80.38	0
C00.D48	Lithuania	108.89	2
C00.D48	Netherlands	787.26	3
C00.D48	Norway	332.58	2
C00.D48	Poland	1419.95	1
C00.D48	Romania	446.41	0
C00.D48	Slovenia	118.51	2
C00.D48	Spain	968.92	1
C00.D48	Sweden	480.46	2
C00.D48	Switzerland	314.72	1
C00.D48	United Kingdom	2260.40	1
C00.D48	Austria	92.05	1
C00.D48	Belgium	108.14	2
C00.D48	Croatia	100.82	2
C00.D48	Czech Republic	192.77	3
C00.D48	Denmark	67.60	0
C00.D48	Estonia	25.52	0
C00.D48	Finland	50.35	0
C00.D48	France	661.52	3
C00.D48	Germany	627.79	1
C00.D48	Hungary	232.15	0
C00.D48	Latvia	56.94	0
C00.D48	Lithuania	54.12	0
C00.D48	Netherlands	113.53	0
C00.D48	Norway	47.04	0
C00.D48	Poland	784.68	3

For peer review only

C00.D48	Romania	611.52	1
C00.D48	Slovenia	35.58	2
C00.D48	Spain	596.62	3
C00.D48	Sweden	64.10	0
C00.D48	Switzerland	90.72	1
C00.D48	United Kingdom	661.79	3
C00.D48	Austria	98.00	2
C00.D48	Belgium	225.75	1
C00.D48	Croatia	63.39	1
C00.D48	Czech Republic	53.75	0
C00.D48	Denmark	97.43	1
C00.D48	Finland	90.62	0
C00.D48	France	1016.65	1
C00.D48	Germany	1250.24	3
C00.D48	Hungary	39.64	0
C00.D48	Latvia	9.49	0
C00.D48	Lithuania	11.52	1
C00.D48	Netherlands	549.80	0
C00.D48	Norway	75.01	2
C00.D48	Poland	200.56	2
C00.D48	Romania	70.17	0
C00.D48	Slovenia	31.74	0
C00.D48	Spain	386.71	3
C00.D48	Sweden	135.07	0
C00.D48	Switzerland	168.30	1
C00.D48	United Kingdom	2464.46	3
C00.D48	Czech Republic	6.86	0
C00.D48	France	14.70	0
C00.D48	Germany	14.28	1
C00.D48	Poland	20.40	0
C00.D48	Romania	29.17	0
C00.D48	Spain	24.35	1
C00.D48	United Kingdom	8.07	0
C00.D48	Austria	6.26	2
C00.D48	Czech Republic	8.82	0
C00.D48	France	23.54	0
C00.D48	Germany	48.92	1
C00.D48	Hungary	7.99	2
C00.D48	Netherlands	9.51	1
C00.D48	Poland	17.23	0
C00.D48	Romania	8.22	0
C00.D48	Spain	18.80	0
C00.D48	Sweden	5.66	1
C00.D48	Switzerland	11.17	2
C00.D48	United Kingdom	30.47	1
C00.D48	Austria	49.04	0
C00.D48	Belgium	28.33	0
C00.D48	Croatia	22.13	1
C00.D48	Czech Republic	92.98	1
C00.D48	Denmark	17.32	0

For peer review only

C00.D48	Estonia	8.21	0
C00.D48	Finland	36.41	1
C00.D48	France	229.29	1
C00.D48	Germany	322.91	1
C00.D48	Hungary	81.28	1
C00.D48	Latvia	17.03	0
C00.D48	Lithuania	31.36	0
C00.D48	Netherlands	16.71	1
C00.D48	Norway	26.18	1
C00.D48	Poland	241.45	0
C00.D48	Romania	488.63	0
C00.D48	Slovenia	19.63	0
C00.D48	Spain	233.13	2
C00.D48	Sweden	35.46	1
C00.D48	Switzerland	40.39	3
C00.D48	United Kingdom	366.17	1
C00.D48	Austria	122.28	0
C00.D48	Belgium	152.98	0
C00.D48	Croatia	37.96	1
C00.D48	Czech Republic	113.01	0
C00.D48	Denmark	57.98	0
C00.D48	Estonia	13.60	0
C00.D48	Finland	80.10	0
C00.D48	France	823.84	1
C00.D48	Germany	1083.73	3
C00.D48	Hungary	123.70	0
C00.D48	Latvia	27.02	0
C00.D48	Lithuania	38.80	0
C00.D48	Netherlands	238.39	1
C00.D48	Norway	55.68	1
C00.D48	Poland	369.40	1
C00.D48	Romania	239.38	1
C00.D48	Slovenia	22.12	0
C00.D48	Spain	523.62	1
C00.D48	Sweden	146.52	0
C00.D48	Switzerland	102.90	1
C00.D48	United Kingdom	816.99	1
C00.D48	Austria	1718.60	2
C00.D48	Belgium	2570.34	3
C00.D48	Croatia	1088.80	1
C00.D48	Czech Republic	2234.32	1
C00.D48	Denmark	1407.10	1
C00.D48	Estonia	270.57	1
C00.D48	Finland	920.10	1
C00.D48	France	12812.81	3
C00.D48	Germany	17880.02	2
C00.D48	Hungary	2511.58	2
C00.D48	Latvia	475.91	0
C00.D48	Lithuania	648.39	0
C00.D48	Netherlands	3915.76	2

For peer review only

C00.D48	Norway	783.47	1
C00.D48	Poland	6860.49	3
C00.D48	Romania	3929.44	0
C00.D48	Slovenia	482.50	1
C00.D48	Spain	6843.63	3
C00.D48	Sweden	1527.74	1
C00.D48	Switzerland	1523.91	1
C00.D48	United Kingdom	13672.05	2
C00.D48	Austria	70.32	0
C00.D48	Belgium	58.16	0
C00.D48	Croatia	48.00	0
C00.D48	Czech Republic	115.56	1
C00.D48	Denmark	38.86	1
C00.D48	Estonia	14.77	0
C00.D48	Finland	37.25	0
C00.D48	France	252.44	0
C00.D48	Germany	735.78	1
C00.D48	Hungary	113.33	0
C00.D48	Latvia	26.70	0
C00.D48	Lithuania	31.94	0
C00.D48	Netherlands	126.22	0
C00.D48	Norway	30.05	0
C00.D48	Poland	345.09	0
C00.D48	Romania	171.86	0
C00.D48	Slovenia	23.56	0
C00.D48	Spain	323.32	1
C00.D48	Sweden	59.05	0
C00.D48	Switzerland	35.80	0
C00.D48	United Kingdom	448.06	0
C00.D48	Austria	24.65	1
C00.D48	Belgium	17.31	0
C00.D48	Croatia	9.97	0
C00.D48	Czech Republic	26.36	1
C00.D48	Denmark	10.92	0
C00.D48	Estonia	5.72	1
C00.D48	Finland	13.12	0
C00.D48	France	84.74	0
C00.D48	Germany	169.62	1
C00.D48	Hungary	35.50	0
C00.D48	Latvia	7.40	0
C00.D48	Lithuania	8.15	0
C00.D48	Netherlands	27.70	0
C00.D48	Norway	5.67	1
C00.D48	Poland	85.81	1
C00.D48	Romania	40.90	2
C00.D48	Slovenia	5.48	0
C00.D48	Spain	55.90	2
C00.D48	Sweden	15.07	1
C00.D48	Switzerland	12.99	0
C00.D48	United Kingdom	109.82	0

For peer review only

C00.D48	Austria	168.19	1
C00.D48	Belgium	183.97	0
C00.D48	Croatia	121.65	1
C00.D48	Czech Republic	399.68	2
C00.D48	Denmark	129.06	1
C00.D48	Estonia	69.62	0
C00.D48	Finland	61.13	1
C00.D48	France	796.17	1
C00.D48	Germany	1608.89	2
C00.D48	Hungary	470.60	2
C00.D48	Latvia	123.85	1
C00.D48	Lithuania	242.80	1
C00.D48	Netherlands	244.67	1
C00.D48	Norway	92.95	1
C00.D48	Poland	2079.94	1
C00.D48	Romania	2005.11	3
C00.D48	Slovenia	51.62	1
C00.D48	Spain	659.79	2
C00.D48	Sweden	151.59	0
C00.D48	Switzerland	91.57	1
C00.D48	United Kingdom	1085.58	2
C00.D48	Austria	168.00	1
C00.D48	Belgium	214.64	0
C00.D48	Croatia	112.98	1
C00.D48	Czech Republic	399.98	1
C00.D48	Denmark	128.80	1
C00.D48	Estonia	41.75	1
C00.D48	Finland	185.56	0
C00.D48	France	799.43	2
C00.D48	Germany	1401.66	3
C00.D48	Hungary	333.97	2
C00.D48	Latvia	98.30	1
C00.D48	Lithuania	151.71	0
C00.D48	Netherlands	418.31	0
C00.D48	Norway	111.81	3
C00.D48	Poland	1310.21	2
C00.D48	Romania	415.01	2
C00.D48	Slovenia	61.83	0
C00.D48	Spain	1081.65	3
C00.D48	Sweden	169.27	0
C00.D48	Switzerland	183.35	1
C00.D48	United Kingdom	1496.84	2
C00.D48	Austria	143.35	3
C00.D48	Belgium	186.48	1
C00.D48	Croatia	140.60	1
C00.D48	Czech Republic	169.79	3
C00.D48	Denmark	69.48	1
C00.D48	Estonia	23.38	1
C00.D48	Finland	10.07	0
C00.D48	France	1812.00	2

For peer review only

C00.D48	Germany	1162.86	2
C00.D48	Hungary	143.49	1
C00.D48	Latvia	61.32	3
C00.D48	Lithuania	18.76	0
C00.D48	Netherlands	113.36	1
C00.D48	Norway	67.75	3
C00.D48	Poland	468.15	1
C00.D48	Romania	329.55	2
C00.D48	Slovenia	51.46	0
C00.D48	Spain	507.04	1
C00.D48	Sweden	178.56	0
C00.D48	Switzerland	55.50	1
C00.D48	United Kingdom	567.18	1
C00.D48	Austria	556.52	1
C00.D48	Belgium	745.47	3
C00.D48	Croatia	327.79	0
C00.D48	Czech Republic	845.55	2
C00.D48	Denmark	443.72	1
C00.D48	Estonia	115.30	1
C00.D48	Finland	349.88	0
C00.D48	France	3736.47	1
C00.D48	Germany	5656.66	3
C00.D48	Hungary	760.92	0
C00.D48	Latvia	212.10	0
C00.D48	Lithuania	302.65	0
C00.D48	Netherlands	1192.67	1
C00.D48	Norway	372.02	1
C00.D48	Poland	3015.81	0
C00.D48	Romania	1149.40	1
C00.D48	Slovenia	153.17	0
C00.D48	Spain	2049.74	1
C00.D48	Sweden	626.73	1
C00.D48	Switzerland	482.04	1
C00.D48	United Kingdom	4873.91	1
C00.D48	Austria	33.41	1
C00.D48	Belgium	50.47	1
C00.D48	Croatia	11.14	0
C00.D48	Czech Republic	131.98	0
C00.D48	Denmark	28.96	0
C00.D48	Estonia	5.85	0
C00.D48	Finland	52.42	2
C00.D48	France	209.21	2
C00.D48	Germany	310.71	2
C00.D48	Hungary	30.33	2
C00.D48	Latvia	7.21	0
C00.D48	Lithuania	8.98	1
C00.D48	Netherlands	41.66	1
C00.D48	Norway	28.85	0
C00.D48	Poland	364.30	1
C00.D48	Romania	89.90	1

For peer review only

C00.D48	Slovenia	8.34	2
C00.D48	Spain	183.25	2
C00.D48	Sweden	93.23	0
C00.D48	Switzerland	40.53	2
C00.D48	United Kingdom	131.36	2
C00.D48	Austria	15.10	0
C00.D48	Belgium	14.50	0
C00.D48	Croatia	10.52	0
C00.D48	Czech Republic	28.71	0
C00.D48	Denmark	13.33	0
C00.D48	Finland	7.45	0
C00.D48	France	81.42	0
C00.D48	Germany	147.75	1
C00.D48	Hungary	28.74	2
C00.D48	Latvia	7.39	0
C00.D48	Lithuania	9.60	0
C00.D48	Netherlands	31.43	1
C00.D48	Norway	8.14	2
C00.D48	Poland	111.95	1
C00.D48	Romania	61.88	0
C00.D48	Spain	115.00	0
C00.D48	Sweden	21.23	0
C00.D48	Switzerland	14.05	1
C00.D48	United Kingdom	125.25	0
C00.D48	Austria	1282.20	2
C00.D48	Belgium	1662.94	2
C00.D48	Croatia	820.40	1
C00.D48	Czech Republic	1851.42	1
C00.D48	Denmark	1387.74	1
C00.D48	Estonia	260.25	1
C00.D48	Finland	957.34	1
C00.D48	France	9967.49	1
C00.D48	Germany	12531.83	2
C00.D48	Hungary	1509.39	2
C00.D48	Latvia	408.57	3
C00.D48	Lithuania	616.74	0
C00.D48	Netherlands	3183.89	1
C00.D48	Norway	1264.97	1
C00.D48	Poland	5643.53	0
C00.D48	Romania	2566.20	2
C00.D48	Slovenia	425.14	0
C00.D48	Spain	6448.51	3
C00.D48	Sweden	2504.28	1
C00.D48	Switzerland	1517.17	1
C00.D48	United Kingdom	12073.19	3
C00.D48	Austria	15.73	0
C00.D48	Belgium	12.01	1
C00.D48	Croatia	15.13	0
C00.D48	Czech Republic	36.01	1
C00.D48	Denmark	12.67	1

For peer review only

C00.D48	Finland	6.50	1
C00.D48	France	100.24	1
C00.D48	Germany	163.72	1
C00.D48	Hungary	47.01	0
C00.D48	Latvia	8.84	0
C00.D48	Lithuania	7.82	1
C00.D48	Netherlands	26.36	0
C00.D48	Norway	10.46	0
C00.D48	Poland	123.73	3
C00.D48	Romania	75.34	1
C00.D48	Slovenia	6.01	0
C00.D48	Spain	44.37	0
C00.D48	Sweden	10.14	0
C00.D48	Switzerland	13.01	1
C00.D48	United Kingdom	72.90	1
C00.D48	Czech Republic	19.72	0
C00.D48	France	18.20	0
C00.D48	Germany	37.55	1
C00.D48	Netherlands	5.43	1
C00.D48	Poland	16.33	0
C00.D48	Romania	13.26	0
C00.D48	Spain	18.66	1
C00.D48	United Kingdom	12.74	0
C00.D48	Austria	469.33	2
C00.D48	Belgium	621.60	1
C00.D48	Croatia	355.04	1
C00.D48	Czech Republic	1346.12	2
C00.D48	Denmark	346.01	1
C00.D48	Estonia	132.05	1
C00.D48	Finland	383.39	1
C00.D48	France	3581.84	2
C00.D48	Germany	5198.87	2
C00.D48	Hungary	825.71	2
C00.D48	Latvia	223.56	0
C00.D48	Lithuania	314.57	0
C00.D48	Netherlands	1112.03	1
C00.D48	Norway	300.70	0
C00.D48	Poland	3214.05	1
C00.D48	Romania	817.78	1
C00.D48	Slovenia	157.74	1
C00.D48	Spain	2026.79	1
C00.D48	Sweden	603.90	3
C00.D48	Switzerland	350.08	1
C00.D48	United Kingdom	4030.98	2
C00.D48	Austria	20.70	2
C00.D48	Belgium	14.09	0
C00.D48	Czech Republic	91.38	2
C00.D48	Denmark	37.42	0
C00.D48	Estonia	6.73	1
C00.D48	Finland	18.35	0

For peer review only

C00.D48	France	18.65	0
C00.D48	Germany	188.08	1
C00.D48	Hungary	32.55	0
C00.D48	Lithuania	10.28	1
C00.D48	Netherlands	17.83	0
C00.D48	Norway	14.74	3
C00.D48	Poland	58.21	2
C00.D48	Romania	71.57	0
C00.D48	Spain	42.96	2
C00.D48	Sweden	50.26	2
C00.D48	Switzerland	30.44	1
C00.D48	United Kingdom	39.53	0
C00.D48	Austria	9.41	0
C00.D48	Belgium	26.60	0
C00.D48	Croatia	8.17	1
C00.D48	Czech Republic	23.00	1
C00.D48	Denmark	13.51	1
C00.D48	Finland	10.76	0
C00.D48	France	79.23	0
C00.D48	Germany	89.30	0
C00.D48	Hungary	19.07	0
C00.D48	Netherlands	23.93	0
C00.D48	Norway	11.85	0
C00.D48	Poland	42.84	1
C00.D48	Romania	34.40	1
C00.D48	Spain	43.31	1
C00.D48	Sweden	16.96	0
C00.D48	Switzerland	19.36	1
C00.D48	United Kingdom	167.86	1
C00.D48	Austria	570.44	2
C00.D48	Belgium	942.82	1
C00.D48	Croatia	434.26	2
C00.D48	Czech Republic	970.23	2
C00.D48	Denmark	623.45	1
C00.D48	Estonia	112.59	0
C00.D48	Finland	288.22	1
C00.D48	France	5240.88	1
C00.D48	Germany	5795.20	2
C00.D48	Hungary	1021.10	0
C00.D48	Latvia	216.68	2
C00.D48	Lithuania	274.20	1
C00.D48	Netherlands	1488.07	2
C00.D48	Norway	409.85	1
C00.D48	Poland	4157.07	0
C00.D48	Romania	1760.75	1
C00.D48	Slovenia	196.58	0
C00.D48	Spain	5261.15	3
C00.D48	Sweden	681.06	1
C00.D48	Switzerland	555.95	0
C00.D48	United Kingdom	5767.70	2

For peer review only

C00.D48	Austria	139.18	2
C00.D48	Belgium	107.88	2
C00.D48	Croatia	5.15	1
C00.D48	Czech Republic	39.90	1
C00.D48	Denmark	15.73	1
C00.D48	Finland	7.67	0
C00.D48	France	774.53	2
C00.D48	Germany	2156.94	3
C00.D48	Hungary	16.43	2
C00.D48	Netherlands	401.59	3
C00.D48	Norway	21.19	1
C00.D48	Poland	61.37	2
C00.D48	Romania	22.21	0
C00.D48	Spain	374.65	3
C00.D48	Sweden	41.12	0
C00.D48	Switzerland	62.63	0
C00.D48	United Kingdom	139.74	2
C00.D48	Austria	24.70	1
C00.D48	Belgium	20.33	0
C00.D48	Croatia	11.06	0
C00.D48	Czech Republic	28.97	1
C00.D48	Denmark	15.07	0
C00.D48	Estonia	5.76	0
C00.D48	Finland	26.59	0
C00.D48	France	160.68	0
C00.D48	Germany	210.44	3
C00.D48	Hungary	35.11	0
C00.D48	Latvia	8.27	2
C00.D48	Lithuania	13.85	0
C00.D48	Netherlands	33.46	0
C00.D48	Norway	8.01	2
C00.D48	Poland	122.04	2
C00.D48	Romania	69.55	0
C00.D48	Spain	73.29	0
C00.D48	Sweden	18.16	0
C00.D48	Switzerland	23.78	2
C00.D48	United Kingdom	106.10	0
C00.D48	Austria	8.91	0
C00.D48	Belgium	5.68	0
C00.D48	Croatia	12.21	2
C00.D48	Czech Republic	29.40	1
C00.D48	Denmark	6.92	0
C00.D48	France	26.17	1
C00.D48	Germany	110.82	3
C00.D48	Hungary	14.27	1
C00.D48	Lithuania	5.29	0
C00.D48	Netherlands	6.64	0
C00.D48	Norway	5.25	0
C00.D48	Poland	68.09	0
C00.D48	Romania	83.36	3

For peer review only

C00.D48	Slovenia	9.64	2
C00.D48	Spain	47.54	1
C00.D48	Sweden	6.87	1
C00.D48	Switzerland	5.41	2
C00.D48	United Kingdom	26.75	0
C00.D48	Austria	525.38	2
C00.D48	Belgium	620.15	0
C00.D48	Croatia	414.13	1
C00.D48	Czech Republic	776.57	1
C00.D48	Denmark	428.34	0
C00.D48	Estonia	99.54	0
C00.D48	Finland	323.65	0
C00.D48	France	3398.97	2
C00.D48	Germany	5460.44	0
C00.D48	Hungary	675.68	2
C00.D48	Latvia	167.98	2
C00.D48	Lithuania	248.65	2
C00.D48	Netherlands	938.20	2
C00.D48	Norway	333.24	0
C00.D48	Poland	3252.55	2
C00.D48	Romania	1554.64	2
C00.D48	Slovenia	140.07	0
C00.D48	Spain	2868.23	0
C00.D48	Sweden	586.26	3
C00.D48	Switzerland	492.74	0
C00.D48	United Kingdom	4087.68	3
C00.D48	Belgium	36.35	0
C00.D48	Croatia	8.38	0
C00.D48	Czech Republic	27.10	0
C00.D48	Denmark	8.56	1
C00.D48	Finland	5.34	1
C00.D48	France	74.59	1
C00.D48	Germany	34.99	3
C00.D48	Hungary	20.93	2
C00.D48	Lithuania	6.27	0
C00.D48	Netherlands	36.29	3
C00.D48	Poland	103.90	1
C00.D48	Romania	42.36	1
C00.D48	Spain	39.69	1
C00.D48	Sweden	6.73	3
C00.D48	United Kingdom	19.53	0
C00.D48	Austria	84.96	1
C00.D48	Belgium	84.17	0
C00.D48	Croatia	41.95	0
C00.D48	Czech Republic	104.84	1
C00.D48	Denmark	41.02	0
C00.D48	Estonia	18.69	0
C00.D48	Finland	53.88	0
C00.D48	France	449.10	1
C00.D48	Germany	758.98	2

For peer review only

C00.D48	Hungary	109.51	1
C00.D48	Latvia	31.23	0
C00.D48	Lithuania	34.29	1
C00.D48	Netherlands	121.77	0
C00.D48	Norway	42.25	0
C00.D48	Poland	365.73	2
C00.D48	Romania	188.22	0
C00.D48	Slovenia	18.58	1
C00.D48	Spain	328.20	0
C00.D48	Sweden	79.34	0
C00.D48	Switzerland	74.97	1
C00.D48	United Kingdom	397.53	0
C00.D48	Austria	19.90	0
C00.D48	Belgium	22.22	1
C00.D48	Croatia	16.71	0
C00.D48	Czech Republic	27.46	0
C00.D48	Denmark	13.17	2
C00.D48	Finland	14.17	0
C00.D48	France	109.02	1
C00.D48	Germany	139.88	0
C00.D48	Hungary	42.53	0
C00.D48	Latvia	9.76	0
C00.D48	Lithuania	11.51	0
C00.D48	Netherlands	39.28	0
C00.D48	Norway	10.98	0
C00.D48	Poland	121.16	1
C00.D48	Romania	66.42	2
C00.D48	Slovenia	7.85	0
C00.D48	Spain	107.99	0
C00.D48	Sweden	19.51	0
C00.D48	Switzerland	15.34	0
C00.D48	United Kingdom	122.87	0
C00.D48	Austria	9.66	3
C00.D48	Belgium	30.11	1
C00.D48	Croatia	6.67	0
C00.D48	Czech Republic	11.29	0
C00.D48	France	56.26	3
C00.D48	Germany	320.15	1
C00.D48	Hungary	15.38	0
C00.D48	Netherlands	61.02	2
C00.D48	Poland	40.57	0
C00.D48	Romania	42.02	0
C00.D48	Spain	82.73	2
C00.D48	Switzerland	31.08	1
C00.D48	United Kingdom	58.79	2
C00.D48	Austria	50.50	1
C00.D48	Belgium	302.33	0
C00.D48	Croatia	161.86	1
C00.D48	Czech Republic	166.51	2
C00.D48	Denmark	212.68	3

For peer review only

C00.D48	Estonia	5.20	0
C00.D48	Finland	74.86	1
C00.D48	France	3044.32	2
C00.D48	Germany	571.36	3
C00.D48	Hungary	148.95	2
C00.D48	Latvia	29.16	3
C00.D48	Lithuania	17.83	1
C00.D48	Netherlands	369.28	1
C00.D48	Norway	58.04	1
C00.D48	Poland	1137.81	0
C00.D48	Romania	315.25	0
C00.D48	Slovenia	23.14	2
C00.D48	Spain	663.90	1
C00.D48	Sweden	217.00	1
C00.D48	Switzerland	71.15	3
C00.D48	United Kingdom	632.16	1
C00.D48	Croatia	8.39	0
C00.D48	Croatia	112.11	2
C00.D48	Czech Republic	51.51	2
C00.D48	Finland	25.16	2
C00.D48	Latvia	14.46	2
C00.D48	Slovenia	58.16	0
C00.D48	Croatia	40.06	1
C00.D48	Czech Republic	25.23	0
C00.D48	Latvia	8.77	1
C00.D48	Slovenia	16.08	0
C00.D48	Austria	805.30	3
C00.D48	Belgium	1193.36	2
C00.D48	Croatia	437.90	0
C00.D48	Czech Republic	1191.43	0
C00.D48	Denmark	917.91	3
C00.D48	Estonia	104.51	1
C00.D48	Finland	361.44	1
C00.D48	France	8321.35	3
C00.D48	Germany	9806.38	0
C00.D48	Hungary	695.39	1
C00.D48	Latvia	175.96	1
C00.D48	Lithuania	224.07	2
C00.D48	Netherlands	2441.59	2
C00.D48	Norway	528.61	1
C00.D48	Poland	7576.47	3
C00.D48	Romania	1547.13	2
C00.D48	Slovenia	108.97	1
C00.D48	Spain	4848.20	1
C00.D48	Sweden	919.76	2
C00.D48	Switzerland	504.34	2
C00.D48	United Kingdom	13226.62	1
C00.D48	Austria	35.30	1
C00.D48	Belgium	55.90	1
C00.D48	Croatia	26.93	0

For peer review only

C00.D48	Czech Republic	76.69	2
C00.D48	Denmark	32.10	0
C00.D48	Estonia	7.76	0
C00.D48	Finland	23.99	2
C00.D48	France	299.70	1
C00.D48	Germany	344.19	1
C00.D48	Hungary	49.75	2
C00.D48	Latvia	14.79	0
C00.D48	Lithuania	22.14	1
C00.D48	Netherlands	91.10	0
C00.D48	Norway	17.80	0
C00.D48	Poland	299.59	3
C00.D48	Romania	146.88	1
C00.D48	Slovenia	11.53	0
C00.D48	Spain	251.90	1
C00.D48	Sweden	37.02	0
C00.D48	Switzerland	34.60	0
C00.D48	United Kingdom	339.75	0
C00.D48	Austria	18.97	1
C00.D48	Belgium	11.58	2
C00.D48	Croatia	5.50	1
C00.D48	Czech Republic	89.80	1
C00.D48	Denmark	20.62	0
C00.D48	Finland	50.14	1
C00.D48	France	128.05	1
C00.D48	Germany	130.04	2
C00.D48	Hungary	19.01	1
C00.D48	Lithuania	14.77	1
C00.D48	Norway	19.53	3
C00.D48	Poland	171.63	0
C00.D48	Romania	40.44	0
C00.D48	Slovenia	10.26	2
C00.D48	Spain	113.21	1
C00.D48	Sweden	29.55	0
C00.D48	Switzerland	23.36	2
C00.D48	United Kingdom	186.55	1
C00.D48	Austria	107.00	1
C00.D48	Belgium	71.89	2
C00.D48	Croatia	25.55	3
C00.D48	Czech Republic	236.37	1
C00.D48	Denmark	91.60	0
C00.D48	Estonia	39.44	2
C00.D48	Finland	359.13	2
C00.D48	France	768.60	1
C00.D48	Germany	687.71	2
C00.D48	Hungary	98.77	2
C00.D48	Latvia	22.97	2
C00.D48	Lithuania	88.83	1
C00.D48	Netherlands	64.83	3
C00.D48	Norway	60.18	3

For peer review only

C00.D48	Poland	517.07	1
C00.D48	Romania	157.91	1
C00.D48	Slovenia	29.17	3
C00.D48	Spain	412.80	2
C00.D48	Sweden	181.90	1
C00.D48	Switzerland	120.29	1
C00.D48	United Kingdom	796.15	1
C00.D48	Austria	46.00	1
C00.D48	Belgium	35.23	2
C00.D48	Czech Republic	46.67	1
C00.D48	Denmark	35.28	2
C00.D48	Estonia	5.41	0
C00.D48	Finland	41.82	0
C00.D48	France	247.15	1
C00.D48	Germany	365.79	2
C00.D48	Hungary	29.81	1
C00.D48	Latvia	6.59	3
C00.D48	Lithuania	15.21	1
C00.D48	Netherlands	63.29	1
C00.D48	Norway	24.68	0
C00.D48	Poland	95.71	1
C00.D48	Romania	15.87	1
C00.D48	Slovenia	9.37	1
C00.D48	Spain	139.75	1
C00.D48	Sweden	49.49	0
C00.D48	Switzerland	43.91	0
C00.D48	United Kingdom	302.92	1
C00.D48	Austria	456.97	1
C00.D48	Belgium	620.71	1
C00.D48	Croatia	255.02	0
C00.D48	Czech Republic	240.56	1
C00.D48	Denmark	232.07	2
C00.D48	Estonia	35.38	2
C00.D48	Finland	73.04	3
C00.D48	France	3549.46	2
C00.D48	Germany	4590.94	1
C00.D48	Hungary	403.28	2
C00.D48	Latvia	84.51	2
C00.D48	Lithuania	38.25	1
C00.D48	Netherlands	1222.77	2
C00.D48	Norway	264.34	2
C00.D48	Poland	1107.55	3
C00.D48	Romania	548.48	0
C00.D48	Slovenia	103.69	0
C00.D48	Spain	2073.90	3
C00.D48	Sweden	444.38	2
C00.D48	Switzerland	386.62	2
C00.D48	United Kingdom	3942.34	2
C00.D48	Austria	11.25	1
C00.D48	Belgium	37.10	0

For peer review only

C00.D48	Croatia	5.98	1
C00.D48	Czech Republic	23.45	0
C00.D48	Denmark	29.46	2
C00.D48	Finland	12.23	0
C00.D48	France	341.98	2
C00.D48	Germany	120.66	1
C00.D48	Hungary	11.72	0
C00.D48	Netherlands	60.51	2
C00.D48	Norway	14.85	0
C00.D48	Poland	51.81	0
C00.D48	Romania	17.59	0
C00.D48	Spain	84.47	2
C00.D48	Sweden	32.80	1
C00.D48	Switzerland	29.36	0
C00.D48	United Kingdom	128.01	1
C00.D48	Austria	362.29	0
C00.D48	Belgium	481.12	1
C00.D48	Croatia	175.92	1
C00.D48	Czech Republic	419.75	1
C00.D48	Denmark	251.94	0
C00.D48	Estonia	54.23	1
C00.D48	Finland	278.59	1
C00.D48	France	2741.28	1
C00.D48	Germany	3750.69	2
C00.D48	Hungary	310.90	2
C00.D48	Latvia	73.05	1
C00.D48	Lithuania	115.17	0
C00.D48	Netherlands	817.41	2
C00.D48	Norway	289.70	0
C00.D48	Poland	1470.37	3
C00.D48	Romania	367.44	1
C00.D48	Slovenia	104.31	0
C00.D48	Spain	1878.22	2
C00.D48	Sweden	483.06	1
C00.D48	Switzerland	356.41	3
C00.D48	United Kingdom	3063.03	2
C00.D48	Austria	301.41	2
C00.D48	Belgium	260.04	1
C00.D48	Croatia	178.44	1
C00.D48	Czech Republic	461.64	1
C00.D48	Denmark	206.49	1
C00.D48	Estonia	64.92	0
C00.D48	Finland	153.42	2
C00.D48	France	1663.61	1
C00.D48	Germany	2453.23	1
C00.D48	Hungary	445.86	2
C00.D48	Latvia	85.98	1
C00.D48	Lithuania	137.69	1
C00.D48	Netherlands	410.70	1
C00.D48	Norway	121.06	1

For peer review only

C00.D48	Poland	1573.64	2
C00.D48	Romania	619.66	0
C00.D48	Slovenia	84.31	0
C00.D48	Spain	1114.86	1
C00.D48	Sweden	287.12	1
C00.D48	Switzerland	218.47	1
C00.D48	United Kingdom	1627.19	2
C00.D48	Austria	413.86	0
C00.D48	Belgium	450.07	0
C00.D48	Croatia	175.38	0
C00.D48	Czech Republic	454.20	1
C00.D48	Denmark	260.97	0
C00.D48	Estonia	61.37	0
C00.D48	Finland	189.20	2
C00.D48	France	2492.26	1
C00.D48	Germany	3729.37	3
C00.D48	Hungary	477.63	2
C00.D48	Latvia	66.89	1
C00.D48	Lithuania	134.91	0
C00.D48	Netherlands	606.45	0
C00.D48	Norway	192.81	0
C00.D48	Poland	1558.72	1
C00.D48	Romania	554.82	1
C00.D48	Slovenia	81.91	1
C00.D48	Spain	1504.17	0
C00.D48	Sweden	356.99	0
C00.D48	Switzerland	310.32	1
C00.D48	United Kingdom	3092.03	0
C00.D48	Austria	6.37	3
C00.D48	Belgium	9.17	2
C00.D48	Czech Republic	22.72	2
C00.D48	Denmark	7.82	1
C00.D48	Finland	7.34	0
C00.D48	France	133.66	2
C00.D48	Germany	85.71	2
C00.D48	Hungary	17.42	2
C00.D48	Lithuania	11.97	1
C00.D48	Netherlands	10.16	3
C00.D48	Poland	49.63	2
C00.D48	Romania	25.92	1
C00.D48	Spain	54.60	2
C00.D48	Sweden	16.37	2
C00.D48	Switzerland	11.16	2
C00.D48	United Kingdom	48.14	3
C00.D48	Czech Republic	36.32	2
C00.D48	Denmark	8.27	3
C00.D48	France	31.15	2
C00.D48	Germany	33.00	1
C00.D48	Hungary	8.34	1
C00.D48	Lithuania	6.88	1

For peer review only

C00.D48	Poland	55.60	1
C00.D48	Romania	27.34	0
C00.D48	Spain	16.51	0
C00.D48	United Kingdom	15.78	1
C00.D48	Austria	74.51	1
C00.D48	Belgium	274.53	0
C00.D48	Croatia	52.13	3
C00.D48	Czech Republic	84.91	1
C00.D48	Denmark	53.12	0
C00.D48	Estonia	8.39	0
C00.D48	Finland	18.01	0
C00.D48	France	1711.85	2
C00.D48	Germany	1055.90	2
C00.D48	Hungary	133.07	2
C00.D48	Latvia	32.88	1
C00.D48	Lithuania	16.46	3
C00.D48	Netherlands	426.60	0
C00.D48	Norway	46.32	0
C00.D48	Poland	228.97	3
C00.D48	Romania	199.77	1
C00.D48	Slovenia	17.77	2
C00.D48	Spain	856.40	3
C00.D48	Sweden	84.87	1
C00.D48	Switzerland	57.28	1
C00.D48	United Kingdom	278.12	1
C00.D48	Belgium	17.24	2
C00.D48	Czech Republic	53.03	2
C00.D48	Denmark	8.38	0
C00.D48	France	138.25	0
C00.D48	Germany	52.68	2
C00.D48	Hungary	88.24	1
C00.D48	Netherlands	15.88	2
C00.D48	Norway	5.32	0
C00.D48	Poland	137.40	1
C00.D48	Romania	40.65	1
C00.D48	Slovenia	26.32	1
C00.D48	Spain	53.94	1
C00.D48	Sweden	14.25	0
C00.D48	Switzerland	5.21	0
C00.D48	United Kingdom	29.71	2
C00.D48	Czech Republic	160.51	0
C00.D48	Slovenia	74.71	0
C00.D48	Switzerland	218.64	2
C00.D48	Germany	8.15	1
C00.D48	Romania	12.74	1
C00.D48	Romania	5.47	1
C00.D48	Romania	7.51	0
C00.D48	Germany	5.21	0
C00.D48	Romania	30.30	3
C00.D48	Romania	9.89	0

For peer review only

C00.D48	Denmark	7.50	0
C00.D48	France	12.65	0
C00.D48	Germany	14.81	0
C00.D48	Hungary	7.02	0
C00.D48	Netherlands	12.61	2
C00.D48	Poland	16.90	0
C00.D48	Spain	19.42	2
C00.D48	United Kingdom	14.04	1
C00.D48	France	17.54	2
C00.D48	Germany	22.25	0
C00.D48	Hungary	7.21	1
C00.D48	Poland	28.70	0
C00.D48	Romania	7.23	1
C00.D48	Spain	16.90	0
C00.D48	United Kingdom	22.73	1
C00.D48	France	5.11	0
C00.D48	Poland	6.49	0
C00.D48	Belgium	5.17	0
C00.D48	France	23.78	0
C00.D48	Germany	12.25	0
C00.D48	Spain	15.29	0
C00.D48	United Kingdom	20.09	0
C00.D48	Austria	5.66	0
C00.D48	Belgium	9.82	0
C00.D48	France	57.21	1
C00.D48	Germany	28.98	0
C00.D48	Netherlands	7.05	0
C00.D48	Poland	9.88	1
C00.D48	Spain	27.95	0
C00.D48	United Kingdom	17.64	0
C00.D48	Germany	6.16	1
C00.D48	France	8.19	2
C00.D48	Germany	5.38	0
C00.D48	Spain	5.46	0
C00.D48	Germany	6.19	0
C00.D48	Germany	5.27	0
C00.D48	United Kingdom	7.58	0
C00.D48	Germany	5.65	1
C00.D48	United Kingdom	6.95	0
C00.D48	Germany	11.91	2
C00.D48	Denmark	6.10	0
C00.D48	Poland	7.15	1
C00.D48	Spain	6.43	3
C00.D48	Austria	68.87	0
C00.D48	Belgium	59.57	0
C00.D48	Croatia	30.49	3
C00.D48	Czech Republic	21.87	0
C00.D48	Denmark	21.10	0
C00.D48	Estonia	7.20	0
C00.D48	Finland	47.68	1

For peer review only

C00.D48	France	412.89	1
C00.D48	Germany	284.09	1
C00.D48	Hungary	56.85	0
C00.D48	Latvia	12.32	0
C00.D48	Lithuania	22.98	0
C00.D48	Netherlands	89.43	0
C00.D48	Norway	21.18	1
C00.D48	Poland	50.29	1
C00.D48	Romania	16.36	1
C00.D48	Slovenia	8.68	2
C00.D48	Spain	292.81	1
C00.D48	Sweden	52.72	1
C00.D48	Switzerland	35.95	1
C00.D48	United Kingdom	384.88	0
C00.D48	Austria	5.37	0
C00.D48	Belgium	10.52	1
C00.D48	Czech Republic	18.65	0
C00.D48	Denmark	18.23	1
C00.D48	Finland	8.25	0
C00.D48	France	52.03	1
C00.D48	Germany	23.51	0
C00.D48	Hungary	19.04	2
C00.D48	Lithuania	10.92	1
C00.D48	Netherlands	6.69	0
C00.D48	Norway	8.50	1
C00.D48	Poland	75.02	0
C00.D48	Romania	17.55	2
C00.D48	Spain	29.14	1
C00.D48	Sweden	5.13	1
C00.D48	United Kingdom	28.51	0
C00.D48	Austria	12.78	1
C00.D48	Belgium	6.56	0
C00.D48	Czech Republic	5.62	0
C00.D48	Finland	5.38	0
C00.D48	France	50.69	0
C00.D48	Germany	32.05	1
C00.D48	Hungary	11.53	0
C00.D48	Lithuania	5.52	1
C00.D48	Netherlands	7.00	2
C00.D48	Poland	12.76	0
C00.D48	Spain	42.85	0
C00.D48	Switzerland	6.20	0
C00.D48	United Kingdom	36.40	1
C00.D48	France	23.15	0
C00.D48	Germany	14.22	3
C00.D48	Spain	9.30	0
C00.D48	United Kingdom	14.72	0
C00.D48	Austria	62.61	3
C00.D48	Belgium	224.18	3
C00.D48	Czech Republic	32.07	1

For peer review only

C00.D48	Denmark	38.92	1
C00.D48	Finland	55.52	0
C00.D48	France	1426.24	3
C00.D48	Germany	1007.20	2
C00.D48	Hungary	150.63	0
C00.D48	Lithuania	6.69	1
C00.D48	Netherlands	273.84	2
C00.D48	Norway	40.25	2
C00.D48	Poland	889.53	2
C00.D48	Romania	24.63	2
C00.D48	Spain	445.46	1
C00.D48	Sweden	181.90	0
C00.D48	Switzerland	76.24	0
C00.D48	United Kingdom	448.33	1
C00.D48	Austria	27.70	3
C00.D48	Belgium	109.20	3
C00.D48	Czech Republic	25.79	1
C00.D48	Denmark	44.94	2
C00.D48	Finland	27.10	0
C00.D48	France	518.56	0
C00.D48	Germany	374.70	1
C00.D48	Hungary	112.67	0
C00.D48	Netherlands	151.08	2
C00.D48	Norway	30.50	0
C00.D48	Poland	1006.96	2
C00.D48	Romania	16.48	0
C00.D48	Spain	184.75	1
C00.D48	Sweden	101.38	0
C00.D48	Switzerland	35.69	0
C00.D48	United Kingdom	158.66	1
C00.D48	Austria	5.66	0
C00.D48	Belgium	16.05	0
C00.D48	Czech Republic	5.45	0
C00.D48	Finland	8.47	0
C00.D48	France	103.34	0
C00.D48	Germany	89.62	1
C00.D48	Hungary	16.85	0
C00.D48	Netherlands	14.75	1
C00.D48	Poland	70.20	1
C00.D48	Spain	44.67	3
C00.D48	Sweden	13.60	0
C00.D48	Switzerland	9.04	0
C00.D48	United Kingdom	40.13	1
C00.D48	Belgium	5.40	2
C00.D48	France	28.45	1
C00.D48	Germany	46.14	2
C00.D48	Hungary	21.72	0
C00.D48	Poland	22.14	0
C00.D48	Spain	19.11	0
C00.D48	Austria	30.43	1

For peer review only

C00.D48	Belgium	76.18	0
C00.D48	Czech Republic	6.38	1
C00.D48	Denmark	16.21	1
C00.D48	Finland	11.44	1
C00.D48	France	518.12	2
C00.D48	Germany	268.23	2
C00.D48	Hungary	30.29	0
C00.D48	Netherlands	77.07	2
C00.D48	Norway	10.54	0
C00.D48	Poland	149.47	2
C00.D48	Romania	12.85	1
C00.D48	Spain	314.25	0
C00.D48	Sweden	32.20	0
C00.D48	Switzerland	15.98	0
C00.D48	United Kingdom	130.31	1
C00.D48	France	5.38	0
C00.D48	Germany	25.05	1
C00.D48	Poland	16.59	0
C00.D48	Spain	25.22	3
C00.D48	Austria	60.23	0
C00.D48	Belgium	164.31	0
C00.D48	Czech Republic	15.83	1
C00.D48	Denmark	44.16	3
C00.D48	Estonia	7.06	1
C00.D48	Finland	11.49	1
C00.D48	France	1411.27	3
C00.D48	Germany	609.86	2
C00.D48	Hungary	50.95	0
C00.D48	Latvia	10.03	0
C00.D48	Lithuania	8.92	0
C00.D48	Netherlands	373.98	2
C00.D48	Norway	34.27	1
C00.D48	Poland	472.70	0
C00.D48	Romania	24.18	0
C00.D48	Slovenia	7.00	2
C00.D48	Spain	700.21	1
C00.D48	Sweden	154.89	2
C00.D48	Switzerland	35.83	0
C00.D48	United Kingdom	759.27	1
C00.D48	Austria	7.55	0
C00.D48	Belgium	12.18	0
C00.D48	France	68.78	1
C00.D48	Germany	72.71	1
C00.D48	Hungary	6.25	1
C00.D48	Netherlands	22.13	2
C00.D48	Norway	5.71	0
C00.D48	Poland	31.30	1
C00.D48	Spain	44.18	0
C00.D48	Sweden	8.32	0
C00.D48	Switzerland	6.45	0

For peer review only

C00.D48	United Kingdom	59.63	1
C00.D48	Austria	29.84	0
C00.D48	Belgium	21.86	1
C00.D48	Croatia	9.04	0
C00.D48	Czech Republic	15.16	1
C00.D48	Denmark	18.07	1
C00.D48	Finland	16.33	0
C00.D48	France	144.41	1
C00.D48	Germany	125.84	0
C00.D48	Hungary	22.96	1
C00.D48	Lithuania	7.06	0
C00.D48	Netherlands	23.67	0
C00.D48	Norway	9.10	0
C00.D48	Poland	70.30	0
C00.D48	Spain	51.76	1
C00.D48	Sweden	23.14	0
C00.D48	Switzerland	21.39	0
C00.D48	United Kingdom	56.35	2
C00.D48	Austria	219.84	2
C00.D48	Belgium	221.62	1
C00.D48	Croatia	72.50	3
C00.D48	Czech Republic	176.62	1
C00.D48	Denmark	104.39	1
C00.D48	Estonia	9.55	3
C00.D48	Finland	84.73	2
C00.D48	France	1146.26	2
C00.D48	Germany	1979.31	1
C00.D48	Hungary	178.24	0
C00.D48	Latvia	25.57	2
C00.D48	Lithuania	38.87	2
C00.D48	Netherlands	327.41	2
C00.D48	Norway	68.70	0
C00.D48	Poland	686.69	1
C00.D48	Romania	38.02	1
C00.D48	Slovenia	30.20	2
C00.D48	Spain	937.25	2
C00.D48	Sweden	140.75	0
C00.D48	Switzerland	173.76	2
C00.D48	United Kingdom	1277.94	2
C00.D48	Austria	48.52	1
C00.D48	Belgium	57.23	1
C00.D48	Croatia	64.18	1
C00.D48	Czech Republic	44.69	2
C00.D48	Denmark	49.68	0
C00.D48	Estonia	9.99	0
C00.D48	Finland	37.52	0
C00.D48	France	584.53	1
C00.D48	Germany	328.32	2
C00.D48	Hungary	76.19	0
C00.D48	Latvia	16.50	1

For peer review only

C00.D48	Lithuania	17.09	0
C00.D48	Netherlands	70.12	2
C00.D48	Norway	22.84	2
C00.D48	Poland	131.24	2
C00.D48	Romania	45.34	0
C00.D48	Slovenia	12.30	1
C00.D48	Spain	455.59	2
C00.D48	Sweden	59.19	0
C00.D48	Switzerland	52.47	0
C00.D48	United Kingdom	524.53	2
C00.D48	Austria	33.10	1
C00.D48	Belgium	92.94	3
C00.D48	Czech Republic	16.74	1
C00.D48	Denmark	39.78	0
C00.D48	Finland	12.83	0
C00.D48	France	495.53	1
C00.D48	Germany	863.01	2
C00.D48	Hungary	34.19	3
C00.D48	Netherlands	260.00	0
C00.D48	Norway	23.59	1
C00.D48	Poland	386.66	0
C00.D48	Romania	76.53	2
C00.D48	Spain	291.16	2
C00.D48	Sweden	85.14	2
C00.D48	Switzerland	78.67	0
C00.D48	United Kingdom	161.20	1
D50.D89	Belgium	20.44	1
D50.D89	Croatia	6.11	3
D50.D89	Czech Republic	11.76	3
D50.D89	Denmark	23.33	0
D50.D89	France	92.87	0
D50.D89	Germany	117.66	2
D50.D89	Hungary	7.44	0
D50.D89	Netherlands	28.79	2
D50.D89	Norway	11.01	1
D50.D89	Poland	24.31	2
D50.D89	Romania	10.61	0
D50.D89	Slovenia	5.50	0
D50.D89	Spain	146.99	0
D50.D89	Sweden	18.39	0
D50.D89	Switzerland	14.49	0
D50.D89	United Kingdom	42.42	0
D50.D89	Denmark	13.35	0
D50.D89	France	27.93	1
D50.D89	Germany	27.60	1
D50.D89	Hungary	15.09	0
D50.D89	Poland	33.67	1
D50.D89	Spain	14.29	0
D50.D89	Sweden	5.29	0
D50.D89	United Kingdom	17.89	1

For peer review only

D50.D89	France	17.96	1
D50.D89	Germany	14.46	1
D50.D89	Spain	19.06	2
D50.D89	France	34.96	0
D50.D89	Germany	28.14	0
D50.D89	Hungary	5.16	1
D50.D89	Netherlands	49.10	1
D50.D89	Poland	12.29	2
D50.D89	Romania	6.47	0
D50.D89	Spain	111.53	2
D50.D89	France	5.38	0
D50.D89	Germany	5.05	0
D50.D89	Spain	6.34	0
D50.D89	France	14.11	1
D50.D89	Germany	5.29	1
D50.D89	United Kingdom	20.61	0
D50.D89	France	11.48	3
D50.D89	Germany	24.91	0
D50.D89	Spain	20.94	0
D50.D89	Sweden	8.53	0
D50.D89	United Kingdom	15.51	1
D50.D89	Belgium	9.56	0
D50.D89	Czech Republic	7.35	0
D50.D89	Denmark	11.18	0
D50.D89	Finland	7.00	1
D50.D89	France	65.09	1
D50.D89	Germany	56.76	0
D50.D89	Hungary	10.14	0
D50.D89	Netherlands	14.76	0
D50.D89	Norway	5.80	0
D50.D89	Poland	31.78	1
D50.D89	Romania	6.70	0
D50.D89	Spain	36.32	1
D50.D89	Switzerland	8.18	0
D50.D89	United Kingdom	45.53	0
D50.D89	Germany	6.55	0
D50.D89	Poland	6.95	1
D50.D89	Romania	9.54	1
D50.D89	Austria	34.63	0
D50.D89	Belgium	50.69	1
D50.D89	Croatia	8.25	1
D50.D89	Czech Republic	13.04	0
D50.D89	Denmark	16.46	0
D50.D89	Finland	10.81	0
D50.D89	France	362.44	2
D50.D89	Germany	319.98	0
D50.D89	Hungary	35.62	2
D50.D89	Latvia	6.67	0
D50.D89	Lithuania	8.33	0
D50.D89	Netherlands	50.50	2

For peer review only

D50.D89	Norway	18.04	2
D50.D89	Poland	164.49	1
D50.D89	Romania	26.92	0
D50.D89	Spain	245.20	3
D50.D89	Sweden	19.32	1
D50.D89	Switzerland	25.77	0
D50.D89	United Kingdom	162.16	3
D50.D89	Germany	44.53	2
D50.D89	Austria	23.23	1
D50.D89	Belgium	123.37	0
D50.D89	Czech Republic	23.83	1
D50.D89	Denmark	127.41	1
D50.D89	Finland	6.15	1
D50.D89	France	692.71	2
D50.D89	Germany	577.43	2
D50.D89	Hungary	48.39	2
D50.D89	Netherlands	308.82	1
D50.D89	Norway	51.73	0
D50.D89	Poland	74.16	3
D50.D89	Romania	23.58	3
D50.D89	Spain	449.37	3
D50.D89	Sweden	121.45	0
D50.D89	Switzerland	48.65	0
D50.D89	United Kingdom	288.77	3
D50.D89	Belgium	15.86	2
D50.D89	Czech Republic	8.82	1
D50.D89	France	105.75	1
D50.D89	Germany	36.93	0
D50.D89	Netherlands	5.19	1
D50.D89	Poland	36.80	1
D50.D89	Romania	12.51	1
D50.D89	Spain	37.45	2
D50.D89	United Kingdom	43.52	1
D50.D89	France	10.39	0
D50.D89	Germany	12.62	0
D50.D89	Hungary	5.84	0
D50.D89	Poland	5.09	1
D50.D89	Spain	5.35	1
D50.D89	United Kingdom	17.46	0
D50.D89	Belgium	45.12	0
D50.D89	Czech Republic	21.62	2
D50.D89	Denmark	10.87	1
D50.D89	France	148.78	2
D50.D89	Germany	426.54	3
D50.D89	Hungary	15.09	2
D50.D89	Netherlands	18.32	0
D50.D89	Norway	5.22	0
D50.D89	Poland	25.43	0
D50.D89	Spain	93.72	1
D50.D89	Sweden	10.61	0

For peer review only

D50.D89	Switzerland	8.70	0
D50.D89	United Kingdom	47.53	0
D50.D89	Austria	21.53	0
D50.D89	Belgium	28.38	0
D50.D89	Croatia	5.66	0
D50.D89	Czech Republic	27.09	1
D50.D89	Denmark	14.21	0
D50.D89	Finland	7.20	2
D50.D89	France	224.15	1
D50.D89	Germany	203.70	0
D50.D89	Hungary	26.78	0
D50.D89	Latvia	5.24	0
D50.D89	Netherlands	33.95	1
D50.D89	Norway	11.27	0
D50.D89	Poland	74.75	1
D50.D89	Romania	15.43	0
D50.D89	Spain	129.89	0
D50.D89	Sweden	12.35	2
D50.D89	Switzerland	15.04	0
D50.D89	United Kingdom	106.80	1
D50.D89	Austria	5.24	1
D50.D89	Belgium	18.26	0
D50.D89	France	61.15	2
D50.D89	Germany	86.34	1
D50.D89	Hungary	8.87	0
D50.D89	Netherlands	7.88	0
D50.D89	Norway	6.54	2
D50.D89	Poland	23.90	2
D50.D89	Spain	46.86	2
D50.D89	Sweden	8.07	0
D50.D89	United Kingdom	151.29	2
D50.D89	France	13.69	0
D50.D89	Germany	13.26	0
D50.D89	Spain	9.06	0
D50.D89	France	24.67	3
D50.D89	Germany	31.95	0
D50.D89	Hungary	5.65	0
D50.D89	Netherlands	8.41	0
D50.D89	Poland	7.15	0
D50.D89	Romania	7.48	1
D50.D89	Spain	27.45	1
D50.D89	United Kingdom	37.60	2
D50.D89	Austria	23.62	2
D50.D89	Belgium	15.26	1
D50.D89	Czech Republic	9.95	1
D50.D89	France	145.80	3
D50.D89	Germany	178.98	2
D50.D89	Hungary	13.72	1
D50.D89	Netherlands	23.52	1
D50.D89	Poland	24.04	1

For peer review only

D50.D89	Spain	47.39	1
D50.D89	Sweden	11.30	1
D50.D89	Switzerland	17.04	1
D50.D89	United Kingdom	18.08	1
D50.D89	France	53.18	2
D50.D89	Germany	14.84	0
D50.D89	Netherlands	6.38	1
D50.D89	Spain	19.31	1
D50.D89	Sweden	6.16	1
D50.D89	United Kingdom	32.24	3
D50.D89	Germany	10.74	1
D50.D89	United Kingdom	10.14	0
D50.D89	Germany	5.65	0
D50.D89	Belgium	22.45	0
D50.D89	France	122.02	1
D50.D89	Germany	36.58	0
D50.D89	Hungary	6.42	1
D50.D89	Norway	6.05	0
D50.D89	Spain	59.29	3
D50.D89	Sweden	7.55	0
D50.D89	United Kingdom	27.73	0
D50.D89	Austria	14.79	2
D50.D89	Belgium	15.51	0
D50.D89	Czech Republic	8.12	2
D50.D89	Denmark	20.52	0
D50.D89	Finland	14.84	0
D50.D89	France	89.81	0
D50.D89	Germany	129.32	1
D50.D89	Hungary	6.66	0
D50.D89	Netherlands	32.22	2
D50.D89	Norway	16.00	0
D50.D89	Poland	21.28	0
D50.D89	Spain	38.98	1
D50.D89	Sweden	24.92	0
D50.D89	Switzerland	15.17	0
D50.D89	United Kingdom	161.13	1
D50.D89	Finland	5.26	1
D50.D89	France	25.48	3
D50.D89	Germany	59.98	2
D50.D89	Netherlands	10.64	0
D50.D89	Spain	13.66	1
D50.D89	Sweden	10.55	2
D50.D89	United Kingdom	30.60	0
E00.E90	Belgium	29.41	1
E00.E90	Czech Republic	13.05	2
E00.E90	Denmark	35.81	0
E00.E90	France	376.42	2
E00.E90	Germany	66.80	3
E00.E90	Hungary	14.97	0
E00.E90	Netherlands	52.44	1

For peer review only

E00.E90	Norway	23.27	0
E00.E90	Poland	30.72	2
E00.E90	Spain	137.02	2
E00.E90	Sweden	24.65	2
E00.E90	Switzerland	12.13	0
E00.E90	United Kingdom	266.26	1
E00.E90	Belgium	9.20	1
E00.E90	France	53.98	3
E00.E90	Germany	44.88	1
E00.E90	Hungary	11.55	0
E00.E90	Netherlands	14.49	0
E00.E90	Poland	6.49	3
E00.E90	Spain	33.39	0
E00.E90	Sweden	6.24	0
E00.E90	United Kingdom	22.05	0
E00.E90	Belgium	47.74	1
E00.E90	Czech Republic	10.93	1
E00.E90	Denmark	33.92	0
E00.E90	France	270.36	2
E00.E90	Germany	196.58	2
E00.E90	Hungary	29.79	2
E00.E90	Latvia	7.08	0
E00.E90	Netherlands	36.83	2
E00.E90	Norway	6.39	0
E00.E90	Poland	105.17	1
E00.E90	Romania	7.51	1
E00.E90	Spain	71.23	0
E00.E90	Sweden	13.45	1
E00.E90	Switzerland	9.72	1
E00.E90	United Kingdom	84.30	2
E00.E90	France	7.55	0
E00.E90	Germany	8.09	0
E00.E90	Belgium	8.71	3
E00.E90	France	56.87	1
E00.E90	Germany	12.97	0
E00.E90	Netherlands	7.93	1
E00.E90	Spain	5.64	0
E00.E90	United Kingdom	6.40	1
E00.E90	Austria	332.22	3
E00.E90	Belgium	163.52	1
E00.E90	Croatia	100.24	1
E00.E90	Czech Republic	1177.68	0
E00.E90	Denmark	278.21	1
E00.E90	Estonia	45.06	3
E00.E90	Finland	95.63	0
E00.E90	France	3046.28	1
E00.E90	Germany	969.94	2
E00.E90	Hungary	755.56	0
E00.E90	Latvia	40.97	1
E00.E90	Lithuania	84.44	1

For peer review only

E00.E90	Netherlands	324.34	2
E00.E90	Norway	82.61	2
E00.E90	Poland	1681.84	0
E00.E90	Romania	717.13	3
E00.E90	Slovenia	40.01	1
E00.E90	Spain	633.13	2
E00.E90	Sweden	77.23	1
E00.E90	Switzerland	61.07	2
E00.E90	United Kingdom	493.96	3
E00.E90	Austria	915.71	3
E00.E90	Belgium	391.17	2
E00.E90	Croatia	293.00	2
E00.E90	Czech Republic	1513.73	2
E00.E90	Denmark	634.73	2
E00.E90	Estonia	103.33	3
E00.E90	Finland	469.87	2
E00.E90	France	3573.42	3
E00.E90	Germany	7613.03	2
E00.E90	Hungary	1514.19	1
E00.E90	Latvia	342.02	3
E00.E90	Lithuania	220.26	1
E00.E90	Netherlands	886.29	2
E00.E90	Norway	183.88	3
E00.E90	Poland	5319.63	1
E00.E90	Romania	1393.68	1
E00.E90	Slovenia	230.28	0
E00.E90	Spain	2224.42	3
E00.E90	Sweden	472.85	0
E00.E90	Switzerland	956.07	2
E00.E90	United Kingdom	2359.69	2
E00.E90	Czech Republic	7.09	1
E00.E90	Germany	13.99	1
E00.E90	Poland	5.00	2
E00.E90	Spain	7.26	0
E00.E90	Czech Republic	16.44	2
E00.E90	Germany	40.32	2
E00.E90	Hungary	19.41	2
E00.E90	Poland	18.80	0
E00.E90	Austria	2326.93	3
E00.E90	Belgium	1298.12	2
E00.E90	Croatia	1086.58	3
E00.E90	Czech Republic	71.83	0
E00.E90	Denmark	373.88	3
E00.E90	Estonia	60.45	1
E00.E90	Finland	7.59	0
E00.E90	France	5649.77	1
E00.E90	Germany	15850.80	1
E00.E90	Hungary	940.47	3
E00.E90	Latvia	53.96	1
E00.E90	Lithuania	36.12	2

For peer review only

E00.E90	Netherlands	3158.20	3
E00.E90	Norway	561.18	2
E00.E90	Poland	1765.40	2
E00.E90	Romania	659.55	3
E00.E90	Slovenia	264.93	2
E00.E90	Spain	8377.34	1
E00.E90	Sweden	1436.72	1
E00.E90	Switzerland	652.69	3
E00.E90	United Kingdom	4537.20	3
E00.E90	Belgium	9.02	0
E00.E90	France	34.84	0
E00.E90	Germany	14.34	0
E00.E90	Netherlands	5.07	0
E00.E90	Poland	6.78	3
E00.E90	Spain	17.30	0
E00.E90	Belgium	14.97	1
E00.E90	France	52.14	0
E00.E90	Germany	47.81	1
E00.E90	Netherlands	12.20	0
E00.E90	Poland	6.88	0
E00.E90	Spain	39.75	2
E00.E90	United Kingdom	23.83	0
E00.E90	Denmark	5.07	0
E00.E90	France	26.61	0
E00.E90	Germany	18.37	3
E00.E90	Netherlands	7.89	2
E00.E90	Spain	11.56	0
E00.E90	Sweden	7.12	0
E00.E90	Switzerland	5.31	0
E00.E90	United Kingdom	28.11	0
E00.E90	Belgium	5.16	0
E00.E90	France	20.17	1
E00.E90	Germany	6.06	0
E00.E90	Spain	10.91	0
E00.E90	United Kingdom	12.43	0
E00.E90	France	31.29	2
E00.E90	Germany	16.18	0
E00.E90	Netherlands	8.64	0
E00.E90	Poland	5.69	0
E00.E90	Romania	6.15	2
E00.E90	Spain	21.09	0
E00.E90	United Kingdom	17.24	1
E00.E90	France	9.28	0
E00.E90	Germany	7.73	0
E00.E90	Spain	8.94	0
E00.E90	United Kingdom	5.94	1
E00.E90	Austria	5.12	0
E00.E90	Belgium	7.09	0
E00.E90	Denmark	6.89	0
E00.E90	France	73.34	1

For peer review only

E00.E90	Germany	34.40	0
E00.E90	Hungary	8.07	1
E00.E90	Netherlands	12.42	0
E00.E90	Norway	5.04	0
E00.E90	Poland	14.81	1
E00.E90	Spain	41.47	2
E00.E90	Sweden	6.17	1
E00.E90	Switzerland	5.85	0
E00.E90	United Kingdom	38.42	1
E00.E90	France	5.96	1
E00.E90	Germany	6.35	1
E00.E90	Poland	6.92	0
E00.E90	Spain	6.14	1
E00.E90	United Kingdom	20.88	1
E00.E90	Belgium	34.35	1
E00.E90	Czech Republic	28.47	1
E00.E90	Denmark	28.04	1
E00.E90	France	237.92	1
E00.E90	Germany	171.16	3
E00.E90	Netherlands	7.84	3
E00.E90	Poland	102.71	2
E00.E90	Romania	33.17	1
E00.E90	Sweden	13.99	1
E00.E90	Switzerland	7.39	0
E00.E90	United Kingdom	8.09	0
E00.E90	Belgium	9.25	1
E00.E90	Czech Republic	44.54	3
E00.E90	France	338.75	3
E00.E90	Hungary	8.65	3
E00.E90	Netherlands	82.91	2
E00.E90	Poland	7.28	0
E00.E90	Romania	36.37	1
E00.E90	Spain	32.61	3
E00.E90	United Kingdom	8.71	0
E00.E90	Belgium	136.62	2
E00.E90	France	2308.57	0
E00.E90	Germany	36.99	2
E00.E90	Hungary	6.28	1
E00.E90	Netherlands	19.95	0
E00.E90	Poland	7.31	2
E00.E90	Romania	17.29	0
E00.E90	Spain	133.97	1
E00.E90	Sweden	43.76	2
E00.E90	Switzerland	27.75	2
E00.E90	United Kingdom	81.65	0
E00.E90	France	12.42	0
E00.E90	Germany	28.66	2
E00.E90	Spain	12.80	0
E00.E90	United Kingdom	5.84	0
E00.E90	France	5.79	2

For peer review only

E00.E90	Germany	8.96	0
E00.E90	United Kingdom	13.11	1
E00.E90	Denmark	5.32	1
E00.E90	Austria	652.00	3
E00.E90	Belgium	134.09	2
E00.E90	Croatia	8.82	2
E00.E90	Czech Republic	34.11	3
E00.E90	Denmark	86.09	1
E00.E90	Estonia	7.61	0
E00.E90	Finland	29.68	1
E00.E90	France	1042.62	3
E00.E90	Germany	1489.35	2
E00.E90	Hungary	110.26	2
E00.E90	Latvia	7.37	2
E00.E90	Lithuania	6.62	0
E00.E90	Netherlands	93.31	2
E00.E90	Norway	26.22	1
E00.E90	Poland	39.36	3
E00.E90	Slovenia	14.74	3
E00.E90	Spain	669.40	2
E00.E90	Sweden	56.74	1
E00.E90	Switzerland	75.06	1
E00.E90	United Kingdom	381.59	1
E00.E90	France	10.51	1
E00.E90	Germany	16.47	1
E00.E90	Spain	8.22	2
E00.E90	United Kingdom	10.66	2
E00.E90	Belgium	20.34	2
E00.E90	France	40.39	0
E00.E90	Germany	13.15	0
E00.E90	Spain	9.61	1
E00.E90	United Kingdom	15.12	0
E00.E90	France	6.39	0
E00.E90	Germany	12.22	0
E00.E90	Hungary	6.83	2
E00.E90	United Kingdom	8.26	0
E00.E90	Austria	6.36	0
E00.E90	Belgium	7.26	1
E00.E90	Finland	8.34	0
E00.E90	France	41.27	0
E00.E90	Germany	53.25	1
E00.E90	Netherlands	15.39	0
E00.E90	Poland	13.57	0
E00.E90	Spain	25.45	0
E00.E90	Sweden	8.34	0
E00.E90	Switzerland	5.41	0
E00.E90	United Kingdom	44.95	0
E00.E90	France	9.34	0
E00.E90	Germany	13.25	0
E00.E90	Poland	6.17	0

For peer review only

E00.E90	Spain	9.70	0
E00.E90	United Kingdom	13.10	0
E00.E90	Finland	5.12	0
E00.E90	Germany	6.94	0
E00.E90	Spain	7.92	0
E00.E90	United Kingdom	8.57	1
E00.E90	Austria	91.14	2
E00.E90	Belgium	52.41	0
E00.E90	Czech Republic	14.84	2
E00.E90	Denmark	55.93	3
E00.E90	France	430.05	1
E00.E90	Germany	307.22	1
E00.E90	Hungary	32.90	2
E00.E90	Netherlands	32.24	3
E00.E90	Norway	35.46	1
E00.E90	Spain	276.40	2
E00.E90	Sweden	46.34	2
E00.E90	Switzerland	12.34	0
E00.E90	United Kingdom	160.49	1
E00.E90	Germany	11.70	2
E00.E90	France	6.00	0
E00.E90	Austria	7.63	0
E00.E90	Belgium	12.21	1
E00.E90	Czech Republic	5.89	2
E00.E90	Denmark	5.30	0
E00.E90	France	139.63	1
E00.E90	Germany	55.95	1
E00.E90	Hungary	6.03	0
E00.E90	Netherlands	13.76	1
E00.E90	Norway	5.74	0
E00.E90	Poland	9.32	2
E00.E90	Spain	36.23	0
E00.E90	Sweden	11.62	0
E00.E90	Switzerland	11.38	0
E00.E90	United Kingdom	75.23	0
E00.E90	Austria	6.84	3
E00.E90	Belgium	7.92	0
E00.E90	Czech Republic	6.27	1
E00.E90	Denmark	5.89	0
E00.E90	France	55.73	1
E00.E90	Germany	64.07	1
E00.E90	Hungary	5.95	0
E00.E90	Netherlands	15.50	0
E00.E90	Poland	16.63	1
E00.E90	Romania	6.35	0
E00.E90	Spain	29.53	0
E00.E90	Switzerland	8.44	1
E00.E90	United Kingdom	115.48	0
E00.E90	Austria	22.15	2
E00.E90	Belgium	50.80	0

For peer review only

E00.E90	Czech Republic	13.40	1
E00.E90	Denmark	17.88	0
E00.E90	Estonia	5.99	0
E00.E90	Finland	49.95	2
E00.E90	France	252.07	2
E00.E90	Germany	194.50	2
E00.E90	Hungary	7.45	0
E00.E90	Lithuania	9.24	0
E00.E90	Netherlands	65.25	1
E00.E90	Norway	27.11	1
E00.E90	Poland	35.14	1
E00.E90	Slovenia	7.33	1
E00.E90	Spain	197.81	2
E00.E90	Sweden	51.34	0
E00.E90	Switzerland	41.45	1
E00.E90	United Kingdom	300.46	2
E00.E90	Belgium	368.30	1
E00.E90	Czech Republic	64.54	2
E00.E90	Denmark	213.48	3
E00.E90	France	2056.06	1
E00.E90	Germany	1537.92	1
E00.E90	Hungary	32.52	2
E00.E90	Netherlands	438.51	3
E00.E90	Norway	124.38	3
E00.E90	Poland	59.66	1
E00.E90	Spain	332.92	3
E00.E90	Sweden	64.28	0
E00.E90	Switzerland	43.66	3
E00.E90	United Kingdom	223.36	2
E00.E90	Austria	5.39	0
E00.E90	Belgium	152.25	1
E00.E90	Czech Republic	31.66	2
E00.E90	Denmark	27.20	2
E00.E90	France	868.82	1
E00.E90	Germany	408.44	3
E00.E90	Hungary	12.73	1
E00.E90	Netherlands	45.71	0
E00.E90	Norway	14.31	1
E00.E90	Poland	35.30	3
E00.E90	Spain	352.57	3
E00.E90	Sweden	20.57	2
E00.E90	Switzerland	13.03	0
E00.E90	United Kingdom	170.39	3
E00.E90	Austria	52.13	1
E00.E90	Belgium	56.20	0
E00.E90	Czech Republic	13.14	1
E00.E90	Denmark	15.78	2
E00.E90	Finland	6.94	0
E00.E90	France	302.03	1
E00.E90	Germany	174.86	2

For peer review only

E00.E90	Hungary	10.25	2
E00.E90	Netherlands	24.62	2
E00.E90	Norway	9.88	0
E00.E90	Poland	10.09	0
E00.E90	Spain	109.99	1
E00.E90	Sweden	22.59	0
E00.E90	Switzerland	30.96	2
E00.E90	United Kingdom	123.69	0
F00.F99	Belgium	530.86	3
F00.F99	Croatia	68.99	1
F00.F99	Czech Republic	368.65	2
F00.F99	Denmark	368.28	2
F00.F99	Finland	1134.25	3
F00.F99	France	1749.57	3
F00.F99	Germany	2155.72	3
F00.F99	Hungary	1702.73	1
F00.F99	Latvia	102.04	1
F00.F99	Lithuania	36.27	2
F00.F99	Netherlands	1106.08	1
F00.F99	Norway	146.85	1
F00.F99	Poland	66.22	1
F00.F99	Spain	2725.66	2
F00.F99	Sweden	818.66	3
F00.F99	Switzerland	548.14	2
F00.F99	United Kingdom	4647.02	2
F00.F99	Austria	359.75	2
F00.F99	Belgium	2944.56	2
F00.F99	Croatia	360.08	2
F00.F99	Denmark	1914.34	1
F00.F99	Finland	1808.33	3
F00.F99	France	11844.02	3
F00.F99	Germany	12396.97	2
F00.F99	Hungary	348.93	3
F00.F99	Latvia	8.93	2
F00.F99	Lithuania	8.84	2
F00.F99	Netherlands	8622.18	3
F00.F99	Norway	1534.12	3
F00.F99	Poland	110.45	2
F00.F99	Spain	12598.62	2
F00.F99	Sweden	3665.26	2
F00.F99	Switzerland	2624.49	1
F00.F99	United Kingdom	23684.95	2
F00.F99	France	54.07	2
F00.F99	Germany	36.32	2
F00.F99	Croatia	24.71	3
F00.F99	Denmark	10.48	1
F00.F99	Germany	68.30	3
F00.F99	Hungary	31.79	0
F00.F99	Lithuania	6.19	0
F00.F99	Netherlands	57.34	0

For peer review only

F00.F99	Spain	15.39	3
F00.F99	Switzerland	24.09	2
F00.F99	United Kingdom	28.03	3
F00.F99	Croatia	8.45	0
F00.F99	Germany	300.40	3
F00.F99	Netherlands	22.84	2
F00.F99	Spain	22.04	3
F00.F99	United Kingdom	6.18	0
F00.F99	Belgium	12.84	1
F00.F99	Croatia	318.48	1
F00.F99	France	201.98	1
F00.F99	Germany	68.75	2
F00.F99	Latvia	5.22	0
F00.F99	Netherlands	50.40	3
F00.F99	Sweden	21.23	1
F00.F99	Switzerland	84.04	2
F00.F99	Austria	432.48	1
F00.F99	Belgium	357.70	2
F00.F99	Croatia	238.99	2
F00.F99	Czech Republic	151.14	3
F00.F99	Denmark	728.40	3
F00.F99	Estonia	114.20	3
F00.F99	Finland	200.74	2
F00.F99	France	3086.12	2
F00.F99	Germany	4754.56	2
F00.F99	Hungary	412.45	3
F00.F99	Latvia	125.22	3
F00.F99	Lithuania	30.13	0
F00.F99	Netherlands	282.39	2
F00.F99	Norway	198.32	1
F00.F99	Poland	1657.21	0
F00.F99	Romania	444.49	2
F00.F99	Slovenia	138.73	2
F00.F99	Spain	325.05	1
F00.F99	Sweden	301.10	3
F00.F99	Switzerland	234.47	1
F00.F99	United Kingdom	883.45	1
F00.F99	Austria	102.31	3
F00.F99	Belgium	8.63	2
F00.F99	Croatia	18.12	1
F00.F99	Denmark	9.90	0
F00.F99	France	24.47	2
F00.F99	Germany	178.87	1
F00.F99	Netherlands	9.63	1
F00.F99	Norway	32.29	3
F00.F99	Spain	5.92	1
F00.F99	Switzerland	32.30	3
F00.F99	United Kingdom	438.12	2
F00.F99	Germany	6.93	0
F00.F99	Germany	6.96	0

For peer review only

F00.F99	United Kingdom	27.18	3
F00.F99	United Kingdom	16.38	3
F00.F99	Belgium	33.92	2
F00.F99	France	447.47	2
F00.F99	Germany	157.99	3
F00.F99	Netherlands	13.39	0
F00.F99	Spain	12.76	1
F00.F99	Switzerland	22.07	0
F00.F99	United Kingdom	17.22	1
F00.F99	Austria	65.05	3
F00.F99	Belgium	35.41	2
F00.F99	Denmark	23.64	0
F00.F99	Finland	15.58	0
F00.F99	France	128.48	3
F00.F99	Germany	527.01	1
F00.F99	Lithuania	5.24	2
F00.F99	Norway	30.44	3
F00.F99	Spain	52.53	3
F00.F99	Sweden	17.56	0
F00.F99	Switzerland	65.50	3
F00.F99	United Kingdom	234.29	2
F00.F99	Belgium	17.19	0
F00.F99	Croatia	43.83	1
F00.F99	Denmark	50.50	0
F00.F99	Finland	55.04	0
F00.F99	France	126.32	1
F00.F99	Germany	78.48	2
F00.F99	Hungary	67.16	1
F00.F99	Latvia	6.76	0
F00.F99	Lithuania	5.97	0
F00.F99	Netherlands	31.74	0
F00.F99	Norway	17.21	0
F00.F99	Poland	27.37	3
F00.F99	Romania	85.98	2
F00.F99	Spain	127.64	1
F00.F99	Sweden	27.56	0
F00.F99	Switzerland	69.47	2
F00.F99	United Kingdom	75.42	1
F00.F99	Belgium	6.21	0
F00.F99	Denmark	13.37	1
F00.F99	France	69.11	1
F00.F99	Germany	8.13	0
F00.F99	Spain	6.41	0
F00.F99	Sweden	6.28	0
F00.F99	Switzerland	5.40	0
F00.F99	United Kingdom	5.92	1
F00.F99	France	7.58	3
F00.F99	Germany	6.97	2
F00.F99	Switzerland	5.94	0
F00.F99	France	50.02	1

For peer review only

F00.F99	Belgium	10.02	0
F00.F99	Croatia	11.51	1
F00.F99	France	221.53	3
F00.F99	Germany	17.94	1
F00.F99	Netherlands	12.16	0
F00.F99	Spain	10.59	0
F00.F99	Sweden	6.31	3
F00.F99	Switzerland	11.84	0
F00.F99	Belgium	5.61	2
F00.F99	Denmark	18.74	0
F00.F99	France	135.85	1
F00.F99	Germany	6.42	2
F00.F99	Netherlands	17.22	0
F00.F99	Norway	6.17	0
F00.F99	Spain	22.13	0
F00.F99	Sweden	13.21	1
F00.F99	Switzerland	9.25	0
F00.F99	United Kingdom	17.77	1
F00.F99	Belgium	84.40	2
F00.F99	Croatia	6.68	1
F00.F99	Denmark	62.62	1
F00.F99	Finland	15.63	1
F00.F99	France	1001.84	1
F00.F99	Germany	111.10	2
F00.F99	Netherlands	42.05	2
F00.F99	Norway	31.47	2
F00.F99	Spain	63.85	3
F00.F99	Sweden	61.83	0
F00.F99	Switzerland	99.47	2
F00.F99	United Kingdom	159.11	1
F00.F99	Belgium	6.07	0
F00.F99	Denmark	6.00	2
F00.F99	France	72.52	1
F00.F99	Germany	8.84	2
F00.F99	Spain	6.63	1
F00.F99	Switzerland	9.19	1
F00.F99	United Kingdom	5.96	1
F00.F99	France	9.61	1
F00.F99	France	149.54	1
F00.F99	Spain	6.85	0
F00.F99	Sweden	5.16	1
F00.F99	Switzerland	6.63	1
F00.F99	France	5.55	2
F00.F99	France	54.03	0
F00.F99	Belgium	10.82	3
F00.F99	France	139.91	2
F00.F99	Spain	34.04	2
F00.F99	France	10.06	0
F00.F99	Austria	6.44	0
F00.F99	Belgium	49.19	2

For peer review only

F00.F99	France	36.19	1
F00.F99	Germany	66.20	2
F00.F99	Netherlands	12.08	0
F00.F99	Poland	6.58	0
F00.F99	Spain	7.47	0
F00.F99	Sweden	11.75	0
F00.F99	Switzerland	11.28	1
F00.F99	United Kingdom	24.82	0
F00.F99	Belgium	5.36	3
F00.F99	France	53.75	2
F00.F99	Belgium	5.50	3
F00.F99	Germany	9.87	0
F00.F99	Netherlands	5.07	0
F00.F99	Finland	6.63	1
F00.F99	Finland	10.95	1
F00.F99	Spain	6.55	2
F00.F99	Finland	13.24	1
F00.F99	France	27.85	1
F00.F99	Spain	15.07	0
F00.F99	Sweden	6.96	0
F00.F99	Belgium	16.39	0
F00.F99	Denmark	10.59	1
F00.F99	France	111.15	0
F00.F99	Germany	7.56	3
F00.F99	Netherlands	41.62	1
F00.F99	Norway	9.18	2
F00.F99	Spain	40.14	0
F00.F99	Sweden	15.61	0
F00.F99	Switzerland	11.54	0
F00.F99	United Kingdom	22.30	1
F00.F99	France	28.28	1
F00.F99	Germany	6.48	1
F00.F99	United Kingdom	7.85	2
F00.F99	Belgium	11.95	2
F00.F99	Denmark	5.53	0
F00.F99	France	71.15	0
F00.F99	Netherlands	14.30	1
F00.F99	Spain	6.45	1
G00.G99	Austria	12.41	1
G00.G99	Belgium	18.30	1
G00.G99	Croatia	16.68	0
G00.G99	Czech Republic	34.39	1
G00.G99	Denmark	24.17	0
G00.G99	Estonia	9.81	1
G00.G99	Finland	13.30	1
G00.G99	France	114.34	1
G00.G99	Germany	96.28	1
G00.G99	Hungary	49.61	3
G00.G99	Latvia	7.58	0
G00.G99	Lithuania	16.26	3

For peer review only

G00.G99	Netherlands	44.96	1
G00.G99	Norway	12.71	1
G00.G99	Poland	108.46	0
G00.G99	Romania	73.85	1
G00.G99	Slovenia	6.42	1
G00.G99	Spain	99.42	3
G00.G99	Sweden	14.82	1
G00.G99	Switzerland	12.45	1
G00.G99	United Kingdom	113.35	1
G00.G99	Austria	11.73	0
G00.G99	Belgium	13.19	0
G00.G99	Czech Republic	7.98	1
G00.G99	Denmark	6.48	1
G00.G99	France	50.30	0
G00.G99	Germany	73.23	1
G00.G99	Hungary	22.27	2
G00.G99	Latvia	5.09	0
G00.G99	Netherlands	40.46	0
G00.G99	Norway	6.96	1
G00.G99	Poland	40.81	0
G00.G99	Romania	24.52	1
G00.G99	Spain	57.22	3
G00.G99	Sweden	7.98	0
G00.G99	United Kingdom	85.11	1
G00.G99	Austria	21.49	2
G00.G99	Belgium	26.54	2
G00.G99	Croatia	11.96	1
G00.G99	Czech Republic	13.35	1
G00.G99	Denmark	5.84	0
G00.G99	Estonia	5.49	1
G00.G99	France	128.44	0
G00.G99	Germany	178.79	1
G00.G99	Hungary	11.81	2
G00.G99	Latvia	17.86	3
G00.G99	Lithuania	22.67	2
G00.G99	Netherlands	22.30	1
G00.G99	Norway	6.35	0
G00.G99	Poland	102.78	1
G00.G99	Romania	113.76	2
G00.G99	Spain	98.48	0
G00.G99	Sweden	7.80	0
G00.G99	Switzerland	14.08	0
G00.G99	United Kingdom	107.27	2
G00.G99	Belgium	9.18	0
G00.G99	Czech Republic	10.01	0
G00.G99	Denmark	6.48	0
G00.G99	France	44.40	0
G00.G99	Germany	47.05	2
G00.G99	Hungary	8.95	0
G00.G99	Netherlands	9.30	0

For peer review only

G00.G99	Poland	27.99	0
G00.G99	Romania	14.81	0
G00.G99	Spain	34.37	2
G00.G99	Sweden	5.69	0
G00.G99	Switzerland	5.82	0
G00.G99	United Kingdom	56.79	3
G00.G99	France	23.38	1
G00.G99	Germany	21.67	1
G00.G99	United Kingdom	15.35	2
G00.G99	Finland	8.03	0
G00.G99	France	30.77	0
G00.G99	Germany	17.08	2
G00.G99	Netherlands	7.08	0
G00.G99	Poland	7.80	0
G00.G99	Spain	15.59	3
G00.G99	Switzerland	5.08	0
G00.G99	United Kingdom	14.22	1
G00.G99	Austria	22.99	1
G00.G99	Belgium	36.35	1
G00.G99	Croatia	6.70	1
G00.G99	Czech Republic	17.50	2
G00.G99	Denmark	18.41	0
G00.G99	Finland	6.73	0
G00.G99	France	163.32	1
G00.G99	Germany	247.56	2
G00.G99	Hungary	11.40	0
G00.G99	Lithuania	5.49	0
G00.G99	Netherlands	55.95	0
G00.G99	Norway	18.75	0
G00.G99	Poland	58.26	1
G00.G99	Romania	6.51	0
G00.G99	Spain	86.86	1
G00.G99	Sweden	27.58	0
G00.G99	Switzerland	16.00	1
G00.G99	United Kingdom	248.01	1
G00.G99	Austria	5.69	0
G00.G99	Belgium	11.39	0
G00.G99	Denmark	7.92	0
G00.G99	Finland	10.68	1
G00.G99	France	59.40	0
G00.G99	Germany	57.42	2
G00.G99	Netherlands	28.69	3
G00.G99	Poland	6.48	0
G00.G99	Romania	6.05	1
G00.G99	Spain	50.53	2
G00.G99	Sweden	9.36	0
G00.G99	Switzerland	10.01	0
G00.G99	United Kingdom	75.35	1
G00.G99	Austria	174.55	1
G00.G99	Belgium	287.30	1

For peer review only

G00.G99	Croatia	42.78	2
G00.G99	Czech Republic	84.61	2
G00.G99	Denmark	148.15	0
G00.G99	Estonia	19.20	1
G00.G99	Finland	201.52	0
G00.G99	France	1628.65	1
G00.G99	Germany	1724.59	1
G00.G99	Hungary	87.74	2
G00.G99	Latvia	20.79	0
G00.G99	Lithuania	39.37	1
G00.G99	Netherlands	508.62	0
G00.G99	Norway	142.35	0
G00.G99	Poland	394.10	2
G00.G99	Romania	69.49	1
G00.G99	Slovenia	37.24	0
G00.G99	Spain	1013.99	3
G00.G99	Sweden	308.07	2
G00.G99	Switzerland	191.83	1
G00.G99	United Kingdom	2285.12	3
G00.G99	Austria	915.81	3
G00.G99	Belgium	847.03	1
G00.G99	Croatia	270.80	2
G00.G99	Czech Republic	281.71	0
G00.G99	Denmark	391.63	2
G00.G99	Estonia	51.67	1
G00.G99	Finland	553.13	2
G00.G99	France	4895.80	0
G00.G99	Germany	6805.50	2
G00.G99	Hungary	307.64	1
G00.G99	Latvia	47.41	3
G00.G99	Lithuania	99.23	2
G00.G99	Netherlands	1534.62	1
G00.G99	Norway	350.83	2
G00.G99	Poland	1038.84	2
G00.G99	Romania	511.51	1
G00.G99	Slovenia	84.56	0
G00.G99	Spain	3070.99	1
G00.G99	Sweden	508.36	3
G00.G99	Switzerland	672.24	0
G00.G99	United Kingdom	5577.69	0
G00.G99	Belgium	6.30	0
G00.G99	Czech Republic	32.18	2
G00.G99	Denmark	7.35	2
G00.G99	France	26.66	0
G00.G99	Germany	48.59	1
G00.G99	Netherlands	13.54	2
G00.G99	Poland	27.92	2
G00.G99	Romania	13.45	3
G00.G99	Spain	53.24	2
G00.G99	Switzerland	31.10	3

For peer review only

G00.G99	United Kingdom	24.89	3
G00.G99	Austria	10.04	1
G00.G99	Belgium	35.09	0
G00.G99	Czech Republic	5.02	1
G00.G99	Denmark	6.62	0
G00.G99	Finland	40.01	2
G00.G99	France	356.21	1
G00.G99	Germany	101.20	2
G00.G99	Netherlands	34.59	3
G00.G99	Norway	10.34	2
G00.G99	Poland	12.25	0
G00.G99	Spain	94.72	2
G00.G99	Sweden	29.64	3
G00.G99	Switzerland	31.24	0
G00.G99	United Kingdom	75.15	3
G00.G99	France	11.22	0
G00.G99	Germany	5.34	1
G00.G99	United Kingdom	12.17	0
G00.G99	Belgium	9.45	0
G00.G99	Finland	8.67	0
G00.G99	France	97.63	1
G00.G99	Germany	15.08	2
G00.G99	Poland	6.78	0
G00.G99	Spain	19.53	1
G00.G99	Switzerland	15.82	2
G00.G99	United Kingdom	13.82	0
G00.G99	Austria	932.64	3
G00.G99	Belgium	2088.76	2
G00.G99	Croatia	245.22	1
G00.G99	Czech Republic	1187.20	2
G00.G99	Denmark	866.86	1
G00.G99	Estonia	54.35	1
G00.G99	Finland	3776.32	3
G00.G99	France	15870.84	2
G00.G99	Germany	5656.99	2
G00.G99	Hungary	322.65	1
G00.G99	Latvia	41.46	2
G00.G99	Lithuania	130.17	1
G00.G99	Netherlands	2254.73	2
G00.G99	Norway	737.85	2
G00.G99	Poland	2573.09	2
G00.G99	Romania	1335.91	2
G00.G99	Slovenia	98.17	2
G00.G99	Spain	11437.72	3
G00.G99	Sweden	1692.67	1
G00.G99	Switzerland	1585.05	0
G00.G99	United Kingdom	9044.26	3
G00.G99	Austria	34.79	2
G00.G99	Belgium	184.78	2
G00.G99	Croatia	20.90	1

For peer review only

G00.G99	Czech Republic	62.19	2
G00.G99	Denmark	37.14	1
G00.G99	Estonia	35.52	1
G00.G99	Finland	237.62	1
G00.G99	France	1516.18	2
G00.G99	Germany	501.35	2
G00.G99	Hungary	580.59	1
G00.G99	Latvia	42.45	3
G00.G99	Lithuania	33.07	2
G00.G99	Netherlands	160.67	3
G00.G99	Norway	77.99	1
G00.G99	Poland	238.66	0
G00.G99	Romania	37.60	1
G00.G99	Slovenia	5.89	1
G00.G99	Spain	741.15	1
G00.G99	Sweden	135.28	1
G00.G99	Switzerland	136.65	2
G00.G99	United Kingdom	882.58	2
G00.G99	Austria	108.60	1
G00.G99	Belgium	136.88	0
G00.G99	Croatia	45.43	1
G00.G99	Czech Republic	107.71	0
G00.G99	Denmark	130.67	0
G00.G99	Estonia	13.76	0
G00.G99	Finland	68.76	0
G00.G99	France	625.70	0
G00.G99	Germany	1063.72	2
G00.G99	Hungary	94.95	0
G00.G99	Latvia	28.56	3
G00.G99	Lithuania	37.13	0
G00.G99	Netherlands	221.42	0
G00.G99	Norway	103.65	0
G00.G99	Poland	468.94	1
G00.G99	Romania	112.47	1
G00.G99	Slovenia	27.14	1
G00.G99	Spain	224.51	0
G00.G99	Sweden	133.55	2
G00.G99	Switzerland	135.58	0
G00.G99	United Kingdom	1317.40	2
G00.G99	Poland	5.50	0
G00.G99	Belgium	9.24	2
G00.G99	Czech Republic	14.35	0
G00.G99	Denmark	5.91	0
G00.G99	France	23.89	0
G00.G99	Germany	21.30	0
G00.G99	Netherlands	8.96	3
G00.G99	Poland	25.54	1
G00.G99	Romania	5.33	0
G00.G99	Spain	28.22	0
G00.G99	United Kingdom	21.68	0

For peer review only

G00.G99	Austria	91.62	2
G00.G99	Belgium	204.83	0
G00.G99	Croatia	66.89	1
G00.G99	Czech Republic	136.74	3
G00.G99	Denmark	96.72	0
G00.G99	Estonia	45.37	3
G00.G99	Finland	79.84	0
G00.G99	France	1233.69	3
G00.G99	Germany	1782.76	1
G00.G99	Hungary	135.32	1
G00.G99	Latvia	54.08	1
G00.G99	Lithuania	52.51	2
G00.G99	Netherlands	218.60	3
G00.G99	Norway	82.61	0
G00.G99	Poland	530.09	1
G00.G99	Romania	293.99	0
G00.G99	Slovenia	21.66	0
G00.G99	Spain	357.71	2
G00.G99	Sweden	99.95	1
G00.G99	Switzerland	92.26	0
G00.G99	United Kingdom	1094.10	2
G00.G99	Austria	18.99	2
G00.G99	Belgium	76.32	0
G00.G99	Croatia	25.76	2
G00.G99	Czech Republic	19.42	2
G00.G99	Denmark	12.51	0
G00.G99	Finland	18.61	0
G00.G99	France	396.90	1
G00.G99	Germany	355.82	1
G00.G99	Hungary	5.40	2
G00.G99	Lithuania	37.12	1
G00.G99	Netherlands	55.70	1
G00.G99	Norway	7.87	0
G00.G99	Poland	172.40	2
G00.G99	Romania	37.11	1
G00.G99	Slovenia	5.78	0
G00.G99	Spain	101.57	3
G00.G99	Sweden	17.53	0
G00.G99	Switzerland	17.60	2
G00.G99	United Kingdom	91.51	1
G00.G99	Austria	50.23	2
G00.G99	Belgium	25.25	1
G00.G99	Denmark	15.61	1
G00.G99	France	148.05	2
G00.G99	Germany	96.43	2
G00.G99	Netherlands	118.53	3
G00.G99	Norway	34.37	3
G00.G99	Romania	70.84	2
G00.G99	Spain	73.24	2
G00.G99	Sweden	8.59	2

For peer review only

G00.G99	United Kingdom	137.74	1
G00.G99	Belgium	10.39	0
G00.G99	France	125.09	2
G00.G99	Germany	59.84	1
G00.G99	Netherlands	13.35	1
G00.G99	Spain	79.42	1
G00.G99	Switzerland	11.60	1
G00.G99	United Kingdom	40.45	2
G00.G99	France	7.91	0
G00.G99	United Kingdom	6.54	0
G00.G99	Belgium	6.40	0
G00.G99	France	25.74	0
G00.G99	Germany	11.51	0
G00.G99	Netherlands	5.87	0
G00.G99	Poland	5.98	2
G00.G99	Spain	11.20	3
G00.G99	United Kingdom	19.02	0
G00.G99	Austria	8.22	0
G00.G99	Belgium	13.13	0
G00.G99	Croatia	6.02	0
G00.G99	Czech Republic	8.10	3
G00.G99	France	34.82	0
G00.G99	Germany	76.12	1
G00.G99	Hungary	13.31	0
G00.G99	Netherlands	15.12	1
G00.G99	Poland	27.81	3
G00.G99	Spain	50.26	0
G00.G99	Switzerland	8.20	1
G00.G99	United Kingdom	53.75	1
G00.G99	Austria	11.15	0
G00.G99	Belgium	24.08	0
G00.G99	Czech Republic	5.13	1
G00.G99	Denmark	13.73	0
G00.G99	Finland	8.82	0
G00.G99	France	97.55	0
G00.G99	Germany	118.44	3
G00.G99	Hungary	33.11	0
G00.G99	Lithuania	7.27	0
G00.G99	Netherlands	29.50	1
G00.G99	Norway	8.26	2
G00.G99	Poland	27.66	0
G00.G99	Spain	55.49	0
G00.G99	Sweden	11.33	0
G00.G99	Switzerland	14.86	1
G00.G99	United Kingdom	67.21	1
G00.G99	Austria	19.54	0
G00.G99	Belgium	16.80	1
G00.G99	Croatia	11.86	1
G00.G99	Czech Republic	13.87	1
G00.G99	Denmark	10.35	0

For peer review only

G00.G99	Finland	6.93	0
G00.G99	France	81.94	0
G00.G99	Germany	120.59	1
G00.G99	Hungary	22.64	0
G00.G99	Netherlands	27.03	0
G00.G99	Norway	5.68	0
G00.G99	Poland	50.87	1
G00.G99	Romania	17.94	0
G00.G99	Slovenia	5.07	0
G00.G99	Spain	102.64	1
G00.G99	Sweden	15.78	1
G00.G99	Switzerland	14.83	0
G00.G99	United Kingdom	119.68	1
G00.G99	Austria	30.12	0
G00.G99	Belgium	41.40	0
G00.G99	Croatia	13.20	0
G00.G99	Czech Republic	14.84	1
G00.G99	Denmark	20.81	1
G00.G99	Finland	22.82	0
G00.G99	France	204.16	1
G00.G99	Germany	252.09	2
G00.G99	Hungary	25.98	0
G00.G99	Netherlands	97.48	1
G00.G99	Norway	28.09	0
G00.G99	Poland	56.77	0
G00.G99	Romania	29.94	1
G00.G99	Slovenia	8.13	0
G00.G99	Spain	225.71	1
G00.G99	Sweden	44.43	1
G00.G99	Switzerland	34.46	0
G00.G99	United Kingdom	281.65	0
G00.G99	Belgium	13.45	0
G00.G99	France	93.42	1
G00.G99	Germany	17.84	1
G00.G99	Netherlands	5.63	0
G00.G99	Poland	6.12	0
G00.G99	Spain	24.26	0
G00.G99	United Kingdom	28.20	1
G00.G99	Austria	18.54	1
G00.G99	Belgium	23.28	0
G00.G99	Croatia	13.79	1
G00.G99	Czech Republic	30.41	1
G00.G99	Denmark	14.78	0
G00.G99	Finland	17.18	2
G00.G99	France	208.86	1
G00.G99	Germany	122.67	1
G00.G99	Hungary	28.80	0
G00.G99	Latvia	7.06	0
G00.G99	Lithuania	20.53	1
G00.G99	Netherlands	5.97	2

For peer review only

G00.G99	Norway	21.95	1
G00.G99	Poland	97.09	1
G00.G99	Romania	5.94	0
G00.G99	Slovenia	9.20	1
G00.G99	Spain	180.60	0
G00.G99	Sweden	23.91	1
G00.G99	Switzerland	26.66	1
G00.G99	United Kingdom	269.89	3
G00.G99	Belgium	33.07	2
G00.G99	France	127.68	3
G00.G99	Germany	53.32	1
G00.G99	Hungary	16.10	0
G00.G99	Poland	5.72	1
G00.G99	Spain	36.81	1
G00.G99	Switzerland	36.54	2
G00.G99	United Kingdom	89.13	2
G00.G99	Austria	11.23	0
G00.G99	Belgium	19.85	2
G00.G99	Croatia	18.44	0
G00.G99	Czech Republic	11.64	3
G00.G99	Denmark	20.16	1
G00.G99	France	166.37	1
G00.G99	Germany	191.26	3
G00.G99	Hungary	18.59	2
G00.G99	Netherlands	18.79	2
G00.G99	Norway	6.28	0
G00.G99	Spain	72.55	0
G00.G99	Sweden	8.20	0
G00.G99	Switzerland	27.86	2
G00.G99	United Kingdom	135.53	2
G00.G99	Denmark	8.18	3
G00.G99	France	27.68	1
G00.G99	Germany	20.06	0
G00.G99	Hungary	5.33	0
G00.G99	Netherlands	8.84	0
G00.G99	Poland	6.66	0
G00.G99	Spain	5.79	0
G00.G99	United Kingdom	8.46	0
G00.G99	Belgium	15.32	2
G00.G99	Finland	17.15	1
G00.G99	France	149.82	3
G00.G99	Germany	121.71	2
G00.G99	Netherlands	78.48	1
G00.G99	Norway	9.44	2
G00.G99	Poland	21.22	1
G00.G99	Spain	22.88	3
G00.G99	Sweden	22.39	0
G00.G99	Switzerland	20.31	3
G00.G99	United Kingdom	141.74	0
G00.G99	Austria	9.70	1

For peer review only

G00.G99	Belgium	31.35	1
G00.G99	Croatia	6.21	2
G00.G99	Czech Republic	26.66	1
G00.G99	Denmark	12.62	0
G00.G99	Finland	21.03	2
G00.G99	France	196.66	1
G00.G99	Germany	130.52	1
G00.G99	Hungary	25.07	0
G00.G99	Lithuania	7.32	0
G00.G99	Netherlands	22.46	2
G00.G99	Norway	10.25	0
G00.G99	Poland	35.36	2
G00.G99	Romania	42.87	1
G00.G99	Spain	139.42	2
G00.G99	Sweden	14.58	0
G00.G99	Switzerland	24.81	2
G00.G99	United Kingdom	87.79	1
G00.G99	Czech Republic	7.04	1
G00.G99	France	9.22	0
G00.G99	Hungary	6.89	2
G00.G99	Latvia	10.94	3
G00.G99	Lithuania	6.45	0
G00.G99	Poland	10.46	0
G00.G99	Romania	10.09	3
G00.G99	Spain	6.65	3
G00.G99	Austria	131.12	1
G00.G99	Belgium	185.82	0
G00.G99	Croatia	10.17	0
G00.G99	Czech Republic	303.60	3
G00.G99	Denmark	31.63	1
G00.G99	Estonia	10.05	1
G00.G99	Finland	11.10	0
G00.G99	France	1032.53	1
G00.G99	Germany	1290.36	2
G00.G99	Hungary	117.62	2
G00.G99	Latvia	41.21	3
G00.G99	Lithuania	11.48	0
G00.G99	Netherlands	72.38	1
G00.G99	Norway	30.35	1
G00.G99	Poland	90.46	3
G00.G99	Romania	172.66	2
G00.G99	Spain	780.64	3
G00.G99	Sweden	67.93	1
G00.G99	Switzerland	67.82	0
G00.G99	United Kingdom	308.38	0
G00.G99	Austria	6.11	0
G00.G99	Belgium	18.17	3
G00.G99	Czech Republic	11.08	0
G00.G99	France	107.09	3
G00.G99	Germany	38.67	1

For peer review only

G00.G99	Hungary	11.28	0
G00.G99	Lithuania	5.79	0
G00.G99	Netherlands	60.17	0
G00.G99	Poland	26.65	1
G00.G99	Romania	12.16	0
G00.G99	Spain	85.76	0
G00.G99	Switzerland	10.68	0
G00.G99	United Kingdom	151.06	2
G00.G99	Czech Republic	7.59	0
G00.G99	France	20.09	1
G00.G99	Germany	7.28	0
G00.G99	Poland	22.87	1
G00.G99	Spain	7.63	3
G00.G99	United Kingdom	6.31	0
G00.G99	Belgium	20.61	2
G00.G99	France	178.09	2
G00.G99	Germany	6.56	0
G00.G99	Netherlands	31.35	1
G00.G99	Norway	6.00	1
G00.G99	Poland	8.67	1
G00.G99	Spain	22.04	3
G00.G99	Sweden	9.52	0
G00.G99	Switzerland	9.88	0
G00.G99	United Kingdom	12.33	0
H00.H59	United Kingdom	5.36	0
H60.H95	United Kingdom	5.23	1
H60.H95	France	14.11	1
H60.H95	Germany	8.38	0
H60.H95	Hungary	7.44	0
H60.H95	Netherlands	9.41	3
H60.H95	United Kingdom	15.78	0
H60.H95	France	5.16	0
I00.I99	Germany	6.53	1
I00.I99	Germany	14.87	1
I00.I99	Romania	5.72	0
I00.I99	Austria	14.00	2
I00.I99	Belgium	164.64	1
I00.I99	Croatia	9.32	3
I00.I99	Czech Republic	87.73	0
I00.I99	Denmark	7.63	1
I00.I99	Estonia	12.48	2
I00.I99	Finland	15.52	1
I00.I99	France	1226.19	1
I00.I99	Germany	541.49	2
I00.I99	Hungary	61.43	2
I00.I99	Latvia	22.74	2
I00.I99	Lithuania	41.68	1
I00.I99	Netherlands	14.20	2
I00.I99	Norway	14.71	3
I00.I99	Poland	237.85	2

For peer review only

I00.I99	Romania	277.55	2
I00.I99	Slovenia	9.38	1
I00.I99	Spain	766.49	1
I00.I99	Sweden	37.72	1
I00.I99	Switzerland	24.18	0
I00.I99	United Kingdom	912.58	2
I00.I99	Croatia	7.30	3
I00.I99	Czech Republic	69.88	0
I00.I99	Denmark	36.80	3
I00.I99	Estonia	9.51	1
I00.I99	France	11.19	0
I00.I99	Germany	89.18	2
I00.I99	Hungary	40.23	2
I00.I99	Latvia	10.41	1
I00.I99	Lithuania	24.45	2
I00.I99	Poland	170.02	2
I00.I99	Romania	129.42	2
I00.I99	Slovenia	7.11	0
I00.I99	Spain	19.59	0
I00.I99	United Kingdom	9.19	0
I00.I99	Belgium	17.92	1
I00.I99	Czech Republic	10.98	2
I00.I99	France	50.54	1
I00.I99	Germany	260.40	2
I00.I99	Poland	24.25	2
I00.I99	Romania	6.16	0
I00.I99	Spain	69.07	1
I00.I99	Sweden	17.83	1
I00.I99	Switzerland	5.38	1
I00.I99	United Kingdom	91.14	1
I00.I99	Austria	219.37	2
I00.I99	Belgium	130.05	1
I00.I99	Croatia	75.30	2
I00.I99	Czech Republic	123.35	2
I00.I99	Denmark	12.45	0
I00.I99	Estonia	15.22	0
I00.I99	Finland	46.68	1
I00.I99	France	439.42	0
I00.I99	Germany	1729.42	3
I00.I99	Hungary	250.47	1
I00.I99	Latvia	27.83	1
I00.I99	Lithuania	67.29	0
I00.I99	Netherlands	36.05	2
I00.I99	Norway	36.12	3
I00.I99	Poland	825.76	1
I00.I99	Romania	100.71	3
I00.I99	Slovenia	89.86	0
I00.I99	Spain	865.40	1
I00.I99	Sweden	71.25	1
I00.I99	Switzerland	85.61	1

For peer review only

I00.I99	United Kingdom	243.66	0
I00.I99	Czech Republic	7.10	3
I00.I99	Estonia	5.42	1
I00.I99	France	19.74	1
I00.I99	Germany	17.20	1
I00.I99	Hungary	13.64	0
I00.I99	Latvia	15.94	0
I00.I99	Lithuania	15.45	3
I00.I99	Netherlands	5.27	1
I00.I99	Poland	6.65	1
I00.I99	Romania	173.72	3
I00.I99	Spain	78.76	0
I00.I99	United Kingdom	127.64	2
I00.I99	Austria	860.76	3
I00.I99	Belgium	363.78	2
I00.I99	Croatia	160.91	1
I00.I99	Czech Republic	1018.84	2
I00.I99	Denmark	505.92	3
I00.I99	Finland	51.63	0
I00.I99	France	4263.26	1
I00.I99	Germany	7612.14	3
I00.I99	Hungary	2114.91	0
I00.I99	Latvia	88.25	3
I00.I99	Lithuania	62.99	2
I00.I99	Netherlands	662.89	2
I00.I99	Norway	281.28	2
I00.I99	Poland	2436.29	3
I00.I99	Romania	19284.79	1
I00.I99	Slovenia	43.47	1
I00.I99	Spain	3489.33	2
I00.I99	Sweden	450.76	1
I00.I99	Switzerland	777.02	0
I00.I99	United Kingdom	1116.31	1
I00.I99	Austria	2145.76	2
I00.I99	Belgium	468.94	3
I00.I99	Croatia	1522.29	1
I00.I99	Czech Republic	1397.25	3
I00.I99	Denmark	216.18	0
I00.I99	Estonia	1449.37	3
I00.I99	Finland	774.81	2
I00.I99	France	3818.20	3
I00.I99	Germany	20709.02	3
I00.I99	Hungary	5079.32	3
I00.I99	Latvia	644.70	2
I00.I99	Lithuania	461.66	2
I00.I99	Netherlands	553.82	2
I00.I99	Norway	119.97	2
I00.I99	Poland	4867.68	2
I00.I99	Romania	17337.23	2
I00.I99	Slovenia	704.71	0

For peer review only

I00.I99	Spain	4065.83	2
I00.I99	Sweden	751.10	1
I00.I99	Switzerland	1410.00	2
I00.I99	United Kingdom	3348.88	3
I00.I99	Austria	297.18	2
I00.I99	Belgium	154.82	1
I00.I99	Croatia	7.93	0
I00.I99	Czech Republic	113.28	1
I00.I99	Denmark	71.25	0
I00.I99	Estonia	12.95	0
I00.I99	Finland	60.81	1
I00.I99	France	770.75	2
I00.I99	Germany	1523.02	1
I00.I99	Hungary	358.91	2
I00.I99	Latvia	39.65	1
I00.I99	Lithuania	24.05	0
I00.I99	Netherlands	123.94	1
I00.I99	Norway	73.14	1
I00.I99	Poland	105.06	0
I00.I99	Romania	110.31	2
I00.I99	Spain	1023.80	3
I00.I99	Sweden	152.45	0
I00.I99	Switzerland	198.50	3
I00.I99	United Kingdom	778.03	0
I00.I99	Austria	299.81	3
I00.I99	Belgium	115.26	1
I00.I99	Czech Republic	129.56	1
I00.I99	Denmark	145.44	1
I00.I99	Estonia	187.78	2
I00.I99	Finland	103.36	2
I00.I99	France	543.52	3
I00.I99	Germany	3198.00	3
I00.I99	Hungary	455.65	1
I00.I99	Latvia	25.24	2
I00.I99	Lithuania	43.94	2
I00.I99	Netherlands	56.14	2
I00.I99	Norway	22.66	2
I00.I99	Poland	147.49	0
I00.I99	Romania	18.42	3
I00.I99	Slovenia	29.19	3
I00.I99	Spain	561.08	2
I00.I99	Sweden	122.46	2
I00.I99	Switzerland	362.03	1
I00.I99	United Kingdom	339.16	3
I00.I99	Austria	130.97	3
I00.I99	Belgium	201.70	2
I00.I99	Croatia	37.00	3
I00.I99	Czech Republic	109.46	0
I00.I99	Denmark	57.37	1
I00.I99	Finland	27.55	1

For peer review only

I00.I99	France	1181.53	3
I00.I99	Germany	294.42	3
I00.I99	Hungary	86.59	3
I00.I99	Latvia	47.45	0
I00.I99	Lithuania	59.55	3
I00.I99	Netherlands	299.58	2
I00.I99	Norway	227.20	2
I00.I99	Poland	1117.33	3
I00.I99	Romania	517.57	2
I00.I99	Slovenia	13.96	1
I00.I99	Spain	378.77	3
I00.I99	Sweden	378.13	2
I00.I99	Switzerland	50.90	1
I00.I99	United Kingdom	135.21	1
I00.I99	Austria	5428.97	2
I00.I99	Belgium	6953.08	1
I00.I99	Croatia	4553.48	3
I00.I99	Czech Republic	9577.10	3
I00.I99	Denmark	3288.88	2
I00.I99	Estonia	595.22	3
I00.I99	Finland	5767.39	3
I00.I99	France	22533.97	3
I00.I99	Germany	59621.37	3
I00.I99	Hungary	9499.88	2
I00.I99	Latvia	1473.12	2
I00.I99	Lithuania	1440.42	3
I00.I99	Netherlands	10579.16	2
I00.I99	Norway	4233.75	1
I00.I99	Poland	27758.72	1
I00.I99	Romania	28379.62	3
I00.I99	Slovenia	1056.55	2
I00.I99	Spain	23596.48	3
I00.I99	Sweden	8513.33	1
I00.I99	Switzerland	3036.82	3
I00.I99	United Kingdom	44576.19	3
I00.I99	Austria	768.65	2
I00.I99	Belgium	70.10	1
I00.I99	Croatia	280.21	3
I00.I99	Czech Republic	145.07	3
I00.I99	Estonia	183.86	3
I00.I99	France	54.97	1
I00.I99	Germany	4152.43	1
I00.I99	Hungary	562.33	2
I00.I99	Latvia	91.26	3
I00.I99	Lithuania	137.71	2
I00.I99	Netherlands	43.28	3
I00.I99	Poland	249.70	2
I00.I99	Romania	83.47	0
I00.I99	Slovenia	93.73	2
I00.I99	Spain	26.91	2

For peer review only

I00.I99	Switzerland	63.70	1
I00.I99	Austria	82.56	2
I00.I99	Belgium	314.22	3
I00.I99	Czech Republic	287.26	1
I00.I99	Denmark	201.75	3
I00.I99	Estonia	73.53	1
I00.I99	Finland	9.45	1
I00.I99	France	2625.54	2
I00.I99	Germany	1097.26	1
I00.I99	Hungary	376.86	2
I00.I99	Latvia	165.06	3
I00.I99	Lithuania	371.42	1
I00.I99	Netherlands	241.18	2
I00.I99	Norway	26.03	2
I00.I99	Poland	1436.09	0
I00.I99	Romania	773.48	3
I00.I99	Slovenia	5.42	1
I00.I99	Spain	1385.64	2
I00.I99	Sweden	99.61	2
I00.I99	Switzerland	94.44	2
I00.I99	United Kingdom	558.00	1
I00.I99	Austria	10704.52	2
I00.I99	Belgium	4344.90	1
I00.I99	Croatia	8948.68	0
I00.I99	Czech Republic	24981.67	2
I00.I99	Denmark	3626.24	2
I00.I99	Estonia	5005.09	1
I00.I99	Finland	7822.66	2
I00.I99	France	16858.87	1
I00.I99	Germany	85468.65	3
I00.I99	Hungary	31532.50	3
I00.I99	Latvia	9150.87	1
I00.I99	Lithuania	17531.29	1
I00.I99	Netherlands	4485.07	2
I00.I99	Norway	2175.81	2
I00.I99	Poland	39223.27	2
I00.I99	Romania	50217.90	1
I00.I99	Slovenia	1558.94	1
I00.I99	Spain	16262.86	1
I00.I99	Sweden	7333.32	1
I00.I99	Switzerland	7136.77	2
I00.I99	United Kingdom	59798.45	3
I00.I99	Austria	304.90	2
I00.I99	Belgium	909.14	1
I00.I99	Croatia	463.27	3
I00.I99	Czech Republic	2021.35	2
I00.I99	Denmark	251.29	1
I00.I99	Estonia	52.83	0
I00.I99	Finland	211.21	1
I00.I99	France	4770.77	3

For peer review only

I00.I99	Germany	7521.30	3
I00.I99	Hungary	336.65	3
I00.I99	Latvia	45.51	1
I00.I99	Lithuania	267.09	0
I00.I99	Netherlands	584.07	3
I00.I99	Norway	158.13	1
I00.I99	Poland	3576.44	3
I00.I99	Romania	1060.17	2
I00.I99	Slovenia	103.19	1
I00.I99	Spain	2453.75	1
I00.I99	Sweden	471.96	2
I00.I99	Switzerland	376.08	2
I00.I99	United Kingdom	3523.93	3
I00.I99	Austria	110.47	3
I00.I99	Belgium	134.46	2
I00.I99	Croatia	122.92	3
I00.I99	Czech Republic	293.08	1
I00.I99	Denmark	56.56	0
I00.I99	Estonia	8.15	2
I00.I99	Finland	18.68	1
I00.I99	France	952.68	1
I00.I99	Germany	1861.30	2
I00.I99	Hungary	381.71	1
I00.I99	Latvia	11.77	1
I00.I99	Lithuania	24.90	2
I00.I99	Netherlands	201.56	2
I00.I99	Norway	37.02	0
I00.I99	Poland	1714.20	3
I00.I99	Romania	7548.71	2
I00.I99	Slovenia	49.63	1
I00.I99	Spain	735.74	3
I00.I99	Sweden	79.14	2
I00.I99	Switzerland	106.50	0
I00.I99	United Kingdom	567.12	2
I00.I99	Czech Republic	8.15	0
I00.I99	France	9.98	0
I00.I99	Germany	7.41	2
I00.I99	Poland	5.92	0
I00.I99	Romania	54.65	1
I00.I99	Spain	6.89	0
I00.I99	United Kingdom	8.04	1
I00.I99	Austria	7.96	0
I00.I99	Czech Republic	17.41	0
I00.I99	Finland	12.39	1
I00.I99	France	10.56	2
I00.I99	Germany	23.31	1
I00.I99	Hungary	18.91	2
I00.I99	Netherlands	5.79	2
I00.I99	Poland	22.94	0
I00.I99	Romania	30.25	0

For peer review only

I00.I99	Spain	13.30	0
I00.I99	Sweden	8.49	1
I00.I99	United Kingdom	69.80	1
I00.I99	Austria	15.17	1
I00.I99	Belgium	33.90	2
I00.I99	Croatia	7.85	0
I00.I99	Czech Republic	56.77	2
I00.I99	Denmark	9.89	0
I00.I99	France	174.81	1
I00.I99	Germany	135.59	3
I00.I99	Hungary	22.95	1
I00.I99	Netherlands	71.77	1
I00.I99	Norway	6.66	3
I00.I99	Poland	39.61	2
I00.I99	Romania	56.68	1
I00.I99	Slovenia	5.64	1
I00.I99	Spain	273.31	2
I00.I99	Sweden	16.82	2
I00.I99	Switzerland	11.63	0
I00.I99	United Kingdom	159.67	0
I00.I99	Austria	11.61	0
I00.I99	Belgium	41.72	2
I00.I99	Croatia	9.34	1
I00.I99	Czech Republic	80.46	0
I00.I99	Denmark	36.68	3
I00.I99	Estonia	19.02	3
I00.I99	Finland	28.83	1
I00.I99	France	273.38	2
I00.I99	Germany	252.22	3
I00.I99	Hungary	76.05	2
I00.I99	Latvia	16.07	0
I00.I99	Lithuania	22.63	0
I00.I99	Netherlands	80.61	2
I00.I99	Norway	12.26	2
I00.I99	Poland	151.30	2
I00.I99	Romania	56.10	1
I00.I99	Slovenia	6.98	0
I00.I99	Spain	236.15	1
I00.I99	Sweden	28.46	0
I00.I99	Switzerland	59.86	0
I00.I99	United Kingdom	378.67	2
I00.I99	Austria	184.86	2
I00.I99	Belgium	100.32	2
I00.I99	Croatia	55.10	3
I00.I99	Czech Republic	174.89	2
I00.I99	Denmark	92.78	1
I00.I99	Estonia	13.34	0
I00.I99	Finland	110.01	0
I00.I99	France	56.18	2
I00.I99	Germany	1611.76	1

For peer review only

I00.I99	Hungary	402.87	1
I00.I99	Latvia	6.38	1
I00.I99	Lithuania	9.36	0
I00.I99	Netherlands	715.26	2
I00.I99	Norway	70.06	0
I00.I99	Poland	422.45	0
I00.I99	Romania	158.26	2
I00.I99	Slovenia	53.56	0
I00.I99	Spain	581.17	1
I00.I99	Sweden	108.79	0
I00.I99	Switzerland	113.95	1
I00.I99	United Kingdom	587.52	0
I00.I99	Austria	639.93	2
I00.I99	Belgium	819.66	3
I00.I99	Croatia	168.43	3
I00.I99	Czech Republic	402.31	2
I00.I99	Denmark	432.67	2
I00.I99	Estonia	65.65	2
I00.I99	Finland	468.98	3
I00.I99	France	3967.82	3
I00.I99	Germany	7199.93	2
I00.I99	Hungary	819.40	1
I00.I99	Latvia	43.68	1
I00.I99	Lithuania	43.91	1
I00.I99	Netherlands	1306.03	2
I00.I99	Norway	495.89	0
I00.I99	Poland	891.90	2
I00.I99	Romania	247.91	1
I00.I99	Slovenia	226.36	2
I00.I99	Spain	2760.54	1
I00.I99	Sweden	673.35	1
I00.I99	Switzerland	487.66	0
I00.I99	United Kingdom	3850.34	2
I00.I99	Austria	24.90	1
I00.I99	Czech Republic	21.51	1
I00.I99	Finland	15.42	2
I00.I99	Germany	139.92	1
I00.I99	Hungary	29.97	0
I00.I99	Netherlands	22.35	2
I00.I99	Poland	32.02	2
I00.I99	Romania	7.67	0
I00.I99	Spain	6.63	0
I00.I99	Germany	9.79	0
I00.I99	Romania	7.66	1
I00.I99	Austria	68.23	0
I00.I99	Belgium	334.37	1
I00.I99	Croatia	7.00	1
I00.I99	Czech Republic	20.41	1
I00.I99	Denmark	38.79	0
I00.I99	France	1453.32	2

For peer review only

I00.I99	Germany	1203.76	2
I00.I99	Hungary	139.45	2
I00.I99	Netherlands	729.10	0
I00.I99	Norway	71.19	1
I00.I99	Poland	160.70	3
I00.I99	Romania	12.12	2
I00.I99	Spain	978.26	2
I00.I99	Sweden	118.20	0
I00.I99	Switzerland	362.19	2
I00.I99	United Kingdom	889.72	1
I00.I99	Czech Republic	15.94	0
I00.I99	Denmark	12.71	1
I00.I99	Finland	34.68	2
I00.I99	France	11.87	0
I00.I99	Germany	93.17	2
I00.I99	Hungary	5.80	2
I00.I99	Netherlands	6.63	2
I00.I99	Norway	5.49	0
I00.I99	Poland	52.89	1
I00.I99	Romania	52.31	1
I00.I99	Spain	10.29	0
I00.I99	Sweden	22.98	0
I00.I99	United Kingdom	57.28	0
I00.I99	Austria	1457.24	3
I00.I99	Belgium	660.91	2
I00.I99	Croatia	326.03	0
I00.I99	Czech Republic	298.99	2
I00.I99	Denmark	156.26	1
I00.I99	Estonia	264.01	1
I00.I99	Finland	452.48	2
I00.I99	France	3765.71	3
I00.I99	Germany	6678.86	1
I00.I99	Hungary	1555.47	2
I00.I99	Latvia	887.15	3
I00.I99	Lithuania	583.02	0
I00.I99	Netherlands	646.93	2
I00.I99	Norway	123.89	0
I00.I99	Poland	3796.33	0
I00.I99	Romania	4263.48	0
I00.I99	Slovenia	854.76	3
I00.I99	Spain	3290.13	3
I00.I99	Sweden	337.48	1
I00.I99	Switzerland	328.21	2
I00.I99	United Kingdom	1673.18	2
I00.I99	Austria	14.71	2
I00.I99	Belgium	81.72	1
I00.I99	Croatia	14.45	1
I00.I99	Czech Republic	26.50	0
I00.I99	Denmark	57.22	3
I00.I99	Finland	14.32	0

For peer review only

I00.I99	France	427.52	1
I00.I99	Germany	467.03	1
I00.I99	Hungary	19.02	1
I00.I99	Netherlands	74.23	0
I00.I99	Norway	25.10	0
I00.I99	Poland	139.77	1
I00.I99	Slovenia	15.60	0
I00.I99	Spain	517.34	3
I00.I99	Sweden	39.02	1
I00.I99	Switzerland	37.59	0
I00.I99	United Kingdom	95.38	0
I00.I99	Belgium	59.43	1
I00.I99	Croatia	5.13	0
I00.I99	Czech Republic	13.46	1
I00.I99	Denmark	13.93	0
I00.I99	France	153.81	1
I00.I99	Germany	84.41	2
I00.I99	Hungary	13.70	1
I00.I99	Netherlands	75.03	1
I00.I99	Poland	48.53	1
I00.I99	Slovenia	5.90	1
I00.I99	Spain	143.60	1
I00.I99	Sweden	7.25	0
I00.I99	Switzerland	12.35	0
I00.I99	United Kingdom	64.24	0
I00.I99	Austria	185.45	3
I00.I99	Belgium	2222.61	2
I00.I99	Croatia	744.62	1
I00.I99	Czech Republic	940.80	2
I00.I99	Denmark	345.02	3
I00.I99	Estonia	40.42	2
I00.I99	Finland	13.57	0
I00.I99	France	5190.04	2
I00.I99	Germany	4653.47	1
I00.I99	Hungary	46.33	2
I00.I99	Latvia	15.69	0
I00.I99	Lithuania	161.88	2
I00.I99	Netherlands	3165.96	1
I00.I99	Norway	181.58	0
I00.I99	Poland	8349.63	0
I00.I99	Slovenia	171.83	3
I00.I99	Spain	86.52	1
I00.I99	Sweden	233.64	2
I00.I99	Switzerland	994.15	3
I00.I99	United Kingdom	148.07	0
I00.I99	Austria	55.90	2
I00.I99	Belgium	39.11	2
I00.I99	Croatia	5.34	0
I00.I99	Czech Republic	15.18	0
I00.I99	Denmark	14.78	0

For peer review only

I00.I99	France	151.27	1
I00.I99	Germany	120.00	1
I00.I99	Hungary	8.42	1
I00.I99	Netherlands	25.82	2
I00.I99	Norway	6.66	0
I00.I99	Poland	40.65	2
I00.I99	Spain	124.77	2
I00.I99	Sweden	7.58	0
I00.I99	Switzerland	10.90	0
I00.I99	United Kingdom	32.96	1
I00.I99	Austria	1117.41	3
I00.I99	Belgium	1349.46	0
I00.I99	Croatia	101.22	1
I00.I99	Czech Republic	508.01	3
I00.I99	Denmark	863.90	3
I00.I99	Estonia	60.90	3
I00.I99	Finland	369.82	3
I00.I99	France	5335.15	1
I00.I99	Germany	13525.87	3
I00.I99	Hungary	566.67	2
I00.I99	Latvia	12.96	3
I00.I99	Lithuania	37.54	0
I00.I99	Netherlands	1862.07	2
I00.I99	Norway	810.08	0
I00.I99	Poland	1769.55	2
I00.I99	Slovenia	133.19	0
I00.I99	Spain	4685.60	2
I00.I99	Sweden	2010.98	1
I00.I99	Switzerland	391.52	1
I00.I99	United Kingdom	4788.59	2
I00.I99	Austria	173.23	3
I00.I99	Belgium	1018.28	2
I00.I99	Croatia	22.91	3
I00.I99	Czech Republic	177.71	0
I00.I99	Denmark	109.75	0
I00.I99	Estonia	6.21	0
I00.I99	Finland	27.35	0
I00.I99	France	5632.77	3
I00.I99	Germany	4955.52	2
I00.I99	Hungary	144.97	0
I00.I99	Lithuania	49.00	1
I00.I99	Netherlands	936.58	1
I00.I99	Norway	90.90	1
I00.I99	Poland	417.00	1
I00.I99	Slovenia	28.86	0
I00.I99	Spain	1489.64	2
I00.I99	Sweden	197.40	0
I00.I99	Switzerland	256.83	1
I00.I99	United Kingdom	246.37	2
I00.I99	Austria	2372.75	2

For peer review only

I00.I99	Belgium	5368.50	2
I00.I99	Croatia	3784.70	3
I00.I99	Czech Republic	3191.96	1
I00.I99	Denmark	1556.62	1
I00.I99	Estonia	177.44	2
I00.I99	Finland	469.68	2
I00.I99	France	23643.30	1
I00.I99	Germany	54870.33	2
I00.I99	Hungary	1866.89	2
I00.I99	Latvia	324.36	3
I00.I99	Lithuania	341.15	3
I00.I99	Netherlands	8571.22	3
I00.I99	Norway	1750.66	2
I00.I99	Poland	41647.45	2
I00.I99	Slovenia	1495.94	3
I00.I99	Spain	21476.66	3
I00.I99	Sweden	3581.84	2
I00.I99	Switzerland	2390.62	2
I00.I99	United Kingdom	10112.86	1
I00.I99	Austria	1981.80	3
I00.I99	Belgium	1551.99	1
I00.I99	Croatia	1442.58	3
I00.I99	Czech Republic	403.14	1
I00.I99	Denmark	452.29	1
I00.I99	Estonia	191.48	3
I00.I99	Finland	241.76	0
I00.I99	France	4168.93	1
I00.I99	Germany	2244.81	3
I00.I99	Hungary	1558.56	2
I00.I99	Latvia	90.16	2
I00.I99	Lithuania	17.03	0
I00.I99	Netherlands	1145.91	3
I00.I99	Norway	438.70	1
I00.I99	Poland	7888.00	1
I00.I99	Romania	8053.11	3
I00.I99	Slovenia	140.02	1
I00.I99	Spain	2782.26	1
I00.I99	Sweden	638.49	3
I00.I99	Switzerland	584.60	1
I00.I99	United Kingdom	3463.52	0
I00.I99	Austria	238.42	0
I00.I99	Belgium	252.28	1
I00.I99	Croatia	130.60	0
I00.I99	Czech Republic	268.38	0
I00.I99	Denmark	200.74	1
I00.I99	Estonia	58.78	2
I00.I99	Finland	329.85	1
I00.I99	France	1364.79	2
I00.I99	Germany	2057.10	1
I00.I99	Hungary	371.19	2

For peer review only

I00.I99	Latvia	100.22	0
I00.I99	Lithuania	155.20	0
I00.I99	Netherlands	465.76	1
I00.I99	Norway	151.86	1
I00.I99	Poland	1593.75	2
I00.I99	Romania	730.16	2
I00.I99	Slovenia	66.94	0
I00.I99	Spain	1041.63	1
I00.I99	Sweden	288.17	1
I00.I99	Switzerland	174.57	2
I00.I99	United Kingdom	2526.85	2
I00.I99	Austria	973.84	3
I00.I99	Belgium	1679.19	1
I00.I99	Croatia	699.59	2
I00.I99	Czech Republic	1529.54	1
I00.I99	Denmark	861.16	3
I00.I99	Estonia	259.16	2
I00.I99	Finland	917.39	2
I00.I99	France	7591.93	1
I00.I99	Germany	8323.13	3
I00.I99	Hungary	2964.78	2
I00.I99	Latvia	724.27	2
I00.I99	Lithuania	689.31	1
I00.I99	Netherlands	2130.66	3
I00.I99	Norway	609.72	2
I00.I99	Poland	7813.51	1
I00.I99	Romania	6881.02	2
I00.I99	Slovenia	312.13	3
I00.I99	Spain	6790.25	3
I00.I99	Sweden	1190.28	2
I00.I99	Switzerland	602.14	1
I00.I99	United Kingdom	5994.60	2
I00.I99	Austria	118.47	3
I00.I99	Belgium	284.39	2
I00.I99	Croatia	50.81	1
I00.I99	Czech Republic	102.64	1
I00.I99	Denmark	65.16	2
I00.I99	Estonia	22.83	0
I00.I99	Finland	28.71	1
I00.I99	France	719.03	1
I00.I99	Germany	854.19	2
I00.I99	Hungary	94.56	0
I00.I99	Latvia	15.05	0
I00.I99	Lithuania	29.65	1
I00.I99	Netherlands	219.65	1
I00.I99	Norway	65.42	3
I00.I99	Poland	370.01	0
I00.I99	Romania	786.94	2
I00.I99	Slovenia	23.29	0
I00.I99	Spain	1155.55	2

For peer review only

I00.I99	Sweden	73.16	0
I00.I99	Switzerland	74.36	0
I00.I99	United Kingdom	1487.96	2
I00.I99	Austria	1525.41	3
I00.I99	Belgium	994.06	3
I00.I99	Croatia	858.73	2
I00.I99	Czech Republic	3387.60	3
I00.I99	Denmark	672.28	2
I00.I99	Estonia	1631.25	3
I00.I99	Finland	2602.50	2
I00.I99	France	7063.35	1
I00.I99	Germany	14657.00	2
I00.I99	Hungary	8888.22	2
I00.I99	Latvia	2486.29	3
I00.I99	Lithuania	3416.08	2
I00.I99	Netherlands	1911.39	3
I00.I99	Norway	538.61	1
I00.I99	Poland	17791.08	3
I00.I99	Romania	3766.43	3
I00.I99	Slovenia	513.60	0
I00.I99	Spain	4966.81	2
I00.I99	Sweden	1951.91	2
I00.I99	Switzerland	565.09	3
I00.I99	United Kingdom	6055.64	2
I00.I99	Austria	2854.49	3
I00.I99	Belgium	4784.77	1
I00.I99	Croatia	6838.57	2
I00.I99	Czech Republic	7456.71	2
I00.I99	Denmark	2666.18	1
I00.I99	Estonia	117.95	1
I00.I99	Finland	205.39	1
I00.I99	France	15826.91	2
I00.I99	Germany	30020.84	2
I00.I99	Hungary	1306.38	2
I00.I99	Latvia	760.31	3
I00.I99	Lithuania	371.06	3
I00.I99	Netherlands	7358.07	2
I00.I99	Norway	2011.62	3
I00.I99	Poland	18658.87	3
I00.I99	Romania	46541.44	1
I00.I99	Slovenia	1061.65	2
I00.I99	Spain	16415.46	3
I00.I99	Sweden	2230.47	1
I00.I99	Switzerland	2501.46	2
I00.I99	United Kingdom	30051.23	3
I00.I99	Austria	499.10	3
I00.I99	Belgium	498.79	2
I00.I99	Croatia	404.42	0
I00.I99	Czech Republic	5792.12	1
I00.I99	Denmark	103.43	2

For peer review only

I00.I99	Estonia	131.46	1
I00.I99	Finland	145.79	1
I00.I99	France	2394.07	3
I00.I99	Germany	8174.79	2
I00.I99	Hungary	3746.83	2
I00.I99	Latvia	2483.25	3
I00.I99	Lithuania	1990.34	0
I00.I99	Netherlands	161.70	2
I00.I99	Norway	86.31	1
I00.I99	Poland	4705.31	1
I00.I99	Romania	16741.32	3
I00.I99	Slovenia	28.07	2
I00.I99	Spain	6026.49	1
I00.I99	Sweden	924.66	2
I00.I99	Switzerland	393.94	1
I00.I99	United Kingdom	11450.12	2
I00.I99	Austria	264.22	1
I00.I99	Belgium	298.15	0
I00.I99	Croatia	1452.77	3
I00.I99	Czech Republic	738.46	2
I00.I99	Denmark	565.13	3
I00.I99	Estonia	81.01	2
I00.I99	Finland	1199.59	2
I00.I99	France	2345.75	2
I00.I99	Germany	5722.16	3
I00.I99	Hungary	1939.16	2
I00.I99	Latvia	356.62	2
I00.I99	Lithuania	524.68	3
I00.I99	Netherlands	1034.02	3
I00.I99	Norway	594.28	2
I00.I99	Poland	4755.96	1
I00.I99	Slovenia	545.15	0
I00.I99	Spain	486.51	2
I00.I99	Sweden	1534.11	1
I00.I99	Switzerland	357.98	3
I00.I99	United Kingdom	3107.00	1
I00.I99	Austria	899.14	3
I00.I99	Belgium	443.52	2
I00.I99	Croatia	1715.82	3
I00.I99	Czech Republic	10307.78	1
I00.I99	Denmark	768.15	3
I00.I99	Estonia	310.65	3
I00.I99	Finland	436.56	0
I00.I99	France	716.86	2
I00.I99	Germany	11478.56	3
I00.I99	Hungary	5403.13	2
I00.I99	Latvia	993.74	3
I00.I99	Lithuania	892.43	3
I00.I99	Netherlands	1574.94	1
I00.I99	Norway	328.23	2

For peer review only

I00.I99	Poland	55059.60	1
I00.I99	Romania	24108.14	3
I00.I99	Slovenia	391.79	1
I00.I99	Spain	3058.62	2
I00.I99	Sweden	1114.11	3
I00.I99	Switzerland	720.35	3
I00.I99	United Kingdom	787.84	2
I00.I99	Austria	383.00	2
I00.I99	Belgium	704.02	1
I00.I99	Croatia	314.53	1
I00.I99	Czech Republic	628.13	1
I00.I99	Denmark	627.28	1
I00.I99	Estonia	85.08	0
I00.I99	Finland	621.21	2
I00.I99	France	3077.05	1
I00.I99	Germany	3740.29	1
I00.I99	Hungary	580.69	2
I00.I99	Latvia	105.00	1
I00.I99	Lithuania	173.47	0
I00.I99	Netherlands	1717.91	1
I00.I99	Norway	578.33	1
I00.I99	Poland	2341.33	2
I00.I99	Romania	483.15	2
I00.I99	Slovenia	118.31	0
I00.I99	Spain	2213.61	1
I00.I99	Sweden	1053.48	1
I00.I99	Switzerland	512.07	1
I00.I99	United Kingdom	9329.53	3
I00.I99	Austria	18.27	2
I00.I99	Belgium	84.62	1
I00.I99	Croatia	8.69	0
I00.I99	Czech Republic	34.74	2
I00.I99	Denmark	20.89	0
I00.I99	Finland	15.56	2
I00.I99	France	556.88	2
I00.I99	Germany	189.49	2
I00.I99	Hungary	26.26	0
I00.I99	Lithuania	7.96	0
I00.I99	Netherlands	293.28	1
I00.I99	Norway	14.23	0
I00.I99	Poland	82.38	0
I00.I99	Romania	103.57	3
I00.I99	Slovenia	6.27	0
I00.I99	Spain	113.50	0
I00.I99	Sweden	35.07	1
I00.I99	Switzerland	28.61	0
I00.I99	United Kingdom	266.73	1
I00.I99	Austria	664.09	1
I00.I99	Belgium	78.30	3
I00.I99	Czech Republic	8.32	1

For peer review only

I00.I99	Denmark	11.58	2
I00.I99	France	506.70	2
I00.I99	Germany	4301.01	3
I00.I99	Hungary	38.08	2
I00.I99	Netherlands	253.32	1
I00.I99	Norway	17.79	3
I00.I99	Poland	37.73	2
I00.I99	Romania	41.86	3
I00.I99	Slovenia	8.57	0
I00.I99	Spain	697.44	2
I00.I99	Sweden	157.34	2
I00.I99	Switzerland	20.83	1
I00.I99	United Kingdom	3044.49	3
I00.I99	Austria	35.17	0
I00.I99	Belgium	188.61	2
I00.I99	Croatia	73.99	3
I00.I99	Czech Republic	176.86	0
I00.I99	Denmark	57.02	1
I00.I99	Estonia	29.06	0
I00.I99	Finland	65.45	2
I00.I99	France	1404.73	2
I00.I99	Germany	2136.50	1
I00.I99	Hungary	302.34	2
I00.I99	Latvia	70.89	2
I00.I99	Lithuania	82.64	0
I00.I99	Netherlands	298.87	1
I00.I99	Norway	39.20	2
I00.I99	Poland	855.25	3
I00.I99	Romania	101.19	0
I00.I99	Slovenia	45.60	1
I00.I99	Spain	360.73	1
I00.I99	Sweden	72.98	3
I00.I99	Switzerland	100.73	1
I00.I99	United Kingdom	195.43	2
I00.I99	Austria	7.14	1
I00.I99	Belgium	224.52	0
I00.I99	Czech Republic	15.16	1
I00.I99	Denmark	12.59	0
I00.I99	Finland	5.52	1
I00.I99	France	4062.33	3
I00.I99	Germany	150.67	0
I00.I99	Hungary	57.71	0
I00.I99	Netherlands	72.10	1
I00.I99	Norway	15.96	1
I00.I99	Poland	19.22	1
I00.I99	Romania	245.97	2
I00.I99	Spain	451.62	0
I00.I99	Sweden	70.02	0
I00.I99	Switzerland	50.76	0
I00.I99	United Kingdom	333.28	2

For peer review only

I00.I99	France	34.65	0
I00.I99	Germany	30.23	3
I00.I99	Netherlands	5.96	0
I00.I99	Spain	13.32	0
I00.I99	United Kingdom	11.19	0
I00.I99	Austria	324.28	3
I00.I99	Belgium	159.13	2
I00.I99	Croatia	31.36	1
I00.I99	Czech Republic	434.17	1
I00.I99	Denmark	45.39	1
I00.I99	Estonia	50.18	1
I00.I99	Finland	166.39	0
I00.I99	France	1089.59	1
I00.I99	Germany	2359.64	3
I00.I99	Hungary	694.52	2
I00.I99	Latvia	45.36	0
I00.I99	Lithuania	113.70	3
I00.I99	Netherlands	113.27	2
I00.I99	Norway	54.75	2
I00.I99	Poland	217.33	2
I00.I99	Romania	44.44	0
I00.I99	Slovenia	75.75	3
I00.I99	Spain	359.72	1
I00.I99	Sweden	92.29	2
I00.I99	Switzerland	79.63	2
I00.I99	United Kingdom	4177.04	1
I00.I99	Czech Republic	8.12	0
I00.I99	France	36.48	0
I00.I99	Germany	29.91	1
I00.I99	Hungary	11.74	0
I00.I99	Netherlands	5.68	0
I00.I99	Poland	12.08	0
I00.I99	Romania	5.86	0
I00.I99	Spain	10.05	2
I00.I99	Sweden	6.94	0
I00.I99	United Kingdom	37.14	1
I00.I99	Austria	15.86	0
I00.I99	Belgium	32.57	1
I00.I99	Croatia	5.49	2
I00.I99	Czech Republic	78.25	1
I00.I99	Denmark	19.51	3
I00.I99	France	75.04	1
I00.I99	Germany	319.29	1
I00.I99	Hungary	66.34	3
I00.I99	Netherlands	23.22	1
I00.I99	Poland	42.65	0
I00.I99	Romania	21.85	1
I00.I99	Slovenia	7.12	1
I00.I99	Spain	93.83	3
I00.I99	Sweden	24.06	0

For peer review only

I00.I99	Switzerland	25.90	0
I00.I99	United Kingdom	80.35	1
I00.I99	Austria	18.47	0
I00.I99	Belgium	13.06	1
I00.I99	Croatia	9.76	0
I00.I99	Czech Republic	79.15	0
I00.I99	Denmark	5.65	1
I00.I99	Finland	7.53	0
I00.I99	France	101.62	1
I00.I99	Germany	147.06	2
I00.I99	Hungary	66.28	0
I00.I99	Latvia	6.94	0
I00.I99	Lithuania	5.24	0
I00.I99	Netherlands	6.43	0
I00.I99	Poland	45.58	1
I00.I99	Slovenia	5.43	0
I00.I99	Spain	31.17	0
I00.I99	Sweden	6.77	0
I00.I99	Switzerland	14.26	1
I00.I99	United Kingdom	133.65	0
I00.I99	Germany	5.44	2
I00.I99	Austria	7.07	0
I00.I99	Belgium	24.35	0
I00.I99	Croatia	19.94	0
I00.I99	Czech Republic	41.42	1
I00.I99	Denmark	8.14	1
I00.I99	France	127.54	1
I00.I99	Germany	233.52	1
I00.I99	Lithuania	11.07	1
I00.I99	Netherlands	13.61	1
I00.I99	Poland	59.18	2
I00.I99	Spain	74.33	1
I00.I99	Sweden	8.79	0
I00.I99	Switzerland	7.01	0
I00.I99	United Kingdom	51.82	1
I00.I99	France	9.98	0
I00.I99	Germany	7.36	0
I00.I99	Austria	6.40	0
I00.I99	Belgium	9.22	0
I00.I99	Czech Republic	6.09	0
I00.I99	France	82.30	3
I00.I99	Germany	76.88	1
I00.I99	Hungary	16.09	1
I00.I99	Netherlands	6.71	0
I00.I99	Spain	74.16	0
I00.I99	Switzerland	17.45	0
I00.I99	United Kingdom	29.05	1
I00.I99	France	28.71	1
I00.I99	Germany	19.45	1
I00.I99	Spain	9.32	0

For peer review only

I00.I99	United Kingdom	31.60	0
I00.I99	Belgium	8.61	3
I00.I99	Denmark	9.36	0
I00.I99	France	20.39	0
I00.I99	Germany	19.11	2
I00.I99	Netherlands	6.36	0
I00.I99	Spain	11.59	0
I00.I99	United Kingdom	8.00	0
I00.I99	Belgium	55.45	1
I00.I99	Denmark	7.82	0
I00.I99	France	181.14	1
I00.I99	Germany	74.12	1
I00.I99	Netherlands	46.25	1
I00.I99	Norway	9.01	1
I00.I99	Poland	33.67	0
I00.I99	Romania	1360.42	3
I00.I99	Spain	90.81	1
I00.I99	Sweden	23.42	0
I00.I99	Switzerland	12.71	2
I00.I99	United Kingdom	23.10	1
J00.J99	Spain	12.34	0
J00.J99	France	10.25	1
J00.J99	United Kingdom	5.62	0
J00.J99	Germany	9.01	0
J00.J99	Czech Republic	5.45	1
J00.J99	France	7.61	1
J00.J99	Germany	10.14	0
J00.J99	United Kingdom	5.34	0
J00.J99	United Kingdom	11.94	0
J00.J99	Austria	14.00	2
J00.J99	Belgium	13.50	1
J00.J99	Czech Republic	7.62	0
J00.J99	Germany	171.62	1
J00.J99	Netherlands	29.65	3
J00.J99	Poland	7.94	0
J00.J99	Spain	18.91	0
J00.J99	Sweden	19.21	0
J00.J99	Switzerland	25.54	1
J00.J99	United Kingdom	22.04	1
J00.J99	Czech Republic	19.53	1
J00.J99	Finland	17.69	3
J00.J99	Germany	18.51	0
J00.J99	Netherlands	15.93	2
J00.J99	Romania	16.16	0
J00.J99	Sweden	13.84	2
J00.J99	United Kingdom	43.98	2
J00.J99	Belgium	133.14	2
J00.J99	Croatia	27.71	0
J00.J99	Czech Republic	66.95	0
J00.J99	Denmark	31.24	1

For peer review only

J00.J99	Finland	22.30	1
J00.J99	France	492.88	0
J00.J99	Germany	156.01	0
J00.J99	Hungary	13.50	0
J00.J99	Netherlands	169.67	0
J00.J99	Norway	39.21	0
J00.J99	Poland	48.77	1
J00.J99	Romania	11.09	2
J00.J99	Spain	177.00	0
J00.J99	Sweden	89.15	0
J00.J99	Switzerland	74.26	2
J00.J99	United Kingdom	58.66	2
J00.J99	Belgium	18.64	1
J00.J99	Czech Republic	35.60	2
J00.J99	Denmark	22.90	0
J00.J99	Estonia	5.68	2
J00.J99	Finland	5.84	2
J00.J99	France	31.44	1
J00.J99	Germany	44.61	1
J00.J99	Hungary	42.22	2
J00.J99	Netherlands	38.56	2
J00.J99	Poland	18.89	0
J00.J99	Romania	49.74	1
J00.J99	Spain	9.49	0
J00.J99	Sweden	9.46	1
J00.J99	Switzerland	6.26	2
J00.J99	United Kingdom	32.11	2
J00.J99	Belgium	51.52	1
J00.J99	Czech Republic	11.91	2
J00.J99	Denmark	30.58	2
J00.J99	Estonia	27.52	3
J00.J99	Finland	21.94	2
J00.J99	France	343.01	2
J00.J99	Germany	65.41	2
J00.J99	Netherlands	56.74	1
J00.J99	Norway	7.35	0
J00.J99	Poland	22.16	3
J00.J99	Spain	173.86	2
J00.J99	Sweden	20.38	1
J00.J99	Switzerland	42.12	1
J00.J99	United Kingdom	126.90	0
J00.J99	United Kingdom	7.65	0
J00.J99	Austria	7.34	0
J00.J99	Belgium	243.19	3
J00.J99	Croatia	19.47	1
J00.J99	Czech Republic	330.87	0
J00.J99	Denmark	557.30	3
J00.J99	Estonia	148.37	2
J00.J99	Finland	340.50	2
J00.J99	France	491.42	1

For peer review only

J00.J99	Germany	523.37	0
J00.J99	Hungary	220.61	2
J00.J99	Lithuania	69.58	0
J00.J99	Netherlands	37.92	1
J00.J99	Norway	14.11	0
J00.J99	Poland	1525.79	2
J00.J99	Romania	53.13	3
J00.J99	Slovenia	105.01	1
J00.J99	Spain	226.82	1
J00.J99	Sweden	37.38	3
J00.J99	Switzerland	29.90	0
J00.J99	United Kingdom	203.68	3
J00.J99	Czech Republic	30.21	0
J00.J99	Germany	233.45	3
J00.J99	Poland	58.92	2
J00.J99	Romania	15.90	0
J00.J99	Austria	1325.03	3
J00.J99	Belgium	4247.27	1
J00.J99	Croatia	1130.44	3
J00.J99	Czech Republic	3426.72	3
J00.J99	Denmark	1602.22	3
J00.J99	Estonia	50.22	0
J00.J99	Finland	847.14	2
J00.J99	France	11093.98	1
J00.J99	Germany	20727.57	3
J00.J99	Hungary	750.86	3
J00.J99	Latvia	422.49	0
J00.J99	Lithuania	548.04	3
J00.J99	Netherlands	6736.34	2
J00.J99	Norway	1989.06	2
J00.J99	Poland	13089.40	0
J00.J99	Romania	7361.71	2
J00.J99	Slovenia	817.32	1
J00.J99	Spain	9094.64	1
J00.J99	Sweden	2093.71	2
J00.J99	Switzerland	1525.09	1
J00.J99	United Kingdom	36743.70	3
J00.J99	Austria	9.05	2
J00.J99	Belgium	204.22	1
J00.J99	Croatia	7.64	1
J00.J99	Czech Republic	156.80	2
J00.J99	Denmark	10.78	0
J00.J99	Finland	7.94	1
J00.J99	France	2866.18	1
J00.J99	Germany	471.63	3
J00.J99	Hungary	149.91	2
J00.J99	Netherlands	47.73	0
J00.J99	Poland	37.24	1
J00.J99	Romania	12.87	1
J00.J99	Slovenia	16.58	1

For peer review only

J00.J99	Spain	370.68	0
J00.J99	Sweden	36.11	1
J00.J99	Switzerland	10.80	1
J00.J99	United Kingdom	231.64	3
J00.J99	Austria	6.20	1
J00.J99	Czech Republic	11.69	1
J00.J99	France	18.48	2
J00.J99	Germany	11.90	1
J00.J99	Hungary	18.97	0
J00.J99	Romania	22.65	1
J00.J99	Spain	14.18	0
J00.J99	United Kingdom	21.80	0
J00.J99	Austria	16.04	3
J00.J99	Belgium	264.83	1
J00.J99	Czech Republic	11.42	1
J00.J99	Denmark	8.37	1
J00.J99	Finland	5.95	1
J00.J99	France	368.08	2
J00.J99	Germany	274.41	2
J00.J99	Hungary	14.38	2
J00.J99	Netherlands	438.20	3
J00.J99	Norway	10.32	0
J00.J99	Poland	27.20	1
J00.J99	Slovenia	47.81	2
J00.J99	Spain	860.55	0
J00.J99	Sweden	42.59	2
J00.J99	Switzerland	15.77	0
J00.J99	United Kingdom	2187.91	1
J00.J99	France	14.59	0
J00.J99	Germany	7.27	2
J00.J99	United Kingdom	8.59	0
J00.J99	Belgium	8.76	0
J00.J99	France	61.96	1
J00.J99	Germany	16.99	0
J00.J99	Netherlands	6.04	0
J00.J99	Spain	21.46	1
J00.J99	United Kingdom	35.53	0
J00.J99	Belgium	11.16	0
J00.J99	France	44.69	0
J00.J99	Germany	34.42	0
J00.J99	Hungary	6.73	3
J00.J99	Netherlands	10.81	0
J00.J99	Norway	5.26	1
J00.J99	Spain	18.15	0
J00.J99	Sweden	8.90	0
J00.J99	United Kingdom	38.60	0
J00.J99	Austria	82.82	1
J00.J99	Belgium	315.59	1
J00.J99	Czech Republic	51.84	3
J00.J99	Denmark	5.20	0

For peer review only

J00.J99	France	500.02	1
J00.J99	Germany	506.78	0
J00.J99	Hungary	94.39	2
J00.J99	Netherlands	42.83	2
J00.J99	Norway	8.60	0
J00.J99	Poland	29.15	1
J00.J99	Spain	277.29	3
J00.J99	Sweden	14.55	0
J00.J99	Switzerland	32.57	1
J00.J99	United Kingdom	88.16	2
J00.J99	Austria	210.29	1
J00.J99	Czech Republic	62.45	0
J00.J99	Denmark	12.99	2
J00.J99	Germany	175.79	1
J00.J99	Hungary	273.51	1
J00.J99	Lithuania	12.35	2
J00.J99	Poland	65.56	3
J00.J99	Romania	39.61	1
J00.J99	Sweden	6.93	2
J00.J99	United Kingdom	42.80	2
J00.J99	Austria	34.89	3
J00.J99	Belgium	139.71	2
J00.J99	Croatia	43.37	3
J00.J99	Czech Republic	187.46	1
J00.J99	Denmark	207.41	2
J00.J99	Finland	5.51	2
J00.J99	France	641.47	2
J00.J99	Germany	576.34	2
J00.J99	Hungary	709.89	2
J00.J99	Latvia	20.75	0
J00.J99	Lithuania	32.05	2
J00.J99	Netherlands	33.32	2
J00.J99	Norway	8.36	2
J00.J99	Poland	399.23	2
J00.J99	Romania	313.72	1
J00.J99	Slovenia	8.02	2
J00.J99	Spain	853.04	1
J00.J99	Sweden	25.84	3
J00.J99	Switzerland	41.42	2
J00.J99	United Kingdom	156.20	2
J00.J99	Austria	606.65	3
J00.J99	Belgium	385.09	2
J00.J99	Croatia	20.83	1
J00.J99	Czech Republic	133.77	3
J00.J99	Denmark	74.21	1
J00.J99	Finland	40.17	0
J00.J99	France	676.32	2
J00.J99	Germany	1691.34	2
J00.J99	Hungary	1065.21	3
J00.J99	Latvia	6.43	0

For peer review only

J00.J99	Lithuania	12.17	1
J00.J99	Netherlands	901.03	2
J00.J99	Norway	113.49	0
J00.J99	Poland	192.37	2
J00.J99	Romania	1058.58	3
J00.J99	Slovenia	18.45	1
J00.J99	Spain	744.38	2
J00.J99	Sweden	260.34	1
J00.J99	Switzerland	44.21	2
J00.J99	United Kingdom	1530.25	2
J00.J99	Austria	2039.82	1
J00.J99	Belgium	4285.28	2
J00.J99	Croatia	1588.64	1
J00.J99	Czech Republic	2512.51	1
J00.J99	Denmark	3655.94	3
J00.J99	Estonia	208.53	0
J00.J99	Finland	1207.98	1
J00.J99	France	6825.11	0
J00.J99	Germany	24056.18	1
J00.J99	Hungary	3169.09	1
J00.J99	Latvia	261.38	0
J00.J99	Lithuania	974.50	1
J00.J99	Netherlands	7233.98	0
J00.J99	Norway	2075.94	2
J00.J99	Poland	9255.15	2
J00.J99	Romania	7041.54	2
J00.J99	Slovenia	568.51	2
J00.J99	Spain	14748.69	1
J00.J99	Sweden	2406.12	1
J00.J99	Switzerland	1895.75	0
J00.J99	United Kingdom	30852.18	2
J00.J99	Austria	139.09	2
J00.J99	Belgium	173.05	2
J00.J99	Croatia	100.31	1
J00.J99	Czech Republic	131.84	2
J00.J99	Denmark	107.01	2
J00.J99	Estonia	48.91	2
J00.J99	Finland	107.29	1
J00.J99	France	1061.87	2
J00.J99	Germany	1624.06	2
J00.J99	Hungary	192.99	3
J00.J99	Latvia	60.62	2
J00.J99	Lithuania	57.76	1
J00.J99	Netherlands	92.67	2
J00.J99	Norway	126.82	3
J00.J99	Poland	761.50	2
J00.J99	Romania	504.10	1
J00.J99	Slovenia	33.18	2
J00.J99	Spain	912.69	1
J00.J99	Sweden	141.29	2

For peer review only

J00.J99	Switzerland	124.12	2
J00.J99	United Kingdom	1371.06	3
J00.J99	Austria	10.57	3
J00.J99	Belgium	29.83	2
J00.J99	Croatia	5.76	1
J00.J99	Czech Republic	8.26	0
J00.J99	Denmark	7.24	1
J00.J99	Finland	5.57	0
J00.J99	France	142.90	2
J00.J99	Germany	136.40	3
J00.J99	Hungary	6.84	0
J00.J99	Netherlands	8.71	1
J00.J99	Poland	134.72	2
J00.J99	Romania	19.81	1
J00.J99	Spain	98.85	3
J00.J99	Sweden	10.08	3
J00.J99	Switzerland	7.15	0
J00.J99	United Kingdom	59.14	2
J00.J99	Belgium	33.42	0
J00.J99	Croatia	9.87	2
J00.J99	Denmark	5.38	0
J00.J99	Finland	22.60	0
J00.J99	France	393.59	3
J00.J99	Germany	28.31	2
J00.J99	Hungary	236.05	3
J00.J99	Lithuania	6.30	0
J00.J99	Netherlands	28.02	3
J00.J99	Norway	7.15	0
J00.J99	Poland	23.53	1
J00.J99	Romania	27.25	1
J00.J99	Spain	445.43	0
J00.J99	Sweden	11.85	0
J00.J99	Switzerland	11.85	0
J00.J99	United Kingdom	1376.88	2
J00.J99	Belgium	109.88	1
J00.J99	Czech Republic	54.57	3
J00.J99	France	13.59	1
J00.J99	Germany	17.26	2
J00.J99	Hungary	15.92	1
J00.J99	Poland	29.54	3
J00.J99	United Kingdom	159.29	2
J00.J99	Belgium	19.85	0
J00.J99	Croatia	6.37	2
J00.J99	Denmark	9.83	1
J00.J99	Finland	25.66	0
J00.J99	France	115.79	2
J00.J99	Germany	135.37	1
J00.J99	Netherlands	12.12	0
J00.J99	Norway	12.42	1
J00.J99	Spain	20.84	1

For peer review only

J00.J99	Sweden	11.82	0
J00.J99	United Kingdom	193.25	1
J00.J99	Austria	12.24	0
J00.J99	Belgium	23.99	1
J00.J99	Czech Republic	10.93	2
J00.J99	France	401.19	2
J00.J99	Germany	294.49	1
J00.J99	Hungary	22.69	1
J00.J99	Netherlands	18.53	2
J00.J99	Poland	12.52	1
J00.J99	Romania	33.89	1
J00.J99	Spain	142.04	1
J00.J99	Sweden	5.34	1
J00.J99	Switzerland	11.30	1
J00.J99	United Kingdom	16.61	3
J00.J99	Germany	6.24	0
J00.J99	Poland	16.78	1
J00.J99	Belgium	27.06	3
J00.J99	Czech Republic	5.59	0
J00.J99	France	14.70	1
J00.J99	Germany	22.70	0
J00.J99	Poland	61.43	2
J00.J99	Romania	26.56	1
J00.J99	Spain	87.34	1
J00.J99	United Kingdom	30.90	1
J00.J99	France	8.39	3
J00.J99	Germany	8.71	2
J00.J99	Spain	6.94	1
J00.J99	Austria	10.45	0
J00.J99	Belgium	7.22	0
J00.J99	Finland	5.53	0
J00.J99	France	38.75	0
J00.J99	Germany	47.63	1
J00.J99	Netherlands	10.26	2
J00.J99	Poland	5.79	1
J00.J99	Romania	10.44	1
J00.J99	Spain	8.63	1
J00.J99	Switzerland	5.15	0
J00.J99	United Kingdom	68.04	2
J00.J99	Germany	10.80	0
J00.J99	Austria	106.51	0
J00.J99	Belgium	827.98	2
J00.J99	Czech Republic	30.71	1
J00.J99	Denmark	49.30	1
J00.J99	Finland	28.22	2
J00.J99	France	2353.73	2
J00.J99	Germany	4354.24	3
J00.J99	Hungary	10.87	1
J00.J99	Netherlands	420.49	2
J00.J99	Norway	49.13	0

For peer review only

J00.J99	Poland	305.79	3
J00.J99	Romania	20.73	1
J00.J99	Slovenia	69.85	2
J00.J99	Spain	1995.92	0
J00.J99	Sweden	133.54	2
J00.J99	Switzerland	166.29	2
J00.J99	United Kingdom	3262.29	2
J00.J99	Belgium	6.68	1
J00.J99	Czech Republic	6.63	1
J00.J99	Denmark	6.36	1
J00.J99	France	74.31	2
J00.J99	Germany	7.59	2
J00.J99	Poland	53.03	1
J00.J99	Belgium	36.75	1
J00.J99	Czech Republic	11.10	0
J00.J99	France	220.94	0
J00.J99	Germany	50.35	2
J00.J99	Norway	5.23	0
J00.J99	Poland	75.42	1
J00.J99	Spain	104.20	1
J00.J99	Switzerland	6.79	0
J00.J99	United Kingdom	65.36	1
J00.J99	Austria	44.12	3
J00.J99	Belgium	312.60	1
J00.J99	Croatia	57.84	2
J00.J99	Czech Republic	56.47	3
J00.J99	Denmark	61.35	1
J00.J99	France	402.62	3
J00.J99	Germany	582.28	1
J00.J99	Netherlands	5.49	0
J00.J99	Norway	38.98	1
J00.J99	Poland	538.32	3
J00.J99	Romania	859.45	0
J00.J99	Spain	2769.46	1
J00.J99	Sweden	40.57	2
J00.J99	Switzerland	42.06	2
J00.J99	United Kingdom	112.37	2
J00.J99	France	8.66	1
J00.J99	Spain	16.54	0
J00.J99	Sweden	5.88	1
J00.J99	Austria	135.47	2
J00.J99	Belgium	306.52	1
J00.J99	Croatia	23.31	0
J00.J99	Czech Republic	227.70	1
J00.J99	Denmark	150.21	2
J00.J99	Estonia	16.18	0
J00.J99	Finland	252.81	1
J00.J99	France	1520.31	2
J00.J99	Germany	2271.04	1
J00.J99	Hungary	184.99	2

For peer review only

J00.J99	Latvia	18.79	0
J00.J99	Lithuania	11.44	1
J00.J99	Netherlands	525.67	1
J00.J99	Norway	154.80	1
J00.J99	Poland	371.76	3
J00.J99	Romania	285.35	1
J00.J99	Slovenia	44.94	1
J00.J99	Spain	2119.58	1
J00.J99	Sweden	365.58	3
J00.J99	Switzerland	222.74	1
J00.J99	United Kingdom	4746.02	2
J00.J99	Austria	7.25	1
J00.J99	Belgium	17.02	0
J00.J99	Croatia	8.96	1
J00.J99	Czech Republic	31.00	0
J00.J99	Denmark	14.56	0
J00.J99	Estonia	33.38	2
J00.J99	Finland	7.12	1
J00.J99	France	89.66	1
J00.J99	Germany	40.85	0
J00.J99	Hungary	104.93	2
J00.J99	Latvia	32.77	0
J00.J99	Lithuania	34.02	1
J00.J99	Netherlands	7.80	0
J00.J99	Norway	9.57	1
J00.J99	Poland	116.61	1
J00.J99	Romania	197.81	1
J00.J99	Spain	50.96	2
J00.J99	Sweden	9.04	0
J00.J99	United Kingdom	141.29	1
J00.J99	Austria	17.26	0
J00.J99	Belgium	27.85	0
J00.J99	Croatia	9.75	1
J00.J99	Czech Republic	18.81	0
J00.J99	Denmark	18.79	0
J00.J99	Finland	15.76	2
J00.J99	France	113.22	1
J00.J99	Germany	179.36	1
J00.J99	Hungary	19.78	0
J00.J99	Latvia	6.11	0
J00.J99	Lithuania	23.21	0
J00.J99	Netherlands	36.40	2
J00.J99	Norway	8.10	0
J00.J99	Poland	141.51	0
J00.J99	Romania	32.96	3
J00.J99	Slovenia	6.28	0
J00.J99	Spain	99.18	1
J00.J99	Sweden	19.72	0
J00.J99	Switzerland	15.05	0
J00.J99	United Kingdom	248.25	3

For peer review only

J00.J99	Belgium	40.98	0
J00.J99	Czech Republic	8.93	2
J00.J99	Denmark	18.27	0
J00.J99	Finland	6.31	1
J00.J99	France	188.10	1
J00.J99	Germany	152.86	2
J00.J99	Netherlands	52.63	3
J00.J99	Norway	7.81	2
J00.J99	Poland	26.37	1
J00.J99	Romania	51.95	2
J00.J99	Spain	241.48	0
J00.J99	Sweden	11.33	0
J00.J99	Switzerland	10.55	0
J00.J99	United Kingdom	127.80	3
J00.J99	France	9.71	0
J00.J99	Spain	17.06	0
J00.J99	United Kingdom	11.86	1
J00.J99	Belgium	17.73	0
J00.J99	Czech Republic	6.01	0
J00.J99	Denmark	9.76	0
J00.J99	France	58.78	1
J00.J99	Germany	49.71	2
J00.J99	Netherlands	19.00	1
J00.J99	Norway	5.97	1
J00.J99	Poland	66.71	1
J00.J99	Romania	18.02	0
J00.J99	Spain	41.86	1
J00.J99	Sweden	6.56	0
J00.J99	United Kingdom	49.87	1
J00.J99	Belgium	11.49	0
J00.J99	Czech Republic	6.41	0
J00.J99	Denmark	9.63	1
J00.J99	France	48.76	0
J00.J99	Germany	43.35	2
J00.J99	Hungary	12.91	2
J00.J99	Netherlands	10.35	0
J00.J99	Poland	13.59	0
J00.J99	Romania	66.85	1
J00.J99	Slovenia	6.99	0
J00.J99	Spain	62.20	2
J00.J99	United Kingdom	36.98	2
J00.J99	Austria	6.42	0
J00.J99	Belgium	321.07	1
J00.J99	Croatia	38.92	0
J00.J99	Czech Republic	178.44	3
J00.J99	Denmark	131.03	1
J00.J99	Finland	6.20	1
J00.J99	France	4050.01	2
J00.J99	Germany	1154.20	2
J00.J99	Netherlands	183.36	3

For peer review only

J00.J99	Norway	32.23	0
J00.J99	Poland	725.08	0
J00.J99	Romania	195.46	2
J00.J99	Slovenia	8.76	2
J00.J99	Spain	3142.76	3
J00.J99	Sweden	24.45	2
J00.J99	Switzerland	57.69	1
J00.J99	United Kingdom	35.34	2
J00.J99	Austria	23.28	1
J00.J99	Belgium	373.80	3
J00.J99	Czech Republic	14.50	0
J00.J99	Denmark	48.44	1
J00.J99	France	1425.54	2
J00.J99	Germany	336.27	1
J00.J99	Hungary	77.78	2
J00.J99	Netherlands	510.39	3
J00.J99	Norway	58.05	2
J00.J99	Poland	24.34	1
J00.J99	Romania	30.94	0
J00.J99	Spain	8706.44	3
J00.J99	Sweden	90.77	2
J00.J99	Switzerland	86.65	0
J00.J99	United Kingdom	6641.62	2
K00.K93	United Kingdom	6.15	0
K00.K93	Netherlands	5.50	1
K00.K93	Germany	17.98	3
K00.K93	Netherlands	12.47	0
K00.K93	United Kingdom	36.25	0
K00.K93	Germany	7.20	1
K00.K93	Spain	5.29	0
K00.K93	United Kingdom	7.08	0
K00.K93	France	11.55	0
K00.K93	Belgium	32.86	1
K00.K93	Czech Republic	10.90	1
K00.K93	Denmark	5.67	0
K00.K93	France	63.94	2
K00.K93	Germany	44.74	0
K00.K93	Netherlands	9.86	1
K00.K93	Norway	7.96	1
K00.K93	Spain	27.03	0
K00.K93	Sweden	12.60	0
K00.K93	United Kingdom	117.07	1
K00.K93	Austria	8.40	0
K00.K93	Belgium	9.17	0
K00.K93	Czech Republic	9.24	1
K00.K93	France	44.86	1
K00.K93	Germany	214.64	3
K00.K93	Hungary	15.54	0
K00.K93	Netherlands	9.44	0
K00.K93	Spain	7.31	1

For peer review only

K00.K93	Switzerland	15.48	0
K00.K93	United Kingdom	46.18	1
K00.K93	Austria	24.31	0
K00.K93	Belgium	59.63	0
K00.K93	Croatia	9.83	0
K00.K93	Czech Republic	52.34	2
K00.K93	Denmark	40.48	2
K00.K93	Estonia	6.83	0
K00.K93	Finland	38.56	0
K00.K93	France	240.13	1
K00.K93	Germany	284.25	1
K00.K93	Hungary	65.51	0
K00.K93	Latvia	11.87	0
K00.K93	Lithuania	20.36	1
K00.K93	Netherlands	78.07	0
K00.K93	Norway	24.89	0
K00.K93	Poland	63.00	1
K00.K93	Romania	111.34	3
K00.K93	Slovenia	11.77	2
K00.K93	Spain	129.83	0
K00.K93	Sweden	56.25	0
K00.K93	Switzerland	31.95	0
K00.K93	United Kingdom	620.20	0
K00.K93	Austria	106.96	1
K00.K93	Belgium	213.70	3
K00.K93	Croatia	154.09	0
K00.K93	Czech Republic	349.81	1
K00.K93	Denmark	244.33	2
K00.K93	Estonia	42.12	1
K00.K93	Finland	125.56	1
K00.K93	France	374.59	1
K00.K93	Germany	1984.61	1
K00.K93	Hungary	478.15	1
K00.K93	Latvia	98.40	2
K00.K93	Lithuania	160.58	0
K00.K93	Netherlands	242.65	2
K00.K93	Norway	108.63	2
K00.K93	Poland	1269.93	1
K00.K93	Romania	340.33	3
K00.K93	Slovenia	62.96	2
K00.K93	Spain	280.86	3
K00.K93	Sweden	188.20	1
K00.K93	Switzerland	94.01	3
K00.K93	United Kingdom	982.61	3
K00.K93	Austria	109.68	2
K00.K93	Belgium	78.18	1
K00.K93	Croatia	114.91	1
K00.K93	Czech Republic	293.37	1
K00.K93	Denmark	170.49	1
K00.K93	Estonia	35.20	0

For peer review only

K00.K93	Finland	92.32	1
K00.K93	France	305.05	2
K00.K93	Germany	1095.36	3
K00.K93	Hungary	458.17	2
K00.K93	Latvia	59.13	0
K00.K93	Lithuania	127.56	1
K00.K93	Netherlands	87.02	2
K00.K93	Norway	72.62	1
K00.K93	Poland	678.38	3
K00.K93	Romania	337.10	2
K00.K93	Slovenia	54.88	1
K00.K93	Spain	238.03	2
K00.K93	Sweden	104.93	1
K00.K93	Switzerland	68.07	1
K00.K93	United Kingdom	2112.32	1
K00.K93	Belgium	26.48	2
K00.K93	Croatia	6.90	1
K00.K93	Czech Republic	36.99	2
K00.K93	Denmark	74.65	1
K00.K93	Finland	7.06	2
K00.K93	France	278.75	2
K00.K93	Germany	115.60	1
K00.K93	Hungary	7.91	1
K00.K93	Netherlands	14.50	3
K00.K93	Norway	52.07	2
K00.K93	Poland	411.42	2
K00.K93	Romania	52.25	0
K00.K93	Spain	105.05	2
K00.K93	Sweden	76.02	0
K00.K93	Switzerland	24.76	1
K00.K93	United Kingdom	507.37	2
K00.K93	Belgium	6.70	0
K00.K93	Czech Republic	11.96	0
K00.K93	Denmark	11.07	0
K00.K93	Germany	66.17	2
K00.K93	Netherlands	28.29	2
K00.K93	Poland	125.79	3
K00.K93	Romania	9.09	1
K00.K93	United Kingdom	18.45	1
K00.K93	Austria	21.96	0
K00.K93	Belgium	14.50	0
K00.K93	Croatia	22.19	1
K00.K93	Czech Republic	39.93	2
K00.K93	Denmark	23.71	0
K00.K93	Finland	22.13	0
K00.K93	France	39.76	1
K00.K93	Germany	324.09	1
K00.K93	Hungary	51.18	1
K00.K93	Latvia	11.77	1
K00.K93	Lithuania	12.10	0

For peer review only

K00.K93	Netherlands	26.04	2
K00.K93	Norway	11.72	2
K00.K93	Poland	168.50	0
K00.K93	Romania	133.12	1
K00.K93	Slovenia	10.64	0
K00.K93	Spain	50.74	0
K00.K93	Sweden	17.02	1
K00.K93	Switzerland	18.05	0
K00.K93	United Kingdom	243.75	0
K00.K93	Czech Republic	7.39	1
K00.K93	Austria	14.35	0
K00.K93	Belgium	41.10	0
K00.K93	Czech Republic	12.91	0
K00.K93	Denmark	9.64	1
K00.K93	France	132.49	1
K00.K93	Germany	268.65	2
K00.K93	Hungary	14.62	1
K00.K93	Netherlands	53.15	2
K00.K93	Norway	5.88	0
K00.K93	Poland	35.00	1
K00.K93	Romania	15.40	0
K00.K93	Spain	107.96	1
K00.K93	Sweden	22.43	0
K00.K93	Switzerland	17.09	2
K00.K93	United Kingdom	222.23	3
K00.K93	Austria	12.16	1
K00.K93	Belgium	12.99	0
K00.K93	Croatia	12.79	0
K00.K93	Czech Republic	22.88	1
K00.K93	Denmark	23.57	3
K00.K93	Finland	10.80	1
K00.K93	France	89.79	1
K00.K93	Germany	146.58	2
K00.K93	Hungary	40.04	0
K00.K93	Lithuania	10.03	0
K00.K93	Netherlands	27.42	1
K00.K93	Norway	7.27	0
K00.K93	Poland	75.83	1
K00.K93	Romania	32.74	1
K00.K93	Spain	62.01	1
K00.K93	Sweden	12.50	1
K00.K93	Switzerland	14.76	2
K00.K93	United Kingdom	134.78	2
K00.K93	France	13.63	1
K00.K93	Germany	9.20	1
K00.K93	Netherlands	5.73	0
K00.K93	Spain	11.29	0
K00.K93	United Kingdom	18.20	0
K00.K93	Spain	7.85	0
K00.K93	United Kingdom	9.27	1

For peer review only

K00.K93	Austria	16.95	1
K00.K93	Belgium	30.92	1
K00.K93	Croatia	32.89	0
K00.K93	Czech Republic	28.68	0
K00.K93	Denmark	22.43	1
K00.K93	Finland	20.72	1
K00.K93	France	166.96	1
K00.K93	Germany	160.62	0
K00.K93	Hungary	60.14	0
K00.K93	Latvia	6.36	2
K00.K93	Lithuania	9.90	0
K00.K93	Netherlands	56.64	0
K00.K93	Norway	9.63	0
K00.K93	Poland	152.40	1
K00.K93	Romania	58.88	1
K00.K93	Slovenia	11.05	0
K00.K93	Spain	152.19	2
K00.K93	Sweden	27.23	1
K00.K93	Switzerland	20.21	0
K00.K93	United Kingdom	239.93	3
K00.K93	Croatia	9.28	1
K00.K93	Czech Republic	7.10	0
K00.K93	Denmark	9.82	0
K00.K93	Finland	7.35	1
K00.K93	France	35.45	1
K00.K93	Germany	42.50	1
K00.K93	Hungary	15.38	1
K00.K93	Lithuania	10.87	1
K00.K93	Netherlands	10.50	0
K00.K93	Poland	129.40	1
K00.K93	Romania	10.56	0
K00.K93	Spain	76.86	2
K00.K93	Sweden	5.18	0
K00.K93	Switzerland	5.49	0
K00.K93	United Kingdom	158.67	1
K00.K93	Austria	6.47	0
K00.K93	Belgium	8.44	0
K00.K93	Croatia	7.70	0
K00.K93	Czech Republic	14.15	0
K00.K93	Finland	9.55	0
K00.K93	France	68.22	0
K00.K93	Germany	57.11	2
K00.K93	Hungary	28.50	0
K00.K93	Lithuania	6.59	0
K00.K93	Netherlands	16.00	0
K00.K93	Poland	50.18	1
K00.K93	Romania	16.76	0
K00.K93	Slovenia	5.30	0
K00.K93	Spain	56.96	0
K00.K93	Sweden	7.47	0

For peer review only

K00.K93	Switzerland	7.58	1
K00.K93	United Kingdom	112.35	2
K00.K93	Austria	12.24	0
K00.K93	Belgium	12.50	0
K00.K93	Croatia	17.92	0
K00.K93	Czech Republic	34.42	1
K00.K93	Denmark	13.01	0
K00.K93	Finland	12.73	0
K00.K93	France	149.82	2
K00.K93	Germany	132.18	2
K00.K93	Hungary	86.31	2
K00.K93	Latvia	7.28	0
K00.K93	Lithuania	10.12	1
K00.K93	Netherlands	29.78	0
K00.K93	Norway	6.01	1
K00.K93	Poland	129.64	0
K00.K93	Romania	21.40	0
K00.K93	Spain	81.47	0
K00.K93	Sweden	15.66	0
K00.K93	Switzerland	18.17	0
K00.K93	United Kingdom	147.09	1
K00.K93	Austria	12.55	0
K00.K93	Belgium	22.95	2
K00.K93	Czech Republic	9.34	3
K00.K93	Denmark	12.97	0
K00.K93	Finland	9.81	0
K00.K93	France	190.70	1
K00.K93	Germany	111.10	2
K00.K93	Hungary	15.47	0
K00.K93	Netherlands	40.85	0
K00.K93	Norway	12.66	0
K00.K93	Poland	12.70	0
K00.K93	Romania	8.64	1
K00.K93	Spain	99.81	0
K00.K93	Sweden	19.12	0
K00.K93	Switzerland	23.39	0
K00.K93	United Kingdom	225.90	3
K00.K93	Czech Republic	8.07	0
K00.K93	Denmark	6.63	1
K00.K93	Germany	15.68	1
K00.K93	Netherlands	6.01	0
K00.K93	Poland	41.39	1
K00.K93	Romania	9.52	0
K00.K93	Spain	8.56	2
K00.K93	United Kingdom	21.96	0
K00.K93	Austria	20.23	1
K00.K93	Belgium	18.91	1
K00.K93	Denmark	9.54	0
K00.K93	France	220.67	2
K00.K93	Germany	67.70	1

For peer review only

K00.K93	Hungary	20.19	2
K00.K93	Netherlands	21.25	0
K00.K93	Norway	8.69	0
K00.K93	Poland	24.80	1
K00.K93	Romania	7.59	0
K00.K93	Spain	143.05	1
K00.K93	Sweden	8.25	1
K00.K93	Switzerland	9.79	0
K00.K93	United Kingdom	203.26	3
K00.K93	Austria	19.92	0
K00.K93	Belgium	28.25	1
K00.K93	Croatia	12.65	2
K00.K93	Czech Republic	28.82	0
K00.K93	Denmark	22.54	1
K00.K93	Finland	6.72	0
K00.K93	France	98.48	0
K00.K93	Germany	217.01	2
K00.K93	Hungary	28.03	0
K00.K93	Netherlands	46.39	1
K00.K93	Norway	11.26	1
K00.K93	Poland	54.06	0
K00.K93	Romania	23.12	1
K00.K93	Spain	76.16	1
K00.K93	Sweden	17.67	0
K00.K93	Switzerland	19.61	0
K00.K93	United Kingdom	251.99	2
K00.K93	Austria	18.51	2
K00.K93	Belgium	13.21	1
K00.K93	Croatia	13.53	2
K00.K93	Czech Republic	38.38	0
K00.K93	Denmark	28.29	2
K00.K93	Estonia	6.31	0
K00.K93	Finland	12.43	1
K00.K93	France	73.16	1
K00.K93	Germany	186.08	2
K00.K93	Hungary	41.02	0
K00.K93	Latvia	9.34	0
K00.K93	Lithuania	9.69	2
K00.K93	Netherlands	33.05	0
K00.K93	Norway	13.95	0
K00.K93	Poland	93.68	0
K00.K93	Romania	47.29	3
K00.K93	Slovenia	5.80	1
K00.K93	Spain	71.20	2
K00.K93	Sweden	16.51	0
K00.K93	Switzerland	14.52	2
K00.K93	United Kingdom	201.18	2
K00.K93	Austria	78.58	2
K00.K93	Belgium	215.58	2
K00.K93	Czech Republic	52.33	2

For peer review only

K00.K93	Denmark	17.06	0
K00.K93	Finland	13.54	1
K00.K93	France	626.59	3
K00.K93	Germany	1359.34	2
K00.K93	Hungary	59.00	0
K00.K93	Netherlands	389.86	2
K00.K93	Norway	8.17	0
K00.K93	Poland	153.57	1
K00.K93	Slovenia	10.32	0
K00.K93	Spain	134.85	3
K00.K93	Sweden	96.89	1
K00.K93	Switzerland	81.20	1
K00.K93	United Kingdom	666.11	2
K00.K93	Austria	327.13	1
K00.K93	Belgium	686.72	1
K00.K93	Croatia	261.51	1
K00.K93	Czech Republic	331.42	1
K00.K93	Denmark	181.23	1
K00.K93	Estonia	140.13	1
K00.K93	Finland	223.86	1
K00.K93	France	2591.91	2
K00.K93	Germany	4651.68	3
K00.K93	Hungary	656.32	2
K00.K93	Latvia	235.96	1
K00.K93	Lithuania	304.18	2
K00.K93	Netherlands	572.79	2
K00.K93	Norway	114.27	2
K00.K93	Poland	1897.72	3
K00.K93	Romania	1057.79	3
K00.K93	Slovenia	129.92	1
K00.K93	Spain	3199.74	2
K00.K93	Sweden	245.06	2
K00.K93	Switzerland	206.98	0
K00.K93	United Kingdom	3144.00	2
K00.K93	Austria	228.74	1
K00.K93	Belgium	457.77	1
K00.K93	Croatia	158.32	0
K00.K93	Czech Republic	332.59	3
K00.K93	Denmark	256.27	2
K00.K93	Estonia	20.58	0
K00.K93	Finland	201.86	3
K00.K93	France	3144.07	1
K00.K93	Germany	3975.83	3
K00.K93	Hungary	277.67	1
K00.K93	Latvia	27.58	0
K00.K93	Lithuania	55.28	1
K00.K93	Netherlands	828.02	1
K00.K93	Norway	157.34	1
K00.K93	Poland	1211.24	1
K00.K93	Romania	741.37	0

For peer review only

K00.K93	Slovenia	83.58	0
K00.K93	Spain	1895.00	3
K00.K93	Sweden	253.59	0
K00.K93	Switzerland	272.57	1
K00.K93	United Kingdom	2317.83	3
K00.K93	Austria	163.25	1
K00.K93	Belgium	148.14	1
K00.K93	Croatia	39.76	1
K00.K93	Czech Republic	172.97	0
K00.K93	Denmark	125.30	1
K00.K93	Estonia	11.31	0
K00.K93	Finland	132.57	1
K00.K93	France	435.62	1
K00.K93	Germany	1261.25	2
K00.K93	Hungary	171.16	1
K00.K93	Latvia	12.15	1
K00.K93	Lithuania	27.92	1
K00.K93	Netherlands	354.35	2
K00.K93	Norway	99.05	0
K00.K93	Poland	319.42	1
K00.K93	Romania	19.78	1
K00.K93	Slovenia	24.42	0
K00.K93	Spain	391.13	0
K00.K93	Sweden	114.30	1
K00.K93	Switzerland	256.80	1
K00.K93	United Kingdom	2268.74	3
K00.K93	Germany	63.56	3
K00.K93	Belgium	15.82	1
K00.K93	Denmark	30.26	0
K00.K93	Finland	7.35	0
K00.K93	France	117.35	1
K00.K93	Germany	153.14	3
K00.K93	Hungary	16.08	1
K00.K93	Netherlands	38.44	1
K00.K93	Norway	10.37	0
K00.K93	Poland	7.98	0
K00.K93	Spain	48.31	2
K00.K93	Sweden	16.67	3
K00.K93	Switzerland	8.27	3
K00.K93	United Kingdom	143.87	1
K00.K93	France	9.18	0
K00.K93	Germany	5.80	0
K00.K93	Belgium	5.44	0
K00.K93	France	18.61	0
K00.K93	Germany	21.57	0
K00.K93	Hungary	11.92	0
K00.K93	Netherlands	8.37	0
K00.K93	Poland	10.15	0
K00.K93	Romania	8.42	0
K00.K93	Spain	16.65	0

For peer review only

K00.K93	United Kingdom	27.08	0
K00.K93	Belgium	25.55	0
K00.K93	Czech Republic	9.39	0
K00.K93	Denmark	24.88	2
K00.K93	France	200.25	1
K00.K93	Germany	70.13	0
K00.K93	Hungary	6.54	0
K00.K93	Netherlands	21.53	2
K00.K93	Norway	13.87	0
K00.K93	Poland	16.54	2
K00.K93	Spain	174.56	2
K00.K93	Sweden	13.53	0
K00.K93	Switzerland	10.03	0
K00.K93	United Kingdom	96.41	2
K00.K93	Austria	81.95	1
K00.K93	Belgium	177.09	2
K00.K93	Croatia	31.06	3
K00.K93	Czech Republic	61.10	0
K00.K93	Denmark	67.44	1
K00.K93	Estonia	5.38	0
K00.K93	Finland	42.28	1
K00.K93	France	564.99	1
K00.K93	Germany	1079.61	2
K00.K93	Hungary	75.04	3
K00.K93	Latvia	7.49	0
K00.K93	Lithuania	19.12	1
K00.K93	Netherlands	345.46	3
K00.K93	Norway	44.28	0
K00.K93	Poland	256.81	3
K00.K93	Romania	20.22	0
K00.K93	Slovenia	28.92	1
K00.K93	Spain	604.23	2
K00.K93	Sweden	78.12	0
K00.K93	Switzerland	93.40	0
K00.K93	United Kingdom	1269.93	3
K00.K93	Austria	14.30	0
K00.K93	Belgium	91.63	2
K00.K93	Croatia	60.06	1
K00.K93	Czech Republic	225.66	3
K00.K93	Denmark	45.15	1
K00.K93	Finland	16.95	0
K00.K93	France	600.82	2
K00.K93	Germany	658.90	2
K00.K93	Hungary	63.54	1
K00.K93	Latvia	6.69	0
K00.K93	Lithuania	19.47	3
K00.K93	Netherlands	106.86	1
K00.K93	Norway	22.43	0
K00.K93	Poland	901.84	3
K00.K93	Romania	572.24	0

For peer review only

K00.K93	Slovenia	11.09	2
K00.K93	Spain	635.15	3
K00.K93	Sweden	48.58	1
K00.K93	Switzerland	36.63	1
K00.K93	United Kingdom	579.41	3
K00.K93	Austria	7.40	1
K00.K93	Belgium	25.70	0
K00.K93	Czech Republic	16.15	0
K00.K93	France	91.46	1
K00.K93	Germany	63.81	1
K00.K93	Hungary	13.95	0
K00.K93	Netherlands	5.37	0
K00.K93	Poland	8.62	1
K00.K93	Romania	7.78	1
K00.K93	Spain	89.65	1
K00.K93	Switzerland	9.24	0
K00.K93	United Kingdom	157.44	1
K00.K93	Austria	706.86	3
K00.K93	Belgium	733.84	1
K00.K93	Croatia	477.28	3
K00.K93	Czech Republic	1176.00	2
K00.K93	Denmark	749.23	2
K00.K93	Estonia	193.43	0
K00.K93	Finland	947.65	2
K00.K93	France	5686.36	2
K00.K93	Germany	8985.32	2
K00.K93	Hungary	3839.75	1
K00.K93	Latvia	110.65	1
K00.K93	Lithuania	517.86	2
K00.K93	Netherlands	479.13	1
K00.K93	Norway	167.78	1
K00.K93	Poland	2599.20	1
K00.K93	Romania	1037.17	2
K00.K93	Slovenia	495.43	3
K00.K93	Spain	1402.28	2
K00.K93	Sweden	375.02	0
K00.K93	Switzerland	581.51	1
K00.K93	United Kingdom	5661.12	3
K00.K93	Austria	7.48	3
K00.K93	Belgium	16.16	0
K00.K93	Croatia	8.08	1
K00.K93	Czech Republic	39.19	0
K00.K93	Denmark	7.60	1
K00.K93	France	72.52	3
K00.K93	Germany	125.97	1
K00.K93	Hungary	92.10	2
K00.K93	Latvia	35.78	0
K00.K93	Lithuania	80.95	2
K00.K93	Poland	155.45	2
K00.K93	Romania	289.66	2

For peer review only

K00.K93	Spain	24.12	0
K00.K93	Switzerland	9.43	0
K00.K93	United Kingdom	6.98	2
K00.K93	Austria	9.48	2
K00.K93	Belgium	104.50	0
K00.K93	Croatia	14.68	0
K00.K93	Czech Republic	120.61	3
K00.K93	Denmark	52.41	0
K00.K93	Finland	12.12	0
K00.K93	France	547.37	1
K00.K93	Germany	389.23	0
K00.K93	Hungary	47.04	1
K00.K93	Lithuania	29.75	2
K00.K93	Netherlands	112.94	2
K00.K93	Norway	38.18	0
K00.K93	Poland	398.11	1
K00.K93	Romania	130.48	2
K00.K93	Spain	568.06	1
K00.K93	Sweden	73.19	0
K00.K93	Switzerland	22.70	1
K00.K93	United Kingdom	304.84	2
K00.K93	Czech Republic	31.28	1
K00.K93	Denmark	6.78	0
K00.K93	France	25.90	1
K00.K93	Germany	41.64	2
K00.K93	Hungary	49.41	1
K00.K93	Lithuania	9.69	0
K00.K93	Netherlands	13.54	3
K00.K93	Poland	24.34	1
K00.K93	Romania	566.78	1
K00.K93	Spain	29.33	1
K00.K93	United Kingdom	62.08	1
K00.K93	Austria	888.21	3
K00.K93	Belgium	529.39	2
K00.K93	Croatia	771.53	1
K00.K93	Czech Republic	787.57	1
K00.K93	Denmark	81.66	1
K00.K93	Estonia	108.09	1
K00.K93	Finland	116.31	0
K00.K93	France	2803.90	2
K00.K93	Germany	5783.95	2
K00.K93	Hungary	983.45	3
K00.K93	Latvia	302.37	1
K00.K93	Lithuania	470.57	2
K00.K93	Netherlands	400.73	0
K00.K93	Norway	61.12	0
K00.K93	Poland	4112.33	2
K00.K93	Romania	10266.86	1
K00.K93	Slovenia	139.30	2
K00.K93	Spain	3938.65	2

For peer review only

K00.K93	Sweden	268.91	2
K00.K93	Switzerland	130.52	0
K00.K93	United Kingdom	2122.36	3
K00.K93	Austria	21.10	0
K00.K93	Belgium	32.02	0
K00.K93	Croatia	6.07	0
K00.K93	Czech Republic	29.31	0
K00.K93	Denmark	9.04	0
K00.K93	Finland	11.15	0
K00.K93	France	145.41	2
K00.K93	Germany	199.70	1
K00.K93	Hungary	31.27	0
K00.K93	Lithuania	6.18	1
K00.K93	Netherlands	25.45	2
K00.K93	Norway	11.30	2
K00.K93	Poland	46.60	0
K00.K93	Romania	23.49	0
K00.K93	Spain	108.52	0
K00.K93	Sweden	19.82	1
K00.K93	Switzerland	19.52	1
K00.K93	United Kingdom	206.00	3
K00.K93	Austria	100.71	2
K00.K93	Belgium	55.32	1
K00.K93	Croatia	22.37	1
K00.K93	Czech Republic	134.42	2
K00.K93	Denmark	19.48	3
K00.K93	Estonia	7.57	1
K00.K93	Finland	7.02	0
K00.K93	France	253.66	0
K00.K93	Germany	386.67	0
K00.K93	Hungary	135.72	2
K00.K93	Latvia	12.98	0
K00.K93	Lithuania	21.38	1
K00.K93	Netherlands	76.22	0
K00.K93	Norway	12.42	0
K00.K93	Poland	129.85	3
K00.K93	Romania	176.43	3
K00.K93	Slovenia	8.70	1
K00.K93	Spain	918.62	1
K00.K93	Sweden	29.91	1
K00.K93	Switzerland	27.01	0
K00.K93	United Kingdom	953.28	2
K00.K93	Austria	73.66	0
K00.K93	Belgium	76.44	2
K00.K93	Croatia	58.62	1
K00.K93	Czech Republic	203.27	2
K00.K93	Denmark	70.36	1
K00.K93	Estonia	14.04	0
K00.K93	Finland	118.70	1
K00.K93	France	388.85	2

For peer review only

K00.K93	Germany	728.12	1
K00.K93	Hungary	308.78	3
K00.K93	Latvia	31.27	0
K00.K93	Lithuania	73.65	2
K00.K93	Netherlands	123.87	1
K00.K93	Norway	43.82	0
K00.K93	Poland	530.47	1
K00.K93	Romania	106.62	0
K00.K93	Slovenia	38.85	0
K00.K93	Spain	521.88	0
K00.K93	Sweden	75.95	0
K00.K93	Switzerland	50.83	0
K00.K93	United Kingdom	804.28	3
K00.K93	Austria	53.73	0
K00.K93	Belgium	145.39	1
K00.K93	Croatia	46.29	0
K00.K93	Czech Republic	76.89	1
K00.K93	Denmark	54.31	1
K00.K93	Estonia	6.30	0
K00.K93	Finland	44.63	1
K00.K93	France	580.13	1
K00.K93	Germany	649.13	1
K00.K93	Hungary	83.99	2
K00.K93	Latvia	14.04	0
K00.K93	Lithuania	14.09	0
K00.K93	Netherlands	164.67	0
K00.K93	Norway	54.39	2
K00.K93	Poland	286.87	1
K00.K93	Romania	125.11	1
K00.K93	Slovenia	31.06	0
K00.K93	Spain	1116.36	0
K00.K93	Sweden	74.09	0
K00.K93	Switzerland	63.12	2
K00.K93	United Kingdom	475.04	3
K00.K93	Austria	11.76	0
K00.K93	Belgium	24.49	0
K00.K93	Croatia	12.58	2
K00.K93	Czech Republic	19.41	0
K00.K93	Denmark	6.71	1
K00.K93	France	170.42	1
K00.K93	Germany	152.23	1
K00.K93	Hungary	15.52	1
K00.K93	Netherlands	40.46	0
K00.K93	Norway	8.44	1
K00.K93	Poland	40.31	3
K00.K93	Romania	7.88	0
K00.K93	Spain	94.83	3
K00.K93	Sweden	5.85	0
K00.K93	Switzerland	5.68	1
K00.K93	United Kingdom	128.72	1

For peer review only

K00.K93	Austria	30.56	0
K00.K93	Belgium	86.89	0
K00.K93	Croatia	12.35	0
K00.K93	Czech Republic	79.92	1
K00.K93	Denmark	41.09	0
K00.K93	Finland	25.30	0
K00.K93	France	487.28	0
K00.K93	Germany	424.88	1
K00.K93	Hungary	49.63	0
K00.K93	Lithuania	8.40	2
K00.K93	Netherlands	115.28	1
K00.K93	Norway	33.95	0
K00.K93	Poland	131.02	3
K00.K93	Romania	22.57	1
K00.K93	Slovenia	11.02	0
K00.K93	Spain	366.98	1
K00.K93	Sweden	65.45	0
K00.K93	Switzerland	44.66	1
K00.K93	United Kingdom	764.39	2
K00.K93	Austria	69.67	2
K00.K93	Belgium	141.25	0
K00.K93	Croatia	150.25	1
K00.K93	Czech Republic	339.96	1
K00.K93	Denmark	71.60	2
K00.K93	Estonia	39.36	1
K00.K93	Finland	97.08	3
K00.K93	France	804.69	2
K00.K93	Germany	1215.96	1
K00.K93	Hungary	339.53	1
K00.K93	Latvia	88.11	3
K00.K93	Lithuania	167.36	1
K00.K93	Netherlands	166.07	0
K00.K93	Norway	47.42	2
K00.K93	Poland	1873.41	2
K00.K93	Romania	1023.90	0
K00.K93	Slovenia	50.84	1
K00.K93	Spain	1314.02	1
K00.K93	Sweden	125.35	0
K00.K93	Switzerland	72.60	0
K00.K93	United Kingdom	1225.06	1
K00.K93	Austria	60.43	0
K00.K93	Belgium	32.97	1
K00.K93	Croatia	19.84	0
K00.K93	Czech Republic	85.60	1
K00.K93	Denmark	80.88	2
K00.K93	Estonia	17.37	1
K00.K93	Finland	77.20	1
K00.K93	France	222.26	1
K00.K93	Germany	491.44	1
K00.K93	Hungary	124.28	1

For peer review only

K00.K93	Latvia	35.60	3
K00.K93	Lithuania	37.99	2
K00.K93	Netherlands	59.91	3
K00.K93	Norway	19.31	2
K00.K93	Poland	249.36	2
K00.K93	Romania	115.27	0
K00.K93	Slovenia	10.54	0
K00.K93	Spain	101.32	0
K00.K93	Sweden	20.32	0
K00.K93	Switzerland	23.77	1
K00.K93	United Kingdom	204.45	2
K00.K93	France	28.15	1
K00.K93	Germany	21.08	0
K00.K93	Hungary	5.84	0
K00.K93	Netherlands	8.51	0
K00.K93	Poland	9.70	1
K00.K93	Spain	22.07	0
K00.K93	Switzerland	5.73	0
K00.K93	United Kingdom	48.18	0
K00.K93	Austria	81.01	0
K00.K93	Belgium	616.89	2
K00.K93	Croatia	93.85	1
K00.K93	Czech Republic	183.26	3
K00.K93	Denmark	246.99	1
K00.K93	Estonia	5.56	0
K00.K93	Finland	57.23	1
K00.K93	France	2482.51	1
K00.K93	Germany	4104.85	3
K00.K93	Hungary	39.36	3
K00.K93	Latvia	12.67	0
K00.K93	Lithuania	22.02	1
K00.K93	Netherlands	1423.87	1
K00.K93	Norway	152.29	0
K00.K93	Poland	1862.57	2
K00.K93	Romania	433.66	0
K00.K93	Slovenia	37.63	2
K00.K93	Spain	2353.02	3
K00.K93	Sweden	327.87	1
K00.K93	Switzerland	307.85	2
K00.K93	United Kingdom	1947.79	1
L00.L99	Austria	5.63	0
L00.L99	Czech Republic	12.75	1
L00.L99	Denmark	8.24	0
L00.L99	France	14.74	2
L00.L99	Germany	65.64	2
L00.L99	Hungary	19.76	2
L00.L99	Lithuania	15.38	0
L00.L99	Netherlands	20.59	2
L00.L99	Poland	13.44	1
L00.L99	Romania	8.21	0

For peer review only

L00.L99	Spain	34.64	0
L00.L99	Sweden	9.09	0
L00.L99	Switzerland	6.89	0
L00.L99	United Kingdom	72.87	1
L00.L99	Austria	5.08	0
L00.L99	Belgium	7.65	1
L00.L99	Czech Republic	23.38	3
L00.L99	Estonia	6.92	0
L00.L99	Germany	68.27	1
L00.L99	Latvia	14.64	0
L00.L99	Lithuania	12.85	2
L00.L99	Netherlands	21.48	1
L00.L99	Poland	8.08	1
L00.L99	Romania	15.25	0
L00.L99	Spain	125.48	1
L00.L99	Switzerland	8.28	0
L00.L99	United Kingdom	984.06	3
L00.L99	Germany	8.80	1
L00.L99	Hungary	7.93	0
L00.L99	Netherlands	37.56	2
L00.L99	Norway	17.31	2
L00.L99	Poland	75.19	3
L00.L99	Romania	8.47	0
L00.L99	Spain	140.60	2
L00.L99	Sweden	18.76	0
L00.L99	Switzerland	8.02	3
L00.L99	United Kingdom	59.80	0
L00.L99	France	10.34	1
L00.L99	Germany	8.37	3
L00.L99	Poland	5.70	0
L00.L99	Spain	12.68	0
L00.L99	United Kingdom	8.16	0
L00.L99	Belgium	8.55	0
L00.L99	France	119.57	1
L00.L99	Germany	26.60	2
L00.L99	Netherlands	17.42	0
L00.L99	Spain	14.52	2
L00.L99	Sweden	6.89	1
L00.L99	United Kingdom	47.72	0
L00.L99	France	19.94	1
L00.L99	United Kingdom	10.57	0
L00.L99	Germany	10.63	1
L00.L99	Netherlands	5.25	0
L00.L99	Spain	8.94	0
L00.L99	United Kingdom	20.58	1
L00.L99	France	12.52	1
L00.L99	Germany	23.12	0
L00.L99	Spain	11.09	0
L00.L99	United Kingdom	19.33	0
L00.L99	France	12.37	0

For peer review only

L00.L99	United Kingdom	7.90	1
L00.L99	France	6.99	0
L00.L99	Germany	6.39	1
L00.L99	United Kingdom	5.80	0
L00.L99	Austria	23.38	0
L00.L99	Belgium	343.39	3
L00.L99	Croatia	10.82	3
L00.L99	Czech Republic	66.97	1
L00.L99	Denmark	26.53	1
L00.L99	France	1519.18	1
L00.L99	Germany	570.29	1
L00.L99	Latvia	17.99	1
L00.L99	Lithuania	8.39	0
L00.L99	Netherlands	326.53	1
L00.L99	Norway	29.80	1
L00.L99	Romania	40.81	1
L00.L99	Slovenia	30.23	0
L00.L99	Spain	745.67	2
L00.L99	Sweden	24.77	2
L00.L99	Switzerland	36.68	1
L00.L99	United Kingdom	267.64	3
L00.L99	Austria	6.07	1
L00.L99	France	19.80	1
L00.L99	Germany	32.41	0
L00.L99	Romania	11.38	1
L00.L99	Spain	16.22	1
L00.L99	United Kingdom	5.98	1
L00.L99	Austria	12.96	1
L00.L99	Belgium	15.78	1
L00.L99	Denmark	16.50	2
L00.L99	France	118.42	3
L00.L99	Germany	77.57	2
L00.L99	Netherlands	52.63	0
L00.L99	Norway	19.13	0
L00.L99	Spain	44.57	1
L00.L99	Sweden	52.94	2
L00.L99	Switzerland	15.22	0
L00.L99	United Kingdom	514.28	3
L00.L99	Denmark	5.73	1
L00.L99	France	10.29	2
L00.L99	Germany	23.54	2
L00.L99	Netherlands	10.22	0
L00.L99	Norway	13.87	1
L00.L99	Spain	155.23	3
L00.L99	Sweden	31.06	2
L00.L99	Switzerland	5.11	0
L00.L99	United Kingdom	116.42	1
M00.M99	Austria	8.06	1
M00.M99	Belgium	15.21	1
M00.M99	Czech Republic	9.48	1

For peer review only

M00.M99	Denmark	6.28	0
M00.M99	Finland	8.27	1
M00.M99	France	100.84	1
M00.M99	Germany	94.29	2
M00.M99	Hungary	8.53	1
M00.M99	Netherlands	39.11	1
M00.M99	Norway	6.83	1
M00.M99	Poland	11.60	2
M00.M99	Spain	48.36	1
M00.M99	Sweden	17.81	0
M00.M99	Switzerland	24.47	1
M00.M99	United Kingdom	195.03	0
M00.M99	Belgium	8.87	2
M00.M99	Czech Republic	11.71	2
M00.M99	Denmark	24.75	2
M00.M99	Estonia	8.27	0
M00.M99	Finland	85.52	1
M00.M99	France	40.82	1
M00.M99	Germany	24.54	0
M00.M99	Hungary	10.63	2
M00.M99	Latvia	9.14	1
M00.M99	Lithuania	18.02	0
M00.M99	Netherlands	10.91	0
M00.M99	Poland	156.21	3
M00.M99	Slovenia	10.07	3
M00.M99	Spain	32.63	0
M00.M99	Sweden	18.54	2
M00.M99	Switzerland	5.82	0
M00.M99	United Kingdom	95.76	2
M00.M99	Austria	54.22	2
M00.M99	Belgium	62.41	1
M00.M99	Croatia	42.10	1
M00.M99	Czech Republic	8.41	2
M00.M99	Denmark	74.80	2
M00.M99	Estonia	9.46	1
M00.M99	Finland	39.67	1
M00.M99	France	426.37	2
M00.M99	Germany	382.12	2
M00.M99	Hungary	55.79	0
M00.M99	Latvia	15.67	0
M00.M99	Lithuania	22.24	1
M00.M99	Netherlands	166.55	2
M00.M99	Norway	61.81	1
M00.M99	Poland	235.66	1
M00.M99	Slovenia	20.87	0
M00.M99	Spain	245.58	2
M00.M99	Sweden	128.50	1
M00.M99	Switzerland	69.96	2
M00.M99	United Kingdom	987.01	2
M00.M99	Denmark	5.53	2

For peer review only

M00.M99	France	18.99	2
M00.M99	Germany	13.94	2
M00.M99	Latvia	5.16	0
M00.M99	Netherlands	11.52	2
M00.M99	Switzerland	5.28	0
M00.M99	United Kingdom	23.90	0
M00.M99	France	5.63	0
M00.M99	Austria	7.11	1
M00.M99	Belgium	11.82	1
M00.M99	Denmark	5.66	0
M00.M99	France	103.92	1
M00.M99	Germany	51.66	1
M00.M99	Hungary	26.64	0
M00.M99	Netherlands	12.85	0
M00.M99	Norway	6.57	3
M00.M99	Spain	16.52	1
M00.M99	Sweden	5.04	3
M00.M99	Switzerland	13.27	0
M00.M99	United Kingdom	88.03	2
M00.M99	Austria	8.70	1
M00.M99	Belgium	20.63	0
M00.M99	Denmark	5.07	0
M00.M99	Finland	9.90	0
M00.M99	France	63.23	1
M00.M99	Germany	90.61	1
M00.M99	Hungary	11.72	2
M00.M99	Netherlands	24.26	0
M00.M99	Poland	5.42	0
M00.M99	Spain	40.48	0
M00.M99	Switzerland	72.97	0
M00.M99	United Kingdom	28.42	1
M00.M99	Belgium	6.77	1
M00.M99	Denmark	15.09	0
M00.M99	Finland	12.61	1
M00.M99	France	53.96	1
M00.M99	Germany	70.48	0
M00.M99	Hungary	28.85	0
M00.M99	Netherlands	78.72	1
M00.M99	Norway	22.95	2
M00.M99	Spain	10.71	0
M00.M99	Sweden	12.52	0
M00.M99	Switzerland	40.31	0
M00.M99	United Kingdom	27.62	1
M00.M99	Belgium	7.65	1
M00.M99	Denmark	7.72	0
M00.M99	Finland	8.95	1
M00.M99	France	22.62	3
M00.M99	Germany	38.85	1
M00.M99	Hungary	7.27	0
M00.M99	Netherlands	24.04	1

For peer review only

M00.M99	Spain	8.37	1
M00.M99	Sweden	6.75	0
M00.M99	Switzerland	18.52	1
M00.M99	United Kingdom	17.81	1
M00.M99	Belgium	14.19	0
M00.M99	Denmark	5.06	1
M00.M99	France	95.29	1
M00.M99	Germany	20.87	0
M00.M99	Netherlands	57.78	2
M00.M99	Spain	51.26	1
M00.M99	Switzerland	11.18	1
M00.M99	United Kingdom	266.48	1
M00.M99	Netherlands	5.60	0
M00.M99	United Kingdom	6.37	1
M00.M99	Belgium	8.54	2
M00.M99	Germany	66.56	0
M00.M99	Netherlands	150.30	1
M00.M99	Spain	103.86	3
M00.M99	Sweden	6.17	0
M00.M99	United Kingdom	391.58	3
M00.M99	Belgium	8.39	1
M00.M99	France	51.00	2
M00.M99	Germany	22.96	2
M00.M99	Poland	8.92	0
M00.M99	Spain	21.24	1
M00.M99	United Kingdom	37.34	2
M00.M99	Austria	20.71	2
M00.M99	Belgium	33.73	0
M00.M99	Czech Republic	11.03	1
M00.M99	Denmark	30.46	0
M00.M99	Finland	19.82	0
M00.M99	France	316.27	2
M00.M99	Germany	178.19	1
M00.M99	Hungary	19.36	2
M00.M99	Lithuania	5.41	0
M00.M99	Netherlands	67.31	2
M00.M99	Norway	16.68	0
M00.M99	Poland	43.33	1
M00.M99	Romania	5.59	0
M00.M99	Slovenia	6.69	1
M00.M99	Spain	89.61	0
M00.M99	Sweden	34.34	0
M00.M99	Switzerland	24.89	0
M00.M99	United Kingdom	168.88	1
M00.M99	Austria	9.35	1
M00.M99	Belgium	9.44	0
M00.M99	Croatia	13.94	0
M00.M99	Czech Republic	11.38	2
M00.M99	Denmark	11.14	1
M00.M99	Finland	7.84	1

For peer review only

M00.M99	France	42.62	2
M00.M99	Germany	53.88	1
M00.M99	Hungary	29.15	0
M00.M99	Latvia	5.85	0
M00.M99	Lithuania	6.12	0
M00.M99	Netherlands	22.45	0
M00.M99	Norway	13.00	1
M00.M99	Poland	91.37	0
M00.M99	Romania	12.96	1
M00.M99	Slovenia	5.69	0
M00.M99	Spain	79.32	0
M00.M99	Sweden	23.04	1
M00.M99	Switzerland	9.68	0
M00.M99	United Kingdom	90.14	0
M00.M99	Belgium	7.76	0
M00.M99	Finland	5.76	2
M00.M99	France	44.56	0
M00.M99	Germany	25.74	1
M00.M99	Hungary	5.69	0
M00.M99	Netherlands	10.48	0
M00.M99	Poland	10.88	0
M00.M99	Spain	25.74	0
M00.M99	Sweden	5.01	3
M00.M99	Switzerland	5.18	0
M00.M99	United Kingdom	44.44	1
M00.M99	Austria	14.38	2
M00.M99	Belgium	22.90	0
M00.M99	Croatia	6.25	0
M00.M99	Denmark	14.98	1
M00.M99	Finland	9.87	0
M00.M99	France	159.45	2
M00.M99	Germany	126.91	1
M00.M99	Hungary	15.01	0
M00.M99	Netherlands	43.27	2
M00.M99	Norway	9.68	0
M00.M99	Poland	51.40	0
M00.M99	Romania	5.77	0
M00.M99	Slovenia	5.88	3
M00.M99	Spain	92.54	1
M00.M99	Sweden	23.17	0
M00.M99	Switzerland	27.72	0
M00.M99	United Kingdom	193.59	1
M00.M99	Austria	11.09	0
M00.M99	Belgium	29.03	1
M00.M99	Croatia	7.73	0
M00.M99	Denmark	26.63	2
M00.M99	Finland	14.14	0
M00.M99	France	167.10	2
M00.M99	Germany	79.49	3
M00.M99	Hungary	15.07	0

For peer review only

M00.M99	Netherlands	57.65	2
M00.M99	Norway	17.11	1
M00.M99	Poland	20.73	0
M00.M99	Slovenia	6.09	0
M00.M99	Spain	65.52	2
M00.M99	Sweden	22.56	0
M00.M99	Switzerland	30.21	2
M00.M99	United Kingdom	76.82	0
M00.M99	Netherlands	6.69	0
M00.M99	Spain	8.91	0
M00.M99	United Kingdom	43.28	0
M00.M99	Austria	8.52	3
M00.M99	Belgium	13.56	3
M00.M99	France	180.46	1
M00.M99	Germany	51.70	0
M00.M99	Netherlands	21.28	1
M00.M99	Spain	55.28	1
M00.M99	Sweden	12.73	2
M00.M99	Switzerland	6.68	0
M00.M99	United Kingdom	162.54	1
M00.M99	Germany	6.13	1
M00.M99	France	7.04	2
M00.M99	Germany	40.61	1
M00.M99	Hungary	7.20	0
M00.M99	Netherlands	5.84	1
M00.M99	Norway	6.24	0
M00.M99	Spain	12.81	0
M00.M99	United Kingdom	21.06	0
M00.M99	Austria	16.85	3
M00.M99	Belgium	13.54	1
M00.M99	Czech Republic	15.56	2
M00.M99	Denmark	9.33	0
M00.M99	France	98.57	1
M00.M99	Germany	196.19	2
M00.M99	Hungary	8.03	1
M00.M99	Latvia	5.56	2
M00.M99	Netherlands	28.36	1
M00.M99	Spain	31.23	2
M00.M99	Sweden	7.40	1
M00.M99	Switzerland	18.15	0
M00.M99	United Kingdom	82.77	2
M00.M99	France	39.66	3
M00.M99	Germany	17.38	1
M00.M99	Hungary	6.77	0
M00.M99	Spain	11.26	0
M00.M99	Switzerland	10.00	3
M00.M99	United Kingdom	30.83	1
M00.M99	Belgium	15.83	0
M00.M99	Denmark	9.63	1
M00.M99	Finland	6.96	0

For peer review only

M00.M99	France	76.36	2
M00.M99	Germany	41.22	1
M00.M99	Netherlands	29.90	3
M00.M99	Norway	8.63	1
M00.M99	Spain	19.99	2
M00.M99	Sweden	13.62	0
M00.M99	Switzerland	12.47	0
M00.M99	United Kingdom	42.46	0
M00.M99	Germany	8.69	0
M00.M99	Spain	6.07	0
M00.M99	France	13.64	0
M00.M99	Germany	10.96	0
M00.M99	Hungary	6.88	1
M00.M99	United Kingdom	7.08	0
M00.M99	Germany	6.08	1
M00.M99	Netherlands	8.55	1
M00.M99	Switzerland	10.29	0
M00.M99	United Kingdom	5.76	2
M00.M99	Germany	19.03	0
M00.M99	Netherlands	14.11	0
M00.M99	Spain	14.36	1
M00.M99	United Kingdom	67.00	3
M00.M99	Austria	9.18	0
M00.M99	Belgium	27.13	0
M00.M99	Denmark	8.56	2
M00.M99	France	206.98	1
M00.M99	Germany	164.55	1
M00.M99	Hungary	13.17	1
M00.M99	Netherlands	31.78	2
M00.M99	Norway	6.50	0
M00.M99	Spain	161.22	2
M00.M99	Sweden	12.09	0
M00.M99	Switzerland	9.92	0
M00.M99	United Kingdom	106.21	1
M00.M99	Germany	6.83	0
M00.M99	United Kingdom	5.35	0
M00.M99	Belgium	10.35	1
M00.M99	Denmark	8.64	1
M00.M99	France	47.01	1
M00.M99	Germany	96.73	1
M00.M99	Netherlands	8.25	2
M00.M99	Spain	53.36	2
M00.M99	Sweden	8.30	1
M00.M99	Switzerland	13.31	1
M00.M99	United Kingdom	119.04	1
M00.M99	Germany	28.50	2
M00.M99	Netherlands	25.84	2
M00.M99	Spain	31.93	3
M00.M99	Sweden	6.46	1
M00.M99	Switzerland	6.19	0

For peer review only

M00.M99	Austria	32.58	0
M00.M99	Belgium	29.66	1
M00.M99	Croatia	14.85	1
M00.M99	Czech Republic	6.90	1
M00.M99	Denmark	41.41	0
M00.M99	Finland	14.14	0
M00.M99	France	75.59	1
M00.M99	Germany	199.09	1
M00.M99	Hungary	66.93	1
M00.M99	Netherlands	67.51	3
M00.M99	Norway	19.15	2
M00.M99	Poland	10.30	2
M00.M99	Spain	182.53	1
M00.M99	Sweden	24.09	0
M00.M99	Switzerland	35.20	0
M00.M99	United Kingdom	1306.36	1
M00.M99	Austria	33.00	1
M00.M99	Belgium	22.42	3
M00.M99	Denmark	72.41	0
M00.M99	France	87.15	1
M00.M99	Germany	218.42	3
M00.M99	Hungary	30.00	0
M00.M99	Netherlands	67.74	0
M00.M99	Norway	28.32	2
M00.M99	Spain	46.03	0
M00.M99	Sweden	25.26	1
M00.M99	Switzerland	57.78	0
M00.M99	United Kingdom	350.52	2
M00.M99	Belgium	5.40	1
M00.M99	Estonia	15.97	1
M00.M99	France	31.97	1
M00.M99	Germany	12.66	0
M00.M99	Spain	2326.13	3
M00.M99	United Kingdom	63.88	2
M00.M99	Austria	11.12	0
M00.M99	Belgium	32.20	0
M00.M99	Croatia	5.33	0
M00.M99	Czech Republic	13.54	2
M00.M99	Denmark	9.76	1
M00.M99	France	228.67	0
M00.M99	Germany	126.91	1
M00.M99	Hungary	42.41	2
M00.M99	Latvia	8.64	0
M00.M99	Lithuania	10.53	0
M00.M99	Netherlands	40.00	1
M00.M99	Norway	21.83	1
M00.M99	Poland	14.65	2
M00.M99	Spain	70.53	0
M00.M99	Sweden	14.23	1
M00.M99	Switzerland	19.42	3

For peer review only

M00.M99	United Kingdom	240.30	2
M00.M99	France	9.17	0
M00.M99	Germany	13.48	2
M00.M99	Hungary	6.24	2
M00.M99	United Kingdom	5.32	0
M00.M99	Belgium	6.81	1
M00.M99	France	40.12	2
M00.M99	Spain	26.99	1
M00.M99	United Kingdom	22.61	2
M00.M99	Belgium	48.21	0
M00.M99	France	1226.53	1
M00.M99	Germany	12.38	2
M00.M99	Spain	8.75	1
M00.M99	United Kingdom	5.89	0
N00.N99	Germany	9.65	0
N00.N99	Hungary	8.26	0
N00.N99	Poland	12.72	3
N00.N99	Romania	8.12	1
N00.N99	United Kingdom	21.92	3
N00.N99	Poland	6.56	1
N00.N99	France	10.43	2
N00.N99	Germany	6.10	0
N00.N99	United Kingdom	22.18	1
N00.N99	Croatia	6.98	0
N00.N99	Czech Republic	9.30	2
N00.N99	Denmark	16.50	1
N00.N99	Estonia	7.60	1
N00.N99	Finland	16.20	1
N00.N99	France	14.37	0
N00.N99	Germany	35.30	0
N00.N99	Hungary	91.24	0
N00.N99	Latvia	62.72	3
N00.N99	Lithuania	24.95	2
N00.N99	Netherlands	6.93	0
N00.N99	Norway	6.32	0
N00.N99	Poland	70.77	2
N00.N99	Romania	67.26	3
N00.N99	Spain	55.64	1
N00.N99	Sweden	22.80	1
N00.N99	Switzerland	8.06	1
N00.N99	United Kingdom	433.22	1
N00.N99	Belgium	9.16	0
N00.N99	Denmark	6.99	1
N00.N99	France	31.66	2
N00.N99	Germany	25.59	0
N00.N99	Hungary	9.22	0
N00.N99	Netherlands	18.97	1
N00.N99	Poland	23.89	0
N00.N99	Romania	5.29	0
N00.N99	Spain	31.87	1

For peer review only

N00.N99	Sweden	6.60	1
N00.N99	Switzerland	6.00	0
N00.N99	United Kingdom	46.86	0
N00.N99	Austria	10.05	0
N00.N99	Belgium	10.53	1
N00.N99	Denmark	20.35	1
N00.N99	France	36.03	1
N00.N99	Germany	58.39	2
N00.N99	Hungary	14.63	0
N00.N99	Netherlands	11.07	2
N00.N99	Norway	7.74	0
N00.N99	Poland	27.56	1
N00.N99	Romania	18.55	1
N00.N99	Spain	33.83	0
N00.N99	Sweden	6.55	2
N00.N99	Switzerland	12.49	1
N00.N99	United Kingdom	64.20	0
N00.N99	Austria	8.97	2
N00.N99	Belgium	11.48	0
N00.N99	Croatia	51.19	2
N00.N99	Czech Republic	115.67	0
N00.N99	Denmark	14.98	0
N00.N99	Estonia	8.65	2
N00.N99	Finland	170.99	1
N00.N99	France	159.24	1
N00.N99	Germany	69.79	1
N00.N99	Hungary	49.37	2
N00.N99	Lithuania	10.67	3
N00.N99	Netherlands	24.72	3
N00.N99	Poland	82.22	3
N00.N99	Romania	54.67	0
N00.N99	Slovenia	17.80	0
N00.N99	Spain	64.44	1
N00.N99	Sweden	43.70	1
N00.N99	United Kingdom	164.73	1
N00.N99	Austria	6.29	2
N00.N99	Belgium	6.66	0
N00.N99	Croatia	89.33	0
N00.N99	Czech Republic	118.21	1
N00.N99	Denmark	10.82	1
N00.N99	Estonia	68.27	1
N00.N99	Finland	28.06	1
N00.N99	France	29.97	2
N00.N99	Germany	80.29	2
N00.N99	Hungary	112.23	2
N00.N99	Latvia	128.51	3
N00.N99	Lithuania	107.99	0
N00.N99	Netherlands	9.16	1
N00.N99	Poland	441.39	3
N00.N99	Romania	193.14	2

For peer review only

N00.N99	Slovenia	9.63	0
N00.N99	Spain	17.44	0
N00.N99	Sweden	12.10	2
N00.N99	Switzerland	12.65	2
N00.N99	United Kingdom	48.45	1
N00.N99	Austria	38.10	1
N00.N99	Belgium	95.87	2
N00.N99	Croatia	6.73	0
N00.N99	Czech Republic	39.34	2
N00.N99	Denmark	20.72	1
N00.N99	Finland	14.79	2
N00.N99	France	450.01	2
N00.N99	Germany	117.94	2
N00.N99	Hungary	33.69	2
N00.N99	Netherlands	36.98	1
N00.N99	Norway	57.69	1
N00.N99	Poland	17.16	0
N00.N99	Romania	44.46	1
N00.N99	Spain	81.25	1
N00.N99	Sweden	31.18	2
N00.N99	Switzerland	31.10	1
N00.N99	United Kingdom	177.41	1
N00.N99	Austria	17.85	0
N00.N99	Belgium	21.85	2
N00.N99	Croatia	7.21	2
N00.N99	Czech Republic	40.90	0
N00.N99	Denmark	24.05	0
N00.N99	Finland	10.47	0
N00.N99	France	87.96	3
N00.N99	Germany	171.56	2
N00.N99	Hungary	27.08	2
N00.N99	Latvia	8.81	2
N00.N99	Lithuania	9.92	0
N00.N99	Netherlands	28.22	2
N00.N99	Norway	11.36	1
N00.N99	Poland	61.04	0
N00.N99	Romania	37.32	0
N00.N99	Spain	102.69	0
N00.N99	Sweden	23.06	0
N00.N99	Switzerland	12.16	0
N00.N99	United Kingdom	277.19	1
N00.N99	Belgium	8.48	1
N00.N99	Denmark	9.97	1
N00.N99	France	20.03	0
N00.N99	Germany	9.56	0
N00.N99	Switzerland	17.70	3
N00.N99	United Kingdom	5.39	0
N00.N99	Belgium	12.64	0
N00.N99	Czech Republic	16.00	0
N00.N99	Denmark	6.40	0

For peer review only

N00.N99	France	25.36	0
N00.N99	Germany	29.28	0
N00.N99	Hungary	13.78	1
N00.N99	Netherlands	5.85	1
N00.N99	Poland	125.96	0
N00.N99	Romania	11.06	0
N00.N99	Spain	36.79	1
N00.N99	United Kingdom	45.97	2
N00.N99	Belgium	231.69	0
N00.N99	Croatia	51.13	3
N00.N99	Czech Republic	342.11	0
N00.N99	Denmark	30.95	3
N00.N99	Finland	12.44	0
N00.N99	France	890.44	2
N00.N99	Germany	2177.88	1
N00.N99	Hungary	38.84	2
N00.N99	Lithuania	9.64	0
N00.N99	Netherlands	207.21	3
N00.N99	Norway	32.60	0
N00.N99	Poland	826.77	3
N00.N99	Romania	519.41	2
N00.N99	Slovenia	35.00	0
N00.N99	Spain	915.66	1
N00.N99	Sweden	78.49	2
N00.N99	Switzerland	27.07	3
N00.N99	United Kingdom	842.07	2
N00.N99	Austria	693.72	2
N00.N99	Belgium	583.16	1
N00.N99	Croatia	408.60	3
N00.N99	Czech Republic	828.89	1
N00.N99	Denmark	264.02	1
N00.N99	Estonia	21.80	1
N00.N99	Finland	112.99	2
N00.N99	France	2571.52	2
N00.N99	Germany	7012.93	1
N00.N99	Hungary	287.74	1
N00.N99	Latvia	33.61	3
N00.N99	Lithuania	84.86	3
N00.N99	Netherlands	1214.29	1
N00.N99	Norway	112.89	2
N00.N99	Poland	3350.13	3
N00.N99	Romania	1877.25	2
N00.N99	Slovenia	158.91	0
N00.N99	Spain	4043.38	3
N00.N99	Sweden	234.10	0
N00.N99	Switzerland	215.64	3
N00.N99	United Kingdom	2035.36	3
N00.N99	Austria	351.07	1
N00.N99	Belgium	647.59	0
N00.N99	Croatia	71.53	3

For peer review only

N00.N99	Czech Republic	50.84	1
N00.N99	Denmark	146.45	3
N00.N99	Finland	13.62	0
N00.N99	France	2657.44	2
N00.N99	Germany	3958.07	2
N00.N99	Hungary	73.15	1
N00.N99	Lithuania	8.06	1
N00.N99	Netherlands	516.53	3
N00.N99	Norway	326.64	2
N00.N99	Poland	913.57	3
N00.N99	Romania	279.05	1
N00.N99	Slovenia	21.93	2
N00.N99	Spain	2177.77	1
N00.N99	Sweden	281.08	0
N00.N99	Switzerland	153.73	3
N00.N99	United Kingdom	1051.17	3
N00.N99	Austria	18.84	0
N00.N99	Belgium	18.83	0
N00.N99	Croatia	7.21	1
N00.N99	Czech Republic	26.97	0
N00.N99	Denmark	18.70	1
N00.N99	Estonia	8.58	2
N00.N99	Finland	6.16	0
N00.N99	France	70.88	0
N00.N99	Germany	84.78	2
N00.N99	Hungary	72.83	0
N00.N99	Latvia	26.09	2
N00.N99	Lithuania	30.93	1
N00.N99	Netherlands	40.65	0
N00.N99	Norway	11.05	0
N00.N99	Poland	32.29	2
N00.N99	Spain	50.43	0
N00.N99	Sweden	19.25	0
N00.N99	Switzerland	8.43	0
N00.N99	United Kingdom	155.45	1
N00.N99	Hungary	5.65	0
N00.N99	Netherlands	5.93	0
N00.N99	United Kingdom	14.03	0
N00.N99	France	5.66	0
N00.N99	United Kingdom	6.02	0
N00.N99	Austria	36.63	1
N00.N99	Czech Republic	5.39	1
N00.N99	Denmark	6.84	3
N00.N99	Finland	5.58	0
N00.N99	Germany	132.87	1
N00.N99	Hungary	46.88	1
N00.N99	Latvia	51.61	1
N00.N99	Lithuania	6.43	2
N00.N99	Netherlands	24.86	1
N00.N99	Romania	6.92	3

For peer review only

N00.N99	Spain	11.30	1
N00.N99	Switzerland	6.20	1
N00.N99	United Kingdom	19.63	0
N00.N99	United Kingdom	10.42	1
N00.N99	Austria	22.47	1
N00.N99	Belgium	35.21	0
N00.N99	Croatia	5.31	0
N00.N99	Czech Republic	14.90	1
N00.N99	Denmark	9.30	0
N00.N99	France	77.86	2
N00.N99	Germany	162.37	3
N00.N99	Hungary	33.34	2
N00.N99	Latvia	13.27	0
N00.N99	Lithuania	12.47	0
N00.N99	Netherlands	112.12	0
N00.N99	Norway	15.50	0
N00.N99	Poland	24.95	0
N00.N99	Romania	11.46	0
N00.N99	Spain	136.35	0
N00.N99	Sweden	10.70	0
N00.N99	Switzerland	10.39	0
N00.N99	United Kingdom	257.02	0
N00.N99	Austria	13.79	0
N00.N99	Belgium	9.18	1
N00.N99	Croatia	5.59	1
N00.N99	Czech Republic	56.96	1
N00.N99	Denmark	80.84	0
N00.N99	Finland	9.49	0
N00.N99	France	18.06	0
N00.N99	Germany	104.10	1
N00.N99	Hungary	43.70	1
N00.N99	Netherlands	73.93	1
N00.N99	Norway	5.17	0
N00.N99	Poland	17.20	2
N00.N99	Slovenia	7.62	1
N00.N99	Spain	11.36	0
N00.N99	Sweden	5.52	2
N00.N99	Switzerland	7.39	0
N00.N99	United Kingdom	49.13	1
N00.N99	Germany	8.46	3
N00.N99	Netherlands	6.07	0
N00.N99	Belgium	17.23	0
N00.N99	Denmark	7.42	1
N00.N99	France	109.06	0
N00.N99	Germany	50.47	1
N00.N99	Hungary	7.10	0
N00.N99	Netherlands	41.48	0
N00.N99	Poland	9.45	1
N00.N99	Spain	42.05	0
N00.N99	Sweden	9.62	0

N00.N99	Switzerland	8.58	1
N00.N99	United Kingdom	121.79	2
N00.N99	Czech Republic	5.10	0
N00.N99	Germany	7.13	0
N00.N99	United Kingdom	7.77	0
N00.N99	France	5.81	0
N00.N99	Austria	150.43	2
N00.N99	Belgium	459.76	3
N00.N99	Croatia	237.29	1
N00.N99	Czech Republic	169.06	3
N00.N99	Denmark	253.27	3
N00.N99	Finland	63.01	1
N00.N99	France	1446.52	1
N00.N99	Germany	3589.75	3
N00.N99	Hungary	43.38	2
N00.N99	Latvia	14.74	2
N00.N99	Lithuania	12.00	3
N00.N99	Netherlands	1686.04	2
N00.N99	Norway	314.94	2
N00.N99	Poland	333.81	3
N00.N99	Romania	29.66	2
N00.N99	Slovenia	79.15	1
N00.N99	Spain	3266.88	1
N00.N99	Sweden	338.09	2
N00.N99	Switzerland	245.82	2
N00.N99	United Kingdom	6824.06	2
N00.N99	Austria	9.44	0
N00.N99	Belgium	21.66	1
N00.N99	Croatia	30.86	3
N00.N99	Czech Republic	36.12	0
N00.N99	Denmark	49.25	1
N00.N99	Estonia	11.55	0
N00.N99	Finland	26.07	1
N00.N99	France	153.65	1
N00.N99	Germany	125.07	2
N00.N99	Hungary	105.51	0
N00.N99	Latvia	41.47	0
N00.N99	Lithuania	26.93	2
N00.N99	Netherlands	74.52	2
N00.N99	Norway	32.69	0
N00.N99	Poland	53.39	2
N00.N99	Romania	307.31	3
N00.N99	Spain	156.19	0
N00.N99	Sweden	41.39	1
N00.N99	Switzerland	40.51	2
N00.N99	United Kingdom	203.83	1
N00.N99	Belgium	6.74	0
N00.N99	France	107.43	2
N00.N99	Germany	7.13	0
N00.N99	Hungary	8.19	0

For peer review only

N00.N99	Netherlands	6.93	1
N00.N99	Spain	9.33	1
N00.N99	United Kingdom	5.63	0
N00.N99	Belgium	20.15	1
N00.N99	France	104.32	2
N00.N99	Germany	9.22	1
N00.N99	Netherlands	13.67	0
N00.N99	Spain	27.61	1
N00.N99	Sweden	6.02	0
N00.N99	United Kingdom	15.20	1
N00.N99	France	7.86	0
N00.N99	Germany	9.96	0
N00.N99	United Kingdom	6.46	0
N00.N99	Belgium	8.59	0
N00.N99	France	27.86	2
N00.N99	Germany	47.83	1
N00.N99	Hungary	6.99	1
N00.N99	Poland	5.49	0
N00.N99	Spain	54.79	0
N00.N99	United Kingdom	39.05	1
N00.N99	Germany	7.91	1
N00.N99	Hungary	6.32	0
N00.N99	United Kingdom	6.36	0
N00.N99	France	6.94	2
N00.N99	Germany	5.43	0
N00.N99	Hungary	5.02	0
N00.N99	United Kingdom	19.79	1
N00.N99	France	17.60	1
N00.N99	Spain	6.61	1
N00.N99	United Kingdom	37.66	1
N00.N99	Germany	17.41	1
N00.N99	Spain	17.58	3
N00.N99	France	11.86	2
N00.N99	Germany	6.23	0
N00.N99	Netherlands	5.60	0
N00.N99	United Kingdom	8.56	2
N00.N99	France	23.85	1
N00.N99	Germany	13.28	1
N00.N99	Netherlands	5.73	1
N00.N99	Spain	10.07	0
N00.N99	United Kingdom	30.16	0
N00.N99	France	12.35	0
N00.N99	United Kingdom	26.21	0
N00.N99	Spain	5.27	0
N00.N99	United Kingdom	18.15	2
N00.N99	Germany	5.27	0
N00.N99	Netherlands	15.07	0
N00.N99	Netherlands	6.01	1
N00.N99	Spain	6.28	0
O00.O99	Romania	5.68	2

For peer review only

O00.O99	France	6.23	0
O00.O99	France	9.85	0
O00.O99	Germany	6.42	0
O00.O99	France	6.56	3
O00.O99	United Kingdom	12.36	0
P00.P96	Belgium	12.53	1
P00.P96	France	31.79	0
P00.P96	Germany	12.09	1
P00.P96	Netherlands	5.19	0
P00.P96	Norway	5.50	1
P00.P96	Belgium	17.52	0
P00.P96	Croatia	13.85	1
P00.P96	Denmark	7.44	0
P00.P96	France	78.58	0
P00.P96	Germany	97.59	2
P00.P96	Netherlands	26.95	2
P00.P96	Norway	11.34	0
P00.P96	Poland	10.76	1
P00.P96	Spain	56.26	1
P00.P96	Sweden	6.15	3
P00.P96	Switzerland	19.07	1
P00.P96	United Kingdom	20.46	0
P00.P96	Austria	17.59	2
P00.P96	Belgium	35.23	2
P00.P96	Croatia	19.81	2
P00.P96	Denmark	12.03	0
P00.P96	France	133.26	1
P00.P96	Germany	131.39	2
P00.P96	Netherlands	25.37	0
P00.P96	Norway	18.74	2
P00.P96	Poland	17.06	0
P00.P96	Romania	5.11	0
P00.P96	Spain	31.86	0
P00.P96	Sweden	12.37	1
P00.P96	Switzerland	27.24	1
P00.P96	United Kingdom	26.61	0
P00.P96	France	21.28	0
P00.P96	Germany	15.76	1
P00.P96	Netherlands	5.18	0
P00.P96	France	29.90	3
P00.P96	Germany	5.44	1
P00.P96	Spain	7.59	0
P00.P96	United Kingdom	9.24	0
P00.P96	Austria	89.65	3
P00.P96	Belgium	15.54	0
P00.P96	Croatia	8.81	0
P00.P96	Czech Republic	11.87	1
P00.P96	Denmark	66.78	0
P00.P96	Finland	9.53	1
P00.P96	France	96.24	2

For peer review only

P00.P96	Germany	701.08	3
P00.P96	Hungary	87.86	2
P00.P96	Lithuania	8.64	1
P00.P96	Netherlands	70.17	2
P00.P96	Norway	5.32	2
P00.P96	Poland	632.69	2
P00.P96	Slovenia	9.56	0
P00.P96	Spain	85.24	1
P00.P96	Sweden	7.63	0
P00.P96	Switzerland	28.79	0
P00.P96	United Kingdom	807.11	3
P00.P96	Poland	10.31	2
P00.P96	Romania	15.32	3
P00.P96	Czech Republic	5.80	0
P00.P96	France	55.60	2
P00.P96	Germany	20.26	1
P00.P96	Hungary	11.74	1
P00.P96	Netherlands	7.14	2
P00.P96	Poland	33.57	1
P00.P96	Spain	28.72	2
P00.P96	United Kingdom	11.86	1
P00.P96	Austria	9.30	2
P00.P96	Belgium	10.70	0
P00.P96	Croatia	5.78	1
P00.P96	Czech Republic	20.81	3
P00.P96	Denmark	7.33	1
P00.P96	France	44.43	1
P00.P96	Germany	83.56	0
P00.P96	Hungary	13.78	0
P00.P96	Latvia	13.49	2
P00.P96	Lithuania	12.63	1
P00.P96	Netherlands	40.91	1
P00.P96	Norway	6.39	0
P00.P96	Poland	81.95	3
P00.P96	Romania	43.00	2
P00.P96	Spain	29.15	2
P00.P96	Sweden	13.91	2
P00.P96	Switzerland	11.26	0
P00.P96	United Kingdom	112.75	2
P00.P96	Belgium	14.73	1
P00.P96	Croatia	25.10	1
P00.P96	Czech Republic	20.25	2
P00.P96	Estonia	6.39	0
P00.P96	Finland	7.04	1
P00.P96	France	46.31	2
P00.P96	Germany	52.37	2
P00.P96	Hungary	43.58	0
P00.P96	Latvia	5.49	0
P00.P96	Lithuania	13.39	3
P00.P96	Netherlands	6.27	0

For peer review only

P00.P96	Poland	52.36	3
P00.P96	Romania	118.96	0
P00.P96	Spain	66.50	2
P00.P96	Sweden	5.49	1
P00.P96	Switzerland	11.78	0
P00.P96	United Kingdom	52.67	2
P00.P96	Hungary	10.82	2
P00.P96	Latvia	5.29	0
P00.P96	Poland	16.05	1
P00.P96	Romania	86.26	1
P00.P96	Spain	6.99	0
P00.P96	United Kingdom	30.51	1
P00.P96	France	9.58	0
P00.P96	Germany	17.28	0
P00.P96	Poland	9.64	1
P00.P96	Romania	33.04	1
P00.P96	Spain	12.31	1
P00.P96	United Kingdom	10.00	1
P00.P96	France	9.24	1
P00.P96	Germany	10.92	0
P00.P96	Spain	14.88	0
P00.P96	United Kingdom	17.03	0
P00.P96	France	19.44	2
P00.P96	Germany	7.66	1
P00.P96	Hungary	9.69	0
P00.P96	Romania	39.03	0
P00.P96	Spain	21.11	1
P00.P96	United Kingdom	6.65	2
P00.P96	France	27.17	1
P00.P96	Germany	26.80	0
P00.P96	Hungary	7.18	1
P00.P96	Netherlands	7.85	0
P00.P96	Poland	10.39	1
P00.P96	Spain	21.57	0
P00.P96	United Kingdom	60.52	2
P00.P96	Belgium	8.80	0
P00.P96	Czech Republic	8.28	1
P00.P96	France	34.08	3
P00.P96	Germany	41.40	1
P00.P96	Hungary	32.53	2
P00.P96	Netherlands	11.02	0
P00.P96	Poland	17.89	1
P00.P96	Romania	175.40	0
P00.P96	Spain	30.71	1
P00.P96	Sweden	5.95	0
P00.P96	Switzerland	11.81	2
P00.P96	United Kingdom	86.63	2
P00.P96	Czech Republic	6.00	0
P00.P96	France	67.90	3
P00.P96	Germany	32.50	1

For peer review only

P00.P96	Hungary	6.32	0
P00.P96	Poland	10.47	1
P00.P96	Spain	35.79	2
P00.P96	Sweden	6.62	2
P00.P96	United Kingdom	11.21	1
P00.P96	Germany	7.49	0
P00.P96	United Kingdom	11.81	0
P00.P96	Belgium	10.76	3
P00.P96	Croatia	11.44	0
P00.P96	Czech Republic	14.49	2
P00.P96	France	37.20	2
P00.P96	Germany	59.22	1
P00.P96	Hungary	9.13	3
P00.P96	Lithuania	7.49	2
P00.P96	Netherlands	27.43	0
P00.P96	Poland	75.45	2
P00.P96	Romania	14.56	0
P00.P96	Spain	97.96	1
P00.P96	Sweden	6.58	2
P00.P96	Switzerland	8.42	0
P00.P96	United Kingdom	41.03	1
P00.P96	France	7.18	0
P00.P96	Spain	6.74	0
P00.P96	United Kingdom	8.86	1
P00.P96	France	46.95	0
P00.P96	Germany	6.95	0
P00.P96	Netherlands	12.77	0
P00.P96	Poland	41.21	3
P00.P96	Spain	5.80	0
P00.P96	United Kingdom	5.36	0
P00.P96	Belgium	19.35	1
P00.P96	Croatia	11.01	2
P00.P96	Czech Republic	25.73	3
P00.P96	Finland	9.07	1
P00.P96	France	125.19	0
P00.P96	Germany	48.54	0
P00.P96	Hungary	78.48	1
P00.P96	Latvia	14.79	1
P00.P96	Netherlands	17.30	2
P00.P96	Poland	56.35	1
P00.P96	Romania	270.83	3
P00.P96	Spain	70.57	1
P00.P96	Sweden	6.79	1
P00.P96	Switzerland	10.06	0
P00.P96	United Kingdom	47.47	3
P00.P96	Romania	63.47	3
P00.P96	Romania	10.67	0
P00.P96	Romania	6.00	1
P00.P96	France	7.44	3
P00.P96	Spain	5.27	0

For peer review only

P00.P96	France	9.57	3
P00.P96	Germany	5.18	1
P00.P96	Belgium	6.04	0
P00.P96	Czech Republic	9.19	1
P00.P96	France	31.85	0
P00.P96	Germany	44.11	1
P00.P96	Hungary	13.86	1
P00.P96	Netherlands	21.57	1
P00.P96	Poland	8.98	3
P00.P96	Romania	12.26	1
P00.P96	Spain	47.63	3
P00.P96	Sweden	5.33	1
P00.P96	Switzerland	5.08	0
P00.P96	United Kingdom	102.30	0
P00.P96	France	18.05	0
P00.P96	Germany	10.82	1
P00.P96	Romania	8.53	1
P00.P96	Spain	12.41	0
P00.P96	France	9.05	1
P00.P96	Germany	16.60	0
P00.P96	Poland	12.03	0
P00.P96	Spain	7.75	2
P00.P96	United Kingdom	6.86	0
P00.P96	France	69.04	1
P00.P96	Germany	9.98	2
P00.P96	Latvia	14.43	2
P00.P96	Spain	43.98	2
P00.P96	United Kingdom	40.96	2
P00.P96	France	30.54	3
P00.P96	Germany	38.06	0
P00.P96	Netherlands	37.55	2
P00.P96	Spain	83.16	0
P00.P96	United Kingdom	12.01	0
Q00.Q99	France	5.01	1
Q00.Q99	Germany	18.32	0
Q00.Q99	Netherlands	6.95	1
Q00.Q99	Poland	28.49	2
Q00.Q99	Spain	5.82	1
Q00.Q99	United Kingdom	19.87	1
Q00.Q99	Poland	9.28	2
Q00.Q99	France	9.56	2
Q00.Q99	Germany	9.67	0
Q00.Q99	United Kingdom	18.28	2
Q00.Q99	Belgium	5.31	1
Q00.Q99	Czech Republic	7.12	1
Q00.Q99	France	24.68	2
Q00.Q99	Germany	28.78	3
Q00.Q99	Hungary	9.82	1
Q00.Q99	Lithuania	6.24	1
Q00.Q99	Poland	41.97	1

For peer review only

Q00.Q99	Romania	69.65	1
Q00.Q99	Spain	11.76	1
Q00.Q99	United Kingdom	28.00	1
Q00.Q99	Austria	8.63	0
Q00.Q99	Belgium	8.04	1
Q00.Q99	Czech Republic	7.15	0
Q00.Q99	Finland	6.31	0
Q00.Q99	France	40.65	1
Q00.Q99	Germany	66.77	0
Q00.Q99	Hungary	18.12	1
Q00.Q99	Netherlands	14.98	0
Q00.Q99	Poland	28.58	2
Q00.Q99	Romania	23.25	0
Q00.Q99	Spain	36.96	0
Q00.Q99	Sweden	7.66	1
Q00.Q99	Switzerland	8.76	0
Q00.Q99	United Kingdom	59.05	1
Q00.Q99	France	22.23	0
Q00.Q99	Germany	20.65	1
Q00.Q99	Netherlands	11.36	2
Q00.Q99	Poland	13.11	1
Q00.Q99	Romania	32.47	1
Q00.Q99	Spain	9.60	0
Q00.Q99	United Kingdom	57.71	0
Q00.Q99	France	8.68	0
Q00.Q99	Germany	13.03	0
Q00.Q99	Poland	9.78	0
Q00.Q99	Romania	5.97	0
Q00.Q99	Spain	11.07	0
Q00.Q99	United Kingdom	11.55	0
Q00.Q99	Belgium	6.48	0
Q00.Q99	Czech Republic	7.50	2
Q00.Q99	Denmark	5.34	1
Q00.Q99	Finland	5.69	1
Q00.Q99	France	47.49	1
Q00.Q99	Germany	52.96	0
Q00.Q99	Hungary	16.29	1
Q00.Q99	Lithuania	8.49	1
Q00.Q99	Netherlands	10.54	0
Q00.Q99	Poland	70.64	2
Q00.Q99	Romania	74.57	1
Q00.Q99	Spain	26.54	2
Q00.Q99	Switzerland	8.62	1
Q00.Q99	United Kingdom	40.05	1
Q00.Q99	Austria	19.61	2
Q00.Q99	Belgium	15.32	0
Q00.Q99	Croatia	9.97	0
Q00.Q99	Czech Republic	17.78	0
Q00.Q99	Denmark	11.28	0
Q00.Q99	Finland	21.44	0

For peer review only

Q00.Q99	France	108.15	1
Q00.Q99	Germany	121.55	2
Q00.Q99	Hungary	37.94	2
Q00.Q99	Latvia	9.97	1
Q00.Q99	Lithuania	15.11	1
Q00.Q99	Netherlands	29.01	1
Q00.Q99	Norway	10.50	0
Q00.Q99	Poland	89.71	2
Q00.Q99	Romania	94.95	1
Q00.Q99	Slovenia	5.44	1
Q00.Q99	Spain	73.68	2
Q00.Q99	Sweden	18.22	0
Q00.Q99	Switzerland	14.50	0
Q00.Q99	United Kingdom	160.80	2
Q00.Q99	France	28.59	1
Q00.Q99	Germany	28.28	0
Q00.Q99	Poland	25.19	0
Q00.Q99	Romania	7.05	0
Q00.Q99	Spain	8.12	0
Q00.Q99	United Kingdom	15.96	0
Q00.Q99	Austria	6.54	0
Q00.Q99	Belgium	10.64	1
Q00.Q99	Croatia	5.95	0
Q00.Q99	Czech Republic	8.97	0
Q00.Q99	Denmark	5.68	2
Q00.Q99	Finland	8.71	0
Q00.Q99	France	47.41	3
Q00.Q99	Germany	92.00	1
Q00.Q99	Hungary	15.87	0
Q00.Q99	Lithuania	9.98	1
Q00.Q99	Netherlands	19.79	1
Q00.Q99	Norway	7.27	1
Q00.Q99	Poland	70.60	0
Q00.Q99	Romania	12.75	0
Q00.Q99	Spain	23.73	2
Q00.Q99	Sweden	7.91	2
Q00.Q99	Switzerland	9.67	3
Q00.Q99	United Kingdom	103.34	1
Q00.Q99	Austria	23.84	1
Q00.Q99	Belgium	23.02	2
Q00.Q99	Croatia	12.33	2
Q00.Q99	Czech Republic	9.91	0
Q00.Q99	Denmark	8.64	1
Q00.Q99	France	164.81	1
Q00.Q99	Germany	134.90	3
Q00.Q99	Hungary	34.21	1
Q00.Q99	Latvia	11.81	1
Q00.Q99	Lithuania	5.63	1
Q00.Q99	Netherlands	38.60	1
Q00.Q99	Norway	8.06	0

For peer review only

Q00.Q99	Poland	124.21	3
Q00.Q99	Romania	147.03	2
Q00.Q99	Spain	164.96	1
Q00.Q99	Sweden	15.09	1
Q00.Q99	Switzerland	18.26	1
Q00.Q99	United Kingdom	228.97	1
Q00.Q99	Austria	5.18	0
Q00.Q99	Belgium	6.20	0
Q00.Q99	Czech Republic	9.81	1
Q00.Q99	Denmark	6.63	0
Q00.Q99	France	46.69	2
Q00.Q99	Germany	83.84	1
Q00.Q99	Hungary	32.56	0
Q00.Q99	Netherlands	9.64	0
Q00.Q99	Poland	27.31	0
Q00.Q99	Romania	27.44	3
Q00.Q99	Spain	43.43	0
Q00.Q99	Sweden	6.85	0
Q00.Q99	Switzerland	5.87	0
Q00.Q99	United Kingdom	42.80	1
Q00.Q99	France	7.16	0
Q00.Q99	Germany	9.13	0
Q00.Q99	Poland	5.59	2
Q00.Q99	United Kingdom	6.97	0
Q00.Q99	France	39.69	1
Q00.Q99	Spain	6.51	0
Q00.Q99	United Kingdom	23.03	1
Q00.Q99	France	22.09	1
Q00.Q99	Germany	24.44	2
Q00.Q99	Hungary	6.95	0
Q00.Q99	Netherlands	5.42	0
Q00.Q99	Poland	7.58	1
Q00.Q99	Romania	8.39	0
Q00.Q99	Spain	31.50	1
Q00.Q99	Switzerland	8.25	0
Q00.Q99	United Kingdom	39.89	2
Q00.Q99	France	6.30	0
Q00.Q99	Germany	6.67	0
Q00.Q99	United Kingdom	8.46	0
Q00.Q99	Austria	5.04	1
Q00.Q99	Belgium	5.70	2
Q00.Q99	France	24.97	0
Q00.Q99	Germany	70.10	1
Q00.Q99	Hungary	5.61	2
Q00.Q99	Netherlands	14.75	1
Q00.Q99	Poland	23.53	2
Q00.Q99	Romania	14.65	0
Q00.Q99	Spain	18.23	1
Q00.Q99	United Kingdom	66.36	1
Q00.Q99	United Kingdom	20.30	1

For peer review only

Q00.Q99	France	12.77	2
Q00.Q99	Germany	7.34	0
Q00.Q99	Poland	6.23	1
Q00.Q99	Romania	17.73	3
Q00.Q99	Spain	9.06	0
Q00.Q99	United Kingdom	16.26	0
Q00.Q99	France	6.22	0
Q00.Q99	Romania	16.56	1
Q00.Q99	France	26.97	1
Q00.Q99	Germany	18.63	0
Q00.Q99	Hungary	5.70	0
Q00.Q99	Netherlands	6.14	0
Q00.Q99	Poland	7.29	0
Q00.Q99	Romania	10.94	2
Q00.Q99	Spain	18.13	0
Q00.Q99	United Kingdom	27.08	1
Q00.Q99	France	27.71	0
Q00.Q99	Germany	15.37	0
Q00.Q99	Romania	12.65	1
Q00.Q99	Spain	20.72	0
Q00.Q99	United Kingdom	13.90	0
Q00.Q99	France	17.45	0
Q00.Q99	Germany	32.99	2
Q00.Q99	Netherlands	6.84	1
Q00.Q99	Poland	27.25	0
Q00.Q99	Spain	7.48	1
Q00.Q99	United Kingdom	20.16	1
Q00.Q99	Austria	12.86	0
Q00.Q99	Belgium	7.40	2
Q00.Q99	Czech Republic	7.16	1
Q00.Q99	Denmark	18.85	3
Q00.Q99	Finland	19.33	0
Q00.Q99	France	69.27	1
Q00.Q99	Germany	44.48	3
Q00.Q99	Hungary	30.30	2
Q00.Q99	Lithuania	5.52	0
Q00.Q99	Netherlands	7.39	0
Q00.Q99	Norway	7.38	0
Q00.Q99	Poland	31.71	0
Q00.Q99	Romania	9.70	1
Q00.Q99	Spain	41.01	0
Q00.Q99	Sweden	24.64	1
Q00.Q99	Switzerland	18.28	1
Q00.Q99	United Kingdom	132.43	0
Q00.Q99	Germany	5.06	0
Q00.Q99	United Kingdom	7.02	0
Q00.Q99	United Kingdom	8.09	0
Q00.Q99	France	8.43	1
Q00.Q99	France	5.55	0
Q00.Q99	Germany	12.43	1

For peer review only

Q00.Q99	United Kingdom	7.36	0
Q00.Q99	France	10.09	0
Q00.Q99	Germany	12.65	1
Q00.Q99	Spain	5.03	0
Q00.Q99	United Kingdom	16.75	0
Q00.Q99	Austria	7.91	0
Q00.Q99	Belgium	8.52	0
Q00.Q99	Croatia	8.53	1
Q00.Q99	France	55.73	1
Q00.Q99	Germany	56.95	0
Q00.Q99	Hungary	8.10	0
Q00.Q99	Netherlands	13.53	0
Q00.Q99	Poland	54.47	0
Q00.Q99	Romania	35.40	1
Q00.Q99	Spain	31.44	0
Q00.Q99	Sweden	5.11	1
Q00.Q99	Switzerland	7.97	0
Q00.Q99	United Kingdom	62.70	0
Q00.Q99	Germany	7.43	1
Q00.Q99	United Kingdom	7.39	0
Q00.Q99	United Kingdom	6.23	0
Q00.Q99	Austria	6.44	0
Q00.Q99	France	30.32	1
Q00.Q99	Germany	28.14	1
Q00.Q99	Netherlands	8.82	0
Q00.Q99	Poland	7.47	0
Q00.Q99	Spain	22.63	0
Q00.Q99	Sweden	5.18	0
Q00.Q99	Switzerland	6.26	0
Q00.Q99	United Kingdom	27.01	0
Q00.Q99	Austria	10.68	0
Q00.Q99	Belgium	10.38	1
Q00.Q99	Denmark	7.20	2
Q00.Q99	Finland	7.46	0
Q00.Q99	France	48.15	0
Q00.Q99	Germany	90.20	1
Q00.Q99	Hungary	5.82	0
Q00.Q99	Netherlands	17.94	0
Q00.Q99	Norway	5.64	0
Q00.Q99	Poland	25.29	2
Q00.Q99	Romania	5.82	0
Q00.Q99	Spain	30.90	0
Q00.Q99	Sweden	8.43	0
Q00.Q99	Switzerland	13.05	2
Q00.Q99	United Kingdom	69.56	0
Q00.Q99	Austria	14.38	0
Q00.Q99	Belgium	21.96	0
Q00.Q99	Croatia	9.60	1
Q00.Q99	Czech Republic	6.39	1
Q00.Q99	France	91.32	0

For peer review only

Q00.Q99	Germany	71.48	0
Q00.Q99	Hungary	22.23	1
Q00.Q99	Latvia	12.93	2
Q00.Q99	Lithuania	17.71	1
Q00.Q99	Netherlands	20.52	1
Q00.Q99	Poland	144.12	1
Q00.Q99	Romania	68.65	1
Q00.Q99	Spain	62.48	1
Q00.Q99	Sweden	5.38	1
Q00.Q99	Switzerland	11.16	0
Q00.Q99	United Kingdom	60.49	0
Q00.Q99	Austria	60.48	3
Q00.Q99	Belgium	35.93	1
Q00.Q99	Croatia	16.56	0
Q00.Q99	Czech Republic	13.40	2
Q00.Q99	Denmark	30.86	0
Q00.Q99	Finland	34.59	1
Q00.Q99	France	223.58	1
Q00.Q99	Germany	329.53	1
Q00.Q99	Hungary	32.26	2
Q00.Q99	Latvia	8.60	0
Q00.Q99	Lithuania	6.51	0
Q00.Q99	Netherlands	122.17	2
Q00.Q99	Norway	34.78	0
Q00.Q99	Poland	27.00	2
Q00.Q99	Romania	6.66	2
Q00.Q99	Slovenia	6.11	0
Q00.Q99	Spain	125.28	1
Q00.Q99	Sweden	49.15	0
Q00.Q99	Switzerland	54.56	0
Q00.Q99	United Kingdom	287.89	3
Q00.Q99	Austria	10.42	0
Q00.Q99	Belgium	8.85	0
Q00.Q99	Croatia	6.73	0
Q00.Q99	Denmark	5.26	1
Q00.Q99	Finland	7.94	1
Q00.Q99	France	28.20	0
Q00.Q99	Germany	103.20	0
Q00.Q99	Hungary	7.52	0
Q00.Q99	Lithuania	5.26	0
Q00.Q99	Netherlands	23.05	1
Q00.Q99	Norway	8.17	0
Q00.Q99	Poland	63.07	3
Q00.Q99	Spain	26.33	1
Q00.Q99	Sweden	13.82	1
Q00.Q99	Switzerland	13.24	0
Q00.Q99	United Kingdom	66.81	0
Q00.Q99	France	31.16	0
Q00.Q99	Germany	15.08	2
Q00.Q99	France	6.55	0

For peer review only

Q00.Q99	Germany	10.31	1
Q00.Q99	United Kingdom	12.08	1
Q00.Q99	France	18.27	1
Q00.Q99	Germany	19.00	0
Q00.Q99	Netherlands	6.92	0
Q00.Q99	Poland	5.40	2
Q00.Q99	Spain	7.64	0
Q00.Q99	United Kingdom	14.27	1
R00.R99	Belgium	5.48	0
R00.R99	Denmark	5.00	0
R00.R99	France	31.93	0
R00.R99	Germany	20.61	1
R00.R99	Netherlands	18.99	1
R00.R99	Spain	20.30	0
R00.R99	Austria	15.04	0
R00.R99	Belgium	6.03	0
R00.R99	Denmark	7.26	2
R00.R99	France	36.35	1
R00.R99	Germany	38.91	0
R00.R99	Spain	7.01	1
R00.R99	United Kingdom	10.03	1
R00.R99	Belgium	10.17	1
R00.R99	France	62.63	1
R00.R99	Germany	36.58	2
R00.R99	Netherlands	49.71	2
R00.R99	Poland	32.00	2
R00.R99	Spain	50.29	1
R00.R99	United Kingdom	9.91	1
R00.R99	Belgium	17.15	1
R00.R99	France	107.03	1
R00.R99	Germany	25.64	0
R00.R99	Netherlands	29.64	3
R00.R99	Spain	19.72	0
R00.R99	Sweden	12.70	2
R00.R99	France	170.05	0
R00.R99	Austria	33.26	2
R00.R99	Belgium	795.57	2
R00.R99	Croatia	187.77	1
R00.R99	Czech Republic	111.02	2
R00.R99	Denmark	12.99	2
R00.R99	France	13352.46	0
R00.R99	Germany	100.73	3
R00.R99	Netherlands	53.35	2
R00.R99	Norway	21.20	1
R00.R99	Poland	1592.80	1
R00.R99	Spain	1273.48	3
R00.R99	Sweden	13.50	0
R00.R99	Switzerland	225.14	3
R00.R99	United Kingdom	28.20	3
R00.R99	Belgium	11.82	0

For peer review only

R00.R99	Croatia	5.10	2
R00.R99	Denmark	20.31	0
R00.R99	France	80.64	1
R00.R99	Germany	92.06	0
R00.R99	Netherlands	137.21	2
R00.R99	Spain	67.51	1
R00.R99	Switzerland	5.09	2
R00.R99	United Kingdom	5.07	0
R00.R99	France	31.42	1
R00.R99	Germany	18.93	1
R00.R99	Netherlands	46.05	2
R00.R99	Belgium	11.15	0
R00.R99	France	27.51	0
R00.R99	Germany	91.04	3
R00.R99	Netherlands	16.56	0
R00.R99	France	6.55	0
R00.R99	Germany	9.53	2
R00.R99	Netherlands	18.95	2
R00.R99	France	8.40	0
R00.R99	Germany	7.01	0
R00.R99	France	11.04	0
R00.R99	Germany	11.31	2
R00.R99	Netherlands	44.32	2
R00.R99	Spain	7.02	0
R00.R99	United Kingdom	51.03	1
R00.R99	France	8.92	0
R00.R99	France	163.31	2
R00.R99	France	8.08	0
R00.R99	Netherlands	9.15	0
R00.R99	Spain	7.98	1
R00.R99	Netherlands	17.10	2
R00.R99	France	5.37	0
R00.R99	Belgium	11.31	0
R00.R99	Croatia	5.57	2
R00.R99	France	183.44	1
R00.R99	Germany	24.69	2
R00.R99	Netherlands	14.21	1
R00.R99	Spain	23.84	3
R00.R99	Belgium	6.55	2
R00.R99	France	106.95	3
R00.R99	Belgium	10.36	0
R00.R99	Denmark	5.73	1
R00.R99	France	83.98	0
R00.R99	Germany	18.66	1
R00.R99	Netherlands	124.59	3
R00.R99	Spain	39.16	1
R00.R99	France	7.64	0
R00.R99	France	7.60	3
R00.R99	Germany	6.43	1
R00.R99	Netherlands	16.70	1

For peer review only

R00.R99	Belgium	139.15	3
R00.R99	Denmark	59.26	0
R00.R99	France	1759.51	1
R00.R99	Germany	17.52	1
R00.R99	Netherlands	188.89	1
R00.R99	Norway	10.85	1
R00.R99	Spain	60.93	3
R00.R99	Sweden	57.44	1
R00.R99	United Kingdom	167.39	3
R00.R99	Austria	702.81	2
R00.R99	Belgium	1137.07	2
R00.R99	Croatia	597.92	1
R00.R99	Czech Republic	181.69	1
R00.R99	Denmark	829.14	3
R00.R99	Estonia	514.48	3
R00.R99	Finland	18.24	3
R00.R99	France	5732.29	3
R00.R99	Germany	4462.96	1
R00.R99	Hungary	86.70	3
R00.R99	Latvia	1861.35	3
R00.R99	Lithuania	48.06	3
R00.R99	Netherlands	2203.33	2
R00.R99	Norway	489.06	2
R00.R99	Poland	17652.87	2
R00.R99	Slovenia	108.95	2
R00.R99	Spain	2955.32	3
R00.R99	Sweden	1301.58	2
R00.R99	Switzerland	472.77	2
R00.R99	United Kingdom	11499.12	2
R00.R99	Belgium	11.53	0
R00.R99	France	228.06	2
R00.R99	Germany	97.39	3
R00.R99	Netherlands	7.39	2
R00.R99	Spain	22.66	1
R00.R99	France	47.74	1
R00.R99	Germany	41.13	0
R00.R99	Netherlands	7.89	1
R00.R99	United Kingdom	8.20	0
R00.R99	Belgium	178.12	2
R00.R99	Croatia	6.96	3
R00.R99	Czech Republic	13.87	0
R00.R99	Denmark	14.24	0
R00.R99	France	1236.35	3
R00.R99	Germany	174.87	2
R00.R99	Netherlands	40.89	3
R00.R99	Poland	271.60	2
R00.R99	Spain	664.65	2
R00.R99	Sweden	119.69	1
R00.R99	Switzerland	9.64	0
R00.R99	United Kingdom	7.66	1

For peer review only

R00.R99	Belgium	27.91	1
R00.R99	Denmark	5.50	0
R00.R99	France	129.16	1
R00.R99	Germany	63.92	0
R00.R99	Netherlands	105.46	2
R00.R99	Spain	144.31	2
R00.R99	United Kingdom	27.09	2
R00.R99	France	32.65	1
R00.R99	Germany	5.22	1
R00.R99	Spain	5.40	1
R00.R99	Belgium	18.84	2
R00.R99	Denmark	6.15	0
R00.R99	France	125.68	1
R00.R99	Germany	78.57	1
R00.R99	Netherlands	45.81	3
R00.R99	Norway	17.22	2
R00.R99	Spain	5.99	0
R00.R99	Austria	55.33	0
R00.R99	Belgium	106.18	3
R00.R99	Croatia	10.20	2
R00.R99	Czech Republic	22.58	1
R00.R99	Denmark	37.56	1
R00.R99	France	807.94	1
R00.R99	Germany	784.25	2
R00.R99	Netherlands	271.66	3
R00.R99	Norway	8.71	0
R00.R99	Poland	105.25	0
R00.R99	Spain	38.65	3
R00.R99	Sweden	17.61	3
R00.R99	Switzerland	7.45	3
R00.R99	United Kingdom	15.23	3
R00.R99	Belgium	32.86	2
R00.R99	France	317.57	1
R00.R99	Germany	321.30	1
R00.R99	Netherlands	12.25	3
R00.R99	Norway	27.49	2
R00.R99	Spain	390.81	3
R00.R99	Sweden	67.76	2
R00.R99	United Kingdom	358.97	3
R00.R99	Germany	6.20	0
R00.R99	Netherlands	7.37	1
R00.R99	France	7.87	2
R00.R99	Austria	21.55	1
R00.R99	Belgium	42.10	2
R00.R99	Croatia	9.00	0
R00.R99	Czech Republic	15.30	0
R00.R99	Denmark	7.09	2
R00.R99	Finland	11.73	0
R00.R99	France	196.16	3
R00.R99	Germany	276.12	2

For peer review only

R00.R99	Hungary	21.45	0
R00.R99	Latvia	17.46	1
R00.R99	Lithuania	8.66	3
R00.R99	Netherlands	14.72	3
R00.R99	Norway	12.19	1
R00.R99	Poland	54.15	2
R00.R99	Romania	11.03	3
R00.R99	Spain	66.19	1
R00.R99	Sweden	18.21	2
R00.R99	Switzerland	12.38	1
R00.R99	United Kingdom	168.31	3
R00.R99	Belgium	593.48	2
R00.R99	Croatia	73.32	2
R00.R99	Czech Republic	71.68	3
R00.R99	Denmark	123.79	1
R00.R99	Finland	10.11	1
R00.R99	France	2006.19	2
R00.R99	Germany	147.04	0
R00.R99	Hungary	7.65	1
R00.R99	Latvia	7.74	0
R00.R99	Lithuania	13.86	0
R00.R99	Netherlands	596.14	2
R00.R99	Norway	698.84	3
R00.R99	Poland	8642.09	2
R00.R99	Spain	208.73	0
R00.R99	Sweden	11.15	1
R00.R99	Switzerland	33.33	0
R00.R99	United Kingdom	64.43	1
R00.R99	Belgium	210.40	1
R00.R99	Croatia	17.69	2
R00.R99	Czech Republic	283.25	2
R00.R99	Denmark	526.00	1
R00.R99	Finland	107.71	0
R00.R99	France	522.46	1
R00.R99	Germany	4226.09	1
R00.R99	Hungary	25.84	1
R00.R99	Latvia	190.76	1
R00.R99	Lithuania	22.69	2
R00.R99	Netherlands	199.23	3
R00.R99	Norway	91.12	1
R00.R99	Poland	1852.61	3
R00.R99	Romania	25.65	0
R00.R99	Spain	439.62	1
R00.R99	Switzerland	29.28	3
R00.R99	United Kingdom	22.35	1
R00.R99	Austria	770.20	1
R00.R99	Belgium	697.17	2
R00.R99	Croatia	152.88	2
R00.R99	Czech Republic	738.46	2
R00.R99	Denmark	2507.71	2

For peer review only

R00.R99	Estonia	158.72	3
R00.R99	Finland	208.05	3
R00.R99	France	15127.84	2
R00.R99	Germany	12495.54	2
R00.R99	Hungary	33.64	0
R00.R99	Latvia	121.54	3
R00.R99	Lithuania	482.17	2
R00.R99	Netherlands	3420.42	3
R00.R99	Norway	1003.78	1
R00.R99	Poland	6181.63	3
R00.R99	Romania	1669.72	1
R00.R99	Slovenia	398.33	2
R00.R99	Spain	5331.01	2
R00.R99	Sweden	1372.78	3
R00.R99	Switzerland	1734.82	0
R00.R99	United Kingdom	1285.04	3
V01.Y98	Germany	5.53	0
V01.Y98	Poland	6.91	1
V01.Y98	Romania	9.22	0
V01.Y98	France	7.32	1
V01.Y98	Germany	14.69	1
V01.Y98	Hungary	6.44	1
V01.Y98	Netherlands	5.32	2
V01.Y98	Poland	14.03	1
V01.Y98	Spain	16.95	0
V01.Y98	United Kingdom	18.51	1
V01.Y98	Austria	52.86	2
V01.Y98	Belgium	57.08	1
V01.Y98	Croatia	74.96	2
V01.Y98	Czech Republic	146.17	2
V01.Y98	Denmark	33.75	1
V01.Y98	Estonia	29.69	3
V01.Y98	Finland	29.70	1
V01.Y98	France	86.00	2
V01.Y98	Germany	407.96	1
V01.Y98	Hungary	262.45	2
V01.Y98	Latvia	95.17	3
V01.Y98	Lithuania	156.10	3
V01.Y98	Netherlands	57.82	3
V01.Y98	Norway	19.67	2
V01.Y98	Poland	1436.65	2
V01.Y98	Romania	211.84	2
V01.Y98	Slovenia	23.43	1
V01.Y98	Spain	208.02	3
V01.Y98	Sweden	34.92	1
V01.Y98	Switzerland	37.34	1
V01.Y98	United Kingdom	289.97	1
V01.Y98	Austria	19.05	0
V01.Y98	Belgium	19.22	0
V01.Y98	Croatia	7.94	0

For peer review only

V01.Y98	Czech Republic	26.68	0
V01.Y98	Denmark	7.88	1
V01.Y98	Estonia	5.39	1
V01.Y98	Finland	10.72	2
V01.Y98	France	31.53	0
V01.Y98	Germany	108.77	1
V01.Y98	Hungary	20.06	1
V01.Y98	Latvia	18.45	1
V01.Y98	Lithuania	19.58	1
V01.Y98	Netherlands	19.12	1
V01.Y98	Norway	6.68	1
V01.Y98	Poland	40.97	1
V01.Y98	Romania	9.31	1
V01.Y98	Spain	48.45	1
V01.Y98	Sweden	10.97	1
V01.Y98	Switzerland	11.77	2
V01.Y98	United Kingdom	101.78	2
V01.Y98	Austria	21.35	1
V01.Y98	Belgium	20.76	0
V01.Y98	Croatia	19.94	1
V01.Y98	Czech Republic	119.73	0
V01.Y98	Denmark	6.60	0
V01.Y98	Estonia	10.68	1
V01.Y98	Finland	6.18	2
V01.Y98	France	76.14	1
V01.Y98	Germany	160.49	1
V01.Y98	Hungary	64.89	1
V01.Y98	Latvia	28.12	1
V01.Y98	Lithuania	24.21	2
V01.Y98	Netherlands	9.05	1
V01.Y98	Poland	257.80	1
V01.Y98	Romania	95.28	1
V01.Y98	Slovenia	6.41	1
V01.Y98	Spain	77.60	3
V01.Y98	Sweden	8.61	0
V01.Y98	Switzerland	16.05	1
V01.Y98	United Kingdom	36.88	0
V01.Y98	Germany	14.09	1
V01.Y98	Hungary	7.41	2
V01.Y98	Austria	8.06	1
V01.Y98	Belgium	8.33	3
V01.Y98	Croatia	13.13	1
V01.Y98	Czech Republic	14.83	1
V01.Y98	Estonia	7.45	2
V01.Y98	France	34.96	1
V01.Y98	Germany	54.19	1
V01.Y98	Hungary	17.92	0
V01.Y98	Latvia	15.38	3
V01.Y98	Lithuania	30.97	2
V01.Y98	Netherlands	6.28	1

For peer review only

V01.Y98	Poland	146.81	3
V01.Y98	Romania	187.59	1
V01.Y98	Slovenia	7.71	1
V01.Y98	Spain	401.58	2
V01.Y98	Switzerland	11.22	1
V01.Y98	United Kingdom	195.24	1
V01.Y98	Netherlands	5.83	1
V01.Y98	Germany	8.47	2
V01.Y98	Netherlands	6.60	0
V01.Y98	Austria	11.42	1
V01.Y98	Belgium	31.72	3
V01.Y98	Croatia	23.66	1
V01.Y98	Czech Republic	44.09	2
V01.Y98	Denmark	19.80	1
V01.Y98	Estonia	8.77	1
V01.Y98	Finland	16.45	1
V01.Y98	France	15.49	0
V01.Y98	Germany	157.10	1
V01.Y98	Hungary	121.55	2
V01.Y98	Latvia	16.67	2
V01.Y98	Lithuania	40.43	1
V01.Y98	Netherlands	99.48	1
V01.Y98	Poland	361.87	3
V01.Y98	Romania	10.93	1
V01.Y98	Slovenia	8.24	0
V01.Y98	Spain	38.93	2
V01.Y98	Sweden	13.09	2
V01.Y98	Switzerland	9.13	1
V01.Y98	United Kingdom	38.27	1
V01.Y98	Belgium	9.93	1
V01.Y98	Czech Republic	8.89	3
V01.Y98	Denmark	10.32	1
V01.Y98	Finland	6.83	3
V01.Y98	Germany	54.93	0
V01.Y98	Hungary	7.09	0
V01.Y98	Lithuania	8.13	1
V01.Y98	Netherlands	30.30	1
V01.Y98	Poland	10.95	2
V01.Y98	Spain	8.44	0
V01.Y98	United Kingdom	21.32	1
V01.Y98	Germany	7.65	0
V01.Y98	Germany	7.10	0
V01.Y98	Netherlands	5.59	0
V01.Y98	Austria	10.90	0
V01.Y98	Belgium	18.68	2
V01.Y98	Croatia	6.88	0
V01.Y98	Czech Republic	15.91	2
V01.Y98	Denmark	6.29	0
V01.Y98	Finland	16.70	1
V01.Y98	France	28.71	0

For peer review only

V01.Y98	Germany	109.89	0
V01.Y98	Hungary	47.81	2
V01.Y98	Lithuania	5.45	0
V01.Y98	Netherlands	49.69	1
V01.Y98	Poland	27.82	3
V01.Y98	Romania	11.68	0
V01.Y98	Spain	14.86	0
V01.Y98	Sweden	8.22	2
V01.Y98	Switzerland	8.48	3
V01.Y98	United Kingdom	22.90	0
V01.Y98	Austria	10.42	3
V01.Y98	Belgium	10.86	0
V01.Y98	Czech Republic	16.69	1
V01.Y98	France	20.36	0
V01.Y98	Germany	63.13	2
V01.Y98	Hungary	10.22	0
V01.Y98	Lithuania	6.93	1
V01.Y98	Netherlands	7.99	0
V01.Y98	Poland	49.78	3
V01.Y98	Romania	22.77	1
V01.Y98	Spain	16.56	0
V01.Y98	Switzerland	7.52	0
V01.Y98	United Kingdom	34.64	2
V01.Y98	Germany	11.05	1
V01.Y98	Spain	6.55	1
V01.Y98	United Kingdom	9.94	1
V01.Y98	Austria	15.88	0
V01.Y98	Belgium	32.28	1
V01.Y98	Croatia	19.12	2
V01.Y98	Czech Republic	24.99	1
V01.Y98	Denmark	23.20	1
V01.Y98	Finland	10.91	0
V01.Y98	France	63.59	0
V01.Y98	Germany	230.08	2
V01.Y98	Hungary	53.70	3
V01.Y98	Latvia	7.40	0
V01.Y98	Lithuania	8.92	0
V01.Y98	Netherlands	47.59	1
V01.Y98	Norway	9.83	1
V01.Y98	Poland	91.92	3
V01.Y98	Slovenia	10.66	0
V01.Y98	Spain	132.39	1
V01.Y98	Sweden	20.04	2
V01.Y98	Switzerland	14.51	0
V01.Y98	United Kingdom	156.97	3
V01.Y98	France	14.24	0
V01.Y98	Germany	46.78	1
V01.Y98	Netherlands	12.86	1
V01.Y98	Spain	29.16	3
V01.Y98	United Kingdom	28.49	1

For peer review only

V01.Y98	Belgium	9.49	0
V01.Y98	Croatia	5.70	1
V01.Y98	Czech Republic	14.05	0
V01.Y98	Denmark	9.10	0
V01.Y98	France	6.30	0
V01.Y98	Germany	93.05	0
V01.Y98	Hungary	14.60	0
V01.Y98	Lithuania	5.34	0
V01.Y98	Netherlands	27.71	0
V01.Y98	Poland	33.58	2
V01.Y98	Spain	56.43	1
V01.Y98	Sweden	7.76	0
V01.Y98	United Kingdom	85.75	1
V01.Y98	Austria	8.75	0
V01.Y98	Belgium	9.80	1
V01.Y98	Croatia	17.87	2
V01.Y98	Denmark	6.24	1
V01.Y98	Finland	10.58	0
V01.Y98	France	18.36	2
V01.Y98	Germany	76.90	1
V01.Y98	Hungary	15.82	0
V01.Y98	Netherlands	16.21	1
V01.Y98	Norway	8.76	1
V01.Y98	Poland	11.21	0
V01.Y98	Slovenia	6.68	0
V01.Y98	Spain	73.81	1
V01.Y98	Sweden	13.01	0
V01.Y98	Switzerland	5.56	1
V01.Y98	United Kingdom	49.47	1
V01.Y98	Austria	33.02	2
V01.Y98	Belgium	36.92	1
V01.Y98	Croatia	12.83	0
V01.Y98	Czech Republic	13.76	1
V01.Y98	France	262.69	3
V01.Y98	Germany	183.64	3
V01.Y98	Hungary	15.69	0
V01.Y98	Netherlands	10.85	3
V01.Y98	Norway	5.64	1
V01.Y98	Poland	46.80	2
V01.Y98	Romania	9.36	2
V01.Y98	Slovenia	7.49	1
V01.Y98	Spain	87.12	1
V01.Y98	Sweden	5.34	0
V01.Y98	Switzerland	32.38	1
V01.Y98	United Kingdom	123.64	2
V01.Y98	Netherlands	6.71	1
V01.Y98	Netherlands	12.49	1
V01.Y98	United Kingdom	5.94	0
V01.Y98	Czech Republic	11.45	1
V01.Y98	Sweden	5.25	0

For peer review only

V01.Y98	Germany	7.06	0
V01.Y98	Austria	27.75	3
V01.Y98	Belgium	29.59	3
V01.Y98	Croatia	51.18	3
V01.Y98	Czech Republic	75.28	3
V01.Y98	Denmark	61.76	1
V01.Y98	Estonia	23.72	1
V01.Y98	Finland	55.84	1
V01.Y98	France	38.58	2
V01.Y98	Germany	358.08	2
V01.Y98	Hungary	202.24	3
V01.Y98	Latvia	39.32	1
V01.Y98	Lithuania	61.74	3
V01.Y98	Netherlands	68.91	3
V01.Y98	Norway	23.76	1
V01.Y98	Poland	820.12	1
V01.Y98	Romania	31.12	0
V01.Y98	Slovenia	25.84	1
V01.Y98	Spain	521.83	3
V01.Y98	Sweden	59.33	2
V01.Y98	Switzerland	11.54	1
V01.Y98	United Kingdom	240.76	3
V01.Y98	Austria	16.20	3
V01.Y98	Belgium	19.80	1
V01.Y98	Croatia	19.14	0
V01.Y98	Czech Republic	42.45	0
V01.Y98	Denmark	25.18	2
V01.Y98	Estonia	5.38	0
V01.Y98	Finland	39.49	1
V01.Y98	France	33.91	1
V01.Y98	Germany	213.51	2
V01.Y98	Hungary	27.96	0
V01.Y98	Latvia	15.09	0
V01.Y98	Lithuania	35.01	1
V01.Y98	Netherlands	40.84	2
V01.Y98	Norway	27.10	1
V01.Y98	Poland	40.80	3
V01.Y98	Slovenia	11.81	3
V01.Y98	Spain	183.23	3
V01.Y98	Sweden	46.31	1
V01.Y98	Switzerland	6.12	2
V01.Y98	United Kingdom	129.99	3
V01.Y98	Austria	9.89	1
V01.Y98	Croatia	6.20	0
V01.Y98	Czech Republic	14.82	0
V01.Y98	France	10.04	0
V01.Y98	Germany	22.34	1
V01.Y98	Hungary	14.04	2
V01.Y98	Netherlands	5.41	1
V01.Y98	Poland	24.11	0

For peer review only

V01.Y98	Romania	6.26	0
V01.Y98	Spain	7.04	1
V01.Y98	Austria	25.37	0
V01.Y98	Belgium	60.65	2
V01.Y98	Croatia	20.11	1
V01.Y98	Czech Republic	89.74	0
V01.Y98	Denmark	34.68	1
V01.Y98	Estonia	14.69	0
V01.Y98	Finland	10.55	1
V01.Y98	France	23.39	0
V01.Y98	Germany	537.17	2
V01.Y98	Hungary	85.87	3
V01.Y98	Latvia	30.76	1
V01.Y98	Lithuania	53.45	3
V01.Y98	Netherlands	122.82	1
V01.Y98	Poland	386.30	2
V01.Y98	Romania	15.33	0
V01.Y98	Slovenia	21.06	0
V01.Y98	Spain	187.59	3
V01.Y98	Sweden	31.57	1
V01.Y98	Switzerland	12.81	1
V01.Y98	United Kingdom	300.00	3
V01.Y98	Austria	19.52	1
V01.Y98	Belgium	22.77	3
V01.Y98	Croatia	48.09	2
V01.Y98	Czech Republic	13.50	0
V01.Y98	Denmark	15.96	2
V01.Y98	Estonia	12.39	0
V01.Y98	Finland	58.14	1
V01.Y98	France	10.06	0
V01.Y98	Germany	143.65	2
V01.Y98	Hungary	48.49	1
V01.Y98	Latvia	30.24	1
V01.Y98	Lithuania	58.13	3
V01.Y98	Netherlands	42.43	2
V01.Y98	Norway	42.19	1
V01.Y98	Poland	53.93	3
V01.Y98	Romania	29.41	3
V01.Y98	Slovenia	11.98	1
V01.Y98	Spain	371.82	2
V01.Y98	Sweden	49.46	1
V01.Y98	Switzerland	8.68	0
V01.Y98	United Kingdom	118.42	3
V01.Y98	Austria	44.46	1
V01.Y98	Belgium	98.88	1
V01.Y98	Croatia	49.97	1
V01.Y98	Czech Republic	46.46	3
V01.Y98	Denmark	10.66	1
V01.Y98	Estonia	9.36	1
V01.Y98	Finland	5.29	2

For peer review only

V01.Y98	France	221.20	2
V01.Y98	Germany	392.35	1
V01.Y98	Hungary	94.44	2
V01.Y98	Latvia	31.15	2
V01.Y98	Lithuania	23.30	1
V01.Y98	Netherlands	36.24	3
V01.Y98	Norway	30.08	1
V01.Y98	Poland	515.77	2
V01.Y98	Romania	252.70	0
V01.Y98	Slovenia	23.46	3
V01.Y98	Spain	111.85	0
V01.Y98	Sweden	13.48	1
V01.Y98	Switzerland	65.85	2
V01.Y98	United Kingdom	442.97	3
V01.Y98	Hungary	9.68	0
V01.Y98	Poland	15.82	1
V01.Y98	Spain	15.68	2
V01.Y98	United Kingdom	5.70	3
V01.Y98	Germany	10.56	0
V01.Y98	Spain	22.74	1
V01.Y98	United Kingdom	10.08	1
V01.Y98	Germany	7.80	0
V01.Y98	Poland	7.50	2
V01.Y98	Spain	6.50	0
V01.Y98	Spain	17.61	0
V01.Y98	Germany	5.62	1
V01.Y98	Hungary	5.81	0
V01.Y98	Poland	12.96	2
V01.Y98	Spain	6.10	1
V01.Y98	Germany	14.54	0
V01.Y98	Spain	17.41	1
V01.Y98	United Kingdom	10.15	1
V01.Y98	Germany	7.49	0
V01.Y98	Spain	5.21	1
V01.Y98	Germany	9.05	1
V01.Y98	Poland	6.21	0
V01.Y98	Spain	36.68	3
V01.Y98	United Kingdom	9.08	1
V01.Y98	France	7.02	1
V01.Y98	Germany	14.59	1
V01.Y98	Poland	6.04	1
V01.Y98	Spain	7.01	0
V01.Y98	United Kingdom	5.52	1
V01.Y98	Spain	6.75	0
V01.Y98	United Kingdom	11.30	0
V01.Y98	Romania	9.84	3
V01.Y98	France	10.53	1
V01.Y98	Germany	16.83	2
V01.Y98	Hungary	10.19	3
V01.Y98	Lithuania	8.25	3

For peer review only

V01.Y98	Poland	7.50	2
V01.Y98	Romania	100.23	1
V01.Y98	Spain	10.63	1
V01.Y98	United Kingdom	15.11	1
V01.Y98	France	14.65	2
V01.Y98	Germany	23.00	0
V01.Y98	Hungary	6.18	2
V01.Y98	Poland	31.95	1
V01.Y98	Romania	200.42	1
V01.Y98	Spain	17.90	0
V01.Y98	Germany	5.11	0
V01.Y98	Germany	5.51	0
V01.Y98	United Kingdom	7.25	0
V01.Y98	Austria	22.33	2
V01.Y98	Croatia	37.35	0
V01.Y98	Czech Republic	5.79	2
V01.Y98	Finland	7.65	1
V01.Y98	Germany	31.67	1
V01.Y98	Latvia	7.35	2
V01.Y98	Lithuania	6.32	0
V01.Y98	Norway	6.85	0
V01.Y98	Poland	57.89	3
V01.Y98	Romania	27.37	2
V01.Y98	Slovenia	18.80	1
V01.Y98	Spain	94.98	1
V01.Y98	Switzerland	9.53	1
V01.Y98	United Kingdom	13.14	0
V01.Y98	Germany	9.12	0
V01.Y98	Finland	13.20	0
V01.Y98	France	11.00	2
V01.Y98	Germany	6.53	1
V01.Y98	Norway	5.51	0
V01.Y98	Spain	6.14	3
V01.Y98	Sweden	15.18	0
V01.Y98	United Kingdom	9.65	0
V01.Y98	France	9.28	0
V01.Y98	Germany	9.39	0
V01.Y98	Romania	7.50	2
V01.Y98	Spain	9.36	1
V01.Y98	Austria	246.87	2
V01.Y98	Belgium	568.08	3
V01.Y98	Croatia	105.59	2
V01.Y98	Czech Republic	258.01	3
V01.Y98	France	3912.36	2
V01.Y98	Germany	1140.98	2
V01.Y98	Lithuania	5.74	0
V01.Y98	Netherlands	10.40	0
V01.Y98	Norway	24.50	0
V01.Y98	Poland	354.92	3
V01.Y98	Romania	1820.37	2

For peer review only

V01.Y98	Spain	845.00	3
V01.Y98	Sweden	15.79	1
V01.Y98	Switzerland	101.97	2
V01.Y98	United Kingdom	149.91	3
V01.Y98	Finland	25.77	2
V01.Y98	Norway	5.24	0
V01.Y98	Spain	15.61	1
V01.Y98	United Kingdom	9.31	0
V01.Y98	Finland	26.02	1
V01.Y98	Norway	18.69	0
V01.Y98	Sweden	15.32	3
V01.Y98	United Kingdom	11.65	0
V01.Y98	United Kingdom	5.67	1
V01.Y98	Austria	5.16	0
V01.Y98	Czech Republic	5.82	0
V01.Y98	France	65.86	3
V01.Y98	Germany	30.62	0
V01.Y98	Netherlands	7.18	0
V01.Y98	Poland	10.41	0
V01.Y98	Spain	32.06	0
V01.Y98	Sweden	5.97	1
V01.Y98	Switzerland	12.71	0
V01.Y98	United Kingdom	24.38	0
V01.Y98	France	16.64	0
V01.Y98	Germany	9.77	0
V01.Y98	Spain	6.85	0
V01.Y98	Switzerland	7.82	0
V01.Y98	Germany	5.72	0
V01.Y98	Czech Republic	11.04	3
V01.Y98	Estonia	5.19	3
V01.Y98	Germany	84.15	2
V01.Y98	Hungary	12.68	0
V01.Y98	Lithuania	5.77	2
V01.Y98	Poland	355.74	3
V01.Y98	Romania	93.41	2
V01.Y98	Finland	11.87	1
V01.Y98	Germany	21.64	0
V01.Y98	Latvia	12.33	3
V01.Y98	Poland	12.94	1
V01.Y98	Sweden	5.22	0
V01.Y98	United Kingdom	13.69	0
V01.Y98	Austria	279.00	0
V01.Y98	Belgium	6.03	0
V01.Y98	Croatia	132.76	1
V01.Y98	Czech Republic	564.98	2
V01.Y98	Denmark	68.01	3
V01.Y98	Estonia	13.04	1
V01.Y98	Finland	793.81	2
V01.Y98	France	33.22	2
V01.Y98	Germany	369.55	2

For peer review only

V01.Y98	Hungary	921.67	2
V01.Y98	Latvia	47.27	1
V01.Y98	Lithuania	67.00	1
V01.Y98	Netherlands	77.45	2
V01.Y98	Norway	6.63	0
V01.Y98	Poland	2609.65	2
V01.Y98	Romania	82.91	0
V01.Y98	Slovenia	251.75	3
V01.Y98	Spain	18.50	2
V01.Y98	Sweden	25.85	1
V01.Y98	Switzerland	38.45	1
V01.Y98	United Kingdom	166.18	3
V01.Y98	Austria	10.65	1
V01.Y98	France	15.97	2
V01.Y98	Germany	6.24	0
V01.Y98	Switzerland	8.63	0
V01.Y98	Poland	12.11	0
V01.Y98	United Kingdom	7.51	0
V01.Y98	Germany	6.29	0
V01.Y98	Austria	5.55	0
V01.Y98	Denmark	5.13	1
V01.Y98	Finland	8.15	0
V01.Y98	Germany	90.25	1
V01.Y98	Hungary	6.12	1
V01.Y98	Netherlands	18.14	3
V01.Y98	Norway	9.03	1
V01.Y98	Spain	6.99	2
V01.Y98	Sweden	12.71	0
V01.Y98	Switzerland	5.64	0
V01.Y98	United Kingdom	18.12	1
V01.Y98	Austria	12.40	3
V01.Y98	Belgium	19.28	1
V01.Y98	Croatia	33.27	1
V01.Y98	Czech Republic	7.47	0
V01.Y98	Denmark	13.90	3
V01.Y98	Finland	46.33	1
V01.Y98	France	53.29	1
V01.Y98	Germany	300.12	0
V01.Y98	Hungary	145.37	2
V01.Y98	Latvia	7.07	0
V01.Y98	Lithuania	8.23	1
V01.Y98	Netherlands	131.70	1
V01.Y98	Norway	16.82	0
V01.Y98	Poland	36.71	1
V01.Y98	Slovenia	16.85	0
V01.Y98	Spain	31.93	2
V01.Y98	Sweden	20.08	0
V01.Y98	Switzerland	13.76	0
V01.Y98	United Kingdom	126.68	3
V01.Y98	Croatia	5.30	0

For peer review only

V01.Y98	Finland	16.68	0
V01.Y98	France	5.56	0
V01.Y98	Germany	53.67	1
V01.Y98	Hungary	24.87	0
V01.Y98	Netherlands	29.94	1
V01.Y98	Norway	6.22	2
V01.Y98	Poland	14.78	1
V01.Y98	Spain	6.95	2
V01.Y98	Sweden	5.86	0
V01.Y98	United Kingdom	61.65	0
V01.Y98	Belgium	5.92	0
V01.Y98	France	7.50	0
V01.Y98	Germany	48.22	0
V01.Y98	Netherlands	54.33	3
V01.Y98	United Kingdom	6.05	0
V01.Y98	Poland	7.85	2
V01.Y98	Austria	71.80	1
V01.Y98	Belgium	235.90	2
V01.Y98	Croatia	50.97	2
V01.Y98	Czech Republic	71.73	0
V01.Y98	Denmark	37.26	0
V01.Y98	Estonia	17.64	1
V01.Y98	Finland	88.72	1
V01.Y98	France	405.10	1
V01.Y98	Germany	1107.65	0
V01.Y98	Hungary	123.09	2
V01.Y98	Latvia	38.91	1
V01.Y98	Lithuania	91.55	1
V01.Y98	Netherlands	257.80	2
V01.Y98	Norway	58.20	2
V01.Y98	Poland	465.75	2
V01.Y98	Slovenia	43.59	0
V01.Y98	Spain	160.03	2
V01.Y98	Sweden	81.91	3
V01.Y98	Switzerland	95.24	1
V01.Y98	United Kingdom	806.04	3
V01.Y98	Austria	15.01	0
V01.Y98	Belgium	18.57	0
V01.Y98	Croatia	10.91	1
V01.Y98	Czech Republic	39.33	2
V01.Y98	Denmark	5.04	0
V01.Y98	Finland	7.08	0
V01.Y98	France	51.67	0
V01.Y98	Germany	122.62	1
V01.Y98	Hungary	37.65	1
V01.Y98	Netherlands	10.91	0
V01.Y98	Norway	5.10	0
V01.Y98	Poland	51.59	1
V01.Y98	Romania	37.86	0
V01.Y98	Slovenia	5.45	0

For peer review only

V01.Y98	Spain	17.33	0
V01.Y98	Sweden	7.37	0
V01.Y98	Switzerland	8.86	0
V01.Y98	United Kingdom	69.10	1
V01.Y98	Czech Republic	5.11	2
V01.Y98	France	8.58	1
V01.Y98	Germany	23.51	1
V01.Y98	Hungary	6.68	2
V01.Y98	Poland	27.64	0
V01.Y98	Spain	20.83	3
V01.Y98	United Kingdom	6.78	1
V01.Y98	Austria	22.96	1
V01.Y98	Belgium	39.32	1
V01.Y98	Croatia	36.04	0
V01.Y98	Czech Republic	59.72	0
V01.Y98	Denmark	13.44	1
V01.Y98	Estonia	23.12	0
V01.Y98	Finland	19.03	1
V01.Y98	France	111.87	1
V01.Y98	Germany	271.76	1
V01.Y98	Hungary	67.31	1
V01.Y98	Latvia	26.63	1
V01.Y98	Lithuania	36.35	1
V01.Y98	Netherlands	27.04	0
V01.Y98	Norway	10.89	3
V01.Y98	Poland	229.23	0
V01.Y98	Romania	81.57	3
V01.Y98	Slovenia	13.36	1
V01.Y98	Spain	119.09	1
V01.Y98	Sweden	16.57	0
V01.Y98	Switzerland	18.15	0
V01.Y98	United Kingdom	107.32	0
V01.Y98	Austria	7.47	1
V01.Y98	Croatia	7.51	0
V01.Y98	Czech Republic	7.43	0
V01.Y98	France	17.78	1
V01.Y98	Germany	30.10	1
V01.Y98	Hungary	14.15	0
V01.Y98	Poland	17.60	1
V01.Y98	Romania	18.11	0
V01.Y98	Spain	13.46	0
V01.Y98	Austria	43.93	1
V01.Y98	France	14.21	0
V01.Y98	Germany	26.90	3
V01.Y98	Norway	7.77	0
V01.Y98	Poland	5.42	0
V01.Y98	Slovenia	11.01	0
V01.Y98	Spain	34.54	0
V01.Y98	Switzerland	42.59	1
V01.Y98	United Kingdom	27.26	0

For peer review only

V01.Y98	France	10.33	1
V01.Y98	Poland	7.08	0
V01.Y98	Austria	30.22	1
V01.Y98	Belgium	15.31	1
V01.Y98	Croatia	19.30	0
V01.Y98	Czech Republic	38.57	2
V01.Y98	Denmark	11.31	1
V01.Y98	Finland	6.23	0
V01.Y98	France	198.68	2
V01.Y98	Germany	111.87	0
V01.Y98	Hungary	22.79	0
V01.Y98	Latvia	20.91	0
V01.Y98	Lithuania	15.86	1
V01.Y98	Netherlands	20.82	0
V01.Y98	Norway	8.53	0
V01.Y98	Poland	188.75	1
V01.Y98	Romania	294.93	2
V01.Y98	Slovenia	18.46	2
V01.Y98	Spain	174.01	1
V01.Y98	Sweden	6.97	0
V01.Y98	Switzerland	34.83	3
V01.Y98	United Kingdom	31.51	0
V01.Y98	Belgium	14.56	0
V01.Y98	Croatia	198.21	1
V01.Y98	Czech Republic	36.52	0
V01.Y98	Denmark	80.85	3
V01.Y98	Estonia	22.67	2
V01.Y98	Finland	211.56	2
V01.Y98	France	430.01	3
V01.Y98	Germany	314.32	3
V01.Y98	Hungary	407.89	2
V01.Y98	Latvia	62.19	1
V01.Y98	Lithuania	68.14	0
V01.Y98	Netherlands	60.43	3
V01.Y98	Norway	101.82	3
V01.Y98	Poland	149.55	3
V01.Y98	Romania	117.54	2
V01.Y98	Slovenia	7.90	0
V01.Y98	Spain	51.28	2
V01.Y98	Sweden	72.38	3
V01.Y98	Switzerland	31.83	3
V01.Y98	United Kingdom	20.28	0
V01.Y98	Austria	531.49	2
V01.Y98	Belgium	1030.74	1
V01.Y98	Croatia	725.31	1
V01.Y98	Czech Republic	1059.20	1
V01.Y98	Denmark	214.91	1
V01.Y98	Estonia	39.96	1
V01.Y98	Finland	137.59	0
V01.Y98	France	4843.32	2

For peer review only

V01.Y98	Germany	6597.94	3
V01.Y98	Hungary	1169.31	3
V01.Y98	Latvia	73.52	2
V01.Y98	Lithuania	172.68	0
V01.Y98	Netherlands	1296.72	3
V01.Y98	Norway	410.67	2
V01.Y98	Poland	2124.08	1
V01.Y98	Romania	947.00	1
V01.Y98	Slovenia	210.13	1
V01.Y98	Spain	1492.20	2
V01.Y98	Sweden	560.82	2
V01.Y98	Switzerland	1189.50	2
V01.Y98	United Kingdom	3385.43	3
V01.Y98	Austria	32.23	0
V01.Y98	Belgium	13.76	0
V01.Y98	Croatia	18.51	0
V01.Y98	Czech Republic	21.72	0
V01.Y98	Denmark	6.53	0
V01.Y98	Estonia	5.15	1
V01.Y98	Finland	12.77	0
V01.Y98	France	67.07	3
V01.Y98	Germany	111.69	1
V01.Y98	Hungary	26.69	0
V01.Y98	Latvia	25.29	0
V01.Y98	Lithuania	22.09	0
V01.Y98	Netherlands	16.25	0
V01.Y98	Norway	7.84	0
V01.Y98	Poland	75.03	1
V01.Y98	Romania	78.40	0
V01.Y98	Slovenia	14.31	3
V01.Y98	Spain	62.23	3
V01.Y98	Sweden	20.16	1
V01.Y98	Switzerland	16.46	0
V01.Y98	United Kingdom	46.68	1
V01.Y98	Germany	11.10	0
V01.Y98	Hungary	7.18	0
V01.Y98	Poland	14.51	1
V01.Y98	Romania	6.36	2
V01.Y98	Spain	5.95	0
V01.Y98	United Kingdom	16.15	0
V01.Y98	Austria	13.04	3
V01.Y98	Belgium	12.71	1
V01.Y98	Czech Republic	9.53	1
V01.Y98	Denmark	6.86	0
V01.Y98	Estonia	6.17	0
V01.Y98	France	30.82	0
V01.Y98	Germany	52.74	0
V01.Y98	Hungary	9.80	1
V01.Y98	Latvia	5.69	3
V01.Y98	Lithuania	5.53	0

For peer review only

V01.Y98	Netherlands	15.49	1
V01.Y98	Norway	10.37	3
V01.Y98	Poland	39.82	0
V01.Y98	Romania	137.04	3
V01.Y98	Spain	15.92	0
V01.Y98	Sweden	6.83	0
V01.Y98	United Kingdom	10.71	0
V01.Y98	Finland	5.20	1
V01.Y98	Germany	15.59	0
V01.Y98	Poland	7.01	0
V01.Y98	Spain	13.27	3
V01.Y98	United Kingdom	7.69	0
V01.Y98	Poland	5.64	0
V01.Y98	Spain	7.81	0
V01.Y98	France	9.31	0
V01.Y98	Germany	7.20	1
V01.Y98	Poland	6.53	2
V01.Y98	Romania	6.74	0
V01.Y98	Belgium	5.16	2
V01.Y98	Croatia	7.29	0
V01.Y98	France	93.79	1
V01.Y98	Germany	14.38	0
V01.Y98	Hungary	7.14	0
V01.Y98	Poland	28.25	0
V01.Y98	Spain	14.80	1
V01.Y98	France	14.04	1
V01.Y98	Germany	19.20	1
V01.Y98	Poland	17.55	3
V01.Y98	Spain	24.41	1
V01.Y98	Sweden	6.38	0
V01.Y98	United Kingdom	12.50	1
V01.Y98	France	15.38	1
V01.Y98	Spain	5.78	0
V01.Y98	France	19.44	0
V01.Y98	Germany	7.24	1
V01.Y98	Romania	9.41	1
V01.Y98	Spain	21.03	1
V01.Y98	Poland	9.27	1
V01.Y98	Belgium	6.35	0
V01.Y98	France	15.95	1
V01.Y98	Germany	12.25	1
V01.Y98	Hungary	5.96	0
V01.Y98	Poland	11.72	0
V01.Y98	Romania	10.97	3
V01.Y98	Spain	13.20	2
V01.Y98	France	6.59	0
V01.Y98	United Kingdom	8.59	0
V01.Y98	Germany	5.35	0
V01.Y98	Poland	55.57	0
V01.Y98	Romania	6.80	0

For peer review only

V01.Y98	United Kingdom	10.09	1
V01.Y98	Poland	29.84	0
V01.Y98	Hungary	5.61	0
V01.Y98	Poland	5.46	1
V01.Y98	France	16.37	1
V01.Y98	Germany	14.50	1
V01.Y98	Lithuania	6.74	1
V01.Y98	Netherlands	5.42	0
V01.Y98	Poland	22.91	1
V01.Y98	Romania	31.56	1
V01.Y98	Spain	15.73	2
V01.Y98	United Kingdom	11.39	0
V01.Y98	Romania	10.06	0
V01.Y98	Austria	6.10	1
V01.Y98	Belgium	6.36	1
V01.Y98	Czech Republic	10.94	0
V01.Y98	France	16.56	1
V01.Y98	Germany	46.94	1
V01.Y98	Hungary	7.74	0
V01.Y98	Lithuania	12.69	1
V01.Y98	Netherlands	6.13	0
V01.Y98	Poland	21.98	1
V01.Y98	Sweden	10.73	1
V01.Y98	United Kingdom	32.07	0
V01.Y98	Germany	8.27	0
V01.Y98	France	20.36	1
V01.Y98	Germany	16.43	0
V01.Y98	Spain	20.43	0
V01.Y98	United Kingdom	5.84	0
V01.Y98	Poland	7.59	0
V01.Y98	Spain	11.94	0
V01.Y98	Austria	22.72	2
V01.Y98	Belgium	8.83	0
V01.Y98	Croatia	60.90	1
V01.Y98	Czech Republic	23.14	0
V01.Y98	Denmark	20.12	0
V01.Y98	Estonia	31.38	1
V01.Y98	Finland	52.01	1
V01.Y98	France	99.38	2
V01.Y98	Germany	108.69	2
V01.Y98	Hungary	100.99	1
V01.Y98	Latvia	98.86	1
V01.Y98	Lithuania	203.12	1
V01.Y98	Netherlands	5.75	0
V01.Y98	Norway	6.61	0
V01.Y98	Poland	509.82	3
V01.Y98	Romania	415.30	1
V01.Y98	Slovenia	13.83	0
V01.Y98	Spain	120.62	1
V01.Y98	Sweden	18.42	2

For peer review only

V01.Y98	Switzerland	5.91	0
V01.Y98	United Kingdom	68.37	0
V01.Y98	Austria	14.53	0
V01.Y98	Belgium	10.30	2
V01.Y98	Czech Republic	53.18	0
V01.Y98	Denmark	6.42	1
V01.Y98	Estonia	8.74	3
V01.Y98	Finland	65.25	3
V01.Y98	France	27.48	0
V01.Y98	Germany	40.98	0
V01.Y98	Hungary	15.11	0
V01.Y98	Lithuania	66.72	0
V01.Y98	Netherlands	22.06	2
V01.Y98	Norway	7.18	3
V01.Y98	Poland	150.40	2
V01.Y98	Romania	27.54	3
V01.Y98	Slovenia	5.44	0
V01.Y98	Spain	31.65	0
V01.Y98	Sweden	16.65	1
V01.Y98	United Kingdom	36.73	0
V01.Y98	Belgium	5.59	2
V01.Y98	Czech Republic	7.78	1
V01.Y98	Estonia	13.07	1
V01.Y98	Finland	6.12	2
V01.Y98	France	10.95	0
V01.Y98	Germany	28.79	1
V01.Y98	Hungary	24.61	1
V01.Y98	Latvia	69.82	3
V01.Y98	Lithuania	13.71	1
V01.Y98	Poland	49.52	1
V01.Y98	Romania	23.48	0
V01.Y98	Spain	16.41	1
V01.Y98	United Kingdom	28.69	0
V01.Y98	Austria	13.78	1
V01.Y98	Belgium	41.12	0
V01.Y98	Croatia	25.09	1
V01.Y98	Czech Republic	93.74	1
V01.Y98	Denmark	7.32	0
V01.Y98	Estonia	14.07	1
V01.Y98	France	882.21	1
V01.Y98	Germany	164.45	0
V01.Y98	Hungary	16.40	1
V01.Y98	Latvia	22.13	3
V01.Y98	Lithuania	13.23	0
V01.Y98	Netherlands	49.45	2
V01.Y98	Norway	49.68	1
V01.Y98	Poland	203.41	3
V01.Y98	Romania	482.75	3
V01.Y98	Spain	309.42	1
V01.Y98	Sweden	50.06	0

For peer review only

V01.Y98	Switzerland	35.94	0
V01.Y98	United Kingdom	59.34	1
V01.Y98	Belgium	11.80	1
V01.Y98	France	13.94	0
V01.Y98	Germany	15.43	0
V01.Y98	Netherlands	16.21	0
V01.Y98	Poland	15.11	1
V01.Y98	United Kingdom	15.78	2
V01.Y98	Belgium	6.05	1
V01.Y98	Finland	5.94	0
V01.Y98	France	21.56	0
V01.Y98	Germany	30.64	0
V01.Y98	Netherlands	10.55	0
V01.Y98	Poland	21.81	1
V01.Y98	Romania	7.86	2
V01.Y98	Spain	10.39	3
V01.Y98	Switzerland	5.04	0
V01.Y98	United Kingdom	148.13	0
V01.Y98	Czech Republic	10.22	0
V01.Y98	France	13.98	0
V01.Y98	Germany	8.79	1
V01.Y98	Hungary	5.08	0
V01.Y98	Poland	26.06	1
V01.Y98	Spain	9.05	1
V01.Y98	Austria	44.98	3
V01.Y98	Belgium	52.06	0
V01.Y98	Croatia	8.56	1
V01.Y98	Czech Republic	209.53	2
V01.Y98	Denmark	8.99	1
V01.Y98	Estonia	56.35	3
V01.Y98	Finland	14.44	3
V01.Y98	France	178.52	2
V01.Y98	Germany	556.89	3
V01.Y98	Hungary	123.58	2
V01.Y98	Latvia	18.15	1
V01.Y98	Lithuania	96.89	1
V01.Y98	Netherlands	9.67	0
V01.Y98	Norway	5.55	0
V01.Y98	Poland	237.04	2
V01.Y98	Romania	270.79	1
V01.Y98	Slovenia	7.71	2
V01.Y98	Spain	93.20	2
V01.Y98	Sweden	9.96	0
V01.Y98	Switzerland	24.07	3
V01.Y98	United Kingdom	129.10	1
V01.Y98	Austria	55.99	1
V01.Y98	Belgium	266.75	2
V01.Y98	Croatia	34.69	0
V01.Y98	Czech Republic	38.30	2
V01.Y98	Denmark	27.98	1

For peer review only

V01.Y98	Estonia	20.05	1
V01.Y98	Finland	69.98	1
V01.Y98	France	2820.38	3
V01.Y98	Germany	538.70	2
V01.Y98	Hungary	139.78	1
V01.Y98	Latvia	34.80	2
V01.Y98	Lithuania	103.68	3
V01.Y98	Netherlands	62.94	0
V01.Y98	Norway	13.85	0
V01.Y98	Poland	368.48	2
V01.Y98	Romania	316.58	1
V01.Y98	Slovenia	24.73	1
V01.Y98	Spain	166.13	1
V01.Y98	Sweden	36.27	1
V01.Y98	Switzerland	67.33	0
V01.Y98	United Kingdom	227.03	0
V01.Y98	Belgium	19.76	0
V01.Y98	Czech Republic	16.58	1
V01.Y98	France	77.21	3
V01.Y98	Germany	84.91	2
V01.Y98	Hungary	26.78	0
V01.Y98	Latvia	10.48	3
V01.Y98	Netherlands	6.18	0
V01.Y98	Norway	22.67	2
V01.Y98	Poland	36.88	1
V01.Y98	Romania	33.44	1
V01.Y98	Spain	777.35	1
V01.Y98	Sweden	31.09	0
V01.Y98	Switzerland	25.13	0
V01.Y98	United Kingdom	37.86	0
V01.Y98	Czech Republic	5.60	0
V01.Y98	France	7.27	0
V01.Y98	Germany	23.57	0
V01.Y98	United Kingdom	7.47	0
V01.Y98	Belgium	114.75	1
V01.Y98	Czech Republic	8.55	0
V01.Y98	Denmark	5.10	0
V01.Y98	France	31.98	0
V01.Y98	Germany	208.18	2
V01.Y98	Hungary	5.35	2
V01.Y98	Netherlands	44.59	2
V01.Y98	Norway	7.31	2
V01.Y98	Poland	20.83	1
V01.Y98	Romania	21.94	1
V01.Y98	Spain	728.81	3
V01.Y98	Switzerland	5.00	1
V01.Y98	United Kingdom	62.23	1
V01.Y98	Czech Republic	6.44	1
V01.Y98	Germany	11.27	1
V01.Y98	Hungary	6.78	1

For peer review only

V01.Y98	Lithuania	12.12	1
V01.Y98	Poland	10.44	2
V01.Y98	Romania	13.13	1
V01.Y98	Germany	14.94	2
V01.Y98	Hungary	11.60	1
V01.Y98	Lithuania	5.06	1
V01.Y98	Poland	37.56	0
V01.Y98	United Kingdom	14.82	1
V01.Y98	Belgium	6.24	0
V01.Y98	Croatia	10.14	3
V01.Y98	Czech Republic	15.75	1
V01.Y98	France	48.62	1
V01.Y98	Germany	27.39	1
V01.Y98	Hungary	21.45	3
V01.Y98	Lithuania	5.02	0
V01.Y98	Poland	77.38	1
V01.Y98	Romania	181.31	1
V01.Y98	Spain	50.44	1
V01.Y98	United Kingdom	9.58	3
V01.Y98	Romania	7.41	2
V01.Y98	Finland	40.38	0
V01.Y98	Germany	5.10	1
V01.Y98	Poland	15.37	1
V01.Y98	Romania	13.92	0
V01.Y98	Austria	26.40	0
V01.Y98	Belgium	63.38	1
V01.Y98	Croatia	14.33	0
V01.Y98	Czech Republic	12.10	2
V01.Y98	Denmark	19.45	0
V01.Y98	Estonia	88.97	3
V01.Y98	Finland	66.88	0
V01.Y98	France	139.77	3
V01.Y98	Germany	227.80	1
V01.Y98	Hungary	61.49	2
V01.Y98	Latvia	140.85	1
V01.Y98	Lithuania	73.06	1
V01.Y98	Netherlands	31.77	0
V01.Y98	Norway	48.37	2
V01.Y98	Poland	359.48	2
V01.Y98	Romania	19.47	0
V01.Y98	Slovenia	5.60	3
V01.Y98	Spain	53.81	3
V01.Y98	Sweden	65.40	3
V01.Y98	Switzerland	12.15	2
V01.Y98	United Kingdom	261.50	1
V01.Y98	Poland	10.90	0
V01.Y98	Croatia	5.37	0
V01.Y98	France	6.80	1
V01.Y98	Germany	9.57	0
V01.Y98	Hungary	12.13	3

For peer review only

V01.Y98	Poland	27.55	3
V01.Y98	Romania	28.82	1
V01.Y98	Spain	8.75	1
V01.Y98	United Kingdom	7.18	0
V01.Y98	Hungary	5.13	0
V01.Y98	Germany	5.61	2
V01.Y98	Poland	11.65	1
V01.Y98	United Kingdom	18.64	1
V01.Y98	Hungary	6.36	2
V01.Y98	Germany	7.55	0
V01.Y98	Hungary	25.48	1
V01.Y98	Latvia	5.76	1
V01.Y98	Poland	20.67	0
V01.Y98	United Kingdom	18.16	0
V01.Y98	Denmark	27.31	0
V01.Y98	Germany	20.02	1
V01.Y98	Hungary	11.93	0
V01.Y98	Latvia	8.66	0
V01.Y98	Lithuania	11.91	0
V01.Y98	Poland	23.80	1
V01.Y98	Romania	63.66	0
V01.Y98	United Kingdom	19.11	0
V01.Y98	Belgium	22.65	1
V01.Y98	Croatia	22.93	3
V01.Y98	Czech Republic	39.88	1
V01.Y98	Denmark	13.33	0
V01.Y98	Estonia	7.99	2
V01.Y98	France	334.22	3
V01.Y98	Germany	143.28	1
V01.Y98	Hungary	43.40	1
V01.Y98	Latvia	11.88	1
V01.Y98	Lithuania	18.94	2
V01.Y98	Netherlands	11.52	1
V01.Y98	Poland	139.66	1
V01.Y98	Romania	365.66	1
V01.Y98	Spain	119.63	1
V01.Y98	Sweden	5.72	0
V01.Y98	Switzerland	14.19	0
V01.Y98	United Kingdom	33.54	2
V01.Y98	France	5.31	1
V01.Y98	Germany	14.10	1
V01.Y98	Hungary	14.61	1
V01.Y98	United Kingdom	22.90	0
V01.Y98	Hungary	5.14	0
V01.Y98	Poland	5.59	0
V01.Y98	Romania	19.55	0
V01.Y98	United Kingdom	6.35	2
V01.Y98	Czech Republic	7.73	1
V01.Y98	Germany	8.68	1
V01.Y98	Poland	52.42	1

For peer review only

V01.Y98	Romania	26.12	1
V01.Y98	France	15.47	0
V01.Y98	Germany	17.57	0
V01.Y98	Hungary	6.01	1
V01.Y98	Poland	7.86	0
V01.Y98	Romania	8.74	0
V01.Y98	Belgium	19.96	0
V01.Y98	France	192.36	0
V01.Y98	Germany	13.33	0
V01.Y98	Poland	6.81	0
V01.Y98	Romania	5.65	0
V01.Y98	Spain	46.43	0
V01.Y98	Austria	31.12	2
V01.Y98	Belgium	15.09	0
V01.Y98	Croatia	30.45	0
V01.Y98	Czech Republic	125.84	2
V01.Y98	Denmark	13.52	0
V01.Y98	Estonia	110.87	2
V01.Y98	Finland	75.26	2
V01.Y98	France	168.94	1
V01.Y98	Germany	119.32	1
V01.Y98	Hungary	251.78	0
V01.Y98	Latvia	209.94	1
V01.Y98	Lithuania	338.59	2
V01.Y98	Netherlands	7.42	0
V01.Y98	Norway	21.84	0
V01.Y98	Poland	421.03	0
V01.Y98	Romania	409.72	1
V01.Y98	Slovenia	22.91	1
V01.Y98	Spain	21.23	1
V01.Y98	Sweden	45.23	1
V01.Y98	Switzerland	6.73	1
V01.Y98	United Kingdom	140.59	3
V01.Y98	Poland	7.08	2
V01.Y98	Romania	38.50	1
V01.Y98	Austria	13.11	0
V01.Y98	France	22.60	0
V01.Y98	Norway	5.72	0
V01.Y98	Switzerland	13.42	3
V01.Y98	Denmark	6.23	0
V01.Y98	Finland	17.92	2
V01.Y98	France	16.91	1
V01.Y98	Germany	5.70	0
V01.Y98	Romania	6.64	0
V01.Y98	Sweden	5.75	2
V01.Y98	United Kingdom	64.78	0
V01.Y98	Belgium	14.08	0
V01.Y98	Czech Republic	25.73	2
V01.Y98	Denmark	12.84	0
V01.Y98	Estonia	6.60	1

For peer review only

V01.Y98	Finland	105.72	2
V01.Y98	France	77.96	1
V01.Y98	Germany	39.13	0
V01.Y98	Latvia	6.29	0
V01.Y98	Lithuania	7.61	2
V01.Y98	Netherlands	7.51	3
V01.Y98	Norway	41.04	0
V01.Y98	Poland	16.00	1
V01.Y98	Romania	11.44	2
V01.Y98	Spain	35.51	0
V01.Y98	Sweden	49.03	1
V01.Y98	Switzerland	8.76	0
V01.Y98	United Kingdom	205.39	2
V01.Y98	Belgium	48.88	2
V01.Y98	Croatia	43.83	2
V01.Y98	Czech Republic	10.39	0
V01.Y98	Denmark	82.22	0
V01.Y98	Estonia	72.87	1
V01.Y98	Finland	96.52	3
V01.Y98	France	119.27	1
V01.Y98	Germany	256.10	1
V01.Y98	Hungary	17.22	0
V01.Y98	Latvia	12.46	2
V01.Y98	Lithuania	44.43	3
V01.Y98	Netherlands	74.50	0
V01.Y98	Norway	144.53	3
V01.Y98	Poland	29.02	2
V01.Y98	Slovenia	13.27	1
V01.Y98	Spain	157.26	3
V01.Y98	Sweden	101.26	2
V01.Y98	Switzerland	52.35	2
V01.Y98	United Kingdom	813.54	3
V01.Y98	Finland	5.40	2
V01.Y98	France	12.71	1
V01.Y98	United Kingdom	6.22	2
V01.Y98	Austria	7.68	2
V01.Y98	Belgium	51.25	0
V01.Y98	Croatia	11.98	3
V01.Y98	Czech Republic	29.82	2
V01.Y98	Denmark	50.34	1
V01.Y98	Finland	17.95	2
V01.Y98	France	829.25	3
V01.Y98	Germany	175.14	3
V01.Y98	Hungary	6.38	0
V01.Y98	Latvia	7.01	3
V01.Y98	Lithuania	6.04	0
V01.Y98	Netherlands	21.23	0
V01.Y98	Norway	63.24	0
V01.Y98	Poland	42.37	1
V01.Y98	Romania	27.65	0

For peer review only

V01.Y98	Spain	309.34	1
V01.Y98	Sweden	86.53	2
V01.Y98	Switzerland	26.76	3
V01.Y98	United Kingdom	367.64	3
V01.Y98	Belgium	23.33	1
V01.Y98	Croatia	17.65	2
V01.Y98	Czech Republic	171.12	1
V01.Y98	Denmark	12.63	0
V01.Y98	Estonia	159.53	2
V01.Y98	Finland	416.69	2
V01.Y98	France	181.98	3
V01.Y98	Germany	137.35	1
V01.Y98	Hungary	23.73	0
V01.Y98	Latvia	173.66	3
V01.Y98	Lithuania	383.90	3
V01.Y98	Netherlands	15.19	0
V01.Y98	Norway	40.24	0
V01.Y98	Poland	1210.37	2
V01.Y98	Romania	321.12	2
V01.Y98	Slovenia	17.83	3
V01.Y98	Spain	40.06	2
V01.Y98	Sweden	114.62	2
V01.Y98	Switzerland	11.17	1
V01.Y98	United Kingdom	319.10	1
V01.Y98	Czech Republic	7.75	2
V01.Y98	Finland	6.67	1
V01.Y98	France	5.10	2
V01.Y98	Hungary	8.83	0
V01.Y98	Latvia	6.82	0
V01.Y98	Poland	14.76	1
V01.Y98	Romania	12.29	0
V01.Y98	United Kingdom	5.80	1
V01.Y98	Austria	14.70	2
V01.Y98	Belgium	34.65	1
V01.Y98	Croatia	22.99	1
V01.Y98	Czech Republic	79.62	3
V01.Y98	Estonia	8.74	2
V01.Y98	Finland	10.84	0
V01.Y98	France	100.52	1
V01.Y98	Germany	49.94	1
V01.Y98	Hungary	63.27	3
V01.Y98	Latvia	39.87	2
V01.Y98	Lithuania	150.13	2
V01.Y98	Netherlands	11.22	0
V01.Y98	Poland	279.75	2
V01.Y98	Romania	491.00	2
V01.Y98	Slovenia	7.40	1
V01.Y98	Spain	82.15	1
V01.Y98	Sweden	5.43	0
V01.Y98	Switzerland	5.60	0

For peer review only

V01.Y98	United Kingdom	50.48	0
V01.Y98	Romania	33.57	1
V01.Y98	Spain	9.17	1
V01.Y98	Belgium	10.77	0
V01.Y98	Czech Republic	10.89	2
V01.Y98	Estonia	5.17	3
V01.Y98	France	104.64	2
V01.Y98	Germany	39.90	1
V01.Y98	Hungary	9.00	1
V01.Y98	Latvia	24.24	2
V01.Y98	Lithuania	18.32	1
V01.Y98	Poland	34.40	1
V01.Y98	Romania	172.62	2
V01.Y98	Spain	107.89	2
V01.Y98	United Kingdom	33.49	0
V01.Y98	Belgium	13.20	0
V01.Y98	France	238.84	1
V01.Y98	Germany	21.91	3
V01.Y98	France	8.08	0
V01.Y98	Germany	14.35	1
V01.Y98	Germany	9.85	0
V01.Y98	Romania	9.17	1
V01.Y98	Austria	630.68	2
V01.Y98	Belgium	922.66	0
V01.Y98	Croatia	113.70	0
V01.Y98	Czech Republic	921.32	1
V01.Y98	Denmark	583.88	1
V01.Y98	Estonia	29.94	3
V01.Y98	Finland	30.43	1
V01.Y98	France	8813.06	2
V01.Y98	Germany	1576.29	2
V01.Y98	Hungary	100.83	0
V01.Y98	Latvia	111.65	3
V01.Y98	Lithuania	62.39	1
V01.Y98	Netherlands	1084.27	3
V01.Y98	Norway	536.20	2
V01.Y98	Poland	968.34	3
V01.Y98	Romania	429.61	0
V01.Y98	Slovenia	6.25	0
V01.Y98	Spain	1957.12	0
V01.Y98	Sweden	929.09	1
V01.Y98	Switzerland	111.33	1
V01.Y98	United Kingdom	3844.82	2
V01.Y98	Czech Republic	5.18	0
V01.Y98	Denmark	17.21	1
V01.Y98	Finland	12.08	2
V01.Y98	France	8.87	0
V01.Y98	Germany	19.27	0
V01.Y98	Hungary	6.19	0
V01.Y98	Netherlands	9.41	0

For peer review only

V01.Y98	Norway	5.06	0
V01.Y98	Sweden	10.87	2
V01.Y98	United Kingdom	84.69	1
V01.Y98	Austria	12.99	0
V01.Y98	Belgium	51.29	3
V01.Y98	Croatia	11.17	0
V01.Y98	Czech Republic	43.91	0
V01.Y98	Denmark	31.91	2
V01.Y98	Finland	157.93	1
V01.Y98	France	210.28	1
V01.Y98	Germany	365.36	0
V01.Y98	Hungary	155.25	0
V01.Y98	Lithuania	8.83	2
V01.Y98	Netherlands	72.89	3
V01.Y98	Norway	36.65	1
V01.Y98	Poland	47.99	1
V01.Y98	Romania	7.36	0
V01.Y98	Slovenia	9.70	0
V01.Y98	Spain	32.10	2
V01.Y98	Sweden	92.45	3
V01.Y98	Switzerland	178.13	3
V01.Y98	United Kingdom	212.98	3
V01.Y98	Belgium	14.14	0
V01.Y98	Czech Republic	6.20	0
V01.Y98	Denmark	26.78	0
V01.Y98	Finland	29.60	0
V01.Y98	France	21.29	2
V01.Y98	Germany	100.95	0
V01.Y98	Hungary	6.13	0
V01.Y98	Netherlands	30.27	3
V01.Y98	Norway	21.92	2
V01.Y98	Poland	5.94	0
V01.Y98	Romania	5.84	0
V01.Y98	Spain	6.55	0
V01.Y98	Sweden	31.02	0
V01.Y98	Switzerland	10.10	0
V01.Y98	United Kingdom	158.50	2
V01.Y98	Finland	16.68	0
V01.Y98	France	14.38	0
V01.Y98	Germany	20.47	0
V01.Y98	Hungary	10.20	0
V01.Y98	United Kingdom	12.71	3
V01.Y98	Austria	85.12	1
V01.Y98	Belgium	167.52	0
V01.Y98	Croatia	16.95	1
V01.Y98	Czech Republic	38.66	1
V01.Y98	Denmark	57.41	0
V01.Y98	Finland	25.15	0
V01.Y98	France	1137.56	3
V01.Y98	Germany	808.10	3

For peer review only

V01.Y98	Hungary	125.03	1
V01.Y98	Lithuania	5.88	2
V01.Y98	Netherlands	160.80	1
V01.Y98	Norway	41.66	1
V01.Y98	Poland	52.83	1
V01.Y98	Romania	26.13	0
V01.Y98	Spain	151.48	1
V01.Y98	Sweden	122.98	2
V01.Y98	Switzerland	60.46	0
V01.Y98	United Kingdom	288.79	1
V01.Y98	France	18.28	3
V01.Y98	Germany	25.23	0
V01.Y98	Netherlands	6.33	1
V01.Y98	Poland	190.62	3
V01.Y98	Romania	30.92	1
V01.Y98	Germany	5.32	0
V01.Y98	Hungary	12.49	1
V01.Y98	Austria	24.11	2
V01.Y98	Belgium	21.91	2
V01.Y98	Croatia	5.55	0
V01.Y98	Czech Republic	29.20	1
V01.Y98	Denmark	38.51	0
V01.Y98	Finland	28.19	1
V01.Y98	France	93.17	1
V01.Y98	Germany	288.27	3
V01.Y98	Hungary	18.13	0
V01.Y98	Lithuania	5.63	0
V01.Y98	Netherlands	23.49	0
V01.Y98	Norway	16.21	2
V01.Y98	Poland	19.12	0
V01.Y98	Slovenia	16.75	3
V01.Y98	Spain	44.73	0
V01.Y98	Sweden	33.59	1
V01.Y98	Switzerland	26.78	0
V01.Y98	United Kingdom	207.68	2
V01.Y98	Belgium	10.87	1
V01.Y98	France	20.62	2
V01.Y98	Germany	42.66	2
V01.Y98	Hungary	48.90	1
V01.Y98	Romania	18.52	1
V01.Y98	Spain	27.35	1
V01.Y98	Belgium	17.01	0
V01.Y98	Croatia	13.20	3
V01.Y98	Czech Republic	9.26	2
V01.Y98	France	102.86	2
V01.Y98	Germany	75.97	0
V01.Y98	Hungary	42.25	3
V01.Y98	Latvia	6.81	1
V01.Y98	Netherlands	11.33	0
V01.Y98	Poland	12.54	1

For peer review only

V01.Y98	Romania	53.55	1
V01.Y98	Spain	81.27	0
V01.Y98	Switzerland	5.02	0
V01.Y98	United Kingdom	12.56	1
V01.Y98	Austria	618.58	3
V01.Y98	Belgium	1036.56	3
V01.Y98	Croatia	478.17	1
V01.Y98	Czech Republic	950.55	1
V01.Y98	Denmark	269.12	2
V01.Y98	Estonia	215.74	2
V01.Y98	Finland	309.30	3
V01.Y98	France	5226.91	0
V01.Y98	Germany	4776.47	2
V01.Y98	Hungary	1621.60	1
V01.Y98	Latvia	429.42	2
V01.Y98	Lithuania	1083.28	2
V01.Y98	Netherlands	733.42	2
V01.Y98	Norway	211.43	0
V01.Y98	Poland	5312.91	3
V01.Y98	Romania	2391.17	1
V01.Y98	Slovenia	309.88	2
V01.Y98	Spain	1718.76	0
V01.Y98	Sweden	487.87	0
V01.Y98	Switzerland	321.11	1
V01.Y98	United Kingdom	2442.85	1
V01.Y98	Austria	60.16	1
V01.Y98	Belgium	157.70	2
V01.Y98	Croatia	44.48	1
V01.Y98	Czech Republic	26.69	2
V01.Y98	Denmark	41.03	1
V01.Y98	Finland	51.21	1
V01.Y98	France	599.82	1
V01.Y98	Germany	295.45	2
V01.Y98	Hungary	55.91	1
V01.Y98	Latvia	6.30	2
V01.Y98	Lithuania	8.88	0
V01.Y98	Netherlands	118.68	2
V01.Y98	Norway	35.39	0
V01.Y98	Poland	37.24	1
V01.Y98	Romania	29.20	1
V01.Y98	Slovenia	22.96	1
V01.Y98	Spain	151.07	1
V01.Y98	Sweden	68.01	2
V01.Y98	Switzerland	65.29	1
V01.Y98	United Kingdom	133.13	0
V01.Y98	Austria	15.19	0
V01.Y98	Belgium	14.79	2
V01.Y98	Croatia	25.61	0
V01.Y98	Czech Republic	72.46	2
V01.Y98	Denmark	15.12	0

For peer review only

V01.Y98	Finland	59.03	1
V01.Y98	France	15.18	0
V01.Y98	Germany	234.90	1
V01.Y98	Hungary	27.24	2
V01.Y98	Lithuania	8.40	0
V01.Y98	Netherlands	7.14	1
V01.Y98	Norway	7.33	0
V01.Y98	Poland	14.66	0
V01.Y98	Slovenia	11.35	0
V01.Y98	Spain	23.91	2
V01.Y98	Sweden	18.78	0
V01.Y98	Switzerland	29.73	0
V01.Y98	Austria	24.68	0
V01.Y98	Belgium	28.19	1
V01.Y98	Croatia	12.88	0
V01.Y98	Czech Republic	20.19	0
V01.Y98	Denmark	46.53	0
V01.Y98	Estonia	5.62	1
V01.Y98	Finland	115.19	1
V01.Y98	France	140.54	3
V01.Y98	Germany	68.01	1
V01.Y98	Hungary	10.18	2
V01.Y98	Latvia	6.72	0
V01.Y98	Norway	29.32	2
V01.Y98	Poland	9.02	2
V01.Y98	Slovenia	5.68	2
V01.Y98	Spain	51.69	3
V01.Y98	Sweden	78.06	2
V01.Y98	Switzerland	20.18	1
V01.Y98	United Kingdom	57.14	1
V01.Y98	Austria	188.90	2
V01.Y98	Belgium	162.93	1
V01.Y98	Croatia	70.08	1
V01.Y98	Czech Republic	70.95	1
V01.Y98	Denmark	5.43	1
V01.Y98	Estonia	10.88	0
V01.Y98	Finland	7.99	0
V01.Y98	France	1511.58	3
V01.Y98	Germany	483.30	1
V01.Y98	Hungary	42.47	2
V01.Y98	Latvia	16.63	1
V01.Y98	Lithuania	11.17	1
V01.Y98	Netherlands	32.71	0
V01.Y98	Norway	51.16	2
V01.Y98	Poland	21.95	2
V01.Y98	Romania	9.27	0
V01.Y98	Slovenia	32.45	0
V01.Y98	Spain	117.31	0
V01.Y98	Sweden	30.78	2
V01.Y98	Switzerland	221.89	2

For peer review only

V01.Y98	United Kingdom	53.59	1
V01.Y98	Croatia	19.61	1
V01.Y98	Austria	6.84	0
V01.Y98	Belgium	10.59	0
V01.Y98	Croatia	5.37	0
V01.Y98	Czech Republic	6.52	0
V01.Y98	Denmark	6.08	1
V01.Y98	Finland	10.76	1
V01.Y98	France	66.24	0
V01.Y98	Germany	70.57	0
V01.Y98	Hungary	13.01	1
V01.Y98	Netherlands	14.52	0
V01.Y98	Poland	11.25	0
V01.Y98	Romania	7.29	1
V01.Y98	Spain	15.89	1
V01.Y98	Sweden	14.87	0
V01.Y98	United Kingdom	48.70	0
V01.Y98	Austria	42.93	0
V01.Y98	Belgium	24.97	0
V01.Y98	Croatia	25.36	0
V01.Y98	Czech Republic	57.01	1
V01.Y98	Denmark	25.75	0
V01.Y98	Estonia	6.79	2
V01.Y98	Finland	20.80	0
V01.Y98	France	109.84	2
V01.Y98	Germany	361.17	0
V01.Y98	Hungary	77.46	0
V01.Y98	Latvia	12.79	0
V01.Y98	Lithuania	13.98	0
V01.Y98	Netherlands	44.92	3
V01.Y98	Norway	14.76	0
V01.Y98	Poland	78.28	1
V01.Y98	Romania	42.35	0
V01.Y98	Slovenia	11.95	0
V01.Y98	Spain	71.57	0
V01.Y98	Sweden	34.17	1
V01.Y98	Switzerland	28.43	0
V01.Y98	United Kingdom	126.21	3
V01.Y98	Austria	144.01	1
V01.Y98	Belgium	111.25	1
V01.Y98	Croatia	54.85	0
V01.Y98	Czech Republic	139.01	2
V01.Y98	Denmark	34.76	0
V01.Y98	Estonia	8.90	2
V01.Y98	Finland	55.38	3
V01.Y98	France	725.25	1
V01.Y98	Germany	960.08	2
V01.Y98	Hungary	165.03	1
V01.Y98	Latvia	18.62	1
V01.Y98	Lithuania	21.39	3

For peer review only

V01.Y98	Netherlands	137.31	0
V01.Y98	Norway	29.02	0
V01.Y98	Poland	133.36	3
V01.Y98	Romania	37.10	3
V01.Y98	Slovenia	23.73	1
V01.Y98	Spain	789.29	2
V01.Y98	Sweden	61.41	0
V01.Y98	Switzerland	146.03	3
V01.Y98	United Kingdom	147.98	1
V01.Y98	Austria	87.03	0
V01.Y98	Belgium	83.81	0
V01.Y98	Croatia	19.12	0
V01.Y98	Czech Republic	48.92	2
V01.Y98	Denmark	23.90	0
V01.Y98	Finland	53.71	0
V01.Y98	France	189.23	1
V01.Y98	Germany	630.08	3
V01.Y98	Hungary	101.65	2
V01.Y98	Netherlands	183.64	0
V01.Y98	Norway	10.08	0
V01.Y98	Poland	20.85	3
V01.Y98	Slovenia	12.33	0
V01.Y98	Spain	93.44	1
V01.Y98	Sweden	78.53	1
V01.Y98	Switzerland	101.40	2
V01.Y98	United Kingdom	192.24	2
V01.Y98	Finland	18.87	1
V01.Y98	France	18.88	0
V01.Y98	Germany	68.20	2
V01.Y98	Hungary	10.93	1
V01.Y98	Netherlands	12.69	0
V01.Y98	Norway	6.11	0
V01.Y98	Poland	11.65	1
V01.Y98	Romania	9.44	0
V01.Y98	Spain	15.04	3
V01.Y98	Sweden	9.22	0
V01.Y98	United Kingdom	9.05	0
V01.Y98	Austria	7.02	0
V01.Y98	Belgium	7.98	0
V01.Y98	Czech Republic	5.34	0
V01.Y98	France	33.96	2
V01.Y98	Germany	95.03	1
V01.Y98	Hungary	32.08	0
V01.Y98	Poland	11.06	0
V01.Y98	Romania	7.80	2
V01.Y98	Sweden	5.87	0
V01.Y98	Switzerland	7.24	0
V01.Y98	United Kingdom	19.81	1
V01.Y98	Austria	13.75	2
V01.Y98	Belgium	103.23	2

For peer review only

V01.Y98	Croatia	19.39	2
V01.Y98	Czech Republic	16.22	2
V01.Y98	France	579.83	1
V01.Y98	Germany	208.49	1
V01.Y98	Netherlands	10.77	2
V01.Y98	Norway	19.58	2
V01.Y98	Poland	17.74	1
V01.Y98	Romania	10.66	3
V01.Y98	Spain	129.95	0
V01.Y98	Switzerland	19.89	0
V01.Y98	United Kingdom	116.18	0
V01.Y98	Austria	6.90	2
V01.Y98	Belgium	18.08	1
V01.Y98	Czech Republic	12.05	1
V01.Y98	Denmark	6.72	0
V01.Y98	Estonia	5.99	3
V01.Y98	Finland	9.74	1
V01.Y98	France	36.67	1
V01.Y98	Germany	65.63	1
V01.Y98	Hungary	17.61	1
V01.Y98	Latvia	11.80	1
V01.Y98	Lithuania	14.27	1
V01.Y98	Netherlands	14.35	0
V01.Y98	Poland	38.54	1
V01.Y98	Romania	50.01	1
V01.Y98	Spain	29.79	1
V01.Y98	Sweden	6.95	0
V01.Y98	Switzerland	7.21	1
V01.Y98	United Kingdom	14.96	1
V01.Y98	Germany	5.65	1
V01.Y98	Croatia	7.40	2
V01.Y98	Czech Republic	10.63	0
V01.Y98	Finland	5.59	0
V01.Y98	Germany	24.89	0
V01.Y98	Spain	11.51	2
V01.Y98	Sweden	5.47	3
V01.Y98	Finland	7.33	1
V01.Y98	France	8.70	0
V01.Y98	Spain	10.98	0
V01.Y98	United Kingdom	5.59	0
V01.Y98	Austria	10.03	0
V01.Y98	Belgium	36.08	1
V01.Y98	Croatia	13.26	1
V01.Y98	Czech Republic	7.69	3
V01.Y98	Finland	5.78	0
V01.Y98	France	123.92	1
V01.Y98	Germany	37.64	1
V01.Y98	Latvia	8.95	2
V01.Y98	Lithuania	5.02	2
V01.Y98	Netherlands	40.46	3

For peer review only

V01.Y98	Norway	7.35	0
V01.Y98	Poland	21.34	2
V01.Y98	Romania	6.94	1
V01.Y98	Spain	49.49	1
V01.Y98	Sweden	10.56	2
V01.Y98	Switzerland	17.25	1
V01.Y98	United Kingdom	21.78	1
V01.Y98	United Kingdom	6.97	1
V01.Y98	Austria	17.57	0
V01.Y98	Belgium	36.89	1
V01.Y98	Croatia	15.50	0
V01.Y98	Czech Republic	36.72	0
V01.Y98	Denmark	13.33	0
V01.Y98	Estonia	36.69	1
V01.Y98	Finland	43.70	1
V01.Y98	France	116.40	2
V01.Y98	Germany	162.97	1
V01.Y98	Hungary	61.84	1
V01.Y98	Latvia	65.82	1
V01.Y98	Lithuania	78.53	1
V01.Y98	Netherlands	50.67	1
V01.Y98	Norway	13.55	0
V01.Y98	Poland	201.28	2
V01.Y98	Romania	136.65	2
V01.Y98	Slovenia	5.99	0
V01.Y98	Spain	140.90	1
V01.Y98	Sweden	36.44	0
V01.Y98	Switzerland	12.94	1
V01.Y98	United Kingdom	70.75	1
V01.Y98	Croatia	5.49	0
V01.Y98	Czech Republic	5.84	0
V01.Y98	Estonia	16.90	3
V01.Y98	Finland	9.43	1
V01.Y98	France	8.22	0
V01.Y98	Germany	38.94	0
V01.Y98	Hungary	20.71	1
V01.Y98	Latvia	9.51	0
V01.Y98	Lithuania	101.90	2
V01.Y98	Netherlands	11.02	0
V01.Y98	Poland	64.80	1
V01.Y98	Romania	59.74	2
V01.Y98	Spain	16.74	0
V01.Y98	Sweden	6.79	0
V01.Y98	United Kingdom	13.22	2
V01.Y98	Belgium	13.49	2
V01.Y98	Croatia	6.85	1
V01.Y98	Czech Republic	9.06	0
V01.Y98	Estonia	13.98	1
V01.Y98	Finland	12.07	1
V01.Y98	France	49.80	0

For peer review only

V01.Y98	Germany	41.10	1
V01.Y98	Hungary	53.27	2
V01.Y98	Latvia	54.25	2
V01.Y98	Lithuania	8.87	2
V01.Y98	Netherlands	11.03	0
V01.Y98	Poland	119.06	2
V01.Y98	Romania	87.26	1
V01.Y98	Spain	12.35	1
V01.Y98	Sweden	5.58	1
V01.Y98	United Kingdom	14.70	3
V01.Y98	Germany	5.71	3
V01.Y98	France	10.55	1
V01.Y98	Germany	15.86	0
V01.Y98	Poland	6.09	1
V01.Y98	Romania	9.24	3
V01.Y98	Germany	7.64	3
V01.Y98	Belgium	22.11	0
V01.Y98	Czech Republic	11.99	1
V01.Y98	Estonia	12.08	3
V01.Y98	France	59.06	1
V01.Y98	Germany	38.41	1
V01.Y98	Hungary	5.09	0
V01.Y98	Latvia	27.52	0
V01.Y98	Netherlands	7.70	3
V01.Y98	Poland	17.46	1
V01.Y98	Romania	189.54	1
V01.Y98	Spain	63.01	1
V01.Y98	Sweden	5.26	1
V01.Y98	United Kingdom	36.68	1
V01.Y98	Sweden	9.14	0
V01.Y98	United Kingdom	60.67	1
V01.Y98	Czech Republic	10.48	2
V01.Y98	Denmark	8.95	1
V01.Y98	Finland	5.25	2
V01.Y98	Germany	30.18	1
V01.Y98	Hungary	12.45	1
V01.Y98	Lithuania	11.36	0
V01.Y98	Poland	21.01	1
V01.Y98	Sweden	56.41	3
V01.Y98	United Kingdom	153.14	1
V01.Y98	Belgium	8.42	1
V01.Y98	Denmark	27.30	1
V01.Y98	Germany	86.69	3
V01.Y98	Poland	47.80	3
V01.Y98	Slovenia	8.38	3
V01.Y98	Sweden	52.51	2
V01.Y98	United Kingdom	199.98	0
V01.Y98	United Kingdom	7.16	0
V01.Y98	Austria	49.85	2
V01.Y98	Belgium	16.13	1

For peer review only

V01.Y98	Czech Republic	12.29	0
V01.Y98	Denmark	20.61	1
V01.Y98	France	25.12	2
V01.Y98	Germany	108.71	2
V01.Y98	Hungary	13.60	3
V01.Y98	Lithuania	5.51	0
V01.Y98	Netherlands	7.43	0
V01.Y98	Poland	74.27	1
V01.Y98	Romania	5.83	0
V01.Y98	Spain	8.31	0
V01.Y98	Sweden	71.62	2
V01.Y98	Switzerland	5.47	0
V01.Y98	United Kingdom	180.15	1
V01.Y98	Czech Republic	10.38	2
V01.Y98	Denmark	6.57	2
V01.Y98	Germany	30.84	0
V01.Y98	Hungary	5.54	0
V01.Y98	Poland	334.76	2
V01.Y98	Romania	20.21	2
V01.Y98	Sweden	20.89	2
V01.Y98	Hungary	6.00	1
V01.Y98	Poland	9.45	1
V01.Y98	Austria	8.61	2
V01.Y98	Czech Republic	5.26	2
V01.Y98	Germany	31.92	0
V01.Y98	Lithuania	9.39	2
V01.Y98	Poland	73.69	3
V01.Y98	Romania	23.50	1
V01.Y98	Spain	7.22	2
V01.Y98	United Kingdom	18.93	1
V01.Y98	Germany	19.91	2
V01.Y98	Hungary	8.98	0
V01.Y98	Lithuania	14.04	1
V01.Y98	Poland	36.82	1
V01.Y98	Romania	21.27	0
V01.Y98	Spain	11.57	3
V01.Y98	Belgium	11.90	2
V01.Y98	Czech Republic	20.73	2
V01.Y98	Estonia	6.12	3
V01.Y98	France	29.46	2
V01.Y98	Germany	230.95	2
V01.Y98	Latvia	11.04	0
V01.Y98	Lithuania	41.19	3
V01.Y98	Poland	388.18	3
V01.Y98	Romania	18.49	1
V01.Y98	United Kingdom	335.77	1
V01.Y98	Austria	36.51	1
V01.Y98	Belgium	28.20	0
V01.Y98	Czech Republic	20.75	0
V01.Y98	Denmark	8.70	1

For peer review only

V01.Y98	Finland	16.92	0
V01.Y98	France	17.44	0
V01.Y98	Germany	158.08	2
V01.Y98	Hungary	31.49	2
V01.Y98	Latvia	13.72	0
V01.Y98	Lithuania	32.22	2
V01.Y98	Netherlands	6.22	1
V01.Y98	Poland	139.57	3
V01.Y98	Romania	8.37	0
V01.Y98	Slovenia	6.14	0
V01.Y98	Spain	11.54	0
V01.Y98	Sweden	34.12	2
V01.Y98	Switzerland	16.93	1
V01.Y98	United Kingdom	192.63	2
V01.Y98	Czech Republic	8.21	0
V01.Y98	Germany	27.76	1
V01.Y98	France	25.27	1
V01.Y98	United Kingdom	5.58	1
V01.Y98	Belgium	12.43	0
V01.Y98	Czech Republic	5.62	0
V01.Y98	France	262.05	3
V01.Y98	Germany	49.12	1
V01.Y98	Latvia	5.01	0
V01.Y98	Lithuania	5.06	0
V01.Y98	Poland	26.68	1
V01.Y98	Switzerland	8.52	1
V01.Y98	United Kingdom	6.47	1
V01.Y98	Denmark	5.99	1
V01.Y98	Germany	39.50	1
V01.Y98	Hungary	7.02	1
V01.Y98	Latvia	15.35	1
V01.Y98	Lithuania	7.29	0
V01.Y98	Poland	9.71	0
V01.Y98	Romania	11.33	1
V01.Y98	Sweden	13.13	1
V01.Y98	United Kingdom	36.70	1
V01.Y98	Czech Republic	5.48	0
V01.Y98	France	25.79	2
V01.Y98	Germany	47.04	1
V01.Y98	Latvia	8.50	0
V01.Y98	Lithuania	9.20	0
V01.Y98	Poland	66.57	2
V01.Y98	Romania	9.96	2
V01.Y98	United Kingdom	25.81	0
V01.Y98	Estonia	5.94	0
V01.Y98	Germany	7.62	0
V01.Y98	Hungary	6.79	3
V01.Y98	Lithuania	91.20	2
V01.Y98	Poland	60.70	3
V01.Y98	Austria	18.02	2

For peer review only

V01.Y98	Belgium	12.87	1
V01.Y98	Czech Republic	25.48	0
V01.Y98	Estonia	8.11	3
V01.Y98	Finland	5.46	0
V01.Y98	France	34.74	2
V01.Y98	Germany	100.68	3
V01.Y98	Hungary	20.24	2
V01.Y98	Latvia	21.63	1
V01.Y98	Lithuania	32.36	1
V01.Y98	Poland	155.43	1
V01.Y98	Romania	37.67	3
V01.Y98	Spain	21.55	1
V01.Y98	Sweden	13.61	0
V01.Y98	Switzerland	8.50	0
V01.Y98	United Kingdom	78.56	1
V01.Y98	Austria	9.77	2
V01.Y98	France	17.68	1
V01.Y98	Germany	51.99	1
V01.Y98	Hungary	15.44	2
V01.Y98	Sweden	8.71	0
V01.Y98	Switzerland	5.16	1
V01.Y98	United Kingdom	30.76	3
V01.Y98	Finland	8.88	0
V01.Y98	Sweden	12.84	0
V01.Y98	Estonia	16.62	1
V01.Y98	Germany	29.16	0
V01.Y98	Latvia	5.30	1
V01.Y98	Lithuania	5.21	1
V01.Y98	Poland	6.64	1
V01.Y98	Romania	7.41	0
V01.Y98	Sweden	7.84	0
V01.Y98	United Kingdom	354.78	2
V01.Y98	Austria	5.56	2
V01.Y98	Belgium	123.55	1
V01.Y98	Czech Republic	218.38	3
V01.Y98	Denmark	9.14	3
V01.Y98	Estonia	29.46	0
V01.Y98	Finland	23.81	0
V01.Y98	France	67.22	1
V01.Y98	Germany	1120.42	2
V01.Y98	Hungary	27.69	0
V01.Y98	Latvia	80.54	2
V01.Y98	Lithuania	99.08	0
V01.Y98	Netherlands	30.67	1
V01.Y98	Poland	1014.04	3
V01.Y98	Romania	111.90	1
V01.Y98	Slovenia	35.66	1
V01.Y98	Spain	9.95	1
V01.Y98	Sweden	7.86	0
V01.Y98	Switzerland	9.06	0

For peer review only

V01.Y98	United Kingdom	167.87	1
V01.Y98	France	12.52	0
V01.Y98	Germany	5.98	1
V01.Y98	United Kingdom	7.29	1
V01.Y98	France	8.67	2
V01.Y98	France	6.93	0
V01.Y98	Belgium	6.12	0
V01.Y98	France	546.05	3
V01.Y98	Germany	14.88	0
V01.Y98	Poland	47.71	2
V01.Y98	Slovenia	8.13	3
V01.Y98	Spain	45.45	3
V01.Y98	United Kingdom	10.41	0
V01.Y98	France	40.93	2
V01.Y98	Germany	8.99	0
V01.Y98	Spain	11.61	2
V01.Y98	United Kingdom	6.35	1
V01.Y98	France	30.90	3
V01.Y98	France	8.77	0
V01.Y98	France	17.43	1
V01.Y98	Spain	62.86	2
V01.Y98	France	15.12	1
V01.Y98	Belgium	5.85	1
V01.Y98	France	48.67	0
V01.Y98	Germany	10.95	0
V01.Y98	Spain	22.20	1
V01.Y98	United Kingdom	17.41	2
V01.Y98	Austria	21.16	2
V01.Y98	Denmark	18.12	0
V01.Y98	France	35.65	0
V01.Y98	Germany	104.83	3
V01.Y98	Hungary	9.40	0
V01.Y98	Poland	8.31	0
V01.Y98	Spain	10.29	1
V01.Y98	United Kingdom	42.18	2
V01.Y98	Germany	83.42	0
V01.Y98	Denmark	5.57	1
V01.Y98	France	7.20	0
V01.Y98	Germany	56.41	0
V01.Y98	France	9.94	2
V01.Y98	Germany	29.04	0
V01.Y98	Germany	7.88	1
V01.Y98	Spain	16.02	2
V01.Y98	Germany	7.80	0
V01.Y98	Austria	110.35	3
V01.Y98	Belgium	85.71	2
V01.Y98	Croatia	28.51	2
V01.Y98	Czech Republic	33.14	2
V01.Y98	Estonia	6.36	3
V01.Y98	France	445.20	2

For peer review only

V01.Y98	Germany	615.33	3
V01.Y98	Latvia	8.20	1
V01.Y98	Lithuania	13.70	0
V01.Y98	Netherlands	27.57	0
V01.Y98	Poland	308.00	0
V01.Y98	Romania	17.79	0
V01.Y98	Slovenia	78.14	0
V01.Y98	Spain	230.79	1
V01.Y98	Sweden	84.58	2
V01.Y98	Switzerland	6.03	0
V01.Y98	United Kingdom	452.11	2
V01.Y98	Austria	7.75	1
V01.Y98	Belgium	10.45	2
V01.Y98	Czech Republic	13.26	2
V01.Y98	France	32.79	2
V01.Y98	Germany	71.53	3
V01.Y98	Poland	9.46	0
V01.Y98	Spain	30.78	0
V01.Y98	Sweden	23.38	0
V01.Y98	United Kingdom	101.34	2
V01.Y98	Austria	9.32	0
V01.Y98	Belgium	6.97	0
V01.Y98	Finland	21.29	0
V01.Y98	France	50.42	0
V01.Y98	Germany	29.05	1
V01.Y98	Netherlands	33.27	0
V01.Y98	Poland	15.08	0
V01.Y98	Spain	15.34	0
V01.Y98	Sweden	13.50	0
V01.Y98	United Kingdom	45.92	1
V01.Y98	Belgium	14.69	0
V01.Y98	Denmark	7.58	1
V01.Y98	Finland	54.77	0
V01.Y98	France	150.78	2
V01.Y98	Germany	56.87	3
V01.Y98	Hungary	18.28	1
V01.Y98	Lithuania	29.26	3
V01.Y98	Netherlands	380.47	2
V01.Y98	Norway	39.31	2
V01.Y98	Poland	79.98	0
V01.Y98	Spain	25.39	1
V01.Y98	Sweden	68.72	1
V01.Y98	Switzerland	24.62	0
V01.Y98	United Kingdom	79.24	2
V01.Y98	Finland	5.69	0
V01.Y98	France	14.87	0
V01.Y98	Germany	37.82	0
V01.Y98	Poland	12.74	0
V01.Y98	United Kingdom	12.95	2
V01.Y98	France	17.90	0

For peer review only

V01.Y98	Germany	17.80	1
V01.Y98	Poland	5.88	0
V01.Y98	United Kingdom	7.91	3
V01.Y98	Austria	53.70	3
V01.Y98	France	9.22	1
V01.Y98	Germany	44.34	1
V01.Y98	Poland	112.91	2
V01.Y98	Spain	6.41	1

For peer review only

**trend**
yes
no
yes
yes
no
yes
no
yes
yes
yes
yes
yes
yes
no
yes
yes
yes
yes
yes
yes
yes
yes
yes
yes
yes
yes
yes
no
no
yes
yes
yes
yes
yes
yes
yes
yes
no
yes
yes
yes
yes
yes
yes
yes
yes
yes
yes
yes

For peer review only

yes
yes
yes
no
yes
yes
yes
yes
yes
yes
yes
yes
yes
yes
yes
no
yes
no
no
yes
yes
yes
no
yes
yes
yes
yes
yes
yes
yes
yes
yes
yes
no
yes
yes
yes
no
yes
yes
yes
yes
yes
yes
no
yes
yes
yes
yes
yes

For peer review only

yes
yes
no
yes
yes
no
yes
yes
yes
yes
no
no
yes
no
yes
yes
no
yes
no
no
yes
yes
yes
no
no
yes
yes
yes
yes
yes
no
no
yes
no
yes
yes
yes
yes
no
yes
yes
yes
no
no
yes
yes
yes
yes
yes
no

For peer review only

yes
no
yes
yes
yes
yes
yes
no
yes
no
no
yes
yes
no
yes
yes
yes
yes
yes
no
yes
yes
no
yes
no
no
yes
yes
yes
yes
no
no
yes
yes
yes
yes
no
yes
no
no
yes
yes
no
yes
yes
yes
yes
yes
yes
yes

For peer review only

no
yes
no
yes
yes
yes
yes
yes
no
yes
yes
yes
yes
yes
yes
yes
yes
yes
yes
yes
no
no
no
no
no
no
no
yes
no
yes
no
yes
yes
no
no
yes
yes
no
no
yes
no
no
yes
yes
no
yes
yes
no
no
yes

For peer review only

yes
yes
no
no
no
no
no
yes
yes
no
yes
yes
yes
no
yes
no
no
no
yes
yes
yes
yes
yes
yes
yes
no
yes
yes
no
yes
yes
yes
no
yes
yes
yes
yes
yes
yes
yes
no
yes
no
yes
yes
yes
yes
yes
no
yes

For peer review only

yes
no
yes
yes
yes
yes
yes
yes
yes
yes
yes
yes
no
yes
yes
yes
yes
yes
yes
no
no
yes
yes
yes
yes
yes
no
yes
yes
yes
yes
yes
yes
yes
yes
no
yes
yes
yes
yes
yes
yes
yes
yes
yes
no
yes
yes
no
yes

For peer review only

yes
yes
yes
no
no
yes
yes
yes
yes
yes
no
yes
yes
no
no
no
no
yes
no
yes
yes
yes
no
yes
yes
yes
yes
yes
no
yes
yes
yes
yes
no
no
no
yes
yes
no
no
yes
no
no
no
no
yes
yes
no
no
no

For peer review only

yes
yes
yes
yes
yes
yes
yes
no
no
no
yes
yes
yes
yes
yes
yes
yes
no
yes
yes
yes
yes
yes
yes
yes
no
yes
no
no
yes
yes
no
no
no
yes
no
no
no
yes
yes
no
yes
no
yes
yes
yes
yes
yes
yes
yes

For peer review only

yes
yes
yes
yes
yes
yes
no
no
yes
no
yes
no
yes
no
yes
yes
yes
no
no
yes
no
no
no
no
yes
no
yes
no
no
yes
no
no
yes
yes
yes
yes
yes
no
no
no
yes
yes
yes
no
no
no
yes
yes
yes
no

For peer review only

no
no
no
yes
yes
no
no
no
yes
yes
yes
no
no
no
no
no
yes
yes
no
yes
yes
yes
no
no
yes
yes
no
yes
yes
no
yes
no
no
yes
yes
yes
yes
yes
yes
yes
yes
no
no
yes
yes
no
yes
no
no
no

For peer review only

no
no
no
no
no
no
no
yes
no
yes
no
no
no
yes
no
no
no
no
yes
yes
yes
yes
yes
no
yes
yes
yes
no
no
no
no
no
no
no
yes
no
yes
no
yes
no
no
no
no
no
no
yes
no
no
no
no

For peer review only

no
no
no
no
no
no
yes
no
no
yes
no
no
no
no
no
no
yes
yes
yes
yes
no
no
no
no
yes
no
yes
no
yes
yes
yes
yes
yes
yes
no
yes
yes
yes
yes
no
no
no
yes
yes
yes
yes
yes
yes
no
yes

For peer review only

yes
yes
yes
yes
yes
no
yes
yes
yes
no
yes
no
no
yes
no
no
no
no
no
no
no
yes
no
no
no
no
yes
yes
yes
no
yes
yes
yes
no
no
no
no
yes
yes
no
no
no
yes
yes
yes
yes
yes
no
no
no

For peer review only

yes
yes
yes
no
no
yes
yes
yes
yes
no
no
yes
yes
yes
no
no
yes
no
yes
no
no
yes
yes
yes
yes
yes
yes
yes
yes
no
no
no
yes
yes
no
yes
yes
yes
no
no
no
yes
no
yes
yes
no
yes
yes
yes
yes

For peer review only

yes
yes
no
yes
yes
yes
yes
no
no
yes
yes
yes
yes
yes
yes
yes
yes
yes
yes
yes
yes
yes
yes
yes
yes
yes
yes
yes
yes
yes
yes
yes
yes
no
yes
no
yes
no
yes
yes
yes
yes
no
no
yes
no
no
yes
no
yes
no

For peer review only

no
yes
yes
yes
yes
yes
yes
no
no
yes
yes
yes
no
yes
yes
yes
no
yes
yes
yes
yes
yes
yes
yes
no
yes
yes
yes
yes
yes
yes
yes
yes
yes
no
no
yes
yes
no
yes
no
no
yes
yes
yes
yes
yes
yes
yes
yes

yes
yes
yes
yes
yes
yes
no
yes
yes
yes
yes
no
no
yes
yes
no
yes
no
yes
yes
no
no
yes
yes
yes
no
yes
no
no
yes
yes
yes
yes
no
no
yes
no
yes
yes
yes
yes
no
no
yes
yes
yes
no
yes
yes
yes

For peer review only

yes
yes
yes
yes
no
yes
yes
yes
yes
yes
yes
no
no
yes
yes
yes
yes
yes
yes
yes
no
yes
yes
yes
yes
yes
yes
yes
no
yes
yes
no
no
yes
yes
yes
yes
yes
no
no
yes
yes
yes
yes
no
yes
yes
no
yes
no

For peer review only

yes
yes
no
yes
yes
no
yes
yes
yes
yes
no
yes
no
no
yes
yes
yes
yes
yes
yes
yes
yes
no
yes
no
yes
no
yes
yes
yes
yes
yes
yes
yes
no
yes
yes
yes
no
yes
yes
no
no
no
no
no
yes
yes
no
yes

For peer review only

yes
no
no
yes
yes
no
no
no
no
no
no
no
yes
no
no
no
yes
yes
no
yes
yes
no
yes
no
no
yes
yes
yes
yes
yes
yes
yes
no
yes
yes
yes
no
no
yes
yes
yes
yes
yes
yes
no
yes
yes
yes
no
no

For peer review only

For peer review only - http://bmjopen.bmj.com/site/about/guidelines.xhtml

yes
no
no
no
yes
no
no
yes
yes
yes
no
yes
yes
yes
yes
yes
no
no
yes
yes
yes
yes
yes
yes
yes
yes
yes
yes
yes
yes
no
yes
no
yes
yes
no
no
yes
no
no
yes
yes
yes
yes
yes
no
no
yes
yes
yes

For peer review only

yes
no
no
yes
yes
yes
no
yes
yes
yes
yes
yes
yes
no
yes
no
yes
yes
yes
yes
yes
yes
yes
no
no
no
no
yes
yes
no
no
yes
no
yes
no
yes
yes
yes
yes
no
no
no
yes
no
no
no
yes
no
no
yes

For peer review only

yes
no
yes
yes
no
no
no
yes
yes
no
no
yes
no
no
no
yes
no
yes
yes
yes
yes
yes
yes
no
no
yes
yes
yes
yes
no
yes
yes
yes
yes
yes
yes
yes
yes
yes
no
no
no
yes
yes
no
no
no
no
no
yes

For peer review only

yes
yes
yes
no
yes
yes
yes
yes
yes
no
yes
no
yes
yes
no
no
yes
no
yes
yes
no
no
yes
no
yes
yes
no
no
yes
no
no
yes
no
yes
no
no
yes
yes
yes
no
no
no
yes
yes
yes
no
no
yes
yes
no

no
yes
yes
yes
yes
no
no
yes
yes
no
no
no
yes
yes
yes
yes
no
no
yes
no
no
no
no
yes
yes
no
no
no
yes
yes
yes
yes
no
yes
no
yes
yes
yes
yes
yes
yes
yes
yes
yes
yes
yes
no
no
yes

For peer review only

yes
yes
no
yes
yes
yes
yes
yes
no
no
no
yes
yes
no
no
no
yes
no
no
no
no
no
no
no
no
yes
no
no
no
yes
no
no
yes
no
yes
no
no
yes
no
no
no
no
yes
yes
yes
no
yes
yes
no
no

For peer review only

yes
no
yes
yes
yes
no
yes
yes
yes
yes
yes
yes
yes
yes
yes
yes
yes
yes
no
yes
yes
yes
no
yes
yes
yes
yes
no
yes
yes
yes
yes
no
no
yes
yes
yes
no
yes
no
yes
yes
yes
yes
yes
yes
yes
yes
no
yes

For peer review only

yes
yes
yes
no
yes
yes
yes
yes
no
yes
no
yes
yes
yes
yes
no
yes
yes
yes
no
yes
yes
no
no
no
yes
yes
no
yes
no
yes
yes
yes
yes
yes
yes
no
no
no
no
yes
yes
yes
yes
no
yes
yes
no
yes
yes

For peer review only

yes
yes
no
yes
yes
no
no
no
no
no
no
no
yes
yes
no
no
yes
yes
yes
no
no
no
yes
no
yes
yes
yes
yes
yes
yes
yes
yes
yes
yes
yes
no
yes
yes
no
yes
no
yes
yes
yes
yes
no
yes
no
yes
yes

For peer review only

yes
yes
yes
no
no
yes
no
no
yes
yes
no
no
no
yes
yes
no
no
yes
yes
no
no
yes
no
yes
yes
yes
yes
yes
yes
yes
yes
yes
yes
no
no
yes
no
yes
yes
yes
yes
yes
yes
yes
yes
no
yes
no
yes
no

For peer review only

no
yes
no
yes
no
yes
yes
no
yes
yes
yes
no
no
no
yes
yes
yes
no
no
no
no
no
no
yes
yes
yes
no
yes
yes
yes
yes
yes
yes
yes
no
yes
yes
yes
no
yes
yes
yes
yes
no
yes
no
yes
yes
no
yes

For peer review only

yes
yes
yes
yes
yes
no
yes
yes
yes
yes
yes
yes
no
yes
no
no
yes
yes
no
no
yes
no
no
no
no
yes
no
yes
no
no
yes
yes
no
no
no
yes
no
no
no
yes
yes
no
yes
yes
yes
no
no
no
no
yes

For peer review only

yes
yes
yes
yes
no
yes
no
yes
yes
no
no
no
yes
no
yes
yes
yes
yes
no
yes
yes
no
no
yes
no
yes
no
no
no
yes
yes
yes
yes
yes
no
yes
yes
yes
yes
yes
no
yes
no
no
yes
no
no
no
yes
yes

For peer review only

yes
no
yes
no
no
yes
no
yes
no
no
yes
no
no
yes
no
no
yes
no
yes
no
no
no
no
no
no
yes
yes
no
no
no
no
no
yes
yes
no
no
yes
yes
no
yes
no
no
yes
yes
yes
yes
no
yes
yes
yes

For peer review only

no
yes
yes
yes
yes
yes
yes
yes
yes
no
no
yes
yes
yes
yes
yes
no
yes
yes
yes
yes
no
yes
no
yes
no
yes
yes
no
no
yes
yes
yes
yes
no
yes
yes
yes
yes
yes
yes
yes
yes
yes
yes
yes
yes
yes
yes
no

For peer review only

yes
no
no
yes
yes
yes
yes
no
yes
no
no
yes
yes
no
yes
no
no
no
yes
yes
yes
yes
no
yes
yes
yes
yes
yes
yes
no
no
yes
yes
no
yes
yes
yes
yes
yes
yes
no
yes
yes
yes
yes
yes
yes
yes
yes
yes

For peer review only

yes
yes
yes
yes
yes
yes
yes
yes
yes
yes
yes
yes
no
no
yes
yes
yes
yes
yes
yes
no
yes
yes
yes
yes
no
no
yes
yes
yes
no
yes
yes
yes
yes
yes
yes
yes
yes
yes
yes
yes
yes
no
no
yes
yes
yes
yes
yes
no

For peer review only

yes
no
yes
no
yes
yes
no
yes
no
no
no
yes
yes
no
yes
no
yes
yes
yes
no
yes
yes
yes
yes
yes
yes
no
yes
no
yes
yes
no
yes
yes
yes
yes
yes
yes
yes
yes
yes
no
yes
yes
yes
yes
yes
yes
yes
yes

For peer review only

yes
no
no
yes
yes
yes
yes
no
no
no
yes
no
no
yes
yes
yes
yes
yes
no
no
no
yes
yes
yes
no
no
yes
no
yes
yes
yes
yes
no
yes
yes
yes
yes
yes
yes
yes
yes
yes
yes
yes
yes
yes
yes
yes
yes
yes

For peer review only

yes
no
no
yes
yes
no
yes
yes
no
no
no
yes
yes
yes
yes
yes
no
no
yes
yes
yes
yes
yes
yes
yes
yes
yes
no
no
yes
yes
yes
no
yes
yes
yes
yes
no
no
yes
no
no
yes
yes
yes
yes
no
no
yes
no

For peer review only

no
no
no
no
yes
no
yes
yes
yes
no
yes
no
yes
no
yes
no
no
no
no
no
no
no
no
no
yes
no
no
yes
no
no
yes
yes
no
no
no
no
no
yes
no
yes
no
yes
yes
no
no
yes
no
no
no
yes

For peer review only

yes
yes
no
no
no
no
yes
yes
yes
yes
yes
yes
yes
no
no
yes
no
yes
no
yes
no
yes
yes
no
yes
no
yes
yes
yes
no
yes
no
no
no
no
yes
no
yes
yes
no
no
no
yes
no
yes
no
no
yes
yes
yes

For peer review only

yes
no
yes
yes
no
yes
yes
yes
yes
yes
yes
no
no
yes
yes
yes
yes
yes
no
no
yes
no
yes
no
yes
no
yes
no
no
yes
no
no
no
no
no
yes
no
yes
yes
yes
no
no
yes
yes
yes
yes
no
no
no
yes

For peer review only

no
yes
yes
yes
yes
yes
no
yes
no
yes
yes
no
no
no
yes
no
yes
no
yes
no
no
yes
yes
yes
yes
yes
yes
no
no
no
yes
yes
no
no
yes
yes
yes
no
yes
no
no
yes
yes
yes
yes
no
yes
no
no
no

For peer review only

yes
no
yes
no
yes
yes
no
yes
no
yes
no
no
no
no
yes
no
no
yes
yes
yes
yes
yes
yes
yes
yes
yes
yes
no
yes
yes
yes
no
yes
yes
yes
yes
no
yes
yes
yes
yes
yes
yes
no
no
no
yes
yes
no
yes

For peer review only

no
yes
yes
yes
no
yes
yes
no
no
yes
yes
yes
yes
no
no
yes
yes
yes
no
yes
no
yes
yes
yes
no
yes
yes
yes
yes
no
no
yes
no
yes
yes
yes
no
no
no
no
no
no
no
yes
yes
no
yes
no
no
yes

For peer review only

yes
yes
yes
no
no
yes
yes
yes
no
yes
no
no
no
yes
yes
no
yes
no
no
no
yes
no
no
no
yes
yes
no
no
no
no
yes
no
yes
no
no
no
yes
yes
no
yes
yes
no
no
no
yes
no
yes
no
no
yes

For peer review only

yes
yes
no
yes
yes
no
yes
yes
yes
no
yes
yes
yes
yes
yes
yes
yes
no
yes
yes
yes
no
no
yes
yes
yes
yes
no
yes
yes
yes
yes
yes
no
no
no
yes
yes
no
no
yes
yes
yes
yes
yes
no
no
no
yes
no

For peer review only

no
no
no
no
no
yes
no
yes
yes
no
no
no
yes
no
yes
no
no
yes
no
yes
yes
no
yes
yes
no
no
yes
yes
yes
no
yes
no
no
no
yes
no
no
no
no
yes
yes
yes
yes
yes
yes
yes
yes
yes
yes
yes

For peer review only

yes
yes
yes
yes
yes
no
yes
yes
yes
yes
yes
no
no
no
yes
no
yes
no
yes
no
no
yes
no
yes
no
no
no
yes
no
yes
no
no
yes
no
no
yes
yes
yes
yes
no
yes
yes
no
yes
yes
no
yes
yes
no
yes

For peer review only

no
yes
yes
yes
no
yes
yes
yes
yes
no
no
yes
no
no
no
yes
yes
no
yes
yes
yes
no
yes
no
yes
yes
no
yes
yes
yes
no
no
yes
yes
no
yes
no
yes
yes
yes
yes
no
yes
yes
no
yes
yes
no
yes
yes

For peer review only

yes
yes
no
yes
yes
yes
yes
yes
yes
yes
yes
yes
yes
yes
yes
yes
yes
yes
yes
yes
yes
yes
yes
yes
yes
no
yes
no
yes
yes
yes
yes
yes
no
yes
yes
yes
no
yes
yes
yes
no
yes
yes
no
yes
yes
yes
yes
yes

yes
yes
yes
yes
yes
yes
yes
yes
yes
no
no
no
no
yes
no
yes
no
yes
no
no
yes
no
no
no
yes
yes
no
no
no
no
no
yes
no
no
no
yes
no
no
no
yes
no
yes
no
no
no
yes
no
no
no
yes

For peer review only

no
yes
no
no
yes
yes
yes
no
yes
yes
yes
no
yes
yes
yes
yes
yes
yes
yes
yes
yes
yes
yes
no
no
yes
yes
yes
yes
yes
no
yes
yes
no
yes
no
yes
yes
no
yes
no
yes
yes
yes
no
no
yes
no
no
yes

For peer review only

no
yes
yes
yes
yes
yes
yes
yes
no
yes
yes
yes
yes
yes
no
yes
yes
yes
yes
yes
yes
yes
yes
yes
yes
yes
yes
yes
no
no
yes
no
no
no
yes
no
no
yes
no
no
yes
no
no
no
no
no
no
no
no
no

For peer review only

no
no
no
no
no
yes
yes
no
yes
yes
yes
yes
yes
yes
yes
yes
yes
no
yes
yes
no
no
yes
yes
no
yes
yes
no
yes
no
yes
no
no
no
no
yes
no
yes
no
yes
yes
no
no
yes
no
no
yes
no
yes
no

For peer review only

yes
no
no
yes
yes
yes
no
no
yes
yes
yes
yes
yes
no
yes
yes
yes
yes
yes
yes
yes
yes
yes
yes
yes
yes
no
yes
yes
no
yes
yes
yes
yes
yes
yes
no
yes
yes
yes
yes
no
yes
yes
no
yes
yes
no
yes
yes

For peer review only

yes
yes
no
no
yes
no
yes
no
yes
yes
yes
yes
yes
yes
yes
yes
yes
yes
yes
yes
yes
yes
yes
yes
yes
yes
yes
yes
yes
yes
yes
yes
yes
yes
yes
yes
yes
yes
yes
yes
yes
yes
yes
yes
yes
yes
yes
no
no
no

For peer review only

yes
yes
yes
no
yes
yes
yes
no
yes
yes
yes
yes
no
yes
yes
yes
yes
yes
yes
yes
yes
yes
yes
yes
yes
yes
yes
no
yes
yes
no
yes
yes
yes
yes
yes
yes
yes
yes
yes
no
yes
yes
yes
yes
yes
yes
yes
no
no

For peer review only

yes
yes
yes
yes
yes
no
yes
no
yes
yes
yes
no
no
yes
yes
yes
yes
yes
no
yes
yes
no
yes
no
no
yes
yes
yes
no
no
no
no
yes
yes
yes
no
yes
yes
no
yes
yes
no
no
no
no
yes
yes
yes
no
yes

For peer review only

no
yes
yes
yes
no
no
yes
no
yes
no
yes
yes
no
no
no
yes
no
yes
yes
yes
yes
yes
yes
yes
yes
yes
yes
no
yes
yes
no
yes
yes
yes
yes
yes
yes
yes
yes
no
yes
yes
yes
no
yes
yes
yes
no
no
yes

For peer review only

yes
yes
no
no
no
no
yes
no
yes
yes
yes
no
no
yes
yes
yes
yes
yes
no
no
no
yes
no
yes
yes
yes
no
no
no
yes
yes
yes
yes
yes
no
no
yes
yes
yes
yes
no
yes
no
yes
yes
yes
yes
yes
no
yes

For peer review only

yes
yes
no
yes
yes
yes
yes
yes
yes
no
no
yes
yes
no
yes
yes
no
no
yes
no
yes
yes
no
yes
yes
yes
yes
yes
no
yes
no
yes
yes
yes
yes
yes
no
yes
yes
no
no
no
yes
no
no
no
no
yes
no
no

For peer review only

no
no
yes
no
no
yes
yes
yes
yes
no
no
yes
no
no
yes
no
yes
yes
yes
yes
yes
no
no
yes
yes
no
no
no
no
yes
no
yes
no
yes
yes
no
no
no
yes
no
yes
yes
no
yes
yes
no
no
yes
yes
yes

For peer review only

yes
yes
no
yes
no
yes
yes
yes
no
yes
no
no
yes
yes
no
yes
yes
yes
yes
yes
yes
yes
no
yes
yes
yes
no
yes
yes
yes
yes
yes
yes
yes
yes
no
yes
yes
no
no
no
yes
yes
no
yes
yes
yes
yes
yes
yes

yes
yes
no
yes
no
yes
yes
yes
yes
yes
no
yes
yes
no
yes
no
yes
no
no
no
yes
yes
no
yes
yes
no
yes
yes
yes
yes
yes
yes
yes
yes
yes
yes
yes
yes
yes
yes
yes
yes
yes
yes
yes
no
yes
yes
yes
yes

For peer review only

yes
yes
yes
yes
yes
yes
yes
yes
yes
yes
yes
yes
no
yes
yes
yes
yes
yes
yes
yes
no
yes
no
no
no
no
no
yes
no
yes
no
no
no
yes
yes
yes
no
yes
no
yes
no
yes
no
no
no
no
yes
yes
no
no
no

For peer review only

yes
no
yes
yes
no
yes
no
yes
yes
yes
yes
yes
yes
no
yes
no
no
yes
yes
no
yes
yes
no
yes
yes
no
no
yes
yes
yes
yes
yes
no
yes
yes
no
yes
no
yes
yes
yes
yes
yes
yes
yes
yes
yes
yes
yes
yes

For peer review only

yes
no
yes
yes
yes
yes
yes
yes
no
no
no
no
no
no
yes
yes
no
no
no
no
yes
no
yes
no
yes
yes
no
yes
yes
no
no
yes
no
no
no
yes
no
no
yes
yes
no
no
no
yes
yes
no
yes
yes
yes
no

For peer review only

no
no
yes
no
no
no
yes
no
no
yes
yes
no
yes
no
no
no
yes
yes
no
yes
yes
no
yes
no
no
yes
no
yes
yes
no
no
no
yes
yes
no
no
no
yes
yes
no
yes
yes
no
yes
yes
yes
no
no
yes
yes

For peer review only

yes
yes
no
yes
no
yes
yes
yes
yes
yes
yes
no
yes
yes
yes
yes
no
yes
no
yes
yes
yes
yes
yes
yes
no
no
no
yes
yes
yes
yes
no
no
no
no
no
no
yes
yes
yes
yes
yes
yes
yes
yes
no
yes
no
yes

For peer review only

yes
yes
yes
no
yes
yes
yes
no
no
yes
no
yes
yes
yes
no
yes
yes
yes
no
yes
yes
no
no
yes
yes
yes
no
no
yes
yes
yes
no
yes
yes
yes
yes
no
yes
yes
yes
yes
yes
yes
no
no
no
yes
no
yes
yes

For peer review only

no
no
no
yes
no
no
no
yes
no
yes
no
yes
yes
no
yes
yes
no
yes
yes
yes
yes
no
no
no
no
yes
yes
no
no
yes
no
no
yes
yes
no
yes
yes
yes
no
yes
yes
yes
yes
yes
yes
yes
yes
yes
yes
yes

For peer review only

yes
yes
yes
yes
no
yes
yes
no
yes
yes
no
yes
yes
yes
yes
yes
yes
no
no
no
yes
yes
yes
yes
yes
no
yes
yes
yes
yes
yes
yes
yes
yes
no
no
yes
no
yes
yes
yes
no
yes
yes
yes
yes
no
yes
yes
yes

For peer review only

no
yes
yes
yes
yes
no
no
yes
yes
yes
yes
no
yes
yes
yes
yes
yes
yes
no
yes
yes
no
yes
yes
yes
yes
yes
yes
yes
yes
yes
no
yes
yes
yes
yes
yes
no
yes
yes
yes
yes
yes
yes
yes
yes
yes
yes
yes
no

For peer review only

yes
yes
yes
yes
yes
yes
no
yes
no
no
yes
yes
yes
yes
yes
no
yes
yes
no
yes
yes
no
yes
no
yes
yes
yes
yes
yes
yes
yes
yes
yes
yes
yes
yes
yes
no
yes
yes
yes
yes
yes
yes
yes
yes
yes
no
yes
yes

For peer review only

yes
yes
yes
no
yes
yes
yes
yes
yes
yes
yes
yes
yes
yes
yes
yes
yes
yes
yes
yes
yes
yes
yes
yes
yes
yes
yes
yes
yes
yes
yes
yes
yes
yes
yes
yes
yes
yes
yes
yes
yes
yes
yes
yes
yes
yes
yes
no
yes
yes

For peer review only

yes
yes
yes
yes
yes
yes
yes
yes
yes
yes
yes
yes
yes
yes
no
yes
yes
yes
yes
yes
yes
yes
yes
no
yes
yes
yes
yes
yes
yes
yes
yes
yes
yes
yes
yes
yes
yes
yes
yes
yes
yes
yes
yes
yes
yes
yes
no
yes
yes

For peer review only

yes
yes
yes
no
yes
yes
yes
yes
yes
yes
yes
yes
yes
yes
yes
yes
yes
yes
yes
yes
no
yes
yes
yes
yes
yes
yes
yes
yes
no
yes
yes
yes
yes
yes
no
yes
no
no
yes
no
yes
no
yes
no
no
yes
yes
yes
yes
yes
no
no

For peer review only

no
yes
yes
yes
yes
no
yes
no
yes
yes
yes
yes
yes
yes
yes
yes
yes
yes
no
no
no
yes
yes
no
yes
yes
yes
yes
yes
yes
no
no
yes
yes
yes
yes
no
yes
no
no
yes
yes
yes
yes
yes
yes
no
no
yes
yes

For peer review only

yes
yes
no
yes
no
no
yes
no
yes
no
yes
no
yes
yes
yes
yes
yes
yes
yes
yes
yes
yes
yes
yes
yes
no
yes
yes
yes
yes
yes
no
yes
yes
yes
yes
yes
no
yes
yes
no
no
no
yes
no
yes
yes
yes
no
yes

For peer review only

yes
yes
no
yes
yes
yes
yes
no
yes
yes
no
yes
yes
no
yes
yes
yes
no
yes
yes
no
no
no
yes
yes
no
yes
yes
yes
yes
yes
yes
yes
yes
no
yes
no
no
no
yes
yes
yes
yes
yes
yes
yes
yes
no
yes
no

For peer review only

yes
yes
yes
no
no
yes
no
yes
yes
no
no
yes
no
yes
no
yes
yes
yes
yes
yes
yes
yes
no
no
no
yes
yes
yes
yes
yes
yes
no
yes
yes
yes
no
yes
yes
no
no
yes
yes
yes
yes
no
yes
yes
no
no
no

For peer review only

yes
yes
yes
yes
no
yes
yes
no
no
yes
yes
no
yes
yes
yes
yes
yes
yes
yes
yes
yes
no
yes
no
yes
no
yes
yes
yes
yes
yes
yes
yes
no
no
no
no
yes
yes
no
yes
yes
yes
yes
no
yes
no
yes
yes
yes

For peer review only

yes
yes
yes
yes
yes
yes
yes
yes
yes
yes
yes
yes
yes
yes
yes
yes
yes
yes
yes
yes
yes
yes
yes
yes
yes
no
yes
yes
yes
yes
no
yes
yes
yes
yes
yes
yes
yes
yes
no
no
yes
no
no
yes
yes
yes
yes
yes
yes

For peer review only

no
no
yes
yes
yes
yes
no
yes
yes
yes
yes
yes
yes
yes
yes
yes
yes
yes
yes
yes
yes
yes
yes
yes
yes
yes
yes
yes
yes
yes
yes
yes
yes
yes
yes
yes
yes
no
yes
yes
yes
no
no
yes
yes
yes
no
yes
no
yes

For peer review only

no
no
yes
yes
yes
yes
yes
yes
yes
yes
yes
yes
yes
yes
yes
yes
yes
yes
yes
no
yes
yes
yes
yes
yes
yes
yes
yes
yes
yes
yes
yes
yes
yes
yes
yes
yes
yes
yes
yes
yes
yes
yes
yes
yes
yes
yes
no
yes
yes

For peer review only

yes
yes
yes
yes
yes
yes
no
yes
yes
yes
yes
yes
yes
yes
yes
yes
yes
no
yes
yes
yes
yes
yes
yes
yes
yes
yes
yes
yes
yes
yes
no
yes
yes
yes
yes
yes
yes
yes
yes
yes
yes
no
yes
yes
yes
yes
yes
yes
yes

For peer review only

yes
yes
yes
yes
yes
yes
yes
yes
yes
yes
yes
yes
no
yes
yes
yes
yes
yes
no
yes
yes
yes
yes
no
yes
yes
yes
yes
yes
yes
no
yes
no
yes
yes
yes
no
no
yes
no
no
yes
no
no
yes
no
yes
yes
yes
yes

For peer review only

yes
yes
yes
yes
yes
yes
yes
yes
no
yes
yes
yes
yes
no
yes
yes
no
yes
no
yes
yes
yes
yes
yes
no
yes
yes
yes
no
yes
yes
yes
yes
yes
yes
no
yes
no
yes
yes
no
no
yes
yes
yes
yes
no
no
no
yes

For peer review only

no
yes
no
no
no
yes
yes
yes
yes
yes
yes
no
yes
yes
yes
no
yes
yes
yes
yes
no
yes
yes
yes
yes
yes
no
no
yes
no
no
no
no
yes
no
yes
no
yes
yes
yes
yes
yes
yes
yes
yes
no
yes
yes
yes
no

For peer review only

no
yes
no
yes
no
no
yes
no
yes
yes
no
no
no
no
yes
no
no
no
yes
no
yes
no
no
no
yes
yes
yes
yes
yes
yes
yes
yes
no
no
yes
no
no
no
no
no
yes
yes
yes
no
no
no
yes
yes
yes
no

For peer review only

no
yes
no
no
yes
no
no
no
yes
no
yes
yes
yes
yes
no
yes
yes
no
yes
yes
no
yes
no
no
yes
yes
no
no
no
yes
yes
no
yes
yes
no
no
no
yes
yes
yes
yes
no
yes
no
yes
yes
yes
no
no
yes

For peer review only

yes
no
no
no
no
no
yes
yes
no
no
yes
yes
yes
yes
no
yes
yes
yes
yes
yes
yes
no
yes
no
yes
yes
yes
yes
yes
yes
yes
yes
yes
yes
yes
no
yes
yes
yes
yes
no
no
no
yes
yes
no
yes
yes
yes
yes

For peer review only

no
yes
no
yes
no
yes
yes
yes
yes
yes
no
yes
no
yes
yes
no
yes
yes
yes
yes
yes
no
yes
yes
yes
yes
no
yes
yes
yes
no
yes
yes
yes
yes
yes
yes
yes
yes
yes
yes
no
yes
yes
yes
yes
no
yes
yes
yes

For peer review only

no
yes
yes
yes
yes
yes
yes
yes
no
yes
no
no
yes
yes
yes
yes
yes
yes
yes
yes
yes
no
yes
yes
no
yes
no
yes
no
yes
no
no
yes
no
no
yes
no
no
no
no
yes
no
yes
no
no
no
yes
yes
yes
no

For peer review only

yes
no
yes
yes
no
yes
yes
no
yes
yes
yes
no
yes
yes
yes
yes
yes
yes
yes
yes
yes
yes
yes
yes
yes
yes
yes
yes
yes
no
yes
yes
yes
yes
yes
yes
yes
yes
yes
yes
yes
yes
yes
yes
yes
no
yes
yes
yes
no

For peer review only

yes
yes
no
yes
yes
yes
yes
yes
yes
yes
yes
yes
yes
yes
yes
no
yes
no
yes
yes
no
yes
no
yes
yes
yes
yes
yes
yes
no
yes
yes
yes
yes
yes
yes
yes
yes
yes
yes
yes
yes
yes
yes
yes
yes
yes
yes
yes
yes

For peer review only

yes
yes
yes
yes
yes
no
yes
no
yes
yes
no
yes
yes
yes
yes
yes
no
yes
no
yes
no
no
yes
yes
yes
no
yes
no
yes
yes
no
no
no
yes
yes
yes
yes
yes
yes
yes
yes
no
yes
yes
no
yes
yes
no
yes
yes

For peer review only

no
yes
no
yes
yes
yes
yes
yes
yes
yes
yes
yes
yes
yes
yes
no
yes
yes
no
yes
no
yes
yes
yes
yes
yes
yes
yes
no
no
no
no
yes
yes
yes
yes
yes
no
yes
no
no
yes
yes
yes
yes
yes
yes
yes
yes
no

For peer review only

yes
yes
yes
no
yes
yes
yes
yes
yes
yes
yes
yes
yes
yes
no
no
yes
no
yes
yes
no
yes
yes
yes
yes
yes
yes
yes
yes
no
yes
yes
no
yes
yes
yes
yes
yes
no
yes
yes
yes
no
yes
yes
no
yes
yes
yes
yes

For peer review only

no
yes
yes
yes
yes
yes
yes
yes
yes
yes
yes
yes
no
yes
no
no
yes
yes
yes
no
yes
no
yes
no
yes
yes
yes
yes
no
yes
no
no
yes
no
no
yes
yes
yes
no
no
no
yes
no
no
yes
no
yes
no
no
yes

For peer review only

no
yes
no
yes
yes
yes
yes
yes
yes
yes
no
no
no
yes
no
no
yes
no
no
no
yes
yes
yes
yes
yes
no
yes
no
yes
no
no
yes
no
yes
yes
no
no
yes
no
yes
yes
no
yes
no
yes
yes
yes
yes
yes
yes

For peer review only

no
no
yes
yes
yes
yes
yes
yes
yes
yes
no
yes
yes
yes
yes
yes
yes
yes
no
yes
yes
no
yes
no
yes
yes
no
no
yes
no
no
no
yes
yes
no
yes
no
yes
yes
no
no
yes
no
no
yes
yes
yes
no
no
yes

For peer review only

no
yes
no
no
no
yes
yes
no
no
yes
yes
no
no
yes
no
no
yes
yes
yes
no
no
no
no
yes
yes
no
yes
yes
yes
yes
yes
yes
yes
yes
no
yes
yes
yes
yes
yes
yes
yes
yes
yes
yes
yes
yes
yes
yes
no

For peer review only

yes
yes
yes
yes
no
yes
yes
yes
yes
yes
yes
yes
yes
yes
yes
yes
yes
yes
yes
yes
yes
yes
yes
yes
yes
yes
no
yes
no
yes
yes
no
no
no
yes
yes
yes
yes
yes
no
no
yes
yes
no
no
yes
yes
yes
yes
no

For peer review only

yes
yes
no
yes
no
no
yes
no
no
yes
no
no
no
yes
yes
yes
yes
yes
no
yes
no
yes
no
yes
yes
yes
no
no
yes
yes
yes
yes
yes
no
no
yes
no
yes
yes
yes
yes
yes
yes
yes
yes
no
no
no
no
yes

For peer review only

yes
yes
no
no
yes
yes
yes
no
no
yes
no
no
no
yes
yes
no
yes
yes
no
yes
yes
no
no
yes
yes
yes
yes
yes
no
yes
no
yes
no
no
yes
no
no
no
no
no
no
yes
no
no
no
yes
no
no
no
no

For peer review only

yes
yes
no
no
no
yes
no
no
yes
yes
yes
no
yes
no
yes
no
no
no
no
no
yes
no
yes
yes
no
no
yes
yes
no
no
no
no
yes
no
no
no
yes
no
yes
yes
no
yes
no
yes
no
yes
yes
no
yes
yes

For peer review only

yes
no
no
yes
no
yes
yes
no
yes
no
yes
yes
no
yes
no
no
yes
no
yes
yes
no
yes
yes
no
no
yes
yes
yes
yes
no
yes
no
yes
yes
yes
no
no
yes
no
no
no
yes
yes
yes
no
yes
yes
yes
yes
yes

For peer review only

no
yes
yes
yes
no
yes
no
yes
no
yes
yes
yes
yes
yes
yes
yes
yes
yes
yes
yes
yes
yes
yes
yes
yes
yes
yes
yes
yes
yes
yes
yes
no
yes
yes
yes
no
yes
yes
no
yes
yes
yes
yes
no
yes
yes
yes
yes
no

For peer review only

no
yes
no
yes
yes
yes
yes
yes
no
yes
no
yes
yes
yes
yes
yes
yes
yes
no
yes
yes
no
no
yes
yes
yes
yes
yes
no
no
yes
yes
yes
yes
no
no
yes
yes
yes
yes
no
no
no
no
no
no
no
no
no
no

For peer review only

no
no
no
yes
yes
no
no
yes
no
yes
yes
no
no
yes
yes
yes
yes
no
yes
no
yes
yes
yes
yes
no
yes
yes
no
yes
no
yes
yes
no
no
yes
no
yes
yes
yes
yes
no
yes
yes
yes
no
yes
yes
no
yes
no

For peer review only

yes
yes
yes
yes
yes
yes
no
no
yes
yes
no
no
yes
yes
yes
no
yes
yes
yes
yes
yes
yes
no
yes
yes
yes
yes
yes
yes
yes
yes
yes
yes
yes
yes
no
yes
yes
yes
no
yes
no
yes
yes
yes
yes
no
yes
yes
yes

For peer review only

no
no
yes
yes
no
no
yes
no
no
yes
no
yes
yes
yes
no
yes
yes
yes
no
yes
yes
yes
no
yes
yes
yes
no
yes
yes
yes
yes
yes
yes
yes
yes
yes
yes
yes
no
yes
yes
yes
yes
yes
no
no
yes
yes
yes
yes

yes
no
yes
no
no
no
no
no
no
yes
yes
no
yes
yes
yes
no
no
no
yes
yes
yes
yes
yes
yes
yes
yes
yes
no
no
no
yes
no
yes
no
no
yes
yes
yes
yes
yes
no
yes
no
yes
yes
yes
yes
no
yes
yes

For peer review only

yes
yes
no
yes
yes
no
yes
no
no
no
no
no
yes
no
yes
no
yes
yes
no
yes
yes
yes
yes
no
no
no
yes
yes
yes
no
no
no
yes
yes
no
no
yes
no
yes
yes
yes
yes
no
yes
yes
no
yes
no
yes
yes

For peer review only

no
no
no
yes
no
no
no
yes
no
yes
yes
no
yes
yes
no
yes
no
yes
yes
yes
no
yes
yes
yes
yes
yes
yes
yes
yes
yes
yes
no
yes
yes
no
yes
yes
no
no
yes
no
yes
no
yes
yes
yes
yes
yes
yes
yes

For peer review only

yes
yes
yes
yes
yes
no
no
no
no
yes
yes
yes
no
no
no
yes
no
no
no
no
yes
yes
yes
yes
no
yes
yes
yes
yes
no
yes
yes
no
yes
no
yes
yes
yes
yes
yes
no
yes
no
yes
yes
yes
no
yes
yes
no

For peer review only

no
no
no
yes
no
yes
yes
no
yes
no
yes
yes
yes
no
yes
no
yes
yes
no
yes
yes
yes
no
yes
no
yes
no
yes
yes
no
no
no
no
yes
yes
no
yes
yes
no
yes
no
yes
no
no
yes
yes
no
no
no
no

For peer review only

yes
no
yes
no
no
yes
yes
yes
yes
yes
yes
yes
no
yes
yes
yes
no
yes
yes
yes
yes
yes
yes
no
yes
yes
yes
yes
yes
yes
yes
yes
no
no
yes
yes
no
yes
yes
yes
yes
no
yes
yes
yes
no
yes
yes
yes
yes

For peer review only

no
yes
yes
yes
yes
yes
yes
yes
yes
no
yes
no
yes
yes
yes
no
yes
yes
no
yes
yes
no
no
yes
yes
no
yes
no
yes
yes
yes
yes
yes
yes
yes
yes
yes
yes
no
no
yes
yes
yes
yes
no
yes
yes
yes
yes
yes

For peer review only

yes
yes
no
yes
no
no
no
yes
yes
no
yes
yes
no
no
yes
yes
yes
no
yes
yes
no
no
no
yes
yes
yes
no
no
no
no
yes
yes
no
yes
yes
no
no
yes
yes
no
no
no
yes
yes
no
yes
yes
yes
no
yes

For peer review only

yes
no
yes
yes
no
yes
yes
no
yes
yes
yes
yes
no
yes
yes
no
yes
yes
no
yes
yes
yes
yes
no
yes
yes
yes
no
yes
no
no
yes
yes
yes
no
yes
no
yes
no
yes
no
no
no
yes
yes
no
no
yes
yes
yes

For peer review only

yes
yes
no
no
no
no
yes
no
yes
no
no
yes
no
no
no
yes
no
yes
no
no
no
no
yes
no
yes
yes
no
no
yes
no
yes
yes
no
yes
no
no
no
yes
yes
no
no
yes
no
yes
no
yes
no
yes
yes
no

For peer review only

yes
yes
no
no
yes
no
yes
no
no
no
no
yes
yes
yes
no
yes
yes
yes
no
yes
yes
yes
yes
no
yes
no
no
no
yes
yes
yes
no
yes
yes
yes
yes
yes
yes
yes
no
yes
yes
yes
no
no
yes
yes
no
yes
no

For peer review only

yes
yes
yes
yes
yes
no
no
no
no
no
no
no
yes
no
yes
yes
no
yes
no
no
yes
yes
no
no
yes
yes
yes
yes
yes
no
yes
no
no
yes
no
no
yes
yes
yes
yes
yes
yes
yes
yes
yes
yes
yes
yes
yes
no

For peer review only

no
yes
yes
yes
no
no
yes
yes
yes
yes
yes
yes
yes
no
no
yes
yes
yes
no
yes
yes
no
no
yes
no
yes
no
yes
yes
yes
yes
no
yes
yes
no
no
no
yes
yes
no
yes
yes
no
no
yes
yes
yes
no
yes
yes

For peer review only

yes
no
yes
yes
no
yes
yes
yes
yes
no
yes
yes
yes
no
no
no
yes
yes
yes
yes
yes
no
yes
no
yes
yes
yes
yes
no
no
no
yes
yes
no
no
yes
yes
yes
yes
yes
yes
no
yes
yes
no
no
yes
no
no
yes

For peer review only

yes
no
no
no
yes
yes
yes
yes
no
yes
no
yes
yes
no
yes
yes
no
yes
no
yes
no
no
yes
yes
yes
yes
yes
yes
yes
yes
no
no
yes
yes
yes
yes
no
no
yes
yes
yes
yes
yes
yes
yes
yes
no
yes
yes
yes

For peer review only

no
no
yes
yes
yes
yes
yes
no
yes
yes
yes
yes
yes
yes
yes
yes
no
yes
yes
yes
yes
yes
no
yes
yes
no
no
no
yes
yes
yes
yes
no
yes
yes
no
no
no
no
no
yes
yes
yes
no
no
yes
no
no
no
no

For peer review only

no
no
yes
yes
no
no
yes
yes
no
yes
no
yes
no
yes
yes
yes
no
yes
no
yes
yes
no
yes
yes
yes
yes
yes
yes
yes
yes
yes
yes
yes
yes
yes
yes
yes
yes
yes
yes
yes
yes
no
yes
no
yes
yes
yes
no
yes

For peer review only

yes
yes
no
yes
yes
yes
yes
yes
yes
yes
yes
yes
yes
no
yes
yes
no
no
yes
no
yes
yes
no
no
yes
no
yes
yes
no
no
yes
no
no
no
yes
no
no
no
no
no
yes
yes
yes
no
yes
yes
yes
yes
yes
yes

For peer review only

yes
yes
no
yes
yes
no
no
yes
no
yes
yes
yes
no
no
no
no
no
no
no
no
no
no
no
yes
yes
yes
no
no
no
yes
yes
yes
no
yes
yes
no
yes
no
yes
yes
no
no
yes
no
yes
no
no
yes
no
no

For peer review only

yes
yes
no
no
no
no
yes
yes
yes
yes
yes
yes
yes
yes
yes
yes
yes
yes
yes
yes
yes
yes
yes
yes
yes
yes
no
yes
yes
no
yes
no
yes
yes
yes
no
no
yes
yes
no
yes
yes
no
yes
yes
yes
no
yes
no
no

For peer review only

yes
yes
no
yes
yes
yes
no
yes
no
yes
no
no
no
no
yes
yes
yes
no
no
yes
yes
no
no
yes
no
no
yes
yes
yes
yes
yes
yes
yes
no
no
yes
yes
yes
yes
no
no
no
no
no
yes
no
no
yes
no
yes

For peer review only

no
no
no
yes
no
yes
no
yes
no
yes
no
yes
no
no
yes
yes
no
yes
yes
yes
yes
no
yes
yes
yes
no
yes
yes
no
yes
no
no
no
yes
yes
no
no
yes
no
yes
yes
no
no
yes
no
no
yes
no
yes
yes

For peer review only

yes
yes
yes
yes
yes
yes
no
yes
no
no
yes
yes
yes
no
yes
yes
yes
yes
yes
yes
yes
yes
no
yes
yes
yes
yes
no
no
yes
yes
yes
no
yes
yes
yes
yes
no
yes
yes
yes
yes
no
yes
yes
yes
no
no
yes
no

For peer review only

yes
no
yes
yes
no
yes
yes
no
yes
yes
no
yes
no
no
yes
yes
yes
yes
yes
no
no
no
yes
yes
no
no
yes
yes
yes
no
yes
no
yes
no
yes
no
yes
yes
yes
yes
no
yes
no
yes
no
yes
yes
no
yes
yes

For peer review only

no
yes
yes
yes
yes
no
no
yes
no
yes
yes
yes
yes
yes
no
yes
no
yes
yes
no
yes
no
no
yes
no
no
no
yes
no
no
yes
yes
yes
yes
no
no
yes
yes
yes
yes
yes
yes
yes
no
yes
yes
no
yes
yes
no

For peer review only

yes
yes
no
yes
no
yes
yes
yes
yes
yes
yes
yes
no
no
no
yes
yes
no
yes
no
no
yes
no
yes
yes
yes
yes
yes
yes
no
yes
no
no
yes
no
yes
yes
yes
yes
yes
yes
no
yes
yes
yes
yes
yes
yes
yes
yes

For peer review only

yes
yes
yes
no
yes
no
no
yes
no
yes
no
yes
no
yes
no
no
yes
no
yes
no
yes
yes
yes
yes
no
no
no
no
no
no
no
no
no
yes
yes
yes
yes
no
yes
yes
no
yes
yes
yes
yes
yes
yes
no
no
no
no
no

For peer review only

yes
yes
yes
yes
yes
yes
no
yes
yes
yes
yes
no
no
yes
yes
no
no
no
no
no
no
yes
no
no
yes
no
yes
yes
no
yes
yes
yes
no
no
yes
yes
yes
yes
yes
yes
yes
no
yes
yes
yes
yes
yes
yes
yes
no

For peer review only

yes
yes
yes
yes
yes
yes
no
no
yes
no
yes
yes
no
no
no
yes
no
no
no
yes
no
no
yes
no
yes
no
yes
yes
yes
no
no
yes
no
yes
no
yes
no
no
no
yes
yes
no
yes
yes
yes
yes
no
yes
yes
yes

For peer review only

yes
no
yes
yes
no
no
no
yes
yes
no
no
no
no
yes
no
yes
no
no
yes
no
no
no
yes
yes
no
yes
yes
no
yes
yes
yes
no
yes
yes
yes
no
no
no
no
yes
no
yes
yes
no
no
no
no
yes
no
yes

For peer review only

no
no
yes
no
no
no
no
yes
yes
no
no
no
no
yes
no
yes
no
no
yes
no
no
no
yes
yes
no
no
no
no
no
no
no
yes
yes
no
no
yes
no
no
no
yes
no
no
no
yes
no
no
no
yes
yes
no

For peer review only

no
yes
yes
yes
yes
yes
yes
yes
yes
no
no
yes
yes
no
yes
no
yes
yes
yes
yes
no
no
yes
no
yes
yes
no
yes
no
no
yes
no
no
no
yes
yes
no
no
no
no
yes
no
yes
yes
yes
no
no
no
yes
no

For peer review only

yes
yes
yes
no
no
yes
no
yes
no
no
no
yes
yes
no
no
no
yes
yes
no
yes
yes
yes
yes
yes
yes
yes
yes
yes
yes
yes
no
yes
no
yes
no
yes
yes
yes
yes
yes
no
yes
yes
yes
yes
yes
no
yes
yes
no

For peer review only

yes
no
yes
no
yes
yes
yes
no
yes
yes
yes
no
no
yes
no
no
yes
yes
no
no
no
yes
yes
no
yes
no
yes
no
no
yes
yes
no
no
yes
yes
yes
yes
yes
yes
yes
no
yes
no
yes
yes
yes
no
yes
yes
yes

For peer review only

For peer review only

yes
no
yes
yes
yes
yes
yes
yes
yes
yes
yes
yes
yes
yes
yes
yes
yes
yes
yes
yes
yes
yes
yes
yes
yes
yes
yes
yes
yes
no
yes
yes
yes
yes
yes
no
yes
no
yes
yes
no
no
yes
yes
yes
yes
yes
yes
no
yes

yes
no
yes
no
yes
yes
yes
yes
yes
yes
yes
no
yes
yes
yes
yes
no
no
yes
yes
yes
yes
yes
yes
yes
no
no
yes
yes
yes
yes
yes
yes
yes
yes
yes
yes
yes
yes
no
yes
yes
yes
yes
no
no
yes
no
yes
yes

For peer review only

no
yes
yes
yes
yes
yes
yes
yes
yes
yes
yes
yes
yes
yes
yes
yes
yes
no
yes
no
no
yes
yes
yes
no
yes
no
yes
yes
yes
yes
yes
no
yes
yes
yes
yes
yes
yes
yes
yes
no
yes
yes
yes
yes
yes
yes
yes
yes

For peer review only

yes
yes
yes
yes
no
yes
yes
yes
yes
yes
yes
yes
yes
yes
no
yes
no
yes
no
yes
yes
yes
yes
yes
no
yes
yes
yes
yes
yes
yes
yes
yes
yes
yes
yes
yes
yes
yes
yes
yes
yes
yes
yes
yes
yes
yes
no
no
no

For peer review only

no
yes
yes
yes
no
yes
yes
yes
yes
yes
yes
yes
yes
yes
yes
yes
yes
yes
no
yes
no
no
yes
yes
yes
yes
yes
yes
yes
yes
yes
yes
yes
yes
no
yes
no
yes
yes
yes
yes
yes
yes
yes
yes
yes
no
yes
yes
yes

For peer review only

yes
yes
yes
yes
yes
yes
yes
yes
no
yes
yes
yes
yes
yes
yes
yes
no
yes
yes
yes
yes
yes
yes
yes
no
yes
yes
yes
yes
yes
yes
yes
yes
no
no
yes
yes
yes
no
yes
no
no
no
no
yes
no
yes
no
yes
no

For peer review only

no
yes
no
yes
yes
no
no
yes
yes
no
yes
no
yes
no
yes
no
yes
no
yes
yes
no
no
yes
yes
yes
yes
no
yes
yes
yes
yes
no
no
yes
yes
no
no
yes
yes
yes
no
yes
yes
no
yes
no
yes
yes
yes
yes

For peer review only

no
yes
no
no
no
no
no
no
no
yes
yes
no
yes
no
yes
yes
yes
no
yes
yes
no
yes
yes
no
no
yes
no
yes
yes
yes
yes
no
yes
yes
yes
no
yes
yes
yes
yes
yes
yes
no
yes
yes
yes
yes
no
yes
no

For peer review only

no
yes
yes
yes
yes
yes
yes
yes
yes
yes
yes
yes
yes
yes
yes
yes
no
yes
yes
yes
yes
yes
yes
yes
no
no
yes
no
yes
yes
yes
no
no
yes
yes
yes
yes
yes
yes
yes
yes
yes
yes
no
no
no
yes
yes
yes
no

For peer review only

no
yes
no
yes
yes
no
yes
no
yes
no
yes
yes
yes
yes
yes
yes
no
no
yes
yes
yes
yes
yes
yes
yes
no
yes
no
yes
no
yes
yes
yes
yes
yes
yes
yes
yes
yes
yes
yes
no
yes
yes
yes
yes
yes
yes
yes
yes

For peer review only

yes
yes
yes
yes
yes
yes
yes
yes
no
yes
no
yes
yes
yes
no
yes
yes
yes
no
yes
yes
no
yes
no
no
yes
no
yes
yes
no
yes
yes
no
yes
yes
no
yes
yes
yes
yes
yes
no
yes
no
no
yes
yes
yes
yes
yes

For peer review only

yes
yes
yes
yes
yes
no
yes
yes
yes
no
no
no
no
yes
no
yes
yes
yes
yes
no
no
yes
yes
yes
yes
yes
no
no
no
yes
yes
no
yes
no
no
no
no
yes
yes
yes
yes
yes
yes
yes
yes
no
no
no
yes
yes

For peer review only

yes
yes
yes
yes
yes
no
yes
no
yes
no
yes
no
yes
no
no
yes
no
no
no
no
yes
no
no
no
no
no
no
no
yes
yes
yes
no
yes
yes
yes
yes
no
yes
yes
no
no
no
no
yes
yes
yes
yes
yes
yes
yes

For peer review only

yes
yes
yes
yes
no
yes
no
yes
yes
yes
yes
yes
yes
yes
no
no
no
no
no
no
yes
no
yes
yes
yes
yes
yes
no
no
yes
yes
yes
yes
no
yes
yes
yes
no
yes
no
yes
yes
no
yes
no
yes
no
no
yes
no

For peer review only

no
no
yes
no
yes
yes
yes
yes
no
no
no
no
no
yes
no
yes
yes
yes
yes
no
no
yes
yes
yes
no
yes
yes
yes
yes
yes
yes
no
yes
yes
yes
yes
no
no
yes
yes
no
no
no
yes
yes
no
no
yes
no
no

For peer review only

no
no
no
yes
yes
yes
yes
yes
no
yes
yes
yes
yes
no
no
yes
no
yes
yes
yes
yes
yes
yes
no
yes
no
yes
yes
yes
no
no
no
yes
no
no
yes
yes
no
yes
no
no
yes
no
yes
no
no
no
no
yes
no

For peer review only

yes
no
yes
yes
no
yes
yes
no
yes
no
no
no
yes
no
no
yes
yes
yes
yes
no
yes
no
no
yes
no
yes
yes
yes
yes
yes
yes
yes
no
yes
yes
yes
yes
no
yes
yes
yes
no
yes
yes
yes
yes
yes
yes
no
yes

For peer review only

yes
yes
yes
no
yes
yes
yes
yes
yes
yes
yes
yes
yes
no
no
no
no
no
yes
no
yes
yes
no
no
no
no
no
yes
no
yes
yes
yes
no
yes
no
no
yes
yes
no
no
yes
yes
yes
no
no
no
no
yes
yes
no

For peer review only

yes
yes
no
yes
no
no
no
yes
no
no
yes
no
no
no
no
yes
yes
no
yes
no
yes
no
no
no
yes
yes
yes
yes
yes
no
yes
yes
no
no
yes
yes
yes
yes
no
yes
yes
no
no
yes
yes
no
no
no
no
no

For peer review only

yes
no
no
yes
yes
yes
yes
no
yes
yes
yes
no
no
yes
yes
no
yes
yes
no
yes
no
yes
yes
no
no
yes
no
no
no
no
no
yes
no
yes
no
no
yes
yes
yes
yes
yes
yes
yes
no
no
yes
yes
no
yes
yes

For peer review only

no
no
no
yes
no
yes
yes
yes
no
no
no
no
yes
yes
yes
yes
no
no
yes
no
yes
yes
yes
yes
no
yes
yes
yes
yes
yes
no
yes
no
yes
no
yes
yes
no
yes
yes
no
yes
yes
no
yes
yes
yes
yes
yes
no

For peer review only

no
yes
yes
no
no
no
yes
yes
yes
no
no
no
no
yes
yes
yes
no
no
no
no
yes
no
yes
yes
yes
no
yes
yes
yes
yes
yes
yes
yes
yes
yes
yes
no
no
yes
yes
yes
yes
no
yes
yes
yes
yes
no
yes
yes

For peer review only

yes
yes
yes
yes
yes
yes
yes
no
no
yes
yes
yes
yes
yes
no
no
no
yes
yes
yes
no
yes
no
yes
yes
yes
yes
no
no
no
no
no
no
no
yes
no
no
no
yes
yes
yes
yes
yes
yes
yes
yes
yes
yes
yes
yes

For peer review only

yes
yes
yes
yes
yes
yes
no
yes
no
yes
yes
yes
yes
yes
no
yes
yes
yes
yes
yes
no
yes
yes
no
no
yes
no
yes
no
yes
no
yes
yes
yes
yes
yes
no
yes
yes
no
yes
yes
yes
yes
yes
no
no
yes
no
yes

For peer review only

yes
yes
yes
no
no
yes
yes
yes
yes
no
yes
yes
no
no
no
yes
no
no
no
yes
no
no
yes
yes
yes
no
yes
yes
yes
yes
yes
yes
yes
yes
yes
yes
no
no
yes
yes
yes
yes
no
no
no
no
yes
yes
yes
yes

For peer review only

yes
no
no
yes
no
no
no
no
no
no
no
no
yes
no
no
yes
no
yes
yes
yes
yes
no
yes
yes
no
no
no
yes
yes
yes
yes
yes
yes
yes
yes
no
no
no
yes
no
yes
yes
no
no
yes
no
no
yes
no
yes

For peer review only

yes
yes
no
no
yes
yes
no
yes
yes
no
yes
no
yes
yes
yes
no
no
yes
yes
yes
yes
no
yes
yes
no
yes
yes
yes
yes
yes
yes
yes
yes
yes
yes
yes
no
yes
yes
yes
yes
yes
yes
no
yes
no
no
no
yes
no

For peer review only

yes
yes
yes
yes
yes
yes
yes
no
yes
yes
yes
yes
no
yes
yes
no
no
yes
yes
yes
yes
yes
yes
yes
yes
yes
yes
no
no
yes
no
yes
yes
yes
yes
yes
yes
no
yes
yes
yes
yes
yes
no
yes
yes
yes
yes
no
no

For peer review only

no
yes
yes
no
yes
yes
yes
yes
yes
yes
yes
yes
yes
yes
no
no
yes
yes
no
yes
no
yes
yes
no
no
yes
yes
yes
yes
yes
yes
no
yes
yes
yes
yes
yes
no
no
no
yes
yes
yes
no
yes
yes
no
no
no
no

For peer review only

no
yes
yes
no
yes
no
no
yes
yes
yes
no
no
yes
yes
yes
yes
no
no
yes
yes
yes
yes
no
no
no
no
yes
no
no
yes
yes
no
no
no
no
no
yes
no
no
no
no
yes
yes
no
yes
yes
no
no
yes
yes

For peer review only

yes
yes
yes
yes
yes
no
yes
yes
no
yes
yes
no
yes
yes
yes
no
yes
yes
yes
no
yes
no
yes
yes
yes
no
no
no
yes
no
yes
no
yes
no
yes
yes
yes
yes
yes
yes
yes
no
yes
yes
yes
no
yes
yes
no
yes

For peer review only

yes
no
no
yes
yes
yes
yes
yes
yes
yes
yes
no
yes
yes
yes
yes
yes
no
yes
yes
yes
no
no
yes
yes
yes
yes
yes
yes
yes
yes
yes
yes
yes
yes
no
yes
no
yes
yes
yes
yes
yes
yes
no
no
yes
no
yes
no

For peer review only

yes
no
yes
yes
no
yes
no
no
no
yes
no
no
no
yes
no
no
no
yes
yes
yes
yes
yes
no
yes
yes
yes
yes
yes
yes
yes
yes
yes
yes
yes
yes
no
no
yes
yes
yes
yes
yes
no
yes
yes
no
no
no
yes
yes

For peer review only

yes
yes
no
no
no
no
yes
yes
no
no
yes
no
no
yes
yes
no
no
no
no
no
yes
no
yes
no
yes
no
no
no
no
yes
no
yes
no
no
no
yes
no
yes
yes
yes
no
yes
no
yes
yes
yes
yes
yes
yes
yes

For peer review only

no
no
yes
yes
yes
yes
no
yes
yes
no
no
no
yes
no
no
yes
yes
yes
no
no
yes
no
yes
yes
yes
yes
yes
no
yes
yes
no
no
yes
no
yes
no
no
no
no
no
yes
yes
no
no
yes
no
no
yes
yes
yes

For peer review only

yes
yes
yes
yes
yes
yes
yes
yes
no
no
no
yes
yes
yes
no
yes
yes
yes
yes
yes
yes
yes
no
yes
yes
yes
no
yes
yes
yes
yes
no
no
no
yes
yes
yes
no
no
no
no
yes
yes
yes
no
yes
yes
yes
yes
yes

no
yes
yes
yes
yes
yes
yes
yes
no
yes
no
no
no
yes
yes
yes
yes
yes
yes
yes
yes
no
yes
yes
no
yes
no
yes
yes
no
no
yes
yes
no
no
yes
no
yes
no
yes
yes
no
no
yes
yes
yes
no
yes
yes
no

For peer review only

yes
yes
yes
yes
no
yes
yes
yes
yes
yes
yes
yes
no
yes
yes
yes
no
yes
yes
yes
yes
no
no
yes
yes
yes
yes
yes
yes
no
yes
yes
yes
yes
yes
yes
no
yes
yes
yes
yes
yes
yes
yes
yes
yes
no
no
yes
yes

For peer review only

no
yes
yes
yes
yes
no
no
yes
no
no
yes
no
yes
yes
yes
no
no
yes
yes
yes
yes
yes
yes
yes
no
yes
yes
yes
yes
yes
yes
no
yes
yes
no
yes
yes
yes
yes
yes
yes
no
yes
yes
yes
yes
yes
no
no
yes

For peer review only

no
no
yes
yes
no
yes
yes
yes
no
no
no
yes
yes
yes
no
yes
yes
yes
no
no
yes
yes
no
no
yes
yes
yes
yes
yes
yes
yes
no
no
yes
yes
yes
no
yes
yes
no
no
yes
yes
no
no
no
yes
yes
yes
yes

For peer review only

yes
no
yes
no
yes
yes
yes
yes
yes
yes
yes
yes
no
no
yes
yes
yes
yes
yes
no
yes
yes
no
no
yes
no
yes
yes
yes
no
no
yes
yes
yes
yes
yes
no
no
yes
yes
no
yes
no
yes
yes
yes
yes
yes
no
no

For peer review only

yes
no
yes
yes
yes
no
no
yes
no
yes
yes
yes
no
yes
no
yes
yes
yes
no
yes
yes
yes
yes
no
no
yes
yes
yes
no
no
yes
no
no
yes
yes
no
yes
no
no
yes
no
yes
yes
no
yes
yes
yes
yes
yes
yes

For peer review only

yes
yes
no
no
no
no
no
yes
yes
no
yes
yes
yes
yes
yes
yes
no
no
no
yes
no
no
no
no
yes
no
no
no
no
yes
no
yes
no
yes
yes
yes
yes
yes
yes
no
yes
yes
no
yes
no
no
no
no
yes
no

For peer review only

yes
no
yes
yes
yes
yes
yes
yes

For peer review only

BMJ Open

How large should a cause of death be in order to be included in mortality trend analysis? deriving a cut-off point from retrospective trend analyses in 21 European countries

Journal:	*BMJ Open*
Manuscript ID	bmjopen-2019-031702.R1
Article Type:	Original research
Date Submitted by the Author:	22-Nov-2019
Complete List of Authors:	Mitratza, Marianna; Amsterdam UMC, University of Amsterdam, Department of Public Health<br>Kardaun, Jan; Statistics Netherlands, Department of Health and Care, ; Amsterdam UMC, University of Amsterdam, Department of Public Health<br>Kunst, Anton; Amsterdam UMC, University of Amsterdam, Department of Public Health
**Primary Subject Heading**:	Epidemiology
Secondary Subject Heading:	Public health, Research methods
Keywords:	mortality, trends, cause of death, size criterion, Europe, EPIDEMIOLOGY

SCHOLARONE™
Manuscripts

Title

How large should a cause of death be in order to be included in mortality trend analysis? deriving a cut-off point from retrospective trend analyses in 21 European countries

Authors

Marianna Mitratza, Department of Public Health, Amsterdam UMC, University of Amsterdam, Amsterdam, the Netherlands

Jan WPF Kardaun, Department of Health and Care, Statistics Netherlands, The Hague, the Netherlands
& Department of Public Health, Amsterdam UMC, University of Amsterdam, Amsterdam, the Netherlands

Anton E Kunst, Department of Public Health, Amsterdam UMC, University of Amsterdam, Amsterdam, the Netherlands

Author responsible for correspondence about the manuscript

Marianna Mitratza

Address: Meibergdreef 9, 1105 AZ Amsterdam, Netherlands

Telephone : +31205664658

Email : m.mitratza@amsterdamumc.nl

Disclaimers

None

Funding

For peer review only - http://bmjopen.bmj.com/site/about/guidelines.xhtml

None

Word count

2762

Number of figures and tables

2 tables, 3 figures

For peer review only

For peer review only - http://bmjopen.bmj.com/site/about/guidelines.xhtml

Abstract

Objectives: The International Classification of Diseases (ICD-10) distinguishes a large number of causes of death (CODs) that could each be studied individually when monitoring time-trends. We aimed to develop recommendations for using the size of CODs as a criterion for their inclusion in long-term trend analysis.

Design: Retrospective trend analysis

Setting: 21 European countries of the WHO Mortality Database

Participants: Deaths from causes of death (3-position ICD10 codes) with ≥5 average annual deaths in a 15-year period between 2000 and 2016

Primary and secondary outcome measures: Fitting polynomial regression models, we examined for each COD in each country whether or not changes over time were statistically significant (with α=0.05).and we assessed correlates of this outcome. Applying ROC curve diagnostics, we derived COD size thresholds for selecting CODs for trends analysis.

Results: Across all countries, 64.0% of CODs had significant long-term trends. The odds of having a significant trend increased by 18% for every 10% increase of COD size. The independent effect of country was negligible. As compared to circulatory system diseases, the probability of a significant trend was lower for neoplasms and digestive system diseases, and higher for infectious diseases, mental diseases and signs-and-symptoms. We derived a general threshold of around 30 (range: 28-33) annual deaths for inclusion of a COD in trend analysis. The relevant threshold for neoplasms was around 65 (range: 61-70) and for infectious diseases was 20 (range: 19-20).

For peer review only

Conclusions: The likelihood that long-term trends are detected with statistical significance is strongly related to COD size and varies between ICD-10 chapters, but has no independent relation to country. We recommend a general size criterion of 30 annual deaths to select CODs for long-term mortality-trends analysis in European countries.

Strengths and limitations of this study

- The first study to develop a criterion to select causes of death for monitoring purposes based on their annual number of deaths.
- The use of a large sample of causes of death covering most European countries, using the WHO Mortality Database.
- Criteria for selection of causes were derived for different types of causes of death.
- Other criteria were not applied, such as causes of death that involve high healthcare costs or that are potentially modifiable.

Introduction

Mortality data are essential for the monitoring of population-wide trends in a large number of diseases and injuries, as well as for the evaluation of health policies. A common source for these data are the statistics maintained by national statistical offices **[1,2]**. National statistics of causes of death (CODs) include many codes of the10th revision of the International Classification of Diseases (ICD-10 codes) **[3].** Given the detail of this classification - there are 1,752 3-position ICD-10 codes - a part of it may not be instrumental for monitoring long-term time-trends due to the small number of deaths for specific codes.

When using these statistics to monitor long-term trends in mortality, a main question is which of the many possible CODs to include. At the very least, the selection should include only

For peer review only - http://bmjopen.bmj.com/site/about/guidelines.xhtml

CODs that are large enough to have a reasonable probability of detecting a long-term mortality trend. This probability may be influenced by several factors. One main factor is the COD size, defined as the mean annual number of deaths, which expresses the rarity of a disease or condition that is selected as underlying cause of death in a population. Incidence changes or effects of interventions are common factors discussed in mortality trends analyses **[4,5]**. In addition, this probability might depend on other factors, such as the type of COD, or the country of interest. Certain types of CODs may be more likely to present a long-term trend. For example, neoplasms have been shown to be more gradual in their annual changes **[6]**, whereas infectious diseases **[7]** may have high year-to-year variation. As regards to different populations, the likelihood to detect a long-term trend for a COD may vary between countries because of differences in population size, COD coding practices that may also influence observed mortality trends **[8]**, trends in prevalence of risk factors **[9,10,11,12]**, implementation of new prevention strategies **[13,14]**, treatment protocols **[5]** or healthcare reforms **[15]**.

Due to the fact that the likelihood to detect a long-term trend of a COD may depend on various factors, there is a need for an empirical assessment of such likelihood. Such analysis may provide an empirical basis for the identification of CODs for which long-trends are likely to be detectable. More specifically, it may be used to define a criterion, or rule of thumb, that identifies eligible CODs in terms of a minimum COD size. When such a criterion allows for variation by COD type and country, it may be used in national and international trend analysis across a broad range of CODs.

The general objective of this study was to determine a COD size criterion for the study of long-term mortality trends in European countries. The specific objectives were: (1) to assess the association between the size and the type of a COD and the probability of detecting a long-term trend in European countries, (2) to assess how this association varies according to country, and (3) to identify a minimum annual number of deaths recommended to monitor trends in cause-specific mortality.

For peer review only - http://bmjopen.bmj.com/site/about/guidelines.xhtml

Methods

Data

We used annual mortality data for 21 European countries of the WHO Mortality Database (1 October 2017 update) **[16]**. We included the 21 countries of the European Union (28 countries) or the European Free Trade Association (4 countries) that had been using ICD-10 (3- or 4-position) coding for at least 15 consecutive years. Iceland, Luxemburg and Malta were excluded because of their small population **[17]**. The most recent 15-year period was selected, which was 2001-2015 for all countries with few exceptions (Belgium, France and Switzerland: 2000-2014; Austria: 2002-2016). If the time series of a COD in a country was interrupted by a year without any data on that COD, we assumed that zero cases occurred.

Statistical analysis

For each year and COD in a country, we calculated an age-standardized count of deaths using the direct method. As reference population, we used the age-distribution of the European Standard Population 2013, scaled to the mid-period population of each country. This method intended to compensate for annual changes in the age-distribution of the population, while keeping the age-standardized count close to the observed absolute numbers.

For further analysis, we analyzed CODs that had at least 5 average age-standardized annual deaths, because most of the smaller CODs had predominantly zero or only zero annual deaths.

Long-term time-trends of the age-standardized count of deaths of each COD in each country were analyzed using ordinary least squares regression models. Trends were fitted by applying linear regression models with polynomial terms of year as continuous, independent covariates

For peer review only - http://bmjopen.bmj.com/site/about/guidelines.xhtml

[18]. We used orthogonal polynomials in order to account for multicollinearity of the polynomial components **[19]**. We fitted four models: the constant, the linear, the quadratic and the cubic model (with zero, first, second and third degree polynomials, respectively). The four models were applied for all CODs in each country. We used the lowest corrected Akaike Information Criterion (AICc) to select the best model for each COD in each country **[20]**. In a next step, the best model was compared with the constant model using the F-test, at the significance level of α=0.05. If the best model performed better than the constant model with statistical significance, it was kept as the final best model. Otherwise, the constant model was selected as the best model for this COD. In the rest of the paper, the constant model is referred to as the absence of a demonstrable trend.

Next, using a multilevel logistic regression model, we determined how the categorization of a COD as having a statistically significant trend (i.e. best model being the linear, quadratic or cubic model) was related to COD size and COD type. These variables were included in the model as fixed effects. The COD size was defined as the mean annual number of deaths and the COD type was defined as the ICD-10 chapter in which it is classified. The chapter of circulatory diseases was the reference category, as it had the largest number of deaths. As the distribution of the number of deaths across CODs was highly skewed, we used its natural logarithm as a measure of COD size. The model also included the level of countries as random effect, in order to investigate the variation of European countries in the likelihood of detecting a long-term trend. We calculated the Intra-class Correlation Coefficients (ICC), which expresses the proportion of the variance in the outcome that is attributable to variations between the countries **[21]**. The ICC was calculated both with and without controlling for the fixed effects of the size and type of the COD.

Finally, we used receiver operating characteristic (ROC) curve diagnostics **[22,23,24]** to derive COD size thresholds for detecting a long-term time-trend. We calculated the Area Under the Curve (AUC) of the logistic model with COD size as the predictor and the binary categorization

For peer review only - http://bmjopen.bmj.com/site/about/guidelines.xhtml

of a COD as having a significant long-term time-trend as the outcome. We derived the COD size thresholds using three indices. Firstly, we used the maximum Youden index **[25,26,27]**, which represents the point of the ROC curve with the maximum sum of sensitivity (se) and specificity (sp). Secondly, we used the index measuring the minimum difference between sensitivity and specificity **[23]**. Thirdly, we estimated the index that represents the point closest to the top-left part of the ROC curve **[22,26]**.

All analyses were conducted using R statistical software (3.5.1 version) **[28]**.

Patient and Public Involvement

No patients were involved in this study.

Results

The number of CODs with at least 5 annual deaths on average varied between 202 (Estonia) and 791 (Germany) **(Table 1)**. Of these CODs, 32.6%, 20.2% and 11.2% had a significant trend following a linear, quadratic or cubic model respectively. The percentage of COD with no significant trend (i.e. constant model) varied from 27.5% to 43.9%, and was highest in the Nordic countries, Switzerland and Slovenia. More detailed information on the best model for each COD in each European country can be found in an additional file **(Online Resource 1)**.

For peer review only

Table 1:

Frequencies of causes of deaths according to estimates of their long-term time-trend in 21 European countries

Country (sorted by mean population)	CODs* analyzed (n)	Type of long-term time-trend (%)				Mean population (thousands)	Mean crude annual deaths from all CODs together	Mean crude annual deaths from CODs analyzed*
		No significant trend	Linear	Quadratic	Cubic			
**Estonia**	202	34.7	35.6	15.3	14.4	1,343	16,642	16,006
**Slovenia**	228	43.9	29.4	17.1	9.6	2,029	18,830	18,163
**Latvia**	259	38.2	28.6	17.0	16.2	2,157	30,840	30,091
**Lithuania**	308	37.7	32.5	19.5	10.4	3,187	42,101	41,243
**Croatia**	315	34.6	37.1	15.9	12.4	4,381	51,444	50,844
**Norway**	356	42.4	26.4	23.6	7.6	4,804	41,764	40,886
**Finland**	355	42.8	35.2	16.6	5.4	5,302	49,869	48,933
**Denmark**	420	40.0	37.4	13.6	9.0	5,497	54,446	53,319
**Switzerland**	451	43.9	31.0	16.4	8.6	7,558	62,183	61,384
**Austria**	374	35.8	25.4	23.5	15.2	8,359	77,256	75,765
**Sweden**	457	43.3	30.6	19.5	6.6	9,259	91,504	90,516
**Hungary**	482	36.1	27.6	29.3	7.1	10,024	130,830	129,695
**Czech Republic**	443	31.8	35.9	23.0	9.3	10,383	107,448	106,636
**Belgium**	517	37.3	33.7	20.9	8.1	10,678	104,344	103,257

For peer review only - http://bmjopen.bmj.com/site/about/guidelines.xhtml

For peer review only

**Netherlands**	554	33.9	28.0	25.1	13.0	16,489	138,373	137,645
**Romania**	444	31.8	36.0	19.4	12.8	21,594	258,000	257,265
**Poland**	581	27.7	33.2	22.9	16.2	38,114	375,231	374,295
**Spain**	672	33.0	33.9	18.2	14.9	44,594	385,512	383,204
**United Kingdom**	710	30.8	33.8	21.1	14.2	61,690	580,733	578,572
**France**	738	27.5	38.8	22.6	11.1	61,709	535,320	532,293
**Germany**	791	27.8	35.4	24.7	12.1	81,980	852,566	849,400

* coded at ICD-10, 3-position level; including all causes of death with at least 5 mean number of deaths in the 15-year-period

Both COD size and COD type were significantly associated with the likelihood of having a significant long-term trend (p-value <0.001) **(Table 2)**. For every 10% increase in the COD size, we observed a 18% increase (1.1^1.73=0.18) in the odds of having a significant trend (OR=1.73, 95%CI=1.67;1.79). Regarding the COD type, neoplasms and digestive system diseases had lower probability for detecting a trend in comparison to the circulatory system diseases. On the other hand, this probability was higher for infectious diseases, mental diseases and signs-and-symptoms. **Fig1** shows for each COD chapter in each country the estimated probability of having a significant long-term trend in relation to COD size. The variation between COD chapters was substantial, irrespective of COD size. Neoplasms (chapter C00.D48) as a group of CODs showed the lowest probability of having a detectable trend.

We found only small variation of countries in the likelihood of detecting a long-term trend, as the ICC for the country-level random effect was only 0.013 (without fixed effects for chapter and size) and 0.003 (with fixed effects) **(Table 2)**. **Fig2** illustrates the small differences between countries in the estimated probability of having a long-term trend.

For peer review only - http://bmjopen.bmj.com/site/about/guidelines.xhtml

For peer review only

Table 2:

Relationship between the likelihood for a cause of death (COD) to have a significant long-term trend with its size, corresponding ICD-10 chapter, and country

COD characteristic		Number of CODs	Total number of deaths	Odds Ratio*** (95% Confidence Interval)
**Size**				
log(mean deaths*)			-	**1.73** (1.67 ; 1.79)
**ICD10 Chapter**				
I00.I99	Diseases of the circulatory system	988	23,610,116	reference
A00.B99	Certain infectious and parasitic diseases	454	823,839	**1.63** (1.25 ; 2.14)
C00.D48	Neoplasms	1871	15,632,543	**0.57** (0.47 ; 0.69)
D50.D89	Diseases of blood and blood-forming organs and certain disorders involving the immune mechanisms	217	143,156	0.82 (0.59 ; 1.14)
E00.E90	Endocrine, nutritional and metabolic diseases	365	1,547,431	1.01 (0.76 ; 1.34)
F00.F99	Mental, behavioural disorders	230	1,725,881	**1.62** (1.13 ; 2.30)
G00.G99	Diseases of the nervous system	536	1,888,629	0.90 (0.70 ; 1.15)
J00.J99	Diseases of the respiratory system	553	4,740,481	1.22 (0.94 ; 1.58)
K00.K93	Diseases of the digestive system	767	2,816,168	**0.75** (0.59 ; 0.94)
M00.M99	Diseases of the musculoskeletal system and connective tissue	367	283,108	1.14 (0.86 ; 1.50)
N00.N99	Diseases of the genitourinary system	385	1,029,118	0.98 (0.74 ; 1.29)
Q00.Q99	Congenital malformations, deformations and chromosomal abnormalities	325	141,848	0.89 (0.67 ; 1.19)
R00.R99	Symptoms, signs and abnormal clinical and laboratory findings, not elsewhere classified (signs-and-symptoms)	258	2,062,587	**1.68** (1.18 ; 2.38)
V01.Y98	External causes of morbidity and mortality	1992	3,005,539	1.20 (0.99 ; 1.46)

For peer review only

Other**	349	240,748	1.29 (0.97 ; 1.71)
**Intra-class correlation for the country level**			
model with fixed effects for size and ICD10 chapter		0.003	
model with no fixed effects		0.013	

*mean deaths : mean of the annual number of deaths for a cause of death monitored in the 15-year-period, measured per country. Only including CODs with 5 or more deaths.

** “Other” consists of the causes of death classified in the ICD-10 chapters H00.H59: Diseases of the eye and adnexa, H60.H95: Diseases of the ear and mastoid process, L00.L99: Diseases of the skin and subcutaneous tissue, O00.O99: Pregnancy, childbirth and the puerperium and P00.P96: Certain conditions originating in the perinatal period.

***odds ratios in bold were statistically significant with p-value < 0.05

Fig3a describes the sensitivity (se) and specificity (sp) for detecting a significant long-term trend using different levels of thresholds in terms of any COD size. The AUC corresponding to these se and sp values was 0.706, with 95%CI:0.695;0.716. The maximized sum index (Youden Index) was 32.7 annual deaths, with sensitivity (se) 61.4% and specificity (sp) 70.3%. The minimum difference index was 27.5 annual deaths (se= 65.5%, sp= 65.5%). The closest top-left index was 29.4 annual deaths (se= 64.0%, sp= 67.5%) (Figure 3a).

The corresponding analysis for the neoplasms yielded a similar ROC curve (AUC= 0.703, 95%CI:0.680;0.727) **(Fig3b)**. The Youden Index was 70.4, with sensitivity 61.5% and specificity 69.6%, and the minimum difference index was 60.5 annual deaths (se= 64.8%, sp= 64.8%). The closest top-left index was also 60.5 annual deaths (se= 64.1%, sp= 66.9%). Infectious and parasitic diseases (AUC= 0.706, 95%CI:0.695;0.716) yielded a Youden Index of 19.7 annual deaths, with sensitivity 67.1% and specificity 69.8%. The closest top-left index was identical, while the minimum difference threshold was 19.3 annual deaths (se= 67.4%, sp= 67.4%) **(Fig3c)**.

For peer review only - http://bmjopen.bmj.com/site/about/guidelines.xhtml

Discussion

COD data are used in widely varying settings, ranging from detailed mortality profiles to macro estimates. Applications include studies in localized areas **[29]**, single countries **[30]** or worldwide **[2,31]**; for a single-disease **[32,33]** or disease group **[9]**; monitored for days or a long-term period **[7]**; for specific age groups **[33,34]** or specific situations (e.g. maternal mortality **[5]**, external causes **[35,36,37]**). These settings all impose different requirements on the collected data. Here we focused on one particular application: national estimates of mortality time trends for a reasonably long period (15 years), for a considerable number of countries (21) that have quite comparable CODs data collection and registration systems **[38]**, covering as many CODs as possible. Our aim was to investigate the effect of the size of a COD on the probability to detect a significant trend, and how this is related to country and type of COD (ICD-10 chapter).

Our results indicate that both the size and the type of a cause of death were associated with the probability of detecting a significant trend, while variations among European countries were negligible. Some types of COD, particularly neoplasms and digestive system diseases, had a lower probability for detecting a significant trend in comparison to the circulatory system diseases, whereas infectious and mental diseases had a higher probability. The results suggest a general size criterion of 30 annual deaths for selecting causes of death to include in long-term mortality trends analysis, and a more specific criterion of 65 deaths for neoplasms and 20 for infectious diseases.

We should outline the limitations of our study. Firstly, due to the exclusion of causes of death with less than 5 annual deaths on average, smaller countries were represented in our analysis with fewer causes of death. However, this is unlikely to have a strong influence on the results, as the suggested COD size threshold of about 30 deaths is much higher than the

For peer review only - http://bmjopen.bmj.com/site/about/guidelines.xhtml

lower limit of 5 mean annual deaths. Secondly, although we proposed the COD size as a criterion to select CODs for long-term trend analysis, we acknowledge that other criteria could be used, such as greater preference to CODs that involve high healthcare costs or that are potentially modifiable by preventive or curative actions. Thirdly, the likelihood to demonstrate a time trend with statistical significance depends on the statistical method that is used to describe these trends. Our results are dependent on the balance between avoiding Type I error and Type II errors. As for Type I errors, we chose a significance level of $\alpha=0.05$. A more restrictive significance level would have the consequence to increase Type II errors, i.e. to reduce the proportion of COD for which a trend would be detected based on our method.

Moreover, our results should be seen as conditional on our use of polynomial models, especially regarding the overall proportion of CODs for which a trend could be detected. An alternative to the classic polynomial regression approach would have been to use Generalized Additive Models (GAMs). These models have the advantage of being able to pick up trends that are not polynomial. In a sensitivity analysis, we applied GAMs with Gaussian process smoothing function to our data. We found that a long-term trend could be detected in 71.7 percent of the CODs, as compared to 64.0 percent in our original analysis. There were virtually no CODs for which a trend could be detected when using polynomial models but not when using GAMs. This would imply that our results are approximately robust to the method used, although somewhat conservative.

We found that mortality from neoplasms was less likely to have a significant trend, for a given size of COD. This may relate to the fact that the neoplasm mortality levels tend to change gradually over time, without short-term trend changes **[6]**. Additionally, cancers are usually coded reliably and consistently over time **[39,40,41]**, so that coding artefacts can rarely induce artificial changes. Conversely, the dynamic nature of infectious diseases may be responsible for their higher likelihood to change over time, and to have significant trends

For peer review only - http://bmjopen.bmj.com/site/about/guidelines.xhtml

even with relatively small numbers of deaths. Similarly, the chapter of signs-and-symptoms is sensitive to changes in the coding rules and practices, thus creating significant changes even with small number of deaths.

Our study showed that European countries did not vary substantially in the probability of detecting a significant long-term trend in CODs of the same size and type. This finding is surprising given the heterogeneity of the countries in terms of demographic characteristics, disease epidemiology, healthcare systems and coding practices. We found that differences between countries in the proportions of CODs with a significant trend (shown in Table 1) can be related to differences in COD size, which is strongly related to the differences in population size. Consequently, our analysis provides support for establishing one common COD size threshold, applicable for all European countries and for use in international trend analyses.

Currently there is no gold standard for the selection of CODs to analyze for long-term trends. In this study we attempted to set such a standard, based on the criterion of the COD size, which is easy to measure for each single COD. We calculated thresholds with three common methods, which came close enough (e.g. in the range of 28 to 33 deaths) to support one general recommendation for practical use. Of course, different thresholds may be preferred, depending on the user's preference to avoid either false positives (by selecting a higher threshold) or false negatives (lower threshold).

In our data, the number of CODs that surpassed our recommended threshold of 30 annual deaths on average is around 500 for the biggest countries, 200-250 for the middle-sized countries and around 100 for the smaller European countries (results not shown). In total, 52 CODs had over 30 annual deaths on average in each country included in our analysis. This implies that at least 52 CODs could be included in the international comparison of long-term trends, but up to 100 if one is to accept a greater risk of false positives in smaller countries.

For peer review only - http://bmjopen.bmj.com/site/about/guidelines.xhtml

From the public health practitioner's perspective, the findings of our study can be used in order to set realistic expectations about the number of CODs that are likely to have a significant long-term trend in populations. We recommend a size criterion of 30 annual deaths to be considered when planning for national or international monitoring and comparisons of cause-specific mortality.

References

1. Mathers CD, Fat DM, Inoue M, Rao C, Lopez AD. Counting the dead and what they died from: an assessment of the global status of cause of death data. Bull World Health Organ. 2005;83(3):171-7.

2. Naghavi M, Abajobir AA, Abbafati C, Abbas KM, Abd-Allah F, Abera SF, et al. Global, regional, and national age-sex specific mortality for 264 causes of death, 1980-2016: a systematic analysis for the Global Burden of Disease Study 2016. Lancet. 2017;390(10100):1151-210.

3. World Health Organization. International statistical classification of diseases and related health problems. Tenth revision, vol. 2. Geneva, Switzerland: WHO; 1993.

4. Aregawi M, Malm KL, Wahjib M, Kofi O, Allotey N-K, Yaw PN, et al. Effect of anti-malarial interventions on trends of malaria cases, hospital admissions and deaths, 2005–2015 ,Ghana. Malar J. 2017;16(1):177.

5. Mnyani CN, Buchmann EJ, Chersich MF, Frank KA, McIntyre JA. Trends in maternal deaths in HIV-infected women, on a background of changing HIV management guidelines in South Africa: 1997 to 2015. J Int AIDS Soc. 2017;20(3): e25022.

For peer review only - http://bmjopen.bmj.com/site/about/guidelines.xhtml

For peer review only

6. Torre LA, Siegel RL, Ward EM, Jemal A. Global cancer incidence and mortality rates and trends-an update. Cancer Epidemiol Biomarkers Prev. 2016;25(1):16-27.

7. Armstrong GL, Conn, LA, Pinner RW. Trends in infectious disease mortality in the United States during the 20th century. JAMA. 1999;281(1):61-6.

8. Griffiths C, Brock A, Rooney C. The impact of introducing ICD-10 on trends in mortality from circulatory diseases in England and Wales. Health statistics quarterly / Office for National Statistics. 2004(22):14-20.

9. Choe YJ, Choe SA, Cho SI. Trends in infectious disease mortality, South Korea, 1983-2015. Emerg Infect Dis. 2018;24(2):320-7.

10. Van Dyke M, Greer S, Odom E, Schieb L, Vaughan A, Kramer M, et al. Heart disease death rates among blacks and whites Aged ≥35 Years - United States, 1968-2015. MMWR Surveill Summ. 2018;67(5):1-11.

11. Hertel-Fernandez AW, Giusti AE, Sotelo JM. The Chilean infant mortality decline: improvement for whom? Socioeconomic and geographic inequalities in infant mortality, 1990-2005. Bull World Health Organ. 2007;85(10):798-804.

12. Mesalles-Naranjo O, Grant I, Wyper GMA, Stockton D, Dobbie R, McFadden M, et al. Trends and inequalities in the burden of mortality in Scotland 2000-2015. PloS One. 2018;13(8):e0196906.

13. Medina-Gomez OS, Medina-Reyes IS. Mortality from type 2 diabetes and implementation of the PREVENIMSS program: a time series study in Mexico, 1998-2015. Cad Saude Publica. 2018;34(5):e00103117.

14. Allanson ER, Pattinson RC. Quality-of-care audits and perinatal mortality in South Africa. Bull World Health Organ. 2015;93(6):424-8.

For peer review only

For peer review only - http://bmjopen.bmj.com/site/about/guidelines.xhtml

15.Notrica DM, Sayrs LW, Krishna N. The effect of verified pediatric trauma centers, state laws, and crash characteristics on time trends in adolescent motor vehicle fatalities, 1999-2015. J Trauma Acute Care Surg. 2018;85(5):944-52.

16. WHO Mortality Database. Geneva: World Health Organization; 2017. Available from: http://www.who.int/healthinfo/mortality_data/en/ [accessed 5 October 2017].

17.Eurostat - European Union. Chapter 7: Demographic challenges-population projections. In: People in the EU: Who are we and how do we live? Eurostat Statistical Books. Luxemburg: Publications Office of the European Union; 2015. p. 159.

18. Mitratza M, Kunst AE, Kardaun JWPF. Detecting Mortality Trends in the Netherlands Across 625 Causes of Death. Int J Environ Res Public Health. 2019;16(21):4150. doi: 10.3390/ijerph16214150.

19.Shacham M, Brauner N. Minimizing the effects of collinearity in polynomial regression. Ind Eng Chem Res. 1997;36:4405-12.

20.Wong CS, Li WK. A note on the corrected Akaike information criterion for threshold autoregressive models. J Time Ser Anal. 1998;19(1):113-24.

21.Diez Roux AV. A glossary for multilevel analysis. J Epidemiol Community Health. 2002;56:588-94.

22.Metz CE. Basic principles of ROC analysis. Semin Nucl Med. 1978;8(4):283-98.

23.Greiner M, Pfeiffer D, Smith RD. Principles and practical application of the receiver-operating characteristic analysis for diagnostic tests. Prev Vet Med. 2000;45(1-2):23–41.

24.Zou KH, O'Malley AJ, Mauri L. Receiver-operating characteristic analysis for evaluating diagnostic tests and predictive models. Circulation. 2007;115(5):654–7.

For peer review only

For peer review only - http://bmjopen.bmj.com/site/about/guidelines.xhtml

25. Youden WJ. Index for rating diagnostic tests. Cancer. 1950;3(1):32–5.

26. Perkins NJ, Schisterman EF. The inconsistency of "optimal" cutpoints obtained using two criteria based on the receiver operating characteristic curve. Am J Epidemiol. 2006;163(7):670–5.

27. Fluss R, Faraggi D, Reiser B. Estimation of the Youden Index and its associated cutoff point. Biom J. 2005;47(4):458–72.

28. R Core Team (2018). R: A language and environment for statistical computing. R Foundation for Statistical Computing, Vienna, Austria: https://www.R-project.org.

29. Bradshaw D, Groenewald P, Bourne DE, Mahomed H, Nojilana B, Daniels J, et al. Making COD statistics useful for public health at local level in the city of Cape Town: utilidad para la salud publica a nivel local en Ciudad del Cabo. Bull World Health Organ. 2006;84(3):211-7.

30. Franca EB, Passos VMA, Malta DC, Duncan BB, Ribeiro ALP, Guimaraes MDC, et al. Cause-specific mortality for 249 causes in Brazil and states during 1990-2015: a systematic analysis for the global burden of disease study 2015. Popul Health Metr. 2017;15(1):39.

31. Ebmeier S, Thayabaran D, Braithwaite I, Benamara C, Weatherall M, Beasley R. Trends in international asthma mortality: analysis of data from the WHO Mortality Database from 46 countries (1993-2012). Lancet. 2017;390(10098):935-45.

32. Burkill S, Montgomery S, Hajiebrahimi M, Hillert J, Olsson T, Bahmanyar S. Mortality trends for multiple sclerosis patients in Sweden from 1968 to 2012. Neurology. 2017;89(6):555-62.

33. Reynales-Shigematsu LM, Guerrero-Lopez CM, Hernandez Avila M, Irving H, Jha P. Divergence and convergence in cause-specific premature adult mortality in Mexico and US Mexican Hispanics from 1995 to 2015: analyses of 4.9 million individual deaths. Int J Epidemiol. 2018;47(1):97-106.

For peer review only

34. Oza S, Lawn JE, Hogan DR, Mathers C, Cousens SN. Neonatal cause-of-death estimates for the early and late neonatal periods for 194 countries: 2000-2013. Bull World Health Organ. 2015;93(1):19-28.

35. Mack K, Clapperton A, Macpherson A, Sleet D, Newton D, Murdoch J, et al. Trends in the leading causes of injury mortality, Australia, Canada, and the United States, 2000-2014. Can J Public Health. 2017;108(2):e185-e91.

36. Padron-Monedero A, Damian J, Pilar Martin M, Fernandez-Cuenca R. Mortality trends for accidental falls in older people in Spain, 2000-2015. BMC Geriatr. 2017;17(1):276.

37. Ajdacic-Gross V, Weiss MG, Ring M, Hepp U, Bopp M, Gutzwiller F, et al. Methods of suicide: international suicide patterns derived from the WHO mortality database. Bull World Health Organ. 2008;86(9):726-32.

38. Commission Regulation (EU) No 328/2011 of 5 April 2011 implementing Regulation (EC) No 1338/2008 of the European Parliament and of the Council on Community statistics on public health and health and safety at work, as regards statistics on causes of death Text with EEA relevance. Official Journal of the European Union.

39. Harteloh P, de Bruin K, Kardaun J. The reliability of cause-of-death coding in The Netherlands. Eur J Epidemiol. 2010;25(8):531–8.

40. Lu TH, Lee MC, Chou MC. Accuracy of cause-of-death coding in Taiwan: types of miscoding and effects on mortality statistics. Int J Epidemiol. 2000;29(2):336–43.

41. Mieno MN, Tanaka N, Arai T, Kawahara T, Kuchiba A, Ishikawa S, et al. Accuracy of death certificates and assessment of factors for misclassification of underlying cause of death. J Epidemiol. 2016;26(4):191-8.

For peer review only - http://bmjopen.bmj.com/site/about/guidelines.xhtml

For peer review only

Footnotes

Contributors: AK, JK and MM designed the study. MM performed the calculations. MM, AK and JK analyzed and interpreted the results and formulated the conclusions, and made major contributions to the manuscript. All authors read and approved the final manuscript.

Funding: This research received no specific grant from any funding agency in the public, commercial or not-for-profit sectors.

Conflict of Interest: The authors declare that they have no conflict of interest.

Ethics approval: No ethical approval was required for this study, as no living subjects were involved and only aggregated administrative data were used.

Data sharing statement: All data used are publically available from the WHO Mortality Database (see https://www.who.int/healthinfo/mortality_data/en/).

Provenance and peer review: Not commissioned; externally peer reviewed.

Patient consent for publication: Not required.

For peer review only

Figure Captions

Fig1

Estimated probability for an underlying cause of death to have a significant long-term trend according to its size, by ICD-10 chapter

Footnote: *See Table 2 for the definition of the chapters.

Fig2

Estimated probability for a disease of the circulatory system to have a significant long-term mortality trend according to its size, by European country

Fig3

Sensitivity and specificity of the cause of death size for the detection of significant long-term time-trends, with thresholds for the optimal cause of death size for trend analysis

For peer review only

For peer review only - http://bmjopen.bmj.com/site/about/guidelines.xhtml

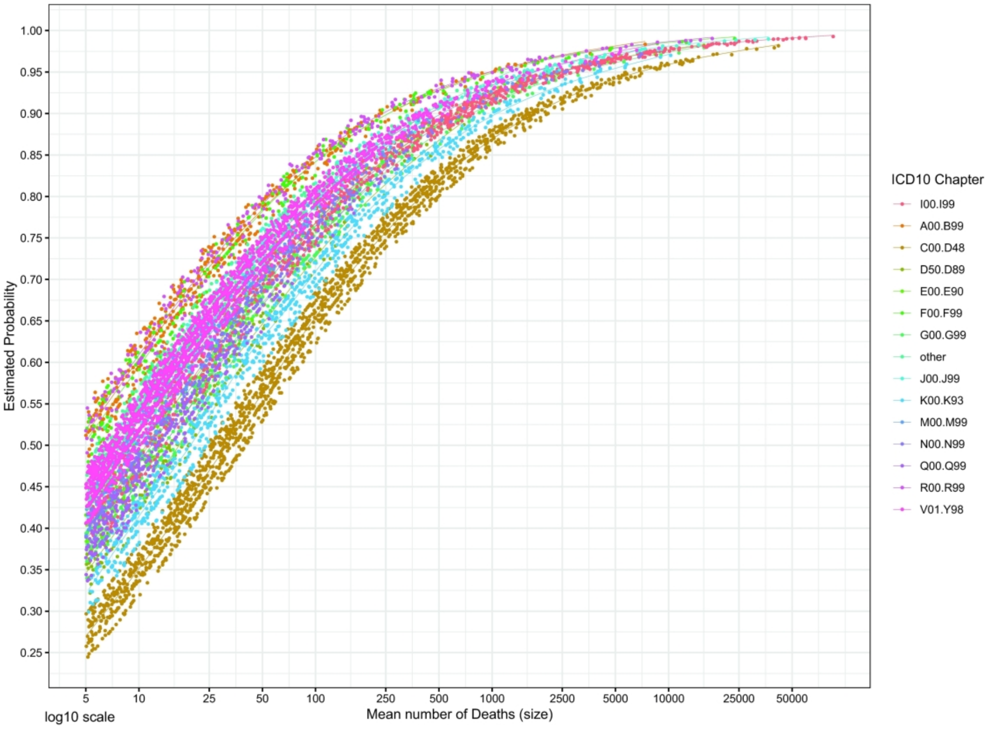

279x215mm (300 x 300 DPI)

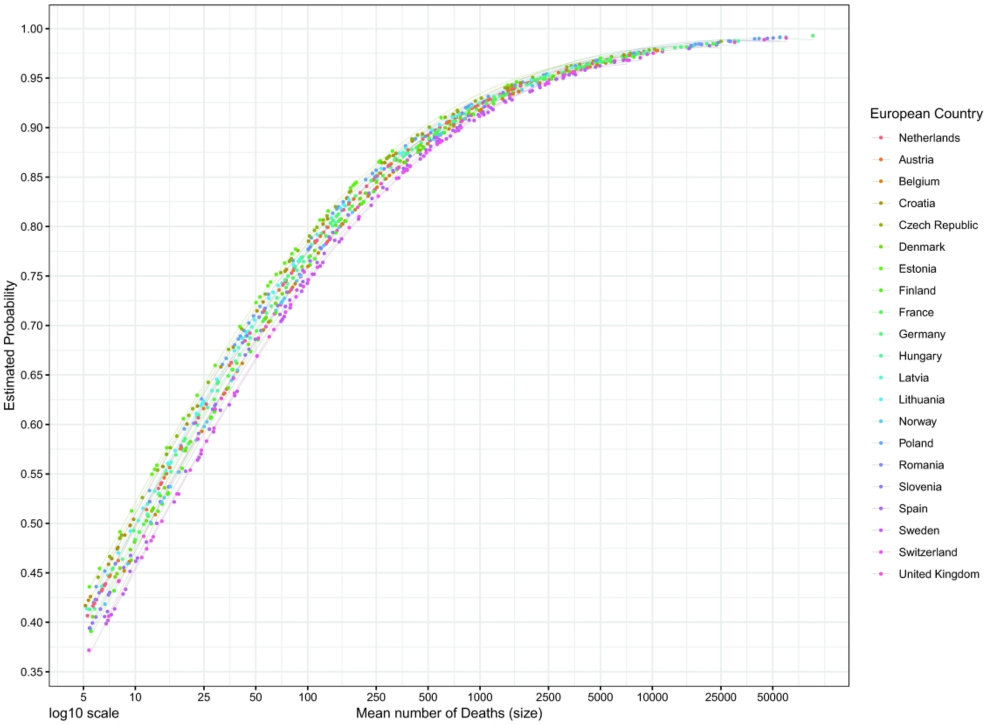

279x215mm (300 x 300 DPI)

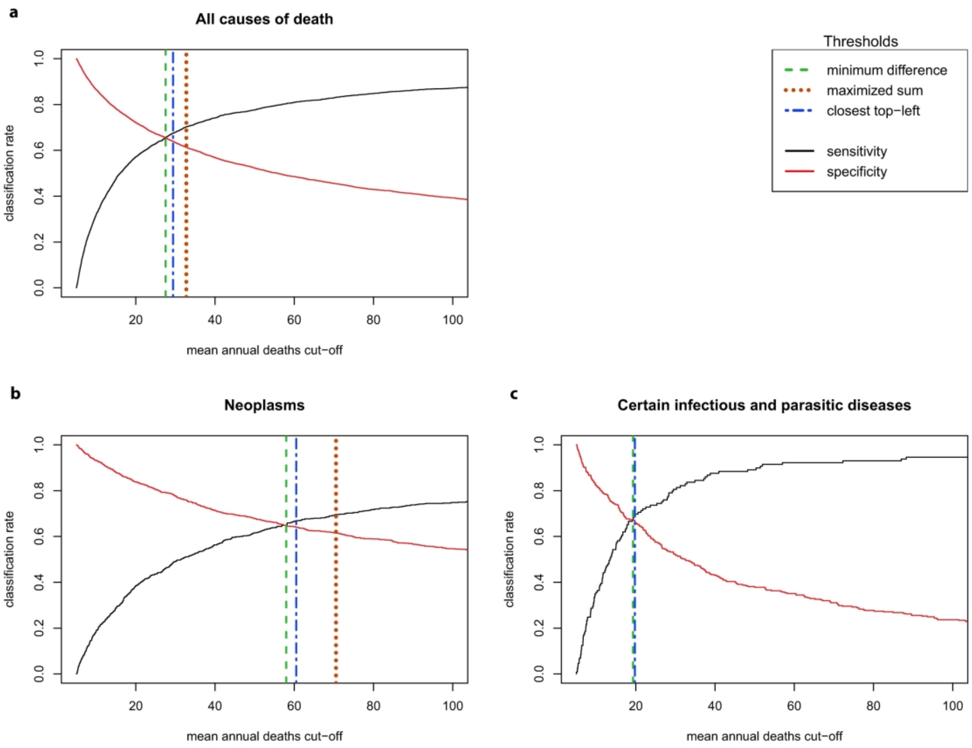

279x217mm (300 x 300 DPI)

ICD-10 code	ICD-10 code label	chapter	country	average age-standardized deaths	final best model polynomial degree	trend
A02	Other salmonella infections	A00.B99	Belgium	9.78	3	yes
A02	Other salmonella infections	A00.B99	Czech Republic	8.64	0	no
A02	Other salmonella infections	A00.B99	France	21.97	1	yes
A02	Other salmonella infections	A00.B99	Germany	54.17	1	yes
A02	Other salmonella infections	A00.B99	Hungary	6.18	0	no
A02	Other salmonella infections	A00.B99	Netherlands	7.44	1	yes
A02	Other salmonella infections	A00.B99	Poland	9.07	0	no
A02	Other salmonella infections	A00.B99	Spain	15.93	1	yes
A02	Other salmonella infections	A00.B99	United Kingdom	16.29	1	yes
A04	Other bacterial intestinal infections	A00.B99	Belgium	132.83	2	yes
A04	Other bacterial intestinal infections	A00.B99	Croatia	5.86	2	yes
A04	Other bacterial intestinal infections	A00.B99	Czech Republic	103.51	3	yes
A04	Other bacterial intestinal infections	A00.B99	Denmark	77.43	3	yes
A04	Other bacterial intestinal infections	A00.B99	Finland	16.69	0	no
A04	Other bacterial intestinal infections	A00.B99	France	290.13	3	yes
A04	Other bacterial intestinal infections	A00.B99	Germany	1221.13	3	yes
A04	Other bacterial intestinal infections	A00.B99	Hungary	108.85	2	yes
A04	Other bacterial intestinal infections	A00.B99	Netherlands	23.73	3	yes
A04	Other bacterial intestinal infections	A00.B99	Norway	47.71	2	yes
A04	Other bacterial intestinal infections	A00.B99	Poland	139.10	3	yes
A04	Other bacterial intestinal infections	A00.B99	Romania	26.54	3	yes
A04	Other bacterial intestinal infections	A00.B99	Spain	62.40	1	yes
A04	Other bacterial intestinal infections	A00.B99	Sweden	65.24	3	yes
A04	Other bacterial intestinal infections	A00.B99	Switzerland	23.09	1	yes
A04	Other bacterial intestinal infections	A00.B99	United Kingdom	1817.62	2	yes
A05	Other bacterial foodborne intoxications	A00.B99	France	11.53	1	yes
A05	Other bacterial foodborne intoxications	A00.B99	Germany	9.54	2	yes
A05	Other bacterial foodborne intoxications	A00.B99	Poland	5.75	0	no
A08	Viral and other specified intestinal infections	A00.B99	Belgium	16.42	0	no
A08	Viral and other specified intestinal infections	A00.B99	Denmark	17.33	2	yes
A08	Viral and other specified intestinal infections	A00.B99	Finland	18.39	2	yes
A08	Viral and other specified intestinal infections	A00.B99	France	33.75	1	yes
A08	Viral and other specified intestinal infections	A00.B99	Germany	171.25	2	yes
A08	Viral and other specified intestinal infections	A00.B99	Hungary	6.08	1	yes
A08	Viral and other specified intestinal infections	A00.B99	Netherlands	70.77	1	yes
A08	Viral and other specified intestinal infections	A00.B99	Norway	7.43	3	yes
A08	Viral and other specified intestinal infections	A00.B99	Poland	5.19	1	yes
A08	Viral and other specified intestinal infections	A00.B99	Spain	7.01	0	no
A08	Viral and other specified intestinal infections	A00.B99	Sweden	40.71	2	yes
A08	Viral and other specified intestinal infections	A00.B99	Switzerland	12.96	3	yes
A08	Viral and other specified intestinal infections	A00.B99	United Kingdom	76.31	3	yes
A09	Diarrhoea and gastroenteritis of presumed infectious origin	A00.B99	Austria	31.23	3	yes
A09	Diarrhoea and gastroenteritis of presumed infectious origin	A00.B99	Belgium	147.79	1	yes
A09	Diarrhoea and gastroenteritis of presumed infectious origin	A00.B99	Croatia	16.85	1	yes

For peer review only

A09	Diarrhoea and gastroenteritis of presumed infectious origin	A00.B99	Czech Republic	40.34	1	yes
A09	Diarrhoea and gastroenteritis of presumed infectious origin	A00.B99	Denmark	96.17	1	yes
A09	Diarrhoea and gastroenteritis of presumed infectious origin	A00.B99	Finland	27.69	1	yes
A09	Diarrhoea and gastroenteritis of presumed infectious origin	A00.B99	France	1031.42	3	yes
A09	Diarrhoea and gastroenteritis of presumed infectious origin	A00.B99	Germany	1349.34	1	yes
A09	Diarrhoea and gastroenteritis of presumed infectious origin	A00.B99	Hungary	62.70	3	yes
A09	Diarrhoea and gastroenteritis of presumed infectious origin	A00.B99	Netherlands	157.63	2	yes
A09	Diarrhoea and gastroenteritis of presumed infectious origin	A00.B99	Norway	121.17	2	yes
A09	Diarrhoea and gastroenteritis of presumed infectious origin	A00.B99	Poland	30.26	0	no
A09	Diarrhoea and gastroenteritis of presumed infectious origin	A00.B99	Romania	60.39	2	yes
A09	Diarrhoea and gastroenteritis of presumed infectious origin	A00.B99	Spain	538.56	1	yes
A09	Diarrhoea and gastroenteritis of presumed infectious origin	A00.B99	Sweden	96.00	3	yes
A09	Diarrhoea and gastroenteritis of presumed infectious origin	A00.B99	Switzerland	42.04	1	yes
A09	Diarrhoea and gastroenteritis of presumed infectious origin	A00.B99	United Kingdom	377.61	1	yes
A15	Respiratory tuberculosis, bacteriologically and histologically confirmed	A00.B99	Croatia	13.42	2	yes
A15	Respiratory tuberculosis, bacteriologically and histologically confirmed	A00.B99	Czech Republic	41.62	2	yes
A15	Respiratory tuberculosis, bacteriologically and histologically confirmed	A00.B99	Estonia	26.69	1	yes
A15	Respiratory tuberculosis, bacteriologically and histologically confirmed	A00.B99	Finland	20.85	2	yes
A15	Respiratory tuberculosis, bacteriologically and histologically confirmed	A00.B99	Germany	52.81	1	yes
A15	Respiratory tuberculosis, bacteriologically and histologically confirmed	A00.B99	Hungary	45.07	2	yes
A15	Respiratory tuberculosis, bacteriologically and histologically confirmed	A00.B99	Poland	345.67	0	no
A15	Respiratory tuberculosis, bacteriologically and histologically confirmed	A00.B99	Romania	1051.27	1	yes
A15	Respiratory tuberculosis, bacteriologically and histologically confirmed	A00.B99	Slovenia	9.30	0	no
A16	Respiratory tuberculosis, not confirmed bacteriologically or histologically	A00.B99	Austria	34.91	0	no
A16	Respiratory tuberculosis, not confirmed bacteriologically or histologically	A00.B99	Belgium	38.61	1	yes
A16	Respiratory tuberculosis, not confirmed bacteriologically or histologically	A00.B99	Croatia	101.52	1	yes
A16	Respiratory tuberculosis, not confirmed bacteriologically or histologically	A00.B99	Czech Republic	23.82	1	yes
A16	Respiratory tuberculosis, not confirmed bacteriologically or histologically	A00.B99	Denmark	20.54	0	no
A16	Respiratory tuberculosis, not confirmed bacteriologically or histologically	A00.B99	Estonia	23.02	2	yes
A16	Respiratory tuberculosis, not confirmed bacteriologically or histologically	A00.B99	France	357.23	3	yes

For peer review only

A16	Respiratory tuberculosis, not confirmed bacteriologically or histologically	A00.B99	Germany	229.49	2	yes
A16	Respiratory tuberculosis, not confirmed bacteriologically or histologically	A00.B99	Hungary	115.32	1	yes
A16	Respiratory tuberculosis, not confirmed bacteriologically or histologically	A00.B99	Latvia	52.58	2	yes
A16	Respiratory tuberculosis, not confirmed bacteriologically or histologically	A00.B99	Lithuania	155.24	1	yes
A16	Respiratory tuberculosis, not confirmed bacteriologically or histologically	A00.B99	Netherlands	24.40	1	yes
A16	Respiratory tuberculosis, not confirmed bacteriologically or histologically	A00.B99	Poland	514.49	2	yes
A16	Respiratory tuberculosis, not confirmed bacteriologically or histologically	A00.B99	Romania	679.40	2	yes
A16	Respiratory tuberculosis, not confirmed bacteriologically or histologically	A00.B99	Spain	247.81	2	yes
A16	Respiratory tuberculosis, not confirmed bacteriologically or histologically	A00.B99	Sweden	13.93	0	no
A16	Respiratory tuberculosis, not confirmed bacteriologically or histologically	A00.B99	Switzerland	10.23	3	yes
A16	Respiratory tuberculosis, not confirmed bacteriologically or histologically	A00.B99	United Kingdom	302.85	1	yes
A17	Tuberculosis of nervous system	A00.B99	France	14.06	1	yes
A17	Tuberculosis of nervous system	A00.B99	Germany	9.64	0	no
A17	Tuberculosis of nervous system	A00.B99	Poland	9.37	1	yes
A17	Tuberculosis of nervous system	A00.B99	Romania	17.09	1	yes
A17	Tuberculosis of nervous system	A00.B99	Spain	16.08	1	yes
A17	Tuberculosis of nervous system	A00.B99	United Kingdom	14.54	1	yes
A18	Tuberculosis of other organs	A00.B99	France	39.71	2	yes
A18	Tuberculosis of other organs	A00.B99	Germany	17.54	1	yes
A18	Tuberculosis of other organs	A00.B99	Poland	12.29	0	no
A18	Tuberculosis of other organs	A00.B99	Romania	14.98	1	yes
A18	Tuberculosis of other organs	A00.B99	Spain	30.10	1	yes
A18	Tuberculosis of other organs	A00.B99	United Kingdom	41.12	1	yes
A19	Miliary tuberculosis	A00.B99	Finland	7.44	3	yes
A19	Miliary tuberculosis	A00.B99	France	26.15	1	yes
A19	Miliary tuberculosis	A00.B99	Germany	21.82	1	yes
A19	Miliary tuberculosis	A00.B99	Hungary	8.07	1	yes
A19	Miliary tuberculosis	A00.B99	Latvia	5.32	0	no
A19	Miliary tuberculosis	A00.B99	Lithuania	74.55	3	yes
A19	Miliary tuberculosis	A00.B99	Poland	9.24	1	yes
A19	Miliary tuberculosis	A00.B99	Romania	13.24	0	no
A19	Miliary tuberculosis	A00.B99	Spain	30.55	1	yes
A19	Miliary tuberculosis	A00.B99	United Kingdom	36.87	3	yes
A26	Erysipeloid	A00.B99	Germany	5.18	2	yes
A27	Leptospirosis	A00.B99	Romania	8.07	1	yes
A31	Infection due to other mycobacteria	A00.B99	Finland	7.60	0	no
A31	Infection due to other mycobacteria	A00.B99	France	19.16	0	no
A31	Infection due to other mycobacteria	A00.B99	Germany	8.02	1	yes
A31	Infection due to other mycobacteria	A00.B99	Poland	5.68	0	no
A31	Infection due to other mycobacteria	A00.B99	Sweden	6.62	1	yes
A31	Infection due to other mycobacteria	A00.B99	United Kingdom	41.26	3	yes
A32	Listeriosis	A00.B99	France	21.74	0	no
A32	Listeriosis	A00.B99	Germany	16.87	3	yes

For peer review only

A32	Listeriosis	A00.B99	Spain	18.11	0	no
A32	Listeriosis	A00.B99	United Kingdom	16.21	0	no
A35	Other tetanus	A00.B99	France	6.02	1	yes
A35	Other tetanus	A00.B99	Poland	8.94	1	yes
A35	Other tetanus	A00.B99	Romania	5.33	1	yes
A37	Whooping cough	A00.B99	France	5.01	0	no
A39	Meningococcal infection	A00.B99	Austria	6.07	0	no
A39	Meningococcal infection	A00.B99	Belgium	13.33	1	yes
A39	Meningococcal infection	A00.B99	Czech Republic	6.68	1	yes
A39	Meningococcal infection	A00.B99	Denmark	5.44	1	yes
A39	Meningococcal infection	A00.B99	France	30.02	1	yes
A39	Meningococcal infection	A00.B99	Germany	50.01	3	yes
A39	Meningococcal infection	A00.B99	Hungary	7.47	0	no
A39	Meningococcal infection	A00.B99	Lithuania	7.37	0	no
A39	Meningococcal infection	A00.B99	Netherlands	12.96	3	yes
A39	Meningococcal infection	A00.B99	Poland	15.10	0	no
A39	Meningococcal infection	A00.B99	Romania	16.17	2	yes
A39	Meningococcal infection	A00.B99	Spain	42.73	1	yes
A39	Meningococcal infection	A00.B99	Switzerland	6.75	2	yes
A39	Meningococcal infection	A00.B99	United Kingdom	82.51	3	yes
A40	Streptococcal septicaemia	A00.B99	Austria	6.16	0	no
A40	Streptococcal septicaemia	A00.B99	Belgium	34.18	2	yes
A40	Streptococcal septicaemia	A00.B99	Czech Republic	10.74	1	yes
A40	Streptococcal septicaemia	A00.B99	Denmark	23.52	2	yes
A40	Streptococcal septicaemia	A00.B99	Finland	28.34	0	no
A40	Streptococcal septicaemia	A00.B99	France	121.57	0	no
A40	Streptococcal septicaemia	A00.B99	Germany	152.87	2	yes
A40	Streptococcal septicaemia	A00.B99	Lithuania	6.30	1	yes
A40	Streptococcal septicaemia	A00.B99	Netherlands	56.51	1	yes
A40	Streptococcal septicaemia	A00.B99	Norway	26.55	1	yes
A40	Streptococcal septicaemia	A00.B99	Poland	22.38	3	yes
A40	Streptococcal septicaemia	A00.B99	Spain	32.47	0	no
A40	Streptococcal septicaemia	A00.B99	Sweden	45.71	1	yes
A40	Streptococcal septicaemia	A00.B99	Switzerland	22.52	0	no
A40	Streptococcal septicaemia	A00.B99	United Kingdom	118.54	3	yes
A41	Other septicaemia	A00.B99	Austria	87.99	2	yes
A41	Other septicaemia	A00.B99	Belgium	1466.24	3	yes
A41	Other septicaemia	A00.B99	Croatia	232.07	2	yes
A41	Other septicaemia	A00.B99	Czech Republic	759.13	1	yes
A41	Other septicaemia	A00.B99	Denmark	402.79	0	no
A41	Other septicaemia	A00.B99	Estonia	13.02	2	yes
A41	Other septicaemia	A00.B99	Finland	129.82	0	no
A41	Other septicaemia	A00.B99	France	4658.94	0	no
A41	Other septicaemia	A00.B99	Germany	7361.90	1	yes
A41	Other septicaemia	A00.B99	Hungary	87.44	1	yes
A41	Other septicaemia	A00.B99	Latvia	29.51	0	no
A41	Other septicaemia	A00.B99	Lithuania	181.40	2	yes
A41	Other septicaemia	A00.B99	Netherlands	1360.05	3	yes
A41	Other septicaemia	A00.B99	Norway	363.57	3	yes
A41	Other septicaemia	A00.B99	Poland	1236.95	3	yes

For peer review only

A41	Other septicaemia	A00.B99	Romania	625.64	2	yes
A41	Other septicaemia	A00.B99	Slovenia	86.99	0	no
A41	Other septicaemia	A00.B99	Spain	3600.30	3	yes
A41	Other septicaemia	A00.B99	Sweden	705.38	1	yes
A41	Other septicaemia	A00.B99	Switzerland	192.17	0	no
A41	Other septicaemia	A00.B99	United Kingdom	2963.53	3	yes
A43	Nocardiosis	A00.B99	France	5.41	0	no
A43	Nocardiosis	A00.B99	Spain	5.24	0	no
A46	Erysipelas	A00.B99	Austria	15.53	2	yes
A46	Erysipelas	A00.B99	Belgium	34.20	1	yes
A46	Erysipelas	A00.B99	Czech Republic	15.71	2	yes
A46	Erysipelas	A00.B99	Denmark	36.34	1	yes
A46	Erysipelas	A00.B99	Estonia	6.24	0	no
A46	Erysipelas	A00.B99	Finland	27.89	0	no
A46	Erysipelas	A00.B99	France	209.88	1	yes
A46	Erysipelas	A00.B99	Germany	236.24	1	yes
A46	Erysipelas	A00.B99	Hungary	26.58	1	yes
A46	Erysipelas	A00.B99	Latvia	15.88	1	yes
A46	Erysipelas	A00.B99	Lithuania	9.46	0	no
A46	Erysipelas	A00.B99	Netherlands	121.16	1	yes
A46	Erysipelas	A00.B99	Norway	26.92	0	no
A46	Erysipelas	A00.B99	Poland	17.45	0	no
A46	Erysipelas	A00.B99	Sweden	46.96	2	yes
A46	Erysipelas	A00.B99	Switzerland	21.43	1	yes
A48	Other bacterial diseases, not elsewhere classified	A00.B99	Belgium	25.29	0	no
A48	Other bacterial diseases, not elsewhere classified	A00.B99	Czech Republic	10.61	2	yes
A48	Other bacterial diseases, not elsewhere classified	A00.B99	Denmark	13.04	1	yes
A48	Other bacterial diseases, not elsewhere classified	A00.B99	France	125.58	2	yes
A48	Other bacterial diseases, not elsewhere classified	A00.B99	Germany	58.22	1	yes
A48	Other bacterial diseases, not elsewhere classified	A00.B99	Hungary	8.70	1	yes
A48	Other bacterial diseases, not elsewhere classified	A00.B99	Netherlands	34.83	1	yes
A48	Other bacterial diseases, not elsewhere classified	A00.B99	Poland	26.50	2	yes
A48	Other bacterial diseases, not elsewhere classified	A00.B99	Romania	31.21	0	no
A48	Other bacterial diseases, not elsewhere classified	A00.B99	Spain	42.04	1	yes
A48	Other bacterial diseases, not elsewhere classified	A00.B99	Sweden	7.39	0	no
A48	Other bacterial diseases, not elsewhere classified	A00.B99	Switzerland	12.08	1	yes
A48	Other bacterial diseases, not elsewhere classified	A00.B99	United Kingdom	36.30	2	yes
A49	Bacterial infection of unspecified site	A00.B99	Belgium	52.93	3	yes
A49	Bacterial infection of unspecified site	A00.B99	Czech Republic	24.85	1	yes
A49	Bacterial infection of unspecified site	A00.B99	Denmark	62.50	2	yes
A49	Bacterial infection of unspecified site	A00.B99	Finland	13.65	0	no
A49	Bacterial infection of unspecified site	A00.B99	France	138.52	1	yes
A49	Bacterial infection of unspecified site	A00.B99	Germany	166.82	3	yes
A49	Bacterial infection of unspecified site	A00.B99	Netherlands	19.82	2	yes
A49	Bacterial infection of unspecified site	A00.B99	Norway	15.89	1	yes
A49	Bacterial infection of unspecified site	A00.B99	Poland	22.69	1	yes
A49	Bacterial infection of unspecified site	A00.B99	Romania	22.55	1	yes
A49	Bacterial infection of unspecified site	A00.B99	Spain	116.89	3	yes
A49	Bacterial infection of unspecified site	A00.B99	Sweden	45.38	2	yes
A49	Bacterial infection of unspecified site	A00.B99	Switzerland	7.34	2	yes

For peer review only

A49	Bacterial infection of unspecified site	A00.B99	United Kingdom	198.85	2	yes
A52	Late syphilis	A00.B99	Germany	6.36	2	yes
A52	Late syphilis	A00.B99	Spain	7.60	0	no
A69	Other spirochaetal infections	A00.B99	Germany	9.70	0	no
A81	Slow virus infections of central nervous system	A00.B99	Austria	23.65	0	no
A81	Slow virus infections of central nervous system	A00.B99	Belgium	19.31	0	no
A81	Slow virus infections of central nervous system	A00.B99	Denmark	8.60	0	no
A81	Slow virus infections of central nervous system	A00.B99	Finland	12.41	0	no
A81	Slow virus infections of central nervous system	A00.B99	France	154.72	0	no
A81	Slow virus infections of central nervous system	A00.B99	Germany	160.70	1	yes
A81	Slow virus infections of central nervous system	A00.B99	Hungary	17.77	0	no
A81	Slow virus infections of central nervous system	A00.B99	Netherlands	31.41	1	yes
A81	Slow virus infections of central nervous system	A00.B99	Norway	8.55	0	no
A81	Slow virus infections of central nervous system	A00.B99	Poland	19.75	2	yes
A81	Slow virus infections of central nervous system	A00.B99	Romania	6.70	1	yes
A81	Slow virus infections of central nervous system	A00.B99	Spain	88.26	0	no
A81	Slow virus infections of central nervous system	A00.B99	Sweden	19.47	0	no
A81	Slow virus infections of central nervous system	A00.B99	Switzerland	20.16	1	yes
A81	Slow virus infections of central nervous system	A00.B99	United Kingdom	123.97	1	yes
A86	Unspecified viral encephalitis	A00.B99	France	15.02	0	no
A86	Unspecified viral encephalitis	A00.B99	Germany	12.12	0	no
A86	Unspecified viral encephalitis	A00.B99	Poland	11.05	1	yes
A86	Unspecified viral encephalitis	A00.B99	Romania	12.95	0	no
A86	Unspecified viral encephalitis	A00.B99	Spain	28.04	0	no
A86	Unspecified viral encephalitis	A00.B99	United Kingdom	42.92	1	yes
A87	Viral meningitis	A00.B99	France	10.96	1	yes
A87	Viral meningitis	A00.B99	Romania	6.33	0	no
A87	Viral meningitis	A00.B99	Spain	9.28	1	yes
B00	Herpesviral [herpes simplex] infections	A00.B99	Austria	5.57	1	yes
B00	Herpesviral [herpes simplex] infections	A00.B99	Belgium	14.94	0	no
B00	Herpesviral [herpes simplex] infections	A00.B99	France	50.36	0	no
B00	Herpesviral [herpes simplex] infections	A00.B99	Germany	45.32	1	yes
B00	Herpesviral [herpes simplex] infections	A00.B99	Netherlands	15.59	3	yes
B00	Herpesviral [herpes simplex] infections	A00.B99	Spain	37.37	2	yes
B00	Herpesviral [herpes simplex] infections	A00.B99	Sweden	6.65	0	no
B00	Herpesviral [herpes simplex] infections	A00.B99	Switzerland	5.24	0	no
B00	Herpesviral [herpes simplex] infections	A00.B99	United Kingdom	32.88	0	no
B01	Varicella [chickenpox]	A00.B99	France	11.50	0	no
B01	Varicella [chickenpox]	A00.B99	Germany	6.71	0	no
B01	Varicella [chickenpox]	A00.B99	Spain	7.71	1	yes
B01	Varicella [chickenpox]	A00.B99	United Kingdom	21.80	3	yes
B02	Zoster [herpes zoster]	A00.B99	Belgium	13.86	0	no
B02	Zoster [herpes zoster]	A00.B99	Denmark	5.27	1	yes
B02	Zoster [herpes zoster]	A00.B99	France	71.15	1	yes
B02	Zoster [herpes zoster]	A00.B99	Germany	75.74	1	yes
B02	Zoster [herpes zoster]	A00.B99	Netherlands	28.33	0	no
B02	Zoster [herpes zoster]	A00.B99	Norway	9.75	2	yes
B02	Zoster [herpes zoster]	A00.B99	Spain	21.28	0	no
B02	Zoster [herpes zoster]	A00.B99	Sweden	12.61	0	no
B02	Zoster [herpes zoster]	A00.B99	Switzerland	9.43	0	no

For peer review only

B02	Zoster [herpes zoster]	A00.B99	United Kingdom	69.17	3	yes
B15	Acute hepatitis A	A00.B99	Germany	8.72	3	yes
B16	Acute hepatitis B	A00.B99	Belgium	6.18	2	yes
B16	Acute hepatitis B	A00.B99	France	13.26	1	yes
B16	Acute hepatitis B	A00.B99	Germany	69.13	2	yes
B16	Acute hepatitis B	A00.B99	Poland	25.77	3	yes
B16	Acute hepatitis B	A00.B99	Romania	11.03	3	yes
B16	Acute hepatitis B	A00.B99	Spain	38.79	0	no
B16	Acute hepatitis B	A00.B99	United Kingdom	37.60	1	yes
B17	Other acute viral hepatitis	A00.B99	Belgium	22.54	3	yes
B17	Other acute viral hepatitis	A00.B99	Czech Republic	17.33	0	no
B17	Other acute viral hepatitis	A00.B99	France	70.84	1	yes
B17	Other acute viral hepatitis	A00.B99	Germany	72.19	2	yes
B17	Other acute viral hepatitis	A00.B99	Netherlands	6.25	2	yes
B17	Other acute viral hepatitis	A00.B99	Poland	9.47	0	no
B17	Other acute viral hepatitis	A00.B99	Romania	7.36	1	yes
B17	Other acute viral hepatitis	A00.B99	Spain	139.58	3	yes
B17	Other acute viral hepatitis	A00.B99	United Kingdom	80.13	1	yes
B18	Chronic viral hepatitis	A00.B99	Austria	277.69	2	yes
B18	Chronic viral hepatitis	A00.B99	Belgium	89.56	2	yes
B18	Chronic viral hepatitis	A00.B99	Croatia	36.46	3	yes
B18	Chronic viral hepatitis	A00.B99	Czech Republic	23.05	1	yes
B18	Chronic viral hepatitis	A00.B99	Denmark	19.70	0	no
B18	Chronic viral hepatitis	A00.B99	Estonia	5.65	1	yes
B18	Chronic viral hepatitis	A00.B99	Finland	8.66	0	no
B18	Chronic viral hepatitis	A00.B99	France	702.43	1	yes
B18	Chronic viral hepatitis	A00.B99	Germany	876.32	1	yes
B18	Chronic viral hepatitis	A00.B99	Latvia	26.31	1	yes
B18	Chronic viral hepatitis	A00.B99	Lithuania	17.12	2	yes
B18	Chronic viral hepatitis	A00.B99	Netherlands	48.28	1	yes
B18	Chronic viral hepatitis	A00.B99	Norway	9.94	0	no
B18	Chronic viral hepatitis	A00.B99	Poland	254.57	1	yes
B18	Chronic viral hepatitis	A00.B99	Romania	34.80	3	yes
B18	Chronic viral hepatitis	A00.B99	Slovenia	6.70	0	no
B18	Chronic viral hepatitis	A00.B99	Spain	860.57	1	yes
B18	Chronic viral hepatitis	A00.B99	Sweden	57.65	1	yes
B18	Chronic viral hepatitis	A00.B99	Switzerland	14.33	1	yes
B18	Chronic viral hepatitis	A00.B99	United Kingdom	140.70	1	yes
B19	Unspecified viral hepatitis	A00.B99	France	19.38	1	yes
B19	Unspecified viral hepatitis	A00.B99	Germany	10.24	1	yes
B19	Unspecified viral hepatitis	A00.B99	Poland	8.67	1	yes
B19	Unspecified viral hepatitis	A00.B99	Spain	19.80	1	yes
B19	Unspecified viral hepatitis	A00.B99	United Kingdom	5.84	1	yes
B20	Human immunodeficiency virus [HIV] disease resulting in infectious and parasitic diseases	A00.B99	Belgium	20.40	1	yes
B20	Human immunodeficiency virus [HIV] disease resulting in infectious and parasitic diseases	A00.B99	Denmark	11.14	0	no
B20	Human immunodeficiency virus [HIV] disease resulting in infectious and parasitic diseases	A00.B99	Estonia	15.62	2	yes
B20	Human immunodeficiency virus [HIV] disease resulting in infectious and parasitic diseases	A00.B99	France	279.73	3	yes

For peer review only

B20	Human immunodeficiency virus [HIV] disease resulting in infectious and parasitic diseases	A00.B99	Germany	93.60	2	yes
B20	Human immunodeficiency virus [HIV] disease resulting in infectious and parasitic diseases	A00.B99	Latvia	31.93	2	yes
B20	Human immunodeficiency virus [HIV] disease resulting in infectious and parasitic diseases	A00.B99	Lithuania	7.86	1	yes
B20	Human immunodeficiency virus [HIV] disease resulting in infectious and parasitic diseases	A00.B99	Netherlands	27.18	3	yes
B20	Human immunodeficiency virus [HIV] disease resulting in infectious and parasitic diseases	A00.B99	Norway	7.12	0	no
B20	Human immunodeficiency virus [HIV] disease resulting in infectious and parasitic diseases	A00.B99	Poland	57.00	0	no
B20	Human immunodeficiency virus [HIV] disease resulting in infectious and parasitic diseases	A00.B99	Romania	77.78	2	yes
B20	Human immunodeficiency virus [HIV] disease resulting in infectious and parasitic diseases	A00.B99	Spain	530.89	2	yes
B20	Human immunodeficiency virus [HIV] disease resulting in infectious and parasitic diseases	A00.B99	Sweden	8.82	1	yes
B20	Human immunodeficiency virus [HIV] disease resulting in infectious and parasitic diseases	A00.B99	Switzerland	20.84	2	yes
B20	Human immunodeficiency virus [HIV] disease resulting in infectious and parasitic diseases	A00.B99	United Kingdom	131.09	2	yes
B21	Human immunodeficiency virus [HIV] disease resulting in malignant neoplasms	A00.B99	Belgium	7.34	0	no
B21	Human immunodeficiency virus [HIV] disease resulting in malignant neoplasms	A00.B99	France	92.20	1	yes
B21	Human immunodeficiency virus [HIV] disease resulting in malignant neoplasms	A00.B99	Germany	52.68	1	yes
B21	Human immunodeficiency virus [HIV] disease resulting in malignant neoplasms	A00.B99	Netherlands	13.24	1	yes
B21	Human immunodeficiency virus [HIV] disease resulting in malignant neoplasms	A00.B99	Poland	11.82	1	yes
B21	Human immunodeficiency virus [HIV] disease resulting in malignant neoplasms	A00.B99	Romania	6.51	2	yes
B21	Human immunodeficiency virus [HIV] disease resulting in malignant neoplasms	A00.B99	Spain	118.70	1	yes
B21	Human immunodeficiency virus [HIV] disease resulting in malignant neoplasms	A00.B99	Switzerland	8.88	2	yes
B21	Human immunodeficiency virus [HIV] disease resulting in malignant neoplasms	A00.B99	United Kingdom	29.58	1	yes
B22	Human immunodeficiency virus [HIV] disease resulting in other specified diseases	A00.B99	Belgium	6.69	0	no
B22	Human immunodeficiency virus [HIV] disease resulting in other specified diseases	A00.B99	Estonia	9.72	1	yes
B22	Human immunodeficiency virus [HIV] disease resulting in other specified diseases	A00.B99	France	145.50	1	yes
B22	Human immunodeficiency virus [HIV] disease resulting in other specified diseases	A00.B99	Germany	29.52	2	yes
B22	Human immunodeficiency virus [HIV] disease resulting in other specified diseases	A00.B99	Netherlands	6.48	2	yes
B22	Human immunodeficiency virus [HIV] disease resulting in other specified diseases	A00.B99	Poland	25.47	3	yes
B22	Human immunodeficiency virus [HIV] disease resulting in other specified diseases	A00.B99	Romania	24.72	3	yes
B22	Human immunodeficiency virus [HIV] disease resulting in other specified diseases	A00.B99	Spain	112.55	1	yes
B22	Human immunodeficiency virus [HIV] disease resulting in other specified diseases	A00.B99	Switzerland	21.14	1	yes
B22	Human immunodeficiency virus [HIV] disease resulting in other specified diseases	A00.B99	United Kingdom	24.31	3	yes

For peer review only

B23	Human immunodeficiency virus [HIV] disease resulting in other conditions	A00.B99	Belgium	6.08	0	no
B23	Human immunodeficiency virus [HIV] disease resulting in other conditions	A00.B99	France	142.60	3	yes
B23	Human immunodeficiency virus [HIV] disease resulting in other conditions	A00.B99	Germany	37.80	3	yes
B23	Human immunodeficiency virus [HIV] disease resulting in other conditions	A00.B99	Poland	15.95	0	no
B23	Human immunodeficiency virus [HIV] disease resulting in other conditions	A00.B99	Romania	5.74	2	yes
B23	Human immunodeficiency virus [HIV] disease resulting in other conditions	A00.B99	Spain	113.78	2	yes
B23	Human immunodeficiency virus [HIV] disease resulting in other conditions	A00.B99	United Kingdom	27.82	2	yes
B24	Unspecified human immunodeficiency virus [HIV] disease	A00.B99	Austria	32.58	1	yes
B24	Unspecified human immunodeficiency virus [HIV] disease	A00.B99	Belgium	11.76	0	no
B24	Unspecified human immunodeficiency virus [HIV] disease	A00.B99	Denmark	9.26	0	no
B24	Unspecified human immunodeficiency virus [HIV] disease	A00.B99	France	63.94	2	yes
B24	Unspecified human immunodeficiency virus [HIV] disease	A00.B99	Germany	219.85	2	yes
B24	Unspecified human immunodeficiency virus [HIV] disease	A00.B99	Netherlands	13.61	1	yes
B24	Unspecified human immunodeficiency virus [HIV] disease	A00.B99	Romania	71.17	3	yes
B24	Unspecified human immunodeficiency virus [HIV] disease	A00.B99	Spain	258.75	3	yes
B24	Unspecified human immunodeficiency virus [HIV] disease	A00.B99	Switzerland	13.21	0	no
B24	Unspecified human immunodeficiency virus [HIV] disease	A00.B99	United Kingdom	17.46	3	yes
B25	Cytomegaloviral disease	A00.B99	France	15.61	3	yes
B25	Cytomegaloviral disease	A00.B99	Germany	11.67	0	no
B25	Cytomegaloviral disease	A00.B99	Spain	7.81	0	no
B25	Cytomegaloviral disease	A00.B99	United Kingdom	17.72	0	no
B33	Other viral diseases, not elsewhere classified	A00.B99	United Kingdom	10.26	0	no
B34	Viral infection of unspecified site	A00.B99	Belgium	24.82	1	yes
B34	Viral infection of unspecified site	A00.B99	Denmark	8.54	0	no
B34	Viral infection of unspecified site	A00.B99	France	63.32	1	yes
B34	Viral infection of unspecified site	A00.B99	Germany	151.72	1	yes
B34	Viral infection of unspecified site	A00.B99	Hungary	21.79	1	yes
B34	Viral infection of unspecified site	A00.B99	Latvia	14.16	0	no
B34	Viral infection of unspecified site	A00.B99	Netherlands	89.62	3	yes
B34	Viral infection of unspecified site	A00.B99	Norway	5.59	1	yes
B34	Viral infection of unspecified site	A00.B99	Spain	13.75	1	yes
B34	Viral infection of unspecified site	A00.B99	Sweden	34.72	1	yes
B34	Viral infection of unspecified site	A00.B99	Switzerland	11.42	1	yes
B34	Viral infection of unspecified site	A00.B99	United Kingdom	38.05	0	no
B37	Candidiasis	A00.B99	Belgium	35.82	1	yes
B37	Candidiasis	A00.B99	Denmark	5.29	1	yes
B37	Candidiasis	A00.B99	France	95.35	3	yes
B37	Candidiasis	A00.B99	Germany	95.35	2	yes
B37	Candidiasis	A00.B99	Netherlands	11.49	0	no

For peer review only

B37	Candidiasis	A00.B99	Poland	5.68	0	no
B37	Candidiasis	A00.B99	Spain	26.54	0	no
B37	Candidiasis	A00.B99	Sweden	8.58	1	yes
B37	Candidiasis	A00.B99	Switzerland	5.20	2	yes
B37	Candidiasis	A00.B99	United Kingdom	29.19	0	no
B44	Aspergillosis	A00.B99	Belgium	40.93	0	no
B44	Aspergillosis	A00.B99	France	67.09	3	yes
B44	Aspergillosis	A00.B99	Germany	51.42	0	no
B44	Aspergillosis	A00.B99	Netherlands	18.15	0	no
B44	Aspergillosis	A00.B99	Poland	13.47	0	no
B44	Aspergillosis	A00.B99	Spain	48.22	0	no
B44	Aspergillosis	A00.B99	United Kingdom	68.90	1	yes
B49	Unspecified mycosis	A00.B99	Belgium	6.29	3	yes
B49	Unspecified mycosis	A00.B99	France	14.11	0	no
B49	Unspecified mycosis	A00.B99	Germany	10.88	0	no
B49	Unspecified mycosis	A00.B99	United Kingdom	16.74	0	no
B50	Plasmodium falciparum malaria	A00.B99	France	12.59	1	yes
B54	Unspecified malaria	A00.B99	France	5.15	1	yes
B55	Leishmaniasis	A00.B99	Spain	6.24	2	yes
B59	Pneumocystosis	A00.B99	Belgium	6.91	1	yes
B59	Pneumocystosis	A00.B99	France	33.29	3	yes
B59	Pneumocystosis	A00.B99	Germany	16.70	1	yes
B59	Pneumocystosis	A00.B99	Netherlands	5.67	1	yes
B59	Pneumocystosis	A00.B99	Spain	5.43	0	no
B59	Pneumocystosis	A00.B99	United Kingdom	37.53	0	no
B67	Echinococcosis	A00.B99	Germany	6.30	0	no
B67	Echinococcosis	A00.B99	Romania	16.87	1	yes
B67	Echinococcosis	A00.B99	Spain	19.74	1	yes
B90	Sequelae of tuberculosis	A00.B99	Belgium	29.48	1	yes
B90	Sequelae of tuberculosis	A00.B99	Finland	38.58	1	yes
B90	Sequelae of tuberculosis	A00.B99	France	382.27	1	yes
B90	Sequelae of tuberculosis	A00.B99	Germany	84.94	1	yes
B90	Sequelae of tuberculosis	A00.B99	Hungary	57.02	2	yes
B90	Sequelae of tuberculosis	A00.B99	Latvia	14.96	0	no
B90	Sequelae of tuberculosis	A00.B99	Netherlands	38.94	1	yes
B90	Sequelae of tuberculosis	A00.B99	Norway	28.05	1	yes
B90	Sequelae of tuberculosis	A00.B99	Poland	32.50	3	yes
B90	Sequelae of tuberculosis	A00.B99	Spain	103.14	1	yes
B90	Sequelae of tuberculosis	A00.B99	Sweden	37.15	1	yes
B90	Sequelae of tuberculosis	A00.B99	Switzerland	11.52	1	yes
B90	Sequelae of tuberculosis	A00.B99	United Kingdom	66.69	2	yes
B91	Sequelae of poliomyelitis	A00.B99	Austria	11.54	0	no
B91	Sequelae of poliomyelitis	A00.B99	Belgium	7.38	1	yes
B91	Sequelae of poliomyelitis	A00.B99	Finland	7.76	0	no
B91	Sequelae of poliomyelitis	A00.B99	France	51.98	0	no
B91	Sequelae of poliomyelitis	A00.B99	Germany	23.72	2	yes
B91	Sequelae of poliomyelitis	A00.B99	Hungary	5.84	1	yes
B91	Sequelae of poliomyelitis	A00.B99	Netherlands	6.80	0	no
B91	Sequelae of poliomyelitis	A00.B99	Norway	15.36	0	no
B91	Sequelae of poliomyelitis	A00.B99	Spain	10.77	0	no

For peer review only

B91	Sequelae of poliomyelitis	A00.B99	Sweden	28.64	1	yes
B91	Sequelae of poliomyelitis	A00.B99	Switzerland	10.82	0	no
B91	Sequelae of poliomyelitis	A00.B99	United Kingdom	37.17	0	no
B94	Sequelae of other and unspecified infectious and parasitic diseases	A00.B99	Finland	6.64	0	no
B94	Sequelae of other and unspecified infectious and parasitic diseases	A00.B99	France	37.63	2	yes
B94	Sequelae of other and unspecified infectious and parasitic diseases	A00.B99	Germany	137.31	2	yes
B94	Sequelae of other and unspecified infectious and parasitic diseases	A00.B99	Poland	11.66	0	no
B94	Sequelae of other and unspecified infectious and parasitic diseases	A00.B99	Sweden	6.05	2	yes
B94	Sequelae of other and unspecified infectious and parasitic diseases	A00.B99	Switzerland	72.26	0	no
B94	Sequelae of other and unspecified infectious and parasitic diseases	A00.B99	United Kingdom	10.02	3	yes
B99	Other and unspecified infectious diseases	A00.B99	Austria	31.26	3	yes
B99	Other and unspecified infectious diseases	A00.B99	Belgium	349.03	1	yes
B99	Other and unspecified infectious diseases	A00.B99	Denmark	60.41	3	yes
B99	Other and unspecified infectious diseases	A00.B99	France	1540.91	2	yes
B99	Other and unspecified infectious diseases	A00.B99	Germany	1104.45	1	yes
B99	Other and unspecified infectious diseases	A00.B99	Netherlands	452.61	3	yes
B99	Other and unspecified infectious diseases	A00.B99	Norway	193.70	2	yes
B99	Other and unspecified infectious diseases	A00.B99	Spain	76.30	3	yes
B99	Other and unspecified infectious diseases	A00.B99	Sweden	352.47	1	yes
B99	Other and unspecified infectious diseases	A00.B99	Switzerland	116.27	1	yes
B99	Other and unspecified infectious diseases	A00.B99	United Kingdom	22.50	2	yes
C00	Malignant neoplasm of lip	C00.D48	Croatia	13.54	1	yes
C00	Malignant neoplasm of lip	C00.D48	Czech Republic	14.75	0	no
C00	Malignant neoplasm of lip	C00.D48	Denmark	6.12	0	no
C00	Malignant neoplasm of lip	C00.D48	France	42.06	1	yes
C00	Malignant neoplasm of lip	C00.D48	Germany	44.31	0	no
C00	Malignant neoplasm of lip	C00.D48	Hungary	31.29	1	yes
C00	Malignant neoplasm of lip	C00.D48	Latvia	10.48	0	no
C00	Malignant neoplasm of lip	C00.D48	Lithuania	11.79	1	yes
C00	Malignant neoplasm of lip	C00.D48	Netherlands	9.01	0	no
C00	Malignant neoplasm of lip	C00.D48	Poland	177.06	3	yes
C00	Malignant neoplasm of lip	C00.D48	Romania	168.30	1	yes
C00	Malignant neoplasm of lip	C00.D48	Spain	66.63	1	yes
C00	Malignant neoplasm of lip	C00.D48	Sweden	5.83	0	no
C00	Malignant neoplasm of lip	C00.D48	United Kingdom	24.67	0	no
C01	Malignant neoplasm of base of tongue	C00.D48	Austria	79.35	2	yes
C01	Malignant neoplasm of base of tongue	C00.D48	Belgium	37.74	0	no
C01	Malignant neoplasm of base of tongue	C00.D48	Croatia	26.88	0	no
C01	Malignant neoplasm of base of tongue	C00.D48	Czech Republic	62.68	0	no
C01	Malignant neoplasm of base of tongue	C00.D48	Denmark	9.64	0	no
C01	Malignant neoplasm of base of tongue	C00.D48	Estonia	5.03	1	yes
C01	Malignant neoplasm of base of tongue	C00.D48	Finland	8.20	0	no
C01	Malignant neoplasm of base of tongue	C00.D48	France	375.84	1	yes
C01	Malignant neoplasm of base of tongue	C00.D48	Germany	525.23	0	no
C01	Malignant neoplasm of base of tongue	C00.D48	Hungary	251.33	0	no

For peer review only

C01	Malignant neoplasm of base of tongue	C00.D48	Latvia	14.33	1	yes
C01	Malignant neoplasm of base of tongue	C00.D48	Lithuania	29.48	0	no
C01	Malignant neoplasm of base of tongue	C00.D48	Netherlands	38.32	0	no
C01	Malignant neoplasm of base of tongue	C00.D48	Norway	6.53	1	yes
C01	Malignant neoplasm of base of tongue	C00.D48	Poland	125.22	3	yes
C01	Malignant neoplasm of base of tongue	C00.D48	Romania	117.99	1	yes
C01	Malignant neoplasm of base of tongue	C00.D48	Spain	133.89	2	yes
C01	Malignant neoplasm of base of tongue	C00.D48	Sweden	30.14	1	yes
C01	Malignant neoplasm of base of tongue	C00.D48	Switzerland	40.96	0	no
C01	Malignant neoplasm of base of tongue	C00.D48	United Kingdom	59.67	0	no
C02	Malignant neoplasm of other and unspecified parts of tongue	C00.D48	Austria	40.61	0	no
C02	Malignant neoplasm of other and unspecified parts of tongue	C00.D48	Belgium	70.54	3	yes
C02	Malignant neoplasm of other and unspecified parts of tongue	C00.D48	Croatia	62.73	1	yes
C02	Malignant neoplasm of other and unspecified parts of tongue	C00.D48	Czech Republic	103.43	1	yes
C02	Malignant neoplasm of other and unspecified parts of tongue	C00.D48	Denmark	44.16	0	no
C02	Malignant neoplasm of other and unspecified parts of tongue	C00.D48	Estonia	19.27	0	no
C02	Malignant neoplasm of other and unspecified parts of tongue	C00.D48	Finland	41.67	0	no
C02	Malignant neoplasm of other and unspecified parts of tongue	C00.D48	France	560.41	1	yes
C02	Malignant neoplasm of other and unspecified parts of tongue	C00.D48	Germany	301.54	3	yes
C02	Malignant neoplasm of other and unspecified parts of tongue	C00.D48	Hungary	174.13	1	yes
C02	Malignant neoplasm of other and unspecified parts of tongue	C00.D48	Latvia	21.68	0	no
C02	Malignant neoplasm of other and unspecified parts of tongue	C00.D48	Lithuania	28.45	0	no
C02	Malignant neoplasm of other and unspecified parts of tongue	C00.D48	Netherlands	88.33	0	no
C02	Malignant neoplasm of other and unspecified parts of tongue	C00.D48	Norway	30.23	0	no
C02	Malignant neoplasm of other and unspecified parts of tongue	C00.D48	Poland	345.93	3	yes
C02	Malignant neoplasm of other and unspecified parts of tongue	C00.D48	Romania	278.13	2	yes
C02	Malignant neoplasm of other and unspecified parts of tongue	C00.D48	Slovenia	13.17	0	no
C02	Malignant neoplasm of other and unspecified parts of tongue	C00.D48	Spain	455.86	0	no
C02	Malignant neoplasm of other and unspecified parts of tongue	C00.D48	Sweden	39.11	0	no
C02	Malignant neoplasm of other and unspecified parts of tongue	C00.D48	Switzerland	37.96	1	yes
C02	Malignant neoplasm of other and unspecified parts of tongue	C00.D48	United Kingdom	586.43	2	yes
C03	Malignant neoplasm of gum	C00.D48	Austria	19.94	3	yes
C03	Malignant neoplasm of gum	C00.D48	Croatia	7.63	0	no
C03	Malignant neoplasm of gum	C00.D48	Czech Republic	17.00	0	no
C03	Malignant neoplasm of gum	C00.D48	Denmark	8.38	0	no
C03	Malignant neoplasm of gum	C00.D48	Finland	16.64	0	no

For peer review only

C03	Malignant neoplasm of gum	C00.D48	France	49.26	0	no
C03	Malignant neoplasm of gum	C00.D48	Germany	49.45	1	yes
C03	Malignant neoplasm of gum	C00.D48	Hungary	33.09	2	yes
C03	Malignant neoplasm of gum	C00.D48	Netherlands	5.92	0	no
C03	Malignant neoplasm of gum	C00.D48	Norway	10.66	2	yes
C03	Malignant neoplasm of gum	C00.D48	Poland	38.53	1	yes
C03	Malignant neoplasm of gum	C00.D48	Romania	26.53	1	yes
C03	Malignant neoplasm of gum	C00.D48	Spain	36.90	0	no
C03	Malignant neoplasm of gum	C00.D48	Sweden	20.83	0	no
C03	Malignant neoplasm of gum	C00.D48	Switzerland	9.34	1	yes
C03	Malignant neoplasm of gum	C00.D48	United Kingdom	95.16	1	yes
C04	Malignant neoplasm of floor of mouth	C00.D48	Austria	68.75	0	no
C04	Malignant neoplasm of floor of mouth	C00.D48	Belgium	90.69	1	yes
C04	Malignant neoplasm of floor of mouth	C00.D48	Croatia	21.15	1	yes
C04	Malignant neoplasm of floor of mouth	C00.D48	Czech Republic	72.96	0	no
C04	Malignant neoplasm of floor of mouth	C00.D48	Denmark	16.38	2	yes
C04	Malignant neoplasm of floor of mouth	C00.D48	Estonia	8.64	0	no
C04	Malignant neoplasm of floor of mouth	C00.D48	Finland	16.71	0	no
C04	Malignant neoplasm of floor of mouth	C00.D48	France	308.86	1	yes
C04	Malignant neoplasm of floor of mouth	C00.D48	Germany	897.46	3	yes
C04	Malignant neoplasm of floor of mouth	C00.D48	Hungary	117.26	1	yes
C04	Malignant neoplasm of floor of mouth	C00.D48	Latvia	14.46	1	yes
C04	Malignant neoplasm of floor of mouth	C00.D48	Lithuania	33.08	1	yes
C04	Malignant neoplasm of floor of mouth	C00.D48	Netherlands	108.32	1	yes
C04	Malignant neoplasm of floor of mouth	C00.D48	Norway	10.56	2	yes
C04	Malignant neoplasm of floor of mouth	C00.D48	Poland	288.98	1	yes
C04	Malignant neoplasm of floor of mouth	C00.D48	Romania	178.40	0	no
C04	Malignant neoplasm of floor of mouth	C00.D48	Slovenia	10.98	0	no
C04	Malignant neoplasm of floor of mouth	C00.D48	Spain	182.01	1	yes
C04	Malignant neoplasm of floor of mouth	C00.D48	Sweden	20.90	2	yes
C04	Malignant neoplasm of floor of mouth	C00.D48	Switzerland	34.98	0	no
C04	Malignant neoplasm of floor of mouth	C00.D48	United Kingdom	76.80	2	yes
C05	Malignant neoplasm of palate	C00.D48	Austria	8.90	0	no
C05	Malignant neoplasm of palate	C00.D48	Belgium	12.69	0	no
C05	Malignant neoplasm of palate	C00.D48	Croatia	12.38	0	no
C05	Malignant neoplasm of palate	C00.D48	Czech Republic	25.30	0	no
C05	Malignant neoplasm of palate	C00.D48	Denmark	5.40	0	no
C05	Malignant neoplasm of palate	C00.D48	Finland	7.06	0	no
C05	Malignant neoplasm of palate	C00.D48	France	125.55	0	no
C05	Malignant neoplasm of palate	C00.D48	Germany	72.64	0	no
C05	Malignant neoplasm of palate	C00.D48	Hungary	43.92	0	no
C05	Malignant neoplasm of palate	C00.D48	Latvia	6.54	0	no
C05	Malignant neoplasm of palate	C00.D48	Lithuania	9.36	1	yes
C05	Malignant neoplasm of palate	C00.D48	Netherlands	10.53	0	no
C05	Malignant neoplasm of palate	C00.D48	Poland	73.60	2	yes
C05	Malignant neoplasm of palate	C00.D48	Romania	58.09	0	no
C05	Malignant neoplasm of palate	C00.D48	Spain	61.92	0	no
C05	Malignant neoplasm of palate	C00.D48	Sweden	7.42	0	no
C05	Malignant neoplasm of palate	C00.D48	Switzerland	9.19	2	yes
C05	Malignant neoplasm of palate	C00.D48	United Kingdom	81.77	0	no

For peer review only

C06	Malignant neoplasm of other and unspecified parts of mouth	C00.D48	Austria	9.67	0	no
C06	Malignant neoplasm of other and unspecified parts of mouth	C00.D48	Belgium	30.27	0	no
C06	Malignant neoplasm of other and unspecified parts of mouth	C00.D48	Croatia	18.86	0	no
C06	Malignant neoplasm of other and unspecified parts of mouth	C00.D48	Czech Republic	33.08	1	yes
C06	Malignant neoplasm of other and unspecified parts of mouth	C00.D48	Denmark	69.13	1	yes
C06	Malignant neoplasm of other and unspecified parts of mouth	C00.D48	Finland	12.15	1	yes
C06	Malignant neoplasm of other and unspecified parts of mouth	C00.D48	France	189.13	2	yes
C06	Malignant neoplasm of other and unspecified parts of mouth	C00.D48	Germany	108.38	2	yes
C06	Malignant neoplasm of other and unspecified parts of mouth	C00.D48	Hungary	76.93	0	no
C06	Malignant neoplasm of other and unspecified parts of mouth	C00.D48	Latvia	15.00	1	yes
C06	Malignant neoplasm of other and unspecified parts of mouth	C00.D48	Lithuania	11.43	1	yes
C06	Malignant neoplasm of other and unspecified parts of mouth	C00.D48	Netherlands	39.38	1	yes
C06	Malignant neoplasm of other and unspecified parts of mouth	C00.D48	Norway	21.22	0	no
C06	Malignant neoplasm of other and unspecified parts of mouth	C00.D48	Poland	138.57	0	no
C06	Malignant neoplasm of other and unspecified parts of mouth	C00.D48	Romania	77.35	0	no
C06	Malignant neoplasm of other and unspecified parts of mouth	C00.D48	Slovenia	14.28	0	no
C06	Malignant neoplasm of other and unspecified parts of mouth	C00.D48	Spain	246.18	0	no
C06	Malignant neoplasm of other and unspecified parts of mouth	C00.D48	Sweden	27.61	0	no
C06	Malignant neoplasm of other and unspecified parts of mouth	C00.D48	Switzerland	16.66	0	no
C06	Malignant neoplasm of other and unspecified parts of mouth	C00.D48	United Kingdom	304.74	2	yes
C07	Malignant neoplasm of parotid gland	C00.D48	Austria	33.33	0	no
C07	Malignant neoplasm of parotid gland	C00.D48	Belgium	29.77	2	yes
C07	Malignant neoplasm of parotid gland	C00.D48	Croatia	16.90	0	no
C07	Malignant neoplasm of parotid gland	C00.D48	Czech Republic	45.97	1	yes
C07	Malignant neoplasm of parotid gland	C00.D48	Denmark	19.56	0	no
C07	Malignant neoplasm of parotid gland	C00.D48	Estonia	6.31	0	no
C07	Malignant neoplasm of parotid gland	C00.D48	Finland	16.02	0	no
C07	Malignant neoplasm of parotid gland	C00.D48	France	197.64	0	no
C07	Malignant neoplasm of parotid gland	C00.D48	Germany	233.79	0	no
C07	Malignant neoplasm of parotid gland	C00.D48	Hungary	52.13	0	no
C07	Malignant neoplasm of parotid gland	C00.D48	Latvia	9.48	1	yes
C07	Malignant neoplasm of parotid gland	C00.D48	Lithuania	14.50	0	no
C07	Malignant neoplasm of parotid gland	C00.D48	Netherlands	41.98	0	no
C07	Malignant neoplasm of parotid gland	C00.D48	Norway	13.37	0	no
C07	Malignant neoplasm of parotid gland	C00.D48	Poland	167.35	0	no
C07	Malignant neoplasm of parotid gland	C00.D48	Romania	87.04	0	no
C07	Malignant neoplasm of parotid gland	C00.D48	Spain	152.25	0	no

For peer review only

C07	Malignant neoplasm of parotid gland	C00.D48	Sweden	22.56	0	no
C07	Malignant neoplasm of parotid gland	C00.D48	Switzerland	20.60	0	no
C07	Malignant neoplasm of parotid gland	C00.D48	United Kingdom	178.31	0	no
C08	Malignant neoplasm of other and unspecified major salivary glands	C00.D48	Austria	8.32	0	no
C08	Malignant neoplasm of other and unspecified major salivary glands	C00.D48	Belgium	6.80	2	yes
C08	Malignant neoplasm of other and unspecified major salivary glands	C00.D48	Croatia	5.62	0	no
C08	Malignant neoplasm of other and unspecified major salivary glands	C00.D48	Czech Republic	19.83	0	no
C08	Malignant neoplasm of other and unspecified major salivary glands	C00.D48	Denmark	6.32	1	yes
C08	Malignant neoplasm of other and unspecified major salivary glands	C00.D48	Finland	5.97	0	no
C08	Malignant neoplasm of other and unspecified major salivary glands	C00.D48	France	39.21	0	no
C08	Malignant neoplasm of other and unspecified major salivary glands	C00.D48	Germany	52.63	0	no
C08	Malignant neoplasm of other and unspecified major salivary glands	C00.D48	Hungary	25.41	0	no
C08	Malignant neoplasm of other and unspecified major salivary glands	C00.D48	Latvia	8.95	0	no
C08	Malignant neoplasm of other and unspecified major salivary glands	C00.D48	Lithuania	9.96	0	no
C08	Malignant neoplasm of other and unspecified major salivary glands	C00.D48	Netherlands	14.73	2	yes
C08	Malignant neoplasm of other and unspecified major salivary glands	C00.D48	Poland	85.62	2	yes
C08	Malignant neoplasm of other and unspecified major salivary glands	C00.D48	Romania	20.21	1	yes
C08	Malignant neoplasm of other and unspecified major salivary glands	C00.D48	Spain	25.15	1	yes
C08	Malignant neoplasm of other and unspecified major salivary glands	C00.D48	Sweden	12.83	0	no
C08	Malignant neoplasm of other and unspecified major salivary glands	C00.D48	Switzerland	5.53	0	no
C08	Malignant neoplasm of other and unspecified major salivary glands	C00.D48	United Kingdom	45.06	0	no
C09	Malignant neoplasm of tonsil	C00.D48	Austria	58.14	0	no
C09	Malignant neoplasm of tonsil	C00.D48	Belgium	45.70	2	yes
C09	Malignant neoplasm of tonsil	C00.D48	Croatia	33.59	0	no
C09	Malignant neoplasm of tonsil	C00.D48	Czech Republic	101.99	1	yes
C09	Malignant neoplasm of tonsil	C00.D48	Denmark	49.01	0	no
C09	Malignant neoplasm of tonsil	C00.D48	Estonia	5.50	1	yes
C09	Malignant neoplasm of tonsil	C00.D48	Finland	19.56	1	yes
C09	Malignant neoplasm of tonsil	C00.D48	France	416.60	2	yes
C09	Malignant neoplasm of tonsil	C00.D48	Germany	373.42	2	yes
C09	Malignant neoplasm of tonsil	C00.D48	Hungary	111.34	1	yes
C09	Malignant neoplasm of tonsil	C00.D48	Latvia	15.96	1	yes
C09	Malignant neoplasm of tonsil	C00.D48	Lithuania	18.23	0	no
C09	Malignant neoplasm of tonsil	C00.D48	Netherlands	32.47	1	yes
C09	Malignant neoplasm of tonsil	C00.D48	Norway	20.45	1	yes
C09	Malignant neoplasm of tonsil	C00.D48	Poland	267.79	1	yes
C09	Malignant neoplasm of tonsil	C00.D48	Romania	240.86	1	yes
C09	Malignant neoplasm of tonsil	C00.D48	Spain	157.66	0	no

For peer review only

C09	Malignant neoplasm of tonsil	C00.D48	Sweden	43.23	0	no
C09	Malignant neoplasm of tonsil	C00.D48	Switzerland	33.90	0	no
C09	Malignant neoplasm of tonsil	C00.D48	United Kingdom	245.10	1	yes
C10	Malignant neoplasm of oropharynx	C00.D48	Austria	57.21	1	yes
C10	Malignant neoplasm of oropharynx	C00.D48	Belgium	49.14	1	yes
C10	Malignant neoplasm of oropharynx	C00.D48	Croatia	37.31	1	yes
C10	Malignant neoplasm of oropharynx	C00.D48	Czech Republic	75.43	1	yes
C10	Malignant neoplasm of oropharynx	C00.D48	Denmark	35.20	2	yes
C10	Malignant neoplasm of oropharynx	C00.D48	Estonia	13.57	0	no
C10	Malignant neoplasm of oropharynx	C00.D48	Finland	5.95	2	yes
C10	Malignant neoplasm of oropharynx	C00.D48	France	567.96	2	yes
C10	Malignant neoplasm of oropharynx	C00.D48	Germany	918.14	1	yes
C10	Malignant neoplasm of oropharynx	C00.D48	Hungary	159.65	1	yes
C10	Malignant neoplasm of oropharynx	C00.D48	Latvia	7.71	3	yes
C10	Malignant neoplasm of oropharynx	C00.D48	Lithuania	37.27	3	yes
C10	Malignant neoplasm of oropharynx	C00.D48	Netherlands	87.43	0	no
C10	Malignant neoplasm of oropharynx	C00.D48	Norway	11.87	2	yes
C10	Malignant neoplasm of oropharynx	C00.D48	Poland	158.88	1	yes
C10	Malignant neoplasm of oropharynx	C00.D48	Romania	238.87	1	yes
C10	Malignant neoplasm of oropharynx	C00.D48	Slovenia	60.48	0	no
C10	Malignant neoplasm of oropharynx	C00.D48	Spain	306.33	2	yes
C10	Malignant neoplasm of oropharynx	C00.D48	Sweden	8.94	0	no
C10	Malignant neoplasm of oropharynx	C00.D48	Switzerland	51.20	0	no
C10	Malignant neoplasm of oropharynx	C00.D48	United Kingdom	233.90	3	yes
C11	Malignant neoplasm of nasopharynx	C00.D48	Austria	20.86	0	no
C11	Malignant neoplasm of nasopharynx	C00.D48	Belgium	18.95	0	no
C11	Malignant neoplasm of nasopharynx	C00.D48	Croatia	16.97	0	no
C11	Malignant neoplasm of nasopharynx	C00.D48	Czech Republic	33.27	0	no
C11	Malignant neoplasm of nasopharynx	C00.D48	Denmark	11.03	0	no
C11	Malignant neoplasm of nasopharynx	C00.D48	Estonia	5.33	0	no
C11	Malignant neoplasm of nasopharynx	C00.D48	Finland	7.62	0	no
C11	Malignant neoplasm of nasopharynx	C00.D48	France	142.89	1	yes
C11	Malignant neoplasm of nasopharynx	C00.D48	Germany	155.82	0	no
C11	Malignant neoplasm of nasopharynx	C00.D48	Hungary	48.80	0	no
C11	Malignant neoplasm of nasopharynx	C00.D48	Latvia	6.37	0	no
C11	Malignant neoplasm of nasopharynx	C00.D48	Lithuania	10.94	0	no
C11	Malignant neoplasm of nasopharynx	C00.D48	Netherlands	34.16	1	yes
C11	Malignant neoplasm of nasopharynx	C00.D48	Norway	6.87	2	yes
C11	Malignant neoplasm of nasopharynx	C00.D48	Poland	160.56	1	yes
C11	Malignant neoplasm of nasopharynx	C00.D48	Romania	183.56	0	no
C11	Malignant neoplasm of nasopharynx	C00.D48	Spain	221.66	1	yes
C11	Malignant neoplasm of nasopharynx	C00.D48	Sweden	16.23	1	yes
C11	Malignant neoplasm of nasopharynx	C00.D48	Switzerland	13.72	1	yes
C11	Malignant neoplasm of nasopharynx	C00.D48	United Kingdom	132.46	0	no
C12	Malignant neoplasm of pyriform sinus	C00.D48	Austria	5.71	0	no
C12	Malignant neoplasm of pyriform sinus	C00.D48	Belgium	20.60	0	no
C12	Malignant neoplasm of pyriform sinus	C00.D48	Czech Republic	12.21	0	no
C12	Malignant neoplasm of pyriform sinus	C00.D48	Estonia	5.59	1	yes
C12	Malignant neoplasm of pyriform sinus	C00.D48	France	452.13	2	yes
C12	Malignant neoplasm of pyriform sinus	C00.D48	Germany	6.04	0	no

For peer review only

C12	Malignant neoplasm of pyriform sinus	C00.D48	Hungary	10.41	0	no
C12	Malignant neoplasm of pyriform sinus	C00.D48	Lithuania	7.18	0	no
C12	Malignant neoplasm of pyriform sinus	C00.D48	Netherlands	10.51	1	yes
C12	Malignant neoplasm of pyriform sinus	C00.D48	Poland	26.12	3	yes
C12	Malignant neoplasm of pyriform sinus	C00.D48	Romania	56.71	1	yes
C12	Malignant neoplasm of pyriform sinus	C00.D48	Spain	113.60	1	yes
C12	Malignant neoplasm of pyriform sinus	C00.D48	Switzerland	14.19	1	yes
C12	Malignant neoplasm of pyriform sinus	C00.D48	United Kingdom	86.45	0	no
C13	Malignant neoplasm of hypopharynx	C00.D48	Austria	77.90	0	no
C13	Malignant neoplasm of hypopharynx	C00.D48	Belgium	37.78	0	no
C13	Malignant neoplasm of hypopharynx	C00.D48	Croatia	84.24	1	yes
C13	Malignant neoplasm of hypopharynx	C00.D48	Czech Republic	81.59	1	yes
C13	Malignant neoplasm of hypopharynx	C00.D48	Denmark	48.97	1	yes
C13	Malignant neoplasm of hypopharynx	C00.D48	Estonia	9.07	0	no
C13	Malignant neoplasm of hypopharynx	C00.D48	Finland	21.90	0	no
C13	Malignant neoplasm of hypopharynx	C00.D48	France	337.88	2	yes
C13	Malignant neoplasm of hypopharynx	C00.D48	Germany	984.18	1	yes
C13	Malignant neoplasm of hypopharynx	C00.D48	Hungary	291.49	1	yes
C13	Malignant neoplasm of hypopharynx	C00.D48	Latvia	24.63	1	yes
C13	Malignant neoplasm of hypopharynx	C00.D48	Lithuania	49.68	0	no
C13	Malignant neoplasm of hypopharynx	C00.D48	Netherlands	54.44	0	no
C13	Malignant neoplasm of hypopharynx	C00.D48	Norway	17.76	1	yes
C13	Malignant neoplasm of hypopharynx	C00.D48	Poland	211.55	2	yes
C13	Malignant neoplasm of hypopharynx	C00.D48	Romania	164.15	3	yes
C13	Malignant neoplasm of hypopharynx	C00.D48	Slovenia	32.51	0	no
C13	Malignant neoplasm of hypopharynx	C00.D48	Spain	189.71	0	no
C13	Malignant neoplasm of hypopharynx	C00.D48	Sweden	41.51	1	yes
C13	Malignant neoplasm of hypopharynx	C00.D48	Switzerland	55.32	0	no
C13	Malignant neoplasm of hypopharynx	C00.D48	United Kingdom	134.21	2	yes
C14	Malignant neoplasm of other and ill-defined sites in the lip, oral cavity and pharynx	C00.D48	Austria	21.73	0	no
C14	Malignant neoplasm of other and ill-defined sites in the lip, oral cavity and pharynx	C00.D48	Belgium	110.39	0	no
C14	Malignant neoplasm of other and ill-defined sites in the lip, oral cavity and pharynx	C00.D48	Croatia	24.02	1	yes
C14	Malignant neoplasm of other and ill-defined sites in the lip, oral cavity and pharynx	C00.D48	Czech Republic	35.50	1	yes
C14	Malignant neoplasm of other and ill-defined sites in the lip, oral cavity and pharynx	C00.D48	Denmark	19.52	1	yes
C14	Malignant neoplasm of other and ill-defined sites in the lip, oral cavity and pharynx	C00.D48	Estonia	5.59	1	yes
C14	Malignant neoplasm of other and ill-defined sites in the lip, oral cavity and pharynx	C00.D48	France	769.81	1	yes
C14	Malignant neoplasm of other and ill-defined sites in the lip, oral cavity and pharynx	C00.D48	Germany	243.93	1	yes
C14	Malignant neoplasm of other and ill-defined sites in the lip, oral cavity and pharynx	C00.D48	Hungary	210.09	2	yes
C14	Malignant neoplasm of other and ill-defined sites in the lip, oral cavity and pharynx	C00.D48	Latvia	19.44	3	yes
C14	Malignant neoplasm of other and ill-defined sites in the lip, oral cavity and pharynx	C00.D48	Lithuania	5.51	0	no
C14	Malignant neoplasm of other and ill-defined sites in the lip, oral cavity and pharynx	C00.D48	Netherlands	99.59	0	no
C14	Malignant neoplasm of other and ill-defined sites in the lip, oral cavity and pharynx	C00.D48	Norway	6.04	0	no

For peer review only

C14	Malignant neoplasm of other and ill-defined sites in the lip, oral cavity and pharynx	C00.D48	Poland	290.56	2	yes
C14	Malignant neoplasm of other and ill-defined sites in the lip, oral cavity and pharynx	C00.D48	Romania	293.84	2	yes
C14	Malignant neoplasm of other and ill-defined sites in the lip, oral cavity and pharynx	C00.D48	Slovenia	9.17	0	no
C14	Malignant neoplasm of other and ill-defined sites in the lip, oral cavity and pharynx	C00.D48	Spain	188.71	1	yes
C14	Malignant neoplasm of other and ill-defined sites in the lip, oral cavity and pharynx	C00.D48	Sweden	8.26	1	yes
C14	Malignant neoplasm of other and ill-defined sites in the lip, oral cavity and pharynx	C00.D48	Switzerland	71.73	1	yes
C14	Malignant neoplasm of other and ill-defined sites in the lip, oral cavity and pharynx	C00.D48	United Kingdom	238.72	0	no
C15	Malignant neoplasm of oesophagus	C00.D48	Austria	356.66	0	no
C15	Malignant neoplasm of oesophagus	C00.D48	Belgium	735.66	0	no
C15	Malignant neoplasm of oesophagus	C00.D48	Croatia	223.12	1	yes
C15	Malignant neoplasm of oesophagus	C00.D48	Czech Republic	515.38	0	no
C15	Malignant neoplasm of oesophagus	C00.D48	Denmark	449.43	1	yes
C15	Malignant neoplasm of oesophagus	C00.D48	Estonia	63.45	1	yes
C15	Malignant neoplasm of oesophagus	C00.D48	Finland	244.80	0	no
C15	Malignant neoplasm of oesophagus	C00.D48	France	4390.10	2	yes
C15	Malignant neoplasm of oesophagus	C00.D48	Germany	4879.38	1	yes
C15	Malignant neoplasm of oesophagus	C00.D48	Hungary	659.75	1	yes
C15	Malignant neoplasm of oesophagus	C00.D48	Latvia	121.07	1	yes
C15	Malignant neoplasm of oesophagus	C00.D48	Lithuania	193.08	1	yes
C15	Malignant neoplasm of oesophagus	C00.D48	Netherlands	1803.26	2	yes
C15	Malignant neoplasm of oesophagus	C00.D48	Norway	224.27	0	no
C15	Malignant neoplasm of oesophagus	C00.D48	Poland	1818.22	1	yes
C15	Malignant neoplasm of oesophagus	C00.D48	Romania	737.89	2	yes
C15	Malignant neoplasm of oesophagus	C00.D48	Slovenia	97.39	1	yes
C15	Malignant neoplasm of oesophagus	C00.D48	Spain	2061.63	2	yes
C15	Malignant neoplasm of oesophagus	C00.D48	Sweden	425.26	0	no
C15	Malignant neoplasm of oesophagus	C00.D48	Switzerland	475.80	0	no
C15	Malignant neoplasm of oesophagus	C00.D48	United Kingdom	8568.89	3	yes
C16	Malignant neoplasm of stomach	C00.D48	Austria	1070.57	2	yes
C16	Malignant neoplasm of stomach	C00.D48	Belgium	926.52	2	yes
C16	Malignant neoplasm of stomach	C00.D48	Croatia	1020.83	1	yes
C16	Malignant neoplasm of stomach	C00.D48	Czech Republic	1573.28	2	yes
C16	Malignant neoplasm of stomach	C00.D48	Denmark	451.24	1	yes
C16	Malignant neoplasm of stomach	C00.D48	Estonia	350.16	1	yes
C16	Malignant neoplasm of stomach	C00.D48	Finland	572.62	1	yes
C16	Malignant neoplasm of stomach	C00.D48	France	5362.07	2	yes
C16	Malignant neoplasm of stomach	C00.D48	Germany	10971.18	2	yes
C16	Malignant neoplasm of stomach	C00.D48	Hungary	2120.23	2	yes
C16	Malignant neoplasm of stomach	C00.D48	Latvia	591.28	1	yes
C16	Malignant neoplasm of stomach	C00.D48	Lithuania	853.87	2	yes
C16	Malignant neoplasm of stomach	C00.D48	Netherlands	1810.99	1	yes
C16	Malignant neoplasm of stomach	C00.D48	Norway	454.29	2	yes
C16	Malignant neoplasm of stomach	C00.D48	Poland	7313.83	2	yes
C16	Malignant neoplasm of stomach	C00.D48	Romania	4575.15	1	yes
C16	Malignant neoplasm of stomach	C00.D48	Slovenia	432.00	1	yes

For peer review only

C16	Malignant neoplasm of stomach	C00.D48	Spain	6473.17	3	yes
C16	Malignant neoplasm of stomach	C00.D48	Sweden	747.80	2	yes
C16	Malignant neoplasm of stomach	C00.D48	Switzerland	612.56	2	yes
C16	Malignant neoplasm of stomach	C00.D48	United Kingdom	6114.85	2	yes
C17	Malignant neoplasm of small intestine	C00.D48	Austria	55.76	1	yes
C17	Malignant neoplasm of small intestine	C00.D48	Belgium	58.20	0	no
C17	Malignant neoplasm of small intestine	C00.D48	Croatia	30.79	1	yes
C17	Malignant neoplasm of small intestine	C00.D48	Czech Republic	87.88	0	no
C17	Malignant neoplasm of small intestine	C00.D48	Denmark	53.46	1	yes
C17	Malignant neoplasm of small intestine	C00.D48	Estonia	12.00	0	no
C17	Malignant neoplasm of small intestine	C00.D48	Finland	49.50	1	yes
C17	Malignant neoplasm of small intestine	C00.D48	France	412.64	2	yes
C17	Malignant neoplasm of small intestine	C00.D48	Germany	454.50	2	yes
C17	Malignant neoplasm of small intestine	C00.D48	Hungary	77.66	2	yes
C17	Malignant neoplasm of small intestine	C00.D48	Latvia	13.67	0	no
C17	Malignant neoplasm of small intestine	C00.D48	Lithuania	19.97	0	no
C17	Malignant neoplasm of small intestine	C00.D48	Netherlands	152.30	2	yes
C17	Malignant neoplasm of small intestine	C00.D48	Norway	59.95	0	no
C17	Malignant neoplasm of small intestine	C00.D48	Poland	213.91	0	no
C17	Malignant neoplasm of small intestine	C00.D48	Romania	113.53	2	yes
C17	Malignant neoplasm of small intestine	C00.D48	Slovenia	15.87	0	no
C17	Malignant neoplasm of small intestine	C00.D48	Spain	198.52	1	yes
C17	Malignant neoplasm of small intestine	C00.D48	Sweden	113.99	0	no
C17	Malignant neoplasm of small intestine	C00.D48	Switzerland	58.17	0	no
C17	Malignant neoplasm of small intestine	C00.D48	United Kingdom	431.82	1	yes
C18	Malignant neoplasm of colon	C00.D48	Austria	1700.02	2	yes
C18	Malignant neoplasm of colon	C00.D48	Belgium	2543.42	2	yes
C18	Malignant neoplasm of colon	C00.D48	Croatia	1285.25	2	yes
C18	Malignant neoplasm of colon	C00.D48	Czech Republic	2985.96	1	yes
C18	Malignant neoplasm of colon	C00.D48	Denmark	1627.29	1	yes
C18	Malignant neoplasm of colon	C00.D48	Estonia	289.28	0	no
C18	Malignant neoplasm of colon	C00.D48	Finland	774.46	0	no
C18	Malignant neoplasm of colon	C00.D48	France	13674.13	2	yes
C18	Malignant neoplasm of colon	C00.D48	Germany	18779.55	3	yes
C18	Malignant neoplasm of colon	C00.D48	Hungary	3741.13	1	yes
C18	Malignant neoplasm of colon	C00.D48	Latvia	443.31	0	no
C18	Malignant neoplasm of colon	C00.D48	Lithuania	574.65	2	yes
C18	Malignant neoplasm of colon	C00.D48	Netherlands	4612.69	2	yes
C18	Malignant neoplasm of colon	C00.D48	Norway	1370.52	1	yes
C18	Malignant neoplasm of colon	C00.D48	Poland	9098.25	0	no
C18	Malignant neoplasm of colon	C00.D48	Romania	3919.18	1	yes
C18	Malignant neoplasm of colon	C00.D48	Slovenia	502.93	2	yes
C18	Malignant neoplasm of colon	C00.D48	Spain	11910.60	3	yes
C18	Malignant neoplasm of colon	C00.D48	Sweden	1885.22	1	yes
C18	Malignant neoplasm of colon	C00.D48	Switzerland	1340.83	3	yes
C18	Malignant neoplasm of colon	C00.D48	United Kingdom	11554.54	1	yes
C19	Malignant neoplasm of rectosigmoid junction	C00.D48	Austria	41.14	1	yes
C19	Malignant neoplasm of rectosigmoid junction	C00.D48	Belgium	137.27	0	no
C19	Malignant neoplasm of rectosigmoid junction	C00.D48	Croatia	97.35	1	yes
C19	Malignant neoplasm of rectosigmoid junction	C00.D48	Czech Republic	662.87	1	yes

For peer review only

C19	Malignant neoplasm of rectosigmoid junction	C00.D48	Denmark	24.36	1	yes
C19	Malignant neoplasm of rectosigmoid junction	C00.D48	Estonia	29.59	1	yes
C19	Malignant neoplasm of rectosigmoid junction	C00.D48	Finland	55.83	1	yes
C19	Malignant neoplasm of rectosigmoid junction	C00.D48	France	1022.25	1	yes
C19	Malignant neoplasm of rectosigmoid junction	C00.D48	Germany	477.69	1	yes
C19	Malignant neoplasm of rectosigmoid junction	C00.D48	Hungary	208.50	2	yes
C19	Malignant neoplasm of rectosigmoid junction	C00.D48	Latvia	42.27	3	yes
C19	Malignant neoplasm of rectosigmoid junction	C00.D48	Lithuania	68.07	0	no
C19	Malignant neoplasm of rectosigmoid junction	C00.D48	Netherlands	133.45	0	no
C19	Malignant neoplasm of rectosigmoid junction	C00.D48	Norway	35.63	2	yes
C19	Malignant neoplasm of rectosigmoid junction	C00.D48	Poland	383.56	2	yes
C19	Malignant neoplasm of rectosigmoid junction	C00.D48	Romania	759.16	0	no
C19	Malignant neoplasm of rectosigmoid junction	C00.D48	Slovenia	73.77	1	yes
C19	Malignant neoplasm of rectosigmoid junction	C00.D48	Spain	710.54	0	no
C19	Malignant neoplasm of rectosigmoid junction	C00.D48	Sweden	55.67	0	no
C19	Malignant neoplasm of rectosigmoid junction	C00.D48	Switzerland	86.25	1	yes
C19	Malignant neoplasm of rectosigmoid junction	C00.D48	United Kingdom	1996.47	1	yes
C20	Malignant neoplasm of rectum	C00.D48	Austria	744.27	1	yes
C20	Malignant neoplasm of rectum	C00.D48	Belgium	572.53	1	yes
C20	Malignant neoplasm of rectum	C00.D48	Croatia	722.33	1	yes
C20	Malignant neoplasm of rectum	C00.D48	Czech Republic	1328.01	1	yes
C20	Malignant neoplasm of rectum	C00.D48	Denmark	609.46	1	yes
C20	Malignant neoplasm of rectum	C00.D48	Estonia	146.78	1	yes
C20	Malignant neoplasm of rectum	C00.D48	Finland	401.33	1	yes
C20	Malignant neoplasm of rectum	C00.D48	France	3471.90	1	yes
C20	Malignant neoplasm of rectum	C00.D48	Germany	7909.38	1	yes
C20	Malignant neoplasm of rectum	C00.D48	Hungary	1790.47	1	yes
C20	Malignant neoplasm of rectum	C00.D48	Latvia	286.46	1	yes
C20	Malignant neoplasm of rectum	C00.D48	Lithuania	435.08	1	yes
C20	Malignant neoplasm of rectum	C00.D48	Netherlands	1189.43	0	no
C20	Malignant neoplasm of rectum	C00.D48	Norway	462.34	2	yes
C20	Malignant neoplasm of rectum	C00.D48	Poland	3669.19	3	yes
C20	Malignant neoplasm of rectum	C00.D48	Romania	1627.47	2	yes
C20	Malignant neoplasm of rectum	C00.D48	Slovenia	293.89	1	yes
C20	Malignant neoplasm of rectum	C00.D48	Spain	2987.13	0	no
C20	Malignant neoplasm of rectum	C00.D48	Sweden	773.71	0	no
C20	Malignant neoplasm of rectum	C00.D48	Switzerland	448.72	1	yes
C20	Malignant neoplasm of rectum	C00.D48	United Kingdom	4641.80	1	yes
C21	Malignant neoplasm of anus and anal canal	C00.D48	Austria	38.45	0	no
C21	Malignant neoplasm of anus and anal canal	C00.D48	Belgium	25.74	1	yes
C21	Malignant neoplasm of anus and anal canal	C00.D48	Croatia	10.64	0	no
C21	Malignant neoplasm of anus and anal canal	C00.D48	Czech Republic	87.52	2	yes
C21	Malignant neoplasm of anus and anal canal	C00.D48	Denmark	31.92	3	yes
C21	Malignant neoplasm of anus and anal canal	C00.D48	Estonia	8.74	0	no
C21	Malignant neoplasm of anus and anal canal	C00.D48	Finland	16.80	0	no
C21	Malignant neoplasm of anus and anal canal	C00.D48	France	321.18	1	yes
C21	Malignant neoplasm of anus and anal canal	C00.D48	Germany	383.44	3	yes
C21	Malignant neoplasm of anus and anal canal	C00.D48	Hungary	37.52	2	yes
C21	Malignant neoplasm of anus and anal canal	C00.D48	Latvia	10.42	0	no
C21	Malignant neoplasm of anus and anal canal	C00.D48	Lithuania	14.96	1	yes

For peer review only

C21	Malignant neoplasm of anus and anal canal	C00.D48	Netherlands	39.39	0	no
C21	Malignant neoplasm of anus and anal canal	C00.D48	Norway	18.97	0	no
C21	Malignant neoplasm of anus and anal canal	C00.D48	Poland	736.10	3	yes
C21	Malignant neoplasm of anus and anal canal	C00.D48	Romania	164.76	2	yes
C21	Malignant neoplasm of anus and anal canal	C00.D48	Slovenia	6.64	1	yes
C21	Malignant neoplasm of anus and anal canal	C00.D48	Spain	94.19	1	yes
C21	Malignant neoplasm of anus and anal canal	C00.D48	Sweden	38.64	0	no
C21	Malignant neoplasm of anus and anal canal	C00.D48	Switzerland	42.06	0	no
C21	Malignant neoplasm of anus and anal canal	C00.D48	United Kingdom	319.50	2	yes
C22	Malignant neoplasm of liver and intrahepatic bile ducts	C00.D48	Austria	901.16	0	no
C22	Malignant neoplasm of liver and intrahepatic bile ducts	C00.D48	Belgium	818.27	3	yes
C22	Malignant neoplasm of liver and intrahepatic bile ducts	C00.D48	Croatia	512.11	3	yes
C22	Malignant neoplasm of liver and intrahepatic bile ducts	C00.D48	Czech Republic	1042.29	2	yes
C22	Malignant neoplasm of liver and intrahepatic bile ducts	C00.D48	Denmark	372.93	1	yes
C22	Malignant neoplasm of liver and intrahepatic bile ducts	C00.D48	Estonia	97.51	0	no
C22	Malignant neoplasm of liver and intrahepatic bile ducts	C00.D48	Finland	473.65	0	no
C22	Malignant neoplasm of liver and intrahepatic bile ducts	C00.D48	France	8425.34	1	yes
C22	Malignant neoplasm of liver and intrahepatic bile ducts	C00.D48	Germany	6839.91	2	yes
C22	Malignant neoplasm of liver and intrahepatic bile ducts	C00.D48	Hungary	940.63	2	yes
C22	Malignant neoplasm of liver and intrahepatic bile ducts	C00.D48	Latvia	149.78	0	no
C22	Malignant neoplasm of liver and intrahepatic bile ducts	C00.D48	Lithuania	190.35	1	yes
C22	Malignant neoplasm of liver and intrahepatic bile ducts	C00.D48	Netherlands	809.49	2	yes
C22	Malignant neoplasm of liver and intrahepatic bile ducts	C00.D48	Norway	201.73	1	yes
C22	Malignant neoplasm of liver and intrahepatic bile ducts	C00.D48	Poland	2658.83	3	yes
C22	Malignant neoplasm of liver and intrahepatic bile ducts	C00.D48	Romania	3121.36	1	yes
C22	Malignant neoplasm of liver and intrahepatic bile ducts	C00.D48	Slovenia	210.22	1	yes
C22	Malignant neoplasm of liver and intrahepatic bile ducts	C00.D48	Spain	5259.94	3	yes
C22	Malignant neoplasm of liver and intrahepatic bile ducts	C00.D48	Sweden	660.07	0	no
C22	Malignant neoplasm of liver and intrahepatic bile ducts	C00.D48	Switzerland	680.96	1	yes
C22	Malignant neoplasm of liver and intrahepatic bile ducts	C00.D48	United Kingdom	4051.88	2	yes
C23	Malignant neoplasm of gallbladder	C00.D48	Austria	156.39	1	yes
C23	Malignant neoplasm of gallbladder	C00.D48	Belgium	84.86	1	yes
C23	Malignant neoplasm of gallbladder	C00.D48	Croatia	190.45	2	yes
C23	Malignant neoplasm of gallbladder	C00.D48	Czech Republic	577.63	1	yes
C23	Malignant neoplasm of gallbladder	C00.D48	Denmark	34.06	0	no
C23	Malignant neoplasm of gallbladder	C00.D48	Estonia	17.86	0	no

For peer review only

C23	Malignant neoplasm of gallbladder	C00.D48	Finland	92.55	2	yes
C23	Malignant neoplasm of gallbladder	C00.D48	France	487.65	2	yes
C23	Malignant neoplasm of gallbladder	C00.D48	Germany	1739.51	2	yes
C23	Malignant neoplasm of gallbladder	C00.D48	Hungary	479.73	2	yes
C23	Malignant neoplasm of gallbladder	C00.D48	Latvia	20.41	1	yes
C23	Malignant neoplasm of gallbladder	C00.D48	Lithuania	54.78	2	yes
C23	Malignant neoplasm of gallbladder	C00.D48	Netherlands	157.11	1	yes
C23	Malignant neoplasm of gallbladder	C00.D48	Norway	29.39	0	no
C23	Malignant neoplasm of gallbladder	C00.D48	Poland	1669.34	2	yes
C23	Malignant neoplasm of gallbladder	C00.D48	Romania	315.12	3	yes
C23	Malignant neoplasm of gallbladder	C00.D48	Slovenia	70.04	2	yes
C23	Malignant neoplasm of gallbladder	C00.D48	Spain	614.57	1	yes
C23	Malignant neoplasm of gallbladder	C00.D48	Sweden	202.71	1	yes
C23	Malignant neoplasm of gallbladder	C00.D48	Switzerland	91.88	1	yes
C23	Malignant neoplasm of gallbladder	C00.D48	United Kingdom	523.34	1	yes
C24	Malignant neoplasm of other and unspecified parts of biliary tract	C00.D48	Austria	186.41	0	no
C24	Malignant neoplasm of other and unspecified parts of biliary tract	C00.D48	Belgium	106.65	1	yes
C24	Malignant neoplasm of other and unspecified parts of biliary tract	C00.D48	Croatia	95.39	1	yes
C24	Malignant neoplasm of other and unspecified parts of biliary tract	C00.D48	Czech Republic	427.51	0	no
C24	Malignant neoplasm of other and unspecified parts of biliary tract	C00.D48	Denmark	104.90	0	no
C24	Malignant neoplasm of other and unspecified parts of biliary tract	C00.D48	Estonia	29.80	1	yes
C24	Malignant neoplasm of other and unspecified parts of biliary tract	C00.D48	Finland	132.69	1	yes
C24	Malignant neoplasm of other and unspecified parts of biliary tract	C00.D48	France	925.30	1	yes
C24	Malignant neoplasm of other and unspecified parts of biliary tract	C00.D48	Germany	2002.50	2	yes
C24	Malignant neoplasm of other and unspecified parts of biliary tract	C00.D48	Hungary	368.39	2	yes
C24	Malignant neoplasm of other and unspecified parts of biliary tract	C00.D48	Latvia	34.53	0	no
C24	Malignant neoplasm of other and unspecified parts of biliary tract	C00.D48	Lithuania	51.98	0	no
C24	Malignant neoplasm of other and unspecified parts of biliary tract	C00.D48	Netherlands	255.09	2	yes
C24	Malignant neoplasm of other and unspecified parts of biliary tract	C00.D48	Norway	62.42	2	yes
C24	Malignant neoplasm of other and unspecified parts of biliary tract	C00.D48	Poland	911.98	1	yes
C24	Malignant neoplasm of other and unspecified parts of biliary tract	C00.D48	Romania	305.84	2	yes
C24	Malignant neoplasm of other and unspecified parts of biliary tract	C00.D48	Slovenia	66.74	0	no
C24	Malignant neoplasm of other and unspecified parts of biliary tract	C00.D48	Spain	835.07	3	yes
C24	Malignant neoplasm of other and unspecified parts of biliary tract	C00.D48	Sweden	325.28	1	yes
C24	Malignant neoplasm of other and unspecified parts of biliary tract	C00.D48	Switzerland	118.69	0	no
C24	Malignant neoplasm of other and unspecified parts of biliary tract	C00.D48	United Kingdom	283.90	2	yes

For peer review only

C25	Malignant neoplasm of pancreas	C00.D48	Austria	1598.11	0	no
C25	Malignant neoplasm of pancreas	C00.D48	Belgium	1606.64	1	yes
C25	Malignant neoplasm of pancreas	C00.D48	Croatia	730.98	1	yes
C25	Malignant neoplasm of pancreas	C00.D48	Czech Republic	2212.87	0	no
C25	Malignant neoplasm of pancreas	C00.D48	Denmark	990.02	2	yes
C25	Malignant neoplasm of pancreas	C00.D48	Estonia	243.19	1	yes
C25	Malignant neoplasm of pancreas	C00.D48	Finland	1050.69	0	no
C25	Malignant neoplasm of pancreas	C00.D48	France	9530.08	1	yes
C25	Malignant neoplasm of pancreas	C00.D48	Germany	14771.19	1	yes
C25	Malignant neoplasm of pancreas	C00.D48	Hungary	2051.51	1	yes
C25	Malignant neoplasm of pancreas	C00.D48	Latvia	395.34	1	yes
C25	Malignant neoplasm of pancreas	C00.D48	Lithuania	513.50	0	no
C25	Malignant neoplasm of pancreas	C00.D48	Netherlands	2768.64	2	yes
C25	Malignant neoplasm of pancreas	C00.D48	Norway	793.44	0	no
C25	Malignant neoplasm of pancreas	C00.D48	Poland	5692.00	0	no
C25	Malignant neoplasm of pancreas	C00.D48	Romania	3110.30	2	yes
C25	Malignant neoplasm of pancreas	C00.D48	Slovenia	363.35	3	yes
C25	Malignant neoplasm of pancreas	C00.D48	Spain	5893.56	1	yes
C25	Malignant neoplasm of pancreas	C00.D48	Sweden	1643.02	1	yes
C25	Malignant neoplasm of pancreas	C00.D48	Switzerland	1169.38	1	yes
C25	Malignant neoplasm of pancreas	C00.D48	United Kingdom	8902.67	1	yes
C26	Malignant neoplasm of other and ill-defined digestive organs	C00.D48	Austria	62.47	1	yes
C26	Malignant neoplasm of other and ill-defined digestive organs	C00.D48	Belgium	450.82	1	yes
C26	Malignant neoplasm of other and ill-defined digestive organs	C00.D48	Croatia	42.32	0	no
C26	Malignant neoplasm of other and ill-defined digestive organs	C00.D48	Czech Republic	251.87	1	yes
C26	Malignant neoplasm of other and ill-defined digestive organs	C00.D48	Denmark	55.63	0	no
C26	Malignant neoplasm of other and ill-defined digestive organs	C00.D48	Estonia	16.87	2	yes
C26	Malignant neoplasm of other and ill-defined digestive organs	C00.D48	Finland	114.67	0	no
C26	Malignant neoplasm of other and ill-defined digestive organs	C00.D48	France	1914.40	1	yes
C26	Malignant neoplasm of other and ill-defined digestive organs	C00.D48	Germany	1202.50	1	yes
C26	Malignant neoplasm of other and ill-defined digestive organs	C00.D48	Hungary	109.11	2	yes
C26	Malignant neoplasm of other and ill-defined digestive organs	C00.D48	Latvia	18.82	1	yes
C26	Malignant neoplasm of other and ill-defined digestive organs	C00.D48	Lithuania	18.99	3	yes
C26	Malignant neoplasm of other and ill-defined digestive organs	C00.D48	Netherlands	1154.93	3	yes
C26	Malignant neoplasm of other and ill-defined digestive organs	C00.D48	Norway	71.38	1	yes
C26	Malignant neoplasm of other and ill-defined digestive organs	C00.D48	Poland	967.63	0	no
C26	Malignant neoplasm of other and ill-defined digestive organs	C00.D48	Romania	95.86	3	yes
C26	Malignant neoplasm of other and ill-defined digestive organs	C00.D48	Slovenia	27.50	2	yes

For peer review only

C26	Malignant neoplasm of other and ill-defined digestive organs	C00.D48	Spain	834.34	3	yes
C26	Malignant neoplasm of other and ill-defined digestive organs	C00.D48	Sweden	173.19	0	no
C26	Malignant neoplasm of other and ill-defined digestive organs	C00.D48	Switzerland	132.65	1	yes
C26	Malignant neoplasm of other and ill-defined digestive organs	C00.D48	United Kingdom	3207.88	2	yes
C30	Malignant neoplasm of nasal cavity and middle ear	C00.D48	Croatia	6.07	0	no
C30	Malignant neoplasm of nasal cavity and middle ear	C00.D48	Czech Republic	16.92	0	no
C30	Malignant neoplasm of nasal cavity and middle ear	C00.D48	Denmark	13.41	0	no
C30	Malignant neoplasm of nasal cavity and middle ear	C00.D48	Finland	8.81	0	no
C30	Malignant neoplasm of nasal cavity and middle ear	C00.D48	France	39.66	0	no
C30	Malignant neoplasm of nasal cavity and middle ear	C00.D48	Germany	32.76	1	yes
C30	Malignant neoplasm of nasal cavity and middle ear	C00.D48	Hungary	12.28	1	yes
C30	Malignant neoplasm of nasal cavity and middle ear	C00.D48	Netherlands	9.26	0	no
C30	Malignant neoplasm of nasal cavity and middle ear	C00.D48	Poland	58.23	2	yes
C30	Malignant neoplasm of nasal cavity and middle ear	C00.D48	Romania	60.71	1	yes
C30	Malignant neoplasm of nasal cavity and middle ear	C00.D48	Spain	31.56	0	no
C30	Malignant neoplasm of nasal cavity and middle ear	C00.D48	Sweden	5.14	0	no
C30	Malignant neoplasm of nasal cavity and middle ear	C00.D48	Switzerland	8.16	1	yes
C30	Malignant neoplasm of nasal cavity and middle ear	C00.D48	United Kingdom	37.21	1	yes
C31	Malignant neoplasm of accessory sinuses	C00.D48	Austria	18.52	0	no
C31	Malignant neoplasm of accessory sinuses	C00.D48	Belgium	26.35	0	no
C31	Malignant neoplasm of accessory sinuses	C00.D48	Croatia	12.34	0	no
C31	Malignant neoplasm of accessory sinuses	C00.D48	Czech Republic	27.32	0	no
C31	Malignant neoplasm of accessory sinuses	C00.D48	Denmark	9.97	0	no
C31	Malignant neoplasm of accessory sinuses	C00.D48	Estonia	6.38	0	no
C31	Malignant neoplasm of accessory sinuses	C00.D48	Finland	11.64	0	no
C31	Malignant neoplasm of accessory sinuses	C00.D48	France	162.75	1	yes
C31	Malignant neoplasm of accessory sinuses	C00.D48	Germany	144.54	0	no
C31	Malignant neoplasm of accessory sinuses	C00.D48	Hungary	29.21	0	no
C31	Malignant neoplasm of accessory sinuses	C00.D48	Latvia	7.52	0	no
C31	Malignant neoplasm of accessory sinuses	C00.D48	Lithuania	19.03	2	yes
C31	Malignant neoplasm of accessory sinuses	C00.D48	Netherlands	27.78	2	yes
C31	Malignant neoplasm of accessory sinuses	C00.D48	Norway	9.38	0	no
C31	Malignant neoplasm of accessory sinuses	C00.D48	Poland	98.28	2	yes
C31	Malignant neoplasm of accessory sinuses	C00.D48	Romania	71.89	2	yes
C31	Malignant neoplasm of accessory sinuses	C00.D48	Slovenia	7.07	0	no
C31	Malignant neoplasm of accessory sinuses	C00.D48	Spain	74.30	1	yes
C31	Malignant neoplasm of accessory sinuses	C00.D48	Sweden	15.03	0	no
C31	Malignant neoplasm of accessory sinuses	C00.D48	Switzerland	13.62	0	no
C31	Malignant neoplasm of accessory sinuses	C00.D48	United Kingdom	107.18	2	yes
C32	Malignant neoplasm of larynx	C00.D48	Austria	152.61	2	yes
C32	Malignant neoplasm of larynx	C00.D48	Belgium	247.59	2	yes
C32	Malignant neoplasm of larynx	C00.D48	Croatia	227.88	1	yes
C32	Malignant neoplasm of larynx	C00.D48	Czech Republic	287.98	1	yes
C32	Malignant neoplasm of larynx	C00.D48	Denmark	113.47	1	yes
C32	Malignant neoplasm of larynx	C00.D48	Estonia	41.42	1	yes
C32	Malignant neoplasm of larynx	C00.D48	Finland	41.83	0	no
C32	Malignant neoplasm of larynx	C00.D48	France	1565.17	2	yes

For peer review only

For peer review only

C32	Malignant neoplasm of larynx	C00.D48	Germany	1495.38	1	yes
C32	Malignant neoplasm of larynx	C00.D48	Hungary	586.14	1	yes
C32	Malignant neoplasm of larynx	C00.D48	Latvia	92.67	0	no
C32	Malignant neoplasm of larynx	C00.D48	Lithuania	143.30	0	no
C32	Malignant neoplasm of larynx	C00.D48	Netherlands	260.40	2	yes
C32	Malignant neoplasm of larynx	C00.D48	Norway	48.05	1	yes
C32	Malignant neoplasm of larynx	C00.D48	Poland	1937.08	1	yes
C32	Malignant neoplasm of larynx	C00.D48	Romania	1118.26	3	yes
C32	Malignant neoplasm of larynx	C00.D48	Slovenia	63.08	1	yes
C32	Malignant neoplasm of larynx	C00.D48	Spain	1765.89	3	yes
C32	Malignant neoplasm of larynx	C00.D48	Sweden	58.28	0	no
C32	Malignant neoplasm of larynx	C00.D48	Switzerland	103.44	2	yes
C32	Malignant neoplasm of larynx	C00.D48	United Kingdom	935.09	2	yes
C33	Malignant neoplasm of trachea	C00.D48	Belgium	8.51	1	yes
C33	Malignant neoplasm of trachea	C00.D48	Croatia	13.96	0	no
C33	Malignant neoplasm of trachea	C00.D48	Czech Republic	14.81	0	no
C33	Malignant neoplasm of trachea	C00.D48	France	62.98	1	yes
C33	Malignant neoplasm of trachea	C00.D48	Germany	35.01	0	no
C33	Malignant neoplasm of trachea	C00.D48	Hungary	28.67	0	no
C33	Malignant neoplasm of trachea	C00.D48	Netherlands	7.86	0	no
C33	Malignant neoplasm of trachea	C00.D48	Poland	51.14	1	yes
C33	Malignant neoplasm of trachea	C00.D48	Romania	14.38	0	no
C33	Malignant neoplasm of trachea	C00.D48	Spain	25.54	0	no
C33	Malignant neoplasm of trachea	C00.D48	United Kingdom	29.53	1	yes
C34	Malignant neoplasm of bronchus and lung	C00.D48	Austria	3922.22	2	yes
C34	Malignant neoplasm of bronchus and lung	C00.D48	Belgium	7008.28	1	yes
C34	Malignant neoplasm of bronchus and lung	C00.D48	Croatia	2920.29	0	no
C34	Malignant neoplasm of bronchus and lung	C00.D48	Czech Republic	6359.29	1	yes
C34	Malignant neoplasm of bronchus and lung	C00.D48	Denmark	4156.65	2	yes
C34	Malignant neoplasm of bronchus and lung	C00.D48	Estonia	735.25	1	yes
C34	Malignant neoplasm of bronchus and lung	C00.D48	Finland	2253.31	1	yes
C34	Malignant neoplasm of bronchus and lung	C00.D48	France	31489.35	2	yes
C34	Malignant neoplasm of bronchus and lung	C00.D48	Germany	41859.76	0	no
C34	Malignant neoplasm of bronchus and lung	C00.D48	Hungary	8998.97	0	no
C34	Malignant neoplasm of bronchus and lung	C00.D48	Latvia	1113.97	2	yes
C34	Malignant neoplasm of bronchus and lung	C00.D48	Lithuania	1571.89	1	yes
C34	Malignant neoplasm of bronchus and lung	C00.D48	Netherlands	11699.38	2	yes
C34	Malignant neoplasm of bronchus and lung	C00.D48	Norway	2501.41	2	yes
C34	Malignant neoplasm of bronchus and lung	C00.D48	Poland	27661.54	1	yes
C34	Malignant neoplasm of bronchus and lung	C00.D48	Romania	10946.56	1	yes
C34	Malignant neoplasm of bronchus and lung	C00.D48	Slovenia	1196.23	3	yes
C34	Malignant neoplasm of bronchus and lung	C00.D48	Spain	22762.25	3	yes
C34	Malignant neoplasm of bronchus and lung	C00.D48	Sweden	3644.45	2	yes
C34	Malignant neoplasm of bronchus and lung	C00.D48	Switzerland	3370.25	1	yes
C34	Malignant neoplasm of bronchus and lung	C00.D48	United Kingdom	39651.31	2	yes
C37	Malignant neoplasm of thymus	C00.D48	Austria	12.19	2	yes
C37	Malignant neoplasm of thymus	C00.D48	Belgium	10.59	0	no
C37	Malignant neoplasm of thymus	C00.D48	Czech Republic	11.42	1	yes
C37	Malignant neoplasm of thymus	C00.D48	France	56.81	0	no
C37	Malignant neoplasm of thymus	C00.D48	Germany	72.24	1	yes

C37	Malignant neoplasm of thymus	C00.D48	Hungary	8.60	1	yes
C37	Malignant neoplasm of thymus	C00.D48	Netherlands	17.64	0	no
C37	Malignant neoplasm of thymus	C00.D48	Poland	16.00	0	no
C37	Malignant neoplasm of thymus	C00.D48	Spain	39.06	1	yes
C37	Malignant neoplasm of thymus	C00.D48	Sweden	7.81	0	no
C37	Malignant neoplasm of thymus	C00.D48	Switzerland	8.41	0	no
C37	Malignant neoplasm of thymus	C00.D48	United Kingdom	45.19	1	yes
C38	Malignant neoplasm of heart, mediastinum and pleura	C00.D48	Austria	18.32	3	yes
C38	Malignant neoplasm of heart, mediastinum and pleura	C00.D48	Belgium	60.23	1	yes
C38	Malignant neoplasm of heart, mediastinum and pleura	C00.D48	Croatia	26.35	1	yes
C38	Malignant neoplasm of heart, mediastinum and pleura	C00.D48	Czech Republic	90.03	1	yes
C38	Malignant neoplasm of heart, mediastinum and pleura	C00.D48	Denmark	31.42	0	no
C38	Malignant neoplasm of heart, mediastinum and pleura	C00.D48	Estonia	7.14	0	no
C38	Malignant neoplasm of heart, mediastinum and pleura	C00.D48	Finland	28.43	1	yes
C38	Malignant neoplasm of heart, mediastinum and pleura	C00.D48	France	501.41	1	yes
C38	Malignant neoplasm of heart, mediastinum and pleura	C00.D48	Germany	225.22	2	yes
C38	Malignant neoplasm of heart, mediastinum and pleura	C00.D48	Hungary	80.11	2	yes
C38	Malignant neoplasm of heart, mediastinum and pleura	C00.D48	Latvia	11.94	0	no
C38	Malignant neoplasm of heart, mediastinum and pleura	C00.D48	Lithuania	19.42	0	no
C38	Malignant neoplasm of heart, mediastinum and pleura	C00.D48	Netherlands	36.72	1	yes
C38	Malignant neoplasm of heart, mediastinum and pleura	C00.D48	Norway	14.73	2	yes
C38	Malignant neoplasm of heart, mediastinum and pleura	C00.D48	Poland	425.78	1	yes
C38	Malignant neoplasm of heart, mediastinum and pleura	C00.D48	Romania	289.76	0	no
C38	Malignant neoplasm of heart, mediastinum and pleura	C00.D48	Slovenia	16.90	1	yes
C38	Malignant neoplasm of heart, mediastinum and pleura	C00.D48	Spain	165.56	1	yes
C38	Malignant neoplasm of heart, mediastinum and pleura	C00.D48	Sweden	33.40	2	yes
C38	Malignant neoplasm of heart, mediastinum and pleura	C00.D48	Switzerland	20.58	1	yes
C38	Malignant neoplasm of heart, mediastinum and pleura	C00.D48	United Kingdom	120.09	1	yes
C39	Malignant neoplasm of other and ill-defined sites in the respiratory system and intrathoracic organs	C00.D48	Belgium	13.66	2	yes
C39	Malignant neoplasm of other and ill-defined sites in the respiratory system and intrathoracic organs	C00.D48	Czech Republic	48.60	0	no
C39	Malignant neoplasm of other and ill-defined sites in the respiratory system and intrathoracic organs	C00.D48	Denmark	18.68	1	yes
C39	Malignant neoplasm of other and ill-defined sites in the respiratory system and intrathoracic organs	C00.D48	Finland	5.04	0	no
C39	Malignant neoplasm of other and ill-defined sites in the respiratory system and intrathoracic organs	C00.D48	France	938.81	2	yes

For peer review only

C39	Malignant neoplasm of other and ill-defined sites in the respiratory system and intrathoracic organs	C00.D48	Germany	24.14	1	yes
C39	Malignant neoplasm of other and ill-defined sites in the respiratory system and intrathoracic organs	C00.D48	Hungary	14.41	1	yes
C39	Malignant neoplasm of other and ill-defined sites in the respiratory system and intrathoracic organs	C00.D48	Poland	75.35	1	yes
C39	Malignant neoplasm of other and ill-defined sites in the respiratory system and intrathoracic organs	C00.D48	Romania	12.86	2	yes
C39	Malignant neoplasm of other and ill-defined sites in the respiratory system and intrathoracic organs	C00.D48	Spain	62.05	1	yes
C39	Malignant neoplasm of other and ill-defined sites in the respiratory system and intrathoracic organs	C00.D48	Sweden	7.08	1	yes
C39	Malignant neoplasm of other and ill-defined sites in the respiratory system and intrathoracic organs	C00.D48	United Kingdom	23.66	0	no
C40	Malignant neoplasm of bone and articular cartilage of limbs	C00.D48	Austria	6.69	0	no
C40	Malignant neoplasm of bone and articular cartilage of limbs	C00.D48	Belgium	9.62	0	no
C40	Malignant neoplasm of bone and articular cartilage of limbs	C00.D48	Croatia	28.36	0	no
C40	Malignant neoplasm of bone and articular cartilage of limbs	C00.D48	Czech Republic	32.02	1	yes
C40	Malignant neoplasm of bone and articular cartilage of limbs	C00.D48	Denmark	10.87	2	yes
C40	Malignant neoplasm of bone and articular cartilage of limbs	C00.D48	Estonia	5.78	0	no
C40	Malignant neoplasm of bone and articular cartilage of limbs	C00.D48	Finland	7.99	0	no
C40	Malignant neoplasm of bone and articular cartilage of limbs	C00.D48	France	62.95	1	yes
C40	Malignant neoplasm of bone and articular cartilage of limbs	C00.D48	Germany	53.13	0	no
C40	Malignant neoplasm of bone and articular cartilage of limbs	C00.D48	Hungary	13.09	2	yes
C40	Malignant neoplasm of bone and articular cartilage of limbs	C00.D48	Latvia	7.76	0	no
C40	Malignant neoplasm of bone and articular cartilage of limbs	C00.D48	Lithuania	11.95	1	yes
C40	Malignant neoplasm of bone and articular cartilage of limbs	C00.D48	Netherlands	7.48	1	yes
C40	Malignant neoplasm of bone and articular cartilage of limbs	C00.D48	Poland	139.48	2	yes
C40	Malignant neoplasm of bone and articular cartilage of limbs	C00.D48	Romania	122.38	1	yes
C40	Malignant neoplasm of bone and articular cartilage of limbs	C00.D48	Slovenia	5.22	0	no
C40	Malignant neoplasm of bone and articular cartilage of limbs	C00.D48	Spain	29.24	0	no
C40	Malignant neoplasm of bone and articular cartilage of limbs	C00.D48	Switzerland	6.21	0	no
C40	Malignant neoplasm of bone and articular cartilage of limbs	C00.D48	United Kingdom	37.37	1	yes
C41	Malignant neoplasm of bone and articular cartilage of other and unspecified sites	C00.D48	Austria	35.90	0	no
C41	Malignant neoplasm of bone and articular cartilage of other and unspecified sites	C00.D48	Belgium	81.49	0	no
C41	Malignant neoplasm of bone and articular cartilage of other and unspecified sites	C00.D48	Croatia	51.52	0	no
C41	Malignant neoplasm of bone and articular cartilage of other and unspecified sites	C00.D48	Czech Republic	68.07	1	yes

For peer review only

C41	Malignant neoplasm of bone and articular cartilage of other and unspecified sites	C00.D48	Denmark	19.75	0	no
C41	Malignant neoplasm of bone and articular cartilage of other and unspecified sites	C00.D48	Estonia	6.76	0	no
C41	Malignant neoplasm of bone and articular cartilage of other and unspecified sites	C00.D48	Finland	13.43	1	yes
C41	Malignant neoplasm of bone and articular cartilage of other and unspecified sites	C00.D48	France	530.20	1	yes
C41	Malignant neoplasm of bone and articular cartilage of other and unspecified sites	C00.D48	Germany	377.75	0	no
C41	Malignant neoplasm of bone and articular cartilage of other and unspecified sites	C00.D48	Hungary	58.02	2	yes
C41	Malignant neoplasm of bone and articular cartilage of other and unspecified sites	C00.D48	Latvia	21.58	1	yes
C41	Malignant neoplasm of bone and articular cartilage of other and unspecified sites	C00.D48	Lithuania	18.49	0	no
C41	Malignant neoplasm of bone and articular cartilage of other and unspecified sites	C00.D48	Netherlands	103.80	0	no
C41	Malignant neoplasm of bone and articular cartilage of other and unspecified sites	C00.D48	Norway	21.07	0	no
C41	Malignant neoplasm of bone and articular cartilage of other and unspecified sites	C00.D48	Poland	320.78	2	yes
C41	Malignant neoplasm of bone and articular cartilage of other and unspecified sites	C00.D48	Romania	441.29	1	yes
C41	Malignant neoplasm of bone and articular cartilage of other and unspecified sites	C00.D48	Slovenia	10.49	0	no
C41	Malignant neoplasm of bone and articular cartilage of other and unspecified sites	C00.D48	Spain	282.85	0	no
C41	Malignant neoplasm of bone and articular cartilage of other and unspecified sites	C00.D48	Sweden	39.14	1	yes
C41	Malignant neoplasm of bone and articular cartilage of other and unspecified sites	C00.D48	Switzerland	34.36	0	no
C41	Malignant neoplasm of bone and articular cartilage of other and unspecified sites	C00.D48	United Kingdom	283.26	0	no
C43	Malignant melanoma of skin	C00.D48	Austria	351.56	0	no
C43	Malignant melanoma of skin	C00.D48	Belgium	321.60	1	yes
C43	Malignant melanoma of skin	C00.D48	Croatia	208.57	0	no
C43	Malignant melanoma of skin	C00.D48	Czech Republic	423.28	1	yes
C43	Malignant melanoma of skin	C00.D48	Denmark	280.41	1	yes
C43	Malignant melanoma of skin	C00.D48	Estonia	57.67	2	yes
C43	Malignant melanoma of skin	C00.D48	Finland	206.27	1	yes
C43	Malignant melanoma of skin	C00.D48	France	1725.80	1	yes
C43	Malignant melanoma of skin	C00.D48	Germany	2579.33	3	yes
C43	Malignant melanoma of skin	C00.D48	Hungary	388.21	0	no
C43	Malignant melanoma of skin	C00.D48	Latvia	80.38	0	no
C43	Malignant melanoma of skin	C00.D48	Lithuania	108.89	2	yes
C43	Malignant melanoma of skin	C00.D48	Netherlands	787.26	3	yes
C43	Malignant melanoma of skin	C00.D48	Norway	332.58	2	yes
C43	Malignant melanoma of skin	C00.D48	Poland	1419.95	1	yes
C43	Malignant melanoma of skin	C00.D48	Romania	446.41	0	no
C43	Malignant melanoma of skin	C00.D48	Slovenia	118.51	2	yes
C43	Malignant melanoma of skin	C00.D48	Spain	968.92	1	yes
C43	Malignant melanoma of skin	C00.D48	Sweden	480.46	2	yes
C43	Malignant melanoma of skin	C00.D48	Switzerland	314.72	1	yes
C43	Malignant melanoma of skin	C00.D48	United Kingdom	2260.40	1	yes
C44	Other malignant neoplasms of skin	C00.D48	Austria	92.05	1	yes

For peer review only

C44	Other malignant neoplasms of skin	C00.D48	Belgium	108.14	2	yes
C44	Other malignant neoplasms of skin	C00.D48	Croatia	100.82	2	yes
C44	Other malignant neoplasms of skin	C00.D48	Czech Republic	192.77	3	yes
C44	Other malignant neoplasms of skin	C00.D48	Denmark	67.60	0	no
C44	Other malignant neoplasms of skin	C00.D48	Estonia	25.52	0	no
C44	Other malignant neoplasms of skin	C00.D48	Finland	50.35	0	no
C44	Other malignant neoplasms of skin	C00.D48	France	661.52	3	yes
C44	Other malignant neoplasms of skin	C00.D48	Germany	627.79	1	yes
C44	Other malignant neoplasms of skin	C00.D48	Hungary	232.15	0	no
C44	Other malignant neoplasms of skin	C00.D48	Latvia	56.94	0	no
C44	Other malignant neoplasms of skin	C00.D48	Lithuania	54.12	0	no
C44	Other malignant neoplasms of skin	C00.D48	Netherlands	113.53	0	no
C44	Other malignant neoplasms of skin	C00.D48	Norway	47.04	0	no
C44	Other malignant neoplasms of skin	C00.D48	Poland	784.68	3	yes
C44	Other malignant neoplasms of skin	C00.D48	Romania	611.52	1	yes
C44	Other malignant neoplasms of skin	C00.D48	Slovenia	35.58	2	yes
C44	Other malignant neoplasms of skin	C00.D48	Spain	596.62	3	yes
C44	Other malignant neoplasms of skin	C00.D48	Sweden	64.10	0	no
C44	Other malignant neoplasms of skin	C00.D48	Switzerland	90.72	1	yes
C44	Other malignant neoplasms of skin	C00.D48	United Kingdom	661.79	3	yes
C45	Mesothelioma	C00.D48	Austria	98.00	2	yes
C45	Mesothelioma	C00.D48	Belgium	225.75	1	yes
C45	Mesothelioma	C00.D48	Croatia	63.39	1	yes
C45	Mesothelioma	C00.D48	Czech Republic	53.75	0	no
C45	Mesothelioma	C00.D48	Denmark	97.43	1	yes
C45	Mesothelioma	C00.D48	Finland	90.62	0	no
C45	Mesothelioma	C00.D48	France	1016.65	1	yes
C45	Mesothelioma	C00.D48	Germany	1250.24	3	yes
C45	Mesothelioma	C00.D48	Hungary	39.64	0	no
C45	Mesothelioma	C00.D48	Latvia	9.49	0	no
C45	Mesothelioma	C00.D48	Lithuania	11.52	1	yes
C45	Mesothelioma	C00.D48	Netherlands	549.80	0	no
C45	Mesothelioma	C00.D48	Norway	75.01	2	yes
C45	Mesothelioma	C00.D48	Poland	200.56	2	yes
C45	Mesothelioma	C00.D48	Romania	70.17	0	no
C45	Mesothelioma	C00.D48	Slovenia	31.74	0	no
C45	Mesothelioma	C00.D48	Spain	386.71	3	yes
C45	Mesothelioma	C00.D48	Sweden	135.07	0	no
C45	Mesothelioma	C00.D48	Switzerland	168.30	1	yes
C45	Mesothelioma	C00.D48	United Kingdom	2464.46	3	yes
C46	Kaposi's sarcoma	C00.D48	Czech Republic	6.86	0	no
C46	Kaposi's sarcoma	C00.D48	France	14.70	0	no
C46	Kaposi's sarcoma	C00.D48	Germany	14.28	1	yes
C46	Kaposi's sarcoma	C00.D48	Poland	20.40	0	no
C46	Kaposi's sarcoma	C00.D48	Romania	29.17	0	no
C46	Kaposi's sarcoma	C00.D48	Spain	24.35	1	yes
C46	Kaposi's sarcoma	C00.D48	United Kingdom	8.07	0	no
C47	Malignant neoplasm of peripheral nerves and autonomic nervous system	C00.D48	Austria	6.26	2	yes
C47	Malignant neoplasm of peripheral nerves and autonomic nervous system	C00.D48	Czech Republic	8.82	0	no

For peer review only

C47	Malignant neoplasm of peripheral nerves and autonomic nervous system	C00.D48	France	23.54	0	no
C47	Malignant neoplasm of peripheral nerves and autonomic nervous system	C00.D48	Germany	48.92	1	yes
C47	Malignant neoplasm of peripheral nerves and autonomic nervous system	C00.D48	Hungary	7.99	2	yes
C47	Malignant neoplasm of peripheral nerves and autonomic nervous system	C00.D48	Netherlands	9.51	1	yes
C47	Malignant neoplasm of peripheral nerves and autonomic nervous system	C00.D48	Poland	17.23	0	no
C47	Malignant neoplasm of peripheral nerves and autonomic nervous system	C00.D48	Romania	8.22	0	no
C47	Malignant neoplasm of peripheral nerves and autonomic nervous system	C00.D48	Spain	18.80	0	no
C47	Malignant neoplasm of peripheral nerves and autonomic nervous system	C00.D48	Sweden	5.66	1	yes
C47	Malignant neoplasm of peripheral nerves and autonomic nervous system	C00.D48	Switzerland	11.17	2	yes
C47	Malignant neoplasm of peripheral nerves and autonomic nervous system	C00.D48	United Kingdom	30.47	1	yes
C48	Malignant neoplasm of retroperitoneum and peritoneum	C00.D48	Austria	49.04	0	no
C48	Malignant neoplasm of retroperitoneum and peritoneum	C00.D48	Belgium	28.33	0	no
C48	Malignant neoplasm of retroperitoneum and peritoneum	C00.D48	Croatia	22.13	1	yes
C48	Malignant neoplasm of retroperitoneum and peritoneum	C00.D48	Czech Republic	92.98	1	yes
C48	Malignant neoplasm of retroperitoneum and peritoneum	C00.D48	Denmark	17.32	0	no
C48	Malignant neoplasm of retroperitoneum and peritoneum	C00.D48	Estonia	8.21	0	no
C48	Malignant neoplasm of retroperitoneum and peritoneum	C00.D48	Finland	36.41	1	yes
C48	Malignant neoplasm of retroperitoneum and peritoneum	C00.D48	France	229.29	1	yes
C48	Malignant neoplasm of retroperitoneum and peritoneum	C00.D48	Germany	322.91	1	yes
C48	Malignant neoplasm of retroperitoneum and peritoneum	C00.D48	Hungary	81.28	1	yes
C48	Malignant neoplasm of retroperitoneum and peritoneum	C00.D48	Latvia	17.03	0	no
C48	Malignant neoplasm of retroperitoneum and peritoneum	C00.D48	Lithuania	31.36	0	no
C48	Malignant neoplasm of retroperitoneum and peritoneum	C00.D48	Netherlands	16.71	1	yes
C48	Malignant neoplasm of retroperitoneum and peritoneum	C00.D48	Norway	26.18	1	yes
C48	Malignant neoplasm of retroperitoneum and peritoneum	C00.D48	Poland	241.45	0	no
C48	Malignant neoplasm of retroperitoneum and peritoneum	C00.D48	Romania	488.63	0	no
C48	Malignant neoplasm of retroperitoneum and peritoneum	C00.D48	Slovenia	19.63	0	no
C48	Malignant neoplasm of retroperitoneum and peritoneum	C00.D48	Spain	233.13	2	yes
C48	Malignant neoplasm of retroperitoneum and peritoneum	C00.D48	Sweden	35.46	1	yes
C48	Malignant neoplasm of retroperitoneum and peritoneum	C00.D48	Switzerland	40.39	3	yes

For peer review only

C48	Malignant neoplasm of retroperitoneum and peritoneum	C00.D48	United Kingdom	366.17	1	yes
C49	Malignant neoplasm of other connective and soft tissue	C00.D48	Austria	122.28	0	no
C49	Malignant neoplasm of other connective and soft tissue	C00.D48	Belgium	152.98	0	no
C49	Malignant neoplasm of other connective and soft tissue	C00.D48	Croatia	37.96	1	yes
C49	Malignant neoplasm of other connective and soft tissue	C00.D48	Czech Republic	113.01	0	no
C49	Malignant neoplasm of other connective and soft tissue	C00.D48	Denmark	57.98	0	no
C49	Malignant neoplasm of other connective and soft tissue	C00.D48	Estonia	13.60	0	no
C49	Malignant neoplasm of other connective and soft tissue	C00.D48	Finland	80.10	0	no
C49	Malignant neoplasm of other connective and soft tissue	C00.D48	France	823.84	1	yes
C49	Malignant neoplasm of other connective and soft tissue	C00.D48	Germany	1083.73	3	yes
C49	Malignant neoplasm of other connective and soft tissue	C00.D48	Hungary	123.70	0	no
C49	Malignant neoplasm of other connective and soft tissue	C00.D48	Latvia	27.02	0	no
C49	Malignant neoplasm of other connective and soft tissue	C00.D48	Lithuania	38.80	0	no
C49	Malignant neoplasm of other connective and soft tissue	C00.D48	Netherlands	238.39	1	yes
C49	Malignant neoplasm of other connective and soft tissue	C00.D48	Norway	55.68	1	yes
C49	Malignant neoplasm of other connective and soft tissue	C00.D48	Poland	369.40	1	yes
C49	Malignant neoplasm of other connective and soft tissue	C00.D48	Romania	239.38	1	yes
C49	Malignant neoplasm of other connective and soft tissue	C00.D48	Slovenia	22.12	0	no
C49	Malignant neoplasm of other connective and soft tissue	C00.D48	Spain	523.62	1	yes
C49	Malignant neoplasm of other connective and soft tissue	C00.D48	Sweden	146.52	0	no
C49	Malignant neoplasm of other connective and soft tissue	C00.D48	Switzerland	102.90	1	yes
C49	Malignant neoplasm of other connective and soft tissue	C00.D48	United Kingdom	816.99	1	yes
C50	Malignant neoplasm of breast	C00.D48	Austria	1718.60	2	yes
C50	Malignant neoplasm of breast	C00.D48	Belgium	2570.34	3	yes
C50	Malignant neoplasm of breast	C00.D48	Croatia	1088.80	1	yes
C50	Malignant neoplasm of breast	C00.D48	Czech Republic	2234.32	1	yes
C50	Malignant neoplasm of breast	C00.D48	Denmark	1407.10	1	yes
C50	Malignant neoplasm of breast	C00.D48	Estonia	270.57	1	yes
C50	Malignant neoplasm of breast	C00.D48	Finland	920.10	1	yes
C50	Malignant neoplasm of breast	C00.D48	France	12812.81	3	yes
C50	Malignant neoplasm of breast	C00.D48	Germany	17880.02	2	yes
C50	Malignant neoplasm of breast	C00.D48	Hungary	2511.58	2	yes
C50	Malignant neoplasm of breast	C00.D48	Latvia	475.91	0	no
C50	Malignant neoplasm of breast	C00.D48	Lithuania	648.39	0	no
C50	Malignant neoplasm of breast	C00.D48	Netherlands	3915.76	2	yes
C50	Malignant neoplasm of breast	C00.D48	Norway	783.47	1	yes

For peer review only

C50	Malignant neoplasm of breast	C00.D48	Poland	6860.49	3	yes
C50	Malignant neoplasm of breast	C00.D48	Romania	3929.44	0	no
C50	Malignant neoplasm of breast	C00.D48	Slovenia	482.50	1	yes
C50	Malignant neoplasm of breast	C00.D48	Spain	6843.63	3	yes
C50	Malignant neoplasm of breast	C00.D48	Sweden	1527.74	1	yes
C50	Malignant neoplasm of breast	C00.D48	Switzerland	1523.91	1	yes
C50	Malignant neoplasm of breast	C00.D48	United Kingdom	13672.05	2	yes
C51	Malignant neoplasm of vulva	C00.D48	Austria	70.32	0	no
C51	Malignant neoplasm of vulva	C00.D48	Belgium	58.16	0	no
C51	Malignant neoplasm of vulva	C00.D48	Croatia	48.00	0	no
C51	Malignant neoplasm of vulva	C00.D48	Czech Republic	115.56	1	yes
C51	Malignant neoplasm of vulva	C00.D48	Denmark	38.86	1	yes
C51	Malignant neoplasm of vulva	C00.D48	Estonia	14.77	0	no
C51	Malignant neoplasm of vulva	C00.D48	Finland	37.25	0	no
C51	Malignant neoplasm of vulva	C00.D48	France	252.44	0	no
C51	Malignant neoplasm of vulva	C00.D48	Germany	735.78	1	yes
C51	Malignant neoplasm of vulva	C00.D48	Hungary	113.33	0	no
C51	Malignant neoplasm of vulva	C00.D48	Latvia	26.70	0	no
C51	Malignant neoplasm of vulva	C00.D48	Lithuania	31.94	0	no
C51	Malignant neoplasm of vulva	C00.D48	Netherlands	126.22	0	no
C51	Malignant neoplasm of vulva	C00.D48	Norway	30.05	0	no
C51	Malignant neoplasm of vulva	C00.D48	Poland	345.09	0	no
C51	Malignant neoplasm of vulva	C00.D48	Romania	171.86	0	no
C51	Malignant neoplasm of vulva	C00.D48	Slovenia	23.56	0	no
C51	Malignant neoplasm of vulva	C00.D48	Spain	323.32	1	yes
C51	Malignant neoplasm of vulva	C00.D48	Sweden	59.05	0	no
C51	Malignant neoplasm of vulva	C00.D48	Switzerland	35.80	0	no
C51	Malignant neoplasm of vulva	C00.D48	United Kingdom	448.06	0	no
C52	Malignant neoplasm of vagina	C00.D48	Austria	24.65	1	yes
C52	Malignant neoplasm of vagina	C00.D48	Belgium	17.31	0	no
C52	Malignant neoplasm of vagina	C00.D48	Croatia	9.97	0	no
C52	Malignant neoplasm of vagina	C00.D48	Czech Republic	26.36	1	yes
C52	Malignant neoplasm of vagina	C00.D48	Denmark	10.92	0	no
C52	Malignant neoplasm of vagina	C00.D48	Estonia	5.72	1	yes
C52	Malignant neoplasm of vagina	C00.D48	Finland	13.12	0	no
C52	Malignant neoplasm of vagina	C00.D48	France	84.74	0	no
C52	Malignant neoplasm of vagina	C00.D48	Germany	169.62	1	yes
C52	Malignant neoplasm of vagina	C00.D48	Hungary	35.50	0	no
C52	Malignant neoplasm of vagina	C00.D48	Latvia	7.40	0	no
C52	Malignant neoplasm of vagina	C00.D48	Lithuania	8.15	0	no
C52	Malignant neoplasm of vagina	C00.D48	Netherlands	27.70	0	no
C52	Malignant neoplasm of vagina	C00.D48	Norway	5.67	1	yes
C52	Malignant neoplasm of vagina	C00.D48	Poland	85.81	1	yes
C52	Malignant neoplasm of vagina	C00.D48	Romania	40.90	2	yes
C52	Malignant neoplasm of vagina	C00.D48	Slovenia	5.48	0	no
C52	Malignant neoplasm of vagina	C00.D48	Spain	55.90	2	yes
C52	Malignant neoplasm of vagina	C00.D48	Sweden	15.07	1	yes
C52	Malignant neoplasm of vagina	C00.D48	Switzerland	12.99	0	no
C52	Malignant neoplasm of vagina	C00.D48	United Kingdom	109.82	0	no
C53	Malignant neoplasm of cervix uteri	C00.D48	Austria	168.19	1	yes

For peer review only

C53	Malignant neoplasm of cervix uteri	C00.D48	Belgium	183.97	0	no
C53	Malignant neoplasm of cervix uteri	C00.D48	Croatia	121.65	1	yes
C53	Malignant neoplasm of cervix uteri	C00.D48	Czech Republic	399.68	2	yes
C53	Malignant neoplasm of cervix uteri	C00.D48	Denmark	129.06	1	yes
C53	Malignant neoplasm of cervix uteri	C00.D48	Estonia	69.62	0	no
C53	Malignant neoplasm of cervix uteri	C00.D48	Finland	61.13	1	yes
C53	Malignant neoplasm of cervix uteri	C00.D48	France	796.17	1	yes
C53	Malignant neoplasm of cervix uteri	C00.D48	Germany	1608.89	2	yes
C53	Malignant neoplasm of cervix uteri	C00.D48	Hungary	470.60	2	yes
C53	Malignant neoplasm of cervix uteri	C00.D48	Latvia	123.85	1	yes
C53	Malignant neoplasm of cervix uteri	C00.D48	Lithuania	242.80	1	yes
C53	Malignant neoplasm of cervix uteri	C00.D48	Netherlands	244.67	1	yes
C53	Malignant neoplasm of cervix uteri	C00.D48	Norway	92.95	1	yes
C53	Malignant neoplasm of cervix uteri	C00.D48	Poland	2079.94	1	yes
C53	Malignant neoplasm of cervix uteri	C00.D48	Romania	2005.11	3	yes
C53	Malignant neoplasm of cervix uteri	C00.D48	Slovenia	51.62	1	yes
C53	Malignant neoplasm of cervix uteri	C00.D48	Spain	659.79	2	yes
C53	Malignant neoplasm of cervix uteri	C00.D48	Sweden	151.59	0	no
C53	Malignant neoplasm of cervix uteri	C00.D48	Switzerland	91.57	1	yes
C53	Malignant neoplasm of cervix uteri	C00.D48	United Kingdom	1085.58	2	yes
C54	Malignant neoplasm of corpus uteri	C00.D48	Austria	168.00	1	yes
C54	Malignant neoplasm of corpus uteri	C00.D48	Belgium	214.64	0	no
C54	Malignant neoplasm of corpus uteri	C00.D48	Croatia	112.98	1	yes
C54	Malignant neoplasm of corpus uteri	C00.D48	Czech Republic	399.98	1	yes
C54	Malignant neoplasm of corpus uteri	C00.D48	Denmark	128.80	1	yes
C54	Malignant neoplasm of corpus uteri	C00.D48	Estonia	41.75	1	yes
C54	Malignant neoplasm of corpus uteri	C00.D48	Finland	185.56	0	no
C54	Malignant neoplasm of corpus uteri	C00.D48	France	799.43	2	yes
C54	Malignant neoplasm of corpus uteri	C00.D48	Germany	1401.66	3	yes
C54	Malignant neoplasm of corpus uteri	C00.D48	Hungary	333.97	2	yes
C54	Malignant neoplasm of corpus uteri	C00.D48	Latvia	98.30	1	yes
C54	Malignant neoplasm of corpus uteri	C00.D48	Lithuania	151.71	0	no
C54	Malignant neoplasm of corpus uteri	C00.D48	Netherlands	418.31	0	no
C54	Malignant neoplasm of corpus uteri	C00.D48	Norway	111.81	3	yes
C54	Malignant neoplasm of corpus uteri	C00.D48	Poland	1310.21	2	yes
C54	Malignant neoplasm of corpus uteri	C00.D48	Romania	415.01	2	yes
C54	Malignant neoplasm of corpus uteri	C00.D48	Slovenia	61.83	0	no
C54	Malignant neoplasm of corpus uteri	C00.D48	Spain	1081.65	3	yes
C54	Malignant neoplasm of corpus uteri	C00.D48	Sweden	169.27	0	no
C54	Malignant neoplasm of corpus uteri	C00.D48	Switzerland	183.35	1	yes
C54	Malignant neoplasm of corpus uteri	C00.D48	United Kingdom	1496.84	2	yes
C55	Malignant neoplasm of uterus, part unspecified	C00.D48	Austria	143.35	3	yes
C55	Malignant neoplasm of uterus, part unspecified	C00.D48	Belgium	186.48	1	yes
C55	Malignant neoplasm of uterus, part unspecified	C00.D48	Croatia	140.60	1	yes
C55	Malignant neoplasm of uterus, part unspecified	C00.D48	Czech Republic	169.79	3	yes
C55	Malignant neoplasm of uterus, part unspecified	C00.D48	Denmark	69.48	1	yes
C55	Malignant neoplasm of uterus, part unspecified	C00.D48	Estonia	23.38	1	yes
C55	Malignant neoplasm of uterus, part unspecified	C00.D48	Finland	10.07	0	no
C55	Malignant neoplasm of uterus, part unspecified	C00.D48	France	1812.00	2	yes
C55	Malignant neoplasm of uterus, part unspecified	C00.D48	Germany	1162.86	2	yes

For peer review only

C55	Malignant neoplasm of uterus, part unspecified	C00.D48	Hungary	143.49	1	yes
C55	Malignant neoplasm of uterus, part unspecified	C00.D48	Latvia	61.32	3	yes
C55	Malignant neoplasm of uterus, part unspecified	C00.D48	Lithuania	18.76	0	no
C55	Malignant neoplasm of uterus, part unspecified	C00.D48	Netherlands	113.36	1	yes
C55	Malignant neoplasm of uterus, part unspecified	C00.D48	Norway	67.75	3	yes
C55	Malignant neoplasm of uterus, part unspecified	C00.D48	Poland	468.15	1	yes
C55	Malignant neoplasm of uterus, part unspecified	C00.D48	Romania	329.55	2	yes
C55	Malignant neoplasm of uterus, part unspecified	C00.D48	Slovenia	51.46	0	no
C55	Malignant neoplasm of uterus, part unspecified	C00.D48	Spain	507.04	1	yes
C55	Malignant neoplasm of uterus, part unspecified	C00.D48	Sweden	178.56	0	no
C55	Malignant neoplasm of uterus, part unspecified	C00.D48	Switzerland	55.50	1	yes
C55	Malignant neoplasm of uterus, part unspecified	C00.D48	United Kingdom	567.18	1	yes
C56	Malignant neoplasm of ovary	C00.D48	Austria	556.52	1	yes
C56	Malignant neoplasm of ovary	C00.D48	Belgium	745.47	3	yes
C56	Malignant neoplasm of ovary	C00.D48	Croatia	327.79	0	no
C56	Malignant neoplasm of ovary	C00.D48	Czech Republic	845.55	2	yes
C56	Malignant neoplasm of ovary	C00.D48	Denmark	443.72	1	yes
C56	Malignant neoplasm of ovary	C00.D48	Estonia	115.30	1	yes
C56	Malignant neoplasm of ovary	C00.D48	Finland	349.88	0	no
C56	Malignant neoplasm of ovary	C00.D48	France	3736.47	1	yes
C56	Malignant neoplasm of ovary	C00.D48	Germany	5656.66	3	yes
C56	Malignant neoplasm of ovary	C00.D48	Hungary	760.92	0	no
C56	Malignant neoplasm of ovary	C00.D48	Latvia	212.10	0	no
C56	Malignant neoplasm of ovary	C00.D48	Lithuania	302.65	0	no
C56	Malignant neoplasm of ovary	C00.D48	Netherlands	1192.67	1	yes
C56	Malignant neoplasm of ovary	C00.D48	Norway	372.02	1	yes
C56	Malignant neoplasm of ovary	C00.D48	Poland	3015.81	0	no
C56	Malignant neoplasm of ovary	C00.D48	Romania	1149.40	1	yes
C56	Malignant neoplasm of ovary	C00.D48	Slovenia	153.17	0	no
C56	Malignant neoplasm of ovary	C00.D48	Spain	2049.74	1	yes
C56	Malignant neoplasm of ovary	C00.D48	Sweden	626.73	1	yes
C56	Malignant neoplasm of ovary	C00.D48	Switzerland	482.04	1	yes
C56	Malignant neoplasm of ovary	C00.D48	United Kingdom	4873.91	1	yes
C57	Malignant neoplasm of other and unspecified female genital organs	C00.D48	Austria	33.41	1	yes
C57	Malignant neoplasm of other and unspecified female genital organs	C00.D48	Belgium	50.47	1	yes
C57	Malignant neoplasm of other and unspecified female genital organs	C00.D48	Croatia	11.14	0	no
C57	Malignant neoplasm of other and unspecified female genital organs	C00.D48	Czech Republic	131.98	0	no
C57	Malignant neoplasm of other and unspecified female genital organs	C00.D48	Denmark	28.96	0	no
C57	Malignant neoplasm of other and unspecified female genital organs	C00.D48	Estonia	5.85	0	no
C57	Malignant neoplasm of other and unspecified female genital organs	C00.D48	Finland	52.42	2	yes
C57	Malignant neoplasm of other and unspecified female genital organs	C00.D48	France	209.21	2	yes
C57	Malignant neoplasm of other and unspecified female genital organs	C00.D48	Germany	310.71	2	yes
C57	Malignant neoplasm of other and unspecified female genital organs	C00.D48	Hungary	30.33	2	yes

For peer review only

C57	Malignant neoplasm of other and unspecified female genital organs	C00.D48	Latvia	7.21	0	no
C57	Malignant neoplasm of other and unspecified female genital organs	C00.D48	Lithuania	8.98	1	yes
C57	Malignant neoplasm of other and unspecified female genital organs	C00.D48	Netherlands	41.66	1	yes
C57	Malignant neoplasm of other and unspecified female genital organs	C00.D48	Norway	28.85	0	no
C57	Malignant neoplasm of other and unspecified female genital organs	C00.D48	Poland	364.30	1	yes
C57	Malignant neoplasm of other and unspecified female genital organs	C00.D48	Romania	89.90	1	yes
C57	Malignant neoplasm of other and unspecified female genital organs	C00.D48	Slovenia	8.34	2	yes
C57	Malignant neoplasm of other and unspecified female genital organs	C00.D48	Spain	183.25	2	yes
C57	Malignant neoplasm of other and unspecified female genital organs	C00.D48	Sweden	93.23	0	no
C57	Malignant neoplasm of other and unspecified female genital organs	C00.D48	Switzerland	40.53	2	yes
C57	Malignant neoplasm of other and unspecified female genital organs	C00.D48	United Kingdom	131.36	2	yes
C60	Malignant neoplasm of penis	C00.D48	Austria	15.10	0	no
C60	Malignant neoplasm of penis	C00.D48	Belgium	14.50	0	no
C60	Malignant neoplasm of penis	C00.D48	Croatia	10.52	0	no
C60	Malignant neoplasm of penis	C00.D48	Czech Republic	28.71	0	no
C60	Malignant neoplasm of penis	C00.D48	Denmark	13.33	0	no
C60	Malignant neoplasm of penis	C00.D48	Finland	7.45	0	no
C60	Malignant neoplasm of penis	C00.D48	France	81.42	0	no
C60	Malignant neoplasm of penis	C00.D48	Germany	147.75	1	yes
C60	Malignant neoplasm of penis	C00.D48	Hungary	28.74	2	yes
C60	Malignant neoplasm of penis	C00.D48	Latvia	7.39	0	no
C60	Malignant neoplasm of penis	C00.D48	Lithuania	9.60	0	no
C60	Malignant neoplasm of penis	C00.D48	Netherlands	31.43	1	yes
C60	Malignant neoplasm of penis	C00.D48	Norway	8.14	2	yes
C60	Malignant neoplasm of penis	C00.D48	Poland	111.95	1	yes
C60	Malignant neoplasm of penis	C00.D48	Romania	61.88	0	no
C60	Malignant neoplasm of penis	C00.D48	Spain	115.00	0	no
C60	Malignant neoplasm of penis	C00.D48	Sweden	21.23	0	no
C60	Malignant neoplasm of penis	C00.D48	Switzerland	14.05	1	yes
C60	Malignant neoplasm of penis	C00.D48	United Kingdom	125.25	0	no
C61	Malignant neoplasm of prostate	C00.D48	Austria	1282.20	2	yes
C61	Malignant neoplasm of prostate	C00.D48	Belgium	1662.94	2	yes
C61	Malignant neoplasm of prostate	C00.D48	Croatia	820.40	1	yes
C61	Malignant neoplasm of prostate	C00.D48	Czech Republic	1851.42	1	yes
C61	Malignant neoplasm of prostate	C00.D48	Denmark	1387.74	1	yes
C61	Malignant neoplasm of prostate	C00.D48	Estonia	260.25	1	yes
C61	Malignant neoplasm of prostate	C00.D48	Finland	957.34	1	yes
C61	Malignant neoplasm of prostate	C00.D48	France	9967.49	1	yes
C61	Malignant neoplasm of prostate	C00.D48	Germany	12531.83	2	yes
C61	Malignant neoplasm of prostate	C00.D48	Hungary	1509.39	2	yes
C61	Malignant neoplasm of prostate	C00.D48	Latvia	408.57	3	yes
C61	Malignant neoplasm of prostate	C00.D48	Lithuania	616.74	0	no
C61	Malignant neoplasm of prostate	C00.D48	Netherlands	3183.89	1	yes

For peer review only

C61	Malignant neoplasm of prostate	C00.D48	Norway	1264.97	1	yes
C61	Malignant neoplasm of prostate	C00.D48	Poland	5643.53	0	no
C61	Malignant neoplasm of prostate	C00.D48	Romania	2566.20	2	yes
C61	Malignant neoplasm of prostate	C00.D48	Slovenia	425.14	0	no
C61	Malignant neoplasm of prostate	C00.D48	Spain	6448.51	3	yes
C61	Malignant neoplasm of prostate	C00.D48	Sweden	2504.28	1	yes
C61	Malignant neoplasm of prostate	C00.D48	Switzerland	1517.17	1	yes
C61	Malignant neoplasm of prostate	C00.D48	United Kingdom	12073.19	3	yes
C62	Malignant neoplasm of testis	C00.D48	Austria	15.73	0	no
C62	Malignant neoplasm of testis	C00.D48	Belgium	12.01	1	yes
C62	Malignant neoplasm of testis	C00.D48	Croatia	15.13	0	no
C62	Malignant neoplasm of testis	C00.D48	Czech Republic	36.01	1	yes
C62	Malignant neoplasm of testis	C00.D48	Denmark	12.67	1	yes
C62	Malignant neoplasm of testis	C00.D48	Finland	6.50	1	yes
C62	Malignant neoplasm of testis	C00.D48	France	100.24	1	yes
C62	Malignant neoplasm of testis	C00.D48	Germany	163.72	1	yes
C62	Malignant neoplasm of testis	C00.D48	Hungary	47.01	0	no
C62	Malignant neoplasm of testis	C00.D48	Latvia	8.84	0	no
C62	Malignant neoplasm of testis	C00.D48	Lithuania	7.82	1	yes
C62	Malignant neoplasm of testis	C00.D48	Netherlands	26.36	0	no
C62	Malignant neoplasm of testis	C00.D48	Norway	10.46	0	no
C62	Malignant neoplasm of testis	C00.D48	Poland	123.73	3	yes
C62	Malignant neoplasm of testis	C00.D48	Romania	75.34	1	yes
C62	Malignant neoplasm of testis	C00.D48	Slovenia	6.01	0	no
C62	Malignant neoplasm of testis	C00.D48	Spain	44.37	0	no
C62	Malignant neoplasm of testis	C00.D48	Sweden	10.14	0	no
C62	Malignant neoplasm of testis	C00.D48	Switzerland	13.01	1	yes
C62	Malignant neoplasm of testis	C00.D48	United Kingdom	72.90	1	yes
C63	Malignant neoplasm of other and unspecified male genital organs	C00.D48	Czech Republic	19.72	0	no
C63	Malignant neoplasm of other and unspecified male genital organs	C00.D48	France	18.20	0	no
C63	Malignant neoplasm of other and unspecified male genital organs	C00.D48	Germany	37.55	1	yes
C63	Malignant neoplasm of other and unspecified male genital organs	C00.D48	Netherlands	5.43	1	yes
C63	Malignant neoplasm of other and unspecified male genital organs	C00.D48	Poland	16.33	0	no
C63	Malignant neoplasm of other and unspecified male genital organs	C00.D48	Romania	13.26	0	no
C63	Malignant neoplasm of other and unspecified male genital organs	C00.D48	Spain	18.66	1	yes
C63	Malignant neoplasm of other and unspecified male genital organs	C00.D48	United Kingdom	12.74	0	no
C64	Malignant neoplasm of kidney, except renal pelvis	C00.D48	Austria	469.33	2	yes
C64	Malignant neoplasm of kidney, except renal pelvis	C00.D48	Belgium	621.60	1	yes
C64	Malignant neoplasm of kidney, except renal pelvis	C00.D48	Croatia	355.04	1	yes
C64	Malignant neoplasm of kidney, except renal pelvis	C00.D48	Czech Republic	1346.12	2	yes
C64	Malignant neoplasm of kidney, except renal pelvis	C00.D48	Denmark	346.01	1	yes
C64	Malignant neoplasm of kidney, except renal pelvis	C00.D48	Estonia	132.05	1	yes
C64	Malignant neoplasm of kidney, except renal pelvis	C00.D48	Finland	383.39	1	yes
C64	Malignant neoplasm of kidney, except renal pelvis	C00.D48	France	3581.84	2	yes
C64	Malignant neoplasm of kidney, except renal pelvis	C00.D48	Germany	5198.87	2	yes

For peer review only

C64	Malignant neoplasm of kidney, except renal pelvis	C00.D48	Hungary	825.71	2	yes
C64	Malignant neoplasm of kidney, except renal pelvis	C00.D48	Latvia	223.56	0	no
C64	Malignant neoplasm of kidney, except renal pelvis	C00.D48	Lithuania	314.57	0	no
C64	Malignant neoplasm of kidney, except renal pelvis	C00.D48	Netherlands	1112.03	1	yes
C64	Malignant neoplasm of kidney, except renal pelvis	C00.D48	Norway	300.70	0	no
C64	Malignant neoplasm of kidney, except renal pelvis	C00.D48	Poland	3214.05	1	yes
C64	Malignant neoplasm of kidney, except renal pelvis	C00.D48	Romania	817.78	1	yes
C64	Malignant neoplasm of kidney, except renal pelvis	C00.D48	Slovenia	157.74	1	yes
C64	Malignant neoplasm of kidney, except renal pelvis	C00.D48	Spain	2026.79	1	yes
C64	Malignant neoplasm of kidney, except renal pelvis	C00.D48	Sweden	603.90	3	yes
C64	Malignant neoplasm of kidney, except renal pelvis	C00.D48	Switzerland	350.08	1	yes
C64	Malignant neoplasm of kidney, except renal pelvis	C00.D48	United Kingdom	4030.98	2	yes
C65	Malignant neoplasm of renal pelvis	C00.D48	Austria	20.70	2	yes
C65	Malignant neoplasm of renal pelvis	C00.D48	Belgium	14.09	0	no
C65	Malignant neoplasm of renal pelvis	C00.D48	Czech Republic	91.38	2	yes
C65	Malignant neoplasm of renal pelvis	C00.D48	Denmark	37.42	0	no
C65	Malignant neoplasm of renal pelvis	C00.D48	Estonia	6.73	1	yes
C65	Malignant neoplasm of renal pelvis	C00.D48	Finland	18.35	0	no
C65	Malignant neoplasm of renal pelvis	C00.D48	France	18.65	0	no
C65	Malignant neoplasm of renal pelvis	C00.D48	Germany	188.08	1	yes
C65	Malignant neoplasm of renal pelvis	C00.D48	Hungary	32.55	0	no
C65	Malignant neoplasm of renal pelvis	C00.D48	Lithuania	10.28	1	yes
C65	Malignant neoplasm of renal pelvis	C00.D48	Netherlands	17.83	0	no
C65	Malignant neoplasm of renal pelvis	C00.D48	Norway	14.74	3	yes
C65	Malignant neoplasm of renal pelvis	C00.D48	Poland	58.21	2	yes
C65	Malignant neoplasm of renal pelvis	C00.D48	Romania	71.57	0	no
C65	Malignant neoplasm of renal pelvis	C00.D48	Spain	42.96	2	yes
C65	Malignant neoplasm of renal pelvis	C00.D48	Sweden	50.26	2	yes
C65	Malignant neoplasm of renal pelvis	C00.D48	Switzerland	30.44	1	yes
C65	Malignant neoplasm of renal pelvis	C00.D48	United Kingdom	39.53	0	no
C66	Malignant neoplasm of ureter	C00.D48	Austria	9.41	0	no
C66	Malignant neoplasm of ureter	C00.D48	Belgium	26.60	0	no
C66	Malignant neoplasm of ureter	C00.D48	Croatia	8.17	1	yes
C66	Malignant neoplasm of ureter	C00.D48	Czech Republic	23.00	1	yes
C66	Malignant neoplasm of ureter	C00.D48	Denmark	13.51	1	yes
C66	Malignant neoplasm of ureter	C00.D48	Finland	10.76	0	no
C66	Malignant neoplasm of ureter	C00.D48	France	79.23	0	no
C66	Malignant neoplasm of ureter	C00.D48	Germany	89.30	0	no
C66	Malignant neoplasm of ureter	C00.D48	Hungary	19.07	0	no
C66	Malignant neoplasm of ureter	C00.D48	Netherlands	23.93	0	no
C66	Malignant neoplasm of ureter	C00.D48	Norway	11.85	0	no
C66	Malignant neoplasm of ureter	C00.D48	Poland	42.84	1	yes
C66	Malignant neoplasm of ureter	C00.D48	Romania	34.40	1	yes
C66	Malignant neoplasm of ureter	C00.D48	Spain	43.31	1	yes
C66	Malignant neoplasm of ureter	C00.D48	Sweden	16.96	0	no
C66	Malignant neoplasm of ureter	C00.D48	Switzerland	19.36	1	yes
C66	Malignant neoplasm of ureter	C00.D48	United Kingdom	167.86	1	yes
C67	Malignant neoplasm of bladder	C00.D48	Austria	570.44	2	yes
C67	Malignant neoplasm of bladder	C00.D48	Belgium	942.82	1	yes
C67	Malignant neoplasm of bladder	C00.D48	Croatia	434.26	2	yes

For peer review only

C67	Malignant neoplasm of bladder	C00.D48	Czech Republic	970.23	2	yes
C67	Malignant neoplasm of bladder	C00.D48	Denmark	623.45	1	yes
C67	Malignant neoplasm of bladder	C00.D48	Estonia	112.59	0	no
C67	Malignant neoplasm of bladder	C00.D48	Finland	288.22	1	yes
C67	Malignant neoplasm of bladder	C00.D48	France	5240.88	1	yes
C67	Malignant neoplasm of bladder	C00.D48	Germany	5795.20	2	yes
C67	Malignant neoplasm of bladder	C00.D48	Hungary	1021.10	0	no
C67	Malignant neoplasm of bladder	C00.D48	Latvia	216.68	2	yes
C67	Malignant neoplasm of bladder	C00.D48	Lithuania	274.20	1	yes
C67	Malignant neoplasm of bladder	C00.D48	Netherlands	1488.07	2	yes
C67	Malignant neoplasm of bladder	C00.D48	Norway	409.85	1	yes
C67	Malignant neoplasm of bladder	C00.D48	Poland	4157.07	0	no
C67	Malignant neoplasm of bladder	C00.D48	Romania	1760.75	1	yes
C67	Malignant neoplasm of bladder	C00.D48	Slovenia	196.58	0	no
C67	Malignant neoplasm of bladder	C00.D48	Spain	5261.15	3	yes
C67	Malignant neoplasm of bladder	C00.D48	Sweden	681.06	1	yes
C67	Malignant neoplasm of bladder	C00.D48	Switzerland	555.95	0	no
C67	Malignant neoplasm of bladder	C00.D48	United Kingdom	5767.70	2	yes
C68	Malignant neoplasm of other and unspecified urinary organs	C00.D48	Austria	139.18	2	yes
C68	Malignant neoplasm of other and unspecified urinary organs	C00.D48	Belgium	107.88	2	yes
C68	Malignant neoplasm of other and unspecified urinary organs	C00.D48	Croatia	5.15	1	yes
C68	Malignant neoplasm of other and unspecified urinary organs	C00.D48	Czech Republic	39.90	1	yes
C68	Malignant neoplasm of other and unspecified urinary organs	C00.D48	Denmark	15.73	1	yes
C68	Malignant neoplasm of other and unspecified urinary organs	C00.D48	Finland	7.67	0	no
C68	Malignant neoplasm of other and unspecified urinary organs	C00.D48	France	774.53	2	yes
C68	Malignant neoplasm of other and unspecified urinary organs	C00.D48	Germany	2156.94	3	yes
C68	Malignant neoplasm of other and unspecified urinary organs	C00.D48	Hungary	16.43	2	yes
C68	Malignant neoplasm of other and unspecified urinary organs	C00.D48	Netherlands	401.59	3	yes
C68	Malignant neoplasm of other and unspecified urinary organs	C00.D48	Norway	21.19	1	yes
C68	Malignant neoplasm of other and unspecified urinary organs	C00.D48	Poland	61.37	2	yes
C68	Malignant neoplasm of other and unspecified urinary organs	C00.D48	Romania	22.21	0	no
C68	Malignant neoplasm of other and unspecified urinary organs	C00.D48	Spain	374.65	3	yes
C68	Malignant neoplasm of other and unspecified urinary organs	C00.D48	Sweden	41.12	0	no
C68	Malignant neoplasm of other and unspecified urinary organs	C00.D48	Switzerland	62.63	0	no
C68	Malignant neoplasm of other and unspecified urinary organs	C00.D48	United Kingdom	139.74	2	yes
C69	Malignant neoplasm of eye and adnexa	C00.D48	Austria	24.70	1	yes
C69	Malignant neoplasm of eye and adnexa	C00.D48	Belgium	20.33	0	no
C69	Malignant neoplasm of eye and adnexa	C00.D48	Croatia	11.06	0	no
C69	Malignant neoplasm of eye and adnexa	C00.D48	Czech Republic	28.97	1	yes

For peer review only

C69	Malignant neoplasm of eye and adnexa	C00.D48	Denmark	15.07	0	no
C69	Malignant neoplasm of eye and adnexa	C00.D48	Estonia	5.76	0	no
C69	Malignant neoplasm of eye and adnexa	C00.D48	Finland	26.59	0	no
C69	Malignant neoplasm of eye and adnexa	C00.D48	France	160.68	0	no
C69	Malignant neoplasm of eye and adnexa	C00.D48	Germany	210.44	3	yes
C69	Malignant neoplasm of eye and adnexa	C00.D48	Hungary	35.11	0	no
C69	Malignant neoplasm of eye and adnexa	C00.D48	Latvia	8.27	2	yes
C69	Malignant neoplasm of eye and adnexa	C00.D48	Lithuania	13.85	0	no
C69	Malignant neoplasm of eye and adnexa	C00.D48	Netherlands	33.46	0	no
C69	Malignant neoplasm of eye and adnexa	C00.D48	Norway	8.01	2	yes
C69	Malignant neoplasm of eye and adnexa	C00.D48	Poland	122.04	2	yes
C69	Malignant neoplasm of eye and adnexa	C00.D48	Romania	69.55	0	no
C69	Malignant neoplasm of eye and adnexa	C00.D48	Spain	73.29	0	no
C69	Malignant neoplasm of eye and adnexa	C00.D48	Sweden	18.16	0	no
C69	Malignant neoplasm of eye and adnexa	C00.D48	Switzerland	23.78	2	yes
C69	Malignant neoplasm of eye and adnexa	C00.D48	United Kingdom	106.10	0	no
C70	Malignant neoplasm of meninges	C00.D48	Austria	8.91	0	no
C70	Malignant neoplasm of meninges	C00.D48	Belgium	5.68	0	no
C70	Malignant neoplasm of meninges	C00.D48	Croatia	12.21	2	yes
C70	Malignant neoplasm of meninges	C00.D48	Czech Republic	29.40	1	yes
C70	Malignant neoplasm of meninges	C00.D48	Denmark	6.92	0	no
C70	Malignant neoplasm of meninges	C00.D48	France	26.17	1	yes
C70	Malignant neoplasm of meninges	C00.D48	Germany	110.82	3	yes
C70	Malignant neoplasm of meninges	C00.D48	Hungary	14.27	1	yes
C70	Malignant neoplasm of meninges	C00.D48	Lithuania	5.29	0	no
C70	Malignant neoplasm of meninges	C00.D48	Netherlands	6.64	0	no
C70	Malignant neoplasm of meninges	C00.D48	Norway	5.25	0	no
C70	Malignant neoplasm of meninges	C00.D48	Poland	68.09	0	no
C70	Malignant neoplasm of meninges	C00.D48	Romania	83.36	3	yes
C70	Malignant neoplasm of meninges	C00.D48	Slovenia	9.64	2	yes
C70	Malignant neoplasm of meninges	C00.D48	Spain	47.54	1	yes
C70	Malignant neoplasm of meninges	C00.D48	Sweden	6.87	1	yes
C70	Malignant neoplasm of meninges	C00.D48	Switzerland	5.41	2	yes
C70	Malignant neoplasm of meninges	C00.D48	United Kingdom	26.75	0	no
C71	Malignant neoplasm of brain	C00.D48	Austria	525.38	2	yes
C71	Malignant neoplasm of brain	C00.D48	Belgium	620.15	0	no
C71	Malignant neoplasm of brain	C00.D48	Croatia	414.13	1	yes
C71	Malignant neoplasm of brain	C00.D48	Czech Republic	776.57	1	yes
C71	Malignant neoplasm of brain	C00.D48	Denmark	428.34	0	no
C71	Malignant neoplasm of brain	C00.D48	Estonia	99.54	0	no
C71	Malignant neoplasm of brain	C00.D48	Finland	323.65	0	no
C71	Malignant neoplasm of brain	C00.D48	France	3398.97	2	yes
C71	Malignant neoplasm of brain	C00.D48	Germany	5460.44	0	no
C71	Malignant neoplasm of brain	C00.D48	Hungary	675.68	2	yes
C71	Malignant neoplasm of brain	C00.D48	Latvia	167.98	2	yes
C71	Malignant neoplasm of brain	C00.D48	Lithuania	248.65	2	yes
C71	Malignant neoplasm of brain	C00.D48	Netherlands	938.20	2	yes
C71	Malignant neoplasm of brain	C00.D48	Norway	333.24	0	no
C71	Malignant neoplasm of brain	C00.D48	Poland	3252.55	2	yes
C71	Malignant neoplasm of brain	C00.D48	Romania	1554.64	2	yes

For peer review only

C71	Malignant neoplasm of brain	C00.D48	Slovenia	140.07	0	no
C71	Malignant neoplasm of brain	C00.D48	Spain	2868.23	0	no
C71	Malignant neoplasm of brain	C00.D48	Sweden	586.26	3	yes
C71	Malignant neoplasm of brain	C00.D48	Switzerland	492.74	0	no
C71	Malignant neoplasm of brain	C00.D48	United Kingdom	4087.68	3	yes
C72	Malignant neoplasm of spinal cord, cranial nerves and other parts of central nervous system	C00.D48	Belgium	36.35	0	no
C72	Malignant neoplasm of spinal cord, cranial nerves and other parts of central nervous system	C00.D48	Croatia	8.38	0	no
C72	Malignant neoplasm of spinal cord, cranial nerves and other parts of central nervous system	C00.D48	Czech Republic	27.10	0	no
C72	Malignant neoplasm of spinal cord, cranial nerves and other parts of central nervous system	C00.D48	Denmark	8.56	1	yes
C72	Malignant neoplasm of spinal cord, cranial nerves and other parts of central nervous system	C00.D48	Finland	5.34	1	yes
C72	Malignant neoplasm of spinal cord, cranial nerves and other parts of central nervous system	C00.D48	France	74.59	1	yes
C72	Malignant neoplasm of spinal cord, cranial nerves and other parts of central nervous system	C00.D48	Germany	34.99	3	yes
C72	Malignant neoplasm of spinal cord, cranial nerves and other parts of central nervous system	C00.D48	Hungary	20.93	2	yes
C72	Malignant neoplasm of spinal cord, cranial nerves and other parts of central nervous system	C00.D48	Lithuania	6.27	0	no
C72	Malignant neoplasm of spinal cord, cranial nerves and other parts of central nervous system	C00.D48	Netherlands	36.29	3	yes
C72	Malignant neoplasm of spinal cord, cranial nerves and other parts of central nervous system	C00.D48	Poland	103.90	1	yes
C72	Malignant neoplasm of spinal cord, cranial nerves and other parts of central nervous system	C00.D48	Romania	42.36	1	yes
C72	Malignant neoplasm of spinal cord, cranial nerves and other parts of central nervous system	C00.D48	Spain	39.69	1	yes
C72	Malignant neoplasm of spinal cord, cranial nerves and other parts of central nervous system	C00.D48	Sweden	6.73	3	yes
C72	Malignant neoplasm of spinal cord, cranial nerves and other parts of central nervous system	C00.D48	United Kingdom	19.53	0	no
C73	Malignant neoplasm of thyroid gland	C00.D48	Austria	84.96	1	yes
C73	Malignant neoplasm of thyroid gland	C00.D48	Belgium	84.17	0	no
C73	Malignant neoplasm of thyroid gland	C00.D48	Croatia	41.95	0	no
C73	Malignant neoplasm of thyroid gland	C00.D48	Czech Republic	104.84	1	yes
C73	Malignant neoplasm of thyroid gland	C00.D48	Denmark	41.02	0	no
C73	Malignant neoplasm of thyroid gland	C00.D48	Estonia	18.69	0	no
C73	Malignant neoplasm of thyroid gland	C00.D48	Finland	53.88	0	no
C73	Malignant neoplasm of thyroid gland	C00.D48	France	449.10	1	yes
C73	Malignant neoplasm of thyroid gland	C00.D48	Germany	758.98	2	yes
C73	Malignant neoplasm of thyroid gland	C00.D48	Hungary	109.51	1	yes
C73	Malignant neoplasm of thyroid gland	C00.D48	Latvia	31.23	0	no
C73	Malignant neoplasm of thyroid gland	C00.D48	Lithuania	34.29	1	yes
C73	Malignant neoplasm of thyroid gland	C00.D48	Netherlands	121.77	0	no
C73	Malignant neoplasm of thyroid gland	C00.D48	Norway	42.25	0	no
C73	Malignant neoplasm of thyroid gland	C00.D48	Poland	365.73	2	yes
C73	Malignant neoplasm of thyroid gland	C00.D48	Romania	188.22	0	no
C73	Malignant neoplasm of thyroid gland	C00.D48	Slovenia	18.58	1	yes
C73	Malignant neoplasm of thyroid gland	C00.D48	Spain	328.20	0	no
C73	Malignant neoplasm of thyroid gland	C00.D48	Sweden	79.34	0	no
C73	Malignant neoplasm of thyroid gland	C00.D48	Switzerland	74.97	1	yes

For peer review only

C73	Malignant neoplasm of thyroid gland	C00.D48	United Kingdom	397.53	0	no
C74	Malignant neoplasm of adrenal gland	C00.D48	Austria	19.90	0	no
C74	Malignant neoplasm of adrenal gland	C00.D48	Belgium	22.22	1	yes
C74	Malignant neoplasm of adrenal gland	C00.D48	Croatia	16.71	0	no
C74	Malignant neoplasm of adrenal gland	C00.D48	Czech Republic	27.46	0	no
C74	Malignant neoplasm of adrenal gland	C00.D48	Denmark	13.17	2	yes
C74	Malignant neoplasm of adrenal gland	C00.D48	Finland	14.17	0	no
C74	Malignant neoplasm of adrenal gland	C00.D48	France	109.02	1	yes
C74	Malignant neoplasm of adrenal gland	C00.D48	Germany	139.88	0	no
C74	Malignant neoplasm of adrenal gland	C00.D48	Hungary	42.53	0	no
C74	Malignant neoplasm of adrenal gland	C00.D48	Latvia	9.76	0	no
C74	Malignant neoplasm of adrenal gland	C00.D48	Lithuania	11.51	0	no
C74	Malignant neoplasm of adrenal gland	C00.D48	Netherlands	39.28	0	no
C74	Malignant neoplasm of adrenal gland	C00.D48	Norway	10.98	0	no
C74	Malignant neoplasm of adrenal gland	C00.D48	Poland	121.16	1	yes
C74	Malignant neoplasm of adrenal gland	C00.D48	Romania	66.42	2	yes
C74	Malignant neoplasm of adrenal gland	C00.D48	Slovenia	7.85	0	no
C74	Malignant neoplasm of adrenal gland	C00.D48	Spain	107.99	0	no
C74	Malignant neoplasm of adrenal gland	C00.D48	Sweden	19.51	0	no
C74	Malignant neoplasm of adrenal gland	C00.D48	Switzerland	15.34	0	no
C74	Malignant neoplasm of adrenal gland	C00.D48	United Kingdom	122.87	0	no
C75	Malignant neoplasm of other endocrine glands and related structures	C00.D48	Austria	9.66	3	yes
C75	Malignant neoplasm of other endocrine glands and related structures	C00.D48	Belgium	30.11	1	yes
C75	Malignant neoplasm of other endocrine glands and related structures	C00.D48	Croatia	6.67	0	no
C75	Malignant neoplasm of other endocrine glands and related structures	C00.D48	Czech Republic	11.29	0	no
C75	Malignant neoplasm of other endocrine glands and related structures	C00.D48	France	56.26	3	yes
C75	Malignant neoplasm of other endocrine glands and related structures	C00.D48	Germany	320.15	1	yes
C75	Malignant neoplasm of other endocrine glands and related structures	C00.D48	Hungary	15.38	0	no
C75	Malignant neoplasm of other endocrine glands and related structures	C00.D48	Netherlands	61.02	2	yes
C75	Malignant neoplasm of other endocrine glands and related structures	C00.D48	Poland	40.57	0	no
C75	Malignant neoplasm of other endocrine glands and related structures	C00.D48	Romania	42.02	0	no
C75	Malignant neoplasm of other endocrine glands and related structures	C00.D48	Spain	82.73	2	yes
C75	Malignant neoplasm of other endocrine glands and related structures	C00.D48	Switzerland	31.08	1	yes
C75	Malignant neoplasm of other endocrine glands and related structures	C00.D48	United Kingdom	58.79	2	yes
C76	Malignant neoplasm of other and ill-defined sites	C00.D48	Austria	50.50	1	yes
C76	Malignant neoplasm of other and ill-defined sites	C00.D48	Belgium	302.33	0	no
C76	Malignant neoplasm of other and ill-defined sites	C00.D48	Croatia	161.86	1	yes
C76	Malignant neoplasm of other and ill-defined sites	C00.D48	Czech Republic	166.51	2	yes
C76	Malignant neoplasm of other and ill-defined sites	C00.D48	Denmark	212.68	3	yes
C76	Malignant neoplasm of other and ill-defined sites	C00.D48	Estonia	5.20	0	no
C76	Malignant neoplasm of other and ill-defined sites	C00.D48	Finland	74.86	1	yes
C76	Malignant neoplasm of other and ill-defined sites	C00.D48	France	3044.32	2	yes

For peer review only

C76	Malignant neoplasm of other and ill-defined sites	C00.D48	Germany	571.36	3	yes
C76	Malignant neoplasm of other and ill-defined sites	C00.D48	Hungary	148.95	2	yes
C76	Malignant neoplasm of other and ill-defined sites	C00.D48	Latvia	29.16	3	yes
C76	Malignant neoplasm of other and ill-defined sites	C00.D48	Lithuania	17.83	1	yes
C76	Malignant neoplasm of other and ill-defined sites	C00.D48	Netherlands	369.28	1	yes
C76	Malignant neoplasm of other and ill-defined sites	C00.D48	Norway	58.04	1	yes
C76	Malignant neoplasm of other and ill-defined sites	C00.D48	Poland	1137.81	0	no
C76	Malignant neoplasm of other and ill-defined sites	C00.D48	Romania	315.25	0	no
C76	Malignant neoplasm of other and ill-defined sites	C00.D48	Slovenia	23.14	2	yes
C76	Malignant neoplasm of other and ill-defined sites	C00.D48	Spain	663.90	1	yes
C76	Malignant neoplasm of other and ill-defined sites	C00.D48	Sweden	217.00	1	yes
C76	Malignant neoplasm of other and ill-defined sites	C00.D48	Switzerland	71.15	3	yes
C76	Malignant neoplasm of other and ill-defined sites	C00.D48	United Kingdom	632.16	1	yes
C77	Secondary and unspecified malignant neoplasm of lymph nodes	C00.D48	Croatia	8.39	0	no
C78	Secondary malignant neoplasm of respiratory and digestive organs	C00.D48	Croatia	112.11	2	yes
C78	Secondary malignant neoplasm of respiratory and digestive organs	C00.D48	Czech Republic	51.51	2	yes
C78	Secondary malignant neoplasm of respiratory and digestive organs	C00.D48	Finland	25.16	2	yes
C78	Secondary malignant neoplasm of respiratory and digestive organs	C00.D48	Latvia	14.46	2	yes
C78	Secondary malignant neoplasm of respiratory and digestive organs	C00.D48	Slovenia	58.16	0	no
C79	Secondary malignant neoplasm of other sites	C00.D48	Croatia	40.06	1	yes
C79	Secondary malignant neoplasm of other sites	C00.D48	Czech Republic	25.23	0	no
C79	Secondary malignant neoplasm of other sites	C00.D48	Latvia	8.77	1	yes
C79	Secondary malignant neoplasm of other sites	C00.D48	Slovenia	16.08	0	no
C80	Malignant neoplasm without specification of site	C00.D48	Austria	805.30	3	yes
C80	Malignant neoplasm without specification of site	C00.D48	Belgium	1193.36	2	yes
C80	Malignant neoplasm without specification of site	C00.D48	Croatia	437.90	0	no
C80	Malignant neoplasm without specification of site	C00.D48	Czech Republic	1191.43	0	no
C80	Malignant neoplasm without specification of site	C00.D48	Denmark	917.91	3	yes
C80	Malignant neoplasm without specification of site	C00.D48	Estonia	104.51	1	yes
C80	Malignant neoplasm without specification of site	C00.D48	Finland	361.44	1	yes
C80	Malignant neoplasm without specification of site	C00.D48	France	8321.35	3	yes
C80	Malignant neoplasm without specification of site	C00.D48	Germany	9806.38	0	no
C80	Malignant neoplasm without specification of site	C00.D48	Hungary	695.39	1	yes
C80	Malignant neoplasm without specification of site	C00.D48	Latvia	175.96	1	yes
C80	Malignant neoplasm without specification of site	C00.D48	Lithuania	224.07	2	yes
C80	Malignant neoplasm without specification of site	C00.D48	Netherlands	2441.59	2	yes
C80	Malignant neoplasm without specification of site	C00.D48	Norway	528.61	1	yes
C80	Malignant neoplasm without specification of site	C00.D48	Poland	7576.47	3	yes
C80	Malignant neoplasm without specification of site	C00.D48	Romania	1547.13	2	yes
C80	Malignant neoplasm without specification of site	C00.D48	Slovenia	108.97	1	yes
C80	Malignant neoplasm without specification of site	C00.D48	Spain	4848.20	1	yes
C80	Malignant neoplasm without specification of site	C00.D48	Sweden	919.76	2	yes
C80	Malignant neoplasm without specification of site	C00.D48	Switzerland	504.34	2	yes
C80	Malignant neoplasm without specification of site	C00.D48	United Kingdom	13226.62	1	yes
C81	Hodgkin's disease	C00.D48	Austria	35.30	1	yes
C81	Hodgkin's disease	C00.D48	Belgium	55.90	1	yes

For peer review only

C81	Hodgkin's disease	C00.D48	Croatia	26.93	0	no
C81	Hodgkin's disease	C00.D48	Czech Republic	76.69	2	yes
C81	Hodgkin's disease	C00.D48	Denmark	32.10	0	no
C81	Hodgkin's disease	C00.D48	Estonia	7.76	0	no
C81	Hodgkin's disease	C00.D48	Finland	23.99	2	yes
C81	Hodgkin's disease	C00.D48	France	299.70	1	yes
C81	Hodgkin's disease	C00.D48	Germany	344.19	1	yes
C81	Hodgkin's disease	C00.D48	Hungary	49.75	2	yes
C81	Hodgkin's disease	C00.D48	Latvia	14.79	0	no
C81	Hodgkin's disease	C00.D48	Lithuania	22.14	1	yes
C81	Hodgkin's disease	C00.D48	Netherlands	91.10	0	no
C81	Hodgkin's disease	C00.D48	Norway	17.80	0	no
C81	Hodgkin's disease	C00.D48	Poland	299.59	3	yes
C81	Hodgkin's disease	C00.D48	Romania	146.88	1	yes
C81	Hodgkin's disease	C00.D48	Slovenia	11.53	0	no
C81	Hodgkin's disease	C00.D48	Spain	251.90	1	yes
C81	Hodgkin's disease	C00.D48	Sweden	37.02	0	no
C81	Hodgkin's disease	C00.D48	Switzerland	34.60	0	no
C81	Hodgkin's disease	C00.D48	United Kingdom	339.75	0	no
C82	Follicular [nodular] non-Hodgkin's lymphoma	C00.D48	Austria	18.97	1	yes
C82	Follicular [nodular] non-Hodgkin's lymphoma	C00.D48	Belgium	11.58	2	yes
C82	Follicular [nodular] non-Hodgkin's lymphoma	C00.D48	Croatia	5.50	1	yes
C82	Follicular [nodular] non-Hodgkin's lymphoma	C00.D48	Czech Republic	89.80	1	yes
C82	Follicular [nodular] non-Hodgkin's lymphoma	C00.D48	Denmark	20.62	0	no
C82	Follicular [nodular] non-Hodgkin's lymphoma	C00.D48	Finland	50.14	1	yes
C82	Follicular [nodular] non-Hodgkin's lymphoma	C00.D48	France	128.05	1	yes
C82	Follicular [nodular] non-Hodgkin's lymphoma	C00.D48	Germany	130.04	2	yes
C82	Follicular [nodular] non-Hodgkin's lymphoma	C00.D48	Hungary	19.01	1	yes
C82	Follicular [nodular] non-Hodgkin's lymphoma	C00.D48	Lithuania	14.77	1	yes
C82	Follicular [nodular] non-Hodgkin's lymphoma	C00.D48	Norway	19.53	3	yes
C82	Follicular [nodular] non-Hodgkin's lymphoma	C00.D48	Poland	171.63	0	no
C82	Follicular [nodular] non-Hodgkin's lymphoma	C00.D48	Romania	40.44	0	no
C82	Follicular [nodular] non-Hodgkin's lymphoma	C00.D48	Slovenia	10.26	2	yes
C82	Follicular [nodular] non-Hodgkin's lymphoma	C00.D48	Spain	113.21	1	yes
C82	Follicular [nodular] non-Hodgkin's lymphoma	C00.D48	Sweden	29.55	0	no
C82	Follicular [nodular] non-Hodgkin's lymphoma	C00.D48	Switzerland	23.36	2	yes
C82	Follicular [nodular] non-Hodgkin's lymphoma	C00.D48	United Kingdom	186.55	1	yes
C83	Diffuse non-Hodgkin's lymphoma	C00.D48	Austria	107.00	1	yes
C83	Diffuse non-Hodgkin's lymphoma	C00.D48	Belgium	71.89	2	yes
C83	Diffuse non-Hodgkin's lymphoma	C00.D48	Croatia	25.55	3	yes
C83	Diffuse non-Hodgkin's lymphoma	C00.D48	Czech Republic	236.37	1	yes
C83	Diffuse non-Hodgkin's lymphoma	C00.D48	Denmark	91.60	0	no
C83	Diffuse non-Hodgkin's lymphoma	C00.D48	Estonia	39.44	2	yes
C83	Diffuse non-Hodgkin's lymphoma	C00.D48	Finland	359.13	2	yes
C83	Diffuse non-Hodgkin's lymphoma	C00.D48	France	768.60	1	yes
C83	Diffuse non-Hodgkin's lymphoma	C00.D48	Germany	687.71	2	yes
C83	Diffuse non-Hodgkin's lymphoma	C00.D48	Hungary	98.77	2	yes
C83	Diffuse non-Hodgkin's lymphoma	C00.D48	Latvia	22.97	2	yes
C83	Diffuse non-Hodgkin's lymphoma	C00.D48	Lithuania	88.83	1	yes
C83	Diffuse non-Hodgkin's lymphoma	C00.D48	Netherlands	64.83	3	yes

For peer review only

C83	Diffuse non-Hodgkin's lymphoma	C00.D48	Norway	60.18	3	yes
C83	Diffuse non-Hodgkin's lymphoma	C00.D48	Poland	517.07	1	yes
C83	Diffuse non-Hodgkin's lymphoma	C00.D48	Romania	157.91	1	yes
C83	Diffuse non-Hodgkin's lymphoma	C00.D48	Slovenia	29.17	3	yes
C83	Diffuse non-Hodgkin's lymphoma	C00.D48	Spain	412.80	2	yes
C83	Diffuse non-Hodgkin's lymphoma	C00.D48	Sweden	181.90	1	yes
C83	Diffuse non-Hodgkin's lymphoma	C00.D48	Switzerland	120.29	1	yes
C83	Diffuse non-Hodgkin's lymphoma	C00.D48	United Kingdom	796.15	1	yes
C84	Peripheral and cutaneous T-cell lymphomas	C00.D48	Austria	46.00	1	yes
C84	Peripheral and cutaneous T-cell lymphomas	C00.D48	Belgium	35.23	2	yes
C84	Peripheral and cutaneous T-cell lymphomas	C00.D48	Czech Republic	46.67	1	yes
C84	Peripheral and cutaneous T-cell lymphomas	C00.D48	Denmark	35.28	2	yes
C84	Peripheral and cutaneous T-cell lymphomas	C00.D48	Estonia	5.41	0	no
C84	Peripheral and cutaneous T-cell lymphomas	C00.D48	Finland	41.82	0	no
C84	Peripheral and cutaneous T-cell lymphomas	C00.D48	France	247.15	1	yes
C84	Peripheral and cutaneous T-cell lymphomas	C00.D48	Germany	365.79	2	yes
C84	Peripheral and cutaneous T-cell lymphomas	C00.D48	Hungary	29.81	1	yes
C84	Peripheral and cutaneous T-cell lymphomas	C00.D48	Latvia	6.59	3	yes
C84	Peripheral and cutaneous T-cell lymphomas	C00.D48	Lithuania	15.21	1	yes
C84	Peripheral and cutaneous T-cell lymphomas	C00.D48	Netherlands	63.29	1	yes
C84	Peripheral and cutaneous T-cell lymphomas	C00.D48	Norway	24.68	0	no
C84	Peripheral and cutaneous T-cell lymphomas	C00.D48	Poland	95.71	1	yes
C84	Peripheral and cutaneous T-cell lymphomas	C00.D48	Romania	15.87	1	yes
C84	Peripheral and cutaneous T-cell lymphomas	C00.D48	Slovenia	9.37	1	yes
C84	Peripheral and cutaneous T-cell lymphomas	C00.D48	Spain	139.75	1	yes
C84	Peripheral and cutaneous T-cell lymphomas	C00.D48	Sweden	49.49	0	no
C84	Peripheral and cutaneous T-cell lymphomas	C00.D48	Switzerland	43.91	0	no
C84	Peripheral and cutaneous T-cell lymphomas	C00.D48	United Kingdom	302.92	1	yes
C85	Other and unspecified types of non-Hodgkin's lymphoma	C00.D48	Austria	456.97	1	yes
C85	Other and unspecified types of non-Hodgkin's lymphoma	C00.D48	Belgium	620.71	1	yes
C85	Other and unspecified types of non-Hodgkin's lymphoma	C00.D48	Croatia	255.02	0	no
C85	Other and unspecified types of non-Hodgkin's lymphoma	C00.D48	Czech Republic	240.56	1	yes
C85	Other and unspecified types of non-Hodgkin's lymphoma	C00.D48	Denmark	232.07	2	yes
C85	Other and unspecified types of non-Hodgkin's lymphoma	C00.D48	Estonia	35.38	2	yes
C85	Other and unspecified types of non-Hodgkin's lymphoma	C00.D48	Finland	73.04	3	yes
C85	Other and unspecified types of non-Hodgkin's lymphoma	C00.D48	France	3549.46	2	yes
C85	Other and unspecified types of non-Hodgkin's lymphoma	C00.D48	Germany	4590.94	1	yes
C85	Other and unspecified types of non-Hodgkin's lymphoma	C00.D48	Hungary	403.28	2	yes
C85	Other and unspecified types of non-Hodgkin's lymphoma	C00.D48	Latvia	84.51	2	yes
C85	Other and unspecified types of non-Hodgkin's lymphoma	C00.D48	Lithuania	38.25	1	yes
C85	Other and unspecified types of non-Hodgkin's lymphoma	C00.D48	Netherlands	1222.77	2	yes

For peer review only

C85	Other and unspecified types of non-Hodgkin's lymphoma	C00.D48	Norway	264.34	2	yes
C85	Other and unspecified types of non-Hodgkin's lymphoma	C00.D48	Poland	1107.55	3	yes
C85	Other and unspecified types of non-Hodgkin's lymphoma	C00.D48	Romania	548.48	0	no
C85	Other and unspecified types of non-Hodgkin's lymphoma	C00.D48	Slovenia	103.69	0	no
C85	Other and unspecified types of non-Hodgkin's lymphoma	C00.D48	Spain	2073.90	3	yes
C85	Other and unspecified types of non-Hodgkin's lymphoma	C00.D48	Sweden	444.38	2	yes
C85	Other and unspecified types of non-Hodgkin's lymphoma	C00.D48	Switzerland	386.62	2	yes
C85	Other and unspecified types of non-Hodgkin's lymphoma	C00.D48	United Kingdom	3942.34	2	yes
C88	Malignant immunoproliferative diseases	C00.D48	Austria	11.25	1	yes
C88	Malignant immunoproliferative diseases	C00.D48	Belgium	37.10	0	no
C88	Malignant immunoproliferative diseases	C00.D48	Croatia	5.98	1	yes
C88	Malignant immunoproliferative diseases	C00.D48	Czech Republic	23.45	0	no
C88	Malignant immunoproliferative diseases	C00.D48	Denmark	29.46	2	yes
C88	Malignant immunoproliferative diseases	C00.D48	Finland	12.23	0	no
C88	Malignant immunoproliferative diseases	C00.D48	France	341.98	2	yes
C88	Malignant immunoproliferative diseases	C00.D48	Germany	120.66	1	yes
C88	Malignant immunoproliferative diseases	C00.D48	Hungary	11.72	0	no
C88	Malignant immunoproliferative diseases	C00.D48	Netherlands	60.51	2	yes
C88	Malignant immunoproliferative diseases	C00.D48	Norway	14.85	0	no
C88	Malignant immunoproliferative diseases	C00.D48	Poland	51.81	0	no
C88	Malignant immunoproliferative diseases	C00.D48	Romania	17.59	0	no
C88	Malignant immunoproliferative diseases	C00.D48	Spain	84.47	2	yes
C88	Malignant immunoproliferative diseases	C00.D48	Sweden	32.80	1	yes
C88	Malignant immunoproliferative diseases	C00.D48	Switzerland	29.36	0	no
C88	Malignant immunoproliferative diseases	C00.D48	United Kingdom	128.01	1	yes
C90	Multiple myeloma and malignant plasma cell neoplasms	C00.D48	Austria	362.29	0	no
C90	Multiple myeloma and malignant plasma cell neoplasms	C00.D48	Belgium	481.12	1	yes
C90	Multiple myeloma and malignant plasma cell neoplasms	C00.D48	Croatia	175.92	1	yes
C90	Multiple myeloma and malignant plasma cell neoplasms	C00.D48	Czech Republic	419.75	1	yes
C90	Multiple myeloma and malignant plasma cell neoplasms	C00.D48	Denmark	251.94	0	no
C90	Multiple myeloma and malignant plasma cell neoplasms	C00.D48	Estonia	54.23	1	yes
C90	Multiple myeloma and malignant plasma cell neoplasms	C00.D48	Finland	278.59	1	yes
C90	Multiple myeloma and malignant plasma cell neoplasms	C00.D48	France	2741.28	1	yes
C90	Multiple myeloma and malignant plasma cell neoplasms	C00.D48	Germany	3750.69	2	yes
C90	Multiple myeloma and malignant plasma cell neoplasms	C00.D48	Hungary	310.90	2	yes
C90	Multiple myeloma and malignant plasma cell neoplasms	C00.D48	Latvia	73.05	1	yes
C90	Multiple myeloma and malignant plasma cell neoplasms	C00.D48	Lithuania	115.17	0	no

For peer review only

C90	Multiple myeloma and malignant plasma cell neoplasms	C00.D48	Netherlands	817.41	2	yes
C90	Multiple myeloma and malignant plasma cell neoplasms	C00.D48	Norway	289.70	0	no
C90	Multiple myeloma and malignant plasma cell neoplasms	C00.D48	Poland	1470.37	3	yes
C90	Multiple myeloma and malignant plasma cell neoplasms	C00.D48	Romania	367.44	1	yes
C90	Multiple myeloma and malignant plasma cell neoplasms	C00.D48	Slovenia	104.31	0	no
C90	Multiple myeloma and malignant plasma cell neoplasms	C00.D48	Spain	1878.22	2	yes
C90	Multiple myeloma and malignant plasma cell neoplasms	C00.D48	Sweden	483.06	1	yes
C90	Multiple myeloma and malignant plasma cell neoplasms	C00.D48	Switzerland	356.41	3	yes
C90	Multiple myeloma and malignant plasma cell neoplasms	C00.D48	United Kingdom	3063.03	2	yes
C91	Lymphoid leukaemia	C00.D48	Austria	301.41	2	yes
C91	Lymphoid leukaemia	C00.D48	Belgium	260.04	1	yes
C91	Lymphoid leukaemia	C00.D48	Croatia	178.44	1	yes
C91	Lymphoid leukaemia	C00.D48	Czech Republic	461.64	1	yes
C91	Lymphoid leukaemia	C00.D48	Denmark	206.49	1	yes
C91	Lymphoid leukaemia	C00.D48	Estonia	64.92	0	no
C91	Lymphoid leukaemia	C00.D48	Finland	153.42	2	yes
C91	Lymphoid leukaemia	C00.D48	France	1663.61	1	yes
C91	Lymphoid leukaemia	C00.D48	Germany	2453.23	1	yes
C91	Lymphoid leukaemia	C00.D48	Hungary	445.86	2	yes
C91	Lymphoid leukaemia	C00.D48	Latvia	85.98	1	yes
C91	Lymphoid leukaemia	C00.D48	Lithuania	137.69	1	yes
C91	Lymphoid leukaemia	C00.D48	Netherlands	410.70	1	yes
C91	Lymphoid leukaemia	C00.D48	Norway	121.06	1	yes
C91	Lymphoid leukaemia	C00.D48	Poland	1573.64	2	yes
C91	Lymphoid leukaemia	C00.D48	Romania	619.66	0	no
C91	Lymphoid leukaemia	C00.D48	Slovenia	84.31	0	no
C91	Lymphoid leukaemia	C00.D48	Spain	1114.86	1	yes
C91	Lymphoid leukaemia	C00.D48	Sweden	287.12	1	yes
C91	Lymphoid leukaemia	C00.D48	Switzerland	218.47	1	yes
C91	Lymphoid leukaemia	C00.D48	United Kingdom	1627.19	2	yes
C92	Myeloid leukaemia	C00.D48	Austria	413.86	0	no
C92	Myeloid leukaemia	C00.D48	Belgium	450.07	0	no
C92	Myeloid leukaemia	C00.D48	Croatia	175.38	0	no
C92	Myeloid leukaemia	C00.D48	Czech Republic	454.20	1	yes
C92	Myeloid leukaemia	C00.D48	Denmark	260.97	0	no
C92	Myeloid leukaemia	C00.D48	Estonia	61.37	0	no
C92	Myeloid leukaemia	C00.D48	Finland	189.20	2	yes
C92	Myeloid leukaemia	C00.D48	France	2492.26	1	yes
C92	Myeloid leukaemia	C00.D48	Germany	3729.37	3	yes
C92	Myeloid leukaemia	C00.D48	Hungary	477.63	2	yes
C92	Myeloid leukaemia	C00.D48	Latvia	66.89	1	yes
C92	Myeloid leukaemia	C00.D48	Lithuania	134.91	0	no
C92	Myeloid leukaemia	C00.D48	Netherlands	606.45	0	no
C92	Myeloid leukaemia	C00.D48	Norway	192.81	0	no

For peer review only

C92	Myeloid leukaemia	C00.D48	Poland	1558.72	1	yes
C92	Myeloid leukaemia	C00.D48	Romania	554.82	1	yes
C92	Myeloid leukaemia	C00.D48	Slovenia	81.91	1	yes
C92	Myeloid leukaemia	C00.D48	Spain	1504.17	0	no
C92	Myeloid leukaemia	C00.D48	Sweden	356.99	0	no
C92	Myeloid leukaemia	C00.D48	Switzerland	310.32	1	yes
C92	Myeloid leukaemia	C00.D48	United Kingdom	3092.03	0	no
C93	Monocytic leukaemia	C00.D48	Austria	6.37	3	yes
C93	Monocytic leukaemia	C00.D48	Belgium	9.17	2	yes
C93	Monocytic leukaemia	C00.D48	Czech Republic	22.72	2	yes
C93	Monocytic leukaemia	C00.D48	Denmark	7.82	1	yes
C93	Monocytic leukaemia	C00.D48	Finland	7.34	0	no
C93	Monocytic leukaemia	C00.D48	France	133.66	2	yes
C93	Monocytic leukaemia	C00.D48	Germany	85.71	2	yes
C93	Monocytic leukaemia	C00.D48	Hungary	17.42	2	yes
C93	Monocytic leukaemia	C00.D48	Lithuania	11.97	1	yes
C93	Monocytic leukaemia	C00.D48	Netherlands	10.16	3	yes
C93	Monocytic leukaemia	C00.D48	Poland	49.63	2	yes
C93	Monocytic leukaemia	C00.D48	Romania	25.92	1	yes
C93	Monocytic leukaemia	C00.D48	Spain	54.60	2	yes
C93	Monocytic leukaemia	C00.D48	Sweden	16.37	2	yes
C93	Monocytic leukaemia	C00.D48	Switzerland	11.16	2	yes
C93	Monocytic leukaemia	C00.D48	United Kingdom	48.14	3	yes
C94	Other leukaemias of specified cell type	C00.D48	Czech Republic	36.32	2	yes
C94	Other leukaemias of specified cell type	C00.D48	Denmark	8.27	3	yes
C94	Other leukaemias of specified cell type	C00.D48	France	31.15	2	yes
C94	Other leukaemias of specified cell type	C00.D48	Germany	33.00	1	yes
C94	Other leukaemias of specified cell type	C00.D48	Hungary	8.34	1	yes
C94	Other leukaemias of specified cell type	C00.D48	Lithuania	6.88	1	yes
C94	Other leukaemias of specified cell type	C00.D48	Poland	55.60	1	yes
C94	Other leukaemias of specified cell type	C00.D48	Romania	27.34	0	no
C94	Other leukaemias of specified cell type	C00.D48	Spain	16.51	0	no
C94	Other leukaemias of specified cell type	C00.D48	United Kingdom	15.78	1	yes
C95	Leukaemia of unspecified cell type	C00.D48	Austria	74.51	1	yes
C95	Leukaemia of unspecified cell type	C00.D48	Belgium	274.53	0	no
C95	Leukaemia of unspecified cell type	C00.D48	Croatia	52.13	3	yes
C95	Leukaemia of unspecified cell type	C00.D48	Czech Republic	84.91	1	yes
C95	Leukaemia of unspecified cell type	C00.D48	Denmark	53.12	0	no
C95	Leukaemia of unspecified cell type	C00.D48	Estonia	8.39	0	no
C95	Leukaemia of unspecified cell type	C00.D48	Finland	18.01	0	no
C95	Leukaemia of unspecified cell type	C00.D48	France	1711.85	2	yes
C95	Leukaemia of unspecified cell type	C00.D48	Germany	1055.90	2	yes
C95	Leukaemia of unspecified cell type	C00.D48	Hungary	133.07	2	yes
C95	Leukaemia of unspecified cell type	C00.D48	Latvia	32.88	1	yes
C95	Leukaemia of unspecified cell type	C00.D48	Lithuania	16.46	3	yes
C95	Leukaemia of unspecified cell type	C00.D48	Netherlands	426.60	0	no
C95	Leukaemia of unspecified cell type	C00.D48	Norway	46.32	0	no
C95	Leukaemia of unspecified cell type	C00.D48	Poland	228.97	3	yes
C95	Leukaemia of unspecified cell type	C00.D48	Romania	199.77	1	yes
C95	Leukaemia of unspecified cell type	C00.D48	Slovenia	17.77	2	yes

For peer review only

C95	Leukaemia of unspecified cell type	C00.D48	Spain	856.40	3	yes
C95	Leukaemia of unspecified cell type	C00.D48	Sweden	84.87	1	yes
C95	Leukaemia of unspecified cell type	C00.D48	Switzerland	57.28	1	yes
C95	Leukaemia of unspecified cell type	C00.D48	United Kingdom	278.12	1	yes
C96	Other and unspecified malignant neoplasms of lymphoid, haematopoietic and related tissue	C00.D48	Belgium	17.24	2	yes
C96	Other and unspecified malignant neoplasms of lymphoid, haematopoietic and related tissue	C00.D48	Czech Republic	53.03	2	yes
C96	Other and unspecified malignant neoplasms of lymphoid, haematopoietic and related tissue	C00.D48	Denmark	8.38	0	no
C96	Other and unspecified malignant neoplasms of lymphoid, haematopoietic and related tissue	C00.D48	France	138.25	0	no
C96	Other and unspecified malignant neoplasms of lymphoid, haematopoietic and related tissue	C00.D48	Germany	52.68	2	yes
C96	Other and unspecified malignant neoplasms of lymphoid, haematopoietic and related tissue	C00.D48	Hungary	88.24	1	yes
C96	Other and unspecified malignant neoplasms of lymphoid, haematopoietic and related tissue	C00.D48	Netherlands	15.88	2	yes
C96	Other and unspecified malignant neoplasms of lymphoid, haematopoietic and related tissue	C00.D48	Norway	5.32	0	no
C96	Other and unspecified malignant neoplasms of lymphoid, haematopoietic and related tissue	C00.D48	Poland	137.40	1	yes
C96	Other and unspecified malignant neoplasms of lymphoid, haematopoietic and related tissue	C00.D48	Romania	40.65	1	yes
C96	Other and unspecified malignant neoplasms of lymphoid, haematopoietic and related tissue	C00.D48	Slovenia	26.32	1	yes
C96	Other and unspecified malignant neoplasms of lymphoid, haematopoietic and related tissue	C00.D48	Spain	53.94	1	yes
C96	Other and unspecified malignant neoplasms of lymphoid, haematopoietic and related tissue	C00.D48	Sweden	14.25	0	no
C96	Other and unspecified malignant neoplasms of lymphoid, haematopoietic and related tissue	C00.D48	Switzerland	5.21	0	no
C96	Other and unspecified malignant neoplasms of lymphoid, haematopoietic and related tissue	C00.D48	United Kingdom	29.71	2	yes
C97	Malignant neoplasms of independent (primary) multiple sites	C00.D48	Czech Republic	160.51	0	no
C97	Malignant neoplasms of independent (primary) multiple sites	C00.D48	Slovenia	74.71	0	no
C97	Malignant neoplasms of independent (primary) multiple sites	C00.D48	Switzerland	218.64	2	yes
D01	Carcinoma in situ of other and unspecified digestive organs	C00.D48	Germany	8.15	1	yes
D01	Carcinoma in situ of other and unspecified digestive organs	C00.D48	Romania	12.74	1	yes
D02	Carcinoma in situ of middle ear and respiratory system	C00.D48	Romania	5.47	1	yes
D04	Carcinoma in situ of skin	C00.D48	Romania	7.51	0	no
D07	Carcinoma in situ of other and unspecified genital organs	C00.D48	Germany	5.21	0	no
D07	Carcinoma in situ of other and unspecified genital organs	C00.D48	Romania	30.30	3	yes
D09	Carcinoma in situ of other and unspecified sites	C00.D48	Romania	9.89	0	no
D12	Benign neoplasm of colon, rectum, anus and anal canal	C00.D48	Denmark	7.50	0	no
D12	Benign neoplasm of colon, rectum, anus and anal canal	C00.D48	France	12.65	0	no
D12	Benign neoplasm of colon, rectum, anus and anal canal	C00.D48	Germany	14.81	0	no
D12	Benign neoplasm of colon, rectum, anus and anal canal	C00.D48	Hungary	7.02	0	no

For peer review only

D12	Benign neoplasm of colon, rectum, anus and anal canal	C00.D48	Netherlands	12.61	2	yes
D12	Benign neoplasm of colon, rectum, anus and anal canal	C00.D48	Poland	16.90	0	no
D12	Benign neoplasm of colon, rectum, anus and anal canal	C00.D48	Spain	19.42	2	yes
D12	Benign neoplasm of colon, rectum, anus and anal canal	C00.D48	United Kingdom	14.04	1	yes
D13	Benign neoplasm of other and ill-defined parts of digestive system	C00.D48	France	17.54	2	yes
D13	Benign neoplasm of other and ill-defined parts of digestive system	C00.D48	Germany	22.25	0	no
D13	Benign neoplasm of other and ill-defined parts of digestive system	C00.D48	Hungary	7.21	1	yes
D13	Benign neoplasm of other and ill-defined parts of digestive system	C00.D48	Poland	28.70	0	no
D13	Benign neoplasm of other and ill-defined parts of digestive system	C00.D48	Romania	7.23	1	yes
D13	Benign neoplasm of other and ill-defined parts of digestive system	C00.D48	Spain	16.90	0	no
D13	Benign neoplasm of other and ill-defined parts of digestive system	C00.D48	United Kingdom	22.73	1	yes
D14	Benign neoplasm of middle ear and respiratory system	C00.D48	France	5.11	0	no
D14	Benign neoplasm of middle ear and respiratory system	C00.D48	Poland	6.49	0	no
D15	Benign neoplasm of other and unspecified intrathoracic organs	C00.D48	Belgium	5.17	0	no
D15	Benign neoplasm of other and unspecified intrathoracic organs	C00.D48	France	23.78	0	no
D15	Benign neoplasm of other and unspecified intrathoracic organs	C00.D48	Germany	12.25	0	no
D15	Benign neoplasm of other and unspecified intrathoracic organs	C00.D48	Spain	15.29	0	no
D15	Benign neoplasm of other and unspecified intrathoracic organs	C00.D48	United Kingdom	20.09	0	no
D18	Haemangioma and lymphangioma, any site	C00.D48	Austria	5.66	0	no
D18	Haemangioma and lymphangioma, any site	C00.D48	Belgium	9.82	0	no
D18	Haemangioma and lymphangioma, any site	C00.D48	France	57.21	1	yes
D18	Haemangioma and lymphangioma, any site	C00.D48	Germany	28.98	0	no
D18	Haemangioma and lymphangioma, any site	C00.D48	Netherlands	7.05	0	no
D18	Haemangioma and lymphangioma, any site	C00.D48	Poland	9.88	1	yes
D18	Haemangioma and lymphangioma, any site	C00.D48	Spain	27.95	0	no
D18	Haemangioma and lymphangioma, any site	C00.D48	United Kingdom	17.64	0	no
D19	Benign neoplasm of mesothelial tissue	C00.D48	Germany	6.16	1	yes
D21	Other benign neoplasms of connective and other soft tissue	C00.D48	France	8.19	2	yes
D21	Other benign neoplasms of connective and other soft tissue	C00.D48	Germany	5.38	0	no
D21	Other benign neoplasms of connective and other soft tissue	C00.D48	Spain	5.46	0	no
D23	Other benign neoplasms of skin	C00.D48	Germany	6.19	0	no
D25	Leiomyoma of uterus	C00.D48	Germany	5.27	0	no
D25	Leiomyoma of uterus	C00.D48	United Kingdom	7.58	0	no
D27	Benign neoplasm of ovary	C00.D48	Germany	5.65	1	yes
D27	Benign neoplasm of ovary	C00.D48	United Kingdom	6.95	0	no
D29	Benign neoplasm of male genital organs	C00.D48	Germany	11.91	2	yes

For peer review only

D30	Benign neoplasm of urinary organs	C00.D48	Denmark	6.10	0	no
D30	Benign neoplasm of urinary organs	C00.D48	Poland	7.15	1	yes
D30	Benign neoplasm of urinary organs	C00.D48	Spain	6.43	3	yes
D32	Benign neoplasm of meninges	C00.D48	Austria	68.87	0	no
D32	Benign neoplasm of meninges	C00.D48	Belgium	59.57	0	no
D32	Benign neoplasm of meninges	C00.D48	Croatia	30.49	3	yes
D32	Benign neoplasm of meninges	C00.D48	Czech Republic	21.87	0	no
D32	Benign neoplasm of meninges	C00.D48	Denmark	21.10	0	no
D32	Benign neoplasm of meninges	C00.D48	Estonia	7.20	0	no
D32	Benign neoplasm of meninges	C00.D48	Finland	47.68	1	yes
D32	Benign neoplasm of meninges	C00.D48	France	412.89	1	yes
D32	Benign neoplasm of meninges	C00.D48	Germany	284.09	1	yes
D32	Benign neoplasm of meninges	C00.D48	Hungary	56.85	0	no
D32	Benign neoplasm of meninges	C00.D48	Latvia	12.32	0	no
D32	Benign neoplasm of meninges	C00.D48	Lithuania	22.98	0	no
D32	Benign neoplasm of meninges	C00.D48	Netherlands	89.43	0	no
D32	Benign neoplasm of meninges	C00.D48	Norway	21.18	1	yes
D32	Benign neoplasm of meninges	C00.D48	Poland	50.29	1	yes
D32	Benign neoplasm of meninges	C00.D48	Romania	16.36	1	yes
D32	Benign neoplasm of meninges	C00.D48	Slovenia	8.68	2	yes
D32	Benign neoplasm of meninges	C00.D48	Spain	292.81	1	yes
D32	Benign neoplasm of meninges	C00.D48	Sweden	52.72	1	yes
D32	Benign neoplasm of meninges	C00.D48	Switzerland	35.95	1	yes
D32	Benign neoplasm of meninges	C00.D48	United Kingdom	384.88	0	no
D33	Benign neoplasm of brain and other parts of central nervous system	C00.D48	Austria	5.37	0	no
D33	Benign neoplasm of brain and other parts of central nervous system	C00.D48	Belgium	10.52	1	yes
D33	Benign neoplasm of brain and other parts of central nervous system	C00.D48	Czech Republic	18.65	0	no
D33	Benign neoplasm of brain and other parts of central nervous system	C00.D48	Denmark	18.23	1	yes
D33	Benign neoplasm of brain and other parts of central nervous system	C00.D48	Finland	8.25	0	no
D33	Benign neoplasm of brain and other parts of central nervous system	C00.D48	France	52.03	1	yes
D33	Benign neoplasm of brain and other parts of central nervous system	C00.D48	Germany	23.51	0	no
D33	Benign neoplasm of brain and other parts of central nervous system	C00.D48	Hungary	19.04	2	yes
D33	Benign neoplasm of brain and other parts of central nervous system	C00.D48	Lithuania	10.92	1	yes
D33	Benign neoplasm of brain and other parts of central nervous system	C00.D48	Netherlands	6.69	0	no
D33	Benign neoplasm of brain and other parts of central nervous system	C00.D48	Norway	8.50	1	yes
D33	Benign neoplasm of brain and other parts of central nervous system	C00.D48	Poland	75.02	0	no
D33	Benign neoplasm of brain and other parts of central nervous system	C00.D48	Romania	17.55	2	yes
D33	Benign neoplasm of brain and other parts of central nervous system	C00.D48	Spain	29.14	1	yes
D33	Benign neoplasm of brain and other parts of central nervous system	C00.D48	Sweden	5.13	1	yes
D33	Benign neoplasm of brain and other parts of central nervous system	C00.D48	United Kingdom	28.51	0	no

For peer review only

D35	Benign neoplasm of other and unspecified endocrine glands	C00.D48	Austria	12.78	1	yes
D35	Benign neoplasm of other and unspecified endocrine glands	C00.D48	Belgium	6.56	0	no
D35	Benign neoplasm of other and unspecified endocrine glands	C00.D48	Czech Republic	5.62	0	no
D35	Benign neoplasm of other and unspecified endocrine glands	C00.D48	Finland	5.38	0	no
D35	Benign neoplasm of other and unspecified endocrine glands	C00.D48	France	50.69	0	no
D35	Benign neoplasm of other and unspecified endocrine glands	C00.D48	Germany	32.05	1	yes
D35	Benign neoplasm of other and unspecified endocrine glands	C00.D48	Hungary	11.53	0	no
D35	Benign neoplasm of other and unspecified endocrine glands	C00.D48	Lithuania	5.52	1	yes
D35	Benign neoplasm of other and unspecified endocrine glands	C00.D48	Netherlands	7.00	2	yes
D35	Benign neoplasm of other and unspecified endocrine glands	C00.D48	Poland	12.76	0	no
D35	Benign neoplasm of other and unspecified endocrine glands	C00.D48	Spain	42.85	0	no
D35	Benign neoplasm of other and unspecified endocrine glands	C00.D48	Switzerland	6.20	0	no
D35	Benign neoplasm of other and unspecified endocrine glands	C00.D48	United Kingdom	36.40	1	yes
D36	Benign neoplasm of other and unspecified sites	C00.D48	France	23.15	0	no
D36	Benign neoplasm of other and unspecified sites	C00.D48	Germany	14.22	3	yes
D36	Benign neoplasm of other and unspecified sites	C00.D48	Spain	9.30	0	no
D36	Benign neoplasm of other and unspecified sites	C00.D48	United Kingdom	14.72	0	no
D37	Neoplasm of uncertain or unknown behaviour of oral cavity and digestive organs	C00.D48	Austria	62.61	3	yes
D37	Neoplasm of uncertain or unknown behaviour of oral cavity and digestive organs	C00.D48	Belgium	224.18	3	yes
D37	Neoplasm of uncertain or unknown behaviour of oral cavity and digestive organs	C00.D48	Czech Republic	32.07	1	yes
D37	Neoplasm of uncertain or unknown behaviour of oral cavity and digestive organs	C00.D48	Denmark	38.92	1	yes
D37	Neoplasm of uncertain or unknown behaviour of oral cavity and digestive organs	C00.D48	Finland	55.52	0	no
D37	Neoplasm of uncertain or unknown behaviour of oral cavity and digestive organs	C00.D48	France	1426.24	3	yes
D37	Neoplasm of uncertain or unknown behaviour of oral cavity and digestive organs	C00.D48	Germany	1007.20	2	yes
D37	Neoplasm of uncertain or unknown behaviour of oral cavity and digestive organs	C00.D48	Hungary	150.63	0	no
D37	Neoplasm of uncertain or unknown behaviour of oral cavity and digestive organs	C00.D48	Lithuania	6.69	1	yes
D37	Neoplasm of uncertain or unknown behaviour of oral cavity and digestive organs	C00.D48	Netherlands	273.84	2	yes
D37	Neoplasm of uncertain or unknown behaviour of oral cavity and digestive organs	C00.D48	Norway	40.25	2	yes
D37	Neoplasm of uncertain or unknown behaviour of oral cavity and digestive organs	C00.D48	Poland	889.53	2	yes
D37	Neoplasm of uncertain or unknown behaviour of oral cavity and digestive organs	C00.D48	Romania	24.63	2	yes
D37	Neoplasm of uncertain or unknown behaviour of oral cavity and digestive organs	C00.D48	Spain	445.46	1	yes
D37	Neoplasm of uncertain or unknown behaviour of oral cavity and digestive organs	C00.D48	Sweden	181.90	0	no

For peer review only

D37	Neoplasm of uncertain or unknown behaviour of oral cavity and digestive organs	C00.D48	Switzerland	76.24	0	no
D37	Neoplasm of uncertain or unknown behaviour of oral cavity and digestive organs	C00.D48	United Kingdom	448.33	1	yes
D38	Neoplasm of uncertain or unknown behaviour of middle ear and respiratory and intrathoracic organs	C00.D48	Austria	27.70	3	yes
D38	Neoplasm of uncertain or unknown behaviour of middle ear and respiratory and intrathoracic organs	C00.D48	Belgium	109.20	3	yes
D38	Neoplasm of uncertain or unknown behaviour of middle ear and respiratory and intrathoracic organs	C00.D48	Czech Republic	25.79	1	yes
D38	Neoplasm of uncertain or unknown behaviour of middle ear and respiratory and intrathoracic organs	C00.D48	Denmark	44.94	2	yes
D38	Neoplasm of uncertain or unknown behaviour of middle ear and respiratory and intrathoracic organs	C00.D48	Finland	27.10	0	no
D38	Neoplasm of uncertain or unknown behaviour of middle ear and respiratory and intrathoracic organs	C00.D48	France	518.56	0	no
D38	Neoplasm of uncertain or unknown behaviour of middle ear and respiratory and intrathoracic organs	C00.D48	Germany	374.70	1	yes
D38	Neoplasm of uncertain or unknown behaviour of middle ear and respiratory and intrathoracic organs	C00.D48	Hungary	112.67	0	no
D38	Neoplasm of uncertain or unknown behaviour of middle ear and respiratory and intrathoracic organs	C00.D48	Netherlands	151.08	2	yes
D38	Neoplasm of uncertain or unknown behaviour of middle ear and respiratory and intrathoracic organs	C00.D48	Norway	30.50	0	no
D38	Neoplasm of uncertain or unknown behaviour of middle ear and respiratory and intrathoracic organs	C00.D48	Poland	1006.96	2	yes
D38	Neoplasm of uncertain or unknown behaviour of middle ear and respiratory and intrathoracic organs	C00.D48	Romania	16.48	0	no
D38	Neoplasm of uncertain or unknown behaviour of middle ear and respiratory and intrathoracic organs	C00.D48	Spain	184.75	1	yes
D38	Neoplasm of uncertain or unknown behaviour of middle ear and respiratory and intrathoracic organs	C00.D48	Sweden	101.38	0	no
D38	Neoplasm of uncertain or unknown behaviour of middle ear and respiratory and intrathoracic organs	C00.D48	Switzerland	35.69	0	no
D38	Neoplasm of uncertain or unknown behaviour of middle ear and respiratory and intrathoracic organs	C00.D48	United Kingdom	158.66	1	yes
D39	Neoplasm of uncertain or unknown behaviour of female genital organs	C00.D48	Austria	5.66	0	no
D39	Neoplasm of uncertain or unknown behaviour of female genital organs	C00.D48	Belgium	16.05	0	no
D39	Neoplasm of uncertain or unknown behaviour of female genital organs	C00.D48	Czech Republic	5.45	0	no
D39	Neoplasm of uncertain or unknown behaviour of female genital organs	C00.D48	Finland	8.47	0	no
D39	Neoplasm of uncertain or unknown behaviour of female genital organs	C00.D48	France	103.34	0	no
D39	Neoplasm of uncertain or unknown behaviour of female genital organs	C00.D48	Germany	89.62	1	yes
D39	Neoplasm of uncertain or unknown behaviour of female genital organs	C00.D48	Hungary	16.85	0	no
D39	Neoplasm of uncertain or unknown behaviour of female genital organs	C00.D48	Netherlands	14.75	1	yes
D39	Neoplasm of uncertain or unknown behaviour of female genital organs	C00.D48	Poland	70.20	1	yes
D39	Neoplasm of uncertain or unknown behaviour of female genital organs	C00.D48	Spain	44.67	3	yes
D39	Neoplasm of uncertain or unknown behaviour of female genital organs	C00.D48	Sweden	13.60	0	no
D39	Neoplasm of uncertain or unknown behaviour of female genital organs	C00.D48	Switzerland	9.04	0	no

For peer review only

D39	Neoplasm of uncertain or unknown behaviour of female genital organs	C00.D48	United Kingdom	40.13	1	yes
D40	Neoplasm of uncertain or unknown behaviour of male genital organs	C00.D48	Belgium	5.40	2	yes
D40	Neoplasm of uncertain or unknown behaviour of male genital organs	C00.D48	France	28.45	1	yes
D40	Neoplasm of uncertain or unknown behaviour of male genital organs	C00.D48	Germany	46.14	2	yes
D40	Neoplasm of uncertain or unknown behaviour of male genital organs	C00.D48	Hungary	21.72	0	no
D40	Neoplasm of uncertain or unknown behaviour of male genital organs	C00.D48	Poland	22.14	0	no
D40	Neoplasm of uncertain or unknown behaviour of male genital organs	C00.D48	Spain	19.11	0	no
D41	Neoplasm of uncertain or unknown behaviour of urinary organs	C00.D48	Austria	30.43	1	yes
D41	Neoplasm of uncertain or unknown behaviour of urinary organs	C00.D48	Belgium	76.18	0	no
D41	Neoplasm of uncertain or unknown behaviour of urinary organs	C00.D48	Czech Republic	6.38	1	yes
D41	Neoplasm of uncertain or unknown behaviour of urinary organs	C00.D48	Denmark	16.21	1	yes
D41	Neoplasm of uncertain or unknown behaviour of urinary organs	C00.D48	Finland	11.44	1	yes
D41	Neoplasm of uncertain or unknown behaviour of urinary organs	C00.D48	France	518.12	2	yes
D41	Neoplasm of uncertain or unknown behaviour of urinary organs	C00.D48	Germany	268.23	2	yes
D41	Neoplasm of uncertain or unknown behaviour of urinary organs	C00.D48	Hungary	30.29	0	no
D41	Neoplasm of uncertain or unknown behaviour of urinary organs	C00.D48	Netherlands	77.07	2	yes
D41	Neoplasm of uncertain or unknown behaviour of urinary organs	C00.D48	Norway	10.54	0	no
D41	Neoplasm of uncertain or unknown behaviour of urinary organs	C00.D48	Poland	149.47	2	yes
D41	Neoplasm of uncertain or unknown behaviour of urinary organs	C00.D48	Romania	12.85	1	yes
D41	Neoplasm of uncertain or unknown behaviour of urinary organs	C00.D48	Spain	314.25	0	no
D41	Neoplasm of uncertain or unknown behaviour of urinary organs	C00.D48	Sweden	32.20	0	no
D41	Neoplasm of uncertain or unknown behaviour of urinary organs	C00.D48	Switzerland	15.98	0	no
D41	Neoplasm of uncertain or unknown behaviour of urinary organs	C00.D48	United Kingdom	130.31	1	yes
D42	Neoplasm of uncertain or unknown behaviour of meninges	C00.D48	France	5.38	0	no
D42	Neoplasm of uncertain or unknown behaviour of meninges	C00.D48	Germany	25.05	1	yes
D42	Neoplasm of uncertain or unknown behaviour of meninges	C00.D48	Poland	16.59	0	no
D42	Neoplasm of uncertain or unknown behaviour of meninges	C00.D48	Spain	25.22	3	yes
D43	Neoplasm of uncertain or unknown behaviour of brain and central nervous system	C00.D48	Austria	60.23	0	no
D43	Neoplasm of uncertain or unknown behaviour of brain and central nervous system	C00.D48	Belgium	164.31	0	no
D43	Neoplasm of uncertain or unknown behaviour of brain and central nervous system	C00.D48	Czech Republic	15.83	1	yes

For peer review only

D43	Neoplasm of uncertain or unknown behaviour of brain and central nervous system	C00.D48	Denmark	44.16	3	yes
D43	Neoplasm of uncertain or unknown behaviour of brain and central nervous system	C00.D48	Estonia	7.06	1	yes
D43	Neoplasm of uncertain or unknown behaviour of brain and central nervous system	C00.D48	Finland	11.49	1	yes
D43	Neoplasm of uncertain or unknown behaviour of brain and central nervous system	C00.D48	France	1411.27	3	yes
D43	Neoplasm of uncertain or unknown behaviour of brain and central nervous system	C00.D48	Germany	609.86	2	yes
D43	Neoplasm of uncertain or unknown behaviour of brain and central nervous system	C00.D48	Hungary	50.95	0	no
D43	Neoplasm of uncertain or unknown behaviour of brain and central nervous system	C00.D48	Latvia	10.03	0	no
D43	Neoplasm of uncertain or unknown behaviour of brain and central nervous system	C00.D48	Lithuania	8.92	0	no
D43	Neoplasm of uncertain or unknown behaviour of brain and central nervous system	C00.D48	Netherlands	373.98	2	yes
D43	Neoplasm of uncertain or unknown behaviour of brain and central nervous system	C00.D48	Norway	34.27	1	yes
D43	Neoplasm of uncertain or unknown behaviour of brain and central nervous system	C00.D48	Poland	472.70	0	no
D43	Neoplasm of uncertain or unknown behaviour of brain and central nervous system	C00.D48	Romania	24.18	0	no
D43	Neoplasm of uncertain or unknown behaviour of brain and central nervous system	C00.D48	Slovenia	7.00	2	yes
D43	Neoplasm of uncertain or unknown behaviour of brain and central nervous system	C00.D48	Spain	700.21	1	yes
D43	Neoplasm of uncertain or unknown behaviour of brain and central nervous system	C00.D48	Sweden	154.89	2	yes
D43	Neoplasm of uncertain or unknown behaviour of brain and central nervous system	C00.D48	Switzerland	35.83	0	no
D43	Neoplasm of uncertain or unknown behaviour of brain and central nervous system	C00.D48	United Kingdom	759.27	1	yes
D44	Neoplasm of uncertain or unknown behaviour of endocrine glands	C00.D48	Austria	7.55	0	no
D44	Neoplasm of uncertain or unknown behaviour of endocrine glands	C00.D48	Belgium	12.18	0	no
D44	Neoplasm of uncertain or unknown behaviour of endocrine glands	C00.D48	France	68.78	1	yes
D44	Neoplasm of uncertain or unknown behaviour of endocrine glands	C00.D48	Germany	72.71	1	yes
D44	Neoplasm of uncertain or unknown behaviour of endocrine glands	C00.D48	Hungary	6.25	1	yes
D44	Neoplasm of uncertain or unknown behaviour of endocrine glands	C00.D48	Netherlands	22.13	2	yes
D44	Neoplasm of uncertain or unknown behaviour of endocrine glands	C00.D48	Norway	5.71	0	no
D44	Neoplasm of uncertain or unknown behaviour of endocrine glands	C00.D48	Poland	31.30	1	yes
D44	Neoplasm of uncertain or unknown behaviour of endocrine glands	C00.D48	Spain	44.18	0	no
D44	Neoplasm of uncertain or unknown behaviour of endocrine glands	C00.D48	Sweden	8.32	0	no
D44	Neoplasm of uncertain or unknown behaviour of endocrine glands	C00.D48	Switzerland	6.45	0	no
D44	Neoplasm of uncertain or unknown behaviour of endocrine glands	C00.D48	United Kingdom	59.63	1	yes
D45	Polycythaemia vera	C00.D48	Austria	29.84	0	no
D45	Polycythaemia vera	C00.D48	Belgium	21.86	1	yes

For peer review only

D45	Polycythaemia vera	C00.D48	Croatia	9.04	0	no
D45	Polycythaemia vera	C00.D48	Czech Republic	15.16	1	yes
D45	Polycythaemia vera	C00.D48	Denmark	18.07	1	yes
D45	Polycythaemia vera	C00.D48	Finland	16.33	0	no
D45	Polycythaemia vera	C00.D48	France	144.41	1	yes
D45	Polycythaemia vera	C00.D48	Germany	125.84	0	no
D45	Polycythaemia vera	C00.D48	Hungary	22.96	1	yes
D45	Polycythaemia vera	C00.D48	Lithuania	7.06	0	no
D45	Polycythaemia vera	C00.D48	Netherlands	23.67	0	no
D45	Polycythaemia vera	C00.D48	Norway	9.10	0	no
D45	Polycythaemia vera	C00.D48	Poland	70.30	0	no
D45	Polycythaemia vera	C00.D48	Spain	51.76	1	yes
D45	Polycythaemia vera	C00.D48	Sweden	23.14	0	no
D45	Polycythaemia vera	C00.D48	Switzerland	21.39	0	no
D45	Polycythaemia vera	C00.D48	United Kingdom	56.35	2	yes
D46	Myelodysplastic syndromes	C00.D48	Austria	219.84	2	yes
D46	Myelodysplastic syndromes	C00.D48	Belgium	221.62	1	yes
D46	Myelodysplastic syndromes	C00.D48	Croatia	72.50	3	yes
D46	Myelodysplastic syndromes	C00.D48	Czech Republic	176.62	1	yes
D46	Myelodysplastic syndromes	C00.D48	Denmark	104.39	1	yes
D46	Myelodysplastic syndromes	C00.D48	Estonia	9.55	3	yes
D46	Myelodysplastic syndromes	C00.D48	Finland	84.73	2	yes
D46	Myelodysplastic syndromes	C00.D48	France	1146.26	2	yes
D46	Myelodysplastic syndromes	C00.D48	Germany	1979.31	1	yes
D46	Myelodysplastic syndromes	C00.D48	Hungary	178.24	0	no
D46	Myelodysplastic syndromes	C00.D48	Latvia	25.57	2	yes
D46	Myelodysplastic syndromes	C00.D48	Lithuania	38.87	2	yes
D46	Myelodysplastic syndromes	C00.D48	Netherlands	327.41	2	yes
D46	Myelodysplastic syndromes	C00.D48	Norway	68.70	0	no
D46	Myelodysplastic syndromes	C00.D48	Poland	686.69	1	yes
D46	Myelodysplastic syndromes	C00.D48	Romania	38.02	1	yes
D46	Myelodysplastic syndromes	C00.D48	Slovenia	30.20	2	yes
D46	Myelodysplastic syndromes	C00.D48	Spain	937.25	2	yes
D46	Myelodysplastic syndromes	C00.D48	Sweden	140.75	0	no
D46	Myelodysplastic syndromes	C00.D48	Switzerland	173.76	2	yes
D46	Myelodysplastic syndromes	C00.D48	United Kingdom	1277.94	2	yes
D47	Other neoplasms of uncertain or unknown behaviour of lymphoid, haematopoietic and related tissue	C00.D48	Austria	48.52	1	yes
D47	Other neoplasms of uncertain or unknown behaviour of lymphoid, haematopoietic and related tissue	C00.D48	Belgium	57.23	1	yes
D47	Other neoplasms of uncertain or unknown behaviour of lymphoid, haematopoietic and related tissue	C00.D48	Croatia	64.18	1	yes
D47	Other neoplasms of uncertain or unknown behaviour of lymphoid, haematopoietic and related tissue	C00.D48	Czech Republic	44.69	2	yes
D47	Other neoplasms of uncertain or unknown behaviour of lymphoid, haematopoietic and related tissue	C00.D48	Denmark	49.68	0	no
D47	Other neoplasms of uncertain or unknown behaviour of lymphoid, haematopoietic and related tissue	C00.D48	Estonia	9.99	0	no
D47	Other neoplasms of uncertain or unknown behaviour of lymphoid, haematopoietic and related tissue	C00.D48	Finland	37.52	0	no
D47	Other neoplasms of uncertain or unknown behaviour of lymphoid, haematopoietic and related tissue	C00.D48	France	584.53	1	yes

For peer review only

D47	Other neoplasms of uncertain or unknown behaviour of lymphoid, haematopoietic and related tissue	C00.D48	Germany	328.32	2	yes
D47	Other neoplasms of uncertain or unknown behaviour of lymphoid, haematopoietic and related tissue	C00.D48	Hungary	76.19	0	no
D47	Other neoplasms of uncertain or unknown behaviour of lymphoid, haematopoietic and related tissue	C00.D48	Latvia	16.50	1	yes
D47	Other neoplasms of uncertain or unknown behaviour of lymphoid, haematopoietic and related tissue	C00.D48	Lithuania	17.09	0	no
D47	Other neoplasms of uncertain or unknown behaviour of lymphoid, haematopoietic and related tissue	C00.D48	Netherlands	70.12	2	yes
D47	Other neoplasms of uncertain or unknown behaviour of lymphoid, haematopoietic and related tissue	C00.D48	Norway	22.84	2	yes
D47	Other neoplasms of uncertain or unknown behaviour of lymphoid, haematopoietic and related tissue	C00.D48	Poland	131.24	2	yes
D47	Other neoplasms of uncertain or unknown behaviour of lymphoid, haematopoietic and related tissue	C00.D48	Romania	45.34	0	no
D47	Other neoplasms of uncertain or unknown behaviour of lymphoid, haematopoietic and related tissue	C00.D48	Slovenia	12.30	1	yes
D47	Other neoplasms of uncertain or unknown behaviour of lymphoid, haematopoietic and related tissue	C00.D48	Spain	455.59	2	yes
D47	Other neoplasms of uncertain or unknown behaviour of lymphoid, haematopoietic and related tissue	C00.D48	Sweden	59.19	0	no
D47	Other neoplasms of uncertain or unknown behaviour of lymphoid, haematopoietic and related tissue	C00.D48	Switzerland	52.47	0	no
D47	Other neoplasms of uncertain or unknown behaviour of lymphoid, haematopoietic and related tissue	C00.D48	United Kingdom	524.53	2	yes
D48	Neoplasm of uncertain or unknown behaviour of other and unspecified sites	C00.D48	Austria	33.10	1	yes
D48	Neoplasm of uncertain or unknown behaviour of other and unspecified sites	C00.D48	Belgium	92.94	3	yes
D48	Neoplasm of uncertain or unknown behaviour of other and unspecified sites	C00.D48	Czech Republic	16.74	1	yes
D48	Neoplasm of uncertain or unknown behaviour of other and unspecified sites	C00.D48	Denmark	39.78	0	no
D48	Neoplasm of uncertain or unknown behaviour of other and unspecified sites	C00.D48	Finland	12.83	0	no
D48	Neoplasm of uncertain or unknown behaviour of other and unspecified sites	C00.D48	France	495.53	1	yes
D48	Neoplasm of uncertain or unknown behaviour of other and unspecified sites	C00.D48	Germany	863.01	2	yes
D48	Neoplasm of uncertain or unknown behaviour of other and unspecified sites	C00.D48	Hungary	34.19	3	yes
D48	Neoplasm of uncertain or unknown behaviour of other and unspecified sites	C00.D48	Netherlands	260.00	0	no
D48	Neoplasm of uncertain or unknown behaviour of other and unspecified sites	C00.D48	Norway	23.59	1	yes
D48	Neoplasm of uncertain or unknown behaviour of other and unspecified sites	C00.D48	Poland	386.66	0	no
D48	Neoplasm of uncertain or unknown behaviour of other and unspecified sites	C00.D48	Romania	76.53	2	yes
D48	Neoplasm of uncertain or unknown behaviour of other and unspecified sites	C00.D48	Spain	291.16	2	yes
D48	Neoplasm of uncertain or unknown behaviour of other and unspecified sites	C00.D48	Sweden	85.14	2	yes
D48	Neoplasm of uncertain or unknown behaviour of other and unspecified sites	C00.D48	Switzerland	78.67	0	no
D48	Neoplasm of uncertain or unknown behaviour of other and unspecified sites	C00.D48	United Kingdom	161.20	1	yes
D50	Iron deficiency anaemia	D50.D89	Belgium	20.44	1	yes
D50	Iron deficiency anaemia	D50.D89	Croatia	6.11	3	yes

For peer review only

D50	Iron deficiency anaemia	D50.D89	Czech Republic	11.76	3	yes
D50	Iron deficiency anaemia	D50.D89	Denmark	23.33	0	no
D50	Iron deficiency anaemia	D50.D89	France	92.87	0	no
D50	Iron deficiency anaemia	D50.D89	Germany	117.66	2	yes
D50	Iron deficiency anaemia	D50.D89	Hungary	7.44	0	no
D50	Iron deficiency anaemia	D50.D89	Netherlands	28.79	2	yes
D50	Iron deficiency anaemia	D50.D89	Norway	11.01	1	yes
D50	Iron deficiency anaemia	D50.D89	Poland	24.31	2	yes
D50	Iron deficiency anaemia	D50.D89	Romania	10.61	0	no
D50	Iron deficiency anaemia	D50.D89	Slovenia	5.50	0	no
D50	Iron deficiency anaemia	D50.D89	Spain	146.99	0	no
D50	Iron deficiency anaemia	D50.D89	Sweden	18.39	0	no
D50	Iron deficiency anaemia	D50.D89	Switzerland	14.49	0	no
D50	Iron deficiency anaemia	D50.D89	United Kingdom	42.42	0	no
D51	Vitamin B12 deficiency anaemia	D50.D89	Denmark	13.35	0	no
D51	Vitamin B12 deficiency anaemia	D50.D89	France	27.93	1	yes
D51	Vitamin B12 deficiency anaemia	D50.D89	Germany	27.60	1	yes
D51	Vitamin B12 deficiency anaemia	D50.D89	Hungary	15.09	0	no
D51	Vitamin B12 deficiency anaemia	D50.D89	Poland	33.67	1	yes
D51	Vitamin B12 deficiency anaemia	D50.D89	Spain	14.29	0	no
D51	Vitamin B12 deficiency anaemia	D50.D89	Sweden	5.29	0	no
D51	Vitamin B12 deficiency anaemia	D50.D89	United Kingdom	17.89	1	yes
D52	Folate deficiency anaemia	D50.D89	France	17.96	1	yes
D52	Folate deficiency anaemia	D50.D89	Germany	14.46	1	yes
D52	Folate deficiency anaemia	D50.D89	Spain	19.06	2	yes
D53	Other nutritional anaemias	D50.D89	France	34.96	0	no
D53	Other nutritional anaemias	D50.D89	Germany	28.14	0	no
D53	Other nutritional anaemias	D50.D89	Hungary	5.16	1	yes
D53	Other nutritional anaemias	D50.D89	Netherlands	49.10	1	yes
D53	Other nutritional anaemias	D50.D89	Poland	12.29	2	yes
D53	Other nutritional anaemias	D50.D89	Romania	6.47	0	no
D53	Other nutritional anaemias	D50.D89	Spain	111.53	2	yes
D56	Thalassaemia	D50.D89	France	5.38	0	no
D56	Thalassaemia	D50.D89	Germany	5.05	0	no
D56	Thalassaemia	D50.D89	Spain	6.34	0	no
D57	Sickle-cell disorders	D50.D89	France	14.11	1	yes
D57	Sickle-cell disorders	D50.D89	Germany	5.29	1	yes
D57	Sickle-cell disorders	D50.D89	United Kingdom	20.61	0	no
D58	Other hereditary haemolytic anaemias	D50.D89	France	11.48	3	yes
D58	Other hereditary haemolytic anaemias	D50.D89	Germany	24.91	0	no
D58	Other hereditary haemolytic anaemias	D50.D89	Spain	20.94	0	no
D58	Other hereditary haemolytic anaemias	D50.D89	Sweden	8.53	0	no
D58	Other hereditary haemolytic anaemias	D50.D89	United Kingdom	15.51	1	yes
D59	Acquired haemolytic anaemia	D50.D89	Belgium	9.56	0	no
D59	Acquired haemolytic anaemia	D50.D89	Czech Republic	7.35	0	no
D59	Acquired haemolytic anaemia	D50.D89	Denmark	11.18	0	no
D59	Acquired haemolytic anaemia	D50.D89	Finland	7.00	1	yes
D59	Acquired haemolytic anaemia	D50.D89	France	65.09	1	yes
D59	Acquired haemolytic anaemia	D50.D89	Germany	56.76	0	no
D59	Acquired haemolytic anaemia	D50.D89	Hungary	10.14	0	no

For peer review only

D59	Acquired haemolytic anaemia	D50.D89	Netherlands	14.76	0	no
D59	Acquired haemolytic anaemia	D50.D89	Norway	5.80	0	no
D59	Acquired haemolytic anaemia	D50.D89	Poland	31.78	1	yes
D59	Acquired haemolytic anaemia	D50.D89	Romania	6.70	0	no
D59	Acquired haemolytic anaemia	D50.D89	Spain	36.32	1	yes
D59	Acquired haemolytic anaemia	D50.D89	Switzerland	8.18	0	no
D59	Acquired haemolytic anaemia	D50.D89	United Kingdom	45.53	0	no
D60	Acquired pure red cell aplasia [erythroblastopenia]	D50.D89	Germany	6.55	0	no
D60	Acquired pure red cell aplasia [erythroblastopenia]	D50.D89	Poland	6.95	1	yes
D60	Acquired pure red cell aplasia [erythroblastopenia]	D50.D89	Romania	9.54	1	yes
D61	Other aplastic anaemias	D50.D89	Austria	34.63	0	no
D61	Other aplastic anaemias	D50.D89	Belgium	50.69	1	yes
D61	Other aplastic anaemias	D50.D89	Croatia	8.25	1	yes
D61	Other aplastic anaemias	D50.D89	Czech Republic	13.04	0	no
D61	Other aplastic anaemias	D50.D89	Denmark	16.46	0	no
D61	Other aplastic anaemias	D50.D89	Finland	10.81	0	no
D61	Other aplastic anaemias	D50.D89	France	362.44	2	yes
D61	Other aplastic anaemias	D50.D89	Germany	319.98	0	no
D61	Other aplastic anaemias	D50.D89	Hungary	35.62	2	yes
D61	Other aplastic anaemias	D50.D89	Latvia	6.67	0	no
D61	Other aplastic anaemias	D50.D89	Lithuania	8.33	0	no
D61	Other aplastic anaemias	D50.D89	Netherlands	50.50	2	yes
D61	Other aplastic anaemias	D50.D89	Norway	18.04	2	yes
D61	Other aplastic anaemias	D50.D89	Poland	164.49	1	yes
D61	Other aplastic anaemias	D50.D89	Romania	26.92	0	no
D61	Other aplastic anaemias	D50.D89	Spain	245.20	3	yes
D61	Other aplastic anaemias	D50.D89	Sweden	19.32	1	yes
D61	Other aplastic anaemias	D50.D89	Switzerland	25.77	0	no
D61	Other aplastic anaemias	D50.D89	United Kingdom	162.16	3	yes
D62	Acute posthaemorrhagic anaemia	D50.D89	Germany	44.53	2	yes
D64	Other anaemias	D50.D89	Austria	23.23	1	yes
D64	Other anaemias	D50.D89	Belgium	123.37	0	no
D64	Other anaemias	D50.D89	Czech Republic	23.83	1	yes
D64	Other anaemias	D50.D89	Denmark	127.41	1	yes
D64	Other anaemias	D50.D89	Finland	6.15	1	yes
D64	Other anaemias	D50.D89	France	692.71	2	yes
D64	Other anaemias	D50.D89	Germany	577.43	2	yes
D64	Other anaemias	D50.D89	Hungary	48.39	2	yes
D64	Other anaemias	D50.D89	Netherlands	308.82	1	yes
D64	Other anaemias	D50.D89	Norway	51.73	0	no
D64	Other anaemias	D50.D89	Poland	74.16	3	yes
D64	Other anaemias	D50.D89	Romania	23.58	3	yes
D64	Other anaemias	D50.D89	Spain	449.37	3	yes
D64	Other anaemias	D50.D89	Sweden	121.45	0	no
D64	Other anaemias	D50.D89	Switzerland	48.65	0	no
D64	Other anaemias	D50.D89	United Kingdom	288.77	3	yes
D65	Disseminated intravascular coagulation [defibrination syndrome]	D50.D89	Belgium	15.86	2	yes
D65	Disseminated intravascular coagulation [defibrination syndrome]	D50.D89	Czech Republic	8.82	1	yes

For peer review only

D65	Disseminated intravascular coagulation [defibrination syndrome]	D50.D89	France	105.75	1	yes
D65	Disseminated intravascular coagulation [defibrination syndrome]	D50.D89	Germany	36.93	0	no
D65	Disseminated intravascular coagulation [defibrination syndrome]	D50.D89	Netherlands	5.19	1	yes
D65	Disseminated intravascular coagulation [defibrination syndrome]	D50.D89	Poland	36.80	1	yes
D65	Disseminated intravascular coagulation [defibrination syndrome]	D50.D89	Romania	12.51	1	yes
D65	Disseminated intravascular coagulation [defibrination syndrome]	D50.D89	Spain	37.45	2	yes
D65	Disseminated intravascular coagulation [defibrination syndrome]	D50.D89	United Kingdom	43.52	1	yes
D66	Hereditary factor VIII deficiency	D50.D89	France	10.39	0	no
D66	Hereditary factor VIII deficiency	D50.D89	Germany	12.62	0	no
D66	Hereditary factor VIII deficiency	D50.D89	Hungary	5.84	0	no
D66	Hereditary factor VIII deficiency	D50.D89	Poland	5.09	1	yes
D66	Hereditary factor VIII deficiency	D50.D89	Spain	5.35	1	yes
D66	Hereditary factor VIII deficiency	D50.D89	United Kingdom	17.46	0	no
D68	Other coagulation defects	D50.D89	Belgium	45.12	0	no
D68	Other coagulation defects	D50.D89	Czech Republic	21.62	2	yes
D68	Other coagulation defects	D50.D89	Denmark	10.87	1	yes
D68	Other coagulation defects	D50.D89	France	148.78	2	yes
D68	Other coagulation defects	D50.D89	Germany	426.54	3	yes
D68	Other coagulation defects	D50.D89	Hungary	15.09	2	yes
D68	Other coagulation defects	D50.D89	Netherlands	18.32	0	no
D68	Other coagulation defects	D50.D89	Norway	5.22	0	no
D68	Other coagulation defects	D50.D89	Poland	25.43	0	no
D68	Other coagulation defects	D50.D89	Spain	93.72	1	yes
D68	Other coagulation defects	D50.D89	Sweden	10.61	0	no
D68	Other coagulation defects	D50.D89	Switzerland	8.70	0	no
D68	Other coagulation defects	D50.D89	United Kingdom	47.53	0	no
D69	Purpura and other haemorrhagic conditions	D50.D89	Austria	21.53	0	no
D69	Purpura and other haemorrhagic conditions	D50.D89	Belgium	28.38	0	no
D69	Purpura and other haemorrhagic conditions	D50.D89	Croatia	5.66	0	no
D69	Purpura and other haemorrhagic conditions	D50.D89	Czech Republic	27.09	1	yes
D69	Purpura and other haemorrhagic conditions	D50.D89	Denmark	14.21	0	no
D69	Purpura and other haemorrhagic conditions	D50.D89	Finland	7.20	2	yes
D69	Purpura and other haemorrhagic conditions	D50.D89	France	224.15	1	yes
D69	Purpura and other haemorrhagic conditions	D50.D89	Germany	203.70	0	no
D69	Purpura and other haemorrhagic conditions	D50.D89	Hungary	26.78	0	no
D69	Purpura and other haemorrhagic conditions	D50.D89	Latvia	5.24	0	no
D69	Purpura and other haemorrhagic conditions	D50.D89	Netherlands	33.95	1	yes
D69	Purpura and other haemorrhagic conditions	D50.D89	Norway	11.27	0	no
D69	Purpura and other haemorrhagic conditions	D50.D89	Poland	74.75	1	yes
D69	Purpura and other haemorrhagic conditions	D50.D89	Romania	15.43	0	no
D69	Purpura and other haemorrhagic conditions	D50.D89	Spain	129.89	0	no
D69	Purpura and other haemorrhagic conditions	D50.D89	Sweden	12.35	2	yes
D69	Purpura and other haemorrhagic conditions	D50.D89	Switzerland	15.04	0	no
D69	Purpura and other haemorrhagic conditions	D50.D89	United Kingdom	106.80	1	yes
D70	Agranulocytosis	D50.D89	Austria	5.24	1	yes

For peer review only

D70	Agranulocytosis	D50.D89	Belgium	18.26	0	no
D70	Agranulocytosis	D50.D89	France	61.15	2	yes
D70	Agranulocytosis	D50.D89	Germany	86.34	1	yes
D70	Agranulocytosis	D50.D89	Hungary	8.87	0	no
D70	Agranulocytosis	D50.D89	Netherlands	7.88	0	no
D70	Agranulocytosis	D50.D89	Norway	6.54	2	yes
D70	Agranulocytosis	D50.D89	Poland	23.90	2	yes
D70	Agranulocytosis	D50.D89	Spain	46.86	2	yes
D70	Agranulocytosis	D50.D89	Sweden	8.07	0	no
D70	Agranulocytosis	D50.D89	United Kingdom	151.29	2	yes
D72	Other disorders of white blood cells	D50.D89	France	13.69	0	no
D72	Other disorders of white blood cells	D50.D89	Germany	13.26	0	no
D72	Other disorders of white blood cells	D50.D89	Spain	9.06	0	no
D73	Diseases of spleen	D50.D89	France	24.67	3	yes
D73	Diseases of spleen	D50.D89	Germany	31.95	0	no
D73	Diseases of spleen	D50.D89	Hungary	5.65	0	no
D73	Diseases of spleen	D50.D89	Netherlands	8.41	0	no
D73	Diseases of spleen	D50.D89	Poland	7.15	0	no
D73	Diseases of spleen	D50.D89	Romania	7.48	1	yes
D73	Diseases of spleen	D50.D89	Spain	27.45	1	yes
D73	Diseases of spleen	D50.D89	United Kingdom	37.60	2	yes
D75	Other diseases of blood and blood-forming organs	D50.D89	Austria	23.62	2	yes
D75	Other diseases of blood and blood-forming organs	D50.D89	Belgium	15.26	1	yes
D75	Other diseases of blood and blood-forming organs	D50.D89	Czech Republic	9.95	1	yes
D75	Other diseases of blood and blood-forming organs	D50.D89	France	145.80	3	yes
D75	Other diseases of blood and blood-forming organs	D50.D89	Germany	178.98	2	yes
D75	Other diseases of blood and blood-forming organs	D50.D89	Hungary	13.72	1	yes
D75	Other diseases of blood and blood-forming organs	D50.D89	Netherlands	23.52	1	yes
D75	Other diseases of blood and blood-forming organs	D50.D89	Poland	24.04	1	yes
D75	Other diseases of blood and blood-forming organs	D50.D89	Spain	47.39	1	yes
D75	Other diseases of blood and blood-forming organs	D50.D89	Sweden	11.30	1	yes
D75	Other diseases of blood and blood-forming organs	D50.D89	Switzerland	17.04	1	yes
D75	Other diseases of blood and blood-forming organs	D50.D89	United Kingdom	18.08	1	yes
D76	Certain diseases involving lymphoreticular tissue and reticulohistiocytic system	D50.D89	France	53.18	2	yes
D76	Certain diseases involving lymphoreticular tissue and reticulohistiocytic system	D50.D89	Germany	14.84	0	no
D76	Certain diseases involving lymphoreticular tissue and reticulohistiocytic system	D50.D89	Netherlands	6.38	1	yes
D76	Certain diseases involving lymphoreticular tissue and reticulohistiocytic system	D50.D89	Spain	19.31	1	yes
D76	Certain diseases involving lymphoreticular tissue and reticulohistiocytic system	D50.D89	Sweden	6.16	1	yes
D76	Certain diseases involving lymphoreticular tissue and reticulohistiocytic system	D50.D89	United Kingdom	32.24	3	yes
D80	Immunodeficiency with predominantly antibody defects	D50.D89	Germany	10.74	1	yes
D80	Immunodeficiency with predominantly antibody defects	D50.D89	United Kingdom	10.14	0	no
D81	Combined immunodeficiencies	D50.D89	Germany	5.65	0	no
D84	Other immunodeficiencies	D50.D89	Belgium	22.45	0	no
D84	Other immunodeficiencies	D50.D89	France	122.02	1	yes
D84	Other immunodeficiencies	D50.D89	Germany	36.58	0	no

For peer review only

D84	Other immunodeficiencies	D50.D89	Hungary	6.42	1	yes
D84	Other immunodeficiencies	D50.D89	Norway	6.05	0	no
D84	Other immunodeficiencies	D50.D89	Spain	59.29	3	yes
D84	Other immunodeficiencies	D50.D89	Sweden	7.55	0	no
D84	Other immunodeficiencies	D50.D89	United Kingdom	27.73	0	no
D86	Sarcoidosis	D50.D89	Austria	14.79	2	yes
D86	Sarcoidosis	D50.D89	Belgium	15.51	0	no
D86	Sarcoidosis	D50.D89	Czech Republic	8.12	2	yes
D86	Sarcoidosis	D50.D89	Denmark	20.52	0	no
D86	Sarcoidosis	D50.D89	Finland	14.84	0	no
D86	Sarcoidosis	D50.D89	France	89.81	0	no
D86	Sarcoidosis	D50.D89	Germany	129.32	1	yes
D86	Sarcoidosis	D50.D89	Hungary	6.66	0	no
D86	Sarcoidosis	D50.D89	Netherlands	32.22	2	yes
D86	Sarcoidosis	D50.D89	Norway	16.00	0	no
D86	Sarcoidosis	D50.D89	Poland	21.28	0	no
D86	Sarcoidosis	D50.D89	Spain	38.98	1	yes
D86	Sarcoidosis	D50.D89	Sweden	24.92	0	no
D86	Sarcoidosis	D50.D89	Switzerland	15.17	0	no
D86	Sarcoidosis	D50.D89	United Kingdom	161.13	1	yes
D89	Other disorders involving the immune mechanism, not elsewhere classified	D50.D89	Finland	5.26	1	yes
D89	Other disorders involving the immune mechanism, not elsewhere classified	D50.D89	France	25.48	3	yes
D89	Other disorders involving the immune mechanism, not elsewhere classified	D50.D89	Germany	59.98	2	yes
D89	Other disorders involving the immune mechanism, not elsewhere classified	D50.D89	Netherlands	10.64	0	no
D89	Other disorders involving the immune mechanism, not elsewhere classified	D50.D89	Spain	13.66	1	yes
D89	Other disorders involving the immune mechanism, not elsewhere classified	D50.D89	Sweden	10.55	2	yes
D89	Other disorders involving the immune mechanism, not elsewhere classified	D50.D89	United Kingdom	30.60	0	no
E03	Other hypothyroidism	E00.E90	Belgium	29.41	1	yes
E03	Other hypothyroidism	E00.E90	Czech Republic	13.05	2	yes
E03	Other hypothyroidism	E00.E90	Denmark	35.81	0	no
E03	Other hypothyroidism	E00.E90	France	376.42	2	yes
E03	Other hypothyroidism	E00.E90	Germany	66.80	3	yes
E03	Other hypothyroidism	E00.E90	Hungary	14.97	0	no
E03	Other hypothyroidism	E00.E90	Netherlands	52.44	1	yes
E03	Other hypothyroidism	E00.E90	Norway	23.27	0	no
E03	Other hypothyroidism	E00.E90	Poland	30.72	2	yes
E03	Other hypothyroidism	E00.E90	Spain	137.02	2	yes
E03	Other hypothyroidism	E00.E90	Sweden	24.65	2	yes
E03	Other hypothyroidism	E00.E90	Switzerland	12.13	0	no
E03	Other hypothyroidism	E00.E90	United Kingdom	266.26	1	yes
E04	Other nontoxic goitre	E00.E90	Belgium	9.20	1	yes
E04	Other nontoxic goitre	E00.E90	France	53.98	3	yes
E04	Other nontoxic goitre	E00.E90	Germany	44.88	1	yes
E04	Other nontoxic goitre	E00.E90	Hungary	11.55	0	no
E04	Other nontoxic goitre	E00.E90	Netherlands	14.49	0	no

For peer review only

E04	Other nontoxic goitre	E00.E90	Poland	6.49	3	yes
E04	Other nontoxic goitre	E00.E90	Spain	33.39	0	no
E04	Other nontoxic goitre	E00.E90	Sweden	6.24	0	no
E04	Other nontoxic goitre	E00.E90	United Kingdom	22.05	0	no
E05	Thyrotoxicosis [hyperthyroidism]	E00.E90	Belgium	47.74	1	yes
E05	Thyrotoxicosis [hyperthyroidism]	E00.E90	Czech Republic	10.93	1	yes
E05	Thyrotoxicosis [hyperthyroidism]	E00.E90	Denmark	33.92	0	no
E05	Thyrotoxicosis [hyperthyroidism]	E00.E90	France	270.36	2	yes
E05	Thyrotoxicosis [hyperthyroidism]	E00.E90	Germany	196.58	2	yes
E05	Thyrotoxicosis [hyperthyroidism]	E00.E90	Hungary	29.79	2	yes
E05	Thyrotoxicosis [hyperthyroidism]	E00.E90	Latvia	7.08	0	no
E05	Thyrotoxicosis [hyperthyroidism]	E00.E90	Netherlands	36.83	2	yes
E05	Thyrotoxicosis [hyperthyroidism]	E00.E90	Norway	6.39	0	no
E05	Thyrotoxicosis [hyperthyroidism]	E00.E90	Poland	105.17	1	yes
E05	Thyrotoxicosis [hyperthyroidism]	E00.E90	Romania	7.51	1	yes
E05	Thyrotoxicosis [hyperthyroidism]	E00.E90	Spain	71.23	0	no
E05	Thyrotoxicosis [hyperthyroidism]	E00.E90	Sweden	13.45	1	yes
E05	Thyrotoxicosis [hyperthyroidism]	E00.E90	Switzerland	9.72	1	yes
E05	Thyrotoxicosis [hyperthyroidism]	E00.E90	United Kingdom	84.30	2	yes
E06	Thyroiditis	E00.E90	France	7.55	0	no
E06	Thyroiditis	E00.E90	Germany	8.09	0	no
E07	Other disorders of thyroid	E00.E90	Belgium	8.71	3	yes
E07	Other disorders of thyroid	E00.E90	France	56.87	1	yes
E07	Other disorders of thyroid	E00.E90	Germany	12.97	0	no
E07	Other disorders of thyroid	E00.E90	Netherlands	7.93	1	yes
E07	Other disorders of thyroid	E00.E90	Spain	5.64	0	no
E07	Other disorders of thyroid	E00.E90	United Kingdom	6.40	1	yes
E10	Insulin-dependent diabetes mellitus	E00.E90	Austria	332.22	3	yes
E10	Insulin-dependent diabetes mellitus	E00.E90	Belgium	163.52	1	yes
E10	Insulin-dependent diabetes mellitus	E00.E90	Croatia	100.24	1	yes
E10	Insulin-dependent diabetes mellitus	E00.E90	Czech Republic	1177.68	0	no
E10	Insulin-dependent diabetes mellitus	E00.E90	Denmark	278.21	1	yes
E10	Insulin-dependent diabetes mellitus	E00.E90	Estonia	45.06	3	yes
E10	Insulin-dependent diabetes mellitus	E00.E90	Finland	95.63	0	no
E10	Insulin-dependent diabetes mellitus	E00.E90	France	3046.28	1	yes
E10	Insulin-dependent diabetes mellitus	E00.E90	Germany	969.94	2	yes
E10	Insulin-dependent diabetes mellitus	E00.E90	Hungary	755.56	0	no
E10	Insulin-dependent diabetes mellitus	E00.E90	Latvia	40.97	1	yes
E10	Insulin-dependent diabetes mellitus	E00.E90	Lithuania	84.44	1	yes
E10	Insulin-dependent diabetes mellitus	E00.E90	Netherlands	324.34	2	yes
E10	Insulin-dependent diabetes mellitus	E00.E90	Norway	82.61	2	yes
E10	Insulin-dependent diabetes mellitus	E00.E90	Poland	1681.84	0	no
E10	Insulin-dependent diabetes mellitus	E00.E90	Romania	717.13	3	yes
E10	Insulin-dependent diabetes mellitus	E00.E90	Slovenia	40.01	1	yes
E10	Insulin-dependent diabetes mellitus	E00.E90	Spain	633.13	2	yes
E10	Insulin-dependent diabetes mellitus	E00.E90	Sweden	77.23	1	yes
E10	Insulin-dependent diabetes mellitus	E00.E90	Switzerland	61.07	2	yes
E10	Insulin-dependent diabetes mellitus	E00.E90	United Kingdom	493.96	3	yes
E11	Non-insulin-dependent diabetes mellitus	E00.E90	Austria	915.71	3	yes
E11	Non-insulin-dependent diabetes mellitus	E00.E90	Belgium	391.17	2	yes

For peer review only

E11	Non-insulin-dependent diabetes mellitus	E00.E90	Croatia	293.00	2	yes
E11	Non-insulin-dependent diabetes mellitus	E00.E90	Czech Republic	1513.73	2	yes
E11	Non-insulin-dependent diabetes mellitus	E00.E90	Denmark	634.73	2	yes
E11	Non-insulin-dependent diabetes mellitus	E00.E90	Estonia	103.33	3	yes
E11	Non-insulin-dependent diabetes mellitus	E00.E90	Finland	469.87	2	yes
E11	Non-insulin-dependent diabetes mellitus	E00.E90	France	3573.42	3	yes
E11	Non-insulin-dependent diabetes mellitus	E00.E90	Germany	7613.03	2	yes
E11	Non-insulin-dependent diabetes mellitus	E00.E90	Hungary	1514.19	1	yes
E11	Non-insulin-dependent diabetes mellitus	E00.E90	Latvia	342.02	3	yes
E11	Non-insulin-dependent diabetes mellitus	E00.E90	Lithuania	220.26	1	yes
E11	Non-insulin-dependent diabetes mellitus	E00.E90	Netherlands	886.29	2	yes
E11	Non-insulin-dependent diabetes mellitus	E00.E90	Norway	183.88	3	yes
E11	Non-insulin-dependent diabetes mellitus	E00.E90	Poland	5319.63	1	yes
E11	Non-insulin-dependent diabetes mellitus	E00.E90	Romania	1393.68	1	yes
E11	Non-insulin-dependent diabetes mellitus	E00.E90	Slovenia	230.28	0	no
E11	Non-insulin-dependent diabetes mellitus	E00.E90	Spain	2224.42	3	yes
E11	Non-insulin-dependent diabetes mellitus	E00.E90	Sweden	472.85	0	no
E11	Non-insulin-dependent diabetes mellitus	E00.E90	Switzerland	956.07	2	yes
E11	Non-insulin-dependent diabetes mellitus	E00.E90	United Kingdom	2359.69	2	yes
E12	Malnutrition-related diabetes mellitus	E00.E90	Czech Republic	7.09	1	yes
E12	Malnutrition-related diabetes mellitus	E00.E90	Germany	13.99	1	yes
E12	Malnutrition-related diabetes mellitus	E00.E90	Poland	5.00	2	yes
E12	Malnutrition-related diabetes mellitus	E00.E90	Spain	7.26	0	no
E13	Other specified diabetes mellitus	E00.E90	Czech Republic	16.44	2	yes
E13	Other specified diabetes mellitus	E00.E90	Germany	40.32	2	yes
E13	Other specified diabetes mellitus	E00.E90	Hungary	19.41	2	yes
E13	Other specified diabetes mellitus	E00.E90	Poland	18.80	0	no
E14	Unspecified diabetes mellitus	E00.E90	Austria	2326.93	3	yes
E14	Unspecified diabetes mellitus	E00.E90	Belgium	1298.12	2	yes
E14	Unspecified diabetes mellitus	E00.E90	Croatia	1086.58	3	yes
E14	Unspecified diabetes mellitus	E00.E90	Czech Republic	71.83	0	no
E14	Unspecified diabetes mellitus	E00.E90	Denmark	373.88	3	yes
E14	Unspecified diabetes mellitus	E00.E90	Estonia	60.45	1	yes
E14	Unspecified diabetes mellitus	E00.E90	Finland	7.59	0	no
E14	Unspecified diabetes mellitus	E00.E90	France	5649.77	1	yes
E14	Unspecified diabetes mellitus	E00.E90	Germany	15850.80	1	yes
E14	Unspecified diabetes mellitus	E00.E90	Hungary	940.47	3	yes
E14	Unspecified diabetes mellitus	E00.E90	Latvia	53.96	1	yes
E14	Unspecified diabetes mellitus	E00.E90	Lithuania	36.12	2	yes
E14	Unspecified diabetes mellitus	E00.E90	Netherlands	3158.20	3	yes
E14	Unspecified diabetes mellitus	E00.E90	Norway	561.18	2	yes
E14	Unspecified diabetes mellitus	E00.E90	Poland	1765.40	2	yes
E14	Unspecified diabetes mellitus	E00.E90	Romania	659.55	3	yes
E14	Unspecified diabetes mellitus	E00.E90	Slovenia	264.93	2	yes
E14	Unspecified diabetes mellitus	E00.E90	Spain	8377.34	1	yes
E14	Unspecified diabetes mellitus	E00.E90	Sweden	1436.72	1	yes
E14	Unspecified diabetes mellitus	E00.E90	Switzerland	652.69	3	yes
E14	Unspecified diabetes mellitus	E00.E90	United Kingdom	4537.20	3	yes
E15	Nondiabetic hypoglycaemic coma	E00.E90	Belgium	9.02	0	no
E15	Nondiabetic hypoglycaemic coma	E00.E90	France	34.84	0	no

For peer review only

E15	Nondiabetic hypoglycaemic coma	E00.E90	Germany	14.34	0	no
E15	Nondiabetic hypoglycaemic coma	E00.E90	Netherlands	5.07	0	no
E15	Nondiabetic hypoglycaemic coma	E00.E90	Poland	6.78	3	yes
E15	Nondiabetic hypoglycaemic coma	E00.E90	Spain	17.30	0	no
E16	Other disorders of pancreatic internal secretion	E00.E90	Belgium	14.97	1	yes
E16	Other disorders of pancreatic internal secretion	E00.E90	France	52.14	0	no
E16	Other disorders of pancreatic internal secretion	E00.E90	Germany	47.81	1	yes
E16	Other disorders of pancreatic internal secretion	E00.E90	Netherlands	12.20	0	no
E16	Other disorders of pancreatic internal secretion	E00.E90	Poland	6.88	0	no
E16	Other disorders of pancreatic internal secretion	E00.E90	Spain	39.75	2	yes
E16	Other disorders of pancreatic internal secretion	E00.E90	United Kingdom	23.83	0	no
E21	Hyperparathyroidism and other disorders of parathyroid gland	E00.E90	Denmark	5.07	0	no
E21	Hyperparathyroidism and other disorders of parathyroid gland	E00.E90	France	26.61	0	no
E21	Hyperparathyroidism and other disorders of parathyroid gland	E00.E90	Germany	18.37	3	yes
E21	Hyperparathyroidism and other disorders of parathyroid gland	E00.E90	Netherlands	7.89	2	yes
E21	Hyperparathyroidism and other disorders of parathyroid gland	E00.E90	Spain	11.56	0	no
E21	Hyperparathyroidism and other disorders of parathyroid gland	E00.E90	Sweden	7.12	0	no
E21	Hyperparathyroidism and other disorders of parathyroid gland	E00.E90	Switzerland	5.31	0	no
E21	Hyperparathyroidism and other disorders of parathyroid gland	E00.E90	United Kingdom	28.11	0	no
E22	Hyperfunction of pituitary gland	E00.E90	Belgium	5.16	0	no
E22	Hyperfunction of pituitary gland	E00.E90	France	20.17	1	yes
E22	Hyperfunction of pituitary gland	E00.E90	Germany	6.06	0	no
E22	Hyperfunction of pituitary gland	E00.E90	Spain	10.91	0	no
E22	Hyperfunction of pituitary gland	E00.E90	United Kingdom	12.43	0	no
E23	Hypofunction and other disorders of pituitary gland	E00.E90	France	31.29	2	yes
E23	Hypofunction and other disorders of pituitary gland	E00.E90	Germany	16.18	0	no
E23	Hypofunction and other disorders of pituitary gland	E00.E90	Netherlands	8.64	0	no
E23	Hypofunction and other disorders of pituitary gland	E00.E90	Poland	5.69	0	no
E23	Hypofunction and other disorders of pituitary gland	E00.E90	Romania	6.15	2	yes
E23	Hypofunction and other disorders of pituitary gland	E00.E90	Spain	21.09	0	no
E23	Hypofunction and other disorders of pituitary gland	E00.E90	United Kingdom	17.24	1	yes
E24	Cushing's syndrome	E00.E90	France	9.28	0	no
E24	Cushing's syndrome	E00.E90	Germany	7.73	0	no
E24	Cushing's syndrome	E00.E90	Spain	8.94	0	no
E24	Cushing's syndrome	E00.E90	United Kingdom	5.94	1	yes
E27	Other disorders of adrenal gland	E00.E90	Austria	5.12	0	no
E27	Other disorders of adrenal gland	E00.E90	Belgium	7.09	0	no
E27	Other disorders of adrenal gland	E00.E90	Denmark	6.89	0	no
E27	Other disorders of adrenal gland	E00.E90	France	73.34	1	yes
E27	Other disorders of adrenal gland	E00.E90	Germany	34.40	0	no
E27	Other disorders of adrenal gland	E00.E90	Hungary	8.07	1	yes
E27	Other disorders of adrenal gland	E00.E90	Netherlands	12.42	0	no
E27	Other disorders of adrenal gland	E00.E90	Norway	5.04	0	no
E27	Other disorders of adrenal gland	E00.E90	Poland	14.81	1	yes
E27	Other disorders of adrenal gland	E00.E90	Spain	41.47	2	yes

For peer review only

E27	Other disorders of adrenal gland	E00.E90	Sweden	6.17	1	yes
E27	Other disorders of adrenal gland	E00.E90	Switzerland	5.85	0	no
E27	Other disorders of adrenal gland	E00.E90	United Kingdom	38.42	1	yes
E34	Other endocrine disorders	E00.E90	France	5.96	1	yes
E34	Other endocrine disorders	E00.E90	Germany	6.35	1	yes
E34	Other endocrine disorders	E00.E90	Poland	6.92	0	no
E34	Other endocrine disorders	E00.E90	Spain	6.14	1	yes
E34	Other endocrine disorders	E00.E90	United Kingdom	20.88	1	yes
E41	Nutritional marasmus	E00.E90	Belgium	34.35	1	yes
E41	Nutritional marasmus	E00.E90	Czech Republic	28.47	1	yes
E41	Nutritional marasmus	E00.E90	Denmark	28.04	1	yes
E41	Nutritional marasmus	E00.E90	France	237.92	1	yes
E41	Nutritional marasmus	E00.E90	Germany	171.16	3	yes
E41	Nutritional marasmus	E00.E90	Netherlands	7.84	3	yes
E41	Nutritional marasmus	E00.E90	Poland	102.71	2	yes
E41	Nutritional marasmus	E00.E90	Romania	33.17	1	yes
E41	Nutritional marasmus	E00.E90	Sweden	13.99	1	yes
E41	Nutritional marasmus	E00.E90	Switzerland	7.39	0	no
E41	Nutritional marasmus	E00.E90	United Kingdom	8.09	0	no
E43	Unspecified severe protein-energy malnutrition	E00.E90	Belgium	9.25	1	yes
E43	Unspecified severe protein-energy malnutrition	E00.E90	Czech Republic	44.54	3	yes
E43	Unspecified severe protein-energy malnutrition	E00.E90	France	338.75	3	yes
E43	Unspecified severe protein-energy malnutrition	E00.E90	Hungary	8.65	3	yes
E43	Unspecified severe protein-energy malnutrition	E00.E90	Netherlands	82.91	2	yes
E43	Unspecified severe protein-energy malnutrition	E00.E90	Poland	7.28	0	no
E43	Unspecified severe protein-energy malnutrition	E00.E90	Romania	36.37	1	yes
E43	Unspecified severe protein-energy malnutrition	E00.E90	Spain	32.61	3	yes
E43	Unspecified severe protein-energy malnutrition	E00.E90	United Kingdom	8.71	0	no
E46	Unspecified protein-energy malnutrition	E00.E90	Belgium	136.62	2	yes
E46	Unspecified protein-energy malnutrition	E00.E90	France	2308.57	0	no
E46	Unspecified protein-energy malnutrition	E00.E90	Germany	36.99	2	yes
E46	Unspecified protein-energy malnutrition	E00.E90	Hungary	6.28	1	yes
E46	Unspecified protein-energy malnutrition	E00.E90	Netherlands	19.95	0	no
E46	Unspecified protein-energy malnutrition	E00.E90	Poland	7.31	2	yes
E46	Unspecified protein-energy malnutrition	E00.E90	Romania	17.29	0	no
E46	Unspecified protein-energy malnutrition	E00.E90	Spain	133.97	1	yes
E46	Unspecified protein-energy malnutrition	E00.E90	Sweden	43.76	2	yes
E46	Unspecified protein-energy malnutrition	E00.E90	Switzerland	27.75	2	yes
E46	Unspecified protein-energy malnutrition	E00.E90	United Kingdom	81.65	0	no
E51	Thiamine deficiency	E00.E90	France	12.42	0	no
E51	Thiamine deficiency	E00.E90	Germany	28.66	2	yes
E51	Thiamine deficiency	E00.E90	Spain	12.80	0	no
E51	Thiamine deficiency	E00.E90	United Kingdom	5.84	0	no
E63	Other nutritional deficiencies	E00.E90	France	5.79	2	yes
E63	Other nutritional deficiencies	E00.E90	Germany	8.96	0	no
E63	Other nutritional deficiencies	E00.E90	United Kingdom	13.11	1	yes
E64	Sequelae of malnutrition and other nutritional deficiencies	E00.E90	Denmark	5.32	1	yes
E66	Obesity	E00.E90	Austria	652.00	3	yes
E66	Obesity	E00.E90	Belgium	134.09	2	yes

For peer review only

E66	Obesity	E00.E90	Croatia	8.82	2	yes
E66	Obesity	E00.E90	Czech Republic	34.11	3	yes
E66	Obesity	E00.E90	Denmark	86.09	1	yes
E66	Obesity	E00.E90	Estonia	7.61	0	no
E66	Obesity	E00.E90	Finland	29.68	1	yes
E66	Obesity	E00.E90	France	1042.62	3	yes
E66	Obesity	E00.E90	Germany	1489.35	2	yes
E66	Obesity	E00.E90	Hungary	110.26	2	yes
E66	Obesity	E00.E90	Latvia	7.37	2	yes
E66	Obesity	E00.E90	Lithuania	6.62	0	no
E66	Obesity	E00.E90	Netherlands	93.31	2	yes
E66	Obesity	E00.E90	Norway	26.22	1	yes
E66	Obesity	E00.E90	Poland	39.36	3	yes
E66	Obesity	E00.E90	Slovenia	14.74	3	yes
E66	Obesity	E00.E90	Spain	669.40	2	yes
E66	Obesity	E00.E90	Sweden	56.74	1	yes
E66	Obesity	E00.E90	Switzerland	75.06	1	yes
E66	Obesity	E00.E90	United Kingdom	381.59	1	yes
E71	Disorders of branched-chain amino-acid metabolism and fatty-acid metabolism	E00.E90	France	10.51	1	yes
E71	Disorders of branched-chain amino-acid metabolism and fatty-acid metabolism	E00.E90	Germany	16.47	1	yes
E71	Disorders of branched-chain amino-acid metabolism and fatty-acid metabolism	E00.E90	Spain	8.22	2	yes
E71	Disorders of branched-chain amino-acid metabolism and fatty-acid metabolism	E00.E90	United Kingdom	10.66	2	yes
E72	Other disorders of amino-acid metabolism	E00.E90	Belgium	20.34	2	yes
E72	Other disorders of amino-acid metabolism	E00.E90	France	40.39	0	no
E72	Other disorders of amino-acid metabolism	E00.E90	Germany	13.15	0	no
E72	Other disorders of amino-acid metabolism	E00.E90	Spain	9.61	1	yes
E72	Other disorders of amino-acid metabolism	E00.E90	United Kingdom	15.12	0	no
E74	Other disorders of carbohydrate metabolism	E00.E90	France	6.39	0	no
E74	Other disorders of carbohydrate metabolism	E00.E90	Germany	12.22	0	no
E74	Other disorders of carbohydrate metabolism	E00.E90	Hungary	6.83	2	yes
E74	Other disorders of carbohydrate metabolism	E00.E90	United Kingdom	8.26	0	no
E75	Disorders of sphingolipid metabolism and other lipid storage disorders	E00.E90	Austria	6.36	0	no
E75	Disorders of sphingolipid metabolism and other lipid storage disorders	E00.E90	Belgium	7.26	1	yes
E75	Disorders of sphingolipid metabolism and other lipid storage disorders	E00.E90	Finland	8.34	0	no
E75	Disorders of sphingolipid metabolism and other lipid storage disorders	E00.E90	France	41.27	0	no
E75	Disorders of sphingolipid metabolism and other lipid storage disorders	E00.E90	Germany	53.25	1	yes
E75	Disorders of sphingolipid metabolism and other lipid storage disorders	E00.E90	Netherlands	15.39	0	no
E75	Disorders of sphingolipid metabolism and other lipid storage disorders	E00.E90	Poland	13.57	0	no
E75	Disorders of sphingolipid metabolism and other lipid storage disorders	E00.E90	Spain	25.45	0	no
E75	Disorders of sphingolipid metabolism and other lipid storage disorders	E00.E90	Sweden	8.34	0	no
E75	Disorders of sphingolipid metabolism and other lipid storage disorders	E00.E90	Switzerland	5.41	0	no

For peer review only

E75	Disorders of sphingolipid metabolism and other lipid storage disorders	E00.E90	United Kingdom	44.95	0	no
E76	Disorders of glycosaminoglycan metabolism	E00.E90	France	9.34	0	no
E76	Disorders of glycosaminoglycan metabolism	E00.E90	Germany	13.25	0	no
E76	Disorders of glycosaminoglycan metabolism	E00.E90	Poland	6.17	0	no
E76	Disorders of glycosaminoglycan metabolism	E00.E90	Spain	9.70	0	no
E76	Disorders of glycosaminoglycan metabolism	E00.E90	United Kingdom	13.10	0	no
E77	Disorders of glycoprotein metabolism	E00.E90	Finland	5.12	0	no
E77	Disorders of glycoprotein metabolism	E00.E90	Germany	6.94	0	no
E77	Disorders of glycoprotein metabolism	E00.E90	Spain	7.92	0	no
E77	Disorders of glycoprotein metabolism	E00.E90	United Kingdom	8.57	1	yes
E78	Disorders of lipoprotein metabolism and other lipidaemias	E00.E90	Austria	91.14	2	yes
E78	Disorders of lipoprotein metabolism and other lipidaemias	E00.E90	Belgium	52.41	0	no
E78	Disorders of lipoprotein metabolism and other lipidaemias	E00.E90	Czech Republic	14.84	2	yes
E78	Disorders of lipoprotein metabolism and other lipidaemias	E00.E90	Denmark	55.93	3	yes
E78	Disorders of lipoprotein metabolism and other lipidaemias	E00.E90	France	430.05	1	yes
E78	Disorders of lipoprotein metabolism and other lipidaemias	E00.E90	Germany	307.22	1	yes
E78	Disorders of lipoprotein metabolism and other lipidaemias	E00.E90	Hungary	32.90	2	yes
E78	Disorders of lipoprotein metabolism and other lipidaemias	E00.E90	Netherlands	32.24	3	yes
E78	Disorders of lipoprotein metabolism and other lipidaemias	E00.E90	Norway	35.46	1	yes
E78	Disorders of lipoprotein metabolism and other lipidaemias	E00.E90	Spain	276.40	2	yes
E78	Disorders of lipoprotein metabolism and other lipidaemias	E00.E90	Sweden	46.34	2	yes
E78	Disorders of lipoprotein metabolism and other lipidaemias	E00.E90	Switzerland	12.34	0	no
E78	Disorders of lipoprotein metabolism and other lipidaemias	E00.E90	United Kingdom	160.49	1	yes
E79	Disorders of purine and pyrimidine metabolism	E00.E90	Germany	11.70	2	yes
E80	Disorders of porphyrin and bilirubin metabolism	E00.E90	France	6.00	0	no
E83	Disorders of mineral metabolism	E00.E90	Austria	7.63	0	no
E83	Disorders of mineral metabolism	E00.E90	Belgium	12.21	1	yes
E83	Disorders of mineral metabolism	E00.E90	Czech Republic	5.89	2	yes
E83	Disorders of mineral metabolism	E00.E90	Denmark	5.30	0	no
E83	Disorders of mineral metabolism	E00.E90	France	139.63	1	yes
E83	Disorders of mineral metabolism	E00.E90	Germany	55.95	1	yes
E83	Disorders of mineral metabolism	E00.E90	Hungary	6.03	0	no
E83	Disorders of mineral metabolism	E00.E90	Netherlands	13.76	1	yes
E83	Disorders of mineral metabolism	E00.E90	Norway	5.74	0	no
E83	Disorders of mineral metabolism	E00.E90	Poland	9.32	2	yes
E83	Disorders of mineral metabolism	E00.E90	Spain	36.23	0	no
E83	Disorders of mineral metabolism	E00.E90	Sweden	11.62	0	no
E83	Disorders of mineral metabolism	E00.E90	Switzerland	11.38	0	no
E83	Disorders of mineral metabolism	E00.E90	United Kingdom	75.23	0	no
E84	Cystic fibrosis	E00.E90	Austria	6.84	3	yes
E84	Cystic fibrosis	E00.E90	Belgium	7.92	0	no

For peer review only

E84	Cystic fibrosis	E00.E90	Czech Republic	6.27	1	yes
E84	Cystic fibrosis	E00.E90	Denmark	5.89	0	no
E84	Cystic fibrosis	E00.E90	France	55.73	1	yes
E84	Cystic fibrosis	E00.E90	Germany	64.07	1	yes
E84	Cystic fibrosis	E00.E90	Hungary	5.95	0	no
E84	Cystic fibrosis	E00.E90	Netherlands	15.50	0	no
E84	Cystic fibrosis	E00.E90	Poland	16.63	1	yes
E84	Cystic fibrosis	E00.E90	Romania	6.35	0	no
E84	Cystic fibrosis	E00.E90	Spain	29.53	0	no
E84	Cystic fibrosis	E00.E90	Switzerland	8.44	1	yes
E84	Cystic fibrosis	E00.E90	United Kingdom	115.48	0	no
E85	Amyloidosis	E00.E90	Austria	22.15	2	yes
E85	Amyloidosis	E00.E90	Belgium	50.80	0	no
E85	Amyloidosis	E00.E90	Czech Republic	13.40	1	yes
E85	Amyloidosis	E00.E90	Denmark	17.88	0	no
E85	Amyloidosis	E00.E90	Estonia	5.99	0	no
E85	Amyloidosis	E00.E90	Finland	49.95	2	yes
E85	Amyloidosis	E00.E90	France	252.07	2	yes
E85	Amyloidosis	E00.E90	Germany	194.50	2	yes
E85	Amyloidosis	E00.E90	Hungary	7.45	0	no
E85	Amyloidosis	E00.E90	Lithuania	9.24	0	no
E85	Amyloidosis	E00.E90	Netherlands	65.25	1	yes
E85	Amyloidosis	E00.E90	Norway	27.11	1	yes
E85	Amyloidosis	E00.E90	Poland	35.14	1	yes
E85	Amyloidosis	E00.E90	Slovenia	7.33	1	yes
E85	Amyloidosis	E00.E90	Spain	197.81	2	yes
E85	Amyloidosis	E00.E90	Sweden	51.34	0	no
E85	Amyloidosis	E00.E90	Switzerland	41.45	1	yes
E85	Amyloidosis	E00.E90	United Kingdom	300.46	2	yes
E86	Volume depletion	E00.E90	Belgium	368.30	1	yes
E86	Volume depletion	E00.E90	Czech Republic	64.54	2	yes
E86	Volume depletion	E00.E90	Denmark	213.48	3	yes
E86	Volume depletion	E00.E90	France	2056.06	1	yes
E86	Volume depletion	E00.E90	Germany	1537.92	1	yes
E86	Volume depletion	E00.E90	Hungary	32.52	2	yes
E86	Volume depletion	E00.E90	Netherlands	438.51	3	yes
E86	Volume depletion	E00.E90	Norway	124.38	3	yes
E86	Volume depletion	E00.E90	Poland	59.66	1	yes
E86	Volume depletion	E00.E90	Spain	332.92	3	yes
E86	Volume depletion	E00.E90	Sweden	64.28	0	no
E86	Volume depletion	E00.E90	Switzerland	43.66	3	yes
E86	Volume depletion	E00.E90	United Kingdom	223.36	2	yes
E87	Other disorders of fluid, electrolyte and acid-base balance	E00.E90	Austria	5.39	0	no
E87	Other disorders of fluid, electrolyte and acid-base balance	E00.E90	Belgium	152.25	1	yes
E87	Other disorders of fluid, electrolyte and acid-base balance	E00.E90	Czech Republic	31.66	2	yes
E87	Other disorders of fluid, electrolyte and acid-base balance	E00.E90	Denmark	27.20	2	yes
E87	Other disorders of fluid, electrolyte and acid-base balance	E00.E90	France	868.82	1	yes

For peer review only

E87	Other disorders of fluid, electrolyte and acid-base balance	E00.E90	Germany	408.44	3	yes
E87	Other disorders of fluid, electrolyte and acid-base balance	E00.E90	Hungary	12.73	1	yes
E87	Other disorders of fluid, electrolyte and acid-base balance	E00.E90	Netherlands	45.71	0	no
E87	Other disorders of fluid, electrolyte and acid-base balance	E00.E90	Norway	14.31	1	yes
E87	Other disorders of fluid, electrolyte and acid-base balance	E00.E90	Poland	35.30	3	yes
E87	Other disorders of fluid, electrolyte and acid-base balance	E00.E90	Spain	352.57	3	yes
E87	Other disorders of fluid, electrolyte and acid-base balance	E00.E90	Sweden	20.57	2	yes
E87	Other disorders of fluid, electrolyte and acid-base balance	E00.E90	Switzerland	13.03	0	no
E87	Other disorders of fluid, electrolyte and acid-base balance	E00.E90	United Kingdom	170.39	3	yes
E88	Other metabolic disorders	E00.E90	Austria	52.13	1	yes
E88	Other metabolic disorders	E00.E90	Belgium	56.20	0	no
E88	Other metabolic disorders	E00.E90	Czech Republic	13.14	1	yes
E88	Other metabolic disorders	E00.E90	Denmark	15.78	2	yes
E88	Other metabolic disorders	E00.E90	Finland	6.94	0	no
E88	Other metabolic disorders	E00.E90	France	302.03	1	yes
E88	Other metabolic disorders	E00.E90	Germany	174.86	2	yes
E88	Other metabolic disorders	E00.E90	Hungary	10.25	2	yes
E88	Other metabolic disorders	E00.E90	Netherlands	24.62	2	yes
E88	Other metabolic disorders	E00.E90	Norway	9.88	0	no
E88	Other metabolic disorders	E00.E90	Poland	10.09	0	no
E88	Other metabolic disorders	E00.E90	Spain	109.99	1	yes
E88	Other metabolic disorders	E00.E90	Sweden	22.59	0	no
E88	Other metabolic disorders	E00.E90	Switzerland	30.96	2	yes
E88	Other metabolic disorders	E00.E90	United Kingdom	123.69	0	no
F01	Vascular dementia	F00.F99	Belgium	530.86	3	yes
F01	Vascular dementia	F00.F99	Croatia	68.99	1	yes
F01	Vascular dementia	F00.F99	Czech Republic	368.65	2	yes
F01	Vascular dementia	F00.F99	Denmark	368.28	2	yes
F01	Vascular dementia	F00.F99	Finland	1134.25	3	yes
F01	Vascular dementia	F00.F99	France	1749.57	3	yes
F01	Vascular dementia	F00.F99	Germany	2155.72	3	yes
F01	Vascular dementia	F00.F99	Hungary	1702.73	1	yes
F01	Vascular dementia	F00.F99	Latvia	102.04	1	yes
F01	Vascular dementia	F00.F99	Lithuania	36.27	2	yes
F01	Vascular dementia	F00.F99	Netherlands	1106.08	1	yes
F01	Vascular dementia	F00.F99	Norway	146.85	1	yes
F01	Vascular dementia	F00.F99	Poland	66.22	1	yes
F01	Vascular dementia	F00.F99	Spain	2725.66	2	yes
F01	Vascular dementia	F00.F99	Sweden	818.66	3	yes
F01	Vascular dementia	F00.F99	Switzerland	548.14	2	yes
F01	Vascular dementia	F00.F99	United Kingdom	4647.02	2	yes
F03	Unspecified dementia	F00.F99	Austria	359.75	2	yes
F03	Unspecified dementia	F00.F99	Belgium	2944.56	2	yes
F03	Unspecified dementia	F00.F99	Croatia	360.08	2	yes

For peer review only

F03	Unspecified dementia	F00.F99	Denmark	1914.34	1	yes
F03	Unspecified dementia	F00.F99	Finland	1808.33	3	yes
F03	Unspecified dementia	F00.F99	France	11844.02	3	yes
F03	Unspecified dementia	F00.F99	Germany	12396.97	2	yes
F03	Unspecified dementia	F00.F99	Hungary	348.93	3	yes
F03	Unspecified dementia	F00.F99	Latvia	8.93	2	yes
F03	Unspecified dementia	F00.F99	Lithuania	8.84	2	yes
F03	Unspecified dementia	F00.F99	Netherlands	8622.18	3	yes
F03	Unspecified dementia	F00.F99	Norway	1534.12	3	yes
F03	Unspecified dementia	F00.F99	Poland	110.45	2	yes
F03	Unspecified dementia	F00.F99	Spain	12598.62	2	yes
F03	Unspecified dementia	F00.F99	Sweden	3665.26	2	yes
F03	Unspecified dementia	F00.F99	Switzerland	2624.49	1	yes
F03	Unspecified dementia	F00.F99	United Kingdom	23684.95	2	yes
F04	Organic amnesic syndrome, not induced by alcohol and other psychoactive substances	F00.F99	France	54.07	2	yes
F04	Organic amnesic syndrome, not induced by alcohol and other psychoactive substances	F00.F99	Germany	36.32	2	yes
F05	Delirium, not induced by alcohol and other psychoactive substances	F00.F99	Croatia	24.71	3	yes
F05	Delirium, not induced by alcohol and other psychoactive substances	F00.F99	Denmark	10.48	1	yes
F05	Delirium, not induced by alcohol and other psychoactive substances	F00.F99	Germany	68.30	3	yes
F05	Delirium, not induced by alcohol and other psychoactive substances	F00.F99	Hungary	31.79	0	no
F05	Delirium, not induced by alcohol and other psychoactive substances	F00.F99	Lithuania	6.19	0	no
F05	Delirium, not induced by alcohol and other psychoactive substances	F00.F99	Netherlands	57.34	0	no
F05	Delirium, not induced by alcohol and other psychoactive substances	F00.F99	Spain	15.39	3	yes
F05	Delirium, not induced by alcohol and other psychoactive substances	F00.F99	Switzerland	24.09	2	yes
F05	Delirium, not induced by alcohol and other psychoactive substances	F00.F99	United Kingdom	28.03	3	yes
F06	Other mental disorders due to brain damage and dysfunction and to physical disease	F00.F99	Croatia	8.45	0	no
F06	Other mental disorders due to brain damage and dysfunction and to physical disease	F00.F99	Germany	300.40	3	yes
F06	Other mental disorders due to brain damage and dysfunction and to physical disease	F00.F99	Netherlands	22.84	2	yes
F06	Other mental disorders due to brain damage and dysfunction and to physical disease	F00.F99	Spain	22.04	3	yes
F06	Other mental disorders due to brain damage and dysfunction and to physical disease	F00.F99	United Kingdom	6.18	0	no
F07	Personality and behavioural disorders due to brain disease, damage and dysfunction	F00.F99	Belgium	12.84	1	yes
F07	Personality and behavioural disorders due to brain disease, damage and dysfunction	F00.F99	Croatia	318.48	1	yes
F07	Personality and behavioural disorders due to brain disease, damage and dysfunction	F00.F99	France	201.98	1	yes
F07	Personality and behavioural disorders due to brain disease, damage and dysfunction	F00.F99	Germany	68.75	2	yes
F07	Personality and behavioural disorders due to brain disease, damage and dysfunction	F00.F99	Latvia	5.22	0	no
F07	Personality and behavioural disorders due to brain disease, damage and dysfunction	F00.F99	Netherlands	50.40	3	yes

For peer review only

F07	Personality and behavioural disorders due to brain disease, damage and dysfunction	F00.F99	Sweden	21.23	1	yes
F07	Personality and behavioural disorders due to brain disease, damage and dysfunction	F00.F99	Switzerland	84.04	2	yes
F10	Mental and behavioural disorders due to use of alcohol	F00.F99	Austria	432.48	1	yes
F10	Mental and behavioural disorders due to use of alcohol	F00.F99	Belgium	357.70	2	yes
F10	Mental and behavioural disorders due to use of alcohol	F00.F99	Croatia	238.99	2	yes
F10	Mental and behavioural disorders due to use of alcohol	F00.F99	Czech Republic	151.14	3	yes
F10	Mental and behavioural disorders due to use of alcohol	F00.F99	Denmark	728.40	3	yes
F10	Mental and behavioural disorders due to use of alcohol	F00.F99	Estonia	114.20	3	yes
F10	Mental and behavioural disorders due to use of alcohol	F00.F99	Finland	200.74	2	yes
F10	Mental and behavioural disorders due to use of alcohol	F00.F99	France	3086.12	2	yes
F10	Mental and behavioural disorders due to use of alcohol	F00.F99	Germany	4754.56	2	yes
F10	Mental and behavioural disorders due to use of alcohol	F00.F99	Hungary	412.45	3	yes
F10	Mental and behavioural disorders due to use of alcohol	F00.F99	Latvia	125.22	3	yes
F10	Mental and behavioural disorders due to use of alcohol	F00.F99	Lithuania	30.13	0	no
F10	Mental and behavioural disorders due to use of alcohol	F00.F99	Netherlands	282.39	2	yes
F10	Mental and behavioural disorders due to use of alcohol	F00.F99	Norway	198.32	1	yes
F10	Mental and behavioural disorders due to use of alcohol	F00.F99	Poland	1657.21	0	no
F10	Mental and behavioural disorders due to use of alcohol	F00.F99	Romania	444.49	2	yes
F10	Mental and behavioural disorders due to use of alcohol	F00.F99	Slovenia	138.73	2	yes
F10	Mental and behavioural disorders due to use of alcohol	F00.F99	Spain	325.05	1	yes
F10	Mental and behavioural disorders due to use of alcohol	F00.F99	Sweden	301.10	3	yes
F10	Mental and behavioural disorders due to use of alcohol	F00.F99	Switzerland	234.47	1	yes
F10	Mental and behavioural disorders due to use of alcohol	F00.F99	United Kingdom	883.45	1	yes
F11	Mental and behavioural disorders due to use of opioids	F00.F99	Austria	102.31	3	yes
F11	Mental and behavioural disorders due to use of opioids	F00.F99	Belgium	8.63	2	yes
F11	Mental and behavioural disorders due to use of opioids	F00.F99	Croatia	18.12	1	yes
F11	Mental and behavioural disorders due to use of opioids	F00.F99	Denmark	9.90	0	no
F11	Mental and behavioural disorders due to use of opioids	F00.F99	France	24.47	2	yes
F11	Mental and behavioural disorders due to use of opioids	F00.F99	Germany	178.87	1	yes
F11	Mental and behavioural disorders due to use of opioids	F00.F99	Netherlands	9.63	1	yes

For peer review only

F11	Mental and behavioural disorders due to use of opioids	F00.F99	Norway	32.29	3	yes
F11	Mental and behavioural disorders due to use of opioids	F00.F99	Spain	5.92	1	yes
F11	Mental and behavioural disorders due to use of opioids	F00.F99	Switzerland	32.30	3	yes
F11	Mental and behavioural disorders due to use of opioids	F00.F99	United Kingdom	438.12	2	yes
F13	Mental and behavioural disorders due to use of sedatives or hypnotics	F00.F99	Germany	6.93	0	no
F14	Mental and behavioural disorders due to use of cocaine	F00.F99	Germany	6.96	0	no
F14	Mental and behavioural disorders due to use of cocaine	F00.F99	United Kingdom	27.18	3	yes
F15	Mental and behavioural disorders due to use of other stimulants, including caffeine	F00.F99	United Kingdom	16.38	3	yes
F17	Mental and behavioural disorders due to use of tobacco	F00.F99	Belgium	33.92	2	yes
F17	Mental and behavioural disorders due to use of tobacco	F00.F99	France	447.47	2	yes
F17	Mental and behavioural disorders due to use of tobacco	F00.F99	Germany	157.99	3	yes
F17	Mental and behavioural disorders due to use of tobacco	F00.F99	Netherlands	13.39	0	no
F17	Mental and behavioural disorders due to use of tobacco	F00.F99	Spain	12.76	1	yes
F17	Mental and behavioural disorders due to use of tobacco	F00.F99	Switzerland	22.07	0	no
F18	Mental and behavioural disorders due to use of volatile solvents	F00.F99	United Kingdom	17.22	1	yes
F19	Mental and behavioural disorders due to multiple drug use and use of other psychoactive substances	F00.F99	Austria	65.05	3	yes
F19	Mental and behavioural disorders due to multiple drug use and use of other psychoactive substances	F00.F99	Belgium	35.41	2	yes
F19	Mental and behavioural disorders due to multiple drug use and use of other psychoactive substances	F00.F99	Denmark	23.64	0	no
F19	Mental and behavioural disorders due to multiple drug use and use of other psychoactive substances	F00.F99	Finland	15.58	0	no
F19	Mental and behavioural disorders due to multiple drug use and use of other psychoactive substances	F00.F99	France	128.48	3	yes
F19	Mental and behavioural disorders due to multiple drug use and use of other psychoactive substances	F00.F99	Germany	527.01	1	yes
F19	Mental and behavioural disorders due to multiple drug use and use of other psychoactive substances	F00.F99	Lithuania	5.24	2	yes
F19	Mental and behavioural disorders due to multiple drug use and use of other psychoactive substances	F00.F99	Norway	30.44	3	yes
F19	Mental and behavioural disorders due to multiple drug use and use of other psychoactive substances	F00.F99	Spain	52.53	3	yes
F19	Mental and behavioural disorders due to multiple drug use and use of other psychoactive substances	F00.F99	Sweden	17.56	0	no
F19	Mental and behavioural disorders due to multiple drug use and use of other psychoactive substances	F00.F99	Switzerland	65.50	3	yes
F19	Mental and behavioural disorders due to multiple drug use and use of other psychoactive substances	F00.F99	United Kingdom	234.29	2	yes
F20	Schizophrenia	F00.F99	Belgium	17.19	0	no
F20	Schizophrenia	F00.F99	Croatia	43.83	1	yes
F20	Schizophrenia	F00.F99	Denmark	50.50	0	no
F20	Schizophrenia	F00.F99	Finland	55.04	0	no
F20	Schizophrenia	F00.F99	France	126.32	1	yes
F20	Schizophrenia	F00.F99	Germany	78.48	2	yes

For peer review only

F20	Schizophrenia	F00.F99	Hungary	67.16	1	yes
F20	Schizophrenia	F00.F99	Latvia	6.76	0	no
F20	Schizophrenia	F00.F99	Lithuania	5.97	0	no
F20	Schizophrenia	F00.F99	Netherlands	31.74	0	no
F20	Schizophrenia	F00.F99	Norway	17.21	0	no
F20	Schizophrenia	F00.F99	Poland	27.37	3	yes
F20	Schizophrenia	F00.F99	Romania	85.98	2	yes
F20	Schizophrenia	F00.F99	Spain	127.64	1	yes
F20	Schizophrenia	F00.F99	Sweden	27.56	0	no
F20	Schizophrenia	F00.F99	Switzerland	69.47	2	yes
F20	Schizophrenia	F00.F99	United Kingdom	75.42	1	yes
F22	Persistent delusional disorders	F00.F99	Belgium	6.21	0	no
F22	Persistent delusional disorders	F00.F99	Denmark	13.37	1	yes
F22	Persistent delusional disorders	F00.F99	France	69.11	1	yes
F22	Persistent delusional disorders	F00.F99	Germany	8.13	0	no
F22	Persistent delusional disorders	F00.F99	Spain	6.41	0	no
F22	Persistent delusional disorders	F00.F99	Sweden	6.28	0	no
F22	Persistent delusional disorders	F00.F99	Switzerland	5.40	0	no
F22	Persistent delusional disorders	F00.F99	United Kingdom	5.92	1	yes
F23	Acute and transient psychotic disorders	F00.F99	France	7.58	3	yes
F25	Schizoaffective disorders	F00.F99	Germany	6.97	2	yes
F25	Schizoaffective disorders	F00.F99	Switzerland	5.94	0	no
F28	Other nonorganic psychotic disorders	F00.F99	France	50.02	1	yes
F29	Unspecified nonorganic psychosis	F00.F99	Belgium	10.02	0	no
F29	Unspecified nonorganic psychosis	F00.F99	Croatia	11.51	1	yes
F29	Unspecified nonorganic psychosis	F00.F99	France	221.53	3	yes
F29	Unspecified nonorganic psychosis	F00.F99	Germany	17.94	1	yes
F29	Unspecified nonorganic psychosis	F00.F99	Netherlands	12.16	0	no
F29	Unspecified nonorganic psychosis	F00.F99	Spain	10.59	0	no
F29	Unspecified nonorganic psychosis	F00.F99	Sweden	6.31	3	yes
F29	Unspecified nonorganic psychosis	F00.F99	Switzerland	11.84	0	no
F31	Bipolar affective disorder	F00.F99	Belgium	5.61	2	yes
F31	Bipolar affective disorder	F00.F99	Denmark	18.74	0	no
F31	Bipolar affective disorder	F00.F99	France	135.85	1	yes
F31	Bipolar affective disorder	F00.F99	Germany	6.42	2	yes
F31	Bipolar affective disorder	F00.F99	Netherlands	17.22	0	no
F31	Bipolar affective disorder	F00.F99	Norway	6.17	0	no
F31	Bipolar affective disorder	F00.F99	Spain	22.13	0	no
F31	Bipolar affective disorder	F00.F99	Sweden	13.21	1	yes
F31	Bipolar affective disorder	F00.F99	Switzerland	9.25	0	no
F31	Bipolar affective disorder	F00.F99	United Kingdom	17.77	1	yes
F32	Depressive episodes	F00.F99	Belgium	84.40	2	yes
F32	Depressive episodes	F00.F99	Croatia	6.68	1	yes
F32	Depressive episodes	F00.F99	Denmark	62.62	1	yes
F32	Depressive episodes	F00.F99	Finland	15.63	1	yes
F32	Depressive episodes	F00.F99	France	1001.84	1	yes
F32	Depressive episodes	F00.F99	Germany	111.10	2	yes
F32	Depressive episodes	F00.F99	Netherlands	42.05	2	yes
F32	Depressive episodes	F00.F99	Norway	31.47	2	yes
F32	Depressive episodes	F00.F99	Spain	63.85	3	yes

For peer review only

F32	Depressive episodes	F00.F99	Sweden	61.83	0	no
F32	Depressive episodes	F00.F99	Switzerland	99.47	2	yes
F32	Depressive episodes	F00.F99	United Kingdom	159.11	1	yes
F33	Recurrent depressive disorder	F00.F99	Belgium	6.07	0	no
F33	Recurrent depressive disorder	F00.F99	Denmark	6.00	2	yes
F33	Recurrent depressive disorder	F00.F99	France	72.52	1	yes
F33	Recurrent depressive disorder	F00.F99	Germany	8.84	2	yes
F33	Recurrent depressive disorder	F00.F99	Spain	6.63	1	yes
F33	Recurrent depressive disorder	F00.F99	Switzerland	9.19	1	yes
F33	Recurrent depressive disorder	F00.F99	United Kingdom	5.96	1	yes
F34	Persistent mood [affective] disorders	F00.F99	France	9.61	1	yes
F41	Other anxiety disorders	F00.F99	France	149.54	1	yes
F41	Other anxiety disorders	F00.F99	Spain	6.85	0	no
F41	Other anxiety disorders	F00.F99	Sweden	5.16	1	yes
F41	Other anxiety disorders	F00.F99	Switzerland	6.63	1	yes
F42	Obsessive-compulsive disorder	F00.F99	France	5.55	2	yes
F43	Reaction to severe stress, and adjustment disorders	F00.F99	France	54.03	0	no
F44	Dissociative [conversion] disorders	F00.F99	Belgium	10.82	3	yes
F44	Dissociative [conversion] disorders	F00.F99	France	139.91	2	yes
F44	Dissociative [conversion] disorders	F00.F99	Spain	34.04	2	yes
F48	Other neurotic disorders	F00.F99	France	10.06	0	no
F50	Eating disorders	F00.F99	Austria	6.44	0	no
F50	Eating disorders	F00.F99	Belgium	49.19	2	yes
F50	Eating disorders	F00.F99	France	36.19	1	yes
F50	Eating disorders	F00.F99	Germany	66.20	2	yes
F50	Eating disorders	F00.F99	Netherlands	12.08	0	no
F50	Eating disorders	F00.F99	Poland	6.58	0	no
F50	Eating disorders	F00.F99	Spain	7.47	0	no
F50	Eating disorders	F00.F99	Sweden	11.75	0	no
F50	Eating disorders	F00.F99	Switzerland	11.28	1	yes
F50	Eating disorders	F00.F99	United Kingdom	24.82	0	no
F54	Psychological and behavioural factors associated with disorders or diseases classified elsewhere	F00.F99	Belgium	5.36	3	yes
F54	Psychological and behavioural factors associated with disorders or diseases classified elsewhere	F00.F99	France	53.75	2	yes
F55	Abuse of non-dependence-producing substances	F00.F99	Belgium	5.50	3	yes
F55	Abuse of non-dependence-producing substances	F00.F99	Germany	9.87	0	no
F60	Specific personality disorders	F00.F99	Netherlands	5.07	0	no
F71	MModerate mental retardation	F00.F99	Finland	6.63	1	yes
F72	MSevere mental retardation	F00.F99	Finland	10.95	1	yes
F72	MSevere mental retardation	F00.F99	Spain	6.55	2	yes
F73	MProfound mental retardation	F00.F99	Finland	13.24	1	yes
F73	MProfound mental retardation	F00.F99	France	27.85	1	yes
F73	MProfound mental retardation	F00.F99	Spain	15.07	0	no
F73	MProfound mental retardation	F00.F99	Sweden	6.96	0	no
F79	MUnspecified mental retardation	F00.F99	Belgium	16.39	0	no
F79	MUnspecified mental retardation	F00.F99	Denmark	10.59	1	yes
F79	MUnspecified mental retardation	F00.F99	France	111.15	0	no
F79	MUnspecified mental retardation	F00.F99	Germany	7.56	3	yes
F79	MUnspecified mental retardation	F00.F99	Netherlands	41.62	1	yes
F79	MUnspecified mental retardation	F00.F99	Norway	9.18	2	yes

For peer review only

F79	MUnspecified mental retardation	F00.F99	Spain	40.14	0	no
F79	MUnspecified mental retardation	F00.F99	Sweden	15.61	0	no
F79	MUnspecified mental retardation	F00.F99	Switzerland	11.54	0	no
F81	Specific developmental disorders of scholastic skills	F00.F99	United Kingdom	22.30	1	yes
F84	Pervasive developmental disorders	F00.F99	France	28.28	1	yes
F84	Pervasive developmental disorders	F00.F99	Germany	6.48	1	yes
F84	Pervasive developmental disorders	F00.F99	United Kingdom	7.85	2	yes
F99	Mental disorder, not otherwise specified	F00.F99	Belgium	11.95	2	yes
F99	Mental disorder, not otherwise specified	F00.F99	Denmark	5.53	0	no
F99	Mental disorder, not otherwise specified	F00.F99	France	71.15	0	no
F99	Mental disorder, not otherwise specified	F00.F99	Netherlands	14.30	1	yes
F99	Mental disorder, not otherwise specified	F00.F99	Spain	6.45	1	yes
G00	Bacterial meningitis, not elsewhere classified	G00.G99	Austria	12.41	1	yes
G00	Bacterial meningitis, not elsewhere classified	G00.G99	Belgium	18.30	1	yes
G00	Bacterial meningitis, not elsewhere classified	G00.G99	Croatia	16.68	0	no
G00	Bacterial meningitis, not elsewhere classified	G00.G99	Czech Republic	34.39	1	yes
G00	Bacterial meningitis, not elsewhere classified	G00.G99	Denmark	24.17	0	no
G00	Bacterial meningitis, not elsewhere classified	G00.G99	Estonia	9.81	1	yes
G00	Bacterial meningitis, not elsewhere classified	G00.G99	Finland	13.30	1	yes
G00	Bacterial meningitis, not elsewhere classified	G00.G99	France	114.34	1	yes
G00	Bacterial meningitis, not elsewhere classified	G00.G99	Germany	96.28	1	yes
G00	Bacterial meningitis, not elsewhere classified	G00.G99	Hungary	49.61	3	yes
G00	Bacterial meningitis, not elsewhere classified	G00.G99	Latvia	7.58	0	no
G00	Bacterial meningitis, not elsewhere classified	G00.G99	Lithuania	16.26	3	yes
G00	Bacterial meningitis, not elsewhere classified	G00.G99	Netherlands	44.96	1	yes
G00	Bacterial meningitis, not elsewhere classified	G00.G99	Norway	12.71	1	yes
G00	Bacterial meningitis, not elsewhere classified	G00.G99	Poland	108.46	0	no
G00	Bacterial meningitis, not elsewhere classified	G00.G99	Romania	73.85	1	yes
G00	Bacterial meningitis, not elsewhere classified	G00.G99	Slovenia	6.42	1	yes
G00	Bacterial meningitis, not elsewhere classified	G00.G99	Spain	99.42	3	yes
G00	Bacterial meningitis, not elsewhere classified	G00.G99	Sweden	14.82	1	yes
G00	Bacterial meningitis, not elsewhere classified	G00.G99	Switzerland	12.45	1	yes
G00	Bacterial meningitis, not elsewhere classified	G00.G99	United Kingdom	113.35	1	yes
G03	Meningitis due to other and unspecified causes	G00.G99	Austria	11.73	0	no
G03	Meningitis due to other and unspecified causes	G00.G99	Belgium	13.19	0	no
G03	Meningitis due to other and unspecified causes	G00.G99	Czech Republic	7.98	1	yes
G03	Meningitis due to other and unspecified causes	G00.G99	Denmark	6.48	1	yes
G03	Meningitis due to other and unspecified causes	G00.G99	France	50.30	0	no
G03	Meningitis due to other and unspecified causes	G00.G99	Germany	73.23	1	yes
G03	Meningitis due to other and unspecified causes	G00.G99	Hungary	22.27	2	yes
G03	Meningitis due to other and unspecified causes	G00.G99	Latvia	5.09	0	no
G03	Meningitis due to other and unspecified causes	G00.G99	Netherlands	40.46	0	no
G03	Meningitis due to other and unspecified causes	G00.G99	Norway	6.96	1	yes
G03	Meningitis due to other and unspecified causes	G00.G99	Poland	40.81	0	no
G03	Meningitis due to other and unspecified causes	G00.G99	Romania	24.52	1	yes
G03	Meningitis due to other and unspecified causes	G00.G99	Spain	57.22	3	yes
G03	Meningitis due to other and unspecified causes	G00.G99	Sweden	7.98	0	no
G03	Meningitis due to other and unspecified causes	G00.G99	United Kingdom	85.11	1	yes
G04	Encephalitis, myelitis and encephalomyelitis	G00.G99	Austria	21.49	2	yes
G04	Encephalitis, myelitis and encephalomyelitis	G00.G99	Belgium	26.54	2	yes

For peer review only

G04	Encephalitis, myelitis and encephalomyelitis	G00.G99	Croatia	11.96	1	yes
G04	Encephalitis, myelitis and encephalomyelitis	G00.G99	Czech Republic	13.35	1	yes
G04	Encephalitis, myelitis and encephalomyelitis	G00.G99	Denmark	5.84	0	no
G04	Encephalitis, myelitis and encephalomyelitis	G00.G99	Estonia	5.49	1	yes
G04	Encephalitis, myelitis and encephalomyelitis	G00.G99	France	128.44	0	no
G04	Encephalitis, myelitis and encephalomyelitis	G00.G99	Germany	178.79	1	yes
G04	Encephalitis, myelitis and encephalomyelitis	G00.G99	Hungary	11.81	2	yes
G04	Encephalitis, myelitis and encephalomyelitis	G00.G99	Latvia	17.86	3	yes
G04	Encephalitis, myelitis and encephalomyelitis	G00.G99	Lithuania	22.67	2	yes
G04	Encephalitis, myelitis and encephalomyelitis	G00.G99	Netherlands	22.30	1	yes
G04	Encephalitis, myelitis and encephalomyelitis	G00.G99	Norway	6.35	0	no
G04	Encephalitis, myelitis and encephalomyelitis	G00.G99	Poland	102.78	1	yes
G04	Encephalitis, myelitis and encephalomyelitis	G00.G99	Romania	113.76	2	yes
G04	Encephalitis, myelitis and encephalomyelitis	G00.G99	Spain	98.48	0	no
G04	Encephalitis, myelitis and encephalomyelitis	G00.G99	Sweden	7.80	0	no
G04	Encephalitis, myelitis and encephalomyelitis	G00.G99	Switzerland	14.08	0	no
G04	Encephalitis, myelitis and encephalomyelitis	G00.G99	United Kingdom	107.27	2	yes
G06	Intracranial and intraspinal abscess and granuloma	G00.G99	Belgium	9.18	0	no
G06	Intracranial and intraspinal abscess and granuloma	G00.G99	Czech Republic	10.01	0	no
G06	Intracranial and intraspinal abscess and granuloma	G00.G99	Denmark	6.48	0	no
G06	Intracranial and intraspinal abscess and granuloma	G00.G99	France	44.40	0	no
G06	Intracranial and intraspinal abscess and granuloma	G00.G99	Germany	47.05	2	yes
G06	Intracranial and intraspinal abscess and granuloma	G00.G99	Hungary	8.95	0	no
G06	Intracranial and intraspinal abscess and granuloma	G00.G99	Netherlands	9.30	0	no
G06	Intracranial and intraspinal abscess and granuloma	G00.G99	Poland	27.99	0	no
G06	Intracranial and intraspinal abscess and granuloma	G00.G99	Romania	14.81	0	no
G06	Intracranial and intraspinal abscess and granuloma	G00.G99	Spain	34.37	2	yes
G06	Intracranial and intraspinal abscess and granuloma	G00.G99	Sweden	5.69	0	no
G06	Intracranial and intraspinal abscess and granuloma	G00.G99	Switzerland	5.82	0	no
G06	Intracranial and intraspinal abscess and granuloma	G00.G99	United Kingdom	56.79	3	yes
G08	Intracranial and intraspinal phlebitis and thrombophlebitis	G00.G99	France	23.38	1	yes
G08	Intracranial and intraspinal phlebitis and thrombophlebitis	G00.G99	Germany	21.67	1	yes
G08	Intracranial and intraspinal phlebitis and thrombophlebitis	G00.G99	United Kingdom	15.35	2	yes
G09	Sequelae of inflammatory diseases of central nervous system	G00.G99	Finland	8.03	0	no
G09	Sequelae of inflammatory diseases of central nervous system	G00.G99	France	30.77	0	no
G09	Sequelae of inflammatory diseases of central nervous system	G00.G99	Germany	17.08	2	yes
G09	Sequelae of inflammatory diseases of central nervous system	G00.G99	Netherlands	7.08	0	no
G09	Sequelae of inflammatory diseases of central nervous system	G00.G99	Poland	7.80	0	no
G09	Sequelae of inflammatory diseases of central nervous system	G00.G99	Spain	15.59	3	yes
G09	Sequelae of inflammatory diseases of central nervous system	G00.G99	Switzerland	5.08	0	no
G09	Sequelae of inflammatory diseases of central nervous system	G00.G99	United Kingdom	14.22	1	yes
G10	Huntington's disease	G00.G99	Austria	22.99	1	yes
G10	Huntington's disease	G00.G99	Belgium	36.35	1	yes

For peer review only

G10	Huntington's disease	G00.G99	Croatia	6.70	1	yes
G10	Huntington's disease	G00.G99	Czech Republic	17.50	2	yes
G10	Huntington's disease	G00.G99	Denmark	18.41	0	no
G10	Huntington's disease	G00.G99	Finland	6.73	0	no
G10	Huntington's disease	G00.G99	France	163.32	1	yes
G10	Huntington's disease	G00.G99	Germany	247.56	2	yes
G10	Huntington's disease	G00.G99	Hungary	11.40	0	no
G10	Huntington's disease	G00.G99	Lithuania	5.49	0	no
G10	Huntington's disease	G00.G99	Netherlands	55.95	0	no
G10	Huntington's disease	G00.G99	Norway	18.75	0	no
G10	Huntington's disease	G00.G99	Poland	58.26	1	yes
G10	Huntington's disease	G00.G99	Romania	6.51	0	no
G10	Huntington's disease	G00.G99	Spain	86.86	1	yes
G10	Huntington's disease	G00.G99	Sweden	27.58	0	no
G10	Huntington's disease	G00.G99	Switzerland	16.00	1	yes
G10	Huntington's disease	G00.G99	United Kingdom	248.01	1	yes
G11	Hereditary ataxia	G00.G99	Austria	5.69	0	no
G11	Hereditary ataxia	G00.G99	Belgium	11.39	0	no
G11	Hereditary ataxia	G00.G99	Denmark	7.92	0	no
G11	Hereditary ataxia	G00.G99	Finland	10.68	1	yes
G11	Hereditary ataxia	G00.G99	France	59.40	0	no
G11	Hereditary ataxia	G00.G99	Germany	57.42	2	yes
G11	Hereditary ataxia	G00.G99	Netherlands	28.69	3	yes
G11	Hereditary ataxia	G00.G99	Poland	6.48	0	no
G11	Hereditary ataxia	G00.G99	Romania	6.05	1	yes
G11	Hereditary ataxia	G00.G99	Spain	50.53	2	yes
G11	Hereditary ataxia	G00.G99	Sweden	9.36	0	no
G11	Hereditary ataxia	G00.G99	Switzerland	10.01	0	no
G11	Hereditary ataxia	G00.G99	United Kingdom	75.35	1	yes
G12	Spinal muscular atrophy and related syndromes	G00.G99	Austria	174.55	1	yes
G12	Spinal muscular atrophy and related syndromes	G00.G99	Belgium	287.30	1	yes
G12	Spinal muscular atrophy and related syndromes	G00.G99	Croatia	42.78	2	yes
G12	Spinal muscular atrophy and related syndromes	G00.G99	Czech Republic	84.61	2	yes
G12	Spinal muscular atrophy and related syndromes	G00.G99	Denmark	148.15	0	no
G12	Spinal muscular atrophy and related syndromes	G00.G99	Estonia	19.20	1	yes
G12	Spinal muscular atrophy and related syndromes	G00.G99	Finland	201.52	0	no
G12	Spinal muscular atrophy and related syndromes	G00.G99	France	1628.65	1	yes
G12	Spinal muscular atrophy and related syndromes	G00.G99	Germany	1724.59	1	yes
G12	Spinal muscular atrophy and related syndromes	G00.G99	Hungary	87.74	2	yes
G12	Spinal muscular atrophy and related syndromes	G00.G99	Latvia	20.79	0	no
G12	Spinal muscular atrophy and related syndromes	G00.G99	Lithuania	39.37	1	yes
G12	Spinal muscular atrophy and related syndromes	G00.G99	Netherlands	508.62	0	no
G12	Spinal muscular atrophy and related syndromes	G00.G99	Norway	142.35	0	no
G12	Spinal muscular atrophy and related syndromes	G00.G99	Poland	394.10	2	yes
G12	Spinal muscular atrophy and related syndromes	G00.G99	Romania	69.49	1	yes
G12	Spinal muscular atrophy and related syndromes	G00.G99	Slovenia	37.24	0	no
G12	Spinal muscular atrophy and related syndromes	G00.G99	Spain	1013.99	3	yes
G12	Spinal muscular atrophy and related syndromes	G00.G99	Sweden	308.07	2	yes
G12	Spinal muscular atrophy and related syndromes	G00.G99	Switzerland	191.83	1	yes
G12	Spinal muscular atrophy and related syndromes	G00.G99	United Kingdom	2285.12	3	yes

For peer review only

G20	Parkinson's disease	G00.G99	Austria	915.81	3	yes
G20	Parkinson's disease	G00.G99	Belgium	847.03	1	yes
G20	Parkinson's disease	G00.G99	Croatia	270.80	2	yes
G20	Parkinson's disease	G00.G99	Czech Republic	281.71	0	no
G20	Parkinson's disease	G00.G99	Denmark	391.63	2	yes
G20	Parkinson's disease	G00.G99	Estonia	51.67	1	yes
G20	Parkinson's disease	G00.G99	Finland	553.13	2	yes
G20	Parkinson's disease	G00.G99	France	4895.80	0	no
G20	Parkinson's disease	G00.G99	Germany	6805.50	2	yes
G20	Parkinson's disease	G00.G99	Hungary	307.64	1	yes
G20	Parkinson's disease	G00.G99	Latvia	47.41	3	yes
G20	Parkinson's disease	G00.G99	Lithuania	99.23	2	yes
G20	Parkinson's disease	G00.G99	Netherlands	1534.62	1	yes
G20	Parkinson's disease	G00.G99	Norway	350.83	2	yes
G20	Parkinson's disease	G00.G99	Poland	1038.84	2	yes
G20	Parkinson's disease	G00.G99	Romania	511.51	1	yes
G20	Parkinson's disease	G00.G99	Slovenia	84.56	0	no
G20	Parkinson's disease	G00.G99	Spain	3070.99	1	yes
G20	Parkinson's disease	G00.G99	Sweden	508.36	3	yes
G20	Parkinson's disease	G00.G99	Switzerland	672.24	0	no
G20	Parkinson's disease	G00.G99	United Kingdom	5577.69	0	no
G21	Secondary parkinsonism	G00.G99	Belgium	6.30	0	no
G21	Secondary parkinsonism	G00.G99	Czech Republic	32.18	2	yes
G21	Secondary parkinsonism	G00.G99	Denmark	7.35	2	yes
G21	Secondary parkinsonism	G00.G99	France	26.66	0	no
G21	Secondary parkinsonism	G00.G99	Germany	48.59	1	yes
G21	Secondary parkinsonism	G00.G99	Netherlands	13.54	2	yes
G21	Secondary parkinsonism	G00.G99	Poland	27.92	2	yes
G21	Secondary parkinsonism	G00.G99	Romania	13.45	3	yes
G21	Secondary parkinsonism	G00.G99	Spain	53.24	2	yes
G21	Secondary parkinsonism	G00.G99	Switzerland	31.10	3	yes
G21	Secondary parkinsonism	G00.G99	United Kingdom	24.89	3	yes
G23	Other degenerative diseases of the basal ganglia	G00.G99	Austria	10.04	1	yes
G23	Other degenerative diseases of the basal ganglia	G00.G99	Belgium	35.09	0	no
G23	Other degenerative diseases of the basal ganglia	G00.G99	Czech Republic	5.02	1	yes
G23	Other degenerative diseases of the basal ganglia	G00.G99	Denmark	6.62	0	no
G23	Other degenerative diseases of the basal ganglia	G00.G99	Finland	40.01	2	yes
G23	Other degenerative diseases of the basal ganglia	G00.G99	France	356.21	1	yes
G23	Other degenerative diseases of the basal ganglia	G00.G99	Germany	101.20	2	yes
G23	Other degenerative diseases of the basal ganglia	G00.G99	Netherlands	34.59	3	yes
G23	Other degenerative diseases of the basal ganglia	G00.G99	Norway	10.34	2	yes
G23	Other degenerative diseases of the basal ganglia	G00.G99	Poland	12.25	0	no
G23	Other degenerative diseases of the basal ganglia	G00.G99	Spain	94.72	2	yes
G23	Other degenerative diseases of the basal ganglia	G00.G99	Sweden	29.64	3	yes
G23	Other degenerative diseases of the basal ganglia	G00.G99	Switzerland	31.24	0	no
G23	Other degenerative diseases of the basal ganglia	G00.G99	United Kingdom	75.15	3	yes
G24	Dystonia	G00.G99	France	11.22	0	no
G24	Dystonia	G00.G99	Germany	5.34	1	yes
G24	Dystonia	G00.G99	United Kingdom	12.17	0	no
G25	Other extrapyramidal and movement disorders	G00.G99	Belgium	9.45	0	no

For peer review only

G25	Other extrapyramidal and movement disorders	G00.G99	Finland	8.67	0	no
G25	Other extrapyramidal and movement disorders	G00.G99	France	97.63	1	yes
G25	Other extrapyramidal and movement disorders	G00.G99	Germany	15.08	2	yes
G25	Other extrapyramidal and movement disorders	G00.G99	Poland	6.78	0	no
G25	Other extrapyramidal and movement disorders	G00.G99	Spain	19.53	1	yes
G25	Other extrapyramidal and movement disorders	G00.G99	Switzerland	15.82	2	yes
G25	Other extrapyramidal and movement disorders	G00.G99	United Kingdom	13.82	0	no
G30	Alzheimer's disease	G00.G99	Austria	932.64	3	yes
G30	Alzheimer's disease	G00.G99	Belgium	2088.76	2	yes
G30	Alzheimer's disease	G00.G99	Croatia	245.22	1	yes
G30	Alzheimer's disease	G00.G99	Czech Republic	1187.20	2	yes
G30	Alzheimer's disease	G00.G99	Denmark	866.86	1	yes
G30	Alzheimer's disease	G00.G99	Estonia	54.35	1	yes
G30	Alzheimer's disease	G00.G99	Finland	3776.32	3	yes
G30	Alzheimer's disease	G00.G99	France	15870.84	2	yes
G30	Alzheimer's disease	G00.G99	Germany	5656.99	2	yes
G30	Alzheimer's disease	G00.G99	Hungary	322.65	1	yes
G30	Alzheimer's disease	G00.G99	Latvia	41.46	2	yes
G30	Alzheimer's disease	G00.G99	Lithuania	130.17	1	yes
G30	Alzheimer's disease	G00.G99	Netherlands	2254.73	2	yes
G30	Alzheimer's disease	G00.G99	Norway	737.85	2	yes
G30	Alzheimer's disease	G00.G99	Poland	2573.09	2	yes
G30	Alzheimer's disease	G00.G99	Romania	1335.91	2	yes
G30	Alzheimer's disease	G00.G99	Slovenia	98.17	2	yes
G30	Alzheimer's disease	G00.G99	Spain	11437.72	3	yes
G30	Alzheimer's disease	G00.G99	Sweden	1692.67	1	yes
G30	Alzheimer's disease	G00.G99	Switzerland	1585.05	0	no
G30	Alzheimer's disease	G00.G99	United Kingdom	9044.26	3	yes
G31	Other degenerative diseases of nervous system, not elsewhere classified	G00.G99	Austria	34.79	2	yes
G31	Other degenerative diseases of nervous system, not elsewhere classified	G00.G99	Belgium	184.78	2	yes
G31	Other degenerative diseases of nervous system, not elsewhere classified	G00.G99	Croatia	20.90	1	yes
G31	Other degenerative diseases of nervous system, not elsewhere classified	G00.G99	Czech Republic	62.19	2	yes
G31	Other degenerative diseases of nervous system, not elsewhere classified	G00.G99	Denmark	37.14	1	yes
G31	Other degenerative diseases of nervous system, not elsewhere classified	G00.G99	Estonia	35.52	1	yes
G31	Other degenerative diseases of nervous system, not elsewhere classified	G00.G99	Finland	237.62	1	yes
G31	Other degenerative diseases of nervous system, not elsewhere classified	G00.G99	France	1516.18	2	yes
G31	Other degenerative diseases of nervous system, not elsewhere classified	G00.G99	Germany	501.35	2	yes
G31	Other degenerative diseases of nervous system, not elsewhere classified	G00.G99	Hungary	580.59	1	yes
G31	Other degenerative diseases of nervous system, not elsewhere classified	G00.G99	Latvia	42.45	3	yes
G31	Other degenerative diseases of nervous system, not elsewhere classified	G00.G99	Lithuania	33.07	2	yes
G31	Other degenerative diseases of nervous system, not elsewhere classified	G00.G99	Netherlands	160.67	3	yes

For peer review only

G31	Other degenerative diseases of nervous system, not elsewhere classified	G00.G99	Norway	77.99	1	yes
G31	Other degenerative diseases of nervous system, not elsewhere classified	G00.G99	Poland	238.66	0	no
G31	Other degenerative diseases of nervous system, not elsewhere classified	G00.G99	Romania	37.60	1	yes
G31	Other degenerative diseases of nervous system, not elsewhere classified	G00.G99	Slovenia	5.89	1	yes
G31	Other degenerative diseases of nervous system, not elsewhere classified	G00.G99	Spain	741.15	1	yes
G31	Other degenerative diseases of nervous system, not elsewhere classified	G00.G99	Sweden	135.28	1	yes
G31	Other degenerative diseases of nervous system, not elsewhere classified	G00.G99	Switzerland	136.65	2	yes
G31	Other degenerative diseases of nervous system, not elsewhere classified	G00.G99	United Kingdom	882.58	2	yes
G35	Multiple sclerosis	G00.G99	Austria	108.60	1	yes
G35	Multiple sclerosis	G00.G99	Belgium	136.88	0	no
G35	Multiple sclerosis	G00.G99	Croatia	45.43	1	yes
G35	Multiple sclerosis	G00.G99	Czech Republic	107.71	0	no
G35	Multiple sclerosis	G00.G99	Denmark	130.67	0	no
G35	Multiple sclerosis	G00.G99	Estonia	13.76	0	no
G35	Multiple sclerosis	G00.G99	Finland	68.76	0	no
G35	Multiple sclerosis	G00.G99	France	625.70	0	no
G35	Multiple sclerosis	G00.G99	Germany	1063.72	2	yes
G35	Multiple sclerosis	G00.G99	Hungary	94.95	0	no
G35	Multiple sclerosis	G00.G99	Latvia	28.56	3	yes
G35	Multiple sclerosis	G00.G99	Lithuania	37.13	0	no
G35	Multiple sclerosis	G00.G99	Netherlands	221.42	0	no
G35	Multiple sclerosis	G00.G99	Norway	103.65	0	no
G35	Multiple sclerosis	G00.G99	Poland	468.94	1	yes
G35	Multiple sclerosis	G00.G99	Romania	112.47	1	yes
G35	Multiple sclerosis	G00.G99	Slovenia	27.14	1	yes
G35	Multiple sclerosis	G00.G99	Spain	224.51	0	no
G35	Multiple sclerosis	G00.G99	Sweden	133.55	2	yes
G35	Multiple sclerosis	G00.G99	Switzerland	135.58	0	no
G35	Multiple sclerosis	G00.G99	United Kingdom	1317.40	2	yes
G36	Other acute disseminated demyelination	G00.G99	Poland	5.50	0	no
G37	Other demyelinating diseases of central nervous system	G00.G99	Belgium	9.24	2	yes
G37	Other demyelinating diseases of central nervous system	G00.G99	Czech Republic	14.35	0	no
G37	Other demyelinating diseases of central nervous system	G00.G99	Denmark	5.91	0	no
G37	Other demyelinating diseases of central nervous system	G00.G99	France	23.89	0	no
G37	Other demyelinating diseases of central nervous system	G00.G99	Germany	21.30	0	no
G37	Other demyelinating diseases of central nervous system	G00.G99	Netherlands	8.96	3	yes
G37	Other demyelinating diseases of central nervous system	G00.G99	Poland	25.54	1	yes
G37	Other demyelinating diseases of central nervous system	G00.G99	Romania	5.33	0	no
G37	Other demyelinating diseases of central nervous system	G00.G99	Spain	28.22	0	no

For peer review only

G37	Other demyelinating diseases of central nervous system	G00.G99	United Kingdom	21.68	0	no
G40	Epilepsy	G00.G99	Austria	91.62	2	yes
G40	Epilepsy	G00.G99	Belgium	204.83	0	no
G40	Epilepsy	G00.G99	Croatia	66.89	1	yes
G40	Epilepsy	G00.G99	Czech Republic	136.74	3	yes
G40	Epilepsy	G00.G99	Denmark	96.72	0	no
G40	Epilepsy	G00.G99	Estonia	45.37	3	yes
G40	Epilepsy	G00.G99	Finland	79.84	0	no
G40	Epilepsy	G00.G99	France	1233.69	3	yes
G40	Epilepsy	G00.G99	Germany	1782.76	1	yes
G40	Epilepsy	G00.G99	Hungary	135.32	1	yes
G40	Epilepsy	G00.G99	Latvia	54.08	1	yes
G40	Epilepsy	G00.G99	Lithuania	52.51	2	yes
G40	Epilepsy	G00.G99	Netherlands	218.60	3	yes
G40	Epilepsy	G00.G99	Norway	82.61	0	no
G40	Epilepsy	G00.G99	Poland	530.09	1	yes
G40	Epilepsy	G00.G99	Romania	293.99	0	no
G40	Epilepsy	G00.G99	Slovenia	21.66	0	no
G40	Epilepsy	G00.G99	Spain	357.71	2	yes
G40	Epilepsy	G00.G99	Sweden	99.95	1	yes
G40	Epilepsy	G00.G99	Switzerland	92.26	0	no
G40	Epilepsy	G00.G99	United Kingdom	1094.10	2	yes
G41	Status epilepticus	G00.G99	Austria	18.99	2	yes
G41	Status epilepticus	G00.G99	Belgium	76.32	0	no
G41	Status epilepticus	G00.G99	Croatia	25.76	2	yes
G41	Status epilepticus	G00.G99	Czech Republic	19.42	2	yes
G41	Status epilepticus	G00.G99	Denmark	12.51	0	no
G41	Status epilepticus	G00.G99	Finland	18.61	0	no
G41	Status epilepticus	G00.G99	France	396.90	1	yes
G41	Status epilepticus	G00.G99	Germany	355.82	1	yes
G41	Status epilepticus	G00.G99	Hungary	5.40	2	yes
G41	Status epilepticus	G00.G99	Lithuania	37.12	1	yes
G41	Status epilepticus	G00.G99	Netherlands	55.70	1	yes
G41	Status epilepticus	G00.G99	Norway	7.87	0	no
G41	Status epilepticus	G00.G99	Poland	172.40	2	yes
G41	Status epilepticus	G00.G99	Romania	37.11	1	yes
G41	Status epilepticus	G00.G99	Slovenia	5.78	0	no
G41	Status epilepticus	G00.G99	Spain	101.57	3	yes
G41	Status epilepticus	G00.G99	Sweden	17.53	0	no
G41	Status epilepticus	G00.G99	Switzerland	17.60	2	yes
G41	Status epilepticus	G00.G99	United Kingdom	91.51	1	yes
G45	Transient cerebral ischaemic attacks and related syndromes	G00.G99	Austria	50.23	2	yes
G45	Transient cerebral ischaemic attacks and related syndromes	G00.G99	Belgium	25.25	1	yes
G45	Transient cerebral ischaemic attacks and related syndromes	G00.G99	Denmark	15.61	1	yes
G45	Transient cerebral ischaemic attacks and related syndromes	G00.G99	France	148.05	2	yes
G45	Transient cerebral ischaemic attacks and related syndromes	G00.G99	Germany	96.43	2	yes

For peer review only

G45	Transient cerebral ischaemic attacks and related syndromes	G00.G99	Netherlands	118.53	3	yes
G45	Transient cerebral ischaemic attacks and related syndromes	G00.G99	Norway	34.37	3	yes
G45	Transient cerebral ischaemic attacks and related syndromes	G00.G99	Romania	70.84	2	yes
G45	Transient cerebral ischaemic attacks and related syndromes	G00.G99	Spain	73.24	2	yes
G45	Transient cerebral ischaemic attacks and related syndromes	G00.G99	Sweden	8.59	2	yes
G45	Transient cerebral ischaemic attacks and related syndromes	G00.G99	United Kingdom	137.74	1	yes
G47	Sleep disorders	G00.G99	Belgium	10.39	0	no
G47	Sleep disorders	G00.G99	France	125.09	2	yes
G47	Sleep disorders	G00.G99	Germany	59.84	1	yes
G47	Sleep disorders	G00.G99	Netherlands	13.35	1	yes
G47	Sleep disorders	G00.G99	Spain	79.42	1	yes
G47	Sleep disorders	G00.G99	Switzerland	11.60	1	yes
G47	Sleep disorders	G00.G99	United Kingdom	40.45	2	yes
G52	Disorders of other cranial nerves	G00.G99	France	7.91	0	no
G58	Other mononeuropathies	G00.G99	United Kingdom	6.54	0	no
G60	Hereditary and idiopathic neuropathy	G00.G99	Belgium	6.40	0	no
G60	Hereditary and idiopathic neuropathy	G00.G99	France	25.74	0	no
G60	Hereditary and idiopathic neuropathy	G00.G99	Germany	11.51	0	no
G60	Hereditary and idiopathic neuropathy	G00.G99	Netherlands	5.87	0	no
G60	Hereditary and idiopathic neuropathy	G00.G99	Poland	5.98	2	yes
G60	Hereditary and idiopathic neuropathy	G00.G99	Spain	11.20	3	yes
G60	Hereditary and idiopathic neuropathy	G00.G99	United Kingdom	19.02	0	no
G61	Inflammatory polyneuropathy	G00.G99	Austria	8.22	0	no
G61	Inflammatory polyneuropathy	G00.G99	Belgium	13.13	0	no
G61	Inflammatory polyneuropathy	G00.G99	Croatia	6.02	0	no
G61	Inflammatory polyneuropathy	G00.G99	Czech Republic	8.10	3	yes
G61	Inflammatory polyneuropathy	G00.G99	France	34.82	0	no
G61	Inflammatory polyneuropathy	G00.G99	Germany	76.12	1	yes
G61	Inflammatory polyneuropathy	G00.G99	Hungary	13.31	0	no
G61	Inflammatory polyneuropathy	G00.G99	Netherlands	15.12	1	yes
G61	Inflammatory polyneuropathy	G00.G99	Poland	27.81	3	yes
G61	Inflammatory polyneuropathy	G00.G99	Spain	50.26	0	no
G61	Inflammatory polyneuropathy	G00.G99	Switzerland	8.20	1	yes
G61	Inflammatory polyneuropathy	G00.G99	United Kingdom	53.75	1	yes
G62	Other polyneuropathies	G00.G99	Austria	11.15	0	no
G62	Other polyneuropathies	G00.G99	Belgium	24.08	0	no
G62	Other polyneuropathies	G00.G99	Czech Republic	5.13	1	yes
G62	Other polyneuropathies	G00.G99	Denmark	13.73	0	no
G62	Other polyneuropathies	G00.G99	Finland	8.82	0	no
G62	Other polyneuropathies	G00.G99	France	97.55	0	no
G62	Other polyneuropathies	G00.G99	Germany	118.44	3	yes
G62	Other polyneuropathies	G00.G99	Hungary	33.11	0	no
G62	Other polyneuropathies	G00.G99	Lithuania	7.27	0	no
G62	Other polyneuropathies	G00.G99	Netherlands	29.50	1	yes
G62	Other polyneuropathies	G00.G99	Norway	8.26	2	yes
G62	Other polyneuropathies	G00.G99	Poland	27.66	0	no

For peer review only

G62	Other polyneuropathies	G00.G99	Spain	55.49	0	no
G62	Other polyneuropathies	G00.G99	Sweden	11.33	0	no
G62	Other polyneuropathies	G00.G99	Switzerland	14.86	1	yes
G62	Other polyneuropathies	G00.G99	United Kingdom	67.21	1	yes
G70	Myasthenia gravis and other myoneural disorders	G00.G99	Austria	19.54	0	no
G70	Myasthenia gravis and other myoneural disorders	G00.G99	Belgium	16.80	1	yes
G70	Myasthenia gravis and other myoneural disorders	G00.G99	Croatia	11.86	1	yes
G70	Myasthenia gravis and other myoneural disorders	G00.G99	Czech Republic	13.87	1	yes
G70	Myasthenia gravis and other myoneural disorders	G00.G99	Denmark	10.35	0	no
G70	Myasthenia gravis and other myoneural disorders	G00.G99	Finland	6.93	0	no
G70	Myasthenia gravis and other myoneural disorders	G00.G99	France	81.94	0	no
G70	Myasthenia gravis and other myoneural disorders	G00.G99	Germany	120.59	1	yes
G70	Myasthenia gravis and other myoneural disorders	G00.G99	Hungary	22.64	0	no
G70	Myasthenia gravis and other myoneural disorders	G00.G99	Netherlands	27.03	0	no
G70	Myasthenia gravis and other myoneural disorders	G00.G99	Norway	5.68	0	no
G70	Myasthenia gravis and other myoneural disorders	G00.G99	Poland	50.87	1	yes
G70	Myasthenia gravis and other myoneural disorders	G00.G99	Romania	17.94	0	no
G70	Myasthenia gravis and other myoneural disorders	G00.G99	Slovenia	5.07	0	no
G70	Myasthenia gravis and other myoneural disorders	G00.G99	Spain	102.64	1	yes
G70	Myasthenia gravis and other myoneural disorders	G00.G99	Sweden	15.78	1	yes
G70	Myasthenia gravis and other myoneural disorders	G00.G99	Switzerland	14.83	0	no
G70	Myasthenia gravis and other myoneural disorders	G00.G99	United Kingdom	119.68	1	yes
G71	Primary disorders of muscles	G00.G99	Austria	30.12	0	no
G71	Primary disorders of muscles	G00.G99	Belgium	41.40	0	no
G71	Primary disorders of muscles	G00.G99	Croatia	13.20	0	no
G71	Primary disorders of muscles	G00.G99	Czech Republic	14.84	1	yes
G71	Primary disorders of muscles	G00.G99	Denmark	20.81	1	yes
G71	Primary disorders of muscles	G00.G99	Finland	22.82	0	no
G71	Primary disorders of muscles	G00.G99	France	204.16	1	yes
G71	Primary disorders of muscles	G00.G99	Germany	252.09	2	yes
G71	Primary disorders of muscles	G00.G99	Hungary	25.98	0	no
G71	Primary disorders of muscles	G00.G99	Netherlands	97.48	1	yes
G71	Primary disorders of muscles	G00.G99	Norway	28.09	0	no
G71	Primary disorders of muscles	G00.G99	Poland	56.77	0	no
G71	Primary disorders of muscles	G00.G99	Romania	29.94	1	yes
G71	Primary disorders of muscles	G00.G99	Slovenia	8.13	0	no
G71	Primary disorders of muscles	G00.G99	Spain	225.71	1	yes
G71	Primary disorders of muscles	G00.G99	Sweden	44.43	1	yes
G71	Primary disorders of muscles	G00.G99	Switzerland	34.46	0	no
G71	Primary disorders of muscles	G00.G99	United Kingdom	281.65	0	no
G72	Other myopathies	G00.G99	Belgium	13.45	0	no
G72	Other myopathies	G00.G99	France	93.42	1	yes
G72	Other myopathies	G00.G99	Germany	17.84	1	yes
G72	Other myopathies	G00.G99	Netherlands	5.63	0	no
G72	Other myopathies	G00.G99	Poland	6.12	0	no
G72	Other myopathies	G00.G99	Spain	24.26	0	no
G72	Other myopathies	G00.G99	United Kingdom	28.20	1	yes
G80	Infantile cerebral palsy	G00.G99	Austria	18.54	1	yes
G80	Infantile cerebral palsy	G00.G99	Belgium	23.28	0	no
G80	Infantile cerebral palsy	G00.G99	Croatia	13.79	1	yes

For peer review only

G80	Infantile cerebral palsy	G00.G99	Czech Republic	30.41	1	yes
G80	Infantile cerebral palsy	G00.G99	Denmark	14.78	0	no
G80	Infantile cerebral palsy	G00.G99	Finland	17.18	2	yes
G80	Infantile cerebral palsy	G00.G99	France	208.86	1	yes
G80	Infantile cerebral palsy	G00.G99	Germany	122.67	1	yes
G80	Infantile cerebral palsy	G00.G99	Hungary	28.80	0	no
G80	Infantile cerebral palsy	G00.G99	Latvia	7.06	0	no
G80	Infantile cerebral palsy	G00.G99	Lithuania	20.53	1	yes
G80	Infantile cerebral palsy	G00.G99	Netherlands	5.97	2	yes
G80	Infantile cerebral palsy	G00.G99	Norway	21.95	1	yes
G80	Infantile cerebral palsy	G00.G99	Poland	97.09	1	yes
G80	Infantile cerebral palsy	G00.G99	Romania	5.94	0	no
G80	Infantile cerebral palsy	G00.G99	Slovenia	9.20	1	yes
G80	Infantile cerebral palsy	G00.G99	Spain	180.60	0	no
G80	Infantile cerebral palsy	G00.G99	Sweden	23.91	1	yes
G80	Infantile cerebral palsy	G00.G99	Switzerland	26.66	1	yes
G80	Infantile cerebral palsy	G00.G99	United Kingdom	269.89	3	yes
G81	Hemiplegia	G00.G99	Belgium	33.07	2	yes
G81	Hemiplegia	G00.G99	France	127.68	3	yes
G81	Hemiplegia	G00.G99	Germany	53.32	1	yes
G81	Hemiplegia	G00.G99	Hungary	16.10	0	no
G81	Hemiplegia	G00.G99	Poland	5.72	1	yes
G81	Hemiplegia	G00.G99	Spain	36.81	1	yes
G81	Hemiplegia	G00.G99	Switzerland	36.54	2	yes
G81	Hemiplegia	G00.G99	United Kingdom	89.13	2	yes
G82	Paraplegia and tetraplegia	G00.G99	Austria	11.23	0	no
G82	Paraplegia and tetraplegia	G00.G99	Belgium	19.85	2	yes
G82	Paraplegia and tetraplegia	G00.G99	Croatia	18.44	0	no
G82	Paraplegia and tetraplegia	G00.G99	Czech Republic	11.64	3	yes
G82	Paraplegia and tetraplegia	G00.G99	Denmark	20.16	1	yes
G82	Paraplegia and tetraplegia	G00.G99	France	166.37	1	yes
G82	Paraplegia and tetraplegia	G00.G99	Germany	191.26	3	yes
G82	Paraplegia and tetraplegia	G00.G99	Hungary	18.59	2	yes
G82	Paraplegia and tetraplegia	G00.G99	Netherlands	18.79	2	yes
G82	Paraplegia and tetraplegia	G00.G99	Norway	6.28	0	no
G82	Paraplegia and tetraplegia	G00.G99	Spain	72.55	0	no
G82	Paraplegia and tetraplegia	G00.G99	Sweden	8.20	0	no
G82	Paraplegia and tetraplegia	G00.G99	Switzerland	27.86	2	yes
G82	Paraplegia and tetraplegia	G00.G99	United Kingdom	135.53	2	yes
G83	Other paralytic syndromes	G00.G99	Denmark	8.18	3	yes
G83	Other paralytic syndromes	G00.G99	France	27.68	1	yes
G83	Other paralytic syndromes	G00.G99	Germany	20.06	0	no
G83	Other paralytic syndromes	G00.G99	Hungary	5.33	0	no
G83	Other paralytic syndromes	G00.G99	Netherlands	8.84	0	no
G83	Other paralytic syndromes	G00.G99	Poland	6.66	0	no
G83	Other paralytic syndromes	G00.G99	Spain	5.79	0	no
G83	Other paralytic syndromes	G00.G99	United Kingdom	8.46	0	no
G90	Disorders of autonomic nervous system	G00.G99	Belgium	15.32	2	yes
G90	Disorders of autonomic nervous system	G00.G99	Finland	17.15	1	yes
G90	Disorders of autonomic nervous system	G00.G99	France	149.82	3	yes

For peer review only

G90	Disorders of autonomic nervous system	G00.G99	Germany	121.71	2	yes
G90	Disorders of autonomic nervous system	G00.G99	Netherlands	78.48	1	yes
G90	Disorders of autonomic nervous system	G00.G99	Norway	9.44	2	yes
G90	Disorders of autonomic nervous system	G00.G99	Poland	21.22	1	yes
G90	Disorders of autonomic nervous system	G00.G99	Spain	22.88	3	yes
G90	Disorders of autonomic nervous system	G00.G99	Sweden	22.39	0	no
G90	Disorders of autonomic nervous system	G00.G99	Switzerland	20.31	3	yes
G90	Disorders of autonomic nervous system	G00.G99	United Kingdom	141.74	0	no
G91	Hydrocephalus	G00.G99	Austria	9.70	1	yes
G91	Hydrocephalus	G00.G99	Belgium	31.35	1	yes
G91	Hydrocephalus	G00.G99	Croatia	6.21	2	yes
G91	Hydrocephalus	G00.G99	Czech Republic	26.66	1	yes
G91	Hydrocephalus	G00.G99	Denmark	12.62	0	no
G91	Hydrocephalus	G00.G99	Finland	21.03	2	yes
G91	Hydrocephalus	G00.G99	France	196.66	1	yes
G91	Hydrocephalus	G00.G99	Germany	130.52	1	yes
G91	Hydrocephalus	G00.G99	Hungary	25.07	0	no
G91	Hydrocephalus	G00.G99	Lithuania	7.32	0	no
G91	Hydrocephalus	G00.G99	Netherlands	22.46	2	yes
G91	Hydrocephalus	G00.G99	Norway	10.25	0	no
G91	Hydrocephalus	G00.G99	Poland	35.36	2	yes
G91	Hydrocephalus	G00.G99	Romania	42.87	1	yes
G91	Hydrocephalus	G00.G99	Spain	139.42	2	yes
G91	Hydrocephalus	G00.G99	Sweden	14.58	0	no
G91	Hydrocephalus	G00.G99	Switzerland	24.81	2	yes
G91	Hydrocephalus	G00.G99	United Kingdom	87.79	1	yes
G92	Toxic encephalopathy	G00.G99	Czech Republic	7.04	1	yes
G92	Toxic encephalopathy	G00.G99	France	9.22	0	no
G92	Toxic encephalopathy	G00.G99	Hungary	6.89	2	yes
G92	Toxic encephalopathy	G00.G99	Latvia	10.94	3	yes
G92	Toxic encephalopathy	G00.G99	Lithuania	6.45	0	no
G92	Toxic encephalopathy	G00.G99	Poland	10.46	0	no
G92	Toxic encephalopathy	G00.G99	Romania	10.09	3	yes
G92	Toxic encephalopathy	G00.G99	Spain	6.65	3	yes
G93	Other disorders of brain	G00.G99	Austria	131.12	1	yes
G93	Other disorders of brain	G00.G99	Belgium	185.82	0	no
G93	Other disorders of brain	G00.G99	Croatia	10.17	0	no
G93	Other disorders of brain	G00.G99	Czech Republic	303.60	3	yes
G93	Other disorders of brain	G00.G99	Denmark	31.63	1	yes
G93	Other disorders of brain	G00.G99	Estonia	10.05	1	yes
G93	Other disorders of brain	G00.G99	Finland	11.10	0	no
G93	Other disorders of brain	G00.G99	France	1032.53	1	yes
G93	Other disorders of brain	G00.G99	Germany	1290.36	2	yes
G93	Other disorders of brain	G00.G99	Hungary	117.62	2	yes
G93	Other disorders of brain	G00.G99	Latvia	41.21	3	yes
G93	Other disorders of brain	G00.G99	Lithuania	11.48	0	no
G93	Other disorders of brain	G00.G99	Netherlands	72.38	1	yes
G93	Other disorders of brain	G00.G99	Norway	30.35	1	yes
G93	Other disorders of brain	G00.G99	Poland	90.46	3	yes
G93	Other disorders of brain	G00.G99	Romania	172.66	2	yes

For peer review only

G93	Other disorders of brain	G00.G99	Spain	780.64	3	yes
G93	Other disorders of brain	G00.G99	Sweden	67.93	1	yes
G93	Other disorders of brain	G00.G99	Switzerland	67.82	0	no
G93	Other disorders of brain	G00.G99	United Kingdom	308.38	0	no
G95	Other diseases of spinal cord	G00.G99	Austria	6.11	0	no
G95	Other diseases of spinal cord	G00.G99	Belgium	18.17	3	yes
G95	Other diseases of spinal cord	G00.G99	Czech Republic	11.08	0	no
G95	Other diseases of spinal cord	G00.G99	France	107.09	3	yes
G95	Other diseases of spinal cord	G00.G99	Germany	38.67	1	yes
G95	Other diseases of spinal cord	G00.G99	Hungary	11.28	0	no
G95	Other diseases of spinal cord	G00.G99	Lithuania	5.79	0	no
G95	Other diseases of spinal cord	G00.G99	Netherlands	60.17	0	no
G95	Other diseases of spinal cord	G00.G99	Poland	26.65	1	yes
G95	Other diseases of spinal cord	G00.G99	Romania	12.16	0	no
G95	Other diseases of spinal cord	G00.G99	Spain	85.76	0	no
G95	Other diseases of spinal cord	G00.G99	Switzerland	10.68	0	no
G95	Other diseases of spinal cord	G00.G99	United Kingdom	151.06	2	yes
G96	Other disorders of central nervous system	G00.G99	Czech Republic	7.59	0	no
G96	Other disorders of central nervous system	G00.G99	France	20.09	1	yes
G96	Other disorders of central nervous system	G00.G99	Germany	7.28	0	no
G96	Other disorders of central nervous system	G00.G99	Poland	22.87	1	yes
G96	Other disorders of central nervous system	G00.G99	Spain	7.63	3	yes
G96	Other disorders of central nervous system	G00.G99	United Kingdom	6.31	0	no
G98	Other disorders of nervous system, not elsewhere classified	G00.G99	Belgium	20.61	2	yes
G98	Other disorders of nervous system, not elsewhere classified	G00.G99	France	178.09	2	yes
G98	Other disorders of nervous system, not elsewhere classified	G00.G99	Germany	6.56	0	no
G98	Other disorders of nervous system, not elsewhere classified	G00.G99	Netherlands	31.35	1	yes
G98	Other disorders of nervous system, not elsewhere classified	G00.G99	Norway	6.00	1	yes
G98	Other disorders of nervous system, not elsewhere classified	G00.G99	Poland	8.67	1	yes
G98	Other disorders of nervous system, not elsewhere classified	G00.G99	Spain	22.04	3	yes
G98	Other disorders of nervous system, not elsewhere classified	G00.G99	Sweden	9.52	0	no
G98	Other disorders of nervous system, not elsewhere classified	G00.G99	Switzerland	9.88	0	no
G98	Other disorders of nervous system, not elsewhere classified	G00.G99	United Kingdom	12.33	0	no
H05	Disorders of orbit	H00.H59	United Kingdom	5.36	0	no
H60	Otitis externa	H60.H95	United Kingdom	5.23	1	yes
H66	Suppurative and unspecified otitis media	H60.H95	France	14.11	1	yes
H66	Suppurative and unspecified otitis media	H60.H95	Germany	8.38	0	no
H66	Suppurative and unspecified otitis media	H60.H95	Hungary	7.44	0	no
H66	Suppurative and unspecified otitis media	H60.H95	Netherlands	9.41	3	yes
H66	Suppurative and unspecified otitis media	H60.H95	United Kingdom	15.78	0	no
H70	Mastoiditis and related conditions	H60.H95	France	5.16	0	no
I00	Rheumatic fever without mention of heart involvement	I00.I99	Germany	6.53	1	yes
I01	Rheumatic fever with heart involvement	I00.I99	Germany	14.87	1	yes

For peer review only

I01	Rheumatic fever with heart involvement	I00.I99	Romania	5.72	0	no
I05	Rheumatic mitral valve diseases	I00.I99	Austria	14.00	2	yes
I05	Rheumatic mitral valve diseases	I00.I99	Belgium	164.64	1	yes
I05	Rheumatic mitral valve diseases	I00.I99	Croatia	9.32	3	yes
I05	Rheumatic mitral valve diseases	I00.I99	Czech Republic	87.73	0	no
I05	Rheumatic mitral valve diseases	I00.I99	Denmark	7.63	1	yes
I05	Rheumatic mitral valve diseases	I00.I99	Estonia	12.48	2	yes
I05	Rheumatic mitral valve diseases	I00.I99	Finland	15.52	1	yes
I05	Rheumatic mitral valve diseases	I00.I99	France	1226.19	1	yes
I05	Rheumatic mitral valve diseases	I00.I99	Germany	541.49	2	yes
I05	Rheumatic mitral valve diseases	I00.I99	Hungary	61.43	2	yes
I05	Rheumatic mitral valve diseases	I00.I99	Latvia	22.74	2	yes
I05	Rheumatic mitral valve diseases	I00.I99	Lithuania	41.68	1	yes
I05	Rheumatic mitral valve diseases	I00.I99	Netherlands	14.20	2	yes
I05	Rheumatic mitral valve diseases	I00.I99	Norway	14.71	3	yes
I05	Rheumatic mitral valve diseases	I00.I99	Poland	237.85	2	yes
I05	Rheumatic mitral valve diseases	I00.I99	Romania	277.55	2	yes
I05	Rheumatic mitral valve diseases	I00.I99	Slovenia	9.38	1	yes
I05	Rheumatic mitral valve diseases	I00.I99	Spain	766.49	1	yes
I05	Rheumatic mitral valve diseases	I00.I99	Sweden	37.72	1	yes
I05	Rheumatic mitral valve diseases	I00.I99	Switzerland	24.18	0	no
I05	Rheumatic mitral valve diseases	I00.I99	United Kingdom	912.58	2	yes
I06	Rheumatic aortic valve diseases	I00.I99	Croatia	7.30	3	yes
I06	Rheumatic aortic valve diseases	I00.I99	Czech Republic	69.88	0	no
I06	Rheumatic aortic valve diseases	I00.I99	Denmark	36.80	3	yes
I06	Rheumatic aortic valve diseases	I00.I99	Estonia	9.51	1	yes
I06	Rheumatic aortic valve diseases	I00.I99	France	11.19	0	no
I06	Rheumatic aortic valve diseases	I00.I99	Germany	89.18	2	yes
I06	Rheumatic aortic valve diseases	I00.I99	Hungary	40.23	2	yes
I06	Rheumatic aortic valve diseases	I00.I99	Latvia	10.41	1	yes
I06	Rheumatic aortic valve diseases	I00.I99	Lithuania	24.45	2	yes
I06	Rheumatic aortic valve diseases	I00.I99	Poland	170.02	2	yes
I06	Rheumatic aortic valve diseases	I00.I99	Romania	129.42	2	yes
I06	Rheumatic aortic valve diseases	I00.I99	Slovenia	7.11	0	no
I06	Rheumatic aortic valve diseases	I00.I99	Spain	19.59	0	no
I06	Rheumatic aortic valve diseases	I00.I99	United Kingdom	9.19	0	no
I07	Rheumatic tricuspid valve diseases	I00.I99	Belgium	17.92	1	yes
I07	Rheumatic tricuspid valve diseases	I00.I99	Czech Republic	10.98	2	yes
I07	Rheumatic tricuspid valve diseases	I00.I99	France	50.54	1	yes
I07	Rheumatic tricuspid valve diseases	I00.I99	Germany	260.40	2	yes
I07	Rheumatic tricuspid valve diseases	I00.I99	Poland	24.25	2	yes
I07	Rheumatic tricuspid valve diseases	I00.I99	Romania	6.16	0	no
I07	Rheumatic tricuspid valve diseases	I00.I99	Spain	69.07	1	yes
I07	Rheumatic tricuspid valve diseases	I00.I99	Sweden	17.83	1	yes
I07	Rheumatic tricuspid valve diseases	I00.I99	Switzerland	5.38	1	yes
I07	Rheumatic tricuspid valve diseases	I00.I99	United Kingdom	91.14	1	yes
I08	Multiple valve diseases	I00.I99	Austria	219.37	2	yes
I08	Multiple valve diseases	I00.I99	Belgium	130.05	1	yes
I08	Multiple valve diseases	I00.I99	Croatia	75.30	2	yes
I08	Multiple valve diseases	I00.I99	Czech Republic	123.35	2	yes

For peer review only

I08	Multiple valve diseases	I00.I99	Denmark	12.45	0	no
I08	Multiple valve diseases	I00.I99	Estonia	15.22	0	no
I08	Multiple valve diseases	I00.I99	Finland	46.68	1	yes
I08	Multiple valve diseases	I00.I99	France	439.42	0	no
I08	Multiple valve diseases	I00.I99	Germany	1729.42	3	yes
I08	Multiple valve diseases	I00.I99	Hungary	250.47	1	yes
I08	Multiple valve diseases	I00.I99	Latvia	27.83	1	yes
I08	Multiple valve diseases	I00.I99	Lithuania	67.29	0	no
I08	Multiple valve diseases	I00.I99	Netherlands	36.05	2	yes
I08	Multiple valve diseases	I00.I99	Norway	36.12	3	yes
I08	Multiple valve diseases	I00.I99	Poland	825.76	1	yes
I08	Multiple valve diseases	I00.I99	Romania	100.71	3	yes
I08	Multiple valve diseases	I00.I99	Slovenia	89.86	0	no
I08	Multiple valve diseases	I00.I99	Spain	865.40	1	yes
I08	Multiple valve diseases	I00.I99	Sweden	71.25	1	yes
I08	Multiple valve diseases	I00.I99	Switzerland	85.61	1	yes
I08	Multiple valve diseases	I00.I99	United Kingdom	243.66	0	no
I09	Other rheumatic heart diseases	I00.I99	Czech Republic	7.10	3	yes
I09	Other rheumatic heart diseases	I00.I99	Estonia	5.42	1	yes
I09	Other rheumatic heart diseases	I00.I99	France	19.74	1	yes
I09	Other rheumatic heart diseases	I00.I99	Germany	17.20	1	yes
I09	Other rheumatic heart diseases	I00.I99	Hungary	13.64	0	no
I09	Other rheumatic heart diseases	I00.I99	Latvia	15.94	0	no
I09	Other rheumatic heart diseases	I00.I99	Lithuania	15.45	3	yes
I09	Other rheumatic heart diseases	I00.I99	Netherlands	5.27	1	yes
I09	Other rheumatic heart diseases	I00.I99	Poland	6.65	1	yes
I09	Other rheumatic heart diseases	I00.I99	Romania	173.72	3	yes
I09	Other rheumatic heart diseases	I00.I99	Spain	78.76	0	no
I09	Other rheumatic heart diseases	I00.I99	United Kingdom	127.64	2	yes
I10	Essential (primary) hypertension	I00.I99	Austria	860.76	3	yes
I10	Essential (primary) hypertension	I00.I99	Belgium	363.78	2	yes
I10	Essential (primary) hypertension	I00.I99	Croatia	160.91	1	yes
I10	Essential (primary) hypertension	I00.I99	Czech Republic	1018.84	2	yes
I10	Essential (primary) hypertension	I00.I99	Denmark	505.92	3	yes
I10	Essential (primary) hypertension	I00.I99	Finland	51.63	0	no
I10	Essential (primary) hypertension	I00.I99	France	4263.26	1	yes
I10	Essential (primary) hypertension	I00.I99	Germany	7612.14	3	yes
I10	Essential (primary) hypertension	I00.I99	Hungary	2114.91	0	no
I10	Essential (primary) hypertension	I00.I99	Latvia	88.25	3	yes
I10	Essential (primary) hypertension	I00.I99	Lithuania	62.99	2	yes
I10	Essential (primary) hypertension	I00.I99	Netherlands	662.89	2	yes
I10	Essential (primary) hypertension	I00.I99	Norway	281.28	2	yes
I10	Essential (primary) hypertension	I00.I99	Poland	2436.29	3	yes
I10	Essential (primary) hypertension	I00.I99	Romania	19284.79	1	yes
I10	Essential (primary) hypertension	I00.I99	Slovenia	43.47	1	yes
I10	Essential (primary) hypertension	I00.I99	Spain	3489.33	2	yes
I10	Essential (primary) hypertension	I00.I99	Sweden	450.76	1	yes
I10	Essential (primary) hypertension	I00.I99	Switzerland	777.02	0	no
I10	Essential (primary) hypertension	I00.I99	United Kingdom	1116.31	1	yes
I11	Hypertensive heart disease	I00.I99	Austria	2145.76	2	yes

For peer review only

I11	Hypertensive heart disease	I00.I99	Belgium	468.94	3	yes
I11	Hypertensive heart disease	I00.I99	Croatia	1522.29	1	yes
I11	Hypertensive heart disease	I00.I99	Czech Republic	1397.25	3	yes
I11	Hypertensive heart disease	I00.I99	Denmark	216.18	0	no
I11	Hypertensive heart disease	I00.I99	Estonia	1449.37	3	yes
I11	Hypertensive heart disease	I00.I99	Finland	774.81	2	yes
I11	Hypertensive heart disease	I00.I99	France	3818.20	3	yes
I11	Hypertensive heart disease	I00.I99	Germany	20709.02	3	yes
I11	Hypertensive heart disease	I00.I99	Hungary	5079.32	3	yes
I11	Hypertensive heart disease	I00.I99	Latvia	644.70	2	yes
I11	Hypertensive heart disease	I00.I99	Lithuania	461.66	2	yes
I11	Hypertensive heart disease	I00.I99	Netherlands	553.82	2	yes
I11	Hypertensive heart disease	I00.I99	Norway	119.97	2	yes
I11	Hypertensive heart disease	I00.I99	Poland	4867.68	2	yes
I11	Hypertensive heart disease	I00.I99	Romania	17337.23	2	yes
I11	Hypertensive heart disease	I00.I99	Slovenia	704.71	0	no
I11	Hypertensive heart disease	I00.I99	Spain	4065.83	2	yes
I11	Hypertensive heart disease	I00.I99	Sweden	751.10	1	yes
I11	Hypertensive heart disease	I00.I99	Switzerland	1410.00	2	yes
I11	Hypertensive heart disease	I00.I99	United Kingdom	3348.88	3	yes
I12	Hypertensive renal disease	I00.I99	Austria	297.18	2	yes
I12	Hypertensive renal disease	I00.I99	Belgium	154.82	1	yes
I12	Hypertensive renal disease	I00.I99	Croatia	7.93	0	no
I12	Hypertensive renal disease	I00.I99	Czech Republic	113.28	1	yes
I12	Hypertensive renal disease	I00.I99	Denmark	71.25	0	no
I12	Hypertensive renal disease	I00.I99	Estonia	12.95	0	no
I12	Hypertensive renal disease	I00.I99	Finland	60.81	1	yes
I12	Hypertensive renal disease	I00.I99	France	770.75	2	yes
I12	Hypertensive renal disease	I00.I99	Germany	1523.02	1	yes
I12	Hypertensive renal disease	I00.I99	Hungary	358.91	2	yes
I12	Hypertensive renal disease	I00.I99	Latvia	39.65	1	yes
I12	Hypertensive renal disease	I00.I99	Lithuania	24.05	0	no
I12	Hypertensive renal disease	I00.I99	Netherlands	123.94	1	yes
I12	Hypertensive renal disease	I00.I99	Norway	73.14	1	yes
I12	Hypertensive renal disease	I00.I99	Poland	105.06	0	no
I12	Hypertensive renal disease	I00.I99	Romania	110.31	2	yes
I12	Hypertensive renal disease	I00.I99	Spain	1023.80	3	yes
I12	Hypertensive renal disease	I00.I99	Sweden	152.45	0	no
I12	Hypertensive renal disease	I00.I99	Switzerland	198.50	3	yes
I12	Hypertensive renal disease	I00.I99	United Kingdom	778.03	0	no
I13	Hypertensive heart and renal disease	I00.I99	Austria	299.81	3	yes
I13	Hypertensive heart and renal disease	I00.I99	Belgium	115.26	1	yes
I13	Hypertensive heart and renal disease	I00.I99	Czech Republic	129.56	1	yes
I13	Hypertensive heart and renal disease	I00.I99	Denmark	145.44	1	yes
I13	Hypertensive heart and renal disease	I00.I99	Estonia	187.78	2	yes
I13	Hypertensive heart and renal disease	I00.I99	Finland	103.36	2	yes
I13	Hypertensive heart and renal disease	I00.I99	France	543.52	3	yes
I13	Hypertensive heart and renal disease	I00.I99	Germany	3198.00	3	yes
I13	Hypertensive heart and renal disease	I00.I99	Hungary	455.65	1	yes
I13	Hypertensive heart and renal disease	I00.I99	Latvia	25.24	2	yes

For peer review only

I13	Hypertensive heart and renal disease	I00.I99	Lithuania	43.94	2	yes
I13	Hypertensive heart and renal disease	I00.I99	Netherlands	56.14	2	yes
I13	Hypertensive heart and renal disease	I00.I99	Norway	22.66	2	yes
I13	Hypertensive heart and renal disease	I00.I99	Poland	147.49	0	no
I13	Hypertensive heart and renal disease	I00.I99	Romania	18.42	3	yes
I13	Hypertensive heart and renal disease	I00.I99	Slovenia	29.19	3	yes
I13	Hypertensive heart and renal disease	I00.I99	Spain	561.08	2	yes
I13	Hypertensive heart and renal disease	I00.I99	Sweden	122.46	2	yes
I13	Hypertensive heart and renal disease	I00.I99	Switzerland	362.03	1	yes
I13	Hypertensive heart and renal disease	I00.I99	United Kingdom	339.16	3	yes
I20	Angina pectoris	I00.I99	Austria	130.97	3	yes
I20	Angina pectoris	I00.I99	Belgium	201.70	2	yes
I20	Angina pectoris	I00.I99	Croatia	37.00	3	yes
I20	Angina pectoris	I00.I99	Czech Republic	109.46	0	no
I20	Angina pectoris	I00.I99	Denmark	57.37	1	yes
I20	Angina pectoris	I00.I99	Finland	27.55	1	yes
I20	Angina pectoris	I00.I99	France	1181.53	3	yes
I20	Angina pectoris	I00.I99	Germany	294.42	3	yes
I20	Angina pectoris	I00.I99	Hungary	86.59	3	yes
I20	Angina pectoris	I00.I99	Latvia	47.45	0	no
I20	Angina pectoris	I00.I99	Lithuania	59.55	3	yes
I20	Angina pectoris	I00.I99	Netherlands	299.58	2	yes
I20	Angina pectoris	I00.I99	Norway	227.20	2	yes
I20	Angina pectoris	I00.I99	Poland	1117.33	3	yes
I20	Angina pectoris	I00.I99	Romania	517.57	2	yes
I20	Angina pectoris	I00.I99	Slovenia	13.96	1	yes
I20	Angina pectoris	I00.I99	Spain	378.77	3	yes
I20	Angina pectoris	I00.I99	Sweden	378.13	2	yes
I20	Angina pectoris	I00.I99	Switzerland	50.90	1	yes
I20	Angina pectoris	I00.I99	United Kingdom	135.21	1	yes
I21	Acute myocardial infarction	I00.I99	Austria	5428.97	2	yes
I21	Acute myocardial infarction	I00.I99	Belgium	6953.08	1	yes
I21	Acute myocardial infarction	I00.I99	Croatia	4553.48	3	yes
I21	Acute myocardial infarction	I00.I99	Czech Republic	9577.10	3	yes
I21	Acute myocardial infarction	I00.I99	Denmark	3288.88	2	yes
I21	Acute myocardial infarction	I00.I99	Estonia	595.22	3	yes
I21	Acute myocardial infarction	I00.I99	Finland	5767.39	3	yes
I21	Acute myocardial infarction	I00.I99	France	22533.97	3	yes
I21	Acute myocardial infarction	I00.I99	Germany	59621.37	3	yes
I21	Acute myocardial infarction	I00.I99	Hungary	9499.88	2	yes
I21	Acute myocardial infarction	I00.I99	Latvia	1473.12	2	yes
I21	Acute myocardial infarction	I00.I99	Lithuania	1440.42	3	yes
I21	Acute myocardial infarction	I00.I99	Netherlands	10579.16	2	yes
I21	Acute myocardial infarction	I00.I99	Norway	4233.75	1	yes
I21	Acute myocardial infarction	I00.I99	Poland	27758.72	1	yes
I21	Acute myocardial infarction	I00.I99	Romania	28379.62	3	yes
I21	Acute myocardial infarction	I00.I99	Slovenia	1056.55	2	yes
I21	Acute myocardial infarction	I00.I99	Spain	23596.48	3	yes
I21	Acute myocardial infarction	I00.I99	Sweden	8513.33	1	yes
I21	Acute myocardial infarction	I00.I99	Switzerland	3036.82	3	yes

For peer review only

I21	Acute myocardial infarction	I00.I99	United Kingdom	44576.19	3	yes
I22	Subsequent myocardial infarction	I00.I99	Austria	768.65	2	yes
I22	Subsequent myocardial infarction	I00.I99	Belgium	70.10	1	yes
I22	Subsequent myocardial infarction	I00.I99	Croatia	280.21	3	yes
I22	Subsequent myocardial infarction	I00.I99	Czech Republic	145.07	3	yes
I22	Subsequent myocardial infarction	I00.I99	Estonia	183.86	3	yes
I22	Subsequent myocardial infarction	I00.I99	France	54.97	1	yes
I22	Subsequent myocardial infarction	I00.I99	Germany	4152.43	1	yes
I22	Subsequent myocardial infarction	I00.I99	Hungary	562.33	2	yes
I22	Subsequent myocardial infarction	I00.I99	Latvia	91.26	3	yes
I22	Subsequent myocardial infarction	I00.I99	Lithuania	137.71	2	yes
I22	Subsequent myocardial infarction	I00.I99	Netherlands	43.28	3	yes
I22	Subsequent myocardial infarction	I00.I99	Poland	249.70	2	yes
I22	Subsequent myocardial infarction	I00.I99	Romania	83.47	0	no
I22	Subsequent myocardial infarction	I00.I99	Slovenia	93.73	2	yes
I22	Subsequent myocardial infarction	I00.I99	Spain	26.91	2	yes
I22	Subsequent myocardial infarction	I00.I99	Switzerland	63.70	1	yes
I24	Other acute ischaemic heart diseases	I00.I99	Austria	82.56	2	yes
I24	Other acute ischaemic heart diseases	I00.I99	Belgium	314.22	3	yes
I24	Other acute ischaemic heart diseases	I00.I99	Czech Republic	287.26	1	yes
I24	Other acute ischaemic heart diseases	I00.I99	Denmark	201.75	3	yes
I24	Other acute ischaemic heart diseases	I00.I99	Estonia	73.53	1	yes
I24	Other acute ischaemic heart diseases	I00.I99	Finland	9.45	1	yes
I24	Other acute ischaemic heart diseases	I00.I99	France	2625.54	2	yes
I24	Other acute ischaemic heart diseases	I00.I99	Germany	1097.26	1	yes
I24	Other acute ischaemic heart diseases	I00.I99	Hungary	376.86	2	yes
I24	Other acute ischaemic heart diseases	I00.I99	Latvia	165.06	3	yes
I24	Other acute ischaemic heart diseases	I00.I99	Lithuania	371.42	1	yes
I24	Other acute ischaemic heart diseases	I00.I99	Netherlands	241.18	2	yes
I24	Other acute ischaemic heart diseases	I00.I99	Norway	26.03	2	yes
I24	Other acute ischaemic heart diseases	I00.I99	Poland	1436.09	0	no
I24	Other acute ischaemic heart diseases	I00.I99	Romania	773.48	3	yes
I24	Other acute ischaemic heart diseases	I00.I99	Slovenia	5.42	1	yes
I24	Other acute ischaemic heart diseases	I00.I99	Spain	1385.64	2	yes
I24	Other acute ischaemic heart diseases	I00.I99	Sweden	99.61	2	yes
I24	Other acute ischaemic heart diseases	I00.I99	Switzerland	94.44	2	yes
I24	Other acute ischaemic heart diseases	I00.I99	United Kingdom	558.00	1	yes
I25	Chronic ischaemic heart disease	I00.I99	Austria	10704.52	2	yes
I25	Chronic ischaemic heart disease	I00.I99	Belgium	4344.90	1	yes
I25	Chronic ischaemic heart disease	I00.I99	Croatia	8948.68	0	no
I25	Chronic ischaemic heart disease	I00.I99	Czech Republic	24981.67	2	yes
I25	Chronic ischaemic heart disease	I00.I99	Denmark	3626.24	2	yes
I25	Chronic ischaemic heart disease	I00.I99	Estonia	5005.09	1	yes
I25	Chronic ischaemic heart disease	I00.I99	Finland	7822.66	2	yes
I25	Chronic ischaemic heart disease	I00.I99	France	16858.87	1	yes
I25	Chronic ischaemic heart disease	I00.I99	Germany	85468.65	3	yes
I25	Chronic ischaemic heart disease	I00.I99	Hungary	31532.50	3	yes
I25	Chronic ischaemic heart disease	I00.I99	Latvia	9150.87	1	yes
I25	Chronic ischaemic heart disease	I00.I99	Lithuania	17531.29	1	yes
I25	Chronic ischaemic heart disease	I00.I99	Netherlands	4485.07	2	yes

For peer review only

I25	Chronic ischaemic heart disease	I00.I99	Norway	2175.81	2	yes
I25	Chronic ischaemic heart disease	I00.I99	Poland	39223.27	2	yes
I25	Chronic ischaemic heart disease	I00.I99	Romania	50217.90	1	yes
I25	Chronic ischaemic heart disease	I00.I99	Slovenia	1558.94	1	yes
I25	Chronic ischaemic heart disease	I00.I99	Spain	16262.86	1	yes
I25	Chronic ischaemic heart disease	I00.I99	Sweden	7333.32	1	yes
I25	Chronic ischaemic heart disease	I00.I99	Switzerland	7136.77	2	yes
I25	Chronic ischaemic heart disease	I00.I99	United Kingdom	59798.45	3	yes
I26	Pulmonary embolism	I00.I99	Austria	304.90	2	yes
I26	Pulmonary embolism	I00.I99	Belgium	909.14	1	yes
I26	Pulmonary embolism	I00.I99	Croatia	463.27	3	yes
I26	Pulmonary embolism	I00.I99	Czech Republic	2021.35	2	yes
I26	Pulmonary embolism	I00.I99	Denmark	251.29	1	yes
I26	Pulmonary embolism	I00.I99	Estonia	52.83	0	no
I26	Pulmonary embolism	I00.I99	Finland	211.21	1	yes
I26	Pulmonary embolism	I00.I99	France	4770.77	3	yes
I26	Pulmonary embolism	I00.I99	Germany	7521.30	3	yes
I26	Pulmonary embolism	I00.I99	Hungary	336.65	3	yes
I26	Pulmonary embolism	I00.I99	Latvia	45.51	1	yes
I26	Pulmonary embolism	I00.I99	Lithuania	267.09	0	no
I26	Pulmonary embolism	I00.I99	Netherlands	584.07	3	yes
I26	Pulmonary embolism	I00.I99	Norway	158.13	1	yes
I26	Pulmonary embolism	I00.I99	Poland	3576.44	3	yes
I26	Pulmonary embolism	I00.I99	Romania	1060.17	2	yes
I26	Pulmonary embolism	I00.I99	Slovenia	103.19	1	yes
I26	Pulmonary embolism	I00.I99	Spain	2453.75	1	yes
I26	Pulmonary embolism	I00.I99	Sweden	471.96	2	yes
I26	Pulmonary embolism	I00.I99	Switzerland	376.08	2	yes
I26	Pulmonary embolism	I00.I99	United Kingdom	3523.93	3	yes
I27	Other pulmonary heart diseases	I00.I99	Austria	110.47	3	yes
I27	Other pulmonary heart diseases	I00.I99	Belgium	134.46	2	yes
I27	Other pulmonary heart diseases	I00.I99	Croatia	122.92	3	yes
I27	Other pulmonary heart diseases	I00.I99	Czech Republic	293.08	1	yes
I27	Other pulmonary heart diseases	I00.I99	Denmark	56.56	0	no
I27	Other pulmonary heart diseases	I00.I99	Estonia	8.15	2	yes
I27	Other pulmonary heart diseases	I00.I99	Finland	18.68	1	yes
I27	Other pulmonary heart diseases	I00.I99	France	952.68	1	yes
I27	Other pulmonary heart diseases	I00.I99	Germany	1861.30	2	yes
I27	Other pulmonary heart diseases	I00.I99	Hungary	381.71	1	yes
I27	Other pulmonary heart diseases	I00.I99	Latvia	11.77	1	yes
I27	Other pulmonary heart diseases	I00.I99	Lithuania	24.90	2	yes
I27	Other pulmonary heart diseases	I00.I99	Netherlands	201.56	2	yes
I27	Other pulmonary heart diseases	I00.I99	Norway	37.02	0	no
I27	Other pulmonary heart diseases	I00.I99	Poland	1714.20	3	yes
I27	Other pulmonary heart diseases	I00.I99	Romania	7548.71	2	yes
I27	Other pulmonary heart diseases	I00.I99	Slovenia	49.63	1	yes
I27	Other pulmonary heart diseases	I00.I99	Spain	735.74	3	yes
I27	Other pulmonary heart diseases	I00.I99	Sweden	79.14	2	yes
I27	Other pulmonary heart diseases	I00.I99	Switzerland	106.50	0	no
I27	Other pulmonary heart diseases	I00.I99	United Kingdom	567.12	2	yes

For peer review only

I28	Other diseases of pulmonary vessels	I00.I99	Czech Republic	8.15	0	no
I28	Other diseases of pulmonary vessels	I00.I99	France	9.98	0	no
I28	Other diseases of pulmonary vessels	I00.I99	Germany	7.41	2	yes
I28	Other diseases of pulmonary vessels	I00.I99	Poland	5.92	0	no
I28	Other diseases of pulmonary vessels	I00.I99	Romania	54.65	1	yes
I28	Other diseases of pulmonary vessels	I00.I99	Spain	6.89	0	no
I28	Other diseases of pulmonary vessels	I00.I99	United Kingdom	8.04	1	yes
I30	Acute pericarditis	I00.I99	Austria	7.96	0	no
I30	Acute pericarditis	I00.I99	Czech Republic	17.41	0	no
I30	Acute pericarditis	I00.I99	Finland	12.39	1	yes
I30	Acute pericarditis	I00.I99	France	10.56	2	yes
I30	Acute pericarditis	I00.I99	Germany	23.31	1	yes
I30	Acute pericarditis	I00.I99	Hungary	18.91	2	yes
I30	Acute pericarditis	I00.I99	Netherlands	5.79	2	yes
I30	Acute pericarditis	I00.I99	Poland	22.94	0	no
I30	Acute pericarditis	I00.I99	Romania	30.25	0	no
I30	Acute pericarditis	I00.I99	Spain	13.30	0	no
I30	Acute pericarditis	I00.I99	Sweden	8.49	1	yes
I30	Acute pericarditis	I00.I99	United Kingdom	69.80	1	yes
I31	Other diseases of pericardium	I00.I99	Austria	15.17	1	yes
I31	Other diseases of pericardium	I00.I99	Belgium	33.90	2	yes
I31	Other diseases of pericardium	I00.I99	Croatia	7.85	0	no
I31	Other diseases of pericardium	I00.I99	Czech Republic	56.77	2	yes
I31	Other diseases of pericardium	I00.I99	Denmark	9.89	0	no
I31	Other diseases of pericardium	I00.I99	France	174.81	1	yes
I31	Other diseases of pericardium	I00.I99	Germany	135.59	3	yes
I31	Other diseases of pericardium	I00.I99	Hungary	22.95	1	yes
I31	Other diseases of pericardium	I00.I99	Netherlands	71.77	1	yes
I31	Other diseases of pericardium	I00.I99	Norway	6.66	3	yes
I31	Other diseases of pericardium	I00.I99	Poland	39.61	2	yes
I31	Other diseases of pericardium	I00.I99	Romania	56.68	1	yes
I31	Other diseases of pericardium	I00.I99	Slovenia	5.64	1	yes
I31	Other diseases of pericardium	I00.I99	Spain	273.31	2	yes
I31	Other diseases of pericardium	I00.I99	Sweden	16.82	2	yes
I31	Other diseases of pericardium	I00.I99	Switzerland	11.63	0	no
I31	Other diseases of pericardium	I00.I99	United Kingdom	159.67	0	no
I33	Acute and subacute endocarditis	I00.I99	Austria	11.61	0	no
I33	Acute and subacute endocarditis	I00.I99	Belgium	41.72	2	yes
I33	Acute and subacute endocarditis	I00.I99	Croatia	9.34	1	yes
I33	Acute and subacute endocarditis	I00.I99	Czech Republic	80.46	0	no
I33	Acute and subacute endocarditis	I00.I99	Denmark	36.68	3	yes
I33	Acute and subacute endocarditis	I00.I99	Estonia	19.02	3	yes
I33	Acute and subacute endocarditis	I00.I99	Finland	28.83	1	yes
I33	Acute and subacute endocarditis	I00.I99	France	273.38	2	yes
I33	Acute and subacute endocarditis	I00.I99	Germany	252.22	3	yes
I33	Acute and subacute endocarditis	I00.I99	Hungary	76.05	2	yes
I33	Acute and subacute endocarditis	I00.I99	Latvia	16.07	0	no
I33	Acute and subacute endocarditis	I00.I99	Lithuania	22.63	0	no
I33	Acute and subacute endocarditis	I00.I99	Netherlands	80.61	2	yes
I33	Acute and subacute endocarditis	I00.I99	Norway	12.26	2	yes

For peer review only

I33	Acute and subacute endocarditis	I00.I99	Poland	151.30	2	yes
I33	Acute and subacute endocarditis	I00.I99	Romania	56.10	1	yes
I33	Acute and subacute endocarditis	I00.I99	Slovenia	6.98	0	no
I33	Acute and subacute endocarditis	I00.I99	Spain	236.15	1	yes
I33	Acute and subacute endocarditis	I00.I99	Sweden	28.46	0	no
I33	Acute and subacute endocarditis	I00.I99	Switzerland	59.86	0	no
I33	Acute and subacute endocarditis	I00.I99	United Kingdom	378.67	2	yes
I34	Nonrheumatic mitral valve disorders	I00.I99	Austria	184.86	2	yes
I34	Nonrheumatic mitral valve disorders	I00.I99	Belgium	100.32	2	yes
I34	Nonrheumatic mitral valve disorders	I00.I99	Croatia	55.10	3	yes
I34	Nonrheumatic mitral valve disorders	I00.I99	Czech Republic	174.89	2	yes
I34	Nonrheumatic mitral valve disorders	I00.I99	Denmark	92.78	1	yes
I34	Nonrheumatic mitral valve disorders	I00.I99	Estonia	13.34	0	no
I34	Nonrheumatic mitral valve disorders	I00.I99	Finland	110.01	0	no
I34	Nonrheumatic mitral valve disorders	I00.I99	France	56.18	2	yes
I34	Nonrheumatic mitral valve disorders	I00.I99	Germany	1611.76	1	yes
I34	Nonrheumatic mitral valve disorders	I00.I99	Hungary	402.87	1	yes
I34	Nonrheumatic mitral valve disorders	I00.I99	Latvia	6.38	1	yes
I34	Nonrheumatic mitral valve disorders	I00.I99	Lithuania	9.36	0	no
I34	Nonrheumatic mitral valve disorders	I00.I99	Netherlands	715.26	2	yes
I34	Nonrheumatic mitral valve disorders	I00.I99	Norway	70.06	0	no
I34	Nonrheumatic mitral valve disorders	I00.I99	Poland	422.45	0	no
I34	Nonrheumatic mitral valve disorders	I00.I99	Romania	158.26	2	yes
I34	Nonrheumatic mitral valve disorders	I00.I99	Slovenia	53.56	0	no
I34	Nonrheumatic mitral valve disorders	I00.I99	Spain	581.17	1	yes
I34	Nonrheumatic mitral valve disorders	I00.I99	Sweden	108.79	0	no
I34	Nonrheumatic mitral valve disorders	I00.I99	Switzerland	113.95	1	yes
I34	Nonrheumatic mitral valve disorders	I00.I99	United Kingdom	587.52	0	no
I35	Nonrheumatic aortic valve disorders	I00.I99	Austria	639.93	2	yes
I35	Nonrheumatic aortic valve disorders	I00.I99	Belgium	819.66	3	yes
I35	Nonrheumatic aortic valve disorders	I00.I99	Croatia	168.43	3	yes
I35	Nonrheumatic aortic valve disorders	I00.I99	Czech Republic	402.31	2	yes
I35	Nonrheumatic aortic valve disorders	I00.I99	Denmark	432.67	2	yes
I35	Nonrheumatic aortic valve disorders	I00.I99	Estonia	65.65	2	yes
I35	Nonrheumatic aortic valve disorders	I00.I99	Finland	468.98	3	yes
I35	Nonrheumatic aortic valve disorders	I00.I99	France	3967.82	3	yes
I35	Nonrheumatic aortic valve disorders	I00.I99	Germany	7199.93	2	yes
I35	Nonrheumatic aortic valve disorders	I00.I99	Hungary	819.40	1	yes
I35	Nonrheumatic aortic valve disorders	I00.I99	Latvia	43.68	1	yes
I35	Nonrheumatic aortic valve disorders	I00.I99	Lithuania	43.91	1	yes
I35	Nonrheumatic aortic valve disorders	I00.I99	Netherlands	1306.03	2	yes
I35	Nonrheumatic aortic valve disorders	I00.I99	Norway	495.89	0	no
I35	Nonrheumatic aortic valve disorders	I00.I99	Poland	891.90	2	yes
I35	Nonrheumatic aortic valve disorders	I00.I99	Romania	247.91	1	yes
I35	Nonrheumatic aortic valve disorders	I00.I99	Slovenia	226.36	2	yes
I35	Nonrheumatic aortic valve disorders	I00.I99	Spain	2760.54	1	yes
I35	Nonrheumatic aortic valve disorders	I00.I99	Sweden	673.35	1	yes
I35	Nonrheumatic aortic valve disorders	I00.I99	Switzerland	487.66	0	no
I35	Nonrheumatic aortic valve disorders	I00.I99	United Kingdom	3850.34	2	yes
I36	Nonrheumatic tricuspid valve disorders	I00.I99	Austria	24.90	1	yes

For peer review only

I36	Nonrheumatic tricuspid valve disorders	I00.I99	Czech Republic	21.51	1	yes
I36	Nonrheumatic tricuspid valve disorders	I00.I99	Finland	15.42	2	yes
I36	Nonrheumatic tricuspid valve disorders	I00.I99	Germany	139.92	1	yes
I36	Nonrheumatic tricuspid valve disorders	I00.I99	Hungary	29.97	0	no
I36	Nonrheumatic tricuspid valve disorders	I00.I99	Netherlands	22.35	2	yes
I36	Nonrheumatic tricuspid valve disorders	I00.I99	Poland	32.02	2	yes
I36	Nonrheumatic tricuspid valve disorders	I00.I99	Romania	7.67	0	no
I36	Nonrheumatic tricuspid valve disorders	I00.I99	Spain	6.63	0	no
I37	Pulmonary valve disorders	I00.I99	Germany	9.79	0	no
I37	Pulmonary valve disorders	I00.I99	Romania	7.66	1	yes
I38	Endocarditis, valve unspecified	I00.I99	Austria	68.23	0	no
I38	Endocarditis, valve unspecified	I00.I99	Belgium	334.37	1	yes
I38	Endocarditis, valve unspecified	I00.I99	Croatia	7.00	1	yes
I38	Endocarditis, valve unspecified	I00.I99	Czech Republic	20.41	1	yes
I38	Endocarditis, valve unspecified	I00.I99	Denmark	38.79	0	no
I38	Endocarditis, valve unspecified	I00.I99	France	1453.32	2	yes
I38	Endocarditis, valve unspecified	I00.I99	Germany	1203.76	2	yes
I38	Endocarditis, valve unspecified	I00.I99	Hungary	139.45	2	yes
I38	Endocarditis, valve unspecified	I00.I99	Netherlands	729.10	0	no
I38	Endocarditis, valve unspecified	I00.I99	Norway	71.19	1	yes
I38	Endocarditis, valve unspecified	I00.I99	Poland	160.70	3	yes
I38	Endocarditis, valve unspecified	I00.I99	Romania	12.12	2	yes
I38	Endocarditis, valve unspecified	I00.I99	Spain	978.26	2	yes
I38	Endocarditis, valve unspecified	I00.I99	Sweden	118.20	0	no
I38	Endocarditis, valve unspecified	I00.I99	Switzerland	362.19	2	yes
I38	Endocarditis, valve unspecified	I00.I99	United Kingdom	889.72	1	yes
I40	Acute myocarditis	I00.I99	Czech Republic	15.94	0	no
I40	Acute myocarditis	I00.I99	Denmark	12.71	1	yes
I40	Acute myocarditis	I00.I99	Finland	34.68	2	yes
I40	Acute myocarditis	I00.I99	France	11.87	0	no
I40	Acute myocarditis	I00.I99	Germany	93.17	2	yes
I40	Acute myocarditis	I00.I99	Hungary	5.80	2	yes
I40	Acute myocarditis	I00.I99	Netherlands	6.63	2	yes
I40	Acute myocarditis	I00.I99	Norway	5.49	0	no
I40	Acute myocarditis	I00.I99	Poland	52.89	1	yes
I40	Acute myocarditis	I00.I99	Romania	52.31	1	yes
I40	Acute myocarditis	I00.I99	Spain	10.29	0	no
I40	Acute myocarditis	I00.I99	Sweden	22.98	0	no
I40	Acute myocarditis	I00.I99	United Kingdom	57.28	0	no
I42	Cardiomyopathy	I00.I99	Austria	1457.24	3	yes
I42	Cardiomyopathy	I00.I99	Belgium	660.91	2	yes
I42	Cardiomyopathy	I00.I99	Croatia	326.03	0	no
I42	Cardiomyopathy	I00.I99	Czech Republic	298.99	2	yes
I42	Cardiomyopathy	I00.I99	Denmark	156.26	1	yes
I42	Cardiomyopathy	I00.I99	Estonia	264.01	1	yes
I42	Cardiomyopathy	I00.I99	Finland	452.48	2	yes
I42	Cardiomyopathy	I00.I99	France	3765.71	3	yes
I42	Cardiomyopathy	I00.I99	Germany	6678.86	1	yes
I42	Cardiomyopathy	I00.I99	Hungary	1555.47	2	yes
I42	Cardiomyopathy	I00.I99	Latvia	887.15	3	yes

For peer review only

I42	Cardiomyopathy	I00.I99	Lithuania	583.02	0	no
I42	Cardiomyopathy	I00.I99	Netherlands	646.93	2	yes
I42	Cardiomyopathy	I00.I99	Norway	123.89	0	no
I42	Cardiomyopathy	I00.I99	Poland	3796.33	0	no
I42	Cardiomyopathy	I00.I99	Romania	4263.48	0	no
I42	Cardiomyopathy	I00.I99	Slovenia	854.76	3	yes
I42	Cardiomyopathy	I00.I99	Spain	3290.13	3	yes
I42	Cardiomyopathy	I00.I99	Sweden	337.48	1	yes
I42	Cardiomyopathy	I00.I99	Switzerland	328.21	2	yes
I42	Cardiomyopathy	I00.I99	United Kingdom	1673.18	2	yes
I44	Atrioventricular and left bundle-branch block	I00.I99	Austria	14.71	2	yes
I44	Atrioventricular and left bundle-branch block	I00.I99	Belgium	81.72	1	yes
I44	Atrioventricular and left bundle-branch block	I00.I99	Croatia	14.45	1	yes
I44	Atrioventricular and left bundle-branch block	I00.I99	Czech Republic	26.50	0	no
I44	Atrioventricular and left bundle-branch block	I00.I99	Denmark	57.22	3	yes
I44	Atrioventricular and left bundle-branch block	I00.I99	Finland	14.32	0	no
I44	Atrioventricular and left bundle-branch block	I00.I99	France	427.52	1	yes
I44	Atrioventricular and left bundle-branch block	I00.I99	Germany	467.03	1	yes
I44	Atrioventricular and left bundle-branch block	I00.I99	Hungary	19.02	1	yes
I44	Atrioventricular and left bundle-branch block	I00.I99	Netherlands	74.23	0	no
I44	Atrioventricular and left bundle-branch block	I00.I99	Norway	25.10	0	no
I44	Atrioventricular and left bundle-branch block	I00.I99	Poland	139.77	1	yes
I44	Atrioventricular and left bundle-branch block	I00.I99	Slovenia	15.60	0	no
I44	Atrioventricular and left bundle-branch block	I00.I99	Spain	517.34	3	yes
I44	Atrioventricular and left bundle-branch block	I00.I99	Sweden	39.02	1	yes
I44	Atrioventricular and left bundle-branch block	I00.I99	Switzerland	37.59	0	no
I44	Atrioventricular and left bundle-branch block	I00.I99	United Kingdom	95.38	0	no
I45	Other conduction disorders	I00.I99	Belgium	59.43	1	yes
I45	Other conduction disorders	I00.I99	Croatia	5.13	0	no
I45	Other conduction disorders	I00.I99	Czech Republic	13.46	1	yes
I45	Other conduction disorders	I00.I99	Denmark	13.93	0	no
I45	Other conduction disorders	I00.I99	France	153.81	1	yes
I45	Other conduction disorders	I00.I99	Germany	84.41	2	yes
I45	Other conduction disorders	I00.I99	Hungary	13.70	1	yes
I45	Other conduction disorders	I00.I99	Netherlands	75.03	1	yes
I45	Other conduction disorders	I00.I99	Poland	48.53	1	yes
I45	Other conduction disorders	I00.I99	Slovenia	5.90	1	yes
I45	Other conduction disorders	I00.I99	Spain	143.60	1	yes
I45	Other conduction disorders	I00.I99	Sweden	7.25	0	no
I45	Other conduction disorders	I00.I99	Switzerland	12.35	0	no
I45	Other conduction disorders	I00.I99	United Kingdom	64.24	0	no
I46	Cardiac arrest	I00.I99	Austria	185.45	3	yes
I46	Cardiac arrest	I00.I99	Belgium	2222.61	2	yes
I46	Cardiac arrest	I00.I99	Croatia	744.62	1	yes
I46	Cardiac arrest	I00.I99	Czech Republic	940.80	2	yes
I46	Cardiac arrest	I00.I99	Denmark	345.02	3	yes
I46	Cardiac arrest	I00.I99	Estonia	40.42	2	yes
I46	Cardiac arrest	I00.I99	Finland	13.57	0	no
I46	Cardiac arrest	I00.I99	France	5190.04	2	yes
I46	Cardiac arrest	I00.I99	Germany	4653.47	1	yes

For peer review only

I46	Cardiac arrest	I00.I99	Hungary	46.33	2	yes
I46	Cardiac arrest	I00.I99	Latvia	15.69	0	no
I46	Cardiac arrest	I00.I99	Lithuania	161.88	2	yes
I46	Cardiac arrest	I00.I99	Netherlands	3165.96	1	yes
I46	Cardiac arrest	I00.I99	Norway	181.58	0	no
I46	Cardiac arrest	I00.I99	Poland	8349.63	0	no
I46	Cardiac arrest	I00.I99	Slovenia	171.83	3	yes
I46	Cardiac arrest	I00.I99	Spain	86.52	1	yes
I46	Cardiac arrest	I00.I99	Sweden	233.64	2	yes
I46	Cardiac arrest	I00.I99	Switzerland	994.15	3	yes
I46	Cardiac arrest	I00.I99	United Kingdom	148.07	0	no
I47	Paroxysmal tachycardia	I00.I99	Austria	55.90	2	yes
I47	Paroxysmal tachycardia	I00.I99	Belgium	39.11	2	yes
I47	Paroxysmal tachycardia	I00.I99	Croatia	5.34	0	no
I47	Paroxysmal tachycardia	I00.I99	Czech Republic	15.18	0	no
I47	Paroxysmal tachycardia	I00.I99	Denmark	14.78	0	no
I47	Paroxysmal tachycardia	I00.I99	France	151.27	1	yes
I47	Paroxysmal tachycardia	I00.I99	Germany	120.00	1	yes
I47	Paroxysmal tachycardia	I00.I99	Hungary	8.42	1	yes
I47	Paroxysmal tachycardia	I00.I99	Netherlands	25.82	2	yes
I47	Paroxysmal tachycardia	I00.I99	Norway	6.66	0	no
I47	Paroxysmal tachycardia	I00.I99	Poland	40.65	2	yes
I47	Paroxysmal tachycardia	I00.I99	Spain	124.77	2	yes
I47	Paroxysmal tachycardia	I00.I99	Sweden	7.58	0	no
I47	Paroxysmal tachycardia	I00.I99	Switzerland	10.90	0	no
I47	Paroxysmal tachycardia	I00.I99	United Kingdom	32.96	1	yes
I48	Atrial fibrillation and flutter	I00.I99	Austria	1117.41	3	yes
I48	Atrial fibrillation and flutter	I00.I99	Belgium	1349.46	0	no
I48	Atrial fibrillation and flutter	I00.I99	Croatia	101.22	1	yes
I48	Atrial fibrillation and flutter	I00.I99	Czech Republic	508.01	3	yes
I48	Atrial fibrillation and flutter	I00.I99	Denmark	863.90	3	yes
I48	Atrial fibrillation and flutter	I00.I99	Estonia	60.90	3	yes
I48	Atrial fibrillation and flutter	I00.I99	Finland	369.82	3	yes
I48	Atrial fibrillation and flutter	I00.I99	France	5335.15	1	yes
I48	Atrial fibrillation and flutter	I00.I99	Germany	13525.87	3	yes
I48	Atrial fibrillation and flutter	I00.I99	Hungary	566.67	2	yes
I48	Atrial fibrillation and flutter	I00.I99	Latvia	12.96	3	yes
I48	Atrial fibrillation and flutter	I00.I99	Lithuania	37.54	0	no
I48	Atrial fibrillation and flutter	I00.I99	Netherlands	1862.07	2	yes
I48	Atrial fibrillation and flutter	I00.I99	Norway	810.08	0	no
I48	Atrial fibrillation and flutter	I00.I99	Poland	1769.55	2	yes
I48	Atrial fibrillation and flutter	I00.I99	Slovenia	133.19	0	no
I48	Atrial fibrillation and flutter	I00.I99	Spain	4685.60	2	yes
I48	Atrial fibrillation and flutter	I00.I99	Sweden	2010.98	1	yes
I48	Atrial fibrillation and flutter	I00.I99	Switzerland	391.52	1	yes
I48	Atrial fibrillation and flutter	I00.I99	United Kingdom	4788.59	2	yes
I49	Other cardiac arrhythmias	I00.I99	Austria	173.23	3	yes
I49	Other cardiac arrhythmias	I00.I99	Belgium	1018.28	2	yes
I49	Other cardiac arrhythmias	I00.I99	Croatia	22.91	3	yes
I49	Other cardiac arrhythmias	I00.I99	Czech Republic	177.71	0	no

For peer review only

I49	Other cardiac arrhythmias	I00.I99	Denmark	109.75	0	no
I49	Other cardiac arrhythmias	I00.I99	Estonia	6.21	0	no
I49	Other cardiac arrhythmias	I00.I99	Finland	27.35	0	no
I49	Other cardiac arrhythmias	I00.I99	France	5632.77	3	yes
I49	Other cardiac arrhythmias	I00.I99	Germany	4955.52	2	yes
I49	Other cardiac arrhythmias	I00.I99	Hungary	144.97	0	no
I49	Other cardiac arrhythmias	I00.I99	Lithuania	49.00	1	yes
I49	Other cardiac arrhythmias	I00.I99	Netherlands	936.58	1	yes
I49	Other cardiac arrhythmias	I00.I99	Norway	90.90	1	yes
I49	Other cardiac arrhythmias	I00.I99	Poland	417.00	1	yes
I49	Other cardiac arrhythmias	I00.I99	Slovenia	28.86	0	no
I49	Other cardiac arrhythmias	I00.I99	Spain	1489.64	2	yes
I49	Other cardiac arrhythmias	I00.I99	Sweden	197.40	0	no
I49	Other cardiac arrhythmias	I00.I99	Switzerland	256.83	1	yes
I49	Other cardiac arrhythmias	I00.I99	United Kingdom	246.37	2	yes
I50	Heart failure	I00.I99	Austria	2372.75	2	yes
I50	Heart failure	I00.I99	Belgium	5368.50	2	yes
I50	Heart failure	I00.I99	Croatia	3784.70	3	yes
I50	Heart failure	I00.I99	Czech Republic	3191.96	1	yes
I50	Heart failure	I00.I99	Denmark	1556.62	1	yes
I50	Heart failure	I00.I99	Estonia	177.44	2	yes
I50	Heart failure	I00.I99	Finland	469.68	2	yes
I50	Heart failure	I00.I99	France	23643.30	1	yes
I50	Heart failure	I00.I99	Germany	54870.33	2	yes
I50	Heart failure	I00.I99	Hungary	1866.89	2	yes
I50	Heart failure	I00.I99	Latvia	324.36	3	yes
I50	Heart failure	I00.I99	Lithuania	341.15	3	yes
I50	Heart failure	I00.I99	Netherlands	8571.22	3	yes
I50	Heart failure	I00.I99	Norway	1750.66	2	yes
I50	Heart failure	I00.I99	Poland	41647.45	2	yes
I50	Heart failure	I00.I99	Slovenia	1495.94	3	yes
I50	Heart failure	I00.I99	Spain	21476.66	3	yes
I50	Heart failure	I00.I99	Sweden	3581.84	2	yes
I50	Heart failure	I00.I99	Switzerland	2390.62	2	yes
I50	Heart failure	I00.I99	United Kingdom	10112.86	1	yes
I51	Complications and ill-defined descriptions of heart disease	I00.I99	Austria	1981.80	3	yes
I51	Complications and ill-defined descriptions of heart disease	I00.I99	Belgium	1551.99	1	yes
I51	Complications and ill-defined descriptions of heart disease	I00.I99	Croatia	1442.58	3	yes
I51	Complications and ill-defined descriptions of heart disease	I00.I99	Czech Republic	403.14	1	yes
I51	Complications and ill-defined descriptions of heart disease	I00.I99	Denmark	452.29	1	yes
I51	Complications and ill-defined descriptions of heart disease	I00.I99	Estonia	191.48	3	yes
I51	Complications and ill-defined descriptions of heart disease	I00.I99	Finland	241.76	0	no
I51	Complications and ill-defined descriptions of heart disease	I00.I99	France	4168.93	1	yes
I51	Complications and ill-defined descriptions of heart disease	I00.I99	Germany	2244.81	3	yes

For peer review only

I51	Complications and ill-defined descriptions of heart disease	I00.I99	Hungary	1558.56	2	yes
I51	Complications and ill-defined descriptions of heart disease	I00.I99	Latvia	90.16	2	yes
I51	Complications and ill-defined descriptions of heart disease	I00.I99	Lithuania	17.03	0	no
I51	Complications and ill-defined descriptions of heart disease	I00.I99	Netherlands	1145.91	3	yes
I51	Complications and ill-defined descriptions of heart disease	I00.I99	Norway	438.70	1	yes
I51	Complications and ill-defined descriptions of heart disease	I00.I99	Poland	7888.00	1	yes
I51	Complications and ill-defined descriptions of heart disease	I00.I99	Romania	8053.11	3	yes
I51	Complications and ill-defined descriptions of heart disease	I00.I99	Slovenia	140.02	1	yes
I51	Complications and ill-defined descriptions of heart disease	I00.I99	Spain	2782.26	1	yes
I51	Complications and ill-defined descriptions of heart disease	I00.I99	Sweden	638.49	3	yes
I51	Complications and ill-defined descriptions of heart disease	I00.I99	Switzerland	584.60	1	yes
I51	Complications and ill-defined descriptions of heart disease	I00.I99	United Kingdom	3463.52	0	no
I60	Subarachnoid haemorrhage	I00.I99	Austria	238.42	0	no
I60	Subarachnoid haemorrhage	I00.I99	Belgium	252.28	1	yes
I60	Subarachnoid haemorrhage	I00.I99	Croatia	130.60	0	no
I60	Subarachnoid haemorrhage	I00.I99	Czech Republic	268.38	0	no
I60	Subarachnoid haemorrhage	I00.I99	Denmark	200.74	1	yes
I60	Subarachnoid haemorrhage	I00.I99	Estonia	58.78	2	yes
I60	Subarachnoid haemorrhage	I00.I99	Finland	329.85	1	yes
I60	Subarachnoid haemorrhage	I00.I99	France	1364.79	2	yes
I60	Subarachnoid haemorrhage	I00.I99	Germany	2057.10	1	yes
I60	Subarachnoid haemorrhage	I00.I99	Hungary	371.19	2	yes
I60	Subarachnoid haemorrhage	I00.I99	Latvia	100.22	0	no
I60	Subarachnoid haemorrhage	I00.I99	Lithuania	155.20	0	no
I60	Subarachnoid haemorrhage	I00.I99	Netherlands	465.76	1	yes
I60	Subarachnoid haemorrhage	I00.I99	Norway	151.86	1	yes
I60	Subarachnoid haemorrhage	I00.I99	Poland	1593.75	2	yes
I60	Subarachnoid haemorrhage	I00.I99	Romania	730.16	2	yes
I60	Subarachnoid haemorrhage	I00.I99	Slovenia	66.94	0	no
I60	Subarachnoid haemorrhage	I00.I99	Spain	1041.63	1	yes
I60	Subarachnoid haemorrhage	I00.I99	Sweden	288.17	1	yes
I60	Subarachnoid haemorrhage	I00.I99	Switzerland	174.57	2	yes
I60	Subarachnoid haemorrhage	I00.I99	United Kingdom	2526.85	2	yes
I61	Intracerebral haemorrhage	I00.I99	Austria	973.84	3	yes
I61	Intracerebral haemorrhage	I00.I99	Belgium	1679.19	1	yes
I61	Intracerebral haemorrhage	I00.I99	Croatia	699.59	2	yes
I61	Intracerebral haemorrhage	I00.I99	Czech Republic	1529.54	1	yes
I61	Intracerebral haemorrhage	I00.I99	Denmark	861.16	3	yes
I61	Intracerebral haemorrhage	I00.I99	Estonia	259.16	2	yes
I61	Intracerebral haemorrhage	I00.I99	Finland	917.39	2	yes
I61	Intracerebral haemorrhage	I00.I99	France	7591.93	1	yes
I61	Intracerebral haemorrhage	I00.I99	Germany	8323.13	3	yes

For peer review only

I61	Intracerebral haemorrhage	I00.I99	Hungary	2964.78	2	yes
I61	Intracerebral haemorrhage	I00.I99	Latvia	724.27	2	yes
I61	Intracerebral haemorrhage	I00.I99	Lithuania	689.31	1	yes
I61	Intracerebral haemorrhage	I00.I99	Netherlands	2130.66	3	yes
I61	Intracerebral haemorrhage	I00.I99	Norway	609.72	2	yes
I61	Intracerebral haemorrhage	I00.I99	Poland	7813.51	1	yes
I61	Intracerebral haemorrhage	I00.I99	Romania	6881.02	2	yes
I61	Intracerebral haemorrhage	I00.I99	Slovenia	312.13	3	yes
I61	Intracerebral haemorrhage	I00.I99	Spain	6790.25	3	yes
I61	Intracerebral haemorrhage	I00.I99	Sweden	1190.28	2	yes
I61	Intracerebral haemorrhage	I00.I99	Switzerland	602.14	1	yes
I61	Intracerebral haemorrhage	I00.I99	United Kingdom	5994.60	2	yes
I62	Other nontraumatic intracranial haemorrhage	I00.I99	Austria	118.47	3	yes
I62	Other nontraumatic intracranial haemorrhage	I00.I99	Belgium	284.39	2	yes
I62	Other nontraumatic intracranial haemorrhage	I00.I99	Croatia	50.81	1	yes
I62	Other nontraumatic intracranial haemorrhage	I00.I99	Czech Republic	102.64	1	yes
I62	Other nontraumatic intracranial haemorrhage	I00.I99	Denmark	65.16	2	yes
I62	Other nontraumatic intracranial haemorrhage	I00.I99	Estonia	22.83	0	no
I62	Other nontraumatic intracranial haemorrhage	I00.I99	Finland	28.71	1	yes
I62	Other nontraumatic intracranial haemorrhage	I00.I99	France	719.03	1	yes
I62	Other nontraumatic intracranial haemorrhage	I00.I99	Germany	854.19	2	yes
I62	Other nontraumatic intracranial haemorrhage	I00.I99	Hungary	94.56	0	no
I62	Other nontraumatic intracranial haemorrhage	I00.I99	Latvia	15.05	0	no
I62	Other nontraumatic intracranial haemorrhage	I00.I99	Lithuania	29.65	1	yes
I62	Other nontraumatic intracranial haemorrhage	I00.I99	Netherlands	219.65	1	yes
I62	Other nontraumatic intracranial haemorrhage	I00.I99	Norway	65.42	3	yes
I62	Other nontraumatic intracranial haemorrhage	I00.I99	Poland	370.01	0	no
I62	Other nontraumatic intracranial haemorrhage	I00.I99	Romania	786.94	2	yes
I62	Other nontraumatic intracranial haemorrhage	I00.I99	Slovenia	23.29	0	no
I62	Other nontraumatic intracranial haemorrhage	I00.I99	Spain	1155.55	2	yes
I62	Other nontraumatic intracranial haemorrhage	I00.I99	Sweden	73.16	0	no
I62	Other nontraumatic intracranial haemorrhage	I00.I99	Switzerland	74.36	0	no
I62	Other nontraumatic intracranial haemorrhage	I00.I99	United Kingdom	1487.96	2	yes
I63	Cerebral infarction	I00.I99	Austria	1525.41	3	yes
I63	Cerebral infarction	I00.I99	Belgium	994.06	3	yes
I63	Cerebral infarction	I00.I99	Croatia	858.73	2	yes
I63	Cerebral infarction	I00.I99	Czech Republic	3387.60	3	yes
I63	Cerebral infarction	I00.I99	Denmark	672.28	2	yes
I63	Cerebral infarction	I00.I99	Estonia	1631.25	3	yes
I63	Cerebral infarction	I00.I99	Finland	2602.50	2	yes
I63	Cerebral infarction	I00.I99	France	7063.35	1	yes
I63	Cerebral infarction	I00.I99	Germany	14657.00	2	yes
I63	Cerebral infarction	I00.I99	Hungary	8888.22	2	yes
I63	Cerebral infarction	I00.I99	Latvia	2486.29	3	yes
I63	Cerebral infarction	I00.I99	Lithuania	3416.08	2	yes
I63	Cerebral infarction	I00.I99	Netherlands	1911.39	3	yes
I63	Cerebral infarction	I00.I99	Norway	538.61	1	yes
I63	Cerebral infarction	I00.I99	Poland	17791.08	3	yes
I63	Cerebral infarction	I00.I99	Romania	3766.43	3	yes
I63	Cerebral infarction	I00.I99	Slovenia	513.60	0	no

For peer review only

I63	Cerebral infarction	I00.I99	Spain	4966.81	2	yes
I63	Cerebral infarction	I00.I99	Sweden	1951.91	2	yes
I63	Cerebral infarction	I00.I99	Switzerland	565.09	3	yes
I63	Cerebral infarction	I00.I99	United Kingdom	6055.64	2	yes
I64	Stroke, not specified as haemorrhage or infarction	I00.I99	Austria	2854.49	3	yes
I64	Stroke, not specified as haemorrhage or infarction	I00.I99	Belgium	4784.77	1	yes
I64	Stroke, not specified as haemorrhage or infarction	I00.I99	Croatia	6838.57	2	yes
I64	Stroke, not specified as haemorrhage or infarction	I00.I99	Czech Republic	7456.71	2	yes
I64	Stroke, not specified as haemorrhage or infarction	I00.I99	Denmark	2666.18	1	yes
I64	Stroke, not specified as haemorrhage or infarction	I00.I99	Estonia	117.95	1	yes
I64	Stroke, not specified as haemorrhage or infarction	I00.I99	Finland	205.39	1	yes
I64	Stroke, not specified as haemorrhage or infarction	I00.I99	France	15826.91	2	yes
I64	Stroke, not specified as haemorrhage or infarction	I00.I99	Germany	30020.84	2	yes
I64	Stroke, not specified as haemorrhage or infarction	I00.I99	Hungary	1306.38	2	yes
I64	Stroke, not specified as haemorrhage or infarction	I00.I99	Latvia	760.31	3	yes
I64	Stroke, not specified as haemorrhage or infarction	I00.I99	Lithuania	371.06	3	yes
I64	Stroke, not specified as haemorrhage or infarction	I00.I99	Netherlands	7358.07	2	yes
I64	Stroke, not specified as haemorrhage or infarction	I00.I99	Norway	2011.62	3	yes
I64	Stroke, not specified as haemorrhage or infarction	I00.I99	Poland	18658.87	3	yes
I64	Stroke, not specified as haemorrhage or infarction	I00.I99	Romania	46541.44	1	yes
I64	Stroke, not specified as haemorrhage or infarction	I00.I99	Slovenia	1061.65	2	yes
I64	Stroke, not specified as haemorrhage or infarction	I00.I99	Spain	16415.46	3	yes
I64	Stroke, not specified as haemorrhage or infarction	I00.I99	Sweden	2230.47	1	yes
I64	Stroke, not specified as haemorrhage or infarction	I00.I99	Switzerland	2501.46	2	yes
I64	Stroke, not specified as haemorrhage or infarction	I00.I99	United Kingdom	30051.23	3	yes
I67	Other cerebrovascular diseases	I00.I99	Austria	499.10	3	yes
I67	Other cerebrovascular diseases	I00.I99	Belgium	498.79	2	yes
I67	Other cerebrovascular diseases	I00.I99	Croatia	404.42	0	no
I67	Other cerebrovascular diseases	I00.I99	Czech Republic	5792.12	1	yes
I67	Other cerebrovascular diseases	I00.I99	Denmark	103.43	2	yes
I67	Other cerebrovascular diseases	I00.I99	Estonia	131.46	1	yes
I67	Other cerebrovascular diseases	I00.I99	Finland	145.79	1	yes
I67	Other cerebrovascular diseases	I00.I99	France	2394.07	3	yes
I67	Other cerebrovascular diseases	I00.I99	Germany	8174.79	2	yes
I67	Other cerebrovascular diseases	I00.I99	Hungary	3746.83	2	yes
I67	Other cerebrovascular diseases	I00.I99	Latvia	2483.25	3	yes
I67	Other cerebrovascular diseases	I00.I99	Lithuania	1990.34	0	no
I67	Other cerebrovascular diseases	I00.I99	Netherlands	161.70	2	yes
I67	Other cerebrovascular diseases	I00.I99	Norway	86.31	1	yes
I67	Other cerebrovascular diseases	I00.I99	Poland	4705.31	1	yes
I67	Other cerebrovascular diseases	I00.I99	Romania	16741.32	3	yes
I67	Other cerebrovascular diseases	I00.I99	Slovenia	28.07	2	yes
I67	Other cerebrovascular diseases	I00.I99	Spain	6026.49	1	yes
I67	Other cerebrovascular diseases	I00.I99	Sweden	924.66	2	yes
I67	Other cerebrovascular diseases	I00.I99	Switzerland	393.94	1	yes
I67	Other cerebrovascular diseases	I00.I99	United Kingdom	11450.12	2	yes
I69	Sequelae of cerebrovascular disease	I00.I99	Austria	264.22	1	yes
I69	Sequelae of cerebrovascular disease	I00.I99	Belgium	298.15	0	no
I69	Sequelae of cerebrovascular disease	I00.I99	Croatia	1452.77	3	yes
I69	Sequelae of cerebrovascular disease	I00.I99	Czech Republic	738.46	2	yes

For peer review only

I69	Sequelae of cerebrovascular disease	I00.I99	Denmark	565.13	3	yes
I69	Sequelae of cerebrovascular disease	I00.I99	Estonia	81.01	2	yes
I69	Sequelae of cerebrovascular disease	I00.I99	Finland	1199.59	2	yes
I69	Sequelae of cerebrovascular disease	I00.I99	France	2345.75	2	yes
I69	Sequelae of cerebrovascular disease	I00.I99	Germany	5722.16	3	yes
I69	Sequelae of cerebrovascular disease	I00.I99	Hungary	1939.16	2	yes
I69	Sequelae of cerebrovascular disease	I00.I99	Latvia	356.62	2	yes
I69	Sequelae of cerebrovascular disease	I00.I99	Lithuania	524.68	3	yes
I69	Sequelae of cerebrovascular disease	I00.I99	Netherlands	1034.02	3	yes
I69	Sequelae of cerebrovascular disease	I00.I99	Norway	594.28	2	yes
I69	Sequelae of cerebrovascular disease	I00.I99	Poland	4755.96	1	yes
I69	Sequelae of cerebrovascular disease	I00.I99	Slovenia	545.15	0	no
I69	Sequelae of cerebrovascular disease	I00.I99	Spain	486.51	2	yes
I69	Sequelae of cerebrovascular disease	I00.I99	Sweden	1534.11	1	yes
I69	Sequelae of cerebrovascular disease	I00.I99	Switzerland	357.98	3	yes
I69	Sequelae of cerebrovascular disease	I00.I99	United Kingdom	3107.00	1	yes
I70	Atherosclerosis	I00.I99	Austria	899.14	3	yes
I70	Atherosclerosis	I00.I99	Belgium	443.52	2	yes
I70	Atherosclerosis	I00.I99	Croatia	1715.82	3	yes
I70	Atherosclerosis	I00.I99	Czech Republic	10307.78	1	yes
I70	Atherosclerosis	I00.I99	Denmark	768.15	3	yes
I70	Atherosclerosis	I00.I99	Estonia	310.65	3	yes
I70	Atherosclerosis	I00.I99	Finland	436.56	0	no
I70	Atherosclerosis	I00.I99	France	716.86	2	yes
I70	Atherosclerosis	I00.I99	Germany	11478.56	3	yes
I70	Atherosclerosis	I00.I99	Hungary	5403.13	2	yes
I70	Atherosclerosis	I00.I99	Latvia	993.74	3	yes
I70	Atherosclerosis	I00.I99	Lithuania	892.43	3	yes
I70	Atherosclerosis	I00.I99	Netherlands	1574.94	1	yes
I70	Atherosclerosis	I00.I99	Norway	328.23	2	yes
I70	Atherosclerosis	I00.I99	Poland	55059.60	1	yes
I70	Atherosclerosis	I00.I99	Romania	24108.14	3	yes
I70	Atherosclerosis	I00.I99	Slovenia	391.79	1	yes
I70	Atherosclerosis	I00.I99	Spain	3058.62	2	yes
I70	Atherosclerosis	I00.I99	Sweden	1114.11	3	yes
I70	Atherosclerosis	I00.I99	Switzerland	720.35	3	yes
I70	Atherosclerosis	I00.I99	United Kingdom	787.84	2	yes
I71	Aortic aneurysm and dissection	I00.I99	Austria	383.00	2	yes
I71	Aortic aneurysm and dissection	I00.I99	Belgium	704.02	1	yes
I71	Aortic aneurysm and dissection	I00.I99	Croatia	314.53	1	yes
I71	Aortic aneurysm and dissection	I00.I99	Czech Republic	628.13	1	yes
I71	Aortic aneurysm and dissection	I00.I99	Denmark	627.28	1	yes
I71	Aortic aneurysm and dissection	I00.I99	Estonia	85.08	0	no
I71	Aortic aneurysm and dissection	I00.I99	Finland	621.21	2	yes
I71	Aortic aneurysm and dissection	I00.I99	France	3077.05	1	yes
I71	Aortic aneurysm and dissection	I00.I99	Germany	3740.29	1	yes
I71	Aortic aneurysm and dissection	I00.I99	Hungary	580.69	2	yes
I71	Aortic aneurysm and dissection	I00.I99	Latvia	105.00	1	yes
I71	Aortic aneurysm and dissection	I00.I99	Lithuania	173.47	0	no
I71	Aortic aneurysm and dissection	I00.I99	Netherlands	1717.91	1	yes

For peer review only

I71	Aortic aneurysm and dissection	I00.I99	Norway	578.33	1	yes
I71	Aortic aneurysm and dissection	I00.I99	Poland	2341.33	2	yes
I71	Aortic aneurysm and dissection	I00.I99	Romania	483.15	2	yes
I71	Aortic aneurysm and dissection	I00.I99	Slovenia	118.31	0	no
I71	Aortic aneurysm and dissection	I00.I99	Spain	2213.61	1	yes
I71	Aortic aneurysm and dissection	I00.I99	Sweden	1053.48	1	yes
I71	Aortic aneurysm and dissection	I00.I99	Switzerland	512.07	1	yes
I71	Aortic aneurysm and dissection	I00.I99	United Kingdom	9329.53	3	yes
I72	Other aneurysm	I00.I99	Austria	18.27	2	yes
I72	Other aneurysm	I00.I99	Belgium	84.62	1	yes
I72	Other aneurysm	I00.I99	Croatia	8.69	0	no
I72	Other aneurysm	I00.I99	Czech Republic	34.74	2	yes
I72	Other aneurysm	I00.I99	Denmark	20.89	0	no
I72	Other aneurysm	I00.I99	Finland	15.56	2	yes
I72	Other aneurysm	I00.I99	France	556.88	2	yes
I72	Other aneurysm	I00.I99	Germany	189.49	2	yes
I72	Other aneurysm	I00.I99	Hungary	26.26	0	no
I72	Other aneurysm	I00.I99	Lithuania	7.96	0	no
I72	Other aneurysm	I00.I99	Netherlands	293.28	1	yes
I72	Other aneurysm	I00.I99	Norway	14.23	0	no
I72	Other aneurysm	I00.I99	Poland	82.38	0	no
I72	Other aneurysm	I00.I99	Romania	103.57	3	yes
I72	Other aneurysm	I00.I99	Slovenia	6.27	0	no
I72	Other aneurysm	I00.I99	Spain	113.50	0	no
I72	Other aneurysm	I00.I99	Sweden	35.07	1	yes
I72	Other aneurysm	I00.I99	Switzerland	28.61	0	no
I72	Other aneurysm	I00.I99	United Kingdom	266.73	1	yes
I73	Other peripheral vascular diseases	I00.I99	Austria	664.09	1	yes
I73	Other peripheral vascular diseases	I00.I99	Belgium	78.30	3	yes
I73	Other peripheral vascular diseases	I00.I99	Czech Republic	8.32	1	yes
I73	Other peripheral vascular diseases	I00.I99	Denmark	11.58	2	yes
I73	Other peripheral vascular diseases	I00.I99	France	506.70	2	yes
I73	Other peripheral vascular diseases	I00.I99	Germany	4301.01	3	yes
I73	Other peripheral vascular diseases	I00.I99	Hungary	38.08	2	yes
I73	Other peripheral vascular diseases	I00.I99	Netherlands	253.32	1	yes
I73	Other peripheral vascular diseases	I00.I99	Norway	17.79	3	yes
I73	Other peripheral vascular diseases	I00.I99	Poland	37.73	2	yes
I73	Other peripheral vascular diseases	I00.I99	Romania	41.86	3	yes
I73	Other peripheral vascular diseases	I00.I99	Slovenia	8.57	0	no
I73	Other peripheral vascular diseases	I00.I99	Spain	697.44	2	yes
I73	Other peripheral vascular diseases	I00.I99	Sweden	157.34	2	yes
I73	Other peripheral vascular diseases	I00.I99	Switzerland	20.83	1	yes
I73	Other peripheral vascular diseases	I00.I99	United Kingdom	3044.49	3	yes
I74	Arterial embolism and thrombosis	I00.I99	Austria	35.17	0	no
I74	Arterial embolism and thrombosis	I00.I99	Belgium	188.61	2	yes
I74	Arterial embolism and thrombosis	I00.I99	Croatia	73.99	3	yes
I74	Arterial embolism and thrombosis	I00.I99	Czech Republic	176.86	0	no
I74	Arterial embolism and thrombosis	I00.I99	Denmark	57.02	1	yes
I74	Arterial embolism and thrombosis	I00.I99	Estonia	29.06	0	no
I74	Arterial embolism and thrombosis	I00.I99	Finland	65.45	2	yes

For peer review only

I74	Arterial embolism and thrombosis	I00.I99	France	1404.73	2	yes
I74	Arterial embolism and thrombosis	I00.I99	Germany	2136.50	1	yes
I74	Arterial embolism and thrombosis	I00.I99	Hungary	302.34	2	yes
I74	Arterial embolism and thrombosis	I00.I99	Latvia	70.89	2	yes
I74	Arterial embolism and thrombosis	I00.I99	Lithuania	82.64	0	no
I74	Arterial embolism and thrombosis	I00.I99	Netherlands	298.87	1	yes
I74	Arterial embolism and thrombosis	I00.I99	Norway	39.20	2	yes
I74	Arterial embolism and thrombosis	I00.I99	Poland	855.25	3	yes
I74	Arterial embolism and thrombosis	I00.I99	Romania	101.19	0	no
I74	Arterial embolism and thrombosis	I00.I99	Slovenia	45.60	1	yes
I74	Arterial embolism and thrombosis	I00.I99	Spain	360.73	1	yes
I74	Arterial embolism and thrombosis	I00.I99	Sweden	72.98	3	yes
I74	Arterial embolism and thrombosis	I00.I99	Switzerland	100.73	1	yes
I74	Arterial embolism and thrombosis	I00.I99	United Kingdom	195.43	2	yes
I77	Other disorders of arteries and arterioles	I00.I99	Austria	7.14	1	yes
I77	Other disorders of arteries and arterioles	I00.I99	Belgium	224.52	0	no
I77	Other disorders of arteries and arterioles	I00.I99	Czech Republic	15.16	1	yes
I77	Other disorders of arteries and arterioles	I00.I99	Denmark	12.59	0	no
I77	Other disorders of arteries and arterioles	I00.I99	Finland	5.52	1	yes
I77	Other disorders of arteries and arterioles	I00.I99	France	4062.33	3	yes
I77	Other disorders of arteries and arterioles	I00.I99	Germany	150.67	0	no
I77	Other disorders of arteries and arterioles	I00.I99	Hungary	57.71	0	no
I77	Other disorders of arteries and arterioles	I00.I99	Netherlands	72.10	1	yes
I77	Other disorders of arteries and arterioles	I00.I99	Norway	15.96	1	yes
I77	Other disorders of arteries and arterioles	I00.I99	Poland	19.22	1	yes
I77	Other disorders of arteries and arterioles	I00.I99	Romania	245.97	2	yes
I77	Other disorders of arteries and arterioles	I00.I99	Spain	451.62	0	no
I77	Other disorders of arteries and arterioles	I00.I99	Sweden	70.02	0	no
I77	Other disorders of arteries and arterioles	I00.I99	Switzerland	50.76	0	no
I77	Other disorders of arteries and arterioles	I00.I99	United Kingdom	333.28	2	yes
I78	Diseases of capillaries	I00.I99	France	34.65	0	no
I78	Diseases of capillaries	I00.I99	Germany	30.23	3	yes
I78	Diseases of capillaries	I00.I99	Netherlands	5.96	0	no
I78	Diseases of capillaries	I00.I99	Spain	13.32	0	no
I78	Diseases of capillaries	I00.I99	United Kingdom	11.19	0	no
I80	Phlebitis and thrombophlebitis	I00.I99	Austria	324.28	3	yes
I80	Phlebitis and thrombophlebitis	I00.I99	Belgium	159.13	2	yes
I80	Phlebitis and thrombophlebitis	I00.I99	Croatia	31.36	1	yes
I80	Phlebitis and thrombophlebitis	I00.I99	Czech Republic	434.17	1	yes
I80	Phlebitis and thrombophlebitis	I00.I99	Denmark	45.39	1	yes
I80	Phlebitis and thrombophlebitis	I00.I99	Estonia	50.18	1	yes
I80	Phlebitis and thrombophlebitis	I00.I99	Finland	166.39	0	no
I80	Phlebitis and thrombophlebitis	I00.I99	France	1089.59	1	yes
I80	Phlebitis and thrombophlebitis	I00.I99	Germany	2359.64	3	yes
I80	Phlebitis and thrombophlebitis	I00.I99	Hungary	694.52	2	yes
I80	Phlebitis and thrombophlebitis	I00.I99	Latvia	45.36	0	no
I80	Phlebitis and thrombophlebitis	I00.I99	Lithuania	113.70	3	yes
I80	Phlebitis and thrombophlebitis	I00.I99	Netherlands	113.27	2	yes
I80	Phlebitis and thrombophlebitis	I00.I99	Norway	54.75	2	yes
I80	Phlebitis and thrombophlebitis	I00.I99	Poland	217.33	2	yes

For peer review only

I80	Phlebitis and thrombophlebitis	I00.I99	Romania	44.44	0	no
I80	Phlebitis and thrombophlebitis	I00.I99	Slovenia	75.75	3	yes
I80	Phlebitis and thrombophlebitis	I00.I99	Spain	359.72	1	yes
I80	Phlebitis and thrombophlebitis	I00.I99	Sweden	92.29	2	yes
I80	Phlebitis and thrombophlebitis	I00.I99	Switzerland	79.63	2	yes
I80	Phlebitis and thrombophlebitis	I00.I99	United Kingdom	4177.04	1	yes
I81	Portal vein thrombosis	I00.I99	Czech Republic	8.12	0	no
I81	Portal vein thrombosis	I00.I99	France	36.48	0	no
I81	Portal vein thrombosis	I00.I99	Germany	29.91	1	yes
I81	Portal vein thrombosis	I00.I99	Hungary	11.74	0	no
I81	Portal vein thrombosis	I00.I99	Netherlands	5.68	0	no
I81	Portal vein thrombosis	I00.I99	Poland	12.08	0	no
I81	Portal vein thrombosis	I00.I99	Romania	5.86	0	no
I81	Portal vein thrombosis	I00.I99	Spain	10.05	2	yes
I81	Portal vein thrombosis	I00.I99	Sweden	6.94	0	no
I81	Portal vein thrombosis	I00.I99	United Kingdom	37.14	1	yes
I82	Other venous embolism and thrombosis	I00.I99	Austria	15.86	0	no
I82	Other venous embolism and thrombosis	I00.I99	Belgium	32.57	1	yes
I82	Other venous embolism and thrombosis	I00.I99	Croatia	5.49	2	yes
I82	Other venous embolism and thrombosis	I00.I99	Czech Republic	78.25	1	yes
I82	Other venous embolism and thrombosis	I00.I99	Denmark	19.51	3	yes
I82	Other venous embolism and thrombosis	I00.I99	France	75.04	1	yes
I82	Other venous embolism and thrombosis	I00.I99	Germany	319.29	1	yes
I82	Other venous embolism and thrombosis	I00.I99	Hungary	66.34	3	yes
I82	Other venous embolism and thrombosis	I00.I99	Netherlands	23.22	1	yes
I82	Other venous embolism and thrombosis	I00.I99	Poland	42.65	0	no
I82	Other venous embolism and thrombosis	I00.I99	Romania	21.85	1	yes
I82	Other venous embolism and thrombosis	I00.I99	Slovenia	7.12	1	yes
I82	Other venous embolism and thrombosis	I00.I99	Spain	93.83	3	yes
I82	Other venous embolism and thrombosis	I00.I99	Sweden	24.06	0	no
I82	Other venous embolism and thrombosis	I00.I99	Switzerland	25.90	0	no
I82	Other venous embolism and thrombosis	I00.I99	United Kingdom	80.35	1	yes
I83	Varicose veins of lower extremities	I00.I99	Austria	18.47	0	no
I83	Varicose veins of lower extremities	I00.I99	Belgium	13.06	1	yes
I83	Varicose veins of lower extremities	I00.I99	Croatia	9.76	0	no
I83	Varicose veins of lower extremities	I00.I99	Czech Republic	79.15	0	no
I83	Varicose veins of lower extremities	I00.I99	Denmark	5.65	1	yes
I83	Varicose veins of lower extremities	I00.I99	Finland	7.53	0	no
I83	Varicose veins of lower extremities	I00.I99	France	101.62	1	yes
I83	Varicose veins of lower extremities	I00.I99	Germany	147.06	2	yes
I83	Varicose veins of lower extremities	I00.I99	Hungary	66.28	0	no
I83	Varicose veins of lower extremities	I00.I99	Latvia	6.94	0	no
I83	Varicose veins of lower extremities	I00.I99	Lithuania	5.24	0	no
I83	Varicose veins of lower extremities	I00.I99	Netherlands	6.43	0	no
I83	Varicose veins of lower extremities	I00.I99	Poland	45.58	1	yes
I83	Varicose veins of lower extremities	I00.I99	Slovenia	5.43	0	no
I83	Varicose veins of lower extremities	I00.I99	Spain	31.17	0	no
I83	Varicose veins of lower extremities	I00.I99	Sweden	6.77	0	no
I83	Varicose veins of lower extremities	I00.I99	Switzerland	14.26	1	yes
I83	Varicose veins of lower extremities	I00.I99	United Kingdom	133.65	0	no

For peer review only

I84	Haemorrhoids	I00.I99	Germany	5.44	2	yes
I85	Oesophageal varices	I00.I99	Austria	7.07	0	no
I85	Oesophageal varices	I00.I99	Belgium	24.35	0	no
I85	Oesophageal varices	I00.I99	Croatia	19.94	0	no
I85	Oesophageal varices	I00.I99	Czech Republic	41.42	1	yes
I85	Oesophageal varices	I00.I99	Denmark	8.14	1	yes
I85	Oesophageal varices	I00.I99	France	127.54	1	yes
I85	Oesophageal varices	I00.I99	Germany	233.52	1	yes
I85	Oesophageal varices	I00.I99	Lithuania	11.07	1	yes
I85	Oesophageal varices	I00.I99	Netherlands	13.61	1	yes
I85	Oesophageal varices	I00.I99	Poland	59.18	2	yes
I85	Oesophageal varices	I00.I99	Spain	74.33	1	yes
I85	Oesophageal varices	I00.I99	Sweden	8.79	0	no
I85	Oesophageal varices	I00.I99	Switzerland	7.01	0	no
I85	Oesophageal varices	I00.I99	United Kingdom	51.82	1	yes
I86	Varicose veins of other sites	I00.I99	France	9.98	0	no
I86	Varicose veins of other sites	I00.I99	Germany	7.36	0	no
I87	Other disorders of veins	I00.I99	Austria	6.40	0	no
I87	Other disorders of veins	I00.I99	Belgium	9.22	0	no
I87	Other disorders of veins	I00.I99	Czech Republic	6.09	0	no
I87	Other disorders of veins	I00.I99	France	82.30	3	yes
I87	Other disorders of veins	I00.I99	Germany	76.88	1	yes
I87	Other disorders of veins	I00.I99	Hungary	16.09	1	yes
I87	Other disorders of veins	I00.I99	Netherlands	6.71	0	no
I87	Other disorders of veins	I00.I99	Spain	74.16	0	no
I87	Other disorders of veins	I00.I99	Switzerland	17.45	0	no
I87	Other disorders of veins	I00.I99	United Kingdom	29.05	1	yes
I89	Other noninfective disorders of lymphatic vessels and lymph nodes	I00.I99	France	28.71	1	yes
I89	Other noninfective disorders of lymphatic vessels and lymph nodes	I00.I99	Germany	19.45	1	yes
I89	Other noninfective disorders of lymphatic vessels and lymph nodes	I00.I99	Spain	9.32	0	no
I89	Other noninfective disorders of lymphatic vessels and lymph nodes	I00.I99	United Kingdom	31.60	0	no
I95	Hypotension	I00.I99	Belgium	8.61	3	yes
I95	Hypotension	I00.I99	Denmark	9.36	0	no
I95	Hypotension	I00.I99	France	20.39	0	no
I95	Hypotension	I00.I99	Germany	19.11	2	yes
I95	Hypotension	I00.I99	Netherlands	6.36	0	no
I95	Hypotension	I00.I99	Spain	11.59	0	no
I95	Hypotension	I00.I99	United Kingdom	8.00	0	no
I99	Other and unspecified disorders of circulatory system	I00.I99	Belgium	55.45	1	yes
I99	Other and unspecified disorders of circulatory system	I00.I99	Denmark	7.82	0	no
I99	Other and unspecified disorders of circulatory system	I00.I99	France	181.14	1	yes
I99	Other and unspecified disorders of circulatory system	I00.I99	Germany	74.12	1	yes
I99	Other and unspecified disorders of circulatory system	I00.I99	Netherlands	46.25	1	yes
I99	Other and unspecified disorders of circulatory system	I00.I99	Norway	9.01	1	yes
I99	Other and unspecified disorders of circulatory system	I00.I99	Poland	33.67	0	no
I99	Other and unspecified disorders of circulatory system	I00.I99	Romania	1360.42	3	yes
I99	Other and unspecified disorders of circulatory system	I00.I99	Spain	90.81	1	yes

For peer review only

I99	Other and unspecified disorders of circulatory system	I00.I99	Sweden	23.42	0	no
I99	Other and unspecified disorders of circulatory system	I00.I99	Switzerland	12.71	2	yes
I99	Other and unspecified disorders of circulatory system	I00.I99	United Kingdom	23.10	1	yes
J00	Acute nasopharyngitis [common cold]	J00.J99	Spain	12.34	0	no
J02	Acute pharyngitis	J00.J99	France	10.25	1	yes
J02	Acute pharyngitis	J00.J99	United Kingdom	5.62	0	no
J03	Acute tonsillitis	J00.J99	Germany	9.01	0	no
J04	Acute laryngitis and tracheitis	J00.J99	Czech Republic	5.45	1	yes
J04	Acute laryngitis and tracheitis	J00.J99	France	7.61	1	yes
J04	Acute laryngitis and tracheitis	J00.J99	Germany	10.14	0	no
J04	Acute laryngitis and tracheitis	J00.J99	United Kingdom	5.34	0	no
J05	Acute obstructive laryngitis [croup] and epiglottitis	J00.J99	United Kingdom	11.94	0	no
J06	Acute upper respiratory infections of multiple or unspecified sites	J00.J99	Austria	14.00	2	yes
J06	Acute upper respiratory infections of multiple or unspecified sites	J00.J99	Belgium	13.50	1	yes
J06	Acute upper respiratory infections of multiple or unspecified sites	J00.J99	Czech Republic	7.62	0	no
J06	Acute upper respiratory infections of multiple or unspecified sites	J00.J99	Germany	171.62	1	yes
J06	Acute upper respiratory infections of multiple or unspecified sites	J00.J99	Netherlands	29.65	3	yes
J06	Acute upper respiratory infections of multiple or unspecified sites	J00.J99	Poland	7.94	0	no
J06	Acute upper respiratory infections of multiple or unspecified sites	J00.J99	Spain	18.91	0	no
J06	Acute upper respiratory infections of multiple or unspecified sites	J00.J99	Sweden	19.21	0	no
J06	Acute upper respiratory infections of multiple or unspecified sites	J00.J99	Switzerland	25.54	1	yes
J06	Acute upper respiratory infections of multiple or unspecified sites	J00.J99	United Kingdom	22.04	1	yes
J10	Influenza due to identified influenza virus	J00.J99	Czech Republic	19.53	1	yes
J10	Influenza due to identified influenza virus	J00.J99	Finland	17.69	3	yes
J10	Influenza due to identified influenza virus	J00.J99	Germany	18.51	0	no
J10	Influenza due to identified influenza virus	J00.J99	Netherlands	15.93	2	yes
J10	Influenza due to identified influenza virus	J00.J99	Romania	16.16	0	no
J10	Influenza due to identified influenza virus	J00.J99	Sweden	13.84	2	yes
J10	Influenza due to identified influenza virus	J00.J99	United Kingdom	43.98	2	yes
J11	Influenza, virus not identified	J00.J99	Belgium	133.14	2	yes
J11	Influenza, virus not identified	J00.J99	Croatia	27.71	0	no
J11	Influenza, virus not identified	J00.J99	Czech Republic	66.95	0	no
J11	Influenza, virus not identified	J00.J99	Denmark	31.24	1	yes
J11	Influenza, virus not identified	J00.J99	Finland	22.30	1	yes
J11	Influenza, virus not identified	J00.J99	France	492.88	0	no
J11	Influenza, virus not identified	J00.J99	Germany	156.01	0	no
J11	Influenza, virus not identified	J00.J99	Hungary	13.50	0	no
J11	Influenza, virus not identified	J00.J99	Netherlands	169.67	0	no
J11	Influenza, virus not identified	J00.J99	Norway	39.21	0	no
J11	Influenza, virus not identified	J00.J99	Poland	48.77	1	yes
J11	Influenza, virus not identified	J00.J99	Romania	11.09	2	yes
J11	Influenza, virus not identified	J00.J99	Spain	177.00	0	no
J11	Influenza, virus not identified	J00.J99	Sweden	89.15	0	no
J11	Influenza, virus not identified	J00.J99	Switzerland	74.26	2	yes

For peer review only

J11	Influenza, virus not identified	J00.J99	United Kingdom	58.66	2	yes
J12	Viral pneumonia, not elsewhere classified	J00.J99	Belgium	18.64	1	yes
J12	Viral pneumonia, not elsewhere classified	J00.J99	Czech Republic	35.60	2	yes
J12	Viral pneumonia, not elsewhere classified	J00.J99	Denmark	22.90	0	no
J12	Viral pneumonia, not elsewhere classified	J00.J99	Estonia	5.68	2	yes
J12	Viral pneumonia, not elsewhere classified	J00.J99	Finland	5.84	2	yes
J12	Viral pneumonia, not elsewhere classified	J00.J99	France	31.44	1	yes
J12	Viral pneumonia, not elsewhere classified	J00.J99	Germany	44.61	1	yes
J12	Viral pneumonia, not elsewhere classified	J00.J99	Hungary	42.22	2	yes
J12	Viral pneumonia, not elsewhere classified	J00.J99	Netherlands	38.56	2	yes
J12	Viral pneumonia, not elsewhere classified	J00.J99	Poland	18.89	0	no
J12	Viral pneumonia, not elsewhere classified	J00.J99	Romania	49.74	1	yes
J12	Viral pneumonia, not elsewhere classified	J00.J99	Spain	9.49	0	no
J12	Viral pneumonia, not elsewhere classified	J00.J99	Sweden	9.46	1	yes
J12	Viral pneumonia, not elsewhere classified	J00.J99	Switzerland	6.26	2	yes
J12	Viral pneumonia, not elsewhere classified	J00.J99	United Kingdom	32.11	2	yes
J13	Pneumonia due to Streptococcus pneumoniae	J00.J99	Belgium	51.52	1	yes
J13	Pneumonia due to Streptococcus pneumoniae	J00.J99	Czech Republic	11.91	2	yes
J13	Pneumonia due to Streptococcus pneumoniae	J00.J99	Denmark	30.58	2	yes
J13	Pneumonia due to Streptococcus pneumoniae	J00.J99	Estonia	27.52	3	yes
J13	Pneumonia due to Streptococcus pneumoniae	J00.J99	Finland	21.94	2	yes
J13	Pneumonia due to Streptococcus pneumoniae	J00.J99	France	343.01	2	yes
J13	Pneumonia due to Streptococcus pneumoniae	J00.J99	Germany	65.41	2	yes
J13	Pneumonia due to Streptococcus pneumoniae	J00.J99	Netherlands	56.74	1	yes
J13	Pneumonia due to Streptococcus pneumoniae	J00.J99	Norway	7.35	0	no
J13	Pneumonia due to Streptococcus pneumoniae	J00.J99	Poland	22.16	3	yes
J13	Pneumonia due to Streptococcus pneumoniae	J00.J99	Spain	173.86	2	yes
J13	Pneumonia due to Streptococcus pneumoniae	J00.J99	Sweden	20.38	1	yes
J13	Pneumonia due to Streptococcus pneumoniae	J00.J99	Switzerland	42.12	1	yes
J13	Pneumonia due to Streptococcus pneumoniae	J00.J99	United Kingdom	126.90	0	no
J14	Pneumonia due to Haemophilus influenzae	J00.J99	United Kingdom	7.65	0	no
J15	Bacterial pneumonia, not elsewhere classified	J00.J99	Austria	7.34	0	no
J15	Bacterial pneumonia, not elsewhere classified	J00.J99	Belgium	243.19	3	yes
J15	Bacterial pneumonia, not elsewhere classified	J00.J99	Croatia	19.47	1	yes
J15	Bacterial pneumonia, not elsewhere classified	J00.J99	Czech Republic	330.87	0	no
J15	Bacterial pneumonia, not elsewhere classified	J00.J99	Denmark	557.30	3	yes
J15	Bacterial pneumonia, not elsewhere classified	J00.J99	Estonia	148.37	2	yes
J15	Bacterial pneumonia, not elsewhere classified	J00.J99	Finland	340.50	2	yes
J15	Bacterial pneumonia, not elsewhere classified	J00.J99	France	491.42	1	yes
J15	Bacterial pneumonia, not elsewhere classified	J00.J99	Germany	523.37	0	no
J15	Bacterial pneumonia, not elsewhere classified	J00.J99	Hungary	220.61	2	yes
J15	Bacterial pneumonia, not elsewhere classified	J00.J99	Lithuania	69.58	0	no
J15	Bacterial pneumonia, not elsewhere classified	J00.J99	Netherlands	37.92	1	yes
J15	Bacterial pneumonia, not elsewhere classified	J00.J99	Norway	14.11	0	no
J15	Bacterial pneumonia, not elsewhere classified	J00.J99	Poland	1525.79	2	yes
J15	Bacterial pneumonia, not elsewhere classified	J00.J99	Romania	53.13	3	yes
J15	Bacterial pneumonia, not elsewhere classified	J00.J99	Slovenia	105.01	1	yes
J15	Bacterial pneumonia, not elsewhere classified	J00.J99	Spain	226.82	1	yes
J15	Bacterial pneumonia, not elsewhere classified	J00.J99	Sweden	37.38	3	yes
J15	Bacterial pneumonia, not elsewhere classified	J00.J99	Switzerland	29.90	0	no

For peer review only

J15	Bacterial pneumonia, not elsewhere classified	J00.J99	United Kingdom	203.68	3	yes
J16	Pneumonia due to other infectious organisms, not elsewhere classified	J00.J99	Czech Republic	30.21	0	no
J16	Pneumonia due to other infectious organisms, not elsewhere classified	J00.J99	Germany	233.45	3	yes
J16	Pneumonia due to other infectious organisms, not elsewhere classified	J00.J99	Poland	58.92	2	yes
J16	Pneumonia due to other infectious organisms, not elsewhere classified	J00.J99	Romania	15.90	0	no
J18	Pneumonia, organism unspecified	J00.J99	Austria	1325.03	3	yes
J18	Pneumonia, organism unspecified	J00.J99	Belgium	4247.27	1	yes
J18	Pneumonia, organism unspecified	J00.J99	Croatia	1130.44	3	yes
J18	Pneumonia, organism unspecified	J00.J99	Czech Republic	3426.72	3	yes
J18	Pneumonia, organism unspecified	J00.J99	Denmark	1602.22	3	yes
J18	Pneumonia, organism unspecified	J00.J99	Estonia	50.22	0	no
J18	Pneumonia, organism unspecified	J00.J99	Finland	847.14	2	yes
J18	Pneumonia, organism unspecified	J00.J99	France	11093.98	1	yes
J18	Pneumonia, organism unspecified	J00.J99	Germany	20727.57	3	yes
J18	Pneumonia, organism unspecified	J00.J99	Hungary	750.86	3	yes
J18	Pneumonia, organism unspecified	J00.J99	Latvia	422.49	0	no
J18	Pneumonia, organism unspecified	J00.J99	Lithuania	548.04	3	yes
J18	Pneumonia, organism unspecified	J00.J99	Netherlands	6736.34	2	yes
J18	Pneumonia, organism unspecified	J00.J99	Norway	1989.06	2	yes
J18	Pneumonia, organism unspecified	J00.J99	Poland	13089.40	0	no
J18	Pneumonia, organism unspecified	J00.J99	Romania	7361.71	2	yes
J18	Pneumonia, organism unspecified	J00.J99	Slovenia	817.32	1	yes
J18	Pneumonia, organism unspecified	J00.J99	Spain	9094.64	1	yes
J18	Pneumonia, organism unspecified	J00.J99	Sweden	2093.71	2	yes
J18	Pneumonia, organism unspecified	J00.J99	Switzerland	1525.09	1	yes
J18	Pneumonia, organism unspecified	J00.J99	United Kingdom	36743.70	3	yes
J20	Acute bronchitis	J00.J99	Austria	9.05	2	yes
J20	Acute bronchitis	J00.J99	Belgium	204.22	1	yes
J20	Acute bronchitis	J00.J99	Croatia	7.64	1	yes
J20	Acute bronchitis	J00.J99	Czech Republic	156.80	2	yes
J20	Acute bronchitis	J00.J99	Denmark	10.78	0	no
J20	Acute bronchitis	J00.J99	Finland	7.94	1	yes
J20	Acute bronchitis	J00.J99	France	2866.18	1	yes
J20	Acute bronchitis	J00.J99	Germany	471.63	3	yes
J20	Acute bronchitis	J00.J99	Hungary	149.91	2	yes
J20	Acute bronchitis	J00.J99	Netherlands	47.73	0	no
J20	Acute bronchitis	J00.J99	Poland	37.24	1	yes
J20	Acute bronchitis	J00.J99	Romania	12.87	1	yes
J20	Acute bronchitis	J00.J99	Slovenia	16.58	1	yes
J20	Acute bronchitis	J00.J99	Spain	370.68	0	no
J20	Acute bronchitis	J00.J99	Sweden	36.11	1	yes
J20	Acute bronchitis	J00.J99	Switzerland	10.80	1	yes
J20	Acute bronchitis	J00.J99	United Kingdom	231.64	3	yes
J21	Acute bronchiolitis	J00.J99	Austria	6.20	1	yes
J21	Acute bronchiolitis	J00.J99	Czech Republic	11.69	1	yes
J21	Acute bronchiolitis	J00.J99	France	18.48	2	yes
J21	Acute bronchiolitis	J00.J99	Germany	11.90	1	yes

For peer review only

For peer review only - http://bmjopen.bmj.com/site/about/guidelines.xhtml

J21	Acute bronchiolitis	J00.J99	Hungary	18.97	0	no
J21	Acute bronchiolitis	J00.J99	Romania	22.65	1	yes
J21	Acute bronchiolitis	J00.J99	Spain	14.18	0	no
J21	Acute bronchiolitis	J00.J99	United Kingdom	21.80	0	no
J22	Unspecified acute lower respiratory infection	J00.J99	Austria	16.04	3	yes
J22	Unspecified acute lower respiratory infection	J00.J99	Belgium	264.83	1	yes
J22	Unspecified acute lower respiratory infection	J00.J99	Czech Republic	11.42	1	yes
J22	Unspecified acute lower respiratory infection	J00.J99	Denmark	8.37	1	yes
J22	Unspecified acute lower respiratory infection	J00.J99	Finland	5.95	1	yes
J22	Unspecified acute lower respiratory infection	J00.J99	France	368.08	2	yes
J22	Unspecified acute lower respiratory infection	J00.J99	Germany	274.41	2	yes
J22	Unspecified acute lower respiratory infection	J00.J99	Hungary	14.38	2	yes
J22	Unspecified acute lower respiratory infection	J00.J99	Netherlands	438.20	3	yes
J22	Unspecified acute lower respiratory infection	J00.J99	Norway	10.32	0	no
J22	Unspecified acute lower respiratory infection	J00.J99	Poland	27.20	1	yes
J22	Unspecified acute lower respiratory infection	J00.J99	Slovenia	47.81	2	yes
J22	Unspecified acute lower respiratory infection	J00.J99	Spain	860.55	0	no
J22	Unspecified acute lower respiratory infection	J00.J99	Sweden	42.59	2	yes
J22	Unspecified acute lower respiratory infection	J00.J99	Switzerland	15.77	0	no
J22	Unspecified acute lower respiratory infection	J00.J99	United Kingdom	2187.91	1	yes
J32	Chronic sinusitis	J00.J99	France	14.59	0	no
J32	Chronic sinusitis	J00.J99	Germany	7.27	2	yes
J32	Chronic sinusitis	J00.J99	United Kingdom	8.59	0	no
J38	Diseases of vocal cords and larynx, not elsewhere classified	J00.J99	Belgium	8.76	0	no
J38	Diseases of vocal cords and larynx, not elsewhere classified	J00.J99	France	61.96	1	yes
J38	Diseases of vocal cords and larynx, not elsewhere classified	J00.J99	Germany	16.99	0	no
J38	Diseases of vocal cords and larynx, not elsewhere classified	J00.J99	Netherlands	6.04	0	no
J38	Diseases of vocal cords and larynx, not elsewhere classified	J00.J99	Spain	21.46	1	yes
J38	Diseases of vocal cords and larynx, not elsewhere classified	J00.J99	United Kingdom	35.53	0	no
J39	Other diseases of upper respiratory tract	J00.J99	Belgium	11.16	0	no
J39	Other diseases of upper respiratory tract	J00.J99	France	44.69	0	no
J39	Other diseases of upper respiratory tract	J00.J99	Germany	34.42	0	no
J39	Other diseases of upper respiratory tract	J00.J99	Hungary	6.73	3	yes
J39	Other diseases of upper respiratory tract	J00.J99	Netherlands	10.81	0	no
J39	Other diseases of upper respiratory tract	J00.J99	Norway	5.26	1	yes
J39	Other diseases of upper respiratory tract	J00.J99	Spain	18.15	0	no
J39	Other diseases of upper respiratory tract	J00.J99	Sweden	8.90	0	no
J39	Other diseases of upper respiratory tract	J00.J99	United Kingdom	38.60	0	no
J40	Bronchitis, not specified as acute or chronic	J00.J99	Austria	82.82	1	yes
J40	Bronchitis, not specified as acute or chronic	J00.J99	Belgium	315.59	1	yes
J40	Bronchitis, not specified as acute or chronic	J00.J99	Czech Republic	51.84	3	yes
J40	Bronchitis, not specified as acute or chronic	J00.J99	Denmark	5.20	0	no
J40	Bronchitis, not specified as acute or chronic	J00.J99	France	500.02	1	yes
J40	Bronchitis, not specified as acute or chronic	J00.J99	Germany	506.78	0	no
J40	Bronchitis, not specified as acute or chronic	J00.J99	Hungary	94.39	2	yes
J40	Bronchitis, not specified as acute or chronic	J00.J99	Netherlands	42.83	2	yes

For peer review only

J40	Bronchitis, not specified as acute or chronic	J00.J99	Norway	8.60	0	no
J40	Bronchitis, not specified as acute or chronic	J00.J99	Poland	29.15	1	yes
J40	Bronchitis, not specified as acute or chronic	J00.J99	Spain	277.29	3	yes
J40	Bronchitis, not specified as acute or chronic	J00.J99	Sweden	14.55	0	no
J40	Bronchitis, not specified as acute or chronic	J00.J99	Switzerland	32.57	1	yes
J40	Bronchitis, not specified as acute or chronic	J00.J99	United Kingdom	88.16	2	yes
J41	Simple and mucopurulent chronic bronchitis	J00.J99	Austria	210.29	1	yes
J41	Simple and mucopurulent chronic bronchitis	J00.J99	Czech Republic	62.45	0	no
J41	Simple and mucopurulent chronic bronchitis	J00.J99	Denmark	12.99	2	yes
J41	Simple and mucopurulent chronic bronchitis	J00.J99	Germany	175.79	1	yes
J41	Simple and mucopurulent chronic bronchitis	J00.J99	Hungary	273.51	1	yes
J41	Simple and mucopurulent chronic bronchitis	J00.J99	Lithuania	12.35	2	yes
J41	Simple and mucopurulent chronic bronchitis	J00.J99	Poland	65.56	3	yes
J41	Simple and mucopurulent chronic bronchitis	J00.J99	Romania	39.61	1	yes
J41	Simple and mucopurulent chronic bronchitis	J00.J99	Sweden	6.93	2	yes
J41	Simple and mucopurulent chronic bronchitis	J00.J99	United Kingdom	42.80	2	yes
J42	Unspecified chronic bronchitis	J00.J99	Austria	34.89	3	yes
J42	Unspecified chronic bronchitis	J00.J99	Belgium	139.71	2	yes
J42	Unspecified chronic bronchitis	J00.J99	Croatia	43.37	3	yes
J42	Unspecified chronic bronchitis	J00.J99	Czech Republic	187.46	1	yes
J42	Unspecified chronic bronchitis	J00.J99	Denmark	207.41	2	yes
J42	Unspecified chronic bronchitis	J00.J99	Finland	5.51	2	yes
J42	Unspecified chronic bronchitis	J00.J99	France	641.47	2	yes
J42	Unspecified chronic bronchitis	J00.J99	Germany	576.34	2	yes
J42	Unspecified chronic bronchitis	J00.J99	Hungary	709.89	2	yes
J42	Unspecified chronic bronchitis	J00.J99	Latvia	20.75	0	no
J42	Unspecified chronic bronchitis	J00.J99	Lithuania	32.05	2	yes
J42	Unspecified chronic bronchitis	J00.J99	Netherlands	33.32	2	yes
J42	Unspecified chronic bronchitis	J00.J99	Norway	8.36	2	yes
J42	Unspecified chronic bronchitis	J00.J99	Poland	399.23	2	yes
J42	Unspecified chronic bronchitis	J00.J99	Romania	313.72	1	yes
J42	Unspecified chronic bronchitis	J00.J99	Slovenia	8.02	2	yes
J42	Unspecified chronic bronchitis	J00.J99	Spain	853.04	1	yes
J42	Unspecified chronic bronchitis	J00.J99	Sweden	25.84	3	yes
J42	Unspecified chronic bronchitis	J00.J99	Switzerland	41.42	2	yes
J42	Unspecified chronic bronchitis	J00.J99	United Kingdom	156.20	2	yes
J43	Emphysema	J00.J99	Austria	606.65	3	yes
J43	Emphysema	J00.J99	Belgium	385.09	2	yes
J43	Emphysema	J00.J99	Croatia	20.83	1	yes
J43	Emphysema	J00.J99	Czech Republic	133.77	3	yes
J43	Emphysema	J00.J99	Denmark	74.21	1	yes
J43	Emphysema	J00.J99	Finland	40.17	0	no
J43	Emphysema	J00.J99	France	676.32	2	yes
J43	Emphysema	J00.J99	Germany	1691.34	2	yes
J43	Emphysema	J00.J99	Hungary	1065.21	3	yes
J43	Emphysema	J00.J99	Latvia	6.43	0	no
J43	Emphysema	J00.J99	Lithuania	12.17	1	yes
J43	Emphysema	J00.J99	Netherlands	901.03	2	yes
J43	Emphysema	J00.J99	Norway	113.49	0	no
J43	Emphysema	J00.J99	Poland	192.37	2	yes

For peer review only

J43	Emphysema	J00.J99	Romania	1058.58	3	yes
J43	Emphysema	J00.J99	Slovenia	18.45	1	yes
J43	Emphysema	J00.J99	Spain	744.38	2	yes
J43	Emphysema	J00.J99	Sweden	260.34	1	yes
J43	Emphysema	J00.J99	Switzerland	44.21	2	yes
J43	Emphysema	J00.J99	United Kingdom	1530.25	2	yes
J44	Other chronic obstructive pulmonary disease	J00.J99	Austria	2039.82	1	yes
J44	Other chronic obstructive pulmonary disease	J00.J99	Belgium	4285.28	2	yes
J44	Other chronic obstructive pulmonary disease	J00.J99	Croatia	1588.64	1	yes
J44	Other chronic obstructive pulmonary disease	J00.J99	Czech Republic	2512.51	1	yes
J44	Other chronic obstructive pulmonary disease	J00.J99	Denmark	3655.94	3	yes
J44	Other chronic obstructive pulmonary disease	J00.J99	Estonia	208.53	0	no
J44	Other chronic obstructive pulmonary disease	J00.J99	Finland	1207.98	1	yes
J44	Other chronic obstructive pulmonary disease	J00.J99	France	6825.11	0	no
J44	Other chronic obstructive pulmonary disease	J00.J99	Germany	24056.18	1	yes
J44	Other chronic obstructive pulmonary disease	J00.J99	Hungary	3169.09	1	yes
J44	Other chronic obstructive pulmonary disease	J00.J99	Latvia	261.38	0	no
J44	Other chronic obstructive pulmonary disease	J00.J99	Lithuania	974.50	1	yes
J44	Other chronic obstructive pulmonary disease	J00.J99	Netherlands	7233.98	0	no
J44	Other chronic obstructive pulmonary disease	J00.J99	Norway	2075.94	2	yes
J44	Other chronic obstructive pulmonary disease	J00.J99	Poland	9255.15	2	yes
J44	Other chronic obstructive pulmonary disease	J00.J99	Romania	7041.54	2	yes
J44	Other chronic obstructive pulmonary disease	J00.J99	Slovenia	568.51	2	yes
J44	Other chronic obstructive pulmonary disease	J00.J99	Spain	14748.69	1	yes
J44	Other chronic obstructive pulmonary disease	J00.J99	Sweden	2406.12	1	yes
J44	Other chronic obstructive pulmonary disease	J00.J99	Switzerland	1895.75	0	no
J44	Other chronic obstructive pulmonary disease	J00.J99	United Kingdom	30852.18	2	yes
J45	Asthma	J00.J99	Austria	139.09	2	yes
J45	Asthma	J00.J99	Belgium	173.05	2	yes
J45	Asthma	J00.J99	Croatia	100.31	1	yes
J45	Asthma	J00.J99	Czech Republic	131.84	2	yes
J45	Asthma	J00.J99	Denmark	107.01	2	yes
J45	Asthma	J00.J99	Estonia	48.91	2	yes
J45	Asthma	J00.J99	Finland	107.29	1	yes
J45	Asthma	J00.J99	France	1061.87	2	yes
J45	Asthma	J00.J99	Germany	1624.06	2	yes
J45	Asthma	J00.J99	Hungary	192.99	3	yes
J45	Asthma	J00.J99	Latvia	60.62	2	yes
J45	Asthma	J00.J99	Lithuania	57.76	1	yes
J45	Asthma	J00.J99	Netherlands	92.67	2	yes
J45	Asthma	J00.J99	Norway	126.82	3	yes
J45	Asthma	J00.J99	Poland	761.50	2	yes
J45	Asthma	J00.J99	Romania	504.10	1	yes
J45	Asthma	J00.J99	Slovenia	33.18	2	yes
J45	Asthma	J00.J99	Spain	912.69	1	yes
J45	Asthma	J00.J99	Sweden	141.29	2	yes
J45	Asthma	J00.J99	Switzerland	124.12	2	yes
J45	Asthma	J00.J99	United Kingdom	1371.06	3	yes
J46	Status asthmaticus	J00.J99	Austria	10.57	3	yes
J46	Status asthmaticus	J00.J99	Belgium	29.83	2	yes

For peer review only

J46	Status asthmaticus	J00.J99	Croatia	5.76	1	yes
J46	Status asthmaticus	J00.J99	Czech Republic	8.26	0	no
J46	Status asthmaticus	J00.J99	Denmark	7.24	1	yes
J46	Status asthmaticus	J00.J99	Finland	5.57	0	no
J46	Status asthmaticus	J00.J99	France	142.90	2	yes
J46	Status asthmaticus	J00.J99	Germany	136.40	3	yes
J46	Status asthmaticus	J00.J99	Hungary	6.84	0	no
J46	Status asthmaticus	J00.J99	Netherlands	8.71	1	yes
J46	Status asthmaticus	J00.J99	Poland	134.72	2	yes
J46	Status asthmaticus	J00.J99	Romania	19.81	1	yes
J46	Status asthmaticus	J00.J99	Spain	98.85	3	yes
J46	Status asthmaticus	J00.J99	Sweden	10.08	3	yes
J46	Status asthmaticus	J00.J99	Switzerland	7.15	0	no
J46	Status asthmaticus	J00.J99	United Kingdom	59.14	2	yes
J47	Bronchiectasis	J00.J99	Belgium	33.42	0	no
J47	Bronchiectasis	J00.J99	Croatia	9.87	2	yes
J47	Bronchiectasis	J00.J99	Denmark	5.38	0	no
J47	Bronchiectasis	J00.J99	Finland	22.60	0	no
J47	Bronchiectasis	J00.J99	France	393.59	3	yes
J47	Bronchiectasis	J00.J99	Germany	28.31	2	yes
J47	Bronchiectasis	J00.J99	Hungary	236.05	3	yes
J47	Bronchiectasis	J00.J99	Lithuania	6.30	0	no
J47	Bronchiectasis	J00.J99	Netherlands	28.02	3	yes
J47	Bronchiectasis	J00.J99	Norway	7.15	0	no
J47	Bronchiectasis	J00.J99	Poland	23.53	1	yes
J47	Bronchiectasis	J00.J99	Romania	27.25	1	yes
J47	Bronchiectasis	J00.J99	Spain	445.43	0	no
J47	Bronchiectasis	J00.J99	Sweden	11.85	0	no
J47	Bronchiectasis	J00.J99	Switzerland	11.85	0	no
J47	Bronchiectasis	J00.J99	United Kingdom	1376.88	2	yes
J60	Coalworker's pneumoconiosis	J00.J99	Belgium	109.88	1	yes
J60	Coalworker's pneumoconiosis	J00.J99	Czech Republic	54.57	3	yes
J60	Coalworker's pneumoconiosis	J00.J99	France	13.59	1	yes
J60	Coalworker's pneumoconiosis	J00.J99	Germany	17.26	2	yes
J60	Coalworker's pneumoconiosis	J00.J99	Hungary	15.92	1	yes
J60	Coalworker's pneumoconiosis	J00.J99	Poland	29.54	3	yes
J60	Coalworker's pneumoconiosis	J00.J99	United Kingdom	159.29	2	yes
J61	Pneumoconiosis due to asbestos and other mineral fibres	J00.J99	Belgium	19.85	0	no
J61	Pneumoconiosis due to asbestos and other mineral fibres	J00.J99	Croatia	6.37	2	yes
J61	Pneumoconiosis due to asbestos and other mineral fibres	J00.J99	Denmark	9.83	1	yes
J61	Pneumoconiosis due to asbestos and other mineral fibres	J00.J99	Finland	25.66	0	no
J61	Pneumoconiosis due to asbestos and other mineral fibres	J00.J99	France	115.79	2	yes
J61	Pneumoconiosis due to asbestos and other mineral fibres	J00.J99	Germany	135.37	1	yes
J61	Pneumoconiosis due to asbestos and other mineral fibres	J00.J99	Netherlands	12.12	0	no
J61	Pneumoconiosis due to asbestos and other mineral fibres	J00.J99	Norway	12.42	1	yes

For peer review only

J61	Pneumoconiosis due to asbestos and other mineral fibres	J00.J99	Spain	20.84	1	yes
J61	Pneumoconiosis due to asbestos and other mineral fibres	J00.J99	Sweden	11.82	0	no
J61	Pneumoconiosis due to asbestos and other mineral fibres	J00.J99	United Kingdom	193.25	1	yes
J62	Pneumoconiosis due to dust containing silica	J00.J99	Austria	12.24	0	no
J62	Pneumoconiosis due to dust containing silica	J00.J99	Belgium	23.99	1	yes
J62	Pneumoconiosis due to dust containing silica	J00.J99	Czech Republic	10.93	2	yes
J62	Pneumoconiosis due to dust containing silica	J00.J99	France	401.19	2	yes
J62	Pneumoconiosis due to dust containing silica	J00.J99	Germany	294.49	1	yes
J62	Pneumoconiosis due to dust containing silica	J00.J99	Hungary	22.69	1	yes
J62	Pneumoconiosis due to dust containing silica	J00.J99	Netherlands	18.53	2	yes
J62	Pneumoconiosis due to dust containing silica	J00.J99	Poland	12.52	1	yes
J62	Pneumoconiosis due to dust containing silica	J00.J99	Romania	33.89	1	yes
J62	Pneumoconiosis due to dust containing silica	J00.J99	Spain	142.04	1	yes
J62	Pneumoconiosis due to dust containing silica	J00.J99	Sweden	5.34	1	yes
J62	Pneumoconiosis due to dust containing silica	J00.J99	Switzerland	11.30	1	yes
J62	Pneumoconiosis due to dust containing silica	J00.J99	United Kingdom	16.61	3	yes
J63	Pneumoconiosis due to other inorganic dusts	J00.J99	Germany	6.24	0	no
J63	Pneumoconiosis due to other inorganic dusts	J00.J99	Poland	16.78	1	yes
J64	Unspecified pneumoconiosis	J00.J99	Belgium	27.06	3	yes
J64	Unspecified pneumoconiosis	J00.J99	Czech Republic	5.59	0	no
J64	Unspecified pneumoconiosis	J00.J99	France	14.70	1	yes
J64	Unspecified pneumoconiosis	J00.J99	Germany	22.70	0	no
J64	Unspecified pneumoconiosis	J00.J99	Poland	61.43	2	yes
J64	Unspecified pneumoconiosis	J00.J99	Romania	26.56	1	yes
J64	Unspecified pneumoconiosis	J00.J99	Spain	87.34	1	yes
J64	Unspecified pneumoconiosis	J00.J99	United Kingdom	30.90	1	yes
J65	Pneumoconiosis associated with tuberculosis	J00.J99	France	8.39	3	yes
J65	Pneumoconiosis associated with tuberculosis	J00.J99	Germany	8.71	2	yes
J65	Pneumoconiosis associated with tuberculosis	J00.J99	Spain	6.94	1	yes
J67	Hypersensitivity pneumonitis due to organic dust	J00.J99	Austria	10.45	0	no
J67	Hypersensitivity pneumonitis due to organic dust	J00.J99	Belgium	7.22	0	no
J67	Hypersensitivity pneumonitis due to organic dust	J00.J99	Finland	5.53	0	no
J67	Hypersensitivity pneumonitis due to organic dust	J00.J99	France	38.75	0	no
J67	Hypersensitivity pneumonitis due to organic dust	J00.J99	Germany	47.63	1	yes
J67	Hypersensitivity pneumonitis due to organic dust	J00.J99	Netherlands	10.26	2	yes
J67	Hypersensitivity pneumonitis due to organic dust	J00.J99	Poland	5.79	1	yes
J67	Hypersensitivity pneumonitis due to organic dust	J00.J99	Romania	10.44	1	yes
J67	Hypersensitivity pneumonitis due to organic dust	J00.J99	Spain	8.63	1	yes
J67	Hypersensitivity pneumonitis due to organic dust	J00.J99	Switzerland	5.15	0	no
J67	Hypersensitivity pneumonitis due to organic dust	J00.J99	United Kingdom	68.04	2	yes
J68	Respiratory conditions due to inhalation of chemicals, gases, fumes and vapours	J00.J99	Germany	10.80	0	no
J69	Pneumonitis due to solids and liquids	J00.J99	Austria	106.51	0	no
J69	Pneumonitis due to solids and liquids	J00.J99	Belgium	827.98	2	yes
J69	Pneumonitis due to solids and liquids	J00.J99	Czech Republic	30.71	1	yes
J69	Pneumonitis due to solids and liquids	J00.J99	Denmark	49.30	1	yes
J69	Pneumonitis due to solids and liquids	J00.J99	Finland	28.22	2	yes
J69	Pneumonitis due to solids and liquids	J00.J99	France	2353.73	2	yes

For peer review only

J69	Pneumonitis due to solids and liquids	J00.J99	Germany	4354.24	3	yes
J69	Pneumonitis due to solids and liquids	J00.J99	Hungary	10.87	1	yes
J69	Pneumonitis due to solids and liquids	J00.J99	Netherlands	420.49	2	yes
J69	Pneumonitis due to solids and liquids	J00.J99	Norway	49.13	0	no
J69	Pneumonitis due to solids and liquids	J00.J99	Poland	305.79	3	yes
J69	Pneumonitis due to solids and liquids	J00.J99	Romania	20.73	1	yes
J69	Pneumonitis due to solids and liquids	J00.J99	Slovenia	69.85	2	yes
J69	Pneumonitis due to solids and liquids	J00.J99	Spain	1995.92	0	no
J69	Pneumonitis due to solids and liquids	J00.J99	Sweden	133.54	2	yes
J69	Pneumonitis due to solids and liquids	J00.J99	Switzerland	166.29	2	yes
J69	Pneumonitis due to solids and liquids	J00.J99	United Kingdom	3262.29	2	yes
J70	Respiratory conditions due to other external agents	J00.J99	Belgium	6.68	1	yes
J70	Respiratory conditions due to other external agents	J00.J99	Czech Republic	6.63	1	yes
J70	Respiratory conditions due to other external agents	J00.J99	Denmark	6.36	1	yes
J70	Respiratory conditions due to other external agents	J00.J99	France	74.31	2	yes
J70	Respiratory conditions due to other external agents	J00.J99	Germany	7.59	2	yes
J70	Respiratory conditions due to other external agents	J00.J99	Poland	53.03	1	yes
J80	Adult respiratory distress syndrome	J00.J99	Belgium	36.75	1	yes
J80	Adult respiratory distress syndrome	J00.J99	Czech Republic	11.10	0	no
J80	Adult respiratory distress syndrome	J00.J99	France	220.94	0	no
J80	Adult respiratory distress syndrome	J00.J99	Germany	50.35	2	yes
J80	Adult respiratory distress syndrome	J00.J99	Norway	5.23	0	no
J80	Adult respiratory distress syndrome	J00.J99	Poland	75.42	1	yes
J80	Adult respiratory distress syndrome	J00.J99	Spain	104.20	1	yes
J80	Adult respiratory distress syndrome	J00.J99	Switzerland	6.79	0	no
J80	Adult respiratory distress syndrome	J00.J99	United Kingdom	65.36	1	yes
J81	Pulmonary oedema	J00.J99	Austria	44.12	3	yes
J81	Pulmonary oedema	J00.J99	Belgium	312.60	1	yes
J81	Pulmonary oedema	J00.J99	Croatia	57.84	2	yes
J81	Pulmonary oedema	J00.J99	Czech Republic	56.47	3	yes
J81	Pulmonary oedema	J00.J99	Denmark	61.35	1	yes
J81	Pulmonary oedema	J00.J99	France	402.62	3	yes
J81	Pulmonary oedema	J00.J99	Germany	582.28	1	yes
J81	Pulmonary oedema	J00.J99	Netherlands	5.49	0	no
J81	Pulmonary oedema	J00.J99	Norway	38.98	1	yes
J81	Pulmonary oedema	J00.J99	Poland	538.32	3	yes
J81	Pulmonary oedema	J00.J99	Romania	859.45	0	no
J81	Pulmonary oedema	J00.J99	Spain	2769.46	1	yes
J81	Pulmonary oedema	J00.J99	Sweden	40.57	2	yes
J81	Pulmonary oedema	J00.J99	Switzerland	42.06	2	yes
J81	Pulmonary oedema	J00.J99	United Kingdom	112.37	2	yes
J82	Pulmonary eosinophilia, not elsewhere classified	J00.J99	France	8.66	1	yes
J82	Pulmonary eosinophilia, not elsewhere classified	J00.J99	Spain	16.54	0	no
J82	Pulmonary eosinophilia, not elsewhere classified	J00.J99	Sweden	5.88	1	yes
J84	Other interstitial pulmonary diseases	J00.J99	Austria	135.47	2	yes
J84	Other interstitial pulmonary diseases	J00.J99	Belgium	306.52	1	yes
J84	Other interstitial pulmonary diseases	J00.J99	Croatia	23.31	0	no
J84	Other interstitial pulmonary diseases	J00.J99	Czech Republic	227.70	1	yes
J84	Other interstitial pulmonary diseases	J00.J99	Denmark	150.21	2	yes
J84	Other interstitial pulmonary diseases	J00.J99	Estonia	16.18	0	no

For peer review only

J84	Other interstitial pulmonary diseases	J00.J99	Finland	252.81	1	yes
J84	Other interstitial pulmonary diseases	J00.J99	France	1520.31	2	yes
J84	Other interstitial pulmonary diseases	J00.J99	Germany	2271.04	1	yes
J84	Other interstitial pulmonary diseases	J00.J99	Hungary	184.99	2	yes
J84	Other interstitial pulmonary diseases	J00.J99	Latvia	18.79	0	no
J84	Other interstitial pulmonary diseases	J00.J99	Lithuania	11.44	1	yes
J84	Other interstitial pulmonary diseases	J00.J99	Netherlands	525.67	1	yes
J84	Other interstitial pulmonary diseases	J00.J99	Norway	154.80	1	yes
J84	Other interstitial pulmonary diseases	J00.J99	Poland	371.76	3	yes
J84	Other interstitial pulmonary diseases	J00.J99	Romania	285.35	1	yes
J84	Other interstitial pulmonary diseases	J00.J99	Slovenia	44.94	1	yes
J84	Other interstitial pulmonary diseases	J00.J99	Spain	2119.58	1	yes
J84	Other interstitial pulmonary diseases	J00.J99	Sweden	365.58	3	yes
J84	Other interstitial pulmonary diseases	J00.J99	Switzerland	222.74	1	yes
J84	Other interstitial pulmonary diseases	J00.J99	United Kingdom	4746.02	2	yes
J85	Abscess of lung and mediastinum	J00.J99	Austria	7.25	1	yes
J85	Abscess of lung and mediastinum	J00.J99	Belgium	17.02	0	no
J85	Abscess of lung and mediastinum	J00.J99	Croatia	8.96	1	yes
J85	Abscess of lung and mediastinum	J00.J99	Czech Republic	31.00	0	no
J85	Abscess of lung and mediastinum	J00.J99	Denmark	14.56	0	no
J85	Abscess of lung and mediastinum	J00.J99	Estonia	33.38	2	yes
J85	Abscess of lung and mediastinum	J00.J99	Finland	7.12	1	yes
J85	Abscess of lung and mediastinum	J00.J99	France	89.66	1	yes
J85	Abscess of lung and mediastinum	J00.J99	Germany	40.85	0	no
J85	Abscess of lung and mediastinum	J00.J99	Hungary	104.93	2	yes
J85	Abscess of lung and mediastinum	J00.J99	Latvia	32.77	0	no
J85	Abscess of lung and mediastinum	J00.J99	Lithuania	34.02	1	yes
J85	Abscess of lung and mediastinum	J00.J99	Netherlands	7.80	0	no
J85	Abscess of lung and mediastinum	J00.J99	Norway	9.57	1	yes
J85	Abscess of lung and mediastinum	J00.J99	Poland	116.61	1	yes
J85	Abscess of lung and mediastinum	J00.J99	Romania	197.81	1	yes
J85	Abscess of lung and mediastinum	J00.J99	Spain	50.96	2	yes
J85	Abscess of lung and mediastinum	J00.J99	Sweden	9.04	0	no
J85	Abscess of lung and mediastinum	J00.J99	United Kingdom	141.29	1	yes
J86	Pyothorax	J00.J99	Austria	17.26	0	no
J86	Pyothorax	J00.J99	Belgium	27.85	0	no
J86	Pyothorax	J00.J99	Croatia	9.75	1	yes
J86	Pyothorax	J00.J99	Czech Republic	18.81	0	no
J86	Pyothorax	J00.J99	Denmark	18.79	0	no
J86	Pyothorax	J00.J99	Finland	15.76	2	yes
J86	Pyothorax	J00.J99	France	113.22	1	yes
J86	Pyothorax	J00.J99	Germany	179.36	1	yes
J86	Pyothorax	J00.J99	Hungary	19.78	0	no
J86	Pyothorax	J00.J99	Latvia	6.11	0	no
J86	Pyothorax	J00.J99	Lithuania	23.21	0	no
J86	Pyothorax	J00.J99	Netherlands	36.40	2	yes
J86	Pyothorax	J00.J99	Norway	8.10	0	no
J86	Pyothorax	J00.J99	Poland	141.51	0	no
J86	Pyothorax	J00.J99	Romania	32.96	3	yes
J86	Pyothorax	J00.J99	Slovenia	6.28	0	no

For peer review only

J86	Pyothorax	J00.J99	Spain	99.18	1	yes
J86	Pyothorax	J00.J99	Sweden	19.72	0	no
J86	Pyothorax	J00.J99	Switzerland	15.05	0	no
J86	Pyothorax	J00.J99	United Kingdom	248.25	3	yes
J90	Pleural effusion, not elsewhere classified	J00.J99	Belgium	40.98	0	no
J90	Pleural effusion, not elsewhere classified	J00.J99	Czech Republic	8.93	2	yes
J90	Pleural effusion, not elsewhere classified	J00.J99	Denmark	18.27	0	no
J90	Pleural effusion, not elsewhere classified	J00.J99	Finland	6.31	1	yes
J90	Pleural effusion, not elsewhere classified	J00.J99	France	188.10	1	yes
J90	Pleural effusion, not elsewhere classified	J00.J99	Germany	152.86	2	yes
J90	Pleural effusion, not elsewhere classified	J00.J99	Netherlands	52.63	3	yes
J90	Pleural effusion, not elsewhere classified	J00.J99	Norway	7.81	2	yes
J90	Pleural effusion, not elsewhere classified	J00.J99	Poland	26.37	1	yes
J90	Pleural effusion, not elsewhere classified	J00.J99	Romania	51.95	2	yes
J90	Pleural effusion, not elsewhere classified	J00.J99	Spain	241.48	0	no
J90	Pleural effusion, not elsewhere classified	J00.J99	Sweden	11.33	0	no
J90	Pleural effusion, not elsewhere classified	J00.J99	Switzerland	10.55	0	no
J90	Pleural effusion, not elsewhere classified	J00.J99	United Kingdom	127.80	3	yes
J92	Pleural plaque	J00.J99	France	9.71	0	no
J92	Pleural plaque	J00.J99	Spain	17.06	0	no
J92	Pleural plaque	J00.J99	United Kingdom	11.86	1	yes
J93	Pneumothorax	J00.J99	Belgium	17.73	0	no
J93	Pneumothorax	J00.J99	Czech Republic	6.01	0	no
J93	Pneumothorax	J00.J99	Denmark	9.76	0	no
J93	Pneumothorax	J00.J99	France	58.78	1	yes
J93	Pneumothorax	J00.J99	Germany	49.71	2	yes
J93	Pneumothorax	J00.J99	Netherlands	19.00	1	yes
J93	Pneumothorax	J00.J99	Norway	5.97	1	yes
J93	Pneumothorax	J00.J99	Poland	66.71	1	yes
J93	Pneumothorax	J00.J99	Romania	18.02	0	no
J93	Pneumothorax	J00.J99	Spain	41.86	1	yes
J93	Pneumothorax	J00.J99	Sweden	6.56	0	no
J93	Pneumothorax	J00.J99	United Kingdom	49.87	1	yes
J94	Other pleural conditions	J00.J99	Belgium	11.49	0	no
J94	Other pleural conditions	J00.J99	Czech Republic	6.41	0	no
J94	Other pleural conditions	J00.J99	Denmark	9.63	1	yes
J94	Other pleural conditions	J00.J99	France	48.76	0	no
J94	Other pleural conditions	J00.J99	Germany	43.35	2	yes
J94	Other pleural conditions	J00.J99	Hungary	12.91	2	yes
J94	Other pleural conditions	J00.J99	Netherlands	10.35	0	no
J94	Other pleural conditions	J00.J99	Poland	13.59	0	no
J94	Other pleural conditions	J00.J99	Romania	66.85	1	yes
J94	Other pleural conditions	J00.J99	Slovenia	6.99	0	no
J94	Other pleural conditions	J00.J99	Spain	62.20	2	yes
J94	Other pleural conditions	J00.J99	United Kingdom	36.98	2	yes
J96	Respiratory failure, not elsewhere classified	J00.J99	Austria	6.42	0	no
J96	Respiratory failure, not elsewhere classified	J00.J99	Belgium	321.07	1	yes
J96	Respiratory failure, not elsewhere classified	J00.J99	Croatia	38.92	0	no
J96	Respiratory failure, not elsewhere classified	J00.J99	Czech Republic	178.44	3	yes
J96	Respiratory failure, not elsewhere classified	J00.J99	Denmark	131.03	1	yes

For peer review only

J96	Respiratory failure, not elsewhere classified	J00.J99	Finland	6.20	1	yes
J96	Respiratory failure, not elsewhere classified	J00.J99	France	4050.01	2	yes
J96	Respiratory failure, not elsewhere classified	J00.J99	Germany	1154.20	2	yes
J96	Respiratory failure, not elsewhere classified	J00.J99	Netherlands	183.36	3	yes
J96	Respiratory failure, not elsewhere classified	J00.J99	Norway	32.23	0	no
J96	Respiratory failure, not elsewhere classified	J00.J99	Poland	725.08	0	no
J96	Respiratory failure, not elsewhere classified	J00.J99	Romania	195.46	2	yes
J96	Respiratory failure, not elsewhere classified	J00.J99	Slovenia	8.76	2	yes
J96	Respiratory failure, not elsewhere classified	J00.J99	Spain	3142.76	3	yes
J96	Respiratory failure, not elsewhere classified	J00.J99	Sweden	24.45	2	yes
J96	Respiratory failure, not elsewhere classified	J00.J99	Switzerland	57.69	1	yes
J96	Respiratory failure, not elsewhere classified	J00.J99	United Kingdom	35.34	2	yes
J98	Other respiratory disorders	J00.J99	Austria	23.28	1	yes
J98	Other respiratory disorders	J00.J99	Belgium	373.80	3	yes
J98	Other respiratory disorders	J00.J99	Czech Republic	14.50	0	no
J98	Other respiratory disorders	J00.J99	Denmark	48.44	1	yes
J98	Other respiratory disorders	J00.J99	France	1425.54	2	yes
J98	Other respiratory disorders	J00.J99	Germany	336.27	1	yes
J98	Other respiratory disorders	J00.J99	Hungary	77.78	2	yes
J98	Other respiratory disorders	J00.J99	Netherlands	510.39	3	yes
J98	Other respiratory disorders	J00.J99	Norway	58.05	2	yes
J98	Other respiratory disorders	J00.J99	Poland	24.34	1	yes
J98	Other respiratory disorders	J00.J99	Romania	30.94	0	no
J98	Other respiratory disorders	J00.J99	Spain	8706.44	3	yes
J98	Other respiratory disorders	J00.J99	Sweden	90.77	2	yes
J98	Other respiratory disorders	J00.J99	Switzerland	86.65	0	no
J98	Other respiratory disorders	J00.J99	United Kingdom	6641.62	2	yes
K04	Diseases of pulp and periapical tissues	K00.K93	United Kingdom	6.15	0	no
K10	Other diseases of jaws	K00.K93	Netherlands	5.50	1	yes
K11	Diseases of salivary glands	K00.K93	Germany	17.98	3	yes
K11	Diseases of salivary glands	K00.K93	Netherlands	12.47	0	no
K11	Diseases of salivary glands	K00.K93	United Kingdom	36.25	0	no
K12	Stomatitis and related lesions	K00.K93	Germany	7.20	1	yes
K12	Stomatitis and related lesions	K00.K93	Spain	5.29	0	no
K12	Stomatitis and related lesions	K00.K93	United Kingdom	7.08	0	no
K13	Other diseases of lip and oral mucosa	K00.K93	France	11.55	0	no
K20	Oesophagitis	K00.K93	Belgium	32.86	1	yes
K20	Oesophagitis	K00.K93	Czech Republic	10.90	1	yes
K20	Oesophagitis	K00.K93	Denmark	5.67	0	no
K20	Oesophagitis	K00.K93	France	63.94	2	yes
K20	Oesophagitis	K00.K93	Germany	44.74	0	no
K20	Oesophagitis	K00.K93	Netherlands	9.86	1	yes
K20	Oesophagitis	K00.K93	Norway	7.96	1	yes
K20	Oesophagitis	K00.K93	Spain	27.03	0	no
K20	Oesophagitis	K00.K93	Sweden	12.60	0	no
K20	Oesophagitis	K00.K93	United Kingdom	117.07	1	yes
K21	Gastro-oesophageal reflux disease	K00.K93	Austria	8.40	0	no
K21	Gastro-oesophageal reflux disease	K00.K93	Belgium	9.17	0	no
K21	Gastro-oesophageal reflux disease	K00.K93	Czech Republic	9.24	1	yes
K21	Gastro-oesophageal reflux disease	K00.K93	France	44.86	1	yes

For peer review only

K21	Gastro-oesophageal reflux disease	K00.K93	Germany	214.64	3	yes
K21	Gastro-oesophageal reflux disease	K00.K93	Hungary	15.54	0	no
K21	Gastro-oesophageal reflux disease	K00.K93	Netherlands	9.44	0	no
K21	Gastro-oesophageal reflux disease	K00.K93	Spain	7.31	1	yes
K21	Gastro-oesophageal reflux disease	K00.K93	Switzerland	15.48	0	no
K21	Gastro-oesophageal reflux disease	K00.K93	United Kingdom	46.18	1	yes
K22	Other diseases of oesophagus	K00.K93	Austria	24.31	0	no
K22	Other diseases of oesophagus	K00.K93	Belgium	59.63	0	no
K22	Other diseases of oesophagus	K00.K93	Croatia	9.83	0	no
K22	Other diseases of oesophagus	K00.K93	Czech Republic	52.34	2	yes
K22	Other diseases of oesophagus	K00.K93	Denmark	40.48	2	yes
K22	Other diseases of oesophagus	K00.K93	Estonia	6.83	0	no
K22	Other diseases of oesophagus	K00.K93	Finland	38.56	0	no
K22	Other diseases of oesophagus	K00.K93	France	240.13	1	yes
K22	Other diseases of oesophagus	K00.K93	Germany	284.25	1	yes
K22	Other diseases of oesophagus	K00.K93	Hungary	65.51	0	no
K22	Other diseases of oesophagus	K00.K93	Latvia	11.87	0	no
K22	Other diseases of oesophagus	K00.K93	Lithuania	20.36	1	yes
K22	Other diseases of oesophagus	K00.K93	Netherlands	78.07	0	no
K22	Other diseases of oesophagus	K00.K93	Norway	24.89	0	no
K22	Other diseases of oesophagus	K00.K93	Poland	63.00	1	yes
K22	Other diseases of oesophagus	K00.K93	Romania	111.34	3	yes
K22	Other diseases of oesophagus	K00.K93	Slovenia	11.77	2	yes
K22	Other diseases of oesophagus	K00.K93	Spain	129.83	0	no
K22	Other diseases of oesophagus	K00.K93	Sweden	56.25	0	no
K22	Other diseases of oesophagus	K00.K93	Switzerland	31.95	0	no
K22	Other diseases of oesophagus	K00.K93	United Kingdom	620.20	0	no
K25	Gastric ulcer	K00.K93	Austria	106.96	1	yes
K25	Gastric ulcer	K00.K93	Belgium	213.70	3	yes
K25	Gastric ulcer	K00.K93	Croatia	154.09	0	no
K25	Gastric ulcer	K00.K93	Czech Republic	349.81	1	yes
K25	Gastric ulcer	K00.K93	Denmark	244.33	2	yes
K25	Gastric ulcer	K00.K93	Estonia	42.12	1	yes
K25	Gastric ulcer	K00.K93	Finland	125.56	1	yes
K25	Gastric ulcer	K00.K93	France	374.59	1	yes
K25	Gastric ulcer	K00.K93	Germany	1984.61	1	yes
K25	Gastric ulcer	K00.K93	Hungary	478.15	1	yes
K25	Gastric ulcer	K00.K93	Latvia	98.40	2	yes
K25	Gastric ulcer	K00.K93	Lithuania	160.58	0	no
K25	Gastric ulcer	K00.K93	Netherlands	242.65	2	yes
K25	Gastric ulcer	K00.K93	Norway	108.63	2	yes
K25	Gastric ulcer	K00.K93	Poland	1269.93	1	yes
K25	Gastric ulcer	K00.K93	Romania	340.33	3	yes
K25	Gastric ulcer	K00.K93	Slovenia	62.96	2	yes
K25	Gastric ulcer	K00.K93	Spain	280.86	3	yes
K25	Gastric ulcer	K00.K93	Sweden	188.20	1	yes
K25	Gastric ulcer	K00.K93	Switzerland	94.01	3	yes
K25	Gastric ulcer	K00.K93	United Kingdom	982.61	3	yes
K26	Duodenal ulcer	K00.K93	Austria	109.68	2	yes
K26	Duodenal ulcer	K00.K93	Belgium	78.18	1	yes

For peer review only

K26	Duodenal ulcer	K00.K93	Croatia	114.91	1	yes
K26	Duodenal ulcer	K00.K93	Czech Republic	293.37	1	yes
K26	Duodenal ulcer	K00.K93	Denmark	170.49	1	yes
K26	Duodenal ulcer	K00.K93	Estonia	35.20	0	no
K26	Duodenal ulcer	K00.K93	Finland	92.32	1	yes
K26	Duodenal ulcer	K00.K93	France	305.05	2	yes
K26	Duodenal ulcer	K00.K93	Germany	1095.36	3	yes
K26	Duodenal ulcer	K00.K93	Hungary	458.17	2	yes
K26	Duodenal ulcer	K00.K93	Latvia	59.13	0	no
K26	Duodenal ulcer	K00.K93	Lithuania	127.56	1	yes
K26	Duodenal ulcer	K00.K93	Netherlands	87.02	2	yes
K26	Duodenal ulcer	K00.K93	Norway	72.62	1	yes
K26	Duodenal ulcer	K00.K93	Poland	678.38	3	yes
K26	Duodenal ulcer	K00.K93	Romania	337.10	2	yes
K26	Duodenal ulcer	K00.K93	Slovenia	54.88	1	yes
K26	Duodenal ulcer	K00.K93	Spain	238.03	2	yes
K26	Duodenal ulcer	K00.K93	Sweden	104.93	1	yes
K26	Duodenal ulcer	K00.K93	Switzerland	68.07	1	yes
K26	Duodenal ulcer	K00.K93	United Kingdom	2112.32	1	yes
K27	Peptic ulcer, site unspecified	K00.K93	Belgium	26.48	2	yes
K27	Peptic ulcer, site unspecified	K00.K93	Croatia	6.90	1	yes
K27	Peptic ulcer, site unspecified	K00.K93	Czech Republic	36.99	2	yes
K27	Peptic ulcer, site unspecified	K00.K93	Denmark	74.65	1	yes
K27	Peptic ulcer, site unspecified	K00.K93	Finland	7.06	2	yes
K27	Peptic ulcer, site unspecified	K00.K93	France	278.75	2	yes
K27	Peptic ulcer, site unspecified	K00.K93	Germany	115.60	1	yes
K27	Peptic ulcer, site unspecified	K00.K93	Hungary	7.91	1	yes
K27	Peptic ulcer, site unspecified	K00.K93	Netherlands	14.50	3	yes
K27	Peptic ulcer, site unspecified	K00.K93	Norway	52.07	2	yes
K27	Peptic ulcer, site unspecified	K00.K93	Poland	411.42	2	yes
K27	Peptic ulcer, site unspecified	K00.K93	Romania	52.25	0	no
K27	Peptic ulcer, site unspecified	K00.K93	Spain	105.05	2	yes
K27	Peptic ulcer, site unspecified	K00.K93	Sweden	76.02	0	no
K27	Peptic ulcer, site unspecified	K00.K93	Switzerland	24.76	1	yes
K27	Peptic ulcer, site unspecified	K00.K93	United Kingdom	507.37	2	yes
K28	Gastrojejunal ulcer	K00.K93	Belgium	6.70	0	no
K28	Gastrojejunal ulcer	K00.K93	Czech Republic	11.96	0	no
K28	Gastrojejunal ulcer	K00.K93	Denmark	11.07	0	no
K28	Gastrojejunal ulcer	K00.K93	Germany	66.17	2	yes
K28	Gastrojejunal ulcer	K00.K93	Netherlands	28.29	2	yes
K28	Gastrojejunal ulcer	K00.K93	Poland	125.79	3	yes
K28	Gastrojejunal ulcer	K00.K93	Romania	9.09	1	yes
K28	Gastrojejunal ulcer	K00.K93	United Kingdom	18.45	1	yes
K29	Gastritis and duodenitis	K00.K93	Austria	21.96	0	no
K29	Gastritis and duodenitis	K00.K93	Belgium	14.50	0	no
K29	Gastritis and duodenitis	K00.K93	Croatia	22.19	1	yes
K29	Gastritis and duodenitis	K00.K93	Czech Republic	39.93	2	yes
K29	Gastritis and duodenitis	K00.K93	Denmark	23.71	0	no
K29	Gastritis and duodenitis	K00.K93	Finland	22.13	0	no
K29	Gastritis and duodenitis	K00.K93	France	39.76	1	yes

For peer review only

K29	Gastritis and duodenitis	K00.K93	Germany	324.09	1	yes
K29	Gastritis and duodenitis	K00.K93	Hungary	51.18	1	yes
K29	Gastritis and duodenitis	K00.K93	Latvia	11.77	1	yes
K29	Gastritis and duodenitis	K00.K93	Lithuania	12.10	0	no
K29	Gastritis and duodenitis	K00.K93	Netherlands	26.04	2	yes
K29	Gastritis and duodenitis	K00.K93	Norway	11.72	2	yes
K29	Gastritis and duodenitis	K00.K93	Poland	168.50	0	no
K29	Gastritis and duodenitis	K00.K93	Romania	133.12	1	yes
K29	Gastritis and duodenitis	K00.K93	Slovenia	10.64	0	no
K29	Gastritis and duodenitis	K00.K93	Spain	50.74	0	no
K29	Gastritis and duodenitis	K00.K93	Sweden	17.02	1	yes
K29	Gastritis and duodenitis	K00.K93	Switzerland	18.05	0	no
K29	Gastritis and duodenitis	K00.K93	United Kingdom	243.75	0	no
K30	Dyspepsia	K00.K93	Czech Republic	7.39	1	yes
K31	Other diseases of stomach and duodenum	K00.K93	Austria	14.35	0	no
K31	Other diseases of stomach and duodenum	K00.K93	Belgium	41.10	0	no
K31	Other diseases of stomach and duodenum	K00.K93	Czech Republic	12.91	0	no
K31	Other diseases of stomach and duodenum	K00.K93	Denmark	9.64	1	yes
K31	Other diseases of stomach and duodenum	K00.K93	France	132.49	1	yes
K31	Other diseases of stomach and duodenum	K00.K93	Germany	268.65	2	yes
K31	Other diseases of stomach and duodenum	K00.K93	Hungary	14.62	1	yes
K31	Other diseases of stomach and duodenum	K00.K93	Netherlands	53.15	2	yes
K31	Other diseases of stomach and duodenum	K00.K93	Norway	5.88	0	no
K31	Other diseases of stomach and duodenum	K00.K93	Poland	35.00	1	yes
K31	Other diseases of stomach and duodenum	K00.K93	Romania	15.40	0	no
K31	Other diseases of stomach and duodenum	K00.K93	Spain	107.96	1	yes
K31	Other diseases of stomach and duodenum	K00.K93	Sweden	22.43	0	no
K31	Other diseases of stomach and duodenum	K00.K93	Switzerland	17.09	2	yes
K31	Other diseases of stomach and duodenum	K00.K93	United Kingdom	222.23	3	yes
K35	Acute appendicitis	K00.K93	Austria	12.16	1	yes
K35	Acute appendicitis	K00.K93	Belgium	12.99	0	no
K35	Acute appendicitis	K00.K93	Croatia	12.79	0	no
K35	Acute appendicitis	K00.K93	Czech Republic	22.88	1	yes
K35	Acute appendicitis	K00.K93	Denmark	23.57	3	yes
K35	Acute appendicitis	K00.K93	Finland	10.80	1	yes
K35	Acute appendicitis	K00.K93	France	89.79	1	yes
K35	Acute appendicitis	K00.K93	Germany	146.58	2	yes
K35	Acute appendicitis	K00.K93	Hungary	40.04	0	no
K35	Acute appendicitis	K00.K93	Lithuania	10.03	0	no
K35	Acute appendicitis	K00.K93	Netherlands	27.42	1	yes
K35	Acute appendicitis	K00.K93	Norway	7.27	0	no
K35	Acute appendicitis	K00.K93	Poland	75.83	1	yes
K35	Acute appendicitis	K00.K93	Romania	32.74	1	yes
K35	Acute appendicitis	K00.K93	Spain	62.01	1	yes
K35	Acute appendicitis	K00.K93	Sweden	12.50	1	yes
K35	Acute appendicitis	K00.K93	Switzerland	14.76	2	yes
K35	Acute appendicitis	K00.K93	United Kingdom	134.78	2	yes
K37	Unspecified appendicitis	K00.K93	France	13.63	1	yes
K37	Unspecified appendicitis	K00.K93	Germany	9.20	1	yes
K37	Unspecified appendicitis	K00.K93	Netherlands	5.73	0	no

For peer review only

K37	Unspecified appendicitis	K00.K93	Spain	11.29	0	no
K37	Unspecified appendicitis	K00.K93	United Kingdom	18.20	0	no
K38	Other diseases of appendix	K00.K93	Spain	7.85	0	no
K38	Other diseases of appendix	K00.K93	United Kingdom	9.27	1	yes
K40	Inguinal hernia	K00.K93	Austria	16.95	1	yes
K40	Inguinal hernia	K00.K93	Belgium	30.92	1	yes
K40	Inguinal hernia	K00.K93	Croatia	32.89	0	no
K40	Inguinal hernia	K00.K93	Czech Republic	28.68	0	no
K40	Inguinal hernia	K00.K93	Denmark	22.43	1	yes
K40	Inguinal hernia	K00.K93	Finland	20.72	1	yes
K40	Inguinal hernia	K00.K93	France	166.96	1	yes
K40	Inguinal hernia	K00.K93	Germany	160.62	0	no
K40	Inguinal hernia	K00.K93	Hungary	60.14	0	no
K40	Inguinal hernia	K00.K93	Latvia	6.36	2	yes
K40	Inguinal hernia	K00.K93	Lithuania	9.90	0	no
K40	Inguinal hernia	K00.K93	Netherlands	56.64	0	no
K40	Inguinal hernia	K00.K93	Norway	9.63	0	no
K40	Inguinal hernia	K00.K93	Poland	152.40	1	yes
K40	Inguinal hernia	K00.K93	Romania	58.88	1	yes
K40	Inguinal hernia	K00.K93	Slovenia	11.05	0	no
K40	Inguinal hernia	K00.K93	Spain	152.19	2	yes
K40	Inguinal hernia	K00.K93	Sweden	27.23	1	yes
K40	Inguinal hernia	K00.K93	Switzerland	20.21	0	no
K40	Inguinal hernia	K00.K93	United Kingdom	239.93	3	yes
K41	Femoral hernia	K00.K93	Croatia	9.28	1	yes
K41	Femoral hernia	K00.K93	Czech Republic	7.10	0	no
K41	Femoral hernia	K00.K93	Denmark	9.82	0	no
K41	Femoral hernia	K00.K93	Finland	7.35	1	yes
K41	Femoral hernia	K00.K93	France	35.45	1	yes
K41	Femoral hernia	K00.K93	Germany	42.50	1	yes
K41	Femoral hernia	K00.K93	Hungary	15.38	1	yes
K41	Femoral hernia	K00.K93	Lithuania	10.87	1	yes
K41	Femoral hernia	K00.K93	Netherlands	10.50	0	no
K41	Femoral hernia	K00.K93	Poland	129.40	1	yes
K41	Femoral hernia	K00.K93	Romania	10.56	0	no
K41	Femoral hernia	K00.K93	Spain	76.86	2	yes
K41	Femoral hernia	K00.K93	Sweden	5.18	0	no
K41	Femoral hernia	K00.K93	Switzerland	5.49	0	no
K41	Femoral hernia	K00.K93	United Kingdom	158.67	1	yes
K42	Umbilical hernia	K00.K93	Austria	6.47	0	no
K42	Umbilical hernia	K00.K93	Belgium	8.44	0	no
K42	Umbilical hernia	K00.K93	Croatia	7.70	0	no
K42	Umbilical hernia	K00.K93	Czech Republic	14.15	0	no
K42	Umbilical hernia	K00.K93	Finland	9.55	0	no
K42	Umbilical hernia	K00.K93	France	68.22	0	no
K42	Umbilical hernia	K00.K93	Germany	57.11	2	yes
K42	Umbilical hernia	K00.K93	Hungary	28.50	0	no
K42	Umbilical hernia	K00.K93	Lithuania	6.59	0	no
K42	Umbilical hernia	K00.K93	Netherlands	16.00	0	no
K42	Umbilical hernia	K00.K93	Poland	50.18	1	yes

For peer review only

K42	Umbilical hernia	K00.K93	Romania	16.76	0	no
K42	Umbilical hernia	K00.K93	Slovenia	5.30	0	no
K42	Umbilical hernia	K00.K93	Spain	56.96	0	no
K42	Umbilical hernia	K00.K93	Sweden	7.47	0	no
K42	Umbilical hernia	K00.K93	Switzerland	7.58	1	yes
K42	Umbilical hernia	K00.K93	United Kingdom	112.35	2	yes
K43	Ventral hernia	K00.K93	Austria	12.24	0	no
K43	Ventral hernia	K00.K93	Belgium	12.50	0	no
K43	Ventral hernia	K00.K93	Croatia	17.92	0	no
K43	Ventral hernia	K00.K93	Czech Republic	34.42	1	yes
K43	Ventral hernia	K00.K93	Denmark	13.01	0	no
K43	Ventral hernia	K00.K93	Finland	12.73	0	no
K43	Ventral hernia	K00.K93	France	149.82	2	yes
K43	Ventral hernia	K00.K93	Germany	132.18	2	yes
K43	Ventral hernia	K00.K93	Hungary	86.31	2	yes
K43	Ventral hernia	K00.K93	Latvia	7.28	0	no
K43	Ventral hernia	K00.K93	Lithuania	10.12	1	yes
K43	Ventral hernia	K00.K93	Netherlands	29.78	0	no
K43	Ventral hernia	K00.K93	Norway	6.01	1	yes
K43	Ventral hernia	K00.K93	Poland	129.64	0	no
K43	Ventral hernia	K00.K93	Romania	21.40	0	no
K43	Ventral hernia	K00.K93	Spain	81.47	0	no
K43	Ventral hernia	K00.K93	Sweden	15.66	0	no
K43	Ventral hernia	K00.K93	Switzerland	18.17	0	no
K43	Ventral hernia	K00.K93	United Kingdom	147.09	1	yes
K44	Diaphragmatic hernia	K00.K93	Austria	12.55	0	no
K44	Diaphragmatic hernia	K00.K93	Belgium	22.95	2	yes
K44	Diaphragmatic hernia	K00.K93	Czech Republic	9.34	3	yes
K44	Diaphragmatic hernia	K00.K93	Denmark	12.97	0	no
K44	Diaphragmatic hernia	K00.K93	Finland	9.81	0	no
K44	Diaphragmatic hernia	K00.K93	France	190.70	1	yes
K44	Diaphragmatic hernia	K00.K93	Germany	111.10	2	yes
K44	Diaphragmatic hernia	K00.K93	Hungary	15.47	0	no
K44	Diaphragmatic hernia	K00.K93	Netherlands	40.85	0	no
K44	Diaphragmatic hernia	K00.K93	Norway	12.66	0	no
K44	Diaphragmatic hernia	K00.K93	Poland	12.70	0	no
K44	Diaphragmatic hernia	K00.K93	Romania	8.64	1	yes
K44	Diaphragmatic hernia	K00.K93	Spain	99.81	0	no
K44	Diaphragmatic hernia	K00.K93	Sweden	19.12	0	no
K44	Diaphragmatic hernia	K00.K93	Switzerland	23.39	0	no
K44	Diaphragmatic hernia	K00.K93	United Kingdom	225.90	3	yes
K45	Other abdominal hernia	K00.K93	Czech Republic	8.07	0	no
K45	Other abdominal hernia	K00.K93	Denmark	6.63	1	yes
K45	Other abdominal hernia	K00.K93	Germany	15.68	1	yes
K45	Other abdominal hernia	K00.K93	Netherlands	6.01	0	no
K45	Other abdominal hernia	K00.K93	Poland	41.39	1	yes
K45	Other abdominal hernia	K00.K93	Romania	9.52	0	no
K45	Other abdominal hernia	K00.K93	Spain	8.56	2	yes
K45	Other abdominal hernia	K00.K93	United Kingdom	21.96	0	no
K46	Unspecified hernia of abdominal cavity	K00.K93	Austria	20.23	1	yes

For peer review only

K46	Unspecified hernia of abdominal cavity	K00.K93	Belgium	18.91	1	yes
K46	Unspecified hernia of abdominal cavity	K00.K93	Denmark	9.54	0	no
K46	Unspecified hernia of abdominal cavity	K00.K93	France	220.67	2	yes
K46	Unspecified hernia of abdominal cavity	K00.K93	Germany	67.70	1	yes
K46	Unspecified hernia of abdominal cavity	K00.K93	Hungary	20.19	2	yes
K46	Unspecified hernia of abdominal cavity	K00.K93	Netherlands	21.25	0	no
K46	Unspecified hernia of abdominal cavity	K00.K93	Norway	8.69	0	no
K46	Unspecified hernia of abdominal cavity	K00.K93	Poland	24.80	1	yes
K46	Unspecified hernia of abdominal cavity	K00.K93	Romania	7.59	0	no
K46	Unspecified hernia of abdominal cavity	K00.K93	Spain	143.05	1	yes
K46	Unspecified hernia of abdominal cavity	K00.K93	Sweden	8.25	1	yes
K46	Unspecified hernia of abdominal cavity	K00.K93	Switzerland	9.79	0	no
K46	Unspecified hernia of abdominal cavity	K00.K93	United Kingdom	203.26	3	yes
K50	Crohn's disease [regional enteritis]	K00.K93	Austria	19.92	0	no
K50	Crohn's disease [regional enteritis]	K00.K93	Belgium	28.25	1	yes
K50	Crohn's disease [regional enteritis]	K00.K93	Croatia	12.65	2	yes
K50	Crohn's disease [regional enteritis]	K00.K93	Czech Republic	28.82	0	no
K50	Crohn's disease [regional enteritis]	K00.K93	Denmark	22.54	1	yes
K50	Crohn's disease [regional enteritis]	K00.K93	Finland	6.72	0	no
K50	Crohn's disease [regional enteritis]	K00.K93	France	98.48	0	no
K50	Crohn's disease [regional enteritis]	K00.K93	Germany	217.01	2	yes
K50	Crohn's disease [regional enteritis]	K00.K93	Hungary	28.03	0	no
K50	Crohn's disease [regional enteritis]	K00.K93	Netherlands	46.39	1	yes
K50	Crohn's disease [regional enteritis]	K00.K93	Norway	11.26	1	yes
K50	Crohn's disease [regional enteritis]	K00.K93	Poland	54.06	0	no
K50	Crohn's disease [regional enteritis]	K00.K93	Romania	23.12	1	yes
K50	Crohn's disease [regional enteritis]	K00.K93	Spain	76.16	1	yes
K50	Crohn's disease [regional enteritis]	K00.K93	Sweden	17.67	0	no
K50	Crohn's disease [regional enteritis]	K00.K93	Switzerland	19.61	0	no
K50	Crohn's disease [regional enteritis]	K00.K93	United Kingdom	251.99	2	yes
K51	Ulcerative colitis	K00.K93	Austria	18.51	2	yes
K51	Ulcerative colitis	K00.K93	Belgium	13.21	1	yes
K51	Ulcerative colitis	K00.K93	Croatia	13.53	2	yes
K51	Ulcerative colitis	K00.K93	Czech Republic	38.38	0	no
K51	Ulcerative colitis	K00.K93	Denmark	28.29	2	yes
K51	Ulcerative colitis	K00.K93	Estonia	6.31	0	no
K51	Ulcerative colitis	K00.K93	Finland	12.43	1	yes
K51	Ulcerative colitis	K00.K93	France	73.16	1	yes
K51	Ulcerative colitis	K00.K93	Germany	186.08	2	yes
K51	Ulcerative colitis	K00.K93	Hungary	41.02	0	no
K51	Ulcerative colitis	K00.K93	Latvia	9.34	0	no
K51	Ulcerative colitis	K00.K93	Lithuania	9.69	2	yes
K51	Ulcerative colitis	K00.K93	Netherlands	33.05	0	no
K51	Ulcerative colitis	K00.K93	Norway	13.95	0	no
K51	Ulcerative colitis	K00.K93	Poland	93.68	0	no
K51	Ulcerative colitis	K00.K93	Romania	47.29	3	yes
K51	Ulcerative colitis	K00.K93	Slovenia	5.80	1	yes
K51	Ulcerative colitis	K00.K93	Spain	71.20	2	yes
K51	Ulcerative colitis	K00.K93	Sweden	16.51	0	no
K51	Ulcerative colitis	K00.K93	Switzerland	14.52	2	yes

For peer review only

K51	Ulcerative colitis	K00.K93	United Kingdom	201.18	2	yes
K52	Other noninfective gastroenteritis and colitis	K00.K93	Austria	78.58	2	yes
K52	Other noninfective gastroenteritis and colitis	K00.K93	Belgium	215.58	2	yes
K52	Other noninfective gastroenteritis and colitis	K00.K93	Czech Republic	52.33	2	yes
K52	Other noninfective gastroenteritis and colitis	K00.K93	Denmark	17.06	0	no
K52	Other noninfective gastroenteritis and colitis	K00.K93	Finland	13.54	1	yes
K52	Other noninfective gastroenteritis and colitis	K00.K93	France	626.59	3	yes
K52	Other noninfective gastroenteritis and colitis	K00.K93	Germany	1359.34	2	yes
K52	Other noninfective gastroenteritis and colitis	K00.K93	Hungary	59.00	0	no
K52	Other noninfective gastroenteritis and colitis	K00.K93	Netherlands	389.86	2	yes
K52	Other noninfective gastroenteritis and colitis	K00.K93	Norway	8.17	0	no
K52	Other noninfective gastroenteritis and colitis	K00.K93	Poland	153.57	1	yes
K52	Other noninfective gastroenteritis and colitis	K00.K93	Slovenia	10.32	0	no
K52	Other noninfective gastroenteritis and colitis	K00.K93	Spain	134.85	3	yes
K52	Other noninfective gastroenteritis and colitis	K00.K93	Sweden	96.89	1	yes
K52	Other noninfective gastroenteritis and colitis	K00.K93	Switzerland	81.20	1	yes
K52	Other noninfective gastroenteritis and colitis	K00.K93	United Kingdom	666.11	2	yes
K55	Vascular disorders of intestine	K00.K93	Austria	327.13	1	yes
K55	Vascular disorders of intestine	K00.K93	Belgium	686.72	1	yes
K55	Vascular disorders of intestine	K00.K93	Croatia	261.51	1	yes
K55	Vascular disorders of intestine	K00.K93	Czech Republic	331.42	1	yes
K55	Vascular disorders of intestine	K00.K93	Denmark	181.23	1	yes
K55	Vascular disorders of intestine	K00.K93	Estonia	140.13	1	yes
K55	Vascular disorders of intestine	K00.K93	Finland	223.86	1	yes
K55	Vascular disorders of intestine	K00.K93	France	2591.91	2	yes
K55	Vascular disorders of intestine	K00.K93	Germany	4651.68	3	yes
K55	Vascular disorders of intestine	K00.K93	Hungary	656.32	2	yes
K55	Vascular disorders of intestine	K00.K93	Latvia	235.96	1	yes
K55	Vascular disorders of intestine	K00.K93	Lithuania	304.18	2	yes
K55	Vascular disorders of intestine	K00.K93	Netherlands	572.79	2	yes
K55	Vascular disorders of intestine	K00.K93	Norway	114.27	2	yes
K55	Vascular disorders of intestine	K00.K93	Poland	1897.72	3	yes
K55	Vascular disorders of intestine	K00.K93	Romania	1057.79	3	yes
K55	Vascular disorders of intestine	K00.K93	Slovenia	129.92	1	yes
K55	Vascular disorders of intestine	K00.K93	Spain	3199.74	2	yes
K55	Vascular disorders of intestine	K00.K93	Sweden	245.06	2	yes
K55	Vascular disorders of intestine	K00.K93	Switzerland	206.98	0	no
K55	Vascular disorders of intestine	K00.K93	United Kingdom	3144.00	2	yes
K56	Paralytic ileus and intestinal obstruction without hernia	K00.K93	Austria	228.74	1	yes
K56	Paralytic ileus and intestinal obstruction without hernia	K00.K93	Belgium	457.77	1	yes
K56	Paralytic ileus and intestinal obstruction without hernia	K00.K93	Croatia	158.32	0	no
K56	Paralytic ileus and intestinal obstruction without hernia	K00.K93	Czech Republic	332.59	3	yes
K56	Paralytic ileus and intestinal obstruction without hernia	K00.K93	Denmark	256.27	2	yes
K56	Paralytic ileus and intestinal obstruction without hernia	K00.K93	Estonia	20.58	0	no
K56	Paralytic ileus and intestinal obstruction without hernia	K00.K93	Finland	201.86	3	yes

For peer review only

K56	Paralytic ileus and intestinal obstruction without hernia	K00.K93	France	3144.07	1	yes
K56	Paralytic ileus and intestinal obstruction without hernia	K00.K93	Germany	3975.83	3	yes
K56	Paralytic ileus and intestinal obstruction without hernia	K00.K93	Hungary	277.67	1	yes
K56	Paralytic ileus and intestinal obstruction without hernia	K00.K93	Latvia	27.58	0	no
K56	Paralytic ileus and intestinal obstruction without hernia	K00.K93	Lithuania	55.28	1	yes
K56	Paralytic ileus and intestinal obstruction without hernia	K00.K93	Netherlands	828.02	1	yes
K56	Paralytic ileus and intestinal obstruction without hernia	K00.K93	Norway	157.34	1	yes
K56	Paralytic ileus and intestinal obstruction without hernia	K00.K93	Poland	1211.24	1	yes
K56	Paralytic ileus and intestinal obstruction without hernia	K00.K93	Romania	741.37	0	no
K56	Paralytic ileus and intestinal obstruction without hernia	K00.K93	Slovenia	83.58	0	no
K56	Paralytic ileus and intestinal obstruction without hernia	K00.K93	Spain	1895.00	3	yes
K56	Paralytic ileus and intestinal obstruction without hernia	K00.K93	Sweden	253.59	0	no
K56	Paralytic ileus and intestinal obstruction without hernia	K00.K93	Switzerland	272.57	1	yes
K56	Paralytic ileus and intestinal obstruction without hernia	K00.K93	United Kingdom	2317.83	3	yes
K57	Diverticular disease of intestine	K00.K93	Austria	163.25	1	yes
K57	Diverticular disease of intestine	K00.K93	Belgium	148.14	1	yes
K57	Diverticular disease of intestine	K00.K93	Croatia	39.76	1	yes
K57	Diverticular disease of intestine	K00.K93	Czech Republic	172.97	0	no
K57	Diverticular disease of intestine	K00.K93	Denmark	125.30	1	yes
K57	Diverticular disease of intestine	K00.K93	Estonia	11.31	0	no
K57	Diverticular disease of intestine	K00.K93	Finland	132.57	1	yes
K57	Diverticular disease of intestine	K00.K93	France	435.62	1	yes
K57	Diverticular disease of intestine	K00.K93	Germany	1261.25	2	yes
K57	Diverticular disease of intestine	K00.K93	Hungary	171.16	1	yes
K57	Diverticular disease of intestine	K00.K93	Latvia	12.15	1	yes
K57	Diverticular disease of intestine	K00.K93	Lithuania	27.92	1	yes
K57	Diverticular disease of intestine	K00.K93	Netherlands	354.35	2	yes
K57	Diverticular disease of intestine	K00.K93	Norway	99.05	0	no
K57	Diverticular disease of intestine	K00.K93	Poland	319.42	1	yes
K57	Diverticular disease of intestine	K00.K93	Romania	19.78	1	yes
K57	Diverticular disease of intestine	K00.K93	Slovenia	24.42	0	no
K57	Diverticular disease of intestine	K00.K93	Spain	391.13	0	no
K57	Diverticular disease of intestine	K00.K93	Sweden	114.30	1	yes
K57	Diverticular disease of intestine	K00.K93	Switzerland	256.80	1	yes
K57	Diverticular disease of intestine	K00.K93	United Kingdom	2268.74	3	yes
K58	Irritable bowel syndrome	K00.K93	Germany	63.56	3	yes
K59	Other functional intestinal disorders	K00.K93	Belgium	15.82	1	yes
K59	Other functional intestinal disorders	K00.K93	Denmark	30.26	0	no
K59	Other functional intestinal disorders	K00.K93	Finland	7.35	0	no
K59	Other functional intestinal disorders	K00.K93	France	117.35	1	yes
K59	Other functional intestinal disorders	K00.K93	Germany	153.14	3	yes

For peer review only

K59	Other functional intestinal disorders	K00.K93	Hungary	16.08	1	yes
K59	Other functional intestinal disorders	K00.K93	Netherlands	38.44	1	yes
K59	Other functional intestinal disorders	K00.K93	Norway	10.37	0	no
K59	Other functional intestinal disorders	K00.K93	Poland	7.98	0	no
K59	Other functional intestinal disorders	K00.K93	Spain	48.31	2	yes
K59	Other functional intestinal disorders	K00.K93	Sweden	16.67	3	yes
K59	Other functional intestinal disorders	K00.K93	Switzerland	8.27	3	yes
K59	Other functional intestinal disorders	K00.K93	United Kingdom	143.87	1	yes
K60	Fissure and fistula of anal and rectal regions	K00.K93	France	9.18	0	no
K60	Fissure and fistula of anal and rectal regions	K00.K93	Germany	5.80	0	no
K61	Abscess of anal and rectal regions	K00.K93	Belgium	5.44	0	no
K61	Abscess of anal and rectal regions	K00.K93	France	18.61	0	no
K61	Abscess of anal and rectal regions	K00.K93	Germany	21.57	0	no
K61	Abscess of anal and rectal regions	K00.K93	Hungary	11.92	0	no
K61	Abscess of anal and rectal regions	K00.K93	Netherlands	8.37	0	no
K61	Abscess of anal and rectal regions	K00.K93	Poland	10.15	0	no
K61	Abscess of anal and rectal regions	K00.K93	Romania	8.42	0	no
K61	Abscess of anal and rectal regions	K00.K93	Spain	16.65	0	no
K61	Abscess of anal and rectal regions	K00.K93	United Kingdom	27.08	0	no
K62	Other diseases of anus and rectum	K00.K93	Belgium	25.55	0	no
K62	Other diseases of anus and rectum	K00.K93	Czech Republic	9.39	0	no
K62	Other diseases of anus and rectum	K00.K93	Denmark	24.88	2	yes
K62	Other diseases of anus and rectum	K00.K93	France	200.25	1	yes
K62	Other diseases of anus and rectum	K00.K93	Germany	70.13	0	no
K62	Other diseases of anus and rectum	K00.K93	Hungary	6.54	0	no
K62	Other diseases of anus and rectum	K00.K93	Netherlands	21.53	2	yes
K62	Other diseases of anus and rectum	K00.K93	Norway	13.87	0	no
K62	Other diseases of anus and rectum	K00.K93	Poland	16.54	2	yes
K62	Other diseases of anus and rectum	K00.K93	Spain	174.56	2	yes
K62	Other diseases of anus and rectum	K00.K93	Sweden	13.53	0	no
K62	Other diseases of anus and rectum	K00.K93	Switzerland	10.03	0	no
K62	Other diseases of anus and rectum	K00.K93	United Kingdom	96.41	2	yes
K63	Other diseases of intestine	K00.K93	Austria	81.95	1	yes
K63	Other diseases of intestine	K00.K93	Belgium	177.09	2	yes
K63	Other diseases of intestine	K00.K93	Croatia	31.06	3	yes
K63	Other diseases of intestine	K00.K93	Czech Republic	61.10	0	no
K63	Other diseases of intestine	K00.K93	Denmark	67.44	1	yes
K63	Other diseases of intestine	K00.K93	Estonia	5.38	0	no
K63	Other diseases of intestine	K00.K93	Finland	42.28	1	yes
K63	Other diseases of intestine	K00.K93	France	564.99	1	yes
K63	Other diseases of intestine	K00.K93	Germany	1079.61	2	yes
K63	Other diseases of intestine	K00.K93	Hungary	75.04	3	yes
K63	Other diseases of intestine	K00.K93	Latvia	7.49	0	no
K63	Other diseases of intestine	K00.K93	Lithuania	19.12	1	yes
K63	Other diseases of intestine	K00.K93	Netherlands	345.46	3	yes
K63	Other diseases of intestine	K00.K93	Norway	44.28	0	no
K63	Other diseases of intestine	K00.K93	Poland	256.81	3	yes
K63	Other diseases of intestine	K00.K93	Romania	20.22	0	no
K63	Other diseases of intestine	K00.K93	Slovenia	28.92	1	yes
K63	Other diseases of intestine	K00.K93	Spain	604.23	2	yes

For peer review only

K63	Other diseases of intestine	K00.K93	Sweden	78.12	0	no
K63	Other diseases of intestine	K00.K93	Switzerland	93.40	0	no
K63	Other diseases of intestine	K00.K93	United Kingdom	1269.93	3	yes
K65	Peritonitis	K00.K93	Austria	14.30	0	no
K65	Peritonitis	K00.K93	Belgium	91.63	2	yes
K65	Peritonitis	K00.K93	Croatia	60.06	1	yes
K65	Peritonitis	K00.K93	Czech Republic	225.66	3	yes
K65	Peritonitis	K00.K93	Denmark	45.15	1	yes
K65	Peritonitis	K00.K93	Finland	16.95	0	no
K65	Peritonitis	K00.K93	France	600.82	2	yes
K65	Peritonitis	K00.K93	Germany	658.90	2	yes
K65	Peritonitis	K00.K93	Hungary	63.54	1	yes
K65	Peritonitis	K00.K93	Latvia	6.69	0	no
K65	Peritonitis	K00.K93	Lithuania	19.47	3	yes
K65	Peritonitis	K00.K93	Netherlands	106.86	1	yes
K65	Peritonitis	K00.K93	Norway	22.43	0	no
K65	Peritonitis	K00.K93	Poland	901.84	3	yes
K65	Peritonitis	K00.K93	Romania	572.24	0	no
K65	Peritonitis	K00.K93	Slovenia	11.09	2	yes
K65	Peritonitis	K00.K93	Spain	635.15	3	yes
K65	Peritonitis	K00.K93	Sweden	48.58	1	yes
K65	Peritonitis	K00.K93	Switzerland	36.63	1	yes
K65	Peritonitis	K00.K93	United Kingdom	579.41	3	yes
K66	Other disorders of peritoneum	K00.K93	Austria	7.40	1	yes
K66	Other disorders of peritoneum	K00.K93	Belgium	25.70	0	no
K66	Other disorders of peritoneum	K00.K93	Czech Republic	16.15	0	no
K66	Other disorders of peritoneum	K00.K93	France	91.46	1	yes
K66	Other disorders of peritoneum	K00.K93	Germany	63.81	1	yes
K66	Other disorders of peritoneum	K00.K93	Hungary	13.95	0	no
K66	Other disorders of peritoneum	K00.K93	Netherlands	5.37	0	no
K66	Other disorders of peritoneum	K00.K93	Poland	8.62	1	yes
K66	Other disorders of peritoneum	K00.K93	Romania	7.78	1	yes
K66	Other disorders of peritoneum	K00.K93	Spain	89.65	1	yes
K66	Other disorders of peritoneum	K00.K93	Switzerland	9.24	0	no
K66	Other disorders of peritoneum	K00.K93	United Kingdom	157.44	1	yes
K70	Alcoholic liver disease	K00.K93	Austria	706.86	3	yes
K70	Alcoholic liver disease	K00.K93	Belgium	733.84	1	yes
K70	Alcoholic liver disease	K00.K93	Croatia	477.28	3	yes
K70	Alcoholic liver disease	K00.K93	Czech Republic	1176.00	2	yes
K70	Alcoholic liver disease	K00.K93	Denmark	749.23	2	yes
K70	Alcoholic liver disease	K00.K93	Estonia	193.43	0	no
K70	Alcoholic liver disease	K00.K93	Finland	947.65	2	yes
K70	Alcoholic liver disease	K00.K93	France	5686.36	2	yes
K70	Alcoholic liver disease	K00.K93	Germany	8985.32	2	yes
K70	Alcoholic liver disease	K00.K93	Hungary	3839.75	1	yes
K70	Alcoholic liver disease	K00.K93	Latvia	110.65	1	yes
K70	Alcoholic liver disease	K00.K93	Lithuania	517.86	2	yes
K70	Alcoholic liver disease	K00.K93	Netherlands	479.13	1	yes
K70	Alcoholic liver disease	K00.K93	Norway	167.78	1	yes
K70	Alcoholic liver disease	K00.K93	Poland	2599.20	1	yes

For peer review only

K70	Alcoholic liver disease	K00.K93	Romania	1037.17	2	yes
K70	Alcoholic liver disease	K00.K93	Slovenia	495.43	3	yes
K70	Alcoholic liver disease	K00.K93	Spain	1402.28	2	yes
K70	Alcoholic liver disease	K00.K93	Sweden	375.02	0	no
K70	Alcoholic liver disease	K00.K93	Switzerland	581.51	1	yes
K70	Alcoholic liver disease	K00.K93	United Kingdom	5661.12	3	yes
K71	Toxic liver disease	K00.K93	Austria	7.48	3	yes
K71	Toxic liver disease	K00.K93	Belgium	16.16	0	no
K71	Toxic liver disease	K00.K93	Croatia	8.08	1	yes
K71	Toxic liver disease	K00.K93	Czech Republic	39.19	0	no
K71	Toxic liver disease	K00.K93	Denmark	7.60	1	yes
K71	Toxic liver disease	K00.K93	France	72.52	3	yes
K71	Toxic liver disease	K00.K93	Germany	125.97	1	yes
K71	Toxic liver disease	K00.K93	Hungary	92.10	2	yes
K71	Toxic liver disease	K00.K93	Latvia	35.78	0	no
K71	Toxic liver disease	K00.K93	Lithuania	80.95	2	yes
K71	Toxic liver disease	K00.K93	Poland	155.45	2	yes
K71	Toxic liver disease	K00.K93	Romania	289.66	2	yes
K71	Toxic liver disease	K00.K93	Spain	24.12	0	no
K71	Toxic liver disease	K00.K93	Switzerland	9.43	0	no
K71	Toxic liver disease	K00.K93	United Kingdom	6.98	2	yes
K72	Hepatic failure, not elsewhere classified	K00.K93	Austria	9.48	2	yes
K72	Hepatic failure, not elsewhere classified	K00.K93	Belgium	104.50	0	no
K72	Hepatic failure, not elsewhere classified	K00.K93	Croatia	14.68	0	no
K72	Hepatic failure, not elsewhere classified	K00.K93	Czech Republic	120.61	3	yes
K72	Hepatic failure, not elsewhere classified	K00.K93	Denmark	52.41	0	no
K72	Hepatic failure, not elsewhere classified	K00.K93	Finland	12.12	0	no
K72	Hepatic failure, not elsewhere classified	K00.K93	France	547.37	1	yes
K72	Hepatic failure, not elsewhere classified	K00.K93	Germany	389.23	0	no
K72	Hepatic failure, not elsewhere classified	K00.K93	Hungary	47.04	1	yes
K72	Hepatic failure, not elsewhere classified	K00.K93	Lithuania	29.75	2	yes
K72	Hepatic failure, not elsewhere classified	K00.K93	Netherlands	112.94	2	yes
K72	Hepatic failure, not elsewhere classified	K00.K93	Norway	38.18	0	no
K72	Hepatic failure, not elsewhere classified	K00.K93	Poland	398.11	1	yes
K72	Hepatic failure, not elsewhere classified	K00.K93	Romania	130.48	2	yes
K72	Hepatic failure, not elsewhere classified	K00.K93	Spain	568.06	1	yes
K72	Hepatic failure, not elsewhere classified	K00.K93	Sweden	73.19	0	no
K72	Hepatic failure, not elsewhere classified	K00.K93	Switzerland	22.70	1	yes
K72	Hepatic failure, not elsewhere classified	K00.K93	United Kingdom	304.84	2	yes
K73	Chronic hepatitis, not elsewhere classified	K00.K93	Czech Republic	31.28	1	yes
K73	Chronic hepatitis, not elsewhere classified	K00.K93	Denmark	6.78	0	no
K73	Chronic hepatitis, not elsewhere classified	K00.K93	France	25.90	1	yes
K73	Chronic hepatitis, not elsewhere classified	K00.K93	Germany	41.64	2	yes
K73	Chronic hepatitis, not elsewhere classified	K00.K93	Hungary	49.41	1	yes
K73	Chronic hepatitis, not elsewhere classified	K00.K93	Lithuania	9.69	0	no
K73	Chronic hepatitis, not elsewhere classified	K00.K93	Netherlands	13.54	3	yes
K73	Chronic hepatitis, not elsewhere classified	K00.K93	Poland	24.34	1	yes
K73	Chronic hepatitis, not elsewhere classified	K00.K93	Romania	566.78	1	yes
K73	Chronic hepatitis, not elsewhere classified	K00.K93	Spain	29.33	1	yes
K73	Chronic hepatitis, not elsewhere classified	K00.K93	United Kingdom	62.08	1	yes

For peer review only

K74	Fibrosis and cirrhosis of liver	K00.K93	Austria	888.21	3	yes
K74	Fibrosis and cirrhosis of liver	K00.K93	Belgium	529.39	2	yes
K74	Fibrosis and cirrhosis of liver	K00.K93	Croatia	771.53	1	yes
K74	Fibrosis and cirrhosis of liver	K00.K93	Czech Republic	787.57	1	yes
K74	Fibrosis and cirrhosis of liver	K00.K93	Denmark	81.66	1	yes
K74	Fibrosis and cirrhosis of liver	K00.K93	Estonia	108.09	1	yes
K74	Fibrosis and cirrhosis of liver	K00.K93	Finland	116.31	0	no
K74	Fibrosis and cirrhosis of liver	K00.K93	France	2803.90	2	yes
K74	Fibrosis and cirrhosis of liver	K00.K93	Germany	5783.95	2	yes
K74	Fibrosis and cirrhosis of liver	K00.K93	Hungary	983.45	3	yes
K74	Fibrosis and cirrhosis of liver	K00.K93	Latvia	302.37	1	yes
K74	Fibrosis and cirrhosis of liver	K00.K93	Lithuania	470.57	2	yes
K74	Fibrosis and cirrhosis of liver	K00.K93	Netherlands	400.73	0	no
K74	Fibrosis and cirrhosis of liver	K00.K93	Norway	61.12	0	no
K74	Fibrosis and cirrhosis of liver	K00.K93	Poland	4112.33	2	yes
K74	Fibrosis and cirrhosis of liver	K00.K93	Romania	10266.86	1	yes
K74	Fibrosis and cirrhosis of liver	K00.K93	Slovenia	139.30	2	yes
K74	Fibrosis and cirrhosis of liver	K00.K93	Spain	3938.65	2	yes
K74	Fibrosis and cirrhosis of liver	K00.K93	Sweden	268.91	2	yes
K74	Fibrosis and cirrhosis of liver	K00.K93	Switzerland	130.52	0	no
K74	Fibrosis and cirrhosis of liver	K00.K93	United Kingdom	2122.36	3	yes
K75	Other inflammatory liver diseases	K00.K93	Austria	21.10	0	no
K75	Other inflammatory liver diseases	K00.K93	Belgium	32.02	0	no
K75	Other inflammatory liver diseases	K00.K93	Croatia	6.07	0	no
K75	Other inflammatory liver diseases	K00.K93	Czech Republic	29.31	0	no
K75	Other inflammatory liver diseases	K00.K93	Denmark	9.04	0	no
K75	Other inflammatory liver diseases	K00.K93	Finland	11.15	0	no
K75	Other inflammatory liver diseases	K00.K93	France	145.41	2	yes
K75	Other inflammatory liver diseases	K00.K93	Germany	199.70	1	yes
K75	Other inflammatory liver diseases	K00.K93	Hungary	31.27	0	no
K75	Other inflammatory liver diseases	K00.K93	Lithuania	6.18	1	yes
K75	Other inflammatory liver diseases	K00.K93	Netherlands	25.45	2	yes
K75	Other inflammatory liver diseases	K00.K93	Norway	11.30	2	yes
K75	Other inflammatory liver diseases	K00.K93	Poland	46.60	0	no
K75	Other inflammatory liver diseases	K00.K93	Romania	23.49	0	no
K75	Other inflammatory liver diseases	K00.K93	Spain	108.52	0	no
K75	Other inflammatory liver diseases	K00.K93	Sweden	19.82	1	yes
K75	Other inflammatory liver diseases	K00.K93	Switzerland	19.52	1	yes
K75	Other inflammatory liver diseases	K00.K93	United Kingdom	206.00	3	yes
K76	Other diseases of liver	K00.K93	Austria	100.71	2	yes
K76	Other diseases of liver	K00.K93	Belgium	55.32	1	yes
K76	Other diseases of liver	K00.K93	Croatia	22.37	1	yes
K76	Other diseases of liver	K00.K93	Czech Republic	134.42	2	yes
K76	Other diseases of liver	K00.K93	Denmark	19.48	3	yes
K76	Other diseases of liver	K00.K93	Estonia	7.57	1	yes
K76	Other diseases of liver	K00.K93	Finland	7.02	0	no
K76	Other diseases of liver	K00.K93	France	253.66	0	no
K76	Other diseases of liver	K00.K93	Germany	386.67	0	no
K76	Other diseases of liver	K00.K93	Hungary	135.72	2	yes
K76	Other diseases of liver	K00.K93	Latvia	12.98	0	no

For peer review only

K76	Other diseases of liver	K00.K93	Lithuania	21.38	1	yes
K76	Other diseases of liver	K00.K93	Netherlands	76.22	0	no
K76	Other diseases of liver	K00.K93	Norway	12.42	0	no
K76	Other diseases of liver	K00.K93	Poland	129.85	3	yes
K76	Other diseases of liver	K00.K93	Romania	176.43	3	yes
K76	Other diseases of liver	K00.K93	Slovenia	8.70	1	yes
K76	Other diseases of liver	K00.K93	Spain	918.62	1	yes
K76	Other diseases of liver	K00.K93	Sweden	29.91	1	yes
K76	Other diseases of liver	K00.K93	Switzerland	27.01	0	no
K76	Other diseases of liver	K00.K93	United Kingdom	953.28	2	yes
K80	Cholelithiasis	K00.K93	Austria	73.66	0	no
K80	Cholelithiasis	K00.K93	Belgium	76.44	2	yes
K80	Cholelithiasis	K00.K93	Croatia	58.62	1	yes
K80	Cholelithiasis	K00.K93	Czech Republic	203.27	2	yes
K80	Cholelithiasis	K00.K93	Denmark	70.36	1	yes
K80	Cholelithiasis	K00.K93	Estonia	14.04	0	no
K80	Cholelithiasis	K00.K93	Finland	118.70	1	yes
K80	Cholelithiasis	K00.K93	France	388.85	2	yes
K80	Cholelithiasis	K00.K93	Germany	728.12	1	yes
K80	Cholelithiasis	K00.K93	Hungary	308.78	3	yes
K80	Cholelithiasis	K00.K93	Latvia	31.27	0	no
K80	Cholelithiasis	K00.K93	Lithuania	73.65	2	yes
K80	Cholelithiasis	K00.K93	Netherlands	123.87	1	yes
K80	Cholelithiasis	K00.K93	Norway	43.82	0	no
K80	Cholelithiasis	K00.K93	Poland	530.47	1	yes
K80	Cholelithiasis	K00.K93	Romania	106.62	0	no
K80	Cholelithiasis	K00.K93	Slovenia	38.85	0	no
K80	Cholelithiasis	K00.K93	Spain	521.88	0	no
K80	Cholelithiasis	K00.K93	Sweden	75.95	0	no
K80	Cholelithiasis	K00.K93	Switzerland	50.83	0	no
K80	Cholelithiasis	K00.K93	United Kingdom	804.28	3	yes
K81	Cholecystitis	K00.K93	Austria	53.73	0	no
K81	Cholecystitis	K00.K93	Belgium	145.39	1	yes
K81	Cholecystitis	K00.K93	Croatia	46.29	0	no
K81	Cholecystitis	K00.K93	Czech Republic	76.89	1	yes
K81	Cholecystitis	K00.K93	Denmark	54.31	1	yes
K81	Cholecystitis	K00.K93	Estonia	6.30	0	no
K81	Cholecystitis	K00.K93	Finland	44.63	1	yes
K81	Cholecystitis	K00.K93	France	580.13	1	yes
K81	Cholecystitis	K00.K93	Germany	649.13	1	yes
K81	Cholecystitis	K00.K93	Hungary	83.99	2	yes
K81	Cholecystitis	K00.K93	Latvia	14.04	0	no
K81	Cholecystitis	K00.K93	Lithuania	14.09	0	no
K81	Cholecystitis	K00.K93	Netherlands	164.67	0	no
K81	Cholecystitis	K00.K93	Norway	54.39	2	yes
K81	Cholecystitis	K00.K93	Poland	286.87	1	yes
K81	Cholecystitis	K00.K93	Romania	125.11	1	yes
K81	Cholecystitis	K00.K93	Slovenia	31.06	0	no
K81	Cholecystitis	K00.K93	Spain	1116.36	0	no
K81	Cholecystitis	K00.K93	Sweden	74.09	0	no

For peer review only

K81	Cholecystitis	K00.K93	Switzerland	63.12	2	yes
K81	Cholecystitis	K00.K93	United Kingdom	475.04	3	yes
K82	Other diseases of gallbladder	K00.K93	Austria	11.76	0	no
K82	Other diseases of gallbladder	K00.K93	Belgium	24.49	0	no
K82	Other diseases of gallbladder	K00.K93	Croatia	12.58	2	yes
K82	Other diseases of gallbladder	K00.K93	Czech Republic	19.41	0	no
K82	Other diseases of gallbladder	K00.K93	Denmark	6.71	1	yes
K82	Other diseases of gallbladder	K00.K93	France	170.42	1	yes
K82	Other diseases of gallbladder	K00.K93	Germany	152.23	1	yes
K82	Other diseases of gallbladder	K00.K93	Hungary	15.52	1	yes
K82	Other diseases of gallbladder	K00.K93	Netherlands	40.46	0	no
K82	Other diseases of gallbladder	K00.K93	Norway	8.44	1	yes
K82	Other diseases of gallbladder	K00.K93	Poland	40.31	3	yes
K82	Other diseases of gallbladder	K00.K93	Romania	7.88	0	no
K82	Other diseases of gallbladder	K00.K93	Spain	94.83	3	yes
K82	Other diseases of gallbladder	K00.K93	Sweden	5.85	0	no
K82	Other diseases of gallbladder	K00.K93	Switzerland	5.68	1	yes
K82	Other diseases of gallbladder	K00.K93	United Kingdom	128.72	1	yes
K83	Other diseases of biliary tract	K00.K93	Austria	30.56	0	no
K83	Other diseases of biliary tract	K00.K93	Belgium	86.89	0	no
K83	Other diseases of biliary tract	K00.K93	Croatia	12.35	0	no
K83	Other diseases of biliary tract	K00.K93	Czech Republic	79.92	1	yes
K83	Other diseases of biliary tract	K00.K93	Denmark	41.09	0	no
K83	Other diseases of biliary tract	K00.K93	Finland	25.30	0	no
K83	Other diseases of biliary tract	K00.K93	France	487.28	0	no
K83	Other diseases of biliary tract	K00.K93	Germany	424.88	1	yes
K83	Other diseases of biliary tract	K00.K93	Hungary	49.63	0	no
K83	Other diseases of biliary tract	K00.K93	Lithuania	8.40	2	yes
K83	Other diseases of biliary tract	K00.K93	Netherlands	115.28	1	yes
K83	Other diseases of biliary tract	K00.K93	Norway	33.95	0	no
K83	Other diseases of biliary tract	K00.K93	Poland	131.02	3	yes
K83	Other diseases of biliary tract	K00.K93	Romania	22.57	1	yes
K83	Other diseases of biliary tract	K00.K93	Slovenia	11.02	0	no
K83	Other diseases of biliary tract	K00.K93	Spain	366.98	1	yes
K83	Other diseases of biliary tract	K00.K93	Sweden	65.45	0	no
K83	Other diseases of biliary tract	K00.K93	Switzerland	44.66	1	yes
K83	Other diseases of biliary tract	K00.K93	United Kingdom	764.39	2	yes
K85	Acute pancreatitis	K00.K93	Austria	69.67	2	yes
K85	Acute pancreatitis	K00.K93	Belgium	141.25	0	no
K85	Acute pancreatitis	K00.K93	Croatia	150.25	1	yes
K85	Acute pancreatitis	K00.K93	Czech Republic	339.96	1	yes
K85	Acute pancreatitis	K00.K93	Denmark	71.60	2	yes
K85	Acute pancreatitis	K00.K93	Estonia	39.36	1	yes
K85	Acute pancreatitis	K00.K93	Finland	97.08	3	yes
K85	Acute pancreatitis	K00.K93	France	804.69	2	yes
K85	Acute pancreatitis	K00.K93	Germany	1215.96	1	yes
K85	Acute pancreatitis	K00.K93	Hungary	339.53	1	yes
K85	Acute pancreatitis	K00.K93	Latvia	88.11	3	yes
K85	Acute pancreatitis	K00.K93	Lithuania	167.36	1	yes
K85	Acute pancreatitis	K00.K93	Netherlands	166.07	0	no

For peer review only

K85	Acute pancreatitis	K00.K93	Norway	47.42	2	yes
K85	Acute pancreatitis	K00.K93	Poland	1873.41	2	yes
K85	Acute pancreatitis	K00.K93	Romania	1023.90	0	no
K85	Acute pancreatitis	K00.K93	Slovenia	50.84	1	yes
K85	Acute pancreatitis	K00.K93	Spain	1314.02	1	yes
K85	Acute pancreatitis	K00.K93	Sweden	125.35	0	no
K85	Acute pancreatitis	K00.K93	Switzerland	72.60	0	no
K85	Acute pancreatitis	K00.K93	United Kingdom	1225.06	1	yes
K86	Other diseases of pancreas	K00.K93	Austria	60.43	0	no
K86	Other diseases of pancreas	K00.K93	Belgium	32.97	1	yes
K86	Other diseases of pancreas	K00.K93	Croatia	19.84	0	no
K86	Other diseases of pancreas	K00.K93	Czech Republic	85.60	1	yes
K86	Other diseases of pancreas	K00.K93	Denmark	80.88	2	yes
K86	Other diseases of pancreas	K00.K93	Estonia	17.37	1	yes
K86	Other diseases of pancreas	K00.K93	Finland	77.20	1	yes
K86	Other diseases of pancreas	K00.K93	France	222.26	1	yes
K86	Other diseases of pancreas	K00.K93	Germany	491.44	1	yes
K86	Other diseases of pancreas	K00.K93	Hungary	124.28	1	yes
K86	Other diseases of pancreas	K00.K93	Latvia	35.60	3	yes
K86	Other diseases of pancreas	K00.K93	Lithuania	37.99	2	yes
K86	Other diseases of pancreas	K00.K93	Netherlands	59.91	3	yes
K86	Other diseases of pancreas	K00.K93	Norway	19.31	2	yes
K86	Other diseases of pancreas	K00.K93	Poland	249.36	2	yes
K86	Other diseases of pancreas	K00.K93	Romania	115.27	0	no
K86	Other diseases of pancreas	K00.K93	Slovenia	10.54	0	no
K86	Other diseases of pancreas	K00.K93	Spain	101.32	0	no
K86	Other diseases of pancreas	K00.K93	Sweden	20.32	0	no
K86	Other diseases of pancreas	K00.K93	Switzerland	23.77	1	yes
K86	Other diseases of pancreas	K00.K93	United Kingdom	204.45	2	yes
K90	Intestinal malabsorption	K00.K93	France	28.15	1	yes
K90	Intestinal malabsorption	K00.K93	Germany	21.08	0	no
K90	Intestinal malabsorption	K00.K93	Hungary	5.84	0	no
K90	Intestinal malabsorption	K00.K93	Netherlands	8.51	0	no
K90	Intestinal malabsorption	K00.K93	Poland	9.70	1	yes
K90	Intestinal malabsorption	K00.K93	Spain	22.07	0	no
K90	Intestinal malabsorption	K00.K93	Switzerland	5.73	0	no
K90	Intestinal malabsorption	K00.K93	United Kingdom	48.18	0	no
K92	Other diseases of digestive system	K00.K93	Austria	81.01	0	no
K92	Other diseases of digestive system	K00.K93	Belgium	616.89	2	yes
K92	Other diseases of digestive system	K00.K93	Croatia	93.85	1	yes
K92	Other diseases of digestive system	K00.K93	Czech Republic	183.26	3	yes
K92	Other diseases of digestive system	K00.K93	Denmark	246.99	1	yes
K92	Other diseases of digestive system	K00.K93	Estonia	5.56	0	no
K92	Other diseases of digestive system	K00.K93	Finland	57.23	1	yes
K92	Other diseases of digestive system	K00.K93	France	2482.51	1	yes
K92	Other diseases of digestive system	K00.K93	Germany	4104.85	3	yes
K92	Other diseases of digestive system	K00.K93	Hungary	39.36	3	yes
K92	Other diseases of digestive system	K00.K93	Latvia	12.67	0	no
K92	Other diseases of digestive system	K00.K93	Lithuania	22.02	1	yes
K92	Other diseases of digestive system	K00.K93	Netherlands	1423.87	1	yes

For peer review only

K92	Other diseases of digestive system	K00.K93	Norway	152.29	0	no
K92	Other diseases of digestive system	K00.K93	Poland	1862.57	2	yes
K92	Other diseases of digestive system	K00.K93	Romania	433.66	0	no
K92	Other diseases of digestive system	K00.K93	Slovenia	37.63	2	yes
K92	Other diseases of digestive system	K00.K93	Spain	2353.02	3	yes
K92	Other diseases of digestive system	K00.K93	Sweden	327.87	1	yes
K92	Other diseases of digestive system	K00.K93	Switzerland	307.85	2	yes
K92	Other diseases of digestive system	K00.K93	United Kingdom	1947.79	1	yes
L02	Cutaneous abscess, furuncle and carbuncle	L00.L99	Austria	5.63	0	no
L02	Cutaneous abscess, furuncle and carbuncle	L00.L99	Czech Republic	12.75	1	yes
L02	Cutaneous abscess, furuncle and carbuncle	L00.L99	Denmark	8.24	0	no
L02	Cutaneous abscess, furuncle and carbuncle	L00.L99	France	14.74	2	yes
L02	Cutaneous abscess, furuncle and carbuncle	L00.L99	Germany	65.64	2	yes
L02	Cutaneous abscess, furuncle and carbuncle	L00.L99	Hungary	19.76	2	yes
L02	Cutaneous abscess, furuncle and carbuncle	L00.L99	Lithuania	15.38	0	no
L02	Cutaneous abscess, furuncle and carbuncle	L00.L99	Netherlands	20.59	2	yes
L02	Cutaneous abscess, furuncle and carbuncle	L00.L99	Poland	13.44	1	yes
L02	Cutaneous abscess, furuncle and carbuncle	L00.L99	Romania	8.21	0	no
L02	Cutaneous abscess, furuncle and carbuncle	L00.L99	Spain	34.64	0	no
L02	Cutaneous abscess, furuncle and carbuncle	L00.L99	Sweden	9.09	0	no
L02	Cutaneous abscess, furuncle and carbuncle	L00.L99	Switzerland	6.89	0	no
L02	Cutaneous abscess, furuncle and carbuncle	L00.L99	United Kingdom	72.87	1	yes
L03	Cellulitis	L00.L99	Austria	5.08	0	no
L03	Cellulitis	L00.L99	Belgium	7.65	1	yes
L03	Cellulitis	L00.L99	Czech Republic	23.38	3	yes
L03	Cellulitis	L00.L99	Estonia	6.92	0	no
L03	Cellulitis	L00.L99	Germany	68.27	1	yes
L03	Cellulitis	L00.L99	Latvia	14.64	0	no
L03	Cellulitis	L00.L99	Lithuania	12.85	2	yes
L03	Cellulitis	L00.L99	Netherlands	21.48	1	yes
L03	Cellulitis	L00.L99	Poland	8.08	1	yes
L03	Cellulitis	L00.L99	Romania	15.25	0	no
L03	Cellulitis	L00.L99	Spain	125.48	1	yes
L03	Cellulitis	L00.L99	Switzerland	8.28	0	no
L03	Cellulitis	L00.L99	United Kingdom	984.06	3	yes
L08	Other local infections of skin and subcutaneous tissue	L00.L99	Germany	8.80	1	yes
L08	Other local infections of skin and subcutaneous tissue	L00.L99	Hungary	7.93	0	no
L08	Other local infections of skin and subcutaneous tissue	L00.L99	Netherlands	37.56	2	yes
L08	Other local infections of skin and subcutaneous tissue	L00.L99	Norway	17.31	2	yes
L08	Other local infections of skin and subcutaneous tissue	L00.L99	Poland	75.19	3	yes
L08	Other local infections of skin and subcutaneous tissue	L00.L99	Romania	8.47	0	no
L08	Other local infections of skin and subcutaneous tissue	L00.L99	Spain	140.60	2	yes
L08	Other local infections of skin and subcutaneous tissue	L00.L99	Sweden	18.76	0	no
L08	Other local infections of skin and subcutaneous tissue	L00.L99	Switzerland	8.02	3	yes

For peer review only

L08	Other local infections of skin and subcutaneous tissue	L00.L99	United Kingdom	59.80	0	no
L10	Pemphigus	L00.L99	France	10.34	1	yes
L10	Pemphigus	L00.L99	Germany	8.37	3	yes
L10	Pemphigus	L00.L99	Poland	5.70	0	no
L10	Pemphigus	L00.L99	Spain	12.68	0	no
L10	Pemphigus	L00.L99	United Kingdom	8.16	0	no
L12	Pemphigoid	L00.L99	Belgium	8.55	0	no
L12	Pemphigoid	L00.L99	France	119.57	1	yes
L12	Pemphigoid	L00.L99	Germany	26.60	2	yes
L12	Pemphigoid	L00.L99	Netherlands	17.42	0	no
L12	Pemphigoid	L00.L99	Spain	14.52	2	yes
L12	Pemphigoid	L00.L99	Sweden	6.89	1	yes
L12	Pemphigoid	L00.L99	United Kingdom	47.72	0	no
L13	Other bullous disorders	L00.L99	France	19.94	1	yes
L30	Other dermatitis	L00.L99	United Kingdom	10.57	0	no
L40	Psoriasis	L00.L99	Germany	10.63	1	yes
L40	Psoriasis	L00.L99	Netherlands	5.25	0	no
L40	Psoriasis	L00.L99	Spain	8.94	0	no
L40	Psoriasis	L00.L99	United Kingdom	20.58	1	yes
L51	Erythema multiforme	L00.L99	France	12.52	1	yes
L51	Erythema multiforme	L00.L99	Germany	23.12	0	no
L51	Erythema multiforme	L00.L99	Spain	11.09	0	no
L51	Erythema multiforme	L00.L99	United Kingdom	19.33	0	no
L53	Other erythematous conditions	L00.L99	France	12.37	0	no
L53	Other erythematous conditions	L00.L99	United Kingdom	7.90	1	yes
L88	Pyoderma gangrenosum	L00.L99	France	6.99	0	no
L88	Pyoderma gangrenosum	L00.L99	Germany	6.39	1	yes
L88	Pyoderma gangrenosum	L00.L99	United Kingdom	5.80	0	no
L89	Decubitus ulcer	L00.L99	Austria	23.38	0	no
L89	Decubitus ulcer	L00.L99	Belgium	343.39	3	yes
L89	Decubitus ulcer	L00.L99	Croatia	10.82	3	yes
L89	Decubitus ulcer	L00.L99	Czech Republic	66.97	1	yes
L89	Decubitus ulcer	L00.L99	Denmark	26.53	1	yes
L89	Decubitus ulcer	L00.L99	France	1519.18	1	yes
L89	Decubitus ulcer	L00.L99	Germany	570.29	1	yes
L89	Decubitus ulcer	L00.L99	Latvia	17.99	1	yes
L89	Decubitus ulcer	L00.L99	Lithuania	8.39	0	no
L89	Decubitus ulcer	L00.L99	Netherlands	326.53	1	yes
L89	Decubitus ulcer	L00.L99	Norway	29.80	1	yes
L89	Decubitus ulcer	L00.L99	Romania	40.81	1	yes
L89	Decubitus ulcer	L00.L99	Slovenia	30.23	0	no
L89	Decubitus ulcer	L00.L99	Spain	745.67	2	yes
L89	Decubitus ulcer	L00.L99	Sweden	24.77	2	yes
L89	Decubitus ulcer	L00.L99	Switzerland	36.68	1	yes
L89	Decubitus ulcer	L00.L99	United Kingdom	267.64	3	yes
L93	Lupus erythematosus	L00.L99	Austria	6.07	1	yes
L93	Lupus erythematosus	L00.L99	France	19.80	1	yes
L93	Lupus erythematosus	L00.L99	Germany	32.41	0	no
L93	Lupus erythematosus	L00.L99	Romania	11.38	1	yes

For peer review only

L93	Lupus erythematosus	L00.L99	Spain	16.22	1	yes
L93	Lupus erythematosus	L00.L99	United Kingdom	5.98	1	yes
L97	Ulcer of lower limb, not elsewhere classified	L00.L99	Austria	12.96	1	yes
L97	Ulcer of lower limb, not elsewhere classified	L00.L99	Belgium	15.78	1	yes
L97	Ulcer of lower limb, not elsewhere classified	L00.L99	Denmark	16.50	2	yes
L97	Ulcer of lower limb, not elsewhere classified	L00.L99	France	118.42	3	yes
L97	Ulcer of lower limb, not elsewhere classified	L00.L99	Germany	77.57	2	yes
L97	Ulcer of lower limb, not elsewhere classified	L00.L99	Netherlands	52.63	0	no
L97	Ulcer of lower limb, not elsewhere classified	L00.L99	Norway	19.13	0	no
L97	Ulcer of lower limb, not elsewhere classified	L00.L99	Spain	44.57	1	yes
L97	Ulcer of lower limb, not elsewhere classified	L00.L99	Sweden	52.94	2	yes
L97	Ulcer of lower limb, not elsewhere classified	L00.L99	Switzerland	15.22	0	no
L97	Ulcer of lower limb, not elsewhere classified	L00.L99	United Kingdom	514.28	3	yes
L98	Other disorders of skin and subcutaneous tissue, not elsewhere classified	L00.L99	Denmark	5.73	1	yes
L98	Other disorders of skin and subcutaneous tissue, not elsewhere classified	L00.L99	France	10.29	2	yes
L98	Other disorders of skin and subcutaneous tissue, not elsewhere classified	L00.L99	Germany	23.54	2	yes
L98	Other disorders of skin and subcutaneous tissue, not elsewhere classified	L00.L99	Netherlands	10.22	0	no
L98	Other disorders of skin and subcutaneous tissue, not elsewhere classified	L00.L99	Norway	13.87	1	yes
L98	Other disorders of skin and subcutaneous tissue, not elsewhere classified	L00.L99	Spain	155.23	3	yes
L98	Other disorders of skin and subcutaneous tissue, not elsewhere classified	L00.L99	Sweden	31.06	2	yes
L98	Other disorders of skin and subcutaneous tissue, not elsewhere classified	L00.L99	Switzerland	5.11	0	no
L98	Other disorders of skin and subcutaneous tissue, not elsewhere classified	L00.L99	United Kingdom	116.42	1	yes
M00	Pyogenic arthritis	M00.M99	Austria	8.06	1	yes
M00	Pyogenic arthritis	M00.M99	Belgium	15.21	1	yes
M00	Pyogenic arthritis	M00.M99	Czech Republic	9.48	1	yes
M00	Pyogenic arthritis	M00.M99	Denmark	6.28	0	no
M00	Pyogenic arthritis	M00.M99	Finland	8.27	1	yes
M00	Pyogenic arthritis	M00.M99	France	100.84	1	yes
M00	Pyogenic arthritis	M00.M99	Germany	94.29	2	yes
M00	Pyogenic arthritis	M00.M99	Hungary	8.53	1	yes
M00	Pyogenic arthritis	M00.M99	Netherlands	39.11	1	yes
M00	Pyogenic arthritis	M00.M99	Norway	6.83	1	yes
M00	Pyogenic arthritis	M00.M99	Poland	11.60	2	yes
M00	Pyogenic arthritis	M00.M99	Spain	48.36	1	yes
M00	Pyogenic arthritis	M00.M99	Sweden	17.81	0	no
M00	Pyogenic arthritis	M00.M99	Switzerland	24.47	1	yes
M00	Pyogenic arthritis	M00.M99	United Kingdom	195.03	0	no
M05	Seropositive rheumatoid arthritis	M00.M99	Belgium	8.87	2	yes
M05	Seropositive rheumatoid arthritis	M00.M99	Czech Republic	11.71	2	yes
M05	Seropositive rheumatoid arthritis	M00.M99	Denmark	24.75	2	yes
M05	Seropositive rheumatoid arthritis	M00.M99	Estonia	8.27	0	no
M05	Seropositive rheumatoid arthritis	M00.M99	Finland	85.52	1	yes
M05	Seropositive rheumatoid arthritis	M00.M99	France	40.82	1	yes
M05	Seropositive rheumatoid arthritis	M00.M99	Germany	24.54	0	no

For peer review only

M05	Seropositive rheumatoid arthritis	M00.M99	Hungary	10.63	2	yes
M05	Seropositive rheumatoid arthritis	M00.M99	Latvia	9.14	1	yes
M05	Seropositive rheumatoid arthritis	M00.M99	Lithuania	18.02	0	no
M05	Seropositive rheumatoid arthritis	M00.M99	Netherlands	10.91	0	no
M05	Seropositive rheumatoid arthritis	M00.M99	Poland	156.21	3	yes
M05	Seropositive rheumatoid arthritis	M00.M99	Slovenia	10.07	3	yes
M05	Seropositive rheumatoid arthritis	M00.M99	Spain	32.63	0	no
M05	Seropositive rheumatoid arthritis	M00.M99	Sweden	18.54	2	yes
M05	Seropositive rheumatoid arthritis	M00.M99	Switzerland	5.82	0	no
M05	Seropositive rheumatoid arthritis	M00.M99	United Kingdom	95.76	2	yes
M06	Other rheumatoid arthritis	M00.M99	Austria	54.22	2	yes
M06	Other rheumatoid arthritis	M00.M99	Belgium	62.41	1	yes
M06	Other rheumatoid arthritis	M00.M99	Croatia	42.10	1	yes
M06	Other rheumatoid arthritis	M00.M99	Czech Republic	8.41	2	yes
M06	Other rheumatoid arthritis	M00.M99	Denmark	74.80	2	yes
M06	Other rheumatoid arthritis	M00.M99	Estonia	9.46	1	yes
M06	Other rheumatoid arthritis	M00.M99	Finland	39.67	1	yes
M06	Other rheumatoid arthritis	M00.M99	France	426.37	2	yes
M06	Other rheumatoid arthritis	M00.M99	Germany	382.12	2	yes
M06	Other rheumatoid arthritis	M00.M99	Hungary	55.79	0	no
M06	Other rheumatoid arthritis	M00.M99	Latvia	15.67	0	no
M06	Other rheumatoid arthritis	M00.M99	Lithuania	22.24	1	yes
M06	Other rheumatoid arthritis	M00.M99	Netherlands	166.55	2	yes
M06	Other rheumatoid arthritis	M00.M99	Norway	61.81	1	yes
M06	Other rheumatoid arthritis	M00.M99	Poland	235.66	1	yes
M06	Other rheumatoid arthritis	M00.M99	Slovenia	20.87	0	no
M06	Other rheumatoid arthritis	M00.M99	Spain	245.58	2	yes
M06	Other rheumatoid arthritis	M00.M99	Sweden	128.50	1	yes
M06	Other rheumatoid arthritis	M00.M99	Switzerland	69.96	2	yes
M06	Other rheumatoid arthritis	M00.M99	United Kingdom	987.01	2	yes
M10	Gout	M00.M99	Denmark	5.53	2	yes
M10	Gout	M00.M99	France	18.99	2	yes
M10	Gout	M00.M99	Germany	13.94	2	yes
M10	Gout	M00.M99	Latvia	5.16	0	no
M10	Gout	M00.M99	Netherlands	11.52	2	yes
M10	Gout	M00.M99	Switzerland	5.28	0	no
M10	Gout	M00.M99	United Kingdom	23.90	0	no
M11	Other crystal arthropathies	M00.M99	France	5.63	0	no
M13	Other arthritis	M00.M99	Austria	7.11	1	yes
M13	Other arthritis	M00.M99	Belgium	11.82	1	yes
M13	Other arthritis	M00.M99	Denmark	5.66	0	no
M13	Other arthritis	M00.M99	France	103.92	1	yes
M13	Other arthritis	M00.M99	Germany	51.66	1	yes
M13	Other arthritis	M00.M99	Hungary	26.64	0	no
M13	Other arthritis	M00.M99	Netherlands	12.85	0	no
M13	Other arthritis	M00.M99	Norway	6.57	3	yes
M13	Other arthritis	M00.M99	Spain	16.52	1	yes
M13	Other arthritis	M00.M99	Sweden	5.04	3	yes
M13	Other arthritis	M00.M99	Switzerland	13.27	0	no
M13	Other arthritis	M00.M99	United Kingdom	88.03	2	yes

For peer review only

M15	Polyarthrosis	M00.M99	Austria	8.70	1	yes
M15	Polyarthrosis	M00.M99	Belgium	20.63	0	no
M15	Polyarthrosis	M00.M99	Denmark	5.07	0	no
M15	Polyarthrosis	M00.M99	Finland	9.90	0	no
M15	Polyarthrosis	M00.M99	France	63.23	1	yes
M15	Polyarthrosis	M00.M99	Germany	90.61	1	yes
M15	Polyarthrosis	M00.M99	Hungary	11.72	2	yes
M15	Polyarthrosis	M00.M99	Netherlands	24.26	0	no
M15	Polyarthrosis	M00.M99	Poland	5.42	0	no
M15	Polyarthrosis	M00.M99	Spain	40.48	0	no
M15	Polyarthrosis	M00.M99	Switzerland	72.97	0	no
M15	Polyarthrosis	M00.M99	United Kingdom	28.42	1	yes
M16	Coxarthrosis [arthrosis of hip]	M00.M99	Belgium	6.77	1	yes
M16	Coxarthrosis [arthrosis of hip]	M00.M99	Denmark	15.09	0	no
M16	Coxarthrosis [arthrosis of hip]	M00.M99	Finland	12.61	1	yes
M16	Coxarthrosis [arthrosis of hip]	M00.M99	France	53.96	1	yes
M16	Coxarthrosis [arthrosis of hip]	M00.M99	Germany	70.48	0	no
M16	Coxarthrosis [arthrosis of hip]	M00.M99	Hungary	28.85	0	no
M16	Coxarthrosis [arthrosis of hip]	M00.M99	Netherlands	78.72	1	yes
M16	Coxarthrosis [arthrosis of hip]	M00.M99	Norway	22.95	2	yes
M16	Coxarthrosis [arthrosis of hip]	M00.M99	Spain	10.71	0	no
M16	Coxarthrosis [arthrosis of hip]	M00.M99	Sweden	12.52	0	no
M16	Coxarthrosis [arthrosis of hip]	M00.M99	Switzerland	40.31	0	no
M16	Coxarthrosis [arthrosis of hip]	M00.M99	United Kingdom	27.62	1	yes
M17	Gonarthrosis [arthrosis of knee]	M00.M99	Belgium	7.65	1	yes
M17	Gonarthrosis [arthrosis of knee]	M00.M99	Denmark	7.72	0	no
M17	Gonarthrosis [arthrosis of knee]	M00.M99	Finland	8.95	1	yes
M17	Gonarthrosis [arthrosis of knee]	M00.M99	France	22.62	3	yes
M17	Gonarthrosis [arthrosis of knee]	M00.M99	Germany	38.85	1	yes
M17	Gonarthrosis [arthrosis of knee]	M00.M99	Hungary	7.27	0	no
M17	Gonarthrosis [arthrosis of knee]	M00.M99	Netherlands	24.04	1	yes
M17	Gonarthrosis [arthrosis of knee]	M00.M99	Spain	8.37	1	yes
M17	Gonarthrosis [arthrosis of knee]	M00.M99	Sweden	6.75	0	no
M17	Gonarthrosis [arthrosis of knee]	M00.M99	Switzerland	18.52	1	yes
M17	Gonarthrosis [arthrosis of knee]	M00.M99	United Kingdom	17.81	1	yes
M19	Other arthrosis	M00.M99	Belgium	14.19	0	no
M19	Other arthrosis	M00.M99	Denmark	5.06	1	yes
M19	Other arthrosis	M00.M99	France	95.29	1	yes
M19	Other arthrosis	M00.M99	Germany	20.87	0	no
M19	Other arthrosis	M00.M99	Netherlands	57.78	2	yes
M19	Other arthrosis	M00.M99	Spain	51.26	1	yes
M19	Other arthrosis	M00.M99	Switzerland	11.18	1	yes
M19	Other arthrosis	M00.M99	United Kingdom	266.48	1	yes
M24	Other specific joint derangements	M00.M99	Netherlands	5.60	0	no
M24	Other specific joint derangements	M00.M99	United Kingdom	6.37	1	yes
M25	Other joint disorders, not elsewhere classified	M00.M99	Belgium	8.54	2	yes
M25	Other joint disorders, not elsewhere classified	M00.M99	Germany	66.56	0	no
M25	Other joint disorders, not elsewhere classified	M00.M99	Netherlands	150.30	1	yes
M25	Other joint disorders, not elsewhere classified	M00.M99	Spain	103.86	3	yes
M25	Other joint disorders, not elsewhere classified	M00.M99	Sweden	6.17	0	no

For peer review only

M25	Other joint disorders, not elsewhere classified	M00.M99	United Kingdom	391.58	3	yes
M30	Polyarteritis nodosa and related conditions	M00.M99	Belgium	8.39	1	yes
M30	Polyarteritis nodosa and related conditions	M00.M99	France	51.00	2	yes
M30	Polyarteritis nodosa and related conditions	M00.M99	Germany	22.96	2	yes
M30	Polyarteritis nodosa and related conditions	M00.M99	Poland	8.92	0	no
M30	Polyarteritis nodosa and related conditions	M00.M99	Spain	21.24	1	yes
M30	Polyarteritis nodosa and related conditions	M00.M99	United Kingdom	37.34	2	yes
M31	Other necrotizing vasculopathies	M00.M99	Austria	20.71	2	yes
M31	Other necrotizing vasculopathies	M00.M99	Belgium	33.73	0	no
M31	Other necrotizing vasculopathies	M00.M99	Czech Republic	11.03	1	yes
M31	Other necrotizing vasculopathies	M00.M99	Denmark	30.46	0	no
M31	Other necrotizing vasculopathies	M00.M99	Finland	19.82	0	no
M31	Other necrotizing vasculopathies	M00.M99	France	316.27	2	yes
M31	Other necrotizing vasculopathies	M00.M99	Germany	178.19	1	yes
M31	Other necrotizing vasculopathies	M00.M99	Hungary	19.36	2	yes
M31	Other necrotizing vasculopathies	M00.M99	Lithuania	5.41	0	no
M31	Other necrotizing vasculopathies	M00.M99	Netherlands	67.31	2	yes
M31	Other necrotizing vasculopathies	M00.M99	Norway	16.68	0	no
M31	Other necrotizing vasculopathies	M00.M99	Poland	43.33	1	yes
M31	Other necrotizing vasculopathies	M00.M99	Romania	5.59	0	no
M31	Other necrotizing vasculopathies	M00.M99	Slovenia	6.69	1	yes
M31	Other necrotizing vasculopathies	M00.M99	Spain	89.61	0	no
M31	Other necrotizing vasculopathies	M00.M99	Sweden	34.34	0	no
M31	Other necrotizing vasculopathies	M00.M99	Switzerland	24.89	0	no
M31	Other necrotizing vasculopathies	M00.M99	United Kingdom	168.88	1	yes
M32	Systemic lupus erythematosus	M00.M99	Austria	9.35	1	yes
M32	Systemic lupus erythematosus	M00.M99	Belgium	9.44	0	no
M32	Systemic lupus erythematosus	M00.M99	Croatia	13.94	0	no
M32	Systemic lupus erythematosus	M00.M99	Czech Republic	11.38	2	yes
M32	Systemic lupus erythematosus	M00.M99	Denmark	11.14	1	yes
M32	Systemic lupus erythematosus	M00.M99	Finland	7.84	1	yes
M32	Systemic lupus erythematosus	M00.M99	France	42.62	2	yes
M32	Systemic lupus erythematosus	M00.M99	Germany	53.88	1	yes
M32	Systemic lupus erythematosus	M00.M99	Hungary	29.15	0	no
M32	Systemic lupus erythematosus	M00.M99	Latvia	5.85	0	no
M32	Systemic lupus erythematosus	M00.M99	Lithuania	6.12	0	no
M32	Systemic lupus erythematosus	M00.M99	Netherlands	22.45	0	no
M32	Systemic lupus erythematosus	M00.M99	Norway	13.00	1	yes
M32	Systemic lupus erythematosus	M00.M99	Poland	91.37	0	no
M32	Systemic lupus erythematosus	M00.M99	Romania	12.96	1	yes
M32	Systemic lupus erythematosus	M00.M99	Slovenia	5.69	0	no
M32	Systemic lupus erythematosus	M00.M99	Spain	79.32	0	no
M32	Systemic lupus erythematosus	M00.M99	Sweden	23.04	1	yes
M32	Systemic lupus erythematosus	M00.M99	Switzerland	9.68	0	no
M32	Systemic lupus erythematosus	M00.M99	United Kingdom	90.14	0	no
M33	Dermatopolymyositis	M00.M99	Belgium	7.76	0	no
M33	Dermatopolymyositis	M00.M99	Finland	5.76	2	yes
M33	Dermatopolymyositis	M00.M99	France	44.56	0	no
M33	Dermatopolymyositis	M00.M99	Germany	25.74	1	yes
M33	Dermatopolymyositis	M00.M99	Hungary	5.69	0	no

For peer review only

M33	Dermatopolymyositis	M00.M99	Netherlands	10.48	0	no
M33	Dermatopolymyositis	M00.M99	Poland	10.88	0	no
M33	Dermatopolymyositis	M00.M99	Spain	25.74	0	no
M33	Dermatopolymyositis	M00.M99	Sweden	5.01	3	yes
M33	Dermatopolymyositis	M00.M99	Switzerland	5.18	0	no
M33	Dermatopolymyositis	M00.M99	United Kingdom	44.44	1	yes
M34	Systemic sclerosis	M00.M99	Austria	14.38	2	yes
M34	Systemic sclerosis	M00.M99	Belgium	22.90	0	no
M34	Systemic sclerosis	M00.M99	Croatia	6.25	0	no
M34	Systemic sclerosis	M00.M99	Denmark	14.98	1	yes
M34	Systemic sclerosis	M00.M99	Finland	9.87	0	no
M34	Systemic sclerosis	M00.M99	France	159.45	2	yes
M34	Systemic sclerosis	M00.M99	Germany	126.91	1	yes
M34	Systemic sclerosis	M00.M99	Hungary	15.01	0	no
M34	Systemic sclerosis	M00.M99	Netherlands	43.27	2	yes
M34	Systemic sclerosis	M00.M99	Norway	9.68	0	no
M34	Systemic sclerosis	M00.M99	Poland	51.40	0	no
M34	Systemic sclerosis	M00.M99	Romania	5.77	0	no
M34	Systemic sclerosis	M00.M99	Slovenia	5.88	3	yes
M34	Systemic sclerosis	M00.M99	Spain	92.54	1	yes
M34	Systemic sclerosis	M00.M99	Sweden	23.17	0	no
M34	Systemic sclerosis	M00.M99	Switzerland	27.72	0	no
M34	Systemic sclerosis	M00.M99	United Kingdom	193.59	1	yes
M35	Other systemic involvement of connective tissue	M00.M99	Austria	11.09	0	no
M35	Other systemic involvement of connective tissue	M00.M99	Belgium	29.03	1	yes
M35	Other systemic involvement of connective tissue	M00.M99	Croatia	7.73	0	no
M35	Other systemic involvement of connective tissue	M00.M99	Denmark	26.63	2	yes
M35	Other systemic involvement of connective tissue	M00.M99	Finland	14.14	0	no
M35	Other systemic involvement of connective tissue	M00.M99	France	167.10	2	yes
M35	Other systemic involvement of connective tissue	M00.M99	Germany	79.49	3	yes
M35	Other systemic involvement of connective tissue	M00.M99	Hungary	15.07	0	no
M35	Other systemic involvement of connective tissue	M00.M99	Netherlands	57.65	2	yes
M35	Other systemic involvement of connective tissue	M00.M99	Norway	17.11	1	yes
M35	Other systemic involvement of connective tissue	M00.M99	Poland	20.73	0	no
M35	Other systemic involvement of connective tissue	M00.M99	Slovenia	6.09	0	no
M35	Other systemic involvement of connective tissue	M00.M99	Spain	65.52	2	yes
M35	Other systemic involvement of connective tissue	M00.M99	Sweden	22.56	0	no
M35	Other systemic involvement of connective tissue	M00.M99	Switzerland	30.21	2	yes
M35	Other systemic involvement of connective tissue	M00.M99	United Kingdom	76.82	0	no
M40	Kyphosis and lordosis	M00.M99	Netherlands	6.69	0	no
M40	Kyphosis and lordosis	M00.M99	Spain	8.91	0	no
M40	Kyphosis and lordosis	M00.M99	United Kingdom	43.28	0	no
M41	Scoliosis	M00.M99	Austria	8.52	3	yes
M41	Scoliosis	M00.M99	Belgium	13.56	3	yes
M41	Scoliosis	M00.M99	France	180.46	1	yes
M41	Scoliosis	M00.M99	Germany	51.70	0	no
M41	Scoliosis	M00.M99	Netherlands	21.28	1	yes
M41	Scoliosis	M00.M99	Spain	55.28	1	yes
M41	Scoliosis	M00.M99	Sweden	12.73	2	yes
M41	Scoliosis	M00.M99	Switzerland	6.68	0	no

For peer review only

M41	Scoliosis	M00.M99	United Kingdom	162.54	1	yes
M43	Other deforming dorsopathies	M00.M99	Germany	6.13	1	yes
M45	Ankylosing spondylitis	M00.M99	France	7.04	2	yes
M45	Ankylosing spondylitis	M00.M99	Germany	40.61	1	yes
M45	Ankylosing spondylitis	M00.M99	Hungary	7.20	0	no
M45	Ankylosing spondylitis	M00.M99	Netherlands	5.84	1	yes
M45	Ankylosing spondylitis	M00.M99	Norway	6.24	0	no
M45	Ankylosing spondylitis	M00.M99	Spain	12.81	0	no
M45	Ankylosing spondylitis	M00.M99	United Kingdom	21.06	0	no
M46	Other inflammatory spondylopathies	M00.M99	Austria	16.85	3	yes
M46	Other inflammatory spondylopathies	M00.M99	Belgium	13.54	1	yes
M46	Other inflammatory spondylopathies	M00.M99	Czech Republic	15.56	2	yes
M46	Other inflammatory spondylopathies	M00.M99	Denmark	9.33	0	no
M46	Other inflammatory spondylopathies	M00.M99	France	98.57	1	yes
M46	Other inflammatory spondylopathies	M00.M99	Germany	196.19	2	yes
M46	Other inflammatory spondylopathies	M00.M99	Hungary	8.03	1	yes
M46	Other inflammatory spondylopathies	M00.M99	Latvia	5.56	2	yes
M46	Other inflammatory spondylopathies	M00.M99	Netherlands	28.36	1	yes
M46	Other inflammatory spondylopathies	M00.M99	Spain	31.23	2	yes
M46	Other inflammatory spondylopathies	M00.M99	Sweden	7.40	1	yes
M46	Other inflammatory spondylopathies	M00.M99	Switzerland	18.15	0	no
M46	Other inflammatory spondylopathies	M00.M99	United Kingdom	82.77	2	yes
M47	Spondylosis	M00.M99	France	39.66	3	yes
M47	Spondylosis	M00.M99	Germany	17.38	1	yes
M47	Spondylosis	M00.M99	Hungary	6.77	0	no
M47	Spondylosis	M00.M99	Spain	11.26	0	no
M47	Spondylosis	M00.M99	Switzerland	10.00	3	yes
M47	Spondylosis	M00.M99	United Kingdom	30.83	1	yes
M48	Other spondylopathies	M00.M99	Belgium	15.83	0	no
M48	Other spondylopathies	M00.M99	Denmark	9.63	1	yes
M48	Other spondylopathies	M00.M99	Finland	6.96	0	no
M48	Other spondylopathies	M00.M99	France	76.36	2	yes
M48	Other spondylopathies	M00.M99	Germany	41.22	1	yes
M48	Other spondylopathies	M00.M99	Netherlands	29.90	3	yes
M48	Other spondylopathies	M00.M99	Norway	8.63	1	yes
M48	Other spondylopathies	M00.M99	Spain	19.99	2	yes
M48	Other spondylopathies	M00.M99	Sweden	13.62	0	no
M48	Other spondylopathies	M00.M99	Switzerland	12.47	0	no
M48	Other spondylopathies	M00.M99	United Kingdom	42.46	0	no
M50	Cervical disc disorders	M00.M99	Germany	8.69	0	no
M50	Cervical disc disorders	M00.M99	Spain	6.07	0	no
M51	Other intervertebral disc disorders	M00.M99	France	13.64	0	no
M51	Other intervertebral disc disorders	M00.M99	Germany	10.96	0	no
M51	Other intervertebral disc disorders	M00.M99	Hungary	6.88	1	yes
M51	Other intervertebral disc disorders	M00.M99	United Kingdom	7.08	0	no
M54	Dorsalgia	M00.M99	Germany	6.08	1	yes
M54	Dorsalgia	M00.M99	Netherlands	8.55	1	yes
M54	Dorsalgia	M00.M99	Switzerland	10.29	0	no
M54	Dorsalgia	M00.M99	United Kingdom	5.76	2	yes
M60	Myositis	M00.M99	Germany	19.03	0	no

For peer review only

M60	Myositis	M00.M99	Netherlands	14.11	0	no
M60	Myositis	M00.M99	Spain	14.36	1	yes
M60	Myositis	M00.M99	United Kingdom	67.00	3	yes
M62	Other disorders of muscle	M00.M99	Austria	9.18	0	no
M62	Other disorders of muscle	M00.M99	Belgium	27.13	0	no
M62	Other disorders of muscle	M00.M99	Denmark	8.56	2	yes
M62	Other disorders of muscle	M00.M99	France	206.98	1	yes
M62	Other disorders of muscle	M00.M99	Germany	164.55	1	yes
M62	Other disorders of muscle	M00.M99	Hungary	13.17	1	yes
M62	Other disorders of muscle	M00.M99	Netherlands	31.78	2	yes
M62	Other disorders of muscle	M00.M99	Norway	6.50	0	no
M62	Other disorders of muscle	M00.M99	Spain	161.22	2	yes
M62	Other disorders of muscle	M00.M99	Sweden	12.09	0	no
M62	Other disorders of muscle	M00.M99	Switzerland	9.92	0	no
M62	Other disorders of muscle	M00.M99	United Kingdom	106.21	1	yes
M70	Soft tissue disorders related to use, overuse and pressure	M00.M99	Germany	6.83	0	no
M70	Soft tissue disorders related to use, overuse and pressure	M00.M99	United Kingdom	5.35	0	no
M72	Fibroblastic disorders	M00.M99	Belgium	10.35	1	yes
M72	Fibroblastic disorders	M00.M99	Denmark	8.64	1	yes
M72	Fibroblastic disorders	M00.M99	France	47.01	1	yes
M72	Fibroblastic disorders	M00.M99	Germany	96.73	1	yes
M72	Fibroblastic disorders	M00.M99	Netherlands	8.25	2	yes
M72	Fibroblastic disorders	M00.M99	Spain	53.36	2	yes
M72	Fibroblastic disorders	M00.M99	Sweden	8.30	1	yes
M72	Fibroblastic disorders	M00.M99	Switzerland	13.31	1	yes
M72	Fibroblastic disorders	M00.M99	United Kingdom	119.04	1	yes
M79	Other soft tissue disorders, not elsewhere classified	M00.M99	Germany	28.50	2	yes
M79	Other soft tissue disorders, not elsewhere classified	M00.M99	Netherlands	25.84	2	yes
M79	Other soft tissue disorders, not elsewhere classified	M00.M99	Spain	31.93	3	yes
M79	Other soft tissue disorders, not elsewhere classified	M00.M99	Sweden	6.46	1	yes
M79	Other soft tissue disorders, not elsewhere classified	M00.M99	Switzerland	6.19	0	no
M80	Osteoporosis with pathological fracture	M00.M99	Austria	32.58	0	no
M80	Osteoporosis with pathological fracture	M00.M99	Belgium	29.66	1	yes
M80	Osteoporosis with pathological fracture	M00.M99	Croatia	14.85	1	yes
M80	Osteoporosis with pathological fracture	M00.M99	Czech Republic	6.90	1	yes
M80	Osteoporosis with pathological fracture	M00.M99	Denmark	41.41	0	no
M80	Osteoporosis with pathological fracture	M00.M99	Finland	14.14	0	no
M80	Osteoporosis with pathological fracture	M00.M99	France	75.59	1	yes
M80	Osteoporosis with pathological fracture	M00.M99	Germany	199.09	1	yes
M80	Osteoporosis with pathological fracture	M00.M99	Hungary	66.93	1	yes
M80	Osteoporosis with pathological fracture	M00.M99	Netherlands	67.51	3	yes
M80	Osteoporosis with pathological fracture	M00.M99	Norway	19.15	2	yes
M80	Osteoporosis with pathological fracture	M00.M99	Poland	10.30	2	yes
M80	Osteoporosis with pathological fracture	M00.M99	Spain	182.53	1	yes
M80	Osteoporosis with pathological fracture	M00.M99	Sweden	24.09	0	no
M80	Osteoporosis with pathological fracture	M00.M99	Switzerland	35.20	0	no
M80	Osteoporosis with pathological fracture	M00.M99	United Kingdom	1306.36	1	yes
M81	Osteoporosis without pathological fracture	M00.M99	Austria	33.00	1	yes
M81	Osteoporosis without pathological fracture	M00.M99	Belgium	22.42	3	yes

For peer review only

M81	Osteoporosis without pathological fracture	M00.M99	Denmark	72.41	0	no
M81	Osteoporosis without pathological fracture	M00.M99	France	87.15	1	yes
M81	Osteoporosis without pathological fracture	M00.M99	Germany	218.42	3	yes
M81	Osteoporosis without pathological fracture	M00.M99	Hungary	30.00	0	no
M81	Osteoporosis without pathological fracture	M00.M99	Netherlands	67.74	0	no
M81	Osteoporosis without pathological fracture	M00.M99	Norway	28.32	2	yes
M81	Osteoporosis without pathological fracture	M00.M99	Spain	46.03	0	no
M81	Osteoporosis without pathological fracture	M00.M99	Sweden	25.26	1	yes
M81	Osteoporosis without pathological fracture	M00.M99	Switzerland	57.78	0	no
M81	Osteoporosis without pathological fracture	M00.M99	United Kingdom	350.52	2	yes
M84	Disorders of continuity of bone	M00.M99	Belgium	5.40	1	yes
M84	Disorders of continuity of bone	M00.M99	Estonia	15.97	1	yes
M84	Disorders of continuity of bone	M00.M99	France	31.97	1	yes
M84	Disorders of continuity of bone	M00.M99	Germany	12.66	0	no
M84	Disorders of continuity of bone	M00.M99	Spain	2326.13	3	yes
M84	Disorders of continuity of bone	M00.M99	United Kingdom	63.88	2	yes
M86	Osteomyelitis	M00.M99	Austria	11.12	0	no
M86	Osteomyelitis	M00.M99	Belgium	32.20	0	no
M86	Osteomyelitis	M00.M99	Croatia	5.33	0	no
M86	Osteomyelitis	M00.M99	Czech Republic	13.54	2	yes
M86	Osteomyelitis	M00.M99	Denmark	9.76	1	yes
M86	Osteomyelitis	M00.M99	France	228.67	0	no
M86	Osteomyelitis	M00.M99	Germany	126.91	1	yes
M86	Osteomyelitis	M00.M99	Hungary	42.41	2	yes
M86	Osteomyelitis	M00.M99	Latvia	8.64	0	no
M86	Osteomyelitis	M00.M99	Lithuania	10.53	0	no
M86	Osteomyelitis	M00.M99	Netherlands	40.00	1	yes
M86	Osteomyelitis	M00.M99	Norway	21.83	1	yes
M86	Osteomyelitis	M00.M99	Poland	14.65	2	yes
M86	Osteomyelitis	M00.M99	Spain	70.53	0	no
M86	Osteomyelitis	M00.M99	Sweden	14.23	1	yes
M86	Osteomyelitis	M00.M99	Switzerland	19.42	3	yes
M86	Osteomyelitis	M00.M99	United Kingdom	240.30	2	yes
M87	Osteonecrosis	M00.M99	France	9.17	0	no
M87	Osteonecrosis	M00.M99	Germany	13.48	2	yes
M87	Osteonecrosis	M00.M99	Hungary	6.24	2	yes
M87	Osteonecrosis	M00.M99	United Kingdom	5.32	0	no
M88	Paget's disease of bone [osteitis deformans]	M00.M99	Belgium	6.81	1	yes
M88	Paget's disease of bone [osteitis deformans]	M00.M99	France	40.12	2	yes
M88	Paget's disease of bone [osteitis deformans]	M00.M99	Spain	26.99	1	yes
M88	Paget's disease of bone [osteitis deformans]	M00.M99	United Kingdom	22.61	2	yes
M89	Other disorders of bone	M00.M99	Belgium	48.21	0	no
M89	Other disorders of bone	M00.M99	France	1226.53	1	yes
M89	Other disorders of bone	M00.M99	Germany	12.38	2	yes
M89	Other disorders of bone	M00.M99	Spain	8.75	1	yes
M95	Other acquired deformities of musculoskeletal system	M00.M99	United Kingdom	5.89	0	no
N00	Acute nephritic syndrome	N00.N99	Germany	9.65	0	no
N00	Acute nephritic syndrome	N00.N99	Hungary	8.26	0	no
N00	Acute nephritic syndrome	N00.N99	Poland	12.72	3	yes

For peer review only

N00	Acute nephritic syndrome	N00.N99	Romania	8.12	1	yes
N00	Acute nephritic syndrome	N00.N99	United Kingdom	21.92	3	yes
N01	Rapidly progressive nephritic syndrome	N00.N99	Poland	6.56	1	yes
N02	Recurrent and persistent haematuria	N00.N99	France	10.43	2	yes
N02	Recurrent and persistent haematuria	N00.N99	Germany	6.10	0	no
N02	Recurrent and persistent haematuria	N00.N99	United Kingdom	22.18	1	yes
N03	Chronic nephritic syndrome	N00.N99	Croatia	6.98	0	no
N03	Chronic nephritic syndrome	N00.N99	Czech Republic	9.30	2	yes
N03	Chronic nephritic syndrome	N00.N99	Denmark	16.50	1	yes
N03	Chronic nephritic syndrome	N00.N99	Estonia	7.60	1	yes
N03	Chronic nephritic syndrome	N00.N99	Finland	16.20	1	yes
N03	Chronic nephritic syndrome	N00.N99	France	14.37	0	no
N03	Chronic nephritic syndrome	N00.N99	Germany	35.30	0	no
N03	Chronic nephritic syndrome	N00.N99	Hungary	91.24	0	no
N03	Chronic nephritic syndrome	N00.N99	Latvia	62.72	3	yes
N03	Chronic nephritic syndrome	N00.N99	Lithuania	24.95	2	yes
N03	Chronic nephritic syndrome	N00.N99	Netherlands	6.93	0	no
N03	Chronic nephritic syndrome	N00.N99	Norway	6.32	0	no
N03	Chronic nephritic syndrome	N00.N99	Poland	70.77	2	yes
N03	Chronic nephritic syndrome	N00.N99	Romania	67.26	3	yes
N03	Chronic nephritic syndrome	N00.N99	Spain	55.64	1	yes
N03	Chronic nephritic syndrome	N00.N99	Sweden	22.80	1	yes
N03	Chronic nephritic syndrome	N00.N99	Switzerland	8.06	1	yes
N03	Chronic nephritic syndrome	N00.N99	United Kingdom	433.22	1	yes
N04	Nephrotic syndrome	N00.N99	Belgium	9.16	0	no
N04	Nephrotic syndrome	N00.N99	Denmark	6.99	1	yes
N04	Nephrotic syndrome	N00.N99	France	31.66	2	yes
N04	Nephrotic syndrome	N00.N99	Germany	25.59	0	no
N04	Nephrotic syndrome	N00.N99	Hungary	9.22	0	no
N04	Nephrotic syndrome	N00.N99	Netherlands	18.97	1	yes
N04	Nephrotic syndrome	N00.N99	Poland	23.89	0	no
N04	Nephrotic syndrome	N00.N99	Romania	5.29	0	no
N04	Nephrotic syndrome	N00.N99	Spain	31.87	1	yes
N04	Nephrotic syndrome	N00.N99	Sweden	6.60	1	yes
N04	Nephrotic syndrome	N00.N99	Switzerland	6.00	0	no
N04	Nephrotic syndrome	N00.N99	United Kingdom	46.86	0	no
N05	Unspecified nephritic syndrome	N00.N99	Austria	10.05	0	no
N05	Unspecified nephritic syndrome	N00.N99	Belgium	10.53	1	yes
N05	Unspecified nephritic syndrome	N00.N99	Denmark	20.35	1	yes
N05	Unspecified nephritic syndrome	N00.N99	France	36.03	1	yes
N05	Unspecified nephritic syndrome	N00.N99	Germany	58.39	2	yes
N05	Unspecified nephritic syndrome	N00.N99	Hungary	14.63	0	no
N05	Unspecified nephritic syndrome	N00.N99	Netherlands	11.07	2	yes
N05	Unspecified nephritic syndrome	N00.N99	Norway	7.74	0	no
N05	Unspecified nephritic syndrome	N00.N99	Poland	27.56	1	yes
N05	Unspecified nephritic syndrome	N00.N99	Romania	18.55	1	yes
N05	Unspecified nephritic syndrome	N00.N99	Spain	33.83	0	no
N05	Unspecified nephritic syndrome	N00.N99	Sweden	6.55	2	yes
N05	Unspecified nephritic syndrome	N00.N99	Switzerland	12.49	1	yes
N05	Unspecified nephritic syndrome	N00.N99	United Kingdom	64.20	0	no

For peer review only

N10	Acute tubulo-interstitial nephritis	N00.N99	Austria	8.97	2	yes
N10	Acute tubulo-interstitial nephritis	N00.N99	Belgium	11.48	0	no
N10	Acute tubulo-interstitial nephritis	N00.N99	Croatia	51.19	2	yes
N10	Acute tubulo-interstitial nephritis	N00.N99	Czech Republic	115.67	0	no
N10	Acute tubulo-interstitial nephritis	N00.N99	Denmark	14.98	0	no
N10	Acute tubulo-interstitial nephritis	N00.N99	Estonia	8.65	2	yes
N10	Acute tubulo-interstitial nephritis	N00.N99	Finland	170.99	1	yes
N10	Acute tubulo-interstitial nephritis	N00.N99	France	159.24	1	yes
N10	Acute tubulo-interstitial nephritis	N00.N99	Germany	69.79	1	yes
N10	Acute tubulo-interstitial nephritis	N00.N99	Hungary	49.37	2	yes
N10	Acute tubulo-interstitial nephritis	N00.N99	Lithuania	10.67	3	yes
N10	Acute tubulo-interstitial nephritis	N00.N99	Netherlands	24.72	3	yes
N10	Acute tubulo-interstitial nephritis	N00.N99	Poland	82.22	3	yes
N10	Acute tubulo-interstitial nephritis	N00.N99	Romania	54.67	0	no
N10	Acute tubulo-interstitial nephritis	N00.N99	Slovenia	17.80	0	no
N10	Acute tubulo-interstitial nephritis	N00.N99	Spain	64.44	1	yes
N10	Acute tubulo-interstitial nephritis	N00.N99	Sweden	43.70	1	yes
N10	Acute tubulo-interstitial nephritis	N00.N99	United Kingdom	164.73	1	yes
N11	Chronic tubulo-interstitial nephritis	N00.N99	Austria	6.29	2	yes
N11	Chronic tubulo-interstitial nephritis	N00.N99	Belgium	6.66	0	no
N11	Chronic tubulo-interstitial nephritis	N00.N99	Croatia	89.33	0	no
N11	Chronic tubulo-interstitial nephritis	N00.N99	Czech Republic	118.21	1	yes
N11	Chronic tubulo-interstitial nephritis	N00.N99	Denmark	10.82	1	yes
N11	Chronic tubulo-interstitial nephritis	N00.N99	Estonia	68.27	1	yes
N11	Chronic tubulo-interstitial nephritis	N00.N99	Finland	28.06	1	yes
N11	Chronic tubulo-interstitial nephritis	N00.N99	France	29.97	2	yes
N11	Chronic tubulo-interstitial nephritis	N00.N99	Germany	80.29	2	yes
N11	Chronic tubulo-interstitial nephritis	N00.N99	Hungary	112.23	2	yes
N11	Chronic tubulo-interstitial nephritis	N00.N99	Latvia	128.51	3	yes
N11	Chronic tubulo-interstitial nephritis	N00.N99	Lithuania	107.99	0	no
N11	Chronic tubulo-interstitial nephritis	N00.N99	Netherlands	9.16	1	yes
N11	Chronic tubulo-interstitial nephritis	N00.N99	Poland	441.39	3	yes
N11	Chronic tubulo-interstitial nephritis	N00.N99	Romania	193.14	2	yes
N11	Chronic tubulo-interstitial nephritis	N00.N99	Slovenia	9.63	0	no
N11	Chronic tubulo-interstitial nephritis	N00.N99	Spain	17.44	0	no
N11	Chronic tubulo-interstitial nephritis	N00.N99	Sweden	12.10	2	yes
N11	Chronic tubulo-interstitial nephritis	N00.N99	Switzerland	12.65	2	yes
N11	Chronic tubulo-interstitial nephritis	N00.N99	United Kingdom	48.45	1	yes
N12	Tubulo-interstitial nephritis, not specified as acute or chronic	N00.N99	Austria	38.10	1	yes
N12	Tubulo-interstitial nephritis, not specified as acute or chronic	N00.N99	Belgium	95.87	2	yes
N12	Tubulo-interstitial nephritis, not specified as acute or chronic	N00.N99	Croatia	6.73	0	no
N12	Tubulo-interstitial nephritis, not specified as acute or chronic	N00.N99	Czech Republic	39.34	2	yes
N12	Tubulo-interstitial nephritis, not specified as acute or chronic	N00.N99	Denmark	20.72	1	yes
N12	Tubulo-interstitial nephritis, not specified as acute or chronic	N00.N99	Finland	14.79	2	yes
N12	Tubulo-interstitial nephritis, not specified as acute or chronic	N00.N99	France	450.01	2	yes

For peer review only

N12	Tubulo-interstitial nephritis, not specified as acute or chronic	N00.N99	Germany	117.94	2	yes
N12	Tubulo-interstitial nephritis, not specified as acute or chronic	N00.N99	Hungary	33.69	2	yes
N12	Tubulo-interstitial nephritis, not specified as acute or chronic	N00.N99	Netherlands	36.98	1	yes
N12	Tubulo-interstitial nephritis, not specified as acute or chronic	N00.N99	Norway	57.69	1	yes
N12	Tubulo-interstitial nephritis, not specified as acute or chronic	N00.N99	Poland	17.16	0	no
N12	Tubulo-interstitial nephritis, not specified as acute or chronic	N00.N99	Romania	44.46	1	yes
N12	Tubulo-interstitial nephritis, not specified as acute or chronic	N00.N99	Spain	81.25	1	yes
N12	Tubulo-interstitial nephritis, not specified as acute or chronic	N00.N99	Sweden	31.18	2	yes
N12	Tubulo-interstitial nephritis, not specified as acute or chronic	N00.N99	Switzerland	31.10	1	yes
N12	Tubulo-interstitial nephritis, not specified as acute or chronic	N00.N99	United Kingdom	177.41	1	yes
N13	Obstructive and reflux uropathy	N00.N99	Austria	17.85	0	no
N13	Obstructive and reflux uropathy	N00.N99	Belgium	21.85	2	yes
N13	Obstructive and reflux uropathy	N00.N99	Croatia	7.21	2	yes
N13	Obstructive and reflux uropathy	N00.N99	Czech Republic	40.90	0	no
N13	Obstructive and reflux uropathy	N00.N99	Denmark	24.05	0	no
N13	Obstructive and reflux uropathy	N00.N99	Finland	10.47	0	no
N13	Obstructive and reflux uropathy	N00.N99	France	87.96	3	yes
N13	Obstructive and reflux uropathy	N00.N99	Germany	171.56	2	yes
N13	Obstructive and reflux uropathy	N00.N99	Hungary	27.08	2	yes
N13	Obstructive and reflux uropathy	N00.N99	Latvia	8.81	2	yes
N13	Obstructive and reflux uropathy	N00.N99	Lithuania	9.92	0	no
N13	Obstructive and reflux uropathy	N00.N99	Netherlands	28.22	2	yes
N13	Obstructive and reflux uropathy	N00.N99	Norway	11.36	1	yes
N13	Obstructive and reflux uropathy	N00.N99	Poland	61.04	0	no
N13	Obstructive and reflux uropathy	N00.N99	Romania	37.32	0	no
N13	Obstructive and reflux uropathy	N00.N99	Spain	102.69	0	no
N13	Obstructive and reflux uropathy	N00.N99	Sweden	23.06	0	no
N13	Obstructive and reflux uropathy	N00.N99	Switzerland	12.16	0	no
N13	Obstructive and reflux uropathy	N00.N99	United Kingdom	277.19	1	yes
N14	Drug- and heavy-metal-induced tubulo-interstitial and tubular conditions	N00.N99	Belgium	8.48	1	yes
N14	Drug- and heavy-metal-induced tubulo-interstitial and tubular conditions	N00.N99	Denmark	9.97	1	yes
N14	Drug- and heavy-metal-induced tubulo-interstitial and tubular conditions	N00.N99	France	20.03	0	no
N14	Drug- and heavy-metal-induced tubulo-interstitial and tubular conditions	N00.N99	Germany	9.56	0	no
N14	Drug- and heavy-metal-induced tubulo-interstitial and tubular conditions	N00.N99	Switzerland	17.70	3	yes
N14	Drug- and heavy-metal-induced tubulo-interstitial and tubular conditions	N00.N99	United Kingdom	5.39	0	no
N15	Other renal tubulo-interstitial diseases	N00.N99	Belgium	12.64	0	no
N15	Other renal tubulo-interstitial diseases	N00.N99	Czech Republic	16.00	0	no
N15	Other renal tubulo-interstitial diseases	N00.N99	Denmark	6.40	0	no
N15	Other renal tubulo-interstitial diseases	N00.N99	France	25.36	0	no
N15	Other renal tubulo-interstitial diseases	N00.N99	Germany	29.28	0	no

For peer review only

N15	Other renal tubulo-interstitial diseases	N00.N99	Hungary	13.78	1	yes
N15	Other renal tubulo-interstitial diseases	N00.N99	Netherlands	5.85	1	yes
N15	Other renal tubulo-interstitial diseases	N00.N99	Poland	125.96	0	no
N15	Other renal tubulo-interstitial diseases	N00.N99	Romania	11.06	0	no
N15	Other renal tubulo-interstitial diseases	N00.N99	Spain	36.79	1	yes
N15	Other renal tubulo-interstitial diseases	N00.N99	United Kingdom	45.97	2	yes
N17	Acute renal failure	N00.N99	Belgium	231.69	0	no
N17	Acute renal failure	N00.N99	Croatia	51.13	3	yes
N17	Acute renal failure	N00.N99	Czech Republic	342.11	0	no
N17	Acute renal failure	N00.N99	Denmark	30.95	3	yes
N17	Acute renal failure	N00.N99	Finland	12.44	0	no
N17	Acute renal failure	N00.N99	France	890.44	2	yes
N17	Acute renal failure	N00.N99	Germany	2177.88	1	yes
N17	Acute renal failure	N00.N99	Hungary	38.84	2	yes
N17	Acute renal failure	N00.N99	Lithuania	9.64	0	no
N17	Acute renal failure	N00.N99	Netherlands	207.21	3	yes
N17	Acute renal failure	N00.N99	Norway	32.60	0	no
N17	Acute renal failure	N00.N99	Poland	826.77	3	yes
N17	Acute renal failure	N00.N99	Romania	519.41	2	yes
N17	Acute renal failure	N00.N99	Slovenia	35.00	0	no
N17	Acute renal failure	N00.N99	Spain	915.66	1	yes
N17	Acute renal failure	N00.N99	Sweden	78.49	2	yes
N17	Acute renal failure	N00.N99	Switzerland	27.07	3	yes
N17	Acute renal failure	N00.N99	United Kingdom	842.07	2	yes
N18	Chronic renal failure	N00.N99	Austria	693.72	2	yes
N18	Chronic renal failure	N00.N99	Belgium	583.16	1	yes
N18	Chronic renal failure	N00.N99	Croatia	408.60	3	yes
N18	Chronic renal failure	N00.N99	Czech Republic	828.89	1	yes
N18	Chronic renal failure	N00.N99	Denmark	264.02	1	yes
N18	Chronic renal failure	N00.N99	Estonia	21.80	1	yes
N18	Chronic renal failure	N00.N99	Finland	112.99	2	yes
N18	Chronic renal failure	N00.N99	France	2571.52	2	yes
N18	Chronic renal failure	N00.N99	Germany	7012.93	1	yes
N18	Chronic renal failure	N00.N99	Hungary	287.74	1	yes
N18	Chronic renal failure	N00.N99	Latvia	33.61	3	yes
N18	Chronic renal failure	N00.N99	Lithuania	84.86	3	yes
N18	Chronic renal failure	N00.N99	Netherlands	1214.29	1	yes
N18	Chronic renal failure	N00.N99	Norway	112.89	2	yes
N18	Chronic renal failure	N00.N99	Poland	3350.13	3	yes
N18	Chronic renal failure	N00.N99	Romania	1877.25	2	yes
N18	Chronic renal failure	N00.N99	Slovenia	158.91	0	no
N18	Chronic renal failure	N00.N99	Spain	4043.38	3	yes
N18	Chronic renal failure	N00.N99	Sweden	234.10	0	no
N18	Chronic renal failure	N00.N99	Switzerland	215.64	3	yes
N18	Chronic renal failure	N00.N99	United Kingdom	2035.36	3	yes
N19	Unspecified renal failure	N00.N99	Austria	351.07	1	yes
N19	Unspecified renal failure	N00.N99	Belgium	647.59	0	no
N19	Unspecified renal failure	N00.N99	Croatia	71.53	3	yes
N19	Unspecified renal failure	N00.N99	Czech Republic	50.84	1	yes
N19	Unspecified renal failure	N00.N99	Denmark	146.45	3	yes

For peer review only

N19	Unspecified renal failure	N00.N99	Finland	13.62	0	no
N19	Unspecified renal failure	N00.N99	France	2657.44	2	yes
N19	Unspecified renal failure	N00.N99	Germany	3958.07	2	yes
N19	Unspecified renal failure	N00.N99	Hungary	73.15	1	yes
N19	Unspecified renal failure	N00.N99	Lithuania	8.06	1	yes
N19	Unspecified renal failure	N00.N99	Netherlands	516.53	3	yes
N19	Unspecified renal failure	N00.N99	Norway	326.64	2	yes
N19	Unspecified renal failure	N00.N99	Poland	913.57	3	yes
N19	Unspecified renal failure	N00.N99	Romania	279.05	1	yes
N19	Unspecified renal failure	N00.N99	Slovenia	21.93	2	yes
N19	Unspecified renal failure	N00.N99	Spain	2177.77	1	yes
N19	Unspecified renal failure	N00.N99	Sweden	281.08	0	no
N19	Unspecified renal failure	N00.N99	Switzerland	153.73	3	yes
N19	Unspecified renal failure	N00.N99	United Kingdom	1051.17	3	yes
N20	Calculus of kidney and ureter	N00.N99	Austria	18.84	0	no
N20	Calculus of kidney and ureter	N00.N99	Belgium	18.83	0	no
N20	Calculus of kidney and ureter	N00.N99	Croatia	7.21	1	yes
N20	Calculus of kidney and ureter	N00.N99	Czech Republic	26.97	0	no
N20	Calculus of kidney and ureter	N00.N99	Denmark	18.70	1	yes
N20	Calculus of kidney and ureter	N00.N99	Estonia	8.58	2	yes
N20	Calculus of kidney and ureter	N00.N99	Finland	6.16	0	no
N20	Calculus of kidney and ureter	N00.N99	France	70.88	0	no
N20	Calculus of kidney and ureter	N00.N99	Germany	84.78	2	yes
N20	Calculus of kidney and ureter	N00.N99	Hungary	72.83	0	no
N20	Calculus of kidney and ureter	N00.N99	Latvia	26.09	2	yes
N20	Calculus of kidney and ureter	N00.N99	Lithuania	30.93	1	yes
N20	Calculus of kidney and ureter	N00.N99	Netherlands	40.65	0	no
N20	Calculus of kidney and ureter	N00.N99	Norway	11.05	0	no
N20	Calculus of kidney and ureter	N00.N99	Poland	32.29	2	yes
N20	Calculus of kidney and ureter	N00.N99	Spain	50.43	0	no
N20	Calculus of kidney and ureter	N00.N99	Sweden	19.25	0	no
N20	Calculus of kidney and ureter	N00.N99	Switzerland	8.43	0	no
N20	Calculus of kidney and ureter	N00.N99	United Kingdom	155.45	1	yes
N21	Calculus of lower urinary tract	N00.N99	Hungary	5.65	0	no
N21	Calculus of lower urinary tract	N00.N99	Netherlands	5.93	0	no
N21	Calculus of lower urinary tract	N00.N99	United Kingdom	14.03	0	no
N23	Unspecified renal colic	N00.N99	France	5.66	0	no
N25	Disorders resulting from impaired renal tubular function	N00.N99	United Kingdom	6.02	0	no
N26	Unspecified contracted kidney	N00.N99	Austria	36.63	1	yes
N26	Unspecified contracted kidney	N00.N99	Czech Republic	5.39	1	yes
N26	Unspecified contracted kidney	N00.N99	Denmark	6.84	3	yes
N26	Unspecified contracted kidney	N00.N99	Finland	5.58	0	no
N26	Unspecified contracted kidney	N00.N99	Germany	132.87	1	yes
N26	Unspecified contracted kidney	N00.N99	Hungary	46.88	1	yes
N26	Unspecified contracted kidney	N00.N99	Latvia	51.61	1	yes
N26	Unspecified contracted kidney	N00.N99	Lithuania	6.43	2	yes
N26	Unspecified contracted kidney	N00.N99	Netherlands	24.86	1	yes
N26	Unspecified contracted kidney	N00.N99	Romania	6.92	3	yes
N26	Unspecified contracted kidney	N00.N99	Spain	11.30	1	yes

For peer review only

N26	Unspecified contracted kidney	N00.N99	Switzerland	6.20	1	yes
N26	Unspecified contracted kidney	N00.N99	United Kingdom	19.63	0	no
N27	Small kidney of unknown cause	N00.N99	United Kingdom	10.42	1	yes
N28	Other disorders of kidney and ureter, not elsewhere classified	N00.N99	Austria	22.47	1	yes
N28	Other disorders of kidney and ureter, not elsewhere classified	N00.N99	Belgium	35.21	0	no
N28	Other disorders of kidney and ureter, not elsewhere classified	N00.N99	Croatia	5.31	0	no
N28	Other disorders of kidney and ureter, not elsewhere classified	N00.N99	Czech Republic	14.90	1	yes
N28	Other disorders of kidney and ureter, not elsewhere classified	N00.N99	Denmark	9.30	0	no
N28	Other disorders of kidney and ureter, not elsewhere classified	N00.N99	France	77.86	2	yes
N28	Other disorders of kidney and ureter, not elsewhere classified	N00.N99	Germany	162.37	3	yes
N28	Other disorders of kidney and ureter, not elsewhere classified	N00.N99	Hungary	33.34	2	yes
N28	Other disorders of kidney and ureter, not elsewhere classified	N00.N99	Latvia	13.27	0	no
N28	Other disorders of kidney and ureter, not elsewhere classified	N00.N99	Lithuania	12.47	0	no
N28	Other disorders of kidney and ureter, not elsewhere classified	N00.N99	Netherlands	112.12	0	no
N28	Other disorders of kidney and ureter, not elsewhere classified	N00.N99	Norway	15.50	0	no
N28	Other disorders of kidney and ureter, not elsewhere classified	N00.N99	Poland	24.95	0	no
N28	Other disorders of kidney and ureter, not elsewhere classified	N00.N99	Romania	11.46	0	no
N28	Other disorders of kidney and ureter, not elsewhere classified	N00.N99	Spain	136.35	0	no
N28	Other disorders of kidney and ureter, not elsewhere classified	N00.N99	Sweden	10.70	0	no
N28	Other disorders of kidney and ureter, not elsewhere classified	N00.N99	Switzerland	10.39	0	no
N28	Other disorders of kidney and ureter, not elsewhere classified	N00.N99	United Kingdom	257.02	0	no
N30	Cystitis	N00.N99	Austria	13.79	0	no
N30	Cystitis	N00.N99	Belgium	9.18	1	yes
N30	Cystitis	N00.N99	Croatia	5.59	1	yes
N30	Cystitis	N00.N99	Czech Republic	56.96	1	yes
N30	Cystitis	N00.N99	Denmark	80.84	0	no
N30	Cystitis	N00.N99	Finland	9.49	0	no
N30	Cystitis	N00.N99	France	18.06	0	no
N30	Cystitis	N00.N99	Germany	104.10	1	yes
N30	Cystitis	N00.N99	Hungary	43.70	1	yes
N30	Cystitis	N00.N99	Netherlands	73.93	1	yes
N30	Cystitis	N00.N99	Norway	5.17	0	no
N30	Cystitis	N00.N99	Poland	17.20	2	yes
N30	Cystitis	N00.N99	Slovenia	7.62	1	yes
N30	Cystitis	N00.N99	Spain	11.36	0	no
N30	Cystitis	N00.N99	Sweden	5.52	2	yes
N30	Cystitis	N00.N99	Switzerland	7.39	0	no
N30	Cystitis	N00.N99	United Kingdom	49.13	1	yes

For peer review only

N31	Neuromuscular dysfunction of bladder, not elsewhere classified	N00.N99	Germany	8.46	3	yes
N31	Neuromuscular dysfunction of bladder, not elsewhere classified	N00.N99	Netherlands	6.07	0	no
N32	Other disorders of bladder	N00.N99	Belgium	17.23	0	no
N32	Other disorders of bladder	N00.N99	Denmark	7.42	1	yes
N32	Other disorders of bladder	N00.N99	France	109.06	0	no
N32	Other disorders of bladder	N00.N99	Germany	50.47	1	yes
N32	Other disorders of bladder	N00.N99	Hungary	7.10	0	no
N32	Other disorders of bladder	N00.N99	Netherlands	41.48	0	no
N32	Other disorders of bladder	N00.N99	Poland	9.45	1	yes
N32	Other disorders of bladder	N00.N99	Spain	42.05	0	no
N32	Other disorders of bladder	N00.N99	Sweden	9.62	0	no
N32	Other disorders of bladder	N00.N99	Switzerland	8.58	1	yes
N32	Other disorders of bladder	N00.N99	United Kingdom	121.79	2	yes
N34	Urethritis and urethral syndrome	N00.N99	Czech Republic	5.10	0	no
N35	Urethral stricture	N00.N99	Germany	7.13	0	no
N35	Urethral stricture	N00.N99	United Kingdom	7.77	0	no
N36	Other disorders of urethra	N00.N99	France	5.81	0	no
N39	Other disorders of urinary system	N00.N99	Austria	150.43	2	yes
N39	Other disorders of urinary system	N00.N99	Belgium	459.76	3	yes
N39	Other disorders of urinary system	N00.N99	Croatia	237.29	1	yes
N39	Other disorders of urinary system	N00.N99	Czech Republic	169.06	3	yes
N39	Other disorders of urinary system	N00.N99	Denmark	253.27	3	yes
N39	Other disorders of urinary system	N00.N99	Finland	63.01	1	yes
N39	Other disorders of urinary system	N00.N99	France	1446.52	1	yes
N39	Other disorders of urinary system	N00.N99	Germany	3589.75	3	yes
N39	Other disorders of urinary system	N00.N99	Hungary	43.38	2	yes
N39	Other disorders of urinary system	N00.N99	Latvia	14.74	2	yes
N39	Other disorders of urinary system	N00.N99	Lithuania	12.00	3	yes
N39	Other disorders of urinary system	N00.N99	Netherlands	1686.04	2	yes
N39	Other disorders of urinary system	N00.N99	Norway	314.94	2	yes
N39	Other disorders of urinary system	N00.N99	Poland	333.81	3	yes
N39	Other disorders of urinary system	N00.N99	Romania	29.66	2	yes
N39	Other disorders of urinary system	N00.N99	Slovenia	79.15	1	yes
N39	Other disorders of urinary system	N00.N99	Spain	3266.88	1	yes
N39	Other disorders of urinary system	N00.N99	Sweden	338.09	2	yes
N39	Other disorders of urinary system	N00.N99	Switzerland	245.82	2	yes
N39	Other disorders of urinary system	N00.N99	United Kingdom	6824.06	2	yes
N40	Hyperplasia of prostate	N00.N99	Austria	9.44	0	no
N40	Hyperplasia of prostate	N00.N99	Belgium	21.66	1	yes
N40	Hyperplasia of prostate	N00.N99	Croatia	30.86	3	yes
N40	Hyperplasia of prostate	N00.N99	Czech Republic	36.12	0	no
N40	Hyperplasia of prostate	N00.N99	Denmark	49.25	1	yes
N40	Hyperplasia of prostate	N00.N99	Estonia	11.55	0	no
N40	Hyperplasia of prostate	N00.N99	Finland	26.07	1	yes
N40	Hyperplasia of prostate	N00.N99	France	153.65	1	yes
N40	Hyperplasia of prostate	N00.N99	Germany	125.07	2	yes
N40	Hyperplasia of prostate	N00.N99	Hungary	105.51	0	no
N40	Hyperplasia of prostate	N00.N99	Latvia	41.47	0	no
N40	Hyperplasia of prostate	N00.N99	Lithuania	26.93	2	yes

For peer review only

N40	Hyperplasia of prostate	N00.N99	Netherlands	74.52	2	yes
N40	Hyperplasia of prostate	N00.N99	Norway	32.69	0	no
N40	Hyperplasia of prostate	N00.N99	Poland	53.39	2	yes
N40	Hyperplasia of prostate	N00.N99	Romania	307.31	3	yes
N40	Hyperplasia of prostate	N00.N99	Spain	156.19	0	no
N40	Hyperplasia of prostate	N00.N99	Sweden	41.39	1	yes
N40	Hyperplasia of prostate	N00.N99	Switzerland	40.51	2	yes
N40	Hyperplasia of prostate	N00.N99	United Kingdom	203.83	1	yes
N41	Inflammatory diseases of prostate	N00.N99	Belgium	6.74	0	no
N41	Inflammatory diseases of prostate	N00.N99	France	107.43	2	yes
N41	Inflammatory diseases of prostate	N00.N99	Germany	7.13	0	no
N41	Inflammatory diseases of prostate	N00.N99	Hungary	8.19	0	no
N41	Inflammatory diseases of prostate	N00.N99	Netherlands	6.93	1	yes
N41	Inflammatory diseases of prostate	N00.N99	Spain	9.33	1	yes
N41	Inflammatory diseases of prostate	N00.N99	United Kingdom	5.63	0	no
N42	Other disorders of prostate	N00.N99	Belgium	20.15	1	yes
N42	Other disorders of prostate	N00.N99	France	104.32	2	yes
N42	Other disorders of prostate	N00.N99	Germany	9.22	1	yes
N42	Other disorders of prostate	N00.N99	Netherlands	13.67	0	no
N42	Other disorders of prostate	N00.N99	Spain	27.61	1	yes
N42	Other disorders of prostate	N00.N99	Sweden	6.02	0	no
N42	Other disorders of prostate	N00.N99	United Kingdom	15.20	1	yes
N45	Orchitis and epididymitis	N00.N99	France	7.86	0	no
N45	Orchitis and epididymitis	N00.N99	Germany	9.96	0	no
N45	Orchitis and epididymitis	N00.N99	United Kingdom	6.46	0	no
N49	Inflammatory disorders of male genital organs, not elsewhere classified	N00.N99	Belgium	8.59	0	no
N49	Inflammatory disorders of male genital organs, not elsewhere classified	N00.N99	France	27.86	2	yes
N49	Inflammatory disorders of male genital organs, not elsewhere classified	N00.N99	Germany	47.83	1	yes
N49	Inflammatory disorders of male genital organs, not elsewhere classified	N00.N99	Hungary	6.99	1	yes
N49	Inflammatory disorders of male genital organs, not elsewhere classified	N00.N99	Poland	5.49	0	no
N49	Inflammatory disorders of male genital organs, not elsewhere classified	N00.N99	Spain	54.79	0	no
N49	Inflammatory disorders of male genital organs, not elsewhere classified	N00.N99	United Kingdom	39.05	1	yes
N70	Salpingitis and oophoritis	N00.N99	Germany	7.91	1	yes
N70	Salpingitis and oophoritis	N00.N99	Hungary	6.32	0	no
N70	Salpingitis and oophoritis	N00.N99	United Kingdom	6.36	0	no
N71	Inflammatory disease of uterus, except cervix	N00.N99	France	6.94	2	yes
N71	Inflammatory disease of uterus, except cervix	N00.N99	Germany	5.43	0	no
N71	Inflammatory disease of uterus, except cervix	N00.N99	Hungary	5.02	0	no
N71	Inflammatory disease of uterus, except cervix	N00.N99	United Kingdom	19.79	1	yes
N73	Other female pelvic inflammatory diseases	N00.N99	France	17.60	1	yes
N73	Other female pelvic inflammatory diseases	N00.N99	Spain	6.61	1	yes
N73	Other female pelvic inflammatory diseases	N00.N99	United Kingdom	37.66	1	yes
N76	Other inflammation of vagina and vulva	N00.N99	Germany	17.41	1	yes
N76	Other inflammation of vagina and vulva	N00.N99	Spain	17.58	3	yes
N81	Female genital prolapse	N00.N99	France	11.86	2	yes

For peer review only

N81	Female genital prolapse	N00.N99	Germany	6.23	0	no
N81	Female genital prolapse	N00.N99	Netherlands	5.60	0	no
N81	Female genital prolapse	N00.N99	United Kingdom	8.56	2	yes
N82	Fistulae involving female genital tract	N00.N99	France	23.85	1	yes
N82	Fistulae involving female genital tract	N00.N99	Germany	13.28	1	yes
N82	Fistulae involving female genital tract	N00.N99	Netherlands	5.73	1	yes
N82	Fistulae involving female genital tract	N00.N99	Spain	10.07	0	no
N82	Fistulae involving female genital tract	N00.N99	United Kingdom	30.16	0	no
N83	Noninflammatory disorders of ovary, fallopian tube and broad ligament	N00.N99	France	12.35	0	no
N83	Noninflammatory disorders of ovary, fallopian tube and broad ligament	N00.N99	United Kingdom	26.21	0	no
N85	Other noninflammatory disorders of uterus, except cervix	N00.N99	Spain	5.27	0	no
N85	Other noninflammatory disorders of uterus, except cervix	N00.N99	United Kingdom	18.15	2	yes
N93	Other abnormal uterine and vaginal bleeding	N00.N99	Germany	5.27	0	no
N93	Other abnormal uterine and vaginal bleeding	N00.N99	Netherlands	15.07	0	no
N95	Menopausal and other perimenopausal disorders	N00.N99	Netherlands	6.01	1	yes
N95	Menopausal and other perimenopausal disorders	N00.N99	Spain	6.28	0	no
O06	Unspecified abortion	O00.O99	Romania	5.68	2	yes
O72	Postpartum haemorrhage	O00.O99	France	6.23	0	no
O88	Obstetric embolism	O00.O99	France	9.85	0	no
O88	Obstetric embolism	O00.O99	Germany	6.42	0	no
O99	Other maternal diseases classifiable elsewhere but complicating pregnancy, childbirth and the puerperium	O00.O99	France	6.56	3	yes
O99	Other maternal diseases classifiable elsewhere but complicating pregnancy, childbirth and the puerperium	O00.O99	United Kingdom	12.36	0	no
P00	Fetus and newborn affected by maternal conditions that may be unrelated to present pregnancy	P00.P96	Belgium	12.53	1	yes
P00	Fetus and newborn affected by maternal conditions that may be unrelated to present pregnancy	P00.P96	France	31.79	0	no
P00	Fetus and newborn affected by maternal conditions that may be unrelated to present pregnancy	P00.P96	Germany	12.09	1	yes
P00	Fetus and newborn affected by maternal conditions that may be unrelated to present pregnancy	P00.P96	Netherlands	5.19	0	no
P00	Fetus and newborn affected by maternal conditions that may be unrelated to present pregnancy	P00.P96	Norway	5.50	1	yes
P01	Fetus and newborn affected by maternal complications of pregnancy	P00.P96	Belgium	17.52	0	no
P01	Fetus and newborn affected by maternal complications of pregnancy	P00.P96	Croatia	13.85	1	yes
P01	Fetus and newborn affected by maternal complications of pregnancy	P00.P96	Denmark	7.44	0	no
P01	Fetus and newborn affected by maternal complications of pregnancy	P00.P96	France	78.58	0	no
P01	Fetus and newborn affected by maternal complications of pregnancy	P00.P96	Germany	97.59	2	yes
P01	Fetus and newborn affected by maternal complications of pregnancy	P00.P96	Netherlands	26.95	2	yes
P01	Fetus and newborn affected by maternal complications of pregnancy	P00.P96	Norway	11.34	0	no
P01	Fetus and newborn affected by maternal complications of pregnancy	P00.P96	Poland	10.76	1	yes

For peer review only

P01	Fetus and newborn affected by maternal complications of pregnancy	P00.P96	Spain	56.26	1	yes
P01	Fetus and newborn affected by maternal complications of pregnancy	P00.P96	Sweden	6.15	3	yes
P01	Fetus and newborn affected by maternal complications of pregnancy	P00.P96	Switzerland	19.07	1	yes
P01	Fetus and newborn affected by maternal complications of pregnancy	P00.P96	United Kingdom	20.46	0	no
P02	Fetus and newborn affected by complications of placenta, cord and membranes	P00.P96	Austria	17.59	2	yes
P02	Fetus and newborn affected by complications of placenta, cord and membranes	P00.P96	Belgium	35.23	2	yes
P02	Fetus and newborn affected by complications of placenta, cord and membranes	P00.P96	Croatia	19.81	2	yes
P02	Fetus and newborn affected by complications of placenta, cord and membranes	P00.P96	Denmark	12.03	0	no
P02	Fetus and newborn affected by complications of placenta, cord and membranes	P00.P96	France	133.26	1	yes
P02	Fetus and newborn affected by complications of placenta, cord and membranes	P00.P96	Germany	131.39	2	yes
P02	Fetus and newborn affected by complications of placenta, cord and membranes	P00.P96	Netherlands	25.37	0	no
P02	Fetus and newborn affected by complications of placenta, cord and membranes	P00.P96	Norway	18.74	2	yes
P02	Fetus and newborn affected by complications of placenta, cord and membranes	P00.P96	Poland	17.06	0	no
P02	Fetus and newborn affected by complications of placenta, cord and membranes	P00.P96	Romania	5.11	0	no
P02	Fetus and newborn affected by complications of placenta, cord and membranes	P00.P96	Spain	31.86	0	no
P02	Fetus and newborn affected by complications of placenta, cord and membranes	P00.P96	Sweden	12.37	1	yes
P02	Fetus and newborn affected by complications of placenta, cord and membranes	P00.P96	Switzerland	27.24	1	yes
P02	Fetus and newborn affected by complications of placenta, cord and membranes	P00.P96	United Kingdom	26.61	0	no
P03	Fetus and newborn affected by other complications of labour and delivery	P00.P96	France	21.28	0	no
P03	Fetus and newborn affected by other complications of labour and delivery	P00.P96	Germany	15.76	1	yes
P03	Fetus and newborn affected by other complications of labour and delivery	P00.P96	Netherlands	5.18	0	no
P05	Slow fetal growth and fetal malnutrition	P00.P96	France	29.90	3	yes
P05	Slow fetal growth and fetal malnutrition	P00.P96	Germany	5.44	1	yes
P05	Slow fetal growth and fetal malnutrition	P00.P96	Spain	7.59	0	no
P05	Slow fetal growth and fetal malnutrition	P00.P96	United Kingdom	9.24	0	no
P07	Disorders related to short gestation and low birth weight, not elsewhere classified	P00.P96	Austria	89.65	3	yes
P07	Disorders related to short gestation and low birth weight, not elsewhere classified	P00.P96	Belgium	15.54	0	no
P07	Disorders related to short gestation and low birth weight, not elsewhere classified	P00.P96	Croatia	8.81	0	no
P07	Disorders related to short gestation and low birth weight, not elsewhere classified	P00.P96	Czech Republic	11.87	1	yes
P07	Disorders related to short gestation and low birth weight, not elsewhere classified	P00.P96	Denmark	66.78	0	no
P07	Disorders related to short gestation and low birth weight, not elsewhere classified	P00.P96	Finland	9.53	1	yes
P07	Disorders related to short gestation and low birth weight, not elsewhere classified	P00.P96	France	96.24	2	yes

For peer review only

P07	Disorders related to short gestation and low birth weight, not elsewhere classified	P00.P96	Germany	701.08	3	yes
P07	Disorders related to short gestation and low birth weight, not elsewhere classified	P00.P96	Hungary	87.86	2	yes
P07	Disorders related to short gestation and low birth weight, not elsewhere classified	P00.P96	Lithuania	8.64	1	yes
P07	Disorders related to short gestation and low birth weight, not elsewhere classified	P00.P96	Netherlands	70.17	2	yes
P07	Disorders related to short gestation and low birth weight, not elsewhere classified	P00.P96	Norway	5.32	2	yes
P07	Disorders related to short gestation and low birth weight, not elsewhere classified	P00.P96	Poland	632.69	2	yes
P07	Disorders related to short gestation and low birth weight, not elsewhere classified	P00.P96	Slovenia	9.56	0	no
P07	Disorders related to short gestation and low birth weight, not elsewhere classified	P00.P96	Spain	85.24	1	yes
P07	Disorders related to short gestation and low birth weight, not elsewhere classified	P00.P96	Sweden	7.63	0	no
P07	Disorders related to short gestation and low birth weight, not elsewhere classified	P00.P96	Switzerland	28.79	0	no
P07	Disorders related to short gestation and low birth weight, not elsewhere classified	P00.P96	United Kingdom	807.11	3	yes
P10	Intracranial laceration and haemorrhage due to birth injury	P00.P96	Poland	10.31	2	yes
P10	Intracranial laceration and haemorrhage due to birth injury	P00.P96	Romania	15.32	3	yes
P20	Intrauterine hypoxia	P00.P96	Czech Republic	5.80	0	no
P20	Intrauterine hypoxia	P00.P96	France	55.60	2	yes
P20	Intrauterine hypoxia	P00.P96	Germany	20.26	1	yes
P20	Intrauterine hypoxia	P00.P96	Hungary	11.74	1	yes
P20	Intrauterine hypoxia	P00.P96	Netherlands	7.14	2	yes
P20	Intrauterine hypoxia	P00.P96	Poland	33.57	1	yes
P20	Intrauterine hypoxia	P00.P96	Spain	28.72	2	yes
P20	Intrauterine hypoxia	P00.P96	United Kingdom	11.86	1	yes
P21	Birth asphyxia	P00.P96	Austria	9.30	2	yes
P21	Birth asphyxia	P00.P96	Belgium	10.70	0	no
P21	Birth asphyxia	P00.P96	Croatia	5.78	1	yes
P21	Birth asphyxia	P00.P96	Czech Republic	20.81	3	yes
P21	Birth asphyxia	P00.P96	Denmark	7.33	1	yes
P21	Birth asphyxia	P00.P96	France	44.43	1	yes
P21	Birth asphyxia	P00.P96	Germany	83.56	0	no
P21	Birth asphyxia	P00.P96	Hungary	13.78	0	no
P21	Birth asphyxia	P00.P96	Latvia	13.49	2	yes
P21	Birth asphyxia	P00.P96	Lithuania	12.63	1	yes
P21	Birth asphyxia	P00.P96	Netherlands	40.91	1	yes
P21	Birth asphyxia	P00.P96	Norway	6.39	0	no
P21	Birth asphyxia	P00.P96	Poland	81.95	3	yes
P21	Birth asphyxia	P00.P96	Romania	43.00	2	yes
P21	Birth asphyxia	P00.P96	Spain	29.15	2	yes
P21	Birth asphyxia	P00.P96	Sweden	13.91	2	yes
P21	Birth asphyxia	P00.P96	Switzerland	11.26	0	no
P21	Birth asphyxia	P00.P96	United Kingdom	112.75	2	yes
P22	Respiratory distress of newborn	P00.P96	Belgium	14.73	1	yes
P22	Respiratory distress of newborn	P00.P96	Croatia	25.10	1	yes
P22	Respiratory distress of newborn	P00.P96	Czech Republic	20.25	2	yes

For peer review only

P22	Respiratory distress of newborn	P00.P96	Estonia	6.39	0	no
P22	Respiratory distress of newborn	P00.P96	Finland	7.04	1	yes
P22	Respiratory distress of newborn	P00.P96	France	46.31	2	yes
P22	Respiratory distress of newborn	P00.P96	Germany	52.37	2	yes
P22	Respiratory distress of newborn	P00.P96	Hungary	43.58	0	no
P22	Respiratory distress of newborn	P00.P96	Latvia	5.49	0	no
P22	Respiratory distress of newborn	P00.P96	Lithuania	13.39	3	yes
P22	Respiratory distress of newborn	P00.P96	Netherlands	6.27	0	no
P22	Respiratory distress of newborn	P00.P96	Poland	52.36	3	yes
P22	Respiratory distress of newborn	P00.P96	Romania	118.96	0	no
P22	Respiratory distress of newborn	P00.P96	Spain	66.50	2	yes
P22	Respiratory distress of newborn	P00.P96	Sweden	5.49	1	yes
P22	Respiratory distress of newborn	P00.P96	Switzerland	11.78	0	no
P22	Respiratory distress of newborn	P00.P96	United Kingdom	52.67	2	yes
P23	Congenital pneumonia	P00.P96	Hungary	10.82	2	yes
P23	Congenital pneumonia	P00.P96	Latvia	5.29	0	no
P23	Congenital pneumonia	P00.P96	Poland	16.05	1	yes
P23	Congenital pneumonia	P00.P96	Romania	86.26	1	yes
P23	Congenital pneumonia	P00.P96	Spain	6.99	0	no
P23	Congenital pneumonia	P00.P96	United Kingdom	30.51	1	yes
P24	Neonatal aspiration syndromes	P00.P96	France	9.58	0	no
P24	Neonatal aspiration syndromes	P00.P96	Germany	17.28	0	no
P24	Neonatal aspiration syndromes	P00.P96	Poland	9.64	1	yes
P24	Neonatal aspiration syndromes	P00.P96	Romania	33.04	1	yes
P24	Neonatal aspiration syndromes	P00.P96	Spain	12.31	1	yes
P24	Neonatal aspiration syndromes	P00.P96	United Kingdom	10.00	1	yes
P25	Interstitial emphysema and related conditions originating in the perinatal period	P00.P96	France	9.24	1	yes
P25	Interstitial emphysema and related conditions originating in the perinatal period	P00.P96	Germany	10.92	0	no
P25	Interstitial emphysema and related conditions originating in the perinatal period	P00.P96	Spain	14.88	0	no
P25	Interstitial emphysema and related conditions originating in the perinatal period	P00.P96	United Kingdom	17.03	0	no
P26	Pulmonary haemorrhage originating in the perinatal period	P00.P96	France	19.44	2	yes
P26	Pulmonary haemorrhage originating in the perinatal period	P00.P96	Germany	7.66	1	yes
P26	Pulmonary haemorrhage originating in the perinatal period	P00.P96	Hungary	9.69	0	no
P26	Pulmonary haemorrhage originating in the perinatal period	P00.P96	Romania	39.03	0	no
P26	Pulmonary haemorrhage originating in the perinatal period	P00.P96	Spain	21.11	1	yes
P26	Pulmonary haemorrhage originating in the perinatal period	P00.P96	United Kingdom	6.65	2	yes
P27	Chronic respiratory disease originating in the perinatal period	P00.P96	France	27.17	1	yes
P27	Chronic respiratory disease originating in the perinatal period	P00.P96	Germany	26.80	0	no
P27	Chronic respiratory disease originating in the perinatal period	P00.P96	Hungary	7.18	1	yes
P27	Chronic respiratory disease originating in the perinatal period	P00.P96	Netherlands	7.85	0	no

For peer review only

P27	Chronic respiratory disease originating in the perinatal period	P00.P96	Poland	10.39	1	yes
P27	Chronic respiratory disease originating in the perinatal period	P00.P96	Spain	21.57	0	no
P27	Chronic respiratory disease originating in the perinatal period	P00.P96	United Kingdom	60.52	2	yes
P28	Other respiratory conditions originating in the perinatal period	P00.P96	Belgium	8.80	0	no
P28	Other respiratory conditions originating in the perinatal period	P00.P96	Czech Republic	8.28	1	yes
P28	Other respiratory conditions originating in the perinatal period	P00.P96	France	34.08	3	yes
P28	Other respiratory conditions originating in the perinatal period	P00.P96	Germany	41.40	1	yes
P28	Other respiratory conditions originating in the perinatal period	P00.P96	Hungary	32.53	2	yes
P28	Other respiratory conditions originating in the perinatal period	P00.P96	Netherlands	11.02	0	no
P28	Other respiratory conditions originating in the perinatal period	P00.P96	Poland	17.89	1	yes
P28	Other respiratory conditions originating in the perinatal period	P00.P96	Romania	175.40	0	no
P28	Other respiratory conditions originating in the perinatal period	P00.P96	Spain	30.71	1	yes
P28	Other respiratory conditions originating in the perinatal period	P00.P96	Sweden	5.95	0	no
P28	Other respiratory conditions originating in the perinatal period	P00.P96	Switzerland	11.81	2	yes
P28	Other respiratory conditions originating in the perinatal period	P00.P96	United Kingdom	86.63	2	yes
P29	Cardiovascular disorders originating in the perinatal period	P00.P96	Czech Republic	6.00	0	no
P29	Cardiovascular disorders originating in the perinatal period	P00.P96	France	67.90	3	yes
P29	Cardiovascular disorders originating in the perinatal period	P00.P96	Germany	32.50	1	yes
P29	Cardiovascular disorders originating in the perinatal period	P00.P96	Hungary	6.32	0	no
P29	Cardiovascular disorders originating in the perinatal period	P00.P96	Poland	10.47	1	yes
P29	Cardiovascular disorders originating in the perinatal period	P00.P96	Spain	35.79	2	yes
P29	Cardiovascular disorders originating in the perinatal period	P00.P96	Sweden	6.62	2	yes
P29	Cardiovascular disorders originating in the perinatal period	P00.P96	United Kingdom	11.21	1	yes
P35	Congenital viral diseases	P00.P96	Germany	7.49	0	no
P35	Congenital viral diseases	P00.P96	United Kingdom	11.81	0	no
P36	Bacterial sepsis of newborn	P00.P96	Belgium	10.76	3	yes
P36	Bacterial sepsis of newborn	P00.P96	Croatia	11.44	0	no
P36	Bacterial sepsis of newborn	P00.P96	Czech Republic	14.49	2	yes
P36	Bacterial sepsis of newborn	P00.P96	France	37.20	2	yes
P36	Bacterial sepsis of newborn	P00.P96	Germany	59.22	1	yes
P36	Bacterial sepsis of newborn	P00.P96	Hungary	9.13	3	yes
P36	Bacterial sepsis of newborn	P00.P96	Lithuania	7.49	2	yes
P36	Bacterial sepsis of newborn	P00.P96	Netherlands	27.43	0	no
P36	Bacterial sepsis of newborn	P00.P96	Poland	75.45	2	yes
P36	Bacterial sepsis of newborn	P00.P96	Romania	14.56	0	no

For peer review only

P36	Bacterial sepsis of newborn	P00.P96	Spain	97.96	1	yes
P36	Bacterial sepsis of newborn	P00.P96	Sweden	6.58	2	yes
P36	Bacterial sepsis of newborn	P00.P96	Switzerland	8.42	0	no
P36	Bacterial sepsis of newborn	P00.P96	United Kingdom	41.03	1	yes
P37	Other congenital infectious and parasitic diseases	P00.P96	France	7.18	0	no
P37	Other congenital infectious and parasitic diseases	P00.P96	Spain	6.74	0	no
P37	Other congenital infectious and parasitic diseases	P00.P96	United Kingdom	8.86	1	yes
P39	Other infections specific to the perinatal period	P00.P96	France	46.95	0	no
P39	Other infections specific to the perinatal period	P00.P96	Germany	6.95	0	no
P39	Other infections specific to the perinatal period	P00.P96	Netherlands	12.77	0	no
P39	Other infections specific to the perinatal period	P00.P96	Poland	41.21	3	yes
P39	Other infections specific to the perinatal period	P00.P96	Spain	5.80	0	no
P39	Other infections specific to the perinatal period	P00.P96	United Kingdom	5.36	0	no
P52	Intracranial nontraumatic haemorrhage of fetus and newborn	P00.P96	Belgium	19.35	1	yes
P52	Intracranial nontraumatic haemorrhage of fetus and newborn	P00.P96	Croatia	11.01	2	yes
P52	Intracranial nontraumatic haemorrhage of fetus and newborn	P00.P96	Czech Republic	25.73	3	yes
P52	Intracranial nontraumatic haemorrhage of fetus and newborn	P00.P96	Finland	9.07	1	yes
P52	Intracranial nontraumatic haemorrhage of fetus and newborn	P00.P96	France	125.19	0	no
P52	Intracranial nontraumatic haemorrhage of fetus and newborn	P00.P96	Germany	48.54	0	no
P52	Intracranial nontraumatic haemorrhage of fetus and newborn	P00.P96	Hungary	78.48	1	yes
P52	Intracranial nontraumatic haemorrhage of fetus and newborn	P00.P96	Latvia	14.79	1	yes
P52	Intracranial nontraumatic haemorrhage of fetus and newborn	P00.P96	Netherlands	17.30	2	yes
P52	Intracranial nontraumatic haemorrhage of fetus and newborn	P00.P96	Poland	56.35	1	yes
P52	Intracranial nontraumatic haemorrhage of fetus and newborn	P00.P96	Romania	270.83	3	yes
P52	Intracranial nontraumatic haemorrhage of fetus and newborn	P00.P96	Spain	70.57	1	yes
P52	Intracranial nontraumatic haemorrhage of fetus and newborn	P00.P96	Sweden	6.79	1	yes
P52	Intracranial nontraumatic haemorrhage of fetus and newborn	P00.P96	Switzerland	10.06	0	no
P52	Intracranial nontraumatic haemorrhage of fetus and newborn	P00.P96	United Kingdom	47.47	3	yes
P53	Haemorrhagic disease of fetus and newborn	P00.P96	Romania	63.47	3	yes
P54	Other neonatal haemorrhages	P00.P96	Romania	10.67	0	no
P55	Haemolytic disease of fetus and newborn	P00.P96	Romania	6.00	1	yes
P60	Disseminated intravascular coagulation of fetus and newborn	P00.P96	France	7.44	3	yes
P60	Disseminated intravascular coagulation of fetus and newborn	P00.P96	Spain	5.27	0	no
P61	Other perinatal haematological disorders	P00.P96	France	9.57	3	yes
P76	Other intestinal obstruction of newborn	P00.P96	Germany	5.18	1	yes
P77	Necrotizing enterocolitis of fetus and newborn	P00.P96	Belgium	6.04	0	no
P77	Necrotizing enterocolitis of fetus and newborn	P00.P96	Czech Republic	9.19	1	yes
P77	Necrotizing enterocolitis of fetus and newborn	P00.P96	France	31.85	0	no
P77	Necrotizing enterocolitis of fetus and newborn	P00.P96	Germany	44.11	1	yes

For peer review only

P77	Necrotizing enterocolitis of fetus and newborn	P00.P96	Hungary	13.86	1	yes
P77	Necrotizing enterocolitis of fetus and newborn	P00.P96	Netherlands	21.57	1	yes
P77	Necrotizing enterocolitis of fetus and newborn	P00.P96	Poland	8.98	3	yes
P77	Necrotizing enterocolitis of fetus and newborn	P00.P96	Romania	12.26	1	yes
P77	Necrotizing enterocolitis of fetus and newborn	P00.P96	Spain	47.63	3	yes
P77	Necrotizing enterocolitis of fetus and newborn	P00.P96	Sweden	5.33	1	yes
P77	Necrotizing enterocolitis of fetus and newborn	P00.P96	Switzerland	5.08	0	no
P77	Necrotizing enterocolitis of fetus and newborn	P00.P96	United Kingdom	102.30	0	no
P78	Other perinatal digestive system disorders	P00.P96	France	18.05	0	no
P78	Other perinatal digestive system disorders	P00.P96	Germany	10.82	1	yes
P78	Other perinatal digestive system disorders	P00.P96	Romania	8.53	1	yes
P78	Other perinatal digestive system disorders	P00.P96	Spain	12.41	0	no
P83	Other conditions of integument specific to fetus and newborn	P00.P96	France	9.05	1	yes
P83	Other conditions of integument specific to fetus and newborn	P00.P96	Germany	16.60	0	no
P83	Other conditions of integument specific to fetus and newborn	P00.P96	Poland	12.03	0	no
P83	Other conditions of integument specific to fetus and newborn	P00.P96	Spain	7.75	2	yes
P83	Other conditions of integument specific to fetus and newborn	P00.P96	United Kingdom	6.86	0	no
P91	Other disturbances of cerebral status of newborn	P00.P96	France	69.04	1	yes
P91	Other disturbances of cerebral status of newborn	P00.P96	Germany	9.98	2	yes
P91	Other disturbances of cerebral status of newborn	P00.P96	Latvia	14.43	2	yes
P91	Other disturbances of cerebral status of newborn	P00.P96	Spain	43.98	2	yes
P91	Other disturbances of cerebral status of newborn	P00.P96	United Kingdom	40.96	2	yes
P96	Other conditions originating in the perinatal period	P00.P96	France	30.54	3	yes
P96	Other conditions originating in the perinatal period	P00.P96	Germany	38.06	0	no
P96	Other conditions originating in the perinatal period	P00.P96	Netherlands	37.55	2	yes
P96	Other conditions originating in the perinatal period	P00.P96	Spain	83.16	0	no
P96	Other conditions originating in the perinatal period	P00.P96	United Kingdom	12.01	0	no
Q00	Anencephaly and similar malformations	Q00.Q99	France	5.01	1	yes
Q00	Anencephaly and similar malformations	Q00.Q99	Germany	18.32	0	no
Q00	Anencephaly and similar malformations	Q00.Q99	Netherlands	6.95	1	yes
Q00	Anencephaly and similar malformations	Q00.Q99	Poland	28.49	2	yes
Q00	Anencephaly and similar malformations	Q00.Q99	Spain	5.82	1	yes
Q00	Anencephaly and similar malformations	Q00.Q99	United Kingdom	19.87	1	yes
Q01	Encephalocele	Q00.Q99	Poland	9.28	2	yes
Q02	Microcephaly	Q00.Q99	France	9.56	2	yes
Q02	Microcephaly	Q00.Q99	Germany	9.67	0	no
Q02	Microcephaly	Q00.Q99	United Kingdom	18.28	2	yes
Q03	Congenital hydrocephalus	Q00.Q99	Belgium	5.31	1	yes
Q03	Congenital hydrocephalus	Q00.Q99	Czech Republic	7.12	1	yes
Q03	Congenital hydrocephalus	Q00.Q99	France	24.68	2	yes
Q03	Congenital hydrocephalus	Q00.Q99	Germany	28.78	3	yes
Q03	Congenital hydrocephalus	Q00.Q99	Hungary	9.82	1	yes
Q03	Congenital hydrocephalus	Q00.Q99	Lithuania	6.24	1	yes
Q03	Congenital hydrocephalus	Q00.Q99	Poland	41.97	1	yes
Q03	Congenital hydrocephalus	Q00.Q99	Romania	69.65	1	yes
Q03	Congenital hydrocephalus	Q00.Q99	Spain	11.76	1	yes
Q03	Congenital hydrocephalus	Q00.Q99	United Kingdom	28.00	1	yes

For peer review only

Q04	Other congenital malformations of brain	Q00.Q99	Austria	8.63	0	no
Q04	Other congenital malformations of brain	Q00.Q99	Belgium	8.04	1	yes
Q04	Other congenital malformations of brain	Q00.Q99	Czech Republic	7.15	0	no
Q04	Other congenital malformations of brain	Q00.Q99	Finland	6.31	0	no
Q04	Other congenital malformations of brain	Q00.Q99	France	40.65	1	yes
Q04	Other congenital malformations of brain	Q00.Q99	Germany	66.77	0	no
Q04	Other congenital malformations of brain	Q00.Q99	Hungary	18.12	1	yes
Q04	Other congenital malformations of brain	Q00.Q99	Netherlands	14.98	0	no
Q04	Other congenital malformations of brain	Q00.Q99	Poland	28.58	2	yes
Q04	Other congenital malformations of brain	Q00.Q99	Romania	23.25	0	no
Q04	Other congenital malformations of brain	Q00.Q99	Spain	36.96	0	no
Q04	Other congenital malformations of brain	Q00.Q99	Sweden	7.66	1	yes
Q04	Other congenital malformations of brain	Q00.Q99	Switzerland	8.76	0	no
Q04	Other congenital malformations of brain	Q00.Q99	United Kingdom	59.05	1	yes
Q05	Spina bifida	Q00.Q99	France	22.23	0	no
Q05	Spina bifida	Q00.Q99	Germany	20.65	1	yes
Q05	Spina bifida	Q00.Q99	Netherlands	11.36	2	yes
Q05	Spina bifida	Q00.Q99	Poland	13.11	1	yes
Q05	Spina bifida	Q00.Q99	Romania	32.47	1	yes
Q05	Spina bifida	Q00.Q99	Spain	9.60	0	no
Q05	Spina bifida	Q00.Q99	United Kingdom	57.71	0	no
Q07	Other congenital malformations of nervous system	Q00.Q99	France	8.68	0	no
Q07	Other congenital malformations of nervous system	Q00.Q99	Germany	13.03	0	no
Q07	Other congenital malformations of nervous system	Q00.Q99	Poland	9.78	0	no
Q07	Other congenital malformations of nervous system	Q00.Q99	Romania	5.97	0	no
Q07	Other congenital malformations of nervous system	Q00.Q99	Spain	11.07	0	no
Q07	Other congenital malformations of nervous system	Q00.Q99	United Kingdom	11.55	0	no
Q20	Congenital malformations of cardiac chambers and connections	Q00.Q99	Belgium	6.48	0	no
Q20	Congenital malformations of cardiac chambers and connections	Q00.Q99	Czech Republic	7.50	2	yes
Q20	Congenital malformations of cardiac chambers and connections	Q00.Q99	Denmark	5.34	1	yes
Q20	Congenital malformations of cardiac chambers and connections	Q00.Q99	Finland	5.69	1	yes
Q20	Congenital malformations of cardiac chambers and connections	Q00.Q99	France	47.49	1	yes
Q20	Congenital malformations of cardiac chambers and connections	Q00.Q99	Germany	52.96	0	no
Q20	Congenital malformations of cardiac chambers and connections	Q00.Q99	Hungary	16.29	1	yes
Q20	Congenital malformations of cardiac chambers and connections	Q00.Q99	Lithuania	8.49	1	yes
Q20	Congenital malformations of cardiac chambers and connections	Q00.Q99	Netherlands	10.54	0	no
Q20	Congenital malformations of cardiac chambers and connections	Q00.Q99	Poland	70.64	2	yes
Q20	Congenital malformations of cardiac chambers and connections	Q00.Q99	Romania	74.57	1	yes
Q20	Congenital malformations of cardiac chambers and connections	Q00.Q99	Spain	26.54	2	yes
Q20	Congenital malformations of cardiac chambers and connections	Q00.Q99	Switzerland	8.62	1	yes
Q20	Congenital malformations of cardiac chambers and connections	Q00.Q99	United Kingdom	40.05	1	yes

For peer review only

Q21	Congenital malformations of cardiac septa	Q00.Q99	Austria	19.61	2	yes
Q21	Congenital malformations of cardiac septa	Q00.Q99	Belgium	15.32	0	no
Q21	Congenital malformations of cardiac septa	Q00.Q99	Croatia	9.97	0	no
Q21	Congenital malformations of cardiac septa	Q00.Q99	Czech Republic	17.78	0	no
Q21	Congenital malformations of cardiac septa	Q00.Q99	Denmark	11.28	0	no
Q21	Congenital malformations of cardiac septa	Q00.Q99	Finland	21.44	0	no
Q21	Congenital malformations of cardiac septa	Q00.Q99	France	108.15	1	yes
Q21	Congenital malformations of cardiac septa	Q00.Q99	Germany	121.55	2	yes
Q21	Congenital malformations of cardiac septa	Q00.Q99	Hungary	37.94	2	yes
Q21	Congenital malformations of cardiac septa	Q00.Q99	Latvia	9.97	1	yes
Q21	Congenital malformations of cardiac septa	Q00.Q99	Lithuania	15.11	1	yes
Q21	Congenital malformations of cardiac septa	Q00.Q99	Netherlands	29.01	1	yes
Q21	Congenital malformations of cardiac septa	Q00.Q99	Norway	10.50	0	no
Q21	Congenital malformations of cardiac septa	Q00.Q99	Poland	89.71	2	yes
Q21	Congenital malformations of cardiac septa	Q00.Q99	Romania	94.95	1	yes
Q21	Congenital malformations of cardiac septa	Q00.Q99	Slovenia	5.44	1	yes
Q21	Congenital malformations of cardiac septa	Q00.Q99	Spain	73.68	2	yes
Q21	Congenital malformations of cardiac septa	Q00.Q99	Sweden	18.22	0	no
Q21	Congenital malformations of cardiac septa	Q00.Q99	Switzerland	14.50	0	no
Q21	Congenital malformations of cardiac septa	Q00.Q99	United Kingdom	160.80	2	yes
Q22	Congenital malformations of pulmonary and tricuspid valves	Q00.Q99	France	28.59	1	yes
Q22	Congenital malformations of pulmonary and tricuspid valves	Q00.Q99	Germany	28.28	0	no
Q22	Congenital malformations of pulmonary and tricuspid valves	Q00.Q99	Poland	25.19	0	no
Q22	Congenital malformations of pulmonary and tricuspid valves	Q00.Q99	Romania	7.05	0	no
Q22	Congenital malformations of pulmonary and tricuspid valves	Q00.Q99	Spain	8.12	0	no
Q22	Congenital malformations of pulmonary and tricuspid valves	Q00.Q99	United Kingdom	15.96	0	no
Q23	Congenital malformations of aortic and mitral valves	Q00.Q99	Austria	6.54	0	no
Q23	Congenital malformations of aortic and mitral valves	Q00.Q99	Belgium	10.64	1	yes
Q23	Congenital malformations of aortic and mitral valves	Q00.Q99	Croatia	5.95	0	no
Q23	Congenital malformations of aortic and mitral valves	Q00.Q99	Czech Republic	8.97	0	no
Q23	Congenital malformations of aortic and mitral valves	Q00.Q99	Denmark	5.68	2	yes
Q23	Congenital malformations of aortic and mitral valves	Q00.Q99	Finland	8.71	0	no
Q23	Congenital malformations of aortic and mitral valves	Q00.Q99	France	47.41	3	yes
Q23	Congenital malformations of aortic and mitral valves	Q00.Q99	Germany	92.00	1	yes
Q23	Congenital malformations of aortic and mitral valves	Q00.Q99	Hungary	15.87	0	no
Q23	Congenital malformations of aortic and mitral valves	Q00.Q99	Lithuania	9.98	1	yes
Q23	Congenital malformations of aortic and mitral valves	Q00.Q99	Netherlands	19.79	1	yes
Q23	Congenital malformations of aortic and mitral valves	Q00.Q99	Norway	7.27	1	yes
Q23	Congenital malformations of aortic and mitral valves	Q00.Q99	Poland	70.60	0	no
Q23	Congenital malformations of aortic and mitral valves	Q00.Q99	Romania	12.75	0	no
Q23	Congenital malformations of aortic and mitral valves	Q00.Q99	Spain	23.73	2	yes
Q23	Congenital malformations of aortic and mitral valves	Q00.Q99	Sweden	7.91	2	yes
Q23	Congenital malformations of aortic and mitral valves	Q00.Q99	Switzerland	9.67	3	yes
Q23	Congenital malformations of aortic and mitral valves	Q00.Q99	United Kingdom	103.34	1	yes
Q24	Other congenital malformations of the heart	Q00.Q99	Austria	23.84	1	yes
Q24	Other congenital malformations of the heart	Q00.Q99	Belgium	23.02	2	yes

For peer review only

Q24	Other congenital malformations of the heart	Q00.Q99	Croatia	12.33	2	yes
Q24	Other congenital malformations of the heart	Q00.Q99	Czech Republic	9.91	0	no
Q24	Other congenital malformations of the heart	Q00.Q99	Denmark	8.64	1	yes
Q24	Other congenital malformations of the heart	Q00.Q99	France	164.81	1	yes
Q24	Other congenital malformations of the heart	Q00.Q99	Germany	134.90	3	yes
Q24	Other congenital malformations of the heart	Q00.Q99	Hungary	34.21	1	yes
Q24	Other congenital malformations of the heart	Q00.Q99	Latvia	11.81	1	yes
Q24	Other congenital malformations of the heart	Q00.Q99	Lithuania	5.63	1	yes
Q24	Other congenital malformations of the heart	Q00.Q99	Netherlands	38.60	1	yes
Q24	Other congenital malformations of the heart	Q00.Q99	Norway	8.06	0	no
Q24	Other congenital malformations of the heart	Q00.Q99	Poland	124.21	3	yes
Q24	Other congenital malformations of the heart	Q00.Q99	Romania	147.03	2	yes
Q24	Other congenital malformations of the heart	Q00.Q99	Spain	164.96	1	yes
Q24	Other congenital malformations of the heart	Q00.Q99	Sweden	15.09	1	yes
Q24	Other congenital malformations of the heart	Q00.Q99	Switzerland	18.26	1	yes
Q24	Other congenital malformations of the heart	Q00.Q99	United Kingdom	228.97	1	yes
Q25	Congenital malformations of great arteries	Q00.Q99	Austria	5.18	0	no
Q25	Congenital malformations of great arteries	Q00.Q99	Belgium	6.20	0	no
Q25	Congenital malformations of great arteries	Q00.Q99	Czech Republic	9.81	1	yes
Q25	Congenital malformations of great arteries	Q00.Q99	Denmark	6.63	0	no
Q25	Congenital malformations of great arteries	Q00.Q99	France	46.69	2	yes
Q25	Congenital malformations of great arteries	Q00.Q99	Germany	83.84	1	yes
Q25	Congenital malformations of great arteries	Q00.Q99	Hungary	32.56	0	no
Q25	Congenital malformations of great arteries	Q00.Q99	Netherlands	9.64	0	no
Q25	Congenital malformations of great arteries	Q00.Q99	Poland	27.31	0	no
Q25	Congenital malformations of great arteries	Q00.Q99	Romania	27.44	3	yes
Q25	Congenital malformations of great arteries	Q00.Q99	Spain	43.43	0	no
Q25	Congenital malformations of great arteries	Q00.Q99	Sweden	6.85	0	no
Q25	Congenital malformations of great arteries	Q00.Q99	Switzerland	5.87	0	no
Q25	Congenital malformations of great arteries	Q00.Q99	United Kingdom	42.80	1	yes
Q26	Congenital malformations of great veins	Q00.Q99	France	7.16	0	no
Q26	Congenital malformations of great veins	Q00.Q99	Germany	9.13	0	no
Q26	Congenital malformations of great veins	Q00.Q99	Poland	5.59	2	yes
Q26	Congenital malformations of great veins	Q00.Q99	United Kingdom	6.97	0	no
Q27	Other congenital malformations of peripheral vascular system	Q00.Q99	France	39.69	1	yes
Q27	Other congenital malformations of peripheral vascular system	Q00.Q99	Spain	6.51	0	no
Q27	Other congenital malformations of peripheral vascular system	Q00.Q99	United Kingdom	23.03	1	yes
Q28	Other congenital malformations of circulatory system	Q00.Q99	France	22.09	1	yes
Q28	Other congenital malformations of circulatory system	Q00.Q99	Germany	24.44	2	yes
Q28	Other congenital malformations of circulatory system	Q00.Q99	Hungary	6.95	0	no
Q28	Other congenital malformations of circulatory system	Q00.Q99	Netherlands	5.42	0	no
Q28	Other congenital malformations of circulatory system	Q00.Q99	Poland	7.58	1	yes
Q28	Other congenital malformations of circulatory system	Q00.Q99	Romania	8.39	0	no
Q28	Other congenital malformations of circulatory system	Q00.Q99	Spain	31.50	1	yes
Q28	Other congenital malformations of circulatory system	Q00.Q99	Switzerland	8.25	0	no
Q28	Other congenital malformations of circulatory system	Q00.Q99	United Kingdom	39.89	2	yes
Q32	Congenital malformations of trachea and bronchus	Q00.Q99	France	6.30	0	no
Q32	Congenital malformations of trachea and bronchus	Q00.Q99	Germany	6.67	0	no

For peer review only

Q32	Congenital malformations of trachea and bronchus	Q00.Q99	United Kingdom	8.46	0	no
Q33	Congenital malformations of lung	Q00.Q99	Austria	5.04	1	yes
Q33	Congenital malformations of lung	Q00.Q99	Belgium	5.70	2	yes
Q33	Congenital malformations of lung	Q00.Q99	France	24.97	0	no
Q33	Congenital malformations of lung	Q00.Q99	Germany	70.10	1	yes
Q33	Congenital malformations of lung	Q00.Q99	Hungary	5.61	2	yes
Q33	Congenital malformations of lung	Q00.Q99	Netherlands	14.75	1	yes
Q33	Congenital malformations of lung	Q00.Q99	Poland	23.53	2	yes
Q33	Congenital malformations of lung	Q00.Q99	Romania	14.65	0	no
Q33	Congenital malformations of lung	Q00.Q99	Spain	18.23	1	yes
Q33	Congenital malformations of lung	Q00.Q99	United Kingdom	66.36	1	yes
Q38	Other congenital malformations of tongue, mouth and pharynx	Q00.Q99	United Kingdom	20.30	1	yes
Q39	Congenital malformations of oesophagus	Q00.Q99	France	12.77	2	yes
Q39	Congenital malformations of oesophagus	Q00.Q99	Germany	7.34	0	no
Q39	Congenital malformations of oesophagus	Q00.Q99	Poland	6.23	1	yes
Q39	Congenital malformations of oesophagus	Q00.Q99	Romania	17.73	3	yes
Q39	Congenital malformations of oesophagus	Q00.Q99	Spain	9.06	0	no
Q39	Congenital malformations of oesophagus	Q00.Q99	United Kingdom	16.26	0	no
Q41	Congenital absence, atresia and stenosis of small intestine	Q00.Q99	France	6.22	0	no
Q41	Congenital absence, atresia and stenosis of small intestine	Q00.Q99	Romania	16.56	1	yes
Q43	Other congenital malformations of intestine	Q00.Q99	France	26.97	1	yes
Q43	Other congenital malformations of intestine	Q00.Q99	Germany	18.63	0	no
Q43	Other congenital malformations of intestine	Q00.Q99	Hungary	5.70	0	no
Q43	Other congenital malformations of intestine	Q00.Q99	Netherlands	6.14	0	no
Q43	Other congenital malformations of intestine	Q00.Q99	Poland	7.29	0	no
Q43	Other congenital malformations of intestine	Q00.Q99	Romania	10.94	2	yes
Q43	Other congenital malformations of intestine	Q00.Q99	Spain	18.13	0	no
Q43	Other congenital malformations of intestine	Q00.Q99	United Kingdom	27.08	1	yes
Q44	Congenital malformations of gallbladder, bile ducts and liver	Q00.Q99	France	27.71	0	no
Q44	Congenital malformations of gallbladder, bile ducts and liver	Q00.Q99	Germany	15.37	0	no
Q44	Congenital malformations of gallbladder, bile ducts and liver	Q00.Q99	Romania	12.65	1	yes
Q44	Congenital malformations of gallbladder, bile ducts and liver	Q00.Q99	Spain	20.72	0	no
Q44	Congenital malformations of gallbladder, bile ducts and liver	Q00.Q99	United Kingdom	13.90	0	no
Q60	Renal agenesis and other reduction defects of kidney	Q00.Q99	France	17.45	0	no
Q60	Renal agenesis and other reduction defects of kidney	Q00.Q99	Germany	32.99	2	yes
Q60	Renal agenesis and other reduction defects of kidney	Q00.Q99	Netherlands	6.84	1	yes
Q60	Renal agenesis and other reduction defects of kidney	Q00.Q99	Poland	27.25	0	no
Q60	Renal agenesis and other reduction defects of kidney	Q00.Q99	Spain	7.48	1	yes
Q60	Renal agenesis and other reduction defects of kidney	Q00.Q99	United Kingdom	20.16	1	yes
Q61	Cystic kidney disease	Q00.Q99	Austria	12.86	0	no
Q61	Cystic kidney disease	Q00.Q99	Belgium	7.40	2	yes

For peer review only

Q61	Cystic kidney disease	Q00.Q99	Czech Republic	7.16	1	yes
Q61	Cystic kidney disease	Q00.Q99	Denmark	18.85	3	yes
Q61	Cystic kidney disease	Q00.Q99	Finland	19.33	0	no
Q61	Cystic kidney disease	Q00.Q99	France	69.27	1	yes
Q61	Cystic kidney disease	Q00.Q99	Germany	44.48	3	yes
Q61	Cystic kidney disease	Q00.Q99	Hungary	30.30	2	yes
Q61	Cystic kidney disease	Q00.Q99	Lithuania	5.52	0	no
Q61	Cystic kidney disease	Q00.Q99	Netherlands	7.39	0	no
Q61	Cystic kidney disease	Q00.Q99	Norway	7.38	0	no
Q61	Cystic kidney disease	Q00.Q99	Poland	31.71	0	no
Q61	Cystic kidney disease	Q00.Q99	Romania	9.70	1	yes
Q61	Cystic kidney disease	Q00.Q99	Spain	41.01	0	no
Q61	Cystic kidney disease	Q00.Q99	Sweden	24.64	1	yes
Q61	Cystic kidney disease	Q00.Q99	Switzerland	18.28	1	yes
Q61	Cystic kidney disease	Q00.Q99	United Kingdom	132.43	0	no
Q63	Other congenital malformations of kidney	Q00.Q99	Germany	5.06	0	no
Q63	Other congenital malformations of kidney	Q00.Q99	United Kingdom	7.02	0	no
Q67	Congenital musculoskeletal deformities of head, face, spine and chest	Q00.Q99	United Kingdom	8.09	0	no
Q76	Congenital malformations of spine and bony thorax	Q00.Q99	France	8.43	1	yes
Q77	Osteochondrodysplasia with defects of growth of tubular bones and spine	Q00.Q99	France	5.55	0	no
Q77	Osteochondrodysplasia with defects of growth of tubular bones and spine	Q00.Q99	Germany	12.43	1	yes
Q77	Osteochondrodysplasia with defects of growth of tubular bones and spine	Q00.Q99	United Kingdom	7.36	0	no
Q78	Other osteochondrodysplasias	Q00.Q99	France	10.09	0	no
Q78	Other osteochondrodysplasias	Q00.Q99	Germany	12.65	1	yes
Q78	Other osteochondrodysplasias	Q00.Q99	Spain	5.03	0	no
Q78	Other osteochondrodysplasias	Q00.Q99	United Kingdom	16.75	0	no
Q79	Congenital malformations of the musculoskeletal system, not elsewhere classified	Q00.Q99	Austria	7.91	0	no
Q79	Congenital malformations of the musculoskeletal system, not elsewhere classified	Q00.Q99	Belgium	8.52	0	no
Q79	Congenital malformations of the musculoskeletal system, not elsewhere classified	Q00.Q99	Croatia	8.53	1	yes
Q79	Congenital malformations of the musculoskeletal system, not elsewhere classified	Q00.Q99	France	55.73	1	yes
Q79	Congenital malformations of the musculoskeletal system, not elsewhere classified	Q00.Q99	Germany	56.95	0	no
Q79	Congenital malformations of the musculoskeletal system, not elsewhere classified	Q00.Q99	Hungary	8.10	0	no
Q79	Congenital malformations of the musculoskeletal system, not elsewhere classified	Q00.Q99	Netherlands	13.53	0	no
Q79	Congenital malformations of the musculoskeletal system, not elsewhere classified	Q00.Q99	Poland	54.47	0	no
Q79	Congenital malformations of the musculoskeletal system, not elsewhere classified	Q00.Q99	Romania	35.40	1	yes
Q79	Congenital malformations of the musculoskeletal system, not elsewhere classified	Q00.Q99	Spain	31.44	0	no
Q79	Congenital malformations of the musculoskeletal system, not elsewhere classified	Q00.Q99	Sweden	5.11	1	yes
Q79	Congenital malformations of the musculoskeletal system, not elsewhere classified	Q00.Q99	Switzerland	7.97	0	no
Q79	Congenital malformations of the musculoskeletal system, not elsewhere classified	Q00.Q99	United Kingdom	62.70	0	no

For peer review only

Q81	Epidermolysis bullosa	Q00.Q99	Germany	7.43	1	yes
Q81	Epidermolysis bullosa	Q00.Q99	United Kingdom	7.39	0	no
Q82	Other congenital malformations of skin	Q00.Q99	United Kingdom	6.23	0	no
Q85	Phakomatoses, not elsewhere classified	Q00.Q99	Austria	6.44	0	no
Q85	Phakomatoses, not elsewhere classified	Q00.Q99	France	30.32	1	yes
Q85	Phakomatoses, not elsewhere classified	Q00.Q99	Germany	28.14	1	yes
Q85	Phakomatoses, not elsewhere classified	Q00.Q99	Netherlands	8.82	0	no
Q85	Phakomatoses, not elsewhere classified	Q00.Q99	Poland	7.47	0	no
Q85	Phakomatoses, not elsewhere classified	Q00.Q99	Spain	22.63	0	no
Q85	Phakomatoses, not elsewhere classified	Q00.Q99	Sweden	5.18	0	no
Q85	Phakomatoses, not elsewhere classified	Q00.Q99	Switzerland	6.26	0	no
Q85	Phakomatoses, not elsewhere classified	Q00.Q99	United Kingdom	27.01	0	no
Q87	Other specified congenital malformation syndromes affecting multiple systems	Q00.Q99	Austria	10.68	0	no
Q87	Other specified congenital malformation syndromes affecting multiple systems	Q00.Q99	Belgium	10.38	1	yes
Q87	Other specified congenital malformation syndromes affecting multiple systems	Q00.Q99	Denmark	7.20	2	yes
Q87	Other specified congenital malformation syndromes affecting multiple systems	Q00.Q99	Finland	7.46	0	no
Q87	Other specified congenital malformation syndromes affecting multiple systems	Q00.Q99	France	48.15	0	no
Q87	Other specified congenital malformation syndromes affecting multiple systems	Q00.Q99	Germany	90.20	1	yes
Q87	Other specified congenital malformation syndromes affecting multiple systems	Q00.Q99	Hungary	5.82	0	no
Q87	Other specified congenital malformation syndromes affecting multiple systems	Q00.Q99	Netherlands	17.94	0	no
Q87	Other specified congenital malformation syndromes affecting multiple systems	Q00.Q99	Norway	5.64	0	no
Q87	Other specified congenital malformation syndromes affecting multiple systems	Q00.Q99	Poland	25.29	2	yes
Q87	Other specified congenital malformation syndromes affecting multiple systems	Q00.Q99	Romania	5.82	0	no
Q87	Other specified congenital malformation syndromes affecting multiple systems	Q00.Q99	Spain	30.90	0	no
Q87	Other specified congenital malformation syndromes affecting multiple systems	Q00.Q99	Sweden	8.43	0	no
Q87	Other specified congenital malformation syndromes affecting multiple systems	Q00.Q99	Switzerland	13.05	2	yes
Q87	Other specified congenital malformation syndromes affecting multiple systems	Q00.Q99	United Kingdom	69.56	0	no
Q89	Other congenital malformations, not elsewhere classified	Q00.Q99	Austria	14.38	0	no
Q89	Other congenital malformations, not elsewhere classified	Q00.Q99	Belgium	21.96	0	no
Q89	Other congenital malformations, not elsewhere classified	Q00.Q99	Croatia	9.60	1	yes
Q89	Other congenital malformations, not elsewhere classified	Q00.Q99	Czech Republic	6.39	1	yes
Q89	Other congenital malformations, not elsewhere classified	Q00.Q99	France	91.32	0	no
Q89	Other congenital malformations, not elsewhere classified	Q00.Q99	Germany	71.48	0	no
Q89	Other congenital malformations, not elsewhere classified	Q00.Q99	Hungary	22.23	1	yes
Q89	Other congenital malformations, not elsewhere classified	Q00.Q99	Latvia	12.93	2	yes

For peer review only

Q89	Other congenital malformations, not elsewhere classified	Q00.Q99	Lithuania	17.71	1	yes
Q89	Other congenital malformations, not elsewhere classified	Q00.Q99	Netherlands	20.52	1	yes
Q89	Other congenital malformations, not elsewhere classified	Q00.Q99	Poland	144.12	1	yes
Q89	Other congenital malformations, not elsewhere classified	Q00.Q99	Romania	68.65	1	yes
Q89	Other congenital malformations, not elsewhere classified	Q00.Q99	Spain	62.48	1	yes
Q89	Other congenital malformations, not elsewhere classified	Q00.Q99	Sweden	5.38	1	yes
Q89	Other congenital malformations, not elsewhere classified	Q00.Q99	Switzerland	11.16	0	no
Q89	Other congenital malformations, not elsewhere classified	Q00.Q99	United Kingdom	60.49	0	no
Q90	Down's syndrome	Q00.Q99	Austria	60.48	3	yes
Q90	Down's syndrome	Q00.Q99	Belgium	35.93	1	yes
Q90	Down's syndrome	Q00.Q99	Croatia	16.56	0	no
Q90	Down's syndrome	Q00.Q99	Czech Republic	13.40	2	yes
Q90	Down's syndrome	Q00.Q99	Denmark	30.86	0	no
Q90	Down's syndrome	Q00.Q99	Finland	34.59	1	yes
Q90	Down's syndrome	Q00.Q99	France	223.58	1	yes
Q90	Down's syndrome	Q00.Q99	Germany	329.53	1	yes
Q90	Down's syndrome	Q00.Q99	Hungary	32.26	2	yes
Q90	Down's syndrome	Q00.Q99	Latvia	8.60	0	no
Q90	Down's syndrome	Q00.Q99	Lithuania	6.51	0	no
Q90	Down's syndrome	Q00.Q99	Netherlands	122.17	2	yes
Q90	Down's syndrome	Q00.Q99	Norway	34.78	0	no
Q90	Down's syndrome	Q00.Q99	Poland	27.00	2	yes
Q90	Down's syndrome	Q00.Q99	Romania	6.66	2	yes
Q90	Down's syndrome	Q00.Q99	Slovenia	6.11	0	no
Q90	Down's syndrome	Q00.Q99	Spain	125.28	1	yes
Q90	Down's syndrome	Q00.Q99	Sweden	49.15	0	no
Q90	Down's syndrome	Q00.Q99	Switzerland	54.56	0	no
Q90	Down's syndrome	Q00.Q99	United Kingdom	287.89	3	yes
Q91	Edwards' syndrome and Patau's syndrome	Q00.Q99	Austria	10.42	0	no
Q91	Edwards' syndrome and Patau's syndrome	Q00.Q99	Belgium	8.85	0	no
Q91	Edwards' syndrome and Patau's syndrome	Q00.Q99	Croatia	6.73	0	no
Q91	Edwards' syndrome and Patau's syndrome	Q00.Q99	Denmark	5.26	1	yes
Q91	Edwards' syndrome and Patau's syndrome	Q00.Q99	Finland	7.94	1	yes
Q91	Edwards' syndrome and Patau's syndrome	Q00.Q99	France	28.20	0	no
Q91	Edwards' syndrome and Patau's syndrome	Q00.Q99	Germany	103.20	0	no
Q91	Edwards' syndrome and Patau's syndrome	Q00.Q99	Hungary	7.52	0	no
Q91	Edwards' syndrome and Patau's syndrome	Q00.Q99	Lithuania	5.26	0	no
Q91	Edwards' syndrome and Patau's syndrome	Q00.Q99	Netherlands	23.05	1	yes
Q91	Edwards' syndrome and Patau's syndrome	Q00.Q99	Norway	8.17	0	no
Q91	Edwards' syndrome and Patau's syndrome	Q00.Q99	Poland	63.07	3	yes
Q91	Edwards' syndrome and Patau's syndrome	Q00.Q99	Spain	26.33	1	yes
Q91	Edwards' syndrome and Patau's syndrome	Q00.Q99	Sweden	13.82	1	yes
Q91	Edwards' syndrome and Patau's syndrome	Q00.Q99	Switzerland	13.24	0	no
Q91	Edwards' syndrome and Patau's syndrome	Q00.Q99	United Kingdom	66.81	0	no

For peer review only

Q92	Other trisomies and partial trisomies of the autosomes, not elsewhere classified	Q00.Q99	France	31.16	0	no
Q92	Other trisomies and partial trisomies of the autosomes, not elsewhere classified	Q00.Q99	Germany	15.08	2	yes
Q93	Monosomies and deletions from the autosomes, not elsewhere classified	Q00.Q99	France	6.55	0	no
Q93	Monosomies and deletions from the autosomes, not elsewhere classified	Q00.Q99	Germany	10.31	1	yes
Q93	Monosomies and deletions from the autosomes, not elsewhere classified	Q00.Q99	United Kingdom	12.08	1	yes
Q99	Other chromosome abnormalities, not elsewhere classified	Q00.Q99	France	18.27	1	yes
Q99	Other chromosome abnormalities, not elsewhere classified	Q00.Q99	Germany	19.00	0	no
Q99	Other chromosome abnormalities, not elsewhere classified	Q00.Q99	Netherlands	6.92	0	no
Q99	Other chromosome abnormalities, not elsewhere classified	Q00.Q99	Poland	5.40	2	yes
Q99	Other chromosome abnormalities, not elsewhere classified	Q00.Q99	Spain	7.64	0	no
Q99	Other chromosome abnormalities, not elsewhere classified	Q00.Q99	United Kingdom	14.27	1	yes
R00	Abnormalities of heart beat	R00.R99	Belgium	5.48	0	no
R00	Abnormalities of heart beat	R00.R99	Denmark	5.00	0	no
R00	Abnormalities of heart beat	R00.R99	France	31.93	0	no
R00	Abnormalities of heart beat	R00.R99	Germany	20.61	1	yes
R00	Abnormalities of heart beat	R00.R99	Netherlands	18.99	1	yes
R00	Abnormalities of heart beat	R00.R99	Spain	20.30	0	no
R02	Gangrene, not elsewhere classified	R00.R99	Austria	15.04	0	no
R02	Gangrene, not elsewhere classified	R00.R99	Belgium	6.03	0	no
R02	Gangrene, not elsewhere classified	R00.R99	Denmark	7.26	2	yes
R02	Gangrene, not elsewhere classified	R00.R99	France	36.35	1	yes
R02	Gangrene, not elsewhere classified	R00.R99	Germany	38.91	0	no
R02	Gangrene, not elsewhere classified	R00.R99	Spain	7.01	1	yes
R02	Gangrene, not elsewhere classified	R00.R99	United Kingdom	10.03	1	yes
R04	Haemorrhage from respiratory passages	R00.R99	Belgium	10.17	1	yes
R04	Haemorrhage from respiratory passages	R00.R99	France	62.63	1	yes
R04	Haemorrhage from respiratory passages	R00.R99	Germany	36.58	2	yes
R04	Haemorrhage from respiratory passages	R00.R99	Netherlands	49.71	2	yes
R04	Haemorrhage from respiratory passages	R00.R99	Poland	32.00	2	yes
R04	Haemorrhage from respiratory passages	R00.R99	Spain	50.29	1	yes
R04	Haemorrhage from respiratory passages	R00.R99	United Kingdom	9.91	1	yes
R06	Abnormalities of breathing	R00.R99	Belgium	17.15	1	yes
R06	Abnormalities of breathing	R00.R99	France	107.03	1	yes
R06	Abnormalities of breathing	R00.R99	Germany	25.64	0	no
R06	Abnormalities of breathing	R00.R99	Netherlands	29.64	3	yes
R06	Abnormalities of breathing	R00.R99	Spain	19.72	0	no
R06	Abnormalities of breathing	R00.R99	Sweden	12.70	2	yes
R07	Pain in throat and chest	R00.R99	France	170.05	0	no
R09	Other symptoms and signs involving the circulatory and respiratory systems	R00.R99	Austria	33.26	2	yes
R09	Other symptoms and signs involving the circulatory and respiratory systems	R00.R99	Belgium	795.57	2	yes
R09	Other symptoms and signs involving the circulatory and respiratory systems	R00.R99	Croatia	187.77	1	yes

For peer review only

R09	Other symptoms and signs involving the circulatory and respiratory systems	R00.R99	Czech Republic	111.02	2	yes
R09	Other symptoms and signs involving the circulatory and respiratory systems	R00.R99	Denmark	12.99	2	yes
R09	Other symptoms and signs involving the circulatory and respiratory systems	R00.R99	France	13352.46	0	no
R09	Other symptoms and signs involving the circulatory and respiratory systems	R00.R99	Germany	100.73	3	yes
R09	Other symptoms and signs involving the circulatory and respiratory systems	R00.R99	Netherlands	53.35	2	yes
R09	Other symptoms and signs involving the circulatory and respiratory systems	R00.R99	Norway	21.20	1	yes
R09	Other symptoms and signs involving the circulatory and respiratory systems	R00.R99	Poland	1592.80	1	yes
R09	Other symptoms and signs involving the circulatory and respiratory systems	R00.R99	Spain	1273.48	3	yes
R09	Other symptoms and signs involving the circulatory and respiratory systems	R00.R99	Sweden	13.50	0	no
R09	Other symptoms and signs involving the circulatory and respiratory systems	R00.R99	Switzerland	225.14	3	yes
R09	Other symptoms and signs involving the circulatory and respiratory systems	R00.R99	United Kingdom	28.20	3	yes
R10	Abdominal and pelvic pain	R00.R99	Belgium	11.82	0	no
R10	Abdominal and pelvic pain	R00.R99	Croatia	5.10	2	yes
R10	Abdominal and pelvic pain	R00.R99	Denmark	20.31	0	no
R10	Abdominal and pelvic pain	R00.R99	France	80.64	1	yes
R10	Abdominal and pelvic pain	R00.R99	Germany	92.06	0	no
R10	Abdominal and pelvic pain	R00.R99	Netherlands	137.21	2	yes
R10	Abdominal and pelvic pain	R00.R99	Spain	67.51	1	yes
R10	Abdominal and pelvic pain	R00.R99	Switzerland	5.09	2	yes
R10	Abdominal and pelvic pain	R00.R99	United Kingdom	5.07	0	no
R11	Nausea and vomiting	R00.R99	France	31.42	1	yes
R11	Nausea and vomiting	R00.R99	Germany	18.93	1	yes
R11	Nausea and vomiting	R00.R99	Netherlands	46.05	2	yes
R13	Dysphagia	R00.R99	Belgium	11.15	0	no
R13	Dysphagia	R00.R99	France	27.51	0	no
R13	Dysphagia	R00.R99	Germany	91.04	3	yes
R13	Dysphagia	R00.R99	Netherlands	16.56	0	no
R17	Unspecified jaundice	R00.R99	France	6.55	0	no
R17	Unspecified jaundice	R00.R99	Germany	9.53	2	yes
R17	Unspecified jaundice	R00.R99	Netherlands	18.95	2	yes
R18	Ascites	R00.R99	France	8.40	0	no
R18	Ascites	R00.R99	Germany	7.01	0	no
R19	Other symptoms and signs involving the digestive system and abdomen	R00.R99	France	11.04	0	no
R19	Other symptoms and signs involving the digestive system and abdomen	R00.R99	Germany	11.31	2	yes
R19	Other symptoms and signs involving the digestive system and abdomen	R00.R99	Netherlands	44.32	2	yes
R19	Other symptoms and signs involving the digestive system and abdomen	R00.R99	Spain	7.02	0	no
R19	Other symptoms and signs involving the digestive system and abdomen	R00.R99	United Kingdom	51.03	1	yes
R23	Other skin changes	R00.R99	France	8.92	0	no
R26	Abnormalities of gait and mobility	R00.R99	France	163.31	2	yes
R31	Unspecified haematuria	R00.R99	France	8.08	0	no

For peer review only

R31	Unspecified haematuria	R00.R99	Netherlands	9.15	0	no
R31	Unspecified haematuria	R00.R99	Spain	7.98	1	yes
R33	Retention of urine	R00.R99	Netherlands	17.10	2	yes
R34	Anuria and oliguria	R00.R99	France	5.37	0	no
R40	Somnolence, stupor and coma	R00.R99	Belgium	11.31	0	no
R40	Somnolence, stupor and coma	R00.R99	Croatia	5.57	2	yes
R40	Somnolence, stupor and coma	R00.R99	France	183.44	1	yes
R40	Somnolence, stupor and coma	R00.R99	Germany	24.69	2	yes
R40	Somnolence, stupor and coma	R00.R99	Netherlands	14.21	1	yes
R40	Somnolence, stupor and coma	R00.R99	Spain	23.84	3	yes
R41	Other symptoms and signs involving cognitive functions and awareness	R00.R99	Belgium	6.55	2	yes
R41	Other symptoms and signs involving cognitive functions and awareness	R00.R99	France	106.95	3	yes
R50	Fever of unknown origin	R00.R99	Belgium	10.36	0	no
R50	Fever of unknown origin	R00.R99	Denmark	5.73	1	yes
R50	Fever of unknown origin	R00.R99	France	83.98	0	no
R50	Fever of unknown origin	R00.R99	Germany	18.66	1	yes
R50	Fever of unknown origin	R00.R99	Netherlands	124.59	3	yes
R50	Fever of unknown origin	R00.R99	Spain	39.16	1	yes
R51	Headache	R00.R99	France	7.64	0	no
R52	Pain, not elsewhere classified	R00.R99	France	7.60	3	yes
R52	Pain, not elsewhere classified	R00.R99	Germany	6.43	1	yes
R52	Pain, not elsewhere classified	R00.R99	Netherlands	16.70	1	yes
R53	Malaise and fatigue	R00.R99	Belgium	139.15	3	yes
R53	Malaise and fatigue	R00.R99	Denmark	59.26	0	no
R53	Malaise and fatigue	R00.R99	France	1759.51	1	yes
R53	Malaise and fatigue	R00.R99	Germany	17.52	1	yes
R53	Malaise and fatigue	R00.R99	Netherlands	188.89	1	yes
R53	Malaise and fatigue	R00.R99	Norway	10.85	1	yes
R53	Malaise and fatigue	R00.R99	Spain	60.93	3	yes
R53	Malaise and fatigue	R00.R99	Sweden	57.44	1	yes
R53	Malaise and fatigue	R00.R99	United Kingdom	167.39	3	yes
R54	Senility	R00.R99	Austria	702.81	2	yes
R54	Senility	R00.R99	Belgium	1137.07	2	yes
R54	Senility	R00.R99	Croatia	597.92	1	yes
R54	Senility	R00.R99	Czech Republic	181.69	1	yes
R54	Senility	R00.R99	Denmark	829.14	3	yes
R54	Senility	R00.R99	Estonia	514.48	3	yes
R54	Senility	R00.R99	Finland	18.24	3	yes
R54	Senility	R00.R99	France	5732.29	3	yes
R54	Senility	R00.R99	Germany	4462.96	1	yes
R54	Senility	R00.R99	Hungary	86.70	3	yes
R54	Senility	R00.R99	Latvia	1861.35	3	yes
R54	Senility	R00.R99	Lithuania	48.06	3	yes
R54	Senility	R00.R99	Netherlands	2203.33	2	yes
R54	Senility	R00.R99	Norway	489.06	2	yes
R54	Senility	R00.R99	Poland	17652.87	2	yes
R54	Senility	R00.R99	Slovenia	108.95	2	yes
R54	Senility	R00.R99	Spain	2955.32	3	yes
R54	Senility	R00.R99	Sweden	1301.58	2	yes

For peer review only

R54	Senility	R00.R99	Switzerland	472.77	2	yes
R54	Senility	R00.R99	United Kingdom	11499.12	2	yes
R55	Syncope and collapse	R00.R99	Belgium	11.53	0	no
R55	Syncope and collapse	R00.R99	France	228.06	2	yes
R55	Syncope and collapse	R00.R99	Germany	97.39	3	yes
R55	Syncope and collapse	R00.R99	Netherlands	7.39	2	yes
R55	Syncope and collapse	R00.R99	Spain	22.66	1	yes
R56	Convulsions, not elsewhere classified	R00.R99	France	47.74	1	yes
R56	Convulsions, not elsewhere classified	R00.R99	Germany	41.13	0	no
R56	Convulsions, not elsewhere classified	R00.R99	Netherlands	7.89	1	yes
R56	Convulsions, not elsewhere classified	R00.R99	United Kingdom	8.20	0	no
R57	Shock, not elsewhere classified	R00.R99	Belgium	178.12	2	yes
R57	Shock, not elsewhere classified	R00.R99	Croatia	6.96	3	yes
R57	Shock, not elsewhere classified	R00.R99	Czech Republic	13.87	0	no
R57	Shock, not elsewhere classified	R00.R99	Denmark	14.24	0	no
R57	Shock, not elsewhere classified	R00.R99	France	1236.35	3	yes
R57	Shock, not elsewhere classified	R00.R99	Germany	174.87	2	yes
R57	Shock, not elsewhere classified	R00.R99	Netherlands	40.89	3	yes
R57	Shock, not elsewhere classified	R00.R99	Poland	271.60	2	yes
R57	Shock, not elsewhere classified	R00.R99	Spain	664.65	2	yes
R57	Shock, not elsewhere classified	R00.R99	Sweden	119.69	1	yes
R57	Shock, not elsewhere classified	R00.R99	Switzerland	9.64	0	no
R57	Shock, not elsewhere classified	R00.R99	United Kingdom	7.66	1	yes
R58	Haemorrhage, not elsewhere classified	R00.R99	Belgium	27.91	1	yes
R58	Haemorrhage, not elsewhere classified	R00.R99	Denmark	5.50	0	no
R58	Haemorrhage, not elsewhere classified	R00.R99	France	129.16	1	yes
R58	Haemorrhage, not elsewhere classified	R00.R99	Germany	63.92	0	no
R58	Haemorrhage, not elsewhere classified	R00.R99	Netherlands	105.46	2	yes
R58	Haemorrhage, not elsewhere classified	R00.R99	Spain	144.31	2	yes
R58	Haemorrhage, not elsewhere classified	R00.R99	United Kingdom	27.09	2	yes
R60	Oedema, not elsewhere classified	R00.R99	France	32.65	1	yes
R60	Oedema, not elsewhere classified	R00.R99	Germany	5.22	1	yes
R60	Oedema, not elsewhere classified	R00.R99	Spain	5.40	1	yes
R63	Symptoms and signs concerning food and fluid intake	R00.R99	Belgium	18.84	2	yes
R63	Symptoms and signs concerning food and fluid intake	R00.R99	Denmark	6.15	0	no
R63	Symptoms and signs concerning food and fluid intake	R00.R99	France	125.68	1	yes
R63	Symptoms and signs concerning food and fluid intake	R00.R99	Germany	78.57	1	yes
R63	Symptoms and signs concerning food and fluid intake	R00.R99	Netherlands	45.81	3	yes
R63	Symptoms and signs concerning food and fluid intake	R00.R99	Norway	17.22	2	yes
R63	Symptoms and signs concerning food and fluid intake	R00.R99	Spain	5.99	0	no
R64	Cachexia	R00.R99	Austria	55.33	0	no
R64	Cachexia	R00.R99	Belgium	106.18	3	yes
R64	Cachexia	R00.R99	Croatia	10.20	2	yes
R64	Cachexia	R00.R99	Czech Republic	22.58	1	yes
R64	Cachexia	R00.R99	Denmark	37.56	1	yes

For peer review only

R64	Cachexia	R00.R99	France	807.94	1	yes
R64	Cachexia	R00.R99	Germany	784.25	2	yes
R64	Cachexia	R00.R99	Netherlands	271.66	3	yes
R64	Cachexia	R00.R99	Norway	8.71	0	no
R64	Cachexia	R00.R99	Poland	105.25	0	no
R64	Cachexia	R00.R99	Spain	38.65	3	yes
R64	Cachexia	R00.R99	Sweden	17.61	3	yes
R64	Cachexia	R00.R99	Switzerland	7.45	3	yes
R64	Cachexia	R00.R99	United Kingdom	15.23	3	yes
R68	Other general symptoms and signs	R00.R99	Belgium	32.86	2	yes
R68	Other general symptoms and signs	R00.R99	France	317.57	1	yes
R68	Other general symptoms and signs	R00.R99	Germany	321.30	1	yes
R68	Other general symptoms and signs	R00.R99	Netherlands	12.25	3	yes
R68	Other general symptoms and signs	R00.R99	Norway	27.49	2	yes
R68	Other general symptoms and signs	R00.R99	Spain	390.81	3	yes
R68	Other general symptoms and signs	R00.R99	Sweden	67.76	2	yes
R68	Other general symptoms and signs	R00.R99	United Kingdom	358.97	3	yes
R73	Elevated blood glucose level	R00.R99	Germany	6.20	0	no
R73	Elevated blood glucose level	R00.R99	Netherlands	7.37	1	yes
R91	Abnormal findings on diagnostic imaging of lung	R00.R99	France	7.87	2	yes
R95	Sudden infant death syndrome	R00.R99	Austria	21.55	1	yes
R95	Sudden infant death syndrome	R00.R99	Belgium	42.10	2	yes
R95	Sudden infant death syndrome	R00.R99	Croatia	9.00	0	no
R95	Sudden infant death syndrome	R00.R99	Czech Republic	15.30	0	no
R95	Sudden infant death syndrome	R00.R99	Denmark	7.09	2	yes
R95	Sudden infant death syndrome	R00.R99	Finland	11.73	0	no
R95	Sudden infant death syndrome	R00.R99	France	196.16	3	yes
R95	Sudden infant death syndrome	R00.R99	Germany	276.12	2	yes
R95	Sudden infant death syndrome	R00.R99	Hungary	21.45	0	no
R95	Sudden infant death syndrome	R00.R99	Latvia	17.46	1	yes
R95	Sudden infant death syndrome	R00.R99	Lithuania	8.66	3	yes
R95	Sudden infant death syndrome	R00.R99	Netherlands	14.72	3	yes
R95	Sudden infant death syndrome	R00.R99	Norway	12.19	1	yes
R95	Sudden infant death syndrome	R00.R99	Poland	54.15	2	yes
R95	Sudden infant death syndrome	R00.R99	Romania	11.03	3	yes
R95	Sudden infant death syndrome	R00.R99	Spain	66.19	1	yes
R95	Sudden infant death syndrome	R00.R99	Sweden	18.21	2	yes
R95	Sudden infant death syndrome	R00.R99	Switzerland	12.38	1	yes
R95	Sudden infant death syndrome	R00.R99	United Kingdom	168.31	3	yes
R96	Other sudden death, cause unknown	R00.R99	Belgium	593.48	2	yes
R96	Other sudden death, cause unknown	R00.R99	Croatia	73.32	2	yes
R96	Other sudden death, cause unknown	R00.R99	Czech Republic	71.68	3	yes
R96	Other sudden death, cause unknown	R00.R99	Denmark	123.79	1	yes
R96	Other sudden death, cause unknown	R00.R99	Finland	10.11	1	yes
R96	Other sudden death, cause unknown	R00.R99	France	2006.19	2	yes
R96	Other sudden death, cause unknown	R00.R99	Germany	147.04	0	no
R96	Other sudden death, cause unknown	R00.R99	Hungary	7.65	1	yes
R96	Other sudden death, cause unknown	R00.R99	Latvia	7.74	0	no
R96	Other sudden death, cause unknown	R00.R99	Lithuania	13.86	0	no
R96	Other sudden death, cause unknown	R00.R99	Netherlands	596.14	2	yes

For peer review only

R96	Other sudden death, cause unknown	R00.R99	Norway	698.84	3	yes
R96	Other sudden death, cause unknown	R00.R99	Poland	8642.09	2	yes
R96	Other sudden death, cause unknown	R00.R99	Spain	208.73	0	no
R96	Other sudden death, cause unknown	R00.R99	Sweden	11.15	1	yes
R96	Other sudden death, cause unknown	R00.R99	Switzerland	33.33	0	no
R96	Other sudden death, cause unknown	R00.R99	United Kingdom	64.43	1	yes
R98	Unattended death	R00.R99	Belgium	210.40	1	yes
R98	Unattended death	R00.R99	Croatia	17.69	2	yes
R98	Unattended death	R00.R99	Czech Republic	283.25	2	yes
R98	Unattended death	R00.R99	Denmark	526.00	1	yes
R98	Unattended death	R00.R99	Finland	107.71	0	no
R98	Unattended death	R00.R99	France	522.46	1	yes
R98	Unattended death	R00.R99	Germany	4226.09	1	yes
R98	Unattended death	R00.R99	Hungary	25.84	1	yes
R98	Unattended death	R00.R99	Latvia	190.76	1	yes
R98	Unattended death	R00.R99	Lithuania	22.69	2	yes
R98	Unattended death	R00.R99	Netherlands	199.23	3	yes
R98	Unattended death	R00.R99	Norway	91.12	1	yes
R98	Unattended death	R00.R99	Poland	1852.61	3	yes
R98	Unattended death	R00.R99	Romania	25.65	0	no
R98	Unattended death	R00.R99	Spain	439.62	1	yes
R98	Unattended death	R00.R99	Switzerland	29.28	3	yes
R98	Unattended death	R00.R99	United Kingdom	22.35	1	yes
R99	Other ill-defined and unspecified causes of mortality	R00.R99	Austria	770.20	1	yes
R99	Other ill-defined and unspecified causes of mortality	R00.R99	Belgium	697.17	2	yes
R99	Other ill-defined and unspecified causes of mortality	R00.R99	Croatia	152.88	2	yes
R99	Other ill-defined and unspecified causes of mortality	R00.R99	Czech Republic	738.46	2	yes
R99	Other ill-defined and unspecified causes of mortality	R00.R99	Denmark	2507.71	2	yes
R99	Other ill-defined and unspecified causes of mortality	R00.R99	Estonia	158.72	3	yes
R99	Other ill-defined and unspecified causes of mortality	R00.R99	Finland	208.05	3	yes
R99	Other ill-defined and unspecified causes of mortality	R00.R99	France	15127.84	2	yes
R99	Other ill-defined and unspecified causes of mortality	R00.R99	Germany	12495.54	2	yes
R99	Other ill-defined and unspecified causes of mortality	R00.R99	Hungary	33.64	0	no
R99	Other ill-defined and unspecified causes of mortality	R00.R99	Latvia	121.54	3	yes
R99	Other ill-defined and unspecified causes of mortality	R00.R99	Lithuania	482.17	2	yes
R99	Other ill-defined and unspecified causes of mortality	R00.R99	Netherlands	3420.42	3	yes
R99	Other ill-defined and unspecified causes of mortality	R00.R99	Norway	1003.78	1	yes
R99	Other ill-defined and unspecified causes of mortality	R00.R99	Poland	6181.63	3	yes
R99	Other ill-defined and unspecified causes of mortality	R00.R99	Romania	1669.72	1	yes
R99	Other ill-defined and unspecified causes of mortality	R00.R99	Slovenia	398.33	2	yes
R99	Other ill-defined and unspecified causes of mortality	R00.R99	Spain	5331.01	2	yes
R99	Other ill-defined and unspecified causes of mortality	R00.R99	Sweden	1372.78	3	yes
R99	Other ill-defined and unspecified causes of mortality	R00.R99	Switzerland	1734.82	0	no
R99	Other ill-defined and unspecified causes of mortality	R00.R99	United Kingdom	1285.04	3	yes
V01	Pedestrian injured in collision with pedal cycle	V01.Y98	Germany	5.53	0	no
V01	Pedestrian injured in collision with pedal cycle	V01.Y98	Poland	6.91	1	yes
V01	Pedestrian injured in collision with pedal cycle	V01.Y98	Romania	9.22	0	no
V02	Pedestrian injured in collision with two- or three-wheeled motor vehicle	V01.Y98	France	7.32	1	yes
V02	Pedestrian injured in collision with two- or three-wheeled motor vehicle	V01.Y98	Germany	14.69	1	yes

For peer review only

V02	Pedestrian injured in collision with two- or three-wheeled motor vehicle	V01.Y98	Hungary	6.44	1	yes
V02	Pedestrian injured in collision with two- or three-wheeled motor vehicle	V01.Y98	Netherlands	5.32	2	yes
V02	Pedestrian injured in collision with two- or three-wheeled motor vehicle	V01.Y98	Poland	14.03	1	yes
V02	Pedestrian injured in collision with two- or three-wheeled motor vehicle	V01.Y98	Spain	16.95	0	no
V02	Pedestrian injured in collision with two- or three-wheeled motor vehicle	V01.Y98	United Kingdom	18.51	1	yes
V03	Pedestrian injured in collision with car, pick-up truck or van	V01.Y98	Austria	52.86	2	yes
V03	Pedestrian injured in collision with car, pick-up truck or van	V01.Y98	Belgium	57.08	1	yes
V03	Pedestrian injured in collision with car, pick-up truck or van	V01.Y98	Croatia	74.96	2	yes
V03	Pedestrian injured in collision with car, pick-up truck or van	V01.Y98	Czech Republic	146.17	2	yes
V03	Pedestrian injured in collision with car, pick-up truck or van	V01.Y98	Denmark	33.75	1	yes
V03	Pedestrian injured in collision with car, pick-up truck or van	V01.Y98	Estonia	29.69	3	yes
V03	Pedestrian injured in collision with car, pick-up truck or van	V01.Y98	Finland	29.70	1	yes
V03	Pedestrian injured in collision with car, pick-up truck or van	V01.Y98	France	86.00	2	yes
V03	Pedestrian injured in collision with car, pick-up truck or van	V01.Y98	Germany	407.96	1	yes
V03	Pedestrian injured in collision with car, pick-up truck or van	V01.Y98	Hungary	262.45	2	yes
V03	Pedestrian injured in collision with car, pick-up truck or van	V01.Y98	Latvia	95.17	3	yes
V03	Pedestrian injured in collision with car, pick-up truck or van	V01.Y98	Lithuania	156.10	3	yes
V03	Pedestrian injured in collision with car, pick-up truck or van	V01.Y98	Netherlands	57.82	3	yes
V03	Pedestrian injured in collision with car, pick-up truck or van	V01.Y98	Norway	19.67	2	yes
V03	Pedestrian injured in collision with car, pick-up truck or van	V01.Y98	Poland	1436.65	2	yes
V03	Pedestrian injured in collision with car, pick-up truck or van	V01.Y98	Romania	211.84	2	yes
V03	Pedestrian injured in collision with car, pick-up truck or van	V01.Y98	Slovenia	23.43	1	yes
V03	Pedestrian injured in collision with car, pick-up truck or van	V01.Y98	Spain	208.02	3	yes
V03	Pedestrian injured in collision with car, pick-up truck or van	V01.Y98	Sweden	34.92	1	yes
V03	Pedestrian injured in collision with car, pick-up truck or van	V01.Y98	Switzerland	37.34	1	yes
V03	Pedestrian injured in collision with car, pick-up truck or van	V01.Y98	United Kingdom	289.97	1	yes
V04	Pedestrian injured in collision with heavy transport vehicle or bus	V01.Y98	Austria	19.05	0	no
V04	Pedestrian injured in collision with heavy transport vehicle or bus	V01.Y98	Belgium	19.22	0	no
V04	Pedestrian injured in collision with heavy transport vehicle or bus	V01.Y98	Croatia	7.94	0	no
V04	Pedestrian injured in collision with heavy transport vehicle or bus	V01.Y98	Czech Republic	26.68	0	no

For peer review only

V04	Pedestrian injured in collision with heavy transport vehicle or bus	V01.Y98	Denmark	7.88	1	yes
V04	Pedestrian injured in collision with heavy transport vehicle or bus	V01.Y98	Estonia	5.39	1	yes
V04	Pedestrian injured in collision with heavy transport vehicle or bus	V01.Y98	Finland	10.72	2	yes
V04	Pedestrian injured in collision with heavy transport vehicle or bus	V01.Y98	France	31.53	0	no
V04	Pedestrian injured in collision with heavy transport vehicle or bus	V01.Y98	Germany	108.77	1	yes
V04	Pedestrian injured in collision with heavy transport vehicle or bus	V01.Y98	Hungary	20.06	1	yes
V04	Pedestrian injured in collision with heavy transport vehicle or bus	V01.Y98	Latvia	18.45	1	yes
V04	Pedestrian injured in collision with heavy transport vehicle or bus	V01.Y98	Lithuania	19.58	1	yes
V04	Pedestrian injured in collision with heavy transport vehicle or bus	V01.Y98	Netherlands	19.12	1	yes
V04	Pedestrian injured in collision with heavy transport vehicle or bus	V01.Y98	Norway	6.68	1	yes
V04	Pedestrian injured in collision with heavy transport vehicle or bus	V01.Y98	Poland	40.97	1	yes
V04	Pedestrian injured in collision with heavy transport vehicle or bus	V01.Y98	Romania	9.31	1	yes
V04	Pedestrian injured in collision with heavy transport vehicle or bus	V01.Y98	Spain	48.45	1	yes
V04	Pedestrian injured in collision with heavy transport vehicle or bus	V01.Y98	Sweden	10.97	1	yes
V04	Pedestrian injured in collision with heavy transport vehicle or bus	V01.Y98	Switzerland	11.77	2	yes
V04	Pedestrian injured in collision with heavy transport vehicle or bus	V01.Y98	United Kingdom	101.78	2	yes
V05	Pedestrian injured in collision with railway train or railway vehicle	V01.Y98	Austria	21.35	1	yes
V05	Pedestrian injured in collision with railway train or railway vehicle	V01.Y98	Belgium	20.76	0	no
V05	Pedestrian injured in collision with railway train or railway vehicle	V01.Y98	Croatia	19.94	1	yes
V05	Pedestrian injured in collision with railway train or railway vehicle	V01.Y98	Czech Republic	119.73	0	no
V05	Pedestrian injured in collision with railway train or railway vehicle	V01.Y98	Denmark	6.60	0	no
V05	Pedestrian injured in collision with railway train or railway vehicle	V01.Y98	Estonia	10.68	1	yes
V05	Pedestrian injured in collision with railway train or railway vehicle	V01.Y98	Finland	6.18	2	yes
V05	Pedestrian injured in collision with railway train or railway vehicle	V01.Y98	France	76.14	1	yes
V05	Pedestrian injured in collision with railway train or railway vehicle	V01.Y98	Germany	160.49	1	yes
V05	Pedestrian injured in collision with railway train or railway vehicle	V01.Y98	Hungary	64.89	1	yes
V05	Pedestrian injured in collision with railway train or railway vehicle	V01.Y98	Latvia	28.12	1	yes
V05	Pedestrian injured in collision with railway train or railway vehicle	V01.Y98	Lithuania	24.21	2	yes
V05	Pedestrian injured in collision with railway train or railway vehicle	V01.Y98	Netherlands	9.05	1	yes
V05	Pedestrian injured in collision with railway train or railway vehicle	V01.Y98	Poland	257.80	1	yes

For peer review only

V05	Pedestrian injured in collision with railway train or railway vehicle	V01.Y98	Romania	95.28	1	yes
V05	Pedestrian injured in collision with railway train or railway vehicle	V01.Y98	Slovenia	6.41	1	yes
V05	Pedestrian injured in collision with railway train or railway vehicle	V01.Y98	Spain	77.60	3	yes
V05	Pedestrian injured in collision with railway train or railway vehicle	V01.Y98	Sweden	8.61	0	no
V05	Pedestrian injured in collision with railway train or railway vehicle	V01.Y98	Switzerland	16.05	1	yes
V05	Pedestrian injured in collision with railway train or railway vehicle	V01.Y98	United Kingdom	36.88	0	no
V06	Pedestrian injured in collision with other nonmotor vehicle	V01.Y98	Germany	14.09	1	yes
V06	Pedestrian injured in collision with other nonmotor vehicle	V01.Y98	Hungary	7.41	2	yes
V09	Pedestrian injured in other and unspecified transport accidents	V01.Y98	Austria	8.06	1	yes
V09	Pedestrian injured in other and unspecified transport accidents	V01.Y98	Belgium	8.33	3	yes
V09	Pedestrian injured in other and unspecified transport accidents	V01.Y98	Croatia	13.13	1	yes
V09	Pedestrian injured in other and unspecified transport accidents	V01.Y98	Czech Republic	14.83	1	yes
V09	Pedestrian injured in other and unspecified transport accidents	V01.Y98	Estonia	7.45	2	yes
V09	Pedestrian injured in other and unspecified transport accidents	V01.Y98	France	34.96	1	yes
V09	Pedestrian injured in other and unspecified transport accidents	V01.Y98	Germany	54.19	1	yes
V09	Pedestrian injured in other and unspecified transport accidents	V01.Y98	Hungary	17.92	0	no
V09	Pedestrian injured in other and unspecified transport accidents	V01.Y98	Latvia	15.38	3	yes
V09	Pedestrian injured in other and unspecified transport accidents	V01.Y98	Lithuania	30.97	2	yes
V09	Pedestrian injured in other and unspecified transport accidents	V01.Y98	Netherlands	6.28	1	yes
V09	Pedestrian injured in other and unspecified transport accidents	V01.Y98	Poland	146.81	3	yes
V09	Pedestrian injured in other and unspecified transport accidents	V01.Y98	Romania	187.59	1	yes
V09	Pedestrian injured in other and unspecified transport accidents	V01.Y98	Slovenia	7.71	1	yes
V09	Pedestrian injured in other and unspecified transport accidents	V01.Y98	Spain	401.58	2	yes
V09	Pedestrian injured in other and unspecified transport accidents	V01.Y98	Switzerland	11.22	1	yes
V09	Pedestrian injured in other and unspecified transport accidents	V01.Y98	United Kingdom	195.24	1	yes
V11	Pedal cyclist injured in collision with other pedal cycle	V01.Y98	Netherlands	5.83	1	yes
V12	Pedal cyclist injured in collision with two- or three-wheeled motor vehicle	V01.Y98	Germany	8.47	2	yes
V12	Pedal cyclist injured in collision with two- or three-wheeled motor vehicle	V01.Y98	Netherlands	6.60	0	no
V13	Pedal cyclist injured in collision with car, pick-up truck or van	V01.Y98	Austria	11.42	1	yes
V13	Pedal cyclist injured in collision with car, pick-up truck or van	V01.Y98	Belgium	31.72	3	yes

For peer review only

V13	Pedal cyclist injured in collision with car, pick-up truck or van	V01.Y98	Croatia	23.66	1	yes
V13	Pedal cyclist injured in collision with car, pick-up truck or van	V01.Y98	Czech Republic	44.09	2	yes
V13	Pedal cyclist injured in collision with car, pick-up truck or van	V01.Y98	Denmark	19.80	1	yes
V13	Pedal cyclist injured in collision with car, pick-up truck or van	V01.Y98	Estonia	8.77	1	yes
V13	Pedal cyclist injured in collision with car, pick-up truck or van	V01.Y98	Finland	16.45	1	yes
V13	Pedal cyclist injured in collision with car, pick-up truck or van	V01.Y98	France	15.49	0	no
V13	Pedal cyclist injured in collision with car, pick-up truck or van	V01.Y98	Germany	157.10	1	yes
V13	Pedal cyclist injured in collision with car, pick-up truck or van	V01.Y98	Hungary	121.55	2	yes
V13	Pedal cyclist injured in collision with car, pick-up truck or van	V01.Y98	Latvia	16.67	2	yes
V13	Pedal cyclist injured in collision with car, pick-up truck or van	V01.Y98	Lithuania	40.43	1	yes
V13	Pedal cyclist injured in collision with car, pick-up truck or van	V01.Y98	Netherlands	99.48	1	yes
V13	Pedal cyclist injured in collision with car, pick-up truck or van	V01.Y98	Poland	361.87	3	yes
V13	Pedal cyclist injured in collision with car, pick-up truck or van	V01.Y98	Romania	10.93	1	yes
V13	Pedal cyclist injured in collision with car, pick-up truck or van	V01.Y98	Slovenia	8.24	0	no
V13	Pedal cyclist injured in collision with car, pick-up truck or van	V01.Y98	Spain	38.93	2	yes
V13	Pedal cyclist injured in collision with car, pick-up truck or van	V01.Y98	Sweden	13.09	2	yes
V13	Pedal cyclist injured in collision with car, pick-up truck or van	V01.Y98	Switzerland	9.13	1	yes
V13	Pedal cyclist injured in collision with car, pick-up truck or van	V01.Y98	United Kingdom	38.27	1	yes
V14	Pedal cyclist injured in collision with heavy transport vehicle or bus	V01.Y98	Belgium	9.93	1	yes
V14	Pedal cyclist injured in collision with heavy transport vehicle or bus	V01.Y98	Czech Republic	8.89	3	yes
V14	Pedal cyclist injured in collision with heavy transport vehicle or bus	V01.Y98	Denmark	10.32	1	yes
V14	Pedal cyclist injured in collision with heavy transport vehicle or bus	V01.Y98	Finland	6.83	3	yes
V14	Pedal cyclist injured in collision with heavy transport vehicle or bus	V01.Y98	Germany	54.93	0	no
V14	Pedal cyclist injured in collision with heavy transport vehicle or bus	V01.Y98	Hungary	7.09	0	no
V14	Pedal cyclist injured in collision with heavy transport vehicle or bus	V01.Y98	Lithuania	8.13	1	yes
V14	Pedal cyclist injured in collision with heavy transport vehicle or bus	V01.Y98	Netherlands	30.30	1	yes
V14	Pedal cyclist injured in collision with heavy transport vehicle or bus	V01.Y98	Poland	10.95	2	yes
V14	Pedal cyclist injured in collision with heavy transport vehicle or bus	V01.Y98	Spain	8.44	0	no
V14	Pedal cyclist injured in collision with heavy transport vehicle or bus	V01.Y98	United Kingdom	21.32	1	yes
V15	Pedal cyclist injured in collision with railway train or railway vehicle	V01.Y98	Germany	7.65	0	no

For peer review only

V17	Pedal cyclist injured in collision with fixed or stationary object	V01.Y98	Germany	7.10	0	no
V17	Pedal cyclist injured in collision with fixed or stationary object	V01.Y98	Netherlands	5.59	0	no
V18	Pedal cyclist injured in noncollision transport accident	V01.Y98	Austria	10.90	0	no
V18	Pedal cyclist injured in noncollision transport accident	V01.Y98	Belgium	18.68	2	yes
V18	Pedal cyclist injured in noncollision transport accident	V01.Y98	Croatia	6.88	0	no
V18	Pedal cyclist injured in noncollision transport accident	V01.Y98	Czech Republic	15.91	2	yes
V18	Pedal cyclist injured in noncollision transport accident	V01.Y98	Denmark	6.29	0	no
V18	Pedal cyclist injured in noncollision transport accident	V01.Y98	Finland	16.70	1	yes
V18	Pedal cyclist injured in noncollision transport accident	V01.Y98	France	28.71	0	no
V18	Pedal cyclist injured in noncollision transport accident	V01.Y98	Germany	109.89	0	no
V18	Pedal cyclist injured in noncollision transport accident	V01.Y98	Hungary	47.81	2	yes
V18	Pedal cyclist injured in noncollision transport accident	V01.Y98	Lithuania	5.45	0	no
V18	Pedal cyclist injured in noncollision transport accident	V01.Y98	Netherlands	49.69	1	yes
V18	Pedal cyclist injured in noncollision transport accident	V01.Y98	Poland	27.82	3	yes
V18	Pedal cyclist injured in noncollision transport accident	V01.Y98	Romania	11.68	0	no
V18	Pedal cyclist injured in noncollision transport accident	V01.Y98	Spain	14.86	0	no
V18	Pedal cyclist injured in noncollision transport accident	V01.Y98	Sweden	8.22	2	yes
V18	Pedal cyclist injured in noncollision transport accident	V01.Y98	Switzerland	8.48	3	yes
V18	Pedal cyclist injured in noncollision transport accident	V01.Y98	United Kingdom	22.90	0	no
V19	Pedal cyclist injured in other and unspecified transport accidents	V01.Y98	Austria	10.42	3	yes
V19	Pedal cyclist injured in other and unspecified transport accidents	V01.Y98	Belgium	10.86	0	no
V19	Pedal cyclist injured in other and unspecified transport accidents	V01.Y98	Czech Republic	16.69	1	yes
V19	Pedal cyclist injured in other and unspecified transport accidents	V01.Y98	France	20.36	0	no
V19	Pedal cyclist injured in other and unspecified transport accidents	V01.Y98	Germany	63.13	2	yes
V19	Pedal cyclist injured in other and unspecified transport accidents	V01.Y98	Hungary	10.22	0	no
V19	Pedal cyclist injured in other and unspecified transport accidents	V01.Y98	Lithuania	6.93	1	yes
V19	Pedal cyclist injured in other and unspecified transport accidents	V01.Y98	Netherlands	7.99	0	no
V19	Pedal cyclist injured in other and unspecified transport accidents	V01.Y98	Poland	49.78	3	yes
V19	Pedal cyclist injured in other and unspecified transport accidents	V01.Y98	Romania	22.77	1	yes
V19	Pedal cyclist injured in other and unspecified transport accidents	V01.Y98	Spain	16.56	0	no

For peer review only

V19	Pedal cyclist injured in other and unspecified transport accidents	V01.Y98	Switzerland	7.52	0	no
V19	Pedal cyclist injured in other and unspecified transport accidents	V01.Y98	United Kingdom	34.64	2	yes
V22	Motorcyle rider injured in collision with two- or three-wheeled motor vehicle	V01.Y98	Germany	11.05	1	yes
V22	Motorcyle rider injured in collision with two- or three-wheeled motor vehicle	V01.Y98	Spain	6.55	1	yes
V22	Motorcyle rider injured in collision with two- or three-wheeled motor vehicle	V01.Y98	United Kingdom	9.94	1	yes
V23	Motorcyle rider injured in collision with car, pick-up truck or van	V01.Y98	Austria	15.88	0	no
V23	Motorcyle rider injured in collision with car, pick-up truck or van	V01.Y98	Belgium	32.28	1	yes
V23	Motorcyle rider injured in collision with car, pick-up truck or van	V01.Y98	Croatia	19.12	2	yes
V23	Motorcyle rider injured in collision with car, pick-up truck or van	V01.Y98	Czech Republic	24.99	1	yes
V23	Motorcyle rider injured in collision with car, pick-up truck or van	V01.Y98	Denmark	23.20	1	yes
V23	Motorcyle rider injured in collision with car, pick-up truck or van	V01.Y98	Finland	10.91	0	no
V23	Motorcyle rider injured in collision with car, pick-up truck or van	V01.Y98	France	63.59	0	no
V23	Motorcyle rider injured in collision with car, pick-up truck or van	V01.Y98	Germany	230.08	2	yes
V23	Motorcyle rider injured in collision with car, pick-up truck or van	V01.Y98	Hungary	53.70	3	yes
V23	Motorcyle rider injured in collision with car, pick-up truck or van	V01.Y98	Latvia	7.40	0	no
V23	Motorcyle rider injured in collision with car, pick-up truck or van	V01.Y98	Lithuania	8.92	0	no
V23	Motorcyle rider injured in collision with car, pick-up truck or van	V01.Y98	Netherlands	47.59	1	yes
V23	Motorcyle rider injured in collision with car, pick-up truck or van	V01.Y98	Norway	9.83	1	yes
V23	Motorcyle rider injured in collision with car, pick-up truck or van	V01.Y98	Poland	91.92	3	yes
V23	Motorcyle rider injured in collision with car, pick-up truck or van	V01.Y98	Slovenia	10.66	0	no
V23	Motorcyle rider injured in collision with car, pick-up truck or van	V01.Y98	Spain	132.39	1	yes
V23	Motorcyle rider injured in collision with car, pick-up truck or van	V01.Y98	Sweden	20.04	2	yes
V23	Motorcyle rider injured in collision with car, pick-up truck or van	V01.Y98	Switzerland	14.51	0	no
V23	Motorcyle rider injured in collision with car, pick-up truck or van	V01.Y98	United Kingdom	156.97	3	yes
V24	Motorcyle rider injured in collision with heavy transport vehicle or bus	V01.Y98	France	14.24	0	no
V24	Motorcyle rider injured in collision with heavy transport vehicle or bus	V01.Y98	Germany	46.78	1	yes
V24	Motorcyle rider injured in collision with heavy transport vehicle or bus	V01.Y98	Netherlands	12.86	1	yes
V24	Motorcyle rider injured in collision with heavy transport vehicle or bus	V01.Y98	Spain	29.16	3	yes
V24	Motorcyle rider injured in collision with heavy transport vehicle or bus	V01.Y98	United Kingdom	28.49	1	yes
V27	Motorcyle rider injured in collision with fixed or stationary object	V01.Y98	Belgium	9.49	0	no

V27	Motorcyle rider injured in collision with fixed or stationary object	V01.Y98	Croatia	5.70	1	yes
V27	Motorcyle rider injured in collision with fixed or stationary object	V01.Y98	Czech Republic	14.05	0	no
V27	Motorcyle rider injured in collision with fixed or stationary object	V01.Y98	Denmark	9.10	0	no
V27	Motorcyle rider injured in collision with fixed or stationary object	V01.Y98	France	6.30	0	no
V27	Motorcyle rider injured in collision with fixed or stationary object	V01.Y98	Germany	93.05	0	no
V27	Motorcyle rider injured in collision with fixed or stationary object	V01.Y98	Hungary	14.60	0	no
V27	Motorcyle rider injured in collision with fixed or stationary object	V01.Y98	Lithuania	5.34	0	no
V27	Motorcyle rider injured in collision with fixed or stationary object	V01.Y98	Netherlands	27.71	0	no
V27	Motorcyle rider injured in collision with fixed or stationary object	V01.Y98	Poland	33.58	2	yes
V27	Motorcyle rider injured in collision with fixed or stationary object	V01.Y98	Spain	56.43	1	yes
V27	Motorcyle rider injured in collision with fixed or stationary object	V01.Y98	Sweden	7.76	0	no
V27	Motorcyle rider injured in collision with fixed or stationary object	V01.Y98	United Kingdom	85.75	1	yes
V28	Motorcyle rider injured in noncollision transport accident	V01.Y98	Austria	8.75	0	no
V28	Motorcyle rider injured in noncollision transport accident	V01.Y98	Belgium	9.80	1	yes
V28	Motorcyle rider injured in noncollision transport accident	V01.Y98	Croatia	17.87	2	yes
V28	Motorcyle rider injured in noncollision transport accident	V01.Y98	Denmark	6.24	1	yes
V28	Motorcyle rider injured in noncollision transport accident	V01.Y98	Finland	10.58	0	no
V28	Motorcyle rider injured in noncollision transport accident	V01.Y98	France	18.36	2	yes
V28	Motorcyle rider injured in noncollision transport accident	V01.Y98	Germany	76.90	1	yes
V28	Motorcyle rider injured in noncollision transport accident	V01.Y98	Hungary	15.82	0	no
V28	Motorcyle rider injured in noncollision transport accident	V01.Y98	Netherlands	16.21	1	yes
V28	Motorcyle rider injured in noncollision transport accident	V01.Y98	Norway	8.76	1	yes
V28	Motorcyle rider injured in noncollision transport accident	V01.Y98	Poland	11.21	0	no
V28	Motorcyle rider injured in noncollision transport accident	V01.Y98	Slovenia	6.68	0	no
V28	Motorcyle rider injured in noncollision transport accident	V01.Y98	Spain	73.81	1	yes
V28	Motorcyle rider injured in noncollision transport accident	V01.Y98	Sweden	13.01	0	no
V28	Motorcyle rider injured in noncollision transport accident	V01.Y98	Switzerland	5.56	1	yes
V28	Motorcyle rider injured in noncollision transport accident	V01.Y98	United Kingdom	49.47	1	yes
V29	Motorcyle rider injured in other and unspecified transport accidents	V01.Y98	Austria	33.02	2	yes
V29	Motorcyle rider injured in other and unspecified transport accidents	V01.Y98	Belgium	36.92	1	yes

For peer review only

V29	Motorcyle rider injured in other and unspecified transport accidents	V01.Y98	Croatia	12.83	0	no
V29	Motorcyle rider injured in other and unspecified transport accidents	V01.Y98	Czech Republic	13.76	1	yes
V29	Motorcyle rider injured in other and unspecified transport accidents	V01.Y98	France	262.69	3	yes
V29	Motorcyle rider injured in other and unspecified transport accidents	V01.Y98	Germany	183.64	3	yes
V29	Motorcyle rider injured in other and unspecified transport accidents	V01.Y98	Hungary	15.69	0	no
V29	Motorcyle rider injured in other and unspecified transport accidents	V01.Y98	Netherlands	10.85	3	yes
V29	Motorcyle rider injured in other and unspecified transport accidents	V01.Y98	Norway	5.64	1	yes
V29	Motorcyle rider injured in other and unspecified transport accidents	V01.Y98	Poland	46.80	2	yes
V29	Motorcyle rider injured in other and unspecified transport accidents	V01.Y98	Romania	9.36	2	yes
V29	Motorcyle rider injured in other and unspecified transport accidents	V01.Y98	Slovenia	7.49	1	yes
V29	Motorcyle rider injured in other and unspecified transport accidents	V01.Y98	Spain	87.12	1	yes
V29	Motorcyle rider injured in other and unspecified transport accidents	V01.Y98	Sweden	5.34	0	no
V29	Motorcyle rider injured in other and unspecified transport accidents	V01.Y98	Switzerland	32.38	1	yes
V29	Motorcyle rider injured in other and unspecified transport accidents	V01.Y98	United Kingdom	123.64	2	yes
V33	Occupant of three-wheeled motor vehicle injured in collision with car, pick-up truck or van	V01.Y98	Netherlands	6.71	1	yes
V38	Occupant of three-wheeled motor vehicle injured in noncollision transport accident	V01.Y98	Netherlands	12.49	1	yes
V38	Occupant of three-wheeled motor vehicle injured in noncollision transport accident	V01.Y98	United Kingdom	5.94	0	no
V40	Car occupant injured in collision with pedestrian or animal	V01.Y98	Czech Republic	11.45	1	yes
V40	Car occupant injured in collision with pedestrian or animal	V01.Y98	Sweden	5.25	0	no
V42	Car occupant injured in collision with two- or three-wheeled motor vehicle	V01.Y98	Germany	7.06	0	no
V43	Car occupant injured in collision with car, pick-up truck or van	V01.Y98	Austria	27.75	3	yes
V43	Car occupant injured in collision with car, pick-up truck or van	V01.Y98	Belgium	29.59	3	yes
V43	Car occupant injured in collision with car, pick-up truck or van	V01.Y98	Croatia	51.18	3	yes
V43	Car occupant injured in collision with car, pick-up truck or van	V01.Y98	Czech Republic	75.28	3	yes
V43	Car occupant injured in collision with car, pick-up truck or van	V01.Y98	Denmark	61.76	1	yes
V43	Car occupant injured in collision with car, pick-up truck or van	V01.Y98	Estonia	23.72	1	yes
V43	Car occupant injured in collision with car, pick-up truck or van	V01.Y98	Finland	55.84	1	yes
V43	Car occupant injured in collision with car, pick-up truck or van	V01.Y98	France	38.58	2	yes
V43	Car occupant injured in collision with car, pick-up truck or van	V01.Y98	Germany	358.08	2	yes
V43	Car occupant injured in collision with car, pick-up truck or van	V01.Y98	Hungary	202.24	3	yes

For peer review only

V43	Car occupant injured in collision with car, pick-up truck or van	V01.Y98	Latvia	39.32	1	yes
V43	Car occupant injured in collision with car, pick-up truck or van	V01.Y98	Lithuania	61.74	3	yes
V43	Car occupant injured in collision with car, pick-up truck or van	V01.Y98	Netherlands	68.91	3	yes
V43	Car occupant injured in collision with car, pick-up truck or van	V01.Y98	Norway	23.76	1	yes
V43	Car occupant injured in collision with car, pick-up truck or van	V01.Y98	Poland	820.12	1	yes
V43	Car occupant injured in collision with car, pick-up truck or van	V01.Y98	Romania	31.12	0	no
V43	Car occupant injured in collision with car, pick-up truck or van	V01.Y98	Slovenia	25.84	1	yes
V43	Car occupant injured in collision with car, pick-up truck or van	V01.Y98	Spain	521.83	3	yes
V43	Car occupant injured in collision with car, pick-up truck or van	V01.Y98	Sweden	59.33	2	yes
V43	Car occupant injured in collision with car, pick-up truck or van	V01.Y98	Switzerland	11.54	1	yes
V43	Car occupant injured in collision with car, pick-up truck or van	V01.Y98	United Kingdom	240.76	3	yes
V44	Car occupant injured in collision with heavy transport vehicle or bus	V01.Y98	Austria	16.20	3	yes
V44	Car occupant injured in collision with heavy transport vehicle or bus	V01.Y98	Belgium	19.80	1	yes
V44	Car occupant injured in collision with heavy transport vehicle or bus	V01.Y98	Croatia	19.14	0	no
V44	Car occupant injured in collision with heavy transport vehicle or bus	V01.Y98	Czech Republic	42.45	0	no
V44	Car occupant injured in collision with heavy transport vehicle or bus	V01.Y98	Denmark	25.18	2	yes
V44	Car occupant injured in collision with heavy transport vehicle or bus	V01.Y98	Estonia	5.38	0	no
V44	Car occupant injured in collision with heavy transport vehicle or bus	V01.Y98	Finland	39.49	1	yes
V44	Car occupant injured in collision with heavy transport vehicle or bus	V01.Y98	France	33.91	1	yes
V44	Car occupant injured in collision with heavy transport vehicle or bus	V01.Y98	Germany	213.51	2	yes
V44	Car occupant injured in collision with heavy transport vehicle or bus	V01.Y98	Hungary	27.96	0	no
V44	Car occupant injured in collision with heavy transport vehicle or bus	V01.Y98	Latvia	15.09	0	no
V44	Car occupant injured in collision with heavy transport vehicle or bus	V01.Y98	Lithuania	35.01	1	yes
V44	Car occupant injured in collision with heavy transport vehicle or bus	V01.Y98	Netherlands	40.84	2	yes
V44	Car occupant injured in collision with heavy transport vehicle or bus	V01.Y98	Norway	27.10	1	yes
V44	Car occupant injured in collision with heavy transport vehicle or bus	V01.Y98	Poland	40.80	3	yes
V44	Car occupant injured in collision with heavy transport vehicle or bus	V01.Y98	Slovenia	11.81	3	yes
V44	Car occupant injured in collision with heavy transport vehicle or bus	V01.Y98	Spain	183.23	3	yes
V44	Car occupant injured in collision with heavy transport vehicle or bus	V01.Y98	Sweden	46.31	1	yes
V44	Car occupant injured in collision with heavy transport vehicle or bus	V01.Y98	Switzerland	6.12	2	yes

For peer review only

V44	Car occupant injured in collision with heavy transport vehicle or bus	V01.Y98	United Kingdom	129.99	3	yes
V45	Car occupant injured in collision with railway train or railway vehicle	V01.Y98	Austria	9.89	1	yes
V45	Car occupant injured in collision with railway train or railway vehicle	V01.Y98	Croatia	6.20	0	no
V45	Car occupant injured in collision with railway train or railway vehicle	V01.Y98	Czech Republic	14.82	0	no
V45	Car occupant injured in collision with railway train or railway vehicle	V01.Y98	France	10.04	0	no
V45	Car occupant injured in collision with railway train or railway vehicle	V01.Y98	Germany	22.34	1	yes
V45	Car occupant injured in collision with railway train or railway vehicle	V01.Y98	Hungary	14.04	2	yes
V45	Car occupant injured in collision with railway train or railway vehicle	V01.Y98	Netherlands	5.41	1	yes
V45	Car occupant injured in collision with railway train or railway vehicle	V01.Y98	Poland	24.11	0	no
V45	Car occupant injured in collision with railway train or railway vehicle	V01.Y98	Romania	6.26	0	no
V45	Car occupant injured in collision with railway train or railway vehicle	V01.Y98	Spain	7.04	1	yes
V47	Car occupant injured in collision with fixed or stationary object	V01.Y98	Austria	25.37	0	no
V47	Car occupant injured in collision with fixed or stationary object	V01.Y98	Belgium	60.65	2	yes
V47	Car occupant injured in collision with fixed or stationary object	V01.Y98	Croatia	20.11	1	yes
V47	Car occupant injured in collision with fixed or stationary object	V01.Y98	Czech Republic	89.74	0	no
V47	Car occupant injured in collision with fixed or stationary object	V01.Y98	Denmark	34.68	1	yes
V47	Car occupant injured in collision with fixed or stationary object	V01.Y98	Estonia	14.69	0	no
V47	Car occupant injured in collision with fixed or stationary object	V01.Y98	Finland	10.55	1	yes
V47	Car occupant injured in collision with fixed or stationary object	V01.Y98	France	23.39	0	no
V47	Car occupant injured in collision with fixed or stationary object	V01.Y98	Germany	537.17	2	yes
V47	Car occupant injured in collision with fixed or stationary object	V01.Y98	Hungary	85.87	3	yes
V47	Car occupant injured in collision with fixed or stationary object	V01.Y98	Latvia	30.76	1	yes
V47	Car occupant injured in collision with fixed or stationary object	V01.Y98	Lithuania	53.45	3	yes
V47	Car occupant injured in collision with fixed or stationary object	V01.Y98	Netherlands	122.82	1	yes
V47	Car occupant injured in collision with fixed or stationary object	V01.Y98	Poland	386.30	2	yes
V47	Car occupant injured in collision with fixed or stationary object	V01.Y98	Romania	15.33	0	no
V47	Car occupant injured in collision with fixed or stationary object	V01.Y98	Slovenia	21.06	0	no
V47	Car occupant injured in collision with fixed or stationary object	V01.Y98	Spain	187.59	3	yes
V47	Car occupant injured in collision with fixed or stationary object	V01.Y98	Sweden	31.57	1	yes
V47	Car occupant injured in collision with fixed or stationary object	V01.Y98	Switzerland	12.81	1	yes

For peer review only

V47	Car occupant injured in collision with fixed or stationary object	V01.Y98	United Kingdom	300.00	3	yes
V48	Car occupant injured in noncollision transport accident	V01.Y98	Austria	19.52	1	yes
V48	Car occupant injured in noncollision transport accident	V01.Y98	Belgium	22.77	3	yes
V48	Car occupant injured in noncollision transport accident	V01.Y98	Croatia	48.09	2	yes
V48	Car occupant injured in noncollision transport accident	V01.Y98	Czech Republic	13.50	0	no
V48	Car occupant injured in noncollision transport accident	V01.Y98	Denmark	15.96	2	yes
V48	Car occupant injured in noncollision transport accident	V01.Y98	Estonia	12.39	0	no
V48	Car occupant injured in noncollision transport accident	V01.Y98	Finland	58.14	1	yes
V48	Car occupant injured in noncollision transport accident	V01.Y98	France	10.06	0	no
V48	Car occupant injured in noncollision transport accident	V01.Y98	Germany	143.65	2	yes
V48	Car occupant injured in noncollision transport accident	V01.Y98	Hungary	48.49	1	yes
V48	Car occupant injured in noncollision transport accident	V01.Y98	Latvia	30.24	1	yes
V48	Car occupant injured in noncollision transport accident	V01.Y98	Lithuania	58.13	3	yes
V48	Car occupant injured in noncollision transport accident	V01.Y98	Netherlands	42.43	2	yes
V48	Car occupant injured in noncollision transport accident	V01.Y98	Norway	42.19	1	yes
V48	Car occupant injured in noncollision transport accident	V01.Y98	Poland	53.93	3	yes
V48	Car occupant injured in noncollision transport accident	V01.Y98	Romania	29.41	3	yes
V48	Car occupant injured in noncollision transport accident	V01.Y98	Slovenia	11.98	1	yes
V48	Car occupant injured in noncollision transport accident	V01.Y98	Spain	371.82	2	yes
V48	Car occupant injured in noncollision transport accident	V01.Y98	Sweden	49.46	1	yes
V48	Car occupant injured in noncollision transport accident	V01.Y98	Switzerland	8.68	0	no
V48	Car occupant injured in noncollision transport accident	V01.Y98	United Kingdom	118.42	3	yes
V49	Car occupant injured in other and unspecified transport accidents	V01.Y98	Austria	44.46	1	yes
V49	Car occupant injured in other and unspecified transport accidents	V01.Y98	Belgium	98.88	1	yes
V49	Car occupant injured in other and unspecified transport accidents	V01.Y98	Croatia	49.97	1	yes
V49	Car occupant injured in other and unspecified transport accidents	V01.Y98	Czech Republic	46.46	3	yes
V49	Car occupant injured in other and unspecified transport accidents	V01.Y98	Denmark	10.66	1	yes
V49	Car occupant injured in other and unspecified transport accidents	V01.Y98	Estonia	9.36	1	yes
V49	Car occupant injured in other and unspecified transport accidents	V01.Y98	Finland	5.29	2	yes
V49	Car occupant injured in other and unspecified transport accidents	V01.Y98	France	221.20	2	yes

For peer review only

V49	Car occupant injured in other and unspecified transport accidents	V01.Y98	Germany	392.35	1	yes
V49	Car occupant injured in other and unspecified transport accidents	V01.Y98	Hungary	94.44	2	yes
V49	Car occupant injured in other and unspecified transport accidents	V01.Y98	Latvia	31.15	2	yes
V49	Car occupant injured in other and unspecified transport accidents	V01.Y98	Lithuania	23.30	1	yes
V49	Car occupant injured in other and unspecified transport accidents	V01.Y98	Netherlands	36.24	3	yes
V49	Car occupant injured in other and unspecified transport accidents	V01.Y98	Norway	30.08	1	yes
V49	Car occupant injured in other and unspecified transport accidents	V01.Y98	Poland	515.77	2	yes
V49	Car occupant injured in other and unspecified transport accidents	V01.Y98	Romania	252.70	0	no
V49	Car occupant injured in other and unspecified transport accidents	V01.Y98	Slovenia	23.46	3	yes
V49	Car occupant injured in other and unspecified transport accidents	V01.Y98	Spain	111.85	0	no
V49	Car occupant injured in other and unspecified transport accidents	V01.Y98	Sweden	13.48	1	yes
V49	Car occupant injured in other and unspecified transport accidents	V01.Y98	Switzerland	65.85	2	yes
V49	Car occupant injured in other and unspecified transport accidents	V01.Y98	United Kingdom	442.97	3	yes
V53	Occupant of pick-up truck or van injured in collision with car, pick-up truck or van	V01.Y98	Hungary	9.68	0	no
V53	Occupant of pick-up truck or van injured in collision with car, pick-up truck or van	V01.Y98	Poland	15.82	1	yes
V53	Occupant of pick-up truck or van injured in collision with car, pick-up truck or van	V01.Y98	Spain	15.68	2	yes
V53	Occupant of pick-up truck or van injured in collision with car, pick-up truck or van	V01.Y98	United Kingdom	5.70	3	yes
V54	Occupant of pick-up truck or van injured in collision with heavy transport vehicle or bus	V01.Y98	Germany	10.56	0	no
V54	Occupant of pick-up truck or van injured in collision with heavy transport vehicle or bus	V01.Y98	Spain	22.74	1	yes
V54	Occupant of pick-up truck or van injured in collision with heavy transport vehicle or bus	V01.Y98	United Kingdom	10.08	1	yes
V57	Occupant of pick-up truck or van injured in collision with fixed or stationary object	V01.Y98	Germany	7.80	0	no
V57	Occupant of pick-up truck or van injured in collision with fixed or stationary object	V01.Y98	Poland	7.50	2	yes
V57	Occupant of pick-up truck or van injured in collision with fixed or stationary object	V01.Y98	Spain	6.50	0	no
V58	Occupant of pick-up truck or van injured in noncollision transport accident	V01.Y98	Spain	17.61	0	no
V59	Occupant of pick-up truck or van injured in other and unspecified transport accidents	V01.Y98	Germany	5.62	1	yes
V59	Occupant of pick-up truck or van injured in other and unspecified transport accidents	V01.Y98	Hungary	5.81	0	no
V59	Occupant of pick-up truck or van injured in other and unspecified transport accidents	V01.Y98	Poland	12.96	2	yes
V63	Occupant of heavy transport vehicle injured in collision with car, pick-up truck or van	V01.Y98	Spain	6.10	1	yes
V64	Occupant of heavy transport vehicle injured in collision with heavy transport vehicle or bus	V01.Y98	Germany	14.54	0	no
V64	Occupant of heavy transport vehicle injured in collision with heavy transport vehicle or bus	V01.Y98	Spain	17.41	1	yes

For peer review only

V64	Occupant of heavy transport vehicle injured in collision with heavy transport vehicle or bus	V01.Y98	United Kingdom	10.15	1	yes
V67	Occupant of heavy transport vehicle injured in collision with fixed or stationary object	V01.Y98	Germany	7.49	0	no
V67	Occupant of heavy transport vehicle injured in collision with fixed or stationary object	V01.Y98	Spain	5.21	1	yes
V68	Occupant of heavy transport vehicle injured in noncollision transport accident	V01.Y98	Germany	9.05	1	yes
V68	Occupant of heavy transport vehicle injured in noncollision transport accident	V01.Y98	Poland	6.21	0	no
V68	Occupant of heavy transport vehicle injured in noncollision transport accident	V01.Y98	Spain	36.68	3	yes
V68	Occupant of heavy transport vehicle injured in noncollision transport accident	V01.Y98	United Kingdom	9.08	1	yes
V69	Occupant of heavy transport vehicle injured in other and unspecified transport accidents	V01.Y98	France	7.02	1	yes
V69	Occupant of heavy transport vehicle injured in other and unspecified transport accidents	V01.Y98	Germany	14.59	1	yes
V69	Occupant of heavy transport vehicle injured in other and unspecified transport accidents	V01.Y98	Poland	6.04	1	yes
V69	Occupant of heavy transport vehicle injured in other and unspecified transport accidents	V01.Y98	Spain	7.01	0	no
V69	Occupant of heavy transport vehicle injured in other and unspecified transport accidents	V01.Y98	United Kingdom	5.52	1	yes
V78	Bus occupant injured in noncollision transport accident	V01.Y98	Spain	6.75	0	no
V78	Bus occupant injured in noncollision transport accident	V01.Y98	United Kingdom	11.30	0	no
V79	Bus occupant injured in other and unspecified transport accidents	V01.Y98	Romania	9.84	3	yes
V80	Animal-rider or occupant of animal-drawn vehicle injured in transport accident	V01.Y98	France	10.53	1	yes
V80	Animal-rider or occupant of animal-drawn vehicle injured in transport accident	V01.Y98	Germany	16.83	2	yes
V80	Animal-rider or occupant of animal-drawn vehicle injured in transport accident	V01.Y98	Hungary	10.19	3	yes
V80	Animal-rider or occupant of animal-drawn vehicle injured in transport accident	V01.Y98	Lithuania	8.25	3	yes
V80	Animal-rider or occupant of animal-drawn vehicle injured in transport accident	V01.Y98	Poland	7.50	2	yes
V80	Animal-rider or occupant of animal-drawn vehicle injured in transport accident	V01.Y98	Romania	100.23	1	yes
V80	Animal-rider or occupant of animal-drawn vehicle injured in transport accident	V01.Y98	Spain	10.63	1	yes
V80	Animal-rider or occupant of animal-drawn vehicle injured in transport accident	V01.Y98	United Kingdom	15.11	1	yes
V81	Occupant of railway train or railway vehicle injured in transport accident	V01.Y98	France	14.65	2	yes
V81	Occupant of railway train or railway vehicle injured in transport accident	V01.Y98	Germany	23.00	0	no
V81	Occupant of railway train or railway vehicle injured in transport accident	V01.Y98	Hungary	6.18	2	yes
V81	Occupant of railway train or railway vehicle injured in transport accident	V01.Y98	Poland	31.95	1	yes
V81	Occupant of railway train or railway vehicle injured in transport accident	V01.Y98	Romania	200.42	1	yes
V81	Occupant of railway train or railway vehicle injured in transport accident	V01.Y98	Spain	17.90	0	no
V82	Occupant of streetcar injured in transport accident	V01.Y98	Germany	5.11	0	no
V83	Occupant of special vehicle mainly used on industrial premises injured in transport accident	V01.Y98	Germany	5.51	0	no

For peer review only

V83	Occupant of special vehicle mainly used on industrial premises injured in transport accident	V01.Y98	United Kingdom	7.25	0	no
V84	Occupant of special vehicle mainly used in agriculture injured in transport accident	V01.Y98	Austria	22.33	2	yes
V84	Occupant of special vehicle mainly used in agriculture injured in transport accident	V01.Y98	Croatia	37.35	0	no
V84	Occupant of special vehicle mainly used in agriculture injured in transport accident	V01.Y98	Czech Republic	5.79	2	yes
V84	Occupant of special vehicle mainly used in agriculture injured in transport accident	V01.Y98	Finland	7.65	1	yes
V84	Occupant of special vehicle mainly used in agriculture injured in transport accident	V01.Y98	Germany	31.67	1	yes
V84	Occupant of special vehicle mainly used in agriculture injured in transport accident	V01.Y98	Latvia	7.35	2	yes
V84	Occupant of special vehicle mainly used in agriculture injured in transport accident	V01.Y98	Lithuania	6.32	0	no
V84	Occupant of special vehicle mainly used in agriculture injured in transport accident	V01.Y98	Norway	6.85	0	no
V84	Occupant of special vehicle mainly used in agriculture injured in transport accident	V01.Y98	Poland	57.89	3	yes
V84	Occupant of special vehicle mainly used in agriculture injured in transport accident	V01.Y98	Romania	27.37	2	yes
V84	Occupant of special vehicle mainly used in agriculture injured in transport accident	V01.Y98	Slovenia	18.80	1	yes
V84	Occupant of special vehicle mainly used in agriculture injured in transport accident	V01.Y98	Spain	94.98	1	yes
V84	Occupant of special vehicle mainly used in agriculture injured in transport accident	V01.Y98	Switzerland	9.53	1	yes
V84	Occupant of special vehicle mainly used in agriculture injured in transport accident	V01.Y98	United Kingdom	13.14	0	no
V85	Occupant of special construction vehicle injured in transport accident	V01.Y98	Germany	9.12	0	no
V86	Occupant of special all-terrain or other motor vehicles designed primarily for off-road use, injured in transport accident	V01.Y98	Finland	13.20	0	no
V86	Occupant of special all-terrain or other motor vehicles designed primarily for off-road use, injured in transport accident	V01.Y98	France	11.00	2	yes
V86	Occupant of special all-terrain or other motor vehicles designed primarily for off-road use, injured in transport accident	V01.Y98	Germany	6.53	1	yes
V86	Occupant of special all-terrain or other motor vehicles designed primarily for off-road use, injured in transport accident	V01.Y98	Norway	5.51	0	no
V86	Occupant of special all-terrain or other motor vehicles designed primarily for off-road use, injured in transport accident	V01.Y98	Spain	6.14	3	yes
V86	Occupant of special all-terrain or other motor vehicles designed primarily for off-road use, injured in transport accident	V01.Y98	Sweden	15.18	0	no
V86	Occupant of special all-terrain or other motor vehicles designed primarily for off-road use, injured in transport accident	V01.Y98	United Kingdom	9.65	0	no
V87	Traffic accident of specified type but victim's mode of transport unknown	V01.Y98	France	9.28	0	no
V87	Traffic accident of specified type but victim's mode of transport unknown	V01.Y98	Germany	9.39	0	no
V87	Traffic accident of specified type but victim's mode of transport unknown	V01.Y98	Romania	7.50	2	yes
V87	Traffic accident of specified type but victim's mode of transport unknown	V01.Y98	Spain	9.36	1	yes

For peer review only

V89	Motor- or nonmotor-vehicle accident, type of vehicle unspecified	V01.Y98	Austria	246.87	2	yes
V89	Motor- or nonmotor-vehicle accident, type of vehicle unspecified	V01.Y98	Belgium	568.08	3	yes
V89	Motor- or nonmotor-vehicle accident, type of vehicle unspecified	V01.Y98	Croatia	105.59	2	yes
V89	Motor- or nonmotor-vehicle accident, type of vehicle unspecified	V01.Y98	Czech Republic	258.01	3	yes
V89	Motor- or nonmotor-vehicle accident, type of vehicle unspecified	V01.Y98	France	3912.36	2	yes
V89	Motor- or nonmotor-vehicle accident, type of vehicle unspecified	V01.Y98	Germany	1140.98	2	yes
V89	Motor- or nonmotor-vehicle accident, type of vehicle unspecified	V01.Y98	Lithuania	5.74	0	no
V89	Motor- or nonmotor-vehicle accident, type of vehicle unspecified	V01.Y98	Netherlands	10.40	0	no
V89	Motor- or nonmotor-vehicle accident, type of vehicle unspecified	V01.Y98	Norway	24.50	0	no
V89	Motor- or nonmotor-vehicle accident, type of vehicle unspecified	V01.Y98	Poland	354.92	3	yes
V89	Motor- or nonmotor-vehicle accident, type of vehicle unspecified	V01.Y98	Romania	1820.37	2	yes
V89	Motor- or nonmotor-vehicle accident, type of vehicle unspecified	V01.Y98	Spain	845.00	3	yes
V89	Motor- or nonmotor-vehicle accident, type of vehicle unspecified	V01.Y98	Sweden	15.79	1	yes
V89	Motor- or nonmotor-vehicle accident, type of vehicle unspecified	V01.Y98	Switzerland	101.97	2	yes
V89	Motor- or nonmotor-vehicle accident, type of vehicle unspecified	V01.Y98	United Kingdom	149.91	3	yes
V90	Accident to watercraft causing drowning and submersion	V01.Y98	Finland	25.77	2	yes
V90	Accident to watercraft causing drowning and submersion	V01.Y98	Norway	5.24	0	no
V90	Accident to watercraft causing drowning and submersion	V01.Y98	Spain	15.61	1	yes
V90	Accident to watercraft causing drowning and submersion	V01.Y98	United Kingdom	9.31	0	no
V92	Water-transport-related drowning and submersion without accident to watercraft	V01.Y98	Finland	26.02	1	yes
V92	Water-transport-related drowning and submersion without accident to watercraft	V01.Y98	Norway	18.69	0	no
V92	Water-transport-related drowning and submersion without accident to watercraft	V01.Y98	Sweden	15.32	3	yes
V92	Water-transport-related drowning and submersion without accident to watercraft	V01.Y98	United Kingdom	11.65	0	no
V93	Accident on board watercraft without accident to watercraft, not causing drowning and submersion	V01.Y98	United Kingdom	5.67	1	yes
V95	Accident to powered aircraft causing injury to occupant	V01.Y98	Austria	5.16	0	no
V95	Accident to powered aircraft causing injury to occupant	V01.Y98	Czech Republic	5.82	0	no
V95	Accident to powered aircraft causing injury to occupant	V01.Y98	France	65.86	3	yes
V95	Accident to powered aircraft causing injury to occupant	V01.Y98	Germany	30.62	0	no
V95	Accident to powered aircraft causing injury to occupant	V01.Y98	Netherlands	7.18	0	no
V95	Accident to powered aircraft causing injury to occupant	V01.Y98	Poland	10.41	0	no

For peer review only

V95	Accident to powered aircraft causing injury to occupant	V01.Y98	Spain	32.06	0	no
V95	Accident to powered aircraft causing injury to occupant	V01.Y98	Sweden	5.97	1	yes
V95	Accident to powered aircraft causing injury to occupant	V01.Y98	Switzerland	12.71	0	no
V95	Accident to powered aircraft causing injury to occupant	V01.Y98	United Kingdom	24.38	0	no
V96	Accident to nonpowered aircraft causing injury to occupant	V01.Y98	France	16.64	0	no
V96	Accident to nonpowered aircraft causing injury to occupant	V01.Y98	Germany	9.77	0	no
V96	Accident to nonpowered aircraft causing injury to occupant	V01.Y98	Spain	6.85	0	no
V96	Accident to nonpowered aircraft causing injury to occupant	V01.Y98	Switzerland	7.82	0	no
V97	Other specified air transport accidents	V01.Y98	Germany	5.72	0	no
V99	Unspecified transport accident	V01.Y98	Czech Republic	11.04	3	yes
V99	Unspecified transport accident	V01.Y98	Estonia	5.19	3	yes
V99	Unspecified transport accident	V01.Y98	Germany	84.15	2	yes
V99	Unspecified transport accident	V01.Y98	Hungary	12.68	0	no
V99	Unspecified transport accident	V01.Y98	Lithuania	5.77	2	yes
V99	Unspecified transport accident	V01.Y98	Poland	355.74	3	yes
V99	Unspecified transport accident	V01.Y98	Romania	93.41	2	yes
W00	Fall on same level involving ice and snow	V01.Y98	Finland	11.87	1	yes
W00	Fall on same level involving ice and snow	V01.Y98	Germany	21.64	0	no
W00	Fall on same level involving ice and snow	V01.Y98	Latvia	12.33	3	yes
W00	Fall on same level involving ice and snow	V01.Y98	Poland	12.94	1	yes
W00	Fall on same level involving ice and snow	V01.Y98	Sweden	5.22	0	no
W00	Fall on same level involving ice and snow	V01.Y98	United Kingdom	13.69	0	no
W01	Fall on same level from slipping, tripping and stumbling	V01.Y98	Austria	279.00	0	no
W01	Fall on same level from slipping, tripping and stumbling	V01.Y98	Belgium	6.03	0	no
W01	Fall on same level from slipping, tripping and stumbling	V01.Y98	Croatia	132.76	1	yes
W01	Fall on same level from slipping, tripping and stumbling	V01.Y98	Czech Republic	564.98	2	yes
W01	Fall on same level from slipping, tripping and stumbling	V01.Y98	Denmark	68.01	3	yes
W01	Fall on same level from slipping, tripping and stumbling	V01.Y98	Estonia	13.04	1	yes
W01	Fall on same level from slipping, tripping and stumbling	V01.Y98	Finland	793.81	2	yes
W01	Fall on same level from slipping, tripping and stumbling	V01.Y98	France	33.22	2	yes
W01	Fall on same level from slipping, tripping and stumbling	V01.Y98	Germany	369.55	2	yes
W01	Fall on same level from slipping, tripping and stumbling	V01.Y98	Hungary	921.67	2	yes
W01	Fall on same level from slipping, tripping and stumbling	V01.Y98	Latvia	47.27	1	yes
W01	Fall on same level from slipping, tripping and stumbling	V01.Y98	Lithuania	67.00	1	yes
W01	Fall on same level from slipping, tripping and stumbling	V01.Y98	Netherlands	77.45	2	yes
W01	Fall on same level from slipping, tripping and stumbling	V01.Y98	Norway	6.63	0	no

For peer review only

W01	Fall on same level from slipping, tripping and stumbling	V01.Y98	Poland	2609.65	2	yes
W01	Fall on same level from slipping, tripping and stumbling	V01.Y98	Romania	82.91	0	no
W01	Fall on same level from slipping, tripping and stumbling	V01.Y98	Slovenia	251.75	3	yes
W01	Fall on same level from slipping, tripping and stumbling	V01.Y98	Spain	18.50	2	yes
W01	Fall on same level from slipping, tripping and stumbling	V01.Y98	Sweden	25.85	1	yes
W01	Fall on same level from slipping, tripping and stumbling	V01.Y98	Switzerland	38.45	1	yes
W01	Fall on same level from slipping, tripping and stumbling	V01.Y98	United Kingdom	166.18	3	yes
W02	Fall involving ice-skates, skis, roller-skates or skateboards	V01.Y98	Austria	10.65	1	yes
W02	Fall involving ice-skates, skis, roller-skates or skateboards	V01.Y98	France	15.97	2	yes
W02	Fall involving ice-skates, skis, roller-skates or skateboards	V01.Y98	Germany	6.24	0	no
W02	Fall involving ice-skates, skis, roller-skates or skateboards	V01.Y98	Switzerland	8.63	0	no
W03	Other fall on same level due to collision with, or pushing by, another person	V01.Y98	Poland	12.11	0	no
W03	Other fall on same level due to collision with, or pushing by, another person	V01.Y98	United Kingdom	7.51	0	no
W04	Fall while being carried or supported by other persons	V01.Y98	Germany	6.29	0	no
W05	Fall involving wheelchair	V01.Y98	Austria	5.55	0	no
W05	Fall involving wheelchair	V01.Y98	Denmark	5.13	1	yes
W05	Fall involving wheelchair	V01.Y98	Finland	8.15	0	no
W05	Fall involving wheelchair	V01.Y98	Germany	90.25	1	yes
W05	Fall involving wheelchair	V01.Y98	Hungary	6.12	1	yes
W05	Fall involving wheelchair	V01.Y98	Netherlands	18.14	3	yes
W05	Fall involving wheelchair	V01.Y98	Norway	9.03	1	yes
W05	Fall involving wheelchair	V01.Y98	Spain	6.99	2	yes
W05	Fall involving wheelchair	V01.Y98	Sweden	12.71	0	no
W05	Fall involving wheelchair	V01.Y98	Switzerland	5.64	0	no
W05	Fall involving wheelchair	V01.Y98	United Kingdom	18.12	1	yes
W06	Fall involving bed	V01.Y98	Austria	12.40	3	yes
W06	Fall involving bed	V01.Y98	Belgium	19.28	1	yes
W06	Fall involving bed	V01.Y98	Croatia	33.27	1	yes
W06	Fall involving bed	V01.Y98	Czech Republic	7.47	0	no
W06	Fall involving bed	V01.Y98	Denmark	13.90	3	yes
W06	Fall involving bed	V01.Y98	Finland	46.33	1	yes
W06	Fall involving bed	V01.Y98	France	53.29	1	yes
W06	Fall involving bed	V01.Y98	Germany	300.12	0	no
W06	Fall involving bed	V01.Y98	Hungary	145.37	2	yes
W06	Fall involving bed	V01.Y98	Latvia	7.07	0	no
W06	Fall involving bed	V01.Y98	Lithuania	8.23	1	yes
W06	Fall involving bed	V01.Y98	Netherlands	131.70	1	yes
W06	Fall involving bed	V01.Y98	Norway	16.82	0	no
W06	Fall involving bed	V01.Y98	Poland	36.71	1	yes
W06	Fall involving bed	V01.Y98	Slovenia	16.85	0	no
W06	Fall involving bed	V01.Y98	Spain	31.93	2	yes

For peer review only

W06	Fall involving bed	V01.Y98	Sweden	20.08	0	no
W06	Fall involving bed	V01.Y98	Switzerland	13.76	0	no
W06	Fall involving bed	V01.Y98	United Kingdom	126.68	3	yes
W07	Fall involving chair	V01.Y98	Croatia	5.30	0	no
W07	Fall involving chair	V01.Y98	Finland	16.68	0	no
W07	Fall involving chair	V01.Y98	France	5.56	0	no
W07	Fall involving chair	V01.Y98	Germany	53.67	1	yes
W07	Fall involving chair	V01.Y98	Hungary	24.87	0	no
W07	Fall involving chair	V01.Y98	Netherlands	29.94	1	yes
W07	Fall involving chair	V01.Y98	Norway	6.22	2	yes
W07	Fall involving chair	V01.Y98	Poland	14.78	1	yes
W07	Fall involving chair	V01.Y98	Spain	6.95	2	yes
W07	Fall involving chair	V01.Y98	Sweden	5.86	0	no
W07	Fall involving chair	V01.Y98	United Kingdom	61.65	0	no
W08	Fall involving other furniture	V01.Y98	Belgium	5.92	0	no
W08	Fall involving other furniture	V01.Y98	France	7.50	0	no
W08	Fall involving other furniture	V01.Y98	Germany	48.22	0	no
W08	Fall involving other furniture	V01.Y98	Netherlands	54.33	3	yes
W08	Fall involving other furniture	V01.Y98	United Kingdom	6.05	0	no
W09	Fall involving playground equipment	V01.Y98	Poland	7.85	2	yes
W10	Fall on and from stairs and steps	V01.Y98	Austria	71.80	1	yes
W10	Fall on and from stairs and steps	V01.Y98	Belgium	235.90	2	yes
W10	Fall on and from stairs and steps	V01.Y98	Croatia	50.97	2	yes
W10	Fall on and from stairs and steps	V01.Y98	Czech Republic	71.73	0	no
W10	Fall on and from stairs and steps	V01.Y98	Denmark	37.26	0	no
W10	Fall on and from stairs and steps	V01.Y98	Estonia	17.64	1	yes
W10	Fall on and from stairs and steps	V01.Y98	Finland	88.72	1	yes
W10	Fall on and from stairs and steps	V01.Y98	France	405.10	1	yes
W10	Fall on and from stairs and steps	V01.Y98	Germany	1107.65	0	no
W10	Fall on and from stairs and steps	V01.Y98	Hungary	123.09	2	yes
W10	Fall on and from stairs and steps	V01.Y98	Latvia	38.91	1	yes
W10	Fall on and from stairs and steps	V01.Y98	Lithuania	91.55	1	yes
W10	Fall on and from stairs and steps	V01.Y98	Netherlands	257.80	2	yes
W10	Fall on and from stairs and steps	V01.Y98	Norway	58.20	2	yes
W10	Fall on and from stairs and steps	V01.Y98	Poland	465.75	2	yes
W10	Fall on and from stairs and steps	V01.Y98	Slovenia	43.59	0	no
W10	Fall on and from stairs and steps	V01.Y98	Spain	160.03	2	yes
W10	Fall on and from stairs and steps	V01.Y98	Sweden	81.91	3	yes
W10	Fall on and from stairs and steps	V01.Y98	Switzerland	95.24	1	yes
W10	Fall on and from stairs and steps	V01.Y98	United Kingdom	806.04	3	yes
W11	Fall on and from ladder	V01.Y98	Austria	15.01	0	no
W11	Fall on and from ladder	V01.Y98	Belgium	18.57	0	no
W11	Fall on and from ladder	V01.Y98	Croatia	10.91	1	yes
W11	Fall on and from ladder	V01.Y98	Czech Republic	39.33	2	yes
W11	Fall on and from ladder	V01.Y98	Denmark	5.04	0	no
W11	Fall on and from ladder	V01.Y98	Finland	7.08	0	no
W11	Fall on and from ladder	V01.Y98	France	51.67	0	no
W11	Fall on and from ladder	V01.Y98	Germany	122.62	1	yes
W11	Fall on and from ladder	V01.Y98	Hungary	37.65	1	yes
W11	Fall on and from ladder	V01.Y98	Netherlands	10.91	0	no

For peer review only

W11	Fall on and from ladder	V01.Y98	Norway	5.10	0	no
W11	Fall on and from ladder	V01.Y98	Poland	51.59	1	yes
W11	Fall on and from ladder	V01.Y98	Romania	37.86	0	no
W11	Fall on and from ladder	V01.Y98	Slovenia	5.45	0	no
W11	Fall on and from ladder	V01.Y98	Spain	17.33	0	no
W11	Fall on and from ladder	V01.Y98	Sweden	7.37	0	no
W11	Fall on and from ladder	V01.Y98	Switzerland	8.86	0	no
W11	Fall on and from ladder	V01.Y98	United Kingdom	69.10	1	yes
W12	Fall on and from scaffolding	V01.Y98	Czech Republic	5.11	2	yes
W12	Fall on and from scaffolding	V01.Y98	France	8.58	1	yes
W12	Fall on and from scaffolding	V01.Y98	Germany	23.51	1	yes
W12	Fall on and from scaffolding	V01.Y98	Hungary	6.68	2	yes
W12	Fall on and from scaffolding	V01.Y98	Poland	27.64	0	no
W12	Fall on and from scaffolding	V01.Y98	Spain	20.83	3	yes
W12	Fall on and from scaffolding	V01.Y98	United Kingdom	6.78	1	yes
W13	Fall from, out of or through building or structure	V01.Y98	Austria	22.96	1	yes
W13	Fall from, out of or through building or structure	V01.Y98	Belgium	39.32	1	yes
W13	Fall from, out of or through building or structure	V01.Y98	Croatia	36.04	0	no
W13	Fall from, out of or through building or structure	V01.Y98	Czech Republic	59.72	0	no
W13	Fall from, out of or through building or structure	V01.Y98	Denmark	13.44	1	yes
W13	Fall from, out of or through building or structure	V01.Y98	Estonia	23.12	0	no
W13	Fall from, out of or through building or structure	V01.Y98	Finland	19.03	1	yes
W13	Fall from, out of or through building or structure	V01.Y98	France	111.87	1	yes
W13	Fall from, out of or through building or structure	V01.Y98	Germany	271.76	1	yes
W13	Fall from, out of or through building or structure	V01.Y98	Hungary	67.31	1	yes
W13	Fall from, out of or through building or structure	V01.Y98	Latvia	26.63	1	yes
W13	Fall from, out of or through building or structure	V01.Y98	Lithuania	36.35	1	yes
W13	Fall from, out of or through building or structure	V01.Y98	Netherlands	27.04	0	no
W13	Fall from, out of or through building or structure	V01.Y98	Norway	10.89	3	yes
W13	Fall from, out of or through building or structure	V01.Y98	Poland	229.23	0	no
W13	Fall from, out of or through building or structure	V01.Y98	Romania	81.57	3	yes
W13	Fall from, out of or through building or structure	V01.Y98	Slovenia	13.36	1	yes
W13	Fall from, out of or through building or structure	V01.Y98	Spain	119.09	1	yes
W13	Fall from, out of or through building or structure	V01.Y98	Sweden	16.57	0	no
W13	Fall from, out of or through building or structure	V01.Y98	Switzerland	18.15	0	no
W13	Fall from, out of or through building or structure	V01.Y98	United Kingdom	107.32	0	no
W14	Fall from tree	V01.Y98	Austria	7.47	1	yes
W14	Fall from tree	V01.Y98	Croatia	7.51	0	no
W14	Fall from tree	V01.Y98	Czech Republic	7.43	0	no
W14	Fall from tree	V01.Y98	France	17.78	1	yes
W14	Fall from tree	V01.Y98	Germany	30.10	1	yes
W14	Fall from tree	V01.Y98	Hungary	14.15	0	no
W14	Fall from tree	V01.Y98	Poland	17.60	1	yes
W14	Fall from tree	V01.Y98	Romania	18.11	0	no
W14	Fall from tree	V01.Y98	Spain	13.46	0	no
W15	Fall from cliff	V01.Y98	Austria	43.93	1	yes
W15	Fall from cliff	V01.Y98	France	14.21	0	no
W15	Fall from cliff	V01.Y98	Germany	26.90	3	yes
W15	Fall from cliff	V01.Y98	Norway	7.77	0	no
W15	Fall from cliff	V01.Y98	Poland	5.42	0	no

For peer review only

W15	Fall from cliff	V01.Y98	Slovenia	11.01	0	no
W15	Fall from cliff	V01.Y98	Spain	34.54	0	no
W15	Fall from cliff	V01.Y98	Switzerland	42.59	1	yes
W15	Fall from cliff	V01.Y98	United Kingdom	27.26	0	no
W16	Diving or jumping into water causing injury other than drowning or submersion	V01.Y98	France	10.33	1	yes
W16	Diving or jumping into water causing injury other than drowning or submersion	V01.Y98	Poland	7.08	0	no
W17	Other fall from one level to another	V01.Y98	Austria	30.22	1	yes
W17	Other fall from one level to another	V01.Y98	Belgium	15.31	1	yes
W17	Other fall from one level to another	V01.Y98	Croatia	19.30	0	no
W17	Other fall from one level to another	V01.Y98	Czech Republic	38.57	2	yes
W17	Other fall from one level to another	V01.Y98	Denmark	11.31	1	yes
W17	Other fall from one level to another	V01.Y98	Finland	6.23	0	no
W17	Other fall from one level to another	V01.Y98	France	198.68	2	yes
W17	Other fall from one level to another	V01.Y98	Germany	111.87	0	no
W17	Other fall from one level to another	V01.Y98	Hungary	22.79	0	no
W17	Other fall from one level to another	V01.Y98	Latvia	20.91	0	no
W17	Other fall from one level to another	V01.Y98	Lithuania	15.86	1	yes
W17	Other fall from one level to another	V01.Y98	Netherlands	20.82	0	no
W17	Other fall from one level to another	V01.Y98	Norway	8.53	0	no
W17	Other fall from one level to another	V01.Y98	Poland	188.75	1	yes
W17	Other fall from one level to another	V01.Y98	Romania	294.93	2	yes
W17	Other fall from one level to another	V01.Y98	Slovenia	18.46	2	yes
W17	Other fall from one level to another	V01.Y98	Spain	174.01	1	yes
W17	Other fall from one level to another	V01.Y98	Sweden	6.97	0	no
W17	Other fall from one level to another	V01.Y98	Switzerland	34.83	3	yes
W17	Other fall from one level to another	V01.Y98	United Kingdom	31.51	0	no
W18	Other fall on same level	V01.Y98	Belgium	14.56	0	no
W18	Other fall on same level	V01.Y98	Croatia	198.21	1	yes
W18	Other fall on same level	V01.Y98	Czech Republic	36.52	0	no
W18	Other fall on same level	V01.Y98	Denmark	80.85	3	yes
W18	Other fall on same level	V01.Y98	Estonia	22.67	2	yes
W18	Other fall on same level	V01.Y98	Finland	211.56	2	yes
W18	Other fall on same level	V01.Y98	France	430.01	3	yes
W18	Other fall on same level	V01.Y98	Germany	314.32	3	yes
W18	Other fall on same level	V01.Y98	Hungary	407.89	2	yes
W18	Other fall on same level	V01.Y98	Latvia	62.19	1	yes
W18	Other fall on same level	V01.Y98	Lithuania	68.14	0	no
W18	Other fall on same level	V01.Y98	Netherlands	60.43	3	yes
W18	Other fall on same level	V01.Y98	Norway	101.82	3	yes
W18	Other fall on same level	V01.Y98	Poland	149.55	3	yes
W18	Other fall on same level	V01.Y98	Romania	117.54	2	yes
W18	Other fall on same level	V01.Y98	Slovenia	7.90	0	no
W18	Other fall on same level	V01.Y98	Spain	51.28	2	yes
W18	Other fall on same level	V01.Y98	Sweden	72.38	3	yes
W18	Other fall on same level	V01.Y98	Switzerland	31.83	3	yes
W18	Other fall on same level	V01.Y98	United Kingdom	20.28	0	no
W19	Unspecified fall	V01.Y98	Austria	531.49	2	yes
W19	Unspecified fall	V01.Y98	Belgium	1030.74	1	yes
W19	Unspecified fall	V01.Y98	Croatia	725.31	1	yes

For peer review only

W19	Unspecified fall	V01.Y98	Czech Republic	1059.20	1	yes
W19	Unspecified fall	V01.Y98	Denmark	214.91	1	yes
W19	Unspecified fall	V01.Y98	Estonia	39.96	1	yes
W19	Unspecified fall	V01.Y98	Finland	137.59	0	no
W19	Unspecified fall	V01.Y98	France	4843.32	2	yes
W19	Unspecified fall	V01.Y98	Germany	6597.94	3	yes
W19	Unspecified fall	V01.Y98	Hungary	1169.31	3	yes
W19	Unspecified fall	V01.Y98	Latvia	73.52	2	yes
W19	Unspecified fall	V01.Y98	Lithuania	172.68	0	no
W19	Unspecified fall	V01.Y98	Netherlands	1296.72	3	yes
W19	Unspecified fall	V01.Y98	Norway	410.67	2	yes
W19	Unspecified fall	V01.Y98	Poland	2124.08	1	yes
W19	Unspecified fall	V01.Y98	Romania	947.00	1	yes
W19	Unspecified fall	V01.Y98	Slovenia	210.13	1	yes
W19	Unspecified fall	V01.Y98	Spain	1492.20	2	yes
W19	Unspecified fall	V01.Y98	Sweden	560.82	2	yes
W19	Unspecified fall	V01.Y98	Switzerland	1189.50	2	yes
W19	Unspecified fall	V01.Y98	United Kingdom	3385.43	3	yes
W20	Struck by thrown, projected or falling object	V01.Y98	Austria	32.23	0	no
W20	Struck by thrown, projected or falling object	V01.Y98	Belgium	13.76	0	no
W20	Struck by thrown, projected or falling object	V01.Y98	Croatia	18.51	0	no
W20	Struck by thrown, projected or falling object	V01.Y98	Czech Republic	21.72	0	no
W20	Struck by thrown, projected or falling object	V01.Y98	Denmark	6.53	0	no
W20	Struck by thrown, projected or falling object	V01.Y98	Estonia	5.15	1	yes
W20	Struck by thrown, projected or falling object	V01.Y98	Finland	12.77	0	no
W20	Struck by thrown, projected or falling object	V01.Y98	France	67.07	3	yes
W20	Struck by thrown, projected or falling object	V01.Y98	Germany	111.69	1	yes
W20	Struck by thrown, projected or falling object	V01.Y98	Hungary	26.69	0	no
W20	Struck by thrown, projected or falling object	V01.Y98	Latvia	25.29	0	no
W20	Struck by thrown, projected or falling object	V01.Y98	Lithuania	22.09	0	no
W20	Struck by thrown, projected or falling object	V01.Y98	Netherlands	16.25	0	no
W20	Struck by thrown, projected or falling object	V01.Y98	Norway	7.84	0	no
W20	Struck by thrown, projected or falling object	V01.Y98	Poland	75.03	1	yes
W20	Struck by thrown, projected or falling object	V01.Y98	Romania	78.40	0	no
W20	Struck by thrown, projected or falling object	V01.Y98	Slovenia	14.31	3	yes
W20	Struck by thrown, projected or falling object	V01.Y98	Spain	62.23	3	yes
W20	Struck by thrown, projected or falling object	V01.Y98	Sweden	20.16	1	yes
W20	Struck by thrown, projected or falling object	V01.Y98	Switzerland	16.46	0	no
W20	Struck by thrown, projected or falling object	V01.Y98	United Kingdom	46.68	1	yes
W22	Striking against or struck by other objects	V01.Y98	Germany	11.10	0	no
W22	Striking against or struck by other objects	V01.Y98	Hungary	7.18	0	no
W22	Striking against or struck by other objects	V01.Y98	Poland	14.51	1	yes
W22	Striking against or struck by other objects	V01.Y98	Romania	6.36	2	yes
W22	Striking against or struck by other objects	V01.Y98	Spain	5.95	0	no
W22	Striking against or struck by other objects	V01.Y98	United Kingdom	16.15	0	no
W23	Caught, crushed, jammed or pinched in or between objects	V01.Y98	Austria	13.04	3	yes
W23	Caught, crushed, jammed or pinched in or between objects	V01.Y98	Belgium	12.71	1	yes
W23	Caught, crushed, jammed or pinched in or between objects	V01.Y98	Czech Republic	9.53	1	yes

For peer review only

W23	Caught, crushed, jammed or pinched in or between objects	V01.Y98	Denmark	6.86	0	no
W23	Caught, crushed, jammed or pinched in or between objects	V01.Y98	Estonia	6.17	0	no
W23	Caught, crushed, jammed or pinched in or between objects	V01.Y98	France	30.82	0	no
W23	Caught, crushed, jammed or pinched in or between objects	V01.Y98	Germany	52.74	0	no
W23	Caught, crushed, jammed or pinched in or between objects	V01.Y98	Hungary	9.80	1	yes
W23	Caught, crushed, jammed or pinched in or between objects	V01.Y98	Latvia	5.69	3	yes
W23	Caught, crushed, jammed or pinched in or between objects	V01.Y98	Lithuania	5.53	0	no
W23	Caught, crushed, jammed or pinched in or between objects	V01.Y98	Netherlands	15.49	1	yes
W23	Caught, crushed, jammed or pinched in or between objects	V01.Y98	Norway	10.37	3	yes
W23	Caught, crushed, jammed or pinched in or between objects	V01.Y98	Poland	39.82	0	no
W23	Caught, crushed, jammed or pinched in or between objects	V01.Y98	Romania	137.04	3	yes
W23	Caught, crushed, jammed or pinched in or between objects	V01.Y98	Spain	15.92	0	no
W23	Caught, crushed, jammed or pinched in or between objects	V01.Y98	Sweden	6.83	0	no
W23	Caught, crushed, jammed or pinched in or between objects	V01.Y98	United Kingdom	10.71	0	no
W24	Contact with lifting and transmission devices, not elsewhere classified	V01.Y98	Finland	5.20	1	yes
W24	Contact with lifting and transmission devices, not elsewhere classified	V01.Y98	Germany	15.59	0	no
W24	Contact with lifting and transmission devices, not elsewhere classified	V01.Y98	Poland	7.01	0	no
W24	Contact with lifting and transmission devices, not elsewhere classified	V01.Y98	Spain	13.27	3	yes
W24	Contact with lifting and transmission devices, not elsewhere classified	V01.Y98	United Kingdom	7.69	0	no
W25	Contact with sharp glass	V01.Y98	Poland	5.64	0	no
W26	Contact with knife, sword or dagger	V01.Y98	Spain	7.81	0	no
W29	Contact with other powered hand tools and household machinery	V01.Y98	France	9.31	0	no
W29	Contact with other powered hand tools and household machinery	V01.Y98	Germany	7.20	1	yes
W29	Contact with other powered hand tools and household machinery	V01.Y98	Poland	6.53	2	yes
W29	Contact with other powered hand tools and household machinery	V01.Y98	Romania	6.74	0	no
W30	Contact with agricultural machinery	V01.Y98	Belgium	5.16	2	yes
W30	Contact with agricultural machinery	V01.Y98	Croatia	7.29	0	no
W30	Contact with agricultural machinery	V01.Y98	France	93.79	1	yes
W30	Contact with agricultural machinery	V01.Y98	Germany	14.38	0	no
W30	Contact with agricultural machinery	V01.Y98	Hungary	7.14	0	no
W30	Contact with agricultural machinery	V01.Y98	Poland	28.25	0	no
W30	Contact with agricultural machinery	V01.Y98	Spain	14.80	1	yes
W31	Contact with other and unspecified machinery	V01.Y98	France	14.04	1	yes
W31	Contact with other and unspecified machinery	V01.Y98	Germany	19.20	1	yes
W31	Contact with other and unspecified machinery	V01.Y98	Poland	17.55	3	yes

For peer review only

W31	Contact with other and unspecified machinery	V01.Y98	Spain	24.41	1	yes
W31	Contact with other and unspecified machinery	V01.Y98	Sweden	6.38	0	no
W31	Contact with other and unspecified machinery	V01.Y98	United Kingdom	12.50	1	yes
W33	Rifle, shotgun and larger firearm discharge	V01.Y98	France	15.38	1	yes
W33	Rifle, shotgun and larger firearm discharge	V01.Y98	Spain	5.78	0	no
W34	Discharge from other and unspecified firearms	V01.Y98	France	19.44	0	no
W34	Discharge from other and unspecified firearms	V01.Y98	Germany	7.24	1	yes
W34	Discharge from other and unspecified firearms	V01.Y98	Romania	9.41	1	yes
W34	Discharge from other and unspecified firearms	V01.Y98	Spain	21.03	1	yes
W36	Explosion and rupture of gas cylinder	V01.Y98	Poland	9.27	1	yes
W40	Explosion of other materials	V01.Y98	Belgium	6.35	0	no
W40	Explosion of other materials	V01.Y98	France	15.95	1	yes
W40	Explosion of other materials	V01.Y98	Germany	12.25	1	yes
W40	Explosion of other materials	V01.Y98	Hungary	5.96	0	no
W40	Explosion of other materials	V01.Y98	Poland	11.72	0	no
W40	Explosion of other materials	V01.Y98	Romania	10.97	3	yes
W40	Explosion of other materials	V01.Y98	Spain	13.20	2	yes
W44	Foreign body entering into or through eye or natural orifice	V01.Y98	France	6.59	0	no
W44	Foreign body entering into or through eye or natural orifice	V01.Y98	United Kingdom	8.59	0	no
W49	Exposure to other and unspecified inanimate mechanical forces	V01.Y98	Germany	5.35	0	no
W49	Exposure to other and unspecified inanimate mechanical forces	V01.Y98	Poland	55.57	0	no
W49	Exposure to other and unspecified inanimate mechanical forces	V01.Y98	Romania	6.80	0	no
W49	Exposure to other and unspecified inanimate mechanical forces	V01.Y98	United Kingdom	10.09	1	yes
W50	Hit, struck, kicked, twisted, bitten or scratched by another person	V01.Y98	Poland	29.84	0	no
W54	Bitten or struck by dog	V01.Y98	Hungary	5.61	0	no
W54	Bitten or struck by dog	V01.Y98	Poland	5.46	1	yes
W55	Bitten or struck by other mammals	V01.Y98	France	16.37	1	yes
W55	Bitten or struck by other mammals	V01.Y98	Germany	14.50	1	yes
W55	Bitten or struck by other mammals	V01.Y98	Lithuania	6.74	1	yes
W55	Bitten or struck by other mammals	V01.Y98	Netherlands	5.42	0	no
W55	Bitten or struck by other mammals	V01.Y98	Poland	22.91	1	yes
W55	Bitten or struck by other mammals	V01.Y98	Romania	31.56	1	yes
W55	Bitten or struck by other mammals	V01.Y98	Spain	15.73	2	yes
W55	Bitten or struck by other mammals	V01.Y98	United Kingdom	11.39	0	no
W64	Exposure to other and unspecified animate mechanical forces	V01.Y98	Romania	10.06	0	no
W65	Drowning and submersion while in bath-tub	V01.Y98	Austria	6.10	1	yes
W65	Drowning and submersion while in bath-tub	V01.Y98	Belgium	6.36	1	yes
W65	Drowning and submersion while in bath-tub	V01.Y98	Czech Republic	10.94	0	no
W65	Drowning and submersion while in bath-tub	V01.Y98	France	16.56	1	yes
W65	Drowning and submersion while in bath-tub	V01.Y98	Germany	46.94	1	yes
W65	Drowning and submersion while in bath-tub	V01.Y98	Hungary	7.74	0	no
W65	Drowning and submersion while in bath-tub	V01.Y98	Lithuania	12.69	1	yes
W65	Drowning and submersion while in bath-tub	V01.Y98	Netherlands	6.13	0	no
W65	Drowning and submersion while in bath-tub	V01.Y98	Poland	21.98	1	yes
W65	Drowning and submersion while in bath-tub	V01.Y98	Sweden	10.73	1	yes

For peer review only

W65	Drowning and submersion while in bath-tub	V01.Y98	United Kingdom	32.07	0	no
W66	Drowning and submersion following fall into bath-tub	V01.Y98	Germany	8.27	0	no
W67	Drowning and submersion while in swimming-pool	V01.Y98	France	20.36	1	yes
W67	Drowning and submersion while in swimming-pool	V01.Y98	Germany	16.43	0	no
W67	Drowning and submersion while in swimming-pool	V01.Y98	Spain	20.43	0	no
W67	Drowning and submersion while in swimming-pool	V01.Y98	United Kingdom	5.84	0	no
W68	Drowning and submersion following fall into swimming-pool	V01.Y98	Poland	7.59	0	no
W68	Drowning and submersion following fall into swimming-pool	V01.Y98	Spain	11.94	0	no
W69	Drowning and submersion while in natural water	V01.Y98	Austria	22.72	2	yes
W69	Drowning and submersion while in natural water	V01.Y98	Belgium	8.83	0	no
W69	Drowning and submersion while in natural water	V01.Y98	Croatia	60.90	1	yes
W69	Drowning and submersion while in natural water	V01.Y98	Czech Republic	23.14	0	no
W69	Drowning and submersion while in natural water	V01.Y98	Denmark	20.12	0	no
W69	Drowning and submersion while in natural water	V01.Y98	Estonia	31.38	1	yes
W69	Drowning and submersion while in natural water	V01.Y98	Finland	52.01	1	yes
W69	Drowning and submersion while in natural water	V01.Y98	France	99.38	2	yes
W69	Drowning and submersion while in natural water	V01.Y98	Germany	108.69	2	yes
W69	Drowning and submersion while in natural water	V01.Y98	Hungary	100.99	1	yes
W69	Drowning and submersion while in natural water	V01.Y98	Latvia	98.86	1	yes
W69	Drowning and submersion while in natural water	V01.Y98	Lithuania	203.12	1	yes
W69	Drowning and submersion while in natural water	V01.Y98	Netherlands	5.75	0	no
W69	Drowning and submersion while in natural water	V01.Y98	Norway	6.61	0	no
W69	Drowning and submersion while in natural water	V01.Y98	Poland	509.82	3	yes
W69	Drowning and submersion while in natural water	V01.Y98	Romania	415.30	1	yes
W69	Drowning and submersion while in natural water	V01.Y98	Slovenia	13.83	0	no
W69	Drowning and submersion while in natural water	V01.Y98	Spain	120.62	1	yes
W69	Drowning and submersion while in natural water	V01.Y98	Sweden	18.42	2	yes
W69	Drowning and submersion while in natural water	V01.Y98	Switzerland	5.91	0	no
W69	Drowning and submersion while in natural water	V01.Y98	United Kingdom	68.37	0	no
W70	Drowning and submersion following fall into natural water	V01.Y98	Austria	14.53	0	no
W70	Drowning and submersion following fall into natural water	V01.Y98	Belgium	10.30	2	yes
W70	Drowning and submersion following fall into natural water	V01.Y98	Czech Republic	53.18	0	no
W70	Drowning and submersion following fall into natural water	V01.Y98	Denmark	6.42	1	yes
W70	Drowning and submersion following fall into natural water	V01.Y98	Estonia	8.74	3	yes
W70	Drowning and submersion following fall into natural water	V01.Y98	Finland	65.25	3	yes
W70	Drowning and submersion following fall into natural water	V01.Y98	France	27.48	0	no
W70	Drowning and submersion following fall into natural water	V01.Y98	Germany	40.98	0	no
W70	Drowning and submersion following fall into natural water	V01.Y98	Hungary	15.11	0	no
W70	Drowning and submersion following fall into natural water	V01.Y98	Lithuania	66.72	0	no
W70	Drowning and submersion following fall into natural water	V01.Y98	Netherlands	22.06	2	yes

For peer review only

W70	Drowning and submersion following fall into natural water	V01.Y98	Norway	7.18	3	yes
W70	Drowning and submersion following fall into natural water	V01.Y98	Poland	150.40	2	yes
W70	Drowning and submersion following fall into natural water	V01.Y98	Romania	27.54	3	yes
W70	Drowning and submersion following fall into natural water	V01.Y98	Slovenia	5.44	0	no
W70	Drowning and submersion following fall into natural water	V01.Y98	Spain	31.65	0	no
W70	Drowning and submersion following fall into natural water	V01.Y98	Sweden	16.65	1	yes
W70	Drowning and submersion following fall into natural water	V01.Y98	United Kingdom	36.73	0	no
W73	Other specified drowning and submersion	V01.Y98	Belgium	5.59	2	yes
W73	Other specified drowning and submersion	V01.Y98	Czech Republic	7.78	1	yes
W73	Other specified drowning and submersion	V01.Y98	Estonia	13.07	1	yes
W73	Other specified drowning and submersion	V01.Y98	Finland	6.12	2	yes
W73	Other specified drowning and submersion	V01.Y98	France	10.95	0	no
W73	Other specified drowning and submersion	V01.Y98	Germany	28.79	1	yes
W73	Other specified drowning and submersion	V01.Y98	Hungary	24.61	1	yes
W73	Other specified drowning and submersion	V01.Y98	Latvia	69.82	3	yes
W73	Other specified drowning and submersion	V01.Y98	Lithuania	13.71	1	yes
W73	Other specified drowning and submersion	V01.Y98	Poland	49.52	1	yes
W73	Other specified drowning and submersion	V01.Y98	Romania	23.48	0	no
W73	Other specified drowning and submersion	V01.Y98	Spain	16.41	1	yes
W73	Other specified drowning and submersion	V01.Y98	United Kingdom	28.69	0	no
W74	Unspecified drowning and submersion	V01.Y98	Austria	13.78	1	yes
W74	Unspecified drowning and submersion	V01.Y98	Belgium	41.12	0	no
W74	Unspecified drowning and submersion	V01.Y98	Croatia	25.09	1	yes
W74	Unspecified drowning and submersion	V01.Y98	Czech Republic	93.74	1	yes
W74	Unspecified drowning and submersion	V01.Y98	Denmark	7.32	0	no
W74	Unspecified drowning and submersion	V01.Y98	Estonia	14.07	1	yes
W74	Unspecified drowning and submersion	V01.Y98	France	882.21	1	yes
W74	Unspecified drowning and submersion	V01.Y98	Germany	164.45	0	no
W74	Unspecified drowning and submersion	V01.Y98	Hungary	16.40	1	yes
W74	Unspecified drowning and submersion	V01.Y98	Latvia	22.13	3	yes
W74	Unspecified drowning and submersion	V01.Y98	Lithuania	13.23	0	no
W74	Unspecified drowning and submersion	V01.Y98	Netherlands	49.45	2	yes
W74	Unspecified drowning and submersion	V01.Y98	Norway	49.68	1	yes
W74	Unspecified drowning and submersion	V01.Y98	Poland	203.41	3	yes
W74	Unspecified drowning and submersion	V01.Y98	Romania	482.75	3	yes
W74	Unspecified drowning and submersion	V01.Y98	Spain	309.42	1	yes
W74	Unspecified drowning and submersion	V01.Y98	Sweden	50.06	0	no
W74	Unspecified drowning and submersion	V01.Y98	Switzerland	35.94	0	no
W74	Unspecified drowning and submersion	V01.Y98	United Kingdom	59.34	1	yes
W75	Accidental suffocation and strangulation in bed	V01.Y98	Belgium	11.80	1	yes
W75	Accidental suffocation and strangulation in bed	V01.Y98	France	13.94	0	no
W75	Accidental suffocation and strangulation in bed	V01.Y98	Germany	15.43	0	no
W75	Accidental suffocation and strangulation in bed	V01.Y98	Netherlands	16.21	0	no
W75	Accidental suffocation and strangulation in bed	V01.Y98	Poland	15.11	1	yes
W75	Accidental suffocation and strangulation in bed	V01.Y98	United Kingdom	15.78	2	yes

For peer review only

W76	Other accidental hanging and strangulation	V01.Y98	Belgium	6.05	1	yes
W76	Other accidental hanging and strangulation	V01.Y98	Finland	5.94	0	no
W76	Other accidental hanging and strangulation	V01.Y98	France	21.56	0	no
W76	Other accidental hanging and strangulation	V01.Y98	Germany	30.64	0	no
W76	Other accidental hanging and strangulation	V01.Y98	Netherlands	10.55	0	no
W76	Other accidental hanging and strangulation	V01.Y98	Poland	21.81	1	yes
W76	Other accidental hanging and strangulation	V01.Y98	Romania	7.86	2	yes
W76	Other accidental hanging and strangulation	V01.Y98	Spain	10.39	3	yes
W76	Other accidental hanging and strangulation	V01.Y98	Switzerland	5.04	0	no
W76	Other accidental hanging and strangulation	V01.Y98	United Kingdom	148.13	0	no
W77	Threat to breathing due to cave-in, falling earth and other substances	V01.Y98	Czech Republic	10.22	0	no
W77	Threat to breathing due to cave-in, falling earth and other substances	V01.Y98	France	13.98	0	no
W77	Threat to breathing due to cave-in, falling earth and other substances	V01.Y98	Germany	8.79	1	yes
W77	Threat to breathing due to cave-in, falling earth and other substances	V01.Y98	Hungary	5.08	0	no
W77	Threat to breathing due to cave-in, falling earth and other substances	V01.Y98	Poland	26.06	1	yes
W77	Threat to breathing due to cave-in, falling earth and other substances	V01.Y98	Spain	9.05	1	yes
W78	Inhalation of gastric contents	V01.Y98	Austria	44.98	3	yes
W78	Inhalation of gastric contents	V01.Y98	Belgium	52.06	0	no
W78	Inhalation of gastric contents	V01.Y98	Croatia	8.56	1	yes
W78	Inhalation of gastric contents	V01.Y98	Czech Republic	209.53	2	yes
W78	Inhalation of gastric contents	V01.Y98	Denmark	8.99	1	yes
W78	Inhalation of gastric contents	V01.Y98	Estonia	56.35	3	yes
W78	Inhalation of gastric contents	V01.Y98	Finland	14.44	3	yes
W78	Inhalation of gastric contents	V01.Y98	France	178.52	2	yes
W78	Inhalation of gastric contents	V01.Y98	Germany	556.89	3	yes
W78	Inhalation of gastric contents	V01.Y98	Hungary	123.58	2	yes
W78	Inhalation of gastric contents	V01.Y98	Latvia	18.15	1	yes
W78	Inhalation of gastric contents	V01.Y98	Lithuania	96.89	1	yes
W78	Inhalation of gastric contents	V01.Y98	Netherlands	9.67	0	no
W78	Inhalation of gastric contents	V01.Y98	Norway	5.55	0	no
W78	Inhalation of gastric contents	V01.Y98	Poland	237.04	2	yes
W78	Inhalation of gastric contents	V01.Y98	Romania	270.79	1	yes
W78	Inhalation of gastric contents	V01.Y98	Slovenia	7.71	2	yes
W78	Inhalation of gastric contents	V01.Y98	Spain	93.20	2	yes
W78	Inhalation of gastric contents	V01.Y98	Sweden	9.96	0	no
W78	Inhalation of gastric contents	V01.Y98	Switzerland	24.07	3	yes
W78	Inhalation of gastric contents	V01.Y98	United Kingdom	129.10	1	yes
W79	Inhalation and ingestion of food causing obstruction of respiratory tract	V01.Y98	Austria	55.99	1	yes
W79	Inhalation and ingestion of food causing obstruction of respiratory tract	V01.Y98	Belgium	266.75	2	yes
W79	Inhalation and ingestion of food causing obstruction of respiratory tract	V01.Y98	Croatia	34.69	0	no
W79	Inhalation and ingestion of food causing obstruction of respiratory tract	V01.Y98	Czech Republic	38.30	2	yes
W79	Inhalation and ingestion of food causing obstruction of respiratory tract	V01.Y98	Denmark	27.98	1	yes

For peer review only

W79	Inhalation and ingestion of food causing obstruction of respiratory tract	V01.Y98	Estonia	20.05	1	yes
W79	Inhalation and ingestion of food causing obstruction of respiratory tract	V01.Y98	Finland	69.98	1	yes
W79	Inhalation and ingestion of food causing obstruction of respiratory tract	V01.Y98	France	2820.38	3	yes
W79	Inhalation and ingestion of food causing obstruction of respiratory tract	V01.Y98	Germany	538.70	2	yes
W79	Inhalation and ingestion of food causing obstruction of respiratory tract	V01.Y98	Hungary	139.78	1	yes
W79	Inhalation and ingestion of food causing obstruction of respiratory tract	V01.Y98	Latvia	34.80	2	yes
W79	Inhalation and ingestion of food causing obstruction of respiratory tract	V01.Y98	Lithuania	103.68	3	yes
W79	Inhalation and ingestion of food causing obstruction of respiratory tract	V01.Y98	Netherlands	62.94	0	no
W79	Inhalation and ingestion of food causing obstruction of respiratory tract	V01.Y98	Norway	13.85	0	no
W79	Inhalation and ingestion of food causing obstruction of respiratory tract	V01.Y98	Poland	368.48	2	yes
W79	Inhalation and ingestion of food causing obstruction of respiratory tract	V01.Y98	Romania	316.58	1	yes
W79	Inhalation and ingestion of food causing obstruction of respiratory tract	V01.Y98	Slovenia	24.73	1	yes
W79	Inhalation and ingestion of food causing obstruction of respiratory tract	V01.Y98	Spain	166.13	1	yes
W79	Inhalation and ingestion of food causing obstruction of respiratory tract	V01.Y98	Sweden	36.27	1	yes
W79	Inhalation and ingestion of food causing obstruction of respiratory tract	V01.Y98	Switzerland	67.33	0	no
W79	Inhalation and ingestion of food causing obstruction of respiratory tract	V01.Y98	United Kingdom	227.03	0	no
W80	Inhalation and ingestion of other objects causing obstruction of respiratory tract	V01.Y98	Belgium	19.76	0	no
W80	Inhalation and ingestion of other objects causing obstruction of respiratory tract	V01.Y98	Czech Republic	16.58	1	yes
W80	Inhalation and ingestion of other objects causing obstruction of respiratory tract	V01.Y98	France	77.21	3	yes
W80	Inhalation and ingestion of other objects causing obstruction of respiratory tract	V01.Y98	Germany	84.91	2	yes
W80	Inhalation and ingestion of other objects causing obstruction of respiratory tract	V01.Y98	Hungary	26.78	0	no
W80	Inhalation and ingestion of other objects causing obstruction of respiratory tract	V01.Y98	Latvia	10.48	3	yes
W80	Inhalation and ingestion of other objects causing obstruction of respiratory tract	V01.Y98	Netherlands	6.18	0	no
W80	Inhalation and ingestion of other objects causing obstruction of respiratory tract	V01.Y98	Norway	22.67	2	yes
W80	Inhalation and ingestion of other objects causing obstruction of respiratory tract	V01.Y98	Poland	36.88	1	yes
W80	Inhalation and ingestion of other objects causing obstruction of respiratory tract	V01.Y98	Romania	33.44	1	yes
W80	Inhalation and ingestion of other objects causing obstruction of respiratory tract	V01.Y98	Spain	777.35	1	yes
W80	Inhalation and ingestion of other objects causing obstruction of respiratory tract	V01.Y98	Sweden	31.09	0	no
W80	Inhalation and ingestion of other objects causing obstruction of respiratory tract	V01.Y98	Switzerland	25.13	0	no
W80	Inhalation and ingestion of other objects causing obstruction of respiratory tract	V01.Y98	United Kingdom	37.86	0	no
W83	Other specified threats to breathing	V01.Y98	Czech Republic	5.60	0	no

For peer review only

W83	Other specified threats to breathing	V01.Y98	France	7.27	0	no
W83	Other specified threats to breathing	V01.Y98	Germany	23.57	0	no
W83	Other specified threats to breathing	V01.Y98	United Kingdom	7.47	0	no
W84	Unspecified threat to breathing	V01.Y98	Belgium	114.75	1	yes
W84	Unspecified threat to breathing	V01.Y98	Czech Republic	8.55	0	no
W84	Unspecified threat to breathing	V01.Y98	Denmark	5.10	0	no
W84	Unspecified threat to breathing	V01.Y98	France	31.98	0	no
W84	Unspecified threat to breathing	V01.Y98	Germany	208.18	2	yes
W84	Unspecified threat to breathing	V01.Y98	Hungary	5.35	2	yes
W84	Unspecified threat to breathing	V01.Y98	Netherlands	44.59	2	yes
W84	Unspecified threat to breathing	V01.Y98	Norway	7.31	2	yes
W84	Unspecified threat to breathing	V01.Y98	Poland	20.83	1	yes
W84	Unspecified threat to breathing	V01.Y98	Romania	21.94	1	yes
W84	Unspecified threat to breathing	V01.Y98	Spain	728.81	3	yes
W84	Unspecified threat to breathing	V01.Y98	Switzerland	5.00	1	yes
W84	Unspecified threat to breathing	V01.Y98	United Kingdom	62.23	1	yes
W85	Exposure to electric transmission lines	V01.Y98	Czech Republic	6.44	1	yes
W85	Exposure to electric transmission lines	V01.Y98	Germany	11.27	1	yes
W85	Exposure to electric transmission lines	V01.Y98	Hungary	6.78	1	yes
W85	Exposure to electric transmission lines	V01.Y98	Lithuania	12.12	1	yes
W85	Exposure to electric transmission lines	V01.Y98	Poland	10.44	2	yes
W85	Exposure to electric transmission lines	V01.Y98	Romania	13.13	1	yes
W86	Exposure to other specified electric current	V01.Y98	Germany	14.94	2	yes
W86	Exposure to other specified electric current	V01.Y98	Hungary	11.60	1	yes
W86	Exposure to other specified electric current	V01.Y98	Lithuania	5.06	1	yes
W86	Exposure to other specified electric current	V01.Y98	Poland	37.56	0	no
W86	Exposure to other specified electric current	V01.Y98	United Kingdom	14.82	1	yes
W87	Exposure to unspecified electric current	V01.Y98	Belgium	6.24	0	no
W87	Exposure to unspecified electric current	V01.Y98	Croatia	10.14	3	yes
W87	Exposure to unspecified electric current	V01.Y98	Czech Republic	15.75	1	yes
W87	Exposure to unspecified electric current	V01.Y98	France	48.62	1	yes
W87	Exposure to unspecified electric current	V01.Y98	Germany	27.39	1	yes
W87	Exposure to unspecified electric current	V01.Y98	Hungary	21.45	3	yes
W87	Exposure to unspecified electric current	V01.Y98	Lithuania	5.02	0	no
W87	Exposure to unspecified electric current	V01.Y98	Poland	77.38	1	yes
W87	Exposure to unspecified electric current	V01.Y98	Romania	181.31	1	yes
W87	Exposure to unspecified electric current	V01.Y98	Spain	50.44	1	yes
W87	Exposure to unspecified electric current	V01.Y98	United Kingdom	9.58	3	yes
W89	Exposure to man-made visible and ultraviolet light	V01.Y98	Romania	7.41	2	yes
W92	Exposure to excessive heat of man-made origin	V01.Y98	Finland	40.38	0	no
W92	Exposure to excessive heat of man-made origin	V01.Y98	Germany	5.10	1	yes
W92	Exposure to excessive heat of man-made origin	V01.Y98	Poland	15.37	1	yes
W93	Exposure to excessive cold of man-made origin	V01.Y98	Romania	13.92	0	no
X00	Exposure to uncontrolled fire in building or structure	V01.Y98	Austria	26.40	0	no
X00	Exposure to uncontrolled fire in building or structure	V01.Y98	Belgium	63.38	1	yes
X00	Exposure to uncontrolled fire in building or structure	V01.Y98	Croatia	14.33	0	no
X00	Exposure to uncontrolled fire in building or structure	V01.Y98	Czech Republic	12.10	2	yes
X00	Exposure to uncontrolled fire in building or structure	V01.Y98	Denmark	19.45	0	no
X00	Exposure to uncontrolled fire in building or structure	V01.Y98	Estonia	88.97	3	yes
X00	Exposure to uncontrolled fire in building or structure	V01.Y98	Finland	66.88	0	no

For peer review only

X00	Exposure to uncontrolled fire in building or structure	V01.Y98	France	139.77	3	yes
X00	Exposure to uncontrolled fire in building or structure	V01.Y98	Germany	227.80	1	yes
X00	Exposure to uncontrolled fire in building or structure	V01.Y98	Hungary	61.49	2	yes
X00	Exposure to uncontrolled fire in building or structure	V01.Y98	Latvia	140.85	1	yes
X00	Exposure to uncontrolled fire in building or structure	V01.Y98	Lithuania	73.06	1	yes
X00	Exposure to uncontrolled fire in building or structure	V01.Y98	Netherlands	31.77	0	no
X00	Exposure to uncontrolled fire in building or structure	V01.Y98	Norway	48.37	2	yes
X00	Exposure to uncontrolled fire in building or structure	V01.Y98	Poland	359.48	2	yes
X00	Exposure to uncontrolled fire in building or structure	V01.Y98	Romania	19.47	0	no
X00	Exposure to uncontrolled fire in building or structure	V01.Y98	Slovenia	5.60	3	yes
X00	Exposure to uncontrolled fire in building or structure	V01.Y98	Spain	53.81	3	yes
X00	Exposure to uncontrolled fire in building or structure	V01.Y98	Sweden	65.40	3	yes
X00	Exposure to uncontrolled fire in building or structure	V01.Y98	Switzerland	12.15	2	yes
X00	Exposure to uncontrolled fire in building or structure	V01.Y98	United Kingdom	261.50	1	yes
X01	Exposure to uncontrolled fire, not in building or structure	V01.Y98	Poland	10.90	0	no
X02	Exposure to controlled fire in building or structure	V01.Y98	Croatia	5.37	0	no
X02	Exposure to controlled fire in building or structure	V01.Y98	France	6.80	1	yes
X02	Exposure to controlled fire in building or structure	V01.Y98	Germany	9.57	0	no
X02	Exposure to controlled fire in building or structure	V01.Y98	Hungary	12.13	3	yes
X02	Exposure to controlled fire in building or structure	V01.Y98	Poland	27.55	3	yes
X02	Exposure to controlled fire in building or structure	V01.Y98	Romania	28.82	1	yes
X02	Exposure to controlled fire in building or structure	V01.Y98	Spain	8.75	1	yes
X02	Exposure to controlled fire in building or structure	V01.Y98	United Kingdom	7.18	0	no
X03	Exposure to controlled fire, not in building or structure	V01.Y98	Hungary	5.13	0	no
X04	Exposure to ignition of highly flammable material	V01.Y98	Germany	5.61	2	yes
X04	Exposure to ignition of highly flammable material	V01.Y98	Poland	11.65	1	yes
X04	Exposure to ignition of highly flammable material	V01.Y98	United Kingdom	18.64	1	yes
X05	Exposure to ignition or melting of nightwear	V01.Y98	Hungary	6.36	2	yes
X06	Exposure to ignition or melting of other clothing and apparel	V01.Y98	Germany	7.55	0	no
X06	Exposure to ignition or melting of other clothing and apparel	V01.Y98	Hungary	25.48	1	yes
X06	Exposure to ignition or melting of other clothing and apparel	V01.Y98	Latvia	5.76	1	yes
X06	Exposure to ignition or melting of other clothing and apparel	V01.Y98	Poland	20.67	0	no
X06	Exposure to ignition or melting of other clothing and apparel	V01.Y98	United Kingdom	18.16	0	no
X08	Exposure to other specified smoke, fire and flames	V01.Y98	Denmark	27.31	0	no
X08	Exposure to other specified smoke, fire and flames	V01.Y98	Germany	20.02	1	yes
X08	Exposure to other specified smoke, fire and flames	V01.Y98	Hungary	11.93	0	no
X08	Exposure to other specified smoke, fire and flames	V01.Y98	Latvia	8.66	0	no
X08	Exposure to other specified smoke, fire and flames	V01.Y98	Lithuania	11.91	0	no
X08	Exposure to other specified smoke, fire and flames	V01.Y98	Poland	23.80	1	yes
X08	Exposure to other specified smoke, fire and flames	V01.Y98	Romania	63.66	0	no
X08	Exposure to other specified smoke, fire and flames	V01.Y98	United Kingdom	19.11	0	no
X09	Exposure to unspecified smoke, fire and flames	V01.Y98	Belgium	22.65	1	yes
X09	Exposure to unspecified smoke, fire and flames	V01.Y98	Croatia	22.93	3	yes
X09	Exposure to unspecified smoke, fire and flames	V01.Y98	Czech Republic	39.88	1	yes
X09	Exposure to unspecified smoke, fire and flames	V01.Y98	Denmark	13.33	0	no

For peer review only

X09	Exposure to unspecified smoke, fire and flames	V01.Y98	Estonia	7.99	2	yes
X09	Exposure to unspecified smoke, fire and flames	V01.Y98	France	334.22	3	yes
X09	Exposure to unspecified smoke, fire and flames	V01.Y98	Germany	143.28	1	yes
X09	Exposure to unspecified smoke, fire and flames	V01.Y98	Hungary	43.40	1	yes
X09	Exposure to unspecified smoke, fire and flames	V01.Y98	Latvia	11.88	1	yes
X09	Exposure to unspecified smoke, fire and flames	V01.Y98	Lithuania	18.94	2	yes
X09	Exposure to unspecified smoke, fire and flames	V01.Y98	Netherlands	11.52	1	yes
X09	Exposure to unspecified smoke, fire and flames	V01.Y98	Poland	139.66	1	yes
X09	Exposure to unspecified smoke, fire and flames	V01.Y98	Romania	365.66	1	yes
X09	Exposure to unspecified smoke, fire and flames	V01.Y98	Spain	119.63	1	yes
X09	Exposure to unspecified smoke, fire and flames	V01.Y98	Sweden	5.72	0	no
X09	Exposure to unspecified smoke, fire and flames	V01.Y98	Switzerland	14.19	0	no
X09	Exposure to unspecified smoke, fire and flames	V01.Y98	United Kingdom	33.54	2	yes
X11	Contact with hot tap-water	V01.Y98	France	5.31	1	yes
X11	Contact with hot tap-water	V01.Y98	Germany	14.10	1	yes
X11	Contact with hot tap-water	V01.Y98	Hungary	14.61	1	yes
X11	Contact with hot tap-water	V01.Y98	United Kingdom	22.90	0	no
X12	Contact with other hot fluids	V01.Y98	Hungary	5.14	0	no
X12	Contact with other hot fluids	V01.Y98	Poland	5.59	0	no
X12	Contact with other hot fluids	V01.Y98	Romania	19.55	0	no
X16	Contact with hot heating appliances, radiators and pipes	V01.Y98	United Kingdom	6.35	2	yes
X19	Contact with other and unspecified heat and hot substances	V01.Y98	Czech Republic	7.73	1	yes
X19	Contact with other and unspecified heat and hot substances	V01.Y98	Germany	8.68	1	yes
X19	Contact with other and unspecified heat and hot substances	V01.Y98	Poland	52.42	1	yes
X19	Contact with other and unspecified heat and hot substances	V01.Y98	Romania	26.12	1	yes
X23	Contact with hornets, wasps and bees	V01.Y98	France	15.47	0	no
X23	Contact with hornets, wasps and bees	V01.Y98	Germany	17.57	0	no
X23	Contact with hornets, wasps and bees	V01.Y98	Hungary	6.01	1	yes
X23	Contact with hornets, wasps and bees	V01.Y98	Poland	7.86	0	no
X23	Contact with hornets, wasps and bees	V01.Y98	Romania	8.74	0	no
X30	Exposure to excessive natural heat	V01.Y98	Belgium	19.96	0	no
X30	Exposure to excessive natural heat	V01.Y98	France	192.36	0	no
X30	Exposure to excessive natural heat	V01.Y98	Germany	13.33	0	no
X30	Exposure to excessive natural heat	V01.Y98	Poland	6.81	0	no
X30	Exposure to excessive natural heat	V01.Y98	Romania	5.65	0	no
X30	Exposure to excessive natural heat	V01.Y98	Spain	46.43	0	no
X31	Exposure to excessive natural cold	V01.Y98	Austria	31.12	2	yes
X31	Exposure to excessive natural cold	V01.Y98	Belgium	15.09	0	no
X31	Exposure to excessive natural cold	V01.Y98	Croatia	30.45	0	no
X31	Exposure to excessive natural cold	V01.Y98	Czech Republic	125.84	2	yes
X31	Exposure to excessive natural cold	V01.Y98	Denmark	13.52	0	no
X31	Exposure to excessive natural cold	V01.Y98	Estonia	110.87	2	yes
X31	Exposure to excessive natural cold	V01.Y98	Finland	75.26	2	yes
X31	Exposure to excessive natural cold	V01.Y98	France	168.94	1	yes
X31	Exposure to excessive natural cold	V01.Y98	Germany	119.32	1	yes
X31	Exposure to excessive natural cold	V01.Y98	Hungary	251.78	0	no
X31	Exposure to excessive natural cold	V01.Y98	Latvia	209.94	1	yes

For peer review only

X31	Exposure to excessive natural cold	V01.Y98	Lithuania	338.59	2	yes
X31	Exposure to excessive natural cold	V01.Y98	Netherlands	7.42	0	no
X31	Exposure to excessive natural cold	V01.Y98	Norway	21.84	0	no
X31	Exposure to excessive natural cold	V01.Y98	Poland	421.03	0	no
X31	Exposure to excessive natural cold	V01.Y98	Romania	409.72	1	yes
X31	Exposure to excessive natural cold	V01.Y98	Slovenia	22.91	1	yes
X31	Exposure to excessive natural cold	V01.Y98	Spain	21.23	1	yes
X31	Exposure to excessive natural cold	V01.Y98	Sweden	45.23	1	yes
X31	Exposure to excessive natural cold	V01.Y98	Switzerland	6.73	1	yes
X31	Exposure to excessive natural cold	V01.Y98	United Kingdom	140.59	3	yes
X33	Victim of lightning	V01.Y98	Poland	7.08	2	yes
X33	Victim of lightning	V01.Y98	Romania	38.50	1	yes
X36	Victim of avalanche, landslide and other earth movements	V01.Y98	Austria	13.11	0	no
X36	Victim of avalanche, landslide and other earth movements	V01.Y98	France	22.60	0	no
X36	Victim of avalanche, landslide and other earth movements	V01.Y98	Norway	5.72	0	no
X36	Victim of avalanche, landslide and other earth movements	V01.Y98	Switzerland	13.42	3	yes
X40	Accidental poisoning by and exposure to nonopioid analgesics, antipyretics and antirheumatics	V01.Y98	Denmark	6.23	0	no
X40	Accidental poisoning by and exposure to nonopioid analgesics, antipyretics and antirheumatics	V01.Y98	Finland	17.92	2	yes
X40	Accidental poisoning by and exposure to nonopioid analgesics, antipyretics and antirheumatics	V01.Y98	France	16.91	1	yes
X40	Accidental poisoning by and exposure to nonopioid analgesics, antipyretics and antirheumatics	V01.Y98	Germany	5.70	0	no
X40	Accidental poisoning by and exposure to nonopioid analgesics, antipyretics and antirheumatics	V01.Y98	Romania	6.64	0	no
X40	Accidental poisoning by and exposure to nonopioid analgesics, antipyretics and antirheumatics	V01.Y98	Sweden	5.75	2	yes
X40	Accidental poisoning by and exposure to nonopioid analgesics, antipyretics and antirheumatics	V01.Y98	United Kingdom	64.78	0	no
X41	Accidental poisoning by and exposure to antiepileptic, sedative-hypnotic, antiparkinsonism and psychotropic drugs, not elsewhere classified	V01.Y98	Belgium	14.08	0	no
X41	Accidental poisoning by and exposure to antiepileptic, sedative-hypnotic, antiparkinsonism and psychotropic drugs, not elsewhere classified	V01.Y98	Czech Republic	25.73	2	yes
X41	Accidental poisoning by and exposure to antiepileptic, sedative-hypnotic, antiparkinsonism and psychotropic drugs, not elsewhere classified	V01.Y98	Denmark	12.84	0	no
X41	Accidental poisoning by and exposure to antiepileptic, sedative-hypnotic, antiparkinsonism and psychotropic drugs, not elsewhere classified	V01.Y98	Estonia	6.60	1	yes
X41	Accidental poisoning by and exposure to antiepileptic, sedative-hypnotic, antiparkinsonism and psychotropic drugs, not elsewhere classified	V01.Y98	Finland	105.72	2	yes
X41	Accidental poisoning by and exposure to antiepileptic, sedative-hypnotic, antiparkinsonism and psychotropic drugs, not elsewhere classified	V01.Y98	France	77.96	1	yes
X41	Accidental poisoning by and exposure to antiepileptic, sedative-hypnotic, antiparkinsonism and psychotropic drugs, not elsewhere classified	V01.Y98	Germany	39.13	0	no
X41	Accidental poisoning by and exposure to antiepileptic, sedative-hypnotic, antiparkinsonism and psychotropic drugs, not elsewhere classified	V01.Y98	Latvia	6.29	0	no

For peer review only

X41	Accidental poisoning by and exposure to antiepileptic, sedative-hypnotic, antiparkinsonism and psychotropic drugs, not elsewhere classified	V01.Y98	Lithuania	7.61	2	yes
X41	Accidental poisoning by and exposure to antiepileptic, sedative-hypnotic, antiparkinsonism and psychotropic drugs, not elsewhere classified	V01.Y98	Netherlands	7.51	3	yes
X41	Accidental poisoning by and exposure to antiepileptic, sedative-hypnotic, antiparkinsonism and psychotropic drugs, not elsewhere classified	V01.Y98	Norway	41.04	0	no
X41	Accidental poisoning by and exposure to antiepileptic, sedative-hypnotic, antiparkinsonism and psychotropic drugs, not elsewhere classified	V01.Y98	Poland	16.00	1	yes
X41	Accidental poisoning by and exposure to antiepileptic, sedative-hypnotic, antiparkinsonism and psychotropic drugs, not elsewhere classified	V01.Y98	Romania	11.44	2	yes
X41	Accidental poisoning by and exposure to antiepileptic, sedative-hypnotic, antiparkinsonism and psychotropic drugs, not elsewhere classified	V01.Y98	Spain	35.51	0	no
X41	Accidental poisoning by and exposure to antiepileptic, sedative-hypnotic, antiparkinsonism and psychotropic drugs, not elsewhere classified	V01.Y98	Sweden	49.03	1	yes
X41	Accidental poisoning by and exposure to antiepileptic, sedative-hypnotic, antiparkinsonism and psychotropic drugs, not elsewhere classified	V01.Y98	Switzerland	8.76	0	no
X41	Accidental poisoning by and exposure to antiepileptic, sedative-hypnotic, antiparkinsonism and psychotropic drugs, not elsewhere classified	V01.Y98	United Kingdom	205.39	2	yes
X42	Accidental poisoning by and exposure to narcotics and psychodysleptics [hallucinogens], not elsewhere classified	V01.Y98	Belgium	48.88	2	yes
X42	Accidental poisoning by and exposure to narcotics and psychodysleptics [hallucinogens], not elsewhere classified	V01.Y98	Croatia	43.83	2	yes
X42	Accidental poisoning by and exposure to narcotics and psychodysleptics [hallucinogens], not elsewhere classified	V01.Y98	Czech Republic	10.39	0	no
X42	Accidental poisoning by and exposure to narcotics and psychodysleptics [hallucinogens], not elsewhere classified	V01.Y98	Denmark	82.22	0	no
X42	Accidental poisoning by and exposure to narcotics and psychodysleptics [hallucinogens], not elsewhere classified	V01.Y98	Estonia	72.87	1	yes
X42	Accidental poisoning by and exposure to narcotics and psychodysleptics [hallucinogens], not elsewhere classified	V01.Y98	Finland	96.52	3	yes
X42	Accidental poisoning by and exposure to narcotics and psychodysleptics [hallucinogens], not elsewhere classified	V01.Y98	France	119.27	1	yes
X42	Accidental poisoning by and exposure to narcotics and psychodysleptics [hallucinogens], not elsewhere classified	V01.Y98	Germany	256.10	1	yes
X42	Accidental poisoning by and exposure to narcotics and psychodysleptics [hallucinogens], not elsewhere classified	V01.Y98	Hungary	17.22	0	no
X42	Accidental poisoning by and exposure to narcotics and psychodysleptics [hallucinogens], not elsewhere classified	V01.Y98	Latvia	12.46	2	yes
X42	Accidental poisoning by and exposure to narcotics and psychodysleptics [hallucinogens], not elsewhere classified	V01.Y98	Lithuania	44.43	3	yes

For peer review only

X42	Accidental poisoning by and exposure to narcotics and psychodysleptics [hallucinogens], not elsewhere classified	V01.Y98	Netherlands	74.50	0	no
X42	Accidental poisoning by and exposure to narcotics and psychodysleptics [hallucinogens], not elsewhere classified	V01.Y98	Norway	144.53	3	yes
X42	Accidental poisoning by and exposure to narcotics and psychodysleptics [hallucinogens], not elsewhere classified	V01.Y98	Poland	29.02	2	yes
X42	Accidental poisoning by and exposure to narcotics and psychodysleptics [hallucinogens], not elsewhere classified	V01.Y98	Slovenia	13.27	1	yes
X42	Accidental poisoning by and exposure to narcotics and psychodysleptics [hallucinogens], not elsewhere classified	V01.Y98	Spain	157.26	3	yes
X42	Accidental poisoning by and exposure to narcotics and psychodysleptics [hallucinogens], not elsewhere classified	V01.Y98	Sweden	101.26	2	yes
X42	Accidental poisoning by and exposure to narcotics and psychodysleptics [hallucinogens], not elsewhere classified	V01.Y98	Switzerland	52.35	2	yes
X42	Accidental poisoning by and exposure to narcotics and psychodysleptics [hallucinogens], not elsewhere classified	V01.Y98	United Kingdom	813.54	3	yes
X43	Accidental poisoning by and exposure to other drugs acting on the autonomic nervous system	V01.Y98	Finland	5.40	2	yes
X43	Accidental poisoning by and exposure to other drugs acting on the autonomic nervous system	V01.Y98	France	12.71	1	yes
X43	Accidental poisoning by and exposure to other drugs acting on the autonomic nervous system	V01.Y98	United Kingdom	6.22	2	yes
X44	Accidental poisoning by and exposure to other and unspecified drugs, medicaments and biological substances	V01.Y98	Austria	7.68	2	yes
X44	Accidental poisoning by and exposure to other and unspecified drugs, medicaments and biological substances	V01.Y98	Belgium	51.25	0	no
X44	Accidental poisoning by and exposure to other and unspecified drugs, medicaments and biological substances	V01.Y98	Croatia	11.98	3	yes
X44	Accidental poisoning by and exposure to other and unspecified drugs, medicaments and biological substances	V01.Y98	Czech Republic	29.82	2	yes
X44	Accidental poisoning by and exposure to other and unspecified drugs, medicaments and biological substances	V01.Y98	Denmark	50.34	1	yes
X44	Accidental poisoning by and exposure to other and unspecified drugs, medicaments and biological substances	V01.Y98	Finland	17.95	2	yes
X44	Accidental poisoning by and exposure to other and unspecified drugs, medicaments and biological substances	V01.Y98	France	829.25	3	yes
X44	Accidental poisoning by and exposure to other and unspecified drugs, medicaments and biological substances	V01.Y98	Germany	175.14	3	yes
X44	Accidental poisoning by and exposure to other and unspecified drugs, medicaments and biological substances	V01.Y98	Hungary	6.38	0	no
X44	Accidental poisoning by and exposure to other and unspecified drugs, medicaments and biological substances	V01.Y98	Latvia	7.01	3	yes

For peer review only

X44	Accidental poisoning by and exposure to other and unspecified drugs, medicaments and biological substances	V01.Y98	Lithuania	6.04	0	no
X44	Accidental poisoning by and exposure to other and unspecified drugs, medicaments and biological substances	V01.Y98	Netherlands	21.23	0	no
X44	Accidental poisoning by and exposure to other and unspecified drugs, medicaments and biological substances	V01.Y98	Norway	63.24	0	no
X44	Accidental poisoning by and exposure to other and unspecified drugs, medicaments and biological substances	V01.Y98	Poland	42.37	1	yes
X44	Accidental poisoning by and exposure to other and unspecified drugs, medicaments and biological substances	V01.Y98	Romania	27.65	0	no
X44	Accidental poisoning by and exposure to other and unspecified drugs, medicaments and biological substances	V01.Y98	Spain	309.34	1	yes
X44	Accidental poisoning by and exposure to other and unspecified drugs, medicaments and biological substances	V01.Y98	Sweden	86.53	2	yes
X44	Accidental poisoning by and exposure to other and unspecified drugs, medicaments and biological substances	V01.Y98	Switzerland	26.76	3	yes
X44	Accidental poisoning by and exposure to other and unspecified drugs, medicaments and biological substances	V01.Y98	United Kingdom	367.64	3	yes
X45	Accidental poisoning by and exposure to alcohol	V01.Y98	Belgium	23.33	1	yes
X45	Accidental poisoning by and exposure to alcohol	V01.Y98	Croatia	17.65	2	yes
X45	Accidental poisoning by and exposure to alcohol	V01.Y98	Czech Republic	171.12	1	yes
X45	Accidental poisoning by and exposure to alcohol	V01.Y98	Denmark	12.63	0	no
X45	Accidental poisoning by and exposure to alcohol	V01.Y98	Estonia	159.53	2	yes
X45	Accidental poisoning by and exposure to alcohol	V01.Y98	Finland	416.69	2	yes
X45	Accidental poisoning by and exposure to alcohol	V01.Y98	France	181.98	3	yes
X45	Accidental poisoning by and exposure to alcohol	V01.Y98	Germany	137.35	1	yes
X45	Accidental poisoning by and exposure to alcohol	V01.Y98	Hungary	23.73	0	no
X45	Accidental poisoning by and exposure to alcohol	V01.Y98	Latvia	173.66	3	yes
X45	Accidental poisoning by and exposure to alcohol	V01.Y98	Lithuania	383.90	3	yes
X45	Accidental poisoning by and exposure to alcohol	V01.Y98	Netherlands	15.19	0	no
X45	Accidental poisoning by and exposure to alcohol	V01.Y98	Norway	40.24	0	no
X45	Accidental poisoning by and exposure to alcohol	V01.Y98	Poland	1210.37	2	yes
X45	Accidental poisoning by and exposure to alcohol	V01.Y98	Romania	321.12	2	yes
X45	Accidental poisoning by and exposure to alcohol	V01.Y98	Slovenia	17.83	3	yes
X45	Accidental poisoning by and exposure to alcohol	V01.Y98	Spain	40.06	2	yes
X45	Accidental poisoning by and exposure to alcohol	V01.Y98	Sweden	114.62	2	yes
X45	Accidental poisoning by and exposure to alcohol	V01.Y98	Switzerland	11.17	1	yes
X45	Accidental poisoning by and exposure to alcohol	V01.Y98	United Kingdom	319.10	1	yes
X46	Accidental poisoning by and exposure to organic solvents and halogenated hydrocarbons and their vapours	V01.Y98	Czech Republic	7.75	2	yes
X46	Accidental poisoning by and exposure to organic solvents and halogenated hydrocarbons and their vapours	V01.Y98	Finland	6.67	1	yes
X46	Accidental poisoning by and exposure to organic solvents and halogenated hydrocarbons and their vapours	V01.Y98	France	5.10	2	yes

For peer review only

X46	Accidental poisoning by and exposure to organic solvents and halogenated hydrocarbons and their vapours	V01.Y98	Hungary	8.83	0	no
X46	Accidental poisoning by and exposure to organic solvents and halogenated hydrocarbons and their vapours	V01.Y98	Latvia	6.82	0	no
X46	Accidental poisoning by and exposure to organic solvents and halogenated hydrocarbons and their vapours	V01.Y98	Poland	14.76	1	yes
X46	Accidental poisoning by and exposure to organic solvents and halogenated hydrocarbons and their vapours	V01.Y98	Romania	12.29	0	no
X46	Accidental poisoning by and exposure to organic solvents and halogenated hydrocarbons and their vapours	V01.Y98	United Kingdom	5.80	1	yes
X47	Accidental poisoning by and exposure to other gases and vapours	V01.Y98	Austria	14.70	2	yes
X47	Accidental poisoning by and exposure to other gases and vapours	V01.Y98	Belgium	34.65	1	yes
X47	Accidental poisoning by and exposure to other gases and vapours	V01.Y98	Croatia	22.99	1	yes
X47	Accidental poisoning by and exposure to other gases and vapours	V01.Y98	Czech Republic	79.62	3	yes
X47	Accidental poisoning by and exposure to other gases and vapours	V01.Y98	Estonia	8.74	2	yes
X47	Accidental poisoning by and exposure to other gases and vapours	V01.Y98	Finland	10.84	0	no
X47	Accidental poisoning by and exposure to other gases and vapours	V01.Y98	France	100.52	1	yes
X47	Accidental poisoning by and exposure to other gases and vapours	V01.Y98	Germany	49.94	1	yes
X47	Accidental poisoning by and exposure to other gases and vapours	V01.Y98	Hungary	63.27	3	yes
X47	Accidental poisoning by and exposure to other gases and vapours	V01.Y98	Latvia	39.87	2	yes
X47	Accidental poisoning by and exposure to other gases and vapours	V01.Y98	Lithuania	150.13	2	yes
X47	Accidental poisoning by and exposure to other gases and vapours	V01.Y98	Netherlands	11.22	0	no
X47	Accidental poisoning by and exposure to other gases and vapours	V01.Y98	Poland	279.75	2	yes
X47	Accidental poisoning by and exposure to other gases and vapours	V01.Y98	Romania	491.00	2	yes
X47	Accidental poisoning by and exposure to other gases and vapours	V01.Y98	Slovenia	7.40	1	yes
X47	Accidental poisoning by and exposure to other gases and vapours	V01.Y98	Spain	82.15	1	yes
X47	Accidental poisoning by and exposure to other gases and vapours	V01.Y98	Sweden	5.43	0	no
X47	Accidental poisoning by and exposure to other gases and vapours	V01.Y98	Switzerland	5.60	0	no
X47	Accidental poisoning by and exposure to other gases and vapours	V01.Y98	United Kingdom	50.48	0	no
X48	Accidental poisoning by and exposure to pesticides	V01.Y98	Romania	33.57	1	yes
X48	Accidental poisoning by and exposure to pesticides	V01.Y98	Spain	9.17	1	yes
X49	Accidental poisoning by and exposure to other and unspecified chemicals and noxious substances	V01.Y98	Belgium	10.77	0	no
X49	Accidental poisoning by and exposure to other and unspecified chemicals and noxious substances	V01.Y98	Czech Republic	10.89	2	yes
X49	Accidental poisoning by and exposure to other and unspecified chemicals and noxious substances	V01.Y98	Estonia	5.17	3	yes

For peer review only

X49	Accidental poisoning by and exposure to other and unspecified chemicals and noxious substances	V01.Y98	France	104.64	2	yes
X49	Accidental poisoning by and exposure to other and unspecified chemicals and noxious substances	V01.Y98	Germany	39.90	1	yes
X49	Accidental poisoning by and exposure to other and unspecified chemicals and noxious substances	V01.Y98	Hungary	9.00	1	yes
X49	Accidental poisoning by and exposure to other and unspecified chemicals and noxious substances	V01.Y98	Latvia	24.24	2	yes
X49	Accidental poisoning by and exposure to other and unspecified chemicals and noxious substances	V01.Y98	Lithuania	18.32	1	yes
X49	Accidental poisoning by and exposure to other and unspecified chemicals and noxious substances	V01.Y98	Poland	34.40	1	yes
X49	Accidental poisoning by and exposure to other and unspecified chemicals and noxious substances	V01.Y98	Romania	172.62	2	yes
X49	Accidental poisoning by and exposure to other and unspecified chemicals and noxious substances	V01.Y98	Spain	107.89	2	yes
X49	Accidental poisoning by and exposure to other and unspecified chemicals and noxious substances	V01.Y98	United Kingdom	33.49	0	no
X50	Overexertion and strenuous or repetitive movements	V01.Y98	Belgium	13.20	0	no
X50	Overexertion and strenuous or repetitive movements	V01.Y98	France	238.84	1	yes
X50	Overexertion and strenuous or repetitive movements	V01.Y98	Germany	21.91	3	yes
X51	Travel and motion	V01.Y98	France	8.08	0	no
X53	Lack of food	V01.Y98	Germany	14.35	1	yes
X58	Exposure to other specified factors	V01.Y98	Germany	9.85	0	no
X58	Exposure to other specified factors	V01.Y98	Romania	9.17	1	yes
X59	Exposure to unspecified factor	V01.Y98	Austria	630.68	2	yes
X59	Exposure to unspecified factor	V01.Y98	Belgium	922.66	0	no
X59	Exposure to unspecified factor	V01.Y98	Croatia	113.70	0	no
X59	Exposure to unspecified factor	V01.Y98	Czech Republic	921.32	1	yes
X59	Exposure to unspecified factor	V01.Y98	Denmark	583.88	1	yes
X59	Exposure to unspecified factor	V01.Y98	Estonia	29.94	3	yes
X59	Exposure to unspecified factor	V01.Y98	Finland	30.43	1	yes
X59	Exposure to unspecified factor	V01.Y98	France	8813.06	2	yes
X59	Exposure to unspecified factor	V01.Y98	Germany	1576.29	2	yes
X59	Exposure to unspecified factor	V01.Y98	Hungary	100.83	0	no
X59	Exposure to unspecified factor	V01.Y98	Latvia	111.65	3	yes
X59	Exposure to unspecified factor	V01.Y98	Lithuania	62.39	1	yes
X59	Exposure to unspecified factor	V01.Y98	Netherlands	1084.27	3	yes
X59	Exposure to unspecified factor	V01.Y98	Norway	536.20	2	yes
X59	Exposure to unspecified factor	V01.Y98	Poland	968.34	3	yes
X59	Exposure to unspecified factor	V01.Y98	Romania	429.61	0	no
X59	Exposure to unspecified factor	V01.Y98	Slovenia	6.25	0	no
X59	Exposure to unspecified factor	V01.Y98	Spain	1957.12	0	no
X59	Exposure to unspecified factor	V01.Y98	Sweden	929.09	1	yes
X59	Exposure to unspecified factor	V01.Y98	Switzerland	111.33	1	yes
X59	Exposure to unspecified factor	V01.Y98	United Kingdom	3844.82	2	yes
X60	Intentional self-poisoning by and exposure to nonopioid analgesics, antipyretics and antirheumatics	V01.Y98	Czech Republic	5.18	0	no
X60	Intentional self-poisoning by and exposure to nonopioid analgesics, antipyretics and antirheumatics	V01.Y98	Denmark	17.21	1	yes
X60	Intentional self-poisoning by and exposure to nonopioid analgesics, antipyretics and antirheumatics	V01.Y98	Finland	12.08	2	yes

For peer review only

X60	Intentional self-poisoning by and exposure to nonopioid analgesics, antipyretics and antirheumatics	V01.Y98	France	8.87	0	no
X60	Intentional self-poisoning by and exposure to nonopioid analgesics, antipyretics and antirheumatics	V01.Y98	Germany	19.27	0	no
X60	Intentional self-poisoning by and exposure to nonopioid analgesics, antipyretics and antirheumatics	V01.Y98	Hungary	6.19	0	no
X60	Intentional self-poisoning by and exposure to nonopioid analgesics, antipyretics and antirheumatics	V01.Y98	Netherlands	9.41	0	no
X60	Intentional self-poisoning by and exposure to nonopioid analgesics, antipyretics and antirheumatics	V01.Y98	Norway	5.06	0	no
X60	Intentional self-poisoning by and exposure to nonopioid analgesics, antipyretics and antirheumatics	V01.Y98	Sweden	10.87	2	yes
X60	Intentional self-poisoning by and exposure to nonopioid analgesics, antipyretics and antirheumatics	V01.Y98	United Kingdom	84.69	1	yes
X61	Intentional self-poisoning by and exposure to antiepileptic, sedative-hypnotic, antiparkinsonism and psychotropic drugs, not elsewhere classified	V01.Y98	Austria	12.99	0	no
X61	Intentional self-poisoning by and exposure to antiepileptic, sedative-hypnotic, antiparkinsonism and psychotropic drugs, not elsewhere classified	V01.Y98	Belgium	51.29	3	yes
X61	Intentional self-poisoning by and exposure to antiepileptic, sedative-hypnotic, antiparkinsonism and psychotropic drugs, not elsewhere classified	V01.Y98	Croatia	11.17	0	no
X61	Intentional self-poisoning by and exposure to antiepileptic, sedative-hypnotic, antiparkinsonism and psychotropic drugs, not elsewhere classified	V01.Y98	Czech Republic	43.91	0	no
X61	Intentional self-poisoning by and exposure to antiepileptic, sedative-hypnotic, antiparkinsonism and psychotropic drugs, not elsewhere classified	V01.Y98	Denmark	31.91	2	yes
X61	Intentional self-poisoning by and exposure to antiepileptic, sedative-hypnotic, antiparkinsonism and psychotropic drugs, not elsewhere classified	V01.Y98	Finland	157.93	1	yes
X61	Intentional self-poisoning by and exposure to antiepileptic, sedative-hypnotic, antiparkinsonism and psychotropic drugs, not elsewhere classified	V01.Y98	France	210.28	1	yes
X61	Intentional self-poisoning by and exposure to antiepileptic, sedative-hypnotic, antiparkinsonism and psychotropic drugs, not elsewhere classified	V01.Y98	Germany	365.36	0	no
X61	Intentional self-poisoning by and exposure to antiepileptic, sedative-hypnotic, antiparkinsonism and psychotropic drugs, not elsewhere classified	V01.Y98	Hungary	155.25	0	no
X61	Intentional self-poisoning by and exposure to antiepileptic, sedative-hypnotic, antiparkinsonism and psychotropic drugs, not elsewhere classified	V01.Y98	Lithuania	8.83	2	yes
X61	Intentional self-poisoning by and exposure to antiepileptic, sedative-hypnotic, antiparkinsonism and psychotropic drugs, not elsewhere classified	V01.Y98	Netherlands	72.89	3	yes
X61	Intentional self-poisoning by and exposure to antiepileptic, sedative-hypnotic, antiparkinsonism and psychotropic drugs, not elsewhere classified	V01.Y98	Norway	36.65	1	yes
X61	Intentional self-poisoning by and exposure to antiepileptic, sedative-hypnotic, antiparkinsonism and psychotropic drugs, not elsewhere classified	V01.Y98	Poland	47.99	1	yes

For peer review only

X61	Intentional self-poisoning by and exposure to antiepileptic, sedative-hypnotic, antiparkinsonism and psychotropic drugs, not elsewhere classified	V01.Y98	Romania	7.36	0	no
X61	Intentional self-poisoning by and exposure to antiepileptic, sedative-hypnotic, antiparkinsonism and psychotropic drugs, not elsewhere classified	V01.Y98	Slovenia	9.70	0	no
X61	Intentional self-poisoning by and exposure to antiepileptic, sedative-hypnotic, antiparkinsonism and psychotropic drugs, not elsewhere classified	V01.Y98	Spain	32.10	2	yes
X61	Intentional self-poisoning by and exposure to antiepileptic, sedative-hypnotic, antiparkinsonism and psychotropic drugs, not elsewhere classified	V01.Y98	Sweden	92.45	3	yes
X61	Intentional self-poisoning by and exposure to antiepileptic, sedative-hypnotic, antiparkinsonism and psychotropic drugs, not elsewhere classified	V01.Y98	Switzerland	178.13	3	yes
X61	Intentional self-poisoning by and exposure to antiepileptic, sedative-hypnotic, antiparkinsonism and psychotropic drugs, not elsewhere classified	V01.Y98	United Kingdom	212.98	3	yes
X62	Intentional self-poisoning by and exposure to narcotics and psychodysleptics [hallucinogens], not elsewhere classified	V01.Y98	Belgium	14.14	0	no
X62	Intentional self-poisoning by and exposure to narcotics and psychodysleptics [hallucinogens], not elsewhere classified	V01.Y98	Czech Republic	6.20	0	no
X62	Intentional self-poisoning by and exposure to narcotics and psychodysleptics [hallucinogens], not elsewhere classified	V01.Y98	Denmark	26.78	0	no
X62	Intentional self-poisoning by and exposure to narcotics and psychodysleptics [hallucinogens], not elsewhere classified	V01.Y98	Finland	29.60	0	no
X62	Intentional self-poisoning by and exposure to narcotics and psychodysleptics [hallucinogens], not elsewhere classified	V01.Y98	France	21.29	2	yes
X62	Intentional self-poisoning by and exposure to narcotics and psychodysleptics [hallucinogens], not elsewhere classified	V01.Y98	Germany	100.95	0	no
X62	Intentional self-poisoning by and exposure to narcotics and psychodysleptics [hallucinogens], not elsewhere classified	V01.Y98	Hungary	6.13	0	no
X62	Intentional self-poisoning by and exposure to narcotics and psychodysleptics [hallucinogens], not elsewhere classified	V01.Y98	Netherlands	30.27	3	yes
X62	Intentional self-poisoning by and exposure to narcotics and psychodysleptics [hallucinogens], not elsewhere classified	V01.Y98	Norway	21.92	2	yes
X62	Intentional self-poisoning by and exposure to narcotics and psychodysleptics [hallucinogens], not elsewhere classified	V01.Y98	Poland	5.94	0	no
X62	Intentional self-poisoning by and exposure to narcotics and psychodysleptics [hallucinogens], not elsewhere classified	V01.Y98	Romania	5.84	0	no
X62	Intentional self-poisoning by and exposure to narcotics and psychodysleptics [hallucinogens], not elsewhere classified	V01.Y98	Spain	6.55	0	no
X62	Intentional self-poisoning by and exposure to narcotics and psychodysleptics [hallucinogens], not elsewhere classified	V01.Y98	Sweden	31.02	0	no
X62	Intentional self-poisoning by and exposure to narcotics and psychodysleptics [hallucinogens], not elsewhere classified	V01.Y98	Switzerland	10.10	0	no

For peer review only

X62	Intentional self-poisoning by and exposure to narcotics and psychodysleptics [hallucinogens], not elsewhere classified	V01.Y98	United Kingdom	158.50	2	yes
X63	Intentional self-poisoning by and exposure to other drugs acting on the autonomic nervous system	V01.Y98	Finland	16.68	0	no
X63	Intentional self-poisoning by and exposure to other drugs acting on the autonomic nervous system	V01.Y98	France	14.38	0	no
X63	Intentional self-poisoning by and exposure to other drugs acting on the autonomic nervous system	V01.Y98	Germany	20.47	0	no
X63	Intentional self-poisoning by and exposure to other drugs acting on the autonomic nervous system	V01.Y98	Hungary	10.20	0	no
X63	Intentional self-poisoning by and exposure to other drugs acting on the autonomic nervous system	V01.Y98	United Kingdom	12.71	3	yes
X64	Intentional self-poisoning by and exposure to other and unspecified drugs, medicaments and biological substances	V01.Y98	Austria	85.12	1	yes
X64	Intentional self-poisoning by and exposure to other and unspecified drugs, medicaments and biological substances	V01.Y98	Belgium	167.52	0	no
X64	Intentional self-poisoning by and exposure to other and unspecified drugs, medicaments and biological substances	V01.Y98	Croatia	16.95	1	yes
X64	Intentional self-poisoning by and exposure to other and unspecified drugs, medicaments and biological substances	V01.Y98	Czech Republic	38.66	1	yes
X64	Intentional self-poisoning by and exposure to other and unspecified drugs, medicaments and biological substances	V01.Y98	Denmark	57.41	0	no
X64	Intentional self-poisoning by and exposure to other and unspecified drugs, medicaments and biological substances	V01.Y98	Finland	25.15	0	no
X64	Intentional self-poisoning by and exposure to other and unspecified drugs, medicaments and biological substances	V01.Y98	France	1137.56	3	yes
X64	Intentional self-poisoning by and exposure to other and unspecified drugs, medicaments and biological substances	V01.Y98	Germany	808.10	3	yes
X64	Intentional self-poisoning by and exposure to other and unspecified drugs, medicaments and biological substances	V01.Y98	Hungary	125.03	1	yes
X64	Intentional self-poisoning by and exposure to other and unspecified drugs, medicaments and biological substances	V01.Y98	Lithuania	5.88	2	yes
X64	Intentional self-poisoning by and exposure to other and unspecified drugs, medicaments and biological substances	V01.Y98	Netherlands	160.80	1	yes
X64	Intentional self-poisoning by and exposure to other and unspecified drugs, medicaments and biological substances	V01.Y98	Norway	41.66	1	yes
X64	Intentional self-poisoning by and exposure to other and unspecified drugs, medicaments and biological substances	V01.Y98	Poland	52.83	1	yes
X64	Intentional self-poisoning by and exposure to other and unspecified drugs, medicaments and biological substances	V01.Y98	Romania	26.13	0	no
X64	Intentional self-poisoning by and exposure to other and unspecified drugs, medicaments and biological substances	V01.Y98	Spain	151.48	1	yes
X64	Intentional self-poisoning by and exposure to other and unspecified drugs, medicaments and biological substances	V01.Y98	Sweden	122.98	2	yes

For peer review only

X64	Intentional self-poisoning by and exposure to other and unspecified drugs, medicaments and biological substances	V01.Y98	Switzerland	60.46	0	no
X64	Intentional self-poisoning by and exposure to other and unspecified drugs, medicaments and biological substances	V01.Y98	United Kingdom	288.79	1	yes
X65	Intentional self-poisoning by and exposure to alcohol	V01.Y98	France	18.28	3	yes
X65	Intentional self-poisoning by and exposure to alcohol	V01.Y98	Germany	25.23	0	no
X65	Intentional self-poisoning by and exposure to alcohol	V01.Y98	Netherlands	6.33	1	yes
X65	Intentional self-poisoning by and exposure to alcohol	V01.Y98	Poland	190.62	3	yes
X65	Intentional self-poisoning by and exposure to alcohol	V01.Y98	Romania	30.92	1	yes
X66	Intentional self-poisoning by and exposure to organic solvents and halogenated hydrocarbons and their vapours	V01.Y98	Germany	5.32	0	no
X66	Intentional self-poisoning by and exposure to organic solvents and halogenated hydrocarbons and their vapours	V01.Y98	Hungary	12.49	1	yes
X67	Intentional self-poisoning by and exposure to other gases and vapours	V01.Y98	Austria	24.11	2	yes
X67	Intentional self-poisoning by and exposure to other gases and vapours	V01.Y98	Belgium	21.91	2	yes
X67	Intentional self-poisoning by and exposure to other gases and vapours	V01.Y98	Croatia	5.55	0	no
X67	Intentional self-poisoning by and exposure to other gases and vapours	V01.Y98	Czech Republic	29.20	1	yes
X67	Intentional self-poisoning by and exposure to other gases and vapours	V01.Y98	Denmark	38.51	0	no
X67	Intentional self-poisoning by and exposure to other gases and vapours	V01.Y98	Finland	28.19	1	yes
X67	Intentional self-poisoning by and exposure to other gases and vapours	V01.Y98	France	93.17	1	yes
X67	Intentional self-poisoning by and exposure to other gases and vapours	V01.Y98	Germany	288.27	3	yes
X67	Intentional self-poisoning by and exposure to other gases and vapours	V01.Y98	Hungary	18.13	0	no
X67	Intentional self-poisoning by and exposure to other gases and vapours	V01.Y98	Lithuania	5.63	0	no
X67	Intentional self-poisoning by and exposure to other gases and vapours	V01.Y98	Netherlands	23.49	0	no
X67	Intentional self-poisoning by and exposure to other gases and vapours	V01.Y98	Norway	16.21	2	yes
X67	Intentional self-poisoning by and exposure to other gases and vapours	V01.Y98	Poland	19.12	0	no
X67	Intentional self-poisoning by and exposure to other gases and vapours	V01.Y98	Slovenia	16.75	3	yes
X67	Intentional self-poisoning by and exposure to other gases and vapours	V01.Y98	Spain	44.73	0	no
X67	Intentional self-poisoning by and exposure to other gases and vapours	V01.Y98	Sweden	33.59	1	yes
X67	Intentional self-poisoning by and exposure to other gases and vapours	V01.Y98	Switzerland	26.78	0	no
X67	Intentional self-poisoning by and exposure to other gases and vapours	V01.Y98	United Kingdom	207.68	2	yes
X68	Intentional self-poisoning by and exposure to pesticides	V01.Y98	Belgium	10.87	1	yes

For peer review only

X68	Intentional self-poisoning by and exposure to pesticides	V01.Y98	France	20.62	2	yes
X68	Intentional self-poisoning by and exposure to pesticides	V01.Y98	Germany	42.66	2	yes
X68	Intentional self-poisoning by and exposure to pesticides	V01.Y98	Hungary	48.90	1	yes
X68	Intentional self-poisoning by and exposure to pesticides	V01.Y98	Romania	18.52	1	yes
X68	Intentional self-poisoning by and exposure to pesticides	V01.Y98	Spain	27.35	1	yes
X69	Intentional self-poisoning by and exposure to other and unspecified chemicals and noxious substances	V01.Y98	Belgium	17.01	0	no
X69	Intentional self-poisoning by and exposure to other and unspecified chemicals and noxious substances	V01.Y98	Croatia	13.20	3	yes
X69	Intentional self-poisoning by and exposure to other and unspecified chemicals and noxious substances	V01.Y98	Czech Republic	9.26	2	yes
X69	Intentional self-poisoning by and exposure to other and unspecified chemicals and noxious substances	V01.Y98	France	102.86	2	yes
X69	Intentional self-poisoning by and exposure to other and unspecified chemicals and noxious substances	V01.Y98	Germany	75.97	0	no
X69	Intentional self-poisoning by and exposure to other and unspecified chemicals and noxious substances	V01.Y98	Hungary	42.25	3	yes
X69	Intentional self-poisoning by and exposure to other and unspecified chemicals and noxious substances	V01.Y98	Latvia	6.81	1	yes
X69	Intentional self-poisoning by and exposure to other and unspecified chemicals and noxious substances	V01.Y98	Netherlands	11.33	0	no
X69	Intentional self-poisoning by and exposure to other and unspecified chemicals and noxious substances	V01.Y98	Poland	12.54	1	yes
X69	Intentional self-poisoning by and exposure to other and unspecified chemicals and noxious substances	V01.Y98	Romania	53.55	1	yes
X69	Intentional self-poisoning by and exposure to other and unspecified chemicals and noxious substances	V01.Y98	Spain	81.27	0	no
X69	Intentional self-poisoning by and exposure to other and unspecified chemicals and noxious substances	V01.Y98	Switzerland	5.02	0	no
X69	Intentional self-poisoning by and exposure to other and unspecified chemicals and noxious substances	V01.Y98	United Kingdom	12.56	1	yes
X70	Intentional self-harm by hanging, strangulation and suffocation	V01.Y98	Austria	618.58	3	yes
X70	Intentional self-harm by hanging, strangulation and suffocation	V01.Y98	Belgium	1036.56	3	yes
X70	Intentional self-harm by hanging, strangulation and suffocation	V01.Y98	Croatia	478.17	1	yes
X70	Intentional self-harm by hanging, strangulation and suffocation	V01.Y98	Czech Republic	950.55	1	yes
X70	Intentional self-harm by hanging, strangulation and suffocation	V01.Y98	Denmark	269.12	2	yes
X70	Intentional self-harm by hanging, strangulation and suffocation	V01.Y98	Estonia	215.74	2	yes
X70	Intentional self-harm by hanging, strangulation and suffocation	V01.Y98	Finland	309.30	3	yes
X70	Intentional self-harm by hanging, strangulation and suffocation	V01.Y98	France	5226.91	0	no
X70	Intentional self-harm by hanging, strangulation and suffocation	V01.Y98	Germany	4776.47	2	yes
X70	Intentional self-harm by hanging, strangulation and suffocation	V01.Y98	Hungary	1621.60	1	yes
X70	Intentional self-harm by hanging, strangulation and suffocation	V01.Y98	Latvia	429.42	2	yes
X70	Intentional self-harm by hanging, strangulation and suffocation	V01.Y98	Lithuania	1083.28	2	yes

For peer review only

X70	Intentional self-harm by hanging, strangulation and suffocation	V01.Y98	Netherlands	733.42	2	yes
X70	Intentional self-harm by hanging, strangulation and suffocation	V01.Y98	Norway	211.43	0	no
X70	Intentional self-harm by hanging, strangulation and suffocation	V01.Y98	Poland	5312.91	3	yes
X70	Intentional self-harm by hanging, strangulation and suffocation	V01.Y98	Romania	2391.17	1	yes
X70	Intentional self-harm by hanging, strangulation and suffocation	V01.Y98	Slovenia	309.88	2	yes
X70	Intentional self-harm by hanging, strangulation and suffocation	V01.Y98	Spain	1718.76	0	no
X70	Intentional self-harm by hanging, strangulation and suffocation	V01.Y98	Sweden	487.87	0	no
X70	Intentional self-harm by hanging, strangulation and suffocation	V01.Y98	Switzerland	321.11	1	yes
X70	Intentional self-harm by hanging, strangulation and suffocation	V01.Y98	United Kingdom	2442.85	1	yes
X71	Intentional self-harm by drowning and submersion	V01.Y98	Austria	60.16	1	yes
X71	Intentional self-harm by drowning and submersion	V01.Y98	Belgium	157.70	2	yes
X71	Intentional self-harm by drowning and submersion	V01.Y98	Croatia	44.48	1	yes
X71	Intentional self-harm by drowning and submersion	V01.Y98	Czech Republic	26.69	2	yes
X71	Intentional self-harm by drowning and submersion	V01.Y98	Denmark	41.03	1	yes
X71	Intentional self-harm by drowning and submersion	V01.Y98	Finland	51.21	1	yes
X71	Intentional self-harm by drowning and submersion	V01.Y98	France	599.82	1	yes
X71	Intentional self-harm by drowning and submersion	V01.Y98	Germany	295.45	2	yes
X71	Intentional self-harm by drowning and submersion	V01.Y98	Hungary	55.91	1	yes
X71	Intentional self-harm by drowning and submersion	V01.Y98	Latvia	6.30	2	yes
X71	Intentional self-harm by drowning and submersion	V01.Y98	Lithuania	8.88	0	no
X71	Intentional self-harm by drowning and submersion	V01.Y98	Netherlands	118.68	2	yes
X71	Intentional self-harm by drowning and submersion	V01.Y98	Norway	35.39	0	no
X71	Intentional self-harm by drowning and submersion	V01.Y98	Poland	37.24	1	yes
X71	Intentional self-harm by drowning and submersion	V01.Y98	Romania	29.20	1	yes
X71	Intentional self-harm by drowning and submersion	V01.Y98	Slovenia	22.96	1	yes
X71	Intentional self-harm by drowning and submersion	V01.Y98	Spain	151.07	1	yes
X71	Intentional self-harm by drowning and submersion	V01.Y98	Sweden	68.01	2	yes
X71	Intentional self-harm by drowning and submersion	V01.Y98	Switzerland	65.29	1	yes
X71	Intentional self-harm by drowning and submersion	V01.Y98	United Kingdom	133.13	0	no
X72	Intentional self-harm by handgun discharge	V01.Y98	Austria	15.19	0	no
X72	Intentional self-harm by handgun discharge	V01.Y98	Belgium	14.79	2	yes
X72	Intentional self-harm by handgun discharge	V01.Y98	Croatia	25.61	0	no
X72	Intentional self-harm by handgun discharge	V01.Y98	Czech Republic	72.46	2	yes
X72	Intentional self-harm by handgun discharge	V01.Y98	Denmark	15.12	0	no
X72	Intentional self-harm by handgun discharge	V01.Y98	Finland	59.03	1	yes
X72	Intentional self-harm by handgun discharge	V01.Y98	France	15.18	0	no
X72	Intentional self-harm by handgun discharge	V01.Y98	Germany	234.90	1	yes
X72	Intentional self-harm by handgun discharge	V01.Y98	Hungary	27.24	2	yes
X72	Intentional self-harm by handgun discharge	V01.Y98	Lithuania	8.40	0	no
X72	Intentional self-harm by handgun discharge	V01.Y98	Netherlands	7.14	1	yes
X72	Intentional self-harm by handgun discharge	V01.Y98	Norway	7.33	0	no
X72	Intentional self-harm by handgun discharge	V01.Y98	Poland	14.66	0	no
X72	Intentional self-harm by handgun discharge	V01.Y98	Slovenia	11.35	0	no
X72	Intentional self-harm by handgun discharge	V01.Y98	Spain	23.91	2	yes

For peer review only

X72	Intentional self-harm by handgun discharge	V01.Y98	Sweden	18.78	0	no
X72	Intentional self-harm by handgun discharge	V01.Y98	Switzerland	29.73	0	no
X73	Intentional self-harm by rifle, shotgun and larger firearm discharge	V01.Y98	Austria	24.68	0	no
X73	Intentional self-harm by rifle, shotgun and larger firearm discharge	V01.Y98	Belgium	28.19	1	yes
X73	Intentional self-harm by rifle, shotgun and larger firearm discharge	V01.Y98	Croatia	12.88	0	no
X73	Intentional self-harm by rifle, shotgun and larger firearm discharge	V01.Y98	Czech Republic	20.19	0	no
X73	Intentional self-harm by rifle, shotgun and larger firearm discharge	V01.Y98	Denmark	46.53	0	no
X73	Intentional self-harm by rifle, shotgun and larger firearm discharge	V01.Y98	Estonia	5.62	1	yes
X73	Intentional self-harm by rifle, shotgun and larger firearm discharge	V01.Y98	Finland	115.19	1	yes
X73	Intentional self-harm by rifle, shotgun and larger firearm discharge	V01.Y98	France	140.54	3	yes
X73	Intentional self-harm by rifle, shotgun and larger firearm discharge	V01.Y98	Germany	68.01	1	yes
X73	Intentional self-harm by rifle, shotgun and larger firearm discharge	V01.Y98	Hungary	10.18	2	yes
X73	Intentional self-harm by rifle, shotgun and larger firearm discharge	V01.Y98	Latvia	6.72	0	no
X73	Intentional self-harm by rifle, shotgun and larger firearm discharge	V01.Y98	Norway	29.32	2	yes
X73	Intentional self-harm by rifle, shotgun and larger firearm discharge	V01.Y98	Poland	9.02	2	yes
X73	Intentional self-harm by rifle, shotgun and larger firearm discharge	V01.Y98	Slovenia	5.68	2	yes
X73	Intentional self-harm by rifle, shotgun and larger firearm discharge	V01.Y98	Spain	51.69	3	yes
X73	Intentional self-harm by rifle, shotgun and larger firearm discharge	V01.Y98	Sweden	78.06	2	yes
X73	Intentional self-harm by rifle, shotgun and larger firearm discharge	V01.Y98	Switzerland	20.18	1	yes
X73	Intentional self-harm by rifle, shotgun and larger firearm discharge	V01.Y98	United Kingdom	57.14	1	yes
X74	Intentional self-harm by other and unspecified firearm discharge	V01.Y98	Austria	188.90	2	yes
X74	Intentional self-harm by other and unspecified firearm discharge	V01.Y98	Belgium	162.93	1	yes
X74	Intentional self-harm by other and unspecified firearm discharge	V01.Y98	Croatia	70.08	1	yes
X74	Intentional self-harm by other and unspecified firearm discharge	V01.Y98	Czech Republic	70.95	1	yes
X74	Intentional self-harm by other and unspecified firearm discharge	V01.Y98	Denmark	5.43	1	yes
X74	Intentional self-harm by other and unspecified firearm discharge	V01.Y98	Estonia	10.88	0	no
X74	Intentional self-harm by other and unspecified firearm discharge	V01.Y98	Finland	7.99	0	no
X74	Intentional self-harm by other and unspecified firearm discharge	V01.Y98	France	1511.58	3	yes
X74	Intentional self-harm by other and unspecified firearm discharge	V01.Y98	Germany	483.30	1	yes
X74	Intentional self-harm by other and unspecified firearm discharge	V01.Y98	Hungary	42.47	2	yes
X74	Intentional self-harm by other and unspecified firearm discharge	V01.Y98	Latvia	16.63	1	yes

For peer review only

X74	Intentional self-harm by other and unspecified firearm discharge	V01.Y98	Lithuania	11.17	1	yes
X74	Intentional self-harm by other and unspecified firearm discharge	V01.Y98	Netherlands	32.71	0	no
X74	Intentional self-harm by other and unspecified firearm discharge	V01.Y98	Norway	51.16	2	yes
X74	Intentional self-harm by other and unspecified firearm discharge	V01.Y98	Poland	21.95	2	yes
X74	Intentional self-harm by other and unspecified firearm discharge	V01.Y98	Romania	9.27	0	no
X74	Intentional self-harm by other and unspecified firearm discharge	V01.Y98	Slovenia	32.45	0	no
X74	Intentional self-harm by other and unspecified firearm discharge	V01.Y98	Spain	117.31	0	no
X74	Intentional self-harm by other and unspecified firearm discharge	V01.Y98	Sweden	30.78	2	yes
X74	Intentional self-harm by other and unspecified firearm discharge	V01.Y98	Switzerland	221.89	2	yes
X74	Intentional self-harm by other and unspecified firearm discharge	V01.Y98	United Kingdom	53.59	1	yes
X75	Intentional self-harm by explosive material	V01.Y98	Croatia	19.61	1	yes
X76	Intentional self-harm by smoke, fire and flames	V01.Y98	Austria	6.84	0	no
X76	Intentional self-harm by smoke, fire and flames	V01.Y98	Belgium	10.59	0	no
X76	Intentional self-harm by smoke, fire and flames	V01.Y98	Croatia	5.37	0	no
X76	Intentional self-harm by smoke, fire and flames	V01.Y98	Czech Republic	6.52	0	no
X76	Intentional self-harm by smoke, fire and flames	V01.Y98	Denmark	6.08	1	yes
X76	Intentional self-harm by smoke, fire and flames	V01.Y98	Finland	10.76	1	yes
X76	Intentional self-harm by smoke, fire and flames	V01.Y98	France	66.24	0	no
X76	Intentional self-harm by smoke, fire and flames	V01.Y98	Germany	70.57	0	no
X76	Intentional self-harm by smoke, fire and flames	V01.Y98	Hungary	13.01	1	yes
X76	Intentional self-harm by smoke, fire and flames	V01.Y98	Netherlands	14.52	0	no
X76	Intentional self-harm by smoke, fire and flames	V01.Y98	Poland	11.25	0	no
X76	Intentional self-harm by smoke, fire and flames	V01.Y98	Romania	7.29	1	yes
X76	Intentional self-harm by smoke, fire and flames	V01.Y98	Spain	15.89	1	yes
X76	Intentional self-harm by smoke, fire and flames	V01.Y98	Sweden	14.87	0	no
X76	Intentional self-harm by smoke, fire and flames	V01.Y98	United Kingdom	48.70	0	no
X78	Intentional self-harm by sharp object	V01.Y98	Austria	42.93	0	no
X78	Intentional self-harm by sharp object	V01.Y98	Belgium	24.97	0	no
X78	Intentional self-harm by sharp object	V01.Y98	Croatia	25.36	0	no
X78	Intentional self-harm by sharp object	V01.Y98	Czech Republic	57.01	1	yes
X78	Intentional self-harm by sharp object	V01.Y98	Denmark	25.75	0	no
X78	Intentional self-harm by sharp object	V01.Y98	Estonia	6.79	2	yes
X78	Intentional self-harm by sharp object	V01.Y98	Finland	20.80	0	no
X78	Intentional self-harm by sharp object	V01.Y98	France	109.84	2	yes
X78	Intentional self-harm by sharp object	V01.Y98	Germany	361.17	0	no
X78	Intentional self-harm by sharp object	V01.Y98	Hungary	77.46	0	no
X78	Intentional self-harm by sharp object	V01.Y98	Latvia	12.79	0	no
X78	Intentional self-harm by sharp object	V01.Y98	Lithuania	13.98	0	no
X78	Intentional self-harm by sharp object	V01.Y98	Netherlands	44.92	3	yes
X78	Intentional self-harm by sharp object	V01.Y98	Norway	14.76	0	no
X78	Intentional self-harm by sharp object	V01.Y98	Poland	78.28	1	yes
X78	Intentional self-harm by sharp object	V01.Y98	Romania	42.35	0	no
X78	Intentional self-harm by sharp object	V01.Y98	Slovenia	11.95	0	no
X78	Intentional self-harm by sharp object	V01.Y98	Spain	71.57	0	no

For peer review only

X78	Intentional self-harm by sharp object	V01.Y98	Sweden	34.17	1	yes
X78	Intentional self-harm by sharp object	V01.Y98	Switzerland	28.43	0	no
X78	Intentional self-harm by sharp object	V01.Y98	United Kingdom	126.21	3	yes
X80	Intentional self-harm by jumping from a high place	V01.Y98	Austria	144.01	1	yes
X80	Intentional self-harm by jumping from a high place	V01.Y98	Belgium	111.25	1	yes
X80	Intentional self-harm by jumping from a high place	V01.Y98	Croatia	54.85	0	no
X80	Intentional self-harm by jumping from a high place	V01.Y98	Czech Republic	139.01	2	yes
X80	Intentional self-harm by jumping from a high place	V01.Y98	Denmark	34.76	0	no
X80	Intentional self-harm by jumping from a high place	V01.Y98	Estonia	8.90	2	yes
X80	Intentional self-harm by jumping from a high place	V01.Y98	Finland	55.38	3	yes
X80	Intentional self-harm by jumping from a high place	V01.Y98	France	725.25	1	yes
X80	Intentional self-harm by jumping from a high place	V01.Y98	Germany	960.08	2	yes
X80	Intentional self-harm by jumping from a high place	V01.Y98	Hungary	165.03	1	yes
X80	Intentional self-harm by jumping from a high place	V01.Y98	Latvia	18.62	1	yes
X80	Intentional self-harm by jumping from a high place	V01.Y98	Lithuania	21.39	3	yes
X80	Intentional self-harm by jumping from a high place	V01.Y98	Netherlands	137.31	0	no
X80	Intentional self-harm by jumping from a high place	V01.Y98	Norway	29.02	0	no
X80	Intentional self-harm by jumping from a high place	V01.Y98	Poland	133.36	3	yes
X80	Intentional self-harm by jumping from a high place	V01.Y98	Romania	37.10	3	yes
X80	Intentional self-harm by jumping from a high place	V01.Y98	Slovenia	23.73	1	yes
X80	Intentional self-harm by jumping from a high place	V01.Y98	Spain	789.29	2	yes
X80	Intentional self-harm by jumping from a high place	V01.Y98	Sweden	61.41	0	no
X80	Intentional self-harm by jumping from a high place	V01.Y98	Switzerland	146.03	3	yes
X80	Intentional self-harm by jumping from a high place	V01.Y98	United Kingdom	147.98	1	yes
X81	Intentional self-harm by jumping or lying before moving object	V01.Y98	Austria	87.03	0	no
X81	Intentional self-harm by jumping or lying before moving object	V01.Y98	Belgium	83.81	0	no
X81	Intentional self-harm by jumping or lying before moving object	V01.Y98	Croatia	19.12	0	no
X81	Intentional self-harm by jumping or lying before moving object	V01.Y98	Czech Republic	48.92	2	yes
X81	Intentional self-harm by jumping or lying before moving object	V01.Y98	Denmark	23.90	0	no
X81	Intentional self-harm by jumping or lying before moving object	V01.Y98	Finland	53.71	0	no
X81	Intentional self-harm by jumping or lying before moving object	V01.Y98	France	189.23	1	yes
X81	Intentional self-harm by jumping or lying before moving object	V01.Y98	Germany	630.08	3	yes
X81	Intentional self-harm by jumping or lying before moving object	V01.Y98	Hungary	101.65	2	yes
X81	Intentional self-harm by jumping or lying before moving object	V01.Y98	Netherlands	183.64	0	no
X81	Intentional self-harm by jumping or lying before moving object	V01.Y98	Norway	10.08	0	no
X81	Intentional self-harm by jumping or lying before moving object	V01.Y98	Poland	20.85	3	yes
X81	Intentional self-harm by jumping or lying before moving object	V01.Y98	Slovenia	12.33	0	no
X81	Intentional self-harm by jumping or lying before moving object	V01.Y98	Spain	93.44	1	yes
X81	Intentional self-harm by jumping or lying before moving object	V01.Y98	Sweden	78.53	1	yes
X81	Intentional self-harm by jumping or lying before moving object	V01.Y98	Switzerland	101.40	2	yes

For peer review only

X81	Intentional self-harm by jumping or lying before moving object	V01.Y98	United Kingdom	192.24	2	yes
X82	Intentional self-harm by crashing of motor vehicle	V01.Y98	Finland	18.87	1	yes
X82	Intentional self-harm by crashing of motor vehicle	V01.Y98	France	18.88	0	no
X82	Intentional self-harm by crashing of motor vehicle	V01.Y98	Germany	68.20	2	yes
X82	Intentional self-harm by crashing of motor vehicle	V01.Y98	Hungary	10.93	1	yes
X82	Intentional self-harm by crashing of motor vehicle	V01.Y98	Netherlands	12.69	0	no
X82	Intentional self-harm by crashing of motor vehicle	V01.Y98	Norway	6.11	0	no
X82	Intentional self-harm by crashing of motor vehicle	V01.Y98	Poland	11.65	1	yes
X82	Intentional self-harm by crashing of motor vehicle	V01.Y98	Romania	9.44	0	no
X82	Intentional self-harm by crashing of motor vehicle	V01.Y98	Spain	15.04	3	yes
X82	Intentional self-harm by crashing of motor vehicle	V01.Y98	Sweden	9.22	0	no
X82	Intentional self-harm by crashing of motor vehicle	V01.Y98	United Kingdom	9.05	0	no
X83	Intentional self-harm by other specified means	V01.Y98	Austria	7.02	0	no
X83	Intentional self-harm by other specified means	V01.Y98	Belgium	7.98	0	no
X83	Intentional self-harm by other specified means	V01.Y98	Czech Republic	5.34	0	no
X83	Intentional self-harm by other specified means	V01.Y98	France	33.96	2	yes
X83	Intentional self-harm by other specified means	V01.Y98	Germany	95.03	1	yes
X83	Intentional self-harm by other specified means	V01.Y98	Hungary	32.08	0	no
X83	Intentional self-harm by other specified means	V01.Y98	Poland	11.06	0	no
X83	Intentional self-harm by other specified means	V01.Y98	Romania	7.80	2	yes
X83	Intentional self-harm by other specified means	V01.Y98	Sweden	5.87	0	no
X83	Intentional self-harm by other specified means	V01.Y98	Switzerland	7.24	0	no
X83	Intentional self-harm by other specified means	V01.Y98	United Kingdom	19.81	1	yes
X84	Intentional self-harm by unspecified means	V01.Y98	Austria	13.75	2	yes
X84	Intentional self-harm by unspecified means	V01.Y98	Belgium	103.23	2	yes
X84	Intentional self-harm by unspecified means	V01.Y98	Croatia	19.39	2	yes
X84	Intentional self-harm by unspecified means	V01.Y98	Czech Republic	16.22	2	yes
X84	Intentional self-harm by unspecified means	V01.Y98	France	579.83	1	yes
X84	Intentional self-harm by unspecified means	V01.Y98	Germany	208.49	1	yes
X84	Intentional self-harm by unspecified means	V01.Y98	Netherlands	10.77	2	yes
X84	Intentional self-harm by unspecified means	V01.Y98	Norway	19.58	2	yes
X84	Intentional self-harm by unspecified means	V01.Y98	Poland	17.74	1	yes
X84	Intentional self-harm by unspecified means	V01.Y98	Romania	10.66	3	yes
X84	Intentional self-harm by unspecified means	V01.Y98	Spain	129.95	0	no
X84	Intentional self-harm by unspecified means	V01.Y98	Switzerland	19.89	0	no
X84	Intentional self-harm by unspecified means	V01.Y98	United Kingdom	116.18	0	no
X91	Assault by hanging, strangulation and suffocation	V01.Y98	Austria	6.90	2	yes
X91	Assault by hanging, strangulation and suffocation	V01.Y98	Belgium	18.08	1	yes
X91	Assault by hanging, strangulation and suffocation	V01.Y98	Czech Republic	12.05	1	yes
X91	Assault by hanging, strangulation and suffocation	V01.Y98	Denmark	6.72	0	no
X91	Assault by hanging, strangulation and suffocation	V01.Y98	Estonia	5.99	3	yes
X91	Assault by hanging, strangulation and suffocation	V01.Y98	Finland	9.74	1	yes
X91	Assault by hanging, strangulation and suffocation	V01.Y98	France	36.67	1	yes
X91	Assault by hanging, strangulation and suffocation	V01.Y98	Germany	65.63	1	yes
X91	Assault by hanging, strangulation and suffocation	V01.Y98	Hungary	17.61	1	yes
X91	Assault by hanging, strangulation and suffocation	V01.Y98	Latvia	11.80	1	yes
X91	Assault by hanging, strangulation and suffocation	V01.Y98	Lithuania	14.27	1	yes
X91	Assault by hanging, strangulation and suffocation	V01.Y98	Netherlands	14.35	0	no
X91	Assault by hanging, strangulation and suffocation	V01.Y98	Poland	38.54	1	yes

For peer review only

X91	Assault by hanging, strangulation and suffocation	V01.Y98	Romania	50.01	1	yes
X91	Assault by hanging, strangulation and suffocation	V01.Y98	Spain	29.79	1	yes
X91	Assault by hanging, strangulation and suffocation	V01.Y98	Sweden	6.95	0	no
X91	Assault by hanging, strangulation and suffocation	V01.Y98	Switzerland	7.21	1	yes
X91	Assault by hanging, strangulation and suffocation	V01.Y98	United Kingdom	14.96	1	yes
X92	Assault by drowning and submersion	V01.Y98	Germany	5.65	1	yes
X93	Assault by handgun discharge	V01.Y98	Croatia	7.40	2	yes
X93	Assault by handgun discharge	V01.Y98	Czech Republic	10.63	0	no
X93	Assault by handgun discharge	V01.Y98	Finland	5.59	0	no
X93	Assault by handgun discharge	V01.Y98	Germany	24.89	0	no
X93	Assault by handgun discharge	V01.Y98	Spain	11.51	2	yes
X93	Assault by handgun discharge	V01.Y98	Sweden	5.47	3	yes
X94	Assault by rifle, shotgun and larger firearm discharge	V01.Y98	Finland	7.33	1	yes
X94	Assault by rifle, shotgun and larger firearm discharge	V01.Y98	France	8.70	0	no
X94	Assault by rifle, shotgun and larger firearm discharge	V01.Y98	Spain	10.98	0	no
X94	Assault by rifle, shotgun and larger firearm discharge	V01.Y98	United Kingdom	5.59	0	no
X95	Assault by other and unspecified firearm discharge	V01.Y98	Austria	10.03	0	no
X95	Assault by other and unspecified firearm discharge	V01.Y98	Belgium	36.08	1	yes
X95	Assault by other and unspecified firearm discharge	V01.Y98	Croatia	13.26	1	yes
X95	Assault by other and unspecified firearm discharge	V01.Y98	Czech Republic	7.69	3	yes
X95	Assault by other and unspecified firearm discharge	V01.Y98	Finland	5.78	0	no
X95	Assault by other and unspecified firearm discharge	V01.Y98	France	123.92	1	yes
X95	Assault by other and unspecified firearm discharge	V01.Y98	Germany	37.64	1	yes
X95	Assault by other and unspecified firearm discharge	V01.Y98	Latvia	8.95	2	yes
X95	Assault by other and unspecified firearm discharge	V01.Y98	Lithuania	5.02	2	yes
X95	Assault by other and unspecified firearm discharge	V01.Y98	Netherlands	40.46	3	yes
X95	Assault by other and unspecified firearm discharge	V01.Y98	Norway	7.35	0	no
X95	Assault by other and unspecified firearm discharge	V01.Y98	Poland	21.34	2	yes
X95	Assault by other and unspecified firearm discharge	V01.Y98	Romania	6.94	1	yes
X95	Assault by other and unspecified firearm discharge	V01.Y98	Spain	49.49	1	yes
X95	Assault by other and unspecified firearm discharge	V01.Y98	Sweden	10.56	2	yes
X95	Assault by other and unspecified firearm discharge	V01.Y98	Switzerland	17.25	1	yes
X95	Assault by other and unspecified firearm discharge	V01.Y98	United Kingdom	21.78	1	yes
X97	Assault by fire and flames	V01.Y98	United Kingdom	6.97	1	yes
X99	Assault by sharp object	V01.Y98	Austria	17.57	0	no
X99	Assault by sharp object	V01.Y98	Belgium	36.89	1	yes
X99	Assault by sharp object	V01.Y98	Croatia	15.50	0	no
X99	Assault by sharp object	V01.Y98	Czech Republic	36.72	0	no
X99	Assault by sharp object	V01.Y98	Denmark	13.33	0	no
X99	Assault by sharp object	V01.Y98	Estonia	36.69	1	yes
X99	Assault by sharp object	V01.Y98	Finland	43.70	1	yes
X99	Assault by sharp object	V01.Y98	France	116.40	2	yes
X99	Assault by sharp object	V01.Y98	Germany	162.97	1	yes
X99	Assault by sharp object	V01.Y98	Hungary	61.84	1	yes
X99	Assault by sharp object	V01.Y98	Latvia	65.82	1	yes
X99	Assault by sharp object	V01.Y98	Lithuania	78.53	1	yes
X99	Assault by sharp object	V01.Y98	Netherlands	50.67	1	yes
X99	Assault by sharp object	V01.Y98	Norway	13.55	0	no
X99	Assault by sharp object	V01.Y98	Poland	201.28	2	yes
X99	Assault by sharp object	V01.Y98	Romania	136.65	2	yes

For peer review only

X99	Assault by sharp object	V01.Y98	Slovenia	5.99	0	no
X99	Assault by sharp object	V01.Y98	Spain	140.90	1	yes
X99	Assault by sharp object	V01.Y98	Sweden	36.44	0	no
X99	Assault by sharp object	V01.Y98	Switzerland	12.94	1	yes
X99	Assault by sharp object	V01.Y98	United Kingdom	70.75	1	yes
Y00	Assault by blunt object	V01.Y98	Croatia	5.49	0	no
Y00	Assault by blunt object	V01.Y98	Czech Republic	5.84	0	no
Y00	Assault by blunt object	V01.Y98	Estonia	16.90	3	yes
Y00	Assault by blunt object	V01.Y98	Finland	9.43	1	yes
Y00	Assault by blunt object	V01.Y98	France	8.22	0	no
Y00	Assault by blunt object	V01.Y98	Germany	38.94	0	no
Y00	Assault by blunt object	V01.Y98	Hungary	20.71	1	yes
Y00	Assault by blunt object	V01.Y98	Latvia	9.51	0	no
Y00	Assault by blunt object	V01.Y98	Lithuania	101.90	2	yes
Y00	Assault by blunt object	V01.Y98	Netherlands	11.02	0	no
Y00	Assault by blunt object	V01.Y98	Poland	64.80	1	yes
Y00	Assault by blunt object	V01.Y98	Romania	59.74	2	yes
Y00	Assault by blunt object	V01.Y98	Spain	16.74	0	no
Y00	Assault by blunt object	V01.Y98	Sweden	6.79	0	no
Y00	Assault by blunt object	V01.Y98	United Kingdom	13.22	2	yes
Y04	Assault by bodily force	V01.Y98	Belgium	13.49	2	yes
Y04	Assault by bodily force	V01.Y98	Croatia	6.85	1	yes
Y04	Assault by bodily force	V01.Y98	Czech Republic	9.06	0	no
Y04	Assault by bodily force	V01.Y98	Estonia	13.98	1	yes
Y04	Assault by bodily force	V01.Y98	Finland	12.07	1	yes
Y04	Assault by bodily force	V01.Y98	France	49.80	0	no
Y04	Assault by bodily force	V01.Y98	Germany	41.10	1	yes
Y04	Assault by bodily force	V01.Y98	Hungary	53.27	2	yes
Y04	Assault by bodily force	V01.Y98	Latvia	54.25	2	yes
Y04	Assault by bodily force	V01.Y98	Lithuania	8.87	2	yes
Y04	Assault by bodily force	V01.Y98	Netherlands	11.03	0	no
Y04	Assault by bodily force	V01.Y98	Poland	119.06	2	yes
Y04	Assault by bodily force	V01.Y98	Romania	87.26	1	yes
Y04	Assault by bodily force	V01.Y98	Spain	12.35	1	yes
Y04	Assault by bodily force	V01.Y98	Sweden	5.58	1	yes
Y04	Assault by bodily force	V01.Y98	United Kingdom	14.70	3	yes
Y06	Neglect and abandonment	V01.Y98	Germany	5.71	3	yes
Y07	Other maltreatment syndromes	V01.Y98	France	10.55	1	yes
Y07	Other maltreatment syndromes	V01.Y98	Germany	15.86	0	no
Y07	Other maltreatment syndromes	V01.Y98	Poland	6.09	1	yes
Y07	Other maltreatment syndromes	V01.Y98	Romania	9.24	3	yes
Y08	Assault by other specified means	V01.Y98	Germany	7.64	3	yes
Y09	Assault by unspecified means	V01.Y98	Belgium	22.11	0	no
Y09	Assault by unspecified means	V01.Y98	Czech Republic	11.99	1	yes
Y09	Assault by unspecified means	V01.Y98	Estonia	12.08	3	yes
Y09	Assault by unspecified means	V01.Y98	France	59.06	1	yes
Y09	Assault by unspecified means	V01.Y98	Germany	38.41	1	yes
Y09	Assault by unspecified means	V01.Y98	Hungary	5.09	0	no
Y09	Assault by unspecified means	V01.Y98	Latvia	27.52	0	no
Y09	Assault by unspecified means	V01.Y98	Netherlands	7.70	3	yes

For peer review only

Y09	Assault by unspecified means	V01.Y98	Poland	17.46	1	yes
Y09	Assault by unspecified means	V01.Y98	Romania	189.54	1	yes
Y09	Assault by unspecified means	V01.Y98	Spain	63.01	1	yes
Y09	Assault by unspecified means	V01.Y98	Sweden	5.26	1	yes
Y09	Assault by unspecified means	V01.Y98	United Kingdom	36.68	1	yes
Y10	Poisoning by and exposure to nonopioid analgesics, antipyretics and antirheumatics, undetermined intent	V01.Y98	Sweden	9.14	0	no
Y10	Poisoning by and exposure to nonopioid analgesics, antipyretics and antirheumatics, undetermined intent	V01.Y98	United Kingdom	60.67	1	yes
Y11	Poisoning by and exposure to antiepileptic, sedative-hypnotic, antiparkinsonism and psychotropic drugs, not elsewhere classified, undetermined intent	V01.Y98	Czech Republic	10.48	2	yes
Y11	Poisoning by and exposure to antiepileptic, sedative-hypnotic, antiparkinsonism and psychotropic drugs, not elsewhere classified, undetermined intent	V01.Y98	Denmark	8.95	1	yes
Y11	Poisoning by and exposure to antiepileptic, sedative-hypnotic, antiparkinsonism and psychotropic drugs, not elsewhere classified, undetermined intent	V01.Y98	Finland	5.25	2	yes
Y11	Poisoning by and exposure to antiepileptic, sedative-hypnotic, antiparkinsonism and psychotropic drugs, not elsewhere classified, undetermined intent	V01.Y98	Germany	30.18	1	yes
Y11	Poisoning by and exposure to antiepileptic, sedative-hypnotic, antiparkinsonism and psychotropic drugs, not elsewhere classified, undetermined intent	V01.Y98	Hungary	12.45	1	yes
Y11	Poisoning by and exposure to antiepileptic, sedative-hypnotic, antiparkinsonism and psychotropic drugs, not elsewhere classified, undetermined intent	V01.Y98	Lithuania	11.36	0	no
Y11	Poisoning by and exposure to antiepileptic, sedative-hypnotic, antiparkinsonism and psychotropic drugs, not elsewhere classified, undetermined intent	V01.Y98	Poland	21.01	1	yes
Y11	Poisoning by and exposure to antiepileptic, sedative-hypnotic, antiparkinsonism and psychotropic drugs, not elsewhere classified, undetermined intent	V01.Y98	Sweden	56.41	3	yes
Y11	Poisoning by and exposure to antiepileptic, sedative-hypnotic, antiparkinsonism and psychotropic drugs, not elsewhere classified, undetermined intent	V01.Y98	United Kingdom	153.14	1	yes
Y12	Poisoning by and exposure to narcotics and psychodysleptics [hallucinogens], not elsewhere classified, undetermined intent	V01.Y98	Belgium	8.42	1	yes
Y12	Poisoning by and exposure to narcotics and psychodysleptics [hallucinogens], not elsewhere classified, undetermined intent	V01.Y98	Denmark	27.30	1	yes
Y12	Poisoning by and exposure to narcotics and psychodysleptics [hallucinogens], not elsewhere classified, undetermined intent	V01.Y98	Germany	86.69	3	yes
Y12	Poisoning by and exposure to narcotics and psychodysleptics [hallucinogens], not elsewhere classified, undetermined intent	V01.Y98	Poland	47.80	3	yes
Y12	Poisoning by and exposure to narcotics and psychodysleptics [hallucinogens], not elsewhere classified, undetermined intent	V01.Y98	Slovenia	8.38	3	yes
Y12	Poisoning by and exposure to narcotics and psychodysleptics [hallucinogens], not elsewhere classified, undetermined intent	V01.Y98	Sweden	52.51	2	yes
Y12	Poisoning by and exposure to narcotics and psychodysleptics [hallucinogens], not elsewhere classified, undetermined intent	V01.Y98	United Kingdom	199.98	0	no

For peer review only

Y13	Poisoning by and exposure to other drugs acting on the autonomic nervous system, undetermined intent	V01.Y98	United Kingdom	7.16	0	no
Y14	Poisoning by and exposure to other and unspecified drugs, medicaments and biological substances, undetermined intent	V01.Y98	Austria	49.85	2	yes
Y14	Poisoning by and exposure to other and unspecified drugs, medicaments and biological substances, undetermined intent	V01.Y98	Belgium	16.13	1	yes
Y14	Poisoning by and exposure to other and unspecified drugs, medicaments and biological substances, undetermined intent	V01.Y98	Czech Republic	12.29	0	no
Y14	Poisoning by and exposure to other and unspecified drugs, medicaments and biological substances, undetermined intent	V01.Y98	Denmark	20.61	1	yes
Y14	Poisoning by and exposure to other and unspecified drugs, medicaments and biological substances, undetermined intent	V01.Y98	France	25.12	2	yes
Y14	Poisoning by and exposure to other and unspecified drugs, medicaments and biological substances, undetermined intent	V01.Y98	Germany	108.71	2	yes
Y14	Poisoning by and exposure to other and unspecified drugs, medicaments and biological substances, undetermined intent	V01.Y98	Hungary	13.60	3	yes
Y14	Poisoning by and exposure to other and unspecified drugs, medicaments and biological substances, undetermined intent	V01.Y98	Lithuania	5.51	0	no
Y14	Poisoning by and exposure to other and unspecified drugs, medicaments and biological substances, undetermined intent	V01.Y98	Netherlands	7.43	0	no
Y14	Poisoning by and exposure to other and unspecified drugs, medicaments and biological substances, undetermined intent	V01.Y98	Poland	74.27	1	yes
Y14	Poisoning by and exposure to other and unspecified drugs, medicaments and biological substances, undetermined intent	V01.Y98	Romania	5.83	0	no
Y14	Poisoning by and exposure to other and unspecified drugs, medicaments and biological substances, undetermined intent	V01.Y98	Spain	8.31	0	no
Y14	Poisoning by and exposure to other and unspecified drugs, medicaments and biological substances, undetermined intent	V01.Y98	Sweden	71.62	2	yes
Y14	Poisoning by and exposure to other and unspecified drugs, medicaments and biological substances, undetermined intent	V01.Y98	Switzerland	5.47	0	no
Y14	Poisoning by and exposure to other and unspecified drugs, medicaments and biological substances, undetermined intent	V01.Y98	United Kingdom	180.15	1	yes
Y15	Poisoning by and exposure to alcohol, undetermined intent	V01.Y98	Czech Republic	10.38	2	yes
Y15	Poisoning by and exposure to alcohol, undetermined intent	V01.Y98	Denmark	6.57	2	yes
Y15	Poisoning by and exposure to alcohol, undetermined intent	V01.Y98	Germany	30.84	0	no
Y15	Poisoning by and exposure to alcohol, undetermined intent	V01.Y98	Hungary	5.54	0	no
Y15	Poisoning by and exposure to alcohol, undetermined intent	V01.Y98	Poland	334.76	2	yes
Y15	Poisoning by and exposure to alcohol, undetermined intent	V01.Y98	Romania	20.21	2	yes
Y15	Poisoning by and exposure to alcohol, undetermined intent	V01.Y98	Sweden	20.89	2	yes

For peer review only

Y16	Poisoning by and exposure to organic solvents and halogenated hydrocarbons and their vapours, undetermined intent	V01.Y98	Hungary	6.00	1	yes
Y16	Poisoning by and exposure to organic solvents and halogenated hydrocarbons and their vapours, undetermined intent	V01.Y98	Poland	9.45	1	yes
Y17	Poisoning by and exposure to other gases and vapours, undetermined intent	V01.Y98	Austria	8.61	2	yes
Y17	Poisoning by and exposure to other gases and vapours, undetermined intent	V01.Y98	Czech Republic	5.26	2	yes
Y17	Poisoning by and exposure to other gases and vapours, undetermined intent	V01.Y98	Germany	31.92	0	no
Y17	Poisoning by and exposure to other gases and vapours, undetermined intent	V01.Y98	Lithuania	9.39	2	yes
Y17	Poisoning by and exposure to other gases and vapours, undetermined intent	V01.Y98	Poland	73.69	3	yes
Y17	Poisoning by and exposure to other gases and vapours, undetermined intent	V01.Y98	Romania	23.50	1	yes
Y17	Poisoning by and exposure to other gases and vapours, undetermined intent	V01.Y98	Spain	7.22	2	yes
Y17	Poisoning by and exposure to other gases and vapours, undetermined intent	V01.Y98	United Kingdom	18.93	1	yes
Y19	Poisoning by and exposure to other and unspecified chemicals and noxious substances, undetermined intent	V01.Y98	Germany	19.91	2	yes
Y19	Poisoning by and exposure to other and unspecified chemicals and noxious substances, undetermined intent	V01.Y98	Hungary	8.98	0	no
Y19	Poisoning by and exposure to other and unspecified chemicals and noxious substances, undetermined intent	V01.Y98	Lithuania	14.04	1	yes
Y19	Poisoning by and exposure to other and unspecified chemicals and noxious substances, undetermined intent	V01.Y98	Poland	36.82	1	yes
Y19	Poisoning by and exposure to other and unspecified chemicals and noxious substances, undetermined intent	V01.Y98	Romania	21.27	0	no
Y19	Poisoning by and exposure to other and unspecified chemicals and noxious substances, undetermined intent	V01.Y98	Spain	11.57	3	yes
Y20	Hanging, strangulation and suffocation, undetermined intent	V01.Y98	Belgium	11.90	2	yes
Y20	Hanging, strangulation and suffocation, undetermined intent	V01.Y98	Czech Republic	20.73	2	yes
Y20	Hanging, strangulation and suffocation, undetermined intent	V01.Y98	Estonia	6.12	3	yes
Y20	Hanging, strangulation and suffocation, undetermined intent	V01.Y98	France	29.46	2	yes
Y20	Hanging, strangulation and suffocation, undetermined intent	V01.Y98	Germany	230.95	2	yes
Y20	Hanging, strangulation and suffocation, undetermined intent	V01.Y98	Latvia	11.04	0	no
Y20	Hanging, strangulation and suffocation, undetermined intent	V01.Y98	Lithuania	41.19	3	yes
Y20	Hanging, strangulation and suffocation, undetermined intent	V01.Y98	Poland	388.18	3	yes
Y20	Hanging, strangulation and suffocation, undetermined intent	V01.Y98	Romania	18.49	1	yes
Y20	Hanging, strangulation and suffocation, undetermined intent	V01.Y98	United Kingdom	335.77	1	yes
Y21	Drowning and submersion, undetermined intent	V01.Y98	Austria	36.51	1	yes

For peer review only

Y21	Drowning and submersion, undetermined intent	V01.Y98	Belgium	28.20	0	no
Y21	Drowning and submersion, undetermined intent	V01.Y98	Czech Republic	20.75	0	no
Y21	Drowning and submersion, undetermined intent	V01.Y98	Denmark	8.70	1	yes
Y21	Drowning and submersion, undetermined intent	V01.Y98	Finland	16.92	0	no
Y21	Drowning and submersion, undetermined intent	V01.Y98	France	17.44	0	no
Y21	Drowning and submersion, undetermined intent	V01.Y98	Germany	158.08	2	yes
Y21	Drowning and submersion, undetermined intent	V01.Y98	Hungary	31.49	2	yes
Y21	Drowning and submersion, undetermined intent	V01.Y98	Latvia	13.72	0	no
Y21	Drowning and submersion, undetermined intent	V01.Y98	Lithuania	32.22	2	yes
Y21	Drowning and submersion, undetermined intent	V01.Y98	Netherlands	6.22	1	yes
Y21	Drowning and submersion, undetermined intent	V01.Y98	Poland	139.57	3	yes
Y21	Drowning and submersion, undetermined intent	V01.Y98	Romania	8.37	0	no
Y21	Drowning and submersion, undetermined intent	V01.Y98	Slovenia	6.14	0	no
Y21	Drowning and submersion, undetermined intent	V01.Y98	Spain	11.54	0	no
Y21	Drowning and submersion, undetermined intent	V01.Y98	Sweden	34.12	2	yes
Y21	Drowning and submersion, undetermined intent	V01.Y98	Switzerland	16.93	1	yes
Y21	Drowning and submersion, undetermined intent	V01.Y98	United Kingdom	192.63	2	yes
Y22	Handgun discharge, undetermined intent	V01.Y98	Czech Republic	8.21	0	no
Y22	Handgun discharge, undetermined intent	V01.Y98	Germany	27.76	1	yes
Y23	Rifle, shotgun and larger firearm discharge, undetermined intent	V01.Y98	France	25.27	1	yes
Y23	Rifle, shotgun and larger firearm discharge, undetermined intent	V01.Y98	United Kingdom	5.58	1	yes
Y24	Other and unspecified firearm discharge, undetermined intent	V01.Y98	Belgium	12.43	0	no
Y24	Other and unspecified firearm discharge, undetermined intent	V01.Y98	Czech Republic	5.62	0	no
Y24	Other and unspecified firearm discharge, undetermined intent	V01.Y98	France	262.05	3	yes
Y24	Other and unspecified firearm discharge, undetermined intent	V01.Y98	Germany	49.12	1	yes
Y24	Other and unspecified firearm discharge, undetermined intent	V01.Y98	Latvia	5.01	0	no
Y24	Other and unspecified firearm discharge, undetermined intent	V01.Y98	Lithuania	5.06	0	no
Y24	Other and unspecified firearm discharge, undetermined intent	V01.Y98	Poland	26.68	1	yes
Y24	Other and unspecified firearm discharge, undetermined intent	V01.Y98	Switzerland	8.52	1	yes
Y24	Other and unspecified firearm discharge, undetermined intent	V01.Y98	United Kingdom	6.47	1	yes
Y26	Exposure to smoke, fire and flames, undetermined intent	V01.Y98	Denmark	5.99	1	yes
Y26	Exposure to smoke, fire and flames, undetermined intent	V01.Y98	Germany	39.50	1	yes
Y26	Exposure to smoke, fire and flames, undetermined intent	V01.Y98	Hungary	7.02	1	yes
Y26	Exposure to smoke, fire and flames, undetermined intent	V01.Y98	Latvia	15.35	1	yes
Y26	Exposure to smoke, fire and flames, undetermined intent	V01.Y98	Lithuania	7.29	0	no
Y26	Exposure to smoke, fire and flames, undetermined intent	V01.Y98	Poland	9.71	0	no
Y26	Exposure to smoke, fire and flames, undetermined intent	V01.Y98	Romania	11.33	1	yes
Y26	Exposure to smoke, fire and flames, undetermined intent	V01.Y98	Sweden	13.13	1	yes

For peer review only

Y26	Exposure to smoke, fire and flames, undetermined intent	V01.Y98	United Kingdom	36.70	1	yes
Y28	Contact with sharp object, undetermined intent	V01.Y98	Czech Republic	5.48	0	no
Y28	Contact with sharp object, undetermined intent	V01.Y98	France	25.79	2	yes
Y28	Contact with sharp object, undetermined intent	V01.Y98	Germany	47.04	1	yes
Y28	Contact with sharp object, undetermined intent	V01.Y98	Latvia	8.50	0	no
Y28	Contact with sharp object, undetermined intent	V01.Y98	Lithuania	9.20	0	no
Y28	Contact with sharp object, undetermined intent	V01.Y98	Poland	66.57	2	yes
Y28	Contact with sharp object, undetermined intent	V01.Y98	Romania	9.96	2	yes
Y28	Contact with sharp object, undetermined intent	V01.Y98	United Kingdom	25.81	0	no
Y29	Contact with blunt object, undetermined intent	V01.Y98	Estonia	5.94	0	no
Y29	Contact with blunt object, undetermined intent	V01.Y98	Germany	7.62	0	no
Y29	Contact with blunt object, undetermined intent	V01.Y98	Hungary	6.79	3	yes
Y29	Contact with blunt object, undetermined intent	V01.Y98	Lithuania	91.20	2	yes
Y29	Contact with blunt object, undetermined intent	V01.Y98	Poland	60.70	3	yes
Y30	Falling, jumping or pushed from a high place, undetermined intent	V01.Y98	Austria	18.02	2	yes
Y30	Falling, jumping or pushed from a high place, undetermined intent	V01.Y98	Belgium	12.87	1	yes
Y30	Falling, jumping or pushed from a high place, undetermined intent	V01.Y98	Czech Republic	25.48	0	no
Y30	Falling, jumping or pushed from a high place, undetermined intent	V01.Y98	Estonia	8.11	3	yes
Y30	Falling, jumping or pushed from a high place, undetermined intent	V01.Y98	Finland	5.46	0	no
Y30	Falling, jumping or pushed from a high place, undetermined intent	V01.Y98	France	34.74	2	yes
Y30	Falling, jumping or pushed from a high place, undetermined intent	V01.Y98	Germany	100.68	3	yes
Y30	Falling, jumping or pushed from a high place, undetermined intent	V01.Y98	Hungary	20.24	2	yes
Y30	Falling, jumping or pushed from a high place, undetermined intent	V01.Y98	Latvia	21.63	1	yes
Y30	Falling, jumping or pushed from a high place, undetermined intent	V01.Y98	Lithuania	32.36	1	yes
Y30	Falling, jumping or pushed from a high place, undetermined intent	V01.Y98	Poland	155.43	1	yes
Y30	Falling, jumping or pushed from a high place, undetermined intent	V01.Y98	Romania	37.67	3	yes
Y30	Falling, jumping or pushed from a high place, undetermined intent	V01.Y98	Spain	21.55	1	yes
Y30	Falling, jumping or pushed from a high place, undetermined intent	V01.Y98	Sweden	13.61	0	no
Y30	Falling, jumping or pushed from a high place, undetermined intent	V01.Y98	Switzerland	8.50	0	no
Y30	Falling, jumping or pushed from a high place, undetermined intent	V01.Y98	United Kingdom	78.56	1	yes
Y31	Falling, lying or running before or into moving object, undetermined intent	V01.Y98	Austria	9.77	2	yes
Y31	Falling, lying or running before or into moving object, undetermined intent	V01.Y98	France	17.68	1	yes
Y31	Falling, lying or running before or into moving object, undetermined intent	V01.Y98	Germany	51.99	1	yes
Y31	Falling, lying or running before or into moving object, undetermined intent	V01.Y98	Hungary	15.44	2	yes
Y31	Falling, lying or running before or into moving object, undetermined intent	V01.Y98	Sweden	8.71	0	no

For peer review only

Y31	Falling, lying or running before or into moving object, undetermined intent	V01.Y98	Switzerland	5.16	1	yes
Y31	Falling, lying or running before or into moving object, undetermined intent	V01.Y98	United Kingdom	30.76	3	yes
Y32	Crashing of motor vehicle, undetermined intent	V01.Y98	Finland	8.88	0	no
Y32	Crashing of motor vehicle, undetermined intent	V01.Y98	Sweden	12.84	0	no
Y33	Other specified events, undetermined intent	V01.Y98	Estonia	16.62	1	yes
Y33	Other specified events, undetermined intent	V01.Y98	Germany	29.16	0	no
Y33	Other specified events, undetermined intent	V01.Y98	Latvia	5.30	1	yes
Y33	Other specified events, undetermined intent	V01.Y98	Lithuania	5.21	1	yes
Y33	Other specified events, undetermined intent	V01.Y98	Poland	6.64	1	yes
Y33	Other specified events, undetermined intent	V01.Y98	Romania	7.41	0	no
Y33	Other specified events, undetermined intent	V01.Y98	Sweden	7.84	0	no
Y33	Other specified events, undetermined intent	V01.Y98	United Kingdom	354.78	2	yes
Y34	Unspecified event, undetermined intent	V01.Y98	Austria	5.56	2	yes
Y34	Unspecified event, undetermined intent	V01.Y98	Belgium	123.55	1	yes
Y34	Unspecified event, undetermined intent	V01.Y98	Czech Republic	218.38	3	yes
Y34	Unspecified event, undetermined intent	V01.Y98	Denmark	9.14	3	yes
Y34	Unspecified event, undetermined intent	V01.Y98	Estonia	29.46	0	no
Y34	Unspecified event, undetermined intent	V01.Y98	Finland	23.81	0	no
Y34	Unspecified event, undetermined intent	V01.Y98	France	67.22	1	yes
Y34	Unspecified event, undetermined intent	V01.Y98	Germany	1120.42	2	yes
Y34	Unspecified event, undetermined intent	V01.Y98	Hungary	27.69	0	no
Y34	Unspecified event, undetermined intent	V01.Y98	Latvia	80.54	2	yes
Y34	Unspecified event, undetermined intent	V01.Y98	Lithuania	99.08	0	no
Y34	Unspecified event, undetermined intent	V01.Y98	Netherlands	30.67	1	yes
Y34	Unspecified event, undetermined intent	V01.Y98	Poland	1014.04	3	yes
Y34	Unspecified event, undetermined intent	V01.Y98	Romania	111.90	1	yes
Y34	Unspecified event, undetermined intent	V01.Y98	Slovenia	35.66	1	yes
Y34	Unspecified event, undetermined intent	V01.Y98	Spain	9.95	1	yes
Y34	Unspecified event, undetermined intent	V01.Y98	Sweden	7.86	0	no
Y34	Unspecified event, undetermined intent	V01.Y98	Switzerland	9.06	0	no
Y34	Unspecified event, undetermined intent	V01.Y98	United Kingdom	167.87	1	yes
Y40	Systemic antibiotics	V01.Y98	France	12.52	0	no
Y40	Systemic antibiotics	V01.Y98	Germany	5.98	1	yes
Y40	Systemic antibiotics	V01.Y98	United Kingdom	7.29	1	yes
Y42	Hormones, and their synthetic substitutes and antagonists, not elsewhere classified	V01.Y98	France	8.67	2	yes
Y43	Primarily systemic agents	V01.Y98	France	6.93	0	no
Y44	Agents primarily affecting blood constituents	V01.Y98	Belgium	6.12	0	no
Y44	Agents primarily affecting blood constituents	V01.Y98	France	546.05	3	yes
Y44	Agents primarily affecting blood constituents	V01.Y98	Germany	14.88	0	no
Y44	Agents primarily affecting blood constituents	V01.Y98	Poland	47.71	2	yes
Y44	Agents primarily affecting blood constituents	V01.Y98	Slovenia	8.13	3	yes
Y44	Agents primarily affecting blood constituents	V01.Y98	Spain	45.45	3	yes
Y44	Agents primarily affecting blood constituents	V01.Y98	United Kingdom	10.41	0	no
Y45	Analgesics, antipyretics and anti-inflammatory drugs	V01.Y98	France	40.93	2	yes
Y45	Analgesics, antipyretics and anti-inflammatory drugs	V01.Y98	Germany	8.99	0	no
Y45	Analgesics, antipyretics and anti-inflammatory drugs	V01.Y98	Spain	11.61	2	yes
Y45	Analgesics, antipyretics and anti-inflammatory drugs	V01.Y98	United Kingdom	6.35	1	yes
Y48	Anaesthetics and therapeutic gases	V01.Y98	France	30.90	3	yes

For peer review only

Y49	Psychotropic drugs, not elsewhere classified	V01.Y98	France	8.77	0	no
Y52	Agents primarily affecting the cardiovascular system	V01.Y98	France	17.43	1	yes
Y52	Agents primarily affecting the cardiovascular system	V01.Y98	Spain	62.86	2	yes
Y54	Agents primarily affecting water-balance and mineral and uric acid metabolism	V01.Y98	France	15.12	1	yes
Y57	Other and unspecified drugs and medicaments	V01.Y98	Belgium	5.85	1	yes
Y57	Other and unspecified drugs and medicaments	V01.Y98	France	48.67	0	no
Y57	Other and unspecified drugs and medicaments	V01.Y98	Germany	10.95	0	no
Y57	Other and unspecified drugs and medicaments	V01.Y98	Spain	22.20	1	yes
Y57	Other and unspecified drugs and medicaments	V01.Y98	United Kingdom	17.41	2	yes
Y60	Unintentional cut, puncture, perforation or haemorrhage during surgical and medical care	V01.Y98	Austria	21.16	2	yes
Y60	Unintentional cut, puncture, perforation or haemorrhage during surgical and medical care	V01.Y98	Denmark	18.12	0	no
Y60	Unintentional cut, puncture, perforation or haemorrhage during surgical and medical care	V01.Y98	France	35.65	0	no
Y60	Unintentional cut, puncture, perforation or haemorrhage during surgical and medical care	V01.Y98	Germany	104.83	3	yes
Y60	Unintentional cut, puncture, perforation or haemorrhage during surgical and medical care	V01.Y98	Hungary	9.40	0	no
Y60	Unintentional cut, puncture, perforation or haemorrhage during surgical and medical care	V01.Y98	Poland	8.31	0	no
Y60	Unintentional cut, puncture, perforation or haemorrhage during surgical and medical care	V01.Y98	Spain	10.29	1	yes
Y60	Unintentional cut, puncture, perforation or haemorrhage during surgical and medical care	V01.Y98	United Kingdom	42.18	2	yes
Y62	Failure of sterile precautions during surgical and medical care	V01.Y98	Germany	83.42	0	no
Y65	Other misadventures during surgical and medical care	V01.Y98	Denmark	5.57	1	yes
Y65	Other misadventures during surgical and medical care	V01.Y98	France	7.20	0	no
Y65	Other misadventures during surgical and medical care	V01.Y98	Germany	56.41	0	no
Y69	Unspecified misadventure during surgical and medical care	V01.Y98	France	9.94	2	yes
Y69	Unspecified misadventure during surgical and medical care	V01.Y98	Germany	29.04	0	no
Y71	Cardiovascular devices associated with adverse incidents	V01.Y98	Germany	7.88	1	yes
Y71	Cardiovascular devices associated with adverse incidents	V01.Y98	Spain	16.02	2	yes
Y73	Gastroenterology and urology devices associated with adverse incidents	V01.Y98	Germany	7.80	0	no
Y83	Surgical operation and other surgical procedures as the cause of abnormal reaction of the patient, or of later complication, without mention of misadventure at the time of procedure	V01.Y98	Austria	110.35	3	yes
Y83	Surgical operation and other surgical procedures as the cause of abnormal reaction of the patient, or of later complication, without mention of misadventure at the time of procedure	V01.Y98	Belgium	85.71	2	yes
Y83	Surgical operation and other surgical procedures as the cause of abnormal reaction of the patient, or of later complication, without mention of misadventure at the time of procedure	V01.Y98	Croatia	28.51	2	yes

For peer review only

Y83	Surgical operation and other surgical procedures as the cause of abnormal reaction of the patient, or of later complication, without mention of misadventure at the time of procedure	V01.Y98	Czech Republic	33.14	2	yes
Y83	Surgical operation and other surgical procedures as the cause of abnormal reaction of the patient, or of later complication, without mention of misadventure at the time of procedure	V01.Y98	Estonia	6.36	3	yes
Y83	Surgical operation and other surgical procedures as the cause of abnormal reaction of the patient, or of later complication, without mention of misadventure at the time of procedure	V01.Y98	France	445.20	2	yes
Y83	Surgical operation and other surgical procedures as the cause of abnormal reaction of the patient, or of later complication, without mention of misadventure at the time of procedure	V01.Y98	Germany	615.33	3	yes
Y83	Surgical operation and other surgical procedures as the cause of abnormal reaction of the patient, or of later complication, without mention of misadventure at the time of procedure	V01.Y98	Latvia	8.20	1	yes
Y83	Surgical operation and other surgical procedures as the cause of abnormal reaction of the patient, or of later complication, without mention of misadventure at the time of procedure	V01.Y98	Lithuania	13.70	0	no
Y83	Surgical operation and other surgical procedures as the cause of abnormal reaction of the patient, or of later complication, without mention of misadventure at the time of procedure	V01.Y98	Netherlands	27.57	0	no
Y83	Surgical operation and other surgical procedures as the cause of abnormal reaction of the patient, or of later complication, without mention of misadventure at the time of procedure	V01.Y98	Poland	308.00	0	no
Y83	Surgical operation and other surgical procedures as the cause of abnormal reaction of the patient, or of later complication, without mention of misadventure at the time of procedure	V01.Y98	Romania	17.79	0	no
Y83	Surgical operation and other surgical procedures as the cause of abnormal reaction of the patient, or of later complication, without mention of misadventure at the time of procedure	V01.Y98	Slovenia	78.14	0	no
Y83	Surgical operation and other surgical procedures as the cause of abnormal reaction of the patient, or of later complication, without mention of misadventure at the time of procedure	V01.Y98	Spain	230.79	1	yes
Y83	Surgical operation and other surgical procedures as the cause of abnormal reaction of the patient, or of later complication, without mention of misadventure at the time of procedure	V01.Y98	Sweden	84.58	2	yes
Y83	Surgical operation and other surgical procedures as the cause of abnormal reaction of the patient, or of later complication, without mention of misadventure at the time of procedure	V01.Y98	Switzerland	6.03	0	no
Y83	Surgical operation and other surgical procedures as the cause of abnormal reaction of the patient, or of later complication, without mention of misadventure at the time of procedure	V01.Y98	United Kingdom	452.11	2	yes
Y84	Other medical procedures as the cause of abnormal reaction of the patient, or of later complication, without mention of misadventure at the time of the procedure	V01.Y98	Austria	7.75	1	yes

For peer review only

Y84	Other medical procedures as the cause of abnormal reaction of the patient, or of later complication, without mention of misadventure at the time of the procedure	V01.Y98	Belgium	10.45	2	yes
Y84	Other medical procedures as the cause of abnormal reaction of the patient, or of later complication, without mention of misadventure at the time of the procedure	V01.Y98	Czech Republic	13.26	2	yes
Y84	Other medical procedures as the cause of abnormal reaction of the patient, or of later complication, without mention of misadventure at the time of the procedure	V01.Y98	France	32.79	2	yes
Y84	Other medical procedures as the cause of abnormal reaction of the patient, or of later complication, without mention of misadventure at the time of the procedure	V01.Y98	Germany	71.53	3	yes
Y84	Other medical procedures as the cause of abnormal reaction of the patient, or of later complication, without mention of misadventure at the time of the procedure	V01.Y98	Poland	9.46	0	no
Y84	Other medical procedures as the cause of abnormal reaction of the patient, or of later complication, without mention of misadventure at the time of the procedure	V01.Y98	Spain	30.78	0	no
Y84	Other medical procedures as the cause of abnormal reaction of the patient, or of later complication, without mention of misadventure at the time of the procedure	V01.Y98	Sweden	23.38	0	no
Y84	Other medical procedures as the cause of abnormal reaction of the patient, or of later complication, without mention of misadventure at the time of the procedure	V01.Y98	United Kingdom	101.34	2	yes
Y85	Sequelae of transport accidents	V01.Y98	Austria	9.32	0	no
Y85	Sequelae of transport accidents	V01.Y98	Belgium	6.97	0	no
Y85	Sequelae of transport accidents	V01.Y98	Finland	21.29	0	no
Y85	Sequelae of transport accidents	V01.Y98	France	50.42	0	no
Y85	Sequelae of transport accidents	V01.Y98	Germany	29.05	1	yes
Y85	Sequelae of transport accidents	V01.Y98	Netherlands	33.27	0	no
Y85	Sequelae of transport accidents	V01.Y98	Poland	15.08	0	no
Y85	Sequelae of transport accidents	V01.Y98	Spain	15.34	0	no
Y85	Sequelae of transport accidents	V01.Y98	Sweden	13.50	0	no
Y85	Sequelae of transport accidents	V01.Y98	United Kingdom	45.92	1	yes
Y86	Sequelae of other accidents	V01.Y98	Belgium	14.69	0	no
Y86	Sequelae of other accidents	V01.Y98	Denmark	7.58	1	yes
Y86	Sequelae of other accidents	V01.Y98	Finland	54.77	0	no
Y86	Sequelae of other accidents	V01.Y98	France	150.78	2	yes
Y86	Sequelae of other accidents	V01.Y98	Germany	56.87	3	yes
Y86	Sequelae of other accidents	V01.Y98	Hungary	18.28	1	yes
Y86	Sequelae of other accidents	V01.Y98	Lithuania	29.26	3	yes
Y86	Sequelae of other accidents	V01.Y98	Netherlands	380.47	2	yes
Y86	Sequelae of other accidents	V01.Y98	Norway	39.31	2	yes
Y86	Sequelae of other accidents	V01.Y98	Poland	79.98	0	no
Y86	Sequelae of other accidents	V01.Y98	Spain	25.39	1	yes
Y86	Sequelae of other accidents	V01.Y98	Sweden	68.72	1	yes
Y86	Sequelae of other accidents	V01.Y98	Switzerland	24.62	0	no
Y86	Sequelae of other accidents	V01.Y98	United Kingdom	79.24	2	yes

For peer review only

Y87	Sequelae of intentional self-harm, assault and events of undetermined intent	V01.Y98	Finland	5.69	0	no
Y87	Sequelae of intentional self-harm, assault and events of undetermined intent	V01.Y98	France	14.87	0	no
Y87	Sequelae of intentional self-harm, assault and events of undetermined intent	V01.Y98	Germany	37.82	0	no
Y87	Sequelae of intentional self-harm, assault and events of undetermined intent	V01.Y98	Poland	12.74	0	no
Y87	Sequelae of intentional self-harm, assault and events of undetermined intent	V01.Y98	United Kingdom	12.95	2	yes
Y88	Sequelae of surgical and medical care as external cause	V01.Y98	France	17.90	0	no
Y88	Sequelae of surgical and medical care as external cause	V01.Y98	Germany	17.80	1	yes
Y88	Sequelae of surgical and medical care as external cause	V01.Y98	Poland	5.88	0	no
Y88	Sequelae of surgical and medical care as external cause	V01.Y98	United Kingdom	7.91	3	yes
Y89	Sequelae of other external causes	V01.Y98	Austria	53.70	3	yes
Y89	Sequelae of other external causes	V01.Y98	France	9.22	1	yes
Y89	Sequelae of other external causes	V01.Y98	Germany	44.34	1	yes
Y89	Sequelae of other external causes	V01.Y98	Poland	112.91	2	yes
Y89	Sequelae of other external causes	V01.Y98	Spain	6.41	1	yes

For peer review only

BMJ Open

How large should a cause of death be in order to be included in mortality trend analysis? deriving a cut-off point from retrospective trend analyses in 21 European countries

Journal:	*BMJ Open*
Manuscript ID	bmjopen-2019-031702.R2
Article Type:	Original research
Date Submitted by the Author:	17-Dec-2019
Complete List of Authors:	Mitratza, Marianna; Amsterdam UMC, University of Amsterdam, Department of Public Health<br>Kardaun, Jan; Statistics Netherlands, Department of Health and Care, ; Amsterdam UMC, University of Amsterdam, Department of Public Health<br>Kunst, Anton; Amsterdam UMC, University of Amsterdam, Department of Public Health
**Primary Subject Heading**:	Epidemiology
Secondary Subject Heading:	Public health, Research methods
Keywords:	mortality, trends, cause of death, size criterion, Europe, EPIDEMIOLOGY

SCHOLARONE™
Manuscripts

Title

How large should a cause of death be in order to be included in mortality trend analysis? deriving a cut-off point from retrospective trend analyses in 21 European countries

Authors

Marianna Mitratza, Department of Public Health, Amsterdam UMC, University of Amsterdam, Amsterdam, the Netherlands

Jan WPF Kardaun, Department of Health and Care, Statistics Netherlands, The Hague, the Netherlands
& Department of Public Health, Amsterdam UMC, University of Amsterdam, Amsterdam, the Netherlands

Anton E Kunst, Department of Public Health, Amsterdam UMC, University of Amsterdam, Amsterdam, the Netherlands

Author responsible for correspondence about the manuscript

Marianna Mitratza

Address: Meibergdreef 9, 1105 AZ Amsterdam, Netherlands

Telephone : +31205664658

Email : m.mitratza@amsterdamumc.nl

Disclaimers

None

Funding

For peer review only

None

Word count

2907

Number of figures and tables

2 tables, 3 figures

For peer review only

For peer review only - http://bmjopen.bmj.com/site/about/guidelines.xhtml

Abstract

Objectives: The International Classification of Diseases (ICD-10) distinguishes a large number of causes of death (CODs) that could each be studied individually when monitoring time-trends. We aimed to develop recommendations for using the size of CODs as a criterion for their inclusion in long-term trend analysis.

Design: Retrospective trend analysis

Setting: 21 European countries of the WHO Mortality Database

Participants: Deaths from causes of death (3-position ICD-10 codes) with ≥5 average annual deaths in a 15-year period between 2000 and 2016

Primary and secondary outcome measures: Fitting polynomial regression models, we examined for each COD in each country whether or not changes over time were statistically significant (with α=0.05) and we assessed correlates of this outcome. Applying ROC curve diagnostics, we derived COD size thresholds for selecting CODs for trends analysis.

Results: Across all countries, 64.0% of CODs had significant long-term trends. The odds of having a significant trend increased by 18% for every 10% increase of COD size. The independent effect of country was negligible. As compared to circulatory system diseases, the probability of a significant trend was lower for neoplasms and digestive system diseases, and higher for infectious diseases, mental diseases and signs-and-symptoms. We derived a general threshold of around 30 (range: 28-33) annual deaths for inclusion of a COD in trend analysis. The relevant threshold for neoplasms was around 65 (range: 61-70) and for infectious diseases was 20 (range: 19-20).

For peer review only

Conclusions: The likelihood that long-term trends are detected with statistical significance is strongly related to COD size and varies between ICD-10 chapters, but has no independent relation to country. We recommend a general size criterion of 30 annual deaths to select CODs for long-term mortality-trends analysis in European countries.

Strengths and limitations of this study

- The first study to develop a criterion to select causes of death for monitoring purposes based on their annual number of deaths.
- The analysis of a large sample of causes of death covering most European countries, using the WHO Mortality Database.
- Criteria for selection of causes were derived for different types of causes of death.
- Other criteria were not applied, such as causes of death that involve high healthcare costs or that are potentially modifiable.

Introduction

Mortality data are essential for the monitoring of population-wide trends in a large number of diseases and injuries, as well as for the evaluation of health policies. A common source for these data are the statistics maintained by national statistical offices **[1,2]**. National statistics of causes of death (CODs) include many codes of the10th revision of the International Classification of Diseases (ICD-10 codes) **[3].** Given the detail of this classification - there are 1,752 3-position ICD-10 codes - a part of it may not be instrumental for monitoring long-term time-trends due to the small number of deaths for specific codes.

When using these statistics to monitor long-term trends in mortality, a main question is which of the many possible CODs to include. At the very least, the selection should include only

For peer review only - http://bmjopen.bmj.com/site/about/guidelines.xhtml

CODs that are large enough to have a reasonable probability of detecting a long-term mortality trend. This probability may be influenced by several factors. One main factor is the COD size, defined as the mean annual number of deaths, which expresses the rarity of a disease or condition that is selected as underlying cause of death in a population. Incidence changes or effects of interventions are common factors discussed in mortality trends analyses **[4,5]**. In addition, this probability might depend on other factors, such as the type of COD, or the country of interest. Certain types of CODs may be more likely to present a long-term trend. For example, neoplasms have been shown to be more gradual in their annual changes **[6]**, whereas infectious diseases **[7]** may have high year-to-year variation. As regards to different populations, the likelihood to detect a long-term trend for a COD may vary between countries because of differences in population size, COD coding practices that may also influence observed mortality trends **[8]**, trends in prevalence of risk factors **[9,10,11,12]**, implementation of new prevention strategies **[13,14]**, treatment protocols **[5]** or healthcare reforms **[15]**.

Due to the fact that the likelihood to detect a long-term trend of a COD may depend on various factors, there is a need for an empirical assessment of such likelihood. Such analysis may provide an empirical basis for the identification of CODs for which long-trends are likely to be detectable. More specifically, it may be used to define a criterion, or rule of thumb, that identifies eligible CODs in terms of a minimum COD size. When such a criterion allows for variation by COD type and country, it may be used in national and international trend analysis across a broad range of CODs.

The general objective of this study was to determine a COD size criterion for the study of long-term mortality trends in European countries. The specific objectives were: (1) to assess the association between the size and the type of a COD and the probability of detecting a long-term trend in European countries, (2) to assess how this association varies according to country, and (3) to identify a minimum annual number of deaths recommended to monitor trends in cause-specific mortality.

For peer review only - http://bmjopen.bmj.com/site/about/guidelines.xhtml

Methods

Data

We used annual mortality data for 21 European countries of the WHO Mortality Database (1 October 2017 update) **[16]**. We included the 21 countries of the European Union (28 countries) or the European Free Trade Association (4 countries) that had been using ICD-10 (3- or 4-position) coding for at least 15 consecutive years. Iceland, Luxemburg and Malta were excluded because of their small population **[17]**. The most recent 15-year period was selected, which was 2001-2015 for all countries with few exceptions (Belgium, France and Switzerland: 2000-2014; Austria: 2002-2016). If the time series of a COD in a country was interrupted by a year without any data on that COD, we assumed that zero cases occurred.

Statistical analysis

For each year and COD in a country, we calculated an age-standardized count of deaths using the direct method. As reference population, we used the age-distribution of the European Standard Population 2013, scaled to the mid-period population of each country. This method intended to compensate for annual changes in the age-distribution of the population, while keeping the age-standardized count close to the observed absolute numbers.

For further analysis, we analyzed CODs that had at least 5 average age-standardized annual deaths, because most of the smaller CODs had predominantly zero or only zero annual deaths.

Long-term time-trends of the age-standardized count of deaths of each COD in each country were analyzed using ordinary least squares regression models. Trends were fitted by applying

For peer review only - http://bmjopen.bmj.com/site/about/guidelines.xhtml

linear regression models with polynomial terms of year as continuous, independent covariates **[18]**. We used orthogonal polynomials in order to account for multicollinearity of the polynomial components **[19]**. We fitted four models: the constant, the linear, the quadratic and the cubic model (with zero, first, second and third degree polynomials, respectively). The four models were applied for all CODs in each country. We used the lowest corrected Akaike Information Criterion (AICc) to select the best model for each COD in each country **[20]**. In a next step, the best model was compared with the constant model using the F-test, at the significance level of $\alpha=0.05$. If the best model performed better than the constant model with statistical significance, it was kept as the final best model. Otherwise, the constant model was selected as the best model for this COD. In the rest of the paper, the constant model is referred to as the absence of a demonstrable trend.

Next, using a multilevel logistic regression model, we determined how the categorization of a COD as having a statistically significant trend (i.e. best model being the linear, quadratic or cubic model) was related to COD size and COD type. These variables were included in the model as fixed effects. The COD size was defined as the mean annual number of deaths and the COD type was defined as the ICD-10 chapter in which it is classified. The chapter of circulatory diseases was the reference category, as it had the largest number of deaths. As the distribution of the number of deaths across CODs was highly skewed, we used its natural logarithm as a measure of COD size. The model also included the level of countries as random effect, in order to investigate the variation of European countries in the likelihood of detecting a long-term trend. We calculated the Intra-class Correlation Coefficient (ICC), which expresses the proportion of the variance in the outcome that is attributable to variations between the countries [**21**]. The ICC was calculated both with and without controlling for the fixed effects of the size and type of the COD.

Finally, we used receiver operating characteristic (ROC) curve diagnostics **[22,23,24]** to derive COD size thresholds for detecting a long-term time-trend. We calculated the Area Under the

For peer review only - http://bmjopen.bmj.com/site/about/guidelines.xhtml

Curve (AUC) of the logistic model with COD size as the predictor and the binary categorization of a COD as having a significant long-term time-trend as the outcome. We derived the COD size thresholds using three indices. Firstly, we used the maximum Youden index **[25,26,27]**, which represents the point of the ROC curve with the maximum sum of sensitivity (se) and specificity (sp). Secondly, we used the index measuring the minimum difference between sensitivity and specificity **[23]**. Thirdly, we estimated the index that represents the point closest to the top-left part of the ROC curve **[22,26]**.

All analyses were conducted using R statistical software (3.5.1 version) **[28]**.

Patient and Public Involvement

No patients were involved in this study.

Results

The number of CODs with at least 5 annual deaths on average varied between 202 (Estonia) and 791 (Germany) **(Table 1)**. Of these CODs, 32.6%, 20.2% and 11.2% had a significant trend following a linear, quadratic or cubic model respectively. The percentage of CODs with no significant trend (i.e. constant model) varied from 27.5% to 43.9%, and was highest in the Nordic countries, Switzerland and Slovenia. More detailed information on the best model for each COD in each European country can be found in an additional file **(Online Resource 1)**.

For peer review only

For peer review only - http://bmjopen.bmj.com/site/about/guidelines.xhtml

Table 1:

Frequencies of causes of deaths according to estimates of their long-term time-trend in 21 European countries

Country (sorted by mean population)	CODs* analyzed (n)	Type of long-term time-trend (%)				Mean population (thousands)	Mean crude annual deaths from all CODs together	Mean crude annual deaths from CODs analyzed*
		No significant trend	Linear	Quadratic	Cubic			
**Estonia**	202	34.7	35.6	15.3	14.4	1,343	16,642	16,006
**Slovenia**	228	43.9	29.4	17.1	9.6	2,029	18,830	18,163
**Latvia**	259	38.2	28.6	17.0	16.2	2,157	30,840	30,091
**Lithuania**	308	37.7	32.5	19.5	10.4	3,187	42,101	41,243
**Croatia**	315	34.6	37.1	15.9	12.4	4,381	51,444	50,844
**Norway**	356	42.4	26.4	23.6	7.6	4,804	41,764	40,886
**Finland**	355	42.8	35.2	16.6	5.4	5,302	49,869	48,933
**Denmark**	420	40.0	37.4	13.6	9.0	5,497	54,446	53,319
**Switzerland**	451	43.9	31.0	16.4	8.6	7,558	62,183	61,384
**Austria**	374	35.8	25.4	23.5	15.2	8,359	77,256	75,765
**Sweden**	457	43.3	30.6	19.5	6.6	9,259	91,504	90,516
**Hungary**	482	36.1	27.6	29.3	7.1	10,024	130,830	129,695
**Czech Republic**	443	31.8	35.9	23.0	9.3	10,383	107,448	106,636

For peer review only

**Belgium**	517	37.3	33.7	20.9	8.1	10,678	104,344	103,257
**Netherlands**	554	33.9	28.0	25.1	13.0	16,489	138,373	137,645
**Romania**	444	31.8	36.0	19.4	12.8	21,594	258,000	257,265
**Poland**	581	27.7	33.2	22.9	16.2	38,114	375,231	374,295
**Spain**	672	33.0	33.9	18.2	14.9	44,594	385,512	383,204
**United Kingdom**	710	30.8	33.8	21.1	14.2	61,690	580,733	578,572
**France**	738	27.5	38.8	22.6	11.1	61,709	535,320	532,293
**Germany**	791	27.8	35.4	24.7	12.1	81,980	852,566	849,400

* coded at ICD-10, 3-position level; including all causes of death with at least 5 mean number of deaths in the 15-year-period

Both COD size and COD type were significantly associated with the likelihood of having a significant long-term trend (p-value <0.001) **(Table 2)**. For every 10% increase in the COD size, we observed a 18% increase (1.1^1.73=0.18) in the odds of having a significant trend (OR=1.73, 95%CI=1.67;1.79). Regarding the COD type, neoplasms and digestive system diseases had lower probability for detecting a trend in comparison to the circulatory system diseases. On the other hand, this probability was higher for infectious diseases, mental diseases and signs-and-symptoms. **Fig1** shows for each COD chapter in each country the estimated probability of having a significant long-term trend in relation to COD size. The variation between COD chapters was substantial, irrespective of COD size. Neoplasms (chapter C00.D48) as a group of CODs showed the lowest probability of having a detectable trend.

We found only small variation of countries in the likelihood of detecting a long-term trend, as the ICC for the country-level random effect was only 0.013 (without fixed effects for chapter and size) and 0.003 (with fixed effects) **(Table 2)**. **Fig2** illustrates the small differences between countries in the estimated probability of having a long-term trend.

For peer review only

For peer review only - http://bmjopen.bmj.com/site/about/guidelines.xhtml

Table 2:

Relationship between the likelihood for a cause of death (COD) to have a significant long-term trend with its size, corresponding ICD-10 chapter, and country

**COD characteristic**		**Number of CODs**	**Total number of deaths**	**Odds Ratio*** (95% Confidence Interval)**
**Size**				
log(mean deaths*)			-	**1.73** (1.67 ; 1.79)
**ICD10 Chapter**				
I00.I99	Diseases of the circulatory system	988	23,610,116	reference
A00.B99	Certain infectious and parasitic diseases	454	823,839	**1.63** (1.25 ; 2.14)
C00.D48	Neoplasms	1871	15,632,543	**0.57** (0.47 ; 0.69)
D50.D89	Diseases of blood and blood-forming organs and certain disorders involving the immune mechanisms	217	143,156	0.82 (0.59 ; 1.14)
E00.E90	Endocrine, nutritional and metabolic diseases	365	1,547,431	1.01 (0.76 ; 1.34)
F00.F99	Mental, behavioural disorders	230	1,725,881	**1.62** (1.13 ; 2.30)
G00.G99	Diseases of the nervous system	536	1,888,629	0.90 (0.70 ; 1.15)
J00.J99	Diseases of the respiratory system	553	4,740,481	1.22 (0.94 ; 1.58)
K00.K93	Diseases of the digestive system	767	2,816,168	**0.75** (0.59 ; 0.94)
M00.M99	Diseases of the musculoskeletal system and connective tissue	367	283,108	1.14 (0.86 ; 1.50)
N00.N99	Diseases of the genitourinary system	385	1,029,118	0.98 (0.74 ; 1.29)
Q00.Q99	Congenital malformations, deformations and chromosomal abnormalities	325	141,848	0.89 (0.67 ; 1.19)

For peer review only

For peer review only - http://bmjopen.bmj.com/site/about/guidelines.xhtml

R00.R99	Symptoms, signs and abnormal clinical and laboratory findings, not elsewhere classified (signs-and-symptoms)	258	2,062,587	**1.68** (1.18 ; 2.38)
V01.Y98	External causes of morbidity and mortality	1992	3,005,539	1.20 (0.99 ; 1.46)
Other**		349	240,748	1.29 (0.97 ; 1.71)
**Intra-class correlation for the country level**				
model with fixed effects for size and ICD10 chapter			0.003	
model with no fixed effects			0.013	

*mean deaths : mean of the annual number of deaths for a cause of death monitored in the 15-year-period, measured per country. Only including CODs with 5 or more deaths.

** “Other” consists of the causes of death classified in the ICD-10 chapters H00.H59: Diseases of the eye and adnexa, H60.H95: Diseases of the ear and mastoid process, L00.L99: Diseases of the skin and subcutaneous tissue, O00.O99: Pregnancy, childbirth and the puerperium and P00.P96: Certain conditions originating in the perinatal period.

***odds ratios in bold were statistically significant with p-value < 0.05

Fig3a describes the sensitivity (se) and specificity (sp) for detecting a significant long-term trend using different levels of thresholds in terms of any COD size. The AUC corresponding to these se and sp values was 0.706, with 95%CI:0.695;0.716. The maximized sum index (Youden Index) was 32.7 annual deaths, with sensitivity (se) 61.4% and specificity (sp) 70.3%. The minimum difference index was 27.5 annual deaths (se= 65.5%, sp= 65.5%). The closest top-left index was 29.4 annual deaths (se= 64.0%, sp= 67.5%) (Figure 3a).

The corresponding analysis for the neoplasms yielded a similar ROC curve (AUC= 0.703, 95%CI:0.680;0.727) **(Fig3b)**. The Youden Index was 70.4, with sensitivity 61.5% and specificity 69.6%, and the minimum difference index was 60.5 annual deaths (se= 64.8%, sp= 64.8%). The closest top-left index was also 60.5 annual deaths (se= 64.1%, sp= 66.9%). Infectious and parasitic diseases (AUC= 0.706, 95%CI:0.695;0.716) yielded a Youden Index

For peer review only

of 19.7 annual deaths, with sensitivity 67.1% and specificity 69.8%. The closest top-left index was identical, while the minimum difference threshold was 19.3 annual deaths (se= 67.4%, sp= 67.4%) **(Fig3c)**.

Discussion

COD data are used in widely varying settings, ranging from detailed mortality profiles to macro estimates. Applications include studies in localized areas **[29]**, single countries **[30]** or worldwide **[2,31]**; for a single-disease **[32,33]** or disease group **[9]**; monitored for days or a long-term period **[7]**; for specific age groups **[33,34]** or specific situations (e.g. maternal mortality **[5]**, external causes **[35,36,37]**). These settings all impose different requirements on the collected data. Here we focused on one particular application: national estimates of mortality time trends for a reasonably long period (15 years), for a considerable number of countries (21) that have quite comparable CODs data collection and registration systems **[38]**, covering as many CODs as possible. Our aim was to investigate the effect of the size of a COD on the probability to detect a significant trend, and how this is related to country and type of COD (ICD-10 chapter).

Our results indicate that both the size and the type of a cause of death were associated with the probability of detecting a significant trend, while variations among European countries were negligible. Some types of CODs, particularly neoplasms and digestive system diseases, had a lower probability for detecting a significant trend in comparison to the circulatory system diseases, whereas infectious and mental diseases had a higher probability. The results suggest a general size criterion of 30 annual deaths for selecting causes of death to include in long-term mortality trends analysis, and a more specific criterion of 65 deaths for neoplasms and 20 for infectious diseases.

For peer review only - http://bmjopen.bmj.com/site/about/guidelines.xhtml

We should outline the limitations of our study. Firstly, due to the exclusion of causes of death with less than 5 annual deaths on average, smaller countries were represented in our analysis with fewer causes of death. However, this is unlikely to have a strong influence on the results, as the suggested COD size threshold of about 30 deaths is much higher than the lower limit of 5 mean annual deaths. Secondly, although we proposed the COD size as a criterion to select CODs for long-term trend analysis, we acknowledge that other criteria could be used, such as greater preference to CODs that involve high healthcare costs or that are potentially modifiable by preventive or curative actions. Thirdly, the likelihood to demonstrate a time trend with statistical significance depends on the statistical method that is used to describe these trends. Our results are dependent on the balance between avoiding Type I error and Type II errors. As for Type I errors, we chose a significance level of $\alpha=0.05$. A more restrictive significance level would have the consequence to increase Type II errors, i.e. to reduce the proportion of CODs for which a trend would be detected based on our method.

Moreover, our results should be seen as conditional on our use of ordinary least squares regression (OLS) models with polynomial terms. The OLS approach may not be appropriate for small counts. However, the approximation of a Poisson by a normal error distribution is generally assumed to be adequate if the mean number of observations is about five or more. For larger counts, OLS has the benefit that a variance can be estimated, rather than postulated.

In addition, an alternative to the classic polynomial regression approach would have been to use Generalized Additive Models (GAMs). These models have the advantage of being able to pick up trends that are not polynomial. In a sensitivity analysis, we applied GAMs with Gaussian process smoothing function to our data. We found that a long-term trend could be detected in 71.7 percent of the CODs, as compared to 64.0 percent in our original analysis. There were virtually no CODs for which a trend could be detected when using polynomial

For peer review only - http://bmjopen.bmj.com/site/about/guidelines.xhtml

models but not when using GAMs. This would imply that our results are approximately robust to the method used, although somewhat conservative.

Finally, including spatial correlation in our model may have altered the chance of detecting a significant trend for CODs with marked geographical patterns. We calculated Moran's I test for spatial correlation among countries regarding the proportion of CODs in each country with a detected long-term trend. The Moran's I test was found to be not statistically significant for all CODs collectively (p-value = 0.988). At the level of COD chapters, we found significant spatial correlation for the chapters C-D (p-value = 0.002), E (p-value = 0.025), and V-Y (p-value = 0.001), but not for other chapters.

We found that mortality from neoplasms was less likely to have a significant trend, for a given size of COD. This may relate to the fact that the neoplasm mortality levels tend to change gradually over time, without short-term trend changes **[6]**. Additionally, cancers are usually coded reliably and consistently over time **[39,40,41]**, so that coding artefacts can rarely induce artificial changes. Conversely, the dynamic nature of infectious diseases may be responsible for their higher likelihood to change over time, and to have significant trends even with relatively small numbers of deaths. Similarly, the chapter of signs-and-symptoms is sensitive to changes in the coding rules and practices, thus creating significant changes even with small number of deaths.

Our study showed that European countries did not vary substantially in the probability of detecting a significant long-term trend in CODs of the same size and type. This finding is surprising given the heterogeneity of the countries in terms of demographic characteristics, disease epidemiology, healthcare systems and coding practices. We found that differences between countries in the proportions of CODs with a significant trend (shown in Table 1) can be related to differences in COD size, which is strongly related to the differences in population size. Consequently, our analysis provides support for establishing one common

For peer review only - http://bmjopen.bmj.com/site/about/guidelines.xhtml

COD size threshold, applicable for all European countries and for use in international trend analyses.

Currently there is no gold standard for the selection of CODs to analyze for long-term trends. In this study we attempted to set such a standard, based on the criterion of the COD size, which is easy to measure for each single COD. We calculated thresholds with three common methods, which came close enough (e.g. in the range of 28 to 33 deaths) to support one general recommendation for practical use. Of course, different thresholds may be preferred, depending on the user's preference to avoid either false positives (by selecting a higher threshold) or false negatives (lower threshold).

In our data, the number of CODs that surpassed our recommended threshold of 30 annual deaths on average was around 500 for the biggest countries, 200-250 for the middle-sized countries and around 100 for the smaller European countries (results not shown). In total, 52 CODs had over 30 annual deaths on average in each country included in our analysis. This implies that at least 52 CODs could be included in the international comparison of long-term trends, but up to 100 if one is to accept a greater risk of false positives in smaller countries.

From the public health practitioner's perspective, the findings of our study can be used in order to set realistic expectations about the number of CODs that are likely to have a significant long-term trend in populations. We recommend a size criterion of 30 annual deaths to be considered when planning for national or international monitoring and comparisons of cause-specific mortality.

References

For peer review only - http://bmjopen.bmj.com/site/about/guidelines.xhtml

1. Mathers CD, Fat DM, Inoue M, Rao C, Lopez AD. Counting the dead and what they died from: an assessment of the global status of cause of death data. Bull World Health Organ. 2005;83(3):171-7.

2. Naghavi M, Abajobir AA, Abbafati C, Abbas KM, Abd-Allah F, Abera SF, et al. Global, regional, and national age-sex specific mortality for 264 causes of death, 1980-2016: a systematic analysis for the Global Burden of Disease Study 2016. Lancet. 2017;390(10100):1151-210.

3. World Health Organization. International statistical classification of diseases and related health problems. Tenth revision, vol. 2. Geneva, Switzerland: WHO; 1993.

4. Aregawi M, Malm KL, Wahjib M, Kofi O, Allotey N-K, Yaw PN, et al. Effect of anti-malarial interventions on trends of malaria cases, hospital admissions and deaths, 2005–2015 ,Ghana. Malar J. 2017;16(1):177.

5. Mnyani CN, Buchmann EJ, Chersich MF, Frank KA, McIntyre JA. Trends in maternal deaths in HIV-infected women, on a background of changing HIV management guidelines in South Africa: 1997 to 2015. J Int AIDS Soc. 2017;20(3): e25022.

6. Torre LA, Siegel RL, Ward EM, Jemal A. Global cancer incidence and mortality rates and trends-an update. Cancer Epidemiol Biomarkers Prev. 2016;25(1):16-27.

7. Armstrong GL, Conn, LA, Pinner RW. Trends in infectious disease mortality in the United States during the 20th century. JAMA. 1999;281(1):61-6.

8. Griffiths C, Brock A, Rooney C. The impact of introducing ICD-10 on trends in mortality from circulatory diseases in England and Wales. Health statistics quarterly / Office for National Statistics. 2004(22):14-20.

For peer review only

For peer review only - http://bmjopen.bmj.com/site/about/guidelines.xhtml

9. Choe YJ, Choe SA, Cho SI. Trends in infectious disease mortality, South Korea, 1983-2015. Emerg Infect Dis. 2018;24(2):320-7.

10. Van Dyke M, Greer S, Odom E, Schieb L, Vaughan A, Kramer M, et al. Heart disease death rates among blacks and whites Aged ≥35 Years - United States, 1968-2015. MMWR Surveill Summ. 2018;67(5):1-11.

11. Hertel-Fernandez AW, Giusti AE, Sotelo JM. The Chilean infant mortality decline: improvement for whom? Socioeconomic and geographic inequalities in infant mortality, 1990-2005. Bull World Health Organ. 2007;85(10):798-804.

12. Mesalles-Naranjo O, Grant I, Wyper GMA, Stockton D, Dobbie R, McFadden M, et al. Trends and inequalities in the burden of mortality in Scotland 2000-2015. PloS One. 2018;13(8):e0196906.

13. Medina-Gomez OS, Medina-Reyes IS. Mortality from type 2 diabetes and implementation of the PREVENIMSS program: a time series study in Mexico, 1998-2015. Cad Saude Publica. 2018;34(5):e00103117.

14. Allanson ER, Pattinson RC. Quality-of-care audits and perinatal mortality in South Africa. Bull World Health Organ. 2015;93(6):424-8.

15. Notrica DM, Sayrs LW, Krishna N. The effect of verified pediatric trauma centers, state laws, and crash characteristics on time trends in adolescent motor vehicle fatalities, 1999-2015. J Trauma Acute Care Surg. 2018;85(5):944-52.

16. WHO Mortality Database. Geneva: World Health Organization; 2017. Available from: http://www.who.int/healthinfo/mortality_data/en/ [accessed 5 October 2017].

For peer review only - http://bmjopen.bmj.com/site/about/guidelines.xhtml

17.Eurostat - European Union. Chapter 7: Demographic challenges-population projections. In: People in the EU: Who are we and how do we live? Eurostat Statistical Books. Luxemburg: Publications Office of the European Union; 2015. p. 159.

18. Mitratza M, Kunst AE, Kardaun JWPF. Detecting Mortality Trends in the Netherlands Across 625 Causes of Death. Int J Environ Res Public Health. 2019;16(21):4150. doi: 10.3390/ijerph16214150.

19.Shacham M, Brauner N. Minimizing the effects of collinearity in polynomial regression. Ind Eng Chem Res. 1997;36:4405-12.

20.Wong CS, Li WK. A note on the corrected Akaike information criterion for threshold autoregressive models. J Time Ser Anal. 1998;19(1):113-24.

21.Diez Roux AV. A glossary for multilevel analysis. J Epidemiol Community Health. 2002;56:588-94.

22.Metz CE. Basic principles of ROC analysis. Semin Nucl Med. 1978;8(4):283-98.

23.Greiner M, Pfeiffer D, Smith RD. Principles and practical application of the receiver-operating characteristic analysis for diagnostic tests. Prev Vet Med. 2000;45(1-2):23–41.

24.Zou KH, O'Malley AJ, Mauri L. Receiver-operating characteristic analysis for evaluating diagnostic tests and predictive models. Circulation. 2007;115(5):654–7.

25.Youden WJ. Index for rating diagnostic tests. Cancer. 1950;3(1):32–5.

26.Perkins NJ, Schisterman EF. The inconsistency of "optimal" cutpoints obtained using two criteria based on the receiver operating characteristic curve. Am J Epidemiol. 2006;163(7):670–5.

27.Fluss R, Faraggi D, Reiser B. Estimation of the Youden Index and its associated cutoff point. Biom J. 2005;47(4):458–72.

For peer review only

28. R Core Team (2018). R: A language and environment for statistical computing. R Foundation for Statistical Computing, Vienna, Austria: https://www.R-project.org.

29. Bradshaw D, Groenewald P, Bourne DE, Mahomed H, Nojilana B, Daniels J, et al. Making COD statistics useful for public health at local level in the city of Cape Town: utilidad para la salud publica a nivel local en Ciudad del Cabo. Bull World Health Organ. 2006;84(3):211-7.

30. Franca EB, Passos VMA, Malta DC, Duncan BB, Ribeiro ALP, Guimaraes MDC, et al. Cause-specific mortality for 249 causes in Brazil and states during 1990-2015: a systematic analysis for the global burden of disease study 2015. Popul Health Metr. 2017;15(1):39.

31. Ebmeier S, Thayabaran D, Braithwaite I, Benamara C, Weatherall M, Beasley R. Trends in international asthma mortality: analysis of data from the WHO Mortality Database from 46 countries (1993-2012). Lancet. 2017;390(10098):935-45.

32. Burkill S, Montgomery S, Hajiebrahimi M, Hillert J, Olsson T, Bahmanyar S. Mortality trends for multiple sclerosis patients in Sweden from 1968 to 2012. Neurology. 2017;89(6):555-62.

33. Reynales-Shigematsu LM, Guerrero-Lopez CM, Hernandez Avila M, Irving H, Jha P. Divergence and convergence in cause-specific premature adult mortality in Mexico and US Mexican Hispanics from 1995 to 2015: analyses of 4.9 million individual deaths. Int J Epidemiol. 2018;47(1):97-106.

34. Oza S, Lawn JE, Hogan DR, Mathers C, Cousens SN. Neonatal cause-of-death estimates for the early and late neonatal periods for 194 countries: 2000-2013. Bull World Health Organ. 2015;93(1):19-28.

35. Mack K, Clapperton A, Macpherson A, Sleet D, Newton D, Murdoch J, et al. Trends in the leading causes of injury mortality, Australia, Canada, and the United States, 2000-2014. Can J Public Health. 2017;108(2):e185-e91.

For peer review only - http://bmjopen.bmj.com/site/about/guidelines.xhtml

36. Padron-Monedero A, Damian J, Pilar Martin M, Fernandez-Cuenca R. Mortality trends for accidental falls in older people in Spain, 2000-2015. BMC Geriatr. 2017;17(1):276.

37. Ajdacic-Gross V, Weiss MG, Ring M, Hepp U, Bopp M, Gutzwiller F, et al. Methods of suicide: international suicide patterns derived from the WHO mortality database. Bull World Health Organ. 2008;86(9):726-32.

38. Commission Regulation (EU) No 328/2011 of 5 April 2011 implementing Regulation (EC) No 1338/2008 of the European Parliament and of the Council on Community statistics on public health and health and safety at work, as regards statistics on causes of death Text with EEA relevance. Official Journal of the European Union.

39. Harteloh P, de Bruin K, Kardaun J. The reliability of cause-of-death coding in The Netherlands. Eur J Epidemiol. 2010;25(8):531–8.

40. Lu TH, Lee MC, Chou MC. Accuracy of cause-of-death coding in Taiwan: types of miscoding and effects on mortality statistics. Int J Epidemiol. 2000;29(2):336–43.

41. Mieno MN, Tanaka N, Arai T, Kawahara T, Kuchiba A, Ishikawa S, et al. Accuracy of death certificates and assessment of factors for misclassification of underlying cause of death. J Epidemiol. 2016;26(4):191-8.

Footnotes

Contributors: AK, JK and MM designed the study. MM performed the calculations. MM, AK and JK analyzed and interpreted the results and formulated the conclusions, and made major contributions to the manuscript. All authors read and approved the final manuscript.

Funding: This research received no specific grant from any funding agency in the public, commercial or not-for-profit sectors.

Conflict of Interest: The authors declare that they have no conflict of interest.

For peer review only - http://bmjopen.bmj.com/site/about/guidelines.xhtml

Ethics approval: No ethical approval was required for this study, as no living subjects were involved and only aggregated administrative data were used.

Data sharing statement: All data used are publically available from the WHO Mortality Database (see https://www.who.int/healthinfo/mortality_data/en/).

Provenance and peer review: Not commissioned; externally peer reviewed.

Patient consent for publication: Not required.

Figure Captions

Fig1

Estimated probability for an underlying cause of death to have a significant long-term trend according to its size, by ICD-10 chapter

Footnote: *See Table 2 for the definition of the chapters.

Fig2

Estimated probability for a disease of the circulatory system to have a significant long-term mortality trend according to its size, by European country

Fig3

Sensitivity and specificity of the cause of death size for the detection of significant long-term time-trends, with thresholds for the optimal cause of death size for trend analysis

For peer review only

For peer review only - http://bmjopen.bmj.com/site/about/guidelines.xhtml

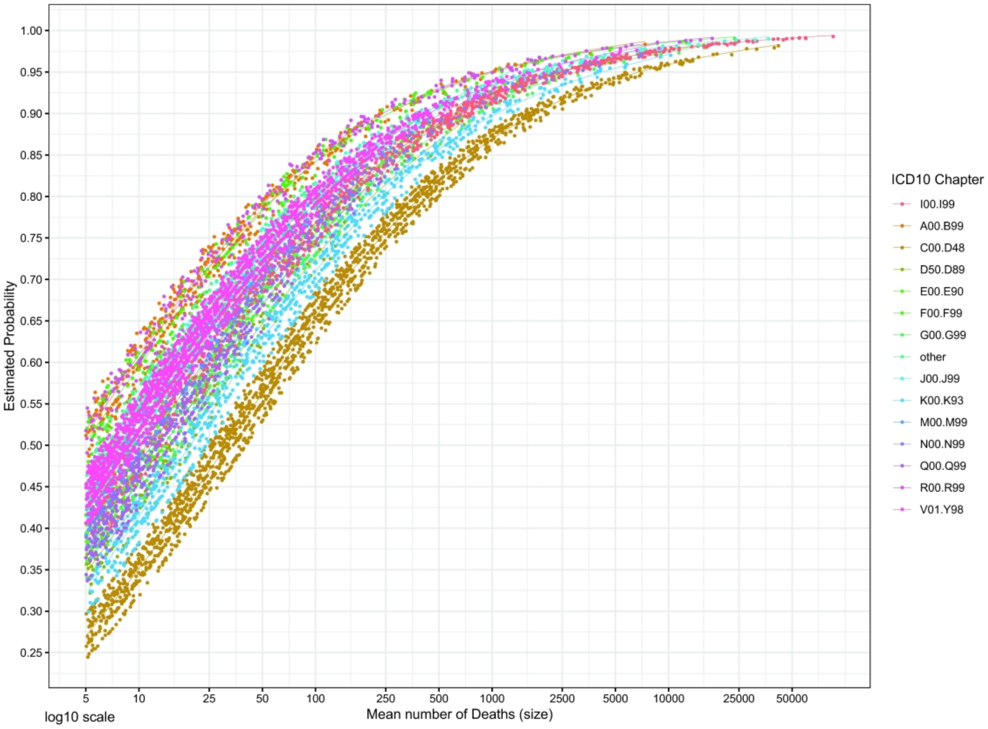

279x215mm (600 x 600 DPI)

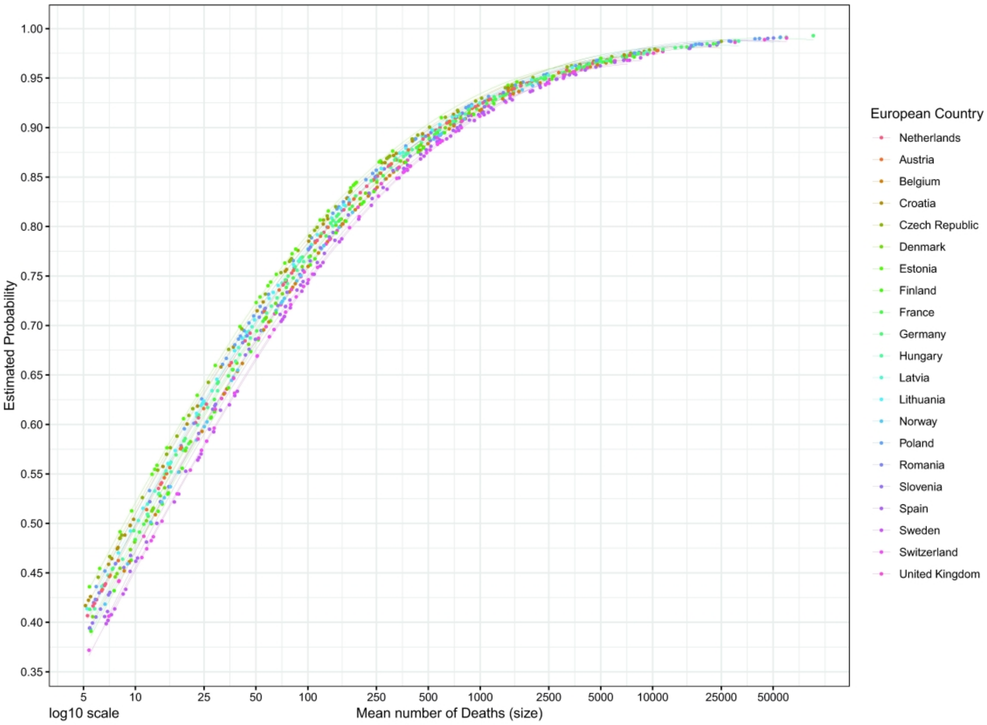

279x215mm (600 x 600 DPI)

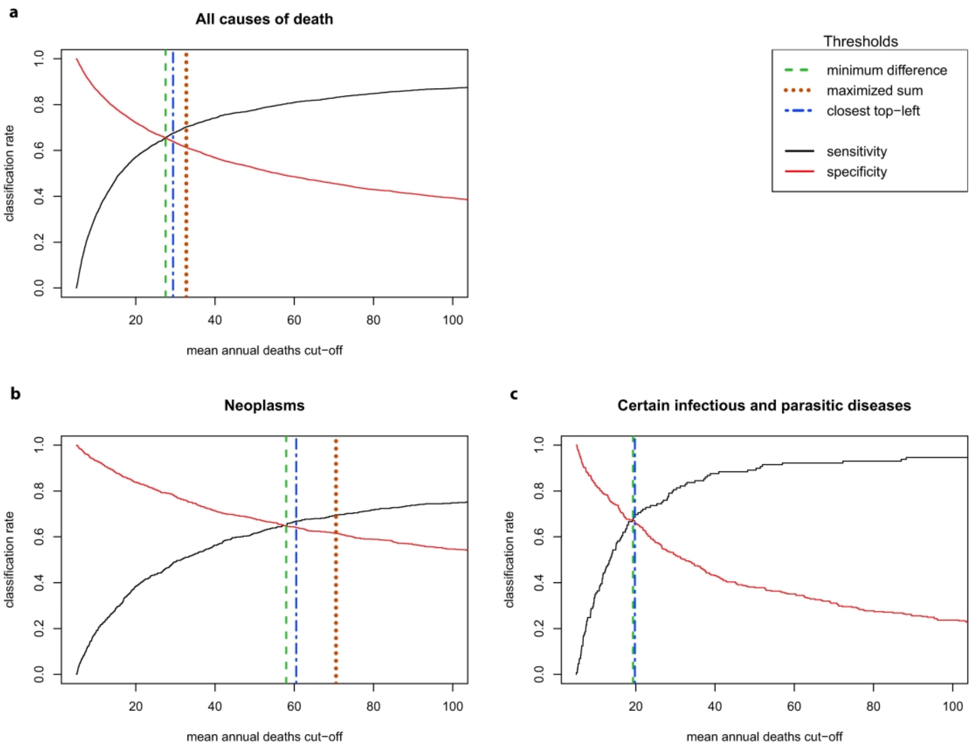

279x217mm (600 x 600 DPI)

ICD-10 code	ICD-10 code label	chapter	country	average age-standardized deaths	final best model polynomial degree	trend
A02	Other salmonella infections	A00.B99	Belgium	9.78	3	yes
A02	Other salmonella infections	A00.B99	Czech Republic	8.64	0	no
A02	Other salmonella infections	A00.B99	France	21.97	1	yes
A02	Other salmonella infections	A00.B99	Germany	54.17	1	yes
A02	Other salmonella infections	A00.B99	Hungary	6.18	0	no
A02	Other salmonella infections	A00.B99	Netherlands	7.44	1	yes
A02	Other salmonella infections	A00.B99	Poland	9.07	0	no
A02	Other salmonella infections	A00.B99	Spain	15.93	1	yes
A02	Other salmonella infections	A00.B99	United Kingdom	16.29	1	yes
A04	Other bacterial intestinal infections	A00.B99	Belgium	132.83	2	yes
A04	Other bacterial intestinal infections	A00.B99	Croatia	5.86	2	yes
A04	Other bacterial intestinal infections	A00.B99	Czech Republic	103.51	3	yes
A04	Other bacterial intestinal infections	A00.B99	Denmark	77.43	3	yes
A04	Other bacterial intestinal infections	A00.B99	Finland	16.69	0	no
A04	Other bacterial intestinal infections	A00.B99	France	290.13	3	yes
A04	Other bacterial intestinal infections	A00.B99	Germany	1221.13	3	yes
A04	Other bacterial intestinal infections	A00.B99	Hungary	108.85	2	yes
A04	Other bacterial intestinal infections	A00.B99	Netherlands	23.73	3	yes
A04	Other bacterial intestinal infections	A00.B99	Norway	47.71	2	yes
A04	Other bacterial intestinal infections	A00.B99	Poland	139.10	3	yes
A04	Other bacterial intestinal infections	A00.B99	Romania	26.54	3	yes
A04	Other bacterial intestinal infections	A00.B99	Spain	62.40	1	yes
A04	Other bacterial intestinal infections	A00.B99	Sweden	65.24	3	yes
A04	Other bacterial intestinal infections	A00.B99	Switzerland	23.09	1	yes
A04	Other bacterial intestinal infections	A00.B99	United Kingdom	1817.62	2	yes
A05	Other bacterial foodborne intoxications	A00.B99	France	11.53	1	yes
A05	Other bacterial foodborne intoxications	A00.B99	Germany	9.54	2	yes
A05	Other bacterial foodborne intoxications	A00.B99	Poland	5.75	0	no
A08	Viral and other specified intestinal infections	A00.B99	Belgium	16.42	0	no
A08	Viral and other specified intestinal infections	A00.B99	Denmark	17.33	2	yes
A08	Viral and other specified intestinal infections	A00.B99	Finland	18.39	2	yes
A08	Viral and other specified intestinal infections	A00.B99	France	33.75	1	yes
A08	Viral and other specified intestinal infections	A00.B99	Germany	171.25	2	yes
A08	Viral and other specified intestinal infections	A00.B99	Hungary	6.08	1	yes
A08	Viral and other specified intestinal infections	A00.B99	Netherlands	70.77	1	yes
A08	Viral and other specified intestinal infections	A00.B99	Norway	7.43	3	yes
A08	Viral and other specified intestinal infections	A00.B99	Poland	5.19	1	yes
A08	Viral and other specified intestinal infections	A00.B99	Spain	7.01	0	no
A08	Viral and other specified intestinal infections	A00.B99	Sweden	40.71	2	yes
A08	Viral and other specified intestinal infections	A00.B99	Switzerland	12.96	3	yes
A08	Viral and other specified intestinal infections	A00.B99	United Kingdom	76.31	3	yes
A09	Diarrhoea and gastroenteritis of presumed infectious origin	A00.B99	Austria	31.23	3	yes
A09	Diarrhoea and gastroenteritis of presumed infectious origin	A00.B99	Belgium	147.79	1	yes
A09	Diarrhoea and gastroenteritis of presumed infectious origin	A00.B99	Croatia	16.85	1	yes

For peer review only

A09	Diarrhoea and gastroenteritis of presumed infectious origin	A00.B99	Czech Republic	40.34	1	yes
A09	Diarrhoea and gastroenteritis of presumed infectious origin	A00.B99	Denmark	96.17	1	yes
A09	Diarrhoea and gastroenteritis of presumed infectious origin	A00.B99	Finland	27.69	1	yes
A09	Diarrhoea and gastroenteritis of presumed infectious origin	A00.B99	France	1031.42	3	yes
A09	Diarrhoea and gastroenteritis of presumed infectious origin	A00.B99	Germany	1349.34	1	yes
A09	Diarrhoea and gastroenteritis of presumed infectious origin	A00.B99	Hungary	62.70	3	yes
A09	Diarrhoea and gastroenteritis of presumed infectious origin	A00.B99	Netherlands	157.63	2	yes
A09	Diarrhoea and gastroenteritis of presumed infectious origin	A00.B99	Norway	121.17	2	yes
A09	Diarrhoea and gastroenteritis of presumed infectious origin	A00.B99	Poland	30.26	0	no
A09	Diarrhoea and gastroenteritis of presumed infectious origin	A00.B99	Romania	60.39	2	yes
A09	Diarrhoea and gastroenteritis of presumed infectious origin	A00.B99	Spain	538.56	1	yes
A09	Diarrhoea and gastroenteritis of presumed infectious origin	A00.B99	Sweden	96.00	3	yes
A09	Diarrhoea and gastroenteritis of presumed infectious origin	A00.B99	Switzerland	42.04	1	yes
A09	Diarrhoea and gastroenteritis of presumed infectious origin	A00.B99	United Kingdom	377.61	1	yes
A15	Respiratory tuberculosis, bacteriologically and histologically confirmed	A00.B99	Croatia	13.42	2	yes
A15	Respiratory tuberculosis, bacteriologically and histologically confirmed	A00.B99	Czech Republic	41.62	2	yes
A15	Respiratory tuberculosis, bacteriologically and histologically confirmed	A00.B99	Estonia	26.69	1	yes
A15	Respiratory tuberculosis, bacteriologically and histologically confirmed	A00.B99	Finland	20.85	2	yes
A15	Respiratory tuberculosis, bacteriologically and histologically confirmed	A00.B99	Germany	52.81	1	yes
A15	Respiratory tuberculosis, bacteriologically and histologically confirmed	A00.B99	Hungary	45.07	2	yes
A15	Respiratory tuberculosis, bacteriologically and histologically confirmed	A00.B99	Poland	345.67	0	no
A15	Respiratory tuberculosis, bacteriologically and histologically confirmed	A00.B99	Romania	1051.27	1	yes
A15	Respiratory tuberculosis, bacteriologically and histologically confirmed	A00.B99	Slovenia	9.30	0	no
A16	Respiratory tuberculosis, not confirmed bacteriologically or histologically	A00.B99	Austria	34.91	0	no
A16	Respiratory tuberculosis, not confirmed bacteriologically or histologically	A00.B99	Belgium	38.61	1	yes
A16	Respiratory tuberculosis, not confirmed bacteriologically or histologically	A00.B99	Croatia	101.52	1	yes
A16	Respiratory tuberculosis, not confirmed bacteriologically or histologically	A00.B99	Czech Republic	23.82	1	yes
A16	Respiratory tuberculosis, not confirmed bacteriologically or histologically	A00.B99	Denmark	20.54	0	no
A16	Respiratory tuberculosis, not confirmed bacteriologically or histologically	A00.B99	Estonia	23.02	2	yes
A16	Respiratory tuberculosis, not confirmed bacteriologically or histologically	A00.B99	France	357.23	3	yes

For peer review only

A16	Respiratory tuberculosis, not confirmed bacteriologically or histologically	A00.B99	Germany	229.49	2	yes
A16	Respiratory tuberculosis, not confirmed bacteriologically or histologically	A00.B99	Hungary	115.32	1	yes
A16	Respiratory tuberculosis, not confirmed bacteriologically or histologically	A00.B99	Latvia	52.58	2	yes
A16	Respiratory tuberculosis, not confirmed bacteriologically or histologically	A00.B99	Lithuania	155.24	1	yes
A16	Respiratory tuberculosis, not confirmed bacteriologically or histologically	A00.B99	Netherlands	24.40	1	yes
A16	Respiratory tuberculosis, not confirmed bacteriologically or histologically	A00.B99	Poland	514.49	2	yes
A16	Respiratory tuberculosis, not confirmed bacteriologically or histologically	A00.B99	Romania	679.40	2	yes
A16	Respiratory tuberculosis, not confirmed bacteriologically or histologically	A00.B99	Spain	247.81	2	yes
A16	Respiratory tuberculosis, not confirmed bacteriologically or histologically	A00.B99	Sweden	13.93	0	no
A16	Respiratory tuberculosis, not confirmed bacteriologically or histologically	A00.B99	Switzerland	10.23	3	yes
A16	Respiratory tuberculosis, not confirmed bacteriologically or histologically	A00.B99	United Kingdom	302.85	1	yes
A17	Tuberculosis of nervous system	A00.B99	France	14.06	1	yes
A17	Tuberculosis of nervous system	A00.B99	Germany	9.64	0	no
A17	Tuberculosis of nervous system	A00.B99	Poland	9.37	1	yes
A17	Tuberculosis of nervous system	A00.B99	Romania	17.09	1	yes
A17	Tuberculosis of nervous system	A00.B99	Spain	16.08	1	yes
A17	Tuberculosis of nervous system	A00.B99	United Kingdom	14.54	1	yes
A18	Tuberculosis of other organs	A00.B99	France	39.71	2	yes
A18	Tuberculosis of other organs	A00.B99	Germany	17.54	1	yes
A18	Tuberculosis of other organs	A00.B99	Poland	12.29	0	no
A18	Tuberculosis of other organs	A00.B99	Romania	14.98	1	yes
A18	Tuberculosis of other organs	A00.B99	Spain	30.10	1	yes
A18	Tuberculosis of other organs	A00.B99	United Kingdom	41.12	1	yes
A19	Miliary tuberculosis	A00.B99	Finland	7.44	3	yes
A19	Miliary tuberculosis	A00.B99	France	26.15	1	yes
A19	Miliary tuberculosis	A00.B99	Germany	21.82	1	yes
A19	Miliary tuberculosis	A00.B99	Hungary	8.07	1	yes
A19	Miliary tuberculosis	A00.B99	Latvia	5.32	0	no
A19	Miliary tuberculosis	A00.B99	Lithuania	74.55	3	yes
A19	Miliary tuberculosis	A00.B99	Poland	9.24	1	yes
A19	Miliary tuberculosis	A00.B99	Romania	13.24	0	no
A19	Miliary tuberculosis	A00.B99	Spain	30.55	1	yes
A19	Miliary tuberculosis	A00.B99	United Kingdom	36.87	3	yes
A26	Erysipeloid	A00.B99	Germany	5.18	2	yes
A27	Leptospirosis	A00.B99	Romania	8.07	1	yes
A31	Infection due to other mycobacteria	A00.B99	Finland	7.60	0	no
A31	Infection due to other mycobacteria	A00.B99	France	19.16	0	no
A31	Infection due to other mycobacteria	A00.B99	Germany	8.02	1	yes
A31	Infection due to other mycobacteria	A00.B99	Poland	5.68	0	no
A31	Infection due to other mycobacteria	A00.B99	Sweden	6.62	1	yes
A31	Infection due to other mycobacteria	A00.B99	United Kingdom	41.26	3	yes
A32	Listeriosis	A00.B99	France	21.74	0	no
A32	Listeriosis	A00.B99	Germany	16.87	3	yes

For peer review only

A32	Listeriosis	A00.B99	Spain	18.11	0	no
A32	Listeriosis	A00.B99	United Kingdom	16.21	0	no
A35	Other tetanus	A00.B99	France	6.02	1	yes
A35	Other tetanus	A00.B99	Poland	8.94	1	yes
A35	Other tetanus	A00.B99	Romania	5.33	1	yes
A37	Whooping cough	A00.B99	France	5.01	0	no
A39	Meningococcal infection	A00.B99	Austria	6.07	0	no
A39	Meningococcal infection	A00.B99	Belgium	13.33	1	yes
A39	Meningococcal infection	A00.B99	Czech Republic	6.68	1	yes
A39	Meningococcal infection	A00.B99	Denmark	5.44	1	yes
A39	Meningococcal infection	A00.B99	France	30.02	1	yes
A39	Meningococcal infection	A00.B99	Germany	50.01	3	yes
A39	Meningococcal infection	A00.B99	Hungary	7.47	0	no
A39	Meningococcal infection	A00.B99	Lithuania	7.37	0	no
A39	Meningococcal infection	A00.B99	Netherlands	12.96	3	yes
A39	Meningococcal infection	A00.B99	Poland	15.10	0	no
A39	Meningococcal infection	A00.B99	Romania	16.17	2	yes
A39	Meningococcal infection	A00.B99	Spain	42.73	1	yes
A39	Meningococcal infection	A00.B99	Switzerland	6.75	2	yes
A39	Meningococcal infection	A00.B99	United Kingdom	82.51	3	yes
A40	Streptococcal septicaemia	A00.B99	Austria	6.16	0	no
A40	Streptococcal septicaemia	A00.B99	Belgium	34.18	2	yes
A40	Streptococcal septicaemia	A00.B99	Czech Republic	10.74	1	yes
A40	Streptococcal septicaemia	A00.B99	Denmark	23.52	2	yes
A40	Streptococcal septicaemia	A00.B99	Finland	28.34	0	no
A40	Streptococcal septicaemia	A00.B99	France	121.57	0	no
A40	Streptococcal septicaemia	A00.B99	Germany	152.87	2	yes
A40	Streptococcal septicaemia	A00.B99	Lithuania	6.30	1	yes
A40	Streptococcal septicaemia	A00.B99	Netherlands	56.51	1	yes
A40	Streptococcal septicaemia	A00.B99	Norway	26.55	1	yes
A40	Streptococcal septicaemia	A00.B99	Poland	22.38	3	yes
A40	Streptococcal septicaemia	A00.B99	Spain	32.47	0	no
A40	Streptococcal septicaemia	A00.B99	Sweden	45.71	1	yes
A40	Streptococcal septicaemia	A00.B99	Switzerland	22.52	0	no
A40	Streptococcal septicaemia	A00.B99	United Kingdom	118.54	3	yes
A41	Other septicaemia	A00.B99	Austria	87.99	2	yes
A41	Other septicaemia	A00.B99	Belgium	1466.24	3	yes
A41	Other septicaemia	A00.B99	Croatia	232.07	2	yes
A41	Other septicaemia	A00.B99	Czech Republic	759.13	1	yes
A41	Other septicaemia	A00.B99	Denmark	402.79	0	no
A41	Other septicaemia	A00.B99	Estonia	13.02	2	yes
A41	Other septicaemia	A00.B99	Finland	129.82	0	no
A41	Other septicaemia	A00.B99	France	4658.94	0	no
A41	Other septicaemia	A00.B99	Germany	7361.90	1	yes
A41	Other septicaemia	A00.B99	Hungary	87.44	1	yes
A41	Other septicaemia	A00.B99	Latvia	29.51	0	no
A41	Other septicaemia	A00.B99	Lithuania	181.40	2	yes
A41	Other septicaemia	A00.B99	Netherlands	1360.05	3	yes
A41	Other septicaemia	A00.B99	Norway	363.57	3	yes
A41	Other septicaemia	A00.B99	Poland	1236.95	3	yes

For peer review only

A41	Other septicaemia	A00.B99	Romania	625.64	2	yes
A41	Other septicaemia	A00.B99	Slovenia	86.99	0	no
A41	Other septicaemia	A00.B99	Spain	3600.30	3	yes
A41	Other septicaemia	A00.B99	Sweden	705.38	1	yes
A41	Other septicaemia	A00.B99	Switzerland	192.17	0	no
A41	Other septicaemia	A00.B99	United Kingdom	2963.53	3	yes
A43	Nocardiosis	A00.B99	France	5.41	0	no
A43	Nocardiosis	A00.B99	Spain	5.24	0	no
A46	Erysipelas	A00.B99	Austria	15.53	2	yes
A46	Erysipelas	A00.B99	Belgium	34.20	1	yes
A46	Erysipelas	A00.B99	Czech Republic	15.71	2	yes
A46	Erysipelas	A00.B99	Denmark	36.34	1	yes
A46	Erysipelas	A00.B99	Estonia	6.24	0	no
A46	Erysipelas	A00.B99	Finland	27.89	0	no
A46	Erysipelas	A00.B99	France	209.88	1	yes
A46	Erysipelas	A00.B99	Germany	236.24	1	yes
A46	Erysipelas	A00.B99	Hungary	26.58	1	yes
A46	Erysipelas	A00.B99	Latvia	15.88	1	yes
A46	Erysipelas	A00.B99	Lithuania	9.46	0	no
A46	Erysipelas	A00.B99	Netherlands	121.16	1	yes
A46	Erysipelas	A00.B99	Norway	26.92	0	no
A46	Erysipelas	A00.B99	Poland	17.45	0	no
A46	Erysipelas	A00.B99	Sweden	46.96	2	yes
A46	Erysipelas	A00.B99	Switzerland	21.43	1	yes
A48	Other bacterial diseases, not elsewhere classified	A00.B99	Belgium	25.29	0	no
A48	Other bacterial diseases, not elsewhere classified	A00.B99	Czech Republic	10.61	2	yes
A48	Other bacterial diseases, not elsewhere classified	A00.B99	Denmark	13.04	1	yes
A48	Other bacterial diseases, not elsewhere classified	A00.B99	France	125.58	2	yes
A48	Other bacterial diseases, not elsewhere classified	A00.B99	Germany	58.22	1	yes
A48	Other bacterial diseases, not elsewhere classified	A00.B99	Hungary	8.70	1	yes
A48	Other bacterial diseases, not elsewhere classified	A00.B99	Netherlands	34.83	1	yes
A48	Other bacterial diseases, not elsewhere classified	A00.B99	Poland	26.50	2	yes
A48	Other bacterial diseases, not elsewhere classified	A00.B99	Romania	31.21	0	no
A48	Other bacterial diseases, not elsewhere classified	A00.B99	Spain	42.04	1	yes
A48	Other bacterial diseases, not elsewhere classified	A00.B99	Sweden	7.39	0	no
A48	Other bacterial diseases, not elsewhere classified	A00.B99	Switzerland	12.08	1	yes
A48	Other bacterial diseases, not elsewhere classified	A00.B99	United Kingdom	36.30	2	yes
A49	Bacterial infection of unspecified site	A00.B99	Belgium	52.93	3	yes
A49	Bacterial infection of unspecified site	A00.B99	Czech Republic	24.85	1	yes
A49	Bacterial infection of unspecified site	A00.B99	Denmark	62.50	2	yes
A49	Bacterial infection of unspecified site	A00.B99	Finland	13.65	0	no
A49	Bacterial infection of unspecified site	A00.B99	France	138.52	1	yes
A49	Bacterial infection of unspecified site	A00.B99	Germany	166.82	3	yes
A49	Bacterial infection of unspecified site	A00.B99	Netherlands	19.82	2	yes
A49	Bacterial infection of unspecified site	A00.B99	Norway	15.89	1	yes
A49	Bacterial infection of unspecified site	A00.B99	Poland	22.69	1	yes
A49	Bacterial infection of unspecified site	A00.B99	Romania	22.55	1	yes
A49	Bacterial infection of unspecified site	A00.B99	Spain	116.89	3	yes
A49	Bacterial infection of unspecified site	A00.B99	Sweden	45.38	2	yes
A49	Bacterial infection of unspecified site	A00.B99	Switzerland	7.34	2	yes

For peer review only

A49	Bacterial infection of unspecified site	A00.B99	United Kingdom	198.85	2	yes
A52	Late syphilis	A00.B99	Germany	6.36	2	yes
A52	Late syphilis	A00.B99	Spain	7.60	0	no
A69	Other spirochaetal infections	A00.B99	Germany	9.70	0	no
A81	Slow virus infections of central nervous system	A00.B99	Austria	23.65	0	no
A81	Slow virus infections of central nervous system	A00.B99	Belgium	19.31	0	no
A81	Slow virus infections of central nervous system	A00.B99	Denmark	8.60	0	no
A81	Slow virus infections of central nervous system	A00.B99	Finland	12.41	0	no
A81	Slow virus infections of central nervous system	A00.B99	France	154.72	0	no
A81	Slow virus infections of central nervous system	A00.B99	Germany	160.70	1	yes
A81	Slow virus infections of central nervous system	A00.B99	Hungary	17.77	0	no
A81	Slow virus infections of central nervous system	A00.B99	Netherlands	31.41	1	yes
A81	Slow virus infections of central nervous system	A00.B99	Norway	8.55	0	no
A81	Slow virus infections of central nervous system	A00.B99	Poland	19.75	2	yes
A81	Slow virus infections of central nervous system	A00.B99	Romania	6.70	1	yes
A81	Slow virus infections of central nervous system	A00.B99	Spain	88.26	0	no
A81	Slow virus infections of central nervous system	A00.B99	Sweden	19.47	0	no
A81	Slow virus infections of central nervous system	A00.B99	Switzerland	20.16	1	yes
A81	Slow virus infections of central nervous system	A00.B99	United Kingdom	123.97	1	yes
A86	Unspecified viral encephalitis	A00.B99	France	15.02	0	no
A86	Unspecified viral encephalitis	A00.B99	Germany	12.12	0	no
A86	Unspecified viral encephalitis	A00.B99	Poland	11.05	1	yes
A86	Unspecified viral encephalitis	A00.B99	Romania	12.95	0	no
A86	Unspecified viral encephalitis	A00.B99	Spain	28.04	0	no
A86	Unspecified viral encephalitis	A00.B99	United Kingdom	42.92	1	yes
A87	Viral meningitis	A00.B99	France	10.96	1	yes
A87	Viral meningitis	A00.B99	Romania	6.33	0	no
A87	Viral meningitis	A00.B99	Spain	9.28	1	yes
B00	Herpesviral [herpes simplex] infections	A00.B99	Austria	5.57	1	yes
B00	Herpesviral [herpes simplex] infections	A00.B99	Belgium	14.94	0	no
B00	Herpesviral [herpes simplex] infections	A00.B99	France	50.36	0	no
B00	Herpesviral [herpes simplex] infections	A00.B99	Germany	45.32	1	yes
B00	Herpesviral [herpes simplex] infections	A00.B99	Netherlands	15.59	3	yes
B00	Herpesviral [herpes simplex] infections	A00.B99	Spain	37.37	2	yes
B00	Herpesviral [herpes simplex] infections	A00.B99	Sweden	6.65	0	no
B00	Herpesviral [herpes simplex] infections	A00.B99	Switzerland	5.24	0	no
B00	Herpesviral [herpes simplex] infections	A00.B99	United Kingdom	32.88	0	no
B01	Varicella [chickenpox]	A00.B99	France	11.50	0	no
B01	Varicella [chickenpox]	A00.B99	Germany	6.71	0	no
B01	Varicella [chickenpox]	A00.B99	Spain	7.71	1	yes
B01	Varicella [chickenpox]	A00.B99	United Kingdom	21.80	3	yes
B02	Zoster [herpes zoster]	A00.B99	Belgium	13.86	0	no
B02	Zoster [herpes zoster]	A00.B99	Denmark	5.27	1	yes
B02	Zoster [herpes zoster]	A00.B99	France	71.15	1	yes
B02	Zoster [herpes zoster]	A00.B99	Germany	75.74	1	yes
B02	Zoster [herpes zoster]	A00.B99	Netherlands	28.33	0	no
B02	Zoster [herpes zoster]	A00.B99	Norway	9.75	2	yes
B02	Zoster [herpes zoster]	A00.B99	Spain	21.28	0	no
B02	Zoster [herpes zoster]	A00.B99	Sweden	12.61	0	no
B02	Zoster [herpes zoster]	A00.B99	Switzerland	9.43	0	no

For peer review only

B02	Zoster [herpes zoster]	A00.B99	United Kingdom	69.17	3	yes
B15	Acute hepatitis A	A00.B99	Germany	8.72	3	yes
B16	Acute hepatitis B	A00.B99	Belgium	6.18	2	yes
B16	Acute hepatitis B	A00.B99	France	13.26	1	yes
B16	Acute hepatitis B	A00.B99	Germany	69.13	2	yes
B16	Acute hepatitis B	A00.B99	Poland	25.77	3	yes
B16	Acute hepatitis B	A00.B99	Romania	11.03	3	yes
B16	Acute hepatitis B	A00.B99	Spain	38.79	0	no
B16	Acute hepatitis B	A00.B99	United Kingdom	37.60	1	yes
B17	Other acute viral hepatitis	A00.B99	Belgium	22.54	3	yes
B17	Other acute viral hepatitis	A00.B99	Czech Republic	17.33	0	no
B17	Other acute viral hepatitis	A00.B99	France	70.84	1	yes
B17	Other acute viral hepatitis	A00.B99	Germany	72.19	2	yes
B17	Other acute viral hepatitis	A00.B99	Netherlands	6.25	2	yes
B17	Other acute viral hepatitis	A00.B99	Poland	9.47	0	no
B17	Other acute viral hepatitis	A00.B99	Romania	7.36	1	yes
B17	Other acute viral hepatitis	A00.B99	Spain	139.58	3	yes
B17	Other acute viral hepatitis	A00.B99	United Kingdom	80.13	1	yes
B18	Chronic viral hepatitis	A00.B99	Austria	277.69	2	yes
B18	Chronic viral hepatitis	A00.B99	Belgium	89.56	2	yes
B18	Chronic viral hepatitis	A00.B99	Croatia	36.46	3	yes
B18	Chronic viral hepatitis	A00.B99	Czech Republic	23.05	1	yes
B18	Chronic viral hepatitis	A00.B99	Denmark	19.70	0	no
B18	Chronic viral hepatitis	A00.B99	Estonia	5.65	1	yes
B18	Chronic viral hepatitis	A00.B99	Finland	8.66	0	no
B18	Chronic viral hepatitis	A00.B99	France	702.43	1	yes
B18	Chronic viral hepatitis	A00.B99	Germany	876.32	1	yes
B18	Chronic viral hepatitis	A00.B99	Latvia	26.31	1	yes
B18	Chronic viral hepatitis	A00.B99	Lithuania	17.12	2	yes
B18	Chronic viral hepatitis	A00.B99	Netherlands	48.28	1	yes
B18	Chronic viral hepatitis	A00.B99	Norway	9.94	0	no
B18	Chronic viral hepatitis	A00.B99	Poland	254.57	1	yes
B18	Chronic viral hepatitis	A00.B99	Romania	34.80	3	yes
B18	Chronic viral hepatitis	A00.B99	Slovenia	6.70	0	no
B18	Chronic viral hepatitis	A00.B99	Spain	860.57	1	yes
B18	Chronic viral hepatitis	A00.B99	Sweden	57.65	1	yes
B18	Chronic viral hepatitis	A00.B99	Switzerland	14.33	1	yes
B18	Chronic viral hepatitis	A00.B99	United Kingdom	140.70	1	yes
B19	Unspecified viral hepatitis	A00.B99	France	19.38	1	yes
B19	Unspecified viral hepatitis	A00.B99	Germany	10.24	1	yes
B19	Unspecified viral hepatitis	A00.B99	Poland	8.67	1	yes
B19	Unspecified viral hepatitis	A00.B99	Spain	19.80	1	yes
B19	Unspecified viral hepatitis	A00.B99	United Kingdom	5.84	1	yes
B20	Human immunodeficiency virus [HIV] disease resulting in infectious and parasitic diseases	A00.B99	Belgium	20.40	1	yes
B20	Human immunodeficiency virus [HIV] disease resulting in infectious and parasitic diseases	A00.B99	Denmark	11.14	0	no
B20	Human immunodeficiency virus [HIV] disease resulting in infectious and parasitic diseases	A00.B99	Estonia	15.62	2	yes
B20	Human immunodeficiency virus [HIV] disease resulting in infectious and parasitic diseases	A00.B99	France	279.73	3	yes

For peer review only

B20	Human immunodeficiency virus [HIV] disease resulting in infectious and parasitic diseases	A00.B99	Germany	93.60	2	yes
B20	Human immunodeficiency virus [HIV] disease resulting in infectious and parasitic diseases	A00.B99	Latvia	31.93	2	yes
B20	Human immunodeficiency virus [HIV] disease resulting in infectious and parasitic diseases	A00.B99	Lithuania	7.86	1	yes
B20	Human immunodeficiency virus [HIV] disease resulting in infectious and parasitic diseases	A00.B99	Netherlands	27.18	3	yes
B20	Human immunodeficiency virus [HIV] disease resulting in infectious and parasitic diseases	A00.B99	Norway	7.12	0	no
B20	Human immunodeficiency virus [HIV] disease resulting in infectious and parasitic diseases	A00.B99	Poland	57.00	0	no
B20	Human immunodeficiency virus [HIV] disease resulting in infectious and parasitic diseases	A00.B99	Romania	77.78	2	yes
B20	Human immunodeficiency virus [HIV] disease resulting in infectious and parasitic diseases	A00.B99	Spain	530.89	2	yes
B20	Human immunodeficiency virus [HIV] disease resulting in infectious and parasitic diseases	A00.B99	Sweden	8.82	1	yes
B20	Human immunodeficiency virus [HIV] disease resulting in infectious and parasitic diseases	A00.B99	Switzerland	20.84	2	yes
B20	Human immunodeficiency virus [HIV] disease resulting in infectious and parasitic diseases	A00.B99	United Kingdom	131.09	2	yes
B21	Human immunodeficiency virus [HIV] disease resulting in malignant neoplasms	A00.B99	Belgium	7.34	0	no
B21	Human immunodeficiency virus [HIV] disease resulting in malignant neoplasms	A00.B99	France	92.20	1	yes
B21	Human immunodeficiency virus [HIV] disease resulting in malignant neoplasms	A00.B99	Germany	52.68	1	yes
B21	Human immunodeficiency virus [HIV] disease resulting in malignant neoplasms	A00.B99	Netherlands	13.24	1	yes
B21	Human immunodeficiency virus [HIV] disease resulting in malignant neoplasms	A00.B99	Poland	11.82	1	yes
B21	Human immunodeficiency virus [HIV] disease resulting in malignant neoplasms	A00.B99	Romania	6.51	2	yes
B21	Human immunodeficiency virus [HIV] disease resulting in malignant neoplasms	A00.B99	Spain	118.70	1	yes
B21	Human immunodeficiency virus [HIV] disease resulting in malignant neoplasms	A00.B99	Switzerland	8.88	2	yes
B21	Human immunodeficiency virus [HIV] disease resulting in malignant neoplasms	A00.B99	United Kingdom	29.58	1	yes
B22	Human immunodeficiency virus [HIV] disease resulting in other specified diseases	A00.B99	Belgium	6.69	0	no
B22	Human immunodeficiency virus [HIV] disease resulting in other specified diseases	A00.B99	Estonia	9.72	1	yes
B22	Human immunodeficiency virus [HIV] disease resulting in other specified diseases	A00.B99	France	145.50	1	yes
B22	Human immunodeficiency virus [HIV] disease resulting in other specified diseases	A00.B99	Germany	29.52	2	yes
B22	Human immunodeficiency virus [HIV] disease resulting in other specified diseases	A00.B99	Netherlands	6.48	2	yes
B22	Human immunodeficiency virus [HIV] disease resulting in other specified diseases	A00.B99	Poland	25.47	3	yes
B22	Human immunodeficiency virus [HIV] disease resulting in other specified diseases	A00.B99	Romania	24.72	3	yes
B22	Human immunodeficiency virus [HIV] disease resulting in other specified diseases	A00.B99	Spain	112.55	1	yes
B22	Human immunodeficiency virus [HIV] disease resulting in other specified diseases	A00.B99	Switzerland	21.14	1	yes
B22	Human immunodeficiency virus [HIV] disease resulting in other specified diseases	A00.B99	United Kingdom	24.31	3	yes

For peer review only

B23	Human immunodeficiency virus [HIV] disease resulting in other conditions	A00.B99	Belgium	6.08	0	no
B23	Human immunodeficiency virus [HIV] disease resulting in other conditions	A00.B99	France	142.60	3	yes
B23	Human immunodeficiency virus [HIV] disease resulting in other conditions	A00.B99	Germany	37.80	3	yes
B23	Human immunodeficiency virus [HIV] disease resulting in other conditions	A00.B99	Poland	15.95	0	no
B23	Human immunodeficiency virus [HIV] disease resulting in other conditions	A00.B99	Romania	5.74	2	yes
B23	Human immunodeficiency virus [HIV] disease resulting in other conditions	A00.B99	Spain	113.78	2	yes
B23	Human immunodeficiency virus [HIV] disease resulting in other conditions	A00.B99	United Kingdom	27.82	2	yes
B24	Unspecified human immunodeficiency virus [HIV] disease	A00.B99	Austria	32.58	1	yes
B24	Unspecified human immunodeficiency virus [HIV] disease	A00.B99	Belgium	11.76	0	no
B24	Unspecified human immunodeficiency virus [HIV] disease	A00.B99	Denmark	9.26	0	no
B24	Unspecified human immunodeficiency virus [HIV] disease	A00.B99	France	63.94	2	yes
B24	Unspecified human immunodeficiency virus [HIV] disease	A00.B99	Germany	219.85	2	yes
B24	Unspecified human immunodeficiency virus [HIV] disease	A00.B99	Netherlands	13.61	1	yes
B24	Unspecified human immunodeficiency virus [HIV] disease	A00.B99	Romania	71.17	3	yes
B24	Unspecified human immunodeficiency virus [HIV] disease	A00.B99	Spain	258.75	3	yes
B24	Unspecified human immunodeficiency virus [HIV] disease	A00.B99	Switzerland	13.21	0	no
B24	Unspecified human immunodeficiency virus [HIV] disease	A00.B99	United Kingdom	17.46	3	yes
B25	Cytomegaloviral disease	A00.B99	France	15.61	3	yes
B25	Cytomegaloviral disease	A00.B99	Germany	11.67	0	no
B25	Cytomegaloviral disease	A00.B99	Spain	7.81	0	no
B25	Cytomegaloviral disease	A00.B99	United Kingdom	17.72	0	no
B33	Other viral diseases, not elsewhere classified	A00.B99	United Kingdom	10.26	0	no
B34	Viral infection of unspecified site	A00.B99	Belgium	24.82	1	yes
B34	Viral infection of unspecified site	A00.B99	Denmark	8.54	0	no
B34	Viral infection of unspecified site	A00.B99	France	63.32	1	yes
B34	Viral infection of unspecified site	A00.B99	Germany	151.72	1	yes
B34	Viral infection of unspecified site	A00.B99	Hungary	21.79	1	yes
B34	Viral infection of unspecified site	A00.B99	Latvia	14.16	0	no
B34	Viral infection of unspecified site	A00.B99	Netherlands	89.62	3	yes
B34	Viral infection of unspecified site	A00.B99	Norway	5.59	1	yes
B34	Viral infection of unspecified site	A00.B99	Spain	13.75	1	yes
B34	Viral infection of unspecified site	A00.B99	Sweden	34.72	1	yes
B34	Viral infection of unspecified site	A00.B99	Switzerland	11.42	1	yes
B34	Viral infection of unspecified site	A00.B99	United Kingdom	38.05	0	no
B37	Candidiasis	A00.B99	Belgium	35.82	1	yes
B37	Candidiasis	A00.B99	Denmark	5.29	1	yes
B37	Candidiasis	A00.B99	France	95.35	3	yes
B37	Candidiasis	A00.B99	Germany	95.35	2	yes
B37	Candidiasis	A00.B99	Netherlands	11.49	0	no

For peer review only

B37	Candidiasis	A00.B99	Poland	5.68	0	no
B37	Candidiasis	A00.B99	Spain	26.54	0	no
B37	Candidiasis	A00.B99	Sweden	8.58	1	yes
B37	Candidiasis	A00.B99	Switzerland	5.20	2	yes
B37	Candidiasis	A00.B99	United Kingdom	29.19	0	no
B44	Aspergillosis	A00.B99	Belgium	40.93	0	no
B44	Aspergillosis	A00.B99	France	67.09	3	yes
B44	Aspergillosis	A00.B99	Germany	51.42	0	no
B44	Aspergillosis	A00.B99	Netherlands	18.15	0	no
B44	Aspergillosis	A00.B99	Poland	13.47	0	no
B44	Aspergillosis	A00.B99	Spain	48.22	0	no
B44	Aspergillosis	A00.B99	United Kingdom	68.90	1	yes
B49	Unspecified mycosis	A00.B99	Belgium	6.29	3	yes
B49	Unspecified mycosis	A00.B99	France	14.11	0	no
B49	Unspecified mycosis	A00.B99	Germany	10.88	0	no
B49	Unspecified mycosis	A00.B99	United Kingdom	16.74	0	no
B50	Plasmodium falciparum malaria	A00.B99	France	12.59	1	yes
B54	Unspecified malaria	A00.B99	France	5.15	1	yes
B55	Leishmaniasis	A00.B99	Spain	6.24	2	yes
B59	Pneumocystosis	A00.B99	Belgium	6.91	1	yes
B59	Pneumocystosis	A00.B99	France	33.29	3	yes
B59	Pneumocystosis	A00.B99	Germany	16.70	1	yes
B59	Pneumocystosis	A00.B99	Netherlands	5.67	1	yes
B59	Pneumocystosis	A00.B99	Spain	5.43	0	no
B59	Pneumocystosis	A00.B99	United Kingdom	37.53	0	no
B67	Echinococcosis	A00.B99	Germany	6.30	0	no
B67	Echinococcosis	A00.B99	Romania	16.87	1	yes
B67	Echinococcosis	A00.B99	Spain	19.74	1	yes
B90	Sequelae of tuberculosis	A00.B99	Belgium	29.48	1	yes
B90	Sequelae of tuberculosis	A00.B99	Finland	38.58	1	yes
B90	Sequelae of tuberculosis	A00.B99	France	382.27	1	yes
B90	Sequelae of tuberculosis	A00.B99	Germany	84.94	1	yes
B90	Sequelae of tuberculosis	A00.B99	Hungary	57.02	2	yes
B90	Sequelae of tuberculosis	A00.B99	Latvia	14.96	0	no
B90	Sequelae of tuberculosis	A00.B99	Netherlands	38.94	1	yes
B90	Sequelae of tuberculosis	A00.B99	Norway	28.05	1	yes
B90	Sequelae of tuberculosis	A00.B99	Poland	32.50	3	yes
B90	Sequelae of tuberculosis	A00.B99	Spain	103.14	1	yes
B90	Sequelae of tuberculosis	A00.B99	Sweden	37.15	1	yes
B90	Sequelae of tuberculosis	A00.B99	Switzerland	11.52	1	yes
B90	Sequelae of tuberculosis	A00.B99	United Kingdom	66.69	2	yes
B91	Sequelae of poliomyelitis	A00.B99	Austria	11.54	0	no
B91	Sequelae of poliomyelitis	A00.B99	Belgium	7.38	1	yes
B91	Sequelae of poliomyelitis	A00.B99	Finland	7.76	0	no
B91	Sequelae of poliomyelitis	A00.B99	France	51.98	0	no
B91	Sequelae of poliomyelitis	A00.B99	Germany	23.72	2	yes
B91	Sequelae of poliomyelitis	A00.B99	Hungary	5.84	1	yes
B91	Sequelae of poliomyelitis	A00.B99	Netherlands	6.80	0	no
B91	Sequelae of poliomyelitis	A00.B99	Norway	15.36	0	no
B91	Sequelae of poliomyelitis	A00.B99	Spain	10.77	0	no

For peer review only

B91	Sequelae of poliomyelitis	A00.B99	Sweden	28.64	1	yes
B91	Sequelae of poliomyelitis	A00.B99	Switzerland	10.82	0	no
B91	Sequelae of poliomyelitis	A00.B99	United Kingdom	37.17	0	no
B94	Sequelae of other and unspecified infectious and parasitic diseases	A00.B99	Finland	6.64	0	no
B94	Sequelae of other and unspecified infectious and parasitic diseases	A00.B99	France	37.63	2	yes
B94	Sequelae of other and unspecified infectious and parasitic diseases	A00.B99	Germany	137.31	2	yes
B94	Sequelae of other and unspecified infectious and parasitic diseases	A00.B99	Poland	11.66	0	no
B94	Sequelae of other and unspecified infectious and parasitic diseases	A00.B99	Sweden	6.05	2	yes
B94	Sequelae of other and unspecified infectious and parasitic diseases	A00.B99	Switzerland	72.26	0	no
B94	Sequelae of other and unspecified infectious and parasitic diseases	A00.B99	United Kingdom	10.02	3	yes
B99	Other and unspecified infectious diseases	A00.B99	Austria	31.26	3	yes
B99	Other and unspecified infectious diseases	A00.B99	Belgium	349.03	1	yes
B99	Other and unspecified infectious diseases	A00.B99	Denmark	60.41	3	yes
B99	Other and unspecified infectious diseases	A00.B99	France	1540.91	2	yes
B99	Other and unspecified infectious diseases	A00.B99	Germany	1104.45	1	yes
B99	Other and unspecified infectious diseases	A00.B99	Netherlands	452.61	3	yes
B99	Other and unspecified infectious diseases	A00.B99	Norway	193.70	2	yes
B99	Other and unspecified infectious diseases	A00.B99	Spain	76.30	3	yes
B99	Other and unspecified infectious diseases	A00.B99	Sweden	352.47	1	yes
B99	Other and unspecified infectious diseases	A00.B99	Switzerland	116.27	1	yes
B99	Other and unspecified infectious diseases	A00.B99	United Kingdom	22.50	2	yes
C00	Malignant neoplasm of lip	C00.D48	Croatia	13.54	1	yes
C00	Malignant neoplasm of lip	C00.D48	Czech Republic	14.75	0	no
C00	Malignant neoplasm of lip	C00.D48	Denmark	6.12	0	no
C00	Malignant neoplasm of lip	C00.D48	France	42.06	1	yes
C00	Malignant neoplasm of lip	C00.D48	Germany	44.31	0	no
C00	Malignant neoplasm of lip	C00.D48	Hungary	31.29	1	yes
C00	Malignant neoplasm of lip	C00.D48	Latvia	10.48	0	no
C00	Malignant neoplasm of lip	C00.D48	Lithuania	11.79	1	yes
C00	Malignant neoplasm of lip	C00.D48	Netherlands	9.01	0	no
C00	Malignant neoplasm of lip	C00.D48	Poland	177.06	3	yes
C00	Malignant neoplasm of lip	C00.D48	Romania	168.30	1	yes
C00	Malignant neoplasm of lip	C00.D48	Spain	66.63	1	yes
C00	Malignant neoplasm of lip	C00.D48	Sweden	5.83	0	no
C00	Malignant neoplasm of lip	C00.D48	United Kingdom	24.67	0	no
C01	Malignant neoplasm of base of tongue	C00.D48	Austria	79.35	2	yes
C01	Malignant neoplasm of base of tongue	C00.D48	Belgium	37.74	0	no
C01	Malignant neoplasm of base of tongue	C00.D48	Croatia	26.88	0	no
C01	Malignant neoplasm of base of tongue	C00.D48	Czech Republic	62.68	0	no
C01	Malignant neoplasm of base of tongue	C00.D48	Denmark	9.64	0	no
C01	Malignant neoplasm of base of tongue	C00.D48	Estonia	5.03	1	yes
C01	Malignant neoplasm of base of tongue	C00.D48	Finland	8.20	0	no
C01	Malignant neoplasm of base of tongue	C00.D48	France	375.84	1	yes
C01	Malignant neoplasm of base of tongue	C00.D48	Germany	525.23	0	no
C01	Malignant neoplasm of base of tongue	C00.D48	Hungary	251.33	0	no

For peer review only

C01	Malignant neoplasm of base of tongue	C00.D48	Latvia	14.33	1	yes
C01	Malignant neoplasm of base of tongue	C00.D48	Lithuania	29.48	0	no
C01	Malignant neoplasm of base of tongue	C00.D48	Netherlands	38.32	0	no
C01	Malignant neoplasm of base of tongue	C00.D48	Norway	6.53	1	yes
C01	Malignant neoplasm of base of tongue	C00.D48	Poland	125.22	3	yes
C01	Malignant neoplasm of base of tongue	C00.D48	Romania	117.99	1	yes
C01	Malignant neoplasm of base of tongue	C00.D48	Spain	133.89	2	yes
C01	Malignant neoplasm of base of tongue	C00.D48	Sweden	30.14	1	yes
C01	Malignant neoplasm of base of tongue	C00.D48	Switzerland	40.96	0	no
C01	Malignant neoplasm of base of tongue	C00.D48	United Kingdom	59.67	0	no
C02	Malignant neoplasm of other and unspecified parts of tongue	C00.D48	Austria	40.61	0	no
C02	Malignant neoplasm of other and unspecified parts of tongue	C00.D48	Belgium	70.54	3	yes
C02	Malignant neoplasm of other and unspecified parts of tongue	C00.D48	Croatia	62.73	1	yes
C02	Malignant neoplasm of other and unspecified parts of tongue	C00.D48	Czech Republic	103.43	1	yes
C02	Malignant neoplasm of other and unspecified parts of tongue	C00.D48	Denmark	44.16	0	no
C02	Malignant neoplasm of other and unspecified parts of tongue	C00.D48	Estonia	19.27	0	no
C02	Malignant neoplasm of other and unspecified parts of tongue	C00.D48	Finland	41.67	0	no
C02	Malignant neoplasm of other and unspecified parts of tongue	C00.D48	France	560.41	1	yes
C02	Malignant neoplasm of other and unspecified parts of tongue	C00.D48	Germany	301.54	3	yes
C02	Malignant neoplasm of other and unspecified parts of tongue	C00.D48	Hungary	174.13	1	yes
C02	Malignant neoplasm of other and unspecified parts of tongue	C00.D48	Latvia	21.68	0	no
C02	Malignant neoplasm of other and unspecified parts of tongue	C00.D48	Lithuania	28.45	0	no
C02	Malignant neoplasm of other and unspecified parts of tongue	C00.D48	Netherlands	88.33	0	no
C02	Malignant neoplasm of other and unspecified parts of tongue	C00.D48	Norway	30.23	0	no
C02	Malignant neoplasm of other and unspecified parts of tongue	C00.D48	Poland	345.93	3	yes
C02	Malignant neoplasm of other and unspecified parts of tongue	C00.D48	Romania	278.13	2	yes
C02	Malignant neoplasm of other and unspecified parts of tongue	C00.D48	Slovenia	13.17	0	no
C02	Malignant neoplasm of other and unspecified parts of tongue	C00.D48	Spain	455.86	0	no
C02	Malignant neoplasm of other and unspecified parts of tongue	C00.D48	Sweden	39.11	0	no
C02	Malignant neoplasm of other and unspecified parts of tongue	C00.D48	Switzerland	37.96	1	yes
C02	Malignant neoplasm of other and unspecified parts of tongue	C00.D48	United Kingdom	586.43	2	yes
C03	Malignant neoplasm of gum	C00.D48	Austria	19.94	3	yes
C03	Malignant neoplasm of gum	C00.D48	Croatia	7.63	0	no
C03	Malignant neoplasm of gum	C00.D48	Czech Republic	17.00	0	no
C03	Malignant neoplasm of gum	C00.D48	Denmark	8.38	0	no
C03	Malignant neoplasm of gum	C00.D48	Finland	16.64	0	no

For peer review only

C03	Malignant neoplasm of gum	C00.D48	France	49.26	0	no
C03	Malignant neoplasm of gum	C00.D48	Germany	49.45	1	yes
C03	Malignant neoplasm of gum	C00.D48	Hungary	33.09	2	yes
C03	Malignant neoplasm of gum	C00.D48	Netherlands	5.92	0	no
C03	Malignant neoplasm of gum	C00.D48	Norway	10.66	2	yes
C03	Malignant neoplasm of gum	C00.D48	Poland	38.53	1	yes
C03	Malignant neoplasm of gum	C00.D48	Romania	26.53	1	yes
C03	Malignant neoplasm of gum	C00.D48	Spain	36.90	0	no
C03	Malignant neoplasm of gum	C00.D48	Sweden	20.83	0	no
C03	Malignant neoplasm of gum	C00.D48	Switzerland	9.34	1	yes
C03	Malignant neoplasm of gum	C00.D48	United Kingdom	95.16	1	yes
C04	Malignant neoplasm of floor of mouth	C00.D48	Austria	68.75	0	no
C04	Malignant neoplasm of floor of mouth	C00.D48	Belgium	90.69	1	yes
C04	Malignant neoplasm of floor of mouth	C00.D48	Croatia	21.15	1	yes
C04	Malignant neoplasm of floor of mouth	C00.D48	Czech Republic	72.96	0	no
C04	Malignant neoplasm of floor of mouth	C00.D48	Denmark	16.38	2	yes
C04	Malignant neoplasm of floor of mouth	C00.D48	Estonia	8.64	0	no
C04	Malignant neoplasm of floor of mouth	C00.D48	Finland	16.71	0	no
C04	Malignant neoplasm of floor of mouth	C00.D48	France	308.86	1	yes
C04	Malignant neoplasm of floor of mouth	C00.D48	Germany	897.46	3	yes
C04	Malignant neoplasm of floor of mouth	C00.D48	Hungary	117.26	1	yes
C04	Malignant neoplasm of floor of mouth	C00.D48	Latvia	14.46	1	yes
C04	Malignant neoplasm of floor of mouth	C00.D48	Lithuania	33.08	1	yes
C04	Malignant neoplasm of floor of mouth	C00.D48	Netherlands	108.32	1	yes
C04	Malignant neoplasm of floor of mouth	C00.D48	Norway	10.56	2	yes
C04	Malignant neoplasm of floor of mouth	C00.D48	Poland	288.98	1	yes
C04	Malignant neoplasm of floor of mouth	C00.D48	Romania	178.40	0	no
C04	Malignant neoplasm of floor of mouth	C00.D48	Slovenia	10.98	0	no
C04	Malignant neoplasm of floor of mouth	C00.D48	Spain	182.01	1	yes
C04	Malignant neoplasm of floor of mouth	C00.D48	Sweden	20.90	2	yes
C04	Malignant neoplasm of floor of mouth	C00.D48	Switzerland	34.98	0	no
C04	Malignant neoplasm of floor of mouth	C00.D48	United Kingdom	76.80	2	yes
C05	Malignant neoplasm of palate	C00.D48	Austria	8.90	0	no
C05	Malignant neoplasm of palate	C00.D48	Belgium	12.69	0	no
C05	Malignant neoplasm of palate	C00.D48	Croatia	12.38	0	no
C05	Malignant neoplasm of palate	C00.D48	Czech Republic	25.30	0	no
C05	Malignant neoplasm of palate	C00.D48	Denmark	5.40	0	no
C05	Malignant neoplasm of palate	C00.D48	Finland	7.06	0	no
C05	Malignant neoplasm of palate	C00.D48	France	125.55	0	no
C05	Malignant neoplasm of palate	C00.D48	Germany	72.64	0	no
C05	Malignant neoplasm of palate	C00.D48	Hungary	43.92	0	no
C05	Malignant neoplasm of palate	C00.D48	Latvia	6.54	0	no
C05	Malignant neoplasm of palate	C00.D48	Lithuania	9.36	1	yes
C05	Malignant neoplasm of palate	C00.D48	Netherlands	10.53	0	no
C05	Malignant neoplasm of palate	C00.D48	Poland	73.60	2	yes
C05	Malignant neoplasm of palate	C00.D48	Romania	58.09	0	no
C05	Malignant neoplasm of palate	C00.D48	Spain	61.92	0	no
C05	Malignant neoplasm of palate	C00.D48	Sweden	7.42	0	no
C05	Malignant neoplasm of palate	C00.D48	Switzerland	9.19	2	yes
C05	Malignant neoplasm of palate	C00.D48	United Kingdom	81.77	0	no

For peer review only

C06	Malignant neoplasm of other and unspecified parts of mouth	C00.D48	Austria	9.67	0	no
C06	Malignant neoplasm of other and unspecified parts of mouth	C00.D48	Belgium	30.27	0	no
C06	Malignant neoplasm of other and unspecified parts of mouth	C00.D48	Croatia	18.86	0	no
C06	Malignant neoplasm of other and unspecified parts of mouth	C00.D48	Czech Republic	33.08	1	yes
C06	Malignant neoplasm of other and unspecified parts of mouth	C00.D48	Denmark	69.13	1	yes
C06	Malignant neoplasm of other and unspecified parts of mouth	C00.D48	Finland	12.15	1	yes
C06	Malignant neoplasm of other and unspecified parts of mouth	C00.D48	France	189.13	2	yes
C06	Malignant neoplasm of other and unspecified parts of mouth	C00.D48	Germany	108.38	2	yes
C06	Malignant neoplasm of other and unspecified parts of mouth	C00.D48	Hungary	76.93	0	no
C06	Malignant neoplasm of other and unspecified parts of mouth	C00.D48	Latvia	15.00	1	yes
C06	Malignant neoplasm of other and unspecified parts of mouth	C00.D48	Lithuania	11.43	1	yes
C06	Malignant neoplasm of other and unspecified parts of mouth	C00.D48	Netherlands	39.38	1	yes
C06	Malignant neoplasm of other and unspecified parts of mouth	C00.D48	Norway	21.22	0	no
C06	Malignant neoplasm of other and unspecified parts of mouth	C00.D48	Poland	138.57	0	no
C06	Malignant neoplasm of other and unspecified parts of mouth	C00.D48	Romania	77.35	0	no
C06	Malignant neoplasm of other and unspecified parts of mouth	C00.D48	Slovenia	14.28	0	no
C06	Malignant neoplasm of other and unspecified parts of mouth	C00.D48	Spain	246.18	0	no
C06	Malignant neoplasm of other and unspecified parts of mouth	C00.D48	Sweden	27.61	0	no
C06	Malignant neoplasm of other and unspecified parts of mouth	C00.D48	Switzerland	16.66	0	no
C06	Malignant neoplasm of other and unspecified parts of mouth	C00.D48	United Kingdom	304.74	2	yes
C07	Malignant neoplasm of parotid gland	C00.D48	Austria	33.33	0	no
C07	Malignant neoplasm of parotid gland	C00.D48	Belgium	29.77	2	yes
C07	Malignant neoplasm of parotid gland	C00.D48	Croatia	16.90	0	no
C07	Malignant neoplasm of parotid gland	C00.D48	Czech Republic	45.97	1	yes
C07	Malignant neoplasm of parotid gland	C00.D48	Denmark	19.56	0	no
C07	Malignant neoplasm of parotid gland	C00.D48	Estonia	6.31	0	no
C07	Malignant neoplasm of parotid gland	C00.D48	Finland	16.02	0	no
C07	Malignant neoplasm of parotid gland	C00.D48	France	197.64	0	no
C07	Malignant neoplasm of parotid gland	C00.D48	Germany	233.79	0	no
C07	Malignant neoplasm of parotid gland	C00.D48	Hungary	52.13	0	no
C07	Malignant neoplasm of parotid gland	C00.D48	Latvia	9.48	1	yes
C07	Malignant neoplasm of parotid gland	C00.D48	Lithuania	14.50	0	no
C07	Malignant neoplasm of parotid gland	C00.D48	Netherlands	41.98	0	no
C07	Malignant neoplasm of parotid gland	C00.D48	Norway	13.37	0	no
C07	Malignant neoplasm of parotid gland	C00.D48	Poland	167.35	0	no
C07	Malignant neoplasm of parotid gland	C00.D48	Romania	87.04	0	no
C07	Malignant neoplasm of parotid gland	C00.D48	Spain	152.25	0	no

For peer review only

C07	Malignant neoplasm of parotid gland	C00.D48	Sweden	22.56	0	no
C07	Malignant neoplasm of parotid gland	C00.D48	Switzerland	20.60	0	no
C07	Malignant neoplasm of parotid gland	C00.D48	United Kingdom	178.31	0	no
C08	Malignant neoplasm of other and unspecified major salivary glands	C00.D48	Austria	8.32	0	no
C08	Malignant neoplasm of other and unspecified major salivary glands	C00.D48	Belgium	6.80	2	yes
C08	Malignant neoplasm of other and unspecified major salivary glands	C00.D48	Croatia	5.62	0	no
C08	Malignant neoplasm of other and unspecified major salivary glands	C00.D48	Czech Republic	19.83	0	no
C08	Malignant neoplasm of other and unspecified major salivary glands	C00.D48	Denmark	6.32	1	yes
C08	Malignant neoplasm of other and unspecified major salivary glands	C00.D48	Finland	5.97	0	no
C08	Malignant neoplasm of other and unspecified major salivary glands	C00.D48	France	39.21	0	no
C08	Malignant neoplasm of other and unspecified major salivary glands	C00.D48	Germany	52.63	0	no
C08	Malignant neoplasm of other and unspecified major salivary glands	C00.D48	Hungary	25.41	0	no
C08	Malignant neoplasm of other and unspecified major salivary glands	C00.D48	Latvia	8.95	0	no
C08	Malignant neoplasm of other and unspecified major salivary glands	C00.D48	Lithuania	9.96	0	no
C08	Malignant neoplasm of other and unspecified major salivary glands	C00.D48	Netherlands	14.73	2	yes
C08	Malignant neoplasm of other and unspecified major salivary glands	C00.D48	Poland	85.62	2	yes
C08	Malignant neoplasm of other and unspecified major salivary glands	C00.D48	Romania	20.21	1	yes
C08	Malignant neoplasm of other and unspecified major salivary glands	C00.D48	Spain	25.15	1	yes
C08	Malignant neoplasm of other and unspecified major salivary glands	C00.D48	Sweden	12.83	0	no
C08	Malignant neoplasm of other and unspecified major salivary glands	C00.D48	Switzerland	5.53	0	no
C08	Malignant neoplasm of other and unspecified major salivary glands	C00.D48	United Kingdom	45.06	0	no
C09	Malignant neoplasm of tonsil	C00.D48	Austria	58.14	0	no
C09	Malignant neoplasm of tonsil	C00.D48	Belgium	45.70	2	yes
C09	Malignant neoplasm of tonsil	C00.D48	Croatia	33.59	0	no
C09	Malignant neoplasm of tonsil	C00.D48	Czech Republic	101.99	1	yes
C09	Malignant neoplasm of tonsil	C00.D48	Denmark	49.01	0	no
C09	Malignant neoplasm of tonsil	C00.D48	Estonia	5.50	1	yes
C09	Malignant neoplasm of tonsil	C00.D48	Finland	19.56	1	yes
C09	Malignant neoplasm of tonsil	C00.D48	France	416.60	2	yes
C09	Malignant neoplasm of tonsil	C00.D48	Germany	373.42	2	yes
C09	Malignant neoplasm of tonsil	C00.D48	Hungary	111.34	1	yes
C09	Malignant neoplasm of tonsil	C00.D48	Latvia	15.96	1	yes
C09	Malignant neoplasm of tonsil	C00.D48	Lithuania	18.23	0	no
C09	Malignant neoplasm of tonsil	C00.D48	Netherlands	32.47	1	yes
C09	Malignant neoplasm of tonsil	C00.D48	Norway	20.45	1	yes
C09	Malignant neoplasm of tonsil	C00.D48	Poland	267.79	1	yes
C09	Malignant neoplasm of tonsil	C00.D48	Romania	240.86	1	yes
C09	Malignant neoplasm of tonsil	C00.D48	Spain	157.66	0	no

For peer review only

C09	Malignant neoplasm of tonsil	C00.D48	Sweden	43.23	0	no
C09	Malignant neoplasm of tonsil	C00.D48	Switzerland	33.90	0	no
C09	Malignant neoplasm of tonsil	C00.D48	United Kingdom	245.10	1	yes
C10	Malignant neoplasm of oropharynx	C00.D48	Austria	57.21	1	yes
C10	Malignant neoplasm of oropharynx	C00.D48	Belgium	49.14	1	yes
C10	Malignant neoplasm of oropharynx	C00.D48	Croatia	37.31	1	yes
C10	Malignant neoplasm of oropharynx	C00.D48	Czech Republic	75.43	1	yes
C10	Malignant neoplasm of oropharynx	C00.D48	Denmark	35.20	2	yes
C10	Malignant neoplasm of oropharynx	C00.D48	Estonia	13.57	0	no
C10	Malignant neoplasm of oropharynx	C00.D48	Finland	5.95	2	yes
C10	Malignant neoplasm of oropharynx	C00.D48	France	567.96	2	yes
C10	Malignant neoplasm of oropharynx	C00.D48	Germany	918.14	1	yes
C10	Malignant neoplasm of oropharynx	C00.D48	Hungary	159.65	1	yes
C10	Malignant neoplasm of oropharynx	C00.D48	Latvia	7.71	3	yes
C10	Malignant neoplasm of oropharynx	C00.D48	Lithuania	37.27	3	yes
C10	Malignant neoplasm of oropharynx	C00.D48	Netherlands	87.43	0	no
C10	Malignant neoplasm of oropharynx	C00.D48	Norway	11.87	2	yes
C10	Malignant neoplasm of oropharynx	C00.D48	Poland	158.88	1	yes
C10	Malignant neoplasm of oropharynx	C00.D48	Romania	238.87	1	yes
C10	Malignant neoplasm of oropharynx	C00.D48	Slovenia	60.48	0	no
C10	Malignant neoplasm of oropharynx	C00.D48	Spain	306.33	2	yes
C10	Malignant neoplasm of oropharynx	C00.D48	Sweden	8.94	0	no
C10	Malignant neoplasm of oropharynx	C00.D48	Switzerland	51.20	0	no
C10	Malignant neoplasm of oropharynx	C00.D48	United Kingdom	233.90	3	yes
C11	Malignant neoplasm of nasopharynx	C00.D48	Austria	20.86	0	no
C11	Malignant neoplasm of nasopharynx	C00.D48	Belgium	18.95	0	no
C11	Malignant neoplasm of nasopharynx	C00.D48	Croatia	16.97	0	no
C11	Malignant neoplasm of nasopharynx	C00.D48	Czech Republic	33.27	0	no
C11	Malignant neoplasm of nasopharynx	C00.D48	Denmark	11.03	0	no
C11	Malignant neoplasm of nasopharynx	C00.D48	Estonia	5.33	0	no
C11	Malignant neoplasm of nasopharynx	C00.D48	Finland	7.62	0	no
C11	Malignant neoplasm of nasopharynx	C00.D48	France	142.89	1	yes
C11	Malignant neoplasm of nasopharynx	C00.D48	Germany	155.82	0	no
C11	Malignant neoplasm of nasopharynx	C00.D48	Hungary	48.80	0	no
C11	Malignant neoplasm of nasopharynx	C00.D48	Latvia	6.37	0	no
C11	Malignant neoplasm of nasopharynx	C00.D48	Lithuania	10.94	0	no
C11	Malignant neoplasm of nasopharynx	C00.D48	Netherlands	34.16	1	yes
C11	Malignant neoplasm of nasopharynx	C00.D48	Norway	6.87	2	yes
C11	Malignant neoplasm of nasopharynx	C00.D48	Poland	160.56	1	yes
C11	Malignant neoplasm of nasopharynx	C00.D48	Romania	183.56	0	no
C11	Malignant neoplasm of nasopharynx	C00.D48	Spain	221.66	1	yes
C11	Malignant neoplasm of nasopharynx	C00.D48	Sweden	16.23	1	yes
C11	Malignant neoplasm of nasopharynx	C00.D48	Switzerland	13.72	1	yes
C11	Malignant neoplasm of nasopharynx	C00.D48	United Kingdom	132.46	0	no
C12	Malignant neoplasm of pyriform sinus	C00.D48	Austria	5.71	0	no
C12	Malignant neoplasm of pyriform sinus	C00.D48	Belgium	20.60	0	no
C12	Malignant neoplasm of pyriform sinus	C00.D48	Czech Republic	12.21	0	no
C12	Malignant neoplasm of pyriform sinus	C00.D48	Estonia	5.59	1	yes
C12	Malignant neoplasm of pyriform sinus	C00.D48	France	452.13	2	yes
C12	Malignant neoplasm of pyriform sinus	C00.D48	Germany	6.04	0	no

For peer review only

C12	Malignant neoplasm of pyriform sinus	C00.D48	Hungary	10.41	0	no
C12	Malignant neoplasm of pyriform sinus	C00.D48	Lithuania	7.18	0	no
C12	Malignant neoplasm of pyriform sinus	C00.D48	Netherlands	10.51	1	yes
C12	Malignant neoplasm of pyriform sinus	C00.D48	Poland	26.12	3	yes
C12	Malignant neoplasm of pyriform sinus	C00.D48	Romania	56.71	1	yes
C12	Malignant neoplasm of pyriform sinus	C00.D48	Spain	113.60	1	yes
C12	Malignant neoplasm of pyriform sinus	C00.D48	Switzerland	14.19	1	yes
C12	Malignant neoplasm of pyriform sinus	C00.D48	United Kingdom	86.45	0	no
C13	Malignant neoplasm of hypopharynx	C00.D48	Austria	77.90	0	no
C13	Malignant neoplasm of hypopharynx	C00.D48	Belgium	37.78	0	no
C13	Malignant neoplasm of hypopharynx	C00.D48	Croatia	84.24	1	yes
C13	Malignant neoplasm of hypopharynx	C00.D48	Czech Republic	81.59	1	yes
C13	Malignant neoplasm of hypopharynx	C00.D48	Denmark	48.97	1	yes
C13	Malignant neoplasm of hypopharynx	C00.D48	Estonia	9.07	0	no
C13	Malignant neoplasm of hypopharynx	C00.D48	Finland	21.90	0	no
C13	Malignant neoplasm of hypopharynx	C00.D48	France	337.88	2	yes
C13	Malignant neoplasm of hypopharynx	C00.D48	Germany	984.18	1	yes
C13	Malignant neoplasm of hypopharynx	C00.D48	Hungary	291.49	1	yes
C13	Malignant neoplasm of hypopharynx	C00.D48	Latvia	24.63	1	yes
C13	Malignant neoplasm of hypopharynx	C00.D48	Lithuania	49.68	0	no
C13	Malignant neoplasm of hypopharynx	C00.D48	Netherlands	54.44	0	no
C13	Malignant neoplasm of hypopharynx	C00.D48	Norway	17.76	1	yes
C13	Malignant neoplasm of hypopharynx	C00.D48	Poland	211.55	2	yes
C13	Malignant neoplasm of hypopharynx	C00.D48	Romania	164.15	3	yes
C13	Malignant neoplasm of hypopharynx	C00.D48	Slovenia	32.51	0	no
C13	Malignant neoplasm of hypopharynx	C00.D48	Spain	189.71	0	no
C13	Malignant neoplasm of hypopharynx	C00.D48	Sweden	41.51	1	yes
C13	Malignant neoplasm of hypopharynx	C00.D48	Switzerland	55.32	0	no
C13	Malignant neoplasm of hypopharynx	C00.D48	United Kingdom	134.21	2	yes
C14	Malignant neoplasm of other and ill-defined sites in the lip, oral cavity and pharynx	C00.D48	Austria	21.73	0	no
C14	Malignant neoplasm of other and ill-defined sites in the lip, oral cavity and pharynx	C00.D48	Belgium	110.39	0	no
C14	Malignant neoplasm of other and ill-defined sites in the lip, oral cavity and pharynx	C00.D48	Croatia	24.02	1	yes
C14	Malignant neoplasm of other and ill-defined sites in the lip, oral cavity and pharynx	C00.D48	Czech Republic	35.50	1	yes
C14	Malignant neoplasm of other and ill-defined sites in the lip, oral cavity and pharynx	C00.D48	Denmark	19.52	1	yes
C14	Malignant neoplasm of other and ill-defined sites in the lip, oral cavity and pharynx	C00.D48	Estonia	5.59	1	yes
C14	Malignant neoplasm of other and ill-defined sites in the lip, oral cavity and pharynx	C00.D48	France	769.81	1	yes
C14	Malignant neoplasm of other and ill-defined sites in the lip, oral cavity and pharynx	C00.D48	Germany	243.93	1	yes
C14	Malignant neoplasm of other and ill-defined sites in the lip, oral cavity and pharynx	C00.D48	Hungary	210.09	2	yes
C14	Malignant neoplasm of other and ill-defined sites in the lip, oral cavity and pharynx	C00.D48	Latvia	19.44	3	yes
C14	Malignant neoplasm of other and ill-defined sites in the lip, oral cavity and pharynx	C00.D48	Lithuania	5.51	0	no
C14	Malignant neoplasm of other and ill-defined sites in the lip, oral cavity and pharynx	C00.D48	Netherlands	99.59	0	no
C14	Malignant neoplasm of other and ill-defined sites in the lip, oral cavity and pharynx	C00.D48	Norway	6.04	0	no

For peer review only

C14	Malignant neoplasm of other and ill-defined sites in the lip, oral cavity and pharynx	C00.D48	Poland	290.56	2	yes
C14	Malignant neoplasm of other and ill-defined sites in the lip, oral cavity and pharynx	C00.D48	Romania	293.84	2	yes
C14	Malignant neoplasm of other and ill-defined sites in the lip, oral cavity and pharynx	C00.D48	Slovenia	9.17	0	no
C14	Malignant neoplasm of other and ill-defined sites in the lip, oral cavity and pharynx	C00.D48	Spain	188.71	1	yes
C14	Malignant neoplasm of other and ill-defined sites in the lip, oral cavity and pharynx	C00.D48	Sweden	8.26	1	yes
C14	Malignant neoplasm of other and ill-defined sites in the lip, oral cavity and pharynx	C00.D48	Switzerland	71.73	1	yes
C14	Malignant neoplasm of other and ill-defined sites in the lip, oral cavity and pharynx	C00.D48	United Kingdom	238.72	0	no
C15	Malignant neoplasm of oesophagus	C00.D48	Austria	356.66	0	no
C15	Malignant neoplasm of oesophagus	C00.D48	Belgium	735.66	0	no
C15	Malignant neoplasm of oesophagus	C00.D48	Croatia	223.12	1	yes
C15	Malignant neoplasm of oesophagus	C00.D48	Czech Republic	515.38	0	no
C15	Malignant neoplasm of oesophagus	C00.D48	Denmark	449.43	1	yes
C15	Malignant neoplasm of oesophagus	C00.D48	Estonia	63.45	1	yes
C15	Malignant neoplasm of oesophagus	C00.D48	Finland	244.80	0	no
C15	Malignant neoplasm of oesophagus	C00.D48	France	4390.10	2	yes
C15	Malignant neoplasm of oesophagus	C00.D48	Germany	4879.38	1	yes
C15	Malignant neoplasm of oesophagus	C00.D48	Hungary	659.75	1	yes
C15	Malignant neoplasm of oesophagus	C00.D48	Latvia	121.07	1	yes
C15	Malignant neoplasm of oesophagus	C00.D48	Lithuania	193.08	1	yes
C15	Malignant neoplasm of oesophagus	C00.D48	Netherlands	1803.26	2	yes
C15	Malignant neoplasm of oesophagus	C00.D48	Norway	224.27	0	no
C15	Malignant neoplasm of oesophagus	C00.D48	Poland	1818.22	1	yes
C15	Malignant neoplasm of oesophagus	C00.D48	Romania	737.89	2	yes
C15	Malignant neoplasm of oesophagus	C00.D48	Slovenia	97.39	1	yes
C15	Malignant neoplasm of oesophagus	C00.D48	Spain	2061.63	2	yes
C15	Malignant neoplasm of oesophagus	C00.D48	Sweden	425.26	0	no
C15	Malignant neoplasm of oesophagus	C00.D48	Switzerland	475.80	0	no
C15	Malignant neoplasm of oesophagus	C00.D48	United Kingdom	8568.89	3	yes
C16	Malignant neoplasm of stomach	C00.D48	Austria	1070.57	2	yes
C16	Malignant neoplasm of stomach	C00.D48	Belgium	926.52	2	yes
C16	Malignant neoplasm of stomach	C00.D48	Croatia	1020.83	1	yes
C16	Malignant neoplasm of stomach	C00.D48	Czech Republic	1573.28	2	yes
C16	Malignant neoplasm of stomach	C00.D48	Denmark	451.24	1	yes
C16	Malignant neoplasm of stomach	C00.D48	Estonia	350.16	1	yes
C16	Malignant neoplasm of stomach	C00.D48	Finland	572.62	1	yes
C16	Malignant neoplasm of stomach	C00.D48	France	5362.07	2	yes
C16	Malignant neoplasm of stomach	C00.D48	Germany	10971.18	2	yes
C16	Malignant neoplasm of stomach	C00.D48	Hungary	2120.23	2	yes
C16	Malignant neoplasm of stomach	C00.D48	Latvia	591.28	1	yes
C16	Malignant neoplasm of stomach	C00.D48	Lithuania	853.87	2	yes
C16	Malignant neoplasm of stomach	C00.D48	Netherlands	1810.99	1	yes
C16	Malignant neoplasm of stomach	C00.D48	Norway	454.29	2	yes
C16	Malignant neoplasm of stomach	C00.D48	Poland	7313.83	2	yes
C16	Malignant neoplasm of stomach	C00.D48	Romania	4575.15	1	yes
C16	Malignant neoplasm of stomach	C00.D48	Slovenia	432.00	1	yes

For peer review only

C16	Malignant neoplasm of stomach	C00.D48	Spain	6473.17	3	yes
C16	Malignant neoplasm of stomach	C00.D48	Sweden	747.80	2	yes
C16	Malignant neoplasm of stomach	C00.D48	Switzerland	612.56	2	yes
C16	Malignant neoplasm of stomach	C00.D48	United Kingdom	6114.85	2	yes
C17	Malignant neoplasm of small intestine	C00.D48	Austria	55.76	1	yes
C17	Malignant neoplasm of small intestine	C00.D48	Belgium	58.20	0	no
C17	Malignant neoplasm of small intestine	C00.D48	Croatia	30.79	1	yes
C17	Malignant neoplasm of small intestine	C00.D48	Czech Republic	87.88	0	no
C17	Malignant neoplasm of small intestine	C00.D48	Denmark	53.46	1	yes
C17	Malignant neoplasm of small intestine	C00.D48	Estonia	12.00	0	no
C17	Malignant neoplasm of small intestine	C00.D48	Finland	49.50	1	yes
C17	Malignant neoplasm of small intestine	C00.D48	France	412.64	2	yes
C17	Malignant neoplasm of small intestine	C00.D48	Germany	454.50	2	yes
C17	Malignant neoplasm of small intestine	C00.D48	Hungary	77.66	2	yes
C17	Malignant neoplasm of small intestine	C00.D48	Latvia	13.67	0	no
C17	Malignant neoplasm of small intestine	C00.D48	Lithuania	19.97	0	no
C17	Malignant neoplasm of small intestine	C00.D48	Netherlands	152.30	2	yes
C17	Malignant neoplasm of small intestine	C00.D48	Norway	59.95	0	no
C17	Malignant neoplasm of small intestine	C00.D48	Poland	213.91	0	no
C17	Malignant neoplasm of small intestine	C00.D48	Romania	113.53	2	yes
C17	Malignant neoplasm of small intestine	C00.D48	Slovenia	15.87	0	no
C17	Malignant neoplasm of small intestine	C00.D48	Spain	198.52	1	yes
C17	Malignant neoplasm of small intestine	C00.D48	Sweden	113.99	0	no
C17	Malignant neoplasm of small intestine	C00.D48	Switzerland	58.17	0	no
C17	Malignant neoplasm of small intestine	C00.D48	United Kingdom	431.82	1	yes
C18	Malignant neoplasm of colon	C00.D48	Austria	1700.02	2	yes
C18	Malignant neoplasm of colon	C00.D48	Belgium	2543.42	2	yes
C18	Malignant neoplasm of colon	C00.D48	Croatia	1285.25	2	yes
C18	Malignant neoplasm of colon	C00.D48	Czech Republic	2985.96	1	yes
C18	Malignant neoplasm of colon	C00.D48	Denmark	1627.29	1	yes
C18	Malignant neoplasm of colon	C00.D48	Estonia	289.28	0	no
C18	Malignant neoplasm of colon	C00.D48	Finland	774.46	0	no
C18	Malignant neoplasm of colon	C00.D48	France	13674.13	2	yes
C18	Malignant neoplasm of colon	C00.D48	Germany	18779.55	3	yes
C18	Malignant neoplasm of colon	C00.D48	Hungary	3741.13	1	yes
C18	Malignant neoplasm of colon	C00.D48	Latvia	443.31	0	no
C18	Malignant neoplasm of colon	C00.D48	Lithuania	574.65	2	yes
C18	Malignant neoplasm of colon	C00.D48	Netherlands	4612.69	2	yes
C18	Malignant neoplasm of colon	C00.D48	Norway	1370.52	1	yes
C18	Malignant neoplasm of colon	C00.D48	Poland	9098.25	0	no
C18	Malignant neoplasm of colon	C00.D48	Romania	3919.18	1	yes
C18	Malignant neoplasm of colon	C00.D48	Slovenia	502.93	2	yes
C18	Malignant neoplasm of colon	C00.D48	Spain	11910.60	3	yes
C18	Malignant neoplasm of colon	C00.D48	Sweden	1885.22	1	yes
C18	Malignant neoplasm of colon	C00.D48	Switzerland	1340.83	3	yes
C18	Malignant neoplasm of colon	C00.D48	United Kingdom	11554.54	1	yes
C19	Malignant neoplasm of rectosigmoid junction	C00.D48	Austria	41.14	1	yes
C19	Malignant neoplasm of rectosigmoid junction	C00.D48	Belgium	137.27	0	no
C19	Malignant neoplasm of rectosigmoid junction	C00.D48	Croatia	97.35	1	yes
C19	Malignant neoplasm of rectosigmoid junction	C00.D48	Czech Republic	662.87	1	yes

For peer review only

For peer review only - http://bmjopen.bmj.com/site/about/guidelines.xhtml

C19	Malignant neoplasm of rectosigmoid junction	C00.D48	Denmark	24.36	1	yes
C19	Malignant neoplasm of rectosigmoid junction	C00.D48	Estonia	29.59	1	yes
C19	Malignant neoplasm of rectosigmoid junction	C00.D48	Finland	55.83	1	yes
C19	Malignant neoplasm of rectosigmoid junction	C00.D48	France	1022.25	1	yes
C19	Malignant neoplasm of rectosigmoid junction	C00.D48	Germany	477.69	1	yes
C19	Malignant neoplasm of rectosigmoid junction	C00.D48	Hungary	208.50	2	yes
C19	Malignant neoplasm of rectosigmoid junction	C00.D48	Latvia	42.27	3	yes
C19	Malignant neoplasm of rectosigmoid junction	C00.D48	Lithuania	68.07	0	no
C19	Malignant neoplasm of rectosigmoid junction	C00.D48	Netherlands	133.45	0	no
C19	Malignant neoplasm of rectosigmoid junction	C00.D48	Norway	35.63	2	yes
C19	Malignant neoplasm of rectosigmoid junction	C00.D48	Poland	383.56	2	yes
C19	Malignant neoplasm of rectosigmoid junction	C00.D48	Romania	759.16	0	no
C19	Malignant neoplasm of rectosigmoid junction	C00.D48	Slovenia	73.77	1	yes
C19	Malignant neoplasm of rectosigmoid junction	C00.D48	Spain	710.54	0	no
C19	Malignant neoplasm of rectosigmoid junction	C00.D48	Sweden	55.67	0	no
C19	Malignant neoplasm of rectosigmoid junction	C00.D48	Switzerland	86.25	1	yes
C19	Malignant neoplasm of rectosigmoid junction	C00.D48	United Kingdom	1996.47	1	yes
C20	Malignant neoplasm of rectum	C00.D48	Austria	744.27	1	yes
C20	Malignant neoplasm of rectum	C00.D48	Belgium	572.53	1	yes
C20	Malignant neoplasm of rectum	C00.D48	Croatia	722.33	1	yes
C20	Malignant neoplasm of rectum	C00.D48	Czech Republic	1328.01	1	yes
C20	Malignant neoplasm of rectum	C00.D48	Denmark	609.46	1	yes
C20	Malignant neoplasm of rectum	C00.D48	Estonia	146.78	1	yes
C20	Malignant neoplasm of rectum	C00.D48	Finland	401.33	1	yes
C20	Malignant neoplasm of rectum	C00.D48	France	3471.90	1	yes
C20	Malignant neoplasm of rectum	C00.D48	Germany	7909.38	1	yes
C20	Malignant neoplasm of rectum	C00.D48	Hungary	1790.47	1	yes
C20	Malignant neoplasm of rectum	C00.D48	Latvia	286.46	1	yes
C20	Malignant neoplasm of rectum	C00.D48	Lithuania	435.08	1	yes
C20	Malignant neoplasm of rectum	C00.D48	Netherlands	1189.43	0	no
C20	Malignant neoplasm of rectum	C00.D48	Norway	462.34	2	yes
C20	Malignant neoplasm of rectum	C00.D48	Poland	3669.19	3	yes
C20	Malignant neoplasm of rectum	C00.D48	Romania	1627.47	2	yes
C20	Malignant neoplasm of rectum	C00.D48	Slovenia	293.89	1	yes
C20	Malignant neoplasm of rectum	C00.D48	Spain	2987.13	0	no
C20	Malignant neoplasm of rectum	C00.D48	Sweden	773.71	0	no
C20	Malignant neoplasm of rectum	C00.D48	Switzerland	448.72	1	yes
C20	Malignant neoplasm of rectum	C00.D48	United Kingdom	4641.80	1	yes
C21	Malignant neoplasm of anus and anal canal	C00.D48	Austria	38.45	0	no
C21	Malignant neoplasm of anus and anal canal	C00.D48	Belgium	25.74	1	yes
C21	Malignant neoplasm of anus and anal canal	C00.D48	Croatia	10.64	0	no
C21	Malignant neoplasm of anus and anal canal	C00.D48	Czech Republic	87.52	2	yes
C21	Malignant neoplasm of anus and anal canal	C00.D48	Denmark	31.92	3	yes
C21	Malignant neoplasm of anus and anal canal	C00.D48	Estonia	8.74	0	no
C21	Malignant neoplasm of anus and anal canal	C00.D48	Finland	16.80	0	no
C21	Malignant neoplasm of anus and anal canal	C00.D48	France	321.18	1	yes
C21	Malignant neoplasm of anus and anal canal	C00.D48	Germany	383.44	3	yes
C21	Malignant neoplasm of anus and anal canal	C00.D48	Hungary	37.52	2	yes
C21	Malignant neoplasm of anus and anal canal	C00.D48	Latvia	10.42	0	no
C21	Malignant neoplasm of anus and anal canal	C00.D48	Lithuania	14.96	1	yes

For peer review only

C21	Malignant neoplasm of anus and anal canal	C00.D48	Netherlands	39.39	0	no
C21	Malignant neoplasm of anus and anal canal	C00.D48	Norway	18.97	0	no
C21	Malignant neoplasm of anus and anal canal	C00.D48	Poland	736.10	3	yes
C21	Malignant neoplasm of anus and anal canal	C00.D48	Romania	164.76	2	yes
C21	Malignant neoplasm of anus and anal canal	C00.D48	Slovenia	6.64	1	yes
C21	Malignant neoplasm of anus and anal canal	C00.D48	Spain	94.19	1	yes
C21	Malignant neoplasm of anus and anal canal	C00.D48	Sweden	38.64	0	no
C21	Malignant neoplasm of anus and anal canal	C00.D48	Switzerland	42.06	0	no
C21	Malignant neoplasm of anus and anal canal	C00.D48	United Kingdom	319.50	2	yes
C22	Malignant neoplasm of liver and intrahepatic bile ducts	C00.D48	Austria	901.16	0	no
C22	Malignant neoplasm of liver and intrahepatic bile ducts	C00.D48	Belgium	818.27	3	yes
C22	Malignant neoplasm of liver and intrahepatic bile ducts	C00.D48	Croatia	512.11	3	yes
C22	Malignant neoplasm of liver and intrahepatic bile ducts	C00.D48	Czech Republic	1042.29	2	yes
C22	Malignant neoplasm of liver and intrahepatic bile ducts	C00.D48	Denmark	372.93	1	yes
C22	Malignant neoplasm of liver and intrahepatic bile ducts	C00.D48	Estonia	97.51	0	no
C22	Malignant neoplasm of liver and intrahepatic bile ducts	C00.D48	Finland	473.65	0	no
C22	Malignant neoplasm of liver and intrahepatic bile ducts	C00.D48	France	8425.34	1	yes
C22	Malignant neoplasm of liver and intrahepatic bile ducts	C00.D48	Germany	6839.91	2	yes
C22	Malignant neoplasm of liver and intrahepatic bile ducts	C00.D48	Hungary	940.63	2	yes
C22	Malignant neoplasm of liver and intrahepatic bile ducts	C00.D48	Latvia	149.78	0	no
C22	Malignant neoplasm of liver and intrahepatic bile ducts	C00.D48	Lithuania	190.35	1	yes
C22	Malignant neoplasm of liver and intrahepatic bile ducts	C00.D48	Netherlands	809.49	2	yes
C22	Malignant neoplasm of liver and intrahepatic bile ducts	C00.D48	Norway	201.73	1	yes
C22	Malignant neoplasm of liver and intrahepatic bile ducts	C00.D48	Poland	2658.83	3	yes
C22	Malignant neoplasm of liver and intrahepatic bile ducts	C00.D48	Romania	3121.36	1	yes
C22	Malignant neoplasm of liver and intrahepatic bile ducts	C00.D48	Slovenia	210.22	1	yes
C22	Malignant neoplasm of liver and intrahepatic bile ducts	C00.D48	Spain	5259.94	3	yes
C22	Malignant neoplasm of liver and intrahepatic bile ducts	C00.D48	Sweden	660.07	0	no
C22	Malignant neoplasm of liver and intrahepatic bile ducts	C00.D48	Switzerland	680.96	1	yes
C22	Malignant neoplasm of liver and intrahepatic bile ducts	C00.D48	United Kingdom	4051.88	2	yes
C23	Malignant neoplasm of gallbladder	C00.D48	Austria	156.39	1	yes
C23	Malignant neoplasm of gallbladder	C00.D48	Belgium	84.86	1	yes
C23	Malignant neoplasm of gallbladder	C00.D48	Croatia	190.45	2	yes
C23	Malignant neoplasm of gallbladder	C00.D48	Czech Republic	577.63	1	yes
C23	Malignant neoplasm of gallbladder	C00.D48	Denmark	34.06	0	no
C23	Malignant neoplasm of gallbladder	C00.D48	Estonia	17.86	0	no

For peer review only

C23	Malignant neoplasm of gallbladder	C00.D48	Finland	92.55	2	yes
C23	Malignant neoplasm of gallbladder	C00.D48	France	487.65	2	yes
C23	Malignant neoplasm of gallbladder	C00.D48	Germany	1739.51	2	yes
C23	Malignant neoplasm of gallbladder	C00.D48	Hungary	479.73	2	yes
C23	Malignant neoplasm of gallbladder	C00.D48	Latvia	20.41	1	yes
C23	Malignant neoplasm of gallbladder	C00.D48	Lithuania	54.78	2	yes
C23	Malignant neoplasm of gallbladder	C00.D48	Netherlands	157.11	1	yes
C23	Malignant neoplasm of gallbladder	C00.D48	Norway	29.39	0	no
C23	Malignant neoplasm of gallbladder	C00.D48	Poland	1669.34	2	yes
C23	Malignant neoplasm of gallbladder	C00.D48	Romania	315.12	3	yes
C23	Malignant neoplasm of gallbladder	C00.D48	Slovenia	70.04	2	yes
C23	Malignant neoplasm of gallbladder	C00.D48	Spain	614.57	1	yes
C23	Malignant neoplasm of gallbladder	C00.D48	Sweden	202.71	1	yes
C23	Malignant neoplasm of gallbladder	C00.D48	Switzerland	91.88	1	yes
C23	Malignant neoplasm of gallbladder	C00.D48	United Kingdom	523.34	1	yes
C24	Malignant neoplasm of other and unspecified parts of biliary tract	C00.D48	Austria	186.41	0	no
C24	Malignant neoplasm of other and unspecified parts of biliary tract	C00.D48	Belgium	106.65	1	yes
C24	Malignant neoplasm of other and unspecified parts of biliary tract	C00.D48	Croatia	95.39	1	yes
C24	Malignant neoplasm of other and unspecified parts of biliary tract	C00.D48	Czech Republic	427.51	0	no
C24	Malignant neoplasm of other and unspecified parts of biliary tract	C00.D48	Denmark	104.90	0	no
C24	Malignant neoplasm of other and unspecified parts of biliary tract	C00.D48	Estonia	29.80	1	yes
C24	Malignant neoplasm of other and unspecified parts of biliary tract	C00.D48	Finland	132.69	1	yes
C24	Malignant neoplasm of other and unspecified parts of biliary tract	C00.D48	France	925.30	1	yes
C24	Malignant neoplasm of other and unspecified parts of biliary tract	C00.D48	Germany	2002.50	2	yes
C24	Malignant neoplasm of other and unspecified parts of biliary tract	C00.D48	Hungary	368.39	2	yes
C24	Malignant neoplasm of other and unspecified parts of biliary tract	C00.D48	Latvia	34.53	0	no
C24	Malignant neoplasm of other and unspecified parts of biliary tract	C00.D48	Lithuania	51.98	0	no
C24	Malignant neoplasm of other and unspecified parts of biliary tract	C00.D48	Netherlands	255.09	2	yes
C24	Malignant neoplasm of other and unspecified parts of biliary tract	C00.D48	Norway	62.42	2	yes
C24	Malignant neoplasm of other and unspecified parts of biliary tract	C00.D48	Poland	911.98	1	yes
C24	Malignant neoplasm of other and unspecified parts of biliary tract	C00.D48	Romania	305.84	2	yes
C24	Malignant neoplasm of other and unspecified parts of biliary tract	C00.D48	Slovenia	66.74	0	no
C24	Malignant neoplasm of other and unspecified parts of biliary tract	C00.D48	Spain	835.07	3	yes
C24	Malignant neoplasm of other and unspecified parts of biliary tract	C00.D48	Sweden	325.28	1	yes
C24	Malignant neoplasm of other and unspecified parts of biliary tract	C00.D48	Switzerland	118.69	0	no
C24	Malignant neoplasm of other and unspecified parts of biliary tract	C00.D48	United Kingdom	283.90	2	yes

C25	Malignant neoplasm of pancreas	C00.D48	Austria	1598.11	0	no
C25	Malignant neoplasm of pancreas	C00.D48	Belgium	1606.64	1	yes
C25	Malignant neoplasm of pancreas	C00.D48	Croatia	730.98	1	yes
C25	Malignant neoplasm of pancreas	C00.D48	Czech Republic	2212.87	0	no
C25	Malignant neoplasm of pancreas	C00.D48	Denmark	990.02	2	yes
C25	Malignant neoplasm of pancreas	C00.D48	Estonia	243.19	1	yes
C25	Malignant neoplasm of pancreas	C00.D48	Finland	1050.69	0	no
C25	Malignant neoplasm of pancreas	C00.D48	France	9530.08	1	yes
C25	Malignant neoplasm of pancreas	C00.D48	Germany	14771.19	1	yes
C25	Malignant neoplasm of pancreas	C00.D48	Hungary	2051.51	1	yes
C25	Malignant neoplasm of pancreas	C00.D48	Latvia	395.34	1	yes
C25	Malignant neoplasm of pancreas	C00.D48	Lithuania	513.50	0	no
C25	Malignant neoplasm of pancreas	C00.D48	Netherlands	2768.64	2	yes
C25	Malignant neoplasm of pancreas	C00.D48	Norway	793.44	0	no
C25	Malignant neoplasm of pancreas	C00.D48	Poland	5692.00	0	no
C25	Malignant neoplasm of pancreas	C00.D48	Romania	3110.30	2	yes
C25	Malignant neoplasm of pancreas	C00.D48	Slovenia	363.35	3	yes
C25	Malignant neoplasm of pancreas	C00.D48	Spain	5893.56	1	yes
C25	Malignant neoplasm of pancreas	C00.D48	Sweden	1643.02	1	yes
C25	Malignant neoplasm of pancreas	C00.D48	Switzerland	1169.38	1	yes
C25	Malignant neoplasm of pancreas	C00.D48	United Kingdom	8902.67	1	yes
C26	Malignant neoplasm of other and ill-defined digestive organs	C00.D48	Austria	62.47	1	yes
C26	Malignant neoplasm of other and ill-defined digestive organs	C00.D48	Belgium	450.82	1	yes
C26	Malignant neoplasm of other and ill-defined digestive organs	C00.D48	Croatia	42.32	0	no
C26	Malignant neoplasm of other and ill-defined digestive organs	C00.D48	Czech Republic	251.87	1	yes
C26	Malignant neoplasm of other and ill-defined digestive organs	C00.D48	Denmark	55.63	0	no
C26	Malignant neoplasm of other and ill-defined digestive organs	C00.D48	Estonia	16.87	2	yes
C26	Malignant neoplasm of other and ill-defined digestive organs	C00.D48	Finland	114.67	0	no
C26	Malignant neoplasm of other and ill-defined digestive organs	C00.D48	France	1914.40	1	yes
C26	Malignant neoplasm of other and ill-defined digestive organs	C00.D48	Germany	1202.50	1	yes
C26	Malignant neoplasm of other and ill-defined digestive organs	C00.D48	Hungary	109.11	2	yes
C26	Malignant neoplasm of other and ill-defined digestive organs	C00.D48	Latvia	18.82	1	yes
C26	Malignant neoplasm of other and ill-defined digestive organs	C00.D48	Lithuania	18.99	3	yes
C26	Malignant neoplasm of other and ill-defined digestive organs	C00.D48	Netherlands	1154.93	3	yes
C26	Malignant neoplasm of other and ill-defined digestive organs	C00.D48	Norway	71.38	1	yes
C26	Malignant neoplasm of other and ill-defined digestive organs	C00.D48	Poland	967.63	0	no
C26	Malignant neoplasm of other and ill-defined digestive organs	C00.D48	Romania	95.86	3	yes
C26	Malignant neoplasm of other and ill-defined digestive organs	C00.D48	Slovenia	27.50	2	yes

For peer review only

C26	Malignant neoplasm of other and ill-defined digestive organs	C00.D48	Spain	834.34	3	yes
C26	Malignant neoplasm of other and ill-defined digestive organs	C00.D48	Sweden	173.19	0	no
C26	Malignant neoplasm of other and ill-defined digestive organs	C00.D48	Switzerland	132.65	1	yes
C26	Malignant neoplasm of other and ill-defined digestive organs	C00.D48	United Kingdom	3207.88	2	yes
C30	Malignant neoplasm of nasal cavity and middle ear	C00.D48	Croatia	6.07	0	no
C30	Malignant neoplasm of nasal cavity and middle ear	C00.D48	Czech Republic	16.92	0	no
C30	Malignant neoplasm of nasal cavity and middle ear	C00.D48	Denmark	13.41	0	no
C30	Malignant neoplasm of nasal cavity and middle ear	C00.D48	Finland	8.81	0	no
C30	Malignant neoplasm of nasal cavity and middle ear	C00.D48	France	39.66	0	no
C30	Malignant neoplasm of nasal cavity and middle ear	C00.D48	Germany	32.76	1	yes
C30	Malignant neoplasm of nasal cavity and middle ear	C00.D48	Hungary	12.28	1	yes
C30	Malignant neoplasm of nasal cavity and middle ear	C00.D48	Netherlands	9.26	0	no
C30	Malignant neoplasm of nasal cavity and middle ear	C00.D48	Poland	58.23	2	yes
C30	Malignant neoplasm of nasal cavity and middle ear	C00.D48	Romania	60.71	1	yes
C30	Malignant neoplasm of nasal cavity and middle ear	C00.D48	Spain	31.56	0	no
C30	Malignant neoplasm of nasal cavity and middle ear	C00.D48	Sweden	5.14	0	no
C30	Malignant neoplasm of nasal cavity and middle ear	C00.D48	Switzerland	8.16	1	yes
C30	Malignant neoplasm of nasal cavity and middle ear	C00.D48	United Kingdom	37.21	1	yes
C31	Malignant neoplasm of accessory sinuses	C00.D48	Austria	18.52	0	no
C31	Malignant neoplasm of accessory sinuses	C00.D48	Belgium	26.35	0	no
C31	Malignant neoplasm of accessory sinuses	C00.D48	Croatia	12.34	0	no
C31	Malignant neoplasm of accessory sinuses	C00.D48	Czech Republic	27.32	0	no
C31	Malignant neoplasm of accessory sinuses	C00.D48	Denmark	9.97	0	no
C31	Malignant neoplasm of accessory sinuses	C00.D48	Estonia	6.38	0	no
C31	Malignant neoplasm of accessory sinuses	C00.D48	Finland	11.64	0	no
C31	Malignant neoplasm of accessory sinuses	C00.D48	France	162.75	1	yes
C31	Malignant neoplasm of accessory sinuses	C00.D48	Germany	144.54	0	no
C31	Malignant neoplasm of accessory sinuses	C00.D48	Hungary	29.21	0	no
C31	Malignant neoplasm of accessory sinuses	C00.D48	Latvia	7.52	0	no
C31	Malignant neoplasm of accessory sinuses	C00.D48	Lithuania	19.03	2	yes
C31	Malignant neoplasm of accessory sinuses	C00.D48	Netherlands	27.78	2	yes
C31	Malignant neoplasm of accessory sinuses	C00.D48	Norway	9.38	0	no
C31	Malignant neoplasm of accessory sinuses	C00.D48	Poland	98.28	2	yes
C31	Malignant neoplasm of accessory sinuses	C00.D48	Romania	71.89	2	yes
C31	Malignant neoplasm of accessory sinuses	C00.D48	Slovenia	7.07	0	no
C31	Malignant neoplasm of accessory sinuses	C00.D48	Spain	74.30	1	yes
C31	Malignant neoplasm of accessory sinuses	C00.D48	Sweden	15.03	0	no
C31	Malignant neoplasm of accessory sinuses	C00.D48	Switzerland	13.62	0	no
C31	Malignant neoplasm of accessory sinuses	C00.D48	United Kingdom	107.18	2	yes
C32	Malignant neoplasm of larynx	C00.D48	Austria	152.61	2	yes
C32	Malignant neoplasm of larynx	C00.D48	Belgium	247.59	2	yes
C32	Malignant neoplasm of larynx	C00.D48	Croatia	227.88	1	yes
C32	Malignant neoplasm of larynx	C00.D48	Czech Republic	287.98	1	yes
C32	Malignant neoplasm of larynx	C00.D48	Denmark	113.47	1	yes
C32	Malignant neoplasm of larynx	C00.D48	Estonia	41.42	1	yes
C32	Malignant neoplasm of larynx	C00.D48	Finland	41.83	0	no
C32	Malignant neoplasm of larynx	C00.D48	France	1565.17	2	yes

For peer review only

C32	Malignant neoplasm of larynx	C00.D48	Germany	1495.38	1	yes
C32	Malignant neoplasm of larynx	C00.D48	Hungary	586.14	1	yes
C32	Malignant neoplasm of larynx	C00.D48	Latvia	92.67	0	no
C32	Malignant neoplasm of larynx	C00.D48	Lithuania	143.30	0	no
C32	Malignant neoplasm of larynx	C00.D48	Netherlands	260.40	2	yes
C32	Malignant neoplasm of larynx	C00.D48	Norway	48.05	1	yes
C32	Malignant neoplasm of larynx	C00.D48	Poland	1937.08	1	yes
C32	Malignant neoplasm of larynx	C00.D48	Romania	1118.26	3	yes
C32	Malignant neoplasm of larynx	C00.D48	Slovenia	63.08	1	yes
C32	Malignant neoplasm of larynx	C00.D48	Spain	1765.89	3	yes
C32	Malignant neoplasm of larynx	C00.D48	Sweden	58.28	0	no
C32	Malignant neoplasm of larynx	C00.D48	Switzerland	103.44	2	yes
C32	Malignant neoplasm of larynx	C00.D48	United Kingdom	935.09	2	yes
C33	Malignant neoplasm of trachea	C00.D48	Belgium	8.51	1	yes
C33	Malignant neoplasm of trachea	C00.D48	Croatia	13.96	0	no
C33	Malignant neoplasm of trachea	C00.D48	Czech Republic	14.81	0	no
C33	Malignant neoplasm of trachea	C00.D48	France	62.98	1	yes
C33	Malignant neoplasm of trachea	C00.D48	Germany	35.01	0	no
C33	Malignant neoplasm of trachea	C00.D48	Hungary	28.67	0	no
C33	Malignant neoplasm of trachea	C00.D48	Netherlands	7.86	0	no
C33	Malignant neoplasm of trachea	C00.D48	Poland	51.14	1	yes
C33	Malignant neoplasm of trachea	C00.D48	Romania	14.38	0	no
C33	Malignant neoplasm of trachea	C00.D48	Spain	25.54	0	no
C33	Malignant neoplasm of trachea	C00.D48	United Kingdom	29.53	1	yes
C34	Malignant neoplasm of bronchus and lung	C00.D48	Austria	3922.22	2	yes
C34	Malignant neoplasm of bronchus and lung	C00.D48	Belgium	7008.28	1	yes
C34	Malignant neoplasm of bronchus and lung	C00.D48	Croatia	2920.29	0	no
C34	Malignant neoplasm of bronchus and lung	C00.D48	Czech Republic	6359.29	1	yes
C34	Malignant neoplasm of bronchus and lung	C00.D48	Denmark	4156.65	2	yes
C34	Malignant neoplasm of bronchus and lung	C00.D48	Estonia	735.25	1	yes
C34	Malignant neoplasm of bronchus and lung	C00.D48	Finland	2253.31	1	yes
C34	Malignant neoplasm of bronchus and lung	C00.D48	France	31489.35	2	yes
C34	Malignant neoplasm of bronchus and lung	C00.D48	Germany	41859.76	0	no
C34	Malignant neoplasm of bronchus and lung	C00.D48	Hungary	8998.97	0	no
C34	Malignant neoplasm of bronchus and lung	C00.D48	Latvia	1113.97	2	yes
C34	Malignant neoplasm of bronchus and lung	C00.D48	Lithuania	1571.89	1	yes
C34	Malignant neoplasm of bronchus and lung	C00.D48	Netherlands	11699.38	2	yes
C34	Malignant neoplasm of bronchus and lung	C00.D48	Norway	2501.41	2	yes
C34	Malignant neoplasm of bronchus and lung	C00.D48	Poland	27661.54	1	yes
C34	Malignant neoplasm of bronchus and lung	C00.D48	Romania	10946.56	1	yes
C34	Malignant neoplasm of bronchus and lung	C00.D48	Slovenia	1196.23	3	yes
C34	Malignant neoplasm of bronchus and lung	C00.D48	Spain	22762.25	3	yes
C34	Malignant neoplasm of bronchus and lung	C00.D48	Sweden	3644.45	2	yes
C34	Malignant neoplasm of bronchus and lung	C00.D48	Switzerland	3370.25	1	yes
C34	Malignant neoplasm of bronchus and lung	C00.D48	United Kingdom	39651.31	2	yes
C37	Malignant neoplasm of thymus	C00.D48	Austria	12.19	2	yes
C37	Malignant neoplasm of thymus	C00.D48	Belgium	10.59	0	no
C37	Malignant neoplasm of thymus	C00.D48	Czech Republic	11.42	1	yes
C37	Malignant neoplasm of thymus	C00.D48	France	56.81	0	no
C37	Malignant neoplasm of thymus	C00.D48	Germany	72.24	1	yes

For peer review only

C37	Malignant neoplasm of thymus	C00.D48	Hungary	8.60	1	yes
C37	Malignant neoplasm of thymus	C00.D48	Netherlands	17.64	0	no
C37	Malignant neoplasm of thymus	C00.D48	Poland	16.00	0	no
C37	Malignant neoplasm of thymus	C00.D48	Spain	39.06	1	yes
C37	Malignant neoplasm of thymus	C00.D48	Sweden	7.81	0	no
C37	Malignant neoplasm of thymus	C00.D48	Switzerland	8.41	0	no
C37	Malignant neoplasm of thymus	C00.D48	United Kingdom	45.19	1	yes
C38	Malignant neoplasm of heart, mediastinum and pleura	C00.D48	Austria	18.32	3	yes
C38	Malignant neoplasm of heart, mediastinum and pleura	C00.D48	Belgium	60.23	1	yes
C38	Malignant neoplasm of heart, mediastinum and pleura	C00.D48	Croatia	26.35	1	yes
C38	Malignant neoplasm of heart, mediastinum and pleura	C00.D48	Czech Republic	90.03	1	yes
C38	Malignant neoplasm of heart, mediastinum and pleura	C00.D48	Denmark	31.42	0	no
C38	Malignant neoplasm of heart, mediastinum and pleura	C00.D48	Estonia	7.14	0	no
C38	Malignant neoplasm of heart, mediastinum and pleura	C00.D48	Finland	28.43	1	yes
C38	Malignant neoplasm of heart, mediastinum and pleura	C00.D48	France	501.41	1	yes
C38	Malignant neoplasm of heart, mediastinum and pleura	C00.D48	Germany	225.22	2	yes
C38	Malignant neoplasm of heart, mediastinum and pleura	C00.D48	Hungary	80.11	2	yes
C38	Malignant neoplasm of heart, mediastinum and pleura	C00.D48	Latvia	11.94	0	no
C38	Malignant neoplasm of heart, mediastinum and pleura	C00.D48	Lithuania	19.42	0	no
C38	Malignant neoplasm of heart, mediastinum and pleura	C00.D48	Netherlands	36.72	1	yes
C38	Malignant neoplasm of heart, mediastinum and pleura	C00.D48	Norway	14.73	2	yes
C38	Malignant neoplasm of heart, mediastinum and pleura	C00.D48	Poland	425.78	1	yes
C38	Malignant neoplasm of heart, mediastinum and pleura	C00.D48	Romania	289.76	0	no
C38	Malignant neoplasm of heart, mediastinum and pleura	C00.D48	Slovenia	16.90	1	yes
C38	Malignant neoplasm of heart, mediastinum and pleura	C00.D48	Spain	165.56	1	yes
C38	Malignant neoplasm of heart, mediastinum and pleura	C00.D48	Sweden	33.40	2	yes
C38	Malignant neoplasm of heart, mediastinum and pleura	C00.D48	Switzerland	20.58	1	yes
C38	Malignant neoplasm of heart, mediastinum and pleura	C00.D48	United Kingdom	120.09	1	yes
C39	Malignant neoplasm of other and ill-defined sites in the respiratory system and intrathoracic organs	C00.D48	Belgium	13.66	2	yes
C39	Malignant neoplasm of other and ill-defined sites in the respiratory system and intrathoracic organs	C00.D48	Czech Republic	48.60	0	no
C39	Malignant neoplasm of other and ill-defined sites in the respiratory system and intrathoracic organs	C00.D48	Denmark	18.68	1	yes
C39	Malignant neoplasm of other and ill-defined sites in the respiratory system and intrathoracic organs	C00.D48	Finland	5.04	0	no
C39	Malignant neoplasm of other and ill-defined sites in the respiratory system and intrathoracic organs	C00.D48	France	938.81	2	yes

For peer review only

C39	Malignant neoplasm of other and ill-defined sites in the respiratory system and intrathoracic organs	C00.D48	Germany	24.14	1	yes
C39	Malignant neoplasm of other and ill-defined sites in the respiratory system and intrathoracic organs	C00.D48	Hungary	14.41	1	yes
C39	Malignant neoplasm of other and ill-defined sites in the respiratory system and intrathoracic organs	C00.D48	Poland	75.35	1	yes
C39	Malignant neoplasm of other and ill-defined sites in the respiratory system and intrathoracic organs	C00.D48	Romania	12.86	2	yes
C39	Malignant neoplasm of other and ill-defined sites in the respiratory system and intrathoracic organs	C00.D48	Spain	62.05	1	yes
C39	Malignant neoplasm of other and ill-defined sites in the respiratory system and intrathoracic organs	C00.D48	Sweden	7.08	1	yes
C39	Malignant neoplasm of other and ill-defined sites in the respiratory system and intrathoracic organs	C00.D48	United Kingdom	23.66	0	no
C40	Malignant neoplasm of bone and articular cartilage of limbs	C00.D48	Austria	6.69	0	no
C40	Malignant neoplasm of bone and articular cartilage of limbs	C00.D48	Belgium	9.62	0	no
C40	Malignant neoplasm of bone and articular cartilage of limbs	C00.D48	Croatia	28.36	0	no
C40	Malignant neoplasm of bone and articular cartilage of limbs	C00.D48	Czech Republic	32.02	1	yes
C40	Malignant neoplasm of bone and articular cartilage of limbs	C00.D48	Denmark	10.87	2	yes
C40	Malignant neoplasm of bone and articular cartilage of limbs	C00.D48	Estonia	5.78	0	no
C40	Malignant neoplasm of bone and articular cartilage of limbs	C00.D48	Finland	7.99	0	no
C40	Malignant neoplasm of bone and articular cartilage of limbs	C00.D48	France	62.95	1	yes
C40	Malignant neoplasm of bone and articular cartilage of limbs	C00.D48	Germany	53.13	0	no
C40	Malignant neoplasm of bone and articular cartilage of limbs	C00.D48	Hungary	13.09	2	yes
C40	Malignant neoplasm of bone and articular cartilage of limbs	C00.D48	Latvia	7.76	0	no
C40	Malignant neoplasm of bone and articular cartilage of limbs	C00.D48	Lithuania	11.95	1	yes
C40	Malignant neoplasm of bone and articular cartilage of limbs	C00.D48	Netherlands	7.48	1	yes
C40	Malignant neoplasm of bone and articular cartilage of limbs	C00.D48	Poland	139.48	2	yes
C40	Malignant neoplasm of bone and articular cartilage of limbs	C00.D48	Romania	122.38	1	yes
C40	Malignant neoplasm of bone and articular cartilage of limbs	C00.D48	Slovenia	5.22	0	no
C40	Malignant neoplasm of bone and articular cartilage of limbs	C00.D48	Spain	29.24	0	no
C40	Malignant neoplasm of bone and articular cartilage of limbs	C00.D48	Switzerland	6.21	0	no
C40	Malignant neoplasm of bone and articular cartilage of limbs	C00.D48	United Kingdom	37.37	1	yes
C41	Malignant neoplasm of bone and articular cartilage of other and unspecified sites	C00.D48	Austria	35.90	0	no
C41	Malignant neoplasm of bone and articular cartilage of other and unspecified sites	C00.D48	Belgium	81.49	0	no
C41	Malignant neoplasm of bone and articular cartilage of other and unspecified sites	C00.D48	Croatia	51.52	0	no
C41	Malignant neoplasm of bone and articular cartilage of other and unspecified sites	C00.D48	Czech Republic	68.07	1	yes

For peer review only

C41	Malignant neoplasm of bone and articular cartilage of other and unspecified sites	C00.D48	Denmark	19.75	0	no
C41	Malignant neoplasm of bone and articular cartilage of other and unspecified sites	C00.D48	Estonia	6.76	0	no
C41	Malignant neoplasm of bone and articular cartilage of other and unspecified sites	C00.D48	Finland	13.43	1	yes
C41	Malignant neoplasm of bone and articular cartilage of other and unspecified sites	C00.D48	France	530.20	1	yes
C41	Malignant neoplasm of bone and articular cartilage of other and unspecified sites	C00.D48	Germany	377.75	0	no
C41	Malignant neoplasm of bone and articular cartilage of other and unspecified sites	C00.D48	Hungary	58.02	2	yes
C41	Malignant neoplasm of bone and articular cartilage of other and unspecified sites	C00.D48	Latvia	21.58	1	yes
C41	Malignant neoplasm of bone and articular cartilage of other and unspecified sites	C00.D48	Lithuania	18.49	0	no
C41	Malignant neoplasm of bone and articular cartilage of other and unspecified sites	C00.D48	Netherlands	103.80	0	no
C41	Malignant neoplasm of bone and articular cartilage of other and unspecified sites	C00.D48	Norway	21.07	0	no
C41	Malignant neoplasm of bone and articular cartilage of other and unspecified sites	C00.D48	Poland	320.78	2	yes
C41	Malignant neoplasm of bone and articular cartilage of other and unspecified sites	C00.D48	Romania	441.29	1	yes
C41	Malignant neoplasm of bone and articular cartilage of other and unspecified sites	C00.D48	Slovenia	10.49	0	no
C41	Malignant neoplasm of bone and articular cartilage of other and unspecified sites	C00.D48	Spain	282.85	0	no
C41	Malignant neoplasm of bone and articular cartilage of other and unspecified sites	C00.D48	Sweden	39.14	1	yes
C41	Malignant neoplasm of bone and articular cartilage of other and unspecified sites	C00.D48	Switzerland	34.36	0	no
C41	Malignant neoplasm of bone and articular cartilage of other and unspecified sites	C00.D48	United Kingdom	283.26	0	no
C43	Malignant melanoma of skin	C00.D48	Austria	351.56	0	no
C43	Malignant melanoma of skin	C00.D48	Belgium	321.60	1	yes
C43	Malignant melanoma of skin	C00.D48	Croatia	208.57	0	no
C43	Malignant melanoma of skin	C00.D48	Czech Republic	423.28	1	yes
C43	Malignant melanoma of skin	C00.D48	Denmark	280.41	1	yes
C43	Malignant melanoma of skin	C00.D48	Estonia	57.67	2	yes
C43	Malignant melanoma of skin	C00.D48	Finland	206.27	1	yes
C43	Malignant melanoma of skin	C00.D48	France	1725.80	1	yes
C43	Malignant melanoma of skin	C00.D48	Germany	2579.33	3	yes
C43	Malignant melanoma of skin	C00.D48	Hungary	388.21	0	no
C43	Malignant melanoma of skin	C00.D48	Latvia	80.38	0	no
C43	Malignant melanoma of skin	C00.D48	Lithuania	108.89	2	yes
C43	Malignant melanoma of skin	C00.D48	Netherlands	787.26	3	yes
C43	Malignant melanoma of skin	C00.D48	Norway	332.58	2	yes
C43	Malignant melanoma of skin	C00.D48	Poland	1419.95	1	yes
C43	Malignant melanoma of skin	C00.D48	Romania	446.41	0	no
C43	Malignant melanoma of skin	C00.D48	Slovenia	118.51	2	yes
C43	Malignant melanoma of skin	C00.D48	Spain	968.92	1	yes
C43	Malignant melanoma of skin	C00.D48	Sweden	480.46	2	yes
C43	Malignant melanoma of skin	C00.D48	Switzerland	314.72	1	yes
C43	Malignant melanoma of skin	C00.D48	United Kingdom	2260.40	1	yes
C44	Other malignant neoplasms of skin	C00.D48	Austria	92.05	1	yes

For peer review only

C44	Other malignant neoplasms of skin	C00.D48	Belgium	108.14	2	yes
C44	Other malignant neoplasms of skin	C00.D48	Croatia	100.82	2	yes
C44	Other malignant neoplasms of skin	C00.D48	Czech Republic	192.77	3	yes
C44	Other malignant neoplasms of skin	C00.D48	Denmark	67.60	0	no
C44	Other malignant neoplasms of skin	C00.D48	Estonia	25.52	0	no
C44	Other malignant neoplasms of skin	C00.D48	Finland	50.35	0	no
C44	Other malignant neoplasms of skin	C00.D48	France	661.52	3	yes
C44	Other malignant neoplasms of skin	C00.D48	Germany	627.79	1	yes
C44	Other malignant neoplasms of skin	C00.D48	Hungary	232.15	0	no
C44	Other malignant neoplasms of skin	C00.D48	Latvia	56.94	0	no
C44	Other malignant neoplasms of skin	C00.D48	Lithuania	54.12	0	no
C44	Other malignant neoplasms of skin	C00.D48	Netherlands	113.53	0	no
C44	Other malignant neoplasms of skin	C00.D48	Norway	47.04	0	no
C44	Other malignant neoplasms of skin	C00.D48	Poland	784.68	3	yes
C44	Other malignant neoplasms of skin	C00.D48	Romania	611.52	1	yes
C44	Other malignant neoplasms of skin	C00.D48	Slovenia	35.58	2	yes
C44	Other malignant neoplasms of skin	C00.D48	Spain	596.62	3	yes
C44	Other malignant neoplasms of skin	C00.D48	Sweden	64.10	0	no
C44	Other malignant neoplasms of skin	C00.D48	Switzerland	90.72	1	yes
C44	Other malignant neoplasms of skin	C00.D48	United Kingdom	661.79	3	yes
C45	Mesothelioma	C00.D48	Austria	98.00	2	yes
C45	Mesothelioma	C00.D48	Belgium	225.75	1	yes
C45	Mesothelioma	C00.D48	Croatia	63.39	1	yes
C45	Mesothelioma	C00.D48	Czech Republic	53.75	0	no
C45	Mesothelioma	C00.D48	Denmark	97.43	1	yes
C45	Mesothelioma	C00.D48	Finland	90.62	0	no
C45	Mesothelioma	C00.D48	France	1016.65	1	yes
C45	Mesothelioma	C00.D48	Germany	1250.24	3	yes
C45	Mesothelioma	C00.D48	Hungary	39.64	0	no
C45	Mesothelioma	C00.D48	Latvia	9.49	0	no
C45	Mesothelioma	C00.D48	Lithuania	11.52	1	yes
C45	Mesothelioma	C00.D48	Netherlands	549.80	0	no
C45	Mesothelioma	C00.D48	Norway	75.01	2	yes
C45	Mesothelioma	C00.D48	Poland	200.56	2	yes
C45	Mesothelioma	C00.D48	Romania	70.17	0	no
C45	Mesothelioma	C00.D48	Slovenia	31.74	0	no
C45	Mesothelioma	C00.D48	Spain	386.71	3	yes
C45	Mesothelioma	C00.D48	Sweden	135.07	0	no
C45	Mesothelioma	C00.D48	Switzerland	168.30	1	yes
C45	Mesothelioma	C00.D48	United Kingdom	2464.46	3	yes
C46	Kaposi's sarcoma	C00.D48	Czech Republic	6.86	0	no
C46	Kaposi's sarcoma	C00.D48	France	14.70	0	no
C46	Kaposi's sarcoma	C00.D48	Germany	14.28	1	yes
C46	Kaposi's sarcoma	C00.D48	Poland	20.40	0	no
C46	Kaposi's sarcoma	C00.D48	Romania	29.17	0	no
C46	Kaposi's sarcoma	C00.D48	Spain	24.35	1	yes
C46	Kaposi's sarcoma	C00.D48	United Kingdom	8.07	0	no
C47	Malignant neoplasm of peripheral nerves and autonomic nervous system	C00.D48	Austria	6.26	2	yes
C47	Malignant neoplasm of peripheral nerves and autonomic nervous system	C00.D48	Czech Republic	8.82	0	no

For peer review only

C47	Malignant neoplasm of peripheral nerves and autonomic nervous system	C00.D48	France	23.54	0	no
C47	Malignant neoplasm of peripheral nerves and autonomic nervous system	C00.D48	Germany	48.92	1	yes
C47	Malignant neoplasm of peripheral nerves and autonomic nervous system	C00.D48	Hungary	7.99	2	yes
C47	Malignant neoplasm of peripheral nerves and autonomic nervous system	C00.D48	Netherlands	9.51	1	yes
C47	Malignant neoplasm of peripheral nerves and autonomic nervous system	C00.D48	Poland	17.23	0	no
C47	Malignant neoplasm of peripheral nerves and autonomic nervous system	C00.D48	Romania	8.22	0	no
C47	Malignant neoplasm of peripheral nerves and autonomic nervous system	C00.D48	Spain	18.80	0	no
C47	Malignant neoplasm of peripheral nerves and autonomic nervous system	C00.D48	Sweden	5.66	1	yes
C47	Malignant neoplasm of peripheral nerves and autonomic nervous system	C00.D48	Switzerland	11.17	2	yes
C47	Malignant neoplasm of peripheral nerves and autonomic nervous system	C00.D48	United Kingdom	30.47	1	yes
C48	Malignant neoplasm of retroperitoneum and peritoneum	C00.D48	Austria	49.04	0	no
C48	Malignant neoplasm of retroperitoneum and peritoneum	C00.D48	Belgium	28.33	0	no
C48	Malignant neoplasm of retroperitoneum and peritoneum	C00.D48	Croatia	22.13	1	yes
C48	Malignant neoplasm of retroperitoneum and peritoneum	C00.D48	Czech Republic	92.98	1	yes
C48	Malignant neoplasm of retroperitoneum and peritoneum	C00.D48	Denmark	17.32	0	no
C48	Malignant neoplasm of retroperitoneum and peritoneum	C00.D48	Estonia	8.21	0	no
C48	Malignant neoplasm of retroperitoneum and peritoneum	C00.D48	Finland	36.41	1	yes
C48	Malignant neoplasm of retroperitoneum and peritoneum	C00.D48	France	229.29	1	yes
C48	Malignant neoplasm of retroperitoneum and peritoneum	C00.D48	Germany	322.91	1	yes
C48	Malignant neoplasm of retroperitoneum and peritoneum	C00.D48	Hungary	81.28	1	yes
C48	Malignant neoplasm of retroperitoneum and peritoneum	C00.D48	Latvia	17.03	0	no
C48	Malignant neoplasm of retroperitoneum and peritoneum	C00.D48	Lithuania	31.36	0	no
C48	Malignant neoplasm of retroperitoneum and peritoneum	C00.D48	Netherlands	16.71	1	yes
C48	Malignant neoplasm of retroperitoneum and peritoneum	C00.D48	Norway	26.18	1	yes
C48	Malignant neoplasm of retroperitoneum and peritoneum	C00.D48	Poland	241.45	0	no
C48	Malignant neoplasm of retroperitoneum and peritoneum	C00.D48	Romania	488.63	0	no
C48	Malignant neoplasm of retroperitoneum and peritoneum	C00.D48	Slovenia	19.63	0	no
C48	Malignant neoplasm of retroperitoneum and peritoneum	C00.D48	Spain	233.13	2	yes
C48	Malignant neoplasm of retroperitoneum and peritoneum	C00.D48	Sweden	35.46	1	yes
C48	Malignant neoplasm of retroperitoneum and peritoneum	C00.D48	Switzerland	40.39	3	yes

For peer review only

C48	Malignant neoplasm of retroperitoneum and peritoneum	C00.D48	United Kingdom	366.17	1	yes
C49	Malignant neoplasm of other connective and soft tissue	C00.D48	Austria	122.28	0	no
C49	Malignant neoplasm of other connective and soft tissue	C00.D48	Belgium	152.98	0	no
C49	Malignant neoplasm of other connective and soft tissue	C00.D48	Croatia	37.96	1	yes
C49	Malignant neoplasm of other connective and soft tissue	C00.D48	Czech Republic	113.01	0	no
C49	Malignant neoplasm of other connective and soft tissue	C00.D48	Denmark	57.98	0	no
C49	Malignant neoplasm of other connective and soft tissue	C00.D48	Estonia	13.60	0	no
C49	Malignant neoplasm of other connective and soft tissue	C00.D48	Finland	80.10	0	no
C49	Malignant neoplasm of other connective and soft tissue	C00.D48	France	823.84	1	yes
C49	Malignant neoplasm of other connective and soft tissue	C00.D48	Germany	1083.73	3	yes
C49	Malignant neoplasm of other connective and soft tissue	C00.D48	Hungary	123.70	0	no
C49	Malignant neoplasm of other connective and soft tissue	C00.D48	Latvia	27.02	0	no
C49	Malignant neoplasm of other connective and soft tissue	C00.D48	Lithuania	38.80	0	no
C49	Malignant neoplasm of other connective and soft tissue	C00.D48	Netherlands	238.39	1	yes
C49	Malignant neoplasm of other connective and soft tissue	C00.D48	Norway	55.68	1	yes
C49	Malignant neoplasm of other connective and soft tissue	C00.D48	Poland	369.40	1	yes
C49	Malignant neoplasm of other connective and soft tissue	C00.D48	Romania	239.38	1	yes
C49	Malignant neoplasm of other connective and soft tissue	C00.D48	Slovenia	22.12	0	no
C49	Malignant neoplasm of other connective and soft tissue	C00.D48	Spain	523.62	1	yes
C49	Malignant neoplasm of other connective and soft tissue	C00.D48	Sweden	146.52	0	no
C49	Malignant neoplasm of other connective and soft tissue	C00.D48	Switzerland	102.90	1	yes
C49	Malignant neoplasm of other connective and soft tissue	C00.D48	United Kingdom	816.99	1	yes
C50	Malignant neoplasm of breast	C00.D48	Austria	1718.60	2	yes
C50	Malignant neoplasm of breast	C00.D48	Belgium	2570.34	3	yes
C50	Malignant neoplasm of breast	C00.D48	Croatia	1088.80	1	yes
C50	Malignant neoplasm of breast	C00.D48	Czech Republic	2234.32	1	yes
C50	Malignant neoplasm of breast	C00.D48	Denmark	1407.10	1	yes
C50	Malignant neoplasm of breast	C00.D48	Estonia	270.57	1	yes
C50	Malignant neoplasm of breast	C00.D48	Finland	920.10	1	yes
C50	Malignant neoplasm of breast	C00.D48	France	12812.81	3	yes
C50	Malignant neoplasm of breast	C00.D48	Germany	17880.02	2	yes
C50	Malignant neoplasm of breast	C00.D48	Hungary	2511.58	2	yes
C50	Malignant neoplasm of breast	C00.D48	Latvia	475.91	0	no
C50	Malignant neoplasm of breast	C00.D48	Lithuania	648.39	0	no
C50	Malignant neoplasm of breast	C00.D48	Netherlands	3915.76	2	yes
C50	Malignant neoplasm of breast	C00.D48	Norway	783.47	1	yes

For peer review only

C50	Malignant neoplasm of breast	C00.D48	Poland	6860.49	3	yes
C50	Malignant neoplasm of breast	C00.D48	Romania	3929.44	0	no
C50	Malignant neoplasm of breast	C00.D48	Slovenia	482.50	1	yes
C50	Malignant neoplasm of breast	C00.D48	Spain	6843.63	3	yes
C50	Malignant neoplasm of breast	C00.D48	Sweden	1527.74	1	yes
C50	Malignant neoplasm of breast	C00.D48	Switzerland	1523.91	1	yes
C50	Malignant neoplasm of breast	C00.D48	United Kingdom	13672.05	2	yes
C51	Malignant neoplasm of vulva	C00.D48	Austria	70.32	0	no
C51	Malignant neoplasm of vulva	C00.D48	Belgium	58.16	0	no
C51	Malignant neoplasm of vulva	C00.D48	Croatia	48.00	0	no
C51	Malignant neoplasm of vulva	C00.D48	Czech Republic	115.56	1	yes
C51	Malignant neoplasm of vulva	C00.D48	Denmark	38.86	1	yes
C51	Malignant neoplasm of vulva	C00.D48	Estonia	14.77	0	no
C51	Malignant neoplasm of vulva	C00.D48	Finland	37.25	0	no
C51	Malignant neoplasm of vulva	C00.D48	France	252.44	0	no
C51	Malignant neoplasm of vulva	C00.D48	Germany	735.78	1	yes
C51	Malignant neoplasm of vulva	C00.D48	Hungary	113.33	0	no
C51	Malignant neoplasm of vulva	C00.D48	Latvia	26.70	0	no
C51	Malignant neoplasm of vulva	C00.D48	Lithuania	31.94	0	no
C51	Malignant neoplasm of vulva	C00.D48	Netherlands	126.22	0	no
C51	Malignant neoplasm of vulva	C00.D48	Norway	30.05	0	no
C51	Malignant neoplasm of vulva	C00.D48	Poland	345.09	0	no
C51	Malignant neoplasm of vulva	C00.D48	Romania	171.86	0	no
C51	Malignant neoplasm of vulva	C00.D48	Slovenia	23.56	0	no
C51	Malignant neoplasm of vulva	C00.D48	Spain	323.32	1	yes
C51	Malignant neoplasm of vulva	C00.D48	Sweden	59.05	0	no
C51	Malignant neoplasm of vulva	C00.D48	Switzerland	35.80	0	no
C51	Malignant neoplasm of vulva	C00.D48	United Kingdom	448.06	0	no
C52	Malignant neoplasm of vagina	C00.D48	Austria	24.65	1	yes
C52	Malignant neoplasm of vagina	C00.D48	Belgium	17.31	0	no
C52	Malignant neoplasm of vagina	C00.D48	Croatia	9.97	0	no
C52	Malignant neoplasm of vagina	C00.D48	Czech Republic	26.36	1	yes
C52	Malignant neoplasm of vagina	C00.D48	Denmark	10.92	0	no
C52	Malignant neoplasm of vagina	C00.D48	Estonia	5.72	1	yes
C52	Malignant neoplasm of vagina	C00.D48	Finland	13.12	0	no
C52	Malignant neoplasm of vagina	C00.D48	France	84.74	0	no
C52	Malignant neoplasm of vagina	C00.D48	Germany	169.62	1	yes
C52	Malignant neoplasm of vagina	C00.D48	Hungary	35.50	0	no
C52	Malignant neoplasm of vagina	C00.D48	Latvia	7.40	0	no
C52	Malignant neoplasm of vagina	C00.D48	Lithuania	8.15	0	no
C52	Malignant neoplasm of vagina	C00.D48	Netherlands	27.70	0	no
C52	Malignant neoplasm of vagina	C00.D48	Norway	5.67	1	yes
C52	Malignant neoplasm of vagina	C00.D48	Poland	85.81	1	yes
C52	Malignant neoplasm of vagina	C00.D48	Romania	40.90	2	yes
C52	Malignant neoplasm of vagina	C00.D48	Slovenia	5.48	0	no
C52	Malignant neoplasm of vagina	C00.D48	Spain	55.90	2	yes
C52	Malignant neoplasm of vagina	C00.D48	Sweden	15.07	1	yes
C52	Malignant neoplasm of vagina	C00.D48	Switzerland	12.99	0	no
C52	Malignant neoplasm of vagina	C00.D48	United Kingdom	109.82	0	no
C53	Malignant neoplasm of cervix uteri	C00.D48	Austria	168.19	1	yes

For peer review only

C53	Malignant neoplasm of cervix uteri	C00.D48	Belgium	183.97	0	no
C53	Malignant neoplasm of cervix uteri	C00.D48	Croatia	121.65	1	yes
C53	Malignant neoplasm of cervix uteri	C00.D48	Czech Republic	399.68	2	yes
C53	Malignant neoplasm of cervix uteri	C00.D48	Denmark	129.06	1	yes
C53	Malignant neoplasm of cervix uteri	C00.D48	Estonia	69.62	0	no
C53	Malignant neoplasm of cervix uteri	C00.D48	Finland	61.13	1	yes
C53	Malignant neoplasm of cervix uteri	C00.D48	France	796.17	1	yes
C53	Malignant neoplasm of cervix uteri	C00.D48	Germany	1608.89	2	yes
C53	Malignant neoplasm of cervix uteri	C00.D48	Hungary	470.60	2	yes
C53	Malignant neoplasm of cervix uteri	C00.D48	Latvia	123.85	1	yes
C53	Malignant neoplasm of cervix uteri	C00.D48	Lithuania	242.80	1	yes
C53	Malignant neoplasm of cervix uteri	C00.D48	Netherlands	244.67	1	yes
C53	Malignant neoplasm of cervix uteri	C00.D48	Norway	92.95	1	yes
C53	Malignant neoplasm of cervix uteri	C00.D48	Poland	2079.94	1	yes
C53	Malignant neoplasm of cervix uteri	C00.D48	Romania	2005.11	3	yes
C53	Malignant neoplasm of cervix uteri	C00.D48	Slovenia	51.62	1	yes
C53	Malignant neoplasm of cervix uteri	C00.D48	Spain	659.79	2	yes
C53	Malignant neoplasm of cervix uteri	C00.D48	Sweden	151.59	0	no
C53	Malignant neoplasm of cervix uteri	C00.D48	Switzerland	91.57	1	yes
C53	Malignant neoplasm of cervix uteri	C00.D48	United Kingdom	1085.58	2	yes
C54	Malignant neoplasm of corpus uteri	C00.D48	Austria	168.00	1	yes
C54	Malignant neoplasm of corpus uteri	C00.D48	Belgium	214.64	0	no
C54	Malignant neoplasm of corpus uteri	C00.D48	Croatia	112.98	1	yes
C54	Malignant neoplasm of corpus uteri	C00.D48	Czech Republic	399.98	1	yes
C54	Malignant neoplasm of corpus uteri	C00.D48	Denmark	128.80	1	yes
C54	Malignant neoplasm of corpus uteri	C00.D48	Estonia	41.75	1	yes
C54	Malignant neoplasm of corpus uteri	C00.D48	Finland	185.56	0	no
C54	Malignant neoplasm of corpus uteri	C00.D48	France	799.43	2	yes
C54	Malignant neoplasm of corpus uteri	C00.D48	Germany	1401.66	3	yes
C54	Malignant neoplasm of corpus uteri	C00.D48	Hungary	333.97	2	yes
C54	Malignant neoplasm of corpus uteri	C00.D48	Latvia	98.30	1	yes
C54	Malignant neoplasm of corpus uteri	C00.D48	Lithuania	151.71	0	no
C54	Malignant neoplasm of corpus uteri	C00.D48	Netherlands	418.31	0	no
C54	Malignant neoplasm of corpus uteri	C00.D48	Norway	111.81	3	yes
C54	Malignant neoplasm of corpus uteri	C00.D48	Poland	1310.21	2	yes
C54	Malignant neoplasm of corpus uteri	C00.D48	Romania	415.01	2	yes
C54	Malignant neoplasm of corpus uteri	C00.D48	Slovenia	61.83	0	no
C54	Malignant neoplasm of corpus uteri	C00.D48	Spain	1081.65	3	yes
C54	Malignant neoplasm of corpus uteri	C00.D48	Sweden	169.27	0	no
C54	Malignant neoplasm of corpus uteri	C00.D48	Switzerland	183.35	1	yes
C54	Malignant neoplasm of corpus uteri	C00.D48	United Kingdom	1496.84	2	yes
C55	Malignant neoplasm of uterus, part unspecified	C00.D48	Austria	143.35	3	yes
C55	Malignant neoplasm of uterus, part unspecified	C00.D48	Belgium	186.48	1	yes
C55	Malignant neoplasm of uterus, part unspecified	C00.D48	Croatia	140.60	1	yes
C55	Malignant neoplasm of uterus, part unspecified	C00.D48	Czech Republic	169.79	3	yes
C55	Malignant neoplasm of uterus, part unspecified	C00.D48	Denmark	69.48	1	yes
C55	Malignant neoplasm of uterus, part unspecified	C00.D48	Estonia	23.38	1	yes
C55	Malignant neoplasm of uterus, part unspecified	C00.D48	Finland	10.07	0	no
C55	Malignant neoplasm of uterus, part unspecified	C00.D48	France	1812.00	2	yes
C55	Malignant neoplasm of uterus, part unspecified	C00.D48	Germany	1162.86	2	yes

For peer review only

C55	Malignant neoplasm of uterus, part unspecified	C00.D48	Hungary	143.49	1	yes
C55	Malignant neoplasm of uterus, part unspecified	C00.D48	Latvia	61.32	3	yes
C55	Malignant neoplasm of uterus, part unspecified	C00.D48	Lithuania	18.76	0	no
C55	Malignant neoplasm of uterus, part unspecified	C00.D48	Netherlands	113.36	1	yes
C55	Malignant neoplasm of uterus, part unspecified	C00.D48	Norway	67.75	3	yes
C55	Malignant neoplasm of uterus, part unspecified	C00.D48	Poland	468.15	1	yes
C55	Malignant neoplasm of uterus, part unspecified	C00.D48	Romania	329.55	2	yes
C55	Malignant neoplasm of uterus, part unspecified	C00.D48	Slovenia	51.46	0	no
C55	Malignant neoplasm of uterus, part unspecified	C00.D48	Spain	507.04	1	yes
C55	Malignant neoplasm of uterus, part unspecified	C00.D48	Sweden	178.56	0	no
C55	Malignant neoplasm of uterus, part unspecified	C00.D48	Switzerland	55.50	1	yes
C55	Malignant neoplasm of uterus, part unspecified	C00.D48	United Kingdom	567.18	1	yes
C56	Malignant neoplasm of ovary	C00.D48	Austria	556.52	1	yes
C56	Malignant neoplasm of ovary	C00.D48	Belgium	745.47	3	yes
C56	Malignant neoplasm of ovary	C00.D48	Croatia	327.79	0	no
C56	Malignant neoplasm of ovary	C00.D48	Czech Republic	845.55	2	yes
C56	Malignant neoplasm of ovary	C00.D48	Denmark	443.72	1	yes
C56	Malignant neoplasm of ovary	C00.D48	Estonia	115.30	1	yes
C56	Malignant neoplasm of ovary	C00.D48	Finland	349.88	0	no
C56	Malignant neoplasm of ovary	C00.D48	France	3736.47	1	yes
C56	Malignant neoplasm of ovary	C00.D48	Germany	5656.66	3	yes
C56	Malignant neoplasm of ovary	C00.D48	Hungary	760.92	0	no
C56	Malignant neoplasm of ovary	C00.D48	Latvia	212.10	0	no
C56	Malignant neoplasm of ovary	C00.D48	Lithuania	302.65	0	no
C56	Malignant neoplasm of ovary	C00.D48	Netherlands	1192.67	1	yes
C56	Malignant neoplasm of ovary	C00.D48	Norway	372.02	1	yes
C56	Malignant neoplasm of ovary	C00.D48	Poland	3015.81	0	no
C56	Malignant neoplasm of ovary	C00.D48	Romania	1149.40	1	yes
C56	Malignant neoplasm of ovary	C00.D48	Slovenia	153.17	0	no
C56	Malignant neoplasm of ovary	C00.D48	Spain	2049.74	1	yes
C56	Malignant neoplasm of ovary	C00.D48	Sweden	626.73	1	yes
C56	Malignant neoplasm of ovary	C00.D48	Switzerland	482.04	1	yes
C56	Malignant neoplasm of ovary	C00.D48	United Kingdom	4873.91	1	yes
C57	Malignant neoplasm of other and unspecified female genital organs	C00.D48	Austria	33.41	1	yes
C57	Malignant neoplasm of other and unspecified female genital organs	C00.D48	Belgium	50.47	1	yes
C57	Malignant neoplasm of other and unspecified female genital organs	C00.D48	Croatia	11.14	0	no
C57	Malignant neoplasm of other and unspecified female genital organs	C00.D48	Czech Republic	131.98	0	no
C57	Malignant neoplasm of other and unspecified female genital organs	C00.D48	Denmark	28.96	0	no
C57	Malignant neoplasm of other and unspecified female genital organs	C00.D48	Estonia	5.85	0	no
C57	Malignant neoplasm of other and unspecified female genital organs	C00.D48	Finland	52.42	2	yes
C57	Malignant neoplasm of other and unspecified female genital organs	C00.D48	France	209.21	2	yes
C57	Malignant neoplasm of other and unspecified female genital organs	C00.D48	Germany	310.71	2	yes
C57	Malignant neoplasm of other and unspecified female genital organs	C00.D48	Hungary	30.33	2	yes

For peer review only

C57	Malignant neoplasm of other and unspecified female genital organs	C00.D48	Latvia	7.21	0	no
C57	Malignant neoplasm of other and unspecified female genital organs	C00.D48	Lithuania	8.98	1	yes
C57	Malignant neoplasm of other and unspecified female genital organs	C00.D48	Netherlands	41.66	1	yes
C57	Malignant neoplasm of other and unspecified female genital organs	C00.D48	Norway	28.85	0	no
C57	Malignant neoplasm of other and unspecified female genital organs	C00.D48	Poland	364.30	1	yes
C57	Malignant neoplasm of other and unspecified female genital organs	C00.D48	Romania	89.90	1	yes
C57	Malignant neoplasm of other and unspecified female genital organs	C00.D48	Slovenia	8.34	2	yes
C57	Malignant neoplasm of other and unspecified female genital organs	C00.D48	Spain	183.25	2	yes
C57	Malignant neoplasm of other and unspecified female genital organs	C00.D48	Sweden	93.23	0	no
C57	Malignant neoplasm of other and unspecified female genital organs	C00.D48	Switzerland	40.53	2	yes
C57	Malignant neoplasm of other and unspecified female genital organs	C00.D48	United Kingdom	131.36	2	yes
C60	Malignant neoplasm of penis	C00.D48	Austria	15.10	0	no
C60	Malignant neoplasm of penis	C00.D48	Belgium	14.50	0	no
C60	Malignant neoplasm of penis	C00.D48	Croatia	10.52	0	no
C60	Malignant neoplasm of penis	C00.D48	Czech Republic	28.71	0	no
C60	Malignant neoplasm of penis	C00.D48	Denmark	13.33	0	no
C60	Malignant neoplasm of penis	C00.D48	Finland	7.45	0	no
C60	Malignant neoplasm of penis	C00.D48	France	81.42	0	no
C60	Malignant neoplasm of penis	C00.D48	Germany	147.75	1	yes
C60	Malignant neoplasm of penis	C00.D48	Hungary	28.74	2	yes
C60	Malignant neoplasm of penis	C00.D48	Latvia	7.39	0	no
C60	Malignant neoplasm of penis	C00.D48	Lithuania	9.60	0	no
C60	Malignant neoplasm of penis	C00.D48	Netherlands	31.43	1	yes
C60	Malignant neoplasm of penis	C00.D48	Norway	8.14	2	yes
C60	Malignant neoplasm of penis	C00.D48	Poland	111.95	1	yes
C60	Malignant neoplasm of penis	C00.D48	Romania	61.88	0	no
C60	Malignant neoplasm of penis	C00.D48	Spain	115.00	0	no
C60	Malignant neoplasm of penis	C00.D48	Sweden	21.23	0	no
C60	Malignant neoplasm of penis	C00.D48	Switzerland	14.05	1	yes
C60	Malignant neoplasm of penis	C00.D48	United Kingdom	125.25	0	no
C61	Malignant neoplasm of prostate	C00.D48	Austria	1282.20	2	yes
C61	Malignant neoplasm of prostate	C00.D48	Belgium	1662.94	2	yes
C61	Malignant neoplasm of prostate	C00.D48	Croatia	820.40	1	yes
C61	Malignant neoplasm of prostate	C00.D48	Czech Republic	1851.42	1	yes
C61	Malignant neoplasm of prostate	C00.D48	Denmark	1387.74	1	yes
C61	Malignant neoplasm of prostate	C00.D48	Estonia	260.25	1	yes
C61	Malignant neoplasm of prostate	C00.D48	Finland	957.34	1	yes
C61	Malignant neoplasm of prostate	C00.D48	France	9967.49	1	yes
C61	Malignant neoplasm of prostate	C00.D48	Germany	12531.83	2	yes
C61	Malignant neoplasm of prostate	C00.D48	Hungary	1509.39	2	yes
C61	Malignant neoplasm of prostate	C00.D48	Latvia	408.57	3	yes
C61	Malignant neoplasm of prostate	C00.D48	Lithuania	616.74	0	no
C61	Malignant neoplasm of prostate	C00.D48	Netherlands	3183.89	1	yes

For peer review only

C61	Malignant neoplasm of prostate	C00.D48	Norway	1264.97	1	yes
C61	Malignant neoplasm of prostate	C00.D48	Poland	5643.53	0	no
C61	Malignant neoplasm of prostate	C00.D48	Romania	2566.20	2	yes
C61	Malignant neoplasm of prostate	C00.D48	Slovenia	425.14	0	no
C61	Malignant neoplasm of prostate	C00.D48	Spain	6448.51	3	yes
C61	Malignant neoplasm of prostate	C00.D48	Sweden	2504.28	1	yes
C61	Malignant neoplasm of prostate	C00.D48	Switzerland	1517.17	1	yes
C61	Malignant neoplasm of prostate	C00.D48	United Kingdom	12073.19	3	yes
C62	Malignant neoplasm of testis	C00.D48	Austria	15.73	0	no
C62	Malignant neoplasm of testis	C00.D48	Belgium	12.01	1	yes
C62	Malignant neoplasm of testis	C00.D48	Croatia	15.13	0	no
C62	Malignant neoplasm of testis	C00.D48	Czech Republic	36.01	1	yes
C62	Malignant neoplasm of testis	C00.D48	Denmark	12.67	1	yes
C62	Malignant neoplasm of testis	C00.D48	Finland	6.50	1	yes
C62	Malignant neoplasm of testis	C00.D48	France	100.24	1	yes
C62	Malignant neoplasm of testis	C00.D48	Germany	163.72	1	yes
C62	Malignant neoplasm of testis	C00.D48	Hungary	47.01	0	no
C62	Malignant neoplasm of testis	C00.D48	Latvia	8.84	0	no
C62	Malignant neoplasm of testis	C00.D48	Lithuania	7.82	1	yes
C62	Malignant neoplasm of testis	C00.D48	Netherlands	26.36	0	no
C62	Malignant neoplasm of testis	C00.D48	Norway	10.46	0	no
C62	Malignant neoplasm of testis	C00.D48	Poland	123.73	3	yes
C62	Malignant neoplasm of testis	C00.D48	Romania	75.34	1	yes
C62	Malignant neoplasm of testis	C00.D48	Slovenia	6.01	0	no
C62	Malignant neoplasm of testis	C00.D48	Spain	44.37	0	no
C62	Malignant neoplasm of testis	C00.D48	Sweden	10.14	0	no
C62	Malignant neoplasm of testis	C00.D48	Switzerland	13.01	1	yes
C62	Malignant neoplasm of testis	C00.D48	United Kingdom	72.90	1	yes
C63	Malignant neoplasm of other and unspecified male genital organs	C00.D48	Czech Republic	19.72	0	no
C63	Malignant neoplasm of other and unspecified male genital organs	C00.D48	France	18.20	0	no
C63	Malignant neoplasm of other and unspecified male genital organs	C00.D48	Germany	37.55	1	yes
C63	Malignant neoplasm of other and unspecified male genital organs	C00.D48	Netherlands	5.43	1	yes
C63	Malignant neoplasm of other and unspecified male genital organs	C00.D48	Poland	16.33	0	no
C63	Malignant neoplasm of other and unspecified male genital organs	C00.D48	Romania	13.26	0	no
C63	Malignant neoplasm of other and unspecified male genital organs	C00.D48	Spain	18.66	1	yes
C63	Malignant neoplasm of other and unspecified male genital organs	C00.D48	United Kingdom	12.74	0	no
C64	Malignant neoplasm of kidney, except renal pelvis	C00.D48	Austria	469.33	2	yes
C64	Malignant neoplasm of kidney, except renal pelvis	C00.D48	Belgium	621.60	1	yes
C64	Malignant neoplasm of kidney, except renal pelvis	C00.D48	Croatia	355.04	1	yes
C64	Malignant neoplasm of kidney, except renal pelvis	C00.D48	Czech Republic	1346.12	2	yes
C64	Malignant neoplasm of kidney, except renal pelvis	C00.D48	Denmark	346.01	1	yes
C64	Malignant neoplasm of kidney, except renal pelvis	C00.D48	Estonia	132.05	1	yes
C64	Malignant neoplasm of kidney, except renal pelvis	C00.D48	Finland	383.39	1	yes
C64	Malignant neoplasm of kidney, except renal pelvis	C00.D48	France	3581.84	2	yes
C64	Malignant neoplasm of kidney, except renal pelvis	C00.D48	Germany	5198.87	2	yes

For peer review only

C64	Malignant neoplasm of kidney, except renal pelvis	C00.D48	Hungary	825.71	2	yes
C64	Malignant neoplasm of kidney, except renal pelvis	C00.D48	Latvia	223.56	0	no
C64	Malignant neoplasm of kidney, except renal pelvis	C00.D48	Lithuania	314.57	0	no
C64	Malignant neoplasm of kidney, except renal pelvis	C00.D48	Netherlands	1112.03	1	yes
C64	Malignant neoplasm of kidney, except renal pelvis	C00.D48	Norway	300.70	0	no
C64	Malignant neoplasm of kidney, except renal pelvis	C00.D48	Poland	3214.05	1	yes
C64	Malignant neoplasm of kidney, except renal pelvis	C00.D48	Romania	817.78	1	yes
C64	Malignant neoplasm of kidney, except renal pelvis	C00.D48	Slovenia	157.74	1	yes
C64	Malignant neoplasm of kidney, except renal pelvis	C00.D48	Spain	2026.79	1	yes
C64	Malignant neoplasm of kidney, except renal pelvis	C00.D48	Sweden	603.90	3	yes
C64	Malignant neoplasm of kidney, except renal pelvis	C00.D48	Switzerland	350.08	1	yes
C64	Malignant neoplasm of kidney, except renal pelvis	C00.D48	United Kingdom	4030.98	2	yes
C65	Malignant neoplasm of renal pelvis	C00.D48	Austria	20.70	2	yes
C65	Malignant neoplasm of renal pelvis	C00.D48	Belgium	14.09	0	no
C65	Malignant neoplasm of renal pelvis	C00.D48	Czech Republic	91.38	2	yes
C65	Malignant neoplasm of renal pelvis	C00.D48	Denmark	37.42	0	no
C65	Malignant neoplasm of renal pelvis	C00.D48	Estonia	6.73	1	yes
C65	Malignant neoplasm of renal pelvis	C00.D48	Finland	18.35	0	no
C65	Malignant neoplasm of renal pelvis	C00.D48	France	18.65	0	no
C65	Malignant neoplasm of renal pelvis	C00.D48	Germany	188.08	1	yes
C65	Malignant neoplasm of renal pelvis	C00.D48	Hungary	32.55	0	no
C65	Malignant neoplasm of renal pelvis	C00.D48	Lithuania	10.28	1	yes
C65	Malignant neoplasm of renal pelvis	C00.D48	Netherlands	17.83	0	no
C65	Malignant neoplasm of renal pelvis	C00.D48	Norway	14.74	3	yes
C65	Malignant neoplasm of renal pelvis	C00.D48	Poland	58.21	2	yes
C65	Malignant neoplasm of renal pelvis	C00.D48	Romania	71.57	0	no
C65	Malignant neoplasm of renal pelvis	C00.D48	Spain	42.96	2	yes
C65	Malignant neoplasm of renal pelvis	C00.D48	Sweden	50.26	2	yes
C65	Malignant neoplasm of renal pelvis	C00.D48	Switzerland	30.44	1	yes
C65	Malignant neoplasm of renal pelvis	C00.D48	United Kingdom	39.53	0	no
C66	Malignant neoplasm of ureter	C00.D48	Austria	9.41	0	no
C66	Malignant neoplasm of ureter	C00.D48	Belgium	26.60	0	no
C66	Malignant neoplasm of ureter	C00.D48	Croatia	8.17	1	yes
C66	Malignant neoplasm of ureter	C00.D48	Czech Republic	23.00	1	yes
C66	Malignant neoplasm of ureter	C00.D48	Denmark	13.51	1	yes
C66	Malignant neoplasm of ureter	C00.D48	Finland	10.76	0	no
C66	Malignant neoplasm of ureter	C00.D48	France	79.23	0	no
C66	Malignant neoplasm of ureter	C00.D48	Germany	89.30	0	no
C66	Malignant neoplasm of ureter	C00.D48	Hungary	19.07	0	no
C66	Malignant neoplasm of ureter	C00.D48	Netherlands	23.93	0	no
C66	Malignant neoplasm of ureter	C00.D48	Norway	11.85	0	no
C66	Malignant neoplasm of ureter	C00.D48	Poland	42.84	1	yes
C66	Malignant neoplasm of ureter	C00.D48	Romania	34.40	1	yes
C66	Malignant neoplasm of ureter	C00.D48	Spain	43.31	1	yes
C66	Malignant neoplasm of ureter	C00.D48	Sweden	16.96	0	no
C66	Malignant neoplasm of ureter	C00.D48	Switzerland	19.36	1	yes
C66	Malignant neoplasm of ureter	C00.D48	United Kingdom	167.86	1	yes
C67	Malignant neoplasm of bladder	C00.D48	Austria	570.44	2	yes
C67	Malignant neoplasm of bladder	C00.D48	Belgium	942.82	1	yes
C67	Malignant neoplasm of bladder	C00.D48	Croatia	434.26	2	yes

For peer review only

C67	Malignant neoplasm of bladder	C00.D48	Czech Republic	970.23	2	yes
C67	Malignant neoplasm of bladder	C00.D48	Denmark	623.45	1	yes
C67	Malignant neoplasm of bladder	C00.D48	Estonia	112.59	0	no
C67	Malignant neoplasm of bladder	C00.D48	Finland	288.22	1	yes
C67	Malignant neoplasm of bladder	C00.D48	France	5240.88	1	yes
C67	Malignant neoplasm of bladder	C00.D48	Germany	5795.20	2	yes
C67	Malignant neoplasm of bladder	C00.D48	Hungary	1021.10	0	no
C67	Malignant neoplasm of bladder	C00.D48	Latvia	216.68	2	yes
C67	Malignant neoplasm of bladder	C00.D48	Lithuania	274.20	1	yes
C67	Malignant neoplasm of bladder	C00.D48	Netherlands	1488.07	2	yes
C67	Malignant neoplasm of bladder	C00.D48	Norway	409.85	1	yes
C67	Malignant neoplasm of bladder	C00.D48	Poland	4157.07	0	no
C67	Malignant neoplasm of bladder	C00.D48	Romania	1760.75	1	yes
C67	Malignant neoplasm of bladder	C00.D48	Slovenia	196.58	0	no
C67	Malignant neoplasm of bladder	C00.D48	Spain	5261.15	3	yes
C67	Malignant neoplasm of bladder	C00.D48	Sweden	681.06	1	yes
C67	Malignant neoplasm of bladder	C00.D48	Switzerland	555.95	0	no
C67	Malignant neoplasm of bladder	C00.D48	United Kingdom	5767.70	2	yes
C68	Malignant neoplasm of other and unspecified urinary organs	C00.D48	Austria	139.18	2	yes
C68	Malignant neoplasm of other and unspecified urinary organs	C00.D48	Belgium	107.88	2	yes
C68	Malignant neoplasm of other and unspecified urinary organs	C00.D48	Croatia	5.15	1	yes
C68	Malignant neoplasm of other and unspecified urinary organs	C00.D48	Czech Republic	39.90	1	yes
C68	Malignant neoplasm of other and unspecified urinary organs	C00.D48	Denmark	15.73	1	yes
C68	Malignant neoplasm of other and unspecified urinary organs	C00.D48	Finland	7.67	0	no
C68	Malignant neoplasm of other and unspecified urinary organs	C00.D48	France	774.53	2	yes
C68	Malignant neoplasm of other and unspecified urinary organs	C00.D48	Germany	2156.94	3	yes
C68	Malignant neoplasm of other and unspecified urinary organs	C00.D48	Hungary	16.43	2	yes
C68	Malignant neoplasm of other and unspecified urinary organs	C00.D48	Netherlands	401.59	3	yes
C68	Malignant neoplasm of other and unspecified urinary organs	C00.D48	Norway	21.19	1	yes
C68	Malignant neoplasm of other and unspecified urinary organs	C00.D48	Poland	61.37	2	yes
C68	Malignant neoplasm of other and unspecified urinary organs	C00.D48	Romania	22.21	0	no
C68	Malignant neoplasm of other and unspecified urinary organs	C00.D48	Spain	374.65	3	yes
C68	Malignant neoplasm of other and unspecified urinary organs	C00.D48	Sweden	41.12	0	no
C68	Malignant neoplasm of other and unspecified urinary organs	C00.D48	Switzerland	62.63	0	no
C68	Malignant neoplasm of other and unspecified urinary organs	C00.D48	United Kingdom	139.74	2	yes
C69	Malignant neoplasm of eye and adnexa	C00.D48	Austria	24.70	1	yes
C69	Malignant neoplasm of eye and adnexa	C00.D48	Belgium	20.33	0	no
C69	Malignant neoplasm of eye and adnexa	C00.D48	Croatia	11.06	0	no
C69	Malignant neoplasm of eye and adnexa	C00.D48	Czech Republic	28.97	1	yes

For peer review only

C69	Malignant neoplasm of eye and adnexa	C00.D48	Denmark	15.07	0	no
C69	Malignant neoplasm of eye and adnexa	C00.D48	Estonia	5.76	0	no
C69	Malignant neoplasm of eye and adnexa	C00.D48	Finland	26.59	0	no
C69	Malignant neoplasm of eye and adnexa	C00.D48	France	160.68	0	no
C69	Malignant neoplasm of eye and adnexa	C00.D48	Germany	210.44	3	yes
C69	Malignant neoplasm of eye and adnexa	C00.D48	Hungary	35.11	0	no
C69	Malignant neoplasm of eye and adnexa	C00.D48	Latvia	8.27	2	yes
C69	Malignant neoplasm of eye and adnexa	C00.D48	Lithuania	13.85	0	no
C69	Malignant neoplasm of eye and adnexa	C00.D48	Netherlands	33.46	0	no
C69	Malignant neoplasm of eye and adnexa	C00.D48	Norway	8.01	2	yes
C69	Malignant neoplasm of eye and adnexa	C00.D48	Poland	122.04	2	yes
C69	Malignant neoplasm of eye and adnexa	C00.D48	Romania	69.55	0	no
C69	Malignant neoplasm of eye and adnexa	C00.D48	Spain	73.29	0	no
C69	Malignant neoplasm of eye and adnexa	C00.D48	Sweden	18.16	0	no
C69	Malignant neoplasm of eye and adnexa	C00.D48	Switzerland	23.78	2	yes
C69	Malignant neoplasm of eye and adnexa	C00.D48	United Kingdom	106.10	0	no
C70	Malignant neoplasm of meninges	C00.D48	Austria	8.91	0	no
C70	Malignant neoplasm of meninges	C00.D48	Belgium	5.68	0	no
C70	Malignant neoplasm of meninges	C00.D48	Croatia	12.21	2	yes
C70	Malignant neoplasm of meninges	C00.D48	Czech Republic	29.40	1	yes
C70	Malignant neoplasm of meninges	C00.D48	Denmark	6.92	0	no
C70	Malignant neoplasm of meninges	C00.D48	France	26.17	1	yes
C70	Malignant neoplasm of meninges	C00.D48	Germany	110.82	3	yes
C70	Malignant neoplasm of meninges	C00.D48	Hungary	14.27	1	yes
C70	Malignant neoplasm of meninges	C00.D48	Lithuania	5.29	0	no
C70	Malignant neoplasm of meninges	C00.D48	Netherlands	6.64	0	no
C70	Malignant neoplasm of meninges	C00.D48	Norway	5.25	0	no
C70	Malignant neoplasm of meninges	C00.D48	Poland	68.09	0	no
C70	Malignant neoplasm of meninges	C00.D48	Romania	83.36	3	yes
C70	Malignant neoplasm of meninges	C00.D48	Slovenia	9.64	2	yes
C70	Malignant neoplasm of meninges	C00.D48	Spain	47.54	1	yes
C70	Malignant neoplasm of meninges	C00.D48	Sweden	6.87	1	yes
C70	Malignant neoplasm of meninges	C00.D48	Switzerland	5.41	2	yes
C70	Malignant neoplasm of meninges	C00.D48	United Kingdom	26.75	0	no
C71	Malignant neoplasm of brain	C00.D48	Austria	525.38	2	yes
C71	Malignant neoplasm of brain	C00.D48	Belgium	620.15	0	no
C71	Malignant neoplasm of brain	C00.D48	Croatia	414.13	1	yes
C71	Malignant neoplasm of brain	C00.D48	Czech Republic	776.57	1	yes
C71	Malignant neoplasm of brain	C00.D48	Denmark	428.34	0	no
C71	Malignant neoplasm of brain	C00.D48	Estonia	99.54	0	no
C71	Malignant neoplasm of brain	C00.D48	Finland	323.65	0	no
C71	Malignant neoplasm of brain	C00.D48	France	3398.97	2	yes
C71	Malignant neoplasm of brain	C00.D48	Germany	5460.44	0	no
C71	Malignant neoplasm of brain	C00.D48	Hungary	675.68	2	yes
C71	Malignant neoplasm of brain	C00.D48	Latvia	167.98	2	yes
C71	Malignant neoplasm of brain	C00.D48	Lithuania	248.65	2	yes
C71	Malignant neoplasm of brain	C00.D48	Netherlands	938.20	2	yes
C71	Malignant neoplasm of brain	C00.D48	Norway	333.24	0	no
C71	Malignant neoplasm of brain	C00.D48	Poland	3252.55	2	yes
C71	Malignant neoplasm of brain	C00.D48	Romania	1554.64	2	yes

For peer review only

C71	Malignant neoplasm of brain	C00.D48	Slovenia	140.07	0	no
C71	Malignant neoplasm of brain	C00.D48	Spain	2868.23	0	no
C71	Malignant neoplasm of brain	C00.D48	Sweden	586.26	3	yes
C71	Malignant neoplasm of brain	C00.D48	Switzerland	492.74	0	no
C71	Malignant neoplasm of brain	C00.D48	United Kingdom	4087.68	3	yes
C72	Malignant neoplasm of spinal cord, cranial nerves and other parts of central nervous system	C00.D48	Belgium	36.35	0	no
C72	Malignant neoplasm of spinal cord, cranial nerves and other parts of central nervous system	C00.D48	Croatia	8.38	0	no
C72	Malignant neoplasm of spinal cord, cranial nerves and other parts of central nervous system	C00.D48	Czech Republic	27.10	0	no
C72	Malignant neoplasm of spinal cord, cranial nerves and other parts of central nervous system	C00.D48	Denmark	8.56	1	yes
C72	Malignant neoplasm of spinal cord, cranial nerves and other parts of central nervous system	C00.D48	Finland	5.34	1	yes
C72	Malignant neoplasm of spinal cord, cranial nerves and other parts of central nervous system	C00.D48	France	74.59	1	yes
C72	Malignant neoplasm of spinal cord, cranial nerves and other parts of central nervous system	C00.D48	Germany	34.99	3	yes
C72	Malignant neoplasm of spinal cord, cranial nerves and other parts of central nervous system	C00.D48	Hungary	20.93	2	yes
C72	Malignant neoplasm of spinal cord, cranial nerves and other parts of central nervous system	C00.D48	Lithuania	6.27	0	no
C72	Malignant neoplasm of spinal cord, cranial nerves and other parts of central nervous system	C00.D48	Netherlands	36.29	3	yes
C72	Malignant neoplasm of spinal cord, cranial nerves and other parts of central nervous system	C00.D48	Poland	103.90	1	yes
C72	Malignant neoplasm of spinal cord, cranial nerves and other parts of central nervous system	C00.D48	Romania	42.36	1	yes
C72	Malignant neoplasm of spinal cord, cranial nerves and other parts of central nervous system	C00.D48	Spain	39.69	1	yes
C72	Malignant neoplasm of spinal cord, cranial nerves and other parts of central nervous system	C00.D48	Sweden	6.73	3	yes
C72	Malignant neoplasm of spinal cord, cranial nerves and other parts of central nervous system	C00.D48	United Kingdom	19.53	0	no
C73	Malignant neoplasm of thyroid gland	C00.D48	Austria	84.96	1	yes
C73	Malignant neoplasm of thyroid gland	C00.D48	Belgium	84.17	0	no
C73	Malignant neoplasm of thyroid gland	C00.D48	Croatia	41.95	0	no
C73	Malignant neoplasm of thyroid gland	C00.D48	Czech Republic	104.84	1	yes
C73	Malignant neoplasm of thyroid gland	C00.D48	Denmark	41.02	0	no
C73	Malignant neoplasm of thyroid gland	C00.D48	Estonia	18.69	0	no
C73	Malignant neoplasm of thyroid gland	C00.D48	Finland	53.88	0	no
C73	Malignant neoplasm of thyroid gland	C00.D48	France	449.10	1	yes
C73	Malignant neoplasm of thyroid gland	C00.D48	Germany	758.98	2	yes
C73	Malignant neoplasm of thyroid gland	C00.D48	Hungary	109.51	1	yes
C73	Malignant neoplasm of thyroid gland	C00.D48	Latvia	31.23	0	no
C73	Malignant neoplasm of thyroid gland	C00.D48	Lithuania	34.29	1	yes
C73	Malignant neoplasm of thyroid gland	C00.D48	Netherlands	121.77	0	no
C73	Malignant neoplasm of thyroid gland	C00.D48	Norway	42.25	0	no
C73	Malignant neoplasm of thyroid gland	C00.D48	Poland	365.73	2	yes
C73	Malignant neoplasm of thyroid gland	C00.D48	Romania	188.22	0	no
C73	Malignant neoplasm of thyroid gland	C00.D48	Slovenia	18.58	1	yes
C73	Malignant neoplasm of thyroid gland	C00.D48	Spain	328.20	0	no
C73	Malignant neoplasm of thyroid gland	C00.D48	Sweden	79.34	0	no
C73	Malignant neoplasm of thyroid gland	C00.D48	Switzerland	74.97	1	yes

For peer review only

C73	Malignant neoplasm of thyroid gland	C00.D48	United Kingdom	397.53	0	no
C74	Malignant neoplasm of adrenal gland	C00.D48	Austria	19.90	0	no
C74	Malignant neoplasm of adrenal gland	C00.D48	Belgium	22.22	1	yes
C74	Malignant neoplasm of adrenal gland	C00.D48	Croatia	16.71	0	no
C74	Malignant neoplasm of adrenal gland	C00.D48	Czech Republic	27.46	0	no
C74	Malignant neoplasm of adrenal gland	C00.D48	Denmark	13.17	2	yes
C74	Malignant neoplasm of adrenal gland	C00.D48	Finland	14.17	0	no
C74	Malignant neoplasm of adrenal gland	C00.D48	France	109.02	1	yes
C74	Malignant neoplasm of adrenal gland	C00.D48	Germany	139.88	0	no
C74	Malignant neoplasm of adrenal gland	C00.D48	Hungary	42.53	0	no
C74	Malignant neoplasm of adrenal gland	C00.D48	Latvia	9.76	0	no
C74	Malignant neoplasm of adrenal gland	C00.D48	Lithuania	11.51	0	no
C74	Malignant neoplasm of adrenal gland	C00.D48	Netherlands	39.28	0	no
C74	Malignant neoplasm of adrenal gland	C00.D48	Norway	10.98	0	no
C74	Malignant neoplasm of adrenal gland	C00.D48	Poland	121.16	1	yes
C74	Malignant neoplasm of adrenal gland	C00.D48	Romania	66.42	2	yes
C74	Malignant neoplasm of adrenal gland	C00.D48	Slovenia	7.85	0	no
C74	Malignant neoplasm of adrenal gland	C00.D48	Spain	107.99	0	no
C74	Malignant neoplasm of adrenal gland	C00.D48	Sweden	19.51	0	no
C74	Malignant neoplasm of adrenal gland	C00.D48	Switzerland	15.34	0	no
C74	Malignant neoplasm of adrenal gland	C00.D48	United Kingdom	122.87	0	no
C75	Malignant neoplasm of other endocrine glands and related structures	C00.D48	Austria	9.66	3	yes
C75	Malignant neoplasm of other endocrine glands and related structures	C00.D48	Belgium	30.11	1	yes
C75	Malignant neoplasm of other endocrine glands and related structures	C00.D48	Croatia	6.67	0	no
C75	Malignant neoplasm of other endocrine glands and related structures	C00.D48	Czech Republic	11.29	0	no
C75	Malignant neoplasm of other endocrine glands and related structures	C00.D48	France	56.26	3	yes
C75	Malignant neoplasm of other endocrine glands and related structures	C00.D48	Germany	320.15	1	yes
C75	Malignant neoplasm of other endocrine glands and related structures	C00.D48	Hungary	15.38	0	no
C75	Malignant neoplasm of other endocrine glands and related structures	C00.D48	Netherlands	61.02	2	yes
C75	Malignant neoplasm of other endocrine glands and related structures	C00.D48	Poland	40.57	0	no
C75	Malignant neoplasm of other endocrine glands and related structures	C00.D48	Romania	42.02	0	no
C75	Malignant neoplasm of other endocrine glands and related structures	C00.D48	Spain	82.73	2	yes
C75	Malignant neoplasm of other endocrine glands and related structures	C00.D48	Switzerland	31.08	1	yes
C75	Malignant neoplasm of other endocrine glands and related structures	C00.D48	United Kingdom	58.79	2	yes
C76	Malignant neoplasm of other and ill-defined sites	C00.D48	Austria	50.50	1	yes
C76	Malignant neoplasm of other and ill-defined sites	C00.D48	Belgium	302.33	0	no
C76	Malignant neoplasm of other and ill-defined sites	C00.D48	Croatia	161.86	1	yes
C76	Malignant neoplasm of other and ill-defined sites	C00.D48	Czech Republic	166.51	2	yes
C76	Malignant neoplasm of other and ill-defined sites	C00.D48	Denmark	212.68	3	yes
C76	Malignant neoplasm of other and ill-defined sites	C00.D48	Estonia	5.20	0	no
C76	Malignant neoplasm of other and ill-defined sites	C00.D48	Finland	74.86	1	yes
C76	Malignant neoplasm of other and ill-defined sites	C00.D48	France	3044.32	2	yes

For peer review only

For peer review only - http://bmjopen.bmj.com/site/about/guidelines.xhtml

C76	Malignant neoplasm of other and ill-defined sites	C00.D48	Germany	571.36	3	yes
C76	Malignant neoplasm of other and ill-defined sites	C00.D48	Hungary	148.95	2	yes
C76	Malignant neoplasm of other and ill-defined sites	C00.D48	Latvia	29.16	3	yes
C76	Malignant neoplasm of other and ill-defined sites	C00.D48	Lithuania	17.83	1	yes
C76	Malignant neoplasm of other and ill-defined sites	C00.D48	Netherlands	369.28	1	yes
C76	Malignant neoplasm of other and ill-defined sites	C00.D48	Norway	58.04	1	yes
C76	Malignant neoplasm of other and ill-defined sites	C00.D48	Poland	1137.81	0	no
C76	Malignant neoplasm of other and ill-defined sites	C00.D48	Romania	315.25	0	no
C76	Malignant neoplasm of other and ill-defined sites	C00.D48	Slovenia	23.14	2	yes
C76	Malignant neoplasm of other and ill-defined sites	C00.D48	Spain	663.90	1	yes
C76	Malignant neoplasm of other and ill-defined sites	C00.D48	Sweden	217.00	1	yes
C76	Malignant neoplasm of other and ill-defined sites	C00.D48	Switzerland	71.15	3	yes
C76	Malignant neoplasm of other and ill-defined sites	C00.D48	United Kingdom	632.16	1	yes
C77	Secondary and unspecified malignant neoplasm of lymph nodes	C00.D48	Croatia	8.39	0	no
C78	Secondary malignant neoplasm of respiratory and digestive organs	C00.D48	Croatia	112.11	2	yes
C78	Secondary malignant neoplasm of respiratory and digestive organs	C00.D48	Czech Republic	51.51	2	yes
C78	Secondary malignant neoplasm of respiratory and digestive organs	C00.D48	Finland	25.16	2	yes
C78	Secondary malignant neoplasm of respiratory and digestive organs	C00.D48	Latvia	14.46	2	yes
C78	Secondary malignant neoplasm of respiratory and digestive organs	C00.D48	Slovenia	58.16	0	no
C79	Secondary malignant neoplasm of other sites	C00.D48	Croatia	40.06	1	yes
C79	Secondary malignant neoplasm of other sites	C00.D48	Czech Republic	25.23	0	no
C79	Secondary malignant neoplasm of other sites	C00.D48	Latvia	8.77	1	yes
C79	Secondary malignant neoplasm of other sites	C00.D48	Slovenia	16.08	0	no
C80	Malignant neoplasm without specification of site	C00.D48	Austria	805.30	3	yes
C80	Malignant neoplasm without specification of site	C00.D48	Belgium	1193.36	2	yes
C80	Malignant neoplasm without specification of site	C00.D48	Croatia	437.90	0	no
C80	Malignant neoplasm without specification of site	C00.D48	Czech Republic	1191.43	0	no
C80	Malignant neoplasm without specification of site	C00.D48	Denmark	917.91	3	yes
C80	Malignant neoplasm without specification of site	C00.D48	Estonia	104.51	1	yes
C80	Malignant neoplasm without specification of site	C00.D48	Finland	361.44	1	yes
C80	Malignant neoplasm without specification of site	C00.D48	France	8321.35	3	yes
C80	Malignant neoplasm without specification of site	C00.D48	Germany	9806.38	0	no
C80	Malignant neoplasm without specification of site	C00.D48	Hungary	695.39	1	yes
C80	Malignant neoplasm without specification of site	C00.D48	Latvia	175.96	1	yes
C80	Malignant neoplasm without specification of site	C00.D48	Lithuania	224.07	2	yes
C80	Malignant neoplasm without specification of site	C00.D48	Netherlands	2441.59	2	yes
C80	Malignant neoplasm without specification of site	C00.D48	Norway	528.61	1	yes
C80	Malignant neoplasm without specification of site	C00.D48	Poland	7576.47	3	yes
C80	Malignant neoplasm without specification of site	C00.D48	Romania	1547.13	2	yes
C80	Malignant neoplasm without specification of site	C00.D48	Slovenia	108.97	1	yes
C80	Malignant neoplasm without specification of site	C00.D48	Spain	4848.20	1	yes
C80	Malignant neoplasm without specification of site	C00.D48	Sweden	919.76	2	yes
C80	Malignant neoplasm without specification of site	C00.D48	Switzerland	504.34	2	yes
C80	Malignant neoplasm without specification of site	C00.D48	United Kingdom	13226.62	1	yes
C81	Hodgkin's disease	C00.D48	Austria	35.30	1	yes
C81	Hodgkin's disease	C00.D48	Belgium	55.90	1	yes

For peer review only

C81	Hodgkin's disease	C00.D48	Croatia	26.93	0	no
C81	Hodgkin's disease	C00.D48	Czech Republic	76.69	2	yes
C81	Hodgkin's disease	C00.D48	Denmark	32.10	0	no
C81	Hodgkin's disease	C00.D48	Estonia	7.76	0	no
C81	Hodgkin's disease	C00.D48	Finland	23.99	2	yes
C81	Hodgkin's disease	C00.D48	France	299.70	1	yes
C81	Hodgkin's disease	C00.D48	Germany	344.19	1	yes
C81	Hodgkin's disease	C00.D48	Hungary	49.75	2	yes
C81	Hodgkin's disease	C00.D48	Latvia	14.79	0	no
C81	Hodgkin's disease	C00.D48	Lithuania	22.14	1	yes
C81	Hodgkin's disease	C00.D48	Netherlands	91.10	0	no
C81	Hodgkin's disease	C00.D48	Norway	17.80	0	no
C81	Hodgkin's disease	C00.D48	Poland	299.59	3	yes
C81	Hodgkin's disease	C00.D48	Romania	146.88	1	yes
C81	Hodgkin's disease	C00.D48	Slovenia	11.53	0	no
C81	Hodgkin's disease	C00.D48	Spain	251.90	1	yes
C81	Hodgkin's disease	C00.D48	Sweden	37.02	0	no
C81	Hodgkin's disease	C00.D48	Switzerland	34.60	0	no
C81	Hodgkin's disease	C00.D48	United Kingdom	339.75	0	no
C82	Follicular [nodular] non-Hodgkin's lymphoma	C00.D48	Austria	18.97	1	yes
C82	Follicular [nodular] non-Hodgkin's lymphoma	C00.D48	Belgium	11.58	2	yes
C82	Follicular [nodular] non-Hodgkin's lymphoma	C00.D48	Croatia	5.50	1	yes
C82	Follicular [nodular] non-Hodgkin's lymphoma	C00.D48	Czech Republic	89.80	1	yes
C82	Follicular [nodular] non-Hodgkin's lymphoma	C00.D48	Denmark	20.62	0	no
C82	Follicular [nodular] non-Hodgkin's lymphoma	C00.D48	Finland	50.14	1	yes
C82	Follicular [nodular] non-Hodgkin's lymphoma	C00.D48	France	128.05	1	yes
C82	Follicular [nodular] non-Hodgkin's lymphoma	C00.D48	Germany	130.04	2	yes
C82	Follicular [nodular] non-Hodgkin's lymphoma	C00.D48	Hungary	19.01	1	yes
C82	Follicular [nodular] non-Hodgkin's lymphoma	C00.D48	Lithuania	14.77	1	yes
C82	Follicular [nodular] non-Hodgkin's lymphoma	C00.D48	Norway	19.53	3	yes
C82	Follicular [nodular] non-Hodgkin's lymphoma	C00.D48	Poland	171.63	0	no
C82	Follicular [nodular] non-Hodgkin's lymphoma	C00.D48	Romania	40.44	0	no
C82	Follicular [nodular] non-Hodgkin's lymphoma	C00.D48	Slovenia	10.26	2	yes
C82	Follicular [nodular] non-Hodgkin's lymphoma	C00.D48	Spain	113.21	1	yes
C82	Follicular [nodular] non-Hodgkin's lymphoma	C00.D48	Sweden	29.55	0	no
C82	Follicular [nodular] non-Hodgkin's lymphoma	C00.D48	Switzerland	23.36	2	yes
C82	Follicular [nodular] non-Hodgkin's lymphoma	C00.D48	United Kingdom	186.55	1	yes
C83	Diffuse non-Hodgkin's lymphoma	C00.D48	Austria	107.00	1	yes
C83	Diffuse non-Hodgkin's lymphoma	C00.D48	Belgium	71.89	2	yes
C83	Diffuse non-Hodgkin's lymphoma	C00.D48	Croatia	25.55	3	yes
C83	Diffuse non-Hodgkin's lymphoma	C00.D48	Czech Republic	236.37	1	yes
C83	Diffuse non-Hodgkin's lymphoma	C00.D48	Denmark	91.60	0	no
C83	Diffuse non-Hodgkin's lymphoma	C00.D48	Estonia	39.44	2	yes
C83	Diffuse non-Hodgkin's lymphoma	C00.D48	Finland	359.13	2	yes
C83	Diffuse non-Hodgkin's lymphoma	C00.D48	France	768.60	1	yes
C83	Diffuse non-Hodgkin's lymphoma	C00.D48	Germany	687.71	2	yes
C83	Diffuse non-Hodgkin's lymphoma	C00.D48	Hungary	98.77	2	yes
C83	Diffuse non-Hodgkin's lymphoma	C00.D48	Latvia	22.97	2	yes
C83	Diffuse non-Hodgkin's lymphoma	C00.D48	Lithuania	88.83	1	yes
C83	Diffuse non-Hodgkin's lymphoma	C00.D48	Netherlands	64.83	3	yes

For peer review only

C83	Diffuse non-Hodgkin's lymphoma	C00.D48	Norway	60.18	3	yes
C83	Diffuse non-Hodgkin's lymphoma	C00.D48	Poland	517.07	1	yes
C83	Diffuse non-Hodgkin's lymphoma	C00.D48	Romania	157.91	1	yes
C83	Diffuse non-Hodgkin's lymphoma	C00.D48	Slovenia	29.17	3	yes
C83	Diffuse non-Hodgkin's lymphoma	C00.D48	Spain	412.80	2	yes
C83	Diffuse non-Hodgkin's lymphoma	C00.D48	Sweden	181.90	1	yes
C83	Diffuse non-Hodgkin's lymphoma	C00.D48	Switzerland	120.29	1	yes
C83	Diffuse non-Hodgkin's lymphoma	C00.D48	United Kingdom	796.15	1	yes
C84	Peripheral and cutaneous T-cell lymphomas	C00.D48	Austria	46.00	1	yes
[truncated: 713,890 more chars]
